# Global burden of diabetes in women from 1990 to 2021, with projections to 2050: population-based study

**DOI:** 10.1186/s12916-025-04361-y

**Published:** 2025-10-08

**Authors:** Fanchao Shi, Qian Zhao, Yi Yang, Lu Liu, Xiao Zhang, Hyun Je Kim, Yanzhong Wang, Jun Pu

**Affiliations:** 1 Department of Cardiology, Renji Hospital, School of Medicine, State Key Laboratory of Systems Medicine for Cancer, Shanghai Cancer Institute, Shanghai Jiao Tong University, Shanghai, 200127 China; 2 BHF Cardiovascular Epidemiology Unit, Department of Public Health and Primary Care, University of Cambridge, Cambridge, CB2 0BB UK; 3 Department of Cardiology, People’s Hospital of Xinjiang Uyghur Autonomous Region, Xinjiang, 830001 China; 4 Department of Geriatrics, Tongji Hospital of Tongji Medical College, Huazhong University of Science and Technology, Wuhan, 430030 China; 5 Key Laboratory of Vascular Aging, Ministry of Education, Tongji Hospital of Tongji Medical College, Huazhong University of Science and Technology, Wuhan, 430030 China; 6 School of Life Course and Population Sciences, King’s College London, London, SE1 1UL UK; 7 Department of Biostatistics, School of Public Health, Peking University, No.38 Xueyuan Road, Beijing, 100191 China; 8 Department of Biomedical Sciences, Seoul National University Graduate School, Seoul, 03080 South Korea

**Keywords:** Type 1 diabetes, Type 2 diabetes, Women, Global burden of disease, Projection

## Abstract

**Background:**

The global burden of women's health is underestimated, with diabetes disproportionately affecting women due to sex-specific health conditions, unequal healthcare access, and underrepresentation in clinical trials. However, none has comprehensively examined the global burden of diabetes in women.

**Methods:**

Diabetes data were extracted from Global Burden of Disease 2021. Age-standardized incidence, mortality, and disability-adjusted life years (DALY), with the corresponding average annual percent change (AAPC), were calculated at global, sociodemographic index (SDI), regional, and national levels. Age-specific patterns, DALY attributable to risk factors, and DALY projections to 2050 were also examined.

**Results:**

In women, the global age-standardized incidence of diabetes was 273 (95% uncertainty interval 253 to 294) per 100 000 population in 2021, with 2.5% being type 1 diabetes (T1D). From 1990 to 2021, age-standardized incidence of T1D increased, with an AAPC of 0.48% (*p* < 0.001); while mortality and DALY decreased (*p* < 0.001). High SDI showed a T1D incidence 1.4 to 2.3 times that of other SDI levels, with a six-fold faster growth in incidence compared to Low-middle SDI (1.07% vs 0.18% per year). T1D incidence exhibited an approximately U-shaped association with age, peaking in women aged < 25 and > 80 years. For type 2 diabetes (T2D) in women, the global age-standardized incidence, mortality, and DALY all significantly increased over time, despite declining mortality at High and High-middle SDI levels (*p* < 0.001). Notably, every 5-year decrease in age was associated with a 0.30% (95% confidence interval 0.27% to 0.33%) higher AAPC in incidence rate and a 0.08% (0.06% to 0.10%) higher AAPC in DALY rate for T2D in women. By 2050, the age-standardized DALY of T1D and T2D are expected to decline and rise respectively, with both experiencing an increasing proportion of their burden attributed to non-fatal ones.

**Conclusions:**

Diabetes imposes a significant burden on women throughout their lifespan. Despite improved survival, T1D incidence in women has increased, especially at High SDI. The growing incidence and DALY of T2D represent another concern, with a notable shift toward younger women. The proportional non-fatal burden of T1D and T2D is forecasted to increase by 2050, highlighting the need for effective prevention and long-term management.

**Supplementary Information:**

The online version contains supplementary material available at 10.1186/s12916-025-04361-y.

## Background

Diabetes, characterised by elevated blood glucose related to the abnormal β-cell function on insulin action [1], has been prioritized as one of the top non-communicable disease targets in the UN and WHO Action Plan to confront the global disease burden [2, 3]. Increasing evidence suggests that diabetes poses a greater risk of adverse outcomes in women than in men [4], underscoring the need for a female-centred approach to better understand and address these sex-specific health challenges. Women experience distinct hormonal fluctuations and reproductive factors— such as polycystic ovary syndrome, gestational diabetes, and menopause— that uniquely influence diabetes risk and progression [5–7]. Additionally, women with diabetes face higher risks of pregnancy complications [8] and are typically diagnosed at later stages and a higher body mass index (BMI) than men [9]. Despite these differences, women with diabetes remain underrepresented in randomized controlled trials [10] and are less likely to achieve treatment targets recommended by guidelines [4, 5]. Given these disparities, women represent a key population requiring targeted attention in global diabetes prevention and management efforts.

Previous studies have reported on the global burden and trend of diabetes over decades [11], as well as its specific impacts on children and adolescents [12–14], young adults [12, 14], and older adults [15]. Although some of them included female subgroups, few have specifically and comprehensively described the global burden and secular development of diabetes in women across different scales. Variations in diabetes burden across the female lifespan—particularly in the context of global population ageing—have not been systematically characterized. The contribution of modifiable risk factors at different life stages and the projected evolution of the diabetes burden among women in the coming decades remain insufficiently understood. These knowledge gaps hinder the development of effective, gender-responsive strategies.

We therefore investigated the global epidemiological characteristics of total and type-specific (type 1 and 2) diabetes in women, along with attributable risk factors and projections to 2050.

## Methods

### Study population and data collection

The Global Burden of Disease (GBD) 2021 project estimated the global burden of 371 diseases and injuries and 88 risk factors across 204 countries and territories from 1990 to 2021 and forecasted the burden of disease scenarios from 2022 to 2050. Details of GBD 2021 have been published previously [16–18]. The study population of our analysis comprised people with diabetes (including both type 1 and 2) across all ages, with a primary focus on women. From GBD 2021, we extracted data on diabetes with corresponding 95% uncertainty intervals (UI) from Global Health Data Exchange [16–20]. The GBD classified countries and territories into seven super-regions based on epidemiological similarity and geographic closeness, which are further subdivided into 21 sub-regions (see Additional file 1: Supplementary Methods) [11, 16–18, 21].

### Estimation of disease burden of diabetes in GBD 2021

In GBD 2021 [16], diabetes was defined as a fasting plasma glucose of ≥ 126 mg/dl (7 mmol/L), or the use of insulin or diabetic medication. Type 1 diabetes (T1D) comprised cases diagnosed by physician, cases recorded in diabetic registries or hospitals, or diabetes cases in persons < 15 years who were on insulin. First, a Bayesian meta-regression modelling tool (DisMod-MR 2.1) was applied to estimate the incidence and prevalence of total diabetes and T1D from 1990 to 2021 using data sources from scientific literature, survey microdata, and insurance claims, with those of type 2 diabetes (T2D) indirectly calculated by subtracting the estimation of T1D from total diabetes. Years lived with disability (YLD) were obtained from prevalence and disability weight after a micro-simulation corrected for comorbidity. Second, mortality from T1D, T2D, and total diabetes was estimated using the Cause of Death Ensemble model (CODEm) approach, by incorporating data from vital registration and verbal autopsy reports in separate models adjusted for selected covariates. To sort out death coded to diabetes which did not specify a type, a regression model was developed to predict the type-specific proportion of death among them. Years of life lost (YLL) were computed by multiplying the number of estimated deaths by the standard life expectancy at age of death. Finally, disability-adjusted life years (DALY) were equal to the sum of YLD and YLL. Risk-attributable DALY were further modelled for 17 detailed factors for diabetes [17]: high air temperature, low air temperature, ambient particulate matter pollution, household air pollution from solid fuels, smoking, second-hand smoke, alcohol use, diet low in fruits, diet low in vegetables, diet low in whole grains, diet high in red meat, diet high in processed meat, diet high in sugar-sweetened beverages, diet low in fibre, low physical activity, high BMI, and high fasting plasma glucose (FPG).

Projections of diabetes burden from 2022 to 2050 were estimated in GBD 2021 from mixed-effects models, using forecasts of key health determinants, including the Sociodemographic Index (SDI) and the comprehensive set of risk factor exposures captured by GBD [18]. A reference forecast (the most likely future) and four alternative scenarios, in which selected sets of risk factors were eliminated, were provided [18]: (i) environmental risks (Safer Environment scenario), (ii) risks associated with communicable, maternal, neonatal, and nutritional diseases (Improved Childhood Nutrition and Vaccination scenario), (iii) risks associated with major non-communicable diseases (Improved Behavioural and Metabolic Risks scenario), and (iv) the combined effects of these three scenarios.

Additional file 1 and other publications [16–18] provide details of relevant definitions and estimation methods in GBD 2021.

### Statistical analysis

We reported primarily on incidence, mortality and DALY because these metrics are particularly salient for characterizing and capturing aspects of the rapid global rise of diabetes, and provided estimates of prevalence, YLD, and YLL in Additional file 2 and 3. To assess the magnitude and direction of temporal trends in these metrics for diabetes, the average annual percent change (AAPC) was estimated by joinpoint regression [22]. By allowing a maximum number of six joinpoints and using a Weighted Bayesian Information Criteria method, joinpoint regression analysis could identify the best fit for joinpoints where there were statistically significant changes in trend. Sensitivity analysis assuming first order autocorrelation estimated from the data was conducted. AAPC is used to represent the average rate of change of a specific variable in a chosen period, calculated from the slope coefficient of each segment in a weighted manner. If AAPC was > 0 (or < 0) and *p*-value < 0.05, we considered the metric on investigation to be in an upward (or downward) trend.

The contribution of each risk factor to disease burden was estimated by dividing the attributable DALY for that factor by total DALY of disease in GBD 2021. Of the 17 detailed factors, we exclude alcohol use in our analysis due to its uncertain causality or its harmfulness being dependent on the amount of consumption [21]. High FPG is also excluded because GBD 2021 assumes a population attributable fraction of 100% for high FPG. The remaining 15 risk factors fall into six categories: non-optimal temperature, air pollution, tobacco use, dietary risks, low physical activity, and high BMI. All risk factors have shown to be associated with T2D, but non-optimal temperatures are the only risk factors associated with T1D.

We further investigated patterns of these metrics by SDI and across age spectrums (per 5-year interval) among women, as well as comparisons with men. A fractional polynomial of degree 2 was applied to characterise the potential linear or non-linear relations between relevant metrics and age. Linear regression adjusting for global and SDI levels was used, where applicable, to quantify the association between AAPC and age.

All statistical analyses were conducted using Stata 15.0, Joinpoint Regression program 5.0.2, and R 4.2.3.

## Results

### Global and SDI-level trends

**Fig. 1:**
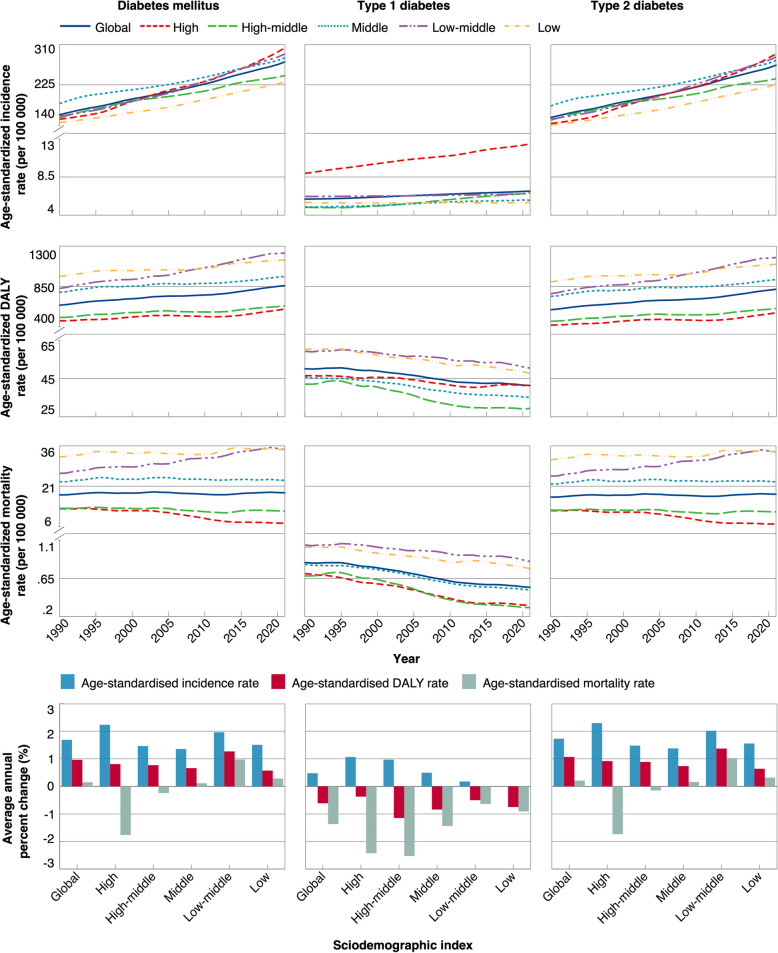
Temporal trends in age-standardized incidence, DALY, and mortality rates among women at the global and SDI regional levels, 1990–2021. DALY: Disability adjusted life years; SDI: Sociodemographic index. This figure displays the temporal trends in age-standardized incidence, DALY and mortality for the burden of total and type-specific diabetes among women globally and by SDI from 1990 to 2021, as well as the corresponding AAPC for the trends. Five SDI categories include countries with a High, High-middle, Middle, Low-middle, or Low SDI. Additional file 3: Fig. S1 presented the trends in prevalence, years lived with disability, and years of life lost. Additional file 2: Table S1-S6 provided detailed numerical data corresponding to these trends, with Additional file 2: Table S7-S8 presenting additional segment-specific annual percentage changes

In 2021, there were an estimated 11.7 million (95% UI 10.9 to 12.7) diabetes cases occurring in women globally, representing 48% of all new-onset diabetes. The corresponding age-standardized incidence of diabetes was 273 (253 to 294) per 100 000 women, with 2.5% being T1D (Fig. 1; Additional file 2: Table S1-S8; Additional file 3: Fig. S1). From 1990 to 2021, the age-standardized incidence of T1D in women increased from 5.9 to 6.8 per 100 000 (AAPC: 0.48%), with a higher AAPC observed at higher SDI levels. Countries with High SDI, which consistently exhibited 1.4 to 2.3 times the T1D incidence of other SDI levels, demonstrated the greatest growth — a staggering six times faster than in countries with Low-middle SDI (AAPC: 1.07% vs. 0.18%). Conversely, the age-standardized mortality and DALY from T1D in women decreased during the same period across SDI levels, among which countries with Low-middle and Low SDI always showed the highest rates. For T2D, the age-standardized incidence and DALY in women largely increased between 1990 and 2021 at global and all SDI levels (AAPC, ranged from 0.64% to 2.29%). In contrast, the global rise in T2D mortality among women was less noticeable (0.21%), with heterogeneous trends by SDI, which declined in High and High-middle SDI but rose in others. Overall, the disease burden (all cause DALY) from diabetes in women has been increasing over the past three decades, consistently surpassing that observed in men (Additional file 3: Fig. S2).

### Regional and national trends

#### Type 1 diabetes

**Fig. 2:**
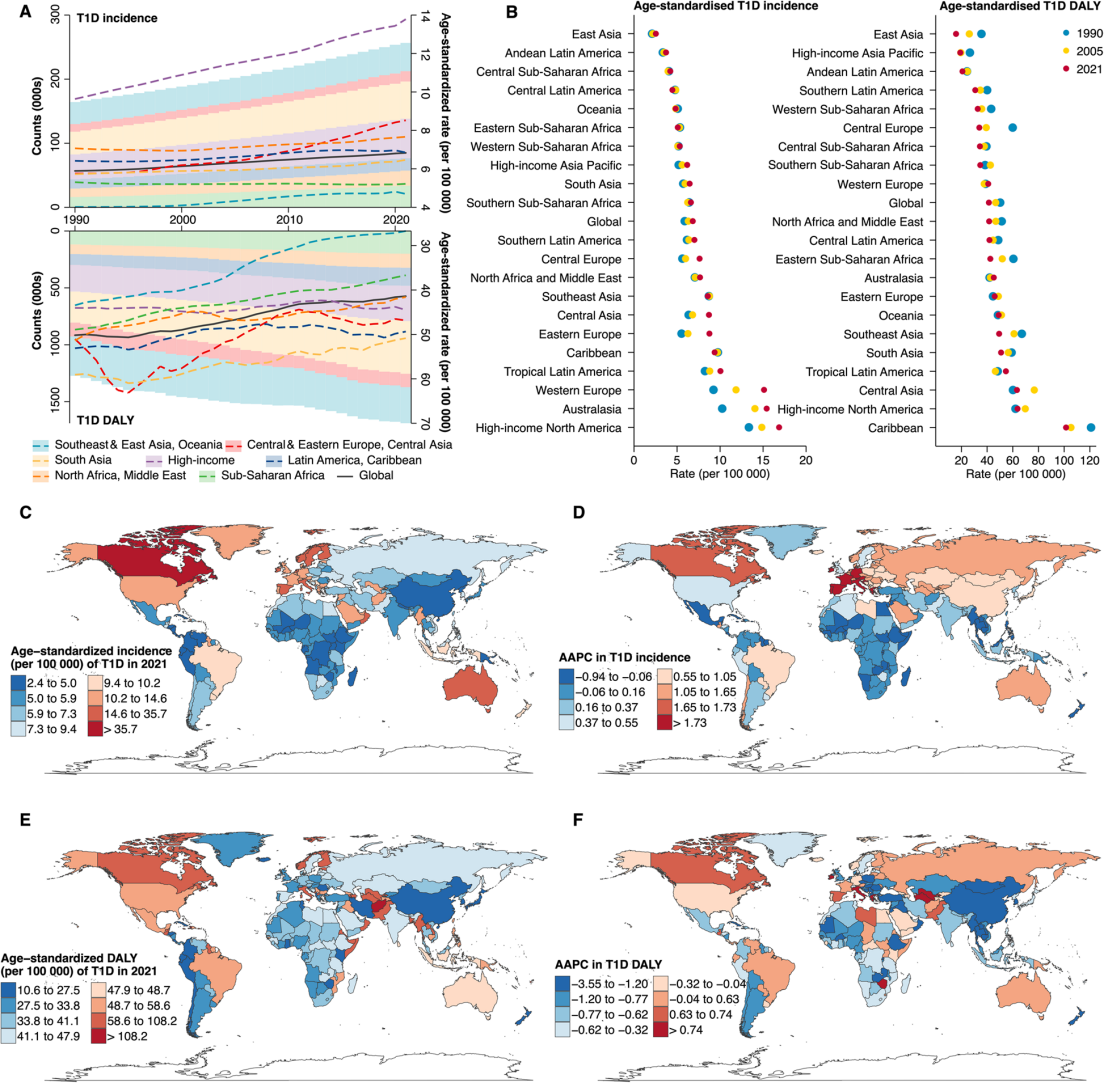
Temporal trends in age-standardized incidence and DALY of type 1 diabetes among women by 7 super-regions, 21 sub-regions, and 204 countries and territories, 1990–2021. AAPC: Average annual percentage change; DALY: Disability adjusted life years; T1D: Type 1 diabetes. Panel **A** illustrates the absolute counts (000 s, shown as shaded areas) and age-standardized rates (per 100 000 population, depicted as lines) of T1D incidence and DALY across seven super-regions from 1990 to 2021. Panel **B** presents the age-standardized incidence and DALY rates (per 100 000 population) of T1D across 21 sub-regions in three distinct years: 1990, 2005, and 2021. Panel **C** displays the age-standardized incidence rate (per 100 000 population) of T1D in 2021, with Panel **D** showing the corresponding AAPC for the trend from 1990 to 2021. Panel **E** displays the age-standardized DALY rate (per 100 000 population) of T1D in 2021, with Panel **F**) showing the corresponding AAPC for the trend from 1990 to 2021. Additional file 3: Fig. S5 presented the map for prevalence, mortality, years lived with disability, and years of life lost. Additional file 2: Table S1-S6 provided detailed numerical data corresponding to these trends, with Additional file 2: Table S7-S8 presenting additional segment-specific annual percentage changes

From 1990 to 2021, all super-regions except Sub-Saharan Africa (AAPC: −0.05%) experienced an increase in T1D incidence among women, while none exhibited a rise in mortality or DALY (Fig. 2; Additional file 2: Table S1, S3, and S6; Additional file 3: Fig. S3). Central Europe, Eastern Europe, and Central Asia (1.28%) and High-income (1.14%) recorded the fastest growth in age-standardized T1D incidence among women, more than twice as rapid as other super-regions. In 2021, High-income exhibited both the highest absolute number (0.06 million) and age-standardized rate (14 per 100 000) of T1D incidence among women, with its three sub-regions ranking at the top globally: High-income North America (17 per 100 000), Australasia (15 per 100 000), and Western Europe (15 per 100 000). Nationwide, most of the top 15 countries with the highest age-standardized incidence (*n* = 14) and the largest growth in incidence (*n* = 13) were from High-income (e.g., highest incidence: Canada; highest AAPC: Cyprus), making it the only super-region without a reduction in total T1D burden among women, despite a substantial decrease in mortality (Additional file 3: Fig. S4).

In 2021, Southeast Asia, Caribbean, North Africa and Middle East, Andean Latin America, and South Asia all demonstrated higher age-standardized incidence, mortality and DALY of T1D in women than in men (Additional file 3: Fig. S3). In contrast, Oceania was the only sub-region where mortality and DALY increased in women but decreased in men between 1990 and 2021. Notably, Caribbean exhibited the highest mortality (1.75 per 100 000) and DALY (101 per 100 000) among women in 2021, surpassing those of any other sub-regions by at least 81% and 59%, respectively. Nationally, Haiti recorded the highest mortality (4.7 per 100 000) and DALY (227 per 100 000) of T1D in women, at least double those in any other countries or territories (Fig. 2; Additional file 2: Table S1-S6; Additional file 3: Fig. S5). In Haiti, the age-standardized T1D mortality and DALY in women were 79% and 73% higher than that in men, respectively. Notably, Afghanistan had the largest sex disparities globally in 2021— with women showing 2.08-fold higher incidence (10.77 [95% UI: 9.14 to 12.91]) vs. 5.18 [4.45 to 6.05] per 100 000), 3.37-fold higher mortality (1.77 [1.13 to 2.84] vs. 0.52 [0.35 to 0.78]), and 2.87-fold higher DALYs (116.19 [83.12 to 169.71] vs. 40.51 [30.53 to 55.02]) than men— and ranked third globally for the total T1D burden in women (Additional file 3: Fig. S6).

#### Type 2 diabetes

**Fig. 3:**
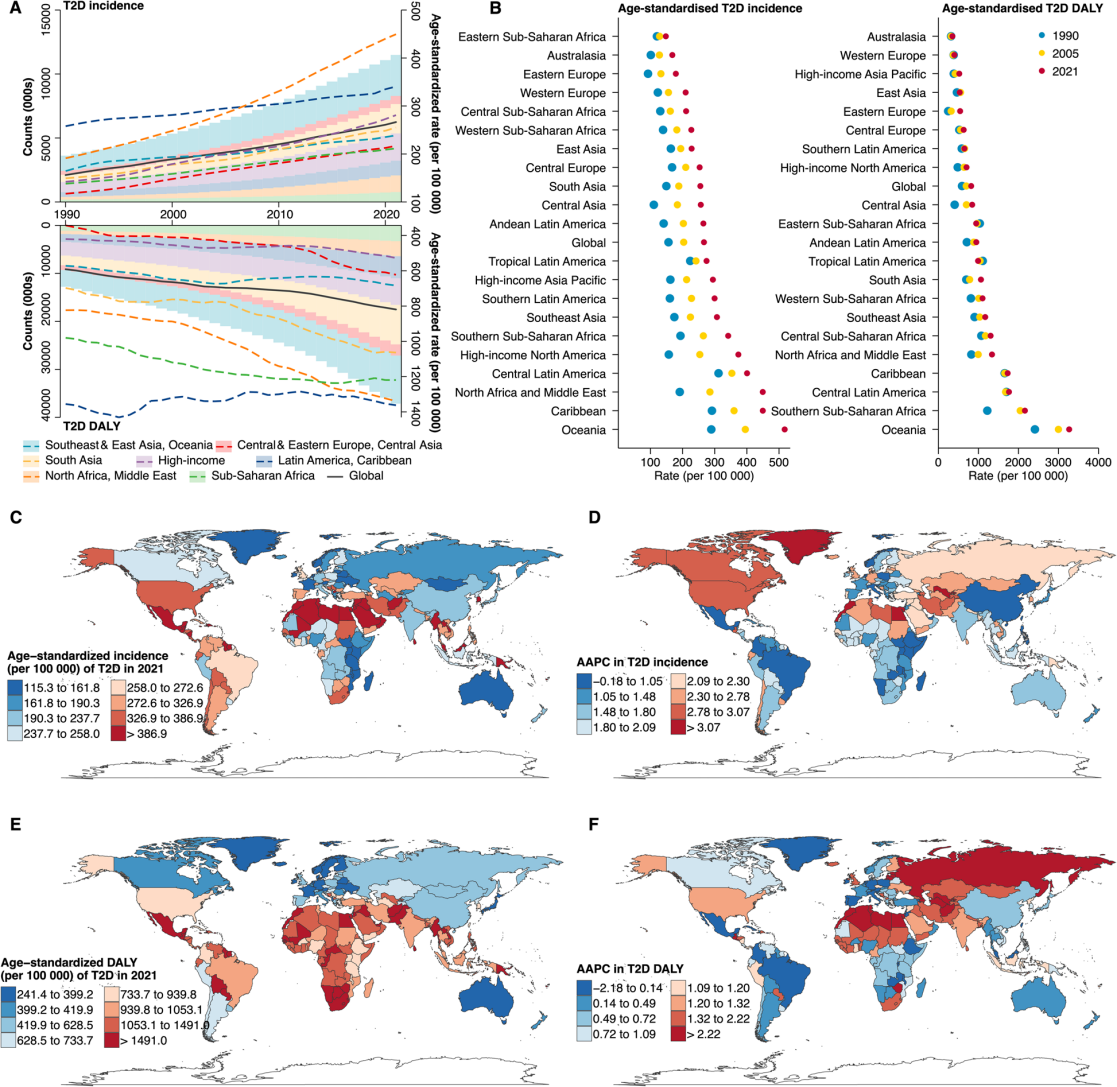
Temporal trends in age-standardized incidence and DALY of type 2 diabetes among women by 7 super-regions, 21 sub-regions, and 204 countries and territories, 1990–2021. AAPC: Average annual percentage change; DALY: Disability adjusted life years; T2D: Type 2 diabetes. Panel **A** illustrates the absolute counts (000 s, shown as shaded areas) and age-standardized rates (per 100 000 population, depicted as lines) of T2D incidence and DALY across seven super-regions from 1990 to 2021. Panel **B** presents the age-standardized incidence and DALY rates (per 100 000 population) of T2D across 21 sub-regions in three distinct years: 1990, 2005, and 2021. Panel **C** displays the age-standardized incidence rate (per 100 000 population) of T2D in 2021, with Panel **D** showing the corresponding AAPC for the trend from 1990 to 2021. Panel **E** displays the age-standardized DALY rate (per 100 000 population) of T2D in 2021, with Panel **F**) showing the corresponding AAPC for the trend from 1990 to 2021. Additional file 3: Fig. S8 presented the map for prevalence, mortality, years lived with disability, and years of life lost. Additional file 2: Table S1-S6 provided detailed numerical data corresponding to these trends, with Additional file 2: Table S7-S8 presenting additional segment-specific annual percentage changes

Between 1990 and 2021, no super-regions witnessed a reduction in age-standardized incidence or DALY of T2D among women (Fig. 3). However, age-standardized mortality declined in High-income (AAPC: −2.14%), Latin America and Caribbean (−0.67%), and Southeast Asia, East Asia, and Oceania (−0.17%) (Additional file 3: Fig. S7). North Africa and Middle East, which reported the highest age-standardized T2D incidence (449 per 100 000) among women in 2021, showed the most rapid increase over time (AAPC: 2.80%), followed by High-income (2.23%). Despite a notable increase in incidence, the lowest age-standardized mortality and the fastest decline position High-income super-region as having the lowest T2D burden among women in 2021 (528 per 100 000). Leading sub-regions (e.g., Australasia and Western Europe) and countries (e.g., France and Ireland) with the lowest total T2D burden were all from High-income (Fig. 3; Additional file 2: Table S1-S6; Additional file 3: Fig. S8**)**.

In contrast, Oceania had the highest age-standardized incidence (518 per 100 000), mortality (99 per 100 000), and DALY (3266 per 100 000) of T2D among women in 2021, exceeding all other sub-regions by more than 15%, 27%, and 51%, respectively (Additional file 3: Fig. S7). Oceania also exhibited the most pronounced sex disparities in AAPC across these metrics, with women experiencing more adverse trends in incidence (AAPC: 1.88% vs 1.61%), mortality (0.63% vs −0.14%) and DALY (0.94% vs 0.34%) compared to men. At the national level, the 14 countries with the highest T2D burden among women in 2021 were all from Oceania, among which Marshall Islands recorded the greatest age-standardized DALY (7462 per 100 000), surpassing the male DALY by 83% (Fig. 3; Additional file 2: Table S6). Additionally, four other countries — United Arab Emirates, Haiti, Afghanistan, and Zimbabwe — had DALY at least 50% higher among women than men in 2021 (Additional file 3: Fig. S9).

### Age- and population-structure characteristics

#### Type 1 diabetes

**Fig. 4:**
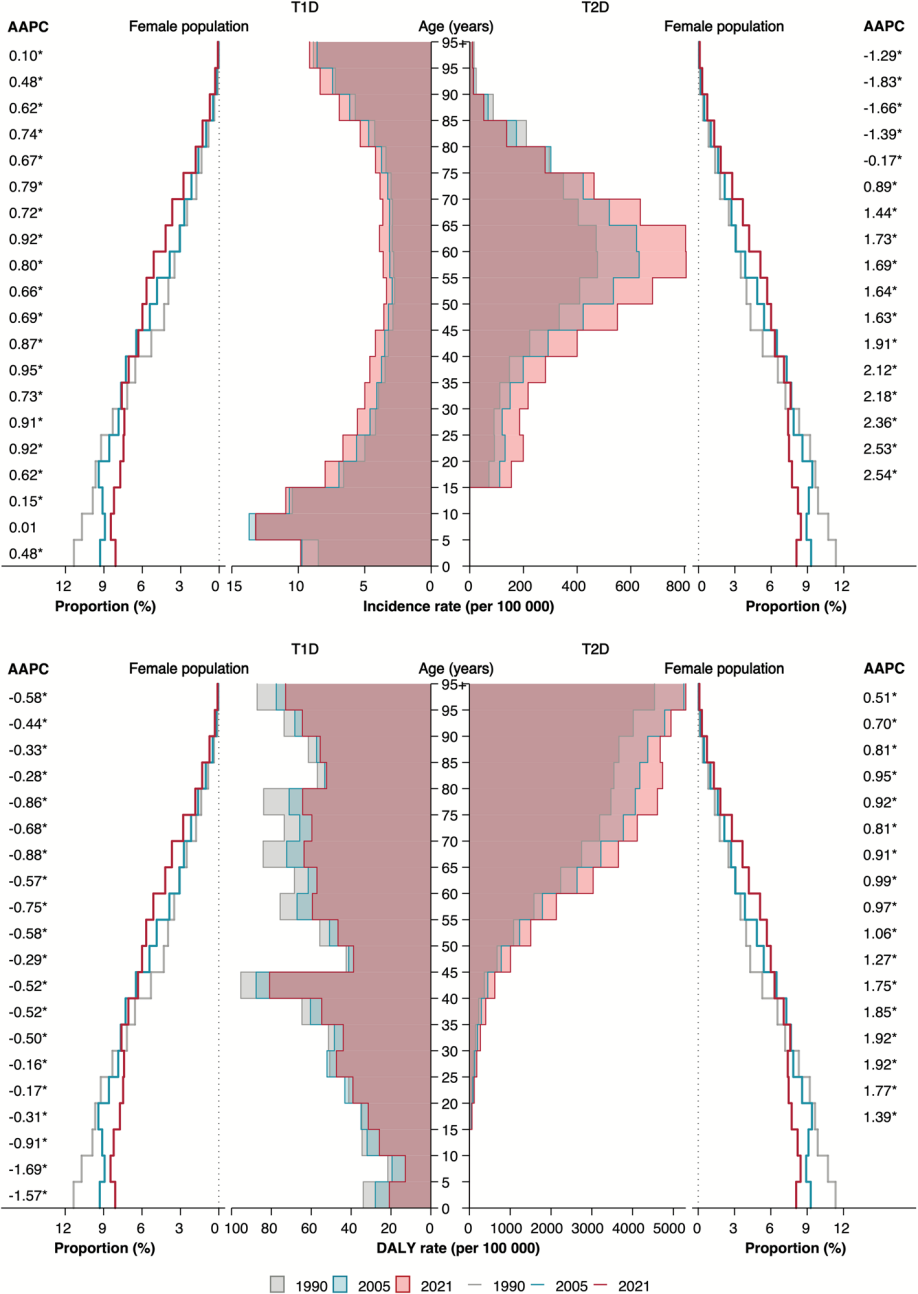
Diabetes incidence and DALY rates by age and subtype groups among women in 1990, 2005, and 2021, along with population structures. AAPC: Average annual percentage change; DALY: Disability adjusted life years; T1D: Type 1 diabetes; T2D: Type 2 diabetes. * *p* < 0.05. The shaded areas display the age-specific incidence rates (per 100 000 population) and DALY rates (per 100 000 population) for T1D and T2D in 1990, 2005, and 2021, respectively. The lines show the proportion (%) of the total female population accounted for by 5-year age-specific female population groups (e.g., 0–4, 5–9 years, et al.) in 1990, 2005, and 2021

Concomitant with an aging female population, the number, rate, and proportion relative to all causes of T1D incidence generally increased from 1990 to 2021 across age groups (Fig. 4; Additional file 3: Fig. S10). Although the absolute number generally declined with age, incidence rates and proportions showed an approximately U-shaped age pattern, peaking in women under 25 and over 80 years. In contrast, age-specific mortality and DALY rates all decreased between 1990 and 2021, despite increases in absolute numbers among women aged 20 years and older. Peaks in DALY and mortality rates occurred at ages 40–45, 75–80, and 95 +. Notably, women over 60 years consistently showed higher T1D incidence numbers and rates than men across SDI levels from 1990 to 2021, despite somewhat overlapping UIs (Additional file 3: Fig. S11-S14).

#### Type 2 diabetes

The age-specific number, rate, and proportion relative to all causes of T2D incidence in women rose with age, peaking at age 55–60 before declining (Fig. 4; Additional file 3: Fig. S10). The incidence rate peak in women was around 5 years earlier than in men (Additional file 3: Fig. S15). Mortality and DALY numbers and proportions showed similar reverse U-shaped patterns, while their rates increased gradually with age (Additional file 3: Fig. S10). From 1990 to 2021, age-specific incidence rates and proportions in women increased initially but decreased after age 75. In contrast, DALY rates and proportions rose across all female age groups during this period. For per five-year reduction in age, the AAPC for incidence and DALY rate in women increased by approximately 0.30% (95% confidence interval [CI] 0.27% to 0.33%) and 0.08% (0.06% to 0.10%), respectively, with similar estimates observed in proportions (Additional file 3: Fig. S16). Mortality rates and proportions declined in women across most age groups at High SDI, while other SDI levels showed variable patterns. Women under 30 years generally exhibited higher numbers and rates of T2D mortality than men— despite overlapping UIs— particularly at Low and Low-middle SDI (Additional file 3: Fig. S17-S20).

### Risk factors

**Fig. 5:**
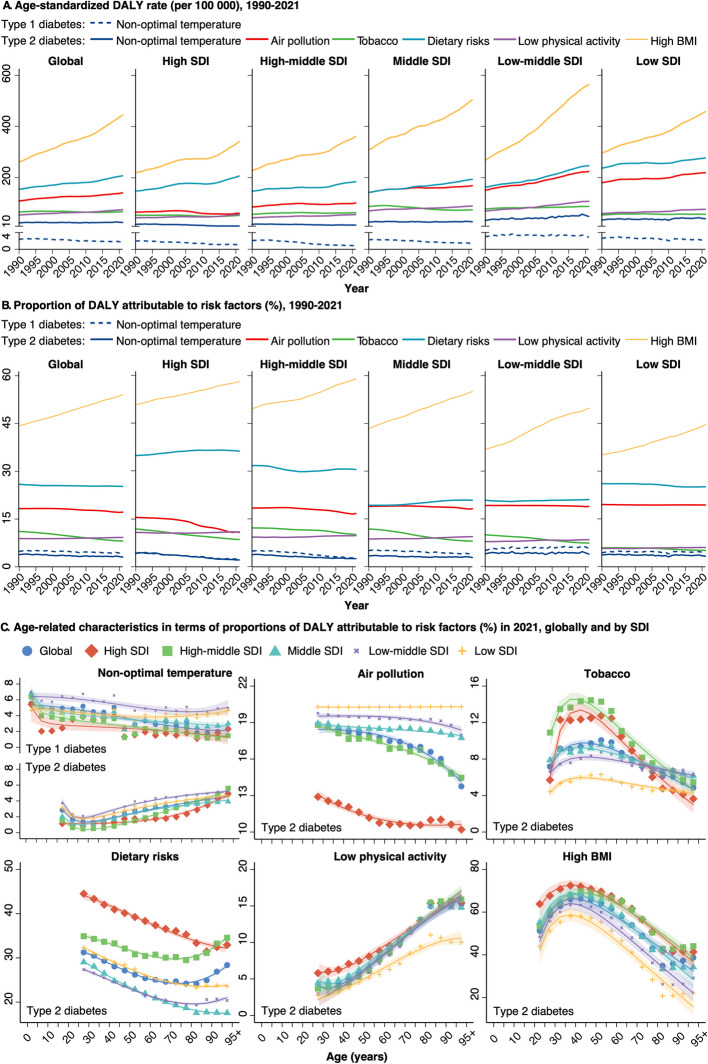
Contributions of risk factors to type 1 and type 2 diabetes among women, by SDI and age groups. DALY: Disability adjusted life years; SDI: Sociodemographic index. Panel **A** illustrates the temporal trends in age-standardized DALY rates (per 100,000 population) for T1D and T2D attributable to risk factors globally and at SDI levels from 1990–2021. Panel **B** shows the temporal trends in the proportion (%) of T1D and T2D DALY attributable to risk factors globally and at SDI levels over the same period (1990–2021). Panel **C** characterizes the age-related patterns of the proportion (%) of DALY attributable to risk factors for T1D and T2D globally and at SDI levels in 2021

Non-optimal temperature, defined as those falling above or below the minimum-risk exposure level, was reported as the main risk factor for T1D in GBD 2021 (Fig. 5). From 1990 to 2021, the T1D burden attributable to low temperature among women decreased substantially, particularly at higher SDI levels, whereas the burden due to high temperature showed an increase (Additional file 3: Fig. S21-S22). In 2021, 2.3% (95%UI: 1.5% to 3.2%) and 1.9% (0.6% to 3.5%) of T1D DALY were from low and high temperature, respectively, with younger women broadly exhibiting higher proportional DALYs.

For T2D, high BMI was the leading contributor (54.0%; 95%UI: 25.8% to 74.0%) to DALY for women in 2021, followed by dietary risks (25.2%; 5.1% to 40.8%), air pollution (17.2%; 10.5% to 24.4%), low physical activity (9.2%; 4.0% to 14.0%), tobacco (8.1%; 4.1% to 12.0%) and non-optimal temperature (3.1%; 1.8% to 4.6%) (Fig. 5). Higher SDI levels typically showed higher proportions of DALY attributable to high BMI, low physical activity, diet, and tobacco use, but lower proportions from air pollution and non-optimal temperature. More specifically, high temperature, ambient particulate matter pollution, diet high in sugar-sweetened beverages, high BMI, low physical activity, and diet high in red meat all showed upwards trends in both age-standardized and proportional DALYs for T2D from 1990 to 2021 (Additional file 3: Fig. S21). Of these factors, high BMI, low physical activity, and diet high in sugar-sweetened beverages generally contributed to higher proportional DALYs in women than in men across age groups in 2021 (Additional file 3: Fig. S23). Furthermore, proportional DALYs attributable to diet high in sugar-sweetened beverages typically declined with age— especially at higher SDI levels— whereas those from low physical activity increased (Fig. 5; Additional file 3: Fig. S24). High BMI showed a reverse U-shaped age pattern in women, peaking at middle age.

### Projections of fatal and non-fatal burden to 2050

**Fig. 6:**
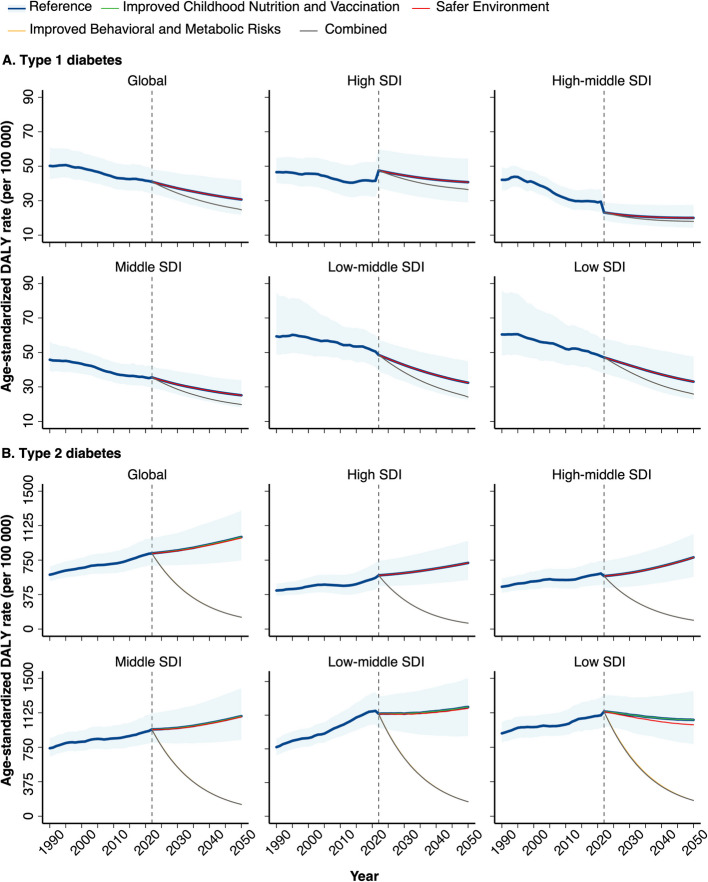
Age-standardized DALY rates of type 1 and type 2 diabetes among women at the global and SDI regional levels, for the past and for five future scenarios, 1990–2050. DALY: Disability adjusted life years; SDI: Sociodemographic index. The dashed vertical line indicates the year 2022 (the first forecast year). The blue shading indicates the 95% uncertainty interval for the reference scenario. The five scenarios include the reference scenario, Safer Environment scenario, Improved Behavioural and Metabolic Risks scenario, Improved Childhood Nutrition and Vaccination scenario, and the combined scenario

Trends of diabetes burden for women will largely be shaped by the evolving patterns in future demography and health scenarios (Fig. 6). In reference scenario, T1D DALY for women is expected to continue declining through 2050 globally and across SDI levels, primarily driven by a substantial decrease in YLL despite rising YLD (Additional file 3: Fig. S25-S26). In contrast, the increasing T2D DALY for women is forecasted to persist worldwide, largely attributed to the significant rise in YLD. Similar T2D patterns are projected across SDI levels, except for a decline in age-standardized DALY at Low SDI (Additional file 3: Fig. S27). For both T1D and T2D, the non-fatal burden measured by the proportion of DALY due to YLD is projected to rise globally, reaching 68% and 69% by 2050, respectively (Additional file 2: Table S9). This pattern is expected across all SDI levels, with higher proportions of non-fatal burden at higher SDI. Nevertheless, lower SDI are forecasted to exhibit a larger shift from fatal to non-fatal burden. The shift in diabetes burden is projected to be more pronounced in women (relative increase from 2022 to 2050: 64% for T1D and 31% for T2D) than in men (47% and 18%, respectively). Compared to the reference scenario, the Improved Behavioural and Metabolic Risks scenario is forecasted to yield the greatest improvements, with considerable reduction in DALY and YLL for T1D and T2D, and in YLD for T2D only; the Safer Environment scenario is expected to result in a slight decrease in YLL and DALY for T2D at Low SDI (Additional file 3: Fig. S25-S27).

## Discussion

### Principal findings

Diabetes presents a severe burden for women throughout their lifespans. Despite previous global burden analyses of diabetes in the general population, none has specifically focused on women. Our analyses of diabetes burden specifically in women uncovered the following novel findings. From 1990 to 2021, T1D incidence and prevalence increased among women across almost all ages despite significant declines in age-standardized mortality and DALY. Total T1D burden was highest in countries with Low-middle and Low SDI, driven by their highest fatal burden; however, we found the highest non-fatal burden, along with the fastest increase, in High SDI countries. Notably, T1D incidence rate exhibited an approximately U-shaped pattern with age, peaking in women aged over 80 and under 25. Over the same period, the T2D burden among women increased globally, with greater DALY at lower SDI levels. Age-standardized T2D incidence increased across SDI levels while age-standardized mortality increased only at Low to Middle SDI levels. The T2D burden exhibited a trend toward younger women, with the AAPC increasing by 0.30% in incidence rate and 0.08% in DALY rate per 5-year decrease in age. We also found patterns of attributable risk factors for T1D and T2D varied by age and SDI levels. Finally, the observed trends in diabetes burden are forecasted to persist by 2050, with a notable shift from fatal to non-fatal burdens. Our findings highlight complex disparities and evolving trends in the diabetes burden among women, underscoring the need for tailored resource allocation and targeted guidelines to address this escalating global challenge.

### Healthy aging at risk: the disproportionate T1D burden among older women

T1D is historically recognized as a common childhood-onset autoimmune disease but can develop at any age [23]. We discovered a roughly U-shaped age pattern, with a potential second peak of T1D incidence in women over 80 comparable to that in women under 25, underscoring the substantial burden of T1D among older women. While Yang et al.’s study on adults over 65 years from 1990 to 2019 indicated that improved survival into older age was a major contributor to the increasing T1D prevalence in older adults [15], our study further suggests that the rising incidence is another crucial factor that should not be overlooked. Several primary studies have reported a T1D incidence peak in older adults of a similar size to or exceeding that in children [24], but more real-world data remain warranted to confirm this pattern and its drivers. Evidence from individual participant data suggested that female T1D patients exhibited an estimated 13-year loss in life expectancy compared to the general population, which was two years more than the reduction observed in males [25], further highlighting the need for improved surveillance and in-time intervention for newly-onset T1D in women. Notably, T1D incidence in older adults may be underestimated, as autoantibody levels can wane over time and even be absent in older individuals who are being evaluated for T1D many years after diabetes onset [26]. Additionally, emerging evidence suggests that immune aging may relate to autoimmunity [27, 28], aligning with our finding of rising T1D incidence rates with advancing age among women over 60 years. In this age group, we observed a consistent trend of higher T1D incidence in women than in men, either in absolute number or rate. While hormonal changes during menopause (e.g., fluctuations in oestrogen, progesterone, testosterone and GnRH) have been hypothesized to influence autoimmune regulations and β-cell function [29], the underlying mechanisms remain unclear. Further research is required to better understand these sex disparities in T1D onset at older ages. As populations age, it is urgent for medical, research, and public health communities to turn their attention to T1D in older women and prioritize healthcare resources to address the growing and complex needs of this population.

### A faster rise in T2D burden among younger populations: a growing concern for women

Different from T1D, we found that T2D incidence rate in women exhibited a reverse U-shaped pattern with age, peaking at 55–60 years — about 5 years earlier than in men. We also identified an approximately linear relationship between age and the change in incidence rates from 1990 to 2021, showing a faster accelerating growth in incidence for younger women. This is further mirrored in our observations of the more rapid increase in the T2D DALY rate as age decreased. These findings underscore the need for more attention and research on the distinct shift in T2D incidence toward younger women, amid the growing global focus on early-onset T2D [12, 30]. Early-onset T2D has been linked to faster deterioration in β-cell function, more aggressive progression to microvascular complications, and higher risks of cardiovascular outcomes and mortality compared to later-onset T2D [30], which may be more pronounced in women [31]. Recent evidence from 1.5 million participants indicated that every decade of earlier diagnosis of diabetes was associated with about four years of lower life expectancy, with the reduction in women being two years greater than in age-matched men [31]. Furthermore, diabetic women are less likely than men to receive appropriate treatments and cardiovascular risk management [4, 5], potentially leading to greater cumulative lifetime exposure to hyperglycemia and greater risks of serious complications and mortality [5, 32]. Beyond these risks, T2D poses unique reproductive health challenges for women, including delayed puberty and menarche, menstrual cycle abnormalities, subfertility, adverse pregnancy outcomes, and early menopause [33]. Given its untreatable nature, the sharp rise in T2D incidence among younger women suggests that more will encounter diabetes-related reproductive health problems throughout their lives. This emphasizes the need for greater awareness and preparation to manage these challenges across their lifespan.

### Regional variations in diabetes burden

Health outcomes and disease burden are closely associated with socioeconomic development [16]. Our analyses found the highest age-standardized DALY and mortality of T1D and T2D among women at Low and Low-middle SDI levels from 1990 to 2021. Additionally, a lower SDI was associated with a higher proportional fatal burden of T1D and T2D— a trend projected to persist through 2050— highlighting the urgent need to enhance diabetes care and resource allocation for women in lower SDI regions. On one hand, recent evidence indicated that lower income regions suffered from narrower diabetic treatment coverage [34]. Despite expansion in treatment access, progress has either stagnated or lagged behind the rising T2D prevalence in women over the past three decades [34]. These findings supported our results and emphasized the necessity to improve treatment accessibility. On the other hand, it is crucial to enhance adherence to treatment among women as failure to treat or delay in treatment both increases risks of diabetes-related complications and mortality. Women are more likely than men to exhibit poorer adherence to therapies, potentially due to more frequent reports of side effects, higher burdens of mental health disorders, and lower quality of life [4, 5]. This largely explains the seemingly conflicting findings in our study, where women showed higher age-standardized mortality and DALY compared to men in some regions— such as the Caribbean— despite reports of higher diabetes treatment coverage for women [34].

Interestingly, countries with High SDI exhibited an age-standardized T1D incidence in women nearly double that of other SDI categories, accompanied by the most rapid increase over time. The diagnostic gaps across SDI levels [35] may be one potential reason, but further research is required as T1D incidence appeared similar across regions ranging from Low to High-middle SDI. Our findings align with prior evidence on women which showed a greater aggregation of autoimmune diseases in more developed areas [36]. The hygiene hypothesis may help elucidate by suggesting that progressive depletion in microbes and parasites and fewer infections due to socioeconomic improvements could disrupt immunoregulatory mechanisms and increase the risk of autoimmune diseases [37]. Targeted resource allocation tailored to sociodemographic status is thus essential for addressing specific health needs effectively.

### Attributable risk factors

We identified high BMI, low physical activity, and diet high in sugar-sweetened beverages as crucial contributors to T2D burden for women. Overweight and obesity, which accounted for over 50% of T2D burden in our analysis, has long been a major driver of the disease. In some of the regions where obesity was prevalent or more increased over time in women [38]— such as countries in Oceania or North Africa and Middle East— T2D incidence was either already high or showed more notable increases as shown in our results. Obesity is up to 50% more prevalent among women than men globally [39], with teenage girls at greater risk of obesity than their male peers in nearly all countries [40]. Women appear to bear a greater obesity burden at the time of their T2D diagnosis than men [5], which may partially explain their more severe clinical outcomes. With menopause and the accompanying loss of estrogen, the redistribution of adipose tissue to the visceral adipose depots in women further warrants attention, given the lower insulin sensitivity and increased cardiometabolic risks associated with visceral fat [41, 42]. As established risk factors for obesity, high intake of sugar-sweetened beverages and low physical activity had detrimental effects on diabetes that were not solely mediated by obesity, but also potentially involved mechanisms such as insulin resistance and metabolic dysfunction [43, 44]. We observed a substantial reduction in T2D burden projected under the Improved Behavioral and Metabolic Risks scenario, reinforcing the importance of weight control and behavioural management in mitigating T2D burden for women. Additionally, environmental factors are emerging concerns in the context of diabetes burden, with high temperature showing the most rapid increase in associated DALY for T1D and T2D in women. While the underlying mechanisms require further investigation, evidence suggests that heat exposure may disrupt fasting glucose regulation, alter insulin sensitivity, and increase morbidity and mortality [45]. Women are reported to be more affected by heat waves [46]. It is crucial to incorporate climate change into the future design, planning, and implementation of diabetes prevention programs to ensure their effectiveness and sustainability.

### Strengths and limitations

Gender-related disparities in diagnosis, access to treatment, and health-seeking behavior may contribute to variations in the disease burden between men and women. Owing to a combination of socioeconomic, environmental and psychosocial factors, as well as inequities within research and healthcare systems, women with diabetes are more likely than men to be diagnosed at more advanced stages, face greater barriers to appropriate healthcare, and exhibit lower adherence to treatment [4, 5, 9, 10]. These challenges may result in gaps in understanding the diabetes burden among women, highlighting the crucial need for a gender-centered strategy in diabetes research and policy formulation. To our knowledge, this is the first study that comprehensively assesses the global, regional, and national burden of total and type-specific diabetes specifically focused on women, offering a foundation for targeted policymaking and health resource allocation. However, there are several limitations. First, data-related limitations exist since our estimates rely on modelled data inferred from available epidemiological information, which may underestimate the burden in Low SDI regions due to variability in healthcare services and reporting mechanisms. Moreover, T2D was indirectly estimated by subtracting T1D from total diabetes cases, a method that may introduce additional uncertainty especially in poorly resourced countries. Second, despite improvements, methodological assumptions inherent in the GBD 2021 estimation process may still introduce biases that affect accuracy, highlighting the need for real-world studies to verify our findings. Third, diabetes data for children under 15 years were assumed to be T1D in GBD 2021, so we did not include this age group for T2D in our analysis. Although incident T2D in those under 15 years is uncommon, further studies are needed to assess whether its exclusion could impact our main findings. Due to the paucity of population-based studies differentiating T1D from T2D in this age group across different times and locations, ongoing monitoring of relevant literature is warranted to address this concern. Additionally, we were unable to include gestational diabetes in our analysis as it was not explicitly modelled in the GBD framework, which may lead to an underestimation of the diabetes burden among women. Latent autoimmune diabetes in adults (a variant of T1D) or rare forms like monogenic diabetes were also not included due to scarce data availability.

## Conclusions

Despite marked progress in improved survival, T1D incidence among women was increasing, particularly in High SDI countries. The U-shaped age pattern of T1D incidence, featuring a potential second peak in older women, underscores a significant T1D burden in this age group that warrants further investigation using real-world data. The growing T2D burden presents another global health challenge for women, especially at Low and Low-middle SDI. The notable shift of T2D burden toward younger populations is also concerning, highlighting the urgent need for earlier prevention. Over the next three decades, the DALY of T1D and T2D are projected to continue their respective downward and upward trends, with both transitioning toward non-fatal burdens. Our study revealed significant variations in diabetes burden among women across multiple dimensions, underscoring the need for adaptable and targeted strategies to enhance early detection and effective management.

## Supplementary Information

Additional file 1: Supplementary Methods.

Additional file 2: Table S1-S6. The burden of total and type-specific diabetes among women at global, SDI, regional, and national levels, 1990-2021. Table S7-S8. Annual percentage changes estimated from joinpoint regression for type 1 and type 2 diabetes among women at global, SDI, regional, and national levels, 1990-2021. Table S9. Proportions of DALY due to YLD for type 1 and type 2 diabetes among women and men at global and SDI levels, forecasted from 2022 to 2050.

Additional file 3: Fig S1. Temporal trends in total and type-specific diabetes prevalence, YLD, and YLL among women at global and SDI level, 1990-2021. Fig S2. Comparison of the global diabetes burden across the full spectrum of diseases between women and men, 1990-2021. Fig S3-S4. Ranking of type 1 diabetes burden across super-regions, sub-regions, and High-income countries and territories, 1990-2021. Fig S5-S6. Maps showing global burden of type 1 diabetes in women and corresponding female-to-male ratios or differences, 1990-2021. Fig S7. Ranking of type 2 diabetes burden across super-regions and sub-regions, 1990-2021. Fig S8-S9. Maps showing global burden of type 2 diabetes in women and corresponding female-to-male ratios or differences, 1990-2021. Fig S10. The age-specific patterns of diabetes burden among women in 1990, 2005, and 2021, along with population structures. Fig S11-S14. Female-to-male ratios of type 1 diabetes burden by age groups, 1990-2021. Fig S15. Comparison of the age-specific type 2 diabetes incidence patterns between men and women in 1990, 2005, and 2021. Fig S16. Correlations between age and AAPCs in the burden of type 1 and type 2 diabetes among women, at global and SDI levels. Fig S17-S20. Female-to-male ratios of type 2 diabetes burden by age groups, 1990-2021. Fig S21. Contributions of detailed risk factors to diabetes DALY among women in 2021 and corresponding AAPCs from 1990 to 2021. Fig S22. Trends of contributions of specific risk factors to diabetes DALY among women at global and SDI levels, 1990-2021. Fig S23. Age-specific differences between women and men in proportional DALY attributable to specific risk factors for diabetes in 2021, globally and by SDI. Fig S24. Age-related characteristics in terms of proportional DALY attributable to specific risk factors for diabetes among women in 2021, globally and by SDI. Fig S25. Diabetes DALY, YLD, and YLL among women globally, for the past and for five future scenarios, 1990-2050. Fig S26-S27. Diabetes DALY, YLD, and YLL among women by SDI levels, for the past and for five future scenarios, 1990-2050.

## Data Availability

GBD 2021 data resources were available online from the Global Health Data Exchange at https://vizhub.healthdata.org/gbd-results and https://vizhub.healthdata.org/gbd-foresight.

## Abbreviations

AAPC: Average annual percent change
BMI: Body mass index
CI: Confidence interval
DALY: Disability-adjusted life years
FPG: Fasting plasma glucose
GBD: Global Burden of Disease
SDI: Sociodemographic index
T1D: Type 1 diabetes
T2D: Type 2 diabetes
UI: Uncertainty interval
YLD: Years lived with disability
YLL: Years of life lost
