# Supplementary material for: Global burden of diabetes in women from 1990 to 2021, with projections to 2050: population-based study
Source: BMC Med. 2025 Oct 8;23:538. doi: 10.1186/s12916-025-04361-y (PMC12506318; doi:10.1186/s12916-025-04361-y)
Supplement: Supplementary file 1 — Additional file 1: Supplementary Methods. [file 12916_2025_4361_MOESM1_ESM.pdf]

Supplementary Methods

1. Data source

The Global Burden of Disease (GBD) 2021 project estimated the global burden of 371 diseases and injuries and 88 risk factors across 204 countries and territories from 1990 to 2021 and forecasted the burden of disease scenarios from 2022 to 2050 [11, 16-18].

All data from 1990 to 2021 used in the present study were extracted from the Global Health Data Exchange at https://vizhub.healthdata.org/gbd-results, including:
(i) Global age- and sex- specific counts, rates, and proportions of incidence, prevalence, mortality, years of life lost (YLL), years lived with disability (YLD), and disability-adjusted life-years (DALY) from 1990 to 2021
(ii) Regional age- and sex- specific counts, rates, and proportions of incidence, prevalence, mortality, YLL, YLD, and DALY from 1990 to 2021
(iii) National age- and sex- specific counts, rates, and proportions of incidence, prevalence, mortality, YLL, YLD, and DALY from 1990 to 2021
(iv) Global, regional, and national age- and sex- specific population from 1990 to 2021
(v) Age- and sex- specific DALY counts and rates attributable to risk factors from 1990 to 2021, along with the contribution of each risk factor to the disease burden

All forecasted data from 2022 to 2050 used in the present study were extracted from the Global Health Data Exchange at https://vizhub.healthdata.org/gbd-foresight. These data include sex-specific counts and rates of DALY, YLL, and YLD at both global and SDI levels under a reference forecast (representing the most likely future) and four alternative scenarios that assess disease burden trajectories assuming the elimination of
selected risk factors from current levels by 2050. The four alternative future scenarios were constructed from various sets of risk factors, including:
(i) Safer Environment scenario
(ii) Improved Childhood Nutrition and Vaccination scenario
(iii) Improved Behavioural and Metabolic Risks scenario
(iv) The combined effects of these three scenarios.

2. Estimation of diabetes burden in GBD 2021

To obtain the overall burden of disease, measured as DALY, GBD 2021 started by estimating the cause-specific mortality and non-fatal health loss. DALY were calculated as the sum of YLL and YLD for each cause, location, age group, sex, and year. The estimations of non-fatal and fatal burden of diabetes were described below, with more details from publications of GBD 2021 [11, 16].

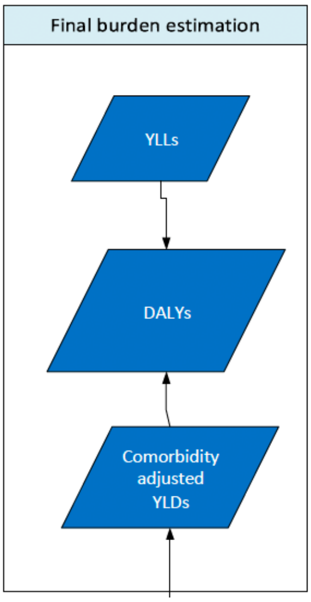

Extracted from **GBD 2021 Diseases and Injuries Collaborators, 2024 [11] (Appendix 1, page 382)**

2.1. non-fatal burdens of diabetes

In GBD 2021 [11, 16], diabetes was defined as fasting plasma glucose greater than or equal to 126 mg/dl (7 mmol/L) or current treatment (insulin or drugs). Type 1 diabetes (T1D) was defined as cases of physician diagnosed type 1 diabetes, or type 1 diabetes cases in a diabetic registry or hospital, or any case of diabetes in persons <15 years who are on insulin. Clinical, reference, and alternative case definitions and diagnostic criteria are presented in the table below.

Quantity of interest	Clinical, reference, or alternative	Definition
DM	Clinical	A metabolic disorder in which the body does not produce enough or does not respond normally to insulin, causing chronic high blood sugar (glucose) levels, which over time leads to serious damage to the heart, blood vessels, eyes, kidneys, and nerves.
DM	Reference	Fasting plasma glucose (FPG) greater than or equal to 126 mg/dl (7 mmol/l) or current treatment (insulin or anti-diabetic drugs).
DM	Alternative	FPG greater than a threshold not equal to 126 mg/dl (7mmol/L) or current treatment (insulin or anti-diabetic drugs).
DM	Alternative	Blood sugar measured using glycated haemoglobin (HbA1c) at a given threshold or current treatment (insulin or anti-diabetic drugs).
DM	Alternative	Blood sugar measured using oral glucose tolerance test (OGTT) at a given threshold or current treatment (insulin or anti-diabetic drugs).
DM	Alternative	Blood sugar measured using post-prandial glucose test (PPG) at a given threshold or current treatment (insulin or anti-diabetic drugs).
DM	Alternative	Any combination and thresholds of FPG/HbA1c/OGTT/PPG or current treatment (insulin or anti-diabetic drugs).
DM	Alternative	Any combination and thresholds of FPG/HbA1c/OGTT/PPG (no treatment).
DM	Alternative	Diabetes as reported in USA claims.
DM	Alternative	Diabetes as reported in Taiwan (province of China) claims.
DM	Alternative	Mean FPG in a representative population.
T1D	Clinical	A metabolic disorder in which the body produces little to no insulin due to autoimmune destruction of pancreatic β-cells, causing chronic high blood sugar (glucose) levels which over time leads to serious damage to the heart, blood vessels, eyes, kidneys, and nerves.
T1D	Reference	Cases of physician-diagnosed type 1 diabetes, or type 1 diabetes cases in a diabetic registry or hospital, or any case of diabetes in persons <15 years who are on insulin.
T1D	Alternative	Cases of type 1 diabetes determined by c-peptide, islet cell autoantibodies (ICA), glutamic acid decarboxylase autoantibodies (GADA).
T1D	Alternative	Cases of type 1 diabetes found using pharmacy data, diabetic camps, or another alternative data collection system that is not a registry.
T2D	Clinical	A metabolic disorder in which the body does not respond normally to insulin, causing chronic high blood sugar (glucose) levels, which over time leads to serious damage to the heart, blood vessels, eyes, kidneys, and nerves.
Neuropathy	Reference	People with diabetes mellitus who have diabetic neuropathy determined by microfilament test.
Neuropathy	Alternative	People with diabetes mellitus who have diabetic neuropathy determined by a test that is not a microfilament test.
Diabetic foot	Reference	People with diabetes mellitus who have diabetic foot (ulcer).
Amputations due to DM	Reference	People with diabetes mellitus who have a lower limb amputation.
Amputations due to DM	Alternative	People with diabetes mellitus who have a specific part of the lower limb amputated (eg, toes, feet, below ankle).
Low vision/blindness due to diabetic retinopathy	Clinical	Vision loss due to damage to the retina among persons with diabetes that is caused by damaged blood vessels that can leak blood into the retina and cause scarring.
Low vision due to diabetic retinopathy	Reference	Low vision (presenting visual acuity of <6/18 ≥3/60 in the better eye using the Snellen chart) from damage to the retina caused by damaged blood vessels due to diabetes. Presenting vision is measured using any corrective lenses currently in use.
Low vision due to diabetic retinopathy	Alternative	Low vision (presenting visual acuity of <6/18 ≥3/60 in the better eye using the Snellen chart) from damage to the retina caused by damaged blood vessels due to diabetes, as measured by Rapid Assessment of Avoidable Blindness (RAAB) surveys.
Blindness due to diabetic retinopathy	Reference	Blindness (acuity in the better eye of <3/60 or <10% visual field around central fixation point) from damage to the retina caused by damaged blood vessels that can leak blood into the retina and cause scarring. Presenting vision is measured using any corrective lenses currently in use.
Blindness due to diabetic retinopathy	Alternative	Blindness (acuity in the better eye of <3/60 or <10% visual field around central fixation point) from damage to the retina caused by damaged blood vessels that can leak blood into the retina and cause scarring as measured by Rapid Assessment of Avoidable Blindness (RAAB) surveys.

The reference and alternative case definitions are limited to those that were modelled. Briefly, data sources obtained through scientific literature, household survey data, epidemiological surveillance data, disease registry data, clinical informatics data, and others were added to DisMod-MR 2.1, a Bayesian meta-regression modelling tool, to estimate the non-fatal burden of total diabetes and T1D from 1990 to 2021. T2D is not modelled due to the inconsistent diagnostic criteria across studies; rather, it was indirectly calculated by subtracting estimated T1D from estimated total diabetes for each age, sex, location and year.

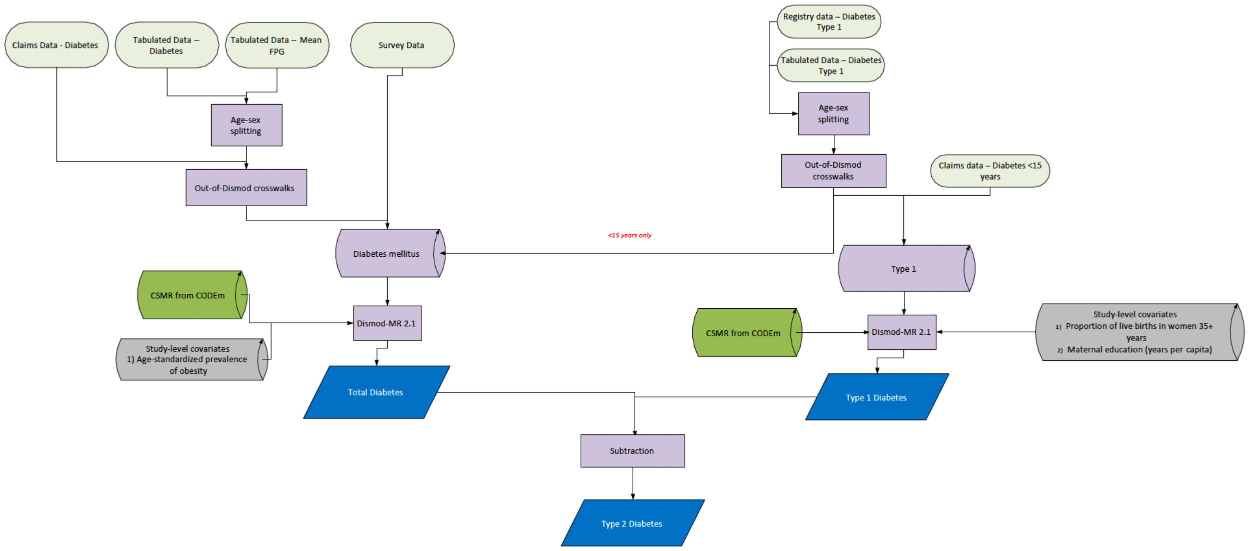

Extracted from **GBD 2021 Diseases and Injuries Collaborators, 2024 [11] (Appendix 1, page 382)**

To incorporate all available population-representative data sources to estimate the nonfatal burden of diabetes, other measures of blood glucose (glycated haemoglobin A1c, oral glucose tolerance test, post-prandial glucose test) in addition to fasting plasma glucose to define diabetes were also accepted. However, studies that used random plasma glucose to define diabetes or self-reporting of diabetes status were not accepted. For T1D, data that reported T1D, juvenile-onset diabetes, and insulin-dependent diabetes among children were all included. The estimates from alternative case definitions were then adjusted to the reference case definition in the modeling procedure. However, based on the assumption that claims data in persons <15 years are T1D and that 100% of people with diabetes are captured in this age group, no adjustments were made to data in these ages and claims data are reported as prevalence.

YLD were the product of the prevalence of each sequela and its corresponding disability weight. Estimates for four diabetic sequelae for each type of diabetes were included: neuropathy, diabetic foot, lower limb amputation, and vision loss due to retinopathy. Each sequela had separate disability weights that were used to calculate YLDs. The crude YLDs were then corrected for comorbidities with all other causes of ill health, assuming independence and a multiplicative function.

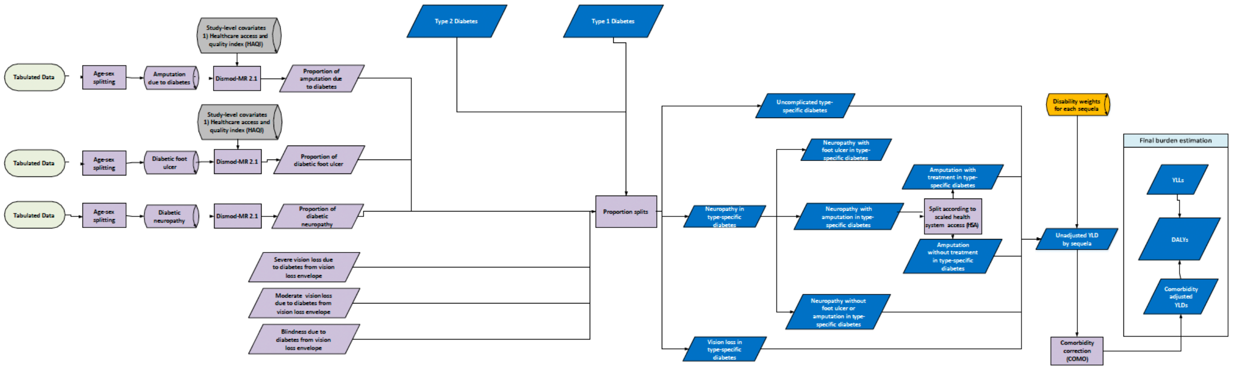

Extracted from **GBD 2021 Diseases and Injuries Collaborators, 2024 [11] (Appendix 1, page 382)**

2.2. Fatal burdens of diabetes

In GBD 2021 [11, 16], the fatal burden was estimated using the Cause of Death Ensemble model (CODEm) approach, a highly automated analytical tool that selects an ensemble of mixed-effects or spatiotemporal Gaussian regression models of mortality rates or cause fractions with varying combinations of predictive covariates. Mortality from T1D, T2D, and total diabetes was estimated by incorporating data from vital registration and verbal autopsy reports in separate models adjusted for selected covariates. Based on known or postulated relationships with development or management of diabetes, 19 covariates were selected including six associated with type 1 diabetes and 13 associated with type 2 diabetes, as shown in the below table. Notably, over 50% of deaths coded to diabetes did not specify a type. A log-linear regression model informed by data that specified the diabetes type was developed to predict the type-specific proportion of deaths among those coded to unspecified diabetes. YLL were computed by multiplying the number of estimated deaths by the standard life expectancy at the age of death.

Model	Level	Covariate	Direction
T1D	1	Healthcare Access and Quality Index	-
	2	Latitude	+
	2	Percentage of births occurring in females >35 years old	+
	2	Percentage of births occurring in females >40 years old	+
	3	Socio-demographic Index	-
	3	Education years per capita	-
T2D	1	Age-standardised mean fasting plasma glucose (mmol/L)	+
	1	Age-standardised prevalence of diabetes	+
	1	Mean BMI	+
	1	Prevalence of obesity	+
	2	Mean cholesterol	+
	2	Mean systolic blood pressure	+
	2	Age- and sex-specific summary exposure variable for low fruit	-
	2	Unadjusted grams of sugar	+
	2	Age- and sex-specific summary exposure variable for low vegetables	-
	2	Age- and sex-specific summary exposure variable for alcohol use	+
	3	Healthcare Access and Quality Index	-
	3	Education years per capita	-
	3	Lag-distributed income per capita	+

3. Estimation of diabetes burdens attributable to risk factors in GBD study 2021

The estimations of diabetes burden attributable to risk factors were described below, with more details from publications of GBD 2021 [17]. The theoretical minimum risk exposure level (TMREL) is the counterfactual level of exposure that would minimise health risk. Population attributable fraction (PAF) represents the proportion of outcomes that would decrease in a given population and time if exposed to a counterfactual level of the TMREL. Summary exposure values (SEVs) represent the age-specific risk-weighted prevalence of exposure. Attributable burden is the reduction in current disease burden that would have been possible if population-level exposure of risk factor(s) had shifted to an alternative or counterfactual distribution of risk exposure.

In GBD 2021 [17], analysis was based on the comparative risk assessment (CRA) framework established to compute risk factor estimates and included seven primary inter-related methodological components: (i) estimating effect size by quantifying the relative risk of the specified health outcome occurring as a function of exposure to the specified risk factor; (ii) collecting exposure data and estimating the levels and distribution of exposure to each risk factor, primarily using two Bayesian statistical models to pool heterogeneous data and to control and adjust for bias; (iii) determining TMREL based on epidemiological evidence; (iv) computing PAFs independently for each risk–outcome pair with estimates of exposure, RR, and the TMREL; (v) calculating SEVs for each risk; (vi) estimation of mediation of different risk factors through other risk factors to compute the burden attributable to various combinations of risk factors; and (vii) estimation of attributable burden for each combination of age group, sex, location, and year.

Risk-attributable DALY were modelled for 17 detailed factors for DM: high air temperature, low air temperature, ambient particulate matter pollution, household air pollution from solid fuels, smoking, second-hand smoke, alcohol use, diet low in fruits, diet low in vegetables, diet low in whole grains, diet high in red meat, diet high in processed meat, diet high in sugar-sweetened beverages, diet low in fibre, low physical activity, high body-mass index (BMI), and high fasting plasma glucose (FPG). All risk factors have been shown to be associated with T2D, but non-optimal temperatures are the only risk factors associated with T1D. We did not include alcohol use and high FPG in our analysis given the debatable effect of alcohol use [21] and the assumption of population attributable fraction by high FPG being 100% in GBD 2021. Definitions of the 15 included attributable risk factors are listed as follows [16]:

Risk factor	Definition
**Non-optimal temperature**	
High temperature	Defined as exposure to temperatures warmer than the TMREL
Low temperature	Defined as temperatures colder than the TMREL
**Air pollution**	
Ambient particulate matter pollution	Defined as the population-weighted annual average mass concentration of particles with an aerodynamic diameter less than 2.5 micrometers (PM2.5) in a cubic meter of air. This measurement is reported in µg/m3.
Household air pollution from solid fuels	Estimated from both the proportion of individuals using solid cooking fuels and the level of exposure to particulate matter less than 2.5 micrometres in diameter (PM2.5) air pollution for these individuals. Solid fuels in analysis include wood, coal/charcoal, dung, and agricultural residues.
**Tobacco use**	
Smoking	Current smokers were defined as individuals who currently use any smoked tobacco product on a daily or occasional basis. Former smokers were defined as individuals who quit using all smoked tobacco products for at least six months, where possible, or according to the definition used by the given survey.

Second-hand smoke	Defined as current exposure to secondhand tobacco smoke at home or at work. Only non-smokers were considered to be exposed to secondhand smoke. Non-smokers are defined as all persons who are not daily smokers. Ex-smokers and occasional smokers are considered non-smokers in analysis. Exposure is evaluated for both children and adults.
**Diet risks**	
Diet low in fruits	Average daily consumption (in grams per day) of fruit including fresh, frozen, cooked, canned, or dried fruit, excluding fruit juices and salted or pickled fruits. Optimal level or range of intake: 340–350 g/day.
Diet low in vegetables	Average daily consumption (in grams per day) of vegetables, including fresh, frozen, cooked, canned, or dried vegetables and excluding legumes and salted or pickled vegetables, juices, nuts and seeds, and starchy vegetables such as potatoes or corn. Optimal level or range of intake: 306–372 g/day.
Diet low in whole grains	Average daily consumption (in grams per day) of whole grains (bran, germ, and endosperm in their natural proportion) from breakfast cereals, bread, rice, pasta, biscuits, muffins, tortillas, pancakes, and other sources. Optimal level or range of intake: 160–210 g/day.
Diet high in red meat	Average daily consumption (in grams per day) of unprocessed red meat including pork and bovine meats such as beef, pork, lamb, and goat, but excluding all processed meats, poultry, fish, and eggs. Optimal level or range of intake: 0–200 g/day.
Diet high in processed meat	Average daily consumption (in grams per day) of meat preserved by smoking, curing, salting, or addition of chemical preservatives. Optimal level or range of intake: 0 g/day.
Diet high in sugar-sweetened beverages	Average daily consumption (in grams per day) of beverages with ⩾50 kcal per 226.8 gram serving, including carbonated beverages, sodas, energy drinks, and fruit drinks, but excluding 100% fruit and vegetable juices. Optimal level or range of intake: 0 g/day.
Diet low in fibre	Average daily consumption (in grams per day) of fibre from all sources including fruits, vegetables, grains, legumes, and pulses. Optimal level or range of intake: 22–25 g/day.
**Low physical activity**	Defined as objectively measured, total physical activity less than 3600 to 4400 MET-minutes per week.
**High body-mass index**	For adults (ages 20+): defined as BMI greater than 20 to 23 kg/m2; for children and adolescents (ages 2–19): defined as being overweight or obese based on International Obesity Task Force standards.

4. Estimation of diabetes burdens projection to 2050 in GBD study 2021

The estimations of disease forecast by 2050 were described below, with more details from publications of GBD 2021 [18]. Briefly, projections of diabetes burden from 2022 to 2050 were estimated using forecasts of key health determinants, including the Socio-demographic Index (SDI) and the comprehensive set of risk factor exposures captured by GBD. The modelling framework is multi-staged, including that (i) drivers of disease are forecasted, such as age-specific fertility rates by location, age-specific educational attainment by location, and projections of risk factor exposure by location, to obtain forecasts of cause-specific and all-cause mortality and YLLs; (ii) cause-specific forecasts for mortality–incidence ratios (MIRs) and mortality–prevalence ratios (MPRs) are combined with forecasts of mortality to produce estimates of non-fatal disease burden (i.e., YLDs) by age, sex, location, and cause; and (iii) forecasts of YLLs and YLDs are combined to produce forecasts of DALYs, and forecasts of mortality are used along with forecasts of fertility and migration to forecast population, which allows for all of the cause-specific burden measures to be aggregated to produce global estimates.

In addition to a reference forecast (the most likely future), alternative scenarios of disease burden were generated, reflecting the potential impact of policies that modify drivers of health [17-18]. Four alternative scenarios given selected sets of risk factors were eliminated were provided: (i) environmental risks (Safer Environment scenario), (ii) risks associated with communicable, maternal, neonatal, and nutritional diseases (CMNNs; Improved Childhood Nutrition and Vaccination scenario), (iii) risks associated with major non-communicable diseases (NCDs; Improved Behavioural and Metabolic Risks scenario), and (iv) the combined effects of these three scenarios. Safer Environment scenario assumes that exposure to unsafe water, unsafe sanitation, unsafe hygiene, and household air pollution will be eliminated linearly by 2050 in all locations. Improved Childhood Nutrition and Vaccination scenario assumes exposure to child growth failure (stunting, wasting, underweight), vitamin A and iron deficiency, and suboptimal breastfeeding (discontinued or non-exclusive) linearly decreases to zero by 2050 and assumes a linear increase in vaccine coverage to 100% in all locations by 2050 for the following vaccines: DTP3, MCV1, MCV2, Hib, PCV3, and Rota. Improved Behavioural and Metabolic Risks scenario assumes (i) exposure to high adult BMI, high systolic blood pressure, high LDL cholesterol, and high fasting plasma glucose are linearly eliminated by 2050 in all locations; (ii) exposure to nonoptimal diet for all GBD diet-related risk factors is likewise eliminated by 2050; ie, all dietary components included in GBD will be consumed at the level that minimises health risk for that dietary component; and (iii) a linear reduction of current tobacco smokers to zero by 2050 as well as no new smokers after 2022 in all locations. These scenarios do not factor in cost or feasibility of eliminating exposure, or analyse specific policies that could be implemented; they simply forecast potential disease burden in the coming decades if risk exposure targets were realized.

5. Data presentation, uncertainty, and socio-demographic index

GBD 2021 metrics were estimated as (i) counts (refer to ‘Number’), (ii) all-age and age-specific rates per 100 000 population (refer to ‘Rate’), (iii) age-standardised rates per 100 000 population, calculated using the GBD standard population structure (refer to ‘Rate’), and (iv) all-age and age-specific proportion of measures for a particular cause relative to those from all causes (%) (refer to ‘Percent’). Definitions of the various metrics were summarized as follows, where ‘population’ refers to the total population:

Measure	Number	Percent	Rate
Incidence	Number new of cases in the population	Proportion of news cases of a particular cause relative to cases from all causes	New cases per 100,000 population
Prevalence	Total number of cases in the population	Proportion of total cases of a particular cause relative to cases from all causes	Total cases per 100,000 population
Mortality	Number of deaths in the population	Proportion of deaths for a particular cause relative to deaths from all causes	Deaths per 100,000 population
Years of life lost (YLLs)	Number of YLLs in the population	Proportion of YLLs for a particular cause relative to YLDs for all causes	YLLs per 100,000 population
Years lived with disability (YLDs)	Number of YLDs in the population	Proportion of YLDs for a particular cause relative to YLDs for all causes	YLDs per 100,000 population
Disability adjusted life years (DALYs)	Number of DALYs in the population	Proportion of DALYs for a particular cause relative to DALYs for all causes	DALYs per 100,000 population

In GBD 2021 [11], calculations were conducted 500 times to generate draw-level estimates throughout all modelling processes. Final estimates represent the mean estimate across 500 draws, and 95% uncertainty intervals (UIs) are represented by the 2.5th and 97.5th percentile values across the draws. Uncertainty was propagated at each step in the estimation process. Different from previous GBD iterations which involved 1000 computations per process, the number of computations per process was reduced to 500 for GBD 2021 because simulation testing revealed the final estimates, and their uncertainty were not affected by this reduction.

The socio-demographic Index (SDI) is a composite indicator of background social and economic conditions that influence health outcomes in each location. It is the geometric mean of 0 to 1 indices of three parameters: the lag-distributed income per capita, average years of schooling, and the fertility rate in females younger than 25 years for a given location. For GBD 2021 after calculating SDI, values were multiplied by 100 for a scale from 0 (lowest income and years of schooling, and highest fertility) to 100 (highest income and years of schooling, and lowest fertility). The cut-off values used to determine quintiles for analysis were then computed by using country-level estimates of SDI after excluding countries with populations less than 1 million. Finally, the 204 countries and territories were divided into five SDI quintiles (low, low-middle, middle, high-middle, and high), with the higher categories indicating more socioeconomically developed. More details are in publications of GBD 2021 [11].

6. Seven super-regions and 21 sub-regions

GBD created seven super-regions based on two criteria: epidemiological similarity and geographic closeness. The seven super-regions are further sub-divided into 21 GBD regions (sub-regions). The names of the super-regions and sub-regions are summarized as follows:

Name of seven super-regions	Name of 21 sub-regions
Southeast Asia, East Asia, and Oceania	East Asia
	Oceania
	Southeast Asia
Central Europe, Eastern Europe, and Central Asia	Eastern Europe
	Central Asia
	Central Europe
High-income	Southern Latin America
	High-income North America
	Australasia
	High-income Asia Pacific
	Western Europe
Latin America and Caribbean	Tropical Latin America
	Caribbean
	Central Latin America
	Andean Latin America
North Africa and Middle East	North Africa and Middle East
South Asia	South Asia
Sub-Saharan Africa	Western Sub-Saharan Africa
	Eastern Sub-Saharan Africa
	Central Sub-Saharan Africa
	Southern Sub-Saharan Africa
