# Supplementary material for: Global burden of diabetes in women from 1990 to 2021, with projections to 2050: population-based study
Source: BMC Med. 2025 Oct 8;23:538. doi: 10.1186/s12916-025-04361-y (PMC12506318; doi:10.1186/s12916-025-04361-y)
Supplement: Supplementary file 2 — Additional file 2: Table S1-S6. The burden of total and type-specific diabetes among women at global, SDI, regional, and national levels, 1990-2021. Table S7-S8. Annual percentage changes estimated from joinpoint regression for type 1 and type 2 diabetes among women at global, SDI, regional, and national levels, 1990-2021. Table S9. Proportions of DALY due to YLD for type 1 and type 2 diabetes among women and men at global and SDI levels, forecasted from 2022 to 2050. [file 12916_2025_4361_MOESM2_ESM.pdf]

Table S1. Incidence number, age-standardized rate, and AAPC of diabetes and two subtypes among **women** globally and by different SDI, regions, countries, and territories, 1990-2021

Location	DM, Number in 1990 (000s)	DM, ASR in 1990 (per 100,000)	DM, Number in 2021 (000s)	DM, ASR in 2021 (per 100,000)	DM, AAPC (95%CI)	T1D, Number in 1990 (000s)	T1D, ASR in 1990 (per 100,000)	T1D, Number in 2021 (000s)	T1D, ASR in 2021 (per 100,000)	T1D, AAPC (95%CI)	T2D, Number in 1990 (000s)	T2D, ASR in 1990 (per 100,000)	T2D, Number in 2021 (000s)	T2D, ASR in 2021 (per 100,000)	T2D, AAPC (95%CI)
**Global**	3784 (3502 to 4083)	162 (149 to 175)	11747 (10867 to 12661)	273 (253 to 294)	1.69 (1.68 to 1.70)	164 (141 to 192)	5.89 (5.11 to 6.86)	257 (220 to 303)	6.83 (5.84 to 8.00)	0.48 (0.48 to 0.48)	3619 (3338 to 3924)	156 (143 to 169)	11491 (10626 to 12401)	266 (247 to 288)	1.73 (1.72 to 1.74)
**By SDI**															
High SDI	786 (728 to 844)	152 (141 to 164)	2213 (2059 to 2367)	303 (284 to 323)	2.23 (2.22 to 2.24)	34.9 (30.8 to 39.7)	8.92 (7.88 to 10.2)	54.4 (46.9 to 63.1)	12.4 (10.8 to 14.3)	1.07 (1.06 to 1.07)	751 (694 to 810)	144 (132 to 155)	2158 (2009 to 2315)	290 (272 to 310)	2.29 (2.28 to 2.31)
High-middle SDI	862 (793 to 943)	155 (143 to 170)	2047 (1868 to 2238)	245 (226 to 265)	1.46 (1.45 to 1.48)	25.4 (22.4 to 29.4)	4.93 (4.33 to 5.70)	37.1 (31.8 to 44.0)	6.66 (5.69 to 7.84)	0.97 (0.96 to 0.99)	837 (767 to 917)	150 (138 to 165)	2010 (1831 to 2203)	238 (219 to 258)	1.48 (1.47 to 1.50)
Middle SDI	1265 (1163 to 1376)	185 (170 to 202)	3965 (3642 to 4323)	282 (260 to 305)	1.36 (1.34 to 1.37)	45.5 (37.7 to 55.2)	4.96 (4.16 to 5.98)	65.0 (54.2 to 78.5)	5.75 (4.79 to 6.98)	0.50 (0.48 to 0.51)	1219 (1120 to 1330)	180 (165 to 197)	3900 (3578 to 4259)	276 (255 to 299)	1.38 (1.36 to 1.39)
Low-middle SDI	642 (592 to 693)	159 (146 to 172)	2598 (2399 to 2801)	290 (269 to 312)	1.96 (1.95 to 1.97)	41.1 (34.2 to 50.2)	6.20 (5.29 to 7.39)	64.1 (53.6 to 76.9)	6.55 (5.56 to 7.79)	0.18 (0.17 to 0.18)	601 (553 to 652)	153 (140 to 166)	2534 (2338 to 2734)	284 (262 to 306)	2.02 (2.01 to 2.03)
Low SDI	224 (207 to 243)	145 (134 to 157)	911 (842 to 987)	231 (214 to 250)	1.51 (1.50 to 1.51)	17.2 (14.6 to 20.4)	5.50 (4.80 to 6.34)	35.8 (30.3 to 42.8)	5.49 (4.75 to 6.38)	-0.00 (-0.01 to 0.00)	207 (190 to 225)	140 (129 to 152)	876 (804 to 949)	226 (209 to 244)	1.55 (1.54 to 1.56)
**Four world regions**															
Africa	291 (271 to 314)	154 (143 to 166)	1351 (1254 to 1446)	278 (259 to 297)	1.93 (1.92 to 1.93)	21.0 (18.1 to 24.4)	5.61 (4.97 to 6.35)	42.6 (36.4 to 50.2)	5.49 (4.80 to 6.30)	-0.07 (-0.08 to -0.07)	270 (250 to 293)	148 (137 to 160)	1308 (1212 to 1407)	272 (253 to 292)	1.98 (1.98 to 1.99)
America	685 (635 to 735)	208 (192 to 224)	2264 (2113 to 2419)	363 (340 to 387)	1.81 (1.79 to 1.82)	33.0 (28.7 to 38.2)	8.85 (7.69 to 10.3)	48.1 (41.3 to 55.5)	10.1 (8.75 to 11.7)	0.43 (0.42 to 0.44)	652 (602 to 703)	200 (183 to 216)	2216 (2067 to 2375)	353 (331 to 377)	1.86 (1.84 to 1.87)
Asia	2133 (1951 to 2325)	164 (149 to 179)	6768 (6198 to 7335)	265 (244 to 287)	1.55 (1.54 to 1.56)	82.2 (68.6 to 99.7)	4.88 (4.10 to 5.85)	123 (103 to 149)	5.86 (4.94 to 7.04)	0.60 (0.59 to 0.61)	2050 (1870 to 2246)	159 (145 to 174)	6644 (6073 to 7224)	259 (238 to 281)	1.57 (1.56 to 1.59)
Europe	663 (613 to 714)	129 (120 to 139)	1340 (1231 to 1458)	230 (214 to 249)	1.88 (1.87 to 1.89)	27.6 (24.3 to 31.6)	7.33 (6.48 to 8.40)	42.3 (36.3 to 49.9)	11.5 (9.95 to 13.5)	1.45 (1.44 to 1.47)	635 (586 to 687)	122 (113 to 132)	1297 (1191 to 1415)	219 (202 to 237)	1.90 (1.89 to 1.91)
**Six WHO regions**															
African Region	221 (205 to 239)	148 (137 to 160)	911 (846 to 976)	234 (217 to 252)	1.49 (1.48 to 1.49)	16.5 (14.1 to 19.3)	5.33 (4.68 to 6.07)	34.2 (29.1 to 40.7)	5.24 (4.55 to 6.02)	-0.05 (-0.06 to -0.05)	205 (189 to 221)	143 (132 to 155)	877 (812 to 942)	229 (212 to 247)	1.54 (1.53 to 1.54)
Region of the Americas	685 (635 to 735)	208 (192 to 224)	2264 (2113 to 2419)	363 (340 to 387)	1.81 (1.79 to 1.82)	33.0 (28.7 to 38.2)	8.85 (7.69 to 10.3)	48.1 (41.3 to 55.5)	10.1 (8.75 to 11.7)	0.43 (0.42 to 0.44)	652 (602 to 703)	200 (183 to 216)	2216 (2067 to 2375)	353 (331 to 377)	1.86 (1.84 to 1.87)
South-East Asia Region	748 (687 to 814)	159 (146 to 174)	2858 (2626 to 3102)	271 (249 to 293)	1.72 (1.70 to 1.73)	45.1 (37.2 to 55.6)	6.30 (5.29 to 7.64)	65.4 (53.8 to 80.7)	6.64 (5.51 to 8.12)	0.17 (0.16 to 0.17)	703 (642 to 771)	153 (140 to 167)	2792 (2559 to 3036)	264 (243 to 287)	1.77 (1.75 to 1.78)
European Region	691 (640 to 743)	129 (120 to 139)	1458 (1345 to 1580)	233 (217 to 252)	1.93 (1.91 to 1.94)	29.6 (26.2 to 33.8)	7.27 (6.43 to 8.34)	46.1 (39.9 to 54.0)	11.3 (9.82 to 13.1)	1.40 (1.38 to 1.42)	661 (611 to 714)	122 (113 to 131)	1411 (1300 to 1533)	222 (206 to 240)	1.96 (1.94 to 1.97)
Eastern Mediterranean Region	223 (207 to 242)	188 (173 to 203)	1384 (1279 to 1491)	438 (407 to 472)	2.78 (2.77 to 2.79)	15.6 (13.0 to 18.8)	7.35 (6.35 to 8.60)	31.5 (26.4 to 37.4)	8.12 (6.91 to 9.56)	0.32 (0.32 to 0.33)	208 (192 to 226)	180 (166 to 196)	1352 (1248 to 1462)	430 (399 to 464)	2.85 (2.84 to 2.86)
Western Pacific Region	1184 (1071 to 1305)	168 (150 to 185)	2794 (2533 to 3066)	238 (220 to 258)	1.13 (1.12 to 1.16)	23.3 (19.9 to 27.8)	3.13 (2.66 to 3.71)	30.0 (25.3 to 35.9)	3.82 (3.20 to 4.64)	0.69 (0.66 to 0.72)	1161 (1049 to 1283)	165 (147 to 182)	2764 (2504 to 3037)	234 (216 to 254)	1.14 (1.12 to 1.16)
**Seven super regions**															
Southeast Asia, East Asia, and Oceania	1227 (1117 to 1348)	168 (152 to 185)	3246 (2942 to 3561)	243 (224 to 264)	1.19 (1.17 to 1.21)	34.4 (28.8 to 41.4)	4.02 (3.42 to 4.80)	44.2 (37.2 to 53.6)	4.70 (3.92 to 5.72)	0.55 (0.52 to 0.58)	1193 (1085 to 1315)	164 (148 to 181)	3202 (2899 to 3517)	239 (220 to 260)	1.20 (1.19 to 1.22)
Central Europe, Eastern Europe, and Central Asia	318 (289 to 347)	123 (113 to 134)	654 (600 to 712)	226 (209 to 244)	1.98 (1.97 to 1.99)	11.9 (10.4 to 13.9)	5.76 (5.02 to 6.70)	16.2 (14.0 to 19.0)	8.52 (7.40 to 10.0)	1.28 (1.27 to 1.29)	306 (277 to 335)	117 (107 to 128)	638 (585 to 696)	217 (200 to 235)	2.01 (2.00 to 2.02)
South Asia	595 (543 to 652)	155 (141 to 169)	2352 (2143 to 2564)	262 (239 to 285)	1.70 (1.68 to 1.72)	35.5 (28.4 to 44.4)	5.72 (4.73 to 7.00)	58.0 (47.3 to 71.9)	6.47 (5.32 to 7.92)	0.39 (0.38 to 0.40)	560 (508 to 618)	149 (136 to 164)	2294 (2084 to 2508)	255 (233 to 279)	1.73 (1.71 to 1.75)
High-income	831 (771 to 891)	152 (141 to 162)	2194 (2040 to 2352)	295 (276 to 314)	2.17 (2.16 to 2.18)	38.8 (34.3 to 44.1)	9.63 (8.55 to 10.9)	61.8 (53.0 to 71.9)	13.8 (12.0 to 15.9)	1.14 (1.12 to 1.16)	793 (731 to 851)	142 (131 to 153)	2133 (1982 to 2291)	281 (263 to 301)	2.23 (2.21 to 2.25)
Latin America and Caribbean	388 (362 to 417)	264 (244 to 284)	1168 (1079 to 1264)	347 (322 to 375)	0.89 (0.88 to 0.90)	14.5 (12.0 to 17.7)	6.42 (5.39 to 7.65)	19.3 (16.3 to 23.2)	6.85 (5.78 to 8.24)	0.24 (0.22 to 0.25)	374 (348 to 402)	258 (238 to 278)	1149 (1060 to 1245)	341 (315 to 368)	0.90 (0.89 to 0.92)
North Africa and Middle East	219 (204 to 236)	198 (184 to 213)	1325 (1225 to 1433)	457 (424 to 494)	2.74 (2.73 to 2.74)	13.3 (11.3 to 15.5)	7.07 (6.22 to 8.12)	23.5 (20.1 to 27.2)	7.67 (6.60 to 8.84)	0.27 (0.26 to 0.27)	206 (191 to 222)	191 (177 to 206)	1301 (1200 to 1411)	449 (417 to 487)	2.80 (2.79 to 2.81)
Sub-Saharan Africa	204 (189 to 221)	143 (133 to 155)	807 (747 to 869)	217 (201 to 233)	1.34 (1.34 to 1.35)	16.0 (13.7 to 18.7)	5.30 (4.66 to 6.04)	33.7 (28.7 to 39.9)	5.22 (4.53 to 6.00)	-0.05 (-0.06 to -0.05)	188 (174 to 204)	138 (128 to 150)	774 (715 to 835)	212 (196 to 228)	1.39 (1.38 to 1.39)
**21 regions**															
East Asia	916 (825 to 1016)	166 (147 to 184)	2011 (1799 to 2225)	231 (211 to 251)	1.06 (1.05 to 1.09)	12.3 (10.4 to 14.7)	2.10 (1.77 to 2.50)	15.0 (12.5 to 18.5)	2.54 (2.11 to 3.11)	0.65 (0.61 to 0.68)	904 (813 to 1004)	163 (145 to 182)	1996 (1785 to 2212)	228 (209 to 249)	1.07 (1.05 to 1.09)
Oceania	6.49 (6.06 to 6.99)	294 (277 to 314)	30.6 (28.3 to 33.0)	523 (486 to 563)	1.86 (1.84 to 1.87)	0.19 (0.17 to 0.22)	5.08 (4.54 to 5.69)	0.36 (0.31 to 0.42)	4.84 (4.26 to 5.53)	-0.16 (-0.16 to -0.15)	6.30 (5.88 to 6.80)	289 (272 to 309)	30.2 (27.9 to 32.7)	518 (482 to 558)	1.88 (1.87 to 1.90)
Southeast Asia	305 (283 to 330)	183 (170 to 198)	1205 (1111 to 1302)	315 (292 to 340)	1.77 (1.76 to 1.78)	21.9 (18.2 to 26.5)	8.70 (7.45 to 10.4)	28.9 (24.2 to 34.8)	8.58 (7.23 to 10.3)	-0.02 (-0.04 to 0.01)	283 (262 to 307)	174 (161 to 189)	1176 (1084 to 1272)	307 (283 to 331)	1.84 (1.83 to 1.85)
Central Asia	35.0 (32.3 to 38.2)	117 (108 to 128)	137 (128 to 147)	265 (248 to 284)	2.69 (2.67 to 2.71)	2.42 (2.12 to 2.77)	6.35 (5.65 to 7.24)	4.09 (3.64 to 4.62)	8.71 (7.75 to 9.84)	1.02 (1.01 to 1.02)	32.6 (29.8 to 35.6)	110 (101 to 122)	133 (124 to 143)	257 (239 to 275)	2.77 (2.75 to 2.79)
Central Europe	135 (123 to 147)	173 (159 to 188)	225 (206 to 244)	261 (240 to 283)	1.32 (1.29 to 1.34)	3.32 (2.93 to 3.80)	5.62 (4.92 to 6.45)	3.65 (3.14 to 4.29)	7.62 (6.51 to 8.93)	0.99 (0.98 to 1.00)	132 (120 to 143)	167 (153 to 182)	221 (202 to 241)	253 (232 to 275)	1.33 (1.30 to 1.35)
Eastern Europe	148 (131 to 165)	97.9 (87.6 to 109)	292 (260 to 325)	188 (168 to 209)	2.13 (2.12 to 2.14)	6.19 (5.24 to 7.45)	5.52 (4.71 to 6.66)	8.42 (7.09 to 10.3)	8.76 (7.40 to 10.6)	1.52 (1.50 to 1.54)	142 (125 to 159)	92.4 (82.0 to 103)	284 (252 to 317)	179 (160 to 199)	2.16 (2.15 to 2.17)
Australasia	12.5 (11.6 to 13.6)	111 (103 to 121)	37.4 (33.7 to 41.0)	183 (166 to 199)	1.63 (1.62 to 1.64)	0.97 (0.92 to 1.03)	10.3 (9.67 to 10.9)	2.06 (1.85 to 2.31)	15.4 (13.9 to 17.2)	1.32 (1.29 to 1.33)	11.5 (10.6 to 12.6)	101 (92.7 to 110)	35.4 (31.6 to 39.0)	168 (151 to 184)	1.66 (1.64 to 1.67)
High-income Asia Pacific	176 (159 to 192)	167 (152 to 182)	399 (364 to 436)	301 (277 to 327)	1.91 (1.89 to 1.92)	3.38 (2.83 to 4.11)	5.19 (4.27 to 6.32)	3.27 (2.74 to 3.97)	6.18 (5.02 to 7.63)	0.53 (0.51 to 0.55)	173 (156 to 189)	162 (147 to 177)	395 (361 to 433)	294 (271 to 321)	1.94 (1.92 to 1.96)
High-income North America	264 (243 to 286)	170 (157 to 185)	987 (925 to 1052)	390 (366 to 413)	2.72 (2.69 to 2.74)	17.3 (15.0 to 20.1)	13.3 (11.6 to 15.5)	27.0 (23.2 to 31.5)	16.9 (14.6 to 19.5)	0.75 (0.74 to 0.76)	246 (226 to 268)	157 (143 to 172)	960 (899 to 1025)	374 (351 to 397)	2.84 (2.82 to 2.87)
Southern Latin America	41.4 (38.8 to 45.0)	167 (156 to 181)	125 (112 to 137)	307 (276 to 335)	1.97 (1.94 to 1.98)	1.58 (1.37 to 1.81)	6.16 (5.37 to 7.06)	2.06 (1.75 to 2.37)	7.02 (5.94 to 8.20)	0.43 (0.42 to 0.44)	39.8 (37.3 to 43.3)	160 (150 to 175)	123 (110 to 135)	299 (269 to 328)	2.02 (1.98 to 2.03)
Western Europe	338 (315 to 361)	132 (123 to 141)	646 (592 to 704)	225 (208 to 243)	1.72 (1.71 to 1.74)	15.6 (13.9 to 17.6)	9.24 (8.26 to 10.4)	27.3 (23.6 to 31.9)	15.1 (13.1 to 17.4)	1.56 (1.53 to 1.59)	322 (299 to 346)	123 (114 to 132)	619 (566 to 677)	209 (193 to 229)	1.74 (1.72 to 1.75)
Andean Latin America	18.7 (17.4 to 20.3)	144 (135 to 157)	87.5 (81.6 to 93.7)	268 (251 to 288)	2.01 (2.00 to 2.02)	0.72 (0.60 to 0.83)	3.35 (2.92 to 3.80)	1.21 (1.05 to 1.40)	3.72 (3.20 to 4.30)	0.36 (0.34 to 0.38)	18.0 (16.7 to 19.6)	141 (132 to 154)	86.3 (80.4 to 92.5)	265 (247 to 284)	2.04 (2.03 to 2.05)
Caribbean	45.3 (42.8 to 48.2)	301 (284 to 321)	121 (113 to 130)	459 (428 to 494)	1.35 (1.34 to 1.36)	1.86 (1.67 to 2.06)	9.75 (8.84 to 10.8)	2.05 (1.85 to 2.30)	9.37 (8.40 to 10.5)	-0.09 (-0.11 to -0.07)	43.4 (41.0 to 46.3)	291 (275 to 312)	119 (111 to 129)	450 (418 to 485)	1.39 (1.38 to 1.40)
Central Latin America	187 (173 to 200)	317 (293 to 342)	568 (528 to 612)	405 (377 to 436)	0.80 (0.77 to 0.83)	4.86 (3.83 to 6.20)	4.77 (3.89 to 5.93)	5.37 (4.43 to 6.49)	4.46 (3.70 to 5.42)	-0.18 (-0.21 to -0.16)	182 (168 to 195)	312 (289 to 337)	563 (523 to 607)	401 (373 to 431)	0.81 (0.79 to 0.85)
Tropical Latin America	138 (126 to 151)	232 (210 to 254)	391 (353 to 431)	285 (257 to 313)	0.65 (0.64 to 0.66)	7.03 (5.63 to 8.73)	8.25 (6.72 to 10.1)	10.7 (8.81 to 13.0)	10.0 (8.21 to 12.3)	0.65 (0.63 to 0.66)	131 (119 to 143)	224 (202 to 245)	380 (342 to 420)	275 (248 to 303)	0.66 (0.64 to 0.68)
North Africa and Middle East	219 (204 to 236)	198 (184 to 213)	1325 (1225 to 1433)	457 (424 to 494)	2.74 (2.73 to 2.74)	13.3 (11.3 to 15.5)	7.07 (6.22 to 8.12)	23.5 (20.1 to 27.2)	7.67 (6.60 to 8.84)	0.27 (0.26 to 0.27)	206 (191 to 222)	191 (177 to 206)	1301 (1200 to 1411)	449 (417 to 487)	2.80 (2.79 to 2.81)
South Asia	595 (543 to 652)	155 (141 to 169)	2352 (2143 to 2564)	262 (239 to 285)	1.70 (1.68 to 1.72)	35.5 (28.4 to 44.4)	5.72 (4.73 to 7.00)	58.0 (47.3 to 71.9)	6.47 (5.32 to 7.92)	0.39 (0.38 to 0.40)	560 (508 to 618)	149 (136 to 164)	2294 (2084 to 2508)	255 (233 to 279)	1.73 (1.71 to 1.75)
Central Sub-Saharan Africa	22.2 (20.3 to 24.4)	135 (124 to 146)	102 (92.6 to 112)	215 (196 to 234)	1.52 (1.51 to 1.53)	1.30 (1.09 to 1.55)	4.06 (3.53 to 4.72)	3.23 (2.71 to 3.81)	4.21 (3.64 to 4.86)	0.12 (0.12 to 0.12)	20.9 (19.1 to 23.0)	131 (120 to 142)	98.9 (89.7 to 108)	211 (192 to 230)	1.56 (1.55 to 1.56)
Eastern Sub-Saharan Africa	64.4 (59.8 to 69.3)	125 (117 to 135)	204 (189 to 219)	153 (143 to 164)	0.63 (0.62 to 0.64)	7.29 (6.28 to 8.34)	5.34 (4.74 to 5.97)	13.3 (11.4 to 15.3)	5.09 (4.44 to 5.74)	-0.14 (-0.15 to -0.12)	57.1 (52.8 to 62.0)	120 (112 to 130)	190 (176 to 205)	148 (138 to 159)	0.66 (0.65 to 0.67)
Southern Sub-Saharan Africa	36.2 (33.2 to 39.2)	200 (183 to 218)	131 (121 to 142)	348 (323 to 377)	1.80 (1.78 to 1.83)	1.82 (1.50 to 2.26)	6.42 (5.36 to 7.74)	2.70 (2.25 to 3.26)	6.61 (5.57 to 7.96)	0.09 (0.08 to 0.10)	34.3 (31.5 to 37.4)	193 (177 to 212)	128 (118 to 140)	342 (316 to 370)	1.85 (1.82 to 1.87)
Western Sub-Saharan Africa	81.5 (75.5 to 88.0)	144 (133 to 156)	370 (342 to 401)	232 (215 to 251)	1.55 (1.53 to 1.57)	5.54 (4.61 to 6.72)	5.17 (4.41 to 6.10)	14.5 (12.0 to 17.8)	5.31 (4.51 to 6.28)	0.08 (0.05 to 0.10)	76.0 (70.0 to 82.2)	139 (128 to 151)	356 (327 to 385)	227 (210 to 246)	1.59 (1.57 to 1.61)
**204 countries and territories**															
Afghanistan	12.5 (11.2 to 13.7)	308 (278 to 338)	76.6 (69.9 to 83.5)	713 (660 to 769)	2.76 (2.74 to 2.77)	0.63 (0.53 to 0.75)	10.5 (9.20 to 12.6)	2.13 (1.73 to 2.59)	10.8 (9.14 to 12.9)	0.09 (0.05 to 0.14)	11.9 (10.6 to 13.1)	298 (268 to 328)	74.4 (67.8 to 81.5)	702 (649 to 758)	2.82 (2.81 to 2.83)
Albania	1.48 (1.33 to 1.63)	120 (107 to 133)	3.42 (3.03 to 3.88)	183 (162 to 207)	1.38 (1.36 to 1.40)	0.07 (0.06 to 0.08)	4.13 (3.55 to 4.83)	0.07 (0.06 to 0.08)	6.11 (5.11 to 7.24)	1.26 (1.25 to 1.27)	1.41 (1.26 to 1.56)	116 (103 to 129)	3.35 (2.96 to 3.82)	177 (156 to 200)	1.39 (1.37 to 1.40)
Algeria	17.8 (16.2 to 19.7)	218 (197 to 244)	108 (99.1 to 117)	494 (453 to 536)	2.67 (2.67 to 2.68)	0.85 (0.71 to 1.00)	5.64 (4.91 to 6.49)	1.42 (1.21 to 1.66)	6.50 (5.59 to 7.60)	0.47 (0.46 to 0.48)	17.0 (15.3 to 18.8)	213 (191 to 238)	107 (97.5 to 116)	488 (446 to 530)	2.71 (2.71 to 2.72)
American Samoa	0.08 (0.07 to 0.09)	449 (412 to 492)	0.22 (0.21 to 0.25)	870 (803 to 946)	2.15 (2.14 to 2.16)	1e-03 (8e-04 to 0.001)	3.66 (3.21 to 4.18)	1e-03 (8e-04 to 0.001)	4.08 (3.49 to 4.65)	0.34 (0.29 to 0.37)	0.08 (0.07 to 0.09)	446 (409 to 488)	0.22 (0.21 to 0.24)	866 (799 to 941)	2.16 (2.15 to 2.17)
Andorra	0.03 (0.02 to 0.03)	94.5 (85.3 to 104)	0.11 (0.10 to 0.12)	195 (177 to 216)	2.37 (2.36 to 2.38)	0.002 (0.002 to 0.002)	8.53 (7.09 to 10.2)	0.005 (0.004 to 0.007)	15.4 (12.8 to 18.3)	1.92 (1.90 to 1.94)	0.02 (0.02 to 0.03)	86.0 (76.9 to 95.4)	0.10 (0.09 to 0.12)	180 (161 to 200)	2.41 (2.40 to 2.42)
Angola	4.25 (3.85 to 4.67)	146 (133 to 160)	25.2 (22.8 to 27.7)	230 (208 to 250)	1.47 (1.45 to 1.48)	0.24 (0.21 to 0.29)	4.12 (3.58 to 4.77)	0.82 (0.68 to 0.98)	4.25 (3.69 to 4.92)	0.10 (0.09 to 0.10)	4.00 (3.61 to 4.44)	142 (129 to 156)	24.4 (22.0 to 26.9)	225 (205 to 246)	1.50 (1.48 to 1.52)
Antigua and Barbuda	0.10 (0.09 to 0.10)	356 (331 to 387)	0.30 (0.28 to 0.34)	523 (478 to 578)	1.22 (1.18 to 1.25)	0.004 (0.003 to 0.005)	12.2 (10.6 to 14.0)	0.004 (0.003 to 0.004)	9.88 (8.62 to 11.5)	-0.62 (-0.66 to -0.59)	0.09 (0.09 to 0.10)	344 (319 to 375)	0.30 (0.27 to 0.34)	514 (468 to 569)	1.27 (1.23 to 1.30)
Argentina	29.5 (27.5 to 32.0)	173 (161 to 187)	77.0 (68.8 to 85.0)	291 (260 to 322)	1.69 (1.67 to 1.70)	1.15 (1.00 to 1.35)	6.63 (5.76 to 7.73)	1.45 (1.22 to 1.70)	7.18 (6.07 to 8.41)	0.29 (0.27 to 0.31)	28.4 (26.4 to 30.8)	166 (155 to 181)	75.5 (67.4 to 83.7)	284 (254 to 315)	1.73 (1.72 to 1.75)
Armenia	2.92 (2.66 to 3.31)	170 (156 to 190)	5.18 (4.61 to 5.99)	256 (233 to 291)	1.31 (1.28 to 1.33)	0.13 (0.11 to 0.14)	7.14 (6.30 to 8.21)	0.11 (0.10 to 0.14)	8.64 (7.36 to 10.2)	0.65 (0.62 to 0.68)	2.79 (2.54 to 3.19)	163 (149 to 183)	5.06 (4.50 to 5.88)	248 (225 to 282)	1.33 (1.30 to 1.35)
Australia	9.81 (8.98 to 10.8)	104 (94.7 to 114)	30.5 (26.9 to 33.8)	178 (158 to 197)	1.77 (1.76 to 1.78)	0.80 (0.77 to 0.85)	10.3 (9.83 to 10.9)	1.84 (1.65 to 2.05)	16.6 (15.0 to 18.5)	1.52 (1.50 to 1.54)	9.01 (8.17 to 10.0)	93.5 (84.5 to 103)	28.6 (25.1 to 32.0)	162 (142 to 180)	1.79 (1.78 to 1.80)
Austria	4.53 (4.24 to 4.79)	79.7 (74.5 to 85.4)	8.93 (8.14 to 9.85)	146 (135 to 160)	1.98 (1.97 to 1.99)	0.26 (0.24 to 0.27)	7.54 (6.90 to 8.26)	0.51 (0.47 to 0.56)	14.3 (13.0 to 15.7)	2.08 (2.07 to 2.10)	4.28 (3.99 to 4.53)	72.2 (67.0 to 77.9)	8.42 (7.61 to 9.34)	132 (121 to 145)	1.97 (1.96 to 1.98)
Azerbaijan	3.93 (3.58 to 4.32)	119 (108 to 131)	18.4 (16.8 to 20.3)	285 (262 to 312)	2.86 (2.84 to 2.88)	0.26 (0.23 to 0.30)	6.69 (5.91 to 7.67)	0.42 (0.37 to 0.49)	9.01 (7.88 to 10.4)	0.96 (0.95 to 0.97)	3.67 (3.31 to 4.06)	112 (102 to 124)	18.0 (16.4 to 19.9)	276 (253 to 303)	2.95 (2.92 to 2.97)
Bahamas	0.33 (0.31 to 0.36)	312 (288 to 334)	1.09 (0.98 to 1.19)	457 (413 to 500)	1.22 (1.21 to 1.23)	0.01 (0.01 to 0.02)	9.87 (8.81 to 11.3)	0.01 (0.01 to 0.02)	8.22 (7.16 to 9.30)	-0.53 (-0.57 to -0.50)	0.32 (0.29 to 0.35)	302 (278 to 324)	1.07 (0.97 to 1.18)	448 (405 to 492)	1.26 (1.25 to 1.27)
Bahrain	0.49 (0.45 to 0.53)	402 (374 to 429)	4.58 (4.27 to 4.91)	769 (727 to 811)	2.10 (2.09 to 2.12)	0.02 (0.02 to 0.02)	8.11 (6.92 to 9.41)	0.05 (0.04 to 0.06)	8.57 (7.29 to 10.0)	0.19 (0.18 to 0.20)	0.47 (0.43 to 0.51)	394 (365 to 421)	4.54 (4.23 to 4.86)	760 (719 to 803)	2.14 (2.12 to 2.15)
Bangladesh	55.4 (51.2 to 59.9)	166 (156 to 177)	264 (245 to 287)	319 (297 to 345)	2.12 (2.09 to 2.14)	5.24 (4.38 to 6.11)	7.33 (6.33 to 8.45)	6.42 (5.40 to 7.44)	7.97 (6.80 to 9.19)	0.30 (0.28 to 0.31)	50.2 (46.3 to 54.5)	159 (149 to 170)	258 (239 to 281)	311 (289 to 336)	2.19 (2.16 to 2.21)
Barbados	0.52 (0.50 to 0.56)	383 (360 to 415)	1.11 (1.04 to 1.18)	509 (478 to 546)	0.92 (0.87 to 0.95)	0.01 (0.01 to 0.02)	12.4 (10.6 to 14.0)	0.01 (0.01 to 0.01)	10.3 (8.88 to 12.0)	-0.60 (-0.62 to -0.58)	0.51 (0.48 to 0.54)	370 (347 to 403)	1.09 (1.03 to 1.17)	499 (468 to 536)	0.96 (0.91 to 1.00)
Belarus	5.62 (5.00 to 6.31)	81.9 (74.2 to 91.6)	9.04 (7.86 to 10.2)	129 (115 to 143)	1.46 (1.43 to 1.48)	0.21 (0.18 to 0.25)	4.22 (3.62 to 4.96)	0.24 (0.20 to 0.30)	5.82 (4.83 to 7.15)	1.05 (1.03 to 1.08)	5.41 (4.78 to 6.09)	77.7 (69.8 to 87.1)	8.80 (7.61 to 9.95)	123 (109 to 137)	1.48 (1.45 to 1.50)
Belgium	8.05 (7.36 to 8.78)	121 (110 to 132)	15.4 (13.6 to 17.4)	208 (186 to 233)	1.75 (1.73 to 1.77)	0.32 (0.28 to 0.38)	7.56 (6.44 to 8.97)	0.53 (0.44 to 0.65)	11.3 (9.34 to 13.8)	1.29 (1.29 to 1.30)	7.73 (7.04 to 8.44)	114 (102 to 125)	14.9 (13.0 to 16.9)	197 (174 to 221)	1.77 (1.76 to 1.79)
Belize	0.17 (0.16 to 0.18)	305 (283 to 329)	0.95 (0.86 to 1.05)	473 (430 to 520)	1.41 (1.38 to 1.43)	0.01 (0.01 to 0.01)	10.00 (8.80 to 11.4)	0.02 (0.02 to 0.02)	8.35 (7.33 to 9.46)	-0.53 (-0.57 to -0.47)	0.16 (0.15 to 0.17)	295 (273 to 319)	0.93 (0.84 to 1.03)	465 (422 to 511)	1.45 (1.42 to 1.48)
Benin	2.26 (2.05 to 2.49)	162 (146 to 177)	13.7 (12.5 to 15.2)	310 (280 to 342)	2.13 (2.09 to 2.17)	0.15 (0.13 to 0.18)	5.30 (4.60 to 6.22)	0.40 (0.33 to 0.47)	5.15 (4.44 to 5.97)	-0.09 (-0.10 to -0.09)	2.11 (1.91 to 2.33)	157 (141 to 172)	13.3 (12.2 to 14.8)	305 (275 to 337)	2.18 (2.14 to 2.23)
Bermuda	0.07 (0.06 to 0.07)	190 (176 to 205)	0.13 (0.12 to 0.15)	275 (248 to 308)	1.20 (1.18 to 1.21)	0.002 (0.002 to 0.002)	6.89 (5.86 to 8.29)	0.002 (0.001 to 0.002)	6.39 (5.44 to 7.57)	-0.20 (-0.24 to -0.17)	0.06 (0.06 to 0.07)	183 (170 to 198)	0.13 (0.12 to 0.15)	269 (242 to 302)	1.24 (1.22 to 1.25)
Bhutan	0.27 (0.24 to 0.29)	138 (126 to 153)	0.85 (0.78 to 0.92)	236 (216 to 257)	1.74 (1.73 to 1.76)	0.02 (0.02 to 0.02)	4.93 (4.27 to 5.63)	0.02 (0.02 to 0.02)	5.41 (4.64 to 6.28)	0.30 (0.29 to 0.30)	0.25 (0.22 to 0.28)	133 (121 to 148)	0.83 (0.76 to 0.90)	230 (211 to 252)	1.79 (1.77 to 1.81)
Bolivia (Plurinational State of)	4.07 (3.77 to 4.36)	194 (180 to 208)	18.3 (16.8 to 19.9)	336 (307 to 366)	1.79 (1.78 to 1.80)	0.18 (0.15 to 0.22)	5.09 (4.44 to 5.85)	0.30 (0.26 to 0.35)	5.06 (4.37 to 5.82)	0.02 (0.00 to 0.04)	3.88 (3.59 to 4.18)	189 (175 to 203)	18.0 (16.5 to 19.6)	331 (302 to 361)	1.83 (1.81 to 1.84)
Bosnia and Herzegovina	4.76 (4.24 to 5.21)	185 (166 to 202)	10.1 (9.10 to 11.0)	381 (348 to 414)	2.36 (2.34 to 2.38)	0.14 (0.12 to 0.16)	6.48 (5.71 to 7.36)	0.11 (0.09 to 0.12)	8.23 (7.17 to 9.57)	0.76 (0.74 to 0.78)	4.62 (4.11 to 5.07)	179 (159 to 195)	9.98 (9.00 to 10.9)	372 (339 to 406)	2.40 (2.38 to 2.42)
Botswana	0.67 (0.62 to 0.73)	178 (165 to 194)	3.11 (2.90 to 3.34)	304 (285 to 325)	1.73 (1.71 to 1.75)	0.05 (0.05 to 0.06)	7.76 (6.81 to 8.96)	0.09 (0.08 to 0.11)	7.91 (6.97 to 9.08)	0.08 (0.06 to 0.10)	0.61 (0.56 to 0.67)	171 (157 to 186)	3.02 (2.80 to 3.24)	296 (277 to 317)	1.79 (1.77 to 1.81)
Brazil	135 (123 to 148)	233 (211 to 255)	379 (341 to 419)	283 (255 to 311)	0.62 (0.61 to 0.64)	6.90 (5.51 to 8.58)	8.31 (6.78 to 10.2)	10.4 (8.59 to 12.8)	10.2 (8.28 to 12.4)	0.66 (0.64 to 0.67)	128 (116 to 141)	224 (203 to 246)	369 (331 to 408)	273 (246 to 301)	0.62 (0.61 to 0.64)
Brunei Darussalam	0.26 (0.25 to 0.28)	367 (343 to 391)	1.49 (1.37 to 1.62)	622 (575 to 676)	1.69 (1.66 to 1.70)	0.02 (0.02 to 0.02)	12.9 (11.4 to 14.4)	0.02 (0.02 to 0.02)	11.9 (10.3 to 13.6)	-0.21 (-0.23 to -0.19)	0.25 (0.23 to 0.26)	354 (329 to 378)	1.47 (1.35 to 1.60)	610 (564 to 664)	1.74 (1.71 to 1.76)
Bulgaria	10.7 (9.40 to 12.1)	176 (158 to 198)	14.9 (13.3 to 16.5)	288 (259 to 317)	1.61 (1.60 to 1.63)	0.32 (0.28 to 0.37)	8.43 (7.20 to 9.85)	0.32 (0.27 to 0.36)	11.4 (9.46 to 13.3)	0.97 (0.95 to 0.98)	10.3 (9.07 to 11.8)	168 (150 to 189)	14.6 (13.0 to 16.2)	277 (248 to 305)	1.64 (1.62 to 1.66)
Burkina Faso	3.47 (3.23 to 3.77)	122 (113 to 132)	15.5 (14.1 to 17.0)	211 (192 to 230)	1.79 (1.78 to 1.81)	0.29 (0.24 to 0.34)	5.21 (4.53 to 6.06)	0.65 (0.54 to 0.77)	4.95 (4.22 to 5.83)	-0.17 (-0.18 to -0.16)	3.18 (2.94 to 3.47)	117 (108 to 127)	14.8 (13.5 to 16.3)	206 (188 to 225)	1.86 (1.85 to 1.87)
Burundi	2.10 (1.96 to 2.26)	128 (118 to 138)	5.76 (5.29 to 6.26)	152 (140 to 165)	0.55 (0.54 to 0.56)	0.22 (0.19 to 0.26)	5.66 (5.06 to 6.31)	0.43 (0.37 to 0.49)	5.09 (4.50 to 5.76)	-0.29 (-0.32 to -0.26)	1.87 (1.73 to 2.04)	122 (113 to 132)	5.34 (4.87 to 5.83)	147 (135 to 159)	0.59 (0.58 to 0.59)
Cabo Verde	0.22 (0.20 to 0.24)	158 (144 to 173)	0.89 (0.80 to 0.98)	325 (293 to 358)	2.36 (2.34 to 2.37)	0.009 (0.008 to 0.01)	4.43 (3.80 to 5.15)	0.01 (0.01 to 0.02)	4.71 (4.06 to 5.52)	0.19 (0.17 to 0.20)	0.21 (0.19 to 0.23)	154 (140 to 168)	0.88 (0.79 to 0.97)	320 (288 to 354)	2.40 (2.39 to 2.42)
Cambodia	4.69 (4.34 to 5.06)	140 (130 to 151)	24.8 (22.6 to 27.2)	303 (275 to 332)	2.52 (2.51 to 2.54)	0.43 (0.37 to 0.50)	7.09 (6.36 to 8.07)	0.57 (0.50 to 0.66)	6.64 (5.85 to 7.65)	-0.16 (-0.20 to -0.13)	4.26 (3.91 to 4.62)	133 (123 to 144)	24.3 (22.0 to 26.6)	296 (268 to 326)	2.62 (2.61 to 2.63)
Cameroon	4.29 (3.96 to 4.67)	141 (130 to 153)	25.5 (23.6 to 27.7)	252 (234 to 272)	1.89 (1.87 to 1.91)	0.33 (0.28 to 0.38)	5.60 (4.95 to 6.45)	0.99 (0.85 to 1.17)	5.66 (4.99 to 6.51)	0.03 (0.03 to 0.03)	3.96 (3.62 to 4.32)	136 (125 to 147)	24.5 (22.7 to 26.7)	246 (229 to 266)	1.95 (1.92 to 1.97)
Canada	19.3 (18.1 to 20.7)	123 (115 to 132)	79.3 (69.6 to 90.1)	296 (263 to 331)	2.87 (2.85 to 2.88)	2.79 (2.62 to 2.98)	22.4 (21.2 to 23.8)	5.88 (5.42 to 6.44)	38.2 (35.4 to 41.3)	1.73 (1.72 to 1.75)	16.5 (15.3 to 18.0)	101 (92.7 to 110)	73.4 (63.9 to 84.3)	258 (225 to 294)	3.07 (3.05 to 3.09)
Central African Republic	1.58 (1.44 to 1.75)	181 (165 to 199)	6.19 (5.60 to 6.75)	296 (268 to 323)	1.59 (1.57 to 1.62)	0.08 (0.07 to 0.09)	5.04 (4.39 to 5.81)	0.16 (0.13 to 0.18)	5.07 (4.42 to 5.86)	0.03 (0.02 to 0.04)	1.51 (1.35 to 1.67)	176 (160 to 194)	6.04 (5.45 to 6.59)	291 (263 to 318)	1.63 (1.60 to 1.65)

Location	DM, Number in 1990 (000s)	DM, ASR in 1990 (per 100,000)	DM, Number in 2021 (000s)	DM, ASR in 2021 (per 100,000)	DM, AAPC (95%CI)	T1D, Number in 1990 (000s)	T1D, ASR in 1990 (per 100,000)	T1D, Number in 2021 (000s)	T1D, ASR in 2021 (per 100,000)	T1D, AAPC (95%CI)	T2D, Number in 1990 (000s)	T2D, ASR in 1990 (per 100,000)	T2D, Number in 2021 (000s)	T2D, ASR in 2021 (per 100,000)	T2D, AAPC (95%CI)
Chad	2.51 (2.31 to 2.73)	135 (125 to 148)	11.9 (10.7 to 13.1)	251 (228 to 275)	2.02 (2.00 to 2.03)	0.18 (0.15 to 0.22)	5.17 (4.42 to 6.11)	0.53 (0.44 to 0.65)	5.16 (4.39 to 6.09)	-0.01 (-0.01 to -0.00)	2.33 (2.14 to 2.55)	130 (119 to 142)	11.3 (10.2 to 12.5)	246 (224 to 270)	2.08 (2.06 to 2.09)
Chile	9.57 (8.60 to 10.7)	160 (143 to 179)	42.4 (37.9 to 48.0)	342 (308 to 384)	2.42 (2.34 to 2.46)	0.30 (0.25 to 0.35)	4.27 (3.65 to 5.00)	0.46 (0.39 to 0.55)	5.98 (4.96 to 7.11)	1.09 (1.07 to 1.10)	9.28 (8.27 to 10.4)	156 (139 to 174)	41.9 (37.5 to 47.5)	336 (303 to 378)	2.44 (2.36 to 2.49)
China	882 (792 to 981)	165 (146 to 184)	1917 (1709 to 2127)	229 (210 to 251)	1.05 (1.03 to 1.07)	11.4 (9.60 to 13.6)	2.00 (1.69 to 2.39)	13.8 (11.5 to 17.2)	2.45 (2.03 to 3.02)	0.68 (0.64 to 0.72)	871 (781 to 970)	163 (145 to 182)	1903 (1696 to 2113)	227 (207 to 248)	1.05 (1.03 to 1.08)
Colombia	26.0 (23.8 to 28.4)	211 (193 to 231)	81.8 (74.7 to 89.2)	285 (260 to 309)	1.01 (0.91 to 1.11)	0.47 (0.39 to 0.55)	2.52 (2.14 to 2.95)	0.55 (0.46 to 0.66)	2.51 (2.10 to 3.03)	-0.01 (-0.03 to 0.00)	25.6 (23.3 to 27.9)	209 (190 to 228)	81.3 (74.1 to 88.6)	282 (258 to 307)	1.02 (0.92 to 1.12)
Comoros	0.20 (0.18 to 0.22)	150 (137 to 165)	0.67 (0.62 to 0.72)	208 (192 to 226)	1.06 (1.04 to 1.07)	0.02 (0.01 to 0.02)	5.36 (4.77 to 5.97)	0.02 (0.02 to 0.02)	5.33 (4.73 to 5.96)	-0.00 (-0.02 to 0.02)	0.18 (0.16 to 0.20)	145 (131 to 160)	0.65 (0.59 to 0.70)	203 (187 to 221)	1.10 (1.08 to 1.11)
Congo	1.22 (1.11 to 1.32)	160 (147 to 172)	5.42 (4.91 to 5.92)	256 (236 to 278)	1.53 (1.51 to 1.54)	0.08 (0.06 to 0.09)	5.59 (4.89 to 6.39)	0.16 (0.14 to 0.19)	5.56 (4.89 to 6.37)	0.00 (-0.02 to 0.03)	1.14 (1.03 to 1.24)	154 (142 to 167)	5.26 (4.75 to 5.74)	250 (230 to 272)	1.57 (1.55 to 1.59)
Cook Islands	0.04 (0.04 to 0.04)	522 (488 to 562)	0.08 (0.08 to 0.09)	806 (749 to 861)	1.40 (1.39 to 1.41)	6e-04 (5e-04 to 6e-04)	5.55 (4.81 to 6.34)	4e-04 (4e-04 to 5e-04)	5.24 (4.56 to 6.03)	-0.17 (-0.18 to -0.17)	0.04 (0.04 to 0.04)	517 (482 to 556)	0.08 (0.08 to 0.09)	801 (744 to 856)	1.41 (1.40 to 1.42)
Costa Rica	2.37 (2.14 to 2.62)	211 (190 to 235)	9.63 (8.64 to 10.8)	339 (304 to 379)	1.56 (1.50 to 1.64)	0.04 (0.03 to 0.05)	2.43 (2.06 to 2.90)	0.05 (0.04 to 0.06)	2.37 (1.95 to 2.89)	-0.07 (-0.08 to -0.06)	2.33 (2.11 to 2.58)	208 (188 to 233)	9.58 (8.59 to 10.7)	336 (302 to 377)	1.58 (1.52 to 1.65)
Croatia	5.98 (5.21 to 6.79)	174 (154 to 195)	8.77 (7.75 to 9.76)	266 (237 to 298)	1.38 (1.34 to 1.40)	0.13 (0.11 to 0.14)	5.70 (5.14 to 6.30)	0.18 (0.15 to 0.21)	10.1 (8.40 to 11.8)	1.86 (1.84 to 1.87)	5.86 (5.09 to 6.66)	168 (148 to 189)	8.59 (7.58 to 9.57)	256 (227 to 287)	1.36 (1.33 to 1.39)
Cuba	11.5 (10.5 to 12.7)	212 (195 to 235)	23.7 (20.4 to 26.3)	301 (269 to 332)	1.11 (1.07 to 1.14)	0.23 (0.20 to 0.27)	5.01 (4.29 to 5.90)	0.16 (0.13 to 0.18)	3.70 (3.07 to 4.46)	-0.91 (-0.96 to -0.85)	11.3 (10.3 to 12.4)	207 (190 to 230)	23.6 (20.3 to 26.2)	298 (266 to 328)	1.14 (1.11 to 1.17)
Cyprus	1.37 (1.30 to 1.45)	298 (281 to 318)	2.61 (2.41 to 2.85)	294 (271 to 319)	-0.05 (-0.08 to -0.04)	0.03 (0.03 to 0.03)	8.07 (7.40 to 8.82)	0.11 (0.10 to 0.12)	19.4 (17.5 to 21.7)	2.89 (2.85 to 2.93)	1.35 (1.27 to 1.42)	290 (273 to 309)	2.50 (2.30 to 2.74)	274 (252 to 300)	-0.18 (-0.20 to -0.16)
Czechia	11.2 (10.1 to 12.7)	166 (150 to 186)	22.6 (19.8 to 25.2)	285 (251 to 317)	1.75 (1.71 to 1.77)	0.30 (0.26 to 0.35)	6.25 (5.36 to 7.27)	0.44 (0.37 to 0.52)	9.20 (7.80 to 10.9)	1.24 (1.22 to 1.27)	10.9 (9.82 to 12.4)	160 (144 to 179)	22.1 (19.3 to 24.8)	276 (242 to 308)	1.77 (1.73 to 1.79)
Côte d'Ivoire	4.54 (4.12 to 5.01)	150 (137 to 163)	23.7 (21.3 to 25.8)	274 (250 to 300)	1.96 (1.95 to 1.98)	0.36 (0.30 to 0.43)	5.34 (4.61 to 6.16)	0.81 (0.69 to 0.97)	5.47 (4.71 to 6.47)	0.07 (0.06 to 0.08)	4.18 (3.76 to 4.61)	145 (132 to 158)	22.9 (20.5 to 25.0)	268 (245 to 294)	2.01 (2.00 to 2.03)
Democratic People's Republic of Korea	14.8 (13.3 to 16.7)	133 (119 to 148)	40.2 (36.1 to 45.2)	242 (221 to 273)	1.96 (1.95 to 1.98)	0.29 (0.25 to 0.34)	2.75 (2.37 to 3.18)	0.33 (0.28 to 0.39)	3.04 (2.60 to 3.57)	0.31 (0.31 to 0.32)	14.5 (13.0 to 16.4)	130 (116 to 145)	39.9 (35.8 to 44.9)	239 (218 to 270)	1.99 (1.98 to 2.00)
Democratic Republic of the Congo	14.4 (13.2 to 15.9)	125 (115 to 137)	61.9 (55.5 to 68.4)	199 (179 to 219)	1.50 (1.49 to 1.51)	0.86 (0.72 to 1.03)	3.85 (3.33 to 4.52)	2.01 (1.69 to 2.38)	4.03 (3.44 to 4.73)	0.15 (0.14 to 0.15)	13.5 (12.3 to 15.0)	122 (111 to 133)	59.9 (53.6 to 66.4)	195 (175 to 216)	1.54 (1.53 to 1.54)
Denmark	2.64 (2.46 to 2.84)	81.4 (75.8 to 87.7)	6.51 (5.82 to 7.33)	171 (154 to 190)	2.41 (2.40 to 2.42)	0.22 (0.19 to 0.25)	10.0 (8.68 to 11.7)	0.33 (0.28 to 0.40)	13.9 (11.7 to 16.6)	1.09 (1.07 to 1.13)	2.43 (2.24 to 2.62)	71.4 (65.6 to 77.8)	6.18 (5.49 to 6.98)	157 (140 to 176)	2.56 (2.54 to 2.57)
Djibouti	0.11 (0.10 to 0.12)	109 (100 to 119)	0.68 (0.62 to 0.74)	161 (147 to 174)	1.25 (1.24 to 1.26)	0.01 (0.01 to 0.02)	5.16 (4.52 to 5.81)	0.03 (0.03 to 0.04)	4.92 (4.27 to 5.61)	-0.12 (-0.14 to -0.09)	0.09 (0.08 to 0.10)	104 (94.9 to 114)	0.65 (0.59 to 0.71)	156 (142 to 169)	1.31 (1.29 to 1.32)
Dominica	0.12 (0.12 to 0.13)	381 (356 to 412)	0.21 (0.19 to 0.24)	550 (498 to 601)	1.17 (1.15 to 1.19)	0.004 (0.004 to 0.005)	10.5 (9.29 to 12.0)	0.003 (0.002 to 0.003)	8.82 (7.67 to 10.1)	-0.50 (-0.55 to -0.44)	0.12 (0.11 to 0.13)	371 (346 to 401)	0.21 (0.19 to 0.23)	541 (489 to 592)	1.22 (1.20 to 1.23)
Dominican Republic	5.81 (5.26 to 6.45)	225 (203 to 249)	23.0 (20.9 to 25.3)	417 (378 to 459)	1.99 (1.96 to 2.02)	0.30 (0.25 to 0.36)	7.12 (6.05 to 8.29)	0.38 (0.33 to 0.44)	7.12 (6.16 to 8.30)	-0.01 (-0.02 to 0.00)	5.51 (4.97 to 6.16)	218 (196 to 243)	22.6 (20.4 to 24.9)	409 (370 to 452)	2.04 (2.01 to 2.07)
Ecuador	6.16 (5.67 to 6.77)	185 (169 to 204)	33.8 (30.8 to 36.8)	379 (345 to 414)	2.36 (2.35 to 2.36)	0.20 (0.16 to 0.23)	3.47 (3.00 to 3.97)	0.34 (0.29 to 0.39)	3.73 (3.20 to 4.36)	0.26 (0.23 to 0.28)	5.96 (5.48 to 6.56)	181 (166 to 200)	33.5 (30.5 to 36.4)	376 (342 to 410)	2.39 (2.38 to 2.39)
Egypt	25.3 (23.1 to 27.5)	134 (123 to 145)	188 (170 to 209)	435 (395 to 480)	3.87 (3.86 to 3.89)	2.17 (1.85 to 2.52)	7.65 (6.69 to 8.74)	4.15 (3.47 to 4.94)	7.53 (6.46 to 8.80)	-0.08 (-0.11 to -0.05)	23.1 (20.9 to 25.3)	126 (116 to 137)	184 (166 to 205)	428 (388 to 472)	4.01 (4.00 to 4.03)
El Salvador	3.12 (2.85 to 3.45)	157 (143 to 175)	11.3 (10.2 to 12.4)	322 (292 to 354)	2.33 (2.27 to 2.39)	0.12 (0.10 to 0.14)	3.69 (3.22 to 4.18)	0.13 (0.11 to 0.15)	3.98 (3.48 to 4.55)	0.25 (0.24 to 0.27)	3.00 (2.73 to 3.34)	154 (140 to 171)	11.1 (10.1 to 12.3)	318 (288 to 350)	2.37 (2.31 to 2.42)
Equatorial Guinea	0.20 (0.18 to 0.22)	144 (132 to 156)	1.35 (1.22 to 1.48)	283 (260 to 308)	2.19 (2.17 to 2.20)	0.01 (0.009 to 0.01)	4.07 (3.56 to 4.69)	0.03 (0.03 to 0.04)	4.65 (4.03 to 5.37)	0.43 (0.42 to 0.43)	0.19 (0.17 to 0.21)	140 (128 to 152)	1.31 (1.19 to 1.44)	278 (255 to 303)	2.23 (2.21 to 2.24)
Eritrea	1.17 (1.08 to 1.28)	132 (122 to 143)	4.50 (4.13 to 4.86)	196 (181 to 211)	1.27 (1.26 to 1.28)	0.13 (0.11 to 0.15)	5.72 (5.03 to 6.46)	0.22 (0.19 to 0.25)	5.74 (5.07 to 6.48)	0.04 (0.02 to 0.06)	1.04 (0.95 to 1.14)	126 (116 to 137)	4.28 (3.92 to 4.65)	190 (175 to 205)	1.32 (1.30 to 1.33)
Estonia	1.07 (0.96 to 1.20)	102 (92.0 to 114)	1.82 (1.61 to 2.07)	199 (177 to 223)	2.16 (2.11 to 2.18)	0.05 (0.05 to 0.06)	7.13 (6.58 to 7.88)	0.07 (0.06 to 0.08)	10.8 (9.15 to 12.9)	1.42 (1.39 to 1.44)	1.02 (0.91 to 1.15)	95.0 (84.7 to 107)	1.76 (1.54 to 2.00)	188 (166 to 213)	2.21 (2.17 to 2.25)
Eswatini	0.47 (0.44 to 0.51)	227 (212 to 246)	1.70 (1.59 to 1.83)	411 (382 to 439)	1.91 (1.89 to 1.93)	0.04 (0.03 to 0.05)	9.18 (8.02 to 10.6)	0.06 (0.05 to 0.07)	9.70 (8.42 to 11.4)	0.18 (0.18 to 0.19)	0.43 (0.40 to 0.47)	218 (202 to 237)	1.65 (1.53 to 1.77)	401 (372 to 429)	1.97 (1.95 to 1.99)
Ethiopia	22.6 (20.7 to 24.7)	165 (151 to 182)	58.2 (52.8 to 63.4)	171 (155 to 187)	0.11 (0.10 to 0.11)	2.16 (1.88 to 2.48)	5.88 (5.16 to 6.66)	3.22 (2.77 to 3.76)	4.92 (4.28 to 5.66)	-0.49 (-0.53 to -0.45)	20.5 (18.5 to 22.5)	159 (145 to 176)	55.0 (49.6 to 60.1)	166 (151 to 182)	0.13 (0.12 to 0.13)
Fiji	1.35 (1.27 to 1.46)	514 (488 to 548)	3.75 (3.54 to 4.01)	809 (766 to 861)	1.47 (1.46 to 1.48)	0.04 (0.04 to 0.05)	9.81 (8.83 to 10.8)	0.04 (0.03 to 0.04)	8.39 (7.43 to 9.61)	-0.45 (-0.51 to -0.37)	1.31 (1.22 to 1.42)	504 (478 to 539)	3.71 (3.50 to 3.97)	800 (758 to 852)	1.50 (1.49 to 1.51)
Finland	4.86 (4.36 to 5.38)	156 (142 to 171)	9.42 (8.52 to 10.4)	279 (256 to 306)	1.90 (1.89 to 1.91)	0.52 (0.50 to 0.54)	24.2 (23.4 to 25.0)	0.78 (0.72 to 0.85)	35.7 (33.4 to 38.6)	1.30 (1.26 to 1.32)	4.34 (3.84 to 4.87)	132 (117 to 147)	8.64 (7.76 to 9.60)	244 (219 to 269)	2.01 (2.00 to 2.02)
France	32.3 (30.0 to 34.7)	87.4 (80.8 to 94.5)	60.3 (53.4 to 67.0)	139 (125 to 153)	1.52 (1.50 to 1.53)	1.54 (1.43 to 1.66)	6.09 (5.57 to 6.63)	3.09 (2.71 to 3.52)	11.3 (9.80 to 13.0)	2.03 (2.01 to 2.05)	30.7 (28.5 to 33.2)	81.4 (74.8 to 88.5)	57.2 (50.3 to 63.9)	128 (114 to 141)	1.47 (1.46 to 1.49)
Gabon	0.57 (0.52 to 0.62)	162 (149 to 175)	2.09 (1.93 to 2.29)	279 (257 to 304)	1.77 (1.76 to 1.78)	0.03 (0.02 to 0.03)	4.89 (4.31 to 5.49)	0.05 (0.04 to 0.06)	4.98 (4.34 to 5.71)	0.08 (0.06 to 0.10)	0.54 (0.50 to 0.59)	157 (144 to 170)	2.04 (1.88 to 2.25)	274 (252 to 299)	1.81 (1.80 to 1.82)
Gambia	0.34 (0.31 to 0.37)	134 (122 to 148)	1.97 (1.80 to 2.16)	261 (239 to 286)	2.16 (2.15 to 2.17)	0.03 (0.02 to 0.03)	5.17 (4.46 to 5.99)	0.07 (0.06 to 0.08)	5.38 (4.66 to 6.29)	0.12 (0.10 to 0.13)	0.31 (0.28 to 0.34)	129 (116 to 143)	1.90 (1.73 to 2.09)	255 (234 to 281)	2.22 (2.22 to 2.23)
Georgia	3.78 (3.40 to 4.21)	113 (102 to 125)	6.05 (5.38 to 6.72)	258 (231 to 288)	2.72 (2.69 to 2.76)	0.18 (0.16 to 0.21)	6.55 (5.74 to 7.59)	0.15 (0.13 to 0.18)	9.15 (8.00 to 10.7)	1.06 (1.04 to 1.09)	3.60 (3.22 to 4.03)	106 (96.0 to 118)	5.90 (5.24 to 6.57)	249 (222 to 279)	2.78 (2.74 to 2.81)
Germany	59.8 (55.4 to 63.7)	98.6 (91.6 to 106)	121 (108 to 137)	205 (187 to 228)	2.44 (2.41 to 2.48)	2.32 (2.12 to 2.53)	7.49 (6.78 to 8.24)	4.26 (3.55 to 5.02)	13.4 (11.2 to 15.7)	1.89 (1.86 to 1.91)	57.4 (53.1 to 61.5)	91.1 (83.9 to 98.5)	117 (104 to 133)	192 (173 to 215)	2.48 (2.45 to 2.52)
Ghana	6.62 (6.04 to 7.23)	145 (133 to 157)	33.4 (30.1 to 36.7)	251 (227 to 275)	1.78 (1.75 to 1.80)	0.41 (0.36 to 0.48)	4.91 (4.35 to 5.59)	0.94 (0.82 to 1.09)	5.04 (4.45 to 5.80)	0.08 (0.06 to 0.09)	6.20 (5.66 to 6.79)	140 (128 to 152)	32.4 (29.2 to 35.8)	246 (222 to 270)	1.82 (1.80 to 1.85)
Greece	9.09 (7.93 to 10.1)	135 (121 to 150)	13.1 (11.5 to 14.6)	201 (181 to 224)	1.26 (1.22 to 1.29)	0.22 (0.19 to 0.25)	5.06 (4.40 to 5.80)	0.42 (0.35 to 0.52)	10.4 (8.63 to 12.5)	2.33 (2.31 to 2.35)	8.87 (7.72 to 9.92)	130 (116 to 145)	12.6 (11.0 to 14.2)	190 (171 to 213)	1.23 (1.19 to 1.26)
Greenland	0.01 (0.01 to 0.02)	68.4 (63.2 to 73.5)	0.06 (0.05 to 0.06)	161 (147 to 179)	2.82 (2.80 to 2.83)	0.003 (0.002 to 0.003)	10.4 (8.72 to 12.4)	0.003 (0.002 to 0.003)	11.6 (9.70 to 13.8)	0.37 (0.36 to 0.37)	0.01 (0.01 to 0.01)	58.0 (52.7 to 63.2)	0.05 (0.05 to 0.06)	150 (135 to 168)	3.11 (3.08 to 3.12)
Grenada	0.13 (0.13 to 0.14)	388 (359 to 416)	0.34 (0.31 to 0.37)	572 (525 to 620)	1.24 (1.21 to 1.26)	0.005 (0.004 to 0.006)	9.44 (8.28 to 10.9)	0.003 (0.003 to 0.004)	7.62 (6.47 to 8.72)	-0.67 (-0.69 to -0.64)	0.13 (0.12 to 0.14)	378 (350 to 407)	0.33 (0.30 to 0.36)	565 (517 to 613)	1.28 (1.25 to 1.30)
Guam	0.13 (0.12 to 0.14)	248 (229 to 269)	0.32 (0.29 to 0.35)	361 (326 to 398)	1.21 (1.21 to 1.22)	0.002 (0.002 to 0.002)	3.10 (2.61 to 3.66)	0.002 (0.002 to 0.003)	3.23 (2.71 to 3.89)	0.14 (0.11 to 0.15)	0.13 (0.12 to 0.14)	245 (226 to 266)	0.32 (0.28 to 0.35)	358 (323 to 395)	1.22 (1.22 to 1.22)
Guatemala	5.12 (4.72 to 5.59)	195 (180 to 214)	33.9 (31.2 to 36.9)	464 (428 to 506)	2.88 (2.84 to 2.93)	0.32 (0.26 to 0.39)	5.41 (4.62 to 6.33)	0.73 (0.64 to 0.84)	8.25 (7.21 to 9.37)	1.33 (1.23 to 1.39)	4.80 (4.42 to 5.27)	189 (174 to 208)	33.2 (30.5 to 36.2)	456 (420 to 498)	2.91 (2.87 to 2.96)
Guinea	2.77 (2.56 to 3.00)	139 (129 to 150)	9.92 (9.17 to 10.8)	234 (216 to 251)	1.69 (1.68 to 1.71)	0.20 (0.17 to 0.24)	5.98 (5.16 to 6.94)	0.46 (0.38 to 0.55)	5.87 (5.05 to 6.85)	-0.06 (-0.07 to -0.06)	2.56 (2.36 to 2.79)	133 (122 to 144)	9.47 (8.70 to 10.3)	228 (210 to 246)	1.75 (1.74 to 1.77)
Guinea-Bissau	0.51 (0.47 to 0.56)	171 (157 to 185)	2.07 (1.89 to 2.27)	306 (282 to 335)	1.90 (1.89 to 1.92)	0.04 (0.03 to 0.05)	6.79 (5.98 to 7.80)	0.08 (0.07 to 0.09)	6.71 (5.87 to 7.82)	-0.04 (-0.04 to -0.03)	0.47 (0.43 to 0.52)	165 (150 to 178)	1.99 (1.82 to 2.20)	299 (276 to 329)	1.96 (1.95 to 1.98)
Guyana	1.44 (1.33 to 1.57)	521 (483 to 569)	2.98 (2.77 to 3.23)	752 (698 to 817)	1.18 (1.17 to 1.19)	0.07 (0.06 to 0.07)	14.4 (13.1 to 15.8)	0.05 (0.04 to 0.05)	12.0 (10.7 to 13.7)	-0.53 (-0.57 to -0.46)	1.38 (1.27 to 1.50)	506 (469 to 555)	2.93 (2.73 to 3.18)	740 (686 to 804)	1.21 (1.20 to 1.23)
Haiti	10.2 (9.41 to 10.9)	445 (414 to 478)	35.7 (32.6 to 39.3)	615 (565 to 674)	1.04 (1.04 to 1.05)	0.64 (0.56 to 0.71)	15.7 (14.1 to 17.3)	0.98 (0.86 to 1.10)	13.9 (12.5 to 15.6)	-0.33 (-0.36 to -0.31)	9.55 (8.79 to 10.3)	430 (398 to 462)	34.7 (31.6 to 38.3)	601 (552 to 661)	1.08 (1.08 to 1.09)
Honduras	2.97 (2.70 to 3.29)	200 (180 to 221)	17.1 (15.5 to 19.0)	376 (341 to 421)	2.07 (1.99 to 2.11)	0.09 (0.07 to 0.11)	2.82 (2.41 to 3.31)	0.18 (0.15 to 0.21)	3.22 (2.75 to 3.78)	0.43 (0.41 to 0.44)	2.89 (2.61 to 3.20)	197 (177 to 218)	16.9 (15.3 to 18.8)	372 (338 to 417)	2.08 (2.01 to 2.13)
Hungary	12.6 (11.1 to 13.9)	173 (153 to 189)	19.5 (17.3 to 21.6)	262 (235 to 289)	1.32 (1.25 to 1.37)	0.23 (0.20 to 0.26)	4.83 (4.18 to 5.46)	0.30 (0.25 to 0.35)	7.37 (6.19 to 8.69)	1.36 (1.35 to 1.37)	12.4 (10.8 to 13.7)	168 (148 to 184)	19.2 (17.0 to 21.3)	254 (227 to 281)	1.33 (1.26 to 1.37)
Iceland	0.12 (0.11 to 0.13)	89.6 (81.2 to 99.3)	0.37 (0.34 to 0.41)	182 (164 to 201)	2.32 (2.31 to 2.33)	0.010 (0.008 to 0.01)	7.90 (6.57 to 9.58)	0.01 (0.01 to 0.02)	9.78 (7.96 to 12.0)	0.71 (0.68 to 0.73)	0.11 (0.10 to 0.12)	81.7 (73.7 to 91.3)	0.36 (0.32 to 0.40)	172 (154 to 191)	2.44 (2.43 to 2.45)
India	472 (426 to 521)	153 (138 to 168)	1719 (1551 to 1886)	244 (221 to 267)	1.50 (1.48 to 1.51)	24.8 (19.5 to 31.7)	5.25 (4.23 to 6.47)	39.5 (31.6 to 49.8)	5.88 (4.76 to 7.28)	0.35 (0.34 to 0.37)	447 (402 to 497)	147 (133 to 163)	1680 (1515 to 1846)	238 (215 to 261)	1.53 (1.51 to 1.55)
Indonesia	99.2 (90.4 to 108)	145 (132 to 160)	392 (353 to 430)	265 (240 to 291)	1.94 (1.91 to 1.97)	8.94 (7.02 to 11.5)	8.82 (7.05 to 11.3)	12.7 (10.1 to 16.3)	9.50 (7.67 to 12.1)	0.24 (0.24 to 0.25)	90.2 (81.7 to 99.4)	136 (124 to 150)	379 (341 to 417)	256 (231 to 282)	2.03 (2.00 to 2.05)
Iran (Islamic Republic of)	27.4 (24.7 to 30.2)	158 (142 to 174)	174 (155 to 194)	369 (331 to 407)	2.73 (2.70 to 2.76)	2.02 (1.59 to 2.57)	6.34 (5.16 to 7.95)	2.65 (2.15 to 3.35)	6.69 (5.39 to 8.45)	0.17 (0.17 to 0.18)	25.3 (22.7 to 28.1)	151 (135 to 168)	172 (153 to 191)	362 (325 to 401)	2.81 (2.77 to 2.84)
Iraq	19.4 (17.8 to 21.0)	344 (312 to 377)	121 (111 to 132)	680 (624 to 740)	2.23 (2.22 to 2.23)	0.71 (0.60 to 0.84)	6.33 (5.50 to 7.30)	1.42 (1.21 to 1.68)	6.50 (5.65 to 7.65)	0.12 (0.10 to 0.15)	18.7 (17.1 to 20.3)	338 (306 to 370)	120 (109 to 131)	673 (618 to 734)	2.26 (2.25 to 2.26)
Ireland	2.01 (1.79 to 2.21)	105 (93.7 to 116)	5.35 (4.84 to 5.94)	179 (163 to 197)	1.76 (1.74 to 1.77)	0.15 (0.13 to 0.17)	8.29 (7.44 to 9.30)	0.40 (0.36 to 0.44)	18.5 (16.8 to 20.5)	2.65 (2.63 to 2.66)	1.86 (1.65 to 2.06)	96.9 (85.4 to 108)	4.95 (4.45 to 5.53)	161 (145 to 178)	1.67 (1.65 to 1.69)
Israel	4.44 (4.08 to 4.77)	182 (166 to 199)	13.6 (12.6 to 14.6)	261 (239 to 282)	1.15 (1.14 to 1.17)	0.20 (0.19 to 0.21)	7.89 (7.49 to 8.32)	0.51 (0.48 to 0.53)	11.1 (10.5 to 11.6)	1.10 (1.09 to 1.12)	4.24 (3.89 to 4.57)	175 (158 to 191)	13.1 (12.1 to 14.1)	250 (228 to 271)	1.16 (1.14 to 1.17)
Italy	68.7 (61.8 to 76.7)	176 (159 to 194)	96.7 (85.5 to 111)	225 (202 to 253)	0.79 (0.76 to 0.81)	3.68 (3.01 to 4.54)	14.3 (11.7 to 17.8)	6.61 (5.47 to 8.36)	27.3 (22.4 to 33.9)	1.97 (1.80 to 2.12)	65.0 (58.0 to 73.3)	161 (145 to 179)	90.1 (78.9 to 104)	197 (174 to 226)	0.66 (0.64 to 0.68)
Jamaica	2.93 (2.77 to 3.09)	322 (302 to 342)	7.02 (6.56 to 7.60)	450 (422 to 489)	1.09 (1.06 to 1.12)	0.13 (0.11 to 0.15)	9.49 (8.23 to 11.2)	0.10 (0.08 to 0.11)	7.54 (6.50 to 8.64)	-0.65 (-0.71 to -0.59)	2.80 (2.64 to 2.96)	312 (294 to 333)	6.92 (6.46 to 7.51)	443 (414 to 481)	1.13 (1.10 to 1.16)
Japan	131 (116 to 147)	157 (140 to 175)	222 (195 to 251)	237 (213 to 264)	1.33 (1.30 to 1.35)	1.98 (1.57 to 2.49)	4.47 (3.52 to 5.70)	2.01 (1.65 to 2.49)	5.65 (4.49 to 7.14)	0.71 (0.64 to 0.74)	129 (114 to 145)	153 (136 to 171)	220 (193 to 249)	231 (208 to 259)	1.35 (1.32 to 1.37)
Jordan	4.07 (3.79 to 4.39)	427 (401 to 457)	32.7 (30.1 to 36.1)	636 (587 to 696)	1.32 (1.28 to 1.36)	0.22 (0.17 to 0.27)	9.09 (7.36 to 10.8)	0.45 (0.37 to 0.54)	7.17 (6.13 to 8.47)	-0.74 (-0.78 to -0.70)	3.85 (3.58 to 4.16)	418 (391 to 447)	32.3 (29.6 to 35.7)	628 (580 to 689)	1.35 (1.31 to 1.39)
Kazakhstan	11.4 (10.0 to 13.1)	142 (125 to 164)	32.4 (29.5 to 35.3)	303 (277 to 328)	2.48 (2.45 to 2.51)	0.49 (0.42 to 0.56)	5.63 (4.92 to 6.47)	0.65 (0.56 to 0.76)	6.91 (5.95 to 8.08)	0.70 (0.66 to 0.76)	10.9 (9.53 to 12.6)	136 (119 to 158)	31.8 (28.8 to 34.6)	296 (271 to 321)	2.54 (2.51 to 2.57)
Kenya	5.89 (5.38 to 6.44)	102 (92.6 to 113)	20.1 (18.4 to 21.9)	119 (110 to 131)	0.53 (0.52 to 0.53)	0.58 (0.49 to 0.69)	3.86 (3.34 to 4.48)	1.12 (0.95 to 1.34)	4.12 (3.54 to 4.81)	0.21 (0.19 to 0.22)	5.31 (4.81 to 5.85)	97.7 (88.7 to 109)	19.0 (17.4 to 20.8)	115 (106 to 127)	0.54 (0.53 to 0.55)
Kiribati	0.13 (0.12 to 0.14)	446 (417 to 475)	0.39 (0.37 to 0.42)	691 (648 to 735)	1.43 (1.42 to 1.43)	0.003 (0.002 to 0.003)	6.50 (5.78 to 7.26)	0.004 (0.004 to 0.005)	6.47 (5.80 to 7.30)	0.00 (-0.01 to 0.01)	0.12 (0.12 to 0.13)	440 (410 to 468)	0.39 (0.36 to 0.42)	684 (641 to 729)	1.44 (1.44 to 1.45)
Kuwait	1.58 (1.42 to 1.75)	359 (326 to 393)	17.4 (15.4 to 19.4)	675 (613 to 740)	2.10 (2.07 to 2.14)	0.06 (0.05 to 0.07)	7.26 (6.01 to 8.56)	0.15 (0.12 to 0.18)	7.46 (6.11 to 8.96)	-0.07 (-0.38 to 0.12)	1.52 (1.36 to 1.69)	352 (319 to 387)	17.2 (15.3 to 19.3)	667 (605 to 732)	2.16 (2.11 to 2.21)
Kyrgyzstan	1.68 (1.52 to 1.85)	89.3 (80.1 to 98.8)	6.35 (5.66 to 7.26)	190 (171 to 217)	2.47 (2.45 to 2.49)	0.09 (0.08 to 0.11)	3.80 (3.22 to 4.44)	0.19 (0.16 to 0.23)	5.34 (4.44 to 6.47)	1.08 (1.05 to 1.10)	1.59 (1.42 to 1.75)	85.5 (76.2 to 95.1)	6.16 (5.47 to 7.07)	185 (165 to 212)	2.52 (2.50 to 2.54)
Lao People's Democratic Republic	2.66 (2.45 to 2.88)	195 (180 to 212)	12.3 (11.2 to 13.3)	406 (369 to 442)	2.40 (2.40 to 2.40)	0.22 (0.19 to 0.25)	8.87 (7.96 to 10.1)	0.29 (0.25 to 0.33)	7.74 (6.83 to 8.91)	-0.40 (-0.44 to -0.34)	2.44 (2.25 to 2.67)	186 (171 to 203)	12.0 (10.9 to 13.0)	398 (361 to 434)	2.49 (2.49 to 2.49)
Latvia	1.74 (1.54 to 1.91)	95.6 (86.7 to 105)	2.77 (2.43 to 3.19)	201 (179 to 226)	2.43 (2.40 to 2.46)	0.05 (0.04 to 0.05)	4.04 (3.56 to 4.62)	0.05 (0.04 to 0.06)	6.25 (5.25 to 7.62)	1.44 (1.43 to 1.46)	1.69 (1.49 to 1.87)	91.6 (82.5 to 101)	2.72 (2.39 to 3.14)	195 (172 to 219)	2.47 (2.44 to 2.50)
Lebanon	3.30 (3.02 to 3.63)	252 (230 to 277)	14.0 (12.7 to 15.2)	458 (419 to 499)	1.97 (1.94 to 2.01)	0.12 (0.10 to 0.14)	7.31 (6.21 to 8.54)	0.18 (0.15 to 0.21)	6.78 (5.75 to 7.95)	-0.17 (-0.20 to -0.14)	3.18 (2.90 to 3.51)	245 (223 to 270)	13.8 (12.6 to 15.0)	451 (412 to 492)	2.02 (1.98 to 2.06)
Lesotho	0.86 (0.79 to 0.93)	144 (134 to 156)	2.54 (2.39 to 2.70)	336 (314 to 357)	2.76 (2.73 to 2.78)	0.07 (0.06 to 0.08)	7.76 (6.77 to 9.16)	0.08 (0.07 to 0.10)	9.05 (7.82 to 10.5)	0.47 (0.43 to 0.50)	0.79 (0.73 to 0.86)	137 (126 to 149)	2.46 (2.30 to 2.62)	327 (306 to 348)	2.85 (2.83 to 2.87)
Liberia	1.03 (0.95 to 1.13)	146 (134 to 160)	4.55 (4.13 to 5.03)	255 (233 to 279)	1.83 (1.81 to 1.84)	0.07 (0.06 to 0.09)	5.16 (4.46 to 5.99)	0.16 (0.13 to 0.19)	5.34 (4.65 to 6.28)	0.10 (0.07 to 0.12)	0.96 (0.88 to 1.06)	141 (129 to 155)	4.40 (3.97 to 4.88)	250 (228 to 274)	1.88 (1.86 to 1.89)
Libya	2.30 (2.09 to 2.52)	193 (174 to 214)	18.0 (16.2 to 20.1)	473 (430 to 524)	2.99 (2.94 to 3.05)	0.14 (0.12 to 0.16)	5.63 (4.91 to 6.45)	0.22 (0.18 to 0.26)	6.86 (5.81 to 8.08)	0.65 (0.63 to 0.67)	2.16 (1.94 to 2.37)	188 (168 to 209)	17.7 (16.0 to 19.9)	466 (423 to 517)	3.04 (2.99 to 3.10)
Lithuania	2.02 (1.79 to 2.27)	85.5 (76.3 to 95.5)	3.29 (2.92 to 3.74)	163 (146 to 183)	2.09 (2.05 to 2.12)	0.07 (0.07 to 0.08)	4.34 (3.94 to 4.83)	0.08 (0.07 to 0.10)	6.60 (5.61 to 7.88)	1.43 (1.38 to 1.47)	1.95 (1.72 to 2.19)	81.2 (71.9 to 91.3)	3.21 (2.85 to 3.66)	156 (140 to 176)	2.12 (2.08 to 2.16)
Luxembourg	0.29 (0.26 to 0.32)	116 (106 to 127)	0.80 (0.72 to 0.89)	199 (179 to 220)	1.76 (1.76 to 1.77)	0.01 (0.01 to 0.01)	7.62 (6.42 to 9.13)	0.03 (0.03 to 0.04)	11.9 (9.92 to 14.3)	1.47 (1.46 to 1.47)	0.28 (0.25 to 0.30)	108 (98.1 to 119)	0.76 (0.68 to 0.86)	187 (167 to 208)	1.78 (1.78 to 1.79)
Madagascar	3.70 (3.41 to 4.01)	111 (103 to 122)	13.8 (12.6 to 15.0)	150 (138 to 161)	0.96 (0.94 to 0.98)	0.39 (0.34 to 0.45)	4.86 (4.33 to 5.54)	0.78 (0.68 to 0.90)	4.63 (4.06 to 5.27)	-0.14 (-0.15 to -0.12)	3.31 (3.04 to 3.60)	107 (97.9 to 117)	13.0 (11.9 to 14.2)	145 (134 to 157)	1.00 (0.98 to 1.02)
Malawi	2.93 (2.70 to 3.15)	110 (102 to 119)	7.13 (6.62 to 7.67)	122 (114 to 132)	0.34 (0.33 to 0.35)	0.38 (0.33 to 0.44)	5.37 (4.75 to 6.00)	0.57 (0.49 to 0.66)	4.91 (4.28 to 5.62)	-0.24 (-0.27 to -0.21)	2.54 (2.34 to 2.77)	105 (96.8 to 113)	6.56 (6.06 to 7.11)	118 (109 to 127)	0.37 (0.36 to 0.39)
Malaysia	15.5 (14.2 to 16.8)	259 (238 to 280)	65.3 (59.6 to 71.5)	413 (376 to 451)	1.51 (1.50 to 1.52)	0.80 (0.69 to 0.94)	9.12 (8.17 to 10.4)	1.19 (1.05 to 1.37)	7.94 (6.98 to 9.01)	-0.39 (-0.43 to -0.33)	14.7 (13.5 to 16.0)	250 (229 to 271)	64.1 (58.4 to 70.3)	405 (367 to 443)	1.56 (1.55 to 1.57)
Maldives	0.11 (0.10 to 0.12)	203 (183 to 224)	0.58 (0.52 to 0.64)	315 (282 to 350)	1.44 (1.42 to 1.46)	0.010 (0.008 to 0.01)	8.36 (7.37 to 9.60)	0.01 (0.01 to 0.02)	7.10 (6.30 to 8.07)	-0.43 (-0.49 to -0.38)	0.10 (0.09 to 0.11)	195 (175 to 215)	0.56 (0.50 to 0.63)	308 (275 to 343)	1.50 (1.48 to 1.52)
Mali	6.00 (5.43 to 6.71)	220 (199 to 246)	28.6 (25.7 to 31.8)	399 (357 to 447)	1.94 (1.93 to 1.95)	0.26 (0.22 to 0.31)	5.42 (4.66 to 6.49)	0.68 (0.56 to 0.82)	5.02 (4.33 to 5.82)	-0.25 (-0.25 to -0.24)	5.74 (5.15 to 6.46)	215 (193 to 240)	27.9 (25.1 to 31.1)	394 (353 to 442)	1.98 (1.97 to 1.99)
Malta	0.33 (0.30 to 0.35)	150 (139 to 163)	0.84 (0.74 to 0.95)	287 (258 to 320)	2.14 (2.12 to 2.15)	0.02 (0.02 to 0.03)	14.4 (13.0 to 15.9)	0.04 (0.04 to 0.05)	24.1 (21.3 to 26.8)	1.67 (1.66 to 1.68)	0.30 (0.28 to 0.33)	135 (124 to 148)	0.80 (0.70 to 0.91)	263 (235 to 296)	2.18 (2.17 to 2.20)
Marshall Islands	0.07 (0.06 to 0.07)	480 (439 to 516)	0.25 (0.23 to 0.27)	899 (837 to 976)	2.03 (2.02 to 2.04)	0.002 (0.002 to 0.002)	6.72 (5.89 to 7.56)	0.002 (0.002 to 0.002)	6.80 (6.01 to 7.68)	0.05 (0.03 to 0.07)	0.06 (0.06 to 0.07)	473 (433 to 509)	0.25 (0.23 to 0.27)	893 (830 to 969)	2.05 (2.04 to 2.07)
Mauritania	0.89 (0.82 to 0.96)	138 (127 to 149)	3.18 (3.01 to 3.35)	213 (203 to 223)	1.40 (1.39 to 1.42)	0.06 (0.05 to 0.07)	5.47 (4.79 to 6.31)	0.13 (0.11 to 0.16)	5.49 (4.80 to 6.38)	0.01 (0.01 to 0.02)	0.82 (0.76 to 0.90)	133 (122 to 144)	3.05 (2.88 to 3.22)	208 (198 to 218)	1.45 (1.43 to 1.46)
Mauritius	1.43 (1.32 to 1.54)	312 (289 to 338)	5.73 (5.35 to 6.15)	618 (580 to 661)	2.24 (2.21 to 2.26)	0.06 (0.05 to 0.06)	9.85 (8.78 to 11.1)	0.06 (0.05 to 0.07)	10.7 (9.71 to 12.0)	0.25 (0.19 to 0.28)	1.37 (1.27 to 1.48)	302 (279 to 328)	5.67 (5.29 to 6.09)	607 (569 to 650)	2.29 (2.26 to 2.31)
Mexico	128 (118 to 139)	420 (383 to 459)	342 (312 to 374)	472 (432 to 515)	0.39 (0.37 to 0.40)	3.35 (2.55 to 4.44)	6.23 (4.84 to 8.23)	3.16 (2.43 to 4.01)	5.14 (3.97 to 6.53)	-0.56 (-0.60 to -0.51)	125 (114 to 136)	413 (376 to 452)	339 (309 to 370)	467 (427 to 509)	0.40 (0.39 to 0.42)
Micronesia (Federated States of)	0.12 (0.11 to 0.13)	361 (338 to 385)	0.34 (0.32 to 0.36)	687 (645 to 726)	2.12 (2.10 to 2.13)	0.004 (0.003 to 0.005)	6.27 (5.55 to 7.14)	0.003 (0.003 to 0.004)	6.32 (5.52 to 7.15)	0.04 (0.02 to 0.06)	0.12 (0.11 to 0.13)	354 (332 to 379)	0.34 (0.32 to 0.36)	680 (639 to 719)	2.15 (2.13 to 2.16)
Monaco	0.02 (0.02 to 0.02)	81.9 (73.7 to 89.9)	0.04 (0.04 to 0.05)	172 (155 to 193)	2.44 (2.43 to 2.45)	0.001 (1e-03 to 0.001)	9.10 (7.59 to 10.9)	0.002 (0.002 to 0.003)	14.1 (11.6 to 17.0)	1.41 (1.39 to 1.43)	0.02 (0.02 to 0.02)	72.8 (64.9 to 81.1)	0.04 (0.04 to 0.05)	158 (142 to 179)	2.54 (2.53 to 2.55)
Mongolia	0.58 (0.53 to 0.64)	76.5 (68.9 to 84.6)	2.74 (2.49 to 3.03)	160 (146 to 176)	2.42 (2.41 to 2.44)	0.05 (0.04 to 0.06)	3.80 (3.28 to 4.40)	0.09 (0.07 to 0.11)	5.14 (4.29 to 6.21)	1.02 (1.00 to 1.04)	0.54 (0.48 to 0.59)	72.7 (65.3 to 80.8)	2.65 (2.41 to 2.95)	155 (142 to 171)	2.48 (2.47 to 2.50)
Montenegro	0.70 (0.62 to 0.79)	202 (180 to 226)	1.40 (1.25 to 1.57)	318 (283 to 352)	1.46 (1.44 to 1.48)	0.03 (0.02 to 0.03)	8.49 (7.55 to 9.57)	0.03 (0.03 to 0.03)	11.5 (9.92 to 13.0)	0.98 (0.97 to 1.00)	0.68 (0.60 to 0.76)	194 (171 to 217)	1.37 (1.22 to 1.54)	306 (271 to 341)	1.48 (1.46 to 1.50)
Morocco	23.3 (21.1 to 25.8)	254 (229 to 285)	132 (120 to 144)	664 (608 to 722)	3.15 (3.14 to 3.17)	0.98 (0.83 to 1.15)	6.95 (6.04 to 7.96)	1.39 (1.20 to 1.60)	7.70 (6.67 to 8.88)	0.32 (0.32 to 0.33)	22.3 (20.2 to 24.9)	247 (222 to 278)	131 (119 to 142)	657 (600 to 714)	3.21 (3.19 to 3.22)
Mozambique	4.20 (3.90 to 4.51)	107 (99.7 to 115)	14.7 (13.5 to 16.1)	157 (144 to 169)	1.23 (1.21 to 1.25)	0.52 (0.45 to 0.60)	5.45 (4.75 to 6.12)	1.14 (0.96 to 1.32)	5.45 (4.74 to 6.20)	0.01 (-0.00 to 0.03)	3.67 (3.39 to 3.97)	101 (94.3 to 109)	13.6 (12.4 to 15.0)	151 (138 to 163)	1.29 (1.26 to 1.30)
Myanmar	39.2 (36.0 to 42.7)	258 (236 to 280)	144 (132 to 157)	475 (435 to 516)	2.00 (1.99 to 2.02)	2.71 (2.34 to 3.16)	12.2 (10.7 to 13.9)	2.96 (2.61 to 3.47)	10.3 (9.16 to 12.0)	-0.46 (-0.51 to -0.39)	36.5 (33.3 to 39.8)	246 (225 to 268)	141 (129 to 154)	465 (425 to 505)	2.09 (2.07 to 2.10)
Namibia	0.78 (0.72 to 0.85)	187 (173 to 203)	2.45 (2.23 to 2.70)	251 (229 to 275)	0.95 (0.94 to 0.96)	0.05 (0.05 to 0.06)	7.46 (6.57 to 8.58)	0.09 (0.07 to 0.10)	6.93 (6.01 to 8.06)	-0.19 (-0.23 to -0.14)	0.73 (0.67 to 0.80)	179 (165 to 195)	2.37 (2.15 to 2.62)	244 (223 to 268)	0.99 (0.97 to 1.00)
Nauru	0.01 (0.01 to 0.02)	446 (416 to 476)	0.04 (0.03 to 0.04)	733 (684 to 781)	1.61 (1.60 to 1.62)	4e-04 (3e-04 to 4e-04)	6.06 (5.17 to 7.00)	3e-04 (3e-04 to 4e-04)	5.43 (4.78 to 6.12)	-0.34 (-0.39 to -0.29)	0.01 (0.01 to 0.02)	440 (410 to 469)	0.03 (0.03 to 0.04)	728 (679 to 776)	1.63 (1.62 to 1.64)
Nepal	10.2 (9.22 to 11.3)	154 (139 to 168)	46.0 (41.9 to 50.1)	293 (266 to 319)	2.08 (2.06 to 2.11)	0.46 (0.38 to 0.55)	3.93 (3.33 to 4.56)	0.72 (0.60 to 0.85)	4.40 (3.75 to 5.19)	0.36 (0.31 to 0.38)	9.79 (8.77 to 10.8)	150 (135 to 164)	45.2 (41.2 to 49.5)	288 (262 to 315)	2.12 (2.09 to 2.15)

Location	DM, Number in 1990 (000s)	DM, ASR in 1990 (per 100,000)	DM, Number in 2021 (000s)	DM, ASR in 2021 (per 100,000)	DM, AAPC (95%CI)	T1D, Number in 1990 (000s)	T1D, ASR in 1990 (per 100,000)	T1D, Number in 2021 (000s)	T1D, ASR in 2021 (per 100,000)	T1D, AAPC (95%CI)	T2D, Number in 1990 (000s)	T2D, ASR in 1990 (per 100,000)	T2D, Number in 2021 (000s)	T2D, ASR in 2021 (per 100,000)	T2D, AAPC (95%CI)
Netherlands	11.4 (10.7 to 12.3)	119 (111 to 130)	19.0 (16.9 to 21.4)	163 (147 to 181)	1.02 (1.00 to 1.04)	0.67 (0.57 to 0.79)	10.1 (8.85 to 11.8)	1.07 (0.91 to 1.26)	14.9 (12.6 to 17.6)	1.26 (1.24 to 1.27)	10.8 (9.97 to 11.6)	109 (100 to 120)	17.9 (15.9 to 20.3)	148 (132 to 166)	1.00 (0.97 to 1.02)
New Zealand	2.69 (2.41 to 2.99)	149 (132 to 166)	6.98 (6.60 to 7.38)	209 (198 to 220)	1.11 (1.09 to 1.13)	0.17 (0.14 to 0.20)	9.97 (8.31 to 11.9)	0.22 (0.19 to 0.27)	9.58 (7.96 to 11.5)	-0.12 (-0.14 to -0.11)	2.53 (2.24 to 2.82)	139 (123 to 156)	6.76 (6.38 to 7.15)	199 (188 to 210)	1.18 (1.17 to 1.21)
Nicaragua	2.82 (2.57 to 3.11)	244 (225 to 269)	13.1 (11.9 to 14.4)	411 (373 to 451)	1.70 (1.67 to 1.74)	0.08 (0.07 to 0.10)	3.12 (2.71 to 3.56)	0.11 (0.09 to 0.12)	3.07 (2.63 to 3.56)	-0.03 (-0.05 to -0.02)	2.74 (2.49 to 3.03)	241 (222 to 265)	13.0 (11.8 to 14.3)	408 (370 to 448)	1.72 (1.69 to 1.75)
Niger	2.94 (2.67 to 3.21)	144 (130 to 156)	17.3 (15.7 to 19.1)	250 (224 to 277)	1.81 (1.80 to 1.82)	0.23 (0.19 to 0.28)	4.95 (4.17 to 5.87)	0.70 (0.58 to 0.87)	4.85 (4.11 to 5.77)	-0.08 (-0.10 to -0.06)	2.72 (2.44 to 2.99)	139 (125 to 151)	16.6 (15.0 to 18.4)	245 (219 to 272)	1.86 (1.85 to 1.87)
Nigeria	36.0 (32.8 to 39.0)	137 (125 to 150)	145 (132 to 158)	194 (177 to 211)	1.13 (1.11 to 1.16)	2.44 (1.94 to 3.13)	5.00 (4.06 to 6.31)	6.86 (5.41 to 8.83)	5.33 (4.35 to 6.67)	0.20 (0.15 to 0.23)	33.6 (30.5 to 36.7)	132 (120 to 145)	138 (125 to 151)	188 (172 to 205)	1.16 (1.14 to 1.19)
Niue	0.004 (0.004 to 0.005)	413 (385 to 444)	0.007 (0.006 to 0.007)	750 (694 to 807)	1.94 (1.93 to 1.95)	6e-05 (6e-05 to 7e-05)	5.04 (4.39 to 5.71)	4e-05 (3e-05 to 4e-05)	4.97 (4.31 to 5.66)	-0.02 (-0.04 to 0.00)	0.004 (0.004 to 0.005)	408 (379 to 439)	0.007 (0.006 to 0.007)	745 (689 to 803)	1.96 (1.95 to 1.97)
North Macedonia	2.44 (2.18 to 2.70)	227 (205 to 252)	6.31 (5.65 to 7.01)	399 (360 to 442)	1.85 (1.82 to 1.87)	0.09 (0.07 to 0.10)	8.76 (7.54 to 10.2)	0.10 (0.09 to 0.12)	10.9 (9.36 to 12.8)	0.73 (0.71 to 0.76)	2.35 (2.10 to 2.62)	218 (196 to 243)	6.21 (5.53 to 6.91)	388 (349 to 431)	1.89 (1.86 to 1.91)
Northern Mariana Islands	0.04 (0.04 to 0.05)	248 (228 to 271)	0.11 (0.10 to 0.13)	416 (375 to 456)	1.69 (1.68 to 1.69)	7e-04 (6e-04 to 9e-04)	3.41 (2.89 to 3.94)	8e-04 (6e-04 to 9e-04)	3.55 (3.01 to 4.16)	0.11 (0.09 to 0.12)	0.04 (0.04 to 0.05)	245 (224 to 268)	0.11 (0.10 to 0.13)	412 (371 to 453)	1.71 (1.70 to 1.71)
Norway	3.96 (3.57 to 4.41)	148 (134 to 164)	6.04 (5.39 to 6.78)	182 (162 to 202)	0.66 (0.63 to 0.68)	0.33 (0.29 to 0.39)	17.3 (14.8 to 20.6)	0.56 (0.48 to 0.65)	24.1 (20.5 to 28.3)	1.07 (1.07 to 1.08)	3.62 (3.23 to 4.07)	131 (116 to 147)	5.48 (4.82 to 6.19)	157 (139 to 177)	0.60 (0.57 to 0.62)
Oman	1.18 (1.08 to 1.29)	257 (237 to 278)	6.30 (5.70 to 7.04)	429 (392 to 467)	1.59 (1.54 to 1.65)	0.19 (0.16 to 0.22)	18.7 (16.5 to 21.3)	0.35 (0.29 to 0.41)	18.4 (16.1 to 21.3)	-0.03 (-0.08 to 0.01)	0.99 (0.90 to 1.09)	238 (218 to 260)	5.96 (5.35 to 6.71)	411 (374 to 449)	1.69 (1.63 to 1.75)
Pakistan	57.3 (52.3 to 63.1)	161 (147 to 178)	322 (294 to 354)	356 (327 to 390)	2.60 (2.57 to 2.64)	5.01 (3.91 to 6.50)	7.78 (6.34 to 9.75)	11.3 (8.98 to 14.3)	8.80 (7.11 to 11.0)	0.39 (0.38 to 0.40)	52.3 (47.5 to 58.2)	153 (139 to 170)	310 (282 to 343)	347 (317 to 381)	2.69 (2.65 to 2.72)
Palau	0.03 (0.03 to 0.03)	447 (417 to 483)	0.08 (0.07 to 0.08)	745 (697 to 790)	1.66 (1.65 to 1.67)	4e-04 (4e-04 to 5e-04)	5.56 (4.85 to 6.34)	4e-04 (3e-04 to 4e-04)	5.55 (4.84 to 6.27)	-0.00 (-0.01 to 0.00)	0.03 (0.03 to 0.03)	442 (411 to 477)	0.08 (0.07 to 0.08)	739 (691 to 785)	1.68 (1.67 to 1.69)
Palestine	1.51 (1.40 to 1.62)	258 (240 to 277)	8.94 (8.21 to 9.75)	482 (444 to 523)	2.05 (2.04 to 2.06)	0.10 (0.08 to 0.12)	8.60 (7.40 to 10.1)	0.25 (0.20 to 0.31)	9.32 (7.81 to 11.1)	0.26 (0.24 to 0.27)	1.41 (1.31 to 1.52)	249 (232 to 268)	8.68 (7.94 to 9.51)	472 (434 to 514)	2.10 (2.09 to 2.11)
Panama	1.83 (1.68 to 2.02)	203 (185 to 225)	7.81 (7.10 to 8.67)	352 (320 to 391)	1.81 (1.77 to 1.87)	0.05 (0.04 to 0.06)	3.92 (3.32 to 4.61)	0.09 (0.08 to 0.11)	4.41 (3.66 to 5.26)	0.37 (0.35 to 0.39)	1.78 (1.63 to 1.97)	199 (181 to 221)	7.71 (7.01 to 8.58)	347 (316 to 386)	1.83 (1.79 to 1.89)
Papua New Guinea	3.33 (3.05 to 3.64)	238 (220 to 258)	20.7 (18.8 to 22.7)	478 (437 to 523)	2.27 (2.25 to 2.29)	0.10 (0.08 to 0.11)	4.13 (3.61 to 4.75)	0.25 (0.21 to 0.29)	4.35 (3.77 to 5.03)	0.16 (0.15 to 0.16)	3.24 (2.95 to 3.55)	234 (215 to 253)	20.4 (18.5 to 22.5)	474 (433 to 519)	2.30 (2.27 to 2.32)
Paraguay	2.76 (2.52 to 3.03)	203 (184 to 223)	12.1 (11.2 to 13.2)	363 (334 to 395)	1.89 (1.87 to 1.90)	0.13 (0.11 to 0.15)	5.85 (5.07 to 6.71)	0.25 (0.21 to 0.29)	7.05 (5.96 to 8.21)	0.61 (0.60 to 0.61)	2.63 (2.39 to 2.91)	197 (179 to 217)	11.9 (11.0 to 12.9)	356 (327 to 388)	1.92 (1.90 to 1.93)
Peru	8.44 (7.69 to 9.40)	112 (102 to 126)	35.4 (32.6 to 38.7)	195 (180 to 213)	1.77 (1.75 to 1.80)	0.34 (0.28 to 0.39)	2.78 (2.42 to 3.22)	0.57 (0.49 to 0.68)	3.26 (2.77 to 3.81)	0.55 (0.53 to 0.56)	8.11 (7.35 to 9.07)	110 (99.1 to 123)	34.9 (32.1 to 38.1)	192 (177 to 210)	1.80 (1.78 to 1.83)
Philippines	41.8 (38.2 to 46.0)	224 (205 to 248)	142 (129 to 156)	284 (259 to 313)	0.76 (0.72 to 0.79)	3.36 (2.61 to 4.27)	9.79 (7.95 to 12.2)	4.78 (3.81 to 6.03)	8.50 (6.92 to 10.6)	-0.41 (-0.46 to -0.35)	38.4 (35.0 to 42.5)	215 (195 to 239)	137 (125 to 152)	275 (250 to 305)	0.80 (0.76 to 0.83)
Poland	45.1 (39.9 to 50.2)	197 (175 to 219)	75.6 (67.3 to 85.6)	264 (235 to 298)	0.93 (0.89 to 0.98)	1.06 (0.85 to 1.33)	5.79 (4.59 to 7.32)	1.07 (0.88 to 1.31)	6.98 (5.53 to 8.69)	0.61 (0.59 to 0.62)	44.1 (38.8 to 49.1)	191 (169 to 212)	74.5 (66.2 to 84.5)	257 (228 to 290)	0.94 (0.90 to 0.99)
Portugal	11.2 (9.97 to 12.4)	171 (155 to 190)	25.6 (23.0 to 28.7)	332 (301 to 367)	2.15 (2.14 to 2.16)	0.33 (0.29 to 0.40)	7.26 (6.23 to 8.42)	0.57 (0.46 to 0.72)	12.7 (10.5 to 15.3)	1.83 (1.82 to 1.85)	10.8 (9.67 to 12.0)	164 (148 to 182)	25.0 (22.3 to 28.2)	319 (287 to 355)	2.16 (2.15 to 2.17)
Puerto Rico	6.70 (6.21 to 7.24)	353 (326 to 384)	11.8 (10.8 to 13.2)	509 (470 to 567)	1.17 (1.16 to 1.19)	0.27 (0.24 to 0.30)	14.7 (12.9 to 16.6)	0.20 (0.18 to 0.22)	12.9 (11.2 to 14.6)	-0.35 (-0.38 to -0.32)	6.43 (5.93 to 6.97)	339 (311 to 369)	11.7 (10.6 to 13.0)	496 (457 to 555)	1.23 (1.21 to 1.24)
Qatar	0.34 (0.30 to 0.38)	500 (466 to 532)	6.50 (5.75 to 7.61)	884 (840 to 938)	1.85 (1.83 to 1.87)	0.02 (0.01 to 0.02)	10.2 (8.76 to 11.8)	0.10 (0.08 to 0.12)	11.8 (9.94 to 13.7)	0.47 (0.45 to 0.47)	0.32 (0.29 to 0.36)	490 (455 to 523)	6.41 (5.64 to 7.50)	872 (829 to 926)	1.87 (1.86 to 1.90)
Republic of Korea	40.2 (37.0 to 43.4)	191 (176 to 207)	163 (150 to 177)	430 (400 to 460)	2.65 (2.62 to 2.67)	1.32 (1.10 to 1.56)	6.63 (5.54 to 7.83)	1.14 (0.95 to 1.38)	7.45 (6.04 to 9.04)	0.40 (0.37 to 0.43)	38.9 (35.7 to 42.0)	185 (170 to 200)	162 (149 to 176)	422 (393 to 453)	2.71 (2.68 to 2.73)
Republic of Moldova	3.26 (2.94 to 3.64)	127 (115 to 142)	6.09 (5.33 to 6.80)	231 (204 to 255)	1.93 (1.91 to 1.95)	0.09 (0.08 to 0.11)	4.14 (3.54 to 4.87)	0.09 (0.08 to 0.11)	5.81 (4.86 to 7.16)	1.13 (1.10 to 1.16)	3.17 (2.84 to 3.54)	123 (111 to 138)	5.99 (5.23 to 6.71)	225 (198 to 249)	1.96 (1.93 to 1.98)
Romania	17.0 (14.5 to 19.8)	118 (103 to 136)	24.9 (22.0 to 28.1)	180 (161 to 202)	1.35 (1.32 to 1.38)	0.42 (0.36 to 0.49)	3.79 (3.26 to 4.38)	0.42 (0.35 to 0.50)	5.32 (4.49 to 6.36)	1.09 (1.07 to 1.10)	16.5 (14.1 to 19.4)	114 (99.0 to 132)	24.4 (21.6 to 27.7)	175 (156 to 197)	1.37 (1.34 to 1.40)
Russian Federation	101 (89.6 to 113)	99.9 (88.8 to 112)	217 (193 to 242)	198 (177 to 220)	2.25 (2.23 to 2.26)	4.34 (3.65 to 5.20)	5.69 (4.83 to 6.81)	6.49 (5.44 to 7.92)	9.39 (7.96 to 11.4)	1.65 (1.63 to 1.68)	96.4 (85.2 to 109)	94.2 (83.1 to 106)	210 (187 to 236)	189 (169 to 211)	2.27 (2.26 to 2.28)
Rwanda	2.46 (2.28 to 2.67)	123 (115 to 132)	6.02 (5.56 to 6.51)	128 (118 to 138)	0.12 (0.11 to 0.13)	0.31 (0.27 to 0.36)	6.13 (5.47 to 6.90)	0.41 (0.35 to 0.47)	5.43 (4.76 to 6.07)	-0.37 (-0.40 to -0.30)	2.15 (1.98 to 2.35)	117 (108 to 126)	5.61 (5.14 to 6.08)	123 (113 to 132)	0.15 (0.13 to 0.16)
Saint Kitts and Nevis	0.07 (0.06 to 0.07)	370 (344 to 400)	0.19 (0.17 to 0.21)	486 (436 to 534)	0.86 (0.84 to 0.88)	0.003 (0.003 to 0.003)	12.4 (11.0 to 14.1)	0.002 (0.002 to 0.003)	9.04 (7.89 to 10.4)	-0.94 (-1.00 to -0.90)	0.06 (0.06 to 0.07)	358 (331 to 387)	0.18 (0.16 to 0.20)	477 (427 to 525)	0.91 (0.89 to 0.93)
Saint Lucia	0.27 (0.25 to 0.28)	521 (486 to 556)	0.68 (0.62 to 0.74)	592 (549 to 639)	0.41 (0.40 to 0.42)	0.008 (0.007 to 0.009)	9.56 (8.31 to 11.1)	0.005 (0.005 to 0.006)	7.28 (6.37 to 8.53)	-0.79 (-0.84 to -0.74)	0.26 (0.24 to 0.28)	511 (477 to 546)	0.67 (0.62 to 0.73)	584 (541 to 631)	0.43 (0.42 to 0.44)
Saint Vincent and the Grenadines	0.19 (0.18 to 0.20)	480 (451 to 510)	0.40 (0.37 to 0.43)	612 (564 to 658)	0.79 (0.78 to 0.79)	0.009 (0.008 to 0.010)	12.8 (11.4 to 14.3)	0.005 (0.004 to 0.006)	9.97 (8.75 to 11.5)	-0.79 (-0.84 to -0.73)	0.18 (0.17 to 0.20)	467 (438 to 498)	0.40 (0.36 to 0.43)	602 (555 to 648)	0.82 (0.82 to 0.83)
Samoa	0.21 (0.19 to 0.22)	365 (336 to 396)	0.60 (0.56 to 0.66)	677 (624 to 739)	2.01 (2.00 to 2.01)	0.004 (0.004 to 0.005)	4.63 (4.04 to 5.32)	0.005 (0.005 to 0.006)	4.62 (4.04 to 5.22)	-0.00 (-0.01 to 0.01)	0.20 (0.18 to 0.22)	360 (331 to 391)	0.60 (0.55 to 0.66)	672 (620 to 734)	2.02 (2.02 to 2.03)
San Marino	0.01 (0.01 to 0.02)	97.4 (87.5 to 108)	0.04 (0.04 to 0.05)	195 (175 to 217)	2.27 (2.26 to 2.28)	1e-03 (8e-04 to 0.001)	8.72 (7.21 to 10.4)	0.002 (0.002 to 0.003)	14.6 (12.1 to 17.3)	1.66 (1.64 to 1.68)	0.01 (0.01 to 0.02)	88.7 (79.0 to 99.3)	0.04 (0.04 to 0.05)	180 (161 to 203)	2.33 (2.32 to 2.34)
Sao Tome and Principe	0.06 (0.05 to 0.06)	145 (130 to 162)	0.23 (0.21 to 0.26)	272 (242 to 302)	2.06 (2.05 to 2.08)	0.004 (0.003 to 0.005)	5.71 (5.03 to 6.57)	0.007 (0.006 to 0.008)	6.01 (5.19 to 6.89)	0.16 (0.16 to 0.17)	0.05 (0.05 to 0.06)	139 (125 to 156)	0.22 (0.20 to 0.25)	265 (236 to 296)	2.12 (2.11 to 2.14)
Saudi Arabia	9.37 (8.52 to 10.1)	241 (218 to 262)	77.6 (69.8 to 87.1)	475 (434 to 521)	2.21 (2.20 to 2.21)	0.80 (0.65 to 1.14)	9.20 (7.60 to 12.5)	1.96 (1.54 to 3.16)	13.5 (10.7 to 22.5)	1.22 (1.20 to 1.24)	8.57 (7.72 to 9.31)	232 (209 to 253)	75.6 (67.9 to 85.2)	461 (419 to 509)	2.24 (2.23 to 2.24)
Senegal	4.17 (3.76 to 4.58)	188 (169 to 208)	19.8 (18.3 to 21.4)	361 (332 to 394)	2.16 (2.14 to 2.18)	0.24 (0.20 to 0.30)	5.57 (4.83 to 6.50)	0.48 (0.41 to 0.58)	5.67 (4.94 to 6.59)	0.05 (0.03 to 0.06)	3.93 (3.51 to 4.33)	183 (163 to 202)	19.3 (17.9 to 21.0)	355 (326 to 389)	2.21 (2.19 to 2.23)
Serbia	13.7 (12.3 to 15.4)	218 (197 to 243)	22.2 (19.9 to 24.8)	342 (308 to 381)	1.46 (1.44 to 1.47)	0.33 (0.30 to 0.37)	7.40 (6.68 to 8.29)	0.36 (0.32 to 0.42)	9.66 (8.39 to 11.3)	0.87 (0.84 to 0.91)	13.4 (11.9 to 15.0)	211 (189 to 235)	21.9 (19.5 to 24.4)	332 (298 to 371)	1.48 (1.46 to 1.49)
Seychelles	0.06 (0.06 to 0.07)	203 (184 to 225)	0.36 (0.33 to 0.40)	588 (540 to 642)	3.50 (3.48 to 3.52)	0.003 (0.002 to 0.003)	7.08 (6.28 to 8.00)	0.003 (0.003 to 0.004)	6.90 (6.04 to 7.92)	-0.08 (-0.09 to -0.08)	0.06 (0.06 to 0.07)	196 (177 to 219)	0.36 (0.32 to 0.39)	581 (532 to 635)	3.58 (3.56 to 3.60)
Sierra Leone	1.77 (1.63 to 1.94)	136 (125 to 149)	7.69 (6.95 to 8.47)	264 (241 to 291)	2.17 (2.16 to 2.18)	0.12 (0.10 to 0.14)	5.12 (4.41 to 5.95)	0.27 (0.23 to 0.32)	5.43 (4.70 to 6.38)	0.17 (0.15 to 0.19)	1.65 (1.50 to 1.83)	131 (120 to 144)	7.43 (6.69 to 8.20)	259 (235 to 285)	2.23 (2.21 to 2.25)
Singapore	4.11 (3.75 to 4.48)	266 (242 to 291)	12.1 (10.7 to 13.5)	320 (286 to 353)	0.60 (0.57 to 0.61)	0.06 (0.05 to 0.07)	4.93 (3.99 to 5.91)	0.10 (0.08 to 0.12)	5.77 (4.61 to 7.14)	0.50 (0.49 to 0.51)	4.05 (3.69 to 4.43)	261 (237 to 286)	12.0 (10.6 to 13.4)	315 (280 to 348)	0.60 (0.58 to 0.62)
Slovakia	4.70 (4.19 to 5.29)	152 (136 to 171)	8.68 (7.58 to 9.65)	215 (190 to 238)	1.14 (1.12 to 1.15)	0.11 (0.10 to 0.13)	4.45 (4.00 to 5.00)	0.15 (0.13 to 0.18)	6.67 (5.69 to 7.84)	1.33 (1.31 to 1.34)	4.59 (4.08 to 5.16)	148 (131 to 166)	8.53 (7.43 to 9.50)	209 (184 to 232)	1.13 (1.11 to 1.15)
Slovenia	2.32 (2.04 to 2.66)	176 (155 to 199)	3.33 (2.92 to 3.74)	213 (190 to 239)	0.62 (0.58 to 0.65)	0.05 (0.04 to 0.05)	5.31 (4.61 to 6.10)	0.06 (0.05 to 0.07)	6.98 (5.79 to 8.41)	0.94 (0.90 to 1.02)	2.27 (1.99 to 2.61)	171 (150 to 193)	3.27 (2.86 to 3.68)	206 (183 to 231)	0.60 (0.56 to 0.63)
Solomon Islands	0.25 (0.24 to 0.28)	255 (239 to 274)	1.31 (1.23 to 1.41)	483 (459 to 509)	2.08 (2.06 to 2.10)	0.01 (0.01 to 0.01)	5.99 (5.30 to 6.69)	0.03 (0.02 to 0.03)	6.77 (6.00 to 7.70)	0.37 (0.36 to 0.39)	0.24 (0.22 to 0.26)	249 (234 to 268)	1.29 (1.20 to 1.38)	476 (452 to 502)	2.11 (2.09 to 2.13)
Somalia	2.38 (2.19 to 2.58)	128 (117 to 139)	10.1 (9.16 to 11.0)	172 (158 to 185)	0.96 (0.95 to 0.97)	0.34 (0.29 to 0.38)	5.90 (5.26 to 6.59)	0.88 (0.74 to 1.03)	5.74 (5.00 to 6.47)	-0.07 (-0.09 to -0.06)	2.05 (1.86 to 2.24)	122 (111 to 133)	9.21 (8.29 to 10.1)	166 (152 to 180)	1.00 (0.99 to 1.01)
South Africa	29.2 (26.7 to 32.0)	212 (193 to 232)	106 (97.3 to 115)	364 (335 to 395)	1.75 (1.72 to 1.78)	1.38 (1.11 to 1.74)	6.90 (5.65 to 8.58)	1.99 (1.63 to 2.51)	6.99 (5.76 to 8.70)	0.04 (0.03 to 0.04)	27.9 (25.4 to 30.5)	205 (187 to 225)	104 (95.5 to 114)	357 (328 to 388)	1.80 (1.76 to 1.83)
South Sudan	1.59 (1.46 to 1.73)	111 (102 to 122)	4.24 (3.85 to 4.61)	146 (135 to 157)	0.89 (0.89 to 0.90)	0.18 (0.15 to 0.21)	4.60 (4.02 to 5.21)	0.28 (0.24 to 0.32)	4.50 (3.97 to 5.13)	-0.06 (-0.08 to -0.05)	1.41 (1.29 to 1.55)	106 (97.4 to 117)	3.96 (3.57 to 4.32)	142 (131 to 153)	0.93 (0.92 to 0.93)
Spain	50.1 (45.0 to 56.6)	199 (179 to 222)	99.7 (90.5 to 109)	316 (290 to 340)	1.46 (1.43 to 1.49)	1.63 (1.52 to 1.75)	9.25 (8.65 to 10.0)	2.99 (2.57 to 3.49)	16.8 (14.8 to 19.3)	1.92 (1.87 to 1.95)	48.5 (43.4 to 55.0)	190 (169 to 213)	96.7 (87.4 to 106)	299 (273 to 323)	1.43 (1.40 to 1.46)
Sri Lanka	18.4 (16.6 to 20.4)	292 (263 to 326)	86.2 (78.9 to 93.4)	590 (541 to 638)	2.29 (2.28 to 2.30)	0.67 (0.59 to 0.78)	7.91 (7.13 to 8.87)	0.96 (0.85 to 1.08)	8.44 (7.47 to 9.72)	0.22 (0.21 to 0.23)	17.8 (16.0 to 19.7)	284 (255 to 319)	85.2 (77.9 to 92.5)	581 (533 to 629)	2.33 (2.32 to 2.34)
Sudan	11.3 (10.4 to 12.4)	184 (167 to 203)	60.0 (54.9 to 65.1)	385 (349 to 419)	2.41 (2.39 to 2.42)	0.60 (0.49 to 0.73)	4.97 (4.21 to 5.82)	1.37 (1.14 to 1.63)	5.50 (4.68 to 6.45)	0.33 (0.33 to 0.33)	10.8 (9.78 to 11.8)	179 (162 to 198)	58.7 (53.6 to 63.6)	379 (344 to 413)	2.45 (2.43 to 2.46)
Suriname	0.48 (0.44 to 0.52)	299 (273 to 328)	1.79 (1.64 to 1.96)	538 (494 to 588)	1.90 (1.88 to 1.92)	0.01 (0.01 to 0.02)	6.22 (5.46 to 7.10)	0.02 (0.01 to 0.02)	5.60 (4.88 to 6.42)	-0.32 (-0.33 to -0.31)	0.46 (0.42 to 0.51)	292 (267 to 322)	1.77 (1.62 to 1.94)	532 (488 to 582)	1.93 (1.91 to 1.95)
Sweden	7.32 (6.56 to 8.22)	130 (117 to 146)	13.0 (11.4 to 14.8)	202 (177 to 227)	1.44 (1.41 to 1.48)	0.49 (0.41 to 0.60)	13.2 (11.1 to 15.9)	0.67 (0.56 to 0.82)	15.5 (12.7 to 18.7)	0.49 (0.48 to 0.50)	6.82 (6.07 to 7.72)	117 (104 to 132)	12.4 (10.7 to 14.2)	187 (163 to 211)	1.53 (1.49 to 1.58)
Switzerland	7.36 (6.63 to 8.14)	164 (147 to 181)	16.8 (15.0 to 18.4)	286 (257 to 311)	1.83 (1.81 to 1.86)	0.26 (0.22 to 0.32)	8.55 (7.11 to 10.2)	0.50 (0.41 to 0.60)	13.1 (10.9 to 15.6)	1.37 (1.34 to 1.39)	7.10 (6.37 to 7.88)	156 (139 to 173)	16.3 (14.5 to 17.9)	273 (245 to 298)	1.85 (1.83 to 1.89)
Syrian Arab Republic	8.24 (7.48 to 9.02)	224 (201 to 247)	34.1 (30.6 to 37.5)	421 (379 to 461)	2.06 (2.05 to 2.06)	0.71 (0.58 to 0.86)	10.0 (8.74 to 11.7)	0.76 (0.64 to 0.89)	10.4 (8.98 to 12.0)	0.14 (0.12 to 0.17)	7.53 (6.80 to 8.31)	214 (192 to 237)	33.4 (29.8 to 36.8)	410 (370 to 451)	2.12 (2.12 to 2.13)
Taiwan (Province of China)	19.0 (18.0 to 20.2)	215 (204 to 228)	53.9 (49.4 to 58.8)	289 (268 to 312)	0.96 (0.95 to 0.97)	0.66 (0.55 to 0.80)	6.77 (5.70 to 8.16)	0.82 (0.68 to 0.97)	7.05 (6.17 to 8.20)	0.14 (0.09 to 0.20)	18.4 (17.3 to 19.6)	208 (198 to 221)	53.0 (48.6 to 57.9)	282 (261 to 305)	0.98 (0.97 to 0.99)
Tajikistan	1.69 (1.55 to 1.86)	89.4 (81.7 to 98.7)	9.41 (8.55 to 10.3)	212 (193 to 233)	2.82 (2.79 to 2.84)	0.16 (0.14 to 0.19)	5.17 (4.49 to 5.93)	0.36 (0.31 to 0.42)	6.67 (5.75 to 7.68)	0.82 (0.82 to 0.83)	1.53 (1.40 to 1.70)	84.2 (76.8 to 93.5)	9.05 (8.16 to 9.91)	205 (186 to 226)	2.91 (2.88 to 2.93)
Thailand	38.4 (35.3 to 42.1)	167 (153 to 184)	162 (144 to 184)	302 (271 to 340)	1.94 (1.92 to 1.96)	2.00 (1.74 to 2.28)	6.70 (5.97 to 7.55)	1.70 (1.50 to 1.95)	5.63 (4.92 to 6.49)	-0.49 (-0.55 to -0.40)	36.4 (33.2 to 40.1)	161 (146 to 177)	160 (143 to 183)	296 (266 to 334)	2.01 (2.00 to 2.03)
Timor-Leste	0.28 (0.26 to 0.30)	139 (128 to 151)	1.73 (1.56 to 1.92)	346 (308 to 386)	3.02 (3.01 to 3.04)	0.02 (0.02 to 0.03)	5.44 (4.77 to 6.31)	0.04 (0.03 to 0.05)	5.62 (4.94 to 6.50)	0.09 (0.08 to 0.10)	0.26 (0.23 to 0.28)	133 (123 to 146)	1.69 (1.52 to 1.88)	340 (302 to 380)	3.10 (3.08 to 3.13)
Togo	1.13 (1.02 to 1.23)	115 (106 to 125)	5.72 (5.25 to 6.18)	188 (173 to 202)	1.59 (1.59 to 1.60)	0.11 (0.09 to 0.13)	5.36 (4.61 to 6.22)	0.24 (0.21 to 0.29)	5.32 (4.61 to 6.14)	-0.03 (-0.04 to -0.02)	1.01 (0.91 to 1.12)	110 (101 to 120)	5.48 (5.02 to 5.94)	183 (168 to 197)	1.65 (1.65 to 1.66)
Tokelau	0.003 (0.003 to 0.003)	430 (396 to 463)	0.005 (0.005 to 0.005)	713 (662 to 769)	1.65 (1.64 to 1.66)	5e-05 (4e-05 to 6e-05)	5.54 (4.81 to 6.27)	4e-05 (3e-05 to 4e-05)	5.39 (4.68 to 6.14)	-0.06 (-0.08 to -0.04)	0.003 (0.003 to 0.003)	424 (390 to 457)	0.005 (0.004 to 0.005)	707 (656 to 764)	1.67 (1.66 to 1.68)
Tonga	0.15 (0.14 to 0.16)	421 (390 to 450)	0.32 (0.29 to 0.34)	667 (615 to 725)	1.49 (1.49 to 1.50)	0.003 (0.002 to 0.003)	4.67 (4.10 to 5.30)	0.003 (0.002 to 0.003)	4.65 (4.05 to 5.37)	-0.01 (-0.02 to -0.01)	0.15 (0.14 to 0.16)	416 (386 to 445)	0.31 (0.29 to 0.34)	662 (610 to 720)	1.51 (1.50 to 1.51)
Trinidad and Tobago	2.50 (2.38 to 2.64)	516 (493 to 544)	5.44 (4.97 to 5.88)	626 (571 to 672)	0.62 (0.60 to 0.63)	0.06 (0.06 to 0.07)	9.55 (8.49 to 10.9)	0.05 (0.04 to 0.05)	7.55 (6.62 to 8.64)	-0.70 (-0.74 to -0.67)	2.44 (2.31 to 2.58)	506 (483 to 534)	5.39 (4.93 to 5.84)	619 (563 to 664)	0.64 (0.62 to 0.65)
Tunisia	5.61 (5.12 to 6.17)	182 (165 to 202)	31.2 (27.7 to 34.5)	442 (395 to 485)	2.89 (2.88 to 2.91)	0.25 (0.21 to 0.30)	5.34 (4.61 to 6.20)	0.33 (0.28 to 0.39)	6.08 (5.21 to 7.18)	0.41 (0.41 to 0.42)	5.36 (4.87 to 5.93)	177 (160 to 197)	30.9 (27.4 to 34.1)	435 (389 to 480)	2.95 (2.93 to 2.96)
Turkmenistan	1.10 (1.02 to 1.20)	78.9 (72.4 to 86.2)	4.45 (4.02 to 4.92)	174 (157 to 192)	2.57 (2.55 to 2.59)	0.13 (0.11 to 0.15)	5.83 (5.16 to 6.75)	0.21 (0.18 to 0.24)	8.17 (7.11 to 9.49)	1.09 (1.08 to 1.10)	0.98 (0.89 to 1.07)	73.1 (66.6 to 80.4)	4.24 (3.81 to 4.72)	166 (150 to 184)	2.67 (2.64 to 2.69)
Tuvalu	0.01 (0.01 to 0.02)	333 (310 to 357)	0.03 (0.03 to 0.04)	582 (547 to 623)	1.82 (1.81 to 1.83)	3e-04 (3e-04 to 4e-04)	6.14 (5.35 to 7.00)	4e-04 (3e-04 to 4e-04)	5.91 (5.15 to 6.70)	-0.11 (-0.12 to -0.09)	0.01 (0.01 to 0.02)	327 (305 to 351)	0.03 (0.03 to 0.04)	576 (541 to 617)	1.85 (1.84 to 1.86)
Türkiye	37.9 (35.5 to 40.3)	179 (168 to 191)	164 (150 to 182)	334 (305 to 370)	2.05 (1.98 to 2.08)	2.20 (1.87 to 2.63)	7.16 (6.25 to 8.31)	2.82 (2.42 to 3.29)	7.26 (6.15 to 8.49)	0.05 (0.03 to 0.06)	35.7 (33.3 to 38.1)	172 (161 to 184)	161 (147 to 180)	327 (298 to 364)	2.11 (2.04 to 2.15)
Uganda	4.56 (4.22 to 4.93)	105 (97.1 to 113)	16.3 (15.1 to 17.6)	134 (124 to 145)	0.79 (0.78 to 0.80)	0.59 (0.50 to 0.69)	4.66 (4.07 to 5.26)	1.31 (1.11 to 1.53)	4.73 (4.14 to 5.36)	0.07 (0.05 to 0.09)	3.97 (3.64 to 4.32)	101 (92.3 to 109)	14.9 (13.8 to 16.2)	129 (119 to 140)	0.82 (0.81 to 0.83)
Ukraine	33.8 (29.4 to 38.0)	94.5 (83.5 to 106)	52.5 (46.1 to 59.6)	164 (146 to 184)	1.80 (1.78 to 1.81)	1.37 (1.12 to 1.73)	5.60 (4.49 to 7.08)	1.40 (1.14 to 1.75)	7.62 (6.13 to 9.60)	1.03 (1.01 to 1.04)	32.4 (28.1 to 36.6)	88.9 (77.9 to 99.7)	51.1 (44.7 to 58.2)	157 (138 to 176)	1.84 (1.82 to 1.86)
United Arab Emirates	0.99 (0.90 to 1.09)	374 (351 to 402)	14.4 (12.4 to 16.2)	502 (457 to 543)	0.95 (0.92 to 0.97)	0.07 (0.06 to 0.09)	12.0 (9.49 to 14.8)	0.30 (0.23 to 0.37)	11.8 (9.80 to 14.3)	-0.01 (-0.07 to 0.06)	0.91 (0.83 to 1.02)	362 (338 to 391)	14.1 (12.1 to 15.9)	490 (445 to 532)	0.98 (0.94 to 1.00)
United Kingdom	47.6 (42.9 to 53.0)	132 (120 to 146)	110 (101 to 120)	277 (254 to 301)	2.39 (2.35 to 2.44)	2.37 (1.99 to 2.85)	9.16 (7.74 to 10.9)	3.30 (2.71 to 4.03)	10.8 (8.92 to 13.0)	0.53 (0.49 to 0.57)	45.2 (40.6 to 50.5)	123 (110 to 137)	107 (97.7 to 117)	266 (244 to 291)	2.51 (2.46 to 2.56)
United Republic of Tanzania	7.25 (6.72 to 7.88)	99.4 (93.2 to 106)	28.6 (26.3 to 30.9)	152 (142 to 163)	1.38 (1.37 to 1.39)	1.12 (0.84 to 1.41)	5.78 (4.72 to 6.85)	2.26 (1.71 to 2.84)	6.00 (4.83 to 7.32)	0.07 (-0.02 to 0.13)	6.14 (5.67 to 6.64)	93.6 (87.5 to 100)	26.3 (24.2 to 28.5)	146 (136 to 157)	1.44 (1.44 to 1.45)
United States Virgin Islands	0.23 (0.20 to 0.25)	412 (372 to 453)	0.37 (0.34 to 0.41)	612 (557 to 667)	1.29 (1.28 to 1.30)	0.005 (0.005 to 0.006)	9.65 (8.48 to 11.0)	0.003 (0.003 to 0.004)	8.61 (7.40 to 9.86)	-0.35 (-0.37 to -0.34)	0.22 (0.20 to 0.25)	402 (363 to 443)	0.37 (0.33 to 0.41)	604 (549 to 659)	1.32 (1.31 to 1.33)
United States of America	244 (224 to 266)	176 (161 to 192)	908 (849 to 965)	401 (377 to 425)	2.71 (2.68 to 2.73)	14.5 (12.3 to 17.1)	12.4 (10.6 to 14.6)	21.1 (17.6 to 25.4)	14.6 (12.3 to 17.5)	0.53 (0.52 to 0.54)	230 (210 to 252)	163 (148 to 180)	886 (829 to 945)	387 (363 to 410)	2.83 (2.80 to 2.85)
Uruguay	2.28 (2.08 to 2.51)	124 (114 to 137)	5.87 (5.19 to 6.51)	267 (238 to 296)	2.47 (2.40 to 2.52)	0.13 (0.11 to 0.15)	8.28 (7.04 to 9.50)	0.16 (0.13 to 0.19)	9.82 (8.04 to 11.8)	0.54 (0.52 to 0.56)	2.15 (1.95 to 2.38)	116 (106 to 128)	5.71 (5.04 to 6.36)	257 (228 to 286)	2.57 (2.50 to 2.62)
Uzbekistan	7.95 (7.33 to 8.70)	100 (92.2 to 110)	51.9 (48.1 to 57.5)	289 (267 to 319)	3.49 (3.46 to 3.52)	0.94 (0.81 to 1.08)	7.66 (6.75 to 8.84)	1.90 (1.67 to 2.17)	11.3 (9.99 to 12.9)	1.25 (1.23 to 1.28)	7.01 (6.38 to 7.76)	92.6 (84.5 to 102)	50.0 (46.0 to 55.6)	277 (256 to 308)	3.63 (3.59 to 3.65)
Vanuatu	0.12 (0.11 to 0.13)	247 (228 to 266)	0.67 (0.62 to 0.73)	507 (469 to 546)	2.35 (2.34 to 2.36)	0.004 (0.004 to 0.005)	4.86 (4.21 to 5.59)	0.009 (0.007 to 0.01)	5.63 (4.37 to 5.73)	0.11 (0.11 to 0.12)	0.11 (0.10 to 0.12)	242 (223 to 261)	0.67 (0.61 to 0.72)	502 (465 to 541)	2.38 (2.37 to 2.39)
Venezuela (Bolivarian Republic of)	14.4 (13.3 to 15.6)	213 (196 to 233)	51.6 (46.8 to 59.3)	327 (298 to 373)	1.43 (1.37 to 1.48)	0.33 (0.28 to 0.39)	3.01 (2.59 to 3.47)	0.36 (0.31 to 0.43)	2.96 (2.52 to 3.48)	-0.05 (-0.06 to -0.03)	14.0 (12.9 to 15.3)	210 (193 to 230)	51.2 (46.5 to 58.9)	324 (295 to 370)	1.44 (1.39 to 1.50)
Viet Nam	42.7 (39.4 to 46.4)	168 (155 to 182)	166 (154 to 180)	287 (266 to 310)	1.73 (1.70 to 1.75)	2.61 (2.23 to 3.10)	7.10 (6.27 to 8.15)	3.55 (3.09 to 4.17)	7.41 (6.45 to 8.70)	0.16 (0.14 to 0.17)	40.1 (36.9 to 43.8)	161 (148 to 175)	162 (150 to 177)	279 (259 to 303)	1.79 (1.76 to 1.81)
Yemen	5.31 (4.79 to 5.87)	145 (131 to 160)	33.6 (30.0 to 37.6)	293 (261 to 328)	2.26 (2.21 to 2.30)	0.41 (0.33 to 0.51)	5.07 (4.34 to 6.03)	1.08 (0.89 to 1.29)	5.57 (4.71 to 6.53)	0.28 (0.26 to 0.30)	4.91 (4.41 to 5.46)	140 (126 to 155)	32.5 (29.0 to 36.5)	287 (256 to 323)	2.31 (2.27 to 2.35)
Zambia	3.21 (2.94 to 3.48)	159 (147 to 173)	12.7 (11.6 to 14.0)	207 (189 to 227)	0.82 (0.80 to 0.84)	0.34 (0.29 to 0.39)	5.75 (5.13 to 6.39)	0.61 (0.53 to 0.72)	5.14 (4.52 to 5.82)	-0.32 (-0.35 to -0.29)	2.87 (2.62 to 3.14)	154 (142 to 167)	12.1 (11.0 to 13.4)	202 (183 to 222)	0.86 (0.84 to 0.88)
Zimbabwe	4.14 (3.77 to 4.48)	158 (145 to 171)	15.3 (14.1 to 16.7)	289 (268 to 313)	1.97 (1.96 to 1.98)	0.23 (0.18 to 0.28)	3.86 (3.27 to 4.47)	0.39 (0.32 to 0.46)	4.49 (3.77 to 5.24)	0.40 (0.36 to 0.45)	3.91 (3.55 to 4.25)	154 (141 to 167)	14.9 (13.7 to 16.3)	285 (264 to 309)	2.00 (1.99 to 2.01)

AAPC: Average annual percentage change; ASR: Age-standardized rate; DM: Diabetes mellitus; SDI: Sociodemographic index; T1D: Type 1 diabetes; T2D: Type 2 diabetes. Results assuming first-order autocorrelation estimated from the data were broadly similar to those of the primary analyses, despite somewhat different estimates.

Table S2. Prevalence number, age-standardized rate, and AAPC of diabetes and two subtypes among **women** globally and by different SDI, regions, countries, and territories, 1990-2021

Location	DM, Number in 1990 (000s)	DM, ASR in 1990 (per 100,000)	DM, Number in 2021 (000s)	DM, ASR in 2021 (per 100,000)	DM, AAPC (95%CI)	T1D, Number in 1990 (000s)	T1D, ASR in 1990 (per 100,000)	T1D, Number in 2021 (000s)	T1D, ASR in 2021 (per 100,000)	T1D, AAPC (95%CI)	T2D, Number in 1990 (000s)	T2D, ASR in 1990 (per 100,000)	T2D, Number in 2021 (000s)	T2D, ASR in 2021 (per 100,000)	T2D, AAPC (95%CI)
**Global**	68905 (63409 to 74618)	3069 (2824 to 3329)	254813 (237402 to 273987)	5743 (5352 to 6185)	2.04 (2.02 to 2.05)	5040 (4232 to 5988)	202 (170 to 239)	9790 (8266 to 11593)	236 (198 to 280)	0.50 (0.50 to 0.51)	63865 (58475 to 69679)	2867 (2629 to 3135)	245023 (227665 to 264112)	5507 (5113 to 5936)	2.13 (2.11 to 2.14)
**By SDI**															
High SDI	16241 (15083 to 17498)	2827 (2627 to 3047)	55389 (52258 to 58921)	6151 (5790 to 6543)	2.54 (2.53 to 2.55)	1566 (1373 to 1794)	318 (277 to 365)	2847 (2461 to 3275)	428 (370 to 493)	0.96 (0.95 to 0.97)	14676 (13521 to 15962)	2509 (2310 to 2739)	52542 (49488 to 56220)	5723 (5364 to 6122)	2.70 (2.68 to 2.71)
High-middle SDI	16585 (15203 to 17989)	2962 (2719 to 3214)	51129 (47480 to 55286)	5377 (4988 to 5825)	1.93 (1.91 to 1.95)	934 (802 to 1088)	169 (146 to 198)	1804 (1523 to 2147)	229 (193 to 270)	0.97 (0.95 to 0.98)	15651 (14319 to 17126)	2793 (2555 to 3053)	49325 (45659 to 53288)	5148 (4766 to 5577)	1.98 (1.96 to 2.01)
Middle SDI	21783 (19965 to 23692)	3555 (3248 to 3892)	82824 (76936 to 89695)	5846 (5430 to 6329)	1.62 (1.61 to 1.64)	1199 (957 to 1497)	149 (122 to 183)	2302 (1876 to 2840)	176 (142 to 217)	0.56 (0.54 to 0.57)	20584 (18772 to 22569)	3405 (3100 to 3754)	80523 (74569 to 87434)	5670 (5250 to 6143)	1.66 (1.65 to 1.68)
Low-middle SDI	10653 (9795 to 11553)	2938 (2699 to 3194)	49272 (45513 to 53029)	5925 (5464 to 6391)	2.28 (2.27 to 2.29)	965 (776 to 1195)	191 (157 to 234)	1963 (1598 to 2416)	208 (171 to 255)	0.27 (0.27 to 0.27)	9688 (8801 to 10587)	2747 (2499 to 3005)	47309 (43411 to 51049)	5717 (5265 to 6183)	2.38 (2.37 to 2.40)
Low SDI	3554 (3269 to 3848)	2550 (2342 to 2770)	15943 (14635 to 17246)	4709 (4330 to 5081)	2.00 (1.99 to 2.00)	370 (308 to 445)	174 (147 to 207)	865 (711 to 1053)	175 (147 to 210)	0.02 (0.02 to 0.03)	3184 (2892 to 3478)	2376 (2168 to 2594)	15078 (13786 to 16431)	4533 (4161 to 4917)	2.10 (2.09 to 2.11)
**Four world regions**															
Africa	4464 (4120 to 4806)	2586 (2385 to 2792)	23202 (21536 to 24872)	5548 (5161 to 5908)	2.49 (2.49 to 2.49)	458 (384 to 546)	173 (148 to 204)	1061 (883 to 1277)	175 (148 to 208)	0.03 (0.02 to 0.03)	4006 (3650 to 4342)	2412 (2207 to 2617)	22141 (20458 to 23786)	5373 (4981 to 5741)	2.62 (2.61 to 2.62)
America	12760 (11873 to 13751)	3810 (3540 to 4115)	48840 (45893 to 51886)	7206 (6767 to 7660)	2.09 (2.07 to 2.10)	1180 (1013 to 1375)	333 (286 to 387)	2164 (1875 to 2516)	367 (317 to 428)	0.32 (0.31 to 0.33)	11579 (10667 to 12607)	3478 (3200 to 3793)	46676 (43840 to 49763)	6839 (6403 to 7311)	2.22 (2.20 to 2.23)
Asia	38145 (34774 to 41609)	3241 (2944 to 3560)	148555 (137597 to 161021)	5703 (5281 to 6173)	1.84 (1.83 to 1.86)	2214 (1789 to 2730)	150 (122 to 183)	4312 (3518 to 5297)	179 (145 to 221)	0.59 (0.58 to 0.60)	35932 (32573 to 39542)	3091 (2805 to 3416)	144243 (133239 to 156943)	5524 (5104 to 6000)	1.89 (1.87 to 1.91)
Europe	13313 (12395 to 14326)	2372 (2210 to 2548)	33606 (31177 to 36301)	4648 (4302 to 5014)	2.18 (2.16 to 2.19)	1173 (1025 to 1339)	252 (219 to 290)	2234 (1914 to 2619)	407 (344 to 473)	1.52 (1.50 to 1.54)	12140 (11207 to 13182)	2119 (1952 to 2304)	31371 (28897 to 34122)	4241 (3895 to 4592)	2.25 (2.23 to 2.26)
**Six WHO regions**															
African Region	3340 (3066 to 3611)	2410 (2216 to 2617)	15401 (14250 to 16573)	4510 (4190 to 4835)	2.04 (2.03 to 2.04)	352 (293 to 420)	163 (139 to 193)	821 (680 to 991)	162 (137 to 195)	-0.02 (-0.03 to -0.02)	2988 (2719 to 3258)	2246 (2051 to 2448)	14580 (13439 to 15799)	4347 (4025 to 4678)	2.14 (2.14 to 2.15)
Region of the Americas	12760 (11873 to 13751)	3810 (3540 to 4115)	48840 (45893 to 51886)	7206 (6767 to 7660)	2.09 (2.07 to 2.10)	1180 (1013 to 1375)	333 (286 to 387)	2164 (1875 to 2516)	367 (317 to 428)	0.32 (0.31 to 0.33)	11579 (10667 to 12607)	3478 (3200 to 3793)	46676 (43840 to 49763)	6839 (6403 to 7311)	2.22 (2.20 to 2.23)
South-East Asia Region	12395 (11309 to 13497)	2921 (2667 to 3176)	55997 (51303 to 60766)	5547 (5082 to 6016)	2.09 (2.07 to 2.11)	1101 (873 to 1378)	190 (153 to 236)	2155 (1733 to 2694)	204 (164 to 254)	0.22 (0.21 to 0.22)	11294 (10187 to 12444)	2731 (2473 to 3006)	53843 (49066 to 58605)	5343 (4878 to 5812)	2.19 (2.17 to 2.21)
European Region	13854 (12897 to 14912)	2376 (2214 to 2552)	35893 (33378 to 38701)	4698 (4352 to 5057)	2.21 (2.20 to 2.22)	1225 (1073 to 1396)	250 (217 to 287)	2361 (2031 to 2754)	399 (340 to 462)	1.49 (1.47 to 1.52)	12629 (11659 to 13701)	2126 (1958 to 2304)	33532 (30934 to 36333)	4299 (3962 to 4643)	2.29 (2.27 to 2.30)
Eastern Mediterranean Region	3592 (3315 to 3901)	3522 (3241 to 3827)	23757 (22057 to 25674)	8960 (8318 to 9659)	3.05 (3.04 to 3.06)	345 (281 to 423)	230 (191 to 276)	899 (730 to 1091)	262 (215 to 314)	0.42 (0.42 to 0.43)	3247 (2961 to 3567)	3292 (3021 to 3601)	22858 (21148 to 24713)	8697 (8059 to 9393)	3.18 (3.17 to 3.19)
Western Pacific Region	22427 (20264 to 24699)	3408 (3082 to 3748)	73184 (67665 to 79351)	5552 (5112 to 6049)	1.58 (1.56 to 1.60)	803 (667 to 979)	106 (88.7 to 129)	1341 (1104 to 1636)	125 (102 to 153)	0.56 (0.53 to 0.58)	21624 (19466 to 23931)	3302 (2970 to 3648)	71844 (66296 to 77909)	5427 (4989 to 5919)	1.61 (1.59 to 1.63)
**Seven super regions**															
Southeast Asia, East Asia, and Oceania	22091 (20036 to 24279)	3345 (3034 to 3686)	76319 (70750 to 82747)	5409 (4996 to 5873)	1.56 (1.53 to 1.59)	940 (761 to 1158)	114 (93.3 to 139)	1574 (1275 to 1944)	133 (107 to 164)	0.53 (0.50 to 0.56)	21151 (19162 to 23391)	3231 (2918 to 3579)	74744 (69158 to 81198)	5276 (4862 to 5723)	1.59 (1.56 to 1.62)
Central Europe, Eastern Europe, and Central Asia	6245 (5767 to 6775)	2261 (2095 to 2448)	14901 (13721 to 16127)	4315 (3983 to 4660)	2.10 (2.09 to 2.11)	419 (356 to 494)	178 (151 to 210)	721 (612 to 856)	279 (234 to 330)	1.47 (1.45 to 1.48)	5825 (5346 to 6359)	2083 (1915 to 2269)	14180 (13023 to 15433)	4037 (3703 to 4381)	2.15 (2.14 to 2.16)
South Asia	10262 (9284 to 11248)	2986 (2714 to 3273)	47549 (43178 to 51921)	5646 (5130 to 6156)	2.05 (2.02 to 2.07)	861 (679 to 1094)	183 (144 to 229)	1876 (1484 to 2364)	204 (162 to 257)	0.35 (0.34 to 0.36)	9401 (8440 to 10430)	2803 (2524 to 3098)	45673 (41221 to 50067)	5442 (4920 to 5957)	2.13 (2.11 to 2.15)
High-income	17326 (16104 to 18644)	2806 (2614 to 3014)	55879 (52592 to 59399)	5994 (5640 to 6364)	2.47 (2.46 to 2.49)	1783 (1568 to 2034)	345 (302 to 394)	3342 (2900 to 3865)	490 (426 to 564)	1.10 (1.08 to 1.12)	15543 (14344 to 16948)	2461 (2266 to 2684)	52537 (49342 to 56260)	5503 (5160 to 5899)	2.63 (2.61 to 2.64)
Latin America and Caribbean	6433 (5989 to 6933)	4854 (4506 to 5229)	23230 (21473 to 25082)	6889 (6372 to 7434)	1.15 (1.13 to 1.16)	389 (316 to 482)	215 (176 to 263)	739 (603 to 897)	229 (186 to 280)	0.24 (0.22 to 0.26)	6044 (5579 to 6551)	4639 (4293 to 5014)	22491 (20766 to 24345)	6659 (6148 to 7205)	1.18 (1.17 to 1.20)
North Africa and Middle East	3491 (3249 to 3748)	3624 (3355 to 3905)	23506 (21853 to 25197)	9197 (8565 to 9849)	3.04 (3.04 to 3.05)	309 (255 to 368)	225 (189 to 265)	747 (610 to 884)	253 (208 to 300)	0.38 (0.38 to 0.39)	3181 (2932 to 3450)	3399 (3134 to 3678)	22759 (21099 to 24473)	8944 (8310 to 9595)	3.16 (3.15 to 3.17)
Sub-Saharan Africa	3057 (2806 to 3316)	2292 (2112 to 2490)	13430 (12397 to 14502)	4075 (3770 to 4387)	1.87 (1.86 to 1.88)	338 (281 to 403)	162 (138 to 192)	792 (656 to 959)	160 (134 to 191)	-0.05 (-0.05 to -0.04)	2719 (2469 to 2976)	2130 (1943 to 2326)	12638 (11583 to 13766)	3915 (3608 to 4235)	1.98 (1.97 to 1.99)
**21 regions**															
East Asia	17639 (15843 to 19562)	3509 (3148 to 3895)	56359 (51658 to 61523)	5608 (5104 to 6122)	1.51 (1.49 to 1.54)	425 (349 to 514)	70.5 (58.3 to 84.7)	700 (573 to 861)	83.9 (68.0 to 103)	0.61 (0.57 to 0.64)	17214 (15449 to 19163)	3439 (3076 to 3829)	55658 (51010 to 60817)	5524 (5023 to 6036)	1.53 (1.50 to 1.56)
Oceania	104 (95.4 to 113)	5441 (5052 to 5872)	562 (517 to 615)	11567 (10719 to 12549)	2.45 (2.44 to 2.46)	4.67 (4.04 to 5.37)	162 (141 to 185)	9.70 (8.17 to 11.4)	152 (129 to 178)	-0.18 (-0.19 to -0.17)	98.8 (90.7 to 108)	5279 (4880 to 5718)	553 (507 to 604)	11415 (10561 to 12382)	2.50 (2.49 to 2.52)
Southeast Asia	4349 (4030 to 4697)	2835 (2623 to 3067)	19398 (18010 to 20810)	5378 (5001 to 5779)	2.11 (2.06 to 2.16)	511 (406 to 631)	237 (191 to 291)	864 (694 to 1076)	233 (187 to 290)	-0.01 (-0.04 to 0.02)	3838 (3484 to 4201)	2598 (2382 to 2847)	18534 (17172 to 19935)	5145 (4771 to 5539)	2.25 (2.20 to 2.30)
Central Asia	669 (619 to 742)	2320 (2143 to 2577)	2685 (2522 to 2860)	5478 (5146 to 5830)	2.81 (2.80 to 2.82)	63.5 (53.4 to 74.7)	193 (164 to 227)	138 (119 to 158)	280 (242 to 322)	1.20 (1.19 to 1.20)	605 (554 to 674)	2127 (1948 to 2369)	2547 (2374 to 2727)	5198 (4843 to 5565)	2.93 (2.92 to 2.94)
Central Europe	2487 (2302 to 2692)	3002 (2781 to 3250)	5259 (4852 to 5693)	4697 (4336 to 5103)	1.45 (1.43 to 1.48)	126 (106 to 147)	182 (153 to 214)	186 (157 to 221)	256 (212 to 305)	1.10 (1.09 to 1.11)	2361 (2177 to 2567)	2820 (2597 to 3064)	5072 (4659 to 5503)	4441 (4080 to 4835)	1.47 (1.43 to 1.49)
Eastern Europe	3089 (2795 to 3408)	1877 (1709 to 2058)	6957 (6275 to 7676)	3655 (3314 to 4026)	2.18 (2.17 to 2.18)	230 (193 to 278)	169 (141 to 204)	397 (331 to 486)	285 (235 to 346)	1.74 (1.72 to 1.76)	2859 (2569 to 3174)	1708 (1540 to 1894)	6560 (5889 to 7312)	3370 (3027 to 3748)	2.22 (2.21 to 2.22)
Australasia	246 (227 to 267)	2031 (1879 to 2202)	848 (773 to 917)	3530 (3230 to 3810)	1.81 (1.79 to 1.83)	40.3 (38.0 to 43.2)	364 (342 to 390)	102 (92.4 to 114)	546 (492 to 612)	1.29 (1.26 to 1.31)	206 (187 to 226)	1667 (1508 to 1838)	745 (673 to 819)	2983 (2679 to 3286)	1.92 (1.90 to 1.94)
High-income Asia Pacific	3507 (3192 to 3821)	3192 (2917 to 3473)	11120 (10257 to 12024)	6188 (5723 to 6651)	2.17 (2.15 to 2.19)	190 (154 to 236)	203 (165 to 252)	266 (216 to 330)	244 (196 to 307)	0.57 (0.55 to 0.59)	3317 (3006 to 3654)	2988 (2710 to 3287)	10853 (10004 to 11771)	5944 (5481 to 6425)	2.24 (2.21 to 2.26)
High-income North America	5765 (5313 to 6251)	3209 (2954 to 3479)	23463 (22181 to 24740)	7711 (7291 to 8141)	2.88 (2.86 to 2.90)	744 (649 to 864)	470 (409 to 547)	1340 (1167 to 1555)	590 (514 to 674)	0.72 (0.71 to 0.73)	5021 (4551 to 5557)	2739 (2477 to 3029)	22123 (20942 to 23398)	7122 (6722 to 7551)	3.15 (3.13 to 3.18)
Southern Latin America	718 (670 to 783)	2817 (2626 to 3070)	2572 (2353 to 2792)	5655 (5192 to 6128)	2.26 (2.24 to 2.28)	58.4 (50.4 to 67.7)	232 (200 to 268)	97.0 (79.9 to 115)	258 (211 to 309)	0.36 (0.35 to 0.37)	660 (612 to 725)	2585 (2393 to 2838)	2475 (2256 to 2702)	5397 (4929 to 5878)	2.40 (2.37 to 2.41)
Western Europe	7090 (6597 to 7629)	2465 (2292 to 2644)	17877 (16562 to 19291)	4851 (4486 to 5253)	2.20 (2.18 to 2.21)	750 (667 to 847)	327 (290 to 370)	1536 (1318 to 1797)	544 (464 to 629)	1.63 (1.57 to 1.66)	6340 (5842 to 6860)	2138 (1965 to 2315)	16341 (15053 to 17755)	4307 (3943 to 4707)	2.28 (2.27 to 2.29)
Andean Latin America	291 (269 to 317)	2448 (2264 to 2668)	1547 (1435 to 1649)	4869 (4517 to 5194)	2.24 (2.23 to 2.25)	17.3 (13.9 to 20.6)	103 (83.6 to 122)	39.1 (32.0 to 46.8)	116 (95.4 to 139)	0.44 (0.42 to 0.46)	274 (252 to 299)	2345 (2161 to 2568)	1508 (1399 to 1611)	4753 (4409 to 5084)	2.30 (2.29 to 2.31)
Caribbean	796 (751 to 853)	5552 (5234 to 5960)	2545 (2380 to 2737)	9259 (8654 to 9958)	1.65 (1.65 to 1.66)	53.9 (47.4 to 61.5)	310 (273 to 352)	70.9 (61.3 to 82.7)	284 (245 to 332)	-0.24 (-0.27 to -0.22)	742 (697 to 798)	5242 (4923 to 5635)	2474 (2307 to 2667)	8975 (8350 to 9667)	1.74 (1.73 to 1.75)
Central Latin America	3134 (2895 to 3368)	6050 (5612 to 6536)	11711 (10864 to 12602)	8457 (7853 to 9097)	1.10 (1.06 to 1.13)	125 (97.2 to 162)	162 (128 to 205)	203 (162 to 251)	150 (119 to 185)	-0.22 (-0.24 to -0.19)	3009 (2774 to 3247)	5889 (5443 to 6370)	11507 (10660 to 12397)	8307 (7708 to 8940)	1.13 (1.09 to 1.16)
Tropical Latin America	2212 (2011 to 2434)	4091 (3701 to 4508)	7427 (6725 to 8203)	5310 (4815 to 5856)	0.87 (0.85 to 0.88)	193 (151 to 243)	270 (215 to 334)	425 (341 to 527)	333 (264 to 412)	0.70 (0.68 to 0.71)	2019 (1817 to 2247)	3822 (3431 to 4243)	7002 (6317 to 7787)	4977 (4493 to 5534)	0.87 (0.86 to 0.88)
North Africa and Middle East	3491 (3249 to 3748)	3624 (3355 to 3905)	23506 (21853 to 25197)	9197 (8565 to 9849)	3.04 (3.04 to 3.05)	309 (255 to 368)	225 (189 to 265)	747 (610 to 884)	253 (208 to 300)	0.38 (0.38 to 0.39)	3181 (2932 to 3450)	3399 (3134 to 3678)	22759 (21099 to 24473)	8944 (8310 to 9595)	3.16 (3.15 to 3.17)
South Asia	10262 (9284 to 11248)	2986 (2714 to 3273)	47549 (43178 to 51921)	5646 (5130 to 6156)	2.05 (2.02 to 2.07)	861 (679 to 1094)	183 (144 to 229)	1876 (1484 to 2364)	204 (162 to 257)	0.35 (0.34 to 0.36)	9401 (8440 to 10430)	2803 (2524 to 3098)	45673 (41221 to 50067)	5442 (4920 to 5957)	2.13 (2.11 to 2.15)
Central Sub-Saharan Africa	346 (316 to 378)	2311 (2113 to 2523)	1802 (1639 to 1971)	4466 (4062 to 4845)	2.15 (2.14 to 2.15)	26.8 (22.0 to 32.6)	119 (100 to 145)	71.5 (57.7 to 86.1)	125 (104 to 149)	0.14 (0.13 to 0.14)	320 (288 to 351)	2192 (1991 to 2405)	1731 (1573 to 1898)	4342 (3938 to 4715)	2.23 (2.22 to 2.23)
Eastern Sub-Saharan Africa	885 (813 to 960)	1784 (1639 to 1938)	3279 (3011 to 3552)	2693 (2489 to 2896)	1.33 (1.31 to 1.34)	155 (132 to 180)	179 (153 to 206)	332 (278 to 392)	168 (142 to 196)	-0.18 (-0.20 to -0.16)	730 (660 to 804)	1606 (1454 to 1760)	2947 (2675 to 3217)	2525 (2313 to 2730)	1.46 (1.44 to 1.48)
Southern Sub-Saharan Africa	535 (486 to 586)	3079 (2803 to 3381)	2132 (1954 to 2325)	5976 (5472 to 6530)	2.15 (2.12 to 2.18)	43.4 (34.6 to 55.2)	188 (152 to 234)	77.0 (62.3 to 97.1)	191 (155 to 239)	0.05 (0.05 to 0.06)	491 (444 to 541)	2891 (2620 to 3197)	2055 (1878 to 2251)	5785 (5290 to 6337)	2.25 (2.22 to 2.28)
Western Sub-Saharan Africa	1290 (1181 to 1400)	2452 (2251 to 2660)	6217 (5726 to 6731)	4497 (4155 to 4858)	1.98 (1.96 to 2.00)	112 (90.9 to 139)	148 (122 to 181)	311 (252 to 391)	153 (126 to 187)	0.10 (0.07 to 0.12)	1178 (1069 to 1285)	2304 (2099 to 2512)	5906 (5404 to 6445)	4344 (4004 to 4706)	2.07 (2.05 to 2.09)
**204 countries and territories**															
Afghanistan	212 (190 to 234)	5856 (5254 to 6449)	1199 (1102 to 1318)	15766 (14549 to 17031)	3.25 (3.24 to 3.26)	14.1 (11.8 to 17.6)	324 (272 to 400)	45.4 (35.4 to 56.9)	358 (289 to 440)	0.34 (0.31 to 0.40)	198 (175 to 220)	5532 (4920 to 6139)	1153 (1055 to 1272)	15408 (14201 to 16669)	3.36 (3.35 to 3.37)
Albania	24.3 (22.0 to 26.9)	2144 (1934 to 2386)	72.8 (64.4 to 82.1)	3428 (3051 to 3836)	1.52 (1.50 to 1.53)	1.98 (1.63 to 2.43)	134 (110 to 161)	3.25 (2.64 to 3.94)	208 (168 to 255)	1.42 (1.40 to 1.43)	22.3 (19.9 to 24.9)	2011 (1795 to 2250)	69.5 (60.9 to 78.8)	3220 (2831 to 3645)	1.52 (1.51 to 1.54)
Algeria	294 (266 to 321)	4097 (3694 to 4502)	2027 (1858 to 2184)	10284 (9465 to 11042)	3.01 (3.00 to 3.02)	19.7 (16.2 to 23.6)	188 (157 to 222)	46.5 (38.7 to 56.3)	215 (180 to 260)	0.45 (0.44 to 0.46)	275 (246 to 302)	3910 (3499 to 4328)	1981 (1807 to 2135)	10069 (9227 to 10827)	3.09 (3.09 to 3.10)
American Samoa	1.35 (1.24 to 1.49)	9297 (8544 to 10258)	5.38 (4.94 to 5.84)	21315 (19645 to 23142)	2.70 (2.68 to 2.72)	0.02 (0.02 to 0.03)	115 (94.8 to 135)	0.03 (0.03 to 0.04)	127 (102 to 152)	0.30 (0.25 to 0.33)	1.33 (1.22 to 1.47)	9182 (8436 to 10140)	5.34 (4.91 to 5.81)	21188 (19527 to 23011)	2.72 (2.70 to 2.74)
Andorra	0.58 (0.53 to 0.64)	2007 (1826 to 2196)	2.79 (2.51 to 3.05)	4299 (3885 to 4710)	2.49 (2.48 to 2.50)	0.08 (0.07 to 0.10)	291 (236 to 355)	0.28 (0.23 to 0.34)	522 (425 to 627)	1.89 (1.86 to 1.91)	0.50 (0.44 to 0.55)	1716 (1526 to 1905)	2.50 (2.23 to 2.77)	3778 (3367 to 4206)	2.58 (2.57 to 2.59)
Angola	65.9 (59.8 to 73.4)	2522 (2287 to 2784)	439 (396 to 487)	4776 (4337 to 5257)	2.07 (2.05 to 2.09)	4.96 (4.05 to 6.12)	121 (101 to 147)	17.3 (14.0 to 20.7)	127 (105 to 153)	0.14 (0.13 to 0.15)	60.9 (55.0 to 68.5)	2401 (2167 to 2664)	422 (379 to 471)	4650 (4210 to 5126)	2.14 (2.12 to 2.16)
Antigua and Barbuda	1.87 (1.72 to 2.05)	6478 (5916 to 7176)	5.99 (5.42 to 6.60)	10405 (9458 to 11433)	1.52 (1.49 to 1.54)	0.13 (0.11 to 0.15)	413 (347 to 492)	0.16 (0.13 to 0.20)	317 (262 to 388)	-0.80 (-0.84 to -0.77)	1.74 (1.59 to 1.92)	6065 (5502 to 6757)	5.83 (5.26 to 6.44)	10088 (9134 to 11128)	1.63 (1.60 to 1.65)
Argentina	515 (475 to 559)	2902 (2680 to 3153)	1584 (1421 to 1747)	5403 (4846 to 5951)	2.03 (2.02 to 2.04)	43.0 (36.9 to 50.4)	256 (219 to 299)	66.3 (54.2 to 78.8)	265 (216 to 318)	0.14 (0.12 to 0.16)	472 (433 to 516)	2647 (2426 to 2895)	1518 (1357 to 1681)	5138 (4577 to 5692)	2.16 (2.15 to 2.17)
Armenia	50.3 (45.9 to 55.5)	3089 (2828 to 3408)	114 (104 to 128)	4978 (4520 to 5548)	1.54 (1.52 to 1.56)	3.80 (3.14 to 4.49)	220 (183 to 259)	5.04 (4.20 to 6.09)	280 (231 to 337)	0.80 (0.78 to 0.83)	46.5 (41.8 to 51.7)	2869 (2594 to 3190)	109 (97.7 to 123)	4698 (4226 to 5261)	1.59 (1.57 to 1.62)
Australia	191 (175 to 208)	1883 (1720 to 2063)	689 (620 to 757)	3405 (3082 to 3722)	1.93 (1.91 to 1.94)	34.2 (32.5 to 36.1)	371 (353 to 392)	92.2 (83.8 to 103)	590 (534 to 662)	1.48 (1.45 to 1.50)	157 (140 to 174)	1512 (1351 to 1690)	597 (527 to 666)	2815 (2484 to 3142)	2.03 (2.02 to 2.04)
Austria	85.7 (79.8 to 92.8)	1401 (1289 to 1524)	226 (208 to 247)	2999 (2743 to 3254)	2.47 (2.46 to 2.49)	12.6 (11.5 to 13.9)	266 (241 to 295)	29.2 (26.5 to 32.0)	508 (455 to 563)	2.10 (2.09 to 2.11)	73.1 (67.0 to 80.5)	1134 (1025 to 1256)	197 (178 to 218)	2490 (2231 to 2774)	2.56 (2.54 to 2.57)
Azerbaijan	72.9 (66.8 to 80.4)	2326 (2125 to 2562)	359 (330 to 393)	5854 (5386 to 6370)	3.03 (3.01 to 3.04)	7.48 (6.26 to 8.99)	207 (174 to 248)	16.8 (14.1 to 19.9)	291 (244 to 344)	1.11 (1.10 to 1.12)	65.5 (58.6 to 72.8)	2119 (1896 to 2352)	342 (313 to 375)	5563 (5102 to 6070)	3.17 (3.15 to 3.19)
Bahamas	5.65 (5.14 to 6.15)	5797 (5273 to 6300)	22.2 (20.1 to 24.5)	9541 (8651 to 10514)	1.61 (1.60 to 1.62)	0.42 (0.35 to 0.50)	324 (275 to 385)	0.58 (0.48 to 0.68)	260 (217 to 308)	-0.64 (-0.69 to -0.61)	5.23 (4.72 to 5.70)	5473 (4957 to 5983)	21.7 (19.5 to 23.9)	9281 (8406 to 10248)	1.70 (1.69 to 1.71)
Bahrain	6.65 (6.02 to 7.26)	6792 (6173 to 7387)	67.9 (62.8 to 73.4)	14697 (13724 to 15724)	2.53 (2.50 to 2.55)	0.48 (0.38 to 0.59)	263 (213 to 317)	1.66 (1.33 to 2.03)	286 (229 to 347)	0.28 (0.27 to 0.29)	6.18 (5.54 to 6.79)	6529 (5916 to 7136)	66.3 (61.1 to 71.8)	14412 (13440 to 15432)	2.59 (2.57 to 2.62)
Bangladesh	918 (845 to 993)	3139 (2914 to 3399)	5451 (5029 to 5938)	7069 (6518 to 7664)	2.65 (2.62 to 2.68)	118 (98.2 to 139)	254 (213 to 296)	234 (192 to 276)	275 (228 to 324)	0.29 (0.26 to 0.31)	800 (726 to 879)	2885 (2647 to 3135)	5218 (4804 to 5711)	6794 (6256 to 7396)	2.80 (2.77 to 2.83)
Barbados	10.1 (9.35 to 11.0)	6473 (5973 to 7182)	25.1 (23.0 to 27.1)	10014 (9182 to 10840)	1.41 (1.36 to 1.45)	0.60 (0.50 to 0.69)	435 (362 to 506)	0.61 (0.51 to 0.74)	347 (287 to 425)	-0.70 (-0.73 to -0.65)	9.46 (8.78 to 10.5)	6038 (5545 to 6756)	24.5 (22.4 to 26.5)	9667 (8851 to 10473)	1.52 (1.49 to 1.56)
Belarus	118 (107 to 131)	1577 (1428 to 1741)	223 (199 to 247)	2595 (2329 to 2859)	1.61 (1.60 to 1.63)	8.39 (7.15 to 10.1)	137 (116 to 166)	12.1 (9.96 to 14.8)	194 (158 to 240)	1.14 (1.12 to 1.17)	109 (98.1 to 123)	1440 (1284 to 1604)	211 (186 to 235)	2401 (2136 to 2671)	1.66 (1.63 to 1.67)
Belgium	177 (161 to 194)	2379 (2164 to 2601)	426 (378 to 475)	4566 (4080 to 5065)	2.11 (2.08 to 2.13)	15.8 (13.2 to 19.1)	269 (222 to 324)	28.8 (23.4 to 35.7)	403 (323 to 503)	1.32 (1.31 to 1.33)	161 (144 to 178)	2110 (1888 to 2336)	397 (349 to 447)	4163 (3678 to 4690)	2.20 (2.17 to 2.22)
Belize	2.79 (2.57 to 3.05)	5392 (4944 to 5933)	16.6 (15.0 to 18.6)	9497 (8582 to 10629)	1.82 (1.79 to 1.84)	0.27 (0.23 to 0.32)	339 (293 to 397)	0.60 (0.50 to 0.72)	275 (232 to 325)	-0.62 (-0.66 to -0.55)	2.52 (2.30 to 2.78)	5053 (4596 to 5588)	16.0 (14.3 to 18.0)	9222 (8314 to 10352)	1.93 (1.90 to 1.95)
Benin	37.9 (34.6 to 41.7)	2916 (2646 to 3228)	251 (229 to 274)	6621 (6017 to 7254)	2.70 (2.64 to 2.75)	2.99 (2.45 to 3.61)	155 (128 to 185)	8.36 (6.87 to 10.2)	151 (126 to 181)	-0.07 (-0.08 to -0.07)	34.9 (31.4 to 38.7)	2761 (2494 to 3066)	242 (220 to 266)	6469 (5861 to 7100)	2.80 (2.74 to 2.86)
Bermuda	1.21 (1.10 to 1.32)	3423 (3135 to 3737)	3.42 (3.08 to 3.81)	5753 (5187 to 6414)	1.68 (1.67 to 1.69)	0.08 (0.06 to 0.10)	236 (191 to 290)	0.09 (0.07 to 0.11)	211 (173 to 253)	-0.32 (-0.35 to -0.29)	1.13 (1.02 to 1.24)	3186 (2892 to 3515)	3.34 (3.00 to 3.73)	5542 (4984 to 6213)	1.79 (1.78 to 1.80)
Bhutan	4.44 (3.97 to 4.90)	2703 (2418 to 2969)	16.4 (14.8 to 17.8)	4918 (4459 to 5328)	1.96 (1.93 to 1.98)	0.42 (0.34 to 0.50)	162 (135 to 190)	0.66 (0.54 to 0.80)	177 (146 to 215)	0.29 (0.29 to 0.29)	4.02 (3.57 to 4.48)	2541 (2254 to 2797)	15.7 (14.2 to 17.1)	4741 (4295 to 5154)	2.04 (2.02 to 2.06)
Bolivia (Plurinational State of)	57.6 (52.6 to 62.5)	2992 (2714 to 3244)	301 (273 to 331)	5870 (5334 to 6456)	2.20 (2.18 to 2.22)	4.16 (3.29 to 5.02)	149 (119 to 176)	8.84 (6.98 to 10.7)	151 (120 to 182)	0.09 (0.06 to 0.11)	53.5 (48.3 to 58.4)	2843 (2569 to 3101)	292 (264 to 322)	5719 (5176 to 6307)	2.28 (2.26 to 2.30)
Bosnia and Herzegovina	73.5 (66.1 to 81.0)	3102 (2791 to 3399)	215 (197 to 233)	6623 (6073 to 7179)	2.48 (2.46 to 2.49)	5.03 (4.18 to 5.97)	211 (174 to 250)	5.64 (4.80 to 6.64)	279 (233 to 331)	0.91 (0.90 to 0.93)	68.5 (60.8 to 75.7)	2891 (2564 to 3185)	209 (191 to 228)	6343 (5794 to 6907)	2.58 (2.56 to 2.59)
Botswana	8.49 (7.75 to 9.41)	2350 (2130 to 2590)	46.6 (43.0 to 50.3)	4925 (4541 to 5319)	2.41 (2.38 to 2.43)	1.16 (0.92 to 1.42)	212 (173 to 256)	2.55 (2.10 to 3.10)	217 (181 to 260)	0.11 (0.08 to 0.13)	7.33 (6.53 to 8.20)	2138 (1929 to 2382)	44.1 (40.3 to 47.8)	4708 (4307 to 5108)	2.59 (2.55 to 2.62)
Brazil	2166 (1967 to 2386)	4103 (3707 to 4528)	7216 (6519 to 7981)	5281 (4776 to 5832)	0.84 (0.83 to 0.85)	189 (149 to 239)	272 (216 to 337)	417 (334 to 518)	336 (267 to 417)	0.70 (0.69 to 0.71)	1977 (1776 to 2205)	3831 (3440 to 4259)	6799 (6120 to 7570)	4944 (4452 to 5504)	0.84 (0.83 to 0.85)
Brunei Darussalam	3.71 (3.44 to 3.98)	6017 (5546 to 6435)	26.1 (24.2 to 28.2)	12520 (11578 to 13527)	2.38 (2.36 to 2.39)	0.59 (0.52 to 0.67)	504 (445 to 571)	1.08 (0.92 to 1.24)	474 (405 to 547)	-0.17 (-0.20 to -0.14)	3.11 (2.85 to 3.40)	5513 (5039 to 5937)	25.0 (23.0 to 27.1)	12046 (11127 to 13055)	2.54 (2.52 to 2.55)
Bulgaria	196 (176 to 221)	3033 (2739 to 3405)	375 (335 to 410)	5271 (4775 to 5755)	1.81 (1.79 to 1.84)	13.4 (10.9 to 16.2)	272 (220 to 330)	16.2 (13.0 to 19.1)	372 (295 to 447)	1.00 (0.98 to 1.02)	183 (163 to 208)	2760 (2473 to 3131)	359 (320 to 393)	4899 (4390 to 5397)	1.89 (1.86 to 1.91)
Burkina Faso	50.5 (46.2 to 55.2)	1874 (1714 to 2053)	273 (250 to 299)	4234 (3872 to 4627)	2.69 (2.67 to 2.70)	5.75 (4.64 to 7.02)	149 (123 to 181)	13.6 (11.1 to 16.5)	144 (118 to 173)	-0.13 (-0.14 to -0.12)	44.8 (40.0 to 49.5)	1725 (1558 to 1907)	259 (236 to 285)	4090 (3723 to 4479)	2.85 (2.83 to 2.86)
Burundi	28.2 (25.9 to 30.8)	1784 (1630 to 1957)	91.6 (83.3 to 101)	2702 (2473 to 2946)	1.34 (1.33 to 1.35)	4.68 (3.95 to 5.48)	182 (158 to 210)	9.75 (8.28 to 11.5)	164 (140 to 192)	-0.28 (-0.31 to -0.23)	23.5 (21.0 to 26.2)	1602 (1446 to 1781)	81.8 (73.7 to 90.7)	2538 (2306 to 2779)	1.48 (1.47 to 1.50)
Cabo Verde	4.00 (3.66 to 4.37)	2961 (2702 to 3247)	17.7 (16.0 to 19.3)	6654 (6041 to 7241)	2.65 (2.64 to 2.67)	0.21 (0.18 to 0.26)	138 (115 to 165)	0.41 (0.34 to 0.49)	145 (122 to 173)	0.17 (0.15 to 0.19)	3.78 (3.44 to 4.16)	2824 (2564 to 3109)	17.3 (15.6 to 18.9)	6509 (5897 to 7100)	2.74 (2.73 to 2.75)
Cambodia	62.4 (57.2 to 68.5)	2049 (1871 to 2230)	386 (353 to 423)	5130 (4669 to 5613)	3.01 (2.99 to 3.03)	9.13 (7.49 to 10.9)	197 (165 to 235)	16.4 (13.4 to 19.7)	187 (154 to 225)	-0.10 (-0.14 to -0.06)	53.3 (48.1 to 59.1)	1852 (1681 to 2034)	370 (336 to 406)	4942 (4483 to 5423)	3.22 (3.20 to 3.24)
Cameroon	64.3 (58.3 to 70.3)	2254 (2047 to 2458)	431 (394 to 473)	4827 (4443 to 5247)	2.51 (2.49 to 2.54)	6.74 (5.57 to 8.10)	160 (134 to 190)	21.4 (17.9 to 25.8)	162 (138 to 195)	0.04 (0.04 to 0.05)	57.5 (51.3 to 63.2)	2094 (1887 to 2303)	409 (372 to 453)	4665 (4274 to 5086)	2.65 (2.62 to 2.68)
Canada	345 (321 to 370)	2027 (1880 to 2175)	1795 (1590 to 2007)	5554 (4995 to 6149)	3.30 (3.27 to 3.31)	122 (116 to 130)	798 (758 to 847)	320 (297 to 344)	1366 (1260 to 1468)	1.75 (1.73 to 1.77)	223 (198 to 250)	1228 (1078 to 1388)	1475 (1271 to 1684)	4188 (3617 to 4811)	4.02 (3.99 to 4.04)
Central African Republic	25.6 (22.8 to 28.3)	3279 (2961 to 3614)	111 (100 to 122)	6456 (5861 to 7063)	2.20 (2.17 to 2.23)	1.63 (1.33 to 1.99)	141 (116 to 168)	3.41 (2.79 to 4.14)	141 (116 to 169)	-0.01 (-0.02 to 0.00)	23.9 (21.3 to 26.6)	3138 (2810 to 3476)	107 (96.7 to 119)	6315 (5720 to 6919)	2.26 (2.23 to 2.28)

Location	DM, Number in 1990 (000s)	DM, ASR in 1990 (per 100,000)	DM, Number in 2021 (000s)	DM, ASR in 2021 (per 100,000)	DM, AAPC (95%CI)	T1D, Number in 1990 (000s)	T1D, ASR in 1990 (per 100,000)	T1D, Number in 2021 (000s)	T1D, ASR in 2021 (per 100,000)	T1D, AAPC (95%CI)	T2D, Number in 1990 (000s)	T2D, ASR in 1990 (per 100,000)	T2D, Number in 2021 (000s)	T2D, ASR in 2021 (per 100,000)	T2D, AAPC (95%CI)
Chad	40.7 (37.2 to 44.6)	2344 (2134 to 2570)	198 (179 to 217)	5012 (4558 to 5514)	2.49 (2.47 to 2.49)	3.58 (2.88 to 4.40)	148 (121 to 181)	9.86 (8.01 to 12.2)	148 (122 to 180)	-0.00 (-0.01 to 0.01)	37.1 (33.5 to 41.0)	2196 (1984 to 2416)	188 (169 to 207)	4864 (4404 to 5358)	2.60 (2.59 to 2.61)
Chile	160 (144 to 180)	2803 (2518 to 3141)	852 (773 to 949)	6312 (5707 to 6999)	2.58 (2.49 to 2.62)	10.5 (8.72 to 12.6)	158 (131 to 189)	23.8 (19.3 to 28.7)	224 (181 to 273)	1.13 (1.12 to 1.13)	150 (133 to 169)	2644 (2350 to 2983)	828 (748 to 925)	6087 (5486 to 6760)	2.64 (2.56 to 2.69)
China	17036 (15280 to 18925)	3522 (3151 to 3916)	54282 (49667 to 59341)	5614 (5101 to 6141)	1.51 (1.48 to 1.54)	395 (325 to 479)	67.9 (56.1 to 81.6)	655 (532 to 810)	81.4 (65.6 to 101)	0.63 (0.60 to 0.67)	16640 (14909 to 18552)	3454 (3083 to 3853)	53628 (49038 to 58691)	5533 (5022 to 6057)	1.53 (1.50 to 1.56)
Colombia	451 (410 to 498)	4125 (3743 to 4519)	1765 (1616 to 1928)	5980 (5472 to 6525)	1.26 (1.17 to 1.38)	13.1 (10.7 to 16.0)	85.3 (69.8 to 103)	23.2 (18.9 to 28.7)	86.0 (69.8 to 106)	0.04 (0.02 to 0.05)	438 (397 to 483)	4040 (3657 to 4435)	1742 (1595 to 1904)	5894 (5396 to 6438)	1.28 (1.19 to 1.40)
Comoros	2.80 (2.57 to 3.05)	2247 (2047 to 2476)	12.1 (11.2 to 13.2)	4011 (3686 to 4342)	1.87 (1.85 to 1.88)	0.37 (0.31 to 0.44)	177 (154 to 206)	0.64 (0.55 to 0.75)	175 (150 to 203)	-0.00 (-0.03 to 0.02)	2.43 (2.21 to 2.68)	2070 (1877 to 2291)	11.5 (10.5 to 12.5)	3836 (3507 to 4168)	1.99 (1.97 to 2.01)
Congo	17.7 (15.9 to 19.4)	2514 (2263 to 2744)	91.9 (82.0 to 100)	4983 (4515 to 5429)	2.23 (2.20 to 2.25)	1.59 (1.30 to 1.95)	156 (128 to 187)	3.89 (3.22 to 4.64)	157 (132 to 188)	0.04 (0.03 to 0.07)	16.1 (14.2 to 17.8)	2358 (2104 to 2590)	88.0 (78.1 to 96.5)	4826 (4360 to 5262)	2.33 (2.30 to 2.35)
Cook Islands	0.71 (0.66 to 0.77)	10468 (9728 to 11327)	2.28 (2.10 to 2.45)	19183 (17596 to 20685)	1.97 (1.95 to 1.98)	0.02 (0.01 to 0.02)	178 (148 to 209)	0.02 (0.01 to 0.02)	168 (139 to 200)	-0.19 (-0.20 to -0.18)	0.70 (0.65 to 0.76)	10290 (9549 to 11130)	2.26 (2.08 to 2.43)	19015 (17422 to 20528)	2.00 (1.98 to 2.01)
Costa Rica	42.9 (38.8 to 46.9)	4146 (3747 to 4576)	216 (196 to 240)	7390 (6714 to 8237)	1.89 (1.83 to 1.96)	1.16 (0.94 to 1.40)	83.3 (67.9 to 101)	2.20 (1.77 to 2.71)	82.5 (66.2 to 102)	-0.02 (-0.03 to -0.01)	41.7 (37.7 to 45.9)	4062 (3664 to 4489)	213 (194 to 238)	7308 (6631 to 8155)	1.92 (1.86 to 1.99)
Croatia	104 (92.6 to 117)	2936 (2619 to 3310)	209 (185 to 233)	4744 (4243 to 5320)	1.56 (1.53 to 1.58)	5.33 (4.74 to 5.96)	188 (166 to 211)	9.49 (7.80 to 11.3)	338 (277 to 401)	1.91 (1.89 to 1.93)	98.4 (87.1 to 112)	2748 (2435 to 3125)	199 (176 to 222)	4406 (3881 to 4961)	1.53 (1.49 to 1.56)
Cuba	222 (202 to 245)	4154 (3802 to 4591)	580 (521 to 638)	6471 (5823 to 7111)	1.42 (1.39 to 1.45)	10.2 (8.58 to 12.3)	181 (151 to 217)	8.48 (6.94 to 10.3)	130 (106 to 159)	-1.00 (-1.05 to -0.93)	211 (193 to 234)	3973 (3624 to 4413)	571 (513 to 629)	6341 (5692 to 6987)	1.50 (1.47 to 1.52)
Cyprus	19.5 (18.3 to 20.9)	4266 (3989 to 4581)	56.1 (51.0 to 61.3)	5557 (5037 to 6070)	0.85 (0.84 to 0.87)	1.15 (1.04 to 1.27)	282 (256 to 312)	5.69 (5.10 to 6.40)	685 (611 to 776)	2.92 (2.89 to 2.96)	18.3 (17.1 to 19.7)	3984 (3700 to 4300)	50.4 (45.2 to 55.5)	4872 (4372 to 5413)	0.65 (0.64 to 0.66)
Czechia	221 (200 to 245)	2894 (2629 to 3203)	535 (472 to 595)	5083 (4533 to 5650)	1.81 (1.78 to 1.83)	12.1 (10.1 to 14.3)	204 (168 to 240)	20.6 (17.1 to 24.6)	300 (245 to 360)	1.24 (1.22 to 1.26)	209 (188 to 233)	2690 (2416 to 3002)	514 (452 to 573)	4783 (4227 to 5347)	1.87 (1.83 to 1.89)
Côte d'Ivoire	70.7 (64.1 to 78.2)	2590 (2330 to 2835)	411 (370 to 446)	5472 (4945 to 5938)	2.45 (2.43 to 2.46)	7.08 (5.75 to 8.46)	154 (128 to 185)	17.8 (14.6 to 21.7)	159 (130 to 193)	0.09 (0.08 to 0.11)	63.6 (56.8 to 71.1)	2436 (2179 to 2682)	393 (353 to 428)	5314 (4790 to 5781)	2.55 (2.54 to 2.57)
Democratic People's Republic of Korea	288 (262 to 318)	2755 (2504 to 3034)	946 (862 to 1047)	5318 (4845 to 5856)	2.14 (2.13 to 2.15)	9.88 (8.18 to 11.7)	89.6 (74.3 to 106)	14.6 (12.1 to 17.6)	99.3 (81.0 to 120)	0.33 (0.32 to 0.34)	279 (252 to 309)	2665 (2414 to 2944)	932 (849 to 1033)	5218 (4738 to 5760)	2.19 (2.18 to 2.20)
Democratic Republic of the Congo	225 (204 to 249)	2155 (1970 to 2373)	1102 (990 to 1216)	4156 (3743 to 4570)	2.14 (2.13 to 2.15)	17.9 (14.5 to 21.6)	114 (95.7 to 141)	44.9 (36.3 to 53.9)	120 (100 to 144)	0.15 (0.14 to 0.16)	207 (186 to 230)	2040 (1849 to 2251)	1057 (947 to 1170)	4036 (3618 to 4445)	2.22 (2.21 to 2.23)
Denmark	60.1 (55.9 to 64.8)	1646 (1517 to 1776)	169 (154 to 187)	3583 (3249 to 3983)	2.53 (2.52 to 2.54)	10.7 (9.14 to 12.6)	352 (300 to 415)	17.7 (14.8 to 21.2)	492 (401 to 598)	1.11 (1.09 to 1.14)	49.4 (44.8 to 54.6)	1294 (1161 to 1436)	151 (135 to 170)	3091 (2728 to 3471)	2.84 (2.82 to 2.86)
Djibouti	1.49 (1.34 to 1.63)	1541 (1397 to 1685)	10.9 (10.0 to 11.9)	2760 (2524 to 3008)	1.90 (1.88 to 1.91)	0.33 (0.28 to 0.39)	179 (152 to 209)	0.95 (0.81 to 1.13)	167 (143 to 198)	-0.15 (-0.19 to -0.11)	1.16 (1.00 to 1.30)	1362 (1213 to 1515)	9.98 (9.03 to 11.0)	2593 (2359 to 2838)	2.10 (2.08 to 2.11)
Dominica	2.38 (2.21 to 2.63)	7011 (6480 to 7774)	4.65 (4.18 to 5.10)	11271 (10158 to 12383)	1.54 (1.53 to 1.55)	0.12 (0.10 to 0.14)	341 (290 to 406)	0.10 (0.09 to 0.12)	281 (235 to 330)	-0.58 (-0.63 to -0.51)	2.26 (2.09 to 2.51)	6670 (6143 to 7440)	4.55 (4.08 to 5.00)	10990 (9874 to 12106)	1.62 (1.60 to 1.63)
Dominican Republic	99.1 (90.1 to 110)	4354 (3925 to 4901)	468 (422 to 513)	8727 (7865 to 9583)	2.25 (2.22 to 2.27)	8.06 (6.56 to 9.85)	241 (196 to 290)	13.1 (10.9 to 15.7)	235 (196 to 281)	-0.09 (-0.10 to -0.08)	91.1 (81.9 to 102)	4113 (3673 to 4651)	454 (408 to 501)	8492 (7628 to 9364)	2.34 (2.31 to 2.37)
Ecuador	96.9 (88.8 to 107)	3173 (2895 to 3517)	621 (564 to 673)	7075 (6423 to 7661)	2.63 (2.61 to 2.64)	4.82 (3.89 to 5.83)	108 (88.4 to 129)	10.9 (8.90 to 13.2)	119 (97.4 to 143)	0.32 (0.28 to 0.34)	92.1 (83.7 to 102)	3064 (2783 to 3420)	611 (554 to 662)	6956 (6304 to 7536)	2.68 (2.67 to 2.70)
Egypt	374 (340 to 407)	2322 (2130 to 2536)	3031 (2735 to 3355)	8504 (7668 to 9411)	4.28 (4.26 to 4.30)	51.3 (42.6 to 61.2)	230 (193 to 272)	117 (94.3 to 145)	249 (203 to 305)	0.22 (0.19 to 0.26)	323 (290 to 357)	2093 (1894 to 2305)	2914 (2618 to 3241)	8254 (7428 to 9159)	4.55 (4.52 to 4.57)
El Salvador	55.4 (50.5 to 61.0)	3060 (2782 to 3405)	231 (208 to 256)	6541 (5901 to 7255)	2.48 (2.42 to 2.54)	3.07 (2.51 to 3.66)	123 (103 to 145)	4.82 (4.10 to 5.67)	137 (117 to 162)	0.36 (0.34 to 0.38)	52.3 (47.2 to 58.0)	2936 (2656 to 3280)	226 (204 to 251)	6403 (5772 to 7120)	2.54 (2.48 to 2.60)
Equatorial Guinea	3.19 (2.86 to 3.53)	2473 (2239 to 2725)	22.6 (20.5 to 24.8)	5567 (5092 to 6084)	2.65 (2.62 to 2.67)	0.21 (0.18 to 0.26)	118 (99.6 to 142)	0.82 (0.66 to 1.00)	138 (113 to 167)	0.50 (0.49 to 0.51)	2.97 (2.64 to 3.31)	2355 (2121 to 2608)	21.8 (19.7 to 23.9)	5429 (4948 to 5957)	2.72 (2.69 to 2.75)
Eritrea	15.9 (14.4 to 17.4)	1862 (1693 to 2046)	73.0 (66.5 to 79.4)	3508 (3201 to 3787)	2.04 (2.02 to 2.05)	2.90 (2.47 to 3.46)	187 (160 to 218)	5.72 (4.86 to 6.81)	186 (160 to 219)	0.00 (-0.02 to 0.04)	13.0 (11.4 to 14.6)	1675 (1505 to 1854)	67.3 (60.7 to 73.8)	3321 (3028 to 3613)	2.20 (2.19 to 2.22)
Estonia	22.9 (20.7 to 25.6)	1943 (1757 to 2176)	50.7 (45.1 to 56.8)	3975 (3525 to 4459)	2.32 (2.30 to 2.34)	2.17 (1.98 to 2.43)	237 (215 to 264)	3.13 (2.62 to 3.78)	349 (287 to 425)	1.30 (1.28 to 1.32)	20.7 (18.5 to 23.4)	1706 (1522 to 1932)	47.6 (41.8 to 53.6)	3626 (3181 to 4110)	2.43 (2.39 to 2.46)
Eswatini	5.89 (5.34 to 6.49)	2984 (2711 to 3300)	25.1 (23.2 to 27.2)	6594 (6054 to 7155)	2.59 (2.56 to 2.61)	0.80 (0.64 to 1.00)	246 (205 to 302)	1.33 (1.07 to 1.65)	254 (209 to 309)	0.11 (0.11 to 0.11)	5.09 (4.57 to 5.71)	2738 (2472 to 3058)	23.8 (21.7 to 25.8)	6340 (5788 to 6888)	2.74 (2.71 to 2.77)
Ethiopia	324 (292 to 355)	2515 (2266 to 2764)	978 (875 to 1087)	3194 (2880 to 3488)	0.77 (0.75 to 0.78)	45.8 (39.3 to 52.9)	196 (170 to 225)	82.0 (69.2 to 98.1)	165 (141 to 192)	-0.45 (-0.51 to -0.40)	278 (247 to 307)	2319 (2057 to 2565)	896 (795 to 1004)	3029 (2698 to 3326)	0.85 (0.84 to 0.87)
Fiji	19.2 (17.6 to 21.2)	8177 (7547 to 8973)	72.0 (65.3 to 79.0)	16271 (14865 to 17769)	2.24 (2.22 to 2.25)	1.16 (1.02 to 1.32)	319 (278 to 364)	1.20 (1.02 to 1.43)	261 (220 to 311)	-0.59 (-0.66 to -0.49)	18.1 (16.4 to 19.9)	7858 (7219 to 8664)	70.8 (64.2 to 77.7)	16011 (14612 to 17482)	2.31 (2.30 to 2.33)
Finland	116 (106 to 127)	3252 (2968 to 3549)	290 (265 to 316)	6262 (5707 to 6832)	2.14 (2.13 to 2.15)	26.1 (25.2 to 27.0)	876 (846 to 908)	45.8 (42.8 to 49.6)	1308 (1225 to 1414)	1.31 (1.27 to 1.34)	90.2 (80.1 to 101)	2376 (2090 to 2679)	244 (218 to 271)	4954 (4376 to 5563)	2.40 (2.38 to 2.41)
France	663 (611 to 714)	1589 (1459 to 1719)	1634 (1475 to 1786)	2955 (2672 to 3252)	2.03 (2.00 to 2.06)	72.8 (66.2 to 79.7)	220 (199 to 242)	169 (147 to 195)	411 (353 to 477)	2.05 (2.04 to 2.07)	590 (538 to 642)	1369 (1236 to 1495)	1465 (1304 to 1614)	2544 (2250 to 2843)	2.03 (2.00 to 2.06)
Gabon	8.92 (8.11 to 9.77)	2673 (2440 to 2936)	36.7 (33.5 to 40.4)	5484 (5046 to 6005)	2.35 (2.33 to 2.36)	0.60 (0.49 to 0.71)	142 (119 to 167)	1.26 (1.04 to 1.52)	146 (122 to 176)	0.12 (0.10 to 0.14)	8.33 (7.52 to 9.16)	2532 (2301 to 2790)	35.4 (32.2 to 39.2)	5337 (4900 to 5868)	2.44 (2.42 to 2.45)
Gambia	5.37 (4.87 to 5.88)	2336 (2122 to 2561)	34.6 (31.5 to 37.7)	5149 (4711 to 5627)	2.59 (2.58 to 2.59)	0.56 (0.46 to 0.69)	151 (125 to 181)	1.56 (1.27 to 1.92)	155 (129 to 189)	0.09 (0.07 to 0.10)	4.81 (4.30 to 5.34)	2185 (1972 to 2409)	33.0 (30.0 to 36.2)	4994 (4550 to 5470)	2.71 (2.70 to 2.72)
Georgia	79.2 (71.9 to 87.3)	2233 (2034 to 2459)	160 (144 to 180)	5401 (4897 to 6018)	2.88 (2.85 to 2.91)	6.15 (5.17 to 7.36)	197 (165 to 237)	6.22 (5.25 to 7.32)	283 (235 to 335)	1.15 (1.12 to 1.17)	73.0 (66.1 to 81.5)	2036 (1839 to 2267)	154 (137 to 173)	5118 (4628 to 5724)	3.02 (2.97 to 3.06)
Germany	1125 (1030 to 1215)	1737 (1594 to 1878)	3260 (2963 to 3622)	4265 (3876 to 4729)	2.99 (2.96 to 3.02)	131 (118 to 145)	273 (245 to 304)	262 (218 to 308)	489 (406 to 581)	1.89 (1.87 to 1.91)	993 (901 to 1086)	1463 (1323 to 1605)	2998 (2705 to 3364)	3776 (3391 to 4237)	3.16 (3.12 to 3.19)
Ghana	108 (97.6 to 119)	2558 (2335 to 2796)	602 (539 to 657)	5038 (4536 to 5512)	2.22 (2.20 to 2.23)	8.74 (7.29 to 10.3)	143 (121 to 168)	23.1 (19.4 to 27.8)	147 (125 to 177)	0.08 (0.07 to 0.10)	98.8 (88.5 to 109)	2415 (2193 to 2649)	579 (516 to 635)	4890 (4395 to 5359)	2.31 (2.29 to 2.32)
Greece	197 (176 to 217)	2691 (2418 to 2969)	410 (371 to 454)	4625 (4193 to 5117)	1.74 (1.72 to 1.76)	11.2 (9.65 to 13.0)	188 (162 to 219)	25.0 (20.6 to 30.4)	370 (300 to 452)	2.19 (2.17 to 2.21)	185 (165 to 206)	2503 (2229 to 2775)	385 (347 to 428)	4255 (3822 to 4753)	1.70 (1.67 to 1.73)
Greenland	0.20 (0.18 to 0.21)	996 (906 to 1087)	1.01 (0.91 to 1.10)	3080 (2824 to 3366)	3.71 (3.69 to 3.73)	0.09 (0.07 to 0.10)	351 (291 to 427)	0.12 (0.09 to 0.14)	389 (320 to 475)	0.33 (0.32 to 0.34)	0.11 (0.09 to 0.13)	644 (548 to 740)	0.89 (0.79 to 1.00)	2690 (2410 to 2997)	4.73 (4.71 to 4.74)
Grenada	2.73 (2.52 to 2.95)	7189 (6576 to 7791)	6.93 (6.16 to 7.62)	11642 (10405 to 12767)	1.53 (1.50 to 1.56)	0.13 (0.11 to 0.15)	319 (270 to 381)	0.13 (0.11 to 0.16)	250 (206 to 296)	-0.76 (-0.78 to -0.73)	2.60 (2.39 to 2.81)	6870 (6253 to 7486)	6.80 (6.01 to 7.47)	11392 (10114 to 12520)	1.61 (1.57 to 1.63)
Guam	2.32 (2.12 to 2.54)	4866 (4448 to 5310)	8.29 (7.56 to 9.10)	8420 (7672 to 9224)	1.78 (1.77 to 1.79)	0.06 (0.05 to 0.07)	100 (81.4 to 122)	0.09 (0.07 to 0.11)	104 (85.1 to 130)	0.12 (0.09 to 0.13)	2.26 (2.06 to 2.47)	4766 (4346 to 5212)	8.20 (7.47 to 9.00)	8316 (7569 to 9108)	1.81 (1.80 to 1.81)
Guatemala	83.3 (76.1 to 91.0)	3733 (3412 to 4096)	625 (568 to 687)	9463 (8609 to 10405)	3.11 (3.06 to 3.17)	6.42 (5.07 to 7.90)	178 (143 to 214)	23.6 (20.0 to 27.6)	285 (243 to 333)	1.50 (1.40 to 1.56)	76.9 (69.5 to 84.8)	3555 (3228 to 3913)	601 (542 to 664)	9178 (8305 to 10111)	3.17 (3.12 to 3.24)
Guinea	43.0 (39.2 to 46.7)	2247 (2054 to 2463)	169 (155 to 185)	4477 (4153 to 4868)	2.24 (2.22 to 2.26)	4.32 (3.53 to 5.23)	171 (141 to 208)	9.65 (7.76 to 11.8)	169 (139 to 206)	-0.04 (-0.04 to -0.03)	38.6 (34.9 to 42.5)	2076 (1883 to 2297)	159 (145 to 175)	4308 (3984 to 4699)	2.38 (2.36 to 2.39)
Guinea-Bissau	8.14 (7.33 to 8.95)	2992 (2707 to 3280)	35.2 (32.5 to 38.6)	6133 (5679 to 6675)	2.36 (2.35 to 2.37)	0.78 (0.64 to 0.94)	188 (157 to 221)	1.64 (1.33 to 2.00)	185 (153 to 223)	-0.04 (-0.05 to -0.04)	7.36 (6.54 to 8.17)	2804 (2516 to 3089)	33.6 (30.9 to 37.0)	5948 (5487 to 6493)	2.47 (2.46 to 2.48)
Guyana	23.4 (21.2 to 25.5)	9907 (8991 to 10899)	61.1 (56.3 to 66.9)	16363 (15099 to 17895)	1.63 (1.62 to 1.64)	1.72 (1.48 to 1.96)	466 (405 to 532)	1.50 (1.27 to 1.80)	378 (321 to 454)	-0.62 (-0.67 to -0.55)	21.6 (19.5 to 23.8)	9442 (8534 to 10440)	59.6 (54.8 to 65.4)	15985 (14732 to 17536)	1.71 (1.70 to 1.72)
Haiti	161 (147 to 176)	8083 (7371 to 8824)	615 (559 to 678)	12740 (11589 to 14089)	1.48 (1.48 to 1.48)	14.9 (12.7 to 17.0)	501 (431 to 572)	27.9 (23.2 to 32.7)	430 (364 to 503)	-0.45 (-0.49 to -0.42)	146 (132 to 161)	7582 (6899 to 8318)	587 (529 to 648)	12310 (11138 to 13625)	1.57 (1.57 to 1.58)
Honduras	51.6 (46.5 to 56.9)	4021 (3632 to 4444)	318 (289 to 350)	7959 (7248 to 8755)	2.22 (2.14 to 2.27)	1.82 (1.44 to 2.27)	90.8 (73.6 to 111)	5.50 (4.49 to 6.66)	107 (87.7 to 129)	0.52 (0.50 to 0.53)	49.8 (44.8 to 55.1)	3930 (3548 to 4354)	312 (284 to 345)	7852 (7149 to 8652)	2.25 (2.17 to 2.30)
Hungary	244 (218 to 267)	2993 (2704 to 3269)	474 (424 to 527)	4742 (4270 to 5264)	1.47 (1.40 to 1.52)	9.75 (8.28 to 11.2)	160 (137 to 184)	15.8 (13.1 to 18.9)	251 (205 to 303)	1.43 (1.42 to 1.45)	234 (209 to 258)	2832 (2538 to 3115)	458 (409 to 511)	4491 (4023 to 5005)	1.48 (1.40 to 1.52)
Iceland	2.74 (2.48 to 3.02)	1957 (1768 to 2163)	9.92 (8.95 to 11.0)	4159 (3744 to 4601)	2.46 (2.45 to 2.47)	0.37 (0.30 to 0.45)	275 (223 to 336)	0.72 (0.58 to 0.88)	363 (291 to 453)	0.91 (0.88 to 0.94)	2.37 (2.11 to 2.65)	1682 (1491 to 1887)	9.20 (8.25 to 10.2)	3796 (3387 to 4231)	2.66 (2.65 to 2.67)
India	8210 (7385 to 9036)	2958 (2666 to 3253)	35711 (32174 to 39234)	5324 (4805 to 5843)	1.89 (1.88 to 1.92)	625 (479 to 822)	169 (130 to 217)	1327 (1034 to 1711)	187 (146 to 240)	0.32 (0.30 to 0.34)	7585 (6765 to 8432)	2789 (2496 to 3089)	34383 (30813 to 38026)	5137 (4608 to 5675)	1.97 (1.95 to 1.99)
Indonesia	1405 (1281 to 1532)	2286 (2080 to 2508)	5971 (5381 to 6611)	4394 (3972 to 4835)	2.14 (2.03 to 2.29)	203 (154 to 266)	235 (181 to 306)	371 (290 to 483)	250 (195 to 323)	0.21 (0.20 to 0.22)	1202 (1061 to 1343)	2051 (1827 to 2278)	5599 (5030 to 6253)	4144 (3734 to 4596)	2.30 (2.18 to 2.45)
Iran (Islamic Republic of)	429 (388 to 473)	2930 (2640 to 3242)	3120 (2805 to 3444)	7308 (6559 to 8072)	2.93 (2.89 to 2.98)	43.7 (33.8 to 56.5)	200 (158 to 255)	100 (79.2 to 128)	222 (176 to 281)	0.35 (0.34 to 0.35)	385 (346 to 431)	2730 (2450 to 3047)	3020 (2705 to 3355)	7086 (6346 to 7867)	3.06 (3.01 to 3.12)
Iraq	334 (308 to 363)	6935 (6367 to 7579)	2212 (2026 to 2416)	14990 (13751 to 16292)	2.54 (2.52 to 2.56)	15.6 (12.8 to 18.7)	214 (180 to 250)	41.6 (34.4 to 50.1)	222 (186 to 265)	0.17 (0.14 to 0.21)	318 (292 to 348)	6721 (6148 to 7364)	2170 (1985 to 2380)	14767 (13526 to 16076)	2.59 (2.57 to 2.61)
Ireland	43.5 (39.1 to 47.7)	2160 (1945 to 2378)	132 (120 to 145)	3827 (3467 to 4226)	1.87 (1.86 to 1.88)	5.50 (4.93 to 6.17)	293 (262 to 329)	19.4 (17.5 to 21.4)	673 (611 to 742)	2.73 (2.71 to 2.74)	38.0 (33.6 to 42.4)	1867 (1650 to 2094)	113 (101 to 127)	3155 (2803 to 3558)	1.72 (1.71 to 1.73)
Israel	85.0 (77.4 to 92.7)	3304 (3010 to 3611)	292 (266 to 317)	4914 (4450 to 5359)	1.30 (1.28 to 1.32)	6.73 (6.39 to 7.10)	272 (259 to 287)	19.7 (18.8 to 20.5)	387 (369 to 402)	1.14 (1.13 to 1.15)	78.3 (70.6 to 85.9)	3031 (2731 to 3335)	272 (246 to 297)	4527 (4064 to 4973)	1.31 (1.29 to 1.33)
Italy	1432 (1289 to 1593)	3228 (2908 to 3581)	2628 (2351 to 2954)	4574 (4103 to 5066)	1.12 (1.08 to 1.14)	173 (140 to 217)	488 (390 to 616)	393 (321 to 489)	939 (752 to 1182)	2.06 (1.85 to 2.17)	1259 (1116 to 1424)	2740 (2416 to 3109)	2234 (1954 to 2558)	3635 (3123 to 4139)	0.92 (0.90 to 0.94)
Jamaica	48.8 (45.6 to 52.7)	5155 (4780 to 5575)	130 (119 to 143)	8224 (7509 to 9039)	1.52 (1.46 to 1.57)	3.62 (2.97 to 4.36)	320 (263 to 384)	3.72 (3.09 to 4.46)	244 (203 to 293)	-0.77 (-0.83 to -0.72)	45.2 (42.0 to 49.2)	4835 (4470 to 5277)	127 (115 to 139)	7980 (7273 to 8778)	1.63 (1.57 to 1.67)
Japan	2724 (2454 to 3037)	3031 (2741 to 3364)	6738 (6079 to 7499)	4849 (4346 to 5353)	1.55 (1.53 to 1.58)	126 (98.5 to 162)	180 (139 to 232)	171 (136 to 216)	224 (174 to 288)	0.64 (0.57 to 0.68)	2599 (2328 to 2923)	2851 (2561 to 3197)	6567 (5918 to 7326)	4625 (4123 to 5142)	1.60 (1.57 to 1.64)
Jordan	55.8 (51.5 to 60.8)	7238 (6694 to 7858)	533 (492 to 582)	12696 (11751 to 13874)	1.86 (1.82 to 1.92)	5.10 (3.73 to 6.33)	338 (258 to 412)	13.5 (10.6 to 17.2)	246 (197 to 308)	-0.98 (-1.02 to -0.94)	50.7 (46.5 to 55.6)	6901 (6354 to 7529)	520 (478 to 569)	12450 (11487 to 13618)	2.04 (1.99 to 2.10)
Kazakhstan	231 (205 to 272)	2919 (2593 to 3439)	701 (645 to 755)	6543 (6019 to 7045)	2.64 (2.63 to 2.65)	14.5 (12.1 to 17.2)	175 (147 to 207)	22.5 (19.0 to 26.9)	224 (188 to 267)	0.83 (0.79 to 0.88)	216 (191 to 257)	2744 (2419 to 3267)	679 (621 to 732)	6319 (5783 to 6805)	2.73 (2.71 to 2.74)
Kenya	84.7 (76.2 to 92.5)	1530 (1383 to 1684)	314 (285 to 343)	2004 (1823 to 2194)	0.87 (0.86 to 0.88)	12.0 (9.99 to 14.5)	123 (105 to 147)	30.2 (25.1 to 36.9)	131 (110 to 157)	0.18 (0.17 to 0.19)	72.7 (64.4 to 80.7)	1407 (1252 to 1560)	283 (254 to 311)	1874 (1686 to 2061)	0.92 (0.91 to 0.93)
Kiribati	2.01 (1.83 to 2.20)	7960 (7242 to 8679)	7.51 (6.88 to 8.13)	14947 (13748 to 16070)	2.06 (2.05 to 2.08)	0.07 (0.06 to 0.08)	201 (170 to 233)	0.12 (0.10 to 0.14)	196 (166 to 231)	-0.04 (-0.07 to -0.03)	1.94 (1.76 to 2.13)	7760 (7052 to 8476)	7.39 (6.76 to 8.02)	14751 (13550 to 15878)	2.10 (2.09 to 2.12)
Kuwait	23.5 (21.3 to 25.6)	6961 (6344 to 7623)	265 (242 to 292)	14150 (12983 to 15367)	2.35 (2.32 to 2.39)	1.55 (1.26 to 1.89)	251 (206 to 301)	5.82 (4.69 to 7.19)	247 (201 to 302)	-0.21 (-0.53 to -0.04)	21.9 (19.8 to 24.0)	6710 (6097 to 7356)	259 (237 to 286)	13903 (12741 to 15115)	2.39 (2.33 to 2.46)
Kyrgyzstan	33.2 (29.9 to 36.5)	1814 (1627 to 2000)	124 (111 to 139)	4063 (3650 to 4567)	2.64 (2.62 to 2.66)	2.46 (2.02 to 2.97)	119 (98.1 to 143)	5.76 (4.65 to 7.06)	174 (140 to 213)	1.19 (1.16 to 1.21)	30.7 (27.4 to 34.0)	1695 (1508 to 1878)	118 (105 to 133)	3890 (3489 to 4392)	2.72 (2.70 to 2.74)
Lao People's Democratic Republic	35.8 (33.1 to 39.0)	2890 (2650 to 3176)	188 (172 to 205)	7058 (6443 to 7653)	2.92 (2.92 to 2.93)	4.58 (3.86 to 5.53)	247 (209 to 293)	7.98 (6.57 to 9.59)	220 (181 to 262)	-0.28 (-0.33 to -0.23)	31.2 (28.5 to 34.6)	2643 (2407 to 2934)	180 (164 to 197)	6838 (6233 to 7453)	3.11 (3.11 to 3.12)
Latvia	37.5 (34.1 to 41.2)	1822 (1654 to 1998)	77.9 (70.0 to 87.8)	3998 (3590 to 4445)	2.57 (2.55 to 2.58)	2.09 (1.82 to 2.43)	132 (115 to 154)	2.71 (2.25 to 3.31)	209 (172 to 261)	1.51 (1.49 to 1.52)	35.4 (32.0 to 39.1)	1689 (1527 to 1864)	75.2 (67.2 to 85.2)	3788 (3368 to 4236)	2.64 (2.62 to 2.66)
Lebanon	58.6 (53.7 to 64.2)	4853 (4468 to 5303)	301 (277 to 327)	9517 (8757 to 10332)	2.23 (2.19 to 2.28)	3.31 (2.67 to 4.02)	237 (192 to 286)	6.82 (5.68 to 8.07)	226 (188 to 268)	-0.08 (-0.11 to -0.05)	55.3 (50.3 to 60.7)	4617 (4214 to 5058)	294 (271 to 320)	9290 (8537 to 10099)	2.32 (2.27 to 2.37)
Lesotho	10.8 (9.69 to 11.9)	1866 (1672 to 2064)	37.0 (34.0 to 40.3)	5153 (4712 to 5613)	3.32 (3.28 to 3.35)	1.51 (1.22 to 1.84)	211 (173 to 258)	2.08 (1.67 to 2.54)	234 (192 to 286)	0.33 (0.30 to 0.35)	9.29 (8.18 to 10.4)	1655 (1462 to 1859)	35.0 (31.9 to 38.2)	4919 (4481 to 5383)	3.57 (3.53 to 3.61)
Liberia	16.6 (15.1 to 18.2)	2499 (2275 to 2755)	78.3 (71.0 to 86.1)	5154 (4710 to 5620)	2.37 (2.36 to 2.38)	1.43 (1.17 to 1.76)	148 (123 to 180)	3.52 (2.89 to 4.28)	155 (128 to 187)	0.13 (0.10 to 0.15)	15.1 (13.6 to 16.8)	2351 (2129 to 2613)	74.8 (67.5 to 82.4)	4999 (4546 to 5464)	2.48 (2.46 to 2.49)
Libya	38.2 (34.8 to 41.8)	3737 (3384 to 4103)	307 (280 to 338)	9856 (9013 to 10902)	3.26 (3.19 to 3.33)	2.94 (2.45 to 3.53)	185 (157 to 218)	7.77 (6.44 to 9.36)	220 (182 to 264)	0.56 (0.54 to 0.58)	35.3 (31.8 to 38.7)	3551 (3193 to 3922)	299 (272 to 330)	9636 (8784 to 10669)	3.35 (3.28 to 3.42)
Lithuania	41.6 (37.3 to 47.0)	1623 (1449 to 1832)	91.9 (83.1 to 103)	3294 (2965 to 3665)	2.31 (2.28 to 2.33)	2.98 (2.66 to 3.37)	141 (126 to 160)	4.16 (3.49 to 5.06)	218 (180 to 270)	1.49 (1.44 to 1.52)	38.6 (34.2 to 44.0)	1481 (1304 to 1691)	87.7 (78.8 to 98.8)	3076 (2740 to 3456)	2.38 (2.34 to 2.41)
Luxembourg	6.38 (5.80 to 6.97)	2310 (2102 to 2531)	20.3 (18.4 to 22.4)	4352 (3935 to 4811)	2.07 (2.06 to 2.07)	0.62 (0.51 to 0.75)	274 (223 to 335)	1.65 (1.35 to 2.00)	426 (343 to 521)	1.44 (1.43 to 1.44)	5.77 (5.15 to 6.35)	2036 (1806 to 2265)	18.7 (16.7 to 20.8)	3926 (3488 to 4379)	2.14 (2.14 to 2.15)
Madagascar	52.5 (48.1 to 57.3)	1635 (1492 to 1793)	221 (202 to 243)	2685 (2449 to 2945)	1.60 (1.58 to 1.61)	8.36 (7.08 to 9.88)	157 (136 to 182)	19.6 (16.6 to 23.1)	148 (127 to 174)	-0.16 (-0.18 to -0.14)	44.2 (39.5 to 48.9)	1478 (1331 to 1635)	201 (183 to 225)	2536 (2309 to 2785)	1.74 (1.71 to 1.75)
Malawi	36.9 (33.6 to 40.2)	1427 (1310 to 1546)	108 (99.4 to 117)	1955 (1801 to 2108)	1.01 (1.00 to 1.02)	8.03 (6.86 to 9.42)	179 (153 to 206)	14.7 (12.2 to 17.7)	161 (136 to 189)	-0.25 (-0.29 to -0.22)	28.9 (25.6 to 32.2)	1248 (1126 to 1378)	93.3 (84.9 to 102)	1793 (1639 to 1947)	1.17 (1.16 to 1.19)
Malaysia	233 (214 to 256)	4322 (3941 to 4727)	1099 (1005 to 1195)	7419 (6786 to 8078)	1.75 (1.74 to 1.77)	18.3 (14.9 to 22.7)	238 (197 to 288)	35.0 (28.2 to 42.3)	217 (177 to 262)	-0.21 (-0.26 to -0.17)	215 (195 to 237)	4084 (3700 to 4490)	1064 (970 to 1159)	7202 (6570 to 7845)	1.84 (1.83 to 1.85)
Maldives	1.44 (1.31 to 1.58)	3131 (2854 to 3464)	9.13 (8.26 to 10.2)	5484 (4965 to 6077)	1.85 (1.83 to 1.86)	0.19 (0.15 to 0.24)	229 (188 to 280)	0.41 (0.33 to 0.50)	198 (160 to 238)	-0.40 (-0.44 to -0.36)	1.25 (1.11 to 1.40)	2901 (2612 to 3243)	8.72 (7.85 to 9.77)	5286 (4759 to 5885)	1.96 (1.94 to 1.98)
Mali	104 (94.8 to 115)	4259 (3874 to 4726)	510 (462 to 564)	8544 (7763 to 9434)	2.28 (2.24 to 2.30)	5.32 (4.33 to 6.51)	154 (127 to 189)	13.4 (10.6 to 16.6)	145 (120 to 174)	-0.20 (-0.20 to -0.19)	98.8 (89.3 to 110)	4105 (3712 to 4570)	497 (448 to 549)	8399 (7613 to 9286)	2.34 (2.30 to 2.37)
Malta	5.95 (5.42 to 6.57)	2633 (2393 to 2898)	23.2 (20.9 to 25.8)	6035 (5494 to 6638)	2.72 (2.70 to 2.74)	1.05 (0.94 to 1.16)	515 (461 to 571)	2.41 (2.13 to 2.69)	871 (759 to 976)	1.71 (1.69 to 1.72)	4.90 (4.34 to 5.52)	2118 (1873 to 2388)	20.8 (18.5 to 23.4)	5164 (4621 to 5766)	2.93 (2.90 to 2.95)
Marshall Islands	1.10 (1.00 to 1.21)	9973 (9131 to 10846)	5.21 (4.77 to 5.74)	22366 (20599 to 24499)	2.62 (2.59 to 2.64)	0.04 (0.03 to 0.05)	208 (175 to 245)	0.06 (0.05 to 0.07)	206 (174 to 244)	-0.01 (-0.04 to 0.02)	1.06 (0.97 to 1.17)	9766 (8924 to 10645)	5.15 (4.72 to 5.69)	22160 (20408 to 24280)	2.65 (2.63 to 2.67)
Mauritania	13.3 (12.2 to 14.6)	2174 (1980 to 2392)	52.9 (49.7 to 56.3)	3960 (3732 to 4195)	1.93 (1.91 to 1.95)	1.31 (1.09 to 1.56)	157 (132 to 186)	2.99 (2.46 to 3.65)	161 (136 to 194)	0.08 (0.07 to 0.09)	12.0 (10.8 to 13.3)	2017 (1822 to 2230)	49.9 (46.7 to 53.2)	3799 (3577 to 4018)	2.04 (2.02 to 2.06)
Mauritius	22.3 (20.6 to 24.2)	5252 (4849 to 5712)	105 (97.4 to 113)	10897 (10183 to 11735)	2.40 (2.36 to 2.44)	1.67 (1.37 to 1.99)	301 (249 to 358)	2.28 (1.95 to 2.67)	309 (263 to 366)	0.07 (0.03 to 0.10)	20.7 (18.9 to 22.6)	4951 (4535 to 5406)	102 (95.1 to 111)	10587 (9878 to 11436)	2.50 (2.46 to 2.54)
Mexico	2127 (1939 to 2318)	8001 (7300 to 8759)	7058 (6439 to 7737)	9976 (9102 to 10921)	0.72 (0.70 to 0.74)	87.1 (64.3 to 118)	216 (163 to 288)	122 (91.8 to 158)	175 (132 to 226)	-0.61 (-0.65 to -0.56)	2040 (1857 to 2236)	7785 (7083 to 8546)	6936 (6316 to 7629)	9801 (8927 to 10758)	0.75 (0.73 to 0.77)
Micronesia (Federated States of)	1.92 (1.76 to 2.10)	6454 (5890 to 7029)	6.58 (6.05 to 7.12)	14645 (13516 to 15803)	2.69 (2.64 to 2.75)	0.09 (0.07 to 0.10)	193 (164 to 225)	0.10 (0.08 to 0.12)	192 (160 to 227)	0.00 (-0.03 to 0.03)	1.84 (1.68 to 2.01)	6261 (5700 to 6828)	6.48 (5.96 to 7.03)	14453 (13338 to 15602)	2.78 (2.74 to 2.82)
Monaco	0.51 (0.46 to 0.56)	1822 (1640 to 2005)	1.37 (1.24 to 1.53)	3944 (3567 to 4381)	2.53 (2.52 to 2.54)	0.07 (0.05 to 0.08)	306 (250 to 372)	0.13 (0.10 to 0.15)	482 (391 to 588)	1.47 (1.45 to 1.49)	0.44 (0.39 to 0.49)	1517 (1324 to 1695)	1.24 (1.11 to 1.40)	3463 (3061 to 3916)	2.70 (2.69 to 2.71)
Mongolia	10.8 (9.82 to 11.7)	1610 (1461 to 1756)	55.6 (50.4 to 61.1)	3585 (3265 to 3917)	2.62 (2.60 to 2.63)	1.05 (0.86 to 1.26)	114 (94.9 to 136)	2.69 (2.17 to 3.27)	164 (133 to 199)	1.18 (1.17 to 1.20)	9.76 (8.79 to 10.7)	1496 (1350 to 1642)	52.9 (47.7 to 58.4)	3421 (3102 to 3762)	2.71 (2.70 to 2.72)
Montenegro	12.1 (10.7 to 13.6)	3483 (3093 to 3911)	29.4 (26.2 to 32.5)	5747 (5144 to 6349)	1.62 (1.58 to 1.64)	0.89 (0.76 to 1.03)	271 (230 to 315)	1.38 (1.18 to 1.61)	378 (318 to 441)	1.09 (1.07 to 1.11)	11.2 (9.81 to 12.7)	3212 (2822 to 3654)	28.0 (24.8 to 31.2)	5369 (4763 to 5981)	1.66 (1.62 to 1.69)
Morocco	400 (361 to 445)	4974 (4454 to 5531)	2695 (2460 to 2946)	14457 (13195 to 15780)	3.50 (3.49 to 3.51)	24.4 (20.0 to 29.4)	221 (185 to 263)	47.9 (39.1 to 57.1)	251 (205 to 299)	0.40 (0.39 to 0.41)	376 (336 to 421)	4753 (4229 to 5309)	2647 (2413 to 2898)	14206 (12969 to 15534)	3.59 (3.58 to 3.60)
Mozambique	54.8 (49.8 to 59.7)	1406 (1279 to 1521)	233 (211 to 256)	2747 (2477 to 3011)	2.16 (2.12 to 2.19)	12.0 (10.2 to 13.8)	186 (158 to 213)	27.2 (22.7 to 32.3)	183 (155 to 214)	0.01 (-0.02 to 0.03)	42.8 (37.7 to 48.0)	1220 (1090 to 1348)	206 (183 to 229)	2564 (2301 to 2829)	2.40 (2.36 to 2.44)
Myanmar	561 (513 to 614)	4078 (3727 to 4470)	2400 (2205 to 2569)	8413 (7730 to 9019)	2.38 (2.36 to 2.40)	61.0 (50.1 to 74.0)	318 (264 to 383)	83.0 (68.1 to 100)	276 (226 to 332)	-0.37 (-0.43 to -0.32)	500 (450 to 554)	3760 (3399 to 4174)	2317 (2116 to 2483)	8138 (7451 to 8755)	2.53 (2.52 to 2.55)
Namibia	10.2 (9.25 to 11.2)	2518 (2300 to 2772)	38.6 (35.0 to 42.5)	4249 (3840 to 4704)	1.69 (1.68 to 1.70)	1.16 (0.94 to 1.45)	202 (166 to 244)	2.22 (1.79 to 2.73)	193 (157 to 234)	-0.10 (-0.13 to -0.06)	8.99 (8.09 to 10.0)	2316 (2092 to 2569)	36.4 (32.6 to 40.3)	4056 (3636 to 4511)	1.81 (1.79 to 1.82)
Nauru	0.23 (0.21 to 0.25)	8112 (7390 to 8880)	0.65 (0.60 to 0.71)	16156 (14913 to 17445)	2.23 (2.21 to 2.25)	0.008 (0.007 to 0.01)	189 (155 to 225)	0.009 (0.008 to 0.01)	168 (142 to 195)	-0.38 (-0.44 to -0.31)	0.22 (0.20 to 0.24)	7923 (7193 to 8691)	0.64 (0.59 to 0.70)	15988 (14737 to 17284)	2.27 (2.25 to 2.29)
Nepal	190 (171 to 209)	3223 (2921 to 3545)	972 (892 to 1055)	6771 (6233 to 7353)	2.37 (2.34 to 2.40)	10.8 (8.75 to 13.2)	130 (107 to 156)	23.7 (19.3 to 28.9)	146 (120 to 176)	0.35 (0.31 to 0.38)	179 (160 to 198)	3093 (2791 to 3417)	948 (869 to 1032)	6625 (6092 to 7201)	2.43 (2.40 to 2.47)

Location	DM, Number in 1990 (000s)	DM, ASR in 1990 (per 100,000)	DM, Number in 2021 (000s)	DM, ASR in 2021 (per 100,000)	DM, AAPC (95%CI)	T1D, Number in 1990 (000s)	T1D, ASR in 1990 (per 100,000)	T1D, Number in 2021 (000s)	T1D, ASR in 2021 (per 100,000)	T1D, AAPC (95%CI)	T2D, Number in 1990 (000s)	T2D, ASR in 1990 (per 100,000)	T2D, Number in 2021 (000s)	T2D, ASR in 2021 (per 100,000)	T2D, AAPC (95%CI)
Netherlands	212 (195 to 233)	2037 (1880 to 2237)	483 (435 to 532)	3407 (3058 to 3756)	1.68 (1.66 to 1.70)	30.2 (26.0 to 35.2)	351 (303 to 405)	56.6 (47.2 to 67.2)	521 (428 to 619)	1.28 (1.26 to 1.29)	182 (165 to 202)	1686 (1508 to 1880)	427 (377 to 476)	2886 (2545 to 3231)	1.76 (1.73 to 1.78)
New Zealand	55.4 (49.7 to 61.9)	2779 (2495 to 3127)	158 (150 to 166)	4166 (3964 to 4365)	1.33 (1.31 to 1.36)	6.15 (5.06 to 7.50)	330 (270 to 401)	10.1 (8.27 to 12.3)	326 (265 to 398)	-0.04 (-0.05 to -0.03)	49.2 (43.5 to 55.9)	2449 (2152 to 2806)	148 (140 to 156)	3840 (3631 to 4042)	1.49 (1.46 to 1.51)
Nicaragua	46.3 (42.1 to 51.1)	4610 (4193 to 5111)	245 (221 to 269)	8313 (7519 to 9132)	1.93 (1.90 to 1.97)	1.80 (1.48 to 2.16)	107 (89.6 to 126)	3.65 (3.06 to 4.30)	107 (90.1 to 126)	0.03 (0.01 to 0.06)	44.5 (40.2 to 49.2)	4503 (4087 to 5004)	242 (217 to 265)	8206 (7400 to 9017)	1.97 (1.93 to 2.00)
Niger	46.4 (42.2 to 50.9)	2512 (2277 to 2753)	291 (263 to 318)	5003 (4547 to 5511)	2.25 (2.25 to 2.26)	4.31 (3.41 to 5.38)	143 (115 to 175)	13.2 (10.4 to 16.5)	141 (115 to 171)	-0.04 (-0.07 to -0.02)	42.1 (37.9 to 47.0)	2370 (2128 to 2613)	278 (250 to 306)	4862 (4397 to 5367)	2.35 (2.34 to 2.36)
Nigeria	561 (508 to 614)	2267 (2043 to 2499)	2280 (2078 to 2496)	3504 (3195 to 3836)	1.38 (1.30 to 1.45)	49.4 (38.4 to 64.5)	142 (112 to 182)	148 (114 to 193)	153 (121 to 196)	0.23 (0.17 to 0.26)	511 (456 to 564)	2125 (1905 to 2355)	2133 (1921 to 2350)	3351 (3049 to 3686)	1.45 (1.36 to 1.51)
Niue	0.09 (0.08 to 0.10)	8014 (7361 to 8695)	0.19 (0.17 to 0.20)	17599 (16223 to 19149)	2.56 (2.55 to 2.58)	0.002 (0.001 to 0.002)	159 (133 to 189)	0.001 (0.001 to 0.002)	156 (128 to 184)	-0.03 (-0.06 to -0.00)	0.09 (0.08 to 0.10)	7855 (7215 to 8533)	0.18 (0.17 to 0.20)	17443 (16067 to 18969)	2.60 (2.58 to 2.61)
North Macedonia	37.9 (34.2 to 42.0)	3767 (3406 to 4165)	120 (109 to 134)	6959 (6310 to 7737)	2.01 (1.99 to 2.02)	2.74 (2.14 to 3.33)	266 (208 to 324)	4.57 (3.78 to 5.56)	352 (288 to 427)	0.92 (0.90 to 0.95)	35.2 (31.5 to 39.3)	3502 (3141 to 3904)	116 (104 to 129)	6607 (5950 to 7379)	2.07 (2.06 to 2.09)
Northern Mariana Islands	0.67 (0.60 to 0.75)	4976 (4530 to 5424)	2.55 (2.29 to 2.80)	9464 (8505 to 10372)	2.10 (2.09 to 2.10)	0.02 (0.02 to 0.03)	108 (87.6 to 129)	0.03 (0.02 to 0.03)	112 (90.8 to 136)	0.09 (0.08 to 0.10)	0.65 (0.58 to 0.73)	4867 (4420 to 5316)	2.52 (2.26 to 2.78)	9352 (8393 to 10255)	2.13 (2.13 to 2.14)
Norway	92.8 (83.8 to 103)	3024 (2740 to 3336)	161 (145 to 177)	3998 (3588 to 4411)	0.90 (0.88 to 0.92)	15.0 (12.7 to 17.9)	593 (497 to 704)	27.7 (23.4 to 32.7)	833 (695 to 991)	1.10 (1.09 to 1.10)	77.8 (67.9 to 87.8)	2431 (2106 to 2758)	133 (117 to 150)	3165 (2761 to 3596)	0.85 (0.83 to 0.87)
Oman	17.3 (15.9 to 18.8)	4562 (4169 to 4997)	100 (91.4 to 111)	8417 (7701 to 9282)	1.94 (1.88 to 1.99)	3.69 (3.13 to 4.42)	613 (522 to 720)	10.4 (8.72 to 12.4)	608 (514 to 725)	-0.01 (-0.05 to 0.04)	13.6 (12.2 to 15.2)	3949 (3543 to 4382)	89.8 (80.4 to 100)	7809 (7084 to 8646)	2.16 (2.09 to 2.22)
Pakistan	940 (851 to 1039)	2994 (2707 to 3310)	5399 (4865 to 5956)	7005 (6360 to 7717)	2.77 (2.73 to 2.80)	107 (81.8 to 142)	240 (186 to 309)	291 (223 to 378)	271 (211 to 347)	0.39 (0.39 to 0.40)	833 (737 to 937)	2754 (2445 to 3094)	5108 (4595 to 5654)	6734 (6082 to 7458)	2.91 (2.87 to 2.95)
Palau	0.49 (0.45 to 0.54)	8432 (7722 to 9269)	1.90 (1.76 to 2.04)	16883 (15707 to 18021)	2.26 (2.25 to 2.27)	0.01 (0.01 to 0.01)	171 (141 to 201)	0.02 (0.01 to 0.02)	169 (140 to 199)	-0.03 (-0.04 to -0.03)	0.48 (0.44 to 0.53)	8262 (7551 to 9108)	1.89 (1.75 to 2.02)	16714 (15542 to 17870)	2.29 (2.28 to 2.30)
Palestine	22.5 (20.9 to 24.4)	4264 (3949 to 4620)	140 (128 to 152)	9056 (8323 to 9867)	2.47 (2.46 to 2.48)	2.01 (1.58 to 2.48)	263 (214 to 315)	6.54 (5.01 to 8.40)	296 (232 to 372)	0.38 (0.35 to 0.39)	20.5 (18.8 to 22.3)	4001 (3667 to 4370)	133 (121 to 146)	8760 (8012 to 9579)	2.57 (2.56 to 2.58)
Panama	32.3 (29.5 to 35.3)	3856 (3510 to 4245)	162 (147 to 180)	7221 (6531 to 7997)	2.07 (2.02 to 2.13)	1.43 (1.13 to 1.79)	131 (105 to 161)	3.27 (2.57 to 3.99)	150 (118 to 183)	0.43 (0.40 to 0.46)	30.8 (28.0 to 33.7)	3725 (3384 to 4109)	159 (143 to 177)	7071 (6394 to 7858)	2.11 (2.07 to 2.17)
Papua New Guinea	54.9 (49.8 to 60.5)	4565 (4171 to 5004)	371 (335 to 411)	10618 (9646 to 11695)	2.75 (2.73 to 2.77)	2.34 (1.96 to 2.74)	131 (111 to 153)	6.50 (5.36 to 7.76)	137 (114 to 163)	0.14 (0.14 to 0.15)	52.5 (47.2 to 58.3)	4434 (4033 to 4871)	364 (329 to 405)	10481 (9509 to 11561)	2.81 (2.79 to 2.83)
Paraguay	45.5 (40.9 to 50.7)	3601 (3237 to 4015)	211 (196 to 230)	6616 (6146 to 7220)	1.99 (1.97 to 2.00)	3.15 (2.59 to 3.76)	186 (154 to 219)	8.13 (6.64 to 9.77)	230 (189 to 275)	0.69 (0.68 to 0.69)	42.3 (37.6 to 47.5)	3415 (3042 to 3827)	203 (187 to 222)	6386 (5920 to 6991)	2.04 (2.03 to 2.06)
Peru	137 (124 to 152)	1971 (1773 to 2197)	625 (575 to 678)	3494 (3212 to 3798)	1.84 (1.82 to 1.87)	8.36 (6.74 to 10.1)	86.8 (71.2 to 103)	19.3 (15.9 to 23.3)	104 (85.7 to 125)	0.61 (0.60 to 0.62)	129 (115 to 144)	1884 (1687 to 2115)	605 (555 to 659)	3389 (3110 to 3695)	1.89 (1.87 to 1.92)
Philippines	607 (554 to 661)	3438 (3132 to 3777)	2169 (1986 to 2371)	4661 (4268 to 5109)	0.98 (0.93 to 1.02)	79.1 (59.8 to 104)	289 (222 to 373)	132 (102 to 171)	241 (188 to 308)	-0.49 (-0.54 to -0.40)	528 (472 to 588)	3149 (2830 to 3507)	2037 (1852 to 2243)	4420 (4021 to 4865)	1.09 (1.03 to 1.14)
Poland	853 (757 to 949)	3433 (3059 to 3822)	1758 (1571 to 1981)	4753 (4277 to 5312)	1.07 (1.03 to 1.10)	38.7 (30.3 to 49.6)	186 (145 to 237)	56.8 (45.0 to 71.3)	238 (185 to 300)	0.81 (0.79 to 0.83)	814 (719 to 912)	3247 (2865 to 3649)	1701 (1517 to 1920)	4516 (4045 to 5081)	1.08 (1.04 to 1.12)
Portugal	238 (216 to 263)	3362 (3051 to 3707)	718 (652 to 791)	7043 (6375 to 7754)	2.40 (2.40 to 2.41)	15.2 (12.8 to 17.8)	257 (216 to 303)	32.5 (26.7 to 39.8)	438 (357 to 531)	1.75 (1.73 to 1.76)	222 (200 to 248)	3105 (2789 to 3454)	685 (619 to 763)	6605 (5924 to 7329)	2.45 (2.44 to 2.46)
Puerto Rico	125 (115 to 137)	6450 (5928 to 7080)	323 (298 to 364)	10522 (9632 to 11696)	1.59 (1.58 to 1.60)	9.02 (7.50 to 10.6)	471 (392 to 556)	8.49 (7.08 to 9.93)	399 (330 to 473)	-0.50 (-0.53 to -0.46)	116 (106 to 128)	5979 (5468 to 6599)	315 (289 to 356)	10123 (9219 to 11278)	1.71 (1.70 to 1.72)
Qatar	4.03 (3.64 to 4.45)	8093 (7377 to 8920)	82.3 (73.1 to 93.3)	16965 (15696 to 18683)	2.42 (2.40 to 2.44)	0.35 (0.28 to 0.45)	303 (249 to 373)	3.08 (2.40 to 3.87)	363 (287 to 448)	0.58 (0.56 to 0.59)	3.67 (3.28 to 4.11)	7790 (7048 to 8618)	79.2 (70.0 to 90.2)	16602 (15314 to 18262)	2.46 (2.44 to 2.48)
Republic of Korea	708 (650 to 771)	3691 (3399 to 3997)	4068 (3801 to 4333)	9321 (8716 to 9948)	3.04 (3.00 to 3.07)	61.0 (50.8 to 72.5)	266 (222 to 316)	87.1 (70.2 to 107)	289 (228 to 361)	0.32 (0.28 to 0.36)	647 (589 to 708)	3425 (3123 to 3744)	3981 (3719 to 4251)	9032 (8439 to 9655)	3.19 (3.15 to 3.23)
Republic of Moldova	63.5 (57.7 to 70.9)	2464 (2243 to 2743)	149 (132 to 164)	4743 (4186 to 5201)	2.14 (2.12 to 2.16)	3.16 (2.65 to 3.80)	131 (110 to 158)	4.52 (3.73 to 5.61)	190 (155 to 236)	1.24 (1.22 to 1.28)	60.4 (54.1 to 67.7)	2332 (2097 to 2611)	145 (127 to 159)	4552 (4011 to 5013)	2.20 (2.17 to 2.22)
Romania	323 (288 to 367)	2143 (1914 to 2420)	619 (554 to 696)	3369 (3017 to 3768)	1.47 (1.45 to 1.50)	15.3 (12.9 to 18.2)	120 (100 to 142)	21.7 (18.0 to 26.4)	181 (149 to 222)	1.33 (1.32 to 1.35)	308 (272 to 351)	2023 (1793 to 2299)	597 (533 to 675)	3188 (2843 to 3584)	1.48 (1.45 to 1.51)
Russian Federation	2037 (1834 to 2256)	1881 (1707 to 2068)	4991 (4494 to 5524)	3780 (3416 to 4171)	2.28 (2.27 to 2.29)	156 (131 to 188)	172 (145 to 205)	298 (250 to 363)	307 (254 to 372)	1.91 (1.88 to 1.94)	1882 (1689 to 2099)	1710 (1530 to 1907)	4693 (4202 to 5249)	3474 (3113 to 3884)	2.32 (2.30 to 2.34)
Rwanda	30.6 (27.9 to 33.5)	1551 (1414 to 1696)	91.2 (83.4 to 99.6)	2038 (1861 to 2220)	0.88 (0.86 to 0.89)	6.51 (5.51 to 7.65)	195 (168 to 229)	11.4 (9.65 to 13.5)	177 (150 to 207)	-0.23 (-0.28 to -0.18)	24.1 (21.4 to 26.9)	1355 (1212 to 1503)	79.8 (71.6 to 88.3)	1861 (1690 to 2033)	1.02 (0.99 to 1.03)
Saint Kitts and Nevis	1.31 (1.21 to 1.42)	6761 (6183 to 7423)	3.73 (3.30 to 4.13)	9751 (8685 to 10762)	1.17 (1.16 to 1.19)	0.08 (0.07 to 0.10)	411 (342 to 488)	0.10 (0.08 to 0.12)	287 (238 to 349)	-1.08 (-1.13 to -1.03)	1.23 (1.13 to 1.34)	6350 (5785 to 7015)	3.63 (3.20 to 4.03)	9465 (8394 to 10479)	1.28 (1.26 to 1.29)
Saint Lucia	4.85 (4.44 to 5.26)	9608 (8712 to 10517)	15.1 (13.9 to 16.3)	12422 (11485 to 13424)	0.82 (0.81 to 0.84)	0.21 (0.18 to 0.25)	324 (274 to 387)	0.24 (0.20 to 0.29)	239 (201 to 288)	-0.91 (-0.96 to -0.86)	4.63 (4.23 to 5.06)	9285 (8412 to 10190)	14.8 (13.7 to 16.1)	12183 (11228 to 13176)	0.88 (0.87 to 0.89)
Saint Vincent and the Grenadines	3.46 (3.21 to 3.73)	8557 (7878 to 9256)	8.59 (7.86 to 9.32)	12571 (11529 to 13632)	1.24 (1.24 to 1.25)	0.23 (0.20 to 0.26)	439 (378 to 501)	0.19 (0.16 to 0.23)	325 (273 to 382)	-0.95 (-1.01 to -0.87)	3.24 (2.98 to 3.51)	8118 (7439 to 8822)	8.40 (7.66 to 9.13)	12246 (11183 to 13313)	1.33 (1.32 to 1.34)
Samoa	3.65 (3.36 to 3.98)	7303 (6689 to 7977)	12.8 (11.8 to 14.1)	15853 (14558 to 17431)	2.52 (2.51 to 2.53)	0.10 (0.08 to 0.12)	145 (121 to 171)	0.14 (0.11 to 0.16)	143 (118 to 169)	-0.01 (-0.03 to -0.00)	3.55 (3.26 to 3.87)	7159 (6541 to 7834)	12.7 (11.6 to 14.0)	15710 (14425 to 17285)	2.56 (2.55 to 2.57)
San Marino	0.33 (0.30 to 0.36)	2050 (1852 to 2259)	1.22 (1.11 to 1.37)	4294 (3885 to 4796)	2.42 (2.41 to 2.43)	0.04 (0.03 to 0.05)	299 (242 to 366)	0.11 (0.09 to 0.14)	498 (403 to 605)	1.66 (1.63 to 1.68)	0.29 (0.26 to 0.32)	1751 (1537 to 1976)	1.11 (0.99 to 1.26)	3796 (3366 to 4316)	2.54 (2.53 to 2.55)
Sao Tome and Principe	1.09 (0.98 to 1.20)	2846 (2568 to 3153)	4.59 (4.19 to 5.00)	6114 (5568 to 6670)	2.50 (2.50 to 2.51)	0.08 (0.07 to 0.10)	165 (138 to 198)	0.17 (0.14 to 0.20)	174 (144 to 207)	0.17 (0.16 to 0.18)	1.01 (0.91 to 1.12)	2681 (2412 to 2986)	4.42 (4.03 to 4.82)	5940 (5408 to 6489)	2.61 (2.60 to 2.61)
Saudi Arabia	139 (127 to 150)	4465 (4056 to 4858)	1203 (1094 to 1324)	9952 (9082 to 10912)	2.62 (2.60 to 2.63)	17.2 (13.9 to 25.0)	311 (253 to 445)	72.9 (55.6 to 135)	458 (352 to 832)	1.23 (1.21 to 1.25)	122 (109 to 134)	4153 (3725 to 4541)	1130 (1002 to 1258)	9494 (8560 to 10506)	2.70 (2.68 to 2.71)
Senegal	69.7 (63.3 to 76.8)	3459 (3123 to 3801)	345 (319 to 371)	7069 (6568 to 7615)	2.37 (2.34 to 2.41)	4.88 (3.98 to 5.94)	161 (132 to 194)	11.2 (9.12 to 13.9)	165 (137 to 203)	0.06 (0.05 to 0.07)	64.9 (58.4 to 72.2)	3298 (2963 to 3659)	333 (308 to 359)	6904 (6383 to 7453)	2.45 (2.42 to 2.49)
Serbia	229 (204 to 256)	3719 (3335 to 4128)	500 (450 to 556)	6074 (5477 to 6733)	1.58 (1.57 to 1.60)	12.4 (10.8 to 14.4)	234 (202 to 273)	17.4 (14.8 to 20.8)	316 (267 to 380)	0.98 (0.95 to 1.02)	217 (192 to 244)	3485 (3092 to 3899)	483 (432 to 537)	5758 (5151 to 6414)	1.61 (1.60 to 1.63)
Seychelles	1.07 (0.97 to 1.18)	3350 (3032 to 3709)	6.57 (6.03 to 7.16)	10912 (10051 to 11858)	3.88 (3.86 to 3.90)	0.07 (0.06 to 0.08)	196 (164 to 234)	0.11 (0.09 to 0.13)	193 (158 to 232)	-0.05 (-0.06 to -0.04)	1.01 (0.91 to 1.12)	3154 (2830 to 3513)	6.46 (5.93 to 7.06)	10719 (9868 to 11661)	4.02 (3.99 to 4.04)
Sierra Leone	29.1 (26.4 to 32.0)	2377 (2178 to 2620)	136 (123 to 150)	5332 (4832 to 5819)	2.65 (2.64 to 2.67)	2.55 (2.07 to 3.11)	148 (122 to 179)	5.91 (4.79 to 7.22)	157 (131 to 190)	0.18 (0.16 to 0.20)	26.5 (23.9 to 29.4)	2229 (2021 to 2469)	130 (118 to 144)	5175 (4668 to 5662)	2.77 (2.76 to 2.78)
Singapore	70.8 (64.9 to 77.1)	5249 (4808 to 5735)	288 (260 to 316)	6999 (6323 to 7666)	0.92 (0.90 to 0.95)	3.16 (2.52 to 3.83)	198 (158 to 241)	7.27 (5.80 to 8.98)	231 (184 to 286)	0.49 (0.47 to 0.51)	67.7 (61.9 to 73.8)	5051 (4605 to 5538)	280 (253 to 308)	6768 (6111 to 7435)	0.94 (0.91 to 0.96)
Slovakia	87.5 (77.6 to 98.3)	2625 (2337 to 2942)	195 (174 to 216)	3941 (3516 to 4348)	1.32 (1.29 to 1.34)	4.23 (3.70 to 4.84)	148 (129 to 170)	7.64 (6.38 to 9.07)	225 (184 to 273)	1.36 (1.34 to 1.38)	83.3 (73.6 to 94.1)	2477 (2187 to 2797)	188 (166 to 209)	3716 (3293 to 4125)	1.32 (1.29 to 1.34)
Slovenia	41.8 (37.0 to 46.8)	2929 (2606 to 3272)	80.7 (71.8 to 90.5)	3897 (3490 to 4386)	0.92 (0.88 to 0.94)	1.99 (1.68 to 2.32)	176 (148 to 208)	3.13 (2.57 to 3.82)	236 (193 to 289)	0.99 (0.95 to 1.05)	39.8 (34.9 to 44.9)	2753 (2428 to 3105)	77.6 (68.7 to 87.5)	3662 (3255 to 4144)	0.92 (0.87 to 0.95)
Solomon Islands	3.54 (3.23 to 3.94)	4037 (3687 to 4473)	21.0 (19.4 to 22.7)	8977 (8322 to 9640)	2.62 (2.59 to 2.64)	0.26 (0.21 to 0.31)	180 (150 to 209)	0.65 (0.53 to 0.78)	203 (168 to 241)	0.38 (0.36 to 0.40)	3.28 (2.98 to 3.67)	3857 (3516 to 4294)	20.4 (18.8 to 22.1)	8774 (8126 to 9425)	2.69 (2.67 to 2.72)
Somalia	32.3 (29.1 to 35.5)	1765 (1590 to 1927)	153 (138 to 168)	2982 (2694 to 3260)	1.70 (1.69 to 1.71)	6.90 (5.89 to 8.03)	197 (169 to 227)	18.4 (15.3 to 21.9)	190 (161 to 222)	-0.08 (-0.09 to -0.06)	25.4 (22.0 to 28.4)	1568 (1386 to 1737)	135 (120 to 151)	2791 (2507 to 3074)	1.87 (1.85 to 1.88)
South Africa	443 (401 to 486)	3338 (3019 to 3695)	1756 (1603 to 1929)	6260 (5712 to 6879)	2.03 (2.00 to 2.06)	33.9 (26.6 to 43.6)	200 (160 to 254)	59.5 (47.3 to 76.1)	200 (159 to 255)	-0.00 (-0.01 to 0.01)	409 (367 to 452)	3138 (2820 to 3498)	1697 (1543 to 1872)	6060 (5523 to 6686)	2.13 (2.09 to 2.16)
South Sudan	21.7 (19.6 to 23.9)	1549 (1395 to 1718)	67.7 (60.8 to 74.7)	2578 (2330 to 2815)	1.65 (1.65 to 1.66)	3.90 (3.34 to 4.64)	157 (134 to 182)	6.69 (5.62 to 8.04)	152 (129 to 179)	-0.08 (-0.10 to -0.06)	17.8 (15.5 to 19.9)	1393 (1239 to 1564)	61.1 (54.0 to 67.8)	2426 (2181 to 2657)	1.81 (1.80 to 1.81)
Spain	1018 (912 to 1138)	3596 (3235 to 4018)	2687 (2482 to 2889)	6565 (6056 to 7053)	1.94 (1.91 to 1.97)	73.9 (68.8 to 79.8)	329 (306 to 356)	179 (157 to 204)	611 (529 to 697)	1.98 (1.93 to 2.01)	944 (840 to 1064)	3267 (2906 to 3688)	2508 (2295 to 2706)	5954 (5417 to 6441)	1.94 (1.91 to 1.97)
Sri Lanka	262 (237 to 289)	4552 (4121 to 5037)	1549 (1419 to 1674)	10478 (9640 to 11319)	2.73 (2.72 to 2.75)	17.1 (14.3 to 20.5)	214 (181 to 254)	29.8 (24.9 to 36.2)	233 (192 to 284)	0.29 (0.28 to 0.30)	245 (220 to 272)	4338 (3907 to 4845)	1519 (1389 to 1646)	10245 (9413 to 11089)	2.82 (2.81 to 2.83)
Sudan	183 (169 to 199)	3488 (3197 to 3815)	969 (893 to 1052)	8001 (7337 to 8679)	2.71 (2.69 to 2.73)	13.8 (10.9 to 17.1)	166 (134 to 203)	35.0 (28.1 to 42.7)	185 (152 to 221)	0.34 (0.34 to 0.35)	169 (154 to 185)	3322 (3021 to 3650)	934 (856 to 1015)	7816 (7149 to 8479)	2.79 (2.77 to 2.81)
Suriname	8.45 (7.72 to 9.31)	5789 (5293 to 6406)	39.1 (35.7 to 42.5)	11476 (10511 to 12474)	2.22 (2.20 to 2.23)	0.38 (0.32 to 0.45)	208 (176 to 244)	0.56 (0.47 to 0.67)	181 (152 to 216)	-0.42 (-0.44 to -0.40)	8.06 (7.32 to 8.94)	5581 (5069 to 6195)	38.5 (35.2 to 42.0)	11295 (10353 to 12304)	2.29 (2.27 to 2.30)
Sweden	176 (159 to 196)	2685 (2419 to 2971)	357 (320 to 398)	4306 (3844 to 4775)	1.55 (1.51 to 1.60)	24.2 (20.1 to 29.1)	468 (384 to 566)	34.7 (28.7 to 42.0)	551 (450 to 671)	0.52 (0.52 to 0.53)	152 (134 to 172)	2218 (1928 to 2501)	322 (283 to 363)	3754 (3252 to 4239)	1.72 (1.66 to 1.77)
Switzerland	165 (149 to 181)	3236 (2918 to 3552)	456 (413 to 498)	6175 (5606 to 6754)	2.12 (2.10 to 2.15)	12.2 (10.0 to 14.7)	292 (239 to 355)	25.8 (21.5 to 31.0)	450 (371 to 553)	1.39 (1.35 to 1.41)	153 (137 to 169)	2943 (2629 to 3270)	430 (387 to 473)	5726 (5153 to 6305)	2.20 (2.18 to 2.23)
Syrian Arab Republic	130 (119 to 142)	4256 (3879 to 4650)	614 (554 to 674)	8531 (7733 to 9302)	2.27 (2.27 to 2.28)	14.6 (11.4 to 18.1)	308 (252 to 367)	24.3 (19.9 to 29.0)	321 (264 to 381)	0.14 (0.13 to 0.17)	116 (105 to 128)	3948 (3570 to 4359)	590 (529 to 649)	8211 (7402 to 8996)	2.39 (2.38 to 2.39)
Taiwan (Province of China)	315 (295 to 337)	3783 (3551 to 4041)	1130 (1055 to 1215)	5484 (5107 to 5903)	1.20 (1.18 to 1.22)	19.3 (15.2 to 24.5)	195 (155 to 244)	31.2 (26.6 to 37.3)	202 (173 to 238)	0.13 (0.09 to 0.19)	296 (275 to 319)	3588 (3356 to 3863)	1099 (1021 to 1185)	5282 (4902 to 5696)	1.26 (1.24 to 1.27)
Tajikistan	30.0 (27.5 to 33.0)	1758 (1616 to 1937)	160 (145 to 175)	4274 (3881 to 4684)	2.90 (2.88 to 2.92)	3.60 (2.96 to 4.26)	158 (132 to 185)	9.87 (8.11 to 11.8)	212 (176 to 251)	0.95 (0.94 to 0.95)	26.4 (23.9 to 29.2)	1600 (1459 to 1782)	150 (135 to 165)	4062 (3675 to 4468)	3.04 (3.02 to 3.06)
Thailand	552 (499 to 607)	2594 (2347 to 2873)	2944 (2676 to 3273)	5251 (4794 to 5798)	2.33 (2.31 to 2.36)	55.5 (45.4 to 66.2)	195 (163 to 230)	69.1 (57.7 to 82.5)	166 (136 to 198)	-0.45 (-0.52 to -0.35)	496 (443 to 551)	2400 (2148 to 2688)	2875 (2607 to 3199)	5085 (4631 to 5616)	2.48 (2.45 to 2.50)
Timor-Leste	3.58 (3.26 to 3.93)	1990 (1815 to 2182)	27.4 (24.7 to 30.3)	5945 (5350 to 6609)	3.63 (3.61 to 3.65)	0.48 (0.39 to 0.58)	150 (125 to 181)	0.96 (0.77 to 1.18)	155 (126 to 189)	0.10 (0.09 to 0.11)	3.10 (2.77 to 3.46)	1840 (1665 to 2045)	26.4 (23.7 to 29.3)	5790 (5199 to 6454)	3.80 (3.78 to 3.83)
Togo	17.1 (15.4 to 18.7)	1880 (1701 to 2060)	96.9 (89.4 to 106)	3520 (3248 to 3825)	2.04 (2.03 to 2.05)	2.24 (1.82 to 2.71)	154 (126 to 185)	5.78 (4.81 to 7.01)	154 (130 to 186)	-0.02 (-0.03 to -0.01)	14.8 (13.1 to 16.5)	1725 (1542 to 1912)	91.1 (83.4 to 100)	3367 (3098 to 3666)	2.16 (2.15 to 2.18)
Tokelau	0.06 (0.05 to 0.06)	8527 (7702 to 9306)	0.12 (0.11 to 0.13)	16249 (15015 to 17579)	2.10 (2.09 to 2.12)	0.001 (0.001 to 0.001)	170 (141 to 202)	0.001 (9e-04 to 0.001)	166 (136 to 195)	-0.05 (-0.07 to -0.01)	0.06 (0.05 to 0.06)	8357 (7524 to 9139)	0.12 (0.11 to 0.13)	16083 (14833 to 17423)	2.14 (2.12 to 2.15)
Tonga	2.57 (2.35 to 2.79)	7926 (7255 to 8599)	6.65 (6.08 to 7.24)	14883 (13586 to 16184)	2.06 (2.05 to 2.07)	0.06 (0.05 to 0.07)	146 (121 to 172)	0.07 (0.06 to 0.09)	144 (118 to 172)	-0.04 (-0.05 to -0.03)	2.51 (2.28 to 2.73)	7780 (7104 to 8456)	6.58 (6.00 to 7.16)	14739 (13433 to 16028)	2.09 (2.08 to 2.10)
Trinidad and Tobago	42.0 (39.3 to 45.1)	9092 (8526 to 9767)	121 (109 to 130)	12624 (11376 to 13619)	1.06 (1.05 to 1.07)	1.81 (1.53 to 2.13)	311 (265 to 363)	1.84 (1.55 to 2.15)	239 (200 to 281)	-0.81 (-0.84 to -0.78)	40.2 (37.5 to 43.4)	8780 (8199 to 9474)	119 (107 to 129)	12385 (11154 to 13376)	1.10 (1.08 to 1.11)
Tunisia	95.1 (86.8 to 104)	3466 (3158 to 3811)	635 (569 to 695)	9072 (8141 to 9923)	3.15 (3.13 to 3.16)	6.88 (5.52 to 8.39)	185 (153 to 222)	13.2 (11.0 to 15.9)	204 (168 to 246)	0.31 (0.31 to 0.32)	88.2 (79.7 to 97.6)	3281 (2968 to 3624)	622 (557 to 682)	8868 (7947 to 9725)	3.26 (3.24 to 3.27)
Turkmenistan	19.1 (17.6 to 20.7)	1514 (1397 to 1651)	89.5 (82.4 to 97.5)	3690 (3385 to 4028)	2.92 (2.90 to 2.94)	2.98 (2.52 to 3.56)	179 (152 to 213)	6.65 (5.63 to 8.02)	265 (225 to 319)	1.27 (1.26 to 1.28)	16.2 (14.5 to 17.9)	1335 (1201 to 1476)	82.9 (75.5 to 91.2)	3425 (3115 to 3771)	3.09 (3.07 to 3.11)
Tuvalu	0.23 (0.21 to 0.26)	5623 (5151 to 6187)	0.69 (0.64 to 0.75)	12108 (11249 to 13187)	2.48 (2.46 to 2.50)	0.009 (0.007 to 0.01)	190 (158 to 227)	0.01 (0.009 to 0.01)	180 (148 to 213)	-0.16 (-0.17 to -0.13)	0.23 (0.21 to 0.25)	5434 (4959 to 5985)	0.68 (0.63 to 0.74)	11928 (11057 to 13014)	2.54 (2.52 to 2.57)
Türkiye	576 (539 to 618)	2883 (2698 to 3097)	3263 (2978 to 3632)	6554 (5985 to 7286)	2.67 (2.57 to 2.74)	59.2 (48.2 to 72.5)	231 (191 to 279)	111 (89.4 to 134)	241 (194 to 293)	0.15 (0.14 to 0.16)	517 (479 to 561)	2652 (2460 to 2871)	3152 (2867 to 3521)	6313 (5746 to 7048)	2.75 (2.69 to 2.82)
Uganda	58.6 (53.6 to 63.8)	1388 (1259 to 1515)	249 (229 to 274)	2199 (2016 to 2410)	1.49 (1.48 to 1.50)	11.5 (9.66 to 13.8)	153 (130 to 179)	30.1 (25.1 to 36.3)	155 (131 to 182)	0.09 (0.07 to 0.11)	47.1 (41.7 to 52.2)	1235 (1108 to 1362)	219 (198 to 243)	2044 (1853 to 2257)	1.63 (1.62 to 1.64)
Ukraine	768 (683 to 862)	1912 (1713 to 2129)	1374 (1215 to 1530)	3413 (3028 to 3801)	1.88 (1.87 to 1.90)	55.3 (43.9 to 71.7)	174 (137 to 223)	72.5 (58.0 to 91.8)	248 (197 to 317)	1.19 (1.17 to 1.20)	713 (627 to 805)	1739 (1534 to 1955)	1301 (1140 to 1458)	3165 (2774 to 3556)	1.95 (1.93 to 1.96)
United Arab Emirates	12.5 (11.4 to 13.8)	5853 (5365 to 6491)	180 (160 to 201)	9547 (8593 to 10404)	1.58 (1.54 to 1.61)	1.56 (1.18 to 2.00)	328 (254 to 407)	9.84 (7.76 to 12.5)	353 (282 to 442)	0.28 (0.25 to 0.31)	10.9 (9.77 to 12.2)	5526 (5022 to 6193)	170 (150 to 191)	9194 (8263 to 10090)	1.64 (1.60 to 1.67)
United Kingdom	1162 (1057 to 1278)	2753 (2503 to 3034)	3418 (3134 to 3713)	6832 (6215 to 7455)	2.94 (2.87 to 3.00)	110 (92.6 to 131)	318 (267 to 379)	158 (130 to 192)	369 (305 to 447)	0.49 (0.44 to 0.53)	1053 (943 to 1175)	2435 (2173 to 2717)	3260 (2975 to 3558)	6463 (5834 to 7115)	3.18 (3.11 to 3.25)
United Republic of Tanzania	93.5 (85.6 to 102)	1308 (1208 to 1417)	451 (413 to 488)	2579 (2367 to 2779)	2.20 (2.18 to 2.21)	24.9 (18.1 to 32.1)	204 (153 to 257)	59.4 (42.5 to 78.2)	210 (155 to 271)	-0.01 (-0.15 to 0.09)	68.6 (59.3 to 77.2)	1104 (990 to 1222)	391 (351 to 432)	2369 (2160 to 2587)	2.48 (2.47 to 2.50)
United States Virgin Islands	3.96 (3.55 to 4.42)	7935 (7128 to 8829)	10.4 (9.47 to 11.4)	12872 (11739 to 13941)	1.58 (1.56 to 1.59)	0.17 (0.15 to 0.21)	318 (265 to 377)	0.15 (0.12 to 0.17)	273 (222 to 326)	-0.49 (-0.50 to -0.47)	3.79 (3.37 to 4.23)	7617 (6785 to 8507)	10.3 (9.32 to 11.2)	12599 (11463 to 13677)	1.64 (1.63 to 1.66)
United States of America	5420 (4979 to 5900)	3336 (3063 to 3625)	21666 (20502 to 22859)	7968 (7536 to 8412)	2.85 (2.83 to 2.88)	622 (531 to 737)	435 (368 to 516)	1020 (859 to 1223)	502 (425 to 594)	0.45 (0.43 to 0.46)	4798 (4346 to 5323)	2901 (2616 to 3219)	20646 (19561 to 21847)	7466 (7052 to 7905)	3.10 (3.08 to 3.14)
Uruguay	43.4 (39.2 to 47.7)	2121 (1917 to 2330)	137 (122 to 151)	5015 (4505 to 5549)	2.78 (2.71 to 2.83)	4.83 (4.04 to 5.65)	280 (235 to 329)	6.93 (5.63 to 8.34)	332 (268 to 400)	0.53 (0.51 to 0.54)	38.6 (34.4 to 43.0)	1841 (1636 to 2052)	130 (115 to 144)	4683 (4152 to 5210)	3.04 (2.97 to 3.09)
Uzbekistan	143 (132 to 155)	1959 (1813 to 2134)	922 (852 to 1005)	5684 (5248 to 6231)	3.51 (3.49 to 3.52)	21.5 (18.1 to 25.5)	232 (196 to 273)	62.1 (52.9 to 72.7)	358 (304 to 418)	1.41 (1.38 to 1.44)	121 (111 to 134)	1728 (1580 to 1910)	860 (790 to 944)	5326 (4887 to 5870)	3.72 (3.70 to 3.73)
Vanuatu	1.84 (1.68 to 2.02)	4566 (4186 to 5050)	12.5 (11.5 to 13.5)	11022 (10193 to 11898)	2.88 (2.87 to 2.89)	0.10 (0.08 to 0.12)	153 (127 to 182)	0.23 (0.19 to 0.27)	157 (129 to 187)	0.08 (0.07 to 0.10)	1.74 (1.58 to 1.92)	4413 (4036 to 4878)	12.2 (11.3 to 13.3)	10865 (10040 to 11748)	2.94 (2.93 to 2.95)
Venezuela (Bolivarian Republic of)	243 (223 to 264)	3998 (3678 to 4383)	1091 (990 to 1232)	6796 (6182 to 7657)	1.77 (1.71 to 1.83)	8.90 (7.31 to 10.7)	102 (83.9 to 120)	14.7 (12.3 to 17.7)	102 (85.2 to 122)	0.02 (0.01 to 0.04)	234 (213 to 255)	3896 (3571 to 4283)	1077 (975 to 1217)	6693 (6073 to 7549)	1.80 (1.75 to 1.86)
Viet Nam	596 (544 to 649)	2438 (2223 to 2671)	2516 (2340 to 2705)	4532 (4222 to 4860)	2.01 (1.97 to 2.04)	60.5 (48.6 to 74.9)	196 (160 to 239)	114 (93.1 to 138)	210 (170 to 255)	0.22 (0.21 to 0.23)	535 (483 to 592)	2242 (2023 to 2477)	2403 (2219 to 2599)	4322 (4000 to 4662)	2.12 (2.08 to 2.15)
Yemen	83.8 (76.1 to 92.3)	2729 (2464 to 3018)	540 (483 to 597)	5871 (5270 to 6544)	2.50 (2.43 to 2.56)	7.93 (6.36 to 9.87)	161 (133 to 195)	25.9 (20.8 to 31.5)	182 (149 to 218)	0.36 (0.34 to 0.38)	75.9 (67.9 to 84.7)	2567 (2309 to 2860)	514 (458 to 574)	5689 (5079 to 6376)	2.61 (2.52 to 2.68)
Zambia	46.8 (42.5 to 51.3)	2466 (2265 to 2680)	223 (201 to 244)	4086 (3716 to 4455)	1.60 (1.57 to 1.63)	7.01 (6.02 to 8.18)	190 (164 to 219)	15.0 (12.7 to 17.8)	167 (142 to 196)	-0.36 (-0.40 to -0.33)	39.8 (35.6 to 44.0)	2276 (2071 to 2490)	207 (186 to 230)	3920 (3542 to 4291)	1.74 (1.71 to 1.77)
Zimbabwe	56.2 (50.6 to 61.1)	2225 (2001 to 2427)	229 (209 to 253)	4824 (4419 to 5332)	2.53 (2.51 to 2.54)	4.87 (3.81 to 5.97)	118 (95.5 to 142)	9.37 (7.45 to 11.6)	134 (109 to 162)	0.33 (0.29 to 0.37)	51.3 (45.6 to 56.4)	2106 (1885 to 2308)	219 (199 to 243)	4690 (4283 to 5199)	2.62 (2.60 to 2.63)

AAPC: Average annual percentage change; ASR: Age-standardized rate; DM: Diabetes mellitus; SDI: Sociodemographic index; T1D: Type 1 diabetes; T2D: Type 2 diabetes. Results assuming first-order autocorrelation estimated from the data were broadly similar to those of the primary analyses, despite somewhat different estimates.

Table S3. Mortality number, age-standardized rate, and AAPC of diabetes and two subtypes among **women** globally and by different SDI, regions, countries, and territories, 1990-2021

Location	DM, Number in 1990 (000s)	DM, ASR in 1990 (per 100,000)	DM, Number in 2021 (000s)	DM, ASR in 2021 (per 100,000)	DM, AAPC (95%CI)	T1D, Number in 1990 (000s)	T1D, ASR in 1990 (per 100,000)	T1D, Number in 2021 (000s)	T1D, ASR in 2021 (per 100,000)	T1D, AAPC (95%CI)	T2D, Number in 1990 (000s)	T2D, ASR in 1990 (per 100,000)	T2D, Number in 2021 (000s)	T2D, ASR in 2021 (per 100,000)	T2D, AAPC (95%CI)
**Global**	366 (340 to 387)	17.8 (16.5 to 18.8)	860 (789 to 920)	18.6 (17.1 to 19.9)	0.15 (0.11 to 0.18)	20.1 (17.4 to 26.1)	0.84 (0.72 to 1.09)	22.8 (19.8 to 26.1)	0.54 (0.47 to 0.62)	-1.36 (-1.39 to -1.34)	346 (322 to 365)	16.9 (15.7 to 17.9)	838 (768 to 896)	18.0 (16.5 to 19.3)	0.21 (0.18 to 0.23)
**By SDI**															
High SDI	85.9 (78.0 to 90.1)	12.6 (11.5 to 13.2)	97.7 (81.9 to 106)	7.29 (6.31 to 7.79)	-1.76 (-1.81 to -1.72)	4.04 (3.85 to 4.18)	0.71 (0.68 to 0.73)	2.61 (2.41 to 2.73)	0.33 (0.32 to 0.35)	-2.42 (-2.49 to -2.37)	81.8 (74.1 to 85.9)	11.9 (10.9 to 12.5)	95.1 (79.5 to 103)	6.96 (5.99 to 7.46)	-1.73 (-1.77 to -1.69)
High-middle SDI	69.3 (64.1 to 73.8)	12.8 (11.8 to 13.6)	134 (117 to 147)	11.8 (10.4 to 13.0)	-0.24 (-0.30 to -0.19)	3.75 (3.21 to 4.50)	0.68 (0.58 to 0.82)	2.54 (2.25 to 3.01)	0.31 (0.27 to 0.36)	-2.52 (-2.61 to -2.41)	65.6 (60.4 to 69.6)	12.1 (11.1 to 12.9)	131 (115 to 144)	11.5 (10.1 to 12.6)	-0.15 (-0.20 to -0.10)
Middle SDI	108 (100 to 116)	22.7 (20.9 to 24.3)	314 (288 to 336)	23.2 (21.1 to 24.8)	0.11 (0.08 to 0.16)	5.68 (4.74 to 7.40)	0.81 (0.67 to 1.07)	6.85 (5.89 to 8.17)	0.52 (0.44 to 0.61)	-1.43 (-1.46 to -1.40)	103 (94.9 to 110)	21.8 (20.1 to 23.4)	307 (281 to 329)	22.6 (20.7 to 24.3)	0.16 (0.13 to 0.20)
Low-middle SDI	69.7 (62.8 to 76.7)	25.8 (23.0 to 28.5)	236 (214 to 258)	34.6 (31.4 to 37.9)	0.97 (0.90 to 1.05)	4.57 (3.71 to 7.00)	1.04 (0.80 to 1.64)	7.39 (6.00 to 8.93)	0.85 (0.68 to 1.03)	-0.64 (-0.67 to -0.60)	65.2 (58.6 to 71.6)	24.7 (22.2 to 27.1)	229 (207 to 251)	33.8 (30.6 to 37.1)	1.02 (0.95 to 1.11)
Low SDI	32.0 (28.4 to 36.9)	31.9 (28.3 to 36.5)	78.0 (69.0 to 88.4)	34.5 (30.8 to 38.9)	0.29 (0.25 to 0.32)	1.99 (1.56 to 2.98)	1.02 (0.74 to 1.62)	3.34 (2.61 to 4.08)	0.76 (0.59 to 0.96)	-0.91 (-0.93 to -0.88)	30.0 (26.7 to 34.3)	30.9 (27.3 to 35.3)	74.6 (66.2 to 84.5)	33.8 (30.1 to 38.0)	0.32 (0.28 to 0.36)
**Four world regions**															
Africa	42.1 (38.3 to 46.5)	33.0 (29.7 to 36.2)	119 (107 to 131)	41.3 (37.5 to 45.5)	0.72 (0.66 to 0.77)	1.90 (1.47 to 2.56)	0.70 (0.51 to 0.97)	2.94 (2.25 to 3.62)	0.49 (0.38 to 0.64)	-1.12 (-1.13 to -1.10)	40.2 (36.6 to 44.2)	32.3 (29.1 to 35.5)	116 (105 to 128)	40.8 (37.1 to 45.0)	0.75 (0.69 to 0.81)
America	80.4 (75.2 to 83.3)	23.4 (21.9 to 24.2)	157 (142 to 168)	20.9 (19.0 to 22.3)	-0.33 (-0.39 to -0.28)	2.88 (2.77 to 3.07)	0.82 (0.79 to 0.87)	3.70 (3.47 to 3.95)	0.61 (0.58 to 0.66)	-0.89 (-0.97 to -0.83)	77.5 (72.3 to 80.4)	22.6 (21.1 to 23.4)	153 (138 to 164)	20.2 (18.4 to 21.7)	-0.32 (-0.38 to -0.26)
Asia	161 (146 to 177)	16.9 (15.1 to 18.6)	473 (429 to 516)	18.2 (16.6 to 19.9)	0.26 (0.22 to 0.30)	11.3 (9.12 to 16.4)	0.85 (0.67 to 1.26)	14.1 (11.7 to 17.1)	0.57 (0.48 to 0.69)	-1.27 (-1.30 to -1.22)	150 (135 to 165)	16.0 (14.3 to 17.6)	459 (415 to 501)	17.7 (16.0 to 19.3)	0.33 (0.29 to 0.36)
Europe	80.4 (73.3 to 84.1)	12.3 (11.3 to 12.9)	109 (93.9 to 117)	10.1 (8.92 to 10.8)	-0.63 (-0.68 to -0.58)	3.87 (3.66 to 4.07)	0.74 (0.70 to 0.78)	1.93 (1.76 to 2.08)	0.31 (0.28 to 0.33)	-2.76 (-2.85 to -2.66)	76.5 (69.6 to 80.2)	11.6 (10.5 to 12.1)	107 (92.2 to 115)	9.78 (8.62 to 10.5)	-0.53 (-0.58 to -0.48)
**Six WHO regions**															
African Region	35.9 (32.2 to 40.0)	35.3 (31.5 to 39.1)	95.2 (86.4 to 106)	41.6 (37.9 to 46.0)	0.52 (0.47 to 0.56)	1.56 (1.17 to 2.14)	0.73 (0.53 to 1.01)	2.39 (1.79 to 2.98)	0.48 (0.36 to 0.64)	-1.33 (-1.34 to -1.31)	34.3 (30.7 to 38.1)	34.5 (30.8 to 38.4)	92.8 (84.1 to 103)	41.1 (37.4 to 45.4)	0.55 (0.50 to 0.59)
Region of the Americas	80.4 (75.2 to 83.3)	23.4 (21.9 to 24.2)	157 (142 to 168)	20.9 (19.0 to 22.3)	-0.33 (-0.39 to -0.28)	2.88 (2.77 to 3.07)	0.82 (0.79 to 0.87)	3.70 (3.47 to 3.95)	0.61 (0.58 to 0.66)	-0.89 (-0.97 to -0.83)	77.5 (72.3 to 80.4)	22.6 (21.1 to 23.4)	153 (138 to 164)	20.2 (18.4 to 21.7)	-0.32 (-0.38 to -0.26)
South-East Asia Region	74.8 (65.1 to 85.8)	24.0 (20.7 to 27.5)	263 (237 to 294)	30.6 (27.4 to 34.1)	0.80 (0.69 to 0.93)	5.52 (4.35 to 8.79)	1.12 (0.84 to 1.83)	8.31 (6.71 to 10.4)	0.82 (0.66 to 1.03)	-0.98 (-1.05 to -0.89)	69.3 (60.5 to 78.8)	22.9 (19.8 to 26.2)	255 (229 to 285)	29.8 (26.6 to 33.2)	0.87 (0.75 to 1.00)
European Region	82.6 (75.5 to 86.4)	12.3 (11.2 to 12.8)	116 (101 to 124)	10.5 (9.33 to 11.3)	-0.49 (-0.56 to -0.43)	4.09 (3.87 to 4.30)	0.75 (0.71 to 0.79)	2.28 (2.09 to 2.44)	0.35 (0.32 to 0.38)	-2.32 (-2.40 to -2.23)	78.5 (71.5 to 82.2)	11.5 (10.5 to 12.1)	113 (98.3 to 122)	10.2 (9.00 to 10.9)	-0.40 (-0.46 to -0.34)
Eastern Mediterranean Region	20.1 (18.3 to 21.9)	26.6 (24.2 to 29.1)	73.7 (63.9 to 84.5)	39.6 (34.6 to 45.0)	1.30 (1.27 to 1.34)	1.21 (0.98 to 1.61)	0.84 (0.63 to 1.14)	2.62 (1.96 to 3.31)	0.82 (0.62 to 1.05)	-0.04 (-0.06 to -0.01)	18.9 (17.2 to 20.6)	25.8 (23.4 to 28.3)	71.1 (61.6 to 81.3)	38.8 (33.9 to 44.1)	1.34 (1.30 to 1.37)
Western Pacific Region	67.5 (59.2 to 76.4)	12.1 (10.6 to 13.5)	147 (126 to 170)	9.66 (8.30 to 11.1)	-0.73 (-0.77 to -0.69)	4.51 (3.51 to 6.05)	0.65 (0.50 to 0.87)	3.18 (2.47 to 4.22)	0.27 (0.22 to 0.35)	-2.76 (-2.79 to -2.73)	63.0 (55.1 to 70.7)	11.4 (10.0 to 12.8)	144 (124 to 166)	9.39 (8.05 to 10.8)	-0.65 (-0.69 to -0.61)
**Seven super regions**															
Southeast Asia, East Asia, and Oceania	86.1 (76.1 to 97.8)	16.0 (14.0 to 18.2)	213 (188 to 239)	14.6 (12.9 to 16.5)	-0.25 (-0.28 to -0.22)	5.99 (4.82 to 8.57)	0.83 (0.65 to 1.19)	5.23 (4.22 to 7.06)	0.41 (0.33 to 0.54)	-2.23 (-2.25 to -2.20)	80.1 (70.4 to 90.6)	15.2 (13.2 to 17.1)	207 (183 to 234)	14.2 (12.5 to 16.0)	-0.17 (-0.20 to -0.13)
Central Europe, Eastern Europe, and Central Asia	22.8 (21.9 to 23.4)	7.65 (7.35 to 7.87)	56.7 (51.8 to 60.2)	13.6 (12.4 to 14.4)	1.95 (1.81 to 2.07)	2.37 (2.25 to 2.50)	0.91 (0.87 to 0.96)	1.53 (1.40 to 1.68)	0.56 (0.52 to 0.62)	-1.41 (-1.53 to -1.29)	20.4 (19.6 to 21.0)	6.74 (6.46 to 6.96)	55.1 (50.4 to 58.6)	13.0 (11.9 to 13.9)	2.23 (2.08 to 2.35)
South Asia	55.7 (47.5 to 61.9)	23.4 (19.8 to 26.3)	219 (193 to 247)	32.3 (28.4 to 36.5)	1.19 (1.08 to 1.38)	4.33 (3.26 to 6.62)	1.10 (0.76 to 1.74)	7.75 (5.82 to 9.92)	0.91 (0.68 to 1.17)	-0.55 (-0.71 to -0.38)	51.4 (43.7 to 57.2)	22.3 (18.8 to 25.1)	212 (186 to 240)	31.4 (27.6 to 35.5)	1.26 (1.15 to 1.45)
High-income	99.4 (90.0 to 104)	13.3 (12.1 to 13.9)	100 (82.8 to 110)	6.81 (5.85 to 7.32)	-2.13 (-2.17 to -2.10)	3.49 (3.33 to 3.62)	0.58 (0.56 to 0.60)	2.33 (2.17 to 2.43)	0.30 (0.29 to 0.30)	-2.15 (-2.20 to -2.11)	95.9 (86.7 to 101)	12.7 (11.5 to 13.3)	98.1 (80.6 to 107)	6.51 (5.56 to 7.02)	-2.14 (-2.17 to -2.11)
Latin America and Caribbean	46.6 (44.5 to 48.1)	43.0 (40.7 to 44.5)	118 (107 to 127)	35.1 (31.9 to 37.9)	-0.65 (-0.71 to -0.59)	1.36 (1.27 to 1.53)	0.89 (0.84 to 1.01)	2.25 (2.04 to 2.49)	0.69 (0.63 to 0.77)	-0.80 (-0.88 to -0.73)	45.3 (43.2 to 46.7)	42.1 (39.9 to 43.5)	115 (105 to 125)	34.4 (31.2 to 37.2)	-0.67 (-0.72 to -0.61)
North Africa and Middle East	19.7 (17.9 to 22.0)	27.1 (24.5 to 30.2)	60.9 (54.3 to 69.1)	31.4 (27.8 to 35.4)	0.47 (0.42 to 0.53)	1.00 (0.77 to 1.45)	0.66 (0.50 to 0.97)	1.30 (1.01 to 1.64)	0.45 (0.36 to 0.57)	-1.13 (-1.18 to -1.09)	18.7 (17.0 to 20.8)	26.5 (23.9 to 29.4)	59.6 (53.1 to 67.6)	30.9 (27.5 to 34.8)	0.51 (0.45 to 0.56)
Sub-Saharan Africa	35.5 (31.7 to 39.7)	36.5 (32.5 to 40.6)	92.7 (83.6 to 103)	42.8 (38.9 to 47.5)	0.50 (0.46 to 0.55)	1.52 (1.13 to 2.09)	0.74 (0.53 to 1.03)	2.37 (1.76 to 2.98)	0.49 (0.36 to 0.65)	-1.30 (-1.32 to -1.29)	34.0 (30.3 to 37.8)	35.8 (31.7 to 39.7)	90.3 (81.3 to 101)	42.3 (38.4 to 46.9)	0.56 (0.52 to 0.59)
**21 regions**															
East Asia	46.2 (39.0 to 55.0)	11.2 (9.42 to 13.2)	98.7 (79.5 to 120)	8.74 (7.07 to 10.6)	-0.77 (-0.85 to -0.68)	3.79 (2.91 to 5.20)	0.71 (0.54 to 0.99)	2.36 (1.76 to 3.21)	0.25 (0.19 to 0.34)	-3.28 (-3.33 to -3.23)	42.4 (35.7 to 50.2)	10.5 (8.82 to 12.3)	96.3 (77.7 to 117)	8.48 (6.87 to 10.3)	-0.65 (-0.73 to -0.56)
Oceania	1.08 (0.91 to 1.33)	82.3 (68.9 to 100)	3.29 (2.79 to 3.91)	100 (85.9 to 117)	0.63 (0.58 to 0.68)	0.02 (0.01 to 0.03)	0.65 (0.42 to 1.00)	0.04 (0.03 to 0.06)	0.66 (0.45 to 0.91)	0.16 (0.09 to 0.25)	1.06 (0.89 to 1.30)	81.7 (68.4 to 99.3)	3.25 (2.75 to 3.86)	99.4 (85.4 to 116)	0.63 (0.59 to 0.68)
Southeast Asia	38.9 (33.5 to 45.8)	30.7 (26.3 to 35.7)	111 (98.1 to 125)	34.0 (30.1 to 38.5)	0.32 (0.29 to 0.35)	2.17 (1.72 to 3.45)	1.18 (0.92 to 1.84)	2.83 (2.22 to 3.90)	0.77 (0.60 to 1.05)	-1.38 (-1.40 to -1.36)	36.7 (31.6 to 42.2)	29.5 (25.2 to 34.2)	108 (95.5 to 122)	33.2 (29.4 to 37.5)	0.37 (0.34 to 0.40)
Central Asia	2.77 (2.59 to 2.96)	9.88 (9.22 to 10.5)	7.81 (6.94 to 8.74)	17.2 (15.3 to 19.2)	1.75 (1.60 to 1.88)	0.33 (0.30 to 0.36)	1.07 (0.97 to 1.18)	0.47 (0.40 to 0.54)	0.97 (0.83 to 1.11)	-0.44 (-0.56 to -0.32)	2.44 (2.26 to 2.62)	8.80 (8.16 to 9.45)	7.34 (6.52 to 8.22)	16.2 (14.4 to 18.0)	1.93 (1.79 to 2.06)
Central Europe	11.8 (11.2 to 12.3)	13.6 (12.9 to 14.1)	17.9 (16.0 to 19.4)	12.2 (11.0 to 13.2)	-0.32 (-0.42 to -0.22)	1.16 (1.09 to 1.21)	1.41 (1.33 to 1.48)	0.46 (0.40 to 0.55)	0.43 (0.38 to 0.51)	-3.80 (-3.88 to -3.73)	10.6 (10.1 to 11.1)	12.2 (11.5 to 12.7)	17.4 (15.5 to 18.8)	11.8 (10.6 to 12.8)	-0.06 (-0.16 to 0.04)
Eastern Europe	8.21 (7.93 to 8.41)	4.56 (4.41 to 4.67)	31.0 (28.0 to 33.5)	13.0 (11.8 to 14.1)	3.68 (3.14 to 4.05)	0.88 (0.82 to 0.96)	0.65 (0.61 to 0.70)	0.60 (0.54 to 0.67)	0.48 (0.43 to 0.53)	-0.61 (-0.96 to -0.20)	7.33 (7.04 to 7.55)	3.91 (3.75 to 4.04)	30.4 (27.4 to 32.8)	12.6 (11.4 to 13.6)	4.11 (3.64 to 4.46)
Australasia	1.34 (1.20 to 1.46)	9.90 (8.84 to 10.7)	2.21 (1.79 to 2.48)	6.43 (5.34 to 7.14)	-1.34 (-1.44 to -1.24)	0.06 (0.05 to 0.06)	0.45 (0.42 to 0.48)	0.04 (0.04 to 0.04)	0.18 (0.17 to 0.19)	-2.85 (-2.96 to -2.72)	1.29 (1.14 to 1.40)	9.45 (8.41 to 10.3)	2.17 (1.75 to 2.44)	6.25 (5.16 to 6.96)	-1.28 (-1.38 to -1.19)
High-income Asia Pacific	9.32 (8.40 to 9.96)	8.27 (7.42 to 8.84)	10.2 (7.74 to 11.8)	2.82 (2.26 to 3.21)	-3.48 (-3.59 to -3.35)	0.31 (0.27 to 0.35)	0.31 (0.27 to 0.34)	0.10 (0.08 to 0.13)	0.07 (0.06 to 0.08)	-4.86 (-4.93 to -4.80)	9.00 (8.10 to 9.63)	7.96 (7.12 to 8.52)	10.1 (7.66 to 11.7)	2.76 (2.20 to 3.14)	-3.46 (-3.58 to -3.34)
High-income North America	30.1 (27.0 to 31.9)	14.1 (12.8 to 14.8)	34.9 (30.5 to 37.5)	9.24 (8.26 to 9.85)	-1.37 (-1.41 to -1.31)	1.42 (1.35 to 1.46)	0.80 (0.77 to 0.82)	1.36 (1.30 to 1.41)	0.58 (0.55 to 0.59)	-1.07 (-1.12 to -1.01)	28.7 (25.7 to 30.4)	13.3 (12.0 to 14.0)	33.5 (29.1 to 36.1)	8.66 (7.69 to 9.26)	-1.40 (-1.44 to -1.34)
Southern Latin America	5.01 (4.68 to 5.29)	19.6 (18.2 to 20.7)	6.39 (5.75 to 6.91)	12.1 (11.0 to 13.0)	-1.52 (-1.66 to -1.39)	0.18 (0.17 to 0.19)	0.69 (0.66 to 0.73)	0.16 (0.15 to 0.17)	0.37 (0.35 to 0.39)	-2.02 (-2.12 to -1.94)	4.83 (4.50 to 5.11)	18.9 (17.5 to 20.0)	6.23 (5.60 to 6.75)	11.7 (10.7 to 12.7)	-1.51 (-1.66 to -1.37)
Western Europe	53.7 (48.0 to 56.6)	13.9 (12.6 to 14.7)	46.8 (37.0 to 52.2)	6.43 (5.26 to 7.07)	-2.47 (-2.52 to -2.42)	1.53 (1.44 to 1.60)	0.52 (0.49 to 0.54)	0.66 (0.58 to 0.71)	0.16 (0.15 to 0.17)	-3.74 (-3.80 to -3.69)	52.1 (46.6 to 55.1)	13.4 (12.1 to 14.1)	46.1 (36.4 to 51.5)	6.27 (5.11 to 6.90)	-2.43 (-2.48 to -2.38)
Andean Latin America	2.29 (2.06 to 2.55)	22.8 (20.5 to 25.4)	7.60 (6.40 to 9.13)	24.9 (20.9 to 29.9)	0.31 (0.03 to 0.51)	0.06 (0.05 to 0.07)	0.36 (0.28 to 0.48)	0.09 (0.06 to 0.12)	0.28 (0.19 to 0.37)	-0.72 (-0.89 to -0.53)	2.23 (2.01 to 2.48)	22.4 (20.1 to 24.9)	7.51 (6.31 to 9.03)	24.6 (20.7 to 29.6)	0.33 (0.04 to 0.52)
Caribbean	7.10 (6.65 to 7.63)	54.4 (50.9 to 58.5)	11.7 (10.0 to 13.4)	40.2 (34.2 to 46.2)	-0.94 (-1.03 to -0.86)	0.36 (0.31 to 0.50)	2.34 (1.99 to 3.28)	0.45 (0.34 to 0.63)	1.75 (1.31 to 2.44)	-0.87 (-0.91 to -0.82)	6.74 (6.30 to 7.24)	52.1 (48.7 to 55.9)	11.3 (9.62 to 12.9)	38.5 (32.7 to 44.0)	-0.94 (-1.04 to -0.86)
Central Latin America	21.5 (20.7 to 22.1)	53.7 (51.3 to 55.2)	61.3 (54.0 to 68.2)	46.0 (40.5 to 51.1)	-0.44 (-0.54 to -0.33)	0.55 (0.54 to 0.57)	0.94 (0.91 to 0.97)	0.91 (0.79 to 1.02)	0.67 (0.58 to 0.75)	-0.97 (-1.10 to -0.83)	21.0 (20.2 to 21.5)	52.8 (50.4 to 54.3)	60.4 (53.2 to 67.2)	45.3 (39.9 to 50.4)	-0.43 (-0.53 to -0.32)
Tropical Latin America	15.7 (14.7 to 16.3)	34.9 (32.3 to 36.4)	37.0 (33.0 to 39.5)	26.1 (23.3 to 27.8)	-0.91 (-0.97 to -0.85)	0.39 (0.37 to 0.41)	0.61 (0.58 to 0.64)	0.79 (0.75 to 0.83)	0.61 (0.58 to 0.64)	0.06 (-0.08 to 0.17)	15.3 (14.3 to 15.9)	34.3 (31.7 to 35.8)	36.2 (32.2 to 38.7)	25.4 (22.7 to 27.2)	-0.93 (-0.99 to -0.87)
North Africa and Middle East	19.7 (17.9 to 22.0)	27.1 (24.5 to 30.2)	60.9 (54.3 to 69.1)	31.4 (27.8 to 35.4)	0.47 (0.42 to 0.53)	1.00 (0.77 to 1.45)	0.66 (0.50 to 0.97)	1.30 (1.01 to 1.64)	0.45 (0.36 to 0.57)	-1.13 (-1.18 to -1.09)	18.7 (17.0 to 20.8)	26.5 (23.9 to 29.4)	59.6 (53.1 to 67.6)	30.9 (27.5 to 34.8)	0.51 (0.45 to 0.56)
South Asia	55.7 (47.5 to 61.9)	23.4 (19.8 to 26.3)	219 (193 to 247)	32.3 (28.4 to 36.5)	1.19 (1.08 to 1.38)	4.33 (3.26 to 6.62)	1.10 (0.76 to 1.74)	7.75 (5.82 to 9.92)	0.91 (0.68 to 1.17)	-0.55 (-0.71 to -0.38)	51.4 (43.7 to 57.2)	22.3 (18.8 to 25.1)	212 (186 to 240)	31.4 (27.6 to 35.5)	1.26 (1.15 to 1.45)
Central Sub-Saharan Africa	4.05 (3.00 to 5.13)	39.8 (29.1 to 49.9)	10.9 (8.15 to 13.9)	42.9 (32.2 to 54.9)	0.24 (0.19 to 0.28)	0.14 (0.10 to 0.19)	0.65 (0.43 to 0.98)	0.28 (0.20 to 0.40)	0.54 (0.36 to 0.82)	-0.62 (-0.66 to -0.58)	3.91 (2.88 to 4.99)	39.2 (28.6 to 49.1)	10.6 (7.91 to 13.6)	42.4 (31.7 to 54.0)	0.25 (0.20 to 0.30)
Eastern Sub-Saharan Africa	13.5 (11.6 to 15.6)	39.9 (34.0 to 45.8)	26.4 (22.9 to 30.7)	34.6 (30.2 to 40.1)	-0.45 (-0.47 to -0.42)	0.76 (0.55 to 1.15)	0.90 (0.63 to 1.39)	1.06 (0.77 to 1.38)	0.57 (0.39 to 0.79)	-1.47 (-1.48 to -1.45)	12.8 (10.9 to 14.8)	39.0 (33.3 to 44.8)	25.3 (21.9 to 29.5)	34.1 (29.7 to 39.4)	-0.44 (-0.46 to -0.42)
Southern Sub-Saharan Africa	6.34 (5.70 to 6.96)	43.9 (39.2 to 48.3)	24.0 (22.3 to 25.9)	78.4 (72.6 to 84.2)	1.85 (1.66 to 2.03)	0.11 (0.08 to 0.14)	0.40 (0.32 to 0.52)	0.14 (0.11 to 0.18)	0.35 (0.26 to 0.43)	-0.45 (-0.56 to -0.33)	6.24 (5.60 to 6.84)	43.5 (38.8 to 47.9)	23.9 (22.1 to 25.7)	78.1 (72.3 to 83.9)	1.86 (1.68 to 2.05)
Western Sub-Saharan Africa	11.6 (9.63 to 13.6)	29.7 (24.7 to 34.7)	31.4 (26.3 to 37.8)	36.5 (31.0 to 43.2)	0.66 (0.65 to 0.67)	0.52 (0.35 to 0.68)	0.72 (0.48 to 0.98)	0.89 (0.58 to 1.17)	0.45 (0.30 to 0.60)	-1.47 (-1.50 to -1.44)	11.1 (9.15 to 13.0)	29.0 (24.1 to 33.9)	30.5 (25.5 to 36.7)	36.0 (30.5 to 42.6)	0.70 (0.69 to 0.71)
**204 countries and territories**															
Afghanistan	1.16 (0.86 to 1.73)	36.2 (27.5 to 52.6)	2.92 (2.24 to 3.99)	59.6 (45.8 to 80.4)	1.65 (1.62 to 1.67)	0.08 (0.04 to 0.14)	1.76 (0.95 to 3.30)	0.22 (0.15 to 0.34)	1.77 (1.13 to 2.84)	0.05 (0.00 to 0.11)	1.08 (0.81 to 1.59)	34.4 (26.1 to 49.7)	2.70 (2.05 to 3.64)	57.9 (44.3 to 77.9)	1.71 (1.69 to 1.73)
Albania	0.05 (0.04 to 0.06)	5.20 (4.27 to 6.31)	0.11 (0.09 to 0.14)	4.79 (3.74 to 6.17)	-0.41 (-0.59 to -0.25)	0.008 (0.006 to 0.01)	0.70 (0.47 to 1.00)	0.007 (0.004 to 0.01)	0.33 (0.20 to 0.51)	-2.52 (-2.65 to -2.41)	0.04 (0.04 to 0.06)	4.50 (3.66 to 5.54)	0.11 (0.08 to 0.14)	4.46 (3.45 to 5.77)	-0.17 (-0.35 to -0.01)
Algeria	0.66 (0.51 to 0.90)	15.5 (12.0 to 20.4)	3.14 (2.51 to 3.85)	24.0 (19.4 to 29.0)	1.44 (1.36 to 1.54)	0.06 (0.04 to 0.10)	0.50 (0.31 to 0.86)	0.08 (0.05 to 0.11)	0.36 (0.24 to 0.54)	-1.04 (-1.08 to -0.99)	0.61 (0.46 to 0.83)	15.1 (11.6 to 19.8)	3.07 (2.45 to 3.76)	23.6 (19.0 to 28.6)	1.49 (1.42 to 1.60)
American Samoa	0.007 (0.006 to 0.009)	77.4 (66.8 to 89.5)	0.03 (0.02 to 0.03)	119 (96.3 to 142)	1.50 (1.38 to 1.61)	7e-05 (5e-05 to 1e-04)	0.29 (0.20 to 0.44)	8e-05 (6e-05 to 1e-04)	0.33 (0.23 to 0.45)	0.50 (0.40 to 0.60)	0.007 (0.006 to 0.009)	77.1 (66.5 to 89.2)	0.03 (0.02 to 0.03)	118 (96.0 to 142)	1.50 (1.38 to 1.61)
Andorra	0.002 (0.002 to 0.003)	8.97 (6.52 to 12.3)	0.006 (0.004 to 0.008)	6.67 (4.79 to 8.88)	-1.16 (-1.42 to -1.03)	2e-04 (1e-04 to 2e-04)	0.61 (0.41 to 0.89)	3e-04 (2e-04 to 4e-04)	0.35 (0.24 to 0.49)	-2.05 (-2.28 to -1.91)	0.002 (0.001 to 0.003)	8.35 (6.05 to 11.5)	0.006 (0.004 to 0.008)	6.32 (4.55 to 8.41)	-1.11 (-1.36 to -0.98)
Angola	0.56 (0.43 to 0.73)	33.0 (25.8 to 42.4)	1.91 (1.42 to 2.54)	35.2 (26.6 to 46.1)	0.17 (0.09 to 0.24)	0.02 (0.02 to 0.03)	0.62 (0.42 to 0.92)	0.06 (0.04 to 0.08)	0.46 (0.29 to 0.68)	-0.94 (-1.03 to -0.82)	0.54 (0.41 to 0.70)	32.4 (25.2 to 41.5)	1.86 (1.38 to 2.48)	34.7 (26.2 to 45.6)	0.19 (0.12 to 0.26)
Antigua and Barbuda	0.02 (0.02 to 0.02)	64.2 (58.2 to 69.9)	0.03 (0.03 to 0.03)	59.3 (55.6 to 63.2)	-0.28 (-0.51 to 0.08)	8e-04 (8e-04 to 9e-04)	2.74 (2.45 to 3.01)	9e-04 (8e-04 to 9e-04)	1.60 (1.49 to 1.72)	-1.78 (-1.89 to -1.68)	0.02 (0.02 to 0.02)	61.5 (55.7 to 66.9)	0.03 (0.03 to 0.03)	57.7 (54.0 to 61.6)	-0.22 (-0.47 to 0.16)
Argentina	3.79 (3.52 to 4.04)	21.1 (19.5 to 22.5)	4.41 (3.97 to 4.80)	13.1 (11.9 to 14.2)	-1.49 (-1.63 to -1.32)	0.13 (0.13 to 0.14)	0.76 (0.71 to 0.81)	0.12 (0.11 to 0.13)	0.43 (0.40 to 0.47)	-1.80 (-1.91 to -1.71)	3.66 (3.39 to 3.90)	20.4 (18.8 to 21.7)	4.29 (3.85 to 4.67)	12.7 (11.5 to 13.7)	-1.48 (-1.62 to -1.32)
Armenia	0.32 (0.29 to 0.35)	20.8 (18.8 to 22.7)	0.39 (0.34 to 0.45)	15.4 (13.4 to 17.7)	-1.12 (-1.44 to -0.85)	0.03 (0.03 to 0.03)	1.74 (1.56 to 1.94)	0.02 (0.02 to 0.02)	1.00 (0.88 to 1.15)	-1.94 (-2.19 to -1.71)	0.29 (0.26 to 0.32)	19.0 (17.2 to 20.8)	0.37 (0.32 to 0.42)	14.4 (12.5 to 16.6)	-1.06 (-1.39 to -0.78)
Australia	1.13 (1.00 to 1.23)	9.94 (8.79 to 10.8)	1.92 (1.54 to 2.16)	6.53 (5.39 to 7.29)	-1.34 (-1.45 to -1.24)	0.04 (0.04 to 0.04)	0.39 (0.36 to 0.42)	0.04 (0.03 to 0.04)	0.20 (0.18 to 0.21)	-2.26 (-2.35 to -2.17)	1.09 (0.96 to 1.18)	9.55 (8.41 to 10.4)	1.88 (1.50 to 2.12)	6.33 (5.20 to 7.09)	-1.31 (-1.42 to -1.21)
Austria	1.17 (1.05 to 1.27)	13.9 (12.6 to 14.9)	1.01 (0.80 to 1.14)	7.34 (6.04 to 8.18)	-1.85 (-2.09 to -1.65)	0.03 (0.03 to 0.03)	0.50 (0.46 to 0.54)	0.01 (0.01 to 0.01)	0.14 (0.13 to 0.16)	-3.95 (-4.05 to -3.86)	1.14 (1.02 to 1.23)	13.4 (12.1 to 14.4)	1.00 (0.79 to 1.13)	7.19 (5.90 to 8.04)	-1.79 (-2.03 to -1.58)
Azerbaijan	0.35 (0.28 to 0.43)	11.8 (9.45 to 14.7)	1.01 (0.71 to 1.32)	18.3 (13.1 to 23.9)	1.45 (1.32 to 1.56)	0.05 (0.03 to 0.06)	1.44 (1.02 to 1.93)	0.07 (0.04 to 0.11)	1.24 (0.73 to 1.90)	-0.47 (-0.56 to -0.37)	0.30 (0.24 to 0.38)	10.4 (8.07 to 13.2)	0.93 (0.66 to 1.22)	17.1 (12.2 to 22.4)	1.59 (1.47 to 1.68)
Bahamas	0.05 (0.04 to 0.05)	55.1 (50.3 to 60.2)	0.07 (0.06 to 0.09)	34.9 (28.7 to 42.2)	-1.33 (-2.00 to -0.98)	0.003 (0.002 to 0.003)	2.32 (2.10 to 2.56)	0.003 (0.002 to 0.004)	1.42 (1.13 to 1.80)	-1.60 (-1.69 to -1.51)	0.04 (0.04 to 0.05)	52.8 (48.2 to 57.7)	0.07 (0.06 to 0.08)	33.5 (27.5 to 40.3)	-1.31 (-2.01 to -0.94)
Bahrain	0.06 (0.05 to 0.07)	95.8 (82.5 to 112)	0.29 (0.24 to 0.34)	124 (105 to 143)	0.71 (0.57 to 0.86)	0.002 (0.001 to 0.002)	1.15 (0.84 to 1.49)	0.003 (0.002 to 0.004)	0.55 (0.42 to 0.74)	-2.35 (-2.45 to -2.26)	0.05 (0.05 to 0.06)	94.6 (81.5 to 111)	0.29 (0.24 to 0.34)	123 (105 to 142)	0.73 (0.60 to 0.89)
Bangladesh	5.49 (4.57 to 6.36)	31.2 (25.4 to 36.5)	22.6 (17.6 to 28.3)	42.6 (33.2 to 53.5)	1.24 (1.08 to 1.39)	0.58 (0.40 to 0.90)	1.63 (1.01 to 2.51)	0.98 (0.64 to 1.34)	1.24 (0.81 to 1.71)	-0.75 (-0.84 to -0.66)	4.91 (4.03 to 5.67)	29.5 (23.8 to 34.5)	21.6 (16.8 to 27.2)	41.4 (32.2 to 52.1)	1.32 (1.15 to 1.48)
Barbados	0.15 (0.14 to 0.16)	82.6 (76.5 to 88.7)	0.18 (0.15 to 0.22)	62.9 (51.3 to 75.6)	-0.71 (-0.96 to -0.47)	0.005 (0.004 to 0.005)	3.02 (2.79 to 3.27)	0.004 (0.003 to 0.006)	1.89 (1.45 to 2.35)	-1.46 (-1.62 to -1.37)	0.15 (0.13 to 0.16)	79.6 (73.7 to 85.4)	0.18 (0.15 to 0.22)	61.1 (49.9 to 73.3)	-0.68 (-0.93 to -0.44)
Belarus	0.38 (0.35 to 0.41)	4.58 (4.28 to 4.92)	0.40 (0.33 to 0.47)	3.94 (3.24 to 4.76)	-0.66 (-1.25 to -0.26)	0.04 (0.04 to 0.05)	0.65 (0.57 to 0.76)	0.03 (0.03 to 0.04)	0.58 (0.46 to 0.72)	0.15 (-0.40 to 0.90)	0.34 (0.31 to 0.36)	3.93 (3.64 to 4.25)	0.36 (0.30 to 0.43)	3.36 (2.77 to 4.05)	-0.63 (-1.16 to -0.25)
Belgium	1.27 (1.12 to 1.40)	12.4 (11.0 to 13.6)	0.76 (0.58 to 0.88)	4.26 (3.35 to 4.82)	-3.32 (-3.58 to -3.06)	0.05 (0.05 to 0.06)	0.65 (0.59 to 0.70)	0.02 (0.02 to 0.02)	0.19 (0.17 to 0.21)	-4.10 (-4.23 to -4.00)	1.22 (1.07 to 1.34)	11.8 (10.4 to 12.9)	0.74 (0.56 to 0.85)	4.07 (3.19 to 4.62)	-3.28 (-3.55 to -3.01)
Belize	0.03 (0.03 to 0.03)	60.1 (55.6 to 65.0)	0.09 (0.08 to 0.10)	63.4 (56.2 to 71.1)	0.12 (-0.19 to 0.44)	0.001 (0.001 to 0.001)	1.74 (1.60 to 1.90)	0.002 (0.002 to 0.002)	1.16 (1.01 to 1.33)	-1.27 (-1.41 to -1.13)	0.03 (0.03 to 0.03)	58.4 (53.9 to 63.1)	0.09 (0.08 to 0.10)	62.2 (55.1 to 69.8)	0.16 (-0.16 to 0.47)
Benin	0.23 (0.18 to 0.28)	24.2 (19.2 to 29.3)	0.82 (0.65 to 1.00)	33.8 (27.3 to 40.8)	1.09 (1.06 to 1.12)	0.01 (0.009 to 0.02)	0.67 (0.43 to 0.97)	0.03 (0.02 to 0.04)	0.47 (0.29 to 0.67)	-1.12 (-1.19 to -1.08)	0.22 (0.17 to 0.27)	23.6 (18.7 to 28.5)	0.79 (0.63 to 0.97)	33.3 (26.9 to 40.2)	1.13 (1.10 to 1.17)
Bermuda	0.01 (0.01 to 0.01)	34.0 (31.0 to 36.9)	0.01 (0.010 to 0.02)	13.7 (11.1 to 17.3)	-2.98 (-3.22 to -2.78)	5e-04 (5e-04 to 6e-04)	1.52 (1.36 to 1.69)	3e-04 (3e-04 to 4e-04)	0.49 (0.40 to 0.61)	-3.67 (-3.79 to -3.56)	0.01 (0.01 to 0.01)	32.5 (29.6 to 35.2)	0.01 (0.010 to 0.02)	13.2 (10.7 to 16.7)	-2.95 (-3.21 to -2.74)
Bhutan	0.03 (0.02 to 0.03)	23.2 (16.8 to 30.6)	0.09 (0.06 to 0.11)	31.4 (22.5 to 40.5)	1.01 (0.97 to 1.04)	0.002 (0.001 to 0.004)	1.07 (0.58 to 1.87)	0.003 (0.002 to 0.004)	0.79 (0.49 to 1.14)	-1.00 (-1.05 to -0.92)	0.02 (0.02 to 0.03)	22.1 (15.9 to 29.2)	0.09 (0.06 to 0.11)	30.7 (21.9 to 39.6)	1.08 (1.04 to 1.11)
Bolivia (Plurinational State of)	0.78 (0.65 to 0.97)	48.6 (40.4 to 60.1)	2.18 (1.72 to 2.86)	48.6 (38.6 to 62.6)	0.02 (-0.01 to 0.04)	0.02 (0.01 to 0.03)	0.78 (0.51 to 1.41)	0.03 (0.01 to 0.05)	0.51 (0.25 to 0.86)	-1.37 (-1.40 to -1.35)	0.76 (0.63 to 0.93)	47.9 (39.8 to 58.6)	2.15 (1.70 to 2.82)	48.1 (38.3 to 61.8)	0.03 (0.01 to 0.06)
Bosnia and Herzegovina	0.38 (0.33 to 0.44)	18.0 (15.4 to 21.2)	1.18 (0.93 to 1.45)	30.9 (24.6 to 38.2)	1.83 (1.69 to 1.95)	0.04 (0.03 to 0.05)	1.79 (1.23 to 2.32)	0.03 (0.02 to 0.05)	0.85 (0.56 to 1.52)	-2.22 (-2.33 to -2.11)	0.34 (0.29 to 0.40)	16.2 (13.7 to 19.0)	1.15 (0.91 to 1.42)	30.0 (23.9 to 37.1)	2.07 (1.95 to 2.19)
Botswana	0.16 (0.11 to 0.21)	58.2 (41.8 to 76.8)	0.43 (0.34 to 0.54)	60.6 (48.0 to 75.6)	-0.03 (-0.19 to 0.16)	0.003 (0.002 to 0.004)	0.42 (0.24 to 0.62)	0.003 (0.002 to 0.005)	0.27 (0.15 to 0.41)	-1.34 (-1.45 to -1.22)	0.15 (0.11 to 0.21)	57.7 (41.5 to 76.2)	0.43 (0.34 to 0.54)	60.3 (47.7 to 75.2)	-0.03 (-0.18 to 0.17)
Brazil	15.4 (14.3 to 16.0)	35.0 (32.3 to 36.6)	35.3 (31.4 to 37.7)	25.4 (22.6 to 27.1)	-1.00 (-1.07 to -0.95)	0.38 (0.36 to 0.40)	0.61 (0.58 to 0.65)	0.77 (0.73 to 0.81)	0.61 (0.58 to 0.64)	0.06 (-0.08 to 0.18)	15.0 (14.0 to 15.6)	34.4 (31.8 to 36.0)	34.6 (30.6 to 36.9)	24.8 (22.0 to 26.5)	-1.02 (-1.10 to -0.97)
Brunei Darussalam	0.04 (0.03 to 0.04)	83.3 (72.8 to 95.4)	0.07 (0.06 to 0.08)	47.8 (40.4 to 56.4)	-1.90 (-2.01 to -1.82)	0.002 (0.001 to 0.003)	2.40 (1.61 to 3.29)	0.002 (0.002 to 0.003)	0.90 (0.70 to 1.24)	-3.17 (-3.24 to -3.10)	0.03 (0.03 to 0.04)	80.9 (70.8 to 92.8)	0.07 (0.06 to 0.08)	46.9 (39.7 to 55.4)	-1.87 (-1.98 to -1.79)
Bulgaria	1.16 (1.06 to 1.26)	17.9 (16.5 to 19.5)	1.31 (1.15 to 1.48)	14.6 (12.8 to 16.6)	-0.63 (-0.77 to -0.48)	0.09 (0.08 to 0.10)	1.51 (1.34 to 1.69)	0.05 (0.04 to 0.06)	0.75 (0.61 to 0.92)	-2.22 (-2.37 to -2.08)	1.07 (0.98 to 1.17)	16.4 (15.1 to 17.9)	1.26 (1.10 to 1.43)	13.9 (12.1 to 15.7)	-0.51 (-0.67 to -0.37)
Burkina Faso	0.56 (0.45 to 0.70)	29.4 (23.5 to 35.9)	1.10 (0.87 to 1.36)	25.8 (21.0 to 31.9)	-0.40 (-0.45 to -0.35)	0.03 (0.02 to 0.05)	0.86 (0.59 to 1.27)	0.05 (0.03 to 0.07)	0.47 (0.31 to 0.69)	-1.90 (-1.95 to -1.86)	0.53 (0.42 to 0.66)	28.5 (22.7 to 35.0)	1.05 (0.83 to 1.30)	25.3 (20.5 to 31.3)	-0.36 (-0.42 to -0.31)
Burundi	0.53 (0.37 to 0.71)	45.9 (31.9 to 60.4)	0.79 (0.55 to 1.06)	40.2 (27.8 to 54.0)	-0.43 (-0.47 to -0.39)	0.02 (0.02 to 0.03)	0.91 (0.58 to 1.32)	0.04 (0.02 to 0.05)	0.68 (0.40 to 1.04)	-0.98 (-1.01 to -0.95)	0.51 (0.35 to 0.68)	45.0 (31.3 to 59.2)	0.76 (0.52 to 1.02)	39.5 (27.4 to 53.0)	-0.42 (-0.46 to -0.38)
Cabo Verde	0.01 (0.01 to 0.02)	10.1 (8.26 to 12.1)	0.08 (0.07 to 0.10)	33.2 (26.4 to 40.7)	3.93 (3.85 to 4.02)	6e-04 (4e-04 to 9e-04)	0.38 (0.22 to 0.61)	7e-04 (4e-04 to 0.001)	0.26 (0.16 to 0.39)	-1.25 (-1.30 to -1.17)	0.01 (0.01 to 0.02)	9.70 (7.86 to 11.8)	0.08 (0.07 to 0.10)	32.9 (26.2 to 40.4)	4.03 (3.95 to 4.13)
Cambodia	0.72 (0.59 to 0.91)	28.8 (23.4 to 36.4)	2.15 (1.54 to 3.01)	32.4 (23.6 to 44.5)	0.38 (0.36 to 0.40)	0.06 (0.04 to 0.11)	1.42 (0.94 to 2.76)	0.07 (0.05 to 0.11)	0.90 (0.57 to 1.34)	-1.47 (-1.50 to -1.44)	0.67 (0.54 to 0.84)	27.4 (22.2 to 34.3)	2.07 (1.47 to 2.93)	31.5 (22.8 to 43.5)	0.46 (0.43 to 0.47)
Cameroon	0.68 (0.50 to 0.86)	34.4 (25.8 to 43.6)	2.47 (1.75 to 3.46)	45.0 (33.1 to 61.1)	0.86 (0.83 to 0.89)	0.03 (0.02 to 0.05)	0.85 (0.52 to 1.27)	0.07 (0.04 to 0.10)	0.55 (0.33 to 0.89)	-1.40 (-1.44 to -1.36)	0.64 (0.48 to 0.82)	33.5 (25.3 to 42.5)	2.41 (1.70 to 3.35)	44.5 (32.7 to 60.3)	0.90 (0.87 to 0.93)
Canada	2.39 (2.14 to 2.58)	12.4 (11.1 to 13.3)	2.82 (2.37 to 3.12)	6.26 (5.44 to 6.83)	-2.10 (-2.20 to -2.00)	0.19 (0.17 to 0.20)	1.06 (0.99 to 1.13)	0.16 (0.14 to 0.17)	0.54 (0.50 to 0.57)	-2.15 (-2.22 to -2.08)	2.20 (1.97 to 2.39)	11.3 (10.1 to 12.2)	2.66 (2.23 to 2.95)	5.72 (4.93 to 6.26)	-2.11 (-2.21 to -2.01)
Central African Republic	0.25 (0.19 to 0.32)	48.1 (35.4 to 60.7)	0.51 (0.37 to 0.69)	50.9 (36.5 to 66.4)	0.17 (0.14 to 0.20)	0.009 (0.006 to 0.01)	0.84 (0.57 to 1.37)	0.02 (0.01 to 0.03)	0.79 (0.53 to 1.19)	-0.16 (-0.24 to -0.07)	0.25 (0.18 to 0.31)	47.3 (34.7 to 59.4)	0.49 (0.35 to 0.67)	50.1 (35.9 to 65.4)	0.18 (0.15 to 0.21)

Location	DM, Number in 1990 (000s)	DM, ASR in 1990 (per 100,000)	DM, Number in 2021 (000s)	DM, ASR in 2021 (per 100,000)	DM, AAPC (95%CI)	T1D, Number in 1990 (000s)	T1D, ASR in 1990 (per 100,000)	T1D, Number in 2021 (000s)	T1D, ASR in 2021 (per 100,000)	T1D, AAPC (95%CI)	T2D, Number in 1990 (000s)	T2D, ASR in 1990 (per 100,000)	T2D, Number in 2021 (000s)	T2D, ASR in 2021 (per 100,000)	T2D, AAPC (95%CI)
Chad	0.31 (0.24 to 0.41)	23.3 (17.6 to 30.3)	0.93 (0.71 to 1.21)	40.0 (31.3 to 51.8)	1.80 (1.77 to 1.82)	0.02 (0.01 to 0.03)	0.68 (0.44 to 1.04)	0.04 (0.03 to 0.06)	0.65 (0.43 to 1.03)	-0.09 (-0.16 to -0.03)	0.30 (0.23 to 0.39)	22.6 (17.1 to 29.4)	0.89 (0.68 to 1.15)	39.4 (30.8 to 51.0)	1.84 (1.82 to 1.86)
Chile	0.83 (0.78 to 0.89)	15.6 (14.5 to 16.6)	1.47 (1.28 to 1.60)	9.67 (8.49 to 10.5)	-1.47 (-1.76 to -1.22)	0.04 (0.03 to 0.04)	0.61 (0.57 to 0.65)	0.03 (0.03 to 0.03)	0.24 (0.22 to 0.26)	-2.89 (-2.99 to -2.79)	0.80 (0.74 to 0.85)	15.0 (13.9 to 16.0)	1.44 (1.25 to 1.57)	9.43 (8.26 to 10.3)	-1.43 (-1.71 to -1.17)
China	41.9 (34.7 to 50.6)	10.5 (8.75 to 12.6)	90.5 (71.6 to 112)	8.31 (6.59 to 10.2)	-0.71 (-0.79 to -0.63)	3.50 (2.62 to 4.89)	0.68 (0.50 to 0.97)	2.04 (1.45 to 2.86)	0.23 (0.17 to 0.32)	-3.45 (-3.51 to -3.39)	38.4 (31.9 to 46.1)	9.84 (8.19 to 11.8)	88.5 (70.1 to 109)	8.08 (6.42 to 9.95)	-0.58 (-0.66 to -0.50)
Colombia	2.08 (1.95 to 2.19)	24.6 (22.8 to 26.0)	3.87 (3.24 to 4.50)	12.4 (10.5 to 14.4)	-2.10 (-2.26 to -1.96)	0.04 (0.04 to 0.05)	0.32 (0.30 to 0.34)	0.06 (0.05 to 0.07)	0.23 (0.19 to 0.27)	-1.17 (-1.38 to -0.97)	2.04 (1.91 to 2.15)	24.3 (22.5 to 25.7)	3.80 (3.19 to 4.42)	12.2 (10.3 to 14.1)	-2.12 (-2.28 to -1.98)
Comoros	0.03 (0.02 to 0.05)	39.2 (27.1 to 53.5)	0.09 (0.06 to 0.13)	38.2 (26.6 to 53.7)	-0.11 (-0.17 to -0.05)	0.002 (0.001 to 0.002)	0.80 (0.49 to 1.19)	0.002 (0.001 to 0.003)	0.65 (0.39 to 1.02)	-0.76 (-1.02 to -0.51)	0.03 (0.02 to 0.05)	38.4 (26.5 to 52.4)	0.09 (0.06 to 0.12)	37.6 (26.2 to 52.7)	-0.11 (-0.16 to -0.06)
Congo	0.27 (0.21 to 0.35)	53.1 (40.8 to 66.8)	0.66 (0.50 to 0.85)	55.4 (42.7 to 70.7)	0.16 (0.11 to 0.21)	0.007 (0.005 to 0.01)	0.74 (0.50 to 1.27)	0.01 (0.009 to 0.02)	0.59 (0.39 to 0.95)	-0.72 (-0.83 to -0.59)	0.27 (0.20 to 0.34)	52.3 (40.1 to 66.0)	0.64 (0.49 to 0.84)	54.8 (42.3 to 70.0)	0.17 (0.12 to 0.22)
Cook Islands	0.008 (0.007 to 0.010)	146 (123 to 174)	0.01 (0.01 to 0.02)	103 (80.9 to 130)	-1.12 (-1.15 to -1.09)	9e-05 (7e-05 to 1e-04)	1.10 (0.79 to 1.57)	6e-05 (4e-05 to 8e-05)	0.61 (0.42 to 0.84)	-1.79 (-2.00 to -1.62)	0.008 (0.007 to 0.009)	145 (122 to 173)	0.01 (0.01 to 0.02)	103 (80.4 to 129)	-1.11 (-1.14 to -1.09)
Costa Rica	0.16 (0.14 to 0.17)	17.8 (16.3 to 19.1)	0.57 (0.49 to 0.64)	18.7 (16.2 to 20.9)	0.47 (-0.18 to 1.15)	0.003 (0.003 to 0.003)	0.28 (0.26 to 0.30)	0.007 (0.007 to 0.008)	0.26 (0.23 to 0.29)	-0.21 (-0.40 to -0.01)	0.15 (0.14 to 0.16)	17.5 (16.1 to 18.9)	0.56 (0.48 to 0.63)	18.5 (16.0 to 20.6)	0.49 (-0.18 to 1.18)
Croatia	0.47 (0.43 to 0.51)	13.5 (12.2 to 14.7)	0.89 (0.76 to 0.99)	14.0 (12.1 to 15.6)	0.05 (-0.13 to 0.21)	0.04 (0.04 to 0.05)	1.25 (1.14 to 1.37)	0.02 (0.01 to 0.02)	0.37 (0.31 to 0.44)	-3.87 (-3.98 to -3.77)	0.43 (0.39 to 0.47)	12.2 (11.0 to 13.4)	0.87 (0.74 to 0.97)	13.6 (11.7 to 15.2)	0.28 (0.09 to 0.45)
Cuba	1.46 (1.36 to 1.55)	28.6 (26.7 to 30.3)	1.17 (1.01 to 1.33)	10.5 (9.07 to 12.0)	-3.19 (-3.39 to -3.00)	0.06 (0.05 to 0.06)	1.04 (0.98 to 1.11)	0.03 (0.03 to 0.04)	0.40 (0.34 to 0.47)	-2.94 (-3.10 to -2.76)	1.40 (1.31 to 1.49)	27.6 (25.7 to 29.2)	1.14 (0.98 to 1.29)	10.1 (8.71 to 11.5)	-3.20 (-3.40 to -3.00)
Cyprus	0.27 (0.22 to 0.32)	78.6 (65.2 to 93.0)	0.27 (0.22 to 0.32)	26.9 (22.3 to 31.9)	-3.36 (-3.52 to -3.19)	0.007 (0.005 to 0.010)	1.86 (1.25 to 2.56)	0.005 (0.004 to 0.007)	0.50 (0.39 to 0.66)	-4.19 (-4.34 to -4.03)	0.26 (0.21 to 0.31)	76.7 (63.6 to 90.7)	0.26 (0.21 to 0.31)	26.4 (21.9 to 31.4)	-3.34 (-3.50 to -3.16)
Czechia	1.22 (1.12 to 1.33)	14.1 (12.9 to 15.4)	2.29 (1.95 to 2.61)	15.7 (13.4 to 17.9)	0.67 (0.25 to 1.14)	0.12 (0.11 to 0.13)	1.53 (1.43 to 1.64)	0.04 (0.03 to 0.05)	0.39 (0.34 to 0.44)	-4.53 (-4.66 to -4.41)	1.10 (1.00 to 1.21)	12.5 (11.4 to 13.8)	2.25 (1.92 to 2.56)	15.3 (13.1 to 17.4)	1.05 (0.61 to 1.56)
Côte d'Ivoire	0.38 (0.29 to 0.48)	25.6 (20.5 to 32.1)	1.70 (1.28 to 2.22)	39.1 (30.4 to 51.2)	1.36 (1.31 to 1.40)	0.03 (0.02 to 0.04)	0.62 (0.36 to 0.92)	0.05 (0.03 to 0.07)	0.47 (0.30 to 0.67)	-0.91 (-0.97 to -0.85)	0.35 (0.27 to 0.45)	25.0 (20.0 to 31.4)	1.65 (1.24 to 2.17)	38.6 (30.1 to 50.6)	1.40 (1.35 to 1.44)
Democratic People's Republic of Korea	1.33 (0.97 to 1.70)	14.5 (10.6 to 18.1)	2.94 (2.22 to 3.94)	15.2 (11.5 to 20.3)	0.16 (0.15 to 0.18)	0.10 (0.06 to 0.16)	0.91 (0.55 to 1.60)	0.12 (0.07 to 0.22)	0.75 (0.44 to 1.32)	-0.64 (-0.65 to -0.61)	1.23 (0.91 to 1.58)	13.6 (9.93 to 17.1)	2.82 (2.13 to 3.80)	14.4 (10.9 to 19.5)	0.22 (0.20 to 0.23)
Democratic Republic of the Congo	2.79 (1.92 to 3.70)	39.4 (26.9 to 51.2)	7.45 (5.12 to 10.0)	43.8 (29.9 to 58.8)	0.36 (0.30 to 0.41)	0.09 (0.06 to 0.13)	0.64 (0.40 to 1.02)	0.19 (0.12 to 0.27)	0.56 (0.35 to 0.88)	-0.48 (-0.54 to -0.42)	2.69 (1.85 to 3.57)	38.7 (26.4 to 50.3)	7.26 (4.98 to 9.77)	43.2 (29.5 to 58.2)	0.37 (0.31 to 0.42)
Denmark	0.44 (0.40 to 0.47)	8.70 (7.98 to 9.33)	0.60 (0.50 to 0.67)	7.53 (6.35 to 8.31)	-0.57 (-0.88 to -0.34)	0.04 (0.03 to 0.04)	0.92 (0.86 to 0.99)	0.02 (0.01 to 0.02)	0.29 (0.26 to 0.32)	-3.65 (-3.75 to -3.55)	0.40 (0.36 to 0.43)	7.78 (7.12 to 8.34)	0.59 (0.48 to 0.65)	7.24 (6.07 to 8.01)	-0.33 (-0.63 to -0.10)
Djibouti	0.02 (0.01 to 0.02)	32.6 (24.2 to 43.4)	0.09 (0.06 to 0.13)	40.2 (28.1 to 57.2)	0.66 (0.63 to 0.68)	1e-03 (7e-04 to 0.001)	0.60 (0.39 to 0.86)	0.003 (0.002 to 0.004)	0.53 (0.31 to 0.81)	-0.22 (-0.37 to -0.07)	0.02 (0.01 to 0.02)	32.0 (23.7 to 42.6)	0.09 (0.06 to 0.13)	39.7 (27.8 to 56.5)	0.68 (0.65 to 0.70)
Dominica	0.03 (0.02 to 0.03)	76.0 (68.1 to 84.3)	0.03 (0.03 to 0.04)	76.7 (66.9 to 89.6)	0.04 (0.00 to 0.07)	7e-04 (5e-04 to 9e-04)	1.96 (1.57 to 2.43)	6e-04 (5e-04 to 8e-04)	1.63 (1.23 to 2.08)	-0.54 (-0.58 to -0.50)	0.03 (0.02 to 0.03)	74.1 (66.3 to 82.3)	0.03 (0.03 to 0.04)	75.0 (65.5 to 87.3)	0.05 (0.02 to 0.09)
Dominican Republic	0.46 (0.40 to 0.53)	27.0 (23.4 to 30.7)	1.54 (1.25 to 1.91)	29.7 (24.2 to 36.9)	0.40 (0.31 to 0.48)	0.03 (0.02 to 0.04)	1.10 (0.85 to 1.57)	0.04 (0.03 to 0.06)	0.82 (0.56 to 1.18)	-0.93 (-1.01 to -0.85)	0.43 (0.37 to 0.49)	25.9 (22.4 to 29.6)	1.49 (1.21 to 1.85)	28.9 (23.5 to 35.9)	0.44 (0.35 to 0.52)
Ecuador	0.67 (0.63 to 0.71)	26.5 (24.8 to 28.1)	2.39 (1.95 to 2.94)	28.4 (23.2 to 34.7)	0.12 (-0.15 to 0.35)	0.01 (0.01 to 0.01)	0.31 (0.29 to 0.33)	0.03 (0.02 to 0.04)	0.35 (0.27 to 0.44)	0.45 (0.29 to 0.58)	0.66 (0.62 to 0.69)	26.2 (24.5 to 27.7)	2.36 (1.92 to 2.90)	28.0 (23.0 to 34.3)	0.12 (-0.16 to 0.35)
Egypt	3.81 (3.40 to 4.25)	38.5 (34.1 to 43.6)	14.5 (11.7 to 17.5)	79.2 (66.1 to 92.9)	2.36 (2.26 to 2.48)	0.14 (0.10 to 0.23)	0.57 (0.39 to 1.04)	0.23 (0.15 to 0.30)	0.57 (0.37 to 0.75)	0.11 (-0.00 to 0.24)	3.67 (3.27 to 4.12)	37.9 (33.5 to 43.0)	14.2 (11.6 to 17.2)	78.6 (65.6 to 92.2)	2.39 (2.29 to 2.51)
El Salvador	0.38 (0.34 to 0.42)	23.7 (21.2 to 26.4)	1.64 (1.31 to 2.06)	44.7 (35.9 to 56.0)	2.23 (2.00 to 2.50)	0.02 (0.01 to 0.02)	0.72 (0.54 to 1.03)	0.02 (0.01 to 0.03)	0.59 (0.38 to 0.82)	-0.57 (-0.73 to -0.35)	0.36 (0.32 to 0.40)	23.0 (20.6 to 25.6)	1.62 (1.30 to 2.03)	44.1 (35.5 to 55.2)	2.30 (2.08 to 2.57)
Equatorial Guinea	0.04 (0.03 to 0.06)	41.7 (31.0 to 57.3)	0.11 (0.07 to 0.16)	44.7 (29.2 to 65.2)	0.29 (0.20 to 0.40)	0.001 (8e-04 to 0.002)	0.74 (0.47 to 1.26)	0.002 (0.001 to 0.003)	0.37 (0.21 to 0.61)	-2.15 (-2.23 to -2.08)	0.04 (0.03 to 0.05)	41.0 (30.5 to 56.4)	0.11 (0.07 to 0.16)	44.4 (29.0 to 64.6)	0.32 (0.23 to 0.43)
Eritrea	0.25 (0.19 to 0.31)	46.0 (35.9 to 58.1)	0.68 (0.50 to 0.89)	52.9 (39.2 to 67.5)	0.44 (0.42 to 0.45)	0.01 (0.009 to 0.02)	0.90 (0.64 to 1.38)	0.02 (0.02 to 0.04)	0.87 (0.56 to 1.26)	-0.13 (-0.16 to -0.10)	0.23 (0.18 to 0.29)	45.1 (35.2 to 57.0)	0.66 (0.48 to 0.85)	52.0 (38.5 to 66.3)	0.45 (0.43 to 0.46)
Estonia	0.06 (0.05 to 0.06)	4.58 (4.19 to 4.96)	0.17 (0.15 to 0.20)	9.16 (7.88 to 10.4)	2.41 (2.02 to 2.72)	0.01 (0.01 to 0.01)	1.07 (0.96 to 1.20)	0.007 (0.006 to 0.007)	0.76 (0.66 to 0.86)	-1.23 (-1.65 to -0.83)	0.05 (0.04 to 0.05)	3.51 (3.15 to 3.86)	0.17 (0.14 to 0.19)	8.40 (7.18 to 9.59)	3.02 (2.63 to 3.36)
Eswatini	0.11 (0.08 to 0.13)	73.4 (56.6 to 91.9)	0.32 (0.22 to 0.44)	109 (76.5 to 145)	1.27 (1.22 to 1.31)	0.002 (0.001 to 0.003)	0.45 (0.29 to 0.63)	0.003 (0.001 to 0.004)	0.46 (0.21 to 0.73)	0.06 (0.02 to 0.10)	0.10 (0.08 to 0.13)	72.9 (56.2 to 91.4)	0.32 (0.22 to 0.44)	108 (76.2 to 144)	1.27 (1.22 to 1.31)
Ethiopia	5.04 (4.17 to 6.35)	59.0 (48.5 to 74.0)	5.87 (4.83 to 7.10)	31.3 (25.9 to 37.5)	-2.01 (-2.06 to -1.96)	0.32 (0.22 to 0.56)	1.47 (1.02 to 2.73)	0.26 (0.17 to 0.37)	0.55 (0.35 to 0.82)	-3.09 (-3.12 to -3.05)	4.72 (3.88 to 6.01)	57.5 (47.4 to 72.2)	5.61 (4.63 to 6.78)	30.7 (25.5 to 36.8)	-1.98 (-2.03 to -1.93)
Fiji	0.29 (0.24 to 0.35)	170 (142 to 207)	0.95 (0.75 to 1.20)	254 (201 to 318)	1.26 (1.10 to 1.41)	0.003 (0.003 to 0.005)	1.02 (0.75 to 1.41)	0.004 (0.003 to 0.005)	0.86 (0.60 to 1.18)	-0.47 (-0.54 to -0.40)	0.28 (0.24 to 0.35)	169 (141 to 206)	0.95 (0.74 to 1.20)	253 (200 to 316)	1.27 (1.11 to 1.42)
Finland	0.36 (0.32 to 0.40)	7.39 (6.56 to 8.07)	0.28 (0.22 to 0.31)	3.06 (2.54 to 3.43)	-2.67 (-2.91 to -2.47)	0.06 (0.05 to 0.06)	1.29 (1.18 to 1.41)	0.02 (0.02 to 0.02)	0.32 (0.28 to 0.35)	-4.36 (-4.52 to -4.19)	0.30 (0.27 to 0.34)	6.10 (5.36 to 6.71)	0.26 (0.20 to 0.29)	2.75 (2.24 to 3.08)	-2.43 (-2.69 to -2.19)
France	5.00 (4.38 to 5.46)	8.59 (7.59 to 9.32)	6.65 (5.31 to 7.47)	5.42 (4.49 to 6.03)	-1.38 (-1.51 to -1.27)	0.18 (0.16 to 0.20)	0.39 (0.36 to 0.42)	0.10 (0.09 to 0.12)	0.15 (0.13 to 0.16)	-3.15 (-3.22 to -3.08)	4.82 (4.22 to 5.28)	8.19 (7.21 to 8.92)	6.55 (5.22 to 7.36)	5.28 (4.36 to 5.88)	-1.32 (-1.45 to -1.20)
Gabon	0.13 (0.10 to 0.17)	46.6 (33.7 to 60.3)	0.23 (0.16 to 0.31)	49.8 (34.2 to 66.0)	0.24 (0.18 to 0.30)	0.002 (0.001 to 0.004)	0.61 (0.35 to 1.00)	0.003 (0.002 to 0.005)	0.41 (0.24 to 0.69)	-1.26 (-1.33 to -1.17)	0.13 (0.10 to 0.17)	46.0 (33.2 to 59.6)	0.23 (0.16 to 0.31)	49.4 (34.0 to 65.6)	0.26 (0.20 to 0.32)
Gambia	0.04 (0.03 to 0.05)	24.8 (19.1 to 31.9)	0.20 (0.15 to 0.26)	43.0 (32.0 to 56.9)	1.90 (1.79 to 2.01)	0.002 (0.001 to 0.003)	0.60 (0.41 to 0.85)	0.005 (0.003 to 0.008)	0.53 (0.36 to 0.92)	-0.31 (-0.59 to -0.07)	0.03 (0.03 to 0.05)	24.2 (18.5 to 31.2)	0.19 (0.14 to 0.25)	42.5 (31.6 to 56.3)	1.94 (1.83 to 2.05)
Georgia	0.40 (0.35 to 0.45)	10.3 (9.04 to 11.7)	0.59 (0.51 to 0.67)	15.7 (13.5 to 18.0)	1.49 (1.04 to 2.00)	0.04 (0.03 to 0.05)	1.21 (1.01 to 1.43)	0.02 (0.02 to 0.03)	0.90 (0.73 to 1.08)	-1.04 (-1.30 to -0.80)	0.36 (0.31 to 0.41)	9.11 (8.00 to 10.4)	0.57 (0.49 to 0.64)	14.8 (12.7 to 16.9)	1.74 (1.34 to 2.25)
Germany	14.4 (12.9 to 15.6)	15.8 (14.3 to 17.1)	10.6 (8.24 to 12.0)	7.21 (5.83 to 8.03)	-2.44 (-2.56 to -2.32)	0.51 (0.47 to 0.55)	0.73 (0.68 to 0.78)	0.17 (0.15 to 0.19)	0.20 (0.19 to 0.22)	-4.02 (-4.10 to -3.95)	13.9 (12.4 to 15.1)	15.1 (13.6 to 16.3)	10.4 (8.09 to 11.8)	7.00 (5.64 to 7.81)	-2.38 (-2.50 to -2.26)
Ghana	0.81 (0.63 to 1.05)	28.9 (22.6 to 36.6)	3.00 (2.30 to 3.93)	38.4 (29.7 to 50.9)	0.91 (0.86 to 0.94)	0.03 (0.02 to 0.04)	0.44 (0.30 to 0.60)	0.05 (0.03 to 0.07)	0.34 (0.21 to 0.51)	-0.85 (-0.89 to -0.82)	0.79 (0.61 to 1.02)	28.5 (22.3 to 36.0)	2.95 (2.26 to 3.87)	38.1 (29.5 to 50.4)	0.93 (0.89 to 0.96)
Greece	0.73 (0.66 to 0.80)	8.73 (7.78 to 9.48)	0.90 (0.75 to 1.00)	5.44 (4.68 to 6.05)	-1.53 (-1.77 to -1.25)	0.01 (0.01 to 0.01)	0.16 (0.15 to 0.18)	0.006 (0.005 to 0.007)	0.06 (0.06 to 0.07)	-3.15 (-3.21 to -3.08)	0.72 (0.65 to 0.79)	8.57 (7.63 to 9.31)	0.89 (0.74 to 1.00)	5.38 (4.62 to 5.98)	-1.51 (-1.75 to -1.22)
Greenland	0.002 (0.002 to 0.002)	14.1 (12.0 to 16.5)	0.002 (0.001 to 0.002)	6.28 (5.16 to 7.72)	-2.45 (-2.57 to -2.35)	6e-05 (4e-05 to 8e-05)	0.25 (0.16 to 0.32)	3e-05 (2e-05 to 4e-05)	0.11 (0.07 to 0.14)	-2.71 (-2.84 to -2.58)	0.002 (0.002 to 0.002)	13.8 (11.8 to 16.2)	0.002 (0.001 to 0.002)	6.18 (5.06 to 7.62)	-2.45 (-2.56 to -2.35)
Grenada	0.05 (0.04 to 0.05)	99.7 (90.8 to 109)	0.05 (0.04 to 0.05)	79.9 (70.9 to 88.3)	-0.52 (-0.78 to -0.21)	1e-03 (9e-04 to 0.001)	2.40 (2.14 to 2.67)	8e-04 (7e-04 to 9e-04)	1.37 (1.18 to 1.57)	-1.76 (-1.90 to -1.64)	0.05 (0.04 to 0.05)	97.3 (88.5 to 106)	0.05 (0.04 to 0.05)	78.5 (69.7 to 86.7)	-0.50 (-0.75 to -0.19)
Guam	0.02 (0.01 to 0.02)	54.7 (47.7 to 62.6)	0.02 (0.02 to 0.02)	18.3 (15.4 to 21.3)	-3.73 (-3.97 to -3.48)	8e-05 (6e-05 to 1e-04)	0.13 (0.10 to 0.18)	5e-05 (4e-05 to 6e-05)	0.06 (0.04 to 0.08)	-2.91 (-3.45 to -2.45)	0.02 (0.01 to 0.02)	54.6 (47.6 to 62.5)	0.02 (0.02 to 0.02)	18.2 (15.3 to 21.2)	-3.73 (-3.98 to -3.48)
Guatemala	0.35 (0.33 to 0.36)	22.0 (20.8 to 23.1)	4.05 (3.52 to 4.62)	70.4 (61.5 to 80.1)	3.98 (3.69 to 4.28)	0.04 (0.04 to 0.04)	1.18 (1.11 to 1.26)	0.09 (0.08 to 0.11)	1.31 (1.10 to 1.52)	0.69 (0.42 to 1.00)	0.31 (0.29 to 0.33)	20.8 (19.6 to 21.9)	3.96 (3.44 to 4.51)	69.1 (60.4 to 78.7)	4.09 (3.80 to 4.40)
Guinea	0.47 (0.34 to 0.61)	31.1 (22.1 to 40.4)	1.13 (0.87 to 1.44)	45.2 (34.9 to 57.8)	1.22 (1.20 to 1.24)	0.02 (0.01 to 0.03)	0.84 (0.50 to 1.27)	0.03 (0.02 to 0.05)	0.64 (0.40 to 0.93)	-0.85 (-0.89 to -0.81)	0.45 (0.32 to 0.58)	30.3 (21.5 to 39.2)	1.09 (0.84 to 1.41)	44.6 (34.2 to 56.8)	1.26 (1.24 to 1.28)
Guinea-Bissau	0.07 (0.05 to 0.09)	35.9 (28.0 to 47.4)	0.18 (0.13 to 0.23)	55.2 (42.2 to 70.6)	1.42 (1.38 to 1.45)	0.004 (0.003 to 0.006)	1.14 (0.77 to 1.85)	0.007 (0.004 to 0.01)	0.83 (0.55 to 1.43)	-1.03 (-1.09 to -0.98)	0.06 (0.05 to 0.08)	34.8 (27.0 to 46.1)	0.17 (0.13 to 0.22)	54.4 (41.6 to 69.4)	1.47 (1.44 to 1.50)
Guyana	0.17 (0.15 to 0.19)	89.2 (78.9 to 99.1)	0.28 (0.22 to 0.35)	87.2 (69.0 to 108)	-0.16 (-0.48 to 0.11)	0.007 (0.006 to 0.009)	2.59 (2.25 to 2.95)	0.006 (0.004 to 0.008)	1.67 (1.21 to 2.18)	-1.22 (-1.38 to -1.07)	0.16 (0.14 to 0.18)	86.7 (76.6 to 96.2)	0.27 (0.21 to 0.34)	85.5 (67.8 to 105)	-0.13 (-0.45 to 0.14)
Haiti	1.84 (1.50 to 2.29)	117 (95.9 to 145)	3.76 (2.78 to 5.09)	112 (82.8 to 150)	-0.10 (-0.14 to -0.06)	0.15 (0.10 to 0.29)	6.23 (4.08 to 12.6)	0.25 (0.14 to 0.42)	4.68 (2.62 to 7.96)	-0.87 (-0.91 to -0.82)	1.70 (1.38 to 2.11)	110 (90.0 to 137)	3.52 (2.60 to 4.73)	107 (80.3 to 142)	-0.07 (-0.10 to -0.04)
Honduras	0.16 (0.14 to 0.19)	15.9 (13.1 to 19.1)	1.07 (0.78 to 1.43)	33.7 (24.4 to 44.9)	2.72 (2.42 to 3.01)	0.007 (0.005 to 0.008)	0.35 (0.27 to 0.45)	0.01 (0.008 to 0.02)	0.31 (0.19 to 0.47)	-0.33 (-0.42 to -0.24)	0.16 (0.13 to 0.18)	15.6 (12.8 to 18.7)	1.05 (0.77 to 1.41)	33.4 (24.2 to 44.5)	2.77 (2.46 to 3.06)
Hungary	1.22 (1.12 to 1.34)	13.7 (12.7 to 14.8)	1.50 (1.29 to 1.66)	11.2 (9.76 to 12.4)	-0.30 (-0.62 to -0.03)	0.09 (0.08 to 0.09)	1.10 (1.01 to 1.19)	0.03 (0.02 to 0.03)	0.30 (0.26 to 0.35)	-4.10 (-4.26 to -3.95)	1.14 (1.04 to 1.24)	12.6 (11.6 to 13.6)	1.47 (1.27 to 1.64)	10.9 (9.48 to 12.1)	-0.09 (-0.41 to 0.18)
Iceland	0.009 (0.008 to 0.010)	4.95 (4.42 to 5.43)	0.01 (0.008 to 0.01)	2.83 (2.28 to 3.20)	-1.94 (-2.06 to -1.84)	7e-04 (6e-04 to 8e-04)	0.44 (0.40 to 0.48)	4e-04 (4e-04 to 5e-04)	0.15 (0.13 to 0.17)	-3.52 (-3.63 to -3.42)	0.008 (0.007 to 0.009)	4.51 (4.00 to 4.95)	0.01 (0.008 to 0.01)	2.68 (2.15 to 3.03)	-1.81 (-1.95 to -1.70)
India	42.4 (35.4 to 48.9)	21.4 (17.6 to 24.9)	166 (144 to 192)	29.4 (25.4 to 34.0)	1.03 (0.83 to 1.27)	3.22 (2.42 to 5.01)	1.02 (0.70 to 1.65)	5.23 (3.90 to 6.91)	0.78 (0.58 to 1.04)	-0.72 (-0.82 to -0.58)	39.2 (32.4 to 44.9)	20.4 (16.7 to 23.6)	161 (139 to 186)	28.6 (24.7 to 33.0)	1.10 (0.90 to 1.34)
Indonesia	10.9 (8.67 to 13.8)	22.3 (17.2 to 28.5)	32.6 (25.3 to 42.1)	29.7 (23.5 to 37.3)	0.94 (0.92 to 0.96)	0.72 (0.53 to 1.17)	0.96 (0.69 to 1.54)	1.00 (0.68 to 1.58)	0.70 (0.48 to 1.10)	-0.97 (-1.00 to -0.95)	10.2 (8.02 to 12.8)	21.4 (16.5 to 27.3)	31.6 (24.5 to 40.8)	29.0 (23.0 to 36.2)	1.01 (0.98 to 1.03)
Iran (Islamic Republic of)	1.56 (1.34 to 1.82)	15.9 (13.5 to 18.8)	7.70 (6.73 to 8.55)	22.8 (19.8 to 25.4)	1.15 (1.07 to 1.21)	0.08 (0.06 to 0.11)	0.33 (0.27 to 0.44)	0.09 (0.06 to 0.12)	0.21 (0.14 to 0.28)	-1.32 (-1.48 to -1.21)	1.48 (1.26 to 1.73)	15.5 (13.2 to 18.4)	7.61 (6.65 to 8.45)	22.6 (19.5 to 25.1)	1.18 (1.10 to 1.24)
Iraq	1.52 (1.24 to 1.84)	37.6 (30.6 to 45.9)	4.59 (3.53 to 5.75)	44.4 (34.6 to 54.4)	0.57 (0.47 to 0.65)	0.10 (0.07 to 0.15)	1.27 (0.84 to 2.02)	0.13 (0.09 to 0.19)	0.70 (0.46 to 1.03)	-1.92 (-1.95 to -1.88)	1.41 (1.15 to 1.72)	36.3 (29.5 to 44.2)	4.46 (3.43 to 5.57)	43.7 (34.0 to 53.7)	0.64 (0.54 to 0.73)
Ireland	0.24 (0.22 to 0.26)	10.1 (9.11 to 10.9)	0.18 (0.14 to 0.21)	3.64 (2.94 to 4.18)	-3.07 (-3.26 to -2.90)	0.01 (0.01 to 0.01)	0.55 (0.50 to 0.59)	0.006 (0.005 to 0.006)	0.14 (0.13 to 0.16)	-4.30 (-4.39 to -4.23)	0.23 (0.21 to 0.25)	9.50 (8.59 to 10.4)	0.17 (0.14 to 0.20)	3.50 (2.82 to 4.02)	-3.02 (-3.22 to -2.83)
Israel	0.54 (0.49 to 0.58)	21.5 (19.3 to 23.1)	1.27 (1.02 to 1.43)	15.6 (12.7 to 17.5)	-1.10 (-1.32 to -0.87)	0.01 (0.01 to 0.02)	0.55 (0.51 to 0.59)	0.009 (0.008 to 0.010)	0.15 (0.13 to 0.16)	-4.16 (-4.24 to -4.08)	0.53 (0.48 to 0.57)	20.9 (18.8 to 22.6)	1.26 (1.01 to 1.42)	15.5 (12.6 to 17.3)	-1.05 (-1.27 to -0.82)
Italy	11.5 (10.2 to 12.2)	20.3 (18.1 to 21.5)	10.0 (7.68 to 11.3)	8.31 (6.61 to 9.26)	-2.87 (-3.00 to -2.70)	0.13 (0.12 to 0.13)	0.32 (0.30 to 0.33)	0.06 (0.05 to 0.07)	0.10 (0.09 to 0.10)	-3.84 (-3.93 to -3.75)	11.4 (10.0 to 12.1)	20.0 (17.8 to 21.2)	9.94 (7.62 to 11.2)	8.21 (6.53 to 9.16)	-2.85 (-2.98 to -2.68)
Jamaica	0.78 (0.74 to 0.83)	78.2 (73.8 to 82.6)	1.34 (1.08 to 1.66)	75.7 (61.0 to 93.8)	0.04 (-0.28 to 0.31)	0.02 (0.02 to 0.02)	1.58 (1.47 to 1.69)	0.02 (0.01 to 0.02)	1.15 (0.85 to 1.50)	-1.13 (-1.32 to -0.92)	0.77 (0.72 to 0.81)	76.7 (72.2 to 81.0)	1.32 (1.07 to 1.64)	74.5 (60.1 to 92.4)	0.06 (-0.27 to 0.33)
Japan	6.24 (5.55 to 6.63)	6.44 (5.70 to 6.84)	4.88 (3.49 to 5.71)	1.47 (1.14 to 1.66)	-4.81 (-4.96 to -4.66)	0.17 (0.16 to 0.17)	0.21 (0.20 to 0.22)	0.06 (0.05 to 0.07)	0.05 (0.05 to 0.05)	-4.58 (-4.71 to -4.45)	6.07 (5.38 to 6.45)	6.22 (5.50 to 6.63)	4.82 (3.44 to 5.64)	1.42 (1.10 to 1.60)	-4.80 (-4.96 to -4.66)
Jordan	0.38 (0.32 to 0.45)	70.4 (58.4 to 83.4)	1.10 (0.89 to 1.36)	42.6 (34.4 to 52.3)	-1.77 (-1.99 to -1.52)	0.01 (0.010 to 0.02)	0.79 (0.60 to 1.05)	0.01 (0.01 to 0.02)	0.26 (0.21 to 0.35)	-3.51 (-3.57 to -3.44)	0.37 (0.30 to 0.44)	69.6 (57.7 to 82.6)	1.09 (0.88 to 1.34)	42.3 (34.2 to 51.9)	-1.75 (-1.98 to -1.50)
Kazakhstan	0.64 (0.56 to 0.74)	8.20 (7.09 to 9.41)	0.95 (0.79 to 1.13)	9.45 (7.81 to 11.2)	0.49 (0.28 to 0.69)	0.08 (0.07 to 0.09)	1.03 (0.91 to 1.16)	0.07 (0.06 to 0.08)	0.65 (0.54 to 0.78)	-1.71 (-1.88 to -1.56)	0.56 (0.48 to 0.65)	7.18 (6.10 to 8.34)	0.89 (0.73 to 1.05)	8.80 (7.23 to 10.4)	0.70 (0.51 to 0.87)
Kenya	0.88 (0.73 to 1.05)	24.0 (19.8 to 28.6)	2.99 (2.24 to 3.84)	29.2 (22.0 to 37.2)	0.64 (0.61 to 0.67)	0.03 (0.02 to 0.05)	0.38 (0.25 to 0.59)	0.07 (0.05 to 0.11)	0.36 (0.23 to 0.58)	-0.14 (-0.18 to -0.11)	0.85 (0.70 to 1.01)	23.6 (19.5 to 28.1)	2.91 (2.19 to 3.72)	28.8 (21.7 to 36.8)	0.65 (0.62 to 0.68)
Kiribati	0.03 (0.02 to 0.03)	137 (108 to 168)	0.07 (0.06 to 0.09)	196 (153 to 243)	1.19 (1.17 to 1.21)	3e-04 (2e-04 to 4e-04)	0.80 (0.49 to 1.07)	5e-04 (3e-04 to 8e-04)	0.86 (0.46 to 1.30)	0.22 (0.19 to 0.25)	0.03 (0.02 to 0.03)	136 (107 to 167)	0.07 (0.06 to 0.09)	195 (153 to 242)	1.19 (1.17 to 1.22)
Kuwait	0.05 (0.05 to 0.06)	26.2 (23.1 to 28.6)	0.19 (0.16 to 0.22)	20.6 (16.7 to 23.9)	-1.00 (-1.55 to -0.31)	0.004 (0.003 to 0.004)	0.74 (0.66 to 0.82)	0.004 (0.004 to 0.005)	0.23 (0.20 to 0.26)	-3.82 (-4.26 to -3.35)	0.05 (0.04 to 0.05)	25.5 (22.4 to 27.8)	0.19 (0.15 to 0.21)	20.4 (16.5 to 23.7)	-0.94 (-1.48 to -0.25)
Kyrgyzstan	0.10 (0.09 to 0.11)	5.71 (5.13 to 6.33)	0.22 (0.18 to 0.26)	8.08 (6.78 to 9.48)	1.08 (0.78 to 1.31)	0.01 (0.010 to 0.01)	0.54 (0.48 to 0.60)	0.01 (0.01 to 0.02)	0.43 (0.36 to 0.51)	-1.03 (-1.48 to -0.70)	0.09 (0.08 to 0.10)	5.18 (4.63 to 5.76)	0.20 (0.17 to 0.24)	7.65 (6.43 to 9.00)	1.24 (0.92 to 1.48)
Lao People's Democratic Republic	0.44 (0.34 to 0.60)	42.2 (32.6 to 55.8)	0.94 (0.71 to 1.19)	42.6 (32.3 to 53.3)	0.01 (-0.01 to 0.02)	0.03 (0.02 to 0.06)	1.86 (1.16 to 3.83)	0.04 (0.02 to 0.06)	1.17 (0.76 to 1.82)	-1.50 (-1.52 to -1.47)	0.42 (0.32 to 0.54)	40.4 (31.1 to 52.5)	0.90 (0.68 to 1.15)	41.4 (31.5 to 51.9)	0.07 (0.05 to 0.08)
Latvia	0.14 (0.13 to 0.15)	6.34 (5.87 to 6.81)	0.35 (0.31 to 0.39)	12.7 (11.1 to 14.1)	2.37 (1.74 to 2.79)	0.01 (0.01 to 0.01)	0.83 (0.77 to 0.89)	0.006 (0.005 to 0.007)	0.56 (0.47 to 0.65)	-1.39 (-2.07 to -0.87)	0.13 (0.12 to 0.14)	5.52 (5.07 to 5.96)	0.35 (0.30 to 0.39)	12.1 (10.6 to 13.5)	2.69 (2.02 to 3.18)
Lebanon	0.33 (0.27 to 0.40)	31.8 (26.1 to 39.1)	0.74 (0.59 to 0.90)	21.1 (16.9 to 25.5)	-1.31 (-1.42 to -1.23)	0.01 (0.008 to 0.02)	0.99 (0.60 to 1.62)	0.01 (0.007 to 0.02)	0.38 (0.22 to 0.58)	-3.05 (-3.11 to -2.99)	0.31 (0.26 to 0.39)	30.8 (25.5 to 38.0)	0.72 (0.58 to 0.88)	20.7 (16.7 to 25.0)	-1.27 (-1.38 to -1.19)
Lesotho	0.21 (0.16 to 0.28)	40.2 (31.1 to 54.6)	0.59 (0.41 to 0.79)	94.8 (67.9 to 125)	2.84 (2.74 to 2.93)	0.003 (0.002 to 0.004)	0.32 (0.19 to 0.50)	0.005 (0.002 to 0.007)	0.49 (0.23 to 0.80)	1.45 (1.33 to 1.57)	0.20 (0.16 to 0.28)	39.9 (30.8 to 54.3)	0.58 (0.41 to 0.78)	94.3 (67.6 to 125)	2.85 (2.75 to 2.94)
Liberia	0.15 (0.12 to 0.18)	30.9 (25.1 to 37.5)	0.41 (0.30 to 0.54)	46.6 (35.0 to 61.5)	1.33 (1.29 to 1.38)	0.007 (0.005 to 0.01)	0.75 (0.47 to 1.07)	0.01 (0.007 to 0.02)	0.54 (0.34 to 0.84)	-1.12 (-1.20 to -1.06)	0.14 (0.11 to 0.17)	30.1 (24.4 to 36.6)	0.39 (0.29 to 0.53)	46.1 (34.5 to 60.8)	1.38 (1.34 to 1.42)
Libya	0.12 (0.10 to 0.15)	14.2 (11.1 to 17.4)	0.61 (0.47 to 0.83)	26.2 (19.8 to 35.1)	2.09 (1.96 to 2.20)	0.007 (0.005 to 0.01)	0.43 (0.28 to 0.70)	0.02 (0.01 to 0.03)	0.57 (0.33 to 0.95)	0.98 (0.81 to 1.14)	0.11 (0.09 to 0.14)	13.8 (10.8 to 17.0)	0.60 (0.45 to 0.80)	25.6 (19.4 to 34.5)	2.11 (1.97 to 2.23)
Lithuania	0.11 (0.10 to 0.12)	3.91 (3.65 to 4.18)	0.31 (0.27 to 0.35)	7.94 (6.93 to 8.91)	2.22 (1.92 to 2.46)	0.02 (0.02 to 0.02)	0.74 (0.66 to 0.83)	0.01 (0.009 to 0.01)	0.55 (0.47 to 0.63)	-1.02 (-1.36 to -0.71)	0.09 (0.08 to 0.10)	3.17 (2.93 to 3.40)	0.30 (0.27 to 0.34)	7.39 (6.44 to 8.30)	2.67 (2.39 to 2.94)
Luxembourg	0.04 (0.03 to 0.04)	11.2 (10.3 to 12.1)	0.04 (0.03 to 0.04)	4.89 (4.11 to 5.57)	-2.66 (-2.97 to -2.43)	0.002 (0.002 to 0.002)	0.68 (0.62 to 0.73)	0.001 (9e-04 to 0.001)	0.18 (0.16 to 0.20)	-4.26 (-4.33 to -4.20)	0.04 (0.03 to 0.04)	10.5 (9.64 to 11.4)	0.03 (0.03 to 0.04)	4.71 (3.94 to 5.37)	-2.58 (-2.90 to -2.35)
Madagascar	0.77 (0.64 to 0.91)	33.8 (28.1 to 39.8)	1.70 (1.24 to 2.29)	35.0 (25.7 to 46.1)	0.10 (0.05 to 0.16)	0.04 (0.03 to 0.05)	0.76 (0.54 to 1.04)	0.08 (0.06 to 0.11)	0.62 (0.44 to 0.89)	-0.63 (-0.70 to -0.58)	0.73 (0.60 to 0.87)	33.0 (27.4 to 39.0)	1.62 (1.18 to 2.19)	34.4 (25.3 to 45.3)	0.12 (0.07 to 0.17)
Malawi	0.66 (0.52 to 0.81)	36.5 (28.7 to 45.1)	1.26 (0.97 to 1.62)	35.6 (27.7 to 44.9)	-0.07 (-0.11 to -0.02)	0.04 (0.03 to 0.05)	0.77 (0.54 to 1.09)	0.05 (0.03 to 0.07)	0.55 (0.35 to 0.81)	-1.11 (-1.16 to -1.06)	0.62 (0.49 to 0.77)	35.7 (28.0 to 44.2)	1.22 (0.93 to 1.56)	35.0 (27.3 to 44.3)	-0.05 (-0.09 to 0.00)
Malaysia	1.40 (1.23 to 1.60)	31.1 (26.9 to 35.5)	2.77 (2.39 to 3.17)	21.5 (18.6 to 24.7)	-1.26 (-1.49 to -1.08)	0.07 (0.05 to 0.09)	1.11 (0.85 to 1.46)	0.10 (0.07 to 0.13)	0.63 (0.43 to 0.83)	-1.79 (-1.94 to -1.63)	1.34 (1.17 to 1.53)	30.0 (25.9 to 34.4)	2.67 (2.31 to 3.07)	20.9 (18.0 to 24.0)	-1.24 (-1.47 to -1.06)
Maldives	0.01 (0.01 to 0.02)	43.3 (36.9 to 52.4)	0.02 (0.02 to 0.03)	18.3 (14.8 to 22.5)	-2.86 (-3.02 to -2.69)	0.001 (7e-04 to 0.002)	1.93 (1.21 to 4.26)	7e-04 (5e-04 to 0.001)	0.40 (0.29 to 0.55)	-5.02 (-5.12 to -4.90)	0.01 (0.01 to 0.02)	41.4 (35.4 to 49.4)	0.02 (0.02 to 0.03)	17.9 (14.5 to 22.0)	-2.79 (-2.95 to -2.62)
Mali	0.62 (0.49 to 0.77)	35.9 (29.2 to 44.5)	1.79 (1.39 to 2.25)	49.5 (38.6 to 61.7)	1.06 (1.03 to 1.09)	0.03 (0.02 to 0.05)	0.99 (0.69 to 1.51)	0.06 (0.04 to 0.09)	0.67 (0.46 to 1.04)	-1.25 (-1.32 to -1.19)	0.58 (0.47 to 0.73)	34.9 (28.3 to 43.4)	1.73 (1.34 to 2.18)	48.8 (38.0 to 60.9)	1.11 (1.08 to 1.13)
Malta	0.06 (0.06 to 0.07)	27.0 (24.4 to 29.3)	0.09 (0.07 to 0.10)	13.0 (10.8 to 14.9)	-2.49 (-2.72 to -2.27)	0.002 (0.002 to 0.002)	0.87 (0.80 to 0.95)	0.001 (0.001 to 0.001)	0.28 (0.25 to 0.32)	-3.67 (-3.83 to -3.54)	0.06 (0.06 to 0.07)	26.1 (23.5 to 28.4)	0.08 (0.07 to 0.10)	12.7 (10.5 to 14.6)	-2.45 (-2.70 to -2.23)
Marshall Islands	0.009 (0.007 to 0.01)	122 (97.1 to 157)	0.04 (0.02 to 0.05)	238 (141 to 330)	2.18 (2.16 to 2.20)	1e-04 (1e-04 to 2e-04)	0.73 (0.50 to 1.12)	3e-04 (1e-04 to 4e-04)	0.96 (0.49 to 1.61)	0.90 (0.87 to 0.91)	0.009 (0.007 to 0.01)	122 (96.5 to 156)	0.04 (0.02 to 0.05)	237 (140 to 328)	2.19 (2.16 to 2.21)
Mauritania	0.15 (0.11 to 0.18)	30.5 (23.4 to 39.1)	0.36 (0.26 to 0.49)	38.6 (28.3 to 52.1)	0.78 (0.73 to 0.83)	0.005 (0.004 to 0.008)	0.68 (0.46 to 1.02)	0.006 (0.004 to 0.01)	0.37 (0.23 to 0.76)	-1.96 (-2.05 to -1.88)	0.14 (0.11 to 0.18)	29.8 (22.8 to 38.4)	0.35 (0.26 to 0.48)	38.2 (28.0 to 51.6)	0.82 (0.77 to 0.88)
Mauritius	0.19 (0.18 to 0.21)	49.0 (45.2 to 52.4)	0.93 (0.85 to 0.98)	92.3 (84.2 to 98.0)	1.91 (1.63 to 2.28)	0.01 (0.01 to 0.02)	3.12 (2.67 to 3.69)	0.02 (0.02 to 0.02)	2.40 (2.16 to 2.67)	-0.86 (-1.03 to -0.67)	0.18 (0.16 to 0.19)	45.9 (42.4 to 49.3)	0.90 (0.83 to 0.96)	89.9 (81.8 to 95.5)	2.04 (1.74 to 2.42)
Mexico	16.3 (15.8 to 16.7)	83.1 (79.8 to 85.4)	42.3 (36.1 to 48.3)	64.9 (55.7 to 73.9)	-0.68 (-0.81 to -0.54)	0.38 (0.37 to 0.40)	1.33 (1.29 to 1.37)	0.58 (0.49 to 0.67)	0.85 (0.72 to 0.98)	-1.36 (-1.49 to -1.20)	15.9 (15.4 to 16.3)	81.8 (78.5 to 84.1)	41.7 (35.7 to 47.6)	64.0 (55.0 to 72.9)	-0.67 (-0.80 to -0.53)
Micronesia (Federated States of)	0.02 (0.02 to 0.03)	86.3 (67.8 to 113)	0.04 (0.03 to 0.06)	127 (99.5 to 165)	1.30 (1.27 to 1.34)	3e-04 (2e-04 to 5e-04)	0.66 (0.41 to 1.12)	3e-04 (2e-04 to 4e-04)	0.53 (0.35 to 0.84)	-0.71 (-0.73 to -0.70)	0.02 (0.02 to 0.03)	85.6 (67.2 to 112)	0.04 (0.03 to 0.06)	127 (99.1 to 164)	1.31 (1.28 to 1.35)
Monaco	0.002 (0.001 to 0.002)	3.07 (2.21 to 4.06)	0.002 (0.001 to 0.002)	2.69 (1.96 to 3.46)	-0.40 (-0.42 to -0.37)	4e-05 (2e-05 to 5e-05)	0.12 (0.07 to 0.16)	3e-05 (2e-05 to 4e-05)	0.08 (0.06 to 0.11)	-1.29 (-1.31 to -1.27)	0.002 (0.001 to 0.002)	2.95 (2.11 to 3.92)	0.002 (0.001 to 0.002)	2.62 (1.90 to 3.36)	-0.36 (-0.39 to -0.33)
Mongolia	0.04 (0.03 to 0.04)	5.89 (4.74 to 7.23)	0.09 (0.08 to 0.11)	7.52 (6.16 to 8.89)	0.76 (0.57 to 0.96)	0.009 (0.005 to 0.01)	1.14 (0.65 to 1.61)	0.009 (0.006 to 0.01)	0.55 (0.38 to 0.73)	-2.31 (-2.39 to -2.22)	0.03 (0.02 to 0.03)	4.75 (3.68 to 6.00)	0.09 (0.07 to 0.10)	6.96 (5.71 to 8.26)	1.19 (0.96 to 1.41)
Montenegro	0.04 (0.04 to 0.05)	12.4 (10.4 to 14.4)	0.10 (0.08 to 0.12)	18.1 (15.4 to 21.3)	1.38 (1.18 to 1.50)	0.006 (0.004 to 0.008)	1.74 (1.24 to 2.40)	0.008 (0.005 to 0.01)	1.54 (1.09 to 2.15)	-0.35 (-0.45 to -0.22)	0.04 (0.03 to 0.04)	10.6 (8.95 to 12.5)	0.09 (0.08 to 0.11)	16.6 (14.0 to 19.5)	1.62 (1.47 to 1.73)
Morocco	0.94 (0.77 to 1.15)	13.8 (11.2 to 16.8)	4.44 (3.18 to 5.61)	27.2 (19.6 to 34.3)	2.21 (2.18 to 2.24)	0.08 (0.05 to 0.14)	0.63 (0.40 to 1.12)	0.10 (0.07 to 0.14)	0.54 (0.37 to 0.81)	-0.54 (-0.57 to -0.50)	0.86 (0.69 to 1.08)	13.1 (10.6 to 16.2)	4.34 (3.09 to 5.52)	26.7 (19.0 to 33.7)	2.30 (2.27 to 2.33)
Mozambique	0.87 (0.72 to 1.07)	31.7 (26.4 to 38.5)	1.93 (1.35 to 2.55)	36.6 (26.3 to 48.4)	0.49 (0.45 to 0.53)	0.05 (0.03 to 0.07)	0.77 (0.50 to 1.06)	0.09 (0.06 to 0.13)	0.65 (0.41 to 0.94)	-0.49 (-0.54 to -0.45)	0.82 (0.68 to 1.01)	30.9 (25.8 to 37.7)	1.84 (1.28 to 2.42)	36.0 (25.8 to 47.4)	0.53 (0.49 to 0.56)
Myanmar	7.32 (5.57 to 9.67)	61.4 (47.3 to 80.6)	14.4 (11.6 to 18.1)	56.0 (45.1 to 70.4)	-0.29 (-0.31 to -0.27)	0.50 (0.31 to 1.04)	3.04 (1.84 to 6.26)	0.46 (0.29 to 0.73)	1.53 (0.99 to 2.46)	-2.19 (-2.21 to -2.17)	6.82 (5.19 to 8.94)	58.4 (45.0 to 75.9)	14.0 (11.2 to 17.5)	54.4 (43.6 to 68.7)	-0.22 (-0.24 to -0.19)
Namibia	0.20 (0.16 to 0.24)	61.7 (51.1 to 74.4)	0.48 (0.34 to 0.64)	68.5 (49.6 to 88.4)	0.36 (0.30 to 0.40)	0.003 (0.002 to 0.004)	0.39 (0.23 to 0.54)	0.003 (0.002 to 0.006)	0.28 (0.15 to 0.44)	-1.10 (-1.17 to -1.03)	0.20 (0.16 to 0.24)	61.3 (50.9 to 74.0)	0.48 (0.34 to 0.63)	68.2 (49.3 to 88.1)	0.37 (0.31 to 0.41)
Nauru	0.002 (0.002 to 0.003)	115 (93.7 to 143)	0.004 (0.003 to 0.006)	151 (118 to 193)	0.87 (0.83 to 0.90)	3e-05 (2e-05 to 5e-05)	0.69 (0.47 to 1.13)	3e-05 (2e-05 to 6e-05)	0.62 (0.37 to 1.05)	-0.36 (-0.39 to -0.34)	0.002 (0.002 to 0.003)	114 (93.0 to 142)	0.004 (0.003 to 0.006)	150 (118 to 192)	0.87 (0.83 to 0.91)
Nepal	0.87 (0.68 to 1.08)	21.4 (17.0 to 26.7)	3.28 (2.50 to 4.32)	30.5 (23.4 to 40.1)	1.17 (1.13 to 1.19)	0.07 (0.05 to 0.12)	1.10 (0.70 to 1.90)	0.13 (0.08 to 0.18)	0.89 (0.57 to 1.27)	-0.68 (-0.72 to -0.64)	0.79 (0.62 to 0.99)	20.3 (16.0 to 25.4)	3.15 (2.38 to 4.15)	29.6 (22.7 to 39.0)	1.24 (1.21 to 1.27)

Location	DM, Number in 1990 (000s)	DM, ASR in 1990 (per 100,000)	DM, Number in 2021 (000s)	DM, ASR in 2021 (per 100,000)	DM, AAPC (95%CI)	T1D, Number in 1990 (000s)	T1D, ASR in 1990 (per 100,000)	T1D, Number in 2021 (000s)	T1D, ASR in 2021 (per 100,000)	T1D, AAPC (95%CI)	T2D, Number in 1990 (000s)	T2D, ASR in 1990 (per 100,000)	T2D, Number in 2021 (000s)	T2D, ASR in 2021 (per 100,000)	T2D, AAPC (95%CI)
Netherlands	2.43 (2.11 to 2.64)	18.8 (16.5 to 20.3)	1.63 (1.33 to 1.85)	6.74 (5.58 to 7.56)	-3.28 (-3.40 to -3.12)	0.13 (0.12 to 0.14)	1.15 (1.06 to 1.24)	0.06 (0.05 to 0.07)	0.34 (0.31 to 0.37)	-3.93 (-4.00 to -3.85)	2.31 (1.99 to 2.51)	17.6 (15.4 to 19.1)	1.57 (1.27 to 1.78)	6.40 (5.27 to 7.20)	-3.24 (-3.37 to -3.07)
New Zealand	0.22 (0.19 to 0.23)	9.72 (8.79 to 10.6)	0.29 (0.24 to 0.32)	5.78 (4.94 to 6.39)	-1.53 (-1.79 to -1.29)	0.02 (0.01 to 0.02)	0.73 (0.67 to 0.79)	0.004 (0.004 to 0.004)	0.11 (0.10 to 0.12)	-5.90 (-6.35 to -5.49)	0.20 (0.18 to 0.22)	8.99 (8.10 to 9.80)	0.28 (0.24 to 0.32)	5.67 (4.83 to 6.27)	-1.32 (-1.62 to -1.04)
Nicaragua	0.21 (0.19 to 0.24)	26.2 (23.1 to 29.2)	0.82 (0.67 to 0.99)	31.6 (25.9 to 38.4)	0.54 (0.31 to 0.75)	0.01 (0.008 to 0.01)	0.65 (0.50 to 0.91)	0.01 (0.01 to 0.02)	0.45 (0.32 to 0.59)	-1.29 (-1.48 to -1.11)	0.20 (0.18 to 0.22)	25.5 (22.4 to 28.5)	0.80 (0.66 to 0.98)	31.1 (25.5 to 37.8)	0.57 (0.34 to 0.78)
Niger	0.23 (0.16 to 0.31)	19.2 (13.3 to 26.5)	0.99 (0.69 to 1.39)	28.0 (19.8 to 38.5)	1.23 (1.20 to 1.25)	0.02 (0.01 to 0.04)	0.70 (0.40 to 1.10)	0.05 (0.03 to 0.08)	0.50 (0.28 to 0.79)	-1.05 (-1.09 to -0.99)	0.20 (0.14 to 0.28)	18.5 (12.9 to 25.7)	0.94 (0.66 to 1.34)	27.5 (19.4 to 37.8)	1.29 (1.27 to 1.32)
Nigeria	6.13 (4.75 to 7.72)	30.9 (24.0 to 38.6)	13.3 (9.75 to 18.1)	33.5 (25.5 to 44.4)	0.24 (0.22 to 0.26)	0.23 (0.14 to 0.33)	0.71 (0.42 to 1.01)	0.37 (0.21 to 0.56)	0.40 (0.23 to 0.59)	-1.90 (-1.94 to -1.86)	5.90 (4.55 to 7.42)	30.2 (23.5 to 37.6)	12.9 (9.41 to 17.6)	33.1 (25.2 to 43.9)	0.28 (0.26 to 0.30)
Niue	0.001 (9e-04 to 0.001)	80.2 (63.2 to 99.5)	0.002 (0.001 to 0.002)	128 (91.5 to 160)	1.53 (1.48 to 1.57)	5e-06 (3e-06 to 7e-06)	0.43 (0.29 to 0.62)	7e-06 (5e-06 to 9e-06)	0.91 (0.63 to 1.20)	2.49 (1.75 to 3.05)	0.001 (9e-04 to 0.001)	79.7 (62.9 to 98.9)	0.002 (0.001 to 0.002)	127 (90.8 to 159)	1.52 (1.48 to 1.56)
North Macedonia	0.23 (0.19 to 0.27)	25.5 (21.2 to 29.9)	0.57 (0.45 to 0.69)	35.7 (28.8 to 43.6)	1.14 (1.07 to 1.21)	0.03 (0.02 to 0.04)	3.11 (2.28 to 3.97)	0.03 (0.02 to 0.04)	1.73 (1.15 to 2.48)	-1.83 (-1.92 to -1.74)	0.20 (0.17 to 0.24)	22.4 (18.6 to 26.4)	0.54 (0.43 to 0.66)	33.9 (27.5 to 41.5)	1.40 (1.33 to 1.48)
Northern Mariana Islands	0.004 (0.003 to 0.005)	69.6 (54.8 to 88.5)	0.01 (0.01 to 0.01)	58.0 (49.5 to 65.0)	-0.63 (-0.92 to -0.41)	3e-05 (2e-05 to 4e-05)	0.13 (0.08 to 0.19)	2e-05 (1e-05 to 2e-05)	0.08 (0.06 to 0.11)	-1.43 (-2.44 to -0.48)	0.004 (0.003 to 0.005)	69.5 (54.7 to 88.4)	0.01 (0.01 to 0.01)	57.9 (49.5 to 64.9)	-0.63 (-0.91 to -0.41)
Norway	0.28 (0.25 to 0.30)	6.21 (5.53 to 6.60)	0.30 (0.24 to 0.33)	4.21 (3.50 to 4.61)	-1.52 (-1.81 to -1.27)	0.02 (0.02 to 0.02)	0.58 (0.56 to 0.60)	0.008 (0.007 to 0.009)	0.20 (0.19 to 0.21)	-3.65 (-3.78 to -3.53)	0.27 (0.23 to 0.29)	5.63 (4.97 to 6.00)	0.29 (0.23 to 0.32)	4.01 (3.31 to 4.40)	-1.36 (-1.67 to -1.09)
Oman	0.12 (0.09 to 0.16)	42.4 (32.4 to 55.5)	0.27 (0.22 to 0.33)	38.2 (31.0 to 46.5)	-0.33 (-0.46 to -0.20)	0.01 (0.007 to 0.01)	2.08 (1.43 to 3.01)	0.01 (0.01 to 0.02)	0.95 (0.72 to 1.25)	-2.39 (-2.49 to -2.27)	0.11 (0.08 to 0.15)	40.4 (30.5 to 52.9)	0.26 (0.21 to 0.32)	37.3 (30.2 to 45.4)	-0.26 (-0.38 to -0.12)
Pakistan	6.99 (5.57 to 8.47)	31.4 (25.0 to 38.5)	27.0 (20.0 to 35.8)	55.0 (41.2 to 71.7)	1.83 (1.81 to 1.85)	0.45 (0.31 to 0.59)	1.30 (0.85 to 1.74)	1.41 (0.91 to 1.98)	1.61 (1.04 to 2.30)	0.74 (0.71 to 0.78)	6.53 (5.09 to 7.99)	30.2 (23.6 to 36.9)	25.6 (19.0 to 33.9)	53.4 (39.9 to 69.5)	1.87 (1.84 to 1.89)
Palau	0.004 (0.003 to 0.005)	82.2 (64.7 to 102)	0.01 (0.008 to 0.01)	128 (102 to 157)	1.50 (1.43 to 1.55)	3e-05 (2e-05 to 4e-05)	0.36 (0.25 to 0.52)	3e-05 (2e-05 to 4e-05)	0.37 (0.27 to 0.51)	0.15 (0.11 to 0.19)	0.004 (0.003 to 0.005)	81.9 (64.4 to 101)	0.01 (0.008 to 0.01)	128 (102 to 156)	1.50 (1.44 to 1.56)
Palestine	0.21 (0.16 to 0.28)	50.2 (39.0 to 64.9)	0.56 (0.47 to 0.66)	53.2 (45.0 to 62.9)	-0.00 (-0.13 to 0.13)	0.005 (0.003 to 0.007)	0.44 (0.31 to 0.63)	0.006 (0.004 to 0.007)	0.24 (0.18 to 0.30)	-1.92 (-2.00 to -1.82)	0.21 (0.16 to 0.27)	49.8 (38.6 to 64.3)	0.56 (0.47 to 0.66)	53.0 (44.7 to 62.6)	0.01 (-0.11 to 0.14)
Panama	0.18 (0.17 to 0.19)	25.1 (23.0 to 26.9)	0.67 (0.53 to 0.79)	28.0 (22.2 to 33.3)	0.32 (0.09 to 0.54)	0.005 (0.004 to 0.005)	0.47 (0.43 to 0.50)	0.010 (0.008 to 0.01)	0.43 (0.35 to 0.52)	-0.08 (-0.20 to 0.09)	0.18 (0.16 to 0.19)	24.6 (22.6 to 26.4)	0.66 (0.52 to 0.78)	27.5 (21.8 to 32.8)	0.33 (0.09 to 0.56)
Papua New Guinea	0.50 (0.37 to 0.67)	60.2 (45.6 to 80.0)	1.56 (1.22 to 2.01)	70.2 (54.7 to 88.6)	0.49 (0.44 to 0.55)	0.01 (0.007 to 0.02)	0.62 (0.34 to 1.03)	0.03 (0.02 to 0.05)	0.65 (0.41 to 0.94)	0.32 (0.20 to 0.47)	0.49 (0.36 to 0.65)	59.6 (45.0 to 79.1)	1.53 (1.19 to 1.97)	69.6 (54.2 to 87.8)	0.50 (0.44 to 0.55)
Paraguay	0.34 (0.28 to 0.40)	29.7 (24.3 to 35.4)	1.71 (1.31 to 2.15)	57.2 (43.7 to 71.9)	2.24 (2.09 to 2.38)	0.010 (0.008 to 0.01)	0.51 (0.39 to 0.69)	0.02 (0.01 to 0.02)	0.48 (0.31 to 0.64)	-0.11 (-0.24 to 0.03)	0.33 (0.27 to 0.39)	29.2 (23.8 to 34.8)	1.69 (1.30 to 2.14)	56.7 (43.4 to 71.4)	2.27 (2.11 to 2.41)
Peru	0.84 (0.70 to 0.99)	14.3 (11.9 to 16.9)	3.03 (2.31 to 4.00)	17.3 (13.2 to 22.8)	0.65 (0.29 to 0.95)	0.02 (0.02 to 0.03)	0.26 (0.19 to 0.33)	0.03 (0.02 to 0.05)	0.18 (0.10 to 0.26)	-1.16 (-1.71 to -0.84)	0.82 (0.68 to 0.97)	14.0 (11.7 to 16.6)	3.00 (2.29 to 3.95)	17.2 (13.1 to 22.6)	0.68 (0.31 to 0.98)
Philippines	3.86 (3.49 to 4.24)	30.4 (27.5 to 33.3)	15.9 (12.8 to 19.4)	38.9 (31.7 to 47.3)	0.86 (0.76 to 1.04)	0.19 (0.16 to 0.24)	0.78 (0.62 to 1.05)	0.39 (0.30 to 0.54)	0.75 (0.57 to 1.03)	-0.12 (-0.18 to -0.06)	3.67 (3.29 to 4.03)	29.7 (26.8 to 32.4)	15.5 (12.5 to 18.9)	38.1 (31.1 to 46.4)	0.88 (0.78 to 1.06)
Poland	3.82 (3.63 to 3.98)	14.3 (13.6 to 14.9)	5.37 (4.61 to 5.94)	10.9 (9.52 to 12.1)	-0.90 (-1.02 to -0.80)	0.42 (0.41 to 0.43)	1.65 (1.60 to 1.69)	0.10 (0.09 to 0.11)	0.30 (0.27 to 0.33)	-5.44 (-5.58 to -5.27)	3.40 (3.22 to 3.56)	12.6 (12.0 to 13.2)	5.26 (4.52 to 5.83)	10.6 (9.23 to 11.7)	-0.58 (-0.70 to -0.47)
Portugal	1.80 (1.65 to 1.94)	22.0 (20.1 to 23.6)	2.27 (1.78 to 2.58)	11.5 (9.22 to 12.9)	-2.20 (-2.39 to -2.03)	0.03 (0.03 to 0.03)	0.43 (0.41 to 0.46)	0.01 (0.009 to 0.01)	0.11 (0.10 to 0.12)	-4.41 (-4.48 to -4.35)	1.78 (1.63 to 1.91)	21.5 (19.7 to 23.1)	2.26 (1.78 to 2.57)	11.4 (9.12 to 12.8)	-2.17 (-2.36 to -2.00)
Puerto Rico	1.07 (1.00 to 1.14)	56.4 (52.4 to 60.0)	1.58 (1.29 to 1.86)	33.5 (27.7 to 39.3)	-1.77 (-2.13 to -1.50)	0.06 (0.05 to 0.06)	3.04 (2.81 to 3.26)	0.06 (0.05 to 0.07)	1.72 (1.41 to 2.05)	-1.72 (-1.89 to -1.54)	1.01 (0.94 to 1.08)	53.4 (49.5 to 56.8)	1.53 (1.25 to 1.80)	31.8 (26.2 to 37.3)	-1.76 (-2.17 to -1.48)
Qatar	0.02 (0.01 to 0.02)	73.0 (58.1 to 93.5)	0.13 (0.10 to 0.16)	75.8 (60.9 to 93.5)	0.16 (-0.19 to 0.49)	7e-04 (5e-04 to 0.001)	0.68 (0.44 to 1.01)	0.002 (0.001 to 0.002)	0.24 (0.17 to 0.32)	-3.40 (-3.56 to -3.24)	0.02 (0.01 to 0.02)	72.3 (57.5 to 92.7)	0.13 (0.10 to 0.16)	75.6 (60.7 to 93.2)	0.18 (-0.17 to 0.52)
Republic of Korea	2.82 (2.45 to 3.19)	17.9 (15.6 to 20.4)	5.19 (3.97 to 6.19)	9.09 (7.01 to 10.8)	-2.21 (-2.32 to -2.15)	0.14 (0.09 to 0.17)	0.67 (0.44 to 0.83)	0.04 (0.03 to 0.07)	0.11 (0.08 to 0.17)	-5.70 (-5.77 to -5.64)	2.68 (2.32 to 3.05)	17.3 (15.0 to 19.7)	5.15 (3.94 to 6.14)	8.98 (6.93 to 10.7)	-2.13 (-2.24 to -2.07)
Republic of Moldova	0.19 (0.17 to 0.21)	7.18 (6.55 to 7.82)	0.30 (0.27 to 0.34)	8.56 (7.51 to 9.61)	0.60 (0.17 to 0.99)	0.02 (0.01 to 0.02)	0.65 (0.60 to 0.70)	0.01 (0.01 to 0.01)	0.60 (0.53 to 0.68)	-0.22 (-0.90 to 0.26)	0.17 (0.16 to 0.19)	6.53 (5.93 to 7.15)	0.29 (0.26 to 0.33)	7.96 (6.97 to 8.95)	0.66 (0.20 to 1.07)
Romania	1.16 (1.09 to 1.25)	7.66 (7.16 to 8.16)	1.46 (1.30 to 1.66)	6.14 (5.47 to 6.95)	-0.81 (-1.04 to -0.64)	0.12 (0.11 to 0.13)	0.85 (0.80 to 0.89)	0.04 (0.04 to 0.05)	0.24 (0.21 to 0.27)	-4.01 (-4.11 to -3.93)	1.05 (0.97 to 1.13)	6.82 (6.32 to 7.33)	1.42 (1.26 to 1.61)	5.90 (5.25 to 6.69)	-0.56 (-0.77 to -0.40)
Russian Federation	5.52 (5.34 to 5.66)	4.67 (4.52 to 4.78)	28.0 (25.1 to 30.5)	17.3 (15.5 to 18.8)	4.57 (4.11 to 4.93)	0.54 (0.50 to 0.58)	0.60 (0.56 to 0.64)	0.44 (0.39 to 0.48)	0.49 (0.44 to 0.54)	-0.56 (-0.90 to -0.21)	4.99 (4.79 to 5.13)	4.07 (3.91 to 4.19)	27.5 (24.8 to 30.0)	16.8 (15.1 to 18.3)	4.94 (4.48 to 5.32)
Rwanda	0.68 (0.44 to 0.99)	48.8 (31.6 to 71.0)	1.01 (0.61 to 1.55)	33.5 (20.1 to 51.5)	-1.23 (-1.28 to -1.18)	0.03 (0.02 to 0.05)	1.10 (0.67 to 1.74)	0.03 (0.02 to 0.05)	0.54 (0.30 to 0.93)	-2.27 (-2.32 to -2.23)	0.65 (0.41 to 0.94)	47.7 (30.6 to 69.7)	0.98 (0.59 to 1.50)	32.9 (19.9 to 50.6)	-1.21 (-1.26 to -1.16)
Saint Kitts and Nevis	0.02 (0.02 to 0.02)	83.1 (76.2 to 90.3)	0.02 (0.01 to 0.02)	46.7 (39.5 to 54.0)	-2.00 (-2.28 to -1.70)	5e-04 (5e-04 to 6e-04)	2.65 (2.37 to 2.93)	3e-04 (3e-04 to 4e-04)	0.92 (0.75 to 1.11)	-3.34 (-3.42 to -3.26)	0.02 (0.02 to 0.02)	80.5 (73.7 to 87.4)	0.01 (0.01 to 0.02)	45.8 (38.8 to 53.0)	-1.96 (-2.25 to -1.65)
Saint Lucia	0.05 (0.05 to 0.05)	108 (101 to 114)	0.08 (0.06 to 0.09)	59.4 (49.5 to 69.8)	-2.27 (-2.53 to -1.99)	0.001 (0.001 to 0.002)	2.65 (2.43 to 2.89)	0.001 (0.001 to 0.002)	1.19 (0.97 to 1.45)	-2.68 (-2.83 to -2.57)	0.05 (0.05 to 0.05)	105 (98.2 to 112)	0.08 (0.06 to 0.09)	58.2 (48.5 to 68.4)	-2.25 (-2.52 to -1.97)
Saint Vincent and the Grenadines	0.05 (0.04 to 0.05)	119 (109 to 128)	0.05 (0.05 to 0.06)	74.1 (65.7 to 83.4)	-1.36 (-1.55 to -1.18)	0.001 (0.001 to 0.002)	3.28 (2.97 to 3.57)	0.001 (0.001 to 0.001)	1.88 (1.60 to 2.17)	-1.83 (-1.91 to -1.74)	0.05 (0.04 to 0.05)	116 (106 to 125)	0.05 (0.04 to 0.06)	72.3 (64.1 to 81.3)	-1.35 (-1.54 to -1.17)
Samoa	0.03 (0.02 to 0.03)	65.9 (51.9 to 84.6)	0.07 (0.05 to 0.09)	105 (79.6 to 137)	1.51 (1.50 to 1.52)	3e-04 (2e-04 to 5e-04)	0.40 (0.26 to 0.58)	4e-04 (3e-04 to 5e-04)	0.40 (0.28 to 0.56)	-0.03 (-0.06 to -0.00)	0.03 (0.02 to 0.03)	65.5 (51.6 to 84.1)	0.07 (0.05 to 0.09)	105 (79.3 to 136)	1.52 (1.51 to 1.53)
San Marino	0.002 (0.001 to 0.002)	7.32 (5.73 to 9.22)	0.002 (0.001 to 0.003)	3.52 (2.32 to 4.87)	-2.56 (-2.83 to -2.37)	5e-05 (3e-05 to 6e-05)	0.26 (0.19 to 0.34)	4e-05 (2e-05 to 6e-05)	0.11 (0.07 to 0.16)	-3.00 (-3.30 to -2.84)	0.002 (0.001 to 0.002)	7.06 (5.51 to 8.90)	0.002 (0.001 to 0.003)	3.41 (2.26 to 4.72)	-2.55 (-2.82 to -2.36)
Sao Tome and Principe	0.004 (0.003 to 0.004)	10.4 (8.69 to 12.4)	0.007 (0.006 to 0.009)	13.9 (11.1 to 17.1)	0.96 (0.92 to 1.00)	3e-04 (2e-04 to 4e-04)	0.60 (0.38 to 0.81)	4e-04 (3e-04 to 6e-04)	0.52 (0.33 to 0.77)	-0.44 (-0.58 to -0.32)	0.003 (0.003 to 0.004)	9.82 (8.21 to 11.7)	0.007 (0.005 to 0.008)	13.4 (10.6 to 16.4)	1.02 (0.94 to 1.09)
Saudi Arabia	0.59 (0.45 to 0.73)	25.6 (19.4 to 31.8)	1.92 (1.53 to 2.40)	28.5 (22.8 to 35.2)	0.30 (0.21 to 0.36)	0.03 (0.02 to 0.04)	0.52 (0.36 to 0.72)	0.05 (0.03 to 0.09)	0.32 (0.19 to 0.49)	-1.57 (-1.63 to -1.51)	0.56 (0.42 to 0.70)	25.1 (19.0 to 31.1)	1.86 (1.49 to 2.34)	28.2 (22.6 to 34.9)	0.32 (0.23 to 0.39)
Senegal	0.44 (0.35 to 0.54)	30.7 (24.0 to 37.7)	1.75 (1.32 to 2.28)	49.9 (37.7 to 64.5)	1.51 (1.39 to 1.59)	0.02 (0.02 to 0.03)	0.75 (0.53 to 1.02)	0.04 (0.02 to 0.05)	0.56 (0.38 to 0.89)	-1.06 (-1.31 to -0.85)	0.42 (0.33 to 0.51)	30.0 (23.4 to 37.0)	1.72 (1.29 to 2.23)	49.4 (37.2 to 63.6)	1.56 (1.44 to 1.63)
Serbia	1.22 (1.04 to 1.42)	23.4 (20.0 to 27.6)	2.15 (1.80 to 2.55)	21.0 (17.6 to 25.0)	-0.33 (-0.48 to -0.18)	0.12 (0.09 to 0.16)	2.27 (1.60 to 2.86)	0.08 (0.06 to 0.12)	0.98 (0.71 to 1.41)	-2.66 (-2.82 to -2.51)	1.10 (0.93 to 1.28)	21.1 (17.9 to 24.9)	2.07 (1.73 to 2.47)	20.1 (16.8 to 24.0)	-0.15 (-0.30 to -0.01)
Seychelles	0.006 (0.005 to 0.007)	17.1 (14.7 to 19.7)	0.02 (0.01 to 0.02)	26.9 (23.0 to 31.1)	1.49 (1.24 to 1.71)	2e-04 (2e-04 to 3e-04)	0.79 (0.60 to 1.06)	3e-04 (2e-04 to 4e-04)	0.56 (0.35 to 0.76)	-1.06 (-1.10 to -1.02)	0.005 (0.005 to 0.006)	16.3 (14.0 to 18.8)	0.02 (0.01 to 0.02)	26.3 (22.6 to 30.4)	1.55 (1.29 to 1.76)
Sierra Leone	0.19 (0.15 to 0.24)	21.0 (17.0 to 26.2)	0.59 (0.45 to 0.77)	35.5 (27.5 to 45.8)	1.73 (1.71 to 1.76)	0.010 (0.006 to 0.01)	0.56 (0.35 to 0.85)	0.02 (0.01 to 0.03)	0.50 (0.32 to 0.75)	-0.38 (-0.43 to -0.34)	0.18 (0.14 to 0.23)	20.4 (16.4 to 25.4)	0.57 (0.44 to 0.74)	35.0 (27.1 to 45.2)	1.77 (1.74 to 1.80)
Singapore	0.22 (0.20 to 0.24)	20.2 (18.5 to 21.9)	0.08 (0.07 to 0.09)	1.79 (1.50 to 2.01)	-7.62 (-7.98 to -7.32)	0.004 (0.004 to 0.004)	0.26 (0.24 to 0.27)	0.001 (9e-04 to 0.001)	0.03 (0.03 to 0.03)	-6.79 (-6.92 to -6.65)	0.22 (0.20 to 0.24)	19.9 (18.3 to 21.6)	0.08 (0.07 to 0.09)	1.76 (1.47 to 1.98)	-7.63 (-7.99 to -7.32)
Slovakia	0.43 (0.36 to 0.50)	11.9 (10.2 to 13.8)	0.48 (0.38 to 0.58)	8.05 (6.39 to 9.81)	-1.25 (-1.40 to -1.11)	0.04 (0.03 to 0.05)	1.08 (0.81 to 1.37)	0.02 (0.02 to 0.03)	0.48 (0.35 to 0.67)	-2.51 (-2.64 to -2.39)	0.39 (0.33 to 0.46)	10.8 (9.18 to 12.6)	0.45 (0.36 to 0.55)	7.57 (6.01 to 9.21)	-1.14 (-1.30 to -1.00)
Slovenia	0.18 (0.17 to 0.20)	11.8 (10.7 to 12.8)	0.21 (0.17 to 0.24)	6.68 (5.57 to 7.77)	-1.70 (-2.05 to -1.30)	0.02 (0.02 to 0.02)	1.18 (1.10 to 1.29)	0.005 (0.004 to 0.005)	0.23 (0.19 to 0.26)	-5.24 (-5.46 to -4.98)	0.17 (0.15 to 0.18)	10.6 (9.58 to 11.6)	0.20 (0.17 to 0.24)	6.45 (5.37 to 7.51)	-1.46 (-1.82 to -1.04)
Solomon Islands	0.05 (0.03 to 0.06)	81.4 (61.5 to 106)	0.18 (0.14 to 0.23)	111 (86.6 to 141)	0.99 (0.92 to 1.05)	6e-04 (3e-04 to 0.001)	0.51 (0.20 to 1.04)	0.002 (0.001 to 0.003)	0.61 (0.40 to 0.97)	0.57 (0.49 to 0.63)	0.04 (0.03 to 0.06)	80.9 (61.2 to 105)	0.18 (0.14 to 0.23)	110 (86.2 to 140)	1.00 (0.92 to 1.05)
Somalia	0.48 (0.35 to 0.63)	44.4 (32.4 to 57.9)	1.47 (1.07 to 1.95)	50.0 (36.1 to 63.9)	0.39 (0.37 to 0.42)	0.03 (0.02 to 0.05)	0.92 (0.60 to 1.55)	0.08 (0.05 to 0.12)	0.92 (0.60 to 1.37)	0.02 (-0.01 to 0.06)	0.45 (0.33 to 0.60)	43.5 (31.8 to 56.8)	1.39 (1.01 to 1.83)	49.1 (35.4 to 62.7)	0.40 (0.38 to 0.42)
South Africa	4.91 (4.35 to 5.42)	43.3 (38.1 to 47.8)	19.5 (17.9 to 21.0)	77.7 (71.1 to 83.9)	1.87 (1.62 to 2.10)	0.09 (0.07 to 0.12)	0.47 (0.38 to 0.63)	0.11 (0.08 to 0.13)	0.36 (0.28 to 0.45)	-0.82 (-0.95 to -0.68)	4.82 (4.26 to 5.31)	42.8 (37.7 to 47.3)	19.4 (17.8 to 20.9)	77.4 (70.8 to 83.5)	1.89 (1.64 to 2.13)
South Sudan	0.36 (0.27 to 0.48)	37.0 (27.3 to 49.4)	0.71 (0.51 to 0.93)	45.5 (32.9 to 59.1)	0.70 (0.68 to 0.73)	0.02 (0.01 to 0.02)	0.67 (0.41 to 0.99)	0.03 (0.02 to 0.04)	0.68 (0.44 to 1.02)	0.09 (-0.00 to 0.20)	0.34 (0.25 to 0.46)	36.3 (26.8 to 48.7)	0.68 (0.49 to 0.90)	44.9 (32.5 to 58.3)	0.71 (0.69 to 0.74)
Spain	6.42 (5.63 to 6.97)	19.0 (16.7 to 20.5)	5.15 (3.92 to 5.96)	5.93 (4.64 to 6.77)	-3.64 (-3.74 to -3.56)	0.08 (0.07 to 0.08)	0.31 (0.29 to 0.33)	0.03 (0.03 to 0.04)	0.08 (0.07 to 0.08)	-4.43 (-4.50 to -4.36)	6.34 (5.56 to 6.89)	18.7 (16.4 to 20.2)	5.12 (3.89 to 5.93)	5.85 (4.57 to 6.69)	-3.63 (-3.73 to -3.55)
Sri Lanka	1.54 (1.30 to 1.78)	35.1 (29.7 to 40.7)	6.79 (4.69 to 9.01)	46.3 (32.0 to 61.7)	0.90 (0.64 to 1.08)	0.08 (0.06 to 0.11)	1.09 (0.80 to 1.55)	0.11 (0.06 to 0.16)	0.77 (0.47 to 1.14)	-0.99 (-1.13 to -0.87)	1.47 (1.23 to 1.70)	34.0 (28.7 to 39.5)	6.68 (4.60 to 8.88)	45.6 (31.5 to 60.6)	0.95 (0.69 to 1.13)
Sudan	0.64 (0.50 to 0.90)	14.6 (11.5 to 20.1)	1.75 (1.29 to 2.38)	21.2 (16.3 to 28.3)	1.21 (1.19 to 1.23)	0.07 (0.04 to 0.12)	0.72 (0.43 to 1.24)	0.11 (0.06 to 0.17)	0.54 (0.32 to 0.87)	-0.91 (-0.96 to -0.86)	0.57 (0.44 to 0.78)	13.9 (10.9 to 19.1)	1.64 (1.22 to 2.23)	20.7 (15.7 to 27.7)	1.29 (1.27 to 1.31)
Suriname	0.06 (0.05 to 0.06)	44.1 (39.4 to 49.9)	0.14 (0.11 to 0.17)	41.1 (33.2 to 50.4)	-0.14 (-0.38 to 0.05)	0.002 (0.001 to 0.002)	1.20 (0.91 to 1.49)	0.003 (0.002 to 0.004)	0.90 (0.65 to 1.18)	-0.88 (-1.02 to -0.74)	0.05 (0.05 to 0.06)	42.9 (38.3 to 48.5)	0.14 (0.11 to 0.17)	40.2 (32.4 to 49.4)	-0.12 (-0.36 to 0.07)
Sweden	0.82 (0.71 to 0.89)	8.21 (7.27 to 8.79)	0.96 (0.76 to 1.12)	6.17 (4.99 to 7.14)	-1.05 (-1.19 to -0.92)	0.04 (0.04 to 0.04)	0.58 (0.54 to 0.61)	0.02 (0.02 to 0.03)	0.24 (0.21 to 0.27)	-2.64 (-2.78 to -2.49)	0.78 (0.68 to 0.85)	7.64 (6.71 to 8.20)	0.94 (0.74 to 1.10)	5.92 (4.78 to 6.87)	-0.88 (-1.05 to -0.75)
Switzerland	0.98 (0.85 to 1.08)	13.5 (11.9 to 14.8)	0.59 (0.45 to 0.68)	4.15 (3.27 to 4.73)	-3.80 (-3.96 to -3.61)	0.02 (0.02 to 0.03)	0.47 (0.44 to 0.51)	0.01 (0.009 to 0.01)	0.11 (0.10 to 0.13)	-4.50 (-4.60 to -4.40)	0.96 (0.82 to 1.05)	13.1 (11.4 to 14.3)	0.58 (0.44 to 0.67)	4.04 (3.17 to 4.61)	-3.77 (-3.93 to -3.58)
Syrian Arab Republic	0.49 (0.39 to 0.59)	22.2 (18.0 to 26.8)	1.11 (0.84 to 1.47)	23.5 (18.3 to 30.2)	0.27 (0.13 to 0.40)	0.03 (0.02 to 0.04)	0.58 (0.42 to 0.78)	0.02 (0.01 to 0.03)	0.32 (0.21 to 0.45)	-1.99 (-2.07 to -1.92)	0.46 (0.37 to 0.56)	21.7 (17.5 to 26.1)	1.09 (0.82 to 1.44)	23.2 (18.1 to 29.8)	0.31 (0.17 to 0.45)
Taiwan (Province of China)	2.94 (2.75 to 3.10)	43.1 (40.1 to 45.7)	5.18 (4.33 to 5.78)	21.3 (18.0 to 23.7)	-2.27 (-2.43 to -2.12)	0.19 (0.18 to 0.21)	2.38 (2.22 to 2.54)	0.20 (0.17 to 0.22)	1.00 (0.90 to 1.10)	-2.78 (-2.90 to -2.65)	2.74 (2.56 to 2.90)	40.8 (37.8 to 43.3)	4.99 (4.15 to 5.58)	20.3 (17.1 to 22.6)	-2.24 (-2.40 to -2.08)
Tajikistan	0.18 (0.16 to 0.22)	11.7 (9.87 to 13.7)	0.45 (0.33 to 0.57)	15.2 (11.2 to 19.3)	0.84 (0.77 to 0.90)	0.02 (0.01 to 0.03)	0.89 (0.60 to 1.27)	0.03 (0.02 to 0.05)	0.77 (0.49 to 1.12)	-0.43 (-0.51 to -0.37)	0.16 (0.14 to 0.20)	10.8 (9.07 to 12.7)	0.41 (0.31 to 0.53)	14.4 (10.6 to 18.3)	0.92 (0.85 to 0.99)
Thailand	4.91 (3.83 to 6.01)	27.3 (21.3 to 33.6)	14.3 (10.8 to 18.3)	23.7 (18.0 to 30.4)	-0.50 (-0.59 to -0.41)	0.25 (0.18 to 0.36)	1.05 (0.73 to 1.55)	0.29 (0.20 to 0.40)	0.59 (0.41 to 0.81)	-1.83 (-1.91 to -1.75)	4.66 (3.64 to 5.70)	26.3 (20.6 to 32.4)	14.0 (10.6 to 17.9)	23.1 (17.6 to 29.6)	-0.43 (-0.53 to -0.34)
Timor-Leste	0.03 (0.02 to 0.04)	23.2 (16.9 to 31.6)	0.11 (0.09 to 0.14)	26.5 (20.8 to 34.8)	0.39 (0.33 to 0.44)	0.002 (0.002 to 0.005)	0.99 (0.62 to 1.89)	0.004 (0.003 to 0.007)	0.78 (0.51 to 1.24)	-0.76 (-0.86 to -0.65)	0.03 (0.02 to 0.04)	22.2 (16.2 to 30.0)	0.10 (0.08 to 0.14)	25.8 (20.1 to 33.9)	0.44 (0.38 to 0.49)
Togo	0.13 (0.10 to 0.16)	22.8 (17.6 to 28.8)	0.58 (0.43 to 0.77)	32.6 (24.6 to 42.8)	1.17 (1.14 to 1.20)	0.009 (0.006 to 0.01)	0.62 (0.43 to 0.91)	0.02 (0.010 to 0.02)	0.45 (0.29 to 0.71)	-1.05 (-1.11 to -1.01)	0.12 (0.10 to 0.15)	22.1 (17.1 to 28.1)	0.56 (0.42 to 0.75)	32.2 (24.3 to 42.3)	1.22 (1.19 to 1.24)
Tokelau	6e-04 (5e-04 to 8e-04)	86.4 (67.0 to 113)	8e-04 (6e-04 to 0.001)	98.1 (73.9 to 128)	0.43 (0.42 to 0.44)	5e-06 (3e-06 to 6e-06)	0.58 (0.39 to 0.83)	8e-06 (5e-06 to 1e-05)	1.24 (0.84 to 1.79)	3.05 (2.27 to 4.26)	6e-04 (4e-04 to 8e-04)	85.8 (66.5 to 113)	8e-04 (6e-04 to 1e-03)	96.9 (72.9 to 126)	0.40 (0.38 to 0.41)
Tonga	0.03 (0.02 to 0.03)	103 (86.1 to 119)	0.05 (0.04 to 0.07)	122 (92.2 to 156)	0.54 (0.48 to 0.60)	3e-04 (2e-04 to 4e-04)	0.54 (0.39 to 0.76)	2e-04 (2e-04 to 4e-04)	0.46 (0.31 to 0.68)	-0.52 (-0.55 to -0.50)	0.03 (0.02 to 0.03)	102 (85.6 to 118)	0.05 (0.04 to 0.07)	122 (91.9 to 155)	0.62 (0.57 to 0.66)
Trinidad and Tobago	0.55 (0.52 to 0.58)	132 (125 to 139)	0.90 (0.70 to 1.11)	87.2 (68.1 to 108)	-1.32 (-1.52 to -1.13)	0.01 (0.01 to 0.02)	2.93 (2.74 to 3.14)	0.01 (0.01 to 0.02)	1.66 (1.26 to 2.13)	-1.65 (-1.80 to -1.51)	0.54 (0.51 to 0.57)	129 (122 to 136)	0.88 (0.69 to 1.10)	85.6 (66.9 to 106)	-1.31 (-1.50 to -1.12)
Tunisia	0.22 (0.18 to 0.27)	10.4 (8.41 to 12.6)	1.01 (0.75 to 1.33)	15.4 (11.4 to 20.3)	1.34 (1.26 to 1.41)	0.01 (0.009 to 0.02)	0.36 (0.23 to 0.67)	0.02 (0.010 to 0.03)	0.25 (0.16 to 0.42)	-1.07 (-1.12 to -1.02)	0.21 (0.17 to 0.26)	10.0 (8.13 to 12.3)	1.00 (0.74 to 1.32)	15.2 (11.2 to 20.1)	1.40 (1.32 to 1.48)
Turkmenistan	0.12 (0.10 to 0.13)	10.4 (9.18 to 11.7)	0.50 (0.39 to 0.65)	21.7 (16.9 to 28.1)	2.52 (2.25 to 2.73)	0.02 (0.02 to 0.02)	1.38 (1.20 to 1.61)	0.04 (0.03 to 0.05)	1.45 (1.08 to 1.95)	0.22 (0.01 to 0.50)	0.10 (0.09 to 0.11)	9.04 (7.84 to 10.3)	0.46 (0.36 to 0.61)	20.3 (15.7 to 26.2)	2.77 (2.51 to 2.98)
Tuvalu	0.003 (0.003 to 0.004)	94.0 (75.4 to 120)	0.006 (0.005 to 0.008)	114 (89.0 to 149)	0.65 (0.62 to 0.67)	3e-05 (2e-05 to 5e-05)	0.66 (0.45 to 1.10)	3e-05 (2e-05 to 4e-05)	0.49 (0.34 to 0.70)	-0.95 (-1.00 to -0.90)	0.003 (0.003 to 0.004)	93.3 (74.8 to 119)	0.006 (0.005 to 0.008)	114 (88.7 to 148)	0.66 (0.63 to 0.68)
Türkiye	6.42 (5.44 to 7.48)	38.9 (32.8 to 45.6)	12.5 (9.92 to 15.4)	26.3 (20.8 to 32.3)	-1.18 (-1.46 to -0.97)	0.22 (0.15 to 0.32)	0.80 (0.54 to 1.21)	0.10 (0.08 to 0.13)	0.23 (0.18 to 0.30)	-3.91 (-4.01 to -3.78)	6.20 (5.25 to 7.28)	38.1 (32.0 to 44.8)	12.4 (9.84 to 15.3)	26.1 (20.6 to 32.0)	-1.14 (-1.42 to -0.93)
Uganda	0.90 (0.60 to 1.26)	31.4 (21.3 to 43.6)	2.61 (1.73 to 3.60)	36.9 (25.1 to 50.9)	0.50 (0.47 to 0.53)	0.04 (0.02 to 0.06)	0.50 (0.28 to 0.84)	0.09 (0.06 to 0.14)	0.51 (0.29 to 0.81)	0.06 (-0.02 to 0.14)	0.86 (0.57 to 1.21)	30.9 (20.9 to 42.8)	2.51 (1.66 to 3.46)	36.4 (24.8 to 50.1)	0.51 (0.48 to 0.54)
Ukraine	1.81 (1.69 to 1.93)	4.11 (3.86 to 4.37)	1.48 (1.00 to 2.07)	3.18 (2.11 to 4.48)	-0.66 (-1.11 to -0.33)	0.24 (0.21 to 0.27)	0.77 (0.70 to 0.86)	0.10 (0.06 to 0.15)	0.39 (0.24 to 0.58)	-2.65 (-3.14 to -2.20)	1.57 (1.44 to 1.70)	3.34 (3.07 to 3.61)	1.38 (0.94 to 1.93)	2.79 (1.87 to 3.93)	-0.35 (-0.86 to 0.03)
United Arab Emirates	0.06 (0.05 to 0.07)	47.0 (36.7 to 58.7)	0.27 (0.22 to 0.34)	107 (85.6 to 129)	2.54 (2.06 to 2.99)	0.002 (0.002 to 0.003)	0.60 (0.43 to 0.85)	0.006 (0.004 to 0.008)	0.61 (0.43 to 0.83)	-0.05 (-0.36 to 0.21)	0.06 (0.04 to 0.07)	46.4 (36.2 to 57.8)	0.27 (0.21 to 0.33)	106 (85.1 to 129)	2.83 (2.29 to 3.33)
United Kingdom	4.85 (4.44 to 5.07)	8.21 (7.60 to 8.54)	3.20 (2.67 to 3.46)	3.63 (3.12 to 3.89)	-2.54 (-2.70 to -2.39)	0.17 (0.17 to 0.18)	0.42 (0.41 to 0.43)	0.08 (0.07 to 0.08)	0.16 (0.16 to 0.17)	-3.16 (-3.35 to -3.03)	4.68 (4.28 to 4.89)	7.79 (7.18 to 8.11)	3.12 (2.59 to 3.38)	3.47 (2.97 to 3.73)	-2.51 (-2.68 to -2.36)
United Republic of Tanzania	1.56 (1.27 to 1.90)	31.6 (25.4 to 38.2)	4.01 (3.18 to 5.20)	33.9 (27.2 to 43.6)	0.26 (0.22 to 0.30)	0.09 (0.06 to 0.12)	0.69 (0.48 to 0.97)	0.15 (0.10 to 0.22)	0.55 (0.36 to 0.81)	-0.73 (-0.77 to -0.70)	1.47 (1.18 to 1.79)	30.9 (24.8 to 37.4)	3.86 (3.05 to 5.01)	33.3 (26.8 to 43.0)	0.28 (0.24 to 0.32)
United States Virgin Islands	0.02 (0.02 to 0.02)	50.7 (42.7 to 59.7)	0.03 (0.02 to 0.03)	24.8 (19.5 to 31.1)	-2.38 (-2.61 to -2.21)	0.001 (8e-04 to 0.001)	2.21 (1.67 to 2.93)	8e-04 (5e-04 to 0.001)	1.13 (0.66 to 1.64)	-2.03 (-2.21 to -1.81)	0.02 (0.02 to 0.02)	48.4 (40.5 to 57.2)	0.02 (0.02 to 0.03)	23.7 (18.5 to 29.6)	-2.41 (-2.66 to -2.22)
United States of America	27.7 (24.9 to 29.4)	14.3 (13.0 to 15.0)	32.1 (27.9 to 34.5)	9.59 (8.56 to 10.2)	-1.30 (-1.35 to -1.25)	1.23 (1.18 to 1.27)	0.78 (0.75 to 0.80)	1.21 (1.15 to 1.25)	0.58 (0.56 to 0.60)	-0.94 (-1.00 to -0.89)	26.5 (23.7 to 28.1)	13.5 (12.2 to 14.2)	30.8 (26.8 to 33.2)	9.01 (7.98 to 9.63)	-1.33 (-1.38 to -1.27)
Uruguay	0.38 (0.34 to 0.42)	16.2 (14.7 to 17.7)	0.51 (0.44 to 0.56)	13.6 (11.9 to 15.0)	-0.68 (-0.82 to -0.55)	0.008 (0.007 to 0.009)	0.41 (0.38 to 0.44)	0.007 (0.006 to 0.008)	0.27 (0.24 to 0.30)	-1.36 (-1.45 to -1.25)	0.37 (0.34 to 0.41)	15.8 (14.4 to 17.3)	0.51 (0.43 to 0.56)	13.4 (11.7 to 14.7)	-0.66 (-0.80 to -0.52)
Uzbekistan	0.62 (0.58 to 0.67)	9.16 (8.47 to 9.89)	3.61 (3.04 to 4.23)	25.2 (21.4 to 29.3)	3.29 (3.08 to 3.46)	0.07 (0.06 to 0.08)	0.83 (0.67 to 1.01)	0.19 (0.15 to 0.23)	1.14 (0.92 to 1.38)	1.04 (0.88 to 1.22)	0.55 (0.50 to 0.60)	8.33 (7.62 to 9.04)	3.42 (2.88 to 4.02)	24.0 (20.3 to 28.1)	3.46 (3.24 to 3.63)
Vanuatu	0.02 (0.01 to 0.03)	73.5 (55.0 to 96.8)	0.08 (0.06 to 0.09)	97.5 (77.7 to 117)	0.91 (0.86 to 0.96)	4e-04 (2e-04 to 5e-04)	0.48 (0.29 to 0.72)	8e-04 (5e-04 to 0.001)	0.51 (0.35 to 0.72)	0.25 (0.15 to 0.36)	0.02 (0.01 to 0.02)	73.0 (54.6 to 96.2)	0.08 (0.06 to 0.09)	97.0 (77.3 to 116)	0.91 (0.87 to 0.96)
Venezuela (Bolivarian Republic of)	1.71 (1.59 to 1.82)	35.6 (32.9 to 37.9)	6.34 (5.05 to 7.97)	39.8 (31.7 to 49.7)	0.45 (0.27 to 0.64)	0.05 (0.05 to 0.05)	0.70 (0.66 to 0.75)	0.10 (0.08 to 0.13)	0.71 (0.54 to 0.90)	0.36 (-0.02 to 0.67)	1.66 (1.55 to 1.77)	34.9 (32.2 to 37.1)	6.24 (4.96 to 7.83)	39.1 (31.2 to 48.8)	0.46 (0.27 to 0.65)
Viet Nam	7.49 (5.94 to 9.66)	33.1 (26.2 to 42.6)	19.6 (15.6 to 24.3)	37.6 (29.8 to 46.4)	0.42 (0.40 to 0.44)	0.26 (0.17 to 0.42)	1.00 (0.62 to 1.65)	0.34 (0.19 to 0.57)	0.61 (0.34 to 1.01)	-1.57 (-1.58 to -1.55)	7.23 (5.73 to 9.30)	32.1 (25.5 to 41.3)	19.3 (15.3 to 23.9)	37.0 (29.4 to 45.8)	0.46 (0.45 to 0.48)
Yemen	0.32 (0.23 to 0.49)	12.9 (9.49 to 19.7)	1.10 (0.77 to 1.71)	16.8 (11.9 to 25.6)	0.85 (0.81 to 0.88)	0.04 (0.02 to 0.07)	0.58 (0.30 to 1.14)	0.07 (0.05 to 0.13)	0.49 (0.30 to 0.84)	-0.49 (-0.57 to -0.39)	0.28 (0.20 to 0.44)	12.3 (9.10 to 18.8)	1.02 (0.71 to 1.58)	16.3 (11.6 to 24.8)	0.90 (0.87 to 0.93)
Zambia	0.48 (0.39 to 0.59)	39.1 (31.6 to 47.7)	1.10 (0.80 to 1.49)	35.6 (26.4 to 47.2)	-0.29 (-0.33 to -0.26)	0.03 (0.02 to 0.05)	0.93 (0.65 to 1.37)	0.05 (0.03 to 0.07)	0.57 (0.38 to 0.86)	-1.54 (-1.57 to -1.51)	0.45 (0.36 to 0.55)	38.2 (30.8 to 46.8)	1.05 (0.76 to 1.42)	35.0 (25.9 to 46.5)	-0.27 (-0.30 to -0.23)
Zimbabwe	0.76 (0.62 to 0.93)	41.4 (33.5 to 50.4)	2.77 (2.08 to 3.63)	79.5 (60.9 to 102)	2.21 (2.15 to 2.27)	0.007 (0.005 to 0.01)	0.16 (0.12 to 0.21)	0.02 (0.01 to 0.04)	0.31 (0.19 to 0.49)	2.09 (1.95 to 2.22)	0.75 (0.61 to 0.92)	41.3 (33.3 to 50.2)	2.74 (2.07 to 3.60)	79.2 (60.7 to 102)	2.21 (2.15 to 2.27)

AAPC: Average annual percentage change; ASR: Age-standardized rate; DM: Diabetes mellitus; SDI: Sociodemographic index; T1D: Type 1 diabetes; T2D: Type 2 diabetes. Results assuming first-order autocorrelation estimated from the data were broadly similar to those of the primary analyses, despite somewhat different estimates.

Table S4. YLD number, age-standardized rate, and AAPC of diabetes and two subtypes among **women** globally and by different SDI, regions, countries, and territories, 1990-2021

Location	DM, Number in 1990 (000s)	DM, ASR in 1990 (per 100,000)	DM, Number in 2021 (000s)	DM, ASR in 2021 (per 100,000)	DM, AAPC (95%CI)	T1D, Number in 1990 (000s)	T1D, ASR in 1990 (per 100,000)	T1D, Number in 2021 (000s)	T1D, ASR in 2021 (per 100,000)	T1D, AAPC (95%CI)	T2D, Number in 1990 (000s)	T2D, ASR in 1990 (per 100,000)	T2D, Number in 2021 (000s)	T2D, ASR in 2021 (per 100,000)	T2D, AAPC (95%CI)
Global	5279 (3737 to 7295)	238 (169 to 329)	20127 (14221 to 27420)	449 (317 to 612)	2.06 (2.05 to 2.07)	332 (216 to 480)	13.7 (8.93 to 19.7)	686 (446 to 996)	16.2 (10.5 to 23.6)	0.54 (0.54 to 0.55)	4947 (3488 to 6878)	225 (159 to 312)	19441 (13699 to 26504)	433 (305 to 591)	2.13 (2.12 to 2.14)
**By SDI**															
High SDI	1208 (860 to 1655)	207 (147 to 286)	4235 (3008 to 5777)	452 (319 to 620)	2.56 (2.54 to 2.57)	102 (69.0 to 143)	20.1 (13.6 to 28.3)	195 (130 to 274)	27.3 (17.9 to 38.6)	0.98 (0.97 to 0.99)	1106 (788 to 1532)	187 (132 to 260)	4040 (2860 to 5503)	425 (299 to 582)	2.69 (2.68 to 2.70)
High-middle SDI	1278 (903 to 1793)	228 (161 to 319)	3977 (2816 to 5483)	404 (283 to 553)	1.87 (1.87 to 1.88)	65.2 (42.4 to 94.5)	11.8 (7.71 to 17.1)	135 (89.3 to 194)	16.0 (10.3 to 23.4)	0.97 (0.94 to 0.99)	1213 (854 to 1695)	216 (153 to 302)	3842 (2712 to 5308)	388 (270 to 532)	1.92 (1.91 to 1.92)
Middle SDI	1669 (1174 to 2298)	283 (201 to 390)	6654 (4695 to 9097)	466 (329 to 636)	1.63 (1.61 to 1.64)	78.6 (50.0 to 116)	10.4 (6.64 to 15.4)	165 (105 to 246)	12.3 (7.87 to 18.3)	0.57 (0.55 to 0.58)	1591 (1119 to 2196)	272 (194 to 377)	6488 (4563 to 8898)	454 (320 to 621)	1.66 (1.64 to 1.68)
Low-middle SDI	830 (586 to 1136)	241 (171 to 328)	3962 (2783 to 5435)	488 (344 to 667)	2.29 (2.28 to 2.30)	61.5 (40.0 to 90.2)	13.2 (8.46 to 19.4)	133 (84.9 to 197)	14.5 (9.36 to 21.5)	0.31 (0.30 to 0.32)	768 (539 to 1062)	228 (161 to 314)	3829 (2689 to 5263)	473 (333 to 649)	2.38 (2.36 to 2.39)
Low SDI	286 (199 to 392)	219 (155 to 302)	1277 (882 to 1765)	402 (281 to 554)	1.97 (1.96 to 1.98)	24.0 (16.1 to 34.4)	12.5 (8.29 to 18.0)	57.0 (37.8 to 82.3)	12.7 (8.40 to 18.4)	0.04 (0.04 to 0.05)	262 (183 to 365)	207 (146 to 286)	1220 (835 to 1695)	389 (272 to 540)	2.05 (2.04 to 2.06)
**Four world regions**															
Africa	369 (258 to 509)	227 (159 to 313)	1910 (1312 to 2655)	480 (328 to 668)	2.45 (2.44 to 2.45)	30.5 (20.3 to 44.0)	12.8 (8.57 to 18.8)	72.4 (48.8 to 106)	13.0 (8.72 to 19.0)	0.05 (0.04 to 0.05)	338 (237 to 471)	214 (150 to 297)	1838 (1258 to 2562)	467 (318 to 652)	2.55 (2.54 to 2.55)
America	1048 (740 to 1430)	315 (223 to 431)	4097 (2916 to 5519)	597 (422 to 805)	2.09 (2.07 to 2.10)	78.6 (52.0 to 112)	22.5 (14.9 to 32.1)	155 (103 to 218)	25.3 (16.6 to 35.8)	0.38 (0.37 to 0.39)	970 (687 to 1328)	292 (207 to 402)	3943 (2806 to 5343)	571 (404 to 774)	2.19 (2.18 to 2.20)
Asia	2762 (1928 to 3808)	242 (171 to 334)	11287 (7967 to 15350)	430 (303 to 585)	1.87 (1.86 to 1.89)	139 (89.6 to 204)	9.89 (6.38 to 14.4)	293 (187 to 433)	11.9 (7.61 to 17.6)	0.60 (0.59 to 0.61)	2623 (1816 to 3614)	232 (163 to 321)	10994 (7756 to 14946)	418 (295 to 569)	1.91 (1.90 to 1.93)
Europe	1079 (760 to 1494)	188 (133 to 261)	2774 (1944 to 3869)	369 (259 to 515)	2.19 (2.17 to 2.20)	82.1 (54.7 to 118)	17.0 (11.3 to 24.5)	164 (110 to 233)	27.8 (17.9 to 40.1)	1.58 (1.55 to 1.60)	997 (701 to 1383)	171 (120 to 238)	2610 (1824 to 3652)	341 (239 to 477)	2.24 (2.23 to 2.26)
**Six WHO regions**															
African Region	283 (198 to 388)	219 (155 to 298)	1304 (899 to 1789)	407 (283 to 559)	2.02 (2.01 to 2.02)	23.6 (15.7 to 33.9)	12.3 (8.18 to 17.9)	56.5 (37.7 to 82.7)	12.3 (8.27 to 18.0)	0.01 (0.00 to 0.01)	259 (182 to 356)	206 (146 to 283)	1247 (861 to 1723)	394 (274 to 544)	2.11 (2.10 to 2.12)
Region of the Americas	1048 (740 to 1430)	315 (223 to 431)	4097 (2916 to 5519)	597 (422 to 805)	2.09 (2.07 to 2.10)	78.6 (52.0 to 112)	22.5 (14.9 to 32.1)	155 (103 to 218)	25.3 (16.6 to 35.8)	0.38 (0.37 to 0.39)	970 (687 to 1328)	292 (207 to 402)	3943 (2806 to 5343)	571 (404 to 774)	2.19 (2.18 to 2.20)
South-East Asia Region	938 (651 to 1281)	233 (165 to 320)	4428 (3111 to 6054)	445 (314 to 604)	2.10 (2.08 to 2.12)	69.4 (44.2 to 103)	12.8 (8.10 to 18.8)	146 (92.6 to 216)	13.9 (8.85 to 20.5)	0.25 (0.24 to 0.26)	869 (605 to 1193)	221 (156 to 303)	4282 (2993 to 5856)	431 (302 to 588)	2.18 (2.16 to 2.20)
European Region	1123 (790 to 1555)	189 (133 to 262)	2968 (2078 to 4134)	375 (262 to 522)	2.23 (2.22 to 2.24)	85.5 (57.0 to 123)	16.9 (11.2 to 24.3)	173 (116 to 245)	27.3 (17.6 to 39.3)	1.54 (1.51 to 1.56)	1038 (729 to 1440)	172 (121 to 240)	2795 (1951 to 3903)	347 (242 to 485)	2.29 (2.28 to 2.30)
Eastern Mediterranean Region	280 (197 to 391)	287 (201 to 403)	1857 (1295 to 2597)	728 (507 to 1015)	3.04 (3.03 to 3.05)	22.2 (14.4 to 32.0)	16.1 (10.3 to 23.6)	59.2 (37.5 to 86.7)	18.1 (11.5 to 26.4)	0.37 (0.36 to 0.38)	258 (180 to 363)	271 (189 to 380)	1798 (1252 to 2518)	709 (493 to 991)	3.15 (3.13 to 3.16)
Western Pacific Region	1558 (1085 to 2192)	241 (169 to 338)	5306 (3713 to 7371)	390 (269 to 546)	1.57 (1.55 to 1.59)	49.9 (32.5 to 72.1)	6.80 (4.44 to 9.84)	92.5 (59.7 to 136)	8.15 (5.23 to 12.1)	0.62 (0.59 to 0.65)	1508 (1046 to 2124)	234 (164 to 328)	5214 (3650 to 7235)	382 (264 to 533)	1.59 (1.57 to 1.61)
**Seven super regions**															
Southeast Asia, East Asia, and Oceania	1559 (1083 to 2183)	243 (170 to 339)	5663 (3972 to 7724)	393 (274 to 539)	1.58 (1.55 to 1.60)	59.5 (37.9 to 88.0)	7.53 (4.77 to 11.1)	110 (69.5 to 164)	8.84 (5.54 to 13.1)	0.57 (0.54 to 0.60)	1500 (1039 to 2096)	236 (164 to 328)	5553 (3891 to 7563)	384 (267 to 526)	1.60 (1.58 to 1.62)
Central Europe, Eastern Europe, and Central Asia	569 (398 to 781)	202 (142 to 278)	1375 (954 to 1889)	386 (270 to 531)	2.11 (2.10 to 2.11)	32.1 (21.3 to 46.4)	13.1 (8.56 to 19.0)	58.2 (39.1 to 83.1)	20.6 (13.5 to 30.0)	1.49 (1.48 to 1.50)	537 (374 to 739)	189 (132 to 261)	1316 (911 to 1812)	365 (254 to 505)	2.15 (2.14 to 2.16)
South Asia	757 (522 to 1039)	233 (163 to 319)	3627 (2554 to 4966)	440 (311 to 601)	2.04 (2.02 to 2.07)	52.9 (33.9 to 77.9)	12.1 (7.76 to 17.6)	122 (78.3 to 181)	13.6 (8.78 to 20.0)	0.36 (0.35 to 0.38)	704 (487 to 965)	221 (155 to 303)	3504 (2466 to 4792)	427 (301 to 583)	2.11 (2.09 to 2.14)
High-income	1272 (907 to 1749)	202 (143 to 281)	4264 (3012 to 5836)	439 (311 to 605)	2.53 (2.52 to 2.54)	116 (78.5 to 164)	21.8 (14.7 to 30.8)	232 (155 to 325)	31.5 (20.8 to 44.7)	1.17 (1.15 to 1.19)	1155 (820 to 1603)	181 (128 to 252)	4032 (2839 to 5544)	408 (289 to 563)	2.66 (2.65 to 2.67)
Latin America and Caribbean	585 (407 to 796)	458 (321 to 620)	2182 (1504 to 2973)	645 (445 to 877)	1.11 (1.09 to 1.14)	27.7 (17.8 to 40.2)	16.6 (10.9 to 24.1)	58.3 (37.6 to 86.0)	17.8 (11.5 to 26.2)	0.27 (0.24 to 0.29)	558 (389 to 760)	442 (310 to 600)	2124 (1466 to 2901)	627 (433 to 856)	1.15 (1.10 to 1.18)
North Africa and Middle East	276 (189 to 394)	298 (202 to 422)	1867 (1274 to 2635)	748 (507 to 1052)	3.01 (3.01 to 3.02)	20.4 (13.1 to 29.8)	16.0 (10.3 to 23.4)	51.0 (32.8 to 74.6)	17.7 (11.4 to 26.1)	0.33 (0.32 to 0.34)	255 (174 to 364)	282 (192 to 399)	1816 (1236 to 2564)	730 (494 to 1027)	3.12 (3.11 to 3.12)
Sub-Saharan Africa	261 (183 to 356)	211 (149 to 286)	1149 (793 to 1575)	375 (263 to 515)	1.87 (1.86 to 1.88)	22.7 (15.1 to 32.6)	12.3 (8.19 to 17.8)	54.4 (36.4 to 79.5)	12.2 (8.22 to 17.9)	-0.02 (-0.02 to -0.01)	238 (167 to 327)	198 (140 to 270)	1095 (758 to 1511)	363 (253 to 498)	1.96 (1.95 to 1.96)
**21 regions**															
East Asia	1185 (816 to 1677)	241 (167 to 341)	3882 (2655 to 5419)	374 (254 to 521)	1.43 (1.41 to 1.45)	24.8 (16.2 to 35.6)	4.23 (2.76 to 6.11)	45.6 (28.8 to 68.2)	5.16 (3.26 to 7.63)	0.72 (0.67 to 0.78)	1160 (799 to 1644)	237 (164 to 336)	3837 (2625 to 5360)	369 (250 to 514)	1.44 (1.42 to 1.46)
Oceania	7.86 (5.31 to 11.1)	442 (300 to 615)	43.3 (29.6 to 60.8)	940 (636 to 1310)	2.45 (2.44 to 2.47)	0.29 (0.19 to 0.41)	10.8 (7.27 to 15.5)	0.61 (0.40 to 0.86)	10.2 (6.63 to 14.5)	-0.18 (-0.19 to -0.16)	7.58 (5.12 to 10.7)	431 (292 to 600)	42.7 (29.2 to 60.1)	930 (629 to 1295)	2.50 (2.48 to 2.51)
Southeast Asia	366 (256 to 501)	250 (174 to 340)	1737 (1207 to 2387)	484 (336 to 660)	2.17 (2.13 to 2.20)	34.4 (21.7 to 51.7)	17.0 (10.8 to 25.3)	63.7 (40.6 to 95.8)	17.0 (10.8 to 25.6)	0.01 (-0.01 to 0.05)	332 (228 to 450)	233 (162 to 315)	1674 (1164 to 2298)	467 (325 to 636)	2.28 (2.25 to 2.32)
Central Asia	57.1 (39.2 to 81.2)	201 (138 to 287)	233 (160 to 328)	478 (328 to 671)	2.83 (2.82 to 2.84)	4.34 (2.82 to 6.33)	13.8 (9.01 to 20.3)	9.99 (6.63 to 14.6)	20.2 (13.4 to 29.4)	1.23 (1.22 to 1.24)	52.7 (35.8 to 75.0)	187 (127 to 266)	223 (153 to 315)	458 (314 to 643)	2.92 (2.91 to 2.94)
Central Europe	235 (161 to 325)	280 (193 to 387)	507 (350 to 696)	441 (306 to 607)	1.48 (1.45 to 1.50)	9.77 (6.55 to 14.1)	13.6 (8.98 to 19.7)	15.7 (10.4 to 22.4)	19.3 (12.5 to 28.4)	1.15 (1.14 to 1.16)	225 (154 to 312)	266 (183 to 369)	492 (339 to 679)	422 (292 to 583)	1.49 (1.47 to 1.52)
Eastern Europe	277 (193 to 380)	164 (115 to 224)	634 (443 to 870)	322 (225 to 444)	2.21 (2.21 to 2.22)	18.0 (11.9 to 25.7)	12.5 (8.17 to 17.9)	32.5 (21.6 to 46.5)	21.1 (13.8 to 30.5)	1.76 (1.73 to 1.78)	259 (181 to 353)	151 (106 to 206)	602 (418 to 827)	301 (211 to 414)	2.25 (2.24 to 2.26)
Australasia	21.0 (14.5 to 29.2)	170 (118 to 237)	70.5 (48.9 to 99.5)	284 (196 to 405)	1.68 (1.66 to 1.70)	2.94 (2.04 to 4.05)	26.0 (18.0 to 35.8)	7.57 (5.16 to 10.6)	37.8 (25.7 to 53.2)	1.21 (1.18 to 1.24)	18.0 (12.5 to 25.4)	144 (99.6 to 201)	62.9 (43.5 to 89.0)	246 (171 to 345)	1.77 (1.74 to 1.80)
High-income Asia Pacific	264 (185 to 371)	238 (167 to 335)	928 (652 to 1269)	490 (341 to 685)	2.35 (2.33 to 2.37)	12.3 (8.01 to 18.1)	12.8 (8.37 to 18.8)	19.9 (12.6 to 28.7)	16.1 (10.2 to 23.9)	0.74 (0.72 to 0.76)	252 (176 to 353)	225 (158 to 316)	908 (635 to 1241)	473 (330 to 662)	2.43 (2.40 to 2.45)
High-income North America	423 (300 to 582)	231 (163 to 318)	1757 (1236 to 2378)	559 (396 to 753)	2.91 (2.89 to 2.93)	48.2 (32.6 to 68.1)	29.6 (19.9 to 42.2)	90.8 (62.1 to 126)	37.5 (25.3 to 52.5)	0.75 (0.74 to 0.76)	374 (264 to 516)	201 (141 to 279)	1666 (1171 to 2262)	522 (368 to 706)	3.14 (3.12 to 3.17)
Southern Latin America	55.8 (37.6 to 76.8)	218 (148 to 300)	201 (135 to 283)	436 (294 to 614)	2.25 (2.22 to 2.26)	3.69 (2.43 to 5.29)	14.7 (9.69 to 21.1)	6.45 (4.16 to 9.46)	16.6 (10.7 to 24.2)	0.41 (0.39 to 0.42)	52.1 (35.0 to 72.0)	203 (137 to 281)	194 (130 to 275)	419 (282 to 589)	2.34 (2.31 to 2.36)
Western Europe	508 (362 to 713)	173 (121 to 244)	1308 (912 to 1840)	339 (233 to 479)	2.19 (2.18 to 2.21)	49.4 (33.1 to 69.4)	20.7 (13.8 to 29.5)	107 (71.1 to 151)	35.1 (22.8 to 50.3)	1.69 (1.63 to 1.73)	459 (325 to 640)	152 (106 to 214)	1201 (836 to 1693)	304 (210 to 430)	2.26 (2.25 to 2.27)
Andean Latin America	25.1 (17.0 to 35.2)	220 (148 to 308)	139 (93.9 to 192)	442 (298 to 609)	2.28 (2.27 to 2.29)	1.16 (0.75 to 1.69)	7.48 (4.86 to 10.8)	2.84 (1.83 to 4.08)	8.53 (5.52 to 12.2)	0.46 (0.44 to 0.48)	23.9 (16.1 to 33.7)	212 (142 to 299)	136 (91.9 to 188)	434 (292 to 598)	2.33 (2.31 to 2.34)
Caribbean	75.9 (51.8 to 105)	539 (367 to 747)	246 (168 to 347)	888 (606 to 1251)	1.61 (1.59 to 1.62)	4.05 (2.75 to 5.82)	24.4 (16.7 to 34.8)	5.67 (3.80 to 8.23)	22.2 (14.9 to 32.2)	-0.26 (-0.29 to -0.24)	71.8 (48.7 to 99.8)	515 (349 to 715)	241 (164 to 338)	866 (589 to 1219)	1.70 (1.68 to 1.71)
Central Latin America	280 (193 to 383)	567 (395 to 774)	1089 (746 to 1481)	787 (540 to 1069)	1.07 (1.03 to 1.10)	8.50 (5.53 to 12.6)	12.1 (7.91 to 17.8)	15.4 (9.76 to 22.6)	11.2 (7.10 to 16.5)	-0.22 (-0.25 to -0.19)	271 (188 to 373)	555 (387 to 758)	1074 (735 to 1463)	775 (532 to 1056)	1.09 (1.06 to 1.13)
Tropical Latin America	204 (141 to 276)	389 (271 to 525)	708 (485 to 965)	503 (344 to 685)	0.84 (0.82 to 0.85)	14.0 (8.73 to 20.8)	21.0 (13.1 to 31.1)	34.5 (22.1 to 50.7)	26.1 (16.7 to 38.8)	0.73 (0.71 to 0.74)	190 (132 to 258)	368 (256 to 497)	674 (465 to 922)	477 (329 to 652)	0.84 (0.83 to 0.85)
North Africa and Middle East	276 (189 to 394)	298 (202 to 422)	1867 (1274 to 2635)	748 (507 to 1052)	3.01 (3.01 to 3.02)	20.4 (13.1 to 29.8)	16.0 (10.3 to 23.4)	51.0 (32.8 to 74.6)	17.7 (11.4 to 26.1)	0.33 (0.32 to 0.34)	255 (174 to 364)	282 (192 to 399)	1816 (1236 to 2564)	730 (494 to 1027)	3.12 (3.11 to 3.12)
South Asia	757 (522 to 1039)	233 (163 to 319)	3627 (2554 to 4966)	440 (311 to 601)	2.04 (2.02 to 2.07)	52.9 (33.9 to 77.9)	12.1 (7.76 to 17.6)	122 (78.3 to 181)	13.6 (8.78 to 20.0)	0.36 (0.35 to 0.38)	704 (487 to 965)	221 (155 to 303)	3504 (2466 to 4792)	427 (301 to 583)	2.11 (2.09 to 2.14)
Central Sub-Saharan Africa	29.4 (20.0 to 41.7)	211 (145 to 301)	153 (103 to 218)	406 (277 to 566)	2.14 (2.14 to 2.15)	1.84 (1.20 to 2.69)	9.13 (6.08 to 13.3)	4.95 (3.19 to 7.28)	9.63 (6.37 to 14.1)	0.17 (0.16 to 0.18)	27.6 (18.8 to 39.2)	202 (139 to 287)	148 (99.4 to 211)	397 (271 to 552)	2.21 (2.20 to 2.22)
Eastern Sub-Saharan Africa	77.1 (54.1 to 104)	171 (122 to 228)	290 (202 to 401)	259 (179 to 352)	1.34 (1.33 to 1.35)	10.2 (6.88 to 14.8)	13.6 (9.14 to 19.3)	22.7 (15.2 to 32.9)	13.0 (8.67 to 18.4)	-0.14 (-0.15 to -0.12)	66.9 (46.6 to 90.1)	157 (111 to 209)	268 (186 to 369)	246 (170 to 336)	1.44 (1.43 to 1.45)
Southern Sub-Saharan Africa	47.8 (33.6 to 65.1)	289 (205 to 392)	199 (139 to 270)	566 (395 to 768)	2.19 (2.15 to 2.21)	3.12 (1.99 to 4.62)	14.8 (9.45 to 21.6)	5.90 (3.87 to 8.83)	15.0 (9.86 to 22.4)	0.05 (0.04 to 0.06)	44.7 (31.1 to 60.9)	274 (193 to 372)	193 (134 to 262)	551 (382 to 749)	2.27 (2.24 to 2.30)
Western Sub-Saharan Africa	106 (74.0 to 147)	217 (152 to 297)	508 (346 to 704)	399 (275 to 549)	1.99 (1.97 to 2.01)	7.45 (4.86 to 11.0)	11.0 (7.16 to 16.3)	20.8 (13.5 to 31.2)	11.4 (7.39 to 17.0)	0.11 (0.09 to 0.14)	99.0 (68.6 to 137)	206 (144 to 283)	487 (333 to 675)	388 (267 to 535)	2.07 (2.05 to 2.09)
**204 countries and territories**															
Afghanistan	16.3 (11.0 to 22.9)	458 (312 to 645)	86.4 (57.7 to 121)	1213 (820 to 1709)	3.20 (3.19 to 3.21)	0.91 (0.59 to 1.36)	21.7 (14.2 to 32.6)	2.77 (1.81 to 4.15)	23.7 (15.6 to 35.0)	0.31 (0.27 to 0.35)	15.4 (10.4 to 21.7)	436 (297 to 619)	83.7 (56.0 to 117)	1190 (805 to 1675)	3.29 (3.28 to 3.30)
Albania	2.20 (1.50 to 3.08)	199 (135 to 279)	6.93 (4.67 to 9.85)	318 (214 to 447)	1.52 (1.51 to 1.53)	0.14 (0.09 to 0.20)	9.98 (6.30 to 14.8)	0.27 (0.17 to 0.39)	15.6 (10.0 to 23.0)	1.45 (1.43 to 1.46)	2.06 (1.40 to 2.90)	189 (128 to 265)	6.66 (4.49 to 9.48)	302 (203 to 427)	1.52 (1.51 to 1.54)
Algeria	22.9 (15.4 to 32.6)	329 (221 to 466)	159 (107 to 219)	818 (550 to 1123)	2.98 (2.97 to 2.99)	1.26 (0.82 to 1.85)	12.9 (8.41 to 19.1)	3.17 (2.04 to 4.59)	14.8 (9.54 to 21.3)	0.46 (0.44 to 0.48)	21.7 (14.5 to 30.9)	316 (212 to 450)	156 (105 to 214)	804 (539 to 1102)	3.05 (3.04 to 3.06)
American Samoa	0.10 (0.07 to 0.15)	765 (521 to 1077)	0.44 (0.30 to 0.62)	1720 (1164 to 2438)	2.64 (2.62 to 2.65)	0.002 (1e-03 to 0.002)	7.92 (5.08 to 11.6)	0.002 (0.001 to 0.003)	8.70 (5.46 to 12.7)	0.29 (0.25 to 0.32)	0.10 (0.07 to 0.14)	757 (515 to 1067)	0.43 (0.29 to 0.61)	1711 (1158 to 2424)	2.65 (2.64 to 2.67)
Andorra	0.04 (0.03 to 0.06)	140 (96.1 to 196)	0.20 (0.13 to 0.28)	300 (200 to 429)	2.49 (2.48 to 2.50)	0.005 (0.003 to 0.008)	18.9 (11.9 to 28.2)	0.02 (0.01 to 0.03)	33.7 (21.4 to 49.4)	1.89 (1.86 to 1.91)	0.04 (0.02 to 0.05)	121 (83.6 to 170)	0.18 (0.12 to 0.26)	267 (179 to 383)	2.57 (2.56 to 2.58)
Angola	5.58 (3.78 to 7.93)	232 (158 to 324)	37.1 (25.0 to 51.9)	436 (295 to 601)	2.04 (2.03 to 2.06)	0.34 (0.22 to 0.49)	9.27 (6.05 to 13.4)	1.18 (0.78 to 1.76)	9.74 (6.51 to 14.1)	0.16 (0.14 to 0.17)	5.24 (3.57 to 7.47)	223 (151 to 312)	35.9 (24.2 to 50.3)	426 (289 to 589)	2.10 (2.08 to 2.12)
Antigua and Barbuda	0.18 (0.12 to 0.25)	633 (426 to 885)	0.59 (0.40 to 0.82)	1013 (686 to 1416)	1.51 (1.48 to 1.53)	0.010 (0.007 to 0.01)	32.9 (22.1 to 48.2)	0.01 (0.009 to 0.02)	25.7 (17.2 to 36.8)	-0.75 (-0.79 to -0.71)	0.17 (0.12 to 0.24)	600 (406 to 842)	0.57 (0.39 to 0.80)	988 (669 to 1380)	1.60 (1.57 to 1.62)
Argentina	40.1 (27.4 to 55.4)	225 (154 to 310)	123 (84.0 to 174)	416 (284 to 584)	2.00 (1.99 to 2.01)	2.71 (1.78 to 3.87)	16.1 (10.5 to 23.0)	4.37 (2.82 to 6.43)	17.0 (10.9 to 25.2)	0.22 (0.19 to 0.24)	37.4 (25.4 to 52.3)	209 (142 to 290)	119 (81.1 to 167)	399 (273 to 562)	2.11 (2.10 to 2.12)
Armenia	4.42 (3.01 to 6.33)	274 (187 to 392)	10.4 (6.89 to 14.8)	441 (296 to 631)	1.54 (1.52 to 1.57)	0.27 (0.18 to 0.40)	16.0 (10.5 to 23.5)	0.40 (0.26 to 0.58)	20.6 (13.3 to 30.6)	0.84 (0.81 to 0.87)	4.14 (2.81 to 5.99)	258 (175 to 372)	9.98 (6.59 to 14.4)	420 (281 to 602)	1.58 (1.55 to 1.61)
Australia	16.5 (11.3 to 23.3)	160 (110 to 225)	57.5 (39.5 to 81.8)	275 (189 to 393)	1.77 (1.75 to 1.80)	2.51 (1.72 to 3.43)	26.6 (18.3 to 36.5)	6.82 (4.63 to 9.54)	40.8 (27.7 to 57.4)	1.37 (1.35 to 1.40)	14.0 (9.50 to 19.7)	133 (90.7 to 188)	50.7 (34.6 to 72.6)	234 (161 to 334)	1.85 (1.82 to 1.87)
Austria	7.12 (4.82 to 9.83)	112 (76.8 to 155)	18.9 (12.9 to 26.8)	239 (163 to 342)	2.47 (2.46 to 2.49)	0.94 (0.63 to 1.36)	18.8 (12.3 to 27.2)	2.27 (1.54 to 3.22)	36.1 (24.4 to 52.0)	2.13 (2.11 to 2.14)	6.17 (4.19 to 8.61)	93.3 (63.1 to 131)	16.7 (11.3 to 23.7)	203 (137 to 291)	2.54 (2.52 to 2.55)
Azerbaijan	6.21 (4.23 to 8.91)	202 (137 to 288)	31.5 (21.6 to 44.3)	513 (353 to 719)	3.05 (3.03 to 3.07)	0.51 (0.33 to 0.73)	14.8 (9.57 to 21.4)	1.25 (0.81 to 1.86)	21.1 (13.8 to 31.3)	1.14 (1.13 to 1.15)	5.70 (3.86 to 8.15)	187 (126 to 266)	30.2 (20.8 to 42.7)	492 (339 to 693)	3.16 (3.15 to 3.18)
Bahamas	0.53 (0.35 to 0.73)	564 (375 to 771)	2.15 (1.44 to 3.06)	920 (615 to 1302)	1.58 (1.57 to 1.59)	0.03 (0.02 to 0.05)	25.8 (17.3 to 37.5)	0.05 (0.03 to 0.07)	20.9 (14.1 to 30.5)	-0.63 (-0.67 to -0.59)	0.50 (0.33 to 0.69)	538 (358 to 740)	2.10 (1.41 to 3.00)	899 (601 to 1272)	1.67 (1.65 to 1.68)
Bahrain	0.53 (0.36 to 0.74)	576 (394 to 790)	5.53 (3.79 to 7.74)	1233 (834 to 1701)	2.48 (2.47 to 2.50)	0.03 (0.02 to 0.05)	18.7 (12.2 to 27.3)	0.11 (0.07 to 0.17)	20.2 (13.2 to 29.7)	0.25 (0.23 to 0.27)	0.50 (0.34 to 0.70)	557 (379 to 766)	5.42 (3.71 to 7.61)	1213 (820 to 1680)	2.54 (2.52 to 2.55)
Bangladesh	64.5 (43.6 to 89.5)	241 (165 to 333)	409 (283 to 570)	545 (375 to 759)	2.67 (2.65 to 2.69)	6.86 (4.47 to 9.93)	16.5 (10.8 to 23.6)	14.9 (9.58 to 21.7)	18.0 (11.5 to 26.1)	0.33 (0.31 to 0.35)	57.7 (39.1 to 80.2)	224 (154 to 310)	394 (272 to 548)	527 (361 to 732)	2.79 (2.77 to 2.81)
Barbados	1.00 (0.68 to 1.38)	637 (430 to 889)	2.50 (1.67 to 3.45)	974 (651 to 1343)	1.38 (1.34 to 1.41)	0.05 (0.03 to 0.07)	34.3 (23.0 to 49.8)	0.05 (0.03 to 0.08)	27.6 (18.1 to 40.3)	-0.70 (-0.73 to -0.67)	0.95 (0.64 to 1.32)	602 (404 to 837)	2.45 (1.64 to 3.38)	946 (629 to 1310)	1.46 (1.42 to 1.49)
Belarus	10.6 (7.05 to 14.9)	138 (92.2 to 194)	20.2 (13.5 to 28.4)	227 (153 to 319)	1.61 (1.59 to 1.63)	0.65 (0.41 to 0.94)	9.97 (6.36 to 14.4)	1.00 (0.63 to 1.47)	14.4 (9.08 to 21.4)	1.20 (1.18 to 1.22)	9.92 (6.63 to 14.1)	128 (85.6 to 181)	19.2 (12.8 to 27.1)	212 (143 to 300)	1.66 (1.64 to 1.68)
Belgium	15.0 (10.1 to 20.9)	194 (131 to 273)	36.1 (24.5 to 51.2)	368 (255 to 527)	2.08 (2.05 to 2.11)	1.20 (0.77 to 1.76)	19.2 (12.3 to 28.1)	2.25 (1.42 to 3.27)	28.9 (17.9 to 42.6)	1.33 (1.30 to 1.34)	13.8 (9.28 to 19.3)	175 (117 to 247)	33.8 (22.9 to 47.8)	339 (232 to 485)	2.15 (2.12 to 2.18)
Belize	0.26 (0.18 to 0.36)	526 (358 to 728)	1.57 (1.05 to 2.22)	920 (620 to 1301)	1.80 (1.77 to 1.83)	0.02 (0.01 to 0.03)	26.8 (18.0 to 37.8)	0.05 (0.03 to 0.07)	21.8 (14.4 to 31.4)	-0.60 (-0.65 to -0.53)	0.24 (0.16 to 0.33)	499 (337 to 692)	1.52 (1.01 to 2.15)	898 (604 to 1272)	1.89 (1.85 to 1.92)
Benin	3.09 (2.08 to 4.32)	257 (172 to 361)	20.1 (13.6 to 28.3)	580 (394 to 815)	2.66 (2.61 to 2.70)	0.20 (0.12 to 0.29)	11.4 (7.30 to 17.0)	0.55 (0.36 to 0.83)	11.2 (7.32 to 16.7)	-0.03 (-0.05 to -0.01)	2.90 (1.93 to 4.08)	246 (165 to 347)	19.6 (13.2 to 27.5)	569 (386 to 800)	2.74 (2.69 to 2.79)
Bermuda	0.12 (0.08 to 0.17)	332 (225 to 470)	0.34 (0.23 to 0.49)	552 (378 to 790)	1.65 (1.64 to 1.66)	0.007 (0.004 to 0.010)	19.2 (12.7 to 28.1)	0.008 (0.005 to 0.01)	17.4 (11.3 to 25.5)	-0.27 (-0.30 to -0.23)	0.11 (0.07 to 0.16)	312 (211 to 445)	0.33 (0.23 to 0.48)	534 (365 to 767)	1.74 (1.73 to 1.75)
Bhutan	0.33 (0.22 to 0.47)	216 (147 to 307)	1.29 (0.88 to 1.82)	397 (271 to 558)	1.98 (1.96 to 2.01)	0.02 (0.02 to 0.04)	10.9 (7.09 to 15.5)	0.04 (0.03 to 0.06)	12.1 (7.80 to 17.7)	0.33 (0.32 to 0.34)	0.30 (0.20 to 0.44)	205 (140 to 291)	1.24 (0.85 to 1.75)	385 (263 to 542)	2.05 (2.03 to 2.08)
Bolivia (Plurinational State of)	4.97 (3.33 to 7.02)	268 (180 to 376)	26.7 (17.9 to 37.9)	529 (355 to 749)	2.20 (2.18 to 2.22)	0.28 (0.18 to 0.40)	10.7 (7.03 to 15.6)	0.63 (0.40 to 0.92)	10.9 (7.10 to 16.0)	0.10 (0.08 to 0.13)	4.70 (3.15 to 6.59)	258 (173 to 360)	26.1 (17.5 to 37.1)	518 (347 to 734)	2.27 (2.25 to 2.28)
Bosnia and Herzegovina	6.90 (4.60 to 9.56)	291 (195 to 400)	20.8 (13.9 to 28.3)	627 (422 to 863)	2.51 (2.50 to 2.52)	0.37 (0.25 to 0.54)	15.5 (10.2 to 22.4)	0.47 (0.31 to 0.66)	20.8 (13.6 to 30.1)	0.94 (0.93 to 0.96)	6.52 (4.35 to 9.01)	276 (185 to 380)	20.3 (13.6 to 27.7)	606 (406 to 836)	2.58 (2.57 to 2.59)
Botswana	0.75 (0.51 to 1.07)	222 (149 to 312)	4.22 (2.88 to 5.89)	462 (315 to 644)	2.39 (2.36 to 2.40)	0.08 (0.05 to 0.12)	16.8 (11.0 to 24.5)	0.19 (0.12 to 0.28)	17.0 (11.2 to 24.7)	0.07 (0.04 to 0.10)	0.67 (0.45 to 0.95)	205 (138 to 289)	4.03 (2.73 to 5.63)	445 (303 to 622)	2.52 (2.49 to 2.54)
Brazil	200 (138 to 270)	390 (272 to 527)	688 (472 to 939)	500 (343 to 682)	0.81 (0.80 to 0.82)	13.7 (8.58 to 20.5)	21.1 (13.2 to 31.3)	33.9 (21.6 to 49.9)	26.4 (16.9 to 39.1)	0.73 (0.72 to 0.75)	186 (129 to 253)	369 (256 to 498)	655 (452 to 896)	474 (327 to 648)	0.81 (0.80 to 0.82)
Brunei Darussalam	0.28 (0.19 to 0.39)	491 (333 to 669)	2.02 (1.37 to 2.83)	989 (673 to 1388)	2.27 (2.26 to 2.29)	0.04 (0.02 to 0.05)	32.9 (21.8 to 46.8)	0.07 (0.05 to 0.10)	30.4 (20.0 to 44.1)	-0.22 (-0.25 to -0.19)	0.25 (0.17 to 0.34)	458 (312 to 627)	1.95 (1.33 to 2.73)	958 (650 to 1342)	2.40 (2.38 to 2.41)
Bulgaria	18.6 (12.2 to 26.3)	281 (187 to 397)	36.1 (24.4 to 49.8)	491 (333 to 683)	1.82 (1.80 to 1.84)	1.06 (0.70 to 1.56)	20.1 (13.3 to 29.7)	1.37 (0.92 to 1.95)	27.9 (18.5 to 40.2)	1.07 (1.05 to 1.09)	17.5 (11.4 to 24.8)	261 (172 to 366)	34.7 (23.4 to 47.6)	463 (311 to 643)	1.87 (1.85 to 1.89)
Burkina Faso	4.20 (2.84 to 5.82)	166 (114 to 230)	22.3 (15.3 to 31.2)	376 (260 to 530)	2.69 (2.67 to 2.70)	0.39 (0.25 to 0.55)	11.0 (7.14 to 15.8)	0.90 (0.56 to 1.33)	10.7 (6.79 to 16.0)	-0.12 (-0.14 to -0.10)	3.81 (2.57 to 5.31)	155 (106 to 215)	21.4 (14.7 to 30.0)	365 (252 to 516)	2.82 (2.80 to 2.83)
Burundi	2.50 (1.71 to 3.46)	171 (118 to 235)	8.00 (5.35 to 11.2)	260 (174 to 362)	1.36 (1.34 to 1.36)	0.31 (0.21 to 0.45)	13.9 (9.33 to 19.7)	0.66 (0.42 to 0.96)	12.7 (8.24 to 18.4)	-0.23 (-0.27 to -0.19)	2.18 (1.50 to 3.06)	157 (108 to 218)	7.34 (4.88 to 10.3)	247 (164 to 344)	1.47 (1.45 to 1.47)
Cabo Verde	0.35 (0.24 to 0.49)	264 (179 to 370)	1.55 (1.04 to 2.13)	596 (398 to 816)	2.67 (2.66 to 2.68)	0.01 (0.010 to 0.02)	10.3 (6.66 to 14.9)	0.03 (0.02 to 0.05)	11.0 (7.08 to 16.3)	0.18 (0.16 to 0.20)	0.33 (0.23 to 0.47)	253 (173 to 355)	1.52 (1.02 to 2.09)	585 (391 to 801)	2.74 (2.73 to 2.75)
Cambodia	5.13 (3.53 to 7.17)	178 (123 to 246)	34.2 (23.4 to 47.7)	460 (316 to 641)	3.12 (3.10 to 3.15)	0.59 (0.39 to 0.89)	13.8 (9.13 to 20.6)	1.16 (0.74 to 1.74)	13.5 (8.65 to 20.2)	-0.02 (-0.06 to 0.01)	4.54 (3.07 to 6.33)	164 (112 to 226)	33.0 (22.6 to 46.3)	447 (307 to 624)	3.29 (3.27 to 3.31)
Cameroon	5.29 (3.61 to 7.49)	201 (138 to 282)	35.0 (23.5 to 48.6)	430 (288 to 591)	2.49 (2.45 to 2.52)	0.45 (0.28 to 0.66)	11.8 (7.63 to 17.3)	1.43 (0.91 to 2.12)	12.1 (7.73 to 17.4)	0.07 (0.06 to 0.08)	4.84 (3.32 to 6.89)	189 (130 to 266)	33.5 (22.6 to 46.8)	418 (280 to 575)	2.60 (2.56 to 2.63)
Canada	25.5 (17.6 to 35.4)	147 (101 to 205)	136 (91.5 to 189)	405 (275 to 567)	3.31 (3.29 to 3.33)	7.98 (5.34 to 11.1)	51.0 (33.8 to 71.1)	22.0 (15.2 to 29.8)	87.2 (59.3 to 121)	1.75 (1.74 to 1.76)	17.6 (11.9 to 24.6)	96.0 (65.1 to 134)	114 (75.7 to 161)	318 (212 to 450)	3.92 (3.90 to 3.95)
Central African Republic	2.16 (1.45 to 3.02)	298 (202 to 416)	9.28 (6.24 to 13.4)	582 (395 to 831)	2.16 (2.13 to 2.18)	0.11 (0.07 to 0.17)	10.7 (6.93 to 16.3)	0.24 (0.15 to 0.35)	10.7 (6.93 to 15.4)	0.02 (0.01 to 0.04)	2.05 (1.37 to 2.87)	287 (195 to 403)	9.04 (6.06 to 13.0)	572 (388 to 817)	2.21 (2.18 to 2.24)
Chad	3.38 (2.28 to 4.74)	206 (140 to 289)	15.6 (10.4 to 22.0)	442 (297 to 623)	2.48 (2.48 to 2.49)	0.24 (0.15 to 0.35)	10.9 (7.05 to 15.8)	0.63 (0.39 to 0.95)	10.9 (6.83 to 16.3)	0.01 (-0.01 to 0.03)	3.14 (2.11 to 4.43)	196 (133 to 275)	15.0 (9.99 to 21.1)	431 (290 to 608)	2.58 (2.57 to 2.59)
Chile	12.3 (8.16 to 16.9)	217 (144 to 299)	66.6 (45.4 to 93.9)	488 (332 to 688)	2.58 (2.50 to 2.63)	0.66 (0.42 to 0.99)	10.1 (6.48 to 15.1)	1.60 (1.02 to 2.35)	14.4 (9.21 to 21.4)	1.14 (1.12 to 1.15)	11.6 (7.67 to 16.1)	207 (137 to 286)	65.0 (44.2 to 91.8)	474 (322 to 668)	2.64 (2.55 to 2.68)

Location	DM, Number in 1990 (000s)	DM, ASR in 1990 (per 100,000)	DM, Number in 2021 (000s)	DM, ASR in 2021 (per 100,000)	DM, AAPC (95%CI)	T1D, Number in 1990 (000s)	T1D, ASR in 1990 (per 100,000)	T1D, Number in 2021 (000s)	T1D, ASR in 2021 (per 100,000)	T1D, AAPC (95%CI)	T2D, Number in 1990 (000s)	T2D, ASR in 1990 (per 100,000)	T2D, Number in 2021 (000s)	T2D, ASR in 2021 (per 100,000)	T2D, AAPC (95%CI)
China	1135 (783 to 1607)	240 (166 to 339)	3699 (2528 to 5167)	370 (251 to 516)	1.42 (1.39 to 1.44)	22.8 (14.8 to 32.9)	4.01 (2.61 to 5.79)	41.9 (26.2 to 62.4)	4.94 (3.09 to 7.33)	0.74 (0.69 to 0.80)	1112 (767 to 1577)	236 (163 to 334)	3657 (2500 to 5112)	365 (248 to 510)	1.43 (1.41 to 1.45)
Colombia	40.0 (26.9 to 57.6)	384 (258 to 556)	165 (112 to 230)	555 (378 to 772)	1.24 (1.15 to 1.35)	0.91 (0.58 to 1.37)	6.46 (4.22 to 9.53)	1.83 (1.15 to 2.73)	6.61 (4.15 to 9.78)	0.09 (0.07 to 0.10)	39.1 (26.2 to 56.5)	378 (254 to 546)	163 (111 to 228)	548 (373 to 764)	1.26 (1.16 to 1.37)
Comoros	0.25 (0.17 to 0.35)	217 (149 to 298)	1.13 (0.77 to 1.58)	384 (265 to 533)	1.85 (1.83 to 1.86)	0.03 (0.02 to 0.04)	13.7 (9.29 to 19.8)	0.05 (0.03 to 0.07)	13.7 (8.98 to 19.3)	0.01 (-0.01 to 0.03)	0.22 (0.15 to 0.31)	203 (140 to 279)	1.08 (0.74 to 1.51)	371 (255 to 516)	1.95 (1.93 to 1.96)
Congo	1.53 (1.05 to 2.12)	231 (159 to 323)	7.95 (5.40 to 11.3)	457 (311 to 646)	2.21 (2.18 to 2.24)	0.11 (0.07 to 0.16)	11.9 (8.00 to 17.1)	0.28 (0.18 to 0.42)	12.2 (7.93 to 18.0)	0.11 (0.08 to 0.14)	1.42 (0.98 to 1.96)	219 (150 to 306)	7.67 (5.20 to 10.9)	445 (302 to 631)	2.30 (2.27 to 2.33)
Cook Islands	0.06 (0.04 to 0.08)	859 (584 to 1210)	0.19 (0.13 to 0.27)	1554 (1068 to 2180)	1.93 (1.91 to 1.94)	1e-03 (6e-04 to 0.001)	12.2 (8.02 to 17.8)	0.001 (8e-04 to 0.002)	11.6 (7.64 to 16.6)	-0.16 (-0.18 to -0.15)	0.06 (0.04 to 0.08)	847 (576 to 1195)	0.19 (0.13 to 0.27)	1542 (1059 to 2165)	1.95 (1.93 to 1.96)
Costa Rica	3.82 (2.62 to 5.43)	386 (262 to 544)	20.0 (13.6 to 28.8)	682 (462 to 981)	1.87 (1.80 to 1.95)	0.08 (0.05 to 0.13)	6.36 (4.13 to 9.51)	0.17 (0.11 to 0.26)	6.34 (3.99 to 9.44)	-0.01 (-0.03 to 0.01)	3.74 (2.55 to 5.31)	379 (258 to 535)	19.9 (13.4 to 28.6)	676 (458 to 973)	1.90 (1.83 to 1.97)
Croatia	9.85 (6.70 to 13.8)	275 (186 to 385)	20.1 (13.3 to 28.3)	444 (299 to 626)	1.56 (1.53 to 1.58)	0.43 (0.28 to 0.61)	14.1 (9.42 to 20.2)	0.81 (0.52 to 1.13)	25.9 (16.3 to 36.3)	1.96 (1.94 to 1.99)	9.43 (6.39 to 13.2)	261 (176 to 365)	19.3 (12.8 to 27.3)	418 (281 to 592)	1.54 (1.51 to 1.56)
Cuba	21.3 (14.7 to 30.5)	404 (278 to 577)	57.9 (39.1 to 81.2)	625 (424 to 869)	1.41 (1.38 to 1.44)	0.80 (0.53 to 1.18)	14.3 (9.59 to 21.2)	0.76 (0.48 to 1.09)	10.5 (6.67 to 15.2)	-0.94 (-0.99 to -0.89)	20.5 (14.1 to 29.5)	389 (268 to 558)	57.2 (38.7 to 80.2)	615 (417 to 856)	1.46 (1.43 to 1.49)
Cyprus	1.45 (1.01 to 2.06)	316 (220 to 443)	4.12 (2.78 to 5.80)	401 (270 to 563)	0.77 (0.76 to 0.78)	0.07 (0.05 to 0.10)	18.1 (12.2 to 25.6)	0.38 (0.25 to 0.55)	44.2 (29.5 to 63.1)	2.93 (2.90 to 2.97)	1.38 (0.95 to 1.97)	298 (206 to 420)	3.73 (2.53 to 5.27)	356 (241 to 502)	0.58 (0.57 to 0.59)
Czechia	20.8 (14.0 to 29.1)	268 (180 to 377)	51.5 (34.6 to 72.6)	477 (323 to 669)	1.85 (1.83 to 1.88)	0.96 (0.64 to 1.37)	15.3 (10.2 to 22.1)	1.76 (1.14 to 2.52)	22.9 (15.1 to 33.3)	1.32 (1.30 to 1.34)	19.9 (13.2 to 27.8)	253 (169 to 356)	49.7 (33.4 to 70.1)	454 (306 to 641)	1.88 (1.85 to 1.91)
Côte d'Ivoire	5.48 (3.69 to 7.64)	226 (155 to 313)	33.0 (22.1 to 46.8)	484 (324 to 679)	2.49 (2.48 to 2.51)	0.45 (0.28 to 0.69)	11.3 (7.12 to 16.9)	1.19 (0.77 to 1.75)	11.8 (7.65 to 17.2)	0.15 (0.13 to 0.16)	5.03 (3.35 to 7.00)	215 (145 to 297)	31.8 (21.2 to 45.1)	472 (314 to 661)	2.58 (2.57 to 2.59)
Democratic People's Republic of Korea	22.1 (14.7 to 31.2)	213 (144 to 302)	75.1 (51.3 to 106)	413 (281 to 579)	2.15 (2.14 to 2.16)	0.64 (0.41 to 0.93)	5.81 (3.75 to 8.44)	1.00 (0.65 to 1.48)	6.49 (4.30 to 9.58)	0.36 (0.34 to 0.38)	21.4 (14.3 to 30.4)	208 (140 to 295)	74.1 (50.6 to 105)	406 (277 to 572)	2.19 (2.18 to 2.20)
Democratic Republic of the Congo	19.1 (12.9 to 27.1)	196 (134 to 277)	93.4 (62.7 to 134)	378 (259 to 531)	2.15 (2.14 to 2.15)	1.22 (0.79 to 1.82)	8.73 (5.70 to 12.9)	3.10 (2.00 to 4.59)	9.27 (6.08 to 13.7)	0.20 (0.18 to 0.21)	17.9 (12.1 to 25.4)	187 (129 to 265)	90.3 (60.6 to 130)	369 (252 to 519)	2.22 (2.21 to 2.22)
Denmark	3.80 (2.64 to 5.21)	102 (69.0 to 142)	10.9 (7.63 to 15.2)	224 (155 to 315)	2.59 (2.57 to 2.60)	0.64 (0.41 to 0.91)	20.4 (13.4 to 29.3)	1.09 (0.68 to 1.58)	28.8 (17.6 to 42.6)	1.13 (1.11 to 1.16)	3.16 (2.19 to 4.30)	81.2 (55.4 to 112)	9.82 (6.77 to 13.8)	195 (132 to 273)	2.87 (2.85 to 2.88)
Djibouti	0.13 (0.09 to 0.18)	149 (102 to 204)	0.99 (0.67 to 1.39)	267 (183 to 378)	1.90 (1.89 to 1.91)	0.02 (0.01 to 0.03)	13.7 (9.01 to 19.8)	0.07 (0.04 to 0.10)	13.0 (8.12 to 18.8)	-0.16 (-0.20 to -0.12)	0.11 (0.07 to 0.15)	135 (93.0 to 184)	0.92 (0.63 to 1.31)	254 (174 to 359)	2.05 (2.04 to 2.06)
Dominica	0.23 (0.16 to 0.32)	682 (461 to 936)	0.46 (0.31 to 0.65)	1091 (738 to 1540)	1.51 (1.50 to 1.53)	0.009 (0.006 to 0.01)	27.3 (18.1 to 39.5)	0.008 (0.006 to 0.01)	22.6 (15.1 to 32.4)	-0.56 (-0.60 to -0.49)	0.22 (0.15 to 0.31)	655 (440 to 903)	0.45 (0.30 to 0.64)	1069 (721 to 1512)	1.58 (1.56 to 1.59)
Dominican Republic	9.11 (6.17 to 12.9)	420 (285 to 596)	44.6 (30.7 to 63.2)	838 (575 to 1187)	2.23 (2.20 to 2.25)	0.59 (0.39 to 0.88)	19.2 (12.9 to 28.0)	1.04 (0.69 to 1.55)	18.7 (12.5 to 28.1)	-0.08 (-0.10 to -0.07)	8.52 (5.75 to 12.3)	401 (272 to 575)	43.5 (29.9 to 61.8)	819 (562 to 1161)	2.31 (2.28 to 2.33)
Ecuador	8.39 (5.57 to 11.8)	287 (190 to 405)	56.7 (38.8 to 78.3)	649 (444 to 897)	2.68 (2.66 to 2.69)	0.32 (0.21 to 0.48)	7.94 (5.06 to 11.9)	0.79 (0.51 to 1.11)	8.70 (5.63 to 12.3)	0.32 (0.29 to 0.35)	8.07 (5.30 to 11.4)	279 (183 to 396)	55.9 (38.2 to 77.3)	640 (438 to 886)	2.72 (2.71 to 2.74)
Egypt	29.5 (20.0 to 42.4)	192 (131 to 271)	243 (160 to 346)	702 (473 to 995)	4.28 (4.27 to 4.31)	3.44 (2.23 to 5.03)	16.5 (10.8 to 24.4)	7.90 (5.03 to 11.9)	17.6 (11.3 to 26.2)	0.17 (0.13 to 0.20)	26.1 (17.9 to 37.5)	175 (120 to 247)	235 (155 to 334)	684 (463 to 972)	4.50 (4.49 to 4.52)
El Salvador	4.89 (3.34 to 6.87)	281 (193 to 396)	21.4 (14.6 to 29.6)	608 (414 to 839)	2.51 (2.46 to 2.57)	0.21 (0.13 to 0.30)	9.19 (5.94 to 13.4)	0.36 (0.24 to 0.52)	10.2 (6.76 to 14.9)	0.37 (0.35 to 0.39)	4.68 (3.21 to 6.58)	272 (186 to 383)	21.1 (14.3 to 29.1)	598 (407 to 825)	2.57 (2.51 to 2.63)
Equatorial Guinea	0.27 (0.19 to 0.39)	225 (156 to 318)	1.91 (1.28 to 2.71)	511 (348 to 720)	2.66 (2.63 to 2.68)	0.01 (0.009 to 0.02)	9.04 (5.74 to 13.2)	0.06 (0.04 to 0.08)	10.7 (7.07 to 15.6)	0.55 (0.54 to 0.57)	0.26 (0.18 to 0.37)	216 (150 to 305)	1.85 (1.25 to 2.62)	500 (341 to 704)	2.72 (2.70 to 2.75)
Eritrea	1.39 (0.94 to 1.93)	179 (122 to 248)	6.59 (4.44 to 9.08)	336 (230 to 456)	2.04 (2.02 to 2.05)	0.20 (0.13 to 0.28)	14.4 (9.68 to 20.6)	0.41 (0.27 to 0.59)	14.4 (9.43 to 20.8)	0.03 (0.01 to 0.06)	1.19 (0.81 to 1.66)	164 (113 to 227)	6.19 (4.19 to 8.51)	322 (221 to 438)	2.16 (2.15 to 2.18)
Estonia	2.06 (1.39 to 2.92)	170 (115 to 239)	4.66 (3.20 to 6.60)	349 (241 to 499)	2.35 (2.32 to 2.37)	0.17 (0.11 to 0.23)	17.1 (11.4 to 23.9)	0.26 (0.17 to 0.38)	26.1 (17.2 to 38.3)	1.42 (1.39 to 1.45)	1.89 (1.28 to 2.69)	153 (103 to 217)	4.40 (3.02 to 6.28)	323 (222 to 464)	2.43 (2.39 to 2.46)
Eswatini	0.52 (0.35 to 0.74)	283 (191 to 404)	2.28 (1.57 to 3.19)	625 (433 to 873)	2.57 (2.54 to 2.60)	0.06 (0.03 to 0.08)	19.5 (12.2 to 28.4)	0.10 (0.06 to 0.14)	20.0 (13.1 to 29.8)	0.09 (0.07 to 0.10)	0.46 (0.31 to 0.67)	264 (176 to 378)	2.19 (1.50 to 3.06)	605 (418 to 849)	2.70 (2.68 to 2.72)
Ethiopia	28.2 (19.7 to 38.1)	242 (169 to 324)	86.2 (59.2 to 117)	308 (214 to 419)	0.78 (0.77 to 0.79)	2.99 (2.01 to 4.30)	14.8 (10.1 to 20.8)	5.60 (3.74 to 8.11)	12.8 (8.60 to 18.1)	-0.38 (-0.43 to -0.33)	25.3 (17.5 to 34.2)	227 (158 to 302)	80.6 (54.6 to 110)	295 (205 to 402)	0.85 (0.83 to 0.86)
Fiji	1.50 (1.00 to 2.10)	679 (452 to 956)	5.94 (3.94 to 8.27)	1351 (903 to 1878)	2.24 (2.23 to 2.25)	0.07 (0.05 to 0.10)	21.0 (14.0 to 30.5)	0.08 (0.05 to 0.11)	17.4 (11.7 to 24.9)	-0.57 (-0.64 to -0.48)	1.42 (0.95 to 2.00)	658 (438 to 926)	5.86 (3.89 to 8.16)	1334 (892 to 1853)	2.30 (2.29 to 2.31)
Finland	8.34 (5.59 to 11.9)	226 (152 to 319)	21.1 (14.1 to 29.3)	434 (291 to 609)	2.12 (2.12 to 2.13)	1.74 (1.20 to 2.44)	55.8 (38.3 to 78.7)	3.15 (2.14 to 4.36)	83.2 (56.5 to 115)	1.31 (1.29 to 1.33)	6.60 (4.47 to 9.37)	171 (115 to 244)	18.0 (12.1 to 25.1)	351 (233 to 495)	2.36 (2.35 to 2.38)
France	42.1 (29.2 to 58.8)	98.8 (68.3 to 138)	106 (73.6 to 149)	186 (126 to 261)	2.06 (2.03 to 2.08)	4.29 (2.91 to 6.11)	12.6 (8.45 to 18.2)	10.4 (6.97 to 15.0)	24.0 (15.9 to 35.0)	2.11 (2.09 to 2.14)	37.8 (26.4 to 52.9)	86.2 (59.9 to 122)	95.8 (66.0 to 134)	162 (110 to 230)	2.06 (2.03 to 2.09)
Gabon	0.80 (0.55 to 1.12)	247 (169 to 347)	3.22 (2.21 to 4.51)	504 (347 to 703)	2.33 (2.32 to 2.34)	0.04 (0.03 to 0.06)	11.0 (7.08 to 16.1)	0.09 (0.06 to 0.13)	11.4 (7.69 to 16.6)	0.14 (0.12 to 0.16)	0.76 (0.52 to 1.06)	236 (162 to 332)	3.12 (2.15 to 4.38)	493 (339 to 690)	2.41 (2.40 to 2.42)
Gambia	0.43 (0.29 to 0.59)	207 (141 to 289)	2.79 (1.90 to 3.95)	454 (312 to 646)	2.58 (2.57 to 2.58)	0.04 (0.02 to 0.05)	11.2 (7.27 to 16.7)	0.10 (0.07 to 0.15)	11.5 (7.53 to 17.1)	0.10 (0.09 to 0.12)	0.39 (0.26 to 0.55)	195 (134 to 275)	2.69 (1.83 to 3.83)	443 (305 to 630)	2.68 (2.67 to 2.68)
Georgia	7.07 (4.82 to 9.95)	195 (133 to 275)	14.5 (9.78 to 20.4)	472 (319 to 663)	2.88 (2.85 to 2.90)	0.47 (0.31 to 0.70)	14.5 (9.76 to 21.6)	0.50 (0.33 to 0.72)	20.9 (13.8 to 30.1)	1.16 (1.14 to 1.18)	6.60 (4.45 to 9.32)	181 (122 to 256)	14.0 (9.40 to 19.7)	451 (303 to 631)	2.98 (2.96 to 3.01)
Germany	75.9 (52.9 to 106)	114 (78.6 to 161)	222 (152 to 309)	278 (190 to 388)	2.97 (2.94 to 3.00)	8.23 (5.55 to 11.5)	16.3 (10.8 to 22.8)	16.9 (11.2 to 23.9)	29.2 (19.2 to 41.4)	1.90 (1.88 to 1.92)	67.7 (46.7 to 93.6)	97.4 (67.0 to 136)	205 (140 to 286)	249 (170 to 347)	3.12 (3.08 to 3.16)
Ghana	8.70 (5.85 to 12.2)	225 (153 to 317)	49.9 (33.4 to 70.5)	446 (300 to 635)	2.23 (2.21 to 2.25)	0.58 (0.39 to 0.87)	10.6 (7.16 to 15.8)	1.60 (1.03 to 2.36)	11.0 (7.17 to 15.9)	0.11 (0.10 to 0.13)	8.12 (5.47 to 11.5)	215 (146 to 302)	48.3 (32.2 to 68.2)	435 (292 to 619)	2.31 (2.29 to 2.32)
Greece	16.3 (11.0 to 22.8)	217 (146 to 303)	34.3 (22.9 to 48.7)	365 (246 to 516)	1.68 (1.66 to 1.71)	0.82 (0.55 to 1.19)	13.2 (8.76 to 18.6)	1.97 (1.28 to 2.90)	26.3 (16.4 to 38.7)	2.23 (2.20 to 2.26)	15.5 (10.4 to 21.8)	204 (137 to 286)	32.4 (21.5 to 46.4)	339 (227 to 484)	1.65 (1.62 to 1.67)
Greenland	0.01 (0.009 to 0.02)	71.4 (49.3 to 101)	0.07 (0.05 to 0.10)	224 (152 to 317)	3.75 (3.73 to 3.77)	0.005 (0.003 to 0.008)	22.4 (14.3 to 32.0)	0.008 (0.005 to 0.01)	25.0 (16.0 to 37.3)	0.34 (0.32 to 0.36)	0.008 (0.006 to 0.01)	49.1 (33.1 to 70.3)	0.07 (0.04 to 0.09)	199 (136 to 279)	4.62 (4.59 to 4.63)
Grenada	0.27 (0.18 to 0.37)	698 (473 to 981)	0.68 (0.45 to 0.95)	1131 (761 to 1578)	1.54 (1.50 to 1.56)	0.010 (0.007 to 0.01)	25.1 (17.1 to 35.2)	0.01 (0.007 to 0.02)	19.9 (13.2 to 28.5)	-0.70 (-0.75 to -0.67)	0.26 (0.17 to 0.36)	673 (455 to 946)	0.67 (0.45 to 0.93)	1111 (749 to 1550)	1.60 (1.56 to 1.62)
Guam	0.18 (0.12 to 0.25)	400 (270 to 552)	0.69 (0.47 to 0.97)	683 (468 to 954)	1.73 (1.73 to 1.74)	0.004 (0.003 to 0.006)	6.99 (4.52 to 10.2)	0.006 (0.004 to 0.010)	7.27 (4.53 to 11.0)	0.13 (0.10 to 0.14)	0.18 (0.12 to 0.25)	393 (266 to 543)	0.69 (0.47 to 0.96)	675 (463 to 944)	1.76 (1.75 to 1.76)
Guatemala	7.18 (4.85 to 10.0)	341 (228 to 482)	56.2 (37.5 to 76.9)	872 (590 to 1194)	3.12 (3.08 to 3.18)	0.42 (0.27 to 0.62)	13.0 (8.38 to 19.1)	1.63 (1.10 to 2.34)	20.7 (14.3 to 30.0)	1.47 (1.39 to 1.52)	6.76 (4.55 to 9.55)	328 (218 to 464)	54.5 (36.4 to 74.8)	852 (575 to 1166)	3.17 (3.13 to 3.23)
Guinea	3.62 (2.45 to 5.08)	199 (135 to 281)	13.7 (9.10 to 19.0)	396 (264 to 550)	2.23 (2.21 to 2.25)	0.29 (0.19 to 0.44)	12.6 (8.00 to 18.5)	0.63 (0.40 to 0.94)	12.4 (7.78 to 18.3)	-0.04 (-0.06 to -0.03)	3.32 (2.25 to 4.74)	186 (127 to 265)	13.0 (8.67 to 18.2)	384 (256 to 534)	2.34 (2.32 to 2.36)
Guinea-Bissau	0.65 (0.44 to 0.92)	262 (173 to 364)	2.80 (1.89 to 3.92)	540 (370 to 757)	2.38 (2.36 to 2.39)	0.05 (0.03 to 0.08)	13.7 (8.84 to 20.3)	0.11 (0.07 to 0.16)	13.6 (8.98 to 19.8)	-0.03 (-0.04 to -0.02)	0.60 (0.40 to 0.85)	248 (164 to 345)	2.69 (1.82 to 3.77)	526 (361 to 737)	2.47 (2.46 to 2.49)
Guyana	2.16 (1.46 to 3.02)	954 (646 to 1336)	5.78 (3.91 to 8.07)	1559 (1056 to 2167)	1.60 (1.59 to 1.60)	0.12 (0.08 to 0.18)	36.1 (24.2 to 51.5)	0.12 (0.08 to 0.17)	29.4 (20.1 to 42.3)	-0.62 (-0.66 to -0.55)	2.03 (1.36 to 2.86)	918 (620 to 1285)	5.66 (3.83 to 7.91)	1529 (1035 to 2124)	1.66 (1.65 to 1.67)
Haiti	14.9 (10.1 to 20.7)	776 (526 to 1078)	56.6 (38.9 to 79.4)	1218 (839 to 1704)	1.46 (1.46 to 1.47)	1.05 (0.71 to 1.50)	38.3 (25.8 to 54.8)	2.05 (1.36 to 3.05)	33.2 (22.2 to 49.2)	-0.42 (-0.45 to -0.39)	13.8 (9.39 to 19.3)	738 (499 to 1029)	54.6 (37.3 to 76.7)	1184 (816 to 1665)	1.54 (1.53 to 1.54)
Honduras	4.51 (3.11 to 6.40)	371 (255 to 526)	28.4 (19.3 to 40.2)	735 (501 to 1042)	2.25 (2.21 to 2.31)	0.12 (0.08 to 0.18)	6.81 (4.34 to 9.97)	0.39 (0.25 to 0.57)	7.96 (5.12 to 11.5)	0.50 (0.49 to 0.52)	4.39 (3.01 to 6.23)	365 (249 to 512)	28.0 (19.0 to 39.7)	727 (495 to 1031)	2.28 (2.23 to 2.33)
Hungary	23.0 (15.4 to 31.6)	276 (187 to 378)	45.5 (30.7 to 63.7)	443 (300 to 621)	1.50 (1.44 to 1.55)	0.77 (0.50 to 1.08)	11.8 (7.76 to 16.5)	1.34 (0.87 to 1.90)	18.9 (12.0 to 27.5)	1.51 (1.49 to 1.53)	22.2 (14.8 to 30.5)	265 (178 to 363)	44.2 (29.7 to 62.2)	424 (286 to 596)	1.50 (1.43 to 1.55)
Iceland	0.19 (0.13 to 0.28)	136 (91.5 to 196)	0.72 (0.48 to 1.01)	291 (194 to 407)	2.48 (2.47 to 2.49)	0.02 (0.02 to 0.03)	17.7 (11.3 to 26.0)	0.05 (0.03 to 0.07)	23.2 (14.4 to 35.4)	0.88 (0.86 to 0.91)	0.17 (0.11 to 0.24)	119 (78.9 to 170)	0.67 (0.45 to 0.94)	268 (180 to 379)	2.67 (2.66 to 2.67)
India	603 (414 to 821)	229 (159 to 312)	2707 (1883 to 3716)	410 (287 to 561)	1.88 (1.85 to 1.90)	38.5 (24.0 to 57.9)	11.1 (7.01 to 16.3)	86.9 (55.3 to 129)	12.4 (7.94 to 18.3)	0.33 (0.31 to 0.36)	564 (390 to 770)	218 (153 to 299)	2620 (1822 to 3589)	398 (278 to 543)	1.94 (1.91 to 1.97)
Indonesia	116 (79.9 to 158)	199 (138 to 270)	526 (368 to 725)	393 (275 to 537)	2.22 (2.12 to 2.34)	13.6 (8.20 to 20.7)	16.8 (10.5 to 25.2)	27.2 (16.8 to 41.3)	18.2 (11.3 to 27.6)	0.27 (0.26 to 0.27)	103 (71.3 to 140)	183 (127 to 248)	499 (350 to 685)	375 (262 to 514)	2.35 (2.25 to 2.48)
Iran (Islamic Republic of)	35.2 (24.9 to 48.4)	252 (178 to 343)	263 (185 to 356)	625 (440 to 844)	2.94 (2.88 to 3.00)	2.94 (1.85 to 4.45)	14.8 (9.34 to 22.0)	7.31 (4.61 to 10.8)	16.1 (10.2 to 23.9)	0.29 (0.28 to 0.30)	32.3 (22.6 to 44.2)	237 (166 to 325)	256 (179 to 348)	609 (426 to 823)	3.05 (2.99 to 3.12)
Iraq	25.3 (17.4 to 35.5)	549 (381 to 761)	168 (113 to 235)	1174 (800 to 1651)	2.49 (2.47 to 2.50)	0.97 (0.62 to 1.40)	14.5 (9.19 to 21.0)	2.69 (1.76 to 3.93)	15.1 (9.82 to 21.7)	0.16 (0.12 to 0.21)	24.3 (16.7 to 34.1)	534 (370 to 741)	165 (111 to 232)	1159 (789 to 1630)	2.53 (2.52 to 2.55)
Ireland	3.08 (2.10 to 4.33)	151 (103 to 214)	9.53 (6.48 to 13.4)	267 (181 to 375)	1.87 (1.85 to 1.88)	0.36 (0.23 to 0.50)	18.7 (12.3 to 26.3)	1.29 (0.87 to 1.81)	42.7 (28.2 to 60.3)	2.71 (2.70 to 2.73)	2.73 (1.84 to 3.82)	132 (89.8 to 187)	8.23 (5.56 to 11.7)	225 (151 to 321)	1.73 (1.72 to 1.74)
Israel	6.17 (4.18 to 8.78)	239 (161 to 342)	21.5 (14.6 to 30.1)	357 (243 to 502)	1.32 (1.30 to 1.34)	0.43 (0.30 to 0.60)	17.6 (12.1 to 24.6)	1.31 (0.91 to 1.83)	25.1 (17.3 to 35.4)	1.14 (1.13 to 1.16)	5.74 (3.89 to 8.16)	221 (149 to 316)	20.2 (13.7 to 28.3)	332 (225 to 465)	1.33 (1.31 to 1.35)
Italy	102 (72.1 to 141)	228 (160 to 315)	210 (147 to 288)	348 (244 to 491)	1.36 (1.32 to 1.39)	11.8 (7.59 to 17.4)	32.3 (20.8 to 47.8)	29.2 (18.8 to 43.7)	63.9 (41.1 to 95.3)	2.06 (1.93 to 2.18)	90.2 (63.2 to 123)	196 (137 to 269)	181 (126 to 249)	284 (198 to 393)	1.21 (1.19 to 1.23)
Jamaica	4.79 (3.27 to 6.60)	510 (345 to 705)	12.8 (8.70 to 17.5)	807 (548 to 1103)	1.48 (1.44 to 1.52)	0.27 (0.18 to 0.39)	25.5 (17.2 to 36.9)	0.30 (0.19 to 0.44)	19.7 (12.6 to 28.7)	-0.74 (-0.80 to -0.69)	4.51 (3.08 to 6.23)	485 (328 to 671)	12.5 (8.50 to 17.0)	787 (535 to 1074)	1.57 (1.53 to 1.60)
Japan	204 (141 to 281)	223 (155 to 308)	574 (399 to 803)	390 (269 to 547)	1.82 (1.79 to 1.84)	8.26 (5.31 to 12.2)	11.3 (7.21 to 16.8)	13.0 (8.37 to 19.0)	14.9 (9.34 to 22.3)	0.82 (0.77 to 0.86)	195 (134 to 270)	212 (146 to 293)	561 (389 to 784)	375 (257 to 526)	1.86 (1.83 to 1.88)
Jordan	4.26 (2.93 to 5.91)	580 (395 to 806)	40.9 (27.8 to 56.6)	1002 (683 to 1372)	1.80 (1.75 to 1.86)	0.31 (0.19 to 0.45)	22.4 (14.4 to 32.7)	0.88 (0.56 to 1.32)	16.6 (10.7 to 24.9)	-0.94 (-1.00 to -0.87)	3.95 (2.71 to 5.50)	557 (380 to 777)	40.0 (27.2 to 55.5)	985 (672 to 1355)	1.88 (1.83 to 1.94)
Kazakhstan	19.8 (13.3 to 27.6)	252 (170 to 352)	60.7 (41.2 to 84.3)	563 (384 to 783)	2.62 (2.61 to 2.64)	1.02 (0.67 to 1.52)	12.5 (8.26 to 18.7)	1.69 (1.08 to 2.46)	16.4 (10.4 to 23.9)	0.90 (0.87 to 0.95)	18.8 (12.7 to 26.4)	240 (162 to 338)	59.0 (40.2 to 82.1)	547 (373 to 762)	2.69 (2.68 to 2.70)
Kenya	7.51 (5.27 to 10.1)	149 (104 to 199)	28.9 (20.0 to 39.5)	197 (137 to 268)	0.90 (0.89 to 0.91)	0.82 (0.54 to 1.21)	9.87 (6.70 to 14.2)	2.20 (1.49 to 3.21)	10.6 (7.16 to 15.3)	0.22 (0.21 to 0.23)	6.69 (4.67 to 9.03)	139 (97.3 to 186)	26.7 (18.4 to 36.4)	187 (130 to 252)	0.94 (0.93 to 0.95)
Kiribati	0.15 (0.10 to 0.21)	641 (437 to 885)	0.59 (0.39 to 0.83)	1211 (804 to 1680)	2.09 (2.07 to 2.10)	0.004 (0.003 to 0.006)	13.1 (8.62 to 19.3)	0.008 (0.005 to 0.01)	13.0 (8.78 to 18.4)	-0.02 (-0.04 to -0.00)	0.15 (0.10 to 0.21)	628 (428 to 866)	0.58 (0.39 to 0.82)	1198 (794 to 1661)	2.12 (2.11 to 2.13)
Kuwait	1.74 (1.20 to 2.43)	562 (391 to 785)	20.0 (13.4 to 28.1)	1130 (767 to 1580)	2.31 (2.28 to 2.35)	0.10 (0.06 to 0.14)	17.5 (11.1 to 24.7)	0.39 (0.25 to 0.59)	17.2 (10.9 to 24.6)	-0.17 (-0.48 to 0.03)	1.64 (1.12 to 2.29)	545 (378 to 764)	19.6 (13.2 to 27.6)	1113 (755 to 1563)	2.35 (2.28 to 2.42)
Kyrgyzstan	2.83 (1.92 to 3.98)	157 (106 to 219)	10.6 (7.23 to 14.9)	354 (243 to 495)	2.67 (2.65 to 2.69)	0.17 (0.11 to 0.25)	8.67 (5.45 to 12.8)	0.41 (0.26 to 0.62)	12.8 (8.03 to 19.1)	1.23 (1.21 to 1.25)	2.65 (1.81 to 3.73)	148 (101 to 207)	10.2 (6.93 to 14.3)	342 (234 to 478)	2.74 (2.72 to 2.75)
Lao People's Democratic Republic	3.00 (2.06 to 4.22)	254 (176 to 356)	16.3 (10.9 to 22.9)	634 (422 to 893)	3.00 (2.99 to 3.00)	0.30 (0.19 to 0.44)	17.3 (11.3 to 25.5)	0.55 (0.35 to 0.83)	15.7 (9.96 to 23.6)	-0.25 (-0.29 to -0.21)	2.70 (1.85 to 3.76)	237 (163 to 330)	15.8 (10.5 to 22.1)	618 (413 to 869)	3.15 (3.14 to 3.15)
Latvia	3.38 (2.27 to 4.76)	160 (108 to 223)	7.18 (4.87 to 10.1)	353 (241 to 497)	2.59 (2.57 to 2.61)	0.16 (0.11 to 0.24)	9.54 (6.21 to 13.8)	0.23 (0.15 to 0.33)	15.5 (9.77 to 23.0)	1.59 (1.57 to 1.61)	3.22 (2.16 to 4.55)	150 (101 to 211)	6.96 (4.70 to 9.79)	337 (230 to 480)	2.64 (2.63 to 2.66)
Lebanon	5.17 (3.52 to 7.42)	436 (297 to 620)	26.8 (18.5 to 37.8)	846 (584 to 1194)	2.20 (2.16 to 2.24)	0.24 (0.16 to 0.36)	18.0 (11.9 to 26.7)	0.53 (0.34 to 0.77)	17.1 (11.2 to 25.1)	-0.10 (-0.14 to -0.06)	4.93 (3.36 to 7.08)	418 (284 to 596)	26.3 (18.1 to 37.1)	829 (572 to 1169)	2.27 (2.23 to 2.31)
Lesotho	0.99 (0.68 to 1.41)	175 (120 to 252)	3.39 (2.32 to 4.73)	484 (331 to 673)	3.32 (3.28 to 3.35)	0.11 (0.07 to 0.16)	16.7 (10.8 to 24.3)	0.15 (0.10 to 0.22)	18.2 (11.7 to 26.7)	0.28 (0.26 to 0.29)	0.87 (0.59 to 1.27)	159 (109 to 228)	3.24 (2.22 to 4.53)	466 (320 to 649)	3.52 (3.49 to 3.56)
Liberia	1.33 (0.92 to 1.86)	217 (148 to 304)	6.13 (4.07 to 8.55)	446 (303 to 620)	2.37 (2.36 to 2.38)	0.09 (0.06 to 0.14)	10.8 (7.13 to 15.7)	0.23 (0.15 to 0.34)	11.3 (7.38 to 16.7)	0.16 (0.14 to 0.18)	1.24 (0.86 to 1.73)	206 (141 to 290)	5.89 (3.92 to 8.29)	435 (296 to 605)	2.45 (2.44 to 2.46)
Libya	2.98 (2.05 to 4.17)	303 (207 to 423)	23.9 (15.9 to 34.1)	790 (532 to 1130)	3.22 (3.15 to 3.29)	0.19 (0.12 to 0.27)	13.0 (8.35 to 18.6)	0.53 (0.35 to 0.77)	15.4 (10.1 to 22.1)	0.57 (0.54 to 0.59)	2.79 (1.91 to 3.95)	290 (197 to 408)	23.4 (15.6 to 33.3)	775 (523 to 1111)	3.31 (3.23 to 3.39)
Lithuania	3.73 (2.55 to 5.29)	142 (97.7 to 200)	8.43 (5.73 to 12.0)	288 (195 to 415)	2.30 (2.28 to 2.33)	0.23 (0.15 to 0.33)	10.2 (6.92 to 15.0)	0.35 (0.23 to 0.51)	16.1 (10.6 to 24.3)	1.62 (1.56 to 1.66)	3.50 (2.39 to 4.99)	132 (90.6 to 186)	8.08 (5.47 to 11.5)	272 (185 to 395)	2.35 (2.32 to 2.38)
Luxembourg	0.46 (0.32 to 0.65)	163 (111 to 230)	1.47 (1.00 to 2.07)	307 (208 to 433)	2.07 (2.07 to 2.08)	0.04 (0.03 to 0.06)	17.4 (11.2 to 25.9)	0.11 (0.07 to 0.16)	27.4 (17.4 to 40.8)	1.48 (1.46 to 1.49)	0.42 (0.29 to 0.60)	145 (99.9 to 205)	1.36 (0.93 to 1.90)	280 (189 to 390)	2.14 (2.13 to 2.14)
Madagascar	4.59 (3.18 to 6.36)	156 (108 to 218)	19.6 (13.3 to 27.6)	258 (177 to 359)	1.63 (1.60 to 1.64)	0.56 (0.37 to 0.80)	12.1 (7.93 to 17.4)	1.37 (0.90 to 1.97)	11.7 (7.73 to 16.4)	-0.11 (-0.12 to -0.09)	4.03 (2.77 to 5.60)	144 (99.6 to 202)	18.2 (12.4 to 25.7)	246 (168 to 342)	1.74 (1.71 to 1.75)
Malawi	3.21 (2.20 to 4.40)	136 (93.4 to 187)	9.47 (6.60 to 12.9)	187 (131 to 259)	1.03 (1.02 to 1.04)	0.53 (0.35 to 0.75)	13.6 (8.97 to 19.6)	1.00 (0.65 to 1.45)	12.6 (8.37 to 17.9)	-0.21 (-0.24 to -0.17)	2.68 (1.83 to 3.74)	123 (84.6 to 170)	8.47 (5.89 to 11.7)	175 (122 to 242)	1.14 (1.13 to 1.15)
Malaysia	19.0 (13.1 to 26.4)	367 (249 to 517)	92.4 (62.9 to 126)	629 (429 to 863)	1.73 (1.70 to 1.76)	1.22 (0.79 to 1.82)	17.0 (11.0 to 24.9)	2.47 (1.61 to 3.65)	15.4 (10.1 to 22.9)	-0.24 (-0.29 to -0.19)	17.8 (11.9 to 24.8)	350 (236 to 495)	89.9 (61.2 to 123)	614 (418 to 844)	1.80 (1.77 to 1.83)
Maldives	0.12 (0.08 to 0.17)	277 (191 to 389)	0.79 (0.55 to 1.11)	491 (342 to 680)	1.87 (1.85 to 1.89)	0.01 (0.008 to 0.02)	16.5 (10.9 to 23.9)	0.03 (0.02 to 0.05)	14.6 (9.32 to 21.8)	-0.32 (-0.36 to -0.27)	0.11 (0.07 to 0.15)	261 (180 to 368)	0.76 (0.52 to 1.06)	476 (330 to 661)	1.96 (1.95 to 1.98)
Mali	8.57 (5.78 to 12.4)	376 (257 to 540)	40.8 (27.3 to 57.4)	752 (508 to 1063)	2.26 (2.24 to 2.28)	0.36 (0.23 to 0.53)	11.4 (7.52 to 16.9)	0.87 (0.55 to 1.29)	10.8 (6.94 to 16.3)	-0.21 (-0.22 to -0.19)	8.22 (5.55 to 11.8)	364 (247 to 523)	40.0 (26.7 to 56.2)	742 (500 to 1049)	2.32 (2.30 to 2.33)
Malta	0.43 (0.29 to 0.61)	189 (128 to 267)	1.72 (1.18 to 2.45)	428 (289 to 617)	2.68 (2.66 to 2.70)	0.07 (0.05 to 0.10)	33.1 (21.8 to 46.8)	0.17 (0.11 to 0.23)	55.9 (37.0 to 77.2)	1.70 (1.69 to 1.72)	0.36 (0.25 to 0.52)	156 (107 to 223)	1.56 (1.05 to 2.20)	372 (252 to 532)	2.86 (2.84 to 2.88)
Marshall Islands	0.08 (0.06 to 0.12)	812 (537 to 1147)	0.40 (0.27 to 0.57)	1786 (1209 to 2515)	2.55 (2.53 to 2.57)	0.002 (0.002 to 0.003)	13.8 (9.10 to 19.6)	0.004 (0.002 to 0.005)	13.7 (8.95 to 19.5)	-0.00 (-0.03 to 0.02)	0.08 (0.05 to 0.11)	798 (528 to 1132)	0.40 (0.27 to 0.56)	1772 (1198 to 2495)	2.58 (2.56 to 2.60)
Mauritania	1.12 (0.77 to 1.58)	194 (133 to 272)	4.47 (2.98 to 6.29)	355 (237 to 499)	1.96 (1.94 to 1.98)	0.09 (0.06 to 0.13)	11.7 (7.73 to 16.9)	0.20 (0.13 to 0.31)	12.1 (7.79 to 18.2)	0.10 (0.08 to 0.11)	1.04 (0.71 to 1.46)	183 (125 to 256)	4.26 (2.84 to 6.00)	343 (228 to 484)	2.05 (2.03 to 2.07)
Mauritius	1.96 (1.32 to 2.73)	470 (319 to 658)	9.60 (6.49 to 13.4)	979 (669 to 1364)	2.41 (2.38 to 2.45)	0.11 (0.07 to 0.16)	21.2 (14.0 to 31.0)	0.18 (0.11 to 0.26)	22.4 (14.5 to 33.4)	0.15 (0.10 to 0.18)	1.84 (1.25 to 2.58)	449 (304 to 628)	9.43 (6.36 to 13.2)	957 (653 to 1335)	2.49 (2.45 to 2.53)
Mexico	191 (131 to 258)	754 (516 to 1012)	658 (454 to 893)	932 (643 to 1260)	0.69 (0.67 to 0.70)	5.92 (3.73 to 9.14)	16.2 (10.2 to 24.6)	9.31 (5.66 to 14.2)	13.2 (8.03 to 20.1)	-0.59 (-0.63 to -0.54)	185 (127 to 250)	738 (507 to 993)	649 (447 to 883)	918 (634 to 1246)	0.71 (0.69 to 0.73)
Micronesia (Federated States of)	0.15 (0.10 to 0.21)	524 (351 to 738)	0.53 (0.37 to 0.74)	1194 (831 to 1671)	2.70 (2.67 to 2.74)	0.005 (0.003 to 0.007)	12.9 (8.69 to 18.1)	0.006 (0.004 to 0.009)	12.9 (8.22 to 19.0)	0.04 (0.01 to 0.07)	0.14 (0.10 to 0.20)	511 (342 to 721)	0.52 (0.36 to 0.73)	1181 (822 to 1652)	2.75 (2.71 to 2.79)
Monaco	0.04 (0.03 to 0.05)	127 (85.8 to 182)	0.10 (0.07 to 0.14)	275 (183 to 387)	2.53 (2.53 to 2.54)	0.005 (0.003 to 0.007)	19.9 (12.3 to 29.9)	0.009 (0.006 to 0.01)	31.2 (19.6 to 47.7)	1.46 (1.44 to 1.48)	0.03 (0.02 to 0.05)	107 (72.7 to 153)	0.09 (0.06 to 0.13)	244 (164 to 343)	2.70 (2.69 to 2.71)
Mongolia	0.87 (0.60 to 1.22)	138 (93.2 to 193)	4.65 (3.14 to 6.76)	306 (209 to 444)	2.61 (2.59 to 2.63)	0.07 (0.04 to 0.10)	8.21 (5.12 to 12.1)	0.19 (0.13 to 0.28)	12.0 (7.77 to 17.4)	1.23 (1.20 to 1.25)	0.81 (0.55 to 1.13)	129 (86.9 to 183)	4.46 (3.02 to 6.53)	294 (202 to 431)	2.69 (2.67 to 2.70)
Montenegro	1.14 (0.76 to 1.63)	326 (217 to 467)	2.81 (1.88 to 3.91)	537 (363 to 755)	1.61 (1.58 to 1.64)	0.07 (0.05 to 0.10)	20.2 (13.6 to 29.2)	0.11 (0.07 to 0.16)	28.3 (18.6 to 40.8)	1.10 (1.08 to 1.12)	1.07 (0.71 to 1.54)	306 (203 to 439)	2.70 (1.80 to 3.76)	509 (342 to 719)	1.64 (1.61 to 1.67)
Morocco	31.0 (20.7 to 44.1)	397 (267 to 566)	210 (142 to 298)	1136 (768 to 1610)	3.45 (3.44 to 3.46)	1.58 (1.04 to 2.32)	15.2 (10.1 to 22.2)	3.26 (2.12 to 4.77)	17.1 (11.1 to 25.0)	0.37 (0.36 to 0.39)	29.4 (19.6 to 42.0)	382 (258 to 547)	207 (140 to 293)	1119 (757 to 1587)	3.53 (3.52 to 3.54)
Mozambique	4.79 (3.27 to 6.56)	133 (91.7 to 182)	20.2 (13.7 to 28.1)	260 (178 to 361)	2.17 (2.14 to 2.20)	0.80 (0.53 to 1.15)	14.0 (9.49 to 20.0)	1.79 (1.14 to 2.61)	13.8 (9.07 to 19.7)	0.02 (-0.01 to 0.05)	3.99 (2.74 to 5.56)	119 (82.2 to 164)	18.4 (12.4 to 25.6)	247 (167 to 344)	2.35 (2.31 to 2.39)
Myanmar	47.2 (32.2 to 66.6)	357 (245 to 504)	213 (146 to 299)	752 (517 to 1048)	2.45 (2.43 to 2.47)	4.05 (2.59 to 6.17)	22.5 (14.4 to 34.0)	5.96 (3.74 to 9.07)	19.8 (12.5 to 30.0)	-0.30 (-0.37 to -0.24)	43.2 (29.5 to 61.0)	334 (229 to 473)	207 (142 to 290)	732 (503 to 1021)	2.58 (2.56 to 2.60)
Namibia	0.91 (0.62 to 1.29)	238 (162 to 336)	3.50 (2.37 to 4.90)	400 (270 to 561)	1.67 (1.66 to 1.68)	0.08 (0.05 to 0.12)	16.1 (10.6 to 23.6)	0.16 (0.11 to 0.25)	15.3 (9.85 to 22.5)	-0.13 (-0.17 to -0.09)	0.83 (0.57 to 1.17)	222 (152 to 313)	3.33 (2.26 to 4.69)	384 (261 to 542)	1.77 (1.75 to 1.78)
Nauru	0.02 (0.01 to 0.02)	664 (449 to 937)	0.05 (0.03 to 0.07)	1309 (893 to 1806)	2.20 (2.18 to 2.22)	5e-04 (3e-04 to 7e-04)	12.5 (8.18 to 17.9)	6e-04 (4e-04 to 8e-04)	11.0 (7.07 to 15.8)	-0.39 (-0.45 to -0.33)	0.02 (0.01 to 0.02)	651 (440 to 922)	0.05 (0.03 to 0.07)	1298 (885 to 1791)	2.23 (2.21 to 2.25)
Nepal	14.0 (9.50 to 19.9)	255 (172 to 361)	74.2 (50.4 to 105)	535 (366 to 752)	2.38 (2.35 to 2.41)	0.67 (0.43 to 0.95)	8.84 (5.79 to 12.7)	1.55 (1.00 to 2.27)	9.95 (6.43 to 14.5)	0.36 (0.33 to 0.39)	13.4 (9.04 to 19.0)	246 (166 to 348)	72.7 (49.3 to 103)	525 (359 to 738)	2.43 (2.41 to 2.46)
Netherlands	17.2 (11.9 to 23.8)	160 (111 to 222)	37.5 (25.3 to 52.5)	253 (171 to 354)	1.49 (1.47 to 1.50)	2.12 (1.43 to 3.02)	23.6 (15.9 to 33.6)	4.10 (2.64 to 5.83)	35.0 (22.8 to 50.5)	1.27 (1.25 to 1.29)	15.1 (10.4 to 20.9)	136 (95.2 to 190)	33.4 (22.4 to 47.5)	218 (146 to 310)	1.52 (1.49 to 1.55)
New Zealand	4.50 (3.21 to 6.23)	223 (159 to 307)	13.0 (9.33 to 17.8)	332 (237 to 452)	1.31 (1.29 to 1.33)	0.43 (0.28 to 0.63)	22.8 (14.8 to 33.4)	0.75 (0.49 to 1.10)	22.7 (14.9 to 33.4)	-0.01 (-0.03 to -0.00)	4.06 (2.87 to 5.62)	200 (141 to 275)	12.3 (8.76 to 16.8)	309 (220 to 421)	1.43 (1.41 to 1.46)

Location	DM, Number in 1990 (000s)	DM, ASR in 1990 (per 100,000)	DM, Number in 2021 (000s)	DM, ASR in 2021 (per 100,000)	DM, AAPC (95%CI)	T1D, Number in 1990 (000s)	T1D, ASR in 1990 (per 100,000)	T1D, Number in 2021 (000s)	T1D, ASR in 2021 (per 100,000)	T1D, AAPC (95%CI)	T2D, Number in 1990 (000s)	T2D, ASR in 1990 (per 100,000)	T2D, Number in 2021 (000s)	T2D, ASR in 2021 (per 100,000)	T2D, AAPC (95%CI)
Nicaragua	4.07 (2.81 to 5.72)	430 (296 to 609)	22.5 (15.1 to 31.6)	777 (518 to 1090)	1.93 (1.90 to 1.97)	0.12 (0.08 to 0.17)	8.01 (5.34 to 11.6)	0.27 (0.18 to 0.40)	8.09 (5.36 to 11.9)	0.05 (0.02 to 0.07)	3.95 (2.73 to 5.54)	422 (290 to 598)	22.2 (14.9 to 31.3)	769 (512 to 1081)	1.96 (1.93 to 2.00)
Niger	3.69 (2.51 to 5.15)	222 (151 to 309)	23.5 (16.0 to 33.0)	443 (303 to 620)	2.26 (2.26 to 2.27)	0.28 (0.17 to 0.40)	10.5 (6.67 to 15.2)	0.85 (0.54 to 1.25)	10.4 (6.64 to 15.0)	-0.04 (-0.06 to -0.03)	3.42 (2.32 to 4.79)	211 (144 to 296)	22.7 (15.4 to 31.8)	433 (297 to 607)	2.34 (2.34 to 2.35)
Nigeria	47.0 (33.2 to 64.4)	201 (143 to 275)	188 (132 to 257)	312 (217 to 425)	1.44 (1.41 to 1.48)	3.29 (2.07 to 4.92)	10.6 (6.65 to 15.6)	9.95 (6.31 to 14.8)	11.5 (7.28 to 17.1)	0.26 (0.21 to 0.29)	43.7 (30.7 to 59.7)	191 (134 to 260)	178 (124 to 243)	300 (210 to 409)	1.49 (1.46 to 1.53)
Niue	0.008 (0.005 to 0.01)	659 (437 to 916)	0.02 (0.01 to 0.02)	1425 (962 to 1985)	2.51 (2.50 to 2.53)	1e-04 (8e-05 to 2e-04)	10.8 (7.20 to 15.6)	1e-04 (6e-05 to 1e-04)	10.6 (6.90 to 15.5)	-0.01 (-0.05 to 0.02)	0.008 (0.005 to 0.01)	648 (430 to 902)	0.02 (0.01 to 0.02)	1415 (955 to 1971)	2.54 (2.53 to 2.56)
North Macedonia	3.56 (2.38 to 5.08)	354 (238 to 506)	11.5 (7.78 to 16.3)	657 (447 to 933)	2.02 (2.00 to 2.03)	0.21 (0.13 to 0.31)	20.0 (12.8 to 29.9)	0.37 (0.24 to 0.56)	26.6 (17.2 to 39.2)	0.94 (0.91 to 0.98)	3.36 (2.25 to 4.79)	334 (224 to 479)	11.2 (7.54 to 15.8)	630 (429 to 896)	2.07 (2.05 to 2.09)
Northern Mariana Islands	0.05 (0.03 to 0.07)	406 (271 to 568)	0.21 (0.14 to 0.30)	770 (518 to 1087)	2.09 (2.08 to 2.10)	0.001 (9e-04 to 0.002)	7.52 (4.83 to 11.2)	0.002 (0.001 to 0.003)	7.80 (4.93 to 11.2)	0.11 (0.09 to 0.13)	0.05 (0.03 to 0.07)	398 (265 to 556)	0.21 (0.14 to 0.30)	762 (512 to 1075)	2.12 (2.11 to 2.13)
Norway	6.71 (4.73 to 9.30)	212 (149 to 295)	11.7 (8.18 to 16.3)	280 (194 to 396)	0.90 (0.88 to 0.91)	1.01 (0.68 to 1.44)	38.3 (25.7 to 55.1)	1.90 (1.28 to 2.70)	54.0 (35.9 to 78.2)	1.11 (1.11 to 1.12)	5.70 (4.00 to 7.99)	174 (121 to 243)	9.75 (6.76 to 13.7)	226 (156 to 320)	0.85 (0.82 to 0.87)
Oman	1.30 (0.88 to 1.86)	362 (244 to 526)	7.54 (4.98 to 10.6)	669 (455 to 946)	1.96 (1.90 to 2.01)	0.23 (0.15 to 0.34)	41.8 (27.3 to 61.7)	0.68 (0.45 to 1.00)	41.8 (27.6 to 61.0)	0.01 (-0.03 to 0.05)	1.07 (0.71 to 1.53)	320 (213 to 462)	6.86 (4.51 to 9.69)	627 (426 to 888)	2.14 (2.07 to 2.20)
Pakistan	75.6 (53.4 to 102)	256 (181 to 346)	435 (308 to 595)	603 (427 to 823)	2.81 (2.77 to 2.84)	6.87 (4.27 to 10.3)	16.9 (10.9 to 25.0)	18.9 (11.7 to 28.3)	19.0 (12.0 to 28.1)	0.37 (0.37 to 0.38)	68.7 (48.8 to 93.5)	239 (170 to 323)	416 (295 to 572)	584 (412 to 800)	2.93 (2.89 to 2.97)
Palau	0.04 (0.03 to 0.05)	691 (466 to 971)	0.16 (0.10 to 0.22)	1366 (904 to 1876)	2.23 (2.21 to 2.24)	8e-04 (5e-04 to 0.001)	11.6 (7.72 to 16.6)	0.001 (7e-04 to 0.002)	11.5 (7.57 to 17.0)	-0.03 (-0.04 to -0.02)	0.04 (0.03 to 0.05)	679 (458 to 958)	0.16 (0.10 to 0.22)	1354 (895 to 1859)	2.25 (2.24 to 2.26)
Palestine	1.79 (1.23 to 2.57)	350 (240 to 501)	10.9 (7.57 to 15.1)	736 (508 to 1021)	2.44 (2.43 to 2.45)	0.13 (0.08 to 0.20)	18.7 (12.0 to 27.9)	0.43 (0.27 to 0.64)	20.7 (13.2 to 30.3)	0.34 (0.30 to 0.36)	1.65 (1.15 to 2.37)	331 (228 to 474)	10.5 (7.30 to 14.6)	715 (494 to 989)	2.53 (2.52 to 2.54)
Panama	2.90 (1.99 to 4.06)	359 (247 to 509)	15.2 (10.4 to 21.7)	674 (464 to 965)	2.07 (2.03 to 2.13)	0.10 (0.06 to 0.15)	10.0 (6.35 to 14.5)	0.25 (0.16 to 0.37)	11.5 (7.24 to 16.8)	0.41 (0.38 to 0.45)	2.79 (1.91 to 3.92)	349 (239 to 496)	14.9 (10.3 to 21.4)	662 (457 to 949)	2.11 (2.06 to 2.17)
Papua New Guinea	4.10 (2.81 to 5.75)	366 (251 to 514)	28.0 (18.9 to 39.5)	856 (584 to 1205)	2.77 (2.74 to 2.79)	0.14 (0.09 to 0.21)	8.73 (5.69 to 12.9)	0.40 (0.26 to 0.58)	9.11 (5.95 to 13.1)	0.14 (0.13 to 0.15)	3.96 (2.71 to 5.57)	358 (244 to 503)	27.6 (18.7 to 38.9)	847 (578 to 1193)	2.81 (2.78 to 2.83)
Paraguay	4.17 (2.84 to 5.84)	340 (230 to 478)	19.7 (13.5 to 27.4)	627 (427 to 869)	1.99 (1.97 to 2.01)	0.23 (0.15 to 0.33)	14.6 (9.64 to 21.6)	0.62 (0.41 to 0.92)	18.0 (11.8 to 26.6)	0.67 (0.66 to 0.67)	3.94 (2.67 to 5.56)	325 (220 to 459)	19.1 (13.0 to 26.5)	609 (413 to 842)	2.04 (2.02 to 2.06)
Peru	11.7 (8.00 to 16.7)	176 (120 to 251)	55.7 (37.1 to 78.5)	314 (209 to 443)	1.88 (1.86 to 1.90)	0.56 (0.36 to 0.81)	6.34 (4.12 to 9.18)	1.42 (0.91 to 2.03)	7.69 (4.93 to 11.1)	0.65 (0.63 to 0.67)	11.2 (7.54 to 15.9)	170 (115 to 244)	54.3 (36.1 to 76.5)	307 (203 to 433)	1.91 (1.89 to 1.94)
Philippines	50.4 (35.1 to 68.3)	304 (210 to 411)	191 (131 to 258)	418 (289 to 560)	1.03 (0.98 to 1.07)	5.20 (3.18 to 7.97)	20.8 (12.6 to 31.3)	9.36 (5.74 to 14.2)	17.5 (10.8 to 26.3)	-0.49 (-0.55 to -0.40)	45.2 (31.2 to 60.8)	283 (195 to 383)	182 (125 to 245)	400 (276 to 535)	1.12 (1.06 to 1.16)
Poland	80.8 (56.2 to 111)	322 (224 to 439)	171 (118 to 232)	450 (312 to 614)	1.10 (1.07 to 1.13)	2.97 (1.84 to 4.46)	13.8 (8.51 to 20.8)	4.76 (2.99 to 7.01)	17.9 (11.1 to 27.0)	0.85 (0.83 to 0.87)	77.9 (54.3 to 107)	308 (215 to 421)	166 (115 to 225)	432 (298 to 589)	1.12 (1.08 to 1.15)
Portugal	17.3 (11.8 to 24.0)	240 (164 to 337)	53.4 (36.5 to 74.7)	500 (344 to 704)	2.39 (2.39 to 2.40)	1.00 (0.66 to 1.46)	16.4 (10.6 to 23.6)	2.28 (1.43 to 3.37)	28.3 (17.8 to 41.8)	1.78 (1.76 to 1.79)	16.3 (11.1 to 22.5)	223 (151 to 311)	51.1 (35.0 to 71.8)	472 (327 to 665)	2.44 (2.43 to 2.45)
Puerto Rico	12.3 (8.19 to 16.9)	633 (424 to 873)	32.5 (22.0 to 45.1)	1022 (684 to 1423)	1.56 (1.55 to 1.57)	0.74 (0.49 to 1.07)	38.4 (25.4 to 55.7)	0.77 (0.51 to 1.10)	33.0 (21.5 to 48.0)	-0.45 (-0.49 to -0.42)	11.5 (7.68 to 16.0)	594 (397 to 823)	31.7 (21.4 to 44.1)	989 (663 to 1378)	1.65 (1.64 to 1.66)
Qatar	0.30 (0.20 to 0.43)	664 (448 to 920)	6.19 (4.15 to 8.69)	1369 (933 to 1878)	2.35 (2.34 to 2.37)	0.02 (0.01 to 0.03)	21.3 (13.7 to 32.5)	0.20 (0.13 to 0.31)	25.3 (16.3 to 38.3)	0.54 (0.53 to 0.55)	0.28 (0.19 to 0.40)	643 (432 to 890)	5.98 (4.02 to 8.39)	1344 (915 to 1845)	2.40 (2.38 to 2.42)
Republic of Korea	54.9 (37.2 to 77.0)	292 (199 to 406)	331 (225 to 465)	729 (497 to 1029)	3.00 (2.97 to 3.03)	3.84 (2.48 to 5.74)	17.2 (11.2 to 25.7)	6.34 (3.99 to 9.61)	19.1 (12.0 to 28.6)	0.34 (0.31 to 0.38)	51.0 (34.4 to 71.1)	275 (186 to 381)	324 (220 to 455)	710 (482 to 1002)	3.12 (3.08 to 3.15)
Republic of Moldova	5.64 (3.84 to 7.91)	217 (149 to 304)	13.5 (9.26 to 18.7)	417 (286 to 579)	2.14 (2.12 to 2.15)	0.24 (0.15 to 0.36)	9.68 (6.22 to 14.5)	0.37 (0.23 to 0.56)	14.2 (8.89 to 21.7)	1.28 (1.24 to 1.32)	5.40 (3.68 to 7.60)	208 (142 to 291)	13.1 (9.01 to 18.2)	403 (276 to 559)	2.17 (2.15 to 2.19)
Romania	30.3 (20.0 to 43.0)	198 (131 to 281)	59.4 (39.3 to 85.5)	314 (207 to 449)	1.51 (1.48 to 1.53)	1.19 (0.76 to 1.77)	8.92 (5.71 to 13.2)	1.84 (1.23 to 2.71)	13.6 (8.86 to 20.0)	1.37 (1.34 to 1.39)	29.1 (19.2 to 41.6)	189 (125 to 271)	57.6 (38.0 to 83.1)	300 (196 to 430)	1.51 (1.48 to 1.54)
Russian Federation	183 (127 to 250)	165 (115 to 224)	456 (317 to 628)	334 (233 to 458)	2.32 (2.30 to 2.33)	12.2 (8.22 to 17.4)	12.8 (8.58 to 18.4)	24.4 (16.2 to 34.3)	22.8 (15.0 to 32.5)	1.93 (1.90 to 1.96)	171 (118 to 234)	152 (106 to 208)	431 (300 to 596)	311 (218 to 429)	2.34 (2.33 to 2.36)
Rwanda	2.67 (1.84 to 3.66)	148 (102 to 200)	8.27 (5.68 to 11.5)	196 (136 to 273)	0.90 (0.88 to 0.91)	0.44 (0.29 to 0.63)	14.9 (9.83 to 21.2)	0.82 (0.53 to 1.15)	13.8 (9.27 to 19.5)	-0.17 (-0.23 to -0.12)	2.23 (1.53 to 3.07)	133 (90.9 to 184)	7.45 (5.17 to 10.4)	182 (126 to 254)	1.00 (0.98 to 1.02)
Saint Kitts and Nevis	0.13 (0.09 to 0.18)	655 (441 to 906)	0.37 (0.24 to 0.51)	948 (628 to 1340)	1.18 (1.17 to 1.20)	0.006 (0.004 to 0.009)	32.4 (21.0 to 47.3)	0.008 (0.005 to 0.01)	23.2 (15.3 to 33.8)	-1.00 (-1.06 to -0.95)	0.12 (0.08 to 0.17)	623 (417 to 862)	0.36 (0.24 to 0.50)	925 (611 to 1304)	1.27 (1.25 to 1.29)
Saint Lucia	0.47 (0.31 to 0.65)	942 (632 to 1311)	1.48 (0.99 to 2.07)	1203 (802 to 1679)	0.78 (0.77 to 0.80)	0.02 (0.01 to 0.02)	25.6 (16.9 to 37.8)	0.02 (0.01 to 0.03)	19.1 (12.8 to 27.2)	-0.85 (-0.91 to -0.80)	0.45 (0.30 to 0.63)	917 (616 to 1279)	1.46 (0.97 to 2.04)	1183 (788 to 1652)	0.82 (0.81 to 0.83)
Saint Vincent and the Grenadines	0.33 (0.23 to 0.46)	836 (563 to 1157)	0.84 (0.57 to 1.18)	1218 (819 to 1698)	1.22 (1.21 to 1.23)	0.02 (0.01 to 0.02)	34.3 (23.0 to 49.0)	0.02 (0.01 to 0.02)	25.8 (17.1 to 37.1)	-0.90 (-0.95 to -0.83)	0.32 (0.21 to 0.44)	802 (539 to 1109)	0.83 (0.56 to 1.16)	1192 (800 to 1663)	1.29 (1.27 to 1.29)
Samoa	0.29 (0.19 to 0.41)	598 (398 to 845)	1.02 (0.68 to 1.43)	1279 (857 to 1817)	2.47 (2.46 to 2.48)	0.006 (0.004 to 0.009)	9.83 (6.45 to 14.3)	0.009 (0.006 to 0.01)	9.70 (6.23 to 13.6)	-0.02 (-0.04 to -0.00)	0.28 (0.19 to 0.40)	588 (392 to 830)	1.01 (0.68 to 1.42)	1270 (850 to 1803)	2.50 (2.49 to 2.51)
San Marino	0.02 (0.02 to 0.03)	144 (96.0 to 198)	0.09 (0.06 to 0.13)	301 (204 to 429)	2.42 (2.42 to 2.43)	0.003 (0.002 to 0.004)	19.4 (12.1 to 28.4)	0.008 (0.005 to 0.01)	32.3 (20.3 to 48.5)	1.66 (1.64 to 1.68)	0.02 (0.01 to 0.03)	124 (84.3 to 175)	0.08 (0.06 to 0.12)	269 (184 to 386)	2.53 (2.52 to 2.54)
Sao Tome and Principe	0.09 (0.06 to 0.13)	253 (173 to 357)	0.37 (0.25 to 0.52)	534 (361 to 752)	2.44 (2.44 to 2.45)	0.005 (0.004 to 0.008)	12.4 (8.02 to 18.3)	0.01 (0.008 to 0.02)	13.1 (8.45 to 19.3)	0.17 (0.16 to 0.19)	0.09 (0.06 to 0.12)	240 (165 to 341)	0.36 (0.24 to 0.51)	521 (352 to 735)	2.53 (2.52 to 2.53)
Saudi Arabia	9.18 (6.31 to 12.7)	308 (219 to 427)	79.2 (54.6 to 112)	688 (478 to 951)	2.64 (2.59 to 2.68)	0.98 (0.60 to 1.54)	19.2 (11.8 to 29.7)	4.39 (2.58 to 8.47)	28.5 (17.2 to 53.8)	1.27 (1.23 to 1.29)	8.19 (5.71 to 11.4)	289 (207 to 399)	74.8 (51.5 to 106)	659 (460 to 920)	2.70 (2.67 to 2.74)
Senegal	5.72 (3.86 to 8.04)	307 (207 to 439)	29.2 (19.7 to 40.6)	636 (428 to 883)	2.41 (2.38 to 2.44)	0.32 (0.21 to 0.47)	11.9 (7.68 to 17.8)	0.76 (0.50 to 1.11)	12.3 (8.12 to 17.7)	0.08 (0.06 to 0.10)	5.40 (3.62 to 7.64)	295 (199 to 425)	28.4 (19.2 to 39.6)	623 (420 to 865)	2.48 (2.44 to 2.51)
Serbia	21.8 (14.3 to 30.5)	349 (230 to 488)	48.2 (32.2 to 67.4)	572 (383 to 810)	1.60 (1.59 to 1.62)	0.96 (0.64 to 1.36)	17.4 (11.5 to 24.4)	1.44 (0.96 to 2.07)	23.9 (15.8 to 34.4)	1.04 (1.00 to 1.07)	20.8 (13.6 to 29.3)	332 (219 to 468)	46.8 (31.1 to 65.6)	548 (365 to 776)	1.62 (1.61 to 1.64)
Seychelles	0.10 (0.06 to 0.13)	301 (201 to 418)	0.60 (0.41 to 0.84)	984 (675 to 1377)	3.89 (3.86 to 3.91)	0.005 (0.003 to 0.007)	14.4 (9.34 to 21.2)	0.008 (0.005 to 0.01)	14.3 (9.51 to 20.6)	-0.03 (-0.04 to -0.02)	0.09 (0.06 to 0.13)	287 (192 to 396)	0.59 (0.40 to 0.83)	969 (664 to 1359)	4.01 (3.99 to 4.03)
Sierra Leone	2.39 (1.61 to 3.33)	210 (144 to 295)	11.0 (7.41 to 15.5)	470 (319 to 664)	2.64 (2.63 to 2.65)	0.17 (0.11 to 0.26)	11.0 (7.05 to 16.5)	0.39 (0.26 to 0.58)	11.6 (7.56 to 17.1)	0.19 (0.16 to 0.21)	2.22 (1.50 to 3.12)	199 (136 to 279)	10.6 (7.12 to 14.9)	458 (310 to 648)	2.74 (2.72 to 2.75)
Singapore	5.29 (3.66 to 7.40)	401 (275 to 566)	21.8 (15.0 to 30.9)	520 (356 to 736)	0.85 (0.83 to 0.86)	0.20 (0.13 to 0.29)	12.5 (7.99 to 18.7)	0.49 (0.31 to 0.73)	14.6 (9.27 to 21.9)	0.50 (0.48 to 0.52)	5.09 (3.50 to 7.16)	388 (264 to 550)	21.3 (14.6 to 30.2)	506 (345 to 717)	0.86 (0.84 to 0.87)
Slovakia	8.24 (5.61 to 11.6)	244 (166 to 346)	18.7 (12.6 to 26.1)	367 (251 to 512)	1.32 (1.29 to 1.35)	0.32 (0.21 to 0.45)	10.9 (7.29 to 15.1)	0.64 (0.42 to 0.93)	17.0 (10.9 to 25.2)	1.45 (1.43 to 1.46)	7.92 (5.40 to 11.2)	234 (160 to 331)	18.0 (12.2 to 25.2)	350 (240 to 491)	1.32 (1.29 to 1.34)
Slovenia	3.96 (2.69 to 5.62)	274 (184 to 389)	7.71 (5.28 to 10.8)	362 (246 to 510)	0.90 (0.86 to 0.92)	0.16 (0.11 to 0.23)	13.1 (8.66 to 19.2)	0.27 (0.17 to 0.39)	17.9 (11.3 to 26.1)	1.05 (1.01 to 1.12)	3.80 (2.59 to 5.41)	260 (177 to 370)	7.44 (5.08 to 10.5)	344 (234 to 485)	0.89 (0.85 to 0.92)
Solomon Islands	0.26 (0.18 to 0.37)	324 (219 to 450)	1.60 (1.08 to 2.27)	725 (488 to 1025)	2.64 (2.61 to 2.66)	0.02 (0.010 to 0.02)	11.9 (7.86 to 17.4)	0.04 (0.03 to 0.06)	13.4 (8.70 to 19.4)	0.38 (0.36 to 0.40)	0.25 (0.17 to 0.35)	312 (211 to 435)	1.56 (1.06 to 2.20)	711 (478 to 1007)	2.70 (2.67 to 2.72)
Somalia	2.76 (1.87 to 3.81)	169 (116 to 231)	13.4 (9.15 to 18.6)	286 (197 to 400)	1.70 (1.69 to 1.71)	0.45 (0.30 to 0.67)	14.8 (10.0 to 21.7)	1.20 (0.79 to 1.75)	14.4 (9.60 to 20.6)	-0.06 (-0.08 to -0.04)	2.31 (1.55 to 3.22)	154 (105 to 211)	12.2 (8.25 to 17.2)	271 (187 to 379)	1.83 (1.82 to 1.84)
South Africa	39.7 (27.9 to 54.2)	312 (220 to 425)	165 (115 to 224)	592 (414 to 804)	2.08 (2.04 to 2.11)	2.46 (1.55 to 3.65)	15.7 (9.91 to 23.3)	4.63 (2.98 to 6.91)	15.7 (10.0 to 23.3)	-0.00 (-0.01 to 0.01)	37.2 (26.1 to 51.0)	296 (210 to 405)	160 (111 to 218)	577 (402 to 786)	2.16 (2.12 to 2.19)
South Sudan	1.87 (1.28 to 2.59)	146 (101 to 200)	5.94 (3.98 to 8.34)	244 (165 to 337)	1.65 (1.64 to 1.65)	0.26 (0.17 to 0.37)	11.9 (7.97 to 17.3)	0.46 (0.29 to 0.67)	11.6 (7.59 to 16.9)	-0.05 (-0.07 to -0.03)	1.61 (1.11 to 2.25)	135 (92.5 to 185)	5.49 (3.68 to 7.72)	232 (156 to 322)	1.76 (1.75 to 1.77)
Spain	85.2 (57.0 to 120)	294 (199 to 413)	224 (153 to 322)	523 (360 to 755)	1.84 (1.81 to 1.87)	5.44 (3.69 to 7.64)	23.2 (15.7 to 32.9)	13.7 (9.34 to 19.4)	42.4 (28.7 to 59.7)	1.93 (1.87 to 1.98)	79.8 (53.3 to 114)	271 (183 to 385)	211 (144 to 304)	481 (329 to 698)	1.83 (1.80 to 1.86)
Sri Lanka	23.3 (15.8 to 31.8)	423 (285 to 576)	149 (100 to 210)	991 (669 to 1397)	2.79 (2.78 to 2.80)	1.19 (0.76 to 1.72)	15.9 (10.3 to 22.5)	2.42 (1.61 to 3.53)	18.2 (12.0 to 26.3)	0.45 (0.42 to 0.47)	22.1 (15.0 to 30.3)	407 (273 to 556)	146 (98.6 to 207)	973 (656 to 1374)	2.85 (2.85 to 2.86)
Sudan	12.2 (8.35 to 16.8)	242 (165 to 335)	64.5 (43.4 to 90.7)	559 (385 to 777)	2.71 (2.69 to 2.73)	0.79 (0.50 to 1.15)	10.1 (6.51 to 14.6)	2.04 (1.30 to 3.02)	11.4 (7.39 to 17.1)	0.40 (0.39 to 0.42)	11.4 (7.82 to 15.9)	232 (158 to 321)	62.4 (42.2 to 87.4)	547 (377 to 760)	2.78 (2.76 to 2.80)
Suriname	0.80 (0.53 to 1.14)	561 (372 to 795)	3.78 (2.56 to 5.25)	1102 (748 to 1521)	2.19 (2.17 to 2.21)	0.03 (0.02 to 0.04)	16.4 (11.1 to 23.7)	0.05 (0.03 to 0.07)	14.4 (9.68 to 20.6)	-0.39 (-0.41 to -0.37)	0.77 (0.51 to 1.10)	544 (362 to 771)	3.74 (2.53 to 5.19)	1087 (737 to 1503)	2.25 (2.23 to 2.26)
Sweden	11.8 (8.27 to 16.6)	174 (121 to 244)	24.0 (16.7 to 34.0)	280 (191 to 399)	1.56 (1.52 to 1.62)	1.52 (1.02 to 2.23)	28.1 (18.6 to 41.8)	2.20 (1.44 to 3.24)	33.1 (21.5 to 48.5)	0.53 (0.52 to 0.54)	10.3 (7.17 to 14.5)	146 (101 to 204)	21.8 (15.3 to 31.1)	247 (170 to 354)	1.71 (1.66 to 1.76)
Switzerland	12.0 (8.29 to 16.7)	229 (157 to 322)	33.3 (22.8 to 47.6)	435 (297 to 627)	2.12 (2.10 to 2.14)	0.82 (0.53 to 1.21)	18.9 (12.0 to 28.2)	1.78 (1.14 to 2.62)	29.0 (18.4 to 43.0)	1.38 (1.35 to 1.41)	11.2 (7.72 to 15.7)	210 (144 to 295)	31.5 (21.7 to 45.0)	406 (277 to 588)	2.17 (2.15 to 2.20)
Syrian Arab Republic	9.89 (6.70 to 13.9)	339 (232 to 477)	48.4 (33.4 to 68.2)	679 (470 to 946)	2.26 (2.26 to 2.27)	0.93 (0.60 to 1.40)	21.4 (14.3 to 32.0)	1.68 (1.07 to 2.47)	22.3 (14.2 to 32.5)	0.15 (0.13 to 0.17)	8.96 (6.10 to 12.6)	318 (216 to 445)	46.7 (32.2 to 65.9)	656 (456 to 919)	2.37 (2.36 to 2.37)
Taiwan (Province of China)	28.1 (18.7 to 39.3)	347 (233 to 482)	108 (74.3 to 150)	507 (345 to 698)	1.22 (1.21 to 1.24)	1.40 (0.87 to 2.12)	14.7 (9.24 to 22.4)	2.66 (1.76 to 3.85)	15.6 (10.2 to 22.6)	0.23 (0.18 to 0.29)	26.7 (17.8 to 37.3)	333 (223 to 461)	106 (72.1 to 147)	491 (333 to 678)	1.26 (1.25 to 1.27)
Tajikistan	2.48 (1.68 to 3.52)	151 (101 to 213)	13.6 (9.23 to 19.5)	375 (255 to 538)	2.96 (2.94 to 2.98)	0.24 (0.15 to 0.34)	11.3 (7.46 to 16.5)	0.68 (0.44 to 0.98)	15.3 (10.2 to 22.1)	0.98 (0.96 to 0.99)	2.24 (1.50 to 3.15)	140 (93.0 to 196)	12.9 (8.72 to 18.6)	360 (246 to 517)	3.08 (3.06 to 3.10)
Thailand	46.9 (32.0 to 65.9)	229 (156 to 321)	270 (184 to 381)	470 (318 to 660)	2.38 (2.36 to 2.41)	3.82 (2.48 to 5.60)	14.1 (9.17 to 20.5)	5.50 (3.57 to 8.00)	12.1 (7.86 to 17.5)	-0.41 (-0.46 to -0.31)	43.1 (29.5 to 61.0)	215 (146 to 302)	264 (180 to 374)	458 (309 to 645)	2.50 (2.47 to 2.53)
Timor-Leste	0.29 (0.19 to 0.40)	175 (119 to 244)	2.41 (1.63 to 3.39)	534 (361 to 746)	3.68 (3.65 to 3.70)	0.03 (0.02 to 0.05)	10.8 (6.95 to 15.9)	0.06 (0.04 to 0.10)	11.3 (7.15 to 16.7)	0.14 (0.12 to 0.15)	0.26 (0.17 to 0.37)	165 (111 to 230)	2.34 (1.58 to 3.30)	522 (353 to 730)	3.82 (3.79 to 3.84)
Togo	1.35 (0.92 to 1.90)	166 (114 to 233)	8.04 (5.49 to 11.1)	313 (213 to 436)	2.05 (2.04 to 2.06)	0.15 (0.09 to 0.21)	11.4 (7.42 to 16.6)	0.40 (0.26 to 0.61)	11.5 (7.49 to 17.3)	0.00 (-0.01 to 0.02)	1.21 (0.82 to 1.71)	155 (106 to 215)	7.65 (5.20 to 10.6)	302 (205 to 420)	2.16 (2.15 to 2.18)
Tokelau	0.005 (0.003 to 0.007)	695 (471 to 986)	0.010 (0.007 to 0.01)	1321 (888 to 1854)	2.10 (2.08 to 2.11)	8e-05 (5e-05 to 1e-04)	11.5 (7.48 to 16.6)	8e-05 (5e-05 to 1e-04)	11.3 (7.40 to 16.4)	-0.03 (-0.06 to -0.00)	0.005 (0.003 to 0.007)	683 (462 to 972)	0.010 (0.006 to 0.01)	1310 (880 to 1839)	2.12 (2.11 to 2.14)
Tonga	0.21 (0.14 to 0.29)	657 (442 to 913)	0.54 (0.37 to 0.77)	1223 (835 to 1741)	2.03 (2.02 to 2.04)	0.004 (0.003 to 0.006)	9.98 (6.57 to 14.2)	0.005 (0.003 to 0.007)	9.92 (6.28 to 14.6)	-0.02 (-0.03 to -0.01)	0.20 (0.14 to 0.28)	647 (435 to 901)	0.54 (0.37 to 0.76)	1213 (828 to 1730)	2.05 (2.04 to 2.06)
Trinidad and Tobago	4.10 (2.75 to 5.68)	899 (603 to 1250)	12.0 (7.95 to 16.9)	1229 (819 to 1742)	1.00 (0.99 to 1.01)	0.14 (0.09 to 0.19)	24.5 (16.5 to 35.1)	0.16 (0.10 to 0.22)	19.1 (12.3 to 27.2)	-0.76 (-0.79 to -0.73)	3.96 (2.66 to 5.50)	874 (586 to 1216)	11.8 (7.85 to 16.7)	1210 (806 to 1720)	1.04 (1.03 to 1.05)
Tunisia	7.43 (5.15 to 10.8)	279 (193 to 402)	50.8 (34.4 to 72.6)	724 (491 to 1034)	3.12 (3.10 to 3.13)	0.44 (0.29 to 0.65)	12.6 (8.37 to 18.5)	0.94 (0.61 to 1.39)	14.1 (9.15 to 20.9)	0.33 (0.32 to 0.35)	6.98 (4.81 to 10.2)	266 (184 to 385)	49.8 (33.8 to 71.1)	710 (481 to 1012)	3.20 (3.19 to 3.22)
Turkmenistan	1.57 (1.06 to 2.24)	130 (87.7 to 185)	7.63 (5.19 to 10.8)	317 (215 to 447)	2.92 (2.91 to 2.94)	0.19 (0.13 to 0.29)	12.6 (8.45 to 18.3)	0.47 (0.31 to 0.68)	18.9 (12.2 to 27.3)	1.30 (1.28 to 1.32)	1.38 (0.92 to 1.96)	117 (79.1 to 167)	7.16 (4.85 to 10.2)	298 (201 to 422)	3.06 (3.04 to 3.07)
Tuvalu	0.02 (0.01 to 0.03)	460 (313 to 640)	0.06 (0.04 to 0.08)	992 (679 to 1389)	2.49 (2.47 to 2.51)	6e-04 (4e-04 to 8e-04)	12.6 (8.21 to 18.2)	7e-04 (5e-04 to 0.001)	12.2 (8.18 to 17.7)	-0.12 (-0.13 to -0.10)	0.02 (0.01 to 0.03)	448 (304 to 621)	0.06 (0.04 to 0.08)	980 (671 to 1374)	2.54 (2.52 to 2.56)
Türkiye	51.2 (34.5 to 72.7)	264 (177 to 372)	298 (200 to 419)	595 (398 to 835)	2.63 (2.54 to 2.69)	4.31 (2.81 to 6.43)	17.8 (11.4 to 26.7)	8.78 (5.81 to 13.0)	18.7 (12.4 to 27.7)	0.15 (0.13 to 0.16)	46.9 (31.6 to 66.4)	246 (166 to 347)	290 (193 to 408)	577 (385 to 812)	2.75 (2.65 to 2.80)
Uganda	5.16 (3.49 to 7.05)	134 (91.0 to 185)	22.0 (14.9 to 30.8)	213 (146 to 298)	1.49 (1.48 to 1.50)	0.77 (0.49 to 1.12)	11.9 (7.77 to 17.0)	2.06 (1.35 to 3.00)	12.3 (8.27 to 17.9)	0.14 (0.12 to 0.16)	4.39 (2.99 to 6.04)	122 (83.1 to 170)	20.0 (13.5 to 28.1)	201 (137 to 282)	1.60 (1.59 to 1.61)
Ukraine	68.6 (48.1 to 95.8)	165 (115 to 229)	125 (86.6 to 172)	296 (208 to 408)	1.92 (1.91 to 1.94)	4.32 (2.67 to 6.39)	12.6 (7.96 to 19.1)	5.95 (3.76 to 8.64)	18.3 (11.4 to 27.1)	1.24 (1.22 to 1.25)	64.3 (44.8 to 88.9)	152 (106 to 209)	119 (82.4 to 165)	278 (194 to 384)	1.97 (1.96 to 1.99)
United Arab Emirates	0.88 (0.60 to 1.23)	448 (304 to 626)	12.7 (8.47 to 18.3)	717 (488 to 1019)	1.54 (1.49 to 1.58)	0.09 (0.06 to 0.14)	22.1 (14.2 to 32.7)	0.64 (0.41 to 0.98)	23.5 (15.3 to 35.0)	0.23 (0.20 to 0.26)	0.78 (0.53 to 1.11)	426 (289 to 593)	12.0 (8.06 to 17.3)	693 (471 to 986)	1.60 (1.54 to 1.64)
United Kingdom	75.4 (52.6 to 105)	175 (120 to 246)	224 (155 to 312)	433 (296 to 606)	2.95 (2.89 to 3.01)	6.77 (4.45 to 9.83)	19.0 (12.4 to 27.8)	10.1 (6.54 to 14.9)	22.4 (14.3 to 33.3)	0.53 (0.48 to 0.57)	68.7 (48.1 to 95.6)	156 (108 to 218)	214 (148 to 298)	410 (280 to 572)	3.17 (3.10 to 3.23)
United Republic of Tanzania	8.02 (5.50 to 10.9)	124 (85.2 to 170)	40.3 (27.5 to 55.7)	248 (170 to 342)	2.24 (2.23 to 2.26)	1.62 (0.98 to 2.45)	15.2 (9.36 to 22.1)	4.01 (2.40 to 6.08)	15.9 (9.67 to 23.4)	0.09 (-0.03 to 0.17)	6.41 (4.42 to 8.95)	109 (75.2 to 150)	36.3 (24.9 to 50.6)	232 (158 to 322)	2.48 (2.47 to 2.49)
United States Virgin Islands	0.38 (0.26 to 0.54)	777 (523 to 1094)	1.05 (0.71 to 1.49)	1251 (852 to 1772)	1.55 (1.54 to 1.57)	0.01 (0.009 to 0.02)	25.7 (17.1 to 37.1)	0.01 (0.009 to 0.02)	22.3 (14.8 to 32.5)	-0.44 (-0.46 to -0.42)	0.37 (0.25 to 0.52)	752 (504 to 1057)	1.04 (0.70 to 1.47)	1229 (836 to 1742)	1.60 (1.59 to 1.62)
United States of America	397 (280 to 548)	240 (169 to 330)	1620 (1142 to 2187)	578 (408 to 780)	2.88 (2.86 to 2.91)	40.2 (26.7 to 58.0)	27.3 (18.1 to 39.6)	68.9 (47.1 to 97.7)	31.8 (21.5 to 45.0)	0.48 (0.47 to 0.50)	357 (251 to 493)	212 (148 to 294)	1552 (1090 to 2100)	546 (385 to 737)	3.10 (3.07 to 3.13)
Uruguay	3.39 (2.30 to 4.68)	163 (112 to 225)	10.7 (7.26 to 15.2)	383 (262 to 546)	2.79 (2.73 to 2.83)	0.33 (0.21 to 0.48)	18.5 (12.1 to 27.8)	0.48 (0.31 to 0.71)	21.8 (14.0 to 32.7)	0.50 (0.48 to 0.52)	3.06 (2.06 to 4.28)	144 (97.9 to 202)	10.2 (6.88 to 14.5)	361 (246 to 514)	2.99 (2.93 to 3.04)
Uzbekistan	11.8 (7.99 to 16.5)	168 (113 to 236)	79.5 (53.6 to 110)	498 (337 to 691)	3.58 (3.56 to 3.60)	1.40 (0.93 to 2.04)	16.3 (11.0 to 23.7)	4.40 (2.91 to 6.50)	25.5 (17.0 to 37.5)	1.45 (1.42 to 1.48)	10.4 (7.00 to 14.6)	152 (102 to 214)	75.1 (50.5 to 105)	472 (319 to 659)	3.75 (3.73 to 3.77)
Vanuatu	0.14 (0.09 to 0.20)	372 (253 to 528)	0.97 (0.67 to 1.37)	895 (606 to 1257)	2.86 (2.85 to 2.87)	0.006 (0.004 to 0.009)	10.2 (6.76 to 15.1)	0.01 (0.010 to 0.02)	10.5 (7.04 to 15.4)	0.10 (0.08 to 0.12)	0.13 (0.09 to 0.19)	362 (246 to 514)	0.95 (0.66 to 1.35)	884 (599 to 1243)	2.92 (2.91 to 2.92)
Venezuela (Bolivarian Republic of)	21.5 (14.5 to 30.5)	371 (251 to 527)	102 (69.0 to 141)	627 (426 to 871)	1.72 (1.67 to 1.78)	0.61 (0.39 to 0.89)	7.61 (4.85 to 11.0)	1.15 (0.73 to 1.77)	7.66 (4.86 to 11.7)	0.02 (-0.01 to 0.05)	20.9 (14.1 to 29.5)	364 (245 to 517)	101 (68.2 to 140)	619 (421 to 859)	1.75 (1.70 to 1.81)
Viet Nam	52.3 (35.3 to 72.9)	219 (148 to 305)	230 (154 to 317)	414 (279 to 570)	2.07 (2.03 to 2.09)	4.20 (2.69 to 6.18)	14.5 (9.36 to 21.3)	8.78 (5.62 to 13.0)	15.8 (10.0 to 23.1)	0.28 (0.26 to 0.29)	48.1 (32.6 to 67.0)	204 (139 to 285)	221 (148 to 306)	398 (267 to 550)	2.16 (2.12 to 2.19)
Yemen	6.28 (4.25 to 8.97)	215 (145 to 305)	40.4 (27.9 to 57.6)	463 (322 to 655)	2.51 (2.48 to 2.55)	0.49 (0.31 to 0.74)	11.0 (7.06 to 16.3)	1.63 (1.02 to 2.44)	12.4 (7.74 to 18.4)	0.35 (0.32 to 0.38)	5.79 (3.93 to 8.30)	204 (139 to 290)	38.8 (26.9 to 55.1)	451 (313 to 637)	2.58 (2.56 to 2.62)
Zambia	4.00 (2.78 to 5.58)	236 (163 to 326)	19.0 (12.9 to 26.6)	385 (265 to 542)	1.58 (1.55 to 1.61)	0.45 (0.29 to 0.66)	14.3 (9.70 to 20.5)	1.01 (0.67 to 1.43)	12.8 (8.43 to 18.4)	-0.30 (-0.34 to -0.26)	3.55 (2.42 to 4.91)	221 (152 to 307)	18.0 (12.1 to 25.2)	373 (256 to 522)	1.68 (1.66 to 1.71)
Zimbabwe	4.91 (3.36 to 6.76)	210 (142 to 294)	20.6 (14.0 to 29.7)	457 (315 to 654)	2.54 (2.53 to 2.55)	0.33 (0.21 to 0.49)	9.35 (5.99 to 13.9)	0.66 (0.43 to 1.01)	10.5 (6.80 to 15.6)	0.31 (0.27 to 0.36)	4.58 (3.11 to 6.38)	201 (135 to 282)	19.9 (13.5 to 28.8)	446 (307 to 640)	2.62 (2.60 to 2.63)

AAPC: Average annual percentage change; ASR: Age-standardized rate; DM: Diabetes mellitus; SDI: Sociodemographic index; T1D: Type 1 diabetes; T2D: Type 2 diabetes; YLD: Years lived with disability. Results assuming first-order autocorrelation estimated from the data were broadly similar to those of the primary analyses, despite somewhat different estimates.

Table S5. YLL number, age-standardized rate, and AAPC of diabetes and two subtypes among **women** globally and by different SDI, regions, countries, and territories, 1990-2021

Location	DM, Number in 1990 (000s)	DM, ASR in 1990 (per 100,000)	DM, Number in 2021 (000s)	DM, ASR in 2021 (per 100,000)	DM, AAPC (95%CI)	T1D, Number in 1990 (000s)	T1D, ASR in 1990 (per 100,000)	T1D, Number in 2021 (000s)	T1D, ASR in 2021 (per 100,000)	T1D, AAPC (95%CI)	T2D, Number in 1990 (000s)	T2D, ASR in 1990 (per 100,000)	T2D, Number in 2021 (000s)	T2D, ASR in 2021 (per 100,000)	T2D, AAPC (95%CI)
Global	8777 (8267 to 9365)	403 (379 to 430)	18711 (17462 to 19948)	411 (384 to 438)	0.07 (0.04 to 0.10)	941 (807 to 1216)	36.5 (31.3 to 47.4)	1004 (865 to 1147)	25.2 (21.7 to 28.7)	-1.18 (-1.21 to -1.15)	7836 (7328 to 8264)	367 (343 to 386)	17707 (16549 to 18919)	386 (361 to 413)	0.17 (0.14 to 0.20)
**By SDI**															
High SDI	1628 (1519 to 1687)	259 (243 to 268)	1612 (1422 to 1709)	148 (135 to 155)	-1.78 (-1.83 to -1.74)	135 (131 to 139)	26.4 (25.6 to 27.2)	88.3 (84.6 to 91.8)	14.3 (13.8 to 14.8)	-2.01 (-2.11 to -1.95)	1493 (1388 to 1550)	233 (217 to 241)	1524 (1335 to 1621)	133 (120 to 140)	-1.77 (-1.82 to -1.73)
High-middle SDI	1534 (1425 to 1640)	275 (255 to 294)	2476 (2220 to 2724)	230 (207 to 252)	-0.57 (-0.63 to -0.53)	164 (141 to 194)	30.3 (25.9 to 35.9)	94.1 (84.0 to 110)	13.4 (12.1 to 15.5)	-2.53 (-2.63 to -2.40)	1369 (1268 to 1459)	245 (226 to 261)	2382 (2133 to 2618)	216 (194 to 237)	-0.39 (-0.44 to -0.34)
Middle SDI	2780 (2577 to 2981)	503 (466 to 538)	6997 (6470 to 7471)	496 (459 to 530)	-0.01 (-0.04 to 0.03)	282 (239 to 357)	35.4 (30.0 to 45.7)	291 (252 to 340)	22.8 (19.8 to 26.5)	-1.37 (-1.40 to -1.33)	2498 (2316 to 2677)	468 (433 to 499)	6706 (6205 to 7163)	473 (438 to 506)	0.08 (0.04 to 0.11)
Low-middle SDI	1900 (1717 to 2107)	588 (532 to 647)	5547 (4998 to 6121)	736 (663 to 810)	0.73 (0.68 to 0.79)	240 (196 to 351)	46.2 (37.4 to 70.8)	339 (275 to 404)	36.0 (29.2 to 43.2)	-0.77 (-0.81 to -0.73)	1660 (1503 to 1816)	541 (488 to 594)	5207 (4687 to 5761)	700 (632 to 771)	0.84 (0.78 to 0.90)
Low SDI	921 (811 to 1068)	743 (659 to 860)	2056 (1798 to 2332)	744 (657 to 844)	0.02 (0.00 to 0.04)	118 (87.4 to 171)	47.9 (37.4 to 72.6)	190 (146 to 234)	35.1 (27.5 to 43.0)	-0.99 (-1.02 to -0.96)	802 (712 to 914)	695 (618 to 793)	1866 (1632 to 2123)	709 (626 to 803)	0.08 (0.06 to 0.10)
**Four world regions**															
Africa	1144 (1030 to 1276)	736 (669 to 815)	2969 (2645 to 3311)	851 (768 to 943)	0.45 (0.39 to 0.50)	120 (87.3 to 166)	35.4 (27.6 to 48.3)	176 (129 to 215)	24.6 (18.7 to 30.2)	-1.17 (-1.18 to -1.15)	1024 (919 to 1133)	701 (636 to 772)	2793 (2476 to 3119)	827 (744 to 915)	0.51 (0.46 to 0.57)
America	1831 (1753 to 1877)	544 (522 to 558)	3331 (3083 to 3555)	472 (439 to 504)	-0.44 (-0.51 to -0.38)	124 (119 to 132)	34.6 (33.2 to 36.8)	156 (146 to 167)	28.4 (26.4 to 30.5)	-0.60 (-0.70 to -0.52)	1707 (1632 to 1751)	510 (488 to 523)	3175 (2931 to 3392)	444 (411 to 474)	-0.44 (-0.51 to -0.38)
Asia	4256 (3841 to 4692)	391 (353 to 429)	10628 (9762 to 11577)	404 (371 to 440)	0.12 (0.09 to 0.14)	558 (460 to 784)	37.7 (31.1 to 53.9)	606 (511 to 721)	25.6 (21.5 to 30.4)	-1.23 (-1.26 to -1.19)	3698 (3337 to 4054)	353 (319 to 388)	10022 (9158 to 10938)	378 (346 to 413)	0.23 (0.21 to 0.26)
Europe	1510 (1416 to 1566)	246 (232 to 255)	1731 (1547 to 1843)	187 (170 to 199)	-0.91 (-1.00 to -0.84)	135 (128 to 144)	30.0 (28.0 to 32.5)	62.9 (58.3 to 67.9)	13.2 (12.2 to 14.2)	-2.63 (-2.79 to -2.50)	1374 (1281 to 1432)	216 (202 to 225)	1668 (1486 to 1778)	174 (157 to 185)	-0.71 (-0.78 to -0.65)
**Six WHO regions**															
African Region	970 (862 to 1093)	788 (706 to 882)	2376 (2115 to 2668)	852 (770 to 948)	0.26 (0.21 to 0.29)	97.4 (68.4 to 132)	36.3 (27.5 to 50.3)	144 (102 to 177)	24.0 (18.0 to 29.9)	-1.34 (-1.36 to -1.31)	872 (775 to 976)	751 (672 to 836)	2232 (1992 to 2498)	828 (748 to 921)	0.32 (0.27 to 0.35)
Region of the Americas	1831 (1753 to 1877)	544 (522 to 558)	3331 (3083 to 3555)	472 (439 to 504)	-0.44 (-0.51 to -0.38)	124 (119 to 132)	34.6 (33.2 to 36.8)	156 (146 to 167)	28.4 (26.4 to 30.5)	-0.60 (-0.70 to -0.52)	1707 (1632 to 1751)	510 (488 to 523)	3175 (2931 to 3392)	444 (411 to 474)	-0.44 (-0.51 to -0.38)
South-East Asia Region	2086 (1839 to 2410)	557 (487 to 640)	6070 (5449 to 6771)	646 (582 to 720)	0.49 (0.42 to 0.55)	278 (222 to 429)	48.3 (38.4 to 76.5)	353 (294 to 435)	33.5 (27.9 to 41.3)	-1.16 (-1.23 to -1.06)	1808 (1592 to 2043)	508 (445 to 577)	5717 (5121 to 6394)	612 (550 to 684)	0.61 (0.54 to 0.68)
European Region	1568 (1472 to 1625)	247 (233 to 256)	1906 (1707 to 2028)	202 (183 to 215)	-0.69 (-0.77 to -0.62)	146 (138 to 155)	30.5 (28.6 to 32.8)	79.5 (73.5 to 85.6)	15.7 (14.6 to 16.9)	-2.12 (-2.24 to -2.01)	1422 (1328 to 1481)	217 (203 to 225)	1827 (1633 to 1946)	186 (168 to 198)	-0.50 (-0.57 to -0.45)
Eastern Mediterranean Region	538 (493 to 587)	588 (537 to 640)	1850 (1582 to 2138)	832 (718 to 955)	1.14 (1.09 to 1.17)	71.5 (57.5 to 96.1)	39.7 (32.5 to 52.9)	140 (105 to 177)	38.6 (29.2 to 48.7)	-0.07 (-0.10 to -0.04)	466 (426 to 510)	548 (500 to 599)	1710 (1460 to 1974)	793 (685 to 909)	1.21 (1.17 to 1.25)
Western Pacific Region	1681 (1483 to 1911)	272 (238 to 308)	3041 (2612 to 3499)	208 (179 to 239)	-0.88 (-0.91 to -0.84)	214 (170 to 275)	28.8 (22.9 to 37.5)	123 (97.3 to 159)	12.2 (9.93 to 15.4)	-2.75 (-2.80 to -2.71)	1467 (1290 to 1661)	243 (213 to 275)	2919 (2511 to 3374)	196 (169 to 226)	-0.71 (-0.74 to -0.67)
**Seven super regions**															
Southeast Asia, East Asia, and Oceania	2276 (2029 to 2582)	372 (330 to 422)	4681 (4142 to 5268)	318 (281 to 357)	-0.50 (-0.53 to -0.47)	286 (232 to 398)	35.9 (28.9 to 50.4)	207 (172 to 272)	17.9 (14.9 to 23.0)	-2.21 (-2.23 to -2.19)	1990 (1762 to 2260)	336 (297 to 380)	4474 (3957 to 5021)	300 (265 to 336)	-0.36 (-0.39 to -0.33)
Central Europe, Eastern Europe, and Central Asia	548 (530 to 562)	192 (186 to 196)	1082 (1003 to 1150)	283 (263 to 302)	1.33 (1.16 to 1.46)	89.7 (86.0 to 93.6)	38.0 (36.5 to 39.5)	59.3 (54.2 to 65.1)	26.2 (24.0 to 28.7)	-1.19 (-1.46 to -1.01)	459 (440 to 473)	154 (148 to 159)	1023 (947 to 1087)	257 (238 to 273)	1.74 (1.58 to 1.86)
South Asia	1527 (1315 to 1690)	524 (448 to 580)	5070 (4464 to 5752)	674 (593 to 763)	0.95 (0.87 to 1.06)	221 (174 to 323)	47.0 (35.8 to 71.5)	341 (263 to 426)	37.2 (28.6 to 46.9)	-0.67 (-0.75 to -0.57)	1307 (1123 to 1450)	477 (407 to 530)	4729 (4124 to 5395)	637 (557 to 723)	1.08 (0.99 to 1.20)
High-income	1809 (1685 to 1877)	262 (246 to 270)	1563 (1367 to 1672)	133 (121 to 139)	-2.16 (-2.19 to -2.12)	117 (114 to 120)	22.2 (21.7 to 22.7)	79.0 (76.4 to 81.0)	13.0 (12.6 to 13.3)	-1.73 (-1.79 to -1.67)	1692 (1570 to 1757)	240 (224 to 248)	1484 (1291 to 1592)	120 (108 to 126)	-2.21 (-2.24 to -2.19)
Latin America and Caribbean	1146 (1110 to 1177)	950 (915 to 977)	2580 (2359 to 2791)	766 (701 to 829)	-0.68 (-0.83 to -0.59)	67.6 (63.1 to 75.3)	36.6 (34.3 to 41.0)	99.5 (89.7 to 111)	31.5 (28.5 to 35.0)	-0.47 (-0.56 to -0.40)	1079 (1042 to 1109)	914 (878 to 940)	2480 (2269 to 2684)	735 (673 to 795)	-0.67 (-0.78 to -0.58)
North Africa and Middle East	508 (460 to 568)	577 (525 to 647)	1403 (1243 to 1610)	633 (564 to 722)	0.34 (0.26 to 0.40)	64.7 (50.0 to 96.9)	35.4 (27.4 to 51.1)	73.1 (56.9 to 93.7)	23.9 (18.6 to 30.9)	-1.17 (-1.22 to -1.13)	443 (405 to 495)	542 (494 to 605)	1330 (1175 to 1525)	609 (542 to 694)	0.41 (0.33 to 0.47)
Sub-Saharan Africa	962 (852 to 1088)	819 (729 to 920)	2333 (2076 to 2626)	881 (793 to 984)	0.23 (0.18 to 0.27)	95.1 (65.5 to 130)	36.6 (27.5 to 51.3)	145 (101 to 180)	24.4 (18.2 to 30.7)	-1.30 (-1.32 to -1.28)	867 (766 to 974)	782 (698 to 873)	2188 (1936 to 2467)	857 (770 to 960)	0.31 (0.27 to 0.34)
**21 regions**															
East Asia	1194 (1007 to 1415)	258 (218 to 306)	1989 (1598 to 2446)	175 (141 to 215)	-1.18 (-1.25 to -1.11)	180 (140 to 233)	31.3 (24.4 to 41.2)	84.6 (64.9 to 114)	10.6 (8.25 to 14.0)	-3.42 (-3.47 to -3.37)	1014 (849 to 1205)	227 (190 to 269)	1905 (1531 to 2345)	165 (132 to 202)	-0.97 (-1.04 to -0.89)
Oceania	32.3 (26.9 to 40.1)	2016 (1694 to 2482)	94.9 (79.3 to 114)	2375 (2009 to 2822)	0.47 (0.43 to 0.52)	1.32 (0.83 to 1.96)	37.6 (24.2 to 57.1)	2.85 (1.94 to 3.98)	38.8 (26.9 to 53.9)	0.23 (0.14 to 0.33)	31.0 (25.7 to 38.5)	1979 (1660 to 2439)	92.1 (77.1 to 111)	2336 (1976 to 2779)	0.48 (0.43 to 0.52)
Southeast Asia	1050 (916 to 1248)	721 (623 to 855)	2597 (2292 to 2971)	734 (648 to 839)	0.05 (0.02 to 0.07)	105 (84.1 to 168)	50.0 (39.7 to 79.9)	120 (94.3 to 157)	32.3 (25.7 to 42.0)	-1.38 (-1.41 to -1.36)	945 (822 to 1099)	671 (578 to 776)	2477 (2181 to 2842)	702 (620 to 801)	0.13 (0.11 to 0.16)
Central Asia	77.3 (72.5 to 82.1)	266 (250 to 283)	207 (182 to 233)	430 (380 to 483)	1.51 (1.32 to 1.64)	15.2 (14.0 to 16.4)	46.3 (42.5 to 50.2)	21.1 (18.1 to 24.1)	43.0 (37.1 to 49.1)	-0.24 (-0.38 to -0.13)	62.0 (57.5 to 66.5)	220 (204 to 236)	186 (163 to 210)	387 (342 to 436)	1.79 (1.61 to 1.94)
Central Europe	259 (248 to 268)	303 (291 to 314)	295 (267 to 318)	224 (205 to 243)	-0.88 (-1.02 to -0.78)	35.3 (33.5 to 36.6)	46.4 (44.2 to 48.1)	12.3 (10.9 to 14.5)	14.6 (13.0 to 17.0)	-3.66 (-3.75 to -3.58)	224 (214 to 232)	257 (245 to 266)	282 (256 to 305)	210 (191 to 227)	-0.66 (-0.75 to -0.55)
Eastern Europe	212 (206 to 217)	127 (124 to 130)	580 (527 to 629)	268 (244 to 290)	2.49 (1.85 to 2.91)	39.3 (37.3 to 42.0)	32.1 (30.8 to 33.9)	25.9 (23.2 to 28.9)	24.8 (22.3 to 27.6)	-0.40 (-0.75 to 0.02)	173 (166 to 178)	94.8 (90.8 to 97.9)	554 (502 to 601)	243 (221 to 264)	3.31 (2.75 to 3.73)
Australasia	24.7 (22.5 to 26.4)	190 (175 to 204)	32.8 (28.0 to 36.1)	112 (97.9 to 121)	-1.68 (-1.81 to -1.57)	1.83 (1.73 to 1.93)	16.0 (15.2 to 16.8)	1.31 (1.23 to 1.39)	7.36 (6.95 to 7.77)	-2.47 (-2.62 to -2.33)	22.8 (20.8 to 24.6)	174 (159 to 187)	31.5 (26.8 to 34.7)	104 (90.7 to 114)	-1.62 (-1.76 to -1.50)
High-income Asia Pacific	193 (180 to 205)	173 (161 to 183)	139 (111 to 159)	51.4 (43.5 to 57.6)	-3.93 (-4.06 to -3.82)	12.9 (11.0 to 14.2)	13.8 (11.9 to 15.2)	3.03 (2.61 to 3.80)	2.98 (2.62 to 3.67)	-4.81 (-4.90 to -4.72)	180 (167 to 192)	159 (147 to 169)	136 (109 to 155)	48.5 (40.8 to 54.4)	-3.87 (-3.99 to -3.75)
High-income North America	608 (565 to 633)	315 (297 to 326)	669 (611 to 705)	207 (193 to 217)	-1.28 (-1.35 to -1.21)	52.8 (51.5 to 53.9)	32.5 (31.8 to 33.1)	52.8 (50.9 to 54.5)	26.2 (25.3 to 27.1)	-0.67 (-0.77 to -0.60)	555 (513 to 580)	282 (265 to 294)	616 (559 to 652)	181 (167 to 190)	-1.38 (-1.45 to -1.32)
Southern Latin America	105 (99.6 to 110)	406 (386 to 425)	119 (109 to 126)	244 (227 to 258)	-1.60 (-1.74 to -1.46)	6.43 (6.16 to 6.71)	25.3 (24.3 to 26.4)	5.48 (5.17 to 5.79)	14.2 (13.4 to 15.0)	-1.88 (-1.99 to -1.78)	98.1 (93.1 to 103)	380 (361 to 400)	113 (104 to 121)	230 (213 to 244)	-1.51 (-1.69 to -1.36)
Western Europe	879 (809 to 917)	248 (231 to 258)	604 (499 to 661)	101 (87.1 to 108)	-2.87 (-2.93 to -2.83)	43.3 (41.9 to 44.6)	18.1 (17.7 to 18.5)	16.4 (15.3 to 17.1)	5.74 (5.53 to 5.91)	-3.66 (-3.76 to -3.57)	836 (767 to 873)	230 (213 to 240)	587 (483 to 644)	94.9 (81.5 to 102)	-2.82 (-2.88 to -2.78)
Andean Latin America	56.5 (50.4 to 63.2)	516 (460 to 577)	163 (136 to 197)	526 (439 to 635)	0.16 (-0.00 to 0.37)	3.14 (2.48 to 4.00)	16.8 (13.4 to 22.1)	4.09 (2.85 to 5.29)	12.3 (8.60 to 15.9)	-1.08 (-1.17 to -0.99)	53.4 (47.5 to 59.6)	499 (445 to 558)	159 (132 to 192)	514 (428 to 619)	0.19 (0.02 to 0.40)
Caribbean	171 (158 to 186)	1238 (1151 to 1344)	265 (223 to 311)	943 (794 to 1111)	-0.83 (-0.91 to -0.77)	16.5 (13.7 to 22.6)	96.5 (81.8 to 134)	19.4 (14.1 to 27.7)	79.3 (57.2 to 113)	-0.62 (-0.68 to -0.57)	154 (143 to 168)	1141 (1063 to 1236)	245 (208 to 288)	864 (729 to 1017)	-0.87 (-0.95 to -0.81)
Central Latin America	531 (517 to 543)	1181 (1143 to 1209)	1388 (1225 to 1550)	1018 (898 to 1137)	-0.45 (-0.57 to -0.33)	27.5 (26.5 to 28.6)	36.5 (35.4 to 37.7)	41.2 (35.6 to 46.4)	30.6 (26.4 to 34.5)	-0.50 (-0.64 to -0.34)	504 (490 to 516)	1144 (1107 to 1172)	1347 (1188 to 1505)	988 (870 to 1103)	-0.45 (-0.57 to -0.33)
Tropical Latin America	388 (370 to 400)	767 (723 to 793)	764 (700 to 803)	543 (498 to 571)	-1.10 (-1.17 to -1.03)	20.5 (19.5 to 21.7)	27.3 (26.1 to 28.7)	34.8 (33.2 to 36.3)	28.5 (27.1 to 29.8)	0.16 (0.06 to 0.27)	367 (350 to 379)	740 (697 to 766)	729 (666 to 768)	514 (470 to 542)	-1.15 (-1.23 to -1.09)
North Africa and Middle East	508 (460 to 568)	577 (525 to 647)	1403 (1243 to 1610)	633 (564 to 722)	0.34 (0.26 to 0.40)	64.7 (50.0 to 96.9)	35.4 (27.4 to 51.1)	73.1 (56.9 to 93.7)	23.9 (18.6 to 30.9)	-1.17 (-1.22 to -1.13)	443 (405 to 495)	542 (494 to 605)	1330 (1175 to 1525)	609 (542 to 694)	0.41 (0.33 to 0.47)
South Asia	1527 (1315 to 1690)	524 (448 to 580)	5070 (4464 to 5752)	674 (593 to 763)	0.95 (0.87 to 1.06)	221 (174 to 323)	47.0 (35.8 to 71.5)	341 (263 to 426)	37.2 (28.6 to 46.9)	-0.67 (-0.75 to -0.57)	1307 (1123 to 1450)	477 (407 to 530)	4729 (4124 to 5395)	637 (557 to 723)	1.08 (0.99 to 1.20)
Central Sub-Saharan Africa	116 (87.2 to 147)	902 (669 to 1140)	292 (222 to 376)	932 (701 to 1193)	0.09 (0.05 to 0.13)	7.81 (5.57 to 10.8)	30.3 (21.5 to 43.5)	15.6 (11.3 to 21.1)	24.8 (17.2 to 35.1)	-0.70 (-0.74 to -0.66)	108 (80.3 to 138)	871 (643 to 1108)	277 (210 to 360)	908 (680 to 1165)	0.11 (0.07 to 0.15)
Eastern Sub-Saharan Africa	398 (341 to 466)	924 (793 to 1073)	699 (603 to 818)	727 (630 to 850)	-0.77 (-0.79 to -0.74)	50.4 (34.3 to 73.7)	46.9 (35.0 to 73.0)	67.2 (47.4 to 85.4)	29.5 (21.4 to 38.5)	-1.48 (-1.50 to -1.46)	348 (292 to 411)	877 (748 to 1024)	632 (544 to 740)	698 (603 to 816)	-0.73 (-0.76 to -0.70)
Southern Sub-Saharan Africa	154 (140 to 169)	972 (880 to 1060)	543 (501 to 588)	1629 (1509 to 1759)	1.61 (1.40 to 1.80)	6.87 (5.08 to 9.39)	23.6 (18.1 to 31.5)	8.28 (5.96 to 10.2)	19.6 (14.1 to 24.2)	-0.70 (-0.83 to -0.56)	147 (134 to 160)	948 (857 to 1035)	534 (493 to 580)	1610 (1489 to 1740)	1.65 (1.44 to 1.84)
Western Sub-Saharan Africa	295 (240 to 348)	639 (524 to 754)	799 (638 to 983)	736 (613 to 890)	0.44 (0.42 to 0.46)	30.1 (18.8 to 40.9)	32.2 (21.8 to 42.5)	53.7 (33.8 to 70.9)	21.3 (13.8 to 28.0)	-1.35 (-1.37 to -1.33)	265 (216 to 313)	607 (499 to 716)	745 (598 to 915)	715 (593 to 863)	0.52 (0.49 to 0.54)
**204 countries and territories**															
Afghanistan	34.0 (25.3 to 51.1)	921 (689 to 1385)	90.6 (69.1 to 122)	1445 (1100 to 1957)	1.49 (1.47 to 1.51)	4.93 (2.76 to 8.83)	92.7 (52.7 to 166)	14.2 (9.89 to 22.7)	92.5 (60.6 to 144)	0.02 (-0.04 to 0.09)	29.1 (21.6 to 43.5)	828 (621 to 1220)	76.4 (56.7 to 106)	1352 (1018 to 1816)	1.64 (1.61 to 1.67)
Albania	1.21 (1.02 to 1.44)	109 (90.7 to 131)	1.96 (1.54 to 2.51)	86.1 (67.7 to 109)	-0.92 (-1.10 to -0.77)	0.32 (0.24 to 0.41)	22.8 (16.9 to 30.2)	0.18 (0.11 to 0.28)	10.3 (6.82 to 15.2)	-2.67 (-2.85 to -2.51)	0.90 (0.72 to 1.10)	86.2 (70.0 to 106)	1.78 (1.38 to 2.30)	75.8 (58.7 to 97.5)	-0.57 (-0.81 to -0.39)
Algeria	18.1 (14.0 to 24.5)	296 (229 to 393)	70.2 (56.0 to 85.4)	431 (347 to 522)	1.30 (1.25 to 1.35)	3.72 (2.37 to 6.51)	27.4 (17.2 to 45.7)	4.07 (2.88 to 5.79)	18.1 (12.8 to 26.0)	-1.31 (-1.34 to -1.26)	14.4 (10.9 to 19.5)	268 (205 to 363)	66.1 (52.5 to 81.1)	413 (332 to 503)	1.48 (1.43 to 1.53)
American Samoa	0.20 (0.17 to 0.23)	1724 (1490 to 1999)	0.66 (0.52 to 0.81)	2690 (2166 to 3287)	1.53 (1.39 to 1.65)	0.005 (0.003 to 0.007)	17.9 (12.3 to 26.8)	0.005 (0.003 to 0.006)	19.3 (13.3 to 26.7)	0.31 (0.22 to 0.39)	0.19 (0.17 to 0.22)	1706 (1476 to 1978)	0.65 (0.52 to 0.80)	2671 (2147 to 3259)	1.53 (1.40 to 1.66)
Andorra	0.04 (0.03 to 0.06)	158 (114 to 218)	0.09 (0.07 to 0.12)	111 (81.7 to 150)	-1.36 (-1.60 to -1.22)	0.006 (0.004 to 0.008)	20.0 (13.5 to 28.4)	0.006 (0.004 to 0.009)	10.9 (7.48 to 15.3)	-2.13 (-2.34 to -2.01)	0.04 (0.03 to 0.05)	138 (99.1 to 192)	0.09 (0.06 to 0.11)	100 (72.6 to 136)	-1.13 (-1.40 to -0.86)
Angola	16.6 (12.6 to 21.2)	766 (586 to 996)	53.2 (38.7 to 70.4)	764 (571 to 1015)	-0.04 (-0.15 to 0.06)	1.39 (0.95 to 2.02)	29.2 (20.0 to 43.3)	3.22 (2.14 to 4.64)	21.1 (13.6 to 30.9)	-0.97 (-1.08 to -0.83)	15.2 (11.5 to 19.9)	737 (564 to 960)	50.0 (36.3 to 66.7)	743 (554 to 988)	-0.01 (-0.12 to 0.08)
Antigua and Barbuda	0.42 (0.38 to 0.45)	1374 (1249 to 1502)	0.65 (0.61 to 0.69)	1157 (1089 to 1233)	-0.61 (-0.80 to -0.37)	0.03 (0.03 to 0.04)	104 (93.1 to 115)	0.03 (0.03 to 0.03)	54.3 (50.4 to 58.8)	-2.11 (-2.21 to -2.01)	0.38 (0.35 to 0.42)	1270 (1154 to 1391)	0.62 (0.58 to 0.66)	1103 (1037 to 1174)	-0.51 (-0.71 to -0.26)
Argentina	78.8 (74.3 to 83.4)	438 (414 to 463)	85.2 (78.6 to 91.3)	275 (255 to 293)	-1.49 (-1.63 to -1.32)	4.80 (4.52 to 5.08)	28.1 (26.5 to 29.7)	4.29 (4.01 to 4.59)	16.8 (15.7 to 17.9)	-1.68 (-1.82 to -1.56)	74.0 (69.5 to 78.5)	410 (385 to 435)	80.9 (74.4 to 86.8)	258 (238 to 276)	-1.48 (-1.63 to -1.31)
Armenia	8.49 (7.81 to 9.15)	526 (482 to 568)	8.13 (7.09 to 9.34)	339 (297 to 388)	-1.62 (-2.02 to -1.32)	1.28 (1.19 to 1.38)	73.7 (68.0 to 79.6)	0.73 (0.64 to 0.84)	41.0 (35.9 to 46.5)	-1.86 (-2.16 to -1.62)	7.20 (6.57 to 7.79)	452 (412 to 490)	7.40 (6.43 to 8.54)	298 (259 to 343)	-1.41 (-1.98 to -1.08)
Australia	20.1 (18.2 to 21.6)	184 (168 to 197)	27.9 (23.7 to 30.9)	111 (97.3 to 122)	-1.60 (-1.73 to -1.48)	1.35 (1.26 to 1.44)	14.3 (13.3 to 15.2)	1.18 (1.10 to 1.26)	7.93 (7.43 to 8.39)	-1.98 (-2.13 to -1.84)	18.7 (16.9 to 20.2)	170 (154 to 183)	26.7 (22.5 to 29.7)	104 (89.7 to 114)	-1.58 (-1.71 to -1.46)
Austria	19.0 (17.5 to 20.5)	245 (227 to 262)	13.3 (11.1 to 14.8)	113 (97.2 to 123)	-2.28 (-2.52 to -2.06)	0.86 (0.81 to 0.92)	17.0 (16.0 to 18.0)	0.30 (0.27 to 0.32)	4.77 (4.41 to 5.09)	-4.03 (-4.13 to -3.95)	18.2 (16.7 to 19.6)	228 (210 to 244)	13.0 (10.8 to 14.5)	108 (92.6 to 118)	-2.17 (-2.44 to -1.93)
Azerbaijan	10.1 (8.24 to 12.2)	321 (262 to 395)	26.4 (18.4 to 34.8)	444 (311 to 583)	1.07 (0.93 to 1.18)	2.23 (1.64 to 2.84)	61.8 (45.1 to 79.4)	2.88 (1.70 to 4.22)	49.9 (30.7 to 71.6)	-0.65 (-0.76 to -0.53)	7.82 (6.18 to 9.77)	259 (203 to 327)	23.5 (16.2 to 31.0)	394 (273 to 520)	1.37 (1.24 to 1.49)
Bahamas	1.16 (1.06 to 1.27)	1285 (1177 to 1400)	1.70 (1.37 to 2.11)	758 (613 to 937)	-1.77 (-2.24 to -1.54)	0.11 (0.10 to 0.13)	93.6 (85.0 to 103)	0.13 (0.10 to 0.16)	56.8 (44.3 to 74.0)	-1.60 (-1.73 to -1.48)	1.05 (0.96 to 1.15)	1192 (1090 to 1303)	1.57 (1.27 to 1.94)	701 (570 to 863)	-1.75 (-2.24 to -1.50)
Bahrain	1.46 (1.25 to 1.72)	1897 (1627 to 2241)	6.74 (5.54 to 7.87)	2156 (1789 to 2504)	0.38 (0.23 to 0.52)	0.11 (0.09 to 0.15)	52.5 (41.4 to 67.3)	0.14 (0.10 to 0.18)	24.2 (18.3 to 31.2)	-2.52 (-2.60 to -2.43)	1.35 (1.15 to 1.59)	1845 (1580 to 2185)	6.61 (5.41 to 7.72)	2132 (1767 to 2479)	0.43 (0.28 to 0.57)
Bangladesh	146 (119 to 170)	620 (516 to 716)	469 (367 to 587)	739 (577 to 923)	0.72 (0.59 to 0.84)	31.1 (21.0 to 47.8)	71.3 (48.0 to 111)	45.8 (31.1 to 62.2)	52.8 (35.4 to 71.5)	-0.84 (-0.93 to -0.74)	115 (94.2 to 136)	549 (454 to 633)	424 (329 to 535)	686 (535 to 860)	0.88 (0.73 to 1.01)
Barbados	2.86 (2.65 to 3.06)	1734 (1613 to 1858)	3.29 (2.64 to 4.00)	1183 (943 to 1447)	-1.10 (-1.47 to -0.83)	0.16 (0.15 to 0.18)	116 (107 to 125)	0.13 (0.10 to 0.17)	67.9 (52.1 to 86.1)	-1.72 (-1.86 to -1.62)	2.70 (2.50 to 2.89)	1617 (1503 to 1736)	3.15 (2.53 to 3.84)	1115 (890 to 1359)	-1.05 (-1.44 to -0.76)
Belarus	9.43 (8.81 to 10.1)	121 (114 to 129)	8.27 (6.73 to 10.0)	101 (81.7 to 123)	-0.14 (-0.54 to 0.25)	1.77 (1.57 to 2.02)	30.1 (27.3 to 33.6)	1.43 (1.13 to 1.78)	29.8 (23.8 to 37.0)	0.42 (-0.13 to 1.11)	7.67 (7.08 to 8.33)	91.2 (84.2 to 98.9)	6.84 (5.58 to 8.26)	71.0 (57.4 to 86.1)	-0.95 (-1.62 to -0.52)
Belgium	20.0 (18.1 to 21.9)	213 (195 to 231)	10.1 (8.05 to 11.4)	69.6 (58.0 to 77.0)	-3.54 (-3.75 to -3.35)	1.38 (1.29 to 1.48)	21.0 (19.8 to 22.3)	0.49 (0.44 to 0.54)	6.11 (5.64 to 6.57)	-4.14 (-4.31 to -4.00)	18.7 (16.8 to 20.5)	192 (174 to 209)	9.63 (7.60 to 10.9)	63.5 (52.1 to 70.7)	-3.49 (-3.71 to -3.28)
Belize	0.69 (0.64 to 0.74)	1379 (1280 to 1482)	2.20 (1.95 to 2.48)	1420 (1260 to 1600)	0.08 (-0.18 to 0.35)	0.07 (0.06 to 0.08)	77.9 (70.6 to 85.8)	0.10 (0.08 to 0.11)	46.9 (40.8 to 54.1)	-1.50 (-1.67 to -1.29)	0.62 (0.57 to 0.66)	1301 (1205 to 1399)	2.10 (1.86 to 2.37)	1374 (1220 to 1545)	0.15 (-0.11 to 0.43)
Benin	6.06 (4.84 to 7.49)	529 (423 to 641)	20.3 (15.9 to 25.3)	686 (543 to 846)	0.85 (0.80 to 0.90)	0.81 (0.51 to 1.21)	31.6 (20.9 to 45.2)	1.62 (1.02 to 2.45)	22.6 (14.7 to 33.1)	-1.05 (-1.10 to -1.00)	5.25 (4.20 to 6.32)	498 (396 to 600)	18.7 (14.3 to 23.4)	664 (524 to 818)	0.94 (0.89 to 0.99)
Bermuda	0.24 (0.22 to 0.26)	680 (619 to 743)	0.19 (0.16 to 0.25)	252 (206 to 317)	-3.28 (-3.47 to -3.12)	0.02 (0.02 to 0.02)	50.5 (44.9 to 56.2)	0.008 (0.006 to 0.010)	16.4 (13.2 to 20.4)	-3.66 (-3.85 to -3.49)	0.22 (0.20 to 0.24)	630 (573 to 687)	0.19 (0.15 to 0.24)	236 (192 to 297)	-3.25 (-3.45 to -3.07)
Bhutan	0.70 (0.52 to 0.92)	531 (390 to 695)	1.84 (1.32 to 2.42)	622 (448 to 810)	0.55 (0.50 to 0.60)	0.10 (0.05 to 0.18)	44.0 (24.5 to 77.8)	0.11 (0.07 to 0.17)	30.7 (18.8 to 45.8)	-1.18 (-1.27 to -1.07)	0.60 (0.43 to 0.79)	487 (359 to 642)	1.73 (1.25 to 2.25)	592 (428 to 766)	0.67 (0.63 to 0.71)
Bolivia (Plurinational State of)	19.9 (16.4 to 25.0)	1120 (923 to 1405)	49.2 (38.2 to 65.0)	1018 (796 to 1333)	-0.31 (-0.34 to -0.28)	1.13 (0.75 to 1.80)	36.0 (24.3 to 60.7)	1.29 (0.66 to 2.01)	22.1 (11.3 to 35.1)	-1.57 (-1.59 to -1.54)	18.8 (15.6 to 23.2)	1084 (897 to 1340)	47.9 (37.3 to 63.1)	996 (778 to 1315)	-0.27 (-0.31 to -0.25)
Bosnia and Herzegovina	8.95 (7.66 to 10.4)	392 (337 to 456)	20.4 (16.1 to 25.3)	563 (444 to 702)	1.26 (1.10 to 1.38)	1.36 (1.02 to 1.70)	57.1 (43.2 to 71.1)	0.67 (0.45 to 1.17)	26.8 (18.2 to 42.5)	-2.41 (-2.59 to -2.29)	7.58 (6.39 to 8.82)	335 (285 to 391)	19.8 (15.5 to 24.4)	536 (422 to 668)	1.60 (1.44 to 1.73)
Botswana	3.87 (2.68 to 5.21)	1236 (861 to 1635)	9.75 (7.58 to 12.4)	1223 (958 to 1539)	-0.19 (-0.47 to 0.11)	0.18 (0.11 to 0.28)	23.7 (13.9 to 35.4)	0.20 (0.12 to 0.31)	16.0 (9.13 to 23.9)	-1.23 (-1.33 to -1.13)	3.69 (2.51 to 4.99)	1212 (840 to 1601)	9.55 (7.43 to 12.1)	1207 (946 to 1520)	-0.17 (-0.46 to 0.13)
Brazil	380 (363 to 392)	770 (726 to 796)	727 (665 to 765)	528 (484 to 556)	-1.20 (-1.27 to -1.13)	19.9 (18.9 to 21.0)	27.3 (26.0 to 28.7)	34.0 (32.5 to 35.5)	28.7 (27.3 to 30.0)	0.18 (0.07 to 0.28)	360 (343 to 371)	742 (700 to 769)	693 (632 to 730)	500 (456 to 526)	-1.26 (-1.34 to -1.19)
Brunei Darussalam	0.91 (0.80 to 1.05)	1742 (1523 to 1993)	1.60 (1.36 to 1.88)	933 (795 to 1105)	-2.08 (-2.16 to -2.01)	0.11 (0.08 to 0.15)	99.2 (68.1 to 139)	0.09 (0.07 to 0.12)	36.3 (27.9 to 50.1)	-3.25 (-3.33 to -3.19)	0.81 (0.71 to 0.92)	1643 (1433 to 1882)	1.51 (1.27 to 1.79)	896 (761 to 1060)	-2.02 (-2.12 to -1.94)
Bulgaria	26.3 (24.3 to 28.6)	398 (369 to 430)	23.8 (20.8 to 27.0)	300 (260 to 344)	-0.89 (-1.07 to -0.69)	2.86 (2.56 to 3.17)	54.0 (49.1 to 59.5)	1.32 (1.06 to 1.64)	27.6 (22.4 to 33.8)	-2.25 (-2.38 to -2.10)	23.4 (21.5 to 25.5)	344 (316 to 373)	22.5 (19.6 to 25.5)	273 (236 to 312)	-0.72 (-0.89 to -0.53)
Burkina Faso	15.3 (12.2 to 19.2)	636 (510 to 795)	29.0 (22.6 to 36.5)	535 (424 to 666)	-0.58 (-0.62 to -0.54)	1.85 (1.15 to 2.74)	39.5 (27.2 to 56.9)	2.98 (1.82 to 4.40)	23.9 (15.3 to 34.5)	-1.60 (-1.66 to -1.55)	13.5 (10.8 to 17.1)	596 (479 to 744)	26.0 (19.9 to 33.0)	511 (402 to 639)	-0.50 (-0.54 to -0.46)
Burundi	14.5 (10.4 to 19.1)	1049 (736 to 1385)	22.0 (15.1 to 29.2)	850 (578 to 1140)	-0.69 (-0.74 to -0.65)	1.46 (0.94 to 2.13)	46.6 (31.6 to 68.0)	2.36 (1.47 to 3.27)	34.1 (21.8 to 49.5)	-1.01 (-1.06 to -0.96)	13.1 (9.04 to 17.4)	1002 (695 to 1324)	19.6 (13.2 to 26.2)	815 (554 to 1098)	-0.67 (-0.72 to -0.63)
Cabo Verde	0.30 (0.24 to 0.36)	216 (178 to 261)	1.54 (1.23 to 1.89)	606 (480 to 749)	3.39 (3.33 to 3.46)	0.03 (0.02 to 0.03)	18.0 (11.6 to 26.2)	0.03 (0.02 to 0.05)	11.6 (7.39 to 16.9)	-1.42 (-1.48 to -1.35)	0.26 (0.21 to 0.32)	198 (162 to 243)	1.51 (1.20 to 1.85)	595 (473 to 735)	3.62 (3.55 to 3.69)
Cambodia	21.2 (17.3 to 27.5)	724 (590 to 922)	53.7 (38.8 to 75.7)	727 (521 to 1007)	0.02 (-0.01 to 0.04)	2.87 (2.07 to 5.29)	61.6 (42.3 to 120)	3.22 (2.22 to 4.79)	37.9 (25.5 to 55.5)	-1.54 (-1.58 to -1.50)	18.3 (14.7 to 23.2)	663 (535 to 835)	50.5 (36.4 to 71.7)	689 (493 to 967)	0.13 (0.10 to 0.16)
Cameroon	18.4 (13.5 to 23.6)	747 (551 to 950)	64.5 (43.9 to 90.9)	933 (649 to 1316)	0.73 (0.70 to 0.75)	1.87 (1.23 to 2.60)	38.2 (24.7 to 55.4)	3.76 (2.27 to 5.65)	24.8 (15.4 to 39.6)	-1.37 (-1.43 to -1.34)	16.5 (12.1 to 21.3)	709 (519 to 903)	60.8 (41.4 to 85.9)	908 (632 to 1275)	0.81 (0.78 to 0.83)
Canada	43.5 (40.1 to 46.2)	238 (221 to 252)	45.7 (40.7 to 49.1)	123 (112 to 131)	-2.11 (-2.21 to -2.03)	5.74 (5.42 to 6.08)	35.3 (33.5 to 37.2)	4.88 (4.58 to 5.18)	21.0 (19.9 to 22.3)	-1.63 (-1.75 to -1.51)	37.8 (34.6 to 40.3)	203 (187 to 216)	40.8 (36.0 to 44.1)	102 (92.1 to 110)	-2.20 (-2.32 to -2.11)
Central African Republic	7.41 (5.56 to 9.42)	1118 (832 to 1416)	14.9 (10.8 to 20.1)	1144 (823 to 1534)	0.16 (0.11 to 0.22)	0.51 (0.36 to 0.78)	39.1 (28.2 to 62.8)	0.99 (0.65 to 1.40)	37.1 (25.3 to 54.7)	-0.18 (-0.25 to -0.11)	6.90 (5.15 to 8.86)	1079 (799 to 1370)	13.9 (9.85 to 18.9)	1107 (796 to 1486)	0.17 (0.12 to 0.23)
Chad	8.02 (6.14 to 10.4)	512 (390 to 671)	26.0 (19.4 to 34.5)	857 (655 to 1121)	1.71 (1.67 to 1.74)	0.96 (0.59 to 1.53)	31.2 (21.0 to 48.0)	2.69 (1.60 to 4.13)	30.1 (19.8 to 46.0)	-0.02 (-0.10 to 0.05)	7.06 (5.39 to 9.17)	481 (366 to 630)	23.3 (17.3 to 31.1)	827 (633 to 1080)	1.79 (1.76 to 1.83)
Chile	18.6 (17.5 to 19.7)	333 (313 to 354)	24.7 (22.2 to 26.7)	172 (156 to 185)	-2.21 (-2.53 to -1.95)	1.35 (1.28 to 1.42)	21.2 (20.1 to 22.3)	0.97 (0.91 to 1.04)	8.70 (8.17 to 9.27)	-2.82 (-2.95 to -2.71)	17.2 (16.2 to 18.4)	312 (292 to 333)	23.8 (21.3 to 25.7)	164 (147 to 176)	-2.17 (-2.50 to -1.90)

Location	DM, Number in 1990 (000s)	DM, ASR in 1990 (per 100,000)	DM, Number in 2021 (000s)	DM, ASR in 2021 (per 100,000)	DM, AAPC (95%CI)	T1D, Number in 1990 (000s)	T1D, ASR in 1990 (per 100,000)	T1D, Number in 2021 (000s)	T1D, ASR in 2021 (per 100,000)	T1D, AAPC (95%CI)	T2D, Number in 1990 (000s)	T2D, ASR in 1990 (per 100,000)	T2D, Number in 2021 (000s)	T2D, ASR in 2021 (per 100,000)	T2D, AAPC (95%CI)
China	1093 (905 to 1316)	245 (203 to 295)	1838 (1448 to 2290)	168 (133 to 208)	-1.19 (-1.26 to -1.13)	169 (128 to 221)	30.4 (22.9 to 40.6)	74.3 (53.8 to 103)	9.74 (7.20 to 13.2)	-3.63 (-3.68 to -3.57)	924 (759 to 1114)	214 (177 to 258)	1764 (1390 to 2194)	158 (125 to 196)	-0.95 (-1.02 to -0.89)
Colombia	51.6 (48.8 to 54.0)	543 (512 to 570)	80.4 (68.3 to 93.5)	266 (226 to 309)	-2.15 (-2.32 to -2.01)	2.29 (2.10 to 2.50)	14.3 (13.3 to 15.4)	2.67 (2.23 to 3.16)	10.2 (8.47 to 12.0)	-1.18 (-1.46 to -0.91)	49.3 (46.5 to 51.6)	529 (498 to 555)	77.7 (66.0 to 90.4)	256 (217 to 297)	-2.20 (-2.36 to -2.06)
Comoros	0.99 (0.66 to 1.38)	885 (603 to 1227)	2.16 (1.50 to 3.05)	808 (552 to 1139)	-0.31 (-0.40 to -0.19)	0.11 (0.07 to 0.16)	42.4 (27.2 to 60.2)	0.13 (0.08 to 0.19)	34.4 (21.5 to 50.8)	-0.78 (-1.12 to -0.46)	0.88 (0.57 to 1.25)	842 (570 to 1172)	2.03 (1.39 to 2.89)	773 (530 to 1095)	-0.30 (-0.38 to -0.20)
Congo	7.48 (5.79 to 9.71)	1207 (930 to 1551)	18.3 (13.6 to 24.0)	1227 (931 to 1589)	0.07 (-0.00 to 0.16)	0.36 (0.24 to 0.57)	33.4 (22.7 to 57.3)	0.71 (0.46 to 1.09)	27.1 (17.5 to 41.3)	-0.65 (-0.78 to -0.48)	7.13 (5.45 to 9.21)	1174 (902 to 1501)	17.5 (13.1 to 23.1)	1200 (911 to 1559)	0.08 (0.02 to 0.15)
Cook Islands	0.20 (0.17 to 0.24)	3291 (2753 to 3932)	0.29 (0.22 to 0.36)	2188 (1704 to 2762)	-1.31 (-1.34 to -1.29)	0.005 (0.004 to 0.007)	58.1 (40.8 to 80.6)	0.003 (0.002 to 0.004)	34.9 (23.6 to 50.3)	-1.54 (-1.81 to -1.33)	0.19 (0.16 to 0.23)	3233 (2713 to 3868)	0.28 (0.22 to 0.36)	2153 (1680 to 2724)	-1.31 (-1.33 to -1.28)
Costa Rica	3.79 (3.51 to 4.04)	412 (380 to 441)	10.9 (9.68 to 12.1)	365 (325 to 406)	-0.29 (-0.90 to 0.33)	0.15 (0.14 to 0.16)	10.7 (9.98 to 11.5)	0.28 (0.25 to 0.31)	10.2 (9.03 to 11.3)	-0.15 (-0.39 to 0.09)	3.65 (3.37 to 3.90)	402 (369 to 430)	10.6 (9.42 to 11.8)	355 (316 to 396)	-0.29 (-0.89 to 0.35)
Croatia	9.70 (8.88 to 10.5)	275 (252 to 297)	13.0 (11.3 to 14.5)	231 (200 to 256)	-0.75 (-0.95 to -0.59)	1.21 (1.11 to 1.32)	38.5 (35.8 to 41.7)	0.39 (0.33 to 0.47)	11.8 (9.85 to 13.8)	-3.75 (-3.89 to -3.61)	8.48 (7.68 to 9.22)	237 (214 to 258)	12.6 (10.9 to 14.1)	219 (190 to 243)	-0.46 (-0.66 to -0.29)
Cuba	32.6 (30.7 to 34.3)	627 (591 to 659)	22.2 (19.2 to 25.4)	216 (187 to 248)	-3.31 (-3.50 to -3.10)	2.23 (2.10 to 2.38)	39.7 (37.3 to 42.3)	1.02 (0.86 to 1.18)	14.5 (12.3 to 16.8)	-3.06 (-3.25 to -2.83)	30.3 (28.5 to 32.1)	587 (552 to 619)	21.2 (18.3 to 24.3)	202 (174 to 232)	-3.33 (-3.53 to -3.11)
Cyprus	4.39 (3.72 to 5.08)	1146 (973 to 1328)	3.89 (3.25 to 4.55)	373 (314 to 435)	-3.50 (-3.65 to -3.35)	0.21 (0.15 to 0.27)	52.2 (36.9 to 67.0)	0.13 (0.10 to 0.17)	14.3 (11.5 to 18.6)	-4.06 (-4.26 to -3.85)	4.17 (3.54 to 4.84)	1094 (929 to 1275)	3.76 (3.14 to 4.39)	358 (302 to 420)	-3.48 (-3.64 to -3.32)
Czechia	23.9 (22.0 to 26.1)	291 (269 to 318)	33.9 (29.2 to 38.8)	257 (220 to 294)	-0.22 (-0.54 to 0.16)	3.21 (3.02 to 3.43)	45.4 (42.6 to 48.5)	1.01 (0.87 to 1.15)	13.1 (11.2 to 14.8)	-4.02 (-4.22 to -3.85)	20.7 (18.9 to 22.8)	246 (225 to 271)	32.9 (28.3 to 37.7)	244 (209 to 280)	0.28 (-0.12 to 0.75)
Côte d'Ivoire	11.1 (8.50 to 14.1)	532 (414 to 676)	44.2 (32.3 to 59.4)	789 (589 to 1040)	1.25 (1.21 to 1.29)	1.61 (0.98 to 2.42)	27.9 (17.0 to 40.6)	2.87 (1.81 to 4.13)	21.6 (13.5 to 30.5)	-0.87 (-0.96 to -0.76)	9.45 (7.24 to 11.9)	504 (396 to 639)	41.3 (29.5 to 55.4)	767 (571 to 1012)	1.34 (1.30 to 1.37)
Democratic People's Republic of Korea	34.4 (24.9 to 44.6)	343 (251 to 440)	67.3 (50.6 to 91.5)	358 (270 to 488)	0.16 (0.14 to 0.17)	4.30 (2.73 to 6.82)	39.5 (24.9 to 63.2)	4.75 (2.81 to 7.96)	33.0 (19.9 to 54.0)	-0.58 (-0.60 to -0.56)	30.1 (21.4 to 38.7)	303 (221 to 390)	62.5 (46.7 to 85.5)	326 (243 to 447)	0.24 (0.23 to 0.25)
Democratic Republic of the Congo	79.9 (56.3 to 105)	889 (621 to 1166)	198 (138 to 265)	950 (655 to 1271)	0.24 (0.17 to 0.31)	5.37 (3.75 to 7.54)	29.8 (20.0 to 43.8)	10.4 (7.11 to 14.3)	25.4 (16.6 to 37.1)	-0.58 (-0.64 to -0.52)	74.5 (51.7 to 99.3)	859 (598 to 1125)	187 (129 to 252)	925 (637 to 1235)	0.26 (0.19 to 0.33)
Denmark	7.98 (7.39 to 8.54)	179 (167 to 190)	8.33 (7.18 to 9.14)	118 (105 to 128)	-1.39 (-1.59 to -1.16)	1.01 (0.95 to 1.08)	29.3 (27.6 to 31.0)	0.38 (0.34 to 0.42)	8.34 (7.66 to 9.04)	-3.99 (-4.19 to -3.80)	6.97 (6.42 to 7.47)	149 (139 to 160)	7.95 (6.81 to 8.75)	110 (96.7 to 120)	-1.03 (-1.22 to -0.82)
Djibouti	0.52 (0.38 to 0.69)	704 (523 to 939)	2.44 (1.61 to 3.65)	818 (559 to 1178)	0.46 (0.42 to 0.50)	0.06 (0.04 to 0.09)	30.2 (20.4 to 41.7)	0.15 (0.09 to 0.23)	25.6 (15.2 to 39.0)	-0.29 (-0.48 to -0.10)	0.46 (0.33 to 0.62)	674 (498 to 903)	2.29 (1.51 to 3.44)	792 (543 to 1140)	0.50 (0.46 to 0.53)
Dominica	0.56 (0.50 to 0.62)	1569 (1408 to 1739)	0.67 (0.57 to 0.79)	1518 (1287 to 1801)	-0.10 (-0.15 to -0.06)	0.03 (0.02 to 0.03)	73.0 (59.9 to 90.0)	0.02 (0.02 to 0.03)	64.3 (47.0 to 84.0)	-0.40 (-0.44 to -0.35)	0.54 (0.48 to 0.60)	1496 (1338 to 1662)	0.64 (0.55 to 0.76)	1454 (1238 to 1715)	-0.09 (-0.14 to -0.05)
Dominican Republic	12.2 (10.5 to 14.1)	595 (516 to 680)	35.3 (28.6 to 43.7)	675 (546 to 836)	0.42 (0.33 to 0.50)	1.69 (1.34 to 2.16)	48.4 (38.4 to 64.4)	1.97 (1.38 to 2.71)	35.6 (24.8 to 48.9)	-1.03 (-1.13 to -0.94)	10.5 (9.08 to 12.1)	546 (470 to 627)	33.4 (27.0 to 41.3)	639 (518 to 794)	0.53 (0.43 to 0.61)
Ecuador	16.2 (15.4 to 17.1)	583 (550 to 616)	49.0 (39.0 to 60.8)	569 (455 to 706)	-0.23 (-0.55 to 0.00)	0.68 (0.63 to 0.74)	14.4 (13.4 to 15.5)	1.37 (1.07 to 1.72)	15.0 (11.8 to 18.8)	0.26 (0.09 to 0.40)	15.5 (14.7 to 16.4)	569 (536 to 601)	47.6 (37.9 to 59.1)	554 (442 to 687)	-0.24 (-0.56 to -0.00)
Egypt	104 (93.6 to 117)	779 (698 to 866)	352 (281 to 429)	1404 (1172 to 1669)	1.96 (1.84 to 2.07)	9.35 (6.53 to 15.1)	30.2 (20.8 to 49.3)	12.8 (8.50 to 16.4)	25.5 (16.9 to 32.2)	-0.41 (-0.53 to -0.29)	95.0 (84.0 to 106)	749 (670 to 842)	340 (272 to 414)	1379 (1151 to 1642)	2.04 (1.91 to 2.14)
El Salvador	9.64 (8.74 to 10.6)	572 (518 to 629)	36.4 (29.1 to 45.2)	1026 (821 to 1278)	2.01 (1.73 to 2.26)	0.85 (0.66 to 1.12)	32.1 (25.4 to 42.3)	0.86 (0.56 to 1.20)	24.6 (16.2 to 34.0)	-0.80 (-0.95 to -0.61)	8.79 (8.00 to 9.66)	540 (491 to 596)	35.5 (28.3 to 44.0)	1001 (799 to 1243)	2.13 (1.92 to 2.36)
Equatorial Guinea	1.10 (0.80 to 1.55)	968 (717 to 1359)	2.84 (1.72 to 4.36)	937 (596 to 1411)	-0.03 (-0.16 to 0.09)	0.07 (0.05 to 0.12)	34.4 (22.9 to 60.1)	0.11 (0.06 to 0.19)	16.8 (9.08 to 27.7)	-2.25 (-2.34 to -2.15)	1.03 (0.75 to 1.46)	934 (687 to 1304)	2.74 (1.67 to 4.19)	921 (584 to 1388)	0.03 (-0.10 to 0.15)
Eritrea	7.64 (6.01 to 9.64)	1063 (835 to 1325)	18.7 (13.6 to 24.5)	1121 (818 to 1451)	0.15 (0.13 to 0.17)	0.76 (0.51 to 1.16)	43.7 (31.7 to 67.0)	1.50 (0.94 to 2.16)	44.4 (28.6 to 63.0)	0.04 (-0.02 to 0.10)	6.88 (5.23 to 8.79)	1020 (794 to 1279)	17.2 (12.3 to 22.7)	1077 (781 to 1392)	0.15 (0.13 to 0.17)
Estonia	1.43 (1.29 to 1.56)	120 (109 to 130)	3.03 (2.61 to 3.47)	205 (177 to 236)	1.91 (1.57 to 2.20)	0.41 (0.37 to 0.46)	42.8 (39.1 to 47.2)	0.24 (0.21 to 0.28)	37.4 (32.5 to 42.3)	-0.47 (-0.87 to -0.09)	1.02 (0.90 to 1.13)	76.8 (68.2 to 85.6)	2.79 (2.39 to 3.19)	168 (144 to 194)	2.73 (2.35 to 3.11)
Eswatini	2.55 (1.98 to 3.21)	1577 (1215 to 1983)	7.78 (5.10 to 10.9)	2323 (1568 to 3183)	1.25 (1.17 to 1.30)	0.12 (0.07 to 0.18)	25.1 (16.0 to 36.8)	0.16 (0.08 to 0.26)	25.9 (11.9 to 41.1)	0.11 (0.06 to 0.16)	2.43 (1.88 to 3.09)	1552 (1192 to 1957)	7.62 (4.98 to 10.7)	2297 (1552 to 3145)	1.26 (1.19 to 1.31)
Ethiopia	159 (131 to 199)	1422 (1176 to 1789)	154 (125 to 190)	650 (534 to 793)	-2.48 (-2.54 to -2.42)	20.6 (13.9 to 34.2)	75.6 (53.0 to 139)	16.6 (11.2 to 22.9)	28.8 (19.2 to 40.8)	-3.08 (-3.12 to -3.05)	139 (111 to 176)	1346 (1110 to 1701)	138 (112 to 171)	622 (511 to 756)	-2.45 (-2.51 to -2.39)
Fiji	8.42 (6.89 to 10.4)	4197 (3473 to 5146)	24.5 (19.2 to 31.5)	5843 (4585 to 7447)	1.02 (0.81 to 1.22)	0.20 (0.15 to 0.27)	52.8 (39.1 to 71.4)	0.21 (0.14 to 0.28)	44.3 (30.7 to 61.6)	-0.48 (-0.56 to -0.40)	8.22 (6.73 to 10.1)	4144 (3431 to 5081)	24.3 (19.0 to 31.3)	5798 (4548 to 7392)	1.03 (0.82 to 1.24)
Finland	6.03 (5.46 to 6.56)	134 (122 to 145)	3.98 (3.34 to 4.44)	55.5 (48.4 to 60.8)	-2.69 (-2.92 to -2.50)	1.29 (1.18 to 1.40)	34.3 (31.9 to 37.0)	0.43 (0.38 to 0.47)	9.89 (9.13 to 10.7)	-3.84 (-4.01 to -3.65)	4.74 (4.26 to 5.20)	99.7 (89.8 to 109)	3.56 (2.94 to 3.98)	45.6 (38.8 to 50.4)	-2.39 (-2.66 to -2.15)
France	74.3 (66.7 to 80.2)	143 (130 to 154)	82.0 (68.1 to 91.0)	84.8 (73.1 to 92.7)	-1.58 (-1.69 to -1.49)	4.65 (4.36 to 4.90)	12.8 (12.2 to 13.5)	2.30 (2.10 to 2.49)	5.04 (4.71 to 5.37)	-2.96 (-3.05 to -2.89)	69.6 (62.2 to 75.4)	130 (118 to 140)	79.6 (66.0 to 88.6)	79.7 (68.3 to 87.4)	-1.47 (-1.60 to -1.36)
Gabon	3.17 (2.34 to 4.03)	1008 (742 to 1296)	5.60 (3.73 to 7.61)	1016 (683 to 1358)	0.06 (-0.01 to 0.12)	0.12 (0.08 to 0.16)	26.7 (17.1 to 41.1)	0.16 (0.10 to 0.26)	18.0 (10.8 to 29.4)	-1.23 (-1.34 to -1.11)	3.05 (2.24 to 3.89)	981 (718 to 1260)	5.44 (3.62 to 7.35)	998 (670 to 1336)	0.09 (0.02 to 0.15)
Gambia	0.98 (0.72 to 1.31)	528 (398 to 703)	4.76 (3.51 to 6.26)	884 (647 to 1183)	1.79 (1.65 to 1.95)	0.13 (0.08 to 0.19)	27.6 (18.7 to 38.3)	0.29 (0.19 to 0.47)	24.6 (16.6 to 40.1)	-0.44 (-0.73 to -0.17)	0.85 (0.62 to 1.16)	500 (376 to 671)	4.47 (3.28 to 5.93)	859 (628 to 1149)	1.77 (1.55 to 1.94)
Georgia	9.78 (8.56 to 11.1)	260 (228 to 296)	11.9 (10.2 to 13.7)	348 (298 to 401)	1.07 (0.73 to 1.47)	1.50 (1.28 to 1.75)	46.6 (40.2 to 54.1)	0.79 (0.65 to 0.95)	35.8 (29.9 to 42.1)	-0.91 (-1.18 to -0.62)	8.28 (7.24 to 9.52)	213 (187 to 245)	11.1 (9.56 to 12.7)	312 (268 to 357)	1.38 (1.00 to 1.87)
Germany	231 (212 to 248)	279 (259 to 296)	140 (115 to 156)	115 (99.6 to 126)	-2.75 (-2.86 to -2.64)	13.1 (12.4 to 14.0)	23.9 (22.8 to 25.1)	4.16 (3.81 to 4.49)	7.13 (6.70 to 7.53)	-3.76 (-3.88 to -3.62)	218 (199 to 235)	255 (236 to 272)	136 (111 to 151)	108 (92.5 to 118)	-2.67 (-2.78 to -2.56)
Ghana	23.0 (17.9 to 29.4)	644 (499 to 821)	74.0 (56.1 to 97.8)	778 (593 to 1023)	0.59 (0.55 to 0.62)	1.61 (1.08 to 2.25)	21.6 (15.1 to 28.9)	2.60 (1.69 to 3.68)	15.2 (9.68 to 21.7)	-1.11 (-1.15 to -1.07)	21.3 (16.5 to 27.3)	623 (482 to 796)	71.4 (54.0 to 94.8)	762 (581 to 1004)	0.64 (0.59 to 0.67)
Greece	12.4 (11.2 to 13.4)	148 (135 to 160)	13.1 (11.3 to 14.6)	94.6 (84.4 to 104)	-1.41 (-1.60 to -1.20)	0.36 (0.33 to 0.38)	6.14 (5.78 to 6.53)	0.15 (0.14 to 0.17)	2.32 (2.15 to 2.52)	-3.20 (-3.30 to -3.10)	12.0 (10.9 to 13.0)	142 (129 to 153)	13.0 (11.2 to 14.4)	92.3 (82.2 to 102)	-1.35 (-1.54 to -1.13)
Greenland	0.05 (0.04 to 0.06)	298 (253 to 345)	0.04 (0.03 to 0.05)	130 (108 to 157)	-2.53 (-2.62 to -2.44)	0.003 (0.002 to 0.004)	11.0 (7.76 to 14.3)	0.001 (9e-04 to 0.002)	4.78 (3.24 to 6.56)	-2.67 (-2.81 to -2.55)	0.05 (0.04 to 0.06)	287 (243 to 332)	0.04 (0.03 to 0.05)	125 (103 to 152)	-2.52 (-2.62 to -2.43)
Grenada	0.90 (0.83 to 0.99)	2176 (1982 to 2377)	0.99 (0.87 to 1.11)	1657 (1459 to 1853)	-0.66 (-0.86 to -0.45)	0.04 (0.04 to 0.05)	107 (95.2 to 120)	0.03 (0.02 to 0.03)	55.1 (47.3 to 63.9)	-2.15 (-2.29 to -2.01)	0.86 (0.78 to 0.94)	2069 (1881 to 2261)	0.96 (0.84 to 1.07)	1602 (1409 to 1791)	-0.60 (-0.80 to -0.39)
Guam	0.40 (0.35 to 0.45)	1101 (964 to 1253)	0.54 (0.45 to 0.63)	497 (421 to 579)	-2.66 (-2.79 to -2.52)	0.006 (0.004 to 0.008)	8.02 (5.75 to 11.5)	0.002 (0.002 to 0.003)	3.30 (2.44 to 4.42)	-3.31 (-3.90 to -2.80)	0.39 (0.34 to 0.45)	1093 (957 to 1246)	0.53 (0.45 to 0.62)	494 (418 to 576)	-2.53 (-2.74 to -2.36)
Guatemala	10.8 (10.3 to 11.3)	506 (482 to 532)	105 (90.3 to 120)	1705 (1473 to 1949)	4.15 (3.79 to 4.51)	2.34 (2.18 to 2.52)	53.9 (50.5 to 57.4)	4.46 (3.74 to 5.25)	56.7 (47.3 to 66.6)	0.60 (-0.33 to 1.03)	8.44 (8.01 to 8.92)	452 (429 to 477)	101 (86.5 to 115)	1648 (1423 to 1884)	4.39 (4.02 to 4.81)
Guinea	12.2 (8.90 to 15.7)	697 (506 to 896)	28.8 (21.9 to 37.6)	955 (734 to 1244)	1.02 (0.99 to 1.04)	1.21 (0.73 to 1.84)	38.9 (24.7 to 58.6)	2.01 (1.25 to 2.95)	29.6 (19.3 to 43.1)	-0.86 (-0.92 to -0.79)	11.0 (8.02 to 14.2)	658 (477 to 847)	26.8 (20.3 to 35.1)	925 (706 to 1208)	1.10 (1.08 to 1.13)
Guinea-Bissau	2.00 (1.55 to 2.57)	847 (648 to 1104)	5.22 (3.93 to 6.77)	1196 (889 to 1549)	1.13 (1.09 to 1.16)	0.26 (0.17 to 0.38)	53.6 (37.4 to 80.4)	0.38 (0.25 to 0.59)	37.8 (25.8 to 62.4)	-1.10 (-1.17 to -1.03)	1.74 (1.31 to 2.25)	793 (602 to 1039)	4.85 (3.61 to 6.32)	1158 (860 to 1502)	1.24 (1.20 to 1.27)
Guyana	4.56 (3.99 to 5.11)	2129 (1868 to 2375)	7.02 (5.36 to 8.92)	1990 (1532 to 2509)	-0.17 (-0.43 to 0.08)	0.37 (0.32 to 0.43)	106 (91.6 to 123)	0.28 (0.20 to 0.37)	71.6 (51.7 to 93.8)	-1.13 (-1.33 to -0.93)	4.19 (3.65 to 4.70)	2023 (1772 to 2256)	6.74 (5.15 to 8.55)	1918 (1479 to 2410)	-0.31 (-0.62 to -0.03)
Haiti	53.2 (43.2 to 66.7)	2863 (2327 to 3565)	105 (76.3 to 144)	2566 (1908 to 3471)	-0.31 (-0.35 to -0.28)	7.47 (5.00 to 13.6)	258 (172 to 500)	12.1 (6.95 to 20.3)	194 (112 to 323)	-0.83 (-0.93 to -0.73)	45.7 (36.9 to 57.6)	2605 (2114 to 3273)	92.9 (67.8 to 127)	2372 (1752 to 3197)	-0.27 (-0.30 to -0.23)
Honduras	4.71 (3.94 to 5.55)	396 (330 to 470)	26.5 (19.4 to 35.7)	755 (552 to 1015)	2.32 (2.06 to 2.57)	0.40 (0.27 to 0.52)	17.0 (12.4 to 21.8)	0.64 (0.39 to 0.96)	13.0 (8.08 to 19.5)	-0.80 (-0.90 to -0.69)	4.31 (3.54 to 5.11)	379 (313 to 452)	25.8 (18.9 to 34.9)	742 (540 to 998)	2.42 (2.15 to 2.68)
Hungary	25.8 (23.6 to 27.9)	303 (280 to 326)	25.2 (22.2 to 28.0)	218 (193 to 241)	-0.78 (-1.06 to -0.53)	2.61 (2.40 to 2.83)	38.5 (35.9 to 41.7)	0.75 (0.65 to 0.86)	11.6 (9.88 to 13.6)	-3.79 (-3.99 to -3.63)	23.2 (21.1 to 25.1)	264 (242 to 286)	24.5 (21.5 to 27.2)	206 (183 to 228)	-0.47 (-0.76 to -0.21)
Iceland	0.14 (0.13 to 0.16)	88.8 (80.9 to 96.1)	0.15 (0.12 to 0.17)	45.5 (38.5 to 50.7)	-2.29 (-2.43 to -2.19)	0.02 (0.02 to 0.02)	12.6 (11.7 to 13.5)	0.01 (0.009 to 0.01)	4.25 (3.80 to 4.70)	-3.38 (-3.48 to -3.31)	0.13 (0.11 to 0.14)	76.2 (68.8 to 83.0)	0.14 (0.11 to 0.16)	41.3 (34.6 to 46.2)	-2.11 (-2.24 to -1.99)
India	1177 (990 to 1342)	492 (410 to 566)	3822 (3309 to 4417)	623 (540 to 720)	0.78 (0.63 to 0.96)	164 (128 to 245)	43.7 (33.5 to 67.5)	219 (171 to 282)	31.2 (24.2 to 40.1)	-1.03 (-1.15 to -0.88)	1013 (842 to 1151)	448 (372 to 513)	3603 (3115 to 4165)	591 (512 to 683)	0.91 (0.74 to 1.12)
Indonesia	319 (262 to 400)	556 (447 to 707)	836 (636 to 1099)	657 (508 to 855)	0.56 (0.53 to 0.58)	36.2 (27.5 to 59.0)	42.3 (31.6 to 69.3)	43.8 (31.1 to 64.9)	30.0 (21.6 to 43.9)	-1.07 (-1.10 to -1.04)	283 (227 to 354)	514 (408 to 648)	792 (600 to 1039)	627 (486 to 810)	0.66 (0.64 to 0.68)
Iran (Islamic Republic of)	40.3 (34.7 to 46.9)	320 (274 to 375)	161 (143 to 178)	432 (382 to 481)	0.96 (0.89 to 1.02)	5.66 (4.14 to 8.02)	18.4 (14.2 to 24.7)	4.65 (2.82 to 5.77)	10.7 (6.63 to 13.3)	-1.74 (-1.87 to -1.64)	34.6 (29.7 to 40.6)	301 (258 to 355)	156 (139 to 174)	422 (371 to 469)	1.07 (1.00 to 1.13)
Iraq	40.6 (33.2 to 49.1)	896 (719 to 1091)	112 (85.0 to 145)	914 (697 to 1158)	0.10 (0.06 to 0.15)	6.53 (4.75 to 8.96)	68.8 (48.6 to 97.4)	7.39 (5.09 to 10.5)	36.6 (24.8 to 52.6)	-2.07 (-2.13 to -2.02)	34.1 (27.0 to 41.5)	827 (661 to 1007)	105 (78.7 to 136)	878 (666 to 1107)	0.24 (0.19 to 0.29)
Ireland	4.15 (3.80 to 4.50)	178 (164 to 192)	2.52 (2.10 to 2.87)	56.4 (47.6 to 63.6)	-3.53 (-3.76 to -3.36)	0.32 (0.30 to 0.34)	15.9 (14.8 to 16.9)	0.14 (0.13 to 0.15)	4.34 (3.99 to 4.69)	-4.13 (-4.26 to -4.02)	3.83 (3.49 to 4.16)	162 (148 to 175)	2.38 (1.97 to 2.72)	52.1 (43.5 to 58.8)	-3.50 (-3.71 to -3.32)
Israel	10.4 (9.62 to 11.1)	401 (371 to 428)	17.5 (14.5 to 19.4)	237 (201 to 261)	-1.75 (-1.96 to -1.55)	0.53 (0.50 to 0.57)	21.0 (19.8 to 22.4)	0.27 (0.25 to 0.30)	5.30 (4.93 to 5.72)	-4.39 (-4.49 to -4.32)	9.91 (9.10 to 10.6)	380 (350 to 406)	17.2 (14.3 to 19.1)	232 (196 to 255)	-1.66 (-1.87 to -1.45)
Italy	195 (177 to 204)	363 (332 to 380)	126 (100 to 140)	126 (106 to 138)	-3.44 (-3.57 to -3.28)	4.11 (4.00 to 4.23)	12.7 (12.4 to 13.0)	1.45 (1.33 to 1.54)	3.54 (3.37 to 3.71)	-4.09 (-4.19 to -3.97)	191 (173 to 200)	350 (319 to 367)	124 (99.1 to 139)	122 (102 to 134)	-3.41 (-3.54 to -3.23)
Jamaica	16.6 (15.8 to 17.5)	1737 (1651 to 1830)	24.9 (19.6 to 31.3)	1510 (1185 to 1900)	-0.24 (-0.65 to 0.10)	0.75 (0.70 to 0.82)	66.3 (61.6 to 71.9)	0.70 (0.51 to 0.92)	46.3 (34.4 to 60.6)	-1.00 (-1.27 to -0.74)	15.8 (15.0 to 16.7)	1670 (1586 to 1764)	24.2 (19.1 to 30.4)	1464 (1150 to 1842)	-0.22 (-0.62 to 0.11)
Japan	116 (107 to 122)	123 (113 to 129)	59.6 (45.5 to 67.9)	26.1 (22.3 to 28.3)	-4.83 (-4.99 to -4.64)	6.27 (6.11 to 6.40)	8.78 (8.60 to 8.95)	1.57 (1.45 to 1.66)	2.18 (2.12 to 2.24)	-4.41 (-4.61 to -4.23)	110 (101 to 116)	114 (104 to 120)	58.0 (44.1 to 66.2)	23.9 (20.1 to 26.1)	-4.87 (-5.02 to -4.67)
Jordan	9.85 (8.13 to 11.8)	1455 (1204 to 1724)	24.7 (19.9 to 30.6)	762 (614 to 936)	-1.94 (-2.33 to -1.73)	0.95 (0.66 to 1.40)	44.7 (32.5 to 60.7)	0.80 (0.61 to 1.10)	14.0 (10.6 to 19.0)	-3.64 (-3.71 to -3.58)	8.91 (7.32 to 10.6)	1410 (1164 to 1675)	23.9 (19.1 to 29.3)	748 (601 to 918)	-1.90 (-2.29 to -1.68)
Kazakhstan	17.4 (15.2 to 19.8)	219 (190 to 248)	22.5 (18.7 to 26.7)	213 (178 to 252)	-0.00 (-0.27 to 0.20)	3.46 (3.10 to 3.83)	42.1 (37.7 to 46.8)	2.70 (2.32 to 3.15)	26.7 (23.0 to 31.0)	-1.49 (-1.75 to -1.28)	13.9 (11.9 to 16.1)	176 (151 to 204)	19.8 (16.3 to 23.6)	187 (154 to 222)	0.18 (-0.04 to 0.38)
Kenya	22.8 (18.9 to 27.4)	505 (418 to 610)	73.9 (54.4 to 95.9)	591 (441 to 763)	0.52 (0.49 to 0.55)	2.11 (1.44 to 3.00)	18.2 (12.8 to 25.6)	4.04 (2.72 to 6.20)	16.7 (11.2 to 26.4)	-0.23 (-0.27 to -0.19)	20.7 (17.0 to 24.9)	487 (403 to 586)	69.9 (51.6 to 91.0)	575 (429 to 738)	0.54 (0.50 to 0.57)
Kiribati	0.73 (0.59 to 0.88)	3369 (2702 to 4061)	2.00 (1.53 to 2.62)	4597 (3533 to 5882)	1.03 (1.00 to 1.05)	0.02 (0.01 to 0.03)	48.5 (28.7 to 67.4)	0.03 (0.02 to 0.05)	51.1 (27.4 to 74.4)	0.15 (0.12 to 0.18)	0.71 (0.57 to 0.86)	3320 (2657 to 4006)	1.96 (1.50 to 2.57)	4546 (3496 to 5801)	1.04 (1.01 to 1.06)
Kuwait	1.34 (1.23 to 1.44)	548 (494 to 594)	4.09 (3.52 to 4.68)	363 (305 to 419)	-1.47 (-2.04 to -0.84)	0.20 (0.19 to 0.22)	30.5 (28.0 to 33.2)	0.23 (0.20 to 0.26)	10.2 (9.00 to 11.8)	-3.45 (-3.89 to -3.01)	1.14 (1.04 to 1.23)	517 (465 to 561)	3.86 (3.31 to 4.43)	353 (296 to 407)	-1.36 (-1.94 to -0.70)
Kyrgyzstan	2.86 (2.60 to 3.14)	154 (140 to 170)	6.04 (5.09 to 7.11)	206 (174 to 242)	0.83 (0.55 to 1.04)	0.51 (0.47 to 0.56)	24.1 (21.8 to 26.4)	0.68 (0.56 to 0.81)	19.8 (16.6 to 23.6)	-0.93 (-1.30 to -0.63)	2.35 (2.11 to 2.60)	130 (117 to 144)	5.36 (4.50 to 6.32)	186 (156 to 219)	1.08 (0.76 to 1.33)
Lao People's Democratic Republic	12.9 (9.77 to 17.4)	1071 (818 to 1444)	24.7 (18.4 to 31.9)	976 (737 to 1249)	-0.29 (-0.31 to -0.27)	1.42 (0.93 to 2.81)	79.6 (50.3 to 164)	1.80 (1.19 to 2.56)	50.6 (33.3 to 73.0)	-1.47 (-1.49 to -1.44)	11.5 (8.75 to 15.2)	991 (760 to 1308)	22.9 (17.2 to 29.7)	926 (700 to 1181)	-0.22 (-0.24 to -0.20)
Latvia	3.54 (3.28 to 3.81)	172 (160 to 184)	6.38 (5.55 to 7.10)	290 (253 to 325)	1.64 (1.01 to 2.09)	0.59 (0.55 to 0.63)	41.6 (38.8 to 44.7)	0.25 (0.21 to 0.29)	30.6 (25.9 to 35.5)	-1.06 (-1.84 to -0.49)	2.95 (2.71 to 3.19)	130 (120 to 142)	6.13 (5.34 to 6.84)	259 (224 to 290)	2.35 (1.61 to 2.88)
Lebanon	8.09 (6.58 to 10.2)	706 (576 to 880)	13.6 (11.0 to 16.2)	412 (335 to 494)	-1.82 (-1.90 to -1.75)	0.73 (0.47 to 1.07)	49.3 (31.2 to 72.8)	0.56 (0.33 to 0.82)	18.7 (11.5 to 27.0)	-3.05 (-3.12 to -2.98)	7.36 (6.00 to 9.30)	657 (538 to 818)	13.0 (10.6 to 15.6)	393 (319 to 472)	-1.73 (-1.81 to -1.66)
Lesotho	4.66 (3.59 to 6.24)	856 (662 to 1152)	13.8 (9.59 to 18.8)	2061 (1430 to 2783)	2.86 (2.76 to 2.96)	0.18 (0.10 to 0.29)	18.5 (11.3 to 30.0)	0.28 (0.14 to 0.44)	27.7 (13.7 to 43.3)	1.36 (1.27 to 1.44)	4.48 (3.47 to 6.07)	838 (648 to 1135)	13.6 (9.38 to 18.5)	2033 (1412 to 2749)	2.89 (2.78 to 2.99)
Liberia	3.83 (2.98 to 4.81)	669 (535 to 824)	11.1 (7.97 to 15.1)	980 (715 to 1315)	1.23 (1.18 to 1.29)	0.44 (0.28 to 0.62)	35.0 (22.8 to 49.7)	0.65 (0.37 to 1.02)	24.8 (15.1 to 38.9)	-1.27 (-1.38 to -1.16)	3.39 (2.64 to 4.26)	634 (504 to 783)	10.4 (7.42 to 14.2)	955 (693 to 1282)	1.32 (1.27 to 1.38)
Libya	3.14 (2.47 to 3.92)	324 (256 to 400)	15.7 (11.7 to 21.3)	583 (436 to 786)	1.92 (1.83 to 2.01)	0.45 (0.30 to 0.76)	21.8 (14.8 to 35.3)	0.98 (0.58 to 1.42)	27.1 (16.6 to 40.9)	0.69 (0.55 to 0.86)	2.69 (2.08 to 3.33)	302 (236 to 372)	14.7 (10.9 to 20.1)	556 (415 to 754)	1.97 (1.86 to 2.07)
Lithuania	2.65 (2.45 to 2.86)	102 (95.2 to 110)	5.79 (5.06 to 6.50)	180 (159 to 204)	1.75 (1.48 to 1.99)	0.66 (0.60 to 0.74)	30.8 (28.2 to 34.0)	0.39 (0.33 to 0.44)	26.6 (22.7 to 30.5)	-0.51 (-0.97 to -0.14)	1.99 (1.81 to 2.15)	71.3 (65.1 to 77.4)	5.40 (4.71 to 6.08)	154 (135 to 174)	2.39 (2.03 to 2.68)
Luxembourg	0.64 (0.59 to 0.69)	198 (184 to 212)	0.49 (0.42 to 0.56)	78.1 (67.7 to 88.3)	-3.02 (-3.29 to -2.82)	0.05 (0.05 to 0.06)	21.3 (19.8 to 22.7)	0.02 (0.02 to 0.03)	5.26 (4.69 to 5.97)	-4.54 (-4.93 to -4.38)	0.59 (0.54 to 0.63)	177 (164 to 190)	0.47 (0.40 to 0.54)	72.8 (62.8 to 82.6)	-2.87 (-3.18 to -2.67)
Madagascar	22.2 (18.4 to 26.0)	778 (645 to 924)	50.8 (36.4 to 68.1)	770 (564 to 1029)	-0.04 (-0.09 to 0.02)	2.55 (1.82 to 3.50)	39.4 (29.7 to 53.5)	4.81 (3.46 to 6.60)	32.3 (23.1 to 45.0)	-0.65 (-0.71 to -0.60)	19.6 (16.0 to 23.3)	738 (609 to 883)	46.0 (33.1 to 62.2)	737 (538 to 991)	-0.01 (-0.07 to 0.05)
Malawi	18.9 (14.9 to 23.4)	816 (648 to 998)	32.8 (24.4 to 43.2)	746 (570 to 962)	-0.28 (-0.33 to -0.23)	2.59 (1.66 to 3.67)	42.0 (29.2 to 59.1)	2.93 (1.90 to 4.22)	28.2 (18.7 to 40.7)	-1.26 (-1.31 to -1.20)	16.3 (12.9 to 20.2)	774 (615 to 955)	29.9 (22.4 to 39.2)	718 (547 to 926)	-0.24 (-0.28 to -0.19)
Malaysia	35.5 (31.2 to 40.2)	721 (629 to 824)	66.2 (57.5 to 75.3)	463 (403 to 524)	-1.23 (-1.41 to -1.04)	2.79 (2.23 to 3.66)	40.5 (32.2 to 53.3)	3.77 (2.67 to 4.96)	23.5 (16.5 to 30.8)	-1.71 (-1.87 to -1.53)	32.7 (28.7 to 37.2)	681 (591 to 776)	62.4 (53.7 to 70.8)	439 (381 to 498)	-1.22 (-1.40 to -1.02)
Maldives	0.46 (0.38 to 0.58)	1057 (885 to 1285)	0.53 (0.43 to 0.64)	360 (289 to 434)	-3.52 (-3.67 to -3.34)	0.06 (0.04 to 0.11)	76.3 (46.8 to 164)	0.03 (0.02 to 0.05)	15.7 (11.3 to 22.0)	-5.07 (-5.14 to -4.96)	0.41 (0.33 to 0.49)	980 (823 to 1176)	0.50 (0.40 to 0.60)	345 (276 to 417)	-3.43 (-3.59 to -3.26)
Mali	17.6 (14.3 to 21.9)	813 (649 to 1010)	48.6 (37.7 to 61.9)	1041 (810 to 1316)	0.81 (0.77 to 0.84)	1.89 (1.19 to 2.77)	45.2 (31.0 to 65.7)	3.46 (2.23 to 5.20)	30.3 (20.7 to 45.9)	-1.28 (-1.36 to -1.21)	15.7 (12.8 to 19.6)	768 (612 to 959)	45.1 (34.3 to 57.7)	1010 (779 to 1276)	0.90 (0.87 to 0.93)
Malta	1.14 (1.05 to 1.23)	480 (441 to 515)	1.23 (1.03 to 1.39)	213 (183 to 240)	-2.78 (-3.04 to -2.54)	0.06 (0.06 to 0.07)	30.0 (27.6 to 32.5)	0.03 (0.03 to 0.03)	9.87 (8.81 to 11.1)	-3.55 (-3.82 to -3.24)	1.08 (0.99 to 1.16)	450 (413 to 484)	1.20 (1.01 to 1.36)	203 (174 to 229)	-2.73 (-3.00 to -2.48)
Marshall Islands	0.26 (0.21 to 0.33)	2965 (2335 to 3813)	1.11 (0.69 to 1.56)	5743 (3526 to 8009)	2.16 (2.14 to 2.18)	0.01 (0.007 to 0.01)	41.1 (27.9 to 61.4)	0.02 (0.008 to 0.03)	52.6 (27.7 to 88.2)	0.80 (0.78 to 0.81)	0.25 (0.20 to 0.32)	2924 (2298 to 3768)	1.10 (0.68 to 1.54)	5690 (3470 to 7947)	2.18 (2.15 to 2.19)
Mauritania	3.61 (2.79 to 4.59)	661 (511 to 839)	8.48 (6.10 to 11.5)	772 (553 to 1049)	0.51 (0.45 to 0.55)	0.29 (0.20 to 0.42)	29.5 (20.5 to 43.7)	0.33 (0.21 to 0.61)	16.0 (10.1 to 30.5)	-2.12 (-2.23 to -2.04)	3.32 (2.56 to 4.19)	632 (487 to 802)	8.15 (5.86 to 11.1)	756 (542 to 1029)	0.59 (0.53 to 0.64)
Mauritius	5.15 (4.79 to 5.53)	1227 (1143 to 1315)	20.4 (18.7 to 21.6)	2060 (1880 to 2173)	1.51 (1.27 to 1.83)	0.58 (0.51 to 0.67)	114 (99.2 to 133)	0.73 (0.65 to 0.81)	95.0 (85.4 to 105)	-0.55 (-0.76 to -0.30)	4.57 (4.20 to 4.94)	1114 (1028 to 1202)	19.7 (18.0 to 20.8)	1965 (1795 to 2076)	1.66 (1.40 to 2.02)
Mexico	399 (389 to 408)	1764 (1712 to 1805)	958 (814 to 1110)	1402 (1193 to 1619)	-0.64 (-0.78 to -0.49)	18.2 (17.5 to 19.1)	48.5 (47.1 to 50.2)	26.8 (22.5 to 30.8)	38.9 (32.8 to 44.7)	-0.74 (-0.96 to -0.52)	381 (371 to 390)	1716 (1664 to 1757)	932 (791 to 1078)	1364 (1160 to 1574)	-0.64 (-0.78 to -0.48)
Micronesia (Federated States of)	0.54 (0.41 to 0.71)	2098 (1610 to 2745)	1.22 (0.92 to 1.62)	2981 (2275 to 3919)	1.18 (1.15 to 1.21)	0.02 (0.01 to 0.03)	39.2 (25.7 to 63.6)	0.02 (0.01 to 0.02)	29.4 (19.6 to 45.1)	-0.92 (-0.94 to -0.90)	0.52 (0.40 to 0.68)	2059 (1581 to 2688)	1.20 (0.91 to 1.60)	2951 (2256 to 3879)	1.21 (1.18 to 1.24)
Monaco	0.02 (0.02 to 0.03)	54.3 (38.7 to 72.2)	0.03 (0.02 to 0.03)	45.2 (33.2 to 57.7)	-0.58 (-0.61 to -0.55)	9e-04 (6e-04 to 0.001)	4.44 (3.02 to 5.87)	7e-04 (5e-04 to 0.001)	3.07 (2.17 to 4.20)	-1.17 (-1.20 to -1.14)	0.02 (0.02 to 0.03)	49.9 (34.9 to 66.8)	0.03 (0.02 to 0.03)	42.1 (30.8 to 54.1)	-0.52 (-0.55 to -0.49)
Mongolia	1.11 (0.91 to 1.33)	168 (136 to 203)	2.63 (2.17 to 3.11)	188 (155 to 220)	0.33 (0.18 to 0.48)	0.41 (0.28 to 0.51)	46.8 (30.7 to 61.7)	0.39 (0.28 to 0.51)	23.8 (17.0 to 30.7)	-2.14 (-2.24 to -2.04)	0.71 (0.54 to 0.89)	121 (93.0 to 153)	2.24 (1.83 to 2.68)	164 (134 to 193)	0.97 (0.78 to 1.17)
Montenegro	0.92 (0.77 to 1.08)	266 (224 to 312)	1.77 (1.46 to 2.09)	328 (274 to 389)	0.81 (0.70 to 0.90)	0.19 (0.14 to 0.24)	55.4 (41.1 to 71.9)	0.20 (0.14 to 0.27)	44.3 (32.5 to 59.5)	-0.61 (-0.79 to -0.41)	0.73 (0.61 to 0.87)	211 (176 to 250)	1.57 (1.30 to 1.87)	284 (236 to 341)	1.09 (0.98 to 1.19)
Morocco	26.1 (21.2 to 32.7)	328 (266 to 400)	107 (74.7 to 141)	606 (429 to 784)	2.00 (1.94 to 2.04)	5.26 (3.33 to 9.71)	36.8 (23.9 to 65.3)	5.19 (3.55 to 7.75)	28.1 (19.5 to 42.7)	-0.87 (-0.91 to -0.84)	20.8 (16.2 to 26.0)	291 (228 to 363)	102 (70.5 to 134)	578 (406 to 747)	2.23 (2.17 to 2.27)
Mozambique	25.2 (20.2 to 31.6)	711 (589 to 877)	53.8 (37.0 to 71.2)	781 (542 to 1037)	0.31 (0.27 to 0.34)	3.55 (2.10 to 5.10)	42.5 (27.8 to 59.6)	6.12 (3.72 to 8.68)	34.9 (22.2 to 49.0)	-0.61 (-0.71 to -0.54)	21.6 (17.6 to 27.1)	669 (557 to 826)	47.7 (32.4 to 64.0)	746 (514 to 990)	0.36 (0.32 to 0.39)
Myanmar	218 (167 to 294)	1595 (1227 to 2143)	356 (280 to 448)	1268 (1000 to 1586)	-0.75 (-0.77 to -0.72)	24.4 (15.2 to 49.7)	134 (82.3 to 279)	19.9 (13.4 to 30.8)	65.8 (44.2 to 102)	-2.29 (-2.33 to -2.25)	193 (146 to 253)	1460 (1111 to 1903)	336 (263 to 423)	1202 (949 to 1509)	-0.63 (-0.65 to -0.60)
Namibia	4.79 (3.97 to 5.80)	1321 (1100 to 1596)	10.8 (7.43 to 14.6)	1386 (968 to 1858)	0.19 (0.15 to 0.23)	0.17 (0.10 to 0.24)	21.4 (12.7 to 30.4)	0.21 (0.11 to 0.34)	15.6 (8.20 to 25.4)	-0.98 (-1.08 to -0.89)	4.63 (3.81 to 5.60)	1300 (1083 to 1571)	10.6 (7.26 to 14.4)	1371 (954 to 1838)	0.21 (0.16 to 0.25)
Nauru	0.06 (0.05 to 0.08)	2824 (2258 to 3596)	0.13 (0.10 to 0.17)	3663 (2820 to 4809)	0.84 (0.79 to 0.87)	0.002 (0.002 to 0.003)	40.4 (27.2 to 64.8)	0.002 (0.001 to 0.003)	35.4 (20.3 to 58.1)	-0.45 (-0.47 to -0.42)	0.06 (0.05 to 0.08)	2783 (2222 to 3545)	0.12 (0.09 to 0.16)	3627 (2784 to 4765)	0.85 (0.81 to 0.89)
Nepal	24.4 (19.0 to 30.4)	492 (385 to 611)	76.6 (58.1 to 101)	625 (475 to 822)	0.79 (0.76 to 0.82)	3.67 (2.41 to 5.87)	45.5 (29.8 to 76.4)	5.58 (3.77 to 7.90)	34.9 (23.3 to 49.2)	-0.85 (-0.91 to -0.80)	20.7 (16.1 to 26.2)	446 (350 to 558)	71.0 (54.3 to 94.0)	590 (446 to 777)	0.92 (0.89 to 0.95)
Netherlands	39.1 (35.2 to 41.8)	325 (296 to 346)	21.4 (18.1 to 23.8)	102 (88.4 to 112)	-3.66 (-3.77 to -3.57)	3.28 (3.06 to 3.50)	33.8 (31.8 to 35.9)	1.29 (1.18 to 1.39)	9.97 (9.29 to 10.6)	-3.93 (-4.03 to -3.81)	35.8 (32.1 to 38.4)	292 (264 to 311)	20.1 (16.9 to 22.4)	91.7 (79.1 to 101)	-3.62 (-3.73 to -3.51)
New Zealand	4.57 (4.20 to 4.93)	222 (203 to 239)	4.86 (4.27 to 5.30)	111 (99.3 to 119)	-2.05 (-2.35 to -1.75)	0.48 (0.44 to 0.51)	24.5 (22.8 to 26.2)	0.13 (0.12 to 0.14)	4.47 (4.20 to 4.79)	-5.32 (-5.64 to -4.99)	4.10 (3.74 to 4.43)	197 (180 to 213)	4.73 (4.14 to 5.16)	106 (94.7 to 115)	-1.84 (-2.13 to -1.54)

Location	DM, Number in 1990 (000s)	DM, ASR in 1990 (per 100,000)	DM, Number in 2021 (000s)	DM, ASR in 2021 (per 100,000)	DM, AAPC (95%CI)	T1D, Number in 1990 (000s)	T1D, ASR in 1990 (per 100,000)	T1D, Number in 2021 (000s)	T1D, ASR in 2021 (per 100,000)	T1D, AAPC (95%CI)	T2D, Number in 1990 (000s)	T2D, ASR in 1990 (per 100,000)	T2D, Number in 2021 (000s)	T2D, ASR in 2021 (per 100,000)	T2D, AAPC (95%CI)
Nicaragua	5.77 (5.21 to 6.35)	626 (562 to 693)	19.8 (16.3 to 24.0)	723 (591 to 874)	0.43 (0.04 to 0.73)	0.61 (0.46 to 0.76)	30.7 (24.3 to 39.6)	0.65 (0.48 to 0.83)	19.5 (14.3 to 25.2)	-1.52 (-1.70 to -1.38)	5.16 (4.64 to 5.73)	596 (531 to 662)	19.2 (15.7 to 23.2)	703 (572 to 852)	0.49 (0.08 to 0.80)
Niger	7.01 (4.97 to 9.31)	431 (305 to 585)	27.3 (18.7 to 37.7)	582 (409 to 816)	0.98 (0.95 to 1.01)	1.55 (0.80 to 2.57)	34.5 (19.6 to 53.5)	3.25 (1.60 to 5.53)	23.9 (13.2 to 37.2)	-1.25 (-1.34 to -1.18)	5.46 (3.95 to 7.35)	396 (281 to 544)	24.0 (16.6 to 33.9)	558 (387 to 788)	1.12 (1.10 to 1.15)
Nigeria	145 (108 to 188)	651 (495 to 835)	332 (226 to 468)	658 (477 to 900)	0.02 (-0.00 to 0.04)	13.0 (7.83 to 18.4)	31.0 (19.1 to 44.5)	22.9 (12.4 to 33.9)	18.6 (10.7 to 27.7)	-1.66 (-1.71 to -1.61)	132 (99.0 to 170)	620 (472 to 795)	309 (209 to 437)	639 (460 to 873)	0.09 (0.06 to 0.11)
Niue	0.02 (0.02 to 0.03)	1815 (1425 to 2290)	0.03 (0.02 to 0.04)	2842 (2077 to 3526)	1.46 (1.40 to 1.50)	3e-04 (2e-04 to 4e-04)	25.6 (17.2 to 37.1)	4e-04 (3e-04 to 6e-04)	62.8 (42.3 to 86.3)	3.04 (2.04 to 3.73)	0.02 (0.02 to 0.03)	1789 (1403 to 2254)	0.03 (0.02 to 0.04)	2779 (2023 to 3455)	1.45 (1.40 to 1.50)
North Macedonia	5.34 (4.49 to 6.23)	553 (466 to 644)	10.7 (8.44 to 13.1)	632 (505 to 770)	0.41 (0.28 to 0.50)	1.01 (0.80 to 1.24)	100 (78.6 to 122)	0.76 (0.51 to 1.08)	50.5 (33.6 to 70.4)	-2.17 (-2.28 to -2.09)	4.32 (3.58 to 5.14)	453 (376 to 538)	9.97 (7.93 to 12.1)	581 (465 to 704)	0.81 (0.70 to 0.90)
Northern Mariana Islands	0.12 (0.09 to 0.16)	1574 (1223 to 2041)	0.33 (0.27 to 0.37)	1268 (1072 to 1431)	-0.70 (-0.80 to -0.61)	0.002 (0.001 to 0.003)	7.97 (5.18 to 12.0)	0.001 (8e-04 to 0.002)	5.05 (3.51 to 7.21)	-1.74 (-3.08 to 0.01)	0.12 (0.09 to 0.16)	1566 (1214 to 2030)	0.33 (0.27 to 0.37)	1263 (1067 to 1425)	-0.70 (-0.79 to -0.60)
Norway	4.53 (4.12 to 4.77)	118 (110 to 124)	3.98 (3.37 to 4.34)	68.9 (60.7 to 74.0)	-2.06 (-2.36 to -1.81)	0.57 (0.55 to 0.59)	24.1 (23.4 to 24.7)	0.24 (0.23 to 0.25)	8.27 (7.97 to 8.57)	-3.41 (-3.68 to -3.21)	3.96 (3.56 to 4.19)	94.1 (86.1 to 99.3)	3.74 (3.13 to 4.09)	60.6 (52.6 to 65.5)	-1.74 (-2.07 to -1.47)
Oman	3.21 (2.42 to 4.24)	986 (737 to 1305)	6.59 (5.23 to 8.10)	764 (614 to 934)	-0.82 (-0.89 to -0.74)	0.55 (0.39 to 0.77)	83.3 (60.4 to 116)	0.64 (0.48 to 0.83)	37.0 (28.5 to 47.3)	-2.51 (-2.74 to -2.35)	2.67 (1.96 to 3.54)	902 (668 to 1194)	5.95 (4.73 to 7.33)	727 (583 to 888)	-0.71 (-0.78 to -0.63)
Pakistan	179 (143 to 217)	682 (544 to 829)	700 (516 to 942)	1155 (860 to 1531)	1.73 (1.70 to 1.75)	22.2 (14.9 to 28.3)	52.9 (36.2 to 67.8)	70.1 (45.8 to 99.3)	66.5 (42.4 to 93.2)	0.77 (0.73 to 0.80)	157 (119 to 192)	629 (484 to 767)	630 (460 to 844)	1089 (805 to 1444)	1.79 (1.77 to 1.81)
Palau	0.09 (0.07 to 0.12)	1852 (1438 to 2345)	0.26 (0.20 to 0.32)	2533 (1989 to 3132)	1.06 (1.00 to 1.12)	0.002 (0.001 to 0.002)	20.6 (14.0 to 29.8)	0.001 (0.001 to 0.002)	21.2 (15.1 to 29.9)	0.12 (0.08 to 0.15)	0.09 (0.07 to 0.12)	1832 (1420 to 2321)	0.26 (0.20 to 0.32)	2512 (1972 to 3109)	1.07 (1.01 to 1.12)
Palestine	4.69 (3.63 to 6.19)	972 (756 to 1276)	12.0 (10.1 to 14.1)	972 (819 to 1144)	-0.18 (-0.32 to -0.05)	0.37 (0.23 to 0.54)	27.0 (18.1 to 38.9)	0.37 (0.26 to 0.51)	13.5 (9.74 to 17.8)	-2.14 (-2.30 to -1.99)	4.32 (3.30 to 5.75)	945 (726 to 1238)	11.6 (9.79 to 13.7)	958 (807 to 1129)	-0.14 (-0.28 to -0.01)
Panama	4.25 (3.98 to 4.52)	551 (514 to 586)	13.5 (10.9 to 16.1)	590 (478 to 705)	0.13 (-0.12 to 0.37)	0.24 (0.22 to 0.26)	21.0 (19.6 to 22.6)	0.40 (0.32 to 0.48)	18.6 (15.1 to 22.2)	-0.35 (-0.47 to -0.25)	4.01 (3.74 to 4.27)	530 (493 to 564)	13.1 (10.6 to 15.7)	571 (461 to 683)	0.15 (-0.10 to 0.38)
Papua New Guinea	15.8 (11.6 to 21.7)	1544 (1149 to 2080)	48.8 (37.5 to 63.3)	1734 (1353 to 2222)	0.38 (0.31 to 0.45)	0.86 (0.48 to 1.33)	37.7 (21.0 to 61.4)	2.25 (1.47 to 3.23)	39.8 (25.8 to 57.9)	0.35 (0.23 to 0.49)	14.9 (10.9 to 20.5)	1506 (1114 to 2022)	46.5 (35.5 to 60.6)	1694 (1321 to 2173)	0.38 (0.32 to 0.46)
Paraguay	8.08 (6.69 to 9.48)	652 (537 to 770)	37.0 (28.6 to 46.8)	1205 (934 to 1521)	2.08 (1.92 to 2.23)	0.62 (0.44 to 0.83)	27.2 (20.8 to 36.0)	0.78 (0.52 to 1.09)	22.1 (14.8 to 31.0)	-0.44 (-0.64 to -0.27)	7.46 (6.14 to 8.83)	625 (513 to 740)	36.2 (28.0 to 45.9)	1183 (916 to 1496)	2.15 (1.99 to 2.31)
Peru	20.4 (17.2 to 24.2)	318 (265 to 380)	65.0 (49.5 to 86.7)	371 (283 to 495)	0.54 (0.16 to 0.89)	1.33 (1.02 to 1.70)	12.3 (9.52 to 15.6)	1.43 (0.79 to 2.10)	7.75 (4.33 to 11.2)	-1.37 (-1.87 to -1.07)	19.1 (15.9 to 22.8)	305 (254 to 367)	63.6 (48.3 to 84.4)	363 (276 to 482)	0.61 (0.22 to 0.96)
Philippines	105 (94.9 to 115)	646 (585 to 709)	396 (314 to 487)	872 (696 to 1070)	1.15 (1.07 to 1.23)	10.7 (8.69 to 12.7)	36.4 (29.9 to 45.1)	18.9 (14.3 to 24.9)	33.9 (25.9 to 45.2)	-0.21 (-0.28 to -0.15)	94.4 (84.8 to 104)	610 (550 to 669)	377 (298 to 464)	838 (667 to 1027)	1.22 (1.15 to 1.29)
Poland	85.7 (82.2 to 89.0)	333 (319 to 345)	83.7 (73.6 to 92.4)	192 (170 to 212)	-1.79 (-1.95 to -1.67)	12.3 (12.0 to 12.5)	51.7 (50.6 to 52.8)	2.92 (2.56 to 3.26)	10.5 (9.23 to 11.7)	-5.13 (-5.31 to -4.98)	73.4 (70.1 to 76.6)	281 (268 to 293)	80.7 (71.0 to 89.3)	181 (161 to 200)	-1.43 (-1.59 to -1.30)
Portugal	33.4 (31.1 to 35.7)	415 (387 to 440)	29.0 (23.6 to 32.5)	173 (146 to 191)	-3.03 (-3.21 to -2.85)	1.01 (0.95 to 1.07)	18.9 (17.8 to 20.0)	0.29 (0.26 to 0.31)	4.62 (4.29 to 4.98)	-4.42 (-4.53 to -4.33)	32.4 (30.1 to 34.7)	396 (369 to 422)	28.7 (23.3 to 32.2)	168 (142 to 186)	-2.98 (-3.16 to -2.79)
Puerto Rico	21.1 (19.9 to 22.3)	1091 (1027 to 1152)	26.3 (21.7 to 31.0)	680 (566 to 807)	-1.57 (-1.85 to -1.30)	1.98 (1.84 to 2.11)	103 (96.1 to 110)	1.45 (1.19 to 1.73)	59.8 (49.2 to 71.8)	-1.57 (-1.76 to -1.36)	19.1 (18.0 to 20.3)	989 (928 to 1045)	24.8 (20.5 to 29.2)	621 (516 to 737)	-1.57 (-1.84 to -1.30)
Qatar	0.47 (0.37 to 0.62)	1387 (1099 to 1800)	3.05 (2.42 to 3.90)	1301 (1043 to 1629)	-0.16 (-0.48 to 0.12)	0.05 (0.03 to 0.08)	31.2 (21.5 to 48.2)	0.09 (0.06 to 0.13)	10.3 (7.50 to 14.0)	-3.58 (-3.80 to -3.34)	0.42 (0.33 to 0.55)	1356 (1072 to 1756)	2.96 (2.34 to 3.78)	1291 (1034 to 1617)	-0.11 (-0.44 to 0.18)
Republic of Korea	70.9 (62.3 to 79.3)	400 (351 to 448)	76.7 (60.5 to 91.0)	145 (115 to 171)	-3.30 (-3.40 to -3.22)	6.36 (4.55 to 7.57)	28.3 (19.9 to 33.7)	1.33 (0.97 to 2.08)	4.75 (3.59 to 7.09)	-5.58 (-5.64 to -5.52)	64.6 (56.6 to 73.0)	371 (326 to 419)	75.4 (59.5 to 89.4)	140 (112 to 166)	-3.13 (-3.22 to -3.07)
Republic of Moldova	5.01 (4.56 to 5.49)	189 (173 to 206)	6.85 (6.01 to 7.71)	214 (188 to 240)	0.42 (-0.05 to 0.85)	0.75 (0.70 to 0.80)	31.9 (29.8 to 33.9)	0.57 (0.50 to 0.66)	33.0 (28.9 to 37.8)	0.13 (-0.67 to 0.67)	4.27 (3.84 to 4.72)	157 (142 to 173)	6.28 (5.47 to 7.07)	181 (158 to 204)	0.45 (-0.00 to 0.88)
Romania	28.3 (26.7 to 30.1)	186 (175 to 197)	26.7 (23.8 to 30.2)	126 (112 to 142)	-1.32 (-1.52 to -1.18)	4.16 (3.94 to 4.35)	32.2 (30.5 to 33.7)	1.13 (0.99 to 1.28)	8.67 (7.57 to 9.86)	-4.09 (-4.20 to -3.99)	24.1 (22.5 to 26.0)	154 (143 to 165)	25.5 (22.7 to 29.0)	118 (104 to 133)	-0.96 (-1.15 to -0.81)
Russian Federation	140 (136 to 143)	126 (123 to 129)	514 (462 to 562)	342 (307 to 374)	3.49 (2.99 to 3.86)	24.0 (22.9 to 25.5)	29.6 (28.4 to 31.1)	18.4 (16.5 to 20.3)	25.1 (22.6 to 27.3)	-0.35 (-0.86 to 0.09)	116 (112 to 119)	96.8 (93.2 to 99.7)	496 (445 to 542)	317 (285 to 347)	4.16 (3.66 to 4.54)
Rwanda	20.2 (13.0 to 29.0)	1145 (728 to 1664)	25.6 (15.5 to 39.8)	684 (412 to 1052)	-1.69 (-1.74 to -1.64)	2.24 (1.48 to 3.30)	57.2 (37.7 to 86.9)	1.96 (1.26 to 2.97)	27.8 (16.8 to 44.0)	-2.31 (-2.40 to -2.23)	17.9 (11.2 to 26.4)	1088 (685 to 1584)	23.6 (14.1 to 36.8)	657 (392 to 1012)	-1.65 (-1.71 to -1.60)
Saint Kitts and Nevis	0.39 (0.35 to 0.42)	1844 (1687 to 2010)	0.32 (0.26 to 0.38)	897 (749 to 1058)	-2.26 (-2.54 to -1.98)	0.02 (0.02 to 0.02)	109 (97.4 to 121)	0.01 (0.008 to 0.01)	32.8 (26.4 to 40.1)	-3.84 (-3.93 to -3.77)	0.37 (0.33 to 0.40)	1735 (1585 to 1892)	0.31 (0.26 to 0.37)	864 (721 to 1020)	-2.18 (-2.47 to -1.90)
Saint Lucia	1.11 (1.04 to 1.19)	2278 (2127 to 2433)	1.48 (1.24 to 1.75)	1171 (983 to 1391)	-2.35 (-2.70 to -2.12)	0.07 (0.06 to 0.07)	107 (97.3 to 117)	0.05 (0.04 to 0.06)	49.5 (40.4 to 60.5)	-2.51 (-2.66 to -2.41)	1.05 (0.98 to 1.12)	2172 (2029 to 2322)	1.43 (1.20 to 1.70)	1122 (941 to 1333)	-2.33 (-2.64 to -2.10)
Saint Vincent and the Grenadines	1.03 (0.95 to 1.11)	2559 (2352 to 2769)	1.09 (0.96 to 1.24)	1574 (1393 to 1791)	-1.45 (-1.62 to -1.29)	0.07 (0.06 to 0.08)	138 (124 to 152)	0.05 (0.04 to 0.06)	80.4 (67.8 to 94.4)	-1.84 (-1.97 to -1.74)	0.96 (0.88 to 1.04)	2421 (2222 to 2622)	1.04 (0.92 to 1.19)	1493 (1320 to 1700)	-1.43 (-1.60 to -1.26)
Samoa	0.67 (0.53 to 0.87)	1512 (1194 to 1942)	1.83 (1.36 to 2.39)	2443 (1830 to 3191)	1.56 (1.54 to 1.57)	0.02 (0.01 to 0.03)	23.9 (15.1 to 33.6)	0.02 (0.02 to 0.03)	22.0 (14.7 to 30.6)	-0.25 (-0.29 to -0.22)	0.65 (0.51 to 0.84)	1488 (1171 to 1913)	1.80 (1.34 to 2.36)	2421 (1812 to 3163)	1.58 (1.56 to 1.60)
San Marino	0.02 (0.02 to 0.03)	119 (94.8 to 146)	0.03 (0.02 to 0.04)	57.8 (37.9 to 81.6)	-2.58 (-2.86 to -2.41)	0.001 (9e-04 to 0.002)	8.65 (6.46 to 11.2)	9e-04 (5e-04 to 0.001)	3.91 (2.48 to 5.81)	-2.71 (-3.00 to -2.56)	0.02 (0.02 to 0.03)	111 (87.3 to 136)	0.02 (0.02 to 0.03)	53.9 (35.5 to 76.0)	-2.56 (-2.85 to -2.40)
Sao Tome and Principe	0.09 (0.07 to 0.11)	231 (190 to 275)	0.18 (0.14 to 0.23)	293 (228 to 369)	0.75 (0.69 to 0.81)	0.02 (0.01 to 0.02)	27.1 (17.6 to 36.5)	0.02 (0.01 to 0.03)	20.8 (13.5 to 31.0)	-0.85 (-1.09 to -0.64)	0.07 (0.06 to 0.09)	204 (166 to 246)	0.16 (0.12 to 0.20)	272 (213 to 338)	0.91 (0.86 to 0.96)
Saudi Arabia	16.1 (12.2 to 20.4)	590 (445 to 743)	60.5 (47.5 to 77.0)	638 (509 to 790)	0.23 (0.16 to 0.28)	1.80 (1.25 to 2.50)	25.5 (18.2 to 34.8)	2.76 (1.36 to 4.55)	14.8 (8.22 to 23.1)	-1.74 (-1.81 to -1.69)	14.3 (10.7 to 18.3)	564 (424 to 715)	57.7 (45.2 to 73.1)	624 (498 to 772)	0.29 (0.23 to 0.35)
Senegal	11.9 (9.50 to 14.6)	671 (526 to 821)	42.1 (31.9 to 55.6)	1013 (763 to 1330)	1.25 (1.03 to 1.39)	1.39 (0.93 to 1.98)	35.6 (25.4 to 48.2)	1.95 (1.32 to 2.91)	25.5 (17.3 to 39.3)	-1.19 (-1.45 to -0.96)	10.5 (8.34 to 13.0)	636 (497 to 780)	40.1 (30.4 to 52.9)	988 (743 to 1299)	1.34 (1.14 to 1.47)
Serbia	26.2 (22.7 to 30.6)	464 (402 to 537)	37.3 (30.9 to 44.5)	392 (324 to 468)	-0.58 (-0.78 to -0.36)	3.86 (2.91 to 4.70)	70.5 (53.8 to 84.7)	2.05 (1.51 to 2.91)	29.8 (22.8 to 40.5)	-2.71 (-2.88 to -2.54)	22.4 (19.1 to 26.3)	394 (338 to 463)	35.2 (29.2 to 42.0)	362 (300 to 433)	-0.24 (-0.40 to -0.05)
Seychelles	0.12 (0.11 to 0.14)	391 (337 to 454)	0.33 (0.28 to 0.38)	559 (479 to 642)	1.09 (0.91 to 1.25)	0.01 (0.008 to 0.01)	31.7 (24.9 to 41.5)	0.01 (0.008 to 0.02)	22.8 (14.4 to 30.1)	-0.96 (-1.06 to -0.81)	0.11 (0.10 to 0.13)	359 (309 to 419)	0.32 (0.28 to 0.37)	537 (461 to 613)	1.22 (1.02 to 1.40)
Sierra Leone	4.73 (3.70 to 6.00)	444 (352 to 561)	15.2 (11.2 to 19.9)	750 (564 to 974)	1.73 (1.70 to 1.76)	0.59 (0.34 to 0.90)	26.6 (16.6 to 38.7)	1.04 (0.65 to 1.55)	23.3 (15.4 to 34.4)	-0.44 (-0.51 to -0.38)	4.14 (3.23 to 5.25)	417 (328 to 526)	14.2 (10.5 to 18.6)	727 (546 to 946)	1.83 (1.79 to 1.85)
Singapore	5.08 (4.73 to 5.45)	425 (393 to 458)	1.38 (1.21 to 1.52)	32.0 (28.4 to 35.0)	-8.11 (-8.44 to -7.83)	0.18 (0.17 to 0.19)	11.1 (10.3 to 11.9)	0.04 (0.03 to 0.04)	1.29 (1.19 to 1.39)	-6.68 (-6.84 to -6.53)	4.90 (4.56 to 5.26)	414 (382 to 447)	1.34 (1.17 to 1.48)	30.7 (27.2 to 33.6)	-8.15 (-8.48 to -7.86)
Slovakia	8.82 (7.56 to 10.2)	254 (219 to 293)	8.70 (6.91 to 10.7)	158 (126 to 193)	-1.48 (-1.64 to -1.34)	1.10 (0.83 to 1.35)	34.9 (27.1 to 42.9)	0.63 (0.46 to 0.87)	16.0 (11.9 to 21.3)	-2.56 (-2.78 to -2.37)	7.73 (6.58 to 9.04)	219 (186 to 254)	8.06 (6.39 to 9.89)	142 (113 to 175)	-1.36 (-1.55 to -1.21)
Slovenia	3.52 (3.23 to 3.81)	237 (218 to 256)	3.15 (2.64 to 3.68)	122 (103 to 144)	-2.02 (-2.28 to -1.79)	0.47 (0.44 to 0.51)	37.0 (34.6 to 39.7)	0.12 (0.10 to 0.13)	7.74 (6.44 to 9.02)	-4.93 (-5.16 to -4.69)	3.05 (2.78 to 3.33)	200 (182 to 217)	3.04 (2.53 to 3.55)	115 (96.8 to 135)	-1.64 (-1.97 to -1.25)
Solomon Islands	1.39 (0.83 to 1.99)	2015 (1370 to 2794)	5.43 (4.10 to 7.07)	2750 (2090 to 3574)	0.98 (0.91 to 1.05)	0.04 (0.02 to 0.07)	25.6 (10.2 to 52.3)	0.10 (0.07 to 0.16)	31.3 (20.6 to 49.4)	0.64 (0.55 to 0.72)	1.35 (0.81 to 1.94)	1989 (1358 to 2750)	5.32 (4.03 to 6.93)	2718 (2068 to 3533)	0.99 (0.91 to 1.05)
Somalia	15.2 (11.0 to 19.9)	1028 (755 to 1343)	44.5 (32.2 to 60.2)	1118 (812 to 1472)	0.29 (0.26 to 0.31)	1.91 (1.16 to 3.07)	45.9 (30.0 to 75.7)	5.18 (3.22 to 7.62)	45.7 (29.5 to 67.0)	0.03 (-0.01 to 0.08)	13.3 (9.46 to 17.7)	982 (713 to 1292)	39.3 (27.8 to 53.8)	1072 (776 to 1402)	0.29 (0.27 to 0.33)
South Africa	120 (108 to 133)	976 (872 to 1076)	430 (397 to 467)	1610 (1484 to 1746)	1.64 (1.39 to 1.87)	5.74 (4.29 to 8.12)	28.0 (21.4 to 38.7)	5.96 (4.48 to 7.48)	20.4 (15.0 to 25.4)	-1.00 (-1.16 to -0.84)	115 (103 to 126)	948 (844 to 1041)	424 (391 to 461)	1589 (1464 to 1725)	1.69 (1.43 to 1.93)
South Sudan	9.49 (7.07 to 12.5)	815 (605 to 1081)	20.6 (14.8 to 27.7)	993 (709 to 1301)	0.67 (0.63 to 0.70)	1.06 (0.68 to 1.57)	34.3 (22.4 to 48.8)	1.83 (1.13 to 2.84)	35.0 (22.5 to 52.4)	0.09 (-0.04 to 0.20)	8.43 (6.11 to 11.3)	780 (575 to 1037)	18.7 (13.1 to 25.3)	958 (686 to 1261)	0.69 (0.66 to 0.72)
Spain	104 (92.4 to 112)	320 (286 to 342)	60.5 (47.7 to 68.9)	85.2 (70.6 to 94.8)	-4.15 (-4.23 to -4.08)	2.68 (2.52 to 2.84)	12.8 (12.0 to 13.5)	0.83 (0.76 to 0.90)	3.09 (2.90 to 3.27)	-4.54 (-4.65 to -4.44)	102 (89.8 to 109)	307 (273 to 329)	59.7 (46.9 to 68.0)	82.1 (67.6 to 91.6)	-4.13 (-4.21 to -4.06)
Sri Lanka	36.5 (31.2 to 42.0)	690 (588 to 790)	138 (94.7 to 185)	918 (627 to 1231)	1.31 (1.07 to 1.48)	3.60 (2.76 to 4.84)	45.0 (34.1 to 62.3)	3.95 (2.41 to 5.74)	30.4 (18.8 to 44.1)	-1.14 (-1.31 to -1.00)	32.9 (28.0 to 38.0)	645 (550 to 741)	134 (91.9 to 180)	888 (604 to 1187)	1.43 (1.19 to 1.60)
Sudan	19.2 (14.8 to 26.3)	359 (279 to 509)	49.0 (35.3 to 67.6)	483 (353 to 659)	0.96 (0.94 to 0.98)	4.82 (2.87 to 8.70)	41.2 (25.0 to 69.8)	6.86 (4.16 to 10.6)	29.7 (17.7 to 46.2)	-1.06 (-1.15 to -0.99)	14.4 (11.2 to 20.1)	318 (247 to 442)	42.1 (30.4 to 58.1)	453 (332 to 618)	1.15 (1.13 to 1.17)
Suriname	1.38 (1.21 to 1.55)	1009 (890 to 1133)	3.24 (2.62 to 3.99)	943 (762 to 1156)	-0.10 (-0.27 to 0.04)	0.09 (0.06 to 0.11)	50.6 (35.9 to 61.0)	0.12 (0.08 to 0.16)	39.5 (27.7 to 51.4)	-0.76 (-0.92 to -0.59)	1.28 (1.14 to 1.45)	958 (847 to 1075)	3.12 (2.51 to 3.83)	904 (728 to 1109)	-0.07 (-0.27 to 0.08)
Sweden	12.9 (11.6 to 13.7)	153 (141 to 161)	12.6 (10.3 to 14.5)	98.6 (82.7 to 113)	-1.53 (-1.72 to -1.38)	1.16 (1.09 to 1.23)	22.1 (21.0 to 23.3)	0.58 (0.50 to 0.65)	8.93 (7.77 to 10.2)	-2.72 (-2.92 to -2.52)	11.8 (10.5 to 12.6)	131 (120 to 139)	12.0 (9.78 to 13.9)	89.7 (74.8 to 103)	-1.27 (-1.49 to -1.11)
Switzerland	15.0 (13.3 to 16.3)	231 (209 to 249)	7.47 (5.96 to 8.43)	64.4 (53.4 to 71.6)	-4.03 (-4.25 to -3.86)	0.73 (0.68 to 0.78)	17.4 (16.4 to 18.3)	0.23 (0.21 to 0.26)	3.92 (3.66 to 4.19)	-4.76 (-4.89 to -4.62)	14.2 (12.6 to 15.5)	214 (192 to 231)	7.24 (5.74 to 8.18)	60.5 (49.7 to 67.6)	-3.97 (-4.22 to -3.77)
Syrian Arab Republic	13.0 (10.4 to 15.8)	472 (377 to 571)	25.8 (19.3 to 34.4)	432 (329 to 568)	-0.22 (-0.32 to -0.12)	1.89 (1.28 to 2.62)	29.2 (21.7 to 39.7)	1.04 (0.70 to 1.46)	14.4 (9.77 to 20.6)	-2.28 (-2.36 to -2.21)	11.1 (8.73 to 13.7)	442 (351 to 539)	24.8 (18.3 to 33.2)	418 (320 to 549)	-0.13 (-0.25 to -0.01)
Taiwan (Province of China)	66.7 (63.2 to 70.3)	897 (845 to 945)	83.6 (72.2 to 91.7)	368 (321 to 403)	-2.91 (-3.08 to -2.75)	6.95 (6.58 to 7.33)	76.6 (72.3 to 81.0)	5.53 (5.03 to 6.04)	34.1 (31.2 to 36.9)	-2.58 (-2.71 to -2.43)	59.8 (56.4 to 63.1)	821 (770 to 866)	78.0 (67.0 to 85.8)	333 (288 to 366)	-2.93 (-3.11 to -2.78)
Tajikistan	5.50 (4.66 to 6.49)	322 (271 to 380)	13.4 (9.84 to 16.8)	388 (287 to 494)	0.59 (0.53 to 0.64)	1.09 (0.80 to 1.47)	43.8 (31.5 to 59.6)	1.91 (1.26 to 2.63)	38.2 (24.8 to 53.3)	-0.41 (-0.48 to -0.34)	4.41 (3.63 to 5.28)	278 (232 to 333)	11.4 (8.42 to 14.7)	350 (258 to 446)	0.72 (0.64 to 0.77)
Thailand	129 (100 to 158)	646 (502 to 791)	300 (229 to 390)	517 (397 to 672)	-0.76 (-0.85 to -0.66)	11.0 (7.85 to 15.0)	41.6 (29.7 to 58.2)	10.3 (7.17 to 14.3)	24.3 (17.1 to 33.5)	-1.64 (-1.75 to -1.54)	118 (91.7 to 144)	605 (468 to 738)	289 (221 to 376)	493 (379 to 640)	-0.71 (-0.80 to -0.61)
Timor-Leste	0.89 (0.65 to 1.26)	561 (412 to 778)	2.70 (2.06 to 3.53)	599 (458 to 787)	0.22 (0.15 to 0.27)	0.13 (0.09 to 0.24)	41.6 (27.6 to 77.5)	0.21 (0.15 to 0.30)	33.1 (22.8 to 48.9)	-0.73 (-0.86 to -0.59)	0.76 (0.56 to 1.04)	519 (381 to 709)	2.49 (1.89 to 3.29)	565 (432 to 747)	0.23 (0.14 to 0.30)
Togo	3.81 (3.04 to 4.69)	503 (394 to 630)	15.1 (11.0 to 20.5)	674 (499 to 907)	0.94 (0.91 to 0.97)	0.53 (0.35 to 0.78)	29.4 (20.5 to 41.0)	0.85 (0.52 to 1.25)	20.4 (12.9 to 31.1)	-1.15 (-1.22 to -1.10)	3.28 (2.58 to 4.15)	473 (369 to 599)	14.2 (10.2 to 19.4)	654 (484 to 882)	1.06 (1.02 to 1.09)
Tokelau	0.01 (0.01 to 0.02)	2067 (1562 to 2718)	0.02 (0.01 to 0.02)	2349 (1813 to 3040)	0.45 (0.40 to 0.50)	3e-04 (2e-04 to 4e-04)	34.9 (23.0 to 49.8)	6e-04 (4e-04 to 8e-04)	92.1 (59.4 to 137)	3.92 (2.96 to 5.40)	0.01 (0.01 to 0.02)	2032 (1531 to 2680)	0.02 (0.01 to 0.02)	2257 (1723 to 2953)	0.35 (0.32 to 0.37)
Tonga	0.74 (0.62 to 0.86)	2468 (2066 to 2857)	1.18 (0.88 to 1.53)	2740 (2048 to 3548)	0.33 (0.29 to 0.37)	0.02 (0.01 to 0.02)	31.0 (22.1 to 42.8)	0.01 (0.010 to 0.02)	26.2 (17.5 to 38.2)	-0.52 (-0.55 to -0.48)	0.72 (0.60 to 0.84)	2438 (2039 to 2826)	1.17 (0.87 to 1.51)	2714 (2030 to 3513)	0.36 (0.29 to 0.40)
Trinidad and Tobago	13.4 (12.7 to 14.2)	3035 (2876 to 3203)	18.9 (14.7 to 23.7)	1880 (1461 to 2363)	-1.42 (-1.62 to -1.20)	0.66 (0.61 to 0.71)	118 (109 to 127)	0.55 (0.41 to 0.71)	70.2 (53.3 to 90.6)	-1.67 (-1.85 to -1.55)	12.7 (12.1 to 13.5)	2917 (2759 to 3085)	18.4 (14.3 to 23.0)	1809 (1407 to 2275)	-1.42 (-1.62 to -1.20)
Tunisia	5.70 (4.63 to 6.96)	223 (182 to 271)	21.4 (15.7 to 28.0)	313 (231 to 410)	1.15 (1.06 to 1.23)	0.87 (0.56 to 1.56)	19.8 (12.9 to 34.7)	0.78 (0.50 to 1.21)	13.0 (8.55 to 20.0)	-1.35 (-1.40 to -1.29)	4.82 (3.97 to 5.99)	203 (167 to 250)	20.6 (15.1 to 27.1)	300 (221 to 396)	1.31 (1.23 to 1.40)
Turkmenistan	3.52 (3.11 to 3.93)	282 (249 to 316)	14.6 (11.2 to 19.2)	598 (460 to 780)	2.87 (2.57 to 3.22)	0.94 (0.86 to 1.03)	58.3 (52.1 to 65.1)	1.75 (1.29 to 2.34)	69.0 (51.1 to 92.1)	0.86 (0.14 to 1.15)	2.58 (2.22 to 2.96)	224 (193 to 257)	12.9 (9.85 to 17.0)	529 (407 to 694)	3.26 (2.96 to 3.59)
Tuvalu	0.09 (0.07 to 0.12)	2321 (1831 to 3009)	0.15 (0.11 to 0.20)	2676 (2024 to 3553)	0.47 (0.44 to 0.50)	0.002 (0.001 to 0.003)	37.4 (25.6 to 59.9)	0.002 (0.001 to 0.002)	27.4 (18.4 to 38.4)	-1.00 (-1.06 to -0.93)	0.09 (0.07 to 0.12)	2284 (1800 to 2969)	0.15 (0.11 to 0.20)	2648 (2003 to 3517)	0.49 (0.46 to 0.52)
Türkiye	146 (125 to 172)	794 (678 to 923)	228 (181 to 281)	465 (371 to 573)	-1.75 (-1.89 to -1.64)	13.6 (9.24 to 20.6)	44.8 (30.4 to 67.0)	4.41 (3.45 to 5.90)	11.2 (8.88 to 15.3)	-4.22 (-4.31 to -4.10)	133 (113 to 157)	750 (641 to 873)	224 (177 to 276)	454 (359 to 558)	-1.65 (-1.79 to -1.53)
Uganda	22.9 (15.3 to 32.0)	650 (433 to 923)	64.7 (44.2 to 88.4)	735 (488 to 1017)	0.37 (0.33 to 0.41)	2.53 (1.60 to 3.75)	24.6 (15.3 to 38.0)	6.05 (3.84 to 8.69)	26.0 (15.8 to 38.1)	0.14 (0.04 to 0.24)	20.3 (13.3 to 29.2)	625 (412 to 891)	58.7 (38.8 to 81.0)	709 (468 to 982)	0.38 (0.35 to 0.42)
Ukraine	50.3 (47.3 to 53.4)	127 (120 to 134)	35.7 (23.4 to 50.7)	89.9 (57.5 to 130)	-1.04 (-1.52 to -0.67)	11.1 (10.1 to 12.2)	39.4 (36.5 to 42.9)	4.53 (2.69 to 6.78)	20.8 (12.7 to 31.0)	-2.42 (-2.93 to -1.95)	39.2 (35.8 to 42.3)	87.4 (79.8 to 94.8)	31.2 (20.8 to 44.0)	69.1 (45.1 to 99.1)	-0.59 (-1.07 to -0.25)
United Arab Emirates	1.53 (1.21 to 1.93)	1016 (785 to 1289)	6.82 (5.43 to 8.34)	1763 (1411 to 2155)	1.66 (1.27 to 2.01)	0.14 (0.10 to 0.21)	24.4 (17.5 to 34.4)	0.29 (0.21 to 0.39)	16.8 (12.5 to 22.3)	-1.22 (-1.43 to -1.02)	1.39 (1.08 to 1.77)	992 (765 to 1257)	6.53 (5.16 to 8.00)	1746 (1395 to 2136)	1.70 (1.31 to 2.06)
United Kingdom	82.4 (77.3 to 85.1)	159 (151 to 164)	45.5 (39.9 to 48.3)	63.8 (58.1 to 66.8)	-3.04 (-3.21 to -2.85)	5.80 (5.69 to 5.88)	17.8 (17.6 to 18.0)	2.64 (2.56 to 2.71)	7.02 (6.85 to 7.15)	-3.15 (-3.46 to -2.97)	76.6 (71.6 to 79.2)	141 (134 to 146)	42.8 (37.3 to 45.7)	56.7 (51.2 to 59.8)	-2.89 (-3.03 to -2.75)
United Republic of Tanzania	43.4 (35.0 to 53.6)	693 (564 to 857)	102 (79.2 to 132)	711 (560 to 917)	0.12 (0.09 to 0.16)	6.45 (4.27 to 9.09)	38.8 (28.2 to 53.1)	10.3 (6.90 to 14.6)	30.5 (20.4 to 42.8)	-0.78 (-0.82 to -0.74)	36.9 (30.1 to 46.3)	654 (530 to 809)	92.1 (71.0 to 120)	681 (534 to 881)	0.16 (0.12 to 0.20)
United States Virgin Islands	0.47 (0.40 to 0.56)	1047 (879 to 1230)	0.47 (0.36 to 0.60)	509 (384 to 655)	-2.36 (-2.51 to -2.23)	0.04 (0.03 to 0.06)	80.2 (63.0 to 104)	0.02 (0.01 to 0.04)	44.7 (23.8 to 66.7)	-1.40 (-1.62 to -1.19)	0.43 (0.36 to 0.52)	967 (808 to 1150)	0.45 (0.34 to 0.57)	464 (353 to 598)	-2.44 (-2.65 to -2.29)
United States of America	564 (525 to 589)	323 (305 to 335)	623 (568 to 657)	217 (201 to 227)	-1.24 (-1.30 to -1.17)	47.0 (45.9 to 48.1)	32.3 (31.6 to 32.9)	47.9 (46.2 to 49.5)	26.8 (25.8 to 27.7)	-0.58 (-0.69 to -0.51)	517 (479 to 542)	291 (273 to 303)	575 (521 to 608)	190 (175 to 200)	-1.34 (-1.40 to -1.28)
Uruguay	7.24 (6.67 to 7.87)	327 (303 to 354)	8.64 (7.59 to 9.47)	270 (241 to 295)	-0.79 (-0.95 to -0.63)	0.27 (0.25 to 0.29)	15.7 (14.6 to 16.8)	0.21 (0.19 to 0.23)	10.2 (9.32 to 11.1)	-1.54 (-1.65 to -1.40)	6.97 (6.40 to 7.59)	311 (287 to 338)	8.43 (7.38 to 9.24)	260 (232 to 284)	-0.74 (-0.90 to -0.58)
Uzbekistan	18.5 (17.2 to 19.9)	257 (238 to 277)	101 (85.3 to 119)	652 (551 to 762)	3.01 (2.80 to 3.18)	3.81 (3.34 to 4.36)	39.8 (33.8 to 46.4)	9.25 (7.53 to 11.1)	52.9 (43.3 to 63.1)	0.96 (0.68 to 1.20)	14.7 (13.4 to 16.1)	218 (198 to 238)	92.1 (76.8 to 108)	599 (502 to 702)	3.29 (3.06 to 3.47)
Vanuatu	0.54 (0.40 to 0.74)	1744 (1298 to 2339)	2.18 (1.71 to 2.64)	2293 (1804 to 2752)	0.87 (0.81 to 0.93)	0.02 (0.01 to 0.04)	28.0 (17.0 to 41.0)	0.05 (0.03 to 0.07)	29.4 (20.2 to 40.1)	0.07 (-0.03 to 0.16)	0.52 (0.38 to 0.70)	1716 (1277 to 2301)	2.13 (1.67 to 2.58)	2264 (1781 to 2709)	0.88 (0.82 to 0.94)
Venezuela (Bolivarian Republic of)	41.7 (39.4 to 44.0)	790 (741 to 837)	137 (107 to 174)	849 (665 to 1076)	0.45 (-0.13 to 0.70)	2.41 (2.27 to 2.56)	28.1 (26.5 to 29.7)	4.51 (3.44 to 5.78)	32.2 (24.5 to 41.1)	0.95 (-0.02 to 1.41)	39.3 (37.1 to 41.6)	762 (714 to 808)	133 (104 to 169)	817 (640 to 1037)	0.27 (0.10 to 0.45)
Viet Nam	165 (130 to 212)	698 (553 to 903)	398 (313 to 505)	728 (575 to 916)	0.15 (0.13 to 0.17)	11.0 (7.30 to 16.3)	38.4 (24.9 to 59.5)	13.0 (7.43 to 20.6)	23.3 (13.6 to 36.6)	-1.60 (-1.61 to -1.59)	154 (121 to 200)	659 (521 to 859)	385 (304 to 488)	704 (554 to 887)	0.23 (0.21 to 0.25)
Yemen	9.68 (6.82 to 14.8)	309 (222 to 489)	30.3 (21.2 to 47.1)	380 (266 to 591)	0.66 (0.61 to 0.70)	2.65 (1.45 to 5.07)	31.3 (17.5 to 59.3)	4.85 (3.10 to 8.15)	26.3 (16.5 to 44.1)	-0.61 (-0.70 to -0.51)	7.03 (4.94 to 11.3)	278 (199 to 439)	25.5 (17.6 to 40.1)	354 (246 to 549)	0.78 (0.73 to 0.81)
Zambia	14.8 (12.0 to 18.2)	901 (729 to 1113)	30.4 (21.4 to 42.0)	754 (547 to 1016)	-0.57 (-0.61 to -0.53)	2.36 (1.58 to 3.43)	50.3 (36.1 to 74.2)	3.18 (2.07 to 4.75)	30.3 (20.1 to 44.8)	-1.58 (-1.61 to -1.54)	12.5 (10.00 to 15.6)	850 (685 to 1059)	27.2 (19.1 to 37.4)	724 (525 to 981)	-0.52 (-0.56 to -0.48)
Zimbabwe	17.7 (14.2 to 21.7)	859 (694 to 1053)	70.2 (51.9 to 93.7)	1729 (1294 to 2264)	2.33 (2.27 to 2.39)	0.48 (0.34 to 0.69)	8.40 (6.21 to 11.5)	1.47 (0.96 to 2.16)	16.9 (10.7 to 25.9)	2.21 (2.05 to 2.37)	17.2 (13.8 to 21.1)	851 (687 to 1043)	68.7 (50.8 to 91.7)	1712 (1284 to 2240)	2.33 (2.27 to 2.39)

AAPC: Average annual percentage change; ASR: Age-standardized rate; DM: Diabetes mellitus; SDI: Sociodemographic index; T1D: Type 1 diabetes; T2D: Type 2 diabetes; YLL: Years of life lost. Results assuming first-order autocorrelation estimated from the data were broadly similar to those of the primary analyses, despite somewhat different estimates.

Table S6. DALY number, age-standardized rate, and AAPC of diabetes and two subtypes among **women** globally and by different SDI, regions, countries, and territories, 1990-2021

Location	DM, Number in 1990 (000s)	DM, ASR in 1990 (per 100,000)	DM, Number in 2021 (000s)	DM, ASR in 2021 (per 100,000)	DM, AAPC (95%CI)	T1D, Number in 1990 (000s)	T1D, ASR in 1990 (per 100,000)	T1D, Number in 2021 (000s)	T1D, ASR in 2021 (per 100,000)	T1D, AAPC (95%CI)	T2D, Number in 1990 (000s)	T2D, ASR in 1990 (per 100,000)	T2D, Number in 2021 (000s)	T2D, ASR in 2021 (per 100,000)	T2D, AAPC (95%CI)
**Global**	14056 (12380 to 16202)	642 (566 to 738)	38838 (33022 to 46503)	861 (729 to 1031)	0.96 (0.95 to 0.98)	1273 (1081 to 1544)	50.2 (42.7 to 61.0)	1689 (1411 to 2026)	41.4 (34.6 to 49.5)	-0.61 (-0.64 to -0.59)	12784 (11243 to 14729)	592 (522 to 680)	37149 (31476 to 44557)	819 (693 to 983)	1.07 (1.05 to 1.09)
**By SDI**															
High SDI	2837 (2476 to 3308)	466 (404 to 546)	5847 (4612 to 7386)	599 (466 to 769)	0.81 (0.78 to 0.83)	237 (204 to 279)	46.5 (40.1 to 54.8)	283 (220 to 361)	41.5 (32.3 to 52.6)	-0.37 (-0.40 to -0.35)	2600 (2268 to 3048)	419 (363 to 496)	5563 (4389 to 7045)	558 (432 to 717)	0.92 (0.89 to 0.94)
High-middle SDI	2812 (2428 to 3345)	503 (434 to 598)	6453 (5247 to 7887)	634 (511 to 778)	0.77 (0.74 to 0.80)	230 (197 to 269)	42.1 (36.1 to 49.5)	229 (182 to 287)	29.4 (23.9 to 36.7)	-1.15 (-1.22 to -1.08)	2582 (2212 to 3079)	461 (394 to 549)	6224 (5068 to 7604)	604 (486 to 741)	0.89 (0.85 to 0.92)
Middle SDI	4449 (3898 to 5139)	786 (693 to 902)	13651 (11648 to 16233)	962 (822 to 1142)	0.66 (0.65 to 0.68)	361 (306 to 439)	45.8 (39.1 to 56.3)	456 (389 to 548)	35.1 (30.0 to 42.3)	-0.84 (-0.86 to -0.82)	4088 (3566 to 4724)	740 (650 to 850)	13194 (11255 to 15699)	927 (792 to 1101)	0.74 (0.72 to 0.75)
Low-middle SDI	2730 (2413 to 3066)	829 (729 to 928)	9508 (8278 to 11174)	1223 (1066 to 1432)	1.27 (1.23 to 1.30)	301 (251 to 415)	59.3 (49.0 to 84.8)	472 (388 to 560)	50.5 (41.5 to 60.1)	-0.50 (-0.53 to -0.47)	2428 (2144 to 2741)	769 (679 to 864)	9036 (7836 to 10684)	1173 (1022 to 1379)	1.37 (1.33 to 1.40)
Low SDI	1207 (1058 to 1369)	962 (848 to 1088)	3333 (2888 to 3941)	1146 (1005 to 1347)	0.57 (0.55 to 0.59)	142 (109 to 195)	60.4 (48.2 to 85.9)	247 (197 to 304)	47.8 (38.4 to 57.9)	-0.75 (-0.77 to -0.73)	1064 (939 to 1210)	901 (800 to 1019)	3086 (2676 to 3670)	1098 (960 to 1293)	0.64 (0.63 to 0.65)
**Four world regions**															
Africa	1513 (1348 to 1684)	963 (862 to 1066)	4879 (4245 to 5706)	1332 (1176 to 1549)	1.04 (1.00 to 1.07)	151 (115 to 199)	48.2 (38.8 to 61.9)	248 (192 to 303)	37.6 (30.2 to 46.5)	-0.80 (-0.81 to -0.79)	1362 (1214 to 1517)	915 (817 to 1017)	4631 (4030 to 5437)	1294 (1141 to 1504)	1.11 (1.07 to 1.14)
America	2879 (2569 to 3285)	859 (766 to 982)	7428 (6203 to 9055)	1069 (891 to 1305)	0.71 (0.69 to 0.74)	203 (177 to 237)	57.1 (49.7 to 66.9)	310 (260 to 374)	53.7 (45.4 to 64.6)	-0.17 (-0.21 to -0.15)	2676 (2385 to 3050)	802 (715 to 915)	7118 (5942 to 8674)	1015 (844 to 1240)	0.77 (0.74 to 0.79)
Asia	7018 (6102 to 8167)	632 (552 to 732)	21915 (18491 to 26118)	834 (703 to 994)	0.90 (0.88 to 0.91)	697 (583 to 918)	47.6 (39.7 to 63.6)	899 (746 to 1079)	37.5 (31.1 to 45.0)	-0.75 (-0.77 to -0.72)	6320 (5463 to 7395)	585 (509 to 678)	21016 (17669 to 25186)	796 (669 to 954)	1.00 (0.98 to 1.02)
Europe	2588 (2262 to 3022)	434 (376 to 511)	4505 (3675 to 5589)	556 (446 to 701)	0.79 (0.76 to 0.82)	217 (189 to 253)	47.0 (40.9 to 54.8)	227 (174 to 297)	41.0 (31.7 to 53.1)	-0.42 (-0.49 to -0.36)	2371 (2069 to 2769)	387 (335 to 456)	4278 (3481 to 5325)	515 (413 to 651)	0.91 (0.88 to 0.94)
**Six WHO regions**															
African Region	1252 (1109 to 1404)	1006 (898 to 1123)	3680 (3226 to 4270)	1259 (1113 to 1444)	0.73 (0.69 to 0.75)	121 (89.1 to 157)	48.6 (38.3 to 63.5)	201 (153 to 249)	36.3 (28.8 to 45.1)	-0.93 (-0.95 to -0.91)	1131 (1003 to 1268)	958 (853 to 1069)	3480 (3056 to 4042)	1222 (1080 to 1400)	0.77 (0.74 to 0.80)
Region of the Americas	2879 (2569 to 3285)	859 (766 to 982)	7428 (6203 to 9055)	1069 (891 to 1305)	0.71 (0.69 to 0.74)	203 (177 to 237)	57.1 (49.7 to 66.9)	310 (260 to 374)	53.7 (45.4 to 64.6)	-0.17 (-0.21 to -0.15)	2676 (2385 to 3050)	802 (715 to 915)	7118 (5942 to 8674)	1015 (844 to 1240)	0.77 (0.74 to 0.79)
South-East Asia Region	3024 (2621 to 3431)	790 (687 to 892)	10498 (9070 to 12367)	1090 (942 to 1278)	1.09 (1.05 to 1.13)	347 (281 to 500)	61.2 (49.6 to 90.6)	499 (414 to 605)	47.4 (39.4 to 57.5)	-0.80 (-0.85 to -0.73)	2677 (2316 to 3035)	729 (633 to 824)	9999 (8589 to 11817)	1043 (898 to 1226)	1.21 (1.17 to 1.26)
European Region	2691 (2351 to 3141)	436 (378 to 513)	4874 (3993 to 6057)	576 (465 to 727)	0.90 (0.86 to 0.92)	231 (202 to 269)	47.4 (41.4 to 54.9)	253 (196 to 327)	43.0 (33.7 to 54.7)	-0.29 (-0.36 to -0.23)	2460 (2143 to 2872)	389 (336 to 458)	4621 (3771 to 5749)	533 (430 to 675)	1.02 (0.98 to 1.05)
Eastern Mediterranean Region	818 (721 to 930)	875 (779 to 995)	3707 (3128 to 4473)	1559 (1321 to 1876)	1.90 (1.87 to 1.91)	93.7 (77.0 to 121)	55.8 (45.8 to 70.8)	199 (155 to 244)	56.6 (44.6 to 69.4)	0.06 (0.04 to 0.08)	724 (638 to 831)	820 (726 to 935)	3508 (2934 to 4262)	1503 (1270 to 1813)	1.99 (1.97 to 2.01)
Western Pacific Region	3238 (2717 to 3917)	513 (433 to 616)	8348 (6680 to 10400)	598 (475 to 753)	0.51 (0.49 to 0.53)	264 (213 to 324)	35.6 (28.8 to 44.2)	215 (171 to 267)	20.3 (16.5 to 25.0)	-1.79 (-1.81 to -1.76)	2975 (2467 to 3589)	478 (400 to 573)	8133 (6497 to 10145)	577 (457 to 729)	0.63 (0.61 to 0.66)
**Seven super regions**															
Southeast Asia, East Asia, and Oceania	3836 (3281 to 4500)	615 (529 to 716)	10344 (8581 to 12532)	710 (588 to 863)	0.48 (0.45 to 0.50)	345 (285 to 456)	43.4 (36.0 to 58.2)	317 (262 to 397)	26.7 (22.3 to 33.1)	-1.53 (-1.55 to -1.50)	3490 (2975 to 4115)	572 (491 to 667)	10027 (8310 to 12189)	683 (564 to 835)	0.59 (0.57 to 0.61)
Central Europe, Eastern Europe, and Central Asia	1117 (947 to 1328)	394 (334 to 470)	2457 (2061 to 2996)	669 (560 to 820)	1.73 (1.68 to 1.77)	122 (110 to 137)	51.0 (46.2 to 57.2)	117 (98.4 to 142)	46.8 (39.9 to 55.9)	-0.16 (-0.28 to -0.05)	995 (835 to 1192)	343 (285 to 412)	2339 (1961 to 2859)	622 (520 to 766)	1.94 (1.90 to 1.98)
South Asia	2285 (1992 to 2605)	757 (659 to 856)	8697 (7425 to 10308)	1114 (957 to 1311)	1.25 (1.17 to 1.31)	274 (223 to 381)	59.1 (47.6 to 84.4)	463 (367 to 563)	50.9 (40.4 to 62.2)	-0.44 (-0.50 to -0.36)	2011 (1740 to 2314)	698 (605 to 794)	8234 (6979 to 9793)	1063 (911 to 1256)	1.36 (1.28 to 1.43)
High-income	3081 (2697 to 3584)	464 (404 to 545)	5827 (4567 to 7420)	572 (442 to 741)	0.67 (0.66 to 0.69)	234 (197 to 281)	44.0 (37.1 to 53.1)	311 (234 to 403)	44.5 (33.8 to 57.6)	0.01 (-0.03 to 0.03)	2847 (2504 to 3321)	420 (365 to 495)	5517 (4329 to 7032)	528 (406 to 686)	0.73 (0.72 to 0.75)
Latin America and Caribbean	1732 (1552 to 1945)	1409 (1271 to 1574)	4762 (4092 to 5685)	1412 (1213 to 1684)	0.01 (-0.03 to 0.05)	95.3 (84.7 to 109)	53.1 (47.0 to 61.2)	158 (136 to 185)	49.3 (42.7 to 57.8)	-0.23 (-0.27 to -0.19)	1636 (1466 to 1838)	1356 (1224 to 1515)	4604 (3950 to 5491)	1362 (1169 to 1625)	0.02 (-0.03 to 0.06)
North Africa and Middle East	783 (685 to 910)	875 (771 to 1011)	3270 (2662 to 4069)	1381 (1141 to 1702)	1.48 (1.45 to 1.51)	85.1 (66.7 to 120)	51.4 (39.9 to 69.9)	124 (100 to 156)	41.6 (33.5 to 52.1)	-0.67 (-0.70 to -0.61)	698 (611 to 815)	824 (731 to 954)	3146 (2558 to 3927)	1339 (1106 to 1655)	1.58 (1.55 to 1.61)
Sub-Saharan Africa	1223 (1088 to 1372)	1029 (918 to 1153)	3482 (3053 to 4028)	1256 (1117 to 1441)	0.64 (0.61 to 0.67)	118 (85.6 to 153)	48.9 (38.5 to 64.5)	199 (150 to 248)	36.7 (29.1 to 45.9)	-0.93 (-0.94 to -0.91)	1105 (979 to 1240)	980 (873 to 1093)	3283 (2881 to 3803)	1219 (1081 to 1398)	0.71 (0.67 to 0.74)
**21 regions**															
East Asia	2379 (1952 to 2910)	499 (412 to 605)	5871 (4586 to 7454)	549 (426 to 704)	0.34 (0.30 to 0.37)	205 (162 to 256)	35.6 (28.0 to 45.2)	130 (103 to 164)	15.8 (12.6 to 19.9)	-2.57 (-2.61 to -2.52)	2175 (1761 to 2672)	463 (379 to 564)	5741 (4471 to 7311)	533 (412 to 684)	0.48 (0.45 to 0.51)
Oceania	40.2 (34.0 to 48.4)	2459 (2106 to 2927)	138 (117 to 164)	3315 (2824 to 3892)	0.92 (0.89 to 0.96)	1.61 (1.11 to 2.21)	48.4 (34.1 to 66.6)	3.46 (2.52 to 4.66)	49.0 (36.1 to 65.4)	0.15 (0.08 to 0.22)	38.6 (32.7 to 46.4)	2410 (2064 to 2875)	135 (114 to 160)	3266 (2781 to 3841)	0.94 (0.90 to 0.97)
Southeast Asia	1416 (1246 to 1624)	971 (853 to 1105)	4334 (3739 to 5088)	1218 (1053 to 1428)	0.74 (0.71 to 0.76)	139 (112 to 200)	67.0 (53.6 to 96.2)	183 (150 to 234)	49.4 (40.4 to 62.7)	-0.97 (-0.98 to -0.95)	1277 (1114 to 1451)	904 (792 to 1024)	4151 (3571 to 4899)	1169 (1009 to 1378)	0.83 (0.80 to 0.86)
Central Asia	134 (116 to 158)	467 (401 to 551)	440 (363 to 543)	908 (751 to 1116)	2.17 (2.08 to 2.25)	19.6 (17.5 to 22.0)	60.2 (53.7 to 68.0)	31.1 (26.4 to 36.1)	63.2 (53.9 to 73.2)	0.18 (0.07 to 0.26)	115 (96.9 to 137)	407 (343 to 487)	409 (336 to 506)	845 (696 to 1042)	2.38 (2.29 to 2.45)
Central Europe	494 (419 to 581)	583 (495 to 687)	802 (643 to 998)	666 (530 to 838)	0.42 (0.37 to 0.47)	44.9 (41.5 to 49.5)	59.9 (55.3 to 66.3)	28.0 (22.7 to 34.6)	33.9 (27.5 to 42.4)	-1.81 (-1.87 to -1.76)	449 (376 to 532)	523 (438 to 622)	774 (620 to 965)	632 (501 to 798)	0.57 (0.52 to 0.62)
Eastern Europe	489 (405 to 592)	291 (242 to 351)	1215 (1025 to 1465)	589 (493 to 718)	2.34 (2.24 to 2.42)	57.3 (50.6 to 66.1)	44.6 (39.9 to 50.8)	58.4 (47.4 to 73.6)	45.9 (38.5 to 56.2)	0.26 (0.07 to 0.43)	432 (351 to 526)	246 (200 to 301)	1156 (978 to 1399)	544 (454 to 663)	2.66 (2.57 to 2.74)
Australasia	45.7 (38.9 to 54.1)	361 (307 to 430)	103 (81.0 to 133)	396 (305 to 517)	0.31 (0.25 to 0.37)	4.77 (3.86 to 5.89)	41.9 (33.9 to 51.9)	8.88 (6.45 to 12.0)	45.2 (33.1 to 60.6)	0.24 (0.21 to 0.27)	40.9 (35.2 to 48.3)	319 (272 to 377)	94.4 (74.3 to 121)	350 (274 to 453)	0.33 (0.27 to 0.38)
High-income Asia Pacific	457 (378 to 565)	411 (339 to 509)	1067 (793 to 1409)	541 (393 to 735)	0.91 (0.87 to 0.94)	25.3 (20.5 to 31.6)	26.6 (21.5 to 33.2)	23.0 (15.6 to 31.5)	19.1 (13.2 to 26.7)	-1.09 (-1.12 to -1.06)	432 (357 to 534)	384 (317 to 476)	1045 (774 to 1380)	522 (379 to 709)	1.01 (0.97 to 1.04)
High-income North America	1030 (903 to 1196)	545 (477 to 635)	2425 (1901 to 3072)	766 (605 to 966)	1.10 (1.07 to 1.12)	101 (85.4 to 121)	62.1 (52.6 to 74.8)	144 (115 to 181)	63.7 (51.6 to 79.1)	0.08 (0.04 to 0.10)	929 (815 to 1080)	483 (422 to 563)	2282 (1781 to 2898)	703 (550 to 890)	1.20 (1.18 to 1.22)
Southern Latin America	160 (142 to 182)	624 (551 to 709)	319 (255 to 403)	680 (540 to 859)	0.29 (0.21 to 0.37)	10.1 (8.79 to 11.7)	40.0 (34.7 to 46.3)	11.9 (9.64 to 14.8)	30.8 (24.9 to 38.4)	-0.84 (-0.93 to -0.78)	150 (133 to 171)	584 (516 to 663)	307 (245 to 388)	649 (514 to 820)	0.37 (0.32 to 0.44)
Western Europe	1388 (1234 to 1598)	421 (370 to 493)	1912 (1513 to 2468)	440 (333 to 579)	0.13 (0.10 to 0.15)	92.6 (77.1 to 112)	38.9 (32.0 to 47.5)	123 (87.7 to 168)	40.9 (28.6 to 56.0)	0.10 (0.06 to 0.14)	1295 (1157 to 1488)	382 (337 to 446)	1789 (1425 to 2289)	399 (305 to 527)	0.13 (0.10 to 0.15)
Andean Latin America	81.6 (71.5 to 92.6)	736 (645 to 835)	302 (253 to 365)	968 (813 to 1168)	0.95 (0.85 to 1.07)	4.30 (3.44 to 5.34)	24.3 (19.7 to 29.9)	6.92 (5.22 to 8.56)	20.8 (15.6 to 25.7)	-0.53 (-0.59 to -0.48)	77.3 (67.5 to 87.9)	711 (621 to 807)	295 (247 to 357)	947 (794 to 1145)	0.99 (0.88 to 1.12)
Caribbean	247 (220 to 281)	1777 (1591 to 2017)	511 (428 to 614)	1831 (1529 to 2204)	0.12 (0.09 to 0.15)	20.5 (17.3 to 27.1)	121 (103 to 159)	25.1 (18.8 to 33.3)	101 (76.0 to 135)	-0.55 (-0.60 to -0.50)	226 (202 to 257)	1656 (1484 to 1878)	486 (406 to 588)	1730 (1438 to 2097)	0.16 (0.12 to 0.19)
Central Latin America	811 (721 to 916)	1748 (1567 to 1958)	2477 (2108 to 2961)	1805 (1538 to 2156)	0.09 (0.02 to 0.16)	36.0 (32.8 to 40.1)	48.7 (44.2 to 54.4)	56.6 (49.2 to 65.9)	41.8 (36.5 to 48.7)	-0.45 (-0.53 to -0.36)	775 (689 to 877)	1699 (1522 to 1906)	2420 (2059 to 2893)	1763 (1502 to 2106)	0.10 (0.04 to 0.16)
Tropical Latin America	592 (528 to 665)	1156 (1031 to 1298)	1472 (1248 to 1743)	1046 (887 to 1238)	-0.38 (-0.48 to -0.30)	34.5 (29.3 to 41.0)	48.2 (40.4 to 57.8)	69.2 (57.3 to 85.7)	54.6 (45.6 to 67.3)	0.42 (0.36 to 0.48)	558 (498 to 624)	1108 (988 to 1239)	1403 (1188 to 1659)	991 (840 to 1171)	-0.41 (-0.52 to -0.32)
North Africa and Middle East	783 (685 to 910)	875 (771 to 1011)	3270 (2662 to 4069)	1381 (1141 to 1702)	1.48 (1.45 to 1.51)	85.1 (66.7 to 120)	51.4 (39.9 to 69.9)	124 (100 to 156)	41.6 (33.5 to 52.1)	-0.67 (-0.70 to -0.61)	698 (611 to 815)	824 (731 to 954)	3146 (2558 to 3927)	1339 (1106 to 1655)	1.58 (1.55 to 1.61)
South Asia	2285 (1992 to 2605)	757 (659 to 856)	8697 (7425 to 10308)	1114 (957 to 1311)	1.25 (1.17 to 1.31)	274 (223 to 381)	59.1 (47.6 to 84.4)	463 (367 to 563)	50.9 (40.4 to 62.2)	-0.44 (-0.50 to -0.36)	2011 (1740 to 2314)	698 (605 to 794)	8234 (6979 to 9793)	1063 (911 to 1256)	1.36 (1.28 to 1.43)
Central Sub-Saharan Africa	145 (114 to 177)	1112 (875 to 1354)	445 (360 to 560)	1339 (1071 to 1675)	0.59 (0.56 to 0.62)	9.65 (7.33 to 12.8)	39.5 (29.9 to 52.6)	20.6 (15.8 to 26.8)	34.4 (26.0 to 45.5)	-0.47 (-0.51 to -0.44)	135 (105 to 166)	1073 (836 to 1309)	425 (340 to 535)	1304 (1041 to 1630)	0.62 (0.59 to 0.65)
Eastern Sub-Saharan Africa	475 (407 to 546)	1095 (946 to 1246)	990 (855 to 1148)	986 (857 to 1134)	-0.34 (-0.36 to -0.32)	60.6 (43.2 to 84.1)	60.5 (46.8 to 86.6)	89.8 (68.7 to 111)	42.5 (33.3 to 53.1)	-1.12 (-1.13 to -1.11)	415 (353 to 477)	1034 (891 to 1175)	900 (780 to 1043)	944 (822 to 1087)	-0.30 (-0.32 to -0.29)
Southern Sub-Saharan Africa	202 (181 to 225)	1260 (1134 to 1407)	741 (672 to 826)	2195 (2000 to 2440)	1.76 (1.58 to 1.91)	9.99 (7.89 to 12.8)	38.4 (30.6 to 49.1)	14.2 (11.1 to 17.6)	34.7 (27.2 to 43.4)	-0.35 (-0.43 to -0.26)	192 (173 to 214)	1222 (1099 to 1366)	727 (660 to 810)	2161 (1970 to 2399)	1.81 (1.64 to 1.96)
Western Sub-Saharan Africa	401 (337 to 467)	856 (729 to 987)	1306 (1087 to 1572)	1135 (954 to 1350)	0.90 (0.88 to 0.91)	37.5 (25.9 to 48.8)	43.2 (32.5 to 55.2)	74.5 (50.9 to 95.3)	32.7 (23.8 to 41.5)	-0.91 (-0.93 to -0.89)	364 (306 to 422)	813 (690 to 934)	1232 (1025 to 1485)	1103 (927 to 1312)	0.99 (0.96 to 1.00)
**204 countries and territories**															
Afghanistan	50.4 (39.8 to 67.2)	1379 (1108 to 1837)	177 (140 to 228)	2658 (2119 to 3401)	2.17 (2.15 to 2.19)	5.84 (3.61 to 9.91)	114 (72.3 to 187)	16.9 (12.5 to 25.5)	116 (83.1 to 170)	0.09 (0.03 to 0.15)	44.5 (35.5 to 59.1)	1264 (1020 to 1681)	160 (125 to 208)	2542 (2015 to 3257)	2.30 (2.29 to 2.32)
Albania	3.41 (2.66 to 4.32)	308 (240 to 391)	8.89 (6.49 to 11.8)	404 (294 to 536)	0.83 (0.79 to 0.86)	0.45 (0.36 to 0.57)	32.8 (25.6 to 41.1)	0.45 (0.32 to 0.59)	25.9 (18.7 to 34.1)	-0.88 (-0.98 to -0.79)	2.96 (2.24 to 3.79)	275 (210 to 352)	8.45 (6.13 to 11.2)	378 (275 to 506)	0.98 (0.94 to 1.02)
Algeria	41.1 (32.1 to 51.5)	625 (493 to 786)	229 (175 to 294)	1249 (973 to 1587)	2.31 (2.29 to 2.34)	4.98 (3.40 to 8.08)	40.3 (27.9 to 61.3)	7.25 (5.62 to 9.57)	32.9 (25.4 to 43.6)	-0.63 (-0.65 to -0.60)	36.1 (28.2 to 46.1)	584 (462 to 740)	222 (169 to 286)	1216 (945 to 1549)	2.44 (2.42 to 2.47)
American Samoa	0.30 (0.26 to 0.35)	2489 (2140 to 2891)	1.09 (0.90 to 1.31)	4410 (3647 to 5279)	1.92 (1.81 to 2.00)	0.006 (0.005 to 0.009)	25.8 (19.0 to 35.2)	0.007 (0.005 to 0.009)	28.0 (21.3 to 37.1)	0.30 (0.24 to 0.35)	0.30 (0.25 to 0.35)	2463 (2119 to 2858)	1.09 (0.90 to 1.31)	4382 (3626 to 5246)	1.93 (1.82 to 2.01)
Andorra	0.08 (0.06 to 0.11)	298 (231 to 383)	0.29 (0.23 to 0.39)	412 (315 to 556)	1.00 (0.93 to 1.04)	0.01 (0.008 to 0.01)	38.8 (28.6 to 51.1)	0.03 (0.02 to 0.03)	44.6 (32.1 to 60.9)	0.42 (0.36 to 0.47)	0.07 (0.06 to 0.09)	260 (203 to 332)	0.27 (0.21 to 0.36)	367 (280 to 496)	1.08 (1.00 to 1.12)
Angola	22.2 (17.8 to 27.4)	998 (804 to 1225)	90.3 (71.3 to 114)	1200 (957 to 1523)	0.59 (0.51 to 0.66)	1.73 (1.25 to 2.38)	38.5 (28.2 to 52.8)	4.40 (3.16 to 5.99)	30.8 (22.4 to 42.1)	-0.66 (-0.74 to -0.54)	20.4 (16.3 to 25.2)	960 (771 to 1175)	85.9 (67.6 to 110)	1169 (930 to 1487)	0.63 (0.56 to 0.69)
Antigua and Barbuda	0.60 (0.53 to 0.67)	2007 (1785 to 2275)	1.23 (1.04 to 1.49)	2171 (1841 to 2608)	0.18 (0.04 to 0.33)	0.04 (0.04 to 0.05)	137 (123 to 155)	0.04 (0.04 to 0.05)	80.0 (70.7 to 92.0)	-1.77 (-1.84 to -1.72)	0.56 (0.49 to 0.63)	1870 (1657 to 2128)	1.19 (1.00 to 1.44)	2091 (1770 to 2521)	0.29 (0.14 to 0.44)
Argentina	119 (105 to 134)	663 (589 to 750)	209 (169 to 259)	691 (557 to 864)	0.17 (0.10 to 0.25)	7.51 (6.53 to 8.65)	44.1 (38.3 to 51.0)	8.67 (7.11 to 10.7)	33.8 (27.7 to 41.7)	-0.85 (-0.95 to -0.78)	111 (98.9 to 127)	619 (549 to 704)	200 (162 to 249)	657 (530 to 823)	0.23 (0.15 to 0.30)
Armenia	12.9 (11.4 to 15.0)	799 (703 to 926)	18.5 (15.0 to 22.8)	780 (637 to 967)	-0.21 (-0.46 to -0.01)	1.56 (1.42 to 1.72)	89.7 (81.6 to 99.5)	1.12 (0.94 to 1.33)	61.6 (52.0 to 71.8)	-1.19 (-1.45 to -1.01)	11.3 (9.88 to 13.2)	710 (618 to 827)	17.4 (14.0 to 21.6)	718 (582 to 897)	-0.09 (-0.35 to 0.12)
Australia	36.6 (31.2 to 43.3)	344 (292 to 410)	85.4 (67.0 to 110)	386 (299 to 505)	0.38 (0.31 to 0.45)	3.86 (3.09 to 4.83)	40.9 (32.6 to 51.1)	8.00 (5.81 to 10.7)	48.7 (35.6 to 65.4)	0.55 (0.52 to 0.58)	32.7 (28.1 to 38.6)	303 (259 to 358)	77.4 (61.1 to 100)	338 (264 to 439)	0.36 (0.28 to 0.43)
Austria	26.2 (23.5 to 29.0)	357 (318 to 405)	32.3 (26.0 to 40.5)	352 (272 to 455)	-0.01 (-0.12 to 0.10)	1.81 (1.48 to 2.20)	35.8 (29.3 to 43.5)	2.57 (1.85 to 3.53)	40.9 (29.2 to 56.7)	0.43 (0.40 to 0.46)	24.4 (21.9 to 27.0)	322 (288 to 360)	29.7 (24.2 to 37.0)	311 (242 to 400)	-0.05 (-0.17 to 0.06)
Azerbaijan	16.3 (13.6 to 19.6)	524 (437 to 631)	57.8 (44.6 to 74.3)	957 (744 to 1224)	1.98 (1.90 to 2.05)	2.74 (2.17 to 3.43)	76.7 (60.3 to 95.9)	4.13 (2.89 to 5.57)	71.0 (50.9 to 93.6)	-0.21 (-0.30 to -0.10)	13.5 (11.1 to 16.5)	447 (365 to 549)	53.7 (41.4 to 69.0)	886 (688 to 1137)	2.24 (2.17 to 2.30)
Bahamas	1.69 (1.48 to 1.94)	1849 (1617 to 2113)	3.85 (3.08 to 4.85)	1678 (1354 to 2099)	-0.27 (-0.40 to -0.13)	0.15 (0.13 to 0.16)	119 (106 to 135)	0.17 (0.14 to 0.21)	77.7 (62.4 to 96.2)	-1.37 (-1.46 to -1.29)	1.55 (1.35 to 1.78)	1730 (1507 to 1980)	3.67 (2.93 to 4.66)	1600 (1283 to 2012)	-0.21 (-0.33 to -0.06)
Bahrain	1.99 (1.71 to 2.31)	2473 (2136 to 2845)	12.3 (10.2 to 14.8)	3389 (2855 to 3983)	0.98 (0.89 to 1.06)	0.14 (0.12 to 0.19)	71.2 (56.7 to 88.0)	0.25 (0.20 to 0.32)	44.3 (35.3 to 55.9)	-1.50 (-1.56 to -1.44)	1.85 (1.58 to 2.14)	2402 (2070 to 2765)	12.0 (9.95 to 14.5)	3344 (2815 to 3938)	1.03 (0.93 to 1.11)
Bangladesh	211 (176 to 246)	861 (734 to 993)	879 (717 to 1088)	1284 (1057 to 1586)	1.36 (1.29 to 1.45)	38.0 (26.7 to 54.9)	87.7 (63.8 to 128)	60.8 (45.5 to 79.3)	70.8 (52.7 to 92.6)	-0.57 (-0.64 to -0.50)	173 (146 to 200)	773 (657 to 896)	818 (666 to 1022)	1213 (1004 to 1497)	1.53 (1.45 to 1.63)
Barbados	3.86 (3.49 to 4.32)	2370 (2134 to 2660)	5.79 (4.83 to 7.04)	2157 (1789 to 2627)	-0.29 (-0.47 to -0.16)	0.21 (0.19 to 0.23)	150 (136 to 168)	0.18 (0.15 to 0.23)	95.5 (77.1 to 118)	-1.51 (-1.59 to -1.45)	3.65 (3.30 to 4.09)	2220 (1998 to 2495)	5.61 (4.67 to 6.81)	2061 (1712 to 2508)	-0.22 (-0.40 to -0.09)
Belarus	20.0 (16.4 to 24.1)	259 (211 to 315)	28.5 (21.7 to 36.9)	328 (252 to 425)	0.85 (0.69 to 0.99)	2.41 (2.08 to 2.82)	40.1 (35.0 to 45.9)	2.43 (1.97 to 3.04)	44.2 (36.2 to 54.1)	0.47 (0.21 to 0.73)	17.6 (14.1 to 21.5)	219 (175 to 270)	26.0 (19.6 to 34.1)	283 (213 to 373)	0.89 (0.77 to 1.01)
Belgium	35.0 (30.0 to 40.9)	407 (343 to 485)	46.2 (34.6 to 62.0)	438 (319 to 599)	0.16 (-0.06 to 0.37)	2.58 (2.18 to 3.13)	40.2 (33.6 to 49.2)	2.74 (1.91 to 3.72)	35.0 (24.0 to 48.4)	-0.47 (-0.52 to -0.44)	32.4 (27.8 to 38.0)	367 (309 to 436)	43.5 (32.5 to 57.6)	403 (293 to 551)	0.23 (0.00 to 0.46)
Belize	0.95 (0.85 to 1.07)	1905 (1714 to 2145)	3.76 (3.22 to 4.45)	2341 (2008 to 2764)	0.63 (0.47 to 0.78)	0.09 (0.08 to 0.10)	105 (93.2 to 118)	0.14 (0.12 to 0.17)	68.7 (58.9 to 80.6)	-1.19 (-1.31 to -1.05)	0.86 (0.77 to 0.97)	1800 (1617 to 2029)	3.62 (3.09 to 4.29)	2272 (1948 to 2686)	0.71 (0.54 to 0.86)
Benin	9.15 (7.47 to 10.9)	787 (649 to 934)	40.4 (32.1 to 49.8)	1266 (1022 to 1533)	1.55 (1.52 to 1.58)	1.01 (0.70 to 1.40)	43.0 (32.2 to 56.5)	2.17 (1.49 to 3.04)	33.9 (24.7 to 45.4)	-0.76 (-0.81 to -0.72)	8.14 (6.68 to 9.74)	744 (612 to 887)	38.3 (30.4 to 47.4)	1232 (993 to 1490)	1.65 (1.62 to 1.67)
Bermuda	0.36 (0.31 to 0.41)	1012 (891 to 1168)	0.54 (0.42 to 0.69)	804 (627 to 1053)	-0.79 (-0.89 to -0.72)	0.02 (0.02 to 0.03)	69.7 (61.3 to 80.6)	0.02 (0.01 to 0.02)	33.9 (26.8 to 43.6)	-2.34 (-2.43 to -2.25)	0.33 (0.29 to 0.38)	942 (828 to 1090)	0.52 (0.41 to 0.67)	770 (600 to 1011)	-0.70 (-0.80 to -0.62)
Bhutan	1.03 (0.82 to 1.30)	747 (598 to 932)	3.13 (2.45 to 3.92)	1019 (800 to 1266)	1.02 (1.00 to 1.05)	0.13 (0.08 to 0.20)	54.8 (34.3 to 88.5)	0.16 (0.11 to 0.22)	42.7 (29.5 to 59.3)	-0.85 (-0.93 to -0.76)	0.90 (0.72 to 1.12)	692 (553 to 860)	2.97 (2.33 to 3.74)	977 (767 to 1221)	1.14 (1.12 to 1.16)
Bolivia (Plurinational State of)	24.9 (20.8 to 29.7)	1388 (1170 to 1652)	75.9 (61.1 to 94.2)	1547 (1254 to 1921)	0.35 (0.33 to 0.38)	1.40 (0.99 to 2.04)	46.7 (33.9 to 70.6)	1.91 (1.22 to 2.70)	33.1 (20.9 to 47.1)	-1.11 (-1.12 to -1.09)	23.5 (19.8 to 28.1)	1342 (1130 to 1602)	74.0 (59.5 to 92.3)	1514 (1227 to 1879)	0.39 (0.37 to 0.42)
Bosnia and Herzegovina	15.8 (13.2 to 18.8)	683 (569 to 814)	41.2 (33.4 to 50.9)	1190 (953 to 1479)	1.83 (1.76 to 1.89)	1.74 (1.39 to 2.11)	72.6 (58.2 to 88.7)	1.14 (0.86 to 1.61)	47.6 (36.4 to 63.6)	-1.37 (-1.47 to -1.28)	14.1 (11.6 to 16.9)	611 (503 to 728)	40.1 (32.4 to 49.6)	1142 (914 to 1427)	2.07 (2.01 to 2.13)
Botswana	4.63 (3.44 to 5.98)	1458 (1081 to 1881)	14.0 (11.4 to 17.2)	1685 (1391 to 2068)	0.32 (0.19 to 0.49)	0.27 (0.18 to 0.37)	40.5 (28.7 to 54.3)	0.39 (0.27 to 0.54)	33.0 (23.1 to 44.3)	-0.64 (-0.69 to -0.58)	4.36 (3.21 to 5.69)	1417 (1047 to 1838)	13.6 (11.0 to 16.8)	1652 (1364 to 2031)	0.35 (0.22 to 0.52)
Brazil	580 (517 to 650)	1160 (1034 to 1300)	1415 (1198 to 1682)	1028 (871 to 1222)	-0.36 (-0.46 to -0.28)	33.6 (28.5 to 40.0)	48.4 (40.6 to 58.1)	67.8 (55.9 to 84.0)	55.0 (45.8 to 67.8)	0.43 (0.38 to 0.49)	546 (488 to 612)	1112 (990 to 1244)	1347 (1142 to 1599)	973 (825 to 1155)	-0.43 (-0.47 to -0.40)
Brunei Darussalam	1.20 (1.06 to 1.36)	2233 (1964 to 2536)	3.62 (2.90 to 4.48)	1922 (1564 to 2343)	-0.53 (-0.58 to -0.47)	0.15 (0.11 to 0.19)	132 (97.6 to 175)	0.16 (0.12 to 0.20)	66.7 (52.4 to 84.3)	-2.21 (-2.27 to -2.17)	1.05 (0.92 to 1.20)	2101 (1839 to 2383)	3.46 (2.76 to 4.30)	1855 (1508 to 2264)	-0.46 (-0.51 to -0.40)
Bulgaria	44.9 (38.1 to 52.7)	679 (579 to 796)	59.8 (48.4 to 74.7)	791 (634 to 995)	0.46 (0.38 to 0.53)	3.92 (3.45 to 4.56)	74.1 (65.0 to 85.2)	2.69 (2.13 to 3.36)	55.5 (44.2 to 69.1)	-0.93 (-1.03 to -0.84)	41.0 (34.6 to 48.2)	604 (510 to 713)	57.2 (46.0 to 71.5)	736 (588 to 928)	0.60 (0.52 to 0.67)
Burkina Faso	19.5 (16.1 to 23.7)	802 (659 to 971)	51.3 (41.7 to 63.7)	911 (747 to 1122)	0.41 (0.38 to 0.43)	2.23 (1.50 to 3.16)	50.6 (36.8 to 68.1)	3.88 (2.58 to 5.48)	34.6 (24.7 to 46.2)	-1.21 (-1.24 to -1.18)	17.3 (14.2 to 21.1)	751 (612 to 911)	47.4 (38.4 to 59.5)	876 (718 to 1081)	0.49 (0.46 to 0.52)
Burundi	17.0 (12.9 to 21.9)	1220 (907 to 1572)	30.0 (22.6 to 37.7)	1110 (834 to 1403)	-0.32 (-0.34 to -0.29)	1.78 (1.25 to 2.42)	60.5 (45.3 to 82.9)	3.02 (2.05 to 3.93)	46.9 (33.2 to 62.6)	-0.82 (-0.85 to -0.78)	15.3 (11.2 to 19.9)	1159 (853 to 1493)	27.0 (20.1 to 34.1)	1063 (794 to 1347)	-0.30 (-0.32 to -0.27)
Cabo Verde	0.65 (0.52 to 0.79)	480 (386 to 592)	3.10 (2.50 to 3.81)	1202 (973 to 1485)	3.03 (3.00 to 3.07)	0.05 (0.03 to 0.06)	28.3 (20.7 to 37.8)	0.06 (0.05 to 0.08)	22.6 (16.9 to 29.6)	-0.74 (-0.78 to -0.69)	0.60 (0.48 to 0.74)	452 (360 to 563)	3.03 (2.45 to 3.74)	1179 (955 to 1456)	3.17 (3.14 to 3.21)
Cambodia	26.3 (22.2 to 33.1)	902 (756 to 1129)	87.9 (68.6 to 115)	1187 (932 to 1541)	0.90 (0.88 to 0.91)	3.46 (2.57 to 5.93)	75.4 (54.6 to 132)	4.43 (3.24 to 6.07)	51.4 (37.8 to 70.3)	-1.24 (-1.26 to -1.16)	22.8 (19.3 to 28.5)	827 (692 to 1032)	83.5 (64.9 to 109)	1135 (889 to 1478)	1.04 (1.02 to 1.05)
Cameroon	23.7 (18.6 to 28.7)	948 (738 to 1145)	99.5 (74.8 to 129)	1362 (1041 to 1749)	1.19 (1.17 to 1.21)	2.32 (1.62 to 3.09)	50.0 (35.7 to 68.6)	5.19 (3.45 to 7.18)	36.9 (25.7 to 52.2)	-0.97 (-1.01 to -0.95)	21.3 (16.7 to 25.9)	898 (700 to 1092)	94.3 (71.4 to 122)	1325 (1011 to 1697)	1.28 (1.26 to 1.30)
Canada	69.1 (60.7 to 79.8)	386 (338 to 447)	182 (137 to 237)	528 (397 to 692)	1.02 (0.92 to 1.07)	13.7 (11.1 to 16.9)	86.3 (69.3 to 107)	26.8 (20.1 to 34.7)	108 (80.3 to 142)	0.74 (0.70 to 0.78)	55.3 (49.1 to 64.0)	299 (265 to 345)	155 (118 to 203)	420 (315 to 555)	1.09 (0.97 to 1.16)
Central African Republic	9.58 (7.58 to 11.6)	1416 (1103 to 1730)	24.2 (18.9 to 30.6)	1726 (1358 to 2163)	0.62 (0.57 to 0.69)	0.62 (0.46 to 0.90)	49.8 (37.7 to 72.4)	1.22 (0.87 to 1.69)	47.8 (34.7 to 65.0)	-0.13 (-0.18 to -0.07)	8.96 (7.03 to 10.9)	1366 (1058 to 1674)	23.0 (18.0 to 29.1)	1678 (1320 to 2102)	0.64 (0.60 to 0.71)
Chad	11.4 (9.20 to 14.1)	718 (580 to 891)	41.6 (33.3 to 51.8)	1299 (1050 to 1605)	1.94 (1.91 to 1.97)	1.20 (0.81 to 1.79)	42.1 (30.2 to 59.2)	3.32 (2.18 to 4.77)	41.0 (29.2 to 57.1)	-0.01 (-0.07 to 0.04)	10.2 (8.25 to 12.7)	676 (542 to 837)	38.3 (30.6 to 48.1)	1258 (1014 to 1557)	2.03 (2.01 to 2.06)
Chile	30.8 (26.5 to 35.6)	550 (473 to 635)	91.4 (69.5 to 119)	660 (499 to 864)	0.51 (0.31 to 0.64)	2.01 (1.75 to 2.35)	31.3 (27.4 to 36.4)	2.57 (2.00 to 3.30)	23.1 (17.9 to 30.2)	-0.90 (-0.96 to -0.84)	28.8 (24.7 to 33.5)	519 (445 to 602)	88.8 (67.5 to 116)	637 (481 to 834)	0.58 (0.38 to 0.71)

Location	DM, Number in 1990 (000s)	DM, ASR in 1990 (per 100,000)	DM, Number in 2021 (000s)	DM, ASR in 2021 (per 100,000)	DM, AAPC (95%CI)	T1D, Number in 1990 (000s)	T1D, ASR in 1990 (per 100,000)	T1D, Number in 2021 (000s)	T1D, ASR in 2021 (per 100,000)	T1D, AAPC (95%CI)	T2D, Number in 1990 (000s)	T2D, ASR in 1990 (per 100,000)	T2D, Number in 2021 (000s)	T2D, ASR in 2021 (per 100,000)	T2D, AAPC (95%CI)
China	2228 (1819 to 2740)	485 (398 to 591)	5537 (4285 to 7081)	538 (414 to 694)	0.37 (0.34 to 0.40)	191 (151 to 242)	34.4 (26.6 to 44.4)	116 (90.9 to 149)	14.7 (11.6 to 18.7)	-2.68 (-2.75 to -2.62)	2037 (1636 to 2519)	450 (365 to 553)	5421 (4186 to 6950)	524 (401 to 677)	0.52 (0.49 to 0.55)
Colombia	91.6 (78.0 to 109)	927 (796 to 1100)	245 (193 to 315)	821 (645 to 1054)	-0.38 (-0.46 to -0.31)	3.21 (2.83 to 3.71)	20.7 (18.2 to 24.3)	4.50 (3.71 to 5.49)	16.8 (13.9 to 20.4)	-0.70 (-0.82 to -0.60)	88.4 (75.0 to 106)	906 (777 to 1076)	241 (189 to 309)	804 (630 to 1034)	-0.37 (-0.45 to -0.30)
Comoros	1.24 (0.90 to 1.65)	1101 (810 to 1453)	3.29 (2.51 to 4.22)	1192 (907 to 1532)	0.23 (0.16 to 0.33)	0.14 (0.09 to 0.18)	56.1 (38.7 to 75.2)	0.18 (0.13 to 0.24)	48.1 (34.4 to 65.0)	-0.57 (-0.78 to -0.35)	1.10 (0.79 to 1.48)	1045 (766 to 1387)	3.11 (2.36 to 4.02)	1144 (868 to 1472)	0.26 (0.20 to 0.36)
Congo	9.01 (7.27 to 11.2)	1439 (1160 to 1775)	26.2 (20.9 to 32.9)	1684 (1341 to 2084)	0.50 (0.45 to 0.58)	0.47 (0.34 to 0.69)	45.3 (33.1 to 70.3)	0.99 (0.69 to 1.38)	39.2 (27.5 to 54.3)	-0.43 (-0.52 to -0.30)	8.55 (6.88 to 10.7)	1393 (1115 to 1719)	25.2 (20.1 to 31.7)	1645 (1312 to 2043)	0.53 (0.47 to 0.60)
Cook Islands	0.26 (0.22 to 0.30)	4151 (3524 to 4866)	0.48 (0.39 to 0.59)	3742 (3035 to 4612)	-0.34 (-0.36 to -0.31)	0.006 (0.005 to 0.009)	70.3 (51.9 to 94.1)	0.004 (0.003 to 0.005)	46.5 (35.1 to 63.3)	-1.21 (-1.35 to -1.04)	0.25 (0.21 to 0.29)	4081 (3466 to 4785)	0.47 (0.39 to 0.58)	3695 (2990 to 4559)	-0.33 (-0.35 to -0.30)
Costa Rica	7.62 (6.35 to 9.25)	798 (665 to 961)	30.9 (24.3 to 40.1)	1047 (824 to 1358)	0.90 (0.75 to 1.05)	0.23 (0.20 to 0.27)	17.1 (14.8 to 20.4)	0.45 (0.38 to 0.54)	16.5 (13.9 to 19.6)	-0.10 (-0.23 to 0.05)	7.39 (6.15 to 8.99)	781 (650 to 941)	30.5 (23.9 to 39.6)	1031 (809 to 1340)	0.92 (0.77 to 1.07)
Croatia	19.5 (16.2 to 23.6)	550 (456 to 660)	33.1 (26.5 to 41.6)	675 (530 to 862)	0.58 (0.48 to 0.64)	1.64 (1.46 to 1.84)	52.6 (47.0 to 59.2)	1.20 (0.90 to 1.55)	37.7 (27.7 to 48.2)	-1.14 (-1.25 to -1.05)	17.9 (14.7 to 21.8)	497 (408 to 602)	31.9 (25.5 to 40.1)	637 (500 to 813)	0.71 (0.61 to 0.78)
Cuba	53.9 (46.9 to 62.3)	1030 (897 to 1190)	80.1 (62.0 to 104)	842 (643 to 1098)	-0.71 (-0.83 to -0.60)	3.03 (2.72 to 3.44)	54.0 (48.5 to 61.4)	1.77 (1.46 to 2.17)	25.0 (20.7 to 30.5)	-2.39 (-2.49 to -2.28)	50.9 (44.1 to 59.2)	976 (846 to 1135)	78.3 (60.5 to 102)	817 (621 to 1069)	-0.63 (-0.76 to -0.51)
Cyprus	5.84 (5.03 to 6.72)	1462 (1274 to 1683)	8.00 (6.54 to 9.89)	773 (635 to 957)	-2.08 (-2.17 to -1.97)	0.28 (0.22 to 0.36)	70.3 (55.3 to 87.5)	0.51 (0.37 to 0.67)	58.5 (42.7 to 77.1)	-0.57 (-0.66 to -0.47)	5.55 (4.79 to 6.42)	1391 (1214 to 1601)	7.49 (6.11 to 9.20)	715 (588 to 878)	-2.18 (-2.27 to -2.09)
Czechia	44.7 (37.6 to 53.6)	560 (469 to 674)	85.4 (68.6 to 106)	734 (580 to 925)	0.92 (0.81 to 1.01)	4.17 (3.80 to 4.68)	60.7 (55.0 to 68.4)	2.77 (2.18 to 3.55)	36.0 (28.1 to 46.7)	-1.74 (-1.84 to -1.68)	40.6 (33.7 to 49.1)	499 (412 to 608)	82.6 (66.3 to 103)	698 (549 to 884)	1.15 (1.02 to 1.25)
Côte d'Ivoire	16.5 (13.3 to 20.1)	758 (613 to 918)	77.2 (61.2 to 96.5)	1272 (1030 to 1593)	1.67 (1.64 to 1.70)	2.06 (1.44 to 2.84)	39.1 (28.1 to 52.5)	4.06 (2.84 to 5.42)	33.4 (24.3 to 43.7)	-0.55 (-0.59 to -0.50)	14.5 (11.6 to 17.8)	719 (581 to 871)	73.2 (58.0 to 92.1)	1239 (1005 to 1554)	1.75 (1.73 to 1.78)
Democratic People's Republic of Korea	56.4 (42.4 to 69.3)	556 (422 to 681)	142 (112 to 180)	771 (610 to 976)	1.07 (1.06 to 1.07)	4.93 (3.29 to 7.52)	45.3 (30.3 to 69.2)	5.75 (3.71 to 9.03)	39.4 (26.1 to 61.1)	-0.45 (-0.47 to -0.43)	51.5 (39.0 to 62.9)	511 (390 to 622)	137 (108 to 173)	732 (580 to 924)	1.17 (1.16 to 1.18)
Democratic Republic of the Congo	99.0 (73.4 to 125)	1084 (805 to 1375)	291 (222 to 376)	1328 (998 to 1717)	0.66 (0.61 to 0.70)	6.59 (4.92 to 8.71)	38.6 (28.2 to 52.4)	13.5 (9.87 to 17.8)	34.7 (25.3 to 46.8)	-0.38 (-0.44 to -0.34)	92.4 (68.4 to 118)	1046 (774 to 1326)	277 (210 to 358)	1293 (973 to 1672)	0.69 (0.64 to 0.73)
Denmark	11.8 (10.4 to 13.4)	280 (245 to 321)	19.2 (15.8 to 24.0)	343 (272 to 435)	0.67 (0.53 to 0.82)	1.65 (1.43 to 1.93)	49.7 (42.7 to 58.8)	1.47 (1.06 to 1.98)	37.2 (26.0 to 50.8)	-0.88 (-0.95 to -0.81)	10.1 (9.01 to 11.5)	231 (202 to 264)	17.8 (14.6 to 22.0)	305 (242 to 385)	0.91 (0.76 to 1.06)
Djibouti	0.65 (0.50 to 0.83)	853 (656 to 1094)	3.43 (2.52 to 4.59)	1085 (814 to 1456)	0.78 (0.75 to 0.81)	0.09 (0.06 to 0.12)	43.9 (32.5 to 59.3)	0.22 (0.16 to 0.31)	38.5 (27.6 to 52.9)	-0.22 (-0.35 to -0.07)	0.56 (0.43 to 0.73)	809 (618 to 1043)	3.21 (2.35 to 4.32)	1047 (784 to 1407)	0.83 (0.80 to 0.86)
Dominica	0.79 (0.70 to 0.91)	2251 (1992 to 2586)	1.12 (0.95 to 1.34)	2610 (2193 to 3124)	0.46 (0.44 to 0.49)	0.03 (0.03 to 0.04)	100 (83.5 to 121)	0.03 (0.02 to 0.04)	86.9 (68.3 to 108)	-0.46 (-0.51 to -0.42)	0.76 (0.67 to 0.87)	2151 (1905 to 2473)	1.09 (0.92 to 1.31)	2523 (2122 to 3024)	0.50 (0.47 to 0.53)
Dominican Republic	21.3 (18.1 to 25.7)	1015 (862 to 1221)	79.9 (64.4 to 98.9)	1513 (1221 to 1870)	1.31 (1.25 to 1.35)	2.28 (1.86 to 2.86)	67.6 (54.9 to 85.9)	3.01 (2.31 to 3.93)	54.4 (41.6 to 70.8)	-0.76 (-0.84 to -0.69)	19.1 (16.0 to 23.3)	947 (800 to 1146)	76.9 (61.6 to 95.2)	1458 (1171 to 1804)	1.38 (1.34 to 1.43)
Ecuador	24.6 (21.8 to 27.9)	870 (775 to 985)	106 (83.6 to 130)	1218 (967 to 1495)	1.03 (0.88 to 1.16)	1.01 (0.87 to 1.18)	22.3 (19.2 to 26.0)	2.16 (1.71 to 2.68)	23.7 (18.8 to 29.3)	0.14 (-0.01 to 0.26)	23.6 (20.9 to 26.8)	848 (755 to 958)	103 (81.9 to 127)	1194 (947 to 1467)	1.05 (0.90 to 1.18)
Egypt	134 (119 to 150)	971 (870 to 1078)	595 (494 to 729)	2107 (1796 to 2521)	2.52 (2.42 to 2.62)	12.8 (9.64 to 19.0)	46.7 (34.5 to 67.9)	20.7 (15.6 to 25.8)	43.1 (32.4 to 53.7)	-0.21 (-0.28 to -0.13)	121 (107 to 135)	924 (828 to 1029)	575 (475 to 702)	2063 (1758 to 2471)	2.61 (2.51 to 2.72)
El Salvador	14.5 (12.7 to 16.5)	854 (748 to 967)	57.8 (48.0 to 69.7)	1634 (1358 to 1971)	2.16 (1.99 to 2.36)	1.06 (0.86 to 1.36)	41.3 (33.7 to 52.4)	1.22 (0.86 to 1.58)	34.9 (24.8 to 45.1)	-0.50 (-0.61 to -0.36)	13.5 (11.8 to 15.4)	812 (711 to 924)	56.6 (47.0 to 68.3)	1599 (1327 to 1931)	2.26 (2.11 to 2.44)
Equatorial Guinea	1.37 (1.07 to 1.79)	1193 (942 to 1588)	4.76 (3.48 to 6.25)	1448 (1070 to 1923)	0.68 (0.57 to 0.78)	0.08 (0.06 to 0.13)	43.4 (31.2 to 69.6)	0.17 (0.11 to 0.25)	27.5 (18.1 to 40.4)	-1.44 (-1.50 to -1.37)	1.29 (1.00 to 1.72)	1149 (903 to 1541)	4.59 (3.36 to 6.03)	1421 (1050 to 1884)	0.73 (0.63 to 0.84)
Eritrea	9.02 (7.28 to 11.1)	1242 (999 to 1517)	25.3 (19.9 to 31.9)	1457 (1142 to 1811)	0.49 (0.48 to 0.51)	0.95 (0.69 to 1.35)	58.1 (44.2 to 81.3)	1.91 (1.33 to 2.58)	58.8 (42.3 to 78.7)	0.06 (0.02 to 0.09)	8.07 (6.37 to 10.0)	1184 (945 to 1454)	23.4 (18.4 to 29.2)	1399 (1099 to 1739)	0.52 (0.50 to 0.53)
Estonia	3.48 (2.82 to 4.32)	289 (235 to 359)	7.69 (6.13 to 9.88)	554 (440 to 712)	2.09 (1.96 to 2.20)	0.58 (0.50 to 0.66)	59.9 (52.3 to 67.5)	0.51 (0.41 to 0.64)	63.5 (53.5 to 76.3)	0.17 (-0.11 to 0.37)	2.91 (2.30 to 3.70)	230 (181 to 292)	7.18 (5.72 to 9.24)	491 (388 to 638)	2.46 (2.35 to 2.56)
Eswatini	3.07 (2.45 to 3.72)	1860 (1481 to 2268)	10.1 (7.29 to 13.3)	2948 (2164 to 3832)	1.45 (1.39 to 1.50)	0.17 (0.12 to 0.23)	44.5 (32.8 to 58.0)	0.26 (0.17 to 0.37)	45.8 (29.5 to 64.0)	0.10 (0.07 to 0.13)	2.89 (2.30 to 3.54)	1815 (1439 to 2225)	9.81 (7.10 to 13.0)	2902 (2134 to 3784)	1.48 (1.42 to 1.53)
Ethiopia	187 (155 to 228)	1663 (1377 to 2034)	240 (199 to 285)	959 (794 to 1117)	-1.75 (-1.79 to -1.71)	23.6 (16.5 to 37.9)	90.4 (67.0 to 155)	22.1 (16.8 to 29.0)	41.6 (31.4 to 55.1)	-2.47 (-2.50 to -2.44)	164 (134 to 201)	1573 (1303 to 1921)	218 (180 to 257)	917 (761 to 1072)	-1.72 (-1.76 to -1.68)
Fiji	9.91 (8.26 to 12.0)	4877 (4086 to 5870)	30.5 (24.6 to 37.9)	7194 (5825 to 8906)	1.19 (1.04 to 1.34)	0.27 (0.21 to 0.35)	73.9 (57.4 to 95.5)	0.29 (0.22 to 0.37)	61.8 (47.0 to 80.8)	-0.54 (-0.61 to -0.47)	9.64 (8.06 to 11.7)	4803 (4026 to 5778)	30.2 (24.3 to 37.5)	7132 (5778 to 8829)	1.21 (1.06 to 1.37)
Finland	14.4 (11.6 to 17.8)	360 (286 to 456)	25.1 (18.2 to 33.2)	490 (348 to 663)	1.01 (0.97 to 1.05)	3.02 (2.43 to 3.70)	90.0 (71.5 to 112)	3.57 (2.56 to 4.80)	93.1 (66.4 to 125)	0.11 (0.05 to 0.15)	11.3 (9.16 to 14.0)	270 (215 to 342)	21.6 (15.7 to 28.5)	397 (282 to 541)	1.26 (1.21 to 1.30)
France	116 (102 to 134)	242 (210 to 283)	188 (154 to 232)	270 (212 to 346)	0.45 (0.34 to 0.53)	8.94 (7.53 to 10.8)	25.4 (21.2 to 31.2)	12.7 (9.29 to 17.2)	29.0 (20.9 to 39.9)	0.46 (0.43 to 0.49)	107 (94.8 to 123)	217 (189 to 253)	175 (144 to 216)	241 (190 to 306)	0.45 (0.31 to 0.55)
Gabon	3.97 (3.12 to 4.90)	1255 (982 to 1560)	8.81 (6.65 to 11.1)	1520 (1139 to 1895)	0.62 (0.58 to 0.67)	0.16 (0.12 to 0.21)	37.7 (26.9 to 51.8)	0.25 (0.18 to 0.36)	29.4 (20.4 to 42.0)	-0.76 (-0.84 to -0.68)	3.81 (2.96 to 4.73)	1217 (947 to 1511)	8.56 (6.46 to 10.8)	1491 (1115 to 1859)	0.66 (0.61 to 0.71)
Gambia	1.41 (1.11 to 1.77)	734 (582 to 909)	7.56 (6.00 to 9.56)	1338 (1055 to 1692)	2.04 (1.95 to 2.15)	0.17 (0.11 to 0.23)	38.8 (28.5 to 50.9)	0.39 (0.28 to 0.57)	36.1 (26.6 to 50.8)	-0.28 (-0.46 to -0.10)	1.24 (0.97 to 1.57)	695 (545 to 865)	7.17 (5.64 to 9.09)	1302 (1029 to 1643)	2.13 (2.03 to 2.23)
Georgia	16.8 (14.2 to 20.2)	455 (384 to 546)	26.5 (21.3 to 32.6)	821 (658 to 1028)	1.81 (1.61 to 2.07)	1.97 (1.67 to 2.28)	61.1 (51.9 to 70.4)	1.30 (1.07 to 1.56)	56.7 (47.2 to 68.0)	-0.09 (-0.34 to 0.12)	14.9 (12.4 to 17.9)	394 (328 to 476)	25.2 (20.2 to 31.1)	764 (607 to 964)	2.03 (1.82 to 2.30)
Germany	307 (278 to 342)	393 (351 to 442)	362 (291 to 446)	393 (306 to 498)	0.08 (-0.00 to 0.16)	21.4 (18.7 to 25.0)	40.2 (35.0 to 47.4)	21.0 (15.4 to 28.0)	36.4 (26.4 to 48.6)	-0.28 (-0.33 to -0.24)	286 (259 to 317)	352 (316 to 396)	341 (275 to 419)	357 (278 to 452)	0.14 (0.05 to 0.23)
Ghana	31.7 (25.6 to 38.4)	870 (704 to 1053)	124 (97.7 to 158)	1224 (972 to 1543)	1.08 (1.05 to 1.11)	2.19 (1.64 to 2.81)	32.1 (24.4 to 40.9)	4.19 (3.07 to 5.56)	26.2 (19.1 to 34.5)	-0.65 (-0.68 to -0.62)	29.5 (23.6 to 36.0)	838 (677 to 1016)	120 (94.4 to 153)	1198 (952 to 1511)	1.13 (1.10 to 1.16)
Greece	28.7 (23.2 to 35.6)	365 (293 to 456)	47.5 (36.2 to 61.8)	459 (340 to 611)	0.71 (0.65 to 0.78)	1.18 (0.91 to 1.54)	19.3 (14.8 to 24.7)	2.12 (1.45 to 3.05)	28.6 (18.7 to 40.9)	1.25 (1.22 to 1.27)	27.5 (22.3 to 34.2)	346 (278 to 431)	45.4 (34.6 to 59.0)	431 (319 to 574)	0.68 (0.60 to 0.74)
Greenland	0.06 (0.06 to 0.07)	369 (319 to 424)	0.11 (0.09 to 0.14)	353 (280 to 440)	-0.09 (-0.13 to -0.04)	0.008 (0.006 to 0.01)	33.4 (24.9 to 44.2)	0.009 (0.006 to 0.01)	29.7 (20.5 to 42.0)	-0.38 (-0.41 to -0.36)	0.06 (0.05 to 0.07)	336 (291 to 387)	0.11 (0.08 to 0.13)	323 (258 to 405)	-0.06 (-0.11 to -0.02)
Grenada	1.17 (1.05 to 1.29)	2874 (2557 to 3181)	1.67 (1.42 to 1.96)	2788 (2365 to 3281)	-0.00 (-0.13 to 0.13)	0.05 (0.05 to 0.06)	132 (117 to 148)	0.04 (0.03 to 0.05)	75.0 (63.8 to 87.8)	-1.79 (-1.87 to -1.71)	1.12 (1.00 to 1.24)	2741 (2431 to 3046)	1.63 (1.38 to 1.91)	2713 (2302 to 3200)	0.07 (-0.07 to 0.21)
Guam	0.58 (0.51 to 0.66)	1501 (1322 to 1694)	1.23 (1.00 to 1.54)	1180 (952 to 1480)	-0.78 (-0.85 to -0.72)	0.009 (0.007 to 0.01)	15.0 (11.3 to 19.7)	0.009 (0.006 to 0.01)	10.6 (7.72 to 14.3)	-1.31 (-1.59 to -1.07)	0.57 (0.50 to 0.65)	1486 (1308 to 1676)	1.22 (0.99 to 1.53)	1169 (945 to 1467)	-0.79 (-0.85 to -0.72)
Guatemala	18.0 (15.4 to 20.8)	847 (728 to 984)	161 (138 to 190)	2577 (2213 to 3020)	3.69 (3.41 to 4.02)	2.76 (2.54 to 3.03)	66.9 (61.3 to 73.4)	6.09 (5.21 to 7.15)	77.3 (66.1 to 90.9)	0.70 (0.32 to 0.98)	15.2 (12.8 to 18.0)	780 (666 to 914)	155 (133 to 183)	2500 (2149 to 2932)	3.86 (3.59 to 4.14)
Guinea	15.8 (12.2 to 19.4)	896 (686 to 1097)	42.5 (34.2 to 52.3)	1351 (1086 to 1651)	1.33 (1.31 to 1.35)	1.51 (1.01 to 2.16)	51.5 (36.6 to 70.9)	2.64 (1.79 to 3.61)	42.0 (30.1 to 57.9)	-0.66 (-0.70 to -0.62)	14.3 (10.9 to 17.6)	844 (641 to 1032)	39.8 (31.6 to 49.3)	1309 (1049 to 1605)	1.42 (1.40 to 1.44)
Guinea-Bissau	2.65 (2.11 to 3.28)	1109 (871 to 1382)	8.02 (6.44 to 10.0)	1735 (1389 to 2128)	1.47 (1.45 to 1.49)	0.31 (0.22 to 0.43)	67.2 (49.4 to 95.0)	0.48 (0.34 to 0.69)	51.4 (37.2 to 74.9)	-0.85 (-0.90 to -0.80)	2.34 (1.83 to 2.90)	1042 (819 to 1304)	7.54 (6.02 to 9.42)	1684 (1348 to 2070)	1.58 (1.56 to 1.60)
Guyana	6.71 (5.80 to 7.74)	3083 (2669 to 3544)	12.8 (10.4 to 15.8)	3549 (2886 to 4377)	0.39 (0.20 to 0.57)	0.49 (0.43 to 0.57)	142 (124 to 164)	0.39 (0.31 to 0.50)	101 (78.9 to 127)	-0.97 (-1.10 to -0.82)	6.22 (5.36 to 7.18)	2940 (2543 to 3380)	12.4 (10.1 to 15.3)	3448 (2808 to 4246)	0.44 (0.25 to 0.62)
Haiti	68.0 (56.4 to 83.7)	3639 (3047 to 4425)	162 (126 to 209)	3784 (2959 to 4821)	0.16 (0.14 to 0.19)	8.52 (6.00 to 14.6)	296 (210 to 543)	14.2 (8.87 to 22.1)	227 (140 to 356)	-0.76 (-0.84 to -0.67)	59.5 (49.4 to 71.4)	3343 (2801 to 4016)	147 (116 to 189)	3557 (2801 to 4535)	0.23 (0.21 to 0.26)
Honduras	9.22 (7.53 to 11.6)	768 (630 to 963)	54.8 (43.0 to 69.5)	1490 (1176 to 1880)	2.16 (2.05 to 2.28)	0.52 (0.39 to 0.66)	23.9 (18.5 to 29.8)	1.03 (0.73 to 1.37)	21.0 (15.2 to 28.1)	-0.37 (-0.44 to -0.29)	8.70 (7.11 to 11.0)	744 (609 to 935)	53.8 (42.2 to 68.2)	1469 (1157 to 1858)	2.22 (2.10 to 2.34)
Hungary	48.8 (40.9 to 57.6)	579 (487 to 683)	70.8 (55.6 to 89.5)	660 (512 to 843)	0.44 (0.35 to 0.52)	3.38 (3.03 to 3.75)	50.4 (45.3 to 55.9)	2.09 (1.62 to 2.67)	30.5 (24.0 to 39.3)	-1.59 (-1.68 to -1.51)	45.4 (37.9 to 53.8)	529 (442 to 627)	68.7 (53.9 to 86.9)	630 (488 to 808)	0.58 (0.50 to 0.66)
Iceland	0.34 (0.27 to 0.42)	225 (181 to 283)	0.87 (0.64 to 1.14)	337 (242 to 452)	1.29 (1.26 to 1.32)	0.04 (0.03 to 0.05)	30.3 (23.6 to 38.5)	0.06 (0.04 to 0.08)	27.5 (18.7 to 39.6)	-0.30 (-0.35 to -0.27)	0.29 (0.24 to 0.36)	195 (155 to 244)	0.81 (0.60 to 1.07)	310 (223 to 416)	1.47 (1.44 to 1.51)
India	1780 (1514 to 2046)	721 (614 to 825)	6529 (5524 to 7757)	1033 (877 to 1226)	1.14 (1.03 to 1.24)	202 (162 to 283)	54.8 (43.5 to 79.3)	306 (245 to 386)	43.6 (34.9 to 54.8)	-0.69 (-0.76 to -0.59)	1578 (1339 to 1828)	666 (568 to 767)	6223 (5240 to 7421)	989 (838 to 1177)	1.28 (1.14 to 1.39)
Indonesia	435 (363 to 521)	756 (622 to 905)	1362 (1131 to 1647)	1050 (884 to 1257)	1.08 (1.05 to 1.13)	49.8 (38.9 to 72.0)	59.1 (45.2 to 86.6)	70.9 (55.0 to 96.2)	48.2 (37.5 to 65.0)	-0.63 (-0.65 to -0.60)	385 (317 to 463)	697 (569 to 841)	1291 (1073 to 1558)	1002 (839 to 1200)	1.20 (1.16 to 1.24)
Iran (Islamic Republic of)	75.5 (63.5 to 89.6)	571 (479 to 678)	424 (347 to 523)	1057 (873 to 1289)	1.96 (1.92 to 2.00)	8.61 (6.54 to 11.4)	33.2 (25.9 to 42.3)	12.0 (8.58 to 15.7)	26.8 (19.4 to 35.1)	-0.66 (-0.72 to -0.62)	66.9 (55.8 to 79.6)	538 (450 to 639)	412 (336 to 508)	1030 (850 to 1258)	2.07 (2.02 to 2.11)
Iraq	65.9 (55.2 to 78.9)	1445 (1210 to 1732)	280 (221 to 357)	2088 (1670 to 2645)	1.21 (1.19 to 1.24)	7.50 (5.57 to 10.3)	83.4 (61.2 to 113)	10.1 (7.62 to 13.5)	51.6 (39.2 to 68.8)	-1.56 (-1.59 to -1.53)	58.4 (48.7 to 70.8)	1361 (1137 to 1649)	270 (212 to 347)	2036 (1626 to 2592)	1.33 (1.31 to 1.36)
Ireland	7.23 (6.17 to 8.54)	329 (277 to 392)	12.1 (9.01 to 16.0)	324 (239 to 431)	-0.03 (-0.10 to 0.03)	0.68 (0.55 to 0.82)	34.6 (28.2 to 42.1)	1.44 (1.01 to 1.95)	47.1 (32.6 to 64.8)	1.00 (0.95 to 1.03)	6.55 (5.61 to 7.70)	294 (249 to 352)	10.6 (8.00 to 14.1)	277 (205 to 372)	-0.18 (-0.27 to -0.11)
Israel	16.6 (14.6 to 19.2)	640 (563 to 741)	39.0 (31.9 to 48.0)	594 (477 to 742)	-0.28 (-0.42 to -0.13)	0.97 (0.83 to 1.13)	38.6 (32.9 to 45.2)	1.58 (1.18 to 2.11)	30.3 (22.5 to 40.7)	-0.76 (-0.80 to -0.72)	15.6 (13.8 to 18.0)	602 (529 to 696)	37.4 (30.7 to 46.0)	564 (454 to 702)	-0.25 (-0.40 to -0.10)
Italy	297 (264 to 337)	591 (521 to 680)	336 (272 to 416)	474 (370 to 616)	-0.73 (-0.82 to -0.68)	15.9 (11.7 to 21.5)	45.0 (33.5 to 60.5)	30.7 (20.3 to 45.2)	67.4 (44.7 to 98.7)	1.22 (1.00 to 1.32)	281 (250 to 318)	546 (484 to 627)	305 (248 to 375)	407 (320 to 516)	-0.96 (-1.03 to -0.90)
Jamaica	21.4 (19.7 to 23.3)	2247 (2062 to 2450)	37.6 (31.1 to 45.9)	2317 (1914 to 2837)	0.15 (-0.00 to 0.27)	1.02 (0.91 to 1.16)	91.8 (81.7 to 105)	1.00 (0.79 to 1.26)	66.0 (51.9 to 83.0)	-1.03 (-1.17 to -0.90)	20.4 (18.7 to 22.2)	2155 (1977 to 2351)	36.6 (30.3 to 44.7)	2251 (1860 to 2754)	0.18 (0.03 to 0.30)
Japan	320 (257 to 400)	346 (278 to 433)	633 (455 to 861)	416 (296 to 572)	0.59 (0.53 to 0.63)	14.5 (11.6 to 18.6)	20.1 (16.0 to 25.6)	14.6 (9.96 to 20.5)	17.1 (11.5 to 24.5)	-0.60 (-0.65 to -0.55)	305 (245 to 383)	326 (261 to 409)	619 (443 to 840)	399 (282 to 549)	0.65 (0.59 to 0.69)
Jordan	14.1 (12.1 to 16.6)	2035 (1756 to 2385)	65.6 (51.3 to 83.6)	1764 (1403 to 2206)	-0.56 (-0.70 to -0.43)	1.25 (0.96 to 1.76)	67.0 (53.3 to 86.3)	1.68 (1.28 to 2.19)	30.7 (23.3 to 40.0)	-2.46 (-2.50 to -2.42)	12.9 (11.0 to 15.2)	1968 (1697 to 2308)	63.9 (49.9 to 81.3)	1733 (1379 to 2168)	-0.51 (-0.65 to -0.38)
Kazakhstan	37.2 (30.5 to 45.5)	471 (385 to 575)	83.2 (63.7 to 107)	777 (596 to 998)	1.69 (1.60 to 1.80)	4.48 (3.94 to 5.06)	54.6 (48.0 to 61.9)	4.39 (3.61 to 5.29)	43.1 (35.6 to 51.7)	-0.80 (-1.03 to -0.64)	32.7 (26.3 to 40.6)	416 (334 to 516)	78.9 (59.7 to 102)	734 (557 to 948)	1.93 (1.83 to 2.04)
Kenya	30.3 (25.8 to 35.4)	654 (554 to 769)	103 (81.9 to 127)	789 (626 to 971)	0.60 (0.57 to 0.62)	2.93 (2.19 to 3.90)	28.1 (21.4 to 36.6)	6.24 (4.64 to 8.57)	27.3 (20.1 to 37.3)	-0.08 (-0.10 to -0.06)	27.4 (23.2 to 32.0)	626 (530 to 736)	96.5 (77.3 to 119)	761 (603 to 935)	0.62 (0.60 to 0.64)
Kiribati	0.89 (0.75 to 1.04)	4009 (3336 to 4776)	2.58 (2.07 to 3.18)	5809 (4670 to 7055)	1.21 (1.19 to 1.23)	0.03 (0.02 to 0.03)	61.6 (41.2 to 80.8)	0.04 (0.03 to 0.06)	64.0 (39.0 to 89.3)	0.11 (0.08 to 0.14)	0.86 (0.72 to 1.02)	3948 (3274 to 4717)	2.54 (2.03 to 3.12)	5745 (4608 to 6959)	1.23 (1.21 to 1.25)
Kuwait	3.09 (2.52 to 3.80)	1110 (923 to 1344)	24.1 (17.7 to 32.6)	1494 (1129 to 1963)	1.01 (0.85 to 1.21)	0.30 (0.27 to 0.35)	48.0 (41.4 to 55.6)	0.62 (0.47 to 0.82)	27.4 (21.4 to 35.2)	-1.73 (-2.14 to -1.45)	2.78 (2.25 to 3.45)	1062 (883 to 1290)	23.5 (17.1 to 31.9)	1466 (1109 to 1930)	1.09 (0.93 to 1.28)
Kyrgyzstan	5.69 (4.71 to 6.79)	311 (256 to 372)	16.6 (13.1 to 21.0)	560 (441 to 703)	1.88 (1.75 to 1.98)	0.68 (0.60 to 0.78)	32.7 (28.7 to 37.6)	1.09 (0.89 to 1.34)	32.6 (26.7 to 39.9)	-0.11 (-0.32 to 0.16)	5.01 (4.10 to 6.07)	278 (227 to 337)	15.6 (12.2 to 19.8)	528 (412 to 669)	2.06 (1.93 to 2.17)
Lao People's Democratic Republic	15.9 (12.7 to 20.5)	1325 (1055 to 1691)	41.0 (32.8 to 51.1)	1611 (1295 to 2005)	0.61 (0.59 to 0.63)	1.72 (1.22 to 3.19)	96.9 (66.1 to 185)	2.35 (1.71 to 3.16)	66.3 (47.9 to 90.3)	-1.20 (-1.23 to -1.16)	14.2 (11.3 to 18.0)	1228 (985 to 1548)	38.7 (30.9 to 48.4)	1544 (1245 to 1934)	0.72 (0.71 to 0.74)
Latvia	6.92 (5.79 to 8.29)	332 (278 to 397)	13.6 (11.2 to 16.9)	642 (525 to 796)	2.17 (1.97 to 2.33)	0.75 (0.68 to 0.84)	51.2 (46.6 to 56.3)	0.48 (0.39 to 0.60)	46.0 (38.9 to 55.3)	-0.39 (-0.75 to -0.16)	6.17 (5.07 to 7.48)	281 (231 to 343)	13.1 (10.8 to 16.3)	596 (486 to 746)	2.49 (2.26 to 2.69)
Lebanon	13.3 (11.1 to 16.3)	1142 (957 to 1394)	40.3 (31.7 to 51.1)	1258 (987 to 1598)	0.31 (0.26 to 0.34)	0.97 (0.68 to 1.34)	67.3 (46.9 to 94.0)	1.09 (0.81 to 1.43)	35.9 (27.2 to 46.5)	-1.98 (-2.04 to -1.91)	12.3 (10.2 to 15.1)	1075 (895 to 1306)	39.2 (30.8 to 49.8)	1222 (956 to 1557)	0.42 (0.37 to 0.45)
Lesotho	5.64 (4.47 to 7.26)	1032 (816 to 1333)	17.2 (12.8 to 22.5)	2545 (1893 to 3288)	2.96 (2.89 to 3.02)	0.29 (0.20 to 0.41)	35.2 (24.7 to 48.5)	0.44 (0.28 to 0.60)	45.9 (29.6 to 63.2)	0.88 (0.83 to 0.92)	5.35 (4.23 to 6.94)	997 (788 to 1294)	16.8 (12.4 to 21.9)	2499 (1852 to 3224)	3.01 (2.94 to 3.08)
Liberia	5.16 (4.31 to 6.24)	886 (744 to 1060)	17.2 (13.5 to 21.8)	1426 (1133 to 1837)	1.55 (1.51 to 1.59)	0.53 (0.36 to 0.72)	45.8 (33.7 to 59.9)	0.88 (0.59 to 1.25)	36.1 (25.6 to 51.0)	-0.89 (-0.96 to -0.81)	4.63 (3.82 to 5.61)	840 (701 to 1010)	16.3 (12.8 to 20.8)	1390 (1099 to 1790)	1.64 (1.60 to 1.68)
Libya	6.11 (4.88 to 7.45)	627 (503 to 761)	39.6 (31.5 to 50.7)	1373 (1101 to 1746)	2.59 (2.53 to 2.64)	0.63 (0.45 to 0.97)	34.8 (25.6 to 49.8)	1.51 (1.08 to 2.01)	42.5 (30.6 to 56.1)	0.72 (0.63 to 0.86)	5.48 (4.36 to 6.70)	592 (471 to 722)	38.1 (30.0 to 48.8)	1331 (1060 to 1690)	2.67 (2.60 to 2.72)
Lithuania	6.38 (5.12 to 8.00)	244 (198 to 305)	14.2 (11.5 to 18.2)	469 (375 to 599)	2.06 (1.97 to 2.14)	0.89 (0.79 to 1.02)	41.0 (36.6 to 46.7)	0.73 (0.60 to 0.91)	42.7 (36.0 to 51.8)	0.02 (-0.38 to 0.27)	5.49 (4.31 to 7.01)	203 (159 to 259)	13.5 (10.9 to 17.3)	426 (338 to 552)	2.38 (2.20 to 2.54)
Luxembourg	1.10 (0.96 to 1.30)	361 (311 to 430)	1.97 (1.49 to 2.56)	385 (285 to 509)	0.19 (0.12 to 0.26)	0.09 (0.08 to 0.11)	38.7 (32.4 to 46.9)	0.14 (0.09 to 0.19)	32.7 (22.7 to 46.0)	-0.56 (-0.60 to -0.53)	1.01 (0.88 to 1.19)	322 (277 to 382)	1.83 (1.38 to 2.38)	352 (262 to 465)	0.28 (0.20 to 0.35)
Madagascar	26.7 (22.8 to 30.9)	934 (795 to 1085)	70.4 (55.3 to 88.6)	1027 (798 to 1301)	0.31 (0.26 to 0.36)	3.11 (2.26 to 4.11)	51.4 (39.6 to 67.5)	6.18 (4.74 to 8.08)	44.0 (33.3 to 57.4)	-0.51 (-0.55 to -0.48)	23.6 (19.8 to 27.8)	882 (745 to 1033)	64.2 (50.1 to 81.4)	983 (763 to 1248)	0.35 (0.31 to 0.40)
Malawi	22.1 (18.0 to 26.6)	952 (780 to 1147)	42.3 (33.6 to 52.8)	934 (751 to 1159)	-0.06 (-0.09 to -0.02)	3.12 (2.15 to 4.28)	55.6 (41.5 to 74.7)	3.93 (2.78 to 5.22)	40.8 (29.8 to 53.6)	-0.97 (-1.01 to -0.91)	19.0 (15.6 to 22.9)	896 (734 to 1079)	38.3 (30.4 to 48.3)	893 (717 to 1106)	-0.01 (-0.04 to 0.03)
Malaysia	54.5 (46.7 to 63.7)	1088 (942 to 1273)	159 (128 to 194)	1092 (884 to 1331)	-0.00 (-0.11 to 0.09)	4.00 (3.29 to 4.95)	57.4 (46.5 to 71.7)	6.23 (4.78 to 7.89)	38.9 (29.7 to 49.5)	-1.15 (-1.23 to -1.07)	50.5 (43.3 to 59.1)	1031 (892 to 1211)	152 (123 to 187)	1053 (850 to 1282)	0.05 (-0.05 to 0.14)
Maldives	0.58 (0.49 to 0.70)	1334 (1148 to 1577)	1.32 (1.07 to 1.67)	851 (694 to 1061)	-1.49 (-1.60 to -1.37)	0.07 (0.05 to 0.13)	92.9 (61.9 to 183)	0.06 (0.05 to 0.08)	30.3 (23.5 to 39.1)	-3.55 (-3.60 to -3.49)	0.51 (0.43 to 0.61)	1241 (1062 to 1453)	1.26 (1.02 to 1.60)	821 (668 to 1023)	-1.43 (-1.57 to -1.28)
Mali	26.2 (21.8 to 31.5)	1189 (990 to 1416)	89.4 (71.4 to 112)	1793 (1445 to 2219)	1.33 (1.31 to 1.35)	2.25 (1.56 to 3.14)	56.6 (41.4 to 78.4)	4.34 (3.03 to 6.17)	41.1 (30.4 to 56.5)	-1.03 (-1.08 to -0.98)	24.0 (20.0 to 28.6)	1132 (939 to 1349)	85.1 (67.9 to 107)	1752 (1412 to 2173)	1.42 (1.40 to 1.44)
Malta	1.57 (1.41 to 1.78)	669 (597 to 759)	2.95 (2.39 to 3.72)	641 (504 to 826)	-0.20 (-0.33 to -0.08)	0.13 (0.11 to 0.16)	63.1 (51.8 to 77.2)	0.20 (0.14 to 0.26)	65.8 (46.8 to 87.1)	0.10 (0.04 to 0.15)	1.44 (1.29 to 1.62)	606 (542 to 683)	2.75 (2.23 to 3.47)	575 (455 to 740)	-0.24 (-0.38 to -0.11)
Marshall Islands	0.35 (0.28 to 0.42)	3777 (3082 to 4663)	1.51 (1.09 to 1.99)	7529 (5419 to 9820)	2.25 (2.24 to 2.27)	0.01 (0.009 to 0.02)	54.8 (41.3 to 76.0)	0.02 (0.01 to 0.03)	66.3 (40.7 to 101)	0.62 (0.60 to 0.63)	0.33 (0.27 to 0.41)	3722 (3034 to 4590)	1.50 (1.07 to 1.96)	7462 (5367 to 9722)	2.27 (2.26 to 2.28)
Mauritania	4.74 (3.85 to 5.82)	855 (689 to 1057)	13.0 (9.97 to 16.2)	1127 (869 to 1434)	0.87 (0.84 to 0.91)	0.38 (0.27 to 0.52)	41.2 (31.3 to 57.0)	0.54 (0.39 to 0.81)	28.0 (20.3 to 41.8)	-1.27 (-1.36 to -1.20)	4.36 (3.52 to 5.37)	814 (654 to 1005)	12.4 (9.58 to 15.6)	1099 (848 to 1395)	0.95 (0.91 to 0.98)
Mauritius	7.10 (6.38 to 7.93)	1698 (1528 to 1897)	30.0 (27.0 to 34.3)	3039 (2731 to 3476)	1.80 (1.64 to 2.01)	0.70 (0.61 to 0.80)	135 (117 to 156)	0.90 (0.80 to 1.01)	117 (104 to 132)	-0.45 (-0.61 to -0.28)	6.41 (5.71 to 7.18)	1563 (1396 to 1750)	29.1 (26.1 to 33.3)	2922 (2618 to 3350)	1.94 (1.76 to 2.16)
Mexico	590 (529 to 661)	2518 (2280 to 2791)	1617 (1374 to 1925)	2334 (1989 to 2769)	-0.23 (-0.30 to -0.16)	24.1 (21.8 to 27.3)	64.7 (58.4 to 73.1)	36.1 (30.5 to 42.6)	52.1 (44.2 to 61.3)	-0.64 (-0.79 to -0.50)	566 (507 to 635)	2453 (2221 to 2721)	1581 (1343 to 1880)	2282 (1943 to 2705)	-0.22 (-0.29 to -0.15)
Micronesia (Federated States of)	0.69 (0.56 to 0.86)	2622 (2114 to 3267)	1.75 (1.41 to 2.19)	4175 (3417 to 5190)	1.53 (1.50 to 1.56)	0.03 (0.02 to 0.04)	52.0 (35.9 to 76.3)	0.02 (0.02 to 0.03)	42.3 (30.5 to 58.9)	-0.66 (-0.68 to -0.65)	0.66 (0.53 to 0.83)	2570 (2068 to 3203)	1.72 (1.39 to 2.15)	4133 (3381 to 5137)	1.58 (1.55 to 1.61)
Monaco	0.06 (0.05 to 0.08)	181 (138 to 235)	0.13 (0.09 to 0.17)	320 (228 to 429)	1.86 (1.85 to 1.87)	0.006 (0.004 to 0.008)	24.4 (16.8 to 34.4)	0.010 (0.006 to 0.01)	34.3 (22.7 to 50.4)	1.10 (1.09 to 1.12)	0.06 (0.04 to 0.07)	157 (120 to 202)	0.12 (0.09 to 0.16)	286 (203 to 384)	1.96 (1.95 to 1.97)
Mongolia	1.99 (1.63 to 2.36)	306 (250 to 368)	7.28 (5.73 to 9.34)	494 (388 to 629)	1.56 (1.44 to 1.66)	0.47 (0.35 to 0.59)	55.0 (38.3 to 70.4)	0.59 (0.46 to 0.73)	35.8 (27.6 to 44.6)	-1.36 (-1.46 to -1.26)	1.51 (1.19 to 1.89)	251 (196 to 313)	6.70 (5.21 to 8.74)	458 (357 to 593)	1.95 (1.84 to 2.04)
Montenegro	2.06 (1.66 to 2.56)	592 (478 to 733)	4.58 (3.62 to 5.73)	865 (688 to 1091)	1.24 (1.21 to 1.28)	0.25 (0.20 to 0.31)	75.6 (59.6 to 92.7)	0.31 (0.24 to 0.39)	72.6 (57.3 to 91.2)	-0.09 (-0.20 to 0.04)	1.80 (1.44 to 2.28)	517 (411 to 652)	4.26 (3.35 to 5.39)	793 (626 to 1008)	1.40 (1.36 to 1.45)
Morocco	57.1 (45.9 to 72.2)	725 (587 to 914)	317 (246 to 416)	1742 (1357 to 2267)	2.87 (2.85 to 2.89)	6.84 (4.71 to 11.4)	52.0 (36.9 to 81.9)	8.45 (6.45 to 11.6)	45.2 (34.8 to 62.9)	-0.46 (-0.48 to -0.43)	50.2 (39.9 to 64.0)	673 (538 to 853)	309 (239 to 406)	1697 (1322 to 2214)	3.03 (3.01 to 3.05)
Mozambique	30.0 (24.6 to 36.5)	845 (710 to 1019)	74.0 (56.0 to 95.5)	1041 (785 to 1330)	0.68 (0.65 to 0.71)	4.35 (2.83 to 6.04)	56.5 (40.1 to 74.7)	7.91 (5.46 to 10.7)	48.7 (35.0 to 64.5)	-0.45 (-0.52 to -0.40)	25.6 (21.3 to 31.2)	788 (660 to 950)	66.1 (49.6 to 86.3)	993 (740 to 1274)	0.75 (0.71 to 0.78)
Myanmar	265 (209 to 341)	1951 (1538 to 2497)	569 (462 to 697)	2020 (1648 to 2470)	0.11 (0.09 to 0.13)	28.5 (18.7 to 53.7)	157 (103 to 300)	25.9 (19.1 to 36.4)	85.6 (63.4 to 120)	-1.92 (-1.96 to -1.88)	237 (185 to 296)	1795 (1416 to 2245)	544 (441 to 667)	1934 (1573 to 2378)	0.24 (0.22 to 0.26)
Namibia	5.70 (4.84 to 6.75)	1560 (1315 to 1858)	14.3 (10.9 to 18.4)	1786 (1377 to 2274)	0.45 (0.41 to 0.48)	0.25 (0.17 to 0.34)	37.4 (26.7 to 48.9)	0.37 (0.25 to 0.52)	30.8 (21.2 to 42.1)	-0.62 (-0.71 to -0.55)	5.45 (4.61 to 6.49)	1522 (1284 to 1817)	13.9 (10.6 to 18.0)	1755 (1354 to 2236)	0.47 (0.43 to 0.50)
Nauru	0.08 (0.07 to 0.10)	3487 (2878 to 4327)	0.18 (0.14 to 0.22)	4971 (4034 to 6188)	1.15 (1.12 to 1.18)	0.003 (0.002 to 0.004)	52.9 (38.2 to 78.3)	0.003 (0.002 to 0.004)	46.4 (30.7 to 69.7)	-0.41 (-0.43 to -0.39)	0.08 (0.06 to 0.10)	3435 (2833 to 4263)	0.17 (0.14 to 0.22)	4925 (4002 to 6131)	1.17 (1.14 to 1.20)
Nepal	38.5 (31.6 to 46.8)	747 (616 to 903)	151 (119 to 191)	1161 (925 to 1456)	1.42 (1.39 to 1.44)	4.34 (3.03 to 6.59)	54.3 (38.6 to 85.7)	7.13 (5.08 to 9.51)	44.8 (31.8 to 60.2)	-0.62 (-0.67 to -0.57)	34.1 (28.0 to 41.4)	693 (569 to 829)	144 (114 to 182)	1116 (888 to 1398)	1.54 (1.51 to 1.56)
Netherlands	56.3 (50.0 to 63.2)	485 (431 to 549)	58.9 (46.6 to 74.9)	354 (271 to 457)	-1.02 (-1.10 to -0.96)	5.39 (4.67 to 6.34)	57.4 (49.4 to 68.0)	5.39 (3.97 to 7.10)	45.0 (32.9 to 60.6)	-0.81 (-0.88 to -0.75)	50.9 (45.2 to 57.2)	428 (380 to 484)	53.5 (42.5 to 68.2)	309 (237 to 399)	-1.06 (-1.16 to -0.98)
New Zealand	9.07 (7.70 to 10.9)	444 (376 to 534)	17.9 (14.0 to 22.7)	443 (346 to 564)	0.12 (0.01 to 0.22)	0.91 (0.75 to 1.11)	47.4 (38.9 to 58.5)	0.88 (0.63 to 1.23)	27.2 (19.4 to 38.1)	-1.74 (-1.79 to -1.69)	8.16 (6.91 to 9.74)	397 (336 to 475)	17.0 (13.4 to 21.5)	416 (325 to 527)	0.30 (0.17 to 0.43)

Location	DM, Number in 1990 (000s)	DM, ASR in 1990 (per 100,000)	DM, Number in 2021 (000s)	DM, ASR in 2021 (per 100,000)	DM, AAPC (95%CI)	T1D, Number in 1990 (000s)	T1D, ASR in 1990 (per 100,000)	T1D, Number in 2021 (000s)	T1D, ASR in 2021 (per 100,000)	T1D, AAPC (95%CI)	T2D, Number in 1990 (000s)	T2D, ASR in 1990 (per 100,000)	T2D, Number in 2021 (000s)	T2D, ASR in 2021 (per 100,000)	T2D, AAPC (95%CI)
Nicaragua	9.84 (8.38 to 11.6)	1057 (903 to 1237)	42.4 (34.5 to 52.2)	1500 (1230 to 1844)	1.10 (1.01 to 1.20)	0.72 (0.56 to 0.90)	38.7 (32.1 to 49.2)	0.92 (0.72 to 1.13)	27.6 (21.6 to 34.2)	-1.14 (-1.25 to -1.04)	9.11 (7.78 to 10.8)	1018 (870 to 1194)	41.4 (33.7 to 51.1)	1472 (1206 to 1814)	1.16 (1.06 to 1.25)
Niger	10.7 (8.33 to 13.5)	653 (512 to 821)	50.8 (38.6 to 64.8)	1025 (796 to 1306)	1.47 (1.45 to 1.49)	1.83 (1.05 to 2.78)	45.0 (29.8 to 64.6)	4.10 (2.37 to 6.32)	34.4 (22.7 to 48.5)	-0.92 (-0.98 to -0.87)	8.88 (7.00 to 11.2)	608 (478 to 764)	46.7 (35.7 to 59.4)	990 (768 to 1266)	1.60 (1.58 to 1.62)
Nigeria	192 (153 to 238)	852 (685 to 1059)	520 (399 to 676)	970 (768 to 1243)	0.41 (0.39 to 0.43)	16.3 (10.9 to 22.1)	41.6 (28.8 to 55.9)	32.9 (21.3 to 44.5)	30.0 (20.5 to 40.7)	-1.07 (-1.11 to -1.04)	175 (140 to 219)	810 (648 to 1011)	487 (372 to 634)	940 (739 to 1204)	0.47 (0.45 to 0.48)
Niue	0.03 (0.03 to 0.04)	2474 (2028 to 3042)	0.05 (0.04 to 0.06)	4267 (3422 to 5197)	1.76 (1.72 to 1.80)	4e-04 (3e-04 to 6e-04)	36.4 (26.8 to 48.8)	5e-04 (4e-04 to 7e-04)	73.5 (52.7 to 96.6)	2.68 (2.12 to 3.50)	0.03 (0.02 to 0.04)	2437 (2000 to 2999)	0.05 (0.04 to 0.06)	4194 (3356 to 5116)	1.77 (1.73 to 1.80)
North Macedonia	8.90 (7.45 to 10.6)	908 (761 to 1078)	22.3 (18.3 to 27.7)	1289 (1057 to 1596)	1.12 (1.05 to 1.17)	1.22 (1.00 to 1.48)	120 (98.2 to 145)	1.13 (0.87 to 1.49)	77.1 (59.6 to 99.9)	-1.40 (-1.48 to -1.34)	7.68 (6.31 to 9.30)	788 (649 to 953)	21.1 (17.1 to 26.4)	1212 (991 to 1506)	1.40 (1.34 to 1.45)
Northern Mariana Islands	0.17 (0.13 to 0.22)	1980 (1600 to 2463)	0.54 (0.45 to 0.64)	2038 (1734 to 2398)	0.10 (0.03 to 0.16)	0.003 (0.002 to 0.004)	15.5 (11.3 to 21.0)	0.003 (0.002 to 0.004)	12.9 (9.31 to 17.0)	-0.74 (-1.10 to -0.33)	0.17 (0.13 to 0.21)	1964 (1587 to 2445)	0.54 (0.44 to 0.63)	2025 (1723 to 2383)	0.10 (0.04 to 0.16)
Norway	11.2 (9.25 to 13.8)	330 (267 to 412)	15.6 (12.1 to 20.2)	349 (262 to 460)	0.18 (0.07 to 0.28)	1.58 (1.26 to 2.02)	62.3 (49.6 to 78.8)	2.14 (1.52 to 2.94)	62.3 (44.2 to 86.2)	-0.04 (-0.10 to 0.02)	9.66 (7.93 to 12.0)	268 (215 to 336)	13.5 (10.5 to 17.5)	286 (217 to 378)	0.22 (0.11 to 0.33)
Oman	4.51 (3.60 to 5.61)	1348 (1075 to 1689)	14.1 (11.3 to 17.4)	1433 (1168 to 1736)	0.14 (0.08 to 0.19)	0.78 (0.59 to 1.02)	125 (95.1 to 161)	1.32 (1.03 to 1.69)	78.9 (61.9 to 101)	-1.43 (-1.51 to -1.35)	3.73 (2.97 to 4.63)	1223 (978 to 1529)	12.8 (10.3 to 15.8)	1354 (1101 to 1641)	0.25 (0.19 to 0.31)
Pakistan	255 (209 to 298)	938 (776 to 1102)	1135 (893 to 1403)	1758 (1390 to 2164)	2.06 (2.04 to 2.08)	29.1 (21.1 to 35.9)	69.8 (51.8 to 86.0)	89.0 (63.6 to 118)	85.5 (62.2 to 113)	0.68 (0.65 to 0.70)	226 (183 to 266)	868 (715 to 1022)	1046 (823 to 1296)	1673 (1320 to 2060)	2.15 (2.13 to 2.17)
Palau	0.13 (0.11 to 0.16)	2543 (2083 to 3032)	0.42 (0.34 to 0.50)	3899 (3205 to 4691)	1.40 (1.37 to 1.43)	0.002 (0.002 to 0.003)	32.2 (23.9 to 42.7)	0.003 (0.002 to 0.003)	32.7 (24.6 to 42.9)	0.07 (0.05 to 0.08)	0.13 (0.11 to 0.16)	2511 (2052 to 2994)	0.41 (0.34 to 0.50)	3866 (3176 to 4652)	1.41 (1.38 to 1.44)
Palestine	6.48 (5.26 to 8.16)	1321 (1069 to 1657)	22.9 (19.0 to 27.7)	1708 (1429 to 2041)	0.75 (0.68 to 0.82)	0.50 (0.35 to 0.68)	45.6 (33.6 to 61.6)	0.80 (0.60 to 1.06)	34.3 (25.7 to 45.2)	-0.90 (-0.95 to -0.85)	5.98 (4.81 to 7.58)	1275 (1028 to 1603)	22.1 (18.3 to 26.8)	1674 (1401 to 2000)	0.79 (0.72 to 0.86)
Panama	7.15 (6.22 to 8.43)	910 (793 to 1070)	28.7 (23.3 to 35.7)	1263 (1025 to 1574)	1.14 (0.92 to 1.37)	0.35 (0.30 to 0.40)	31.0 (27.1 to 35.8)	0.65 (0.53 to 0.80)	30.1 (24.5 to 36.8)	-0.02 (-0.11 to 0.10)	6.80 (5.89 to 8.03)	879 (764 to 1035)	28.0 (22.8 to 34.9)	1233 (1001 to 1541)	1.17 (0.95 to 1.41)
Papua New Guinea	19.9 (15.2 to 25.5)	1910 (1479 to 2450)	76.8 (61.9 to 95.9)	2589 (2122 to 3199)	0.99 (0.94 to 1.04)	1.00 (0.62 to 1.48)	46.4 (29.0 to 69.0)	2.65 (1.84 to 3.68)	48.9 (34.3 to 67.8)	0.30 (0.20 to 0.42)	18.9 (14.3 to 24.2)	1864 (1443 to 2390)	74.1 (59.7 to 92.8)	2540 (2078 to 3147)	1.00 (0.96 to 1.05)
Paraguay	12.2 (10.4 to 14.7)	992 (835 to 1198)	56.7 (46.0 to 68.9)	1832 (1486 to 2225)	2.05 (1.95 to 2.15)	0.84 (0.64 to 1.08)	41.8 (32.8 to 52.5)	1.40 (1.04 to 1.80)	40.1 (29.8 to 51.3)	-0.01 (-0.09 to 0.07)	11.4 (9.58 to 13.8)	951 (797 to 1153)	55.3 (44.9 to 67.3)	1791 (1454 to 2176)	2.11 (2.01 to 2.22)
Peru	32.1 (27.3 to 37.6)	494 (418 to 579)	121 (96.9 to 152)	685 (550 to 862)	1.10 (0.95 to 1.27)	1.89 (1.51 to 2.37)	18.7 (15.0 to 23.0)	2.85 (2.02 to 3.75)	15.4 (10.9 to 20.2)	-0.61 (-0.80 to -0.44)	30.2 (25.7 to 35.7)	475 (402 to 559)	118 (94.7 to 148)	670 (537 to 843)	1.15 (1.00 to 1.33)
Philippines	155 (136 to 177)	950 (836 to 1071)	587 (485 to 718)	1290 (1069 to 1575)	1.00 (0.92 to 1.08)	15.9 (13.0 to 19.3)	57.2 (47.0 to 69.0)	28.2 (22.4 to 36.1)	51.4 (40.8 to 65.6)	-0.32 (-0.36 to -0.28)	140 (122 to 158)	893 (787 to 1009)	559 (461 to 685)	1239 (1025 to 1514)	1.07 (0.98 to 1.15)
Poland	167 (142 to 197)	654 (558 to 773)	254 (201 to 316)	642 (503 to 807)	-0.15 (-0.21 to -0.10)	15.2 (14.1 to 16.7)	65.5 (60.3 to 72.7)	7.68 (5.89 to 9.96)	28.4 (21.9 to 37.6)	-2.64 (-2.72 to -2.57)	151 (128 to 181)	589 (497 to 703)	247 (195 to 306)	613 (478 to 769)	0.06 (-0.01 to 0.12)
Portugal	50.8 (44.5 to 57.7)	655 (574 to 753)	82.4 (65.3 to 105)	673 (516 to 876)	0.00 (-0.07 to 0.08)	2.01 (1.65 to 2.47)	35.3 (29.4 to 42.7)	2.57 (1.72 to 3.65)	32.9 (22.4 to 46.6)	-0.23 (-0.26 to -0.20)	48.8 (42.8 to 55.4)	620 (543 to 709)	79.8 (63.4 to 102)	640 (492 to 838)	0.02 (-0.06 to 0.10)
Puerto Rico	33.4 (29.5 to 38.2)	1724 (1524 to 1973)	58.8 (48.6 to 72.9)	1702 (1374 to 2136)	-0.06 (-0.20 to 0.05)	2.71 (2.45 to 3.08)	141 (127 to 161)	2.22 (1.85 to 2.65)	92.8 (77.6 to 111)	-1.27 (-1.39 to -1.14)	30.7 (27.0 to 35.3)	1583 (1393 to 1823)	56.6 (46.6 to 70.4)	1609 (1295 to 2023)	0.03 (-0.10 to 0.14)
Qatar	0.77 (0.62 to 0.95)	2052 (1667 to 2507)	9.24 (7.04 to 11.9)	2670 (2123 to 3288)	0.83 (0.57 to 1.05)	0.07 (0.05 to 0.10)	52.5 (38.3 to 71.8)	0.29 (0.21 to 0.41)	35.6 (25.9 to 48.1)	-1.19 (-1.29 to -1.08)	0.70 (0.57 to 0.86)	1999 (1632 to 2440)	8.94 (6.82 to 11.5)	2634 (2096 to 3247)	0.87 (0.61 to 1.10)
Republic of Korea	126 (107 to 149)	692 (595 to 814)	407 (299 to 542)	873 (635 to 1172)	0.74 (0.69 to 0.77)	10.2 (7.93 to 12.5)	45.5 (35.4 to 55.8)	7.67 (5.19 to 10.8)	23.8 (16.5 to 33.1)	-2.04 (-2.07 to -2.00)	116 (98.0 to 137)	647 (551 to 763)	400 (293 to 532)	850 (617 to 1142)	0.87 (0.82 to 0.90)
Republic of Moldova	10.7 (8.89 to 12.9)	406 (339 to 492)	20.3 (16.0 to 25.7)	631 (497 to 801)	1.56 (1.32 to 1.82)	0.99 (0.89 to 1.11)	41.6 (37.8 to 46.8)	0.94 (0.78 to 1.15)	47.2 (40.1 to 56.2)	0.36 (-0.12 to 0.65)	9.67 (7.94 to 11.8)	365 (299 to 447)	19.4 (15.2 to 24.6)	584 (456 to 746)	1.63 (1.44 to 1.83)
Romania	58.6 (48.3 to 70.4)	384 (318 to 462)	86.1 (66.2 to 111)	440 (334 to 576)	0.42 (0.36 to 0.47)	5.35 (4.87 to 5.93)	41.1 (37.6 to 45.5)	2.97 (2.30 to 3.82)	22.3 (17.5 to 28.6)	-1.94 (-2.03 to -1.88)	53.2 (43.4 to 64.4)	343 (280 to 417)	83.1 (64.0 to 108)	417 (318 to 549)	0.61 (0.55 to 0.66)
Russian Federation	323 (266 to 389)	291 (241 to 351)	970 (832 to 1155)	676 (578 to 808)	2.82 (2.72 to 2.93)	36.3 (32.0 to 41.6)	42.4 (37.8 to 48.1)	42.8 (34.8 to 53.5)	47.9 (40.1 to 58.1)	0.63 (0.43 to 0.81)	286 (233 to 349)	249 (202 to 304)	927 (797 to 1106)	628 (535 to 754)	3.11 (3.02 to 3.21)
Rwanda	22.8 (15.8 to 31.8)	1293 (876 to 1825)	33.9 (23.4 to 48.1)	880 (599 to 1268)	-1.24 (-1.28 to -1.20)	2.68 (1.86 to 3.78)	72.1 (51.6 to 104)	2.78 (1.97 to 3.94)	41.6 (29.6 to 58.8)	-1.74 (-1.81 to -1.68)	20.2 (13.5 to 28.6)	1221 (817 to 1743)	31.1 (21.0 to 44.3)	839 (565 to 1215)	-1.21 (-1.26 to -1.17)
Saint Kitts and Nevis	0.52 (0.46 to 0.58)	2500 (2234 to 2827)	0.69 (0.56 to 0.85)	1846 (1516 to 2269)	-0.94 (-1.08 to -0.79)	0.03 (0.02 to 0.03)	141 (126 to 162)	0.02 (0.02 to 0.02)	56.0 (45.9 to 68.9)	-2.98 (-3.03 to -2.94)	0.49 (0.43 to 0.55)	2358 (2106 to 2662)	0.67 (0.54 to 0.83)	1790 (1470 to 2202)	-0.85 (-1.00 to -0.70)
Saint Lucia	1.58 (1.42 to 1.80)	3221 (2883 to 3667)	2.96 (2.43 to 3.61)	2374 (1950 to 2913)	-1.04 (-1.16 to -0.93)	0.08 (0.07 to 0.09)	132 (118 to 148)	0.07 (0.06 to 0.08)	68.6 (56.7 to 83.4)	-2.13 (-2.21 to -2.07)	1.50 (1.34 to 1.71)	3088 (2759 to 3519)	2.89 (2.38 to 3.53)	2305 (1893 to 2827)	-1.00 (-1.12 to -0.88)
Saint Vincent and the Grenadines	1.36 (1.24 to 1.52)	3395 (3075 to 3787)	1.93 (1.62 to 2.33)	2792 (2344 to 3362)	-0.57 (-0.69 to -0.47)	0.09 (0.08 to 0.10)	173 (154 to 193)	0.06 (0.05 to 0.07)	106 (91.7 to 124)	-1.61 (-1.71 to -1.53)	1.28 (1.16 to 1.42)	3222 (2912 to 3597)	1.87 (1.56 to 2.26)	2686 (2252 to 3243)	-0.53 (-0.66 to -0.42)
Samoa	0.96 (0.80 to 1.18)	2110 (1742 to 2588)	2.84 (2.26 to 3.46)	3722 (2975 to 4523)	1.84 (1.83 to 1.85)	0.03 (0.02 to 0.04)	33.7 (24.2 to 44.6)	0.03 (0.02 to 0.04)	31.7 (23.5 to 41.4)	-0.19 (-0.22 to -0.17)	0.93 (0.77 to 1.15)	2076 (1711 to 2544)	2.81 (2.24 to 3.42)	3691 (2951 to 4484)	1.87 (1.86 to 1.87)
San Marino	0.05 (0.04 to 0.06)	263 (208 to 327)	0.12 (0.09 to 0.15)	359 (264 to 489)	0.99 (0.93 to 1.02)	0.004 (0.003 to 0.005)	28.0 (20.2 to 36.9)	0.009 (0.006 to 0.01)	36.2 (23.9 to 52.3)	0.81 (0.76 to 0.84)	0.04 (0.04 to 0.05)	235 (187 to 291)	0.11 (0.08 to 0.14)	323 (239 to 439)	1.00 (0.95 to 1.04)
Sao Tome and Principe	0.18 (0.15 to 0.23)	484 (392 to 608)	0.55 (0.43 to 0.72)	827 (650 to 1068)	1.71 (1.68 to 1.75)	0.02 (0.02 to 0.03)	39.4 (28.8 to 50.9)	0.03 (0.02 to 0.04)	33.9 (24.9 to 45.5)	-0.44 (-0.57 to -0.31)	0.16 (0.13 to 0.20)	445 (357 to 558)	0.52 (0.40 to 0.68)	793 (623 to 1026)	1.85 (1.83 to 1.88)
Saudi Arabia	25.3 (20.6 to 30.4)	898 (730 to 1078)	140 (111 to 174)	1326 (1086 to 1632)	1.26 (1.23 to 1.29)	2.78 (2.11 to 3.71)	44.7 (33.9 to 58.1)	7.14 (4.75 to 11.2)	43.3 (29.4 to 68.2)	-0.10 (-0.13 to -0.07)	22.5 (18.1 to 27.3)	853 (691 to 1027)	133 (106 to 165)	1283 (1047 to 1579)	1.32 (1.29 to 1.35)
Senegal	17.6 (14.2 to 21.5)	979 (788 to 1189)	71.3 (55.5 to 87.1)	1649 (1288 to 2043)	1.68 (1.57 to 1.75)	1.71 (1.23 to 2.29)	47.5 (36.4 to 61.9)	2.72 (2.00 to 3.80)	37.8 (28.6 to 51.5)	-0.83 (-1.00 to -0.69)	15.9 (12.8 to 19.4)	931 (749 to 1135)	68.6 (53.5 to 84.4)	1611 (1259 to 2004)	1.77 (1.67 to 1.84)
Serbia	48.0 (39.5 to 57.5)	814 (673 to 972)	85.5 (67.6 to 106)	964 (757 to 1209)	0.56 (0.49 to 0.64)	4.82 (3.86 to 5.82)	87.9 (71.1 to 106)	3.50 (2.69 to 4.55)	53.7 (41.5 to 68.2)	-1.51 (-1.60 to -1.41)	43.1 (34.7 to 52.1)	726 (591 to 876)	82.0 (64.9 to 102)	910 (716 to 1145)	0.73 (0.65 to 0.82)
Seychelles	0.22 (0.19 to 0.26)	692 (586 to 825)	0.93 (0.74 to 1.19)	1543 (1227 to 1961)	2.60 (2.53 to 2.64)	0.02 (0.01 to 0.02)	46.1 (36.7 to 57.3)	0.02 (0.01 to 0.03)	37.1 (26.7 to 46.9)	-0.49 (-0.57 to -0.40)	0.21 (0.17 to 0.25)	646 (547 to 772)	0.91 (0.72 to 1.16)	1506 (1194 to 1920)	2.74 (2.66 to 2.79)
Sierra Leone	7.12 (5.72 to 8.66)	654 (527 to 798)	26.2 (20.7 to 33.7)	1220 (966 to 1538)	2.04 (2.02 to 2.06)	0.76 (0.50 to 1.08)	37.6 (27.0 to 51.7)	1.43 (1.00 to 1.98)	34.9 (25.7 to 47.4)	-0.25 (-0.30 to -0.21)	6.36 (5.13 to 7.80)	617 (498 to 753)	24.8 (19.5 to 32.0)	1185 (936 to 1494)	2.14 (2.12 to 2.16)
Singapore	10.4 (8.65 to 12.5)	826 (693 to 993)	23.2 (16.3 to 32.2)	552 (386 to 768)	-1.43 (-1.55 to -1.31)	0.38 (0.31 to 0.48)	23.6 (19.1 to 29.8)	0.53 (0.35 to 0.77)	15.9 (10.6 to 23.2)	-1.27 (-1.33 to -1.24)	9.99 (8.31 to 12.1)	802 (672 to 967)	22.6 (15.9 to 31.5)	536 (375 to 746)	-1.43 (-1.55 to -1.31)
Slovakia	17.1 (13.9 to 20.5)	498 (406 to 601)	27.4 (21.2 to 35.5)	526 (406 to 685)	0.16 (0.10 to 0.22)	1.42 (1.12 to 1.73)	45.8 (36.6 to 55.5)	1.27 (0.97 to 1.62)	33.0 (25.7 to 41.6)	-1.03 (-1.18 to -0.90)	15.6 (12.6 to 18.8)	452 (363 to 548)	26.1 (20.1 to 34.1)	493 (377 to 646)	0.26 (0.20 to 0.31)
Slovenia	7.48 (6.19 to 9.15)	510 (422 to 624)	10.9 (8.46 to 14.2)	484 (369 to 638)	-0.20 (-0.30 to -0.11)	0.63 (0.57 to 0.70)	50.1 (44.8 to 56.6)	0.38 (0.29 to 0.51)	25.6 (19.2 to 34.0)	-2.08 (-2.21 to -1.96)	6.85 (5.59 to 8.45)	460 (375 to 567)	10.5 (8.18 to 13.7)	458 (350 to 608)	-0.05 (-0.16 to 0.05)
Solomon Islands	1.65 (1.08 to 2.24)	2339 (1654 to 3102)	7.03 (5.59 to 8.70)	3474 (2750 to 4271)	1.26 (1.19 to 1.32)	0.05 (0.03 to 0.09)	37.5 (21.6 to 63.5)	0.14 (0.11 to 0.21)	44.8 (33.3 to 63.8)	0.58 (0.52 to 0.65)	1.60 (1.05 to 2.16)	2301 (1626 to 3045)	6.89 (5.47 to 8.53)	3429 (2718 to 4213)	1.27 (1.20 to 1.33)
Somalia	17.9 (13.7 to 22.6)	1197 (908 to 1514)	57.8 (43.8 to 73.5)	1404 (1064 to 1745)	0.53 (0.51 to 0.55)	2.36 (1.56 to 3.50)	60.7 (43.6 to 92.8)	6.37 (4.31 to 8.88)	60.1 (43.8 to 83.0)	-0.02 (-0.06 to 0.03)	15.6 (11.5 to 20.1)	1136 (862 to 1443)	51.4 (38.9 to 65.5)	1344 (1016 to 1676)	0.56 (0.54 to 0.58)
South Africa	160 (144 to 180)	1287 (1155 to 1442)	595 (536 to 666)	2202 (1992 to 2460)	1.70 (1.50 to 1.86)	8.19 (6.48 to 10.8)	43.7 (35.0 to 56.7)	10.6 (8.43 to 13.2)	36.1 (28.6 to 45.2)	-0.58 (-0.65 to -0.51)	152 (136 to 170)	1244 (1117 to 1391)	584 (527 to 654)	2166 (1960 to 2417)	1.76 (1.55 to 1.93)
South Sudan	11.4 (8.92 to 14.5)	961 (748 to 1245)	26.5 (20.5 to 34.1)	1236 (951 to 1561)	0.84 (0.81 to 0.87)	1.32 (0.91 to 1.81)	46.2 (33.4 to 62.6)	2.29 (1.53 to 3.27)	46.6 (33.8 to 65.3)	0.06 (-0.04 to 0.14)	10.0 (7.77 to 12.9)	915 (709 to 1187)	24.2 (18.5 to 31.1)	1190 (909 to 1503)	0.87 (0.85 to 0.90)
Spain	189 (161 to 224)	614 (516 to 732)	285 (213 to 382)	608 (445 to 835)	-0.05 (-0.08 to -0.02)	8.12 (6.32 to 10.3)	36.0 (28.4 to 45.3)	14.6 (10.2 to 20.2)	45.5 (31.8 to 62.7)	0.73 (0.67 to 0.77)	181 (154 to 214)	578 (488 to 688)	270 (202 to 361)	563 (412 to 774)	-0.09 (-0.12 to -0.06)
Sri Lanka	59.8 (51.2 to 69.3)	1114 (957 to 1284)	287 (223 to 361)	1909 (1490 to 2410)	1.76 (1.67 to 1.83)	4.80 (3.77 to 6.17)	60.9 (47.4 to 77.9)	6.37 (4.57 to 8.49)	48.6 (35.1 to 64.8)	-0.62 (-0.75 to -0.51)	55.0 (46.8 to 63.7)	1053 (903 to 1214)	280 (218 to 352)	1860 (1452 to 2349)	1.86 (1.77 to 1.93)
Sudan	31.4 (25.4 to 39.5)	601 (487 to 758)	113 (87.9 to 147)	1041 (823 to 1327)	1.79 (1.78 to 1.80)	5.61 (3.55 to 9.62)	51.3 (33.6 to 81.2)	8.90 (6.10 to 12.8)	41.1 (28.6 to 58.9)	-0.72 (-0.78 to -0.68)	25.8 (20.7 to 32.6)	549 (443 to 689)	105 (80.7 to 136)	1000 (785 to 1275)	1.95 (1.94 to 1.97)
Suriname	2.18 (1.86 to 2.52)	1569 (1350 to 1806)	7.02 (5.69 to 8.71)	2045 (1652 to 2537)	0.89 (0.80 to 0.96)	0.12 (0.09 to 0.14)	67.0 (51.7 to 79.3)	0.16 (0.13 to 0.21)	53.9 (41.0 to 67.6)	-0.66 (-0.78 to -0.55)	2.06 (1.76 to 2.39)	1502 (1286 to 1736)	6.85 (5.54 to 8.50)	1991 (1609 to 2469)	0.95 (0.88 to 1.00)
Sweden	24.8 (21.2 to 29.6)	327 (274 to 398)	36.6 (29.1 to 46.8)	379 (292 to 499)	0.46 (0.40 to 0.52)	2.68 (2.16 to 3.39)	50.2 (40.3 to 64.3)	2.78 (2.01 to 3.82)	42.1 (30.4 to 57.9)	-0.53 (-0.58 to -0.47)	22.1 (18.9 to 26.2)	277 (232 to 335)	33.8 (26.9 to 43.1)	337 (260 to 443)	0.61 (0.54 to 0.68)
Switzerland	26.9 (23.3 to 31.9)	460 (390 to 556)	40.8 (30.6 to 55.1)	499 (366 to 691)	0.30 (0.21 to 0.37)	1.55 (1.26 to 1.95)	36.2 (29.4 to 45.6)	2.01 (1.38 to 2.85)	32.9 (22.3 to 46.9)	-0.38 (-0.42 to -0.34)	25.4 (21.9 to 30.1)	424 (359 to 514)	38.8 (29.2 to 52.4)	467 (340 to 647)	0.38 (0.29 to 0.46)
Syrian Arab Republic	22.9 (18.7 to 27.1)	811 (663 to 969)	74.2 (57.6 to 96.8)	1111 (872 to 1436)	1.05 (0.99 to 1.10)	2.82 (2.10 to 3.67)	50.6 (39.1 to 65.0)	2.72 (2.00 to 3.59)	36.7 (27.2 to 47.9)	-0.99 (-1.03 to -0.94)	20.0 (16.2 to 24.1)	760 (619 to 914)	71.5 (55.6 to 93.5)	1074 (842 to 1391)	1.15 (1.09 to 1.20)
Taiwan (Province of China)	94.9 (85.2 to 107)	1244 (1122 to 1393)	192 (155 to 235)	874 (708 to 1072)	-1.23 (-1.39 to -1.10)	8.35 (7.70 to 9.18)	91.3 (84.4 to 100)	8.19 (7.09 to 9.48)	49.8 (43.5 to 57.0)	-1.89 (-2.00 to -1.79)	86.5 (77.3 to 97.9)	1153 (1035 to 1293)	184 (148 to 225)	825 (664 to 1017)	-1.18 (-1.34 to -1.04)
Tajikistan	7.98 (6.79 to 9.35)	473 (401 to 558)	26.9 (21.5 to 34.1)	764 (619 to 966)	1.55 (1.51 to 1.58)	1.33 (1.03 to 1.73)	55.1 (41.9 to 71.7)	2.59 (1.89 to 3.37)	53.5 (38.8 to 70.0)	-0.08 (-0.13 to -0.02)	6.65 (5.58 to 7.81)	418 (353 to 493)	24.3 (19.3 to 31.0)	710 (572 to 904)	1.71 (1.67 to 1.74)
Thailand	176 (142 to 207)	875 (704 to 1034)	570 (454 to 713)	987 (786 to 1232)	0.36 (0.29 to 0.43)	14.8 (11.1 to 19.1)	55.7 (41.8 to 72.8)	15.8 (12.0 to 20.9)	36.5 (28.0 to 47.6)	-1.29 (-1.38 to -1.21)	161 (130 to 191)	820 (655 to 968)	554 (441 to 694)	951 (756 to 1189)	0.48 (0.41 to 0.55)
Timor-Leste	1.18 (0.92 to 1.57)	736 (577 to 957)	5.11 (4.09 to 6.43)	1132 (909 to 1430)	1.40 (1.35 to 1.44)	0.16 (0.12 to 0.27)	52.4 (37.0 to 90.7)	0.28 (0.20 to 0.37)	44.4 (32.4 to 61.7)	-0.54 (-0.64 to -0.41)	1.02 (0.79 to 1.32)	684 (533 to 878)	4.83 (3.84 to 6.11)	1088 (872 to 1373)	1.52 (1.47 to 1.56)
Togo	5.17 (4.24 to 6.28)	669 (546 to 806)	23.1 (18.2 to 29.4)	987 (782 to 1248)	1.27 (1.25 to 1.29)	0.68 (0.48 to 0.93)	40.8 (30.0 to 53.8)	1.25 (0.86 to 1.72)	31.9 (22.4 to 43.2)	-0.79 (-0.83 to -0.75)	4.49 (3.66 to 5.46)	628 (513 to 756)	21.9 (17.3 to 28.0)	956 (757 to 1212)	1.36 (1.34 to 1.38)
Tokelau	0.02 (0.02 to 0.02)	2762 (2164 to 3482)	0.03 (0.02 to 0.03)	3670 (2965 to 4510)	0.94 (0.91 to 0.98)	4e-04 (3e-04 to 5e-04)	46.4 (32.7 to 63.3)	6e-04 (4e-04 to 9e-04)	103 (69.3 to 149)	3.23 (2.44 to 4.42)	0.02 (0.01 to 0.02)	2715 (2131 to 3425)	0.03 (0.02 to 0.03)	3566 (2875 to 4403)	0.88 (0.86 to 0.90)
Tonga	0.95 (0.81 to 1.09)	3125 (2677 to 3586)	1.72 (1.37 to 2.16)	3963 (3164 to 4981)	0.78 (0.76 to 0.81)	0.02 (0.02 to 0.03)	40.9 (30.9 to 54.4)	0.02 (0.01 to 0.03)	36.1 (26.8 to 49.1)	-0.40 (-0.43 to -0.37)	0.93 (0.79 to 1.07)	3084 (2642 to 3542)	1.70 (1.36 to 2.13)	3927 (3132 to 4937)	0.80 (0.77 to 0.82)
Trinidad and Tobago	17.5 (16.1 to 19.2)	3934 (3623 to 4308)	30.9 (25.5 to 38.0)	3109 (2572 to 3828)	-0.73 (-0.88 to -0.61)	0.80 (0.73 to 0.88)	143 (130 to 157)	0.70 (0.56 to 0.88)	89.3 (71.1 to 113)	-1.50 (-1.64 to -1.41)	16.7 (15.3 to 18.3)	3791 (3488 to 4158)	30.2 (25.0 to 37.2)	3020 (2499 to 3726)	-0.71 (-0.85 to -0.59)
Tunisia	13.1 (10.5 to 16.9)	501 (404 to 648)	72.1 (54.9 to 94.7)	1037 (794 to 1356)	2.36 (2.33 to 2.38)	1.31 (0.93 to 2.11)	32.5 (23.1 to 51.1)	1.72 (1.28 to 2.35)	27.1 (20.2 to 36.8)	-0.58 (-0.61 to -0.55)	11.8 (9.33 to 15.3)	469 (375 to 600)	70.4 (53.5 to 92.8)	1010 (769 to 1326)	2.49 (2.47 to 2.52)
Turkmenistan	5.09 (4.43 to 5.82)	412 (359 to 475)	22.3 (18.3 to 27.9)	915 (755 to 1142)	2.67 (2.51 to 2.81)	1.14 (1.03 to 1.26)	70.9 (63.5 to 80.1)	2.22 (1.75 to 2.86)	87.9 (69.6 to 113)	0.89 (0.45 to 1.12)	3.95 (3.37 to 4.57)	341 (292 to 394)	20.0 (16.4 to 25.0)	827 (679 to 1029)	2.97 (2.80 to 3.11)
Tuvalu	0.11 (0.09 to 0.14)	2782 (2273 to 3484)	0.21 (0.17 to 0.26)	3668 (2950 to 4599)	0.91 (0.88 to 0.93)	0.003 (0.002 to 0.004)	50.1 (37.6 to 71.7)	0.002 (0.002 to 0.003)	39.5 (29.5 to 52.1)	-0.74 (-0.79 to -0.70)	0.11 (0.09 to 0.14)	2731 (2229 to 3400)	0.20 (0.16 to 0.26)	3628 (2920 to 4549)	0.93 (0.91 to 0.95)
Türkiye	198 (170 to 229)	1059 (908 to 1225)	527 (425 to 659)	1061 (857 to 1325)	0.05 (-0.08 to 0.13)	17.9 (12.7 to 25.7)	62.6 (45.2 to 86.4)	13.2 (9.96 to 17.4)	29.8 (22.8 to 39.1)	-2.36 (-2.43 to -2.27)	180 (154 to 209)	996 (855 to 1150)	513 (415 to 643)	1031 (831 to 1287)	0.16 (0.08 to 0.22)
Uganda	28.0 (20.6 to 37.7)	784 (565 to 1052)	86.8 (64.6 to 114)	949 (694 to 1238)	0.59 (0.56 to 0.62)	3.29 (2.28 to 4.59)	36.5 (25.9 to 49.9)	8.10 (5.75 to 11.1)	38.3 (27.5 to 53.2)	0.14 (0.07 to 0.21)	24.7 (17.8 to 33.6)	748 (534 to 1009)	78.7 (56.9 to 103)	910 (664 to 1186)	0.61 (0.58 to 0.64)
Ukraine	119 (98.0 to 146)	291 (241 to 355)	160 (120 to 209)	386 (291 to 501)	0.90 (0.81 to 0.98)	15.4 (13.5 to 17.9)	52.0 (46.1 to 59.6)	10.5 (7.57 to 14.1)	39.1 (28.2 to 52.7)	-0.98 (-1.15 to -0.84)	103 (84.1 to 128)	239 (192 to 295)	150 (112 to 197)	347 (257 to 457)	1.21 (1.13 to 1.28)
United Arab Emirates	2.41 (1.99 to 2.95)	1464 (1214 to 1795)	19.5 (15.2 to 24.9)	2480 (2050 to 2993)	1.62 (1.35 to 1.85)	0.24 (0.18 to 0.32)	46.4 (34.9 to 61.8)	0.93 (0.68 to 1.27)	40.3 (30.3 to 53.1)	-0.46 (-0.54 to -0.39)	2.17 (1.79 to 2.66)	1418 (1175 to 1741)	18.6 (14.4 to 23.8)	2439 (2013 to 2948)	1.67 (1.39 to 1.90)
United Kingdom	158 (136 to 188)	334 (280 to 403)	269 (201 to 355)	496 (361 to 670)	1.22 (1.16 to 1.28)	12.6 (10.2 to 15.7)	36.8 (30.2 to 45.6)	12.7 (9.21 to 17.6)	29.4 (21.4 to 40.4)	-0.77 (-0.86 to -0.71)	145 (125 to 172)	297 (250 to 359)	257 (192 to 338)	467 (337 to 629)	1.41 (1.33 to 1.48)
United Republic of Tanzania	51.4 (43.1 to 61.5)	817 (684 to 978)	143 (115 to 176)	960 (776 to 1173)	0.55 (0.51 to 0.58)	8.07 (5.77 to 10.9)	54.1 (41.4 to 69.9)	14.3 (10.3 to 19.6)	46.4 (33.8 to 62.7)	-0.52 (-0.56 to -0.48)	43.3 (36.0 to 52.5)	763 (636 to 920)	128 (102 to 159)	913 (735 to 1124)	0.60 (0.57 to 0.64)
United States Virgin Islands	0.86 (0.71 to 1.03)	1824 (1538 to 2167)	1.52 (1.18 to 1.98)	1760 (1343 to 2277)	-0.13 (-0.17 to -0.09)	0.06 (0.05 to 0.07)	106 (85.8 to 131)	0.04 (0.03 to 0.05)	67.1 (45.2 to 88.7)	-1.32 (-1.46 to -1.08)	0.80 (0.67 to 0.97)	1718 (1447 to 2054)	1.49 (1.15 to 1.93)	1693 (1280 to 2193)	-0.07 (-0.12 to -0.03)
United States of America	961 (844 to 1119)	563 (492 to 657)	2243 (1758 to 2830)	795 (627 to 1001)	1.12 (1.09 to 1.15)	87.2 (73.6 to 105)	59.6 (50.3 to 72.2)	117 (95.1 to 146)	58.6 (48.1 to 72.3)	-0.06 (-0.09 to -0.03)	874 (766 to 1018)	503 (439 to 588)	2127 (1659 to 2686)	736 (575 to 932)	1.23 (1.21 to 1.25)
Uruguay	10.6 (9.41 to 12.2)	490 (431 to 562)	19.3 (15.8 to 23.8)	653 (527 to 818)	0.94 (0.87 to 1.01)	0.60 (0.48 to 0.76)	34.2 (27.5 to 43.4)	0.70 (0.52 to 0.93)	32.0 (24.2 to 43.0)	-0.24 (-0.29 to -0.18)	10.0 (8.91 to 11.4)	456 (404 to 523)	18.6 (15.3 to 23.0)	621 (502 to 779)	1.01 (0.94 to 1.09)
Uzbekistan	30.3 (26.2 to 34.7)	425 (367 to 490)	181 (153 to 221)	1150 (972 to 1390)	3.31 (3.18 to 3.41)	5.21 (4.53 to 6.07)	56.1 (47.5 to 65.6)	13.6 (11.4 to 16.3)	78.5 (65.6 to 93.7)	1.13 (0.99 to 1.26)	25.1 (21.5 to 29.2)	369 (317 to 429)	167 (140 to 203)	1071 (900 to 1290)	3.54 (3.42 to 3.65)
Vanuatu	0.68 (0.52 to 0.87)	2117 (1624 to 2677)	3.15 (2.60 to 3.73)	3188 (2670 to 3725)	1.30 (1.26 to 1.35)	0.03 (0.02 to 0.04)	38.2 (26.3 to 52.7)	0.06 (0.05 to 0.08)	39.9 (30.1 to 52.6)	0.09 (0.01 to 0.15)	0.65 (0.50 to 0.83)	2079 (1595 to 2637)	3.08 (2.55 to 3.66)	3148 (2638 to 3676)	1.32 (1.27 to 1.36)
Venezuela (Bolivarian Republic of)	63.2 (56.0 to 72.8)	1162 (1032 to 1318)	239 (196 to 298)	1476 (1209 to 1836)	0.77 (0.66 to 0.87)	3.02 (2.77 to 3.32)	35.8 (32.8 to 39.6)	5.66 (4.45 to 7.00)	39.8 (31.5 to 49.2)	0.36 (-0.08 to 0.61)	60.2 (53.1 to 69.5)	1126 (998 to 1277)	233 (191 to 291)	1436 (1174 to 1787)	0.78 (0.67 to 0.88)
Viet Nam	217 (180 to 270)	916 (759 to 1137)	628 (511 to 771)	1142 (928 to 1395)	0.71 (0.70 to 0.72)	15.2 (11.2 to 20.7)	52.9 (38.8 to 73.5)	21.8 (15.0 to 30.2)	39.1 (27.2 to 53.7)	-0.96 (-0.97 to -0.94)	202 (167 to 249)	863 (714 to 1066)	607 (490 to 744)	1103 (891 to 1353)	0.79 (0.78 to 0.80)
Yemen	16.0 (12.4 to 21.1)	524 (412 to 697)	70.8 (54.9 to 94.7)	843 (651 to 1122)	1.56 (1.54 to 1.59)	3.14 (1.90 to 5.71)	42.3 (27.0 to 69.3)	6.48 (4.53 to 9.77)	38.7 (27.0 to 58.1)	-0.26 (-0.32 to -0.19)	12.8 (10.1 to 16.8)	482 (382 to 634)	64.3 (49.9 to 86.8)	804 (624 to 1079)	1.69 (1.65 to 1.72)
Zambia	18.8 (15.6 to 22.4)	1136 (943 to 1350)	49.4 (38.8 to 61.9)	1140 (909 to 1403)	0.00 (-0.02 to 0.03)	2.81 (1.97 to 3.90)	64.6 (48.8 to 91.3)	4.19 (2.95 to 5.79)	43.1 (31.6 to 58.5)	-1.24 (-1.28 to -1.21)	16.0 (13.3 to 19.2)	1072 (890 to 1279)	45.2 (35.4 to 56.9)	1097 (871 to 1356)	0.06 (0.04 to 0.09)
Zimbabwe	22.6 (18.8 to 26.9)	1069 (879 to 1278)	90.8 (69.9 to 113)	2185 (1702 to 2707)	2.39 (2.33 to 2.46)	0.82 (0.62 to 1.08)	17.7 (13.2 to 22.8)	2.13 (1.49 to 2.92)	27.3 (19.4 to 37.5)	1.29 (1.18 to 1.38)	21.8 (18.0 to 26.0)	1051 (864 to 1258)	88.7 (67.9 to 111)	2158 (1680 to 2677)	2.41 (2.34 to 2.47)

AAPC: Average annual percentage change; ASR: Age-standardized rate; DALY: Disability adjusted life years; DM: Diabetes mellitus; SDI: Sociodemographic index; T1D: Type 1 diabetes; T2D: Type 2 diabetes. Results assuming first-order autocorrelation estimated from the data were broadly similar to those of the primary analyses, despite somewhat different estimates.

Table S7. Annual percentage changes estimated from joinpoint regression for age-standardized **incidence, prevalence, mortality, YLD, YLL, and DALY** of type 1 diabetes among **women** globally and by different SDI, regions, countries, and territories, 1990-2021

Location	Segment	Incidence		Prevalence		Mortality		YLD		YLL		DALY	
		Period	APC (95%CI)	Period	APC (95%CI)	Period	APC (95%CI)	Period	APC (95%CI)	Period	APC (95%CI)	Period	APC (95%CI)
Global	0	1990-1993	0.22 (0.15 to 0.26)	1990-1996	0.32 (0.28 to 0.35)	1990-1995	0.01 (-0.22 to 0.20)	1990-2001	0.45 (0.43 to 0.46)	1990-1995	0.15 (-0.14 to 0.47)	1990-1995	0.20 (0.03 to 0.40)
	1	1993-1996	0.37 (0.33 to 0.55)	1996-2006	0.52 (0.32 to 0.54)	1995-2003	-1.52 (-1.62 to -1.42)	2001-2006	0.58 (0.50 to 0.63)	1995-2004	-1.25 (-1.40 to -0.49)	1995-2003	-0.72 (-0.82 to -0.61)
	2	1996-2004	0.58 (0.57 to 0.61)	2006-2009	0.66 (0.50 to 0.69)	2003-2011	-2.48 (-2.60 to -2.36)	2006-2009	0.78 (0.70 to 0.83)	2004-2011	-2.26 (-2.54 to -1.18)	2003-2012	-1.21 (-1.34 to -1.13)
	3	2004-2013	0.52 (0.51 to 0.53)	2009-2014	0.59 (0.54 to 0.63)	2011-2014	-1.30 (-2.38 to -1.14)	2009-2014	0.64 (0.58 to 0.68)	2011-2014	-1.10 (-2.34 to -0.92)	2012-2017	-0.11 (-0.29 to 0.24)
	4	2013-2017	0.41 (0.37 to 0.45)	2014-2019	0.46 (0.40 to 0.48)	2014-2017	-0.32 (-0.61 to -0.13)	2014-2021	0.50 (0.47 to 0.52)	2014-2017	-0.27 (-0.67 to -0.02)	2017-2021	-0.71 (-1.09 to -0.49)
	5	2017-2021	0.52 (0.49 to 0.59)	2019-2021	0.64 (0.55 to 0.70)	2017-2021	-1.35 (-1.56 to -1.23)			2017-2021	-1.50 (-1.79 to -1.33)		
**By SDI**													
High SDI	0	1990-1994	1.33 (1.28 to 1.40)	1990-1993	1.41 (1.31 to 1.51)	1990-1994	-1.13 (-1.51 to -0.57)	1990-1992	1.53 (1.32 to 1.68)	1990-1998	-1.72 (-2.66 to -1.41)	1990-1994	-0.05 (-0.21 to 0.25)
	1	1994-2004	1.14 (1.12 to 1.15)	1993-1999	1.03 (1.00 to 1.10)	1994-1998	-2.83 (-3.44 to -2.45)	1992-2000	1.20 (1.13 to 1.23)	1998-2003	-0.96 (-3.87 to -0.33)	1994-1998	-0.61 (-0.90 to -0.42)
	2	2004-2011	0.75 (0.73 to 0.77)	1999-2004	0.95 (0.64 to 0.98)	1998-2002	-1.43 (-1.84 to -0.84)	2000-2011	0.77 (0.74 to 0.79)	2003-2014	-3.72 (-3.96 to -3.47)	1998-2002	0.26 (-0.03 to 0.54)
	3	2011-2015	1.40 (1.36 to 1.43)	2004-2011	0.67 (0.64 to 0.72)	2002-2006	-3.43 (-3.91 to -2.87)	2011-2015	1.17 (1.07 to 1.29)	2014-2017	1.89 (0.85 to 2.51)	2002-2013	-1.18 (-1.23 to -1.12)
	4	2015-2019	0.83 (0.77 to 0.88)	2011-2015	1.22 (1.18 to 1.28)	2006-2013	-4.41 (-4.81 to -4.24)	2015-2021	0.78 (0.72 to 0.83)	2017-2021	-2.03 (-3.07 to -1.51)	2013-2017	0.95 (0.74 to 1.22)
	5	2019-2021	1.09 (0.96 to 1.18)	2015-2019	0.68 (0.59 to 0.73)	2013-2018	-0.37 (-0.64 to 0.07)					2017-2021	-0.17 (-0.54 to 0.08)
	6			2019-2021	1.14 (0.97 to 1.25)	2018-2021	-2.23 (-3.23 to -1.62)						
High-middle SDI	0	1990-1996	-0.08 (-0.21 to 0.01)	1990-1995	-0.11 (-0.32 to -0.00)	1990-1995	1.22 (0.41 to 2.16)	1990-1995	-0.09 (-0.30 to 0.04)	1990-1994	2.07 (0.84 to 3.60)	1990-1994	1.29 (0.65 to 2.01)
	1	1996-2003	1.02 (0.91 to 1.12)	1995-2002	0.78 (0.15 to 0.85)	1995-2003	-2.67 (-3.05 to -2.27)	1995-2002	0.85 (0.71 to 0.96)	1994-2003	-2.51 (-2.88 to -2.14)	1994-2002	-1.45 (-1.69 to -1.17)
	2	2003-2012	1.53 (1.47 to 1.63)	2002-2006	1.08 (0.82 to 1.53)	2003-2012	-5.20 (-5.67 to -4.93)	2002-2010	1.34 (1.26 to 1.55)	2003-2011	-5.55 (-6.20 to -5.18)	2002-2011	-2.81 (-3.22 to -2.59)
	3	2012-2021	1.08 (1.00 to 1.14)	2006-2009	1.66 (1.21 to 1.79)	2012-2021	-1.72 (-2.03 to -1.32)	2010-2019	1.11 (0.86 to 1.16)	2011-2021	-1.90 (-2.19 to -1.58)	2011-2021	-0.36 (-0.62 to -0.06)
	4			2009-2019	1.20 (1.08 to 1.24)			2019-2021	1.93 (1.39 to 2.22)				
	5			2019-2021	1.95 (1.53 to 2.17)								
Middle SDI	0	1990-1994	0.17 (-0.10 to 0.33)	1990-2000	0.28 (0.22 to 0.33)	1990-1996	-0.27 (-0.49 to 0.00)	1990-2001	0.29 (0.23 to 0.34)	1990-1996	-0.40 (-0.63 to -0.02)	1990-1996	-0.28 (-0.43 to -0.12)
	1	1994-1999	0.43 (0.33 to 0.84)	2000-2011	0.86 (0.81 to 0.92)	1996-2003	-1.48 (-1.63 to -1.29)	2001-2011	0.91 (0.85 to 1.01)	1996-2003	-1.33 (-1.57 to -1.14)	1996-2003	-0.86 (-0.99 to -0.29)
	2	1999-2010	0.80 (0.30 to 0.89)	2011-2021	0.50 (0.44 to 0.55)	2003-2007	-2.95 (-3.26 to -2.52)	2011-2021	0.53 (0.46 to 0.59)	2003-2011	-2.50 (-2.71 to -2.38)	2003-2007	-1.71 (-1.91 to -0.81)
	3	2010-2021	0.34 (0.28 to 0.40)			2007-2012	-2.34 (-2.60 to -1.27)			2011-2021	-1.07 (-1.16 to -0.96)	2007-2012	-1.29 (-1.74 to -0.80)
	4					2012-2021	-0.99 (-1.08 to -0.85)					2012-2019	-0.45 (-1.26 to -0.15)
	5											2019-2021	-0.96 (-1.33 to -0.51)
Low-middle SDI	0	1990-2006	0.09 (0.08 to 0.10)	1990-1995	0.07 (0.03 to 0.09)	1990-1996	0.36 (0.10 to 0.67)	1990-1992	0.77 (0.65 to 0.89)	1990-1996	0.20 (-0.08 to 0.63)	1990-1996	0.28 (0.10 to 0.52)
	1	2006-2009	0.29 (0.23 to 0.32)	1995-2006	0.20 (0.12 to 0.22)	1996-2004	-0.94 (-1.44 to -0.80)	1992-1995	0.34 (0.25 to 0.41)	1996-2008	-0.76 (-0.84 to -0.64)	1996-2004	-0.71 (-1.06 to -0.61)
	2	2009-2016	0.16 (0.12 to 0.18)	2006-2009	0.50 (0.20 to 0.52)	2004-2008	-0.29 (-0.56 to 0.09)	1995-2000	-0.32 (-0.36 to -0.28)	2008-2011	-1.69 (-1.94 to -1.20)	2004-2008	-0.19 (-0.41 to 0.10)
	3	2016-2021	0.40 (0.37 to 0.45)	2009-2013	0.35 (0.33 to 0.52)	2008-2011	-1.58 (-1.82 to -1.14)	2000-2005	0.17 (0.13 to 0.21)	2011-2017	-0.55 (-0.72 to -0.14)	2008-2011	-1.20 (-1.39 to -0.87)
	4			2013-2016	0.28 (0.25 to 0.33)	2011-2018	-0.29 (-0.39 to -0.10)	2005-2013	0.72 (0.70 to 0.74)	2017-2021	-1.92 (-2.25 to -1.63)	2011-2017	-0.30 (-0.41 to -0.01)
	5			2016-2021	0.40 (0.38 to 0.44)	2018-2021	-2.10 (-2.42 to -1.71)	2013-2021	0.26 (0.24 to 0.28)			2017-2021	-1.33 (-1.57 to -1.12)
Low SDI	0	1990-1994	-0.26 (-0.31 to -0.22)	1990-1992	-0.32 (-0.39 to -0.21)	1990-1995	0.06 (-0.22 to 0.33)	1990-1995	0.08 (0.04 to 0.12)	1990-1995	0.00 (-0.25 to 0.24)	1990-1995	0.01 (-0.20 to 0.19)
	1	1994-2002	-0.01 (-0.03 to 0.00)	1992-1995	-0.06 (-0.09 to 0.09)	1995-2000	-1.51 (-1.96 to -1.24)	1995-2000	-0.25 (-0.31 to -0.20)	1995-2002	-1.31 (-1.62 to -1.17)	1995-2002	-1.07 (-1.26 to -0.95)
	2	2002-2010	0.11 (0.09 to 0.12)	1995-2005	0.10 (0.09 to 0.13)	2000-2006	-0.78 (-0.94 to -0.34)	2000-2005	0.09 (0.02 to 0.14)	2002-2006	-0.52 (-0.85 to -0.19)	2002-2006	-0.36 (-0.62 to -0.09)
	3	2010-2016	-0.15 (-0.17 to -0.13)	2005-2010	0.22 (0.09 to 0.27)	2006-2010	-1.71 (-2.05 to -1.47)	2005-2010	0.41 (0.37 to 0.48)	2006-2010	-2.12 (-2.39 to -1.84)	2006-2010	-1.53 (-1.73 to -1.32)
	4	2016-2021	0.21 (0.19 to 0.24)	2010-2016	-0.21 (-0.24 to 0.22)	2010-2013	0.79 (0.36 to 1.06)	2010-2013	0.01 (-0.04 to 0.28)	2010-2013	0.21 (-0.16 to 0.46)	2010-2013	0.17 (-0.12 to 0.35)
	5			2016-2019	0.05 (-0.21 to 0.08)	2013-2017	-1.11 (-1.31 to -0.85)	2013-2018	-0.13 (-0.22 to -0.10)	2013-2017	-1.05 (-1.28 to -0.82)	2013-2017	-0.83 (-0.99 to -0.65)
	6			2019-2021	0.25 (0.14 to 0.32)	2017-2021	-1.79 (-2.14 to -1.62)	2018-2021	0.10 (0.02 to 0.21)	2017-2021	-1.83 (-2.12 to -1.66)	2017-2021	-1.35 (-1.59 to -1.22)
**Four world regions**													
Africa	0	1990-1994	-0.39 (-0.50 to -0.34)	1990-1994	-0.38 (-0.44 to -0.34)	1990-1994	-1.21 (-1.38 to -0.98)	1990-1994	-0.40 (-0.52 to -0.34)	1990-1994	-1.12 (-1.29 to -0.81)	1990-1994	-0.92 (-1.03 to -0.73)
	1	1994-1999	-0.10 (-0.24 to -0.05)	1994-2001	0.00 (-0.03 to 0.03)	1994-1998	-1.94 (-2.19 to -1.79)	1994-2000	0.01 (-0.06 to 0.06)	1994-1998	-1.87 (-2.16 to -1.68)	1994-1998	-1.36 (-1.55 to -1.24)
	2	1999-2010	-0.02 (-0.04 to 0.02)	2001-2010	0.18 (0.16 to 0.20)	1998-2001	-1.22 (-1.41 to -0.69)	2000-2010	0.20 (0.09 to 0.23)	1998-2001	-1.04 (-1.43 to -0.00)	1998-2001	-0.71 (-0.93 to -0.26)
	3	2010-2015	-0.23 (-0.30 to -0.18)	2010-2015	-0.14 (-0.20 to -0.10)	2001-2005	0.28 (0.15 to 0.42)	2010-2015	-0.10 (-0.18 to 0.22)	2001-2005	0.14 (-1.12 to 0.40)	2001-2005	0.16 (0.03 to 0.31)
	4	2015-2019	0.10 (-0.08 to 0.18)	2015-2019	0.12 (0.03 to 0.20)	2005-2011	-1.14 (-1.25 to -1.06)	2015-2019	0.17 (-0.12 to 0.23)	2005-2011	-1.28 (-1.43 to -1.12)	2005-2011	-0.84 (-0.94 to -0.77)
	5	2019-2021	0.42 (0.26 to 0.51)	2019-2021	0.52 (0.38 to 0.62)	2011-2015	-0.40 (-0.53 to -0.21)	2019-2021	0.41 (0.22 to 0.52)	2011-2015	-0.42 (-0.63 to -0.18)	2011-2015	-0.34 (-0.47 to -0.19)
	6					2015-2021	-1.83 (-1.90 to -1.76)			2015-2021	-2.05 (-2.14 to -1.96)	2015-2021	-1.30 (-1.37 to -1.24)
America	0	1990-1993	0.56 (0.46 to 0.72)	1990-1994	0.72 (0.67 to 0.77)	1990-1995	1.01 (0.60 to 1.48)	1990-1994	0.84 (0.77 to 0.91)	1990-1996	0.48 (0.16 to 1.12)	1990-1995	0.65 (0.45 to 0.95)
	1	1993-2002	0.27 (0.21 to 0.29)	1994-2010	0.19 (0.18 to 0.20)	1995-2003	-0.98 (-1.18 to -0.79)	1994-2010	0.25 (0.24 to 0.27)	1996-2003	-0.60 (-1.01 to -0.30)	1995-2002	-0.17 (-0.36 to -0.01)
	2	2002-2010	0.38 (0.35 to 0.42)	2010-2015	0.57 (0.52 to 0.65)	2003-2013	-2.50 (-2.73 to -2.35)	2010-2015	0.68 (0.32 to 0.80)	2003-2013	-2.01 (-2.36 to -1.83)	2002-2013	-0.93 (-1.03 to -0.86)
	3	2010-2015	0.81 (0.75 to 0.89)	2015-2021	0.19 (0.15 to 0.23)	2013-2017	1.17 (0.61 to 2.28)	2015-2019	0.26 (0.20 to 0.63)	2013-2017	1.78 (1.03 to 3.00)	2013-2017	1.26 (0.93 to 1.74)
	4	2015-2019	0.47 (0.40 to 0.66)			2017-2021	-1.01 (-1.91 to -0.51)	2019-2021	-0.11 (-0.27 to 0.10)	2017-2021	-0.98 (-2.21 to -0.31)	2017-2021	-0.55 (-0.93 to -0.22)
	5	2019-2021	0.09 (-0.03 to 0.26)										
Asia	0	1990-1995	0.28 (0.19 to 0.34)	1990-2000	0.25 (0.21 to 0.28)	1990-1998	-0.29 (-0.48 to 0.01)	1990-1994	0.45 (0.33 to 0.66)	1990-1999	-0.28 (-0.45 to -0.08)	1990-1995	0.03 (-0.20 to 0.56)
	1	1995-2000	0.61 (0.51 to 0.71)	2000-2006	0.77 (0.33 to 0.84)	1998-2003	-1.40 (-1.91 to -0.94)	1994-2001	0.09 (-0.03 to 0.14)	1999-2006	-1.68 (-1.90 to -1.36)	1995-2000	-0.50 (-1.12 to -0.27)
	2	2000-2010	0.82 (0.80 to 0.86)	2006-2010	0.98 (0.77 to 1.12)	2003-2011	-2.46 (-2.70 to -2.31)	2001-2006	0.84 (0.63 to 0.92)	2006-2011	-2.56 (-3.05 to -2.29)	2000-2006	-1.16 (-1.63 to -0.95)
	3	2010-2021	0.54 (0.52 to 0.56)	2010-2021	0.67 (0.62 to 0.70)	2011-2021	-1.02 (-1.11 to -0.92)	2006-2010	1.18 (1.05 to 1.34)	2011-2021	-1.09 (-1.18 to -0.99)	2006-2011	-1.60 (-1.90 to -0.50)
	4							2010-2021	0.67 (0.64 to 0.70)			2011-2021	-0.58 (-0.68 to -0.47)
Europe	0	1990-1995	0.75 (0.50 to 0.91)	1990-1995	0.90 (0.57 to 1.08)	1990-1994	1.52 (0.33 to 2.38)	1990-1996	1.06 (0.71 to 1.22)	1990-1994	2.37 (0.99 to 3.58)	1990-1994	1.89 (1.18 to 2.36)

Location	Segment	Incidence Period	Incidence APC (95%CI)	Prevalence Period	Prevalence APC (95%CI)	Mortality Period	Mortality APC (95%CI)	YLD Period	YLD APC (95%CI)	YLL Period	YLL APC (95%CI)	DALY Period	DALY APC (95%CI)
	1	1995-2000	1.83 (1.68 to 2.18)	1995-1999	1.93 (1.66 to 2.23)	1994-2003	-3.73 (-3.98 to -3.45)	1996-1999	2.43 (1.94 to 2.66)	1994-2002	-3.38 (-3.74 to -2.92)	1994-2011	-1.50 (-1.61 to -1.42)
	2	2000-2021	1.53 (1.50 to 1.55)	1999-2021	1.59 (1.55 to 1.61)	2003-2011	-5.11 (-5.85 to -4.79)	1999-2021	1.60 (1.57 to 1.62)	2002-2012	-5.03 (-5.44 to -4.78)	2011-2021	0.51 (0.22 to 0.85)
	3					2011-2021	-1.64 (-1.97 to -1.26)			2012-2018	-0.23 (-0.74 to 0.81)		
	4									2018-2021	-3.79 (-6.71 to -2.29)		
**Six WHO regions**													
African Region	0	1990-1994	-0.41 (-0.46 to -0.37)	1990-1993	-0.47 (-0.58 to -0.42)	1990-1994	-1.17 (-1.36 to -0.96)	1990-1992	-0.50 (-0.57 to -0.41)	1990-1994	-1.07 (-1.29 to -0.77)	1990-1992	-0.50 (-0.92 to -0.17)
	1	1994-2000	-0.08 (-0.12 to -0.05)	1993-1996	-0.17 (-0.24 to -0.03)	1994-1998	-2.17 (-2.42 to -1.97)	1992-1995	-0.29 (-0.33 to 0.03)	1994-1999	-1.94 (-2.35 to -1.76)	1992-1999	-1.34 (-1.48 to -1.27)
	2	2000-2010	0.03 (0.01 to 0.05)	1996-2005	0.11 (0.09 to 0.12)	1998-2001	-1.44 (-1.81 to -0.65)	1995-2001	0.07 (0.03 to 0.13)	1999-2005	-0.75 (-0.92 to -0.53)	1999-2005	-0.52 (-0.62 to -0.38)
	3	2010-2016	-0.13 (-0.18 to 0.03)	2005-2010	0.24 (0.21 to 0.28)	2001-2004	-0.42 (-1.49 to -0.26)	2001-2006	0.18 (0.11 to 0.25)	2005-2011	-1.71 (-1.90 to -1.56)	2005-2011	-1.12 (-1.26 to -1.02)
	4	2016-2019	0.06 (-0.13 to 0.09)	2010-2018	-0.17 (-0.19 to -0.16)	2004-2011	-1.51 (-1.60 to -1.30)	2006-2010	0.26 (-0.10 to 0.30)	2011-2014	-0.08 (-0.62 to 0.11)	2011-2015	-0.21 (-0.39 to 0.00)
	5	2019-2021	0.36 (0.23 to 0.44)	2018-2021	0.16 (0.10 to 0.22)	2011-2015	-0.34 (-0.63 to -0.21)	2010-2018	-0.09 (-0.12 to -0.06)	2014-2017	-1.33 (-1.64 to -0.91)	2015-2021	-1.30 (-1.39 to -1.22)
	6					2015-2021	-1.70 (-1.79 to -1.61)	2018-2021	0.14 (0.08 to 0.25)	2017-2021	-2.11 (-2.37 to -1.97)		
Region of the Americas	0	1990-1993	0.56 (0.46 to 0.72)	1990-1994	0.72 (0.67 to 0.77)	1990-1995	1.01 (0.60 to 1.48)	1990-1994	0.84 (0.77 to 0.91)	1990-1996	0.48 (0.16 to 1.12)	1990-1995	0.65 (0.45 to 0.95)
	1	1993-2002	0.27 (0.21 to 0.29)	1994-2010	0.19 (0.18 to 0.20)	1995-2003	-0.98 (-1.18 to -0.79)	1994-2010	0.25 (0.24 to 0.27)	1996-2003	-0.60 (-1.01 to -0.30)	1995-2002	-0.17 (-0.36 to -0.01)
	2	2002-2010	0.38 (0.35 to 0.42)	2010-2015	0.57 (0.52 to 0.65)	2003-2013	-2.50 (-2.73 to -2.35)	2010-2015	0.68 (0.32 to 0.80)	2003-2013	-2.01 (-2.36 to -1.83)	2002-2013	-0.93 (-1.03 to -0.86)
	3	2010-2015	0.81 (0.75 to 0.89)	2015-2021	0.19 (0.15 to 0.23)	2013-2017	1.17 (0.61 to 2.28)	2015-2019	0.26 (0.20 to 0.63)	2013-2017	1.78 (1.03 to 3.00)	2013-2017	1.26 (0.93 to 1.74)
	4	2015-2019	0.47 (0.40 to 0.66)			2017-2021	-1.01 (-1.91 to -0.51)	2019-2021	-0.11 (-0.27 to 0.10)	2017-2021	-0.98 (-2.21 to -0.31)	2017-2021	-0.55 (-0.93 to -0.22)
	5	2019-2021	0.09 (-0.03 to 0.26)										
South-East Asia Region	0	1990-1994	0.16 (0.12 to 0.21)	1990-1997	0.10 (0.06 to 0.12)	1990-1996	0.15 (-0.45 to 1.03)	1990-1992	1.21 (1.06 to 1.37)	1990-1998	-0.12 (-0.58 to 0.55)	1990-1996	0.18 (-0.29 to 0.91)
	1	1994-2006	0.05 (0.04 to 0.06)	1997-2005	0.18 (0.15 to 0.20)	1996-2011	-1.34 (-1.66 to -1.19)	1992-1995	0.49 (0.40 to 0.57)	1998-2021	-1.52 (-1.59 to -1.45)	1996-2021	-1.03 (-1.08 to -0.99)
	2	2006-2009	0.34 (0.27 to 0.37)	2005-2010	0.42 (0.37 to 0.46)	2011-2019	-0.80 (-1.16 to -0.11)	1995-2000	-0.63 (-0.68 to -0.59)				
	3	2009-2015	0.12 (0.09 to 0.14)	2010-2016	0.20 (0.16 to 0.24)	2019-2021	-2.34 (-3.28 to -1.07)	2000-2005	0.07 (0.02 to 0.11)				
	4	2015-2018	0.46 (0.41 to 0.50)	2016-2019	0.36 (0.27 to 0.39)			2005-2010	0.82 (0.77 to 0.90)				
	5	2018-2021	0.28 (0.20 to 0.32)	2019-2021	0.15 (0.08 to 0.26)			2010-2014	0.59 (0.43 to 0.65)				
	6							2014-2021	0.05 (0.01 to 0.08)				
European Region	0	1990-1994	0.66 (0.15 to 0.94)	1990-1994	0.88 (0.13 to 1.19)	1990-1994	2.48 (1.84 to 3.19)	1990-1995	0.91 (0.47 to 1.11)	1990-1994	3.50 (2.48 to 4.37)	1990-1994	2.64 (1.94 to 3.12)
	1	1994-2021	1.51 (1.49 to 1.53)	1994-2021	1.58 (1.56 to 1.61)	1994-2003	-3.49 (-3.69 to -3.18)	1995-1999	2.08 (1.79 to 2.45)	1994-2002	-3.03 (-3.30 to -2.68)	1994-2010	-1.40 (-1.52 to -1.31)
	2					2003-2011	-4.37 (-5.21 to -4.09)	1999-2021	1.58 (1.55 to 1.60)	2002-2011	-4.39 (-4.78 to -4.16)	2010-2021	0.29 (0.03 to 0.57)
	3					2011-2021	-1.46 (-1.77 to -1.15)			2011-2017	-0.44 (-0.88 to 0.55)		
	4									2017-2021	-3.07 (-4.72 to -2.21)		
Eastern Mediterranean Region	0	1990-1993	-0.01 (-0.12 to 0.06)	1990-1995	0.09 (0.05 to 0.17)	1990-1992	1.20 (0.79 to 1.74)	1990-2000	-0.07 (-0.09 to -0.05)	1990-1992	0.94 (0.50 to 1.48)	1990-1992	0.63 (0.30 to 1.05)
	1	1993-2001	0.15 (0.13 to 0.18)	1995-2001	0.02 (-0.03 to 0.35)	1992-1996	2.53 (2.35 to 2.84)	2000-2010	0.49 (0.47 to 0.51)	1992-1996	2.23 (2.02 to 2.55)	1992-1996	1.59 (1.44 to 1.82)
	2	2001-2009	0.34 (0.33 to 0.39)	2001-2007	0.42 (0.35 to 0.46)	1996-2001	0.10 (-0.17 to 0.26)	2010-2015	0.30 (0.23 to 0.35)	1996-2001	-0.06 (-0.32 to 0.13)	1996-2001	-0.04 (-0.22 to 0.11)
	3	2009-2015	0.27 (0.23 to 0.30)	2007-2015	0.50 (0.47 to 0.53)	2001-2005	0.96 (0.74 to 1.25)	2015-2018	1.16 (1.06 to 1.23)	2001-2005	1.17 (0.89 to 1.50)	2001-2005	1.00 (0.78 to 1.24)
	4	2015-2021	0.74 (0.72 to 0.76)	2015-2018	1.08 (1.01 to 1.12)	2005-2012	-1.07 (-1.24 to -0.99)	2018-2021	0.80 (0.66 to 0.87)	2005-2012	-0.97 (-1.16 to -0.87)	2005-2012	-0.58 (-0.74 to -0.51)
	5			2018-2021	0.91 (0.81 to 0.97)	2012-2015	-0.33 (-0.70 to -0.16)			2012-2015	-0.12 (-0.57 to 0.08)	2012-2015	-0.01 (-0.33 to 0.15)
	6					2015-2021	-1.56 (-1.70 to -1.46)			2015-2021	-1.67 (-1.84 to -1.54)	2015-2021	-0.88 (-1.02 to -0.78)
Western Pacific Region	0	1990-1997	-0.01 (-0.25 to 0.18)	1990-2000	0.04 (-0.05 to 0.13)	1990-1997	-1.92 (-2.23 to -1.78)	1990-1995	-0.21 (-0.74 to 0.01)	1990-1997	-2.17 (-2.84 to -1.91)	1990-1997	-1.73 (-1.92 to -1.61)
	1	1997-2010	1.44 (1.37 to 1.54)	2000-2010	1.22 (1.12 to 1.33)	1997-2000	-1.06 (-1.51 to -0.82)	1995-2001	0.50 (0.26 to 0.92)	1997-2000	-1.33 (-2.46 to -1.02)	1997-2000	-0.94 (-1.44 to -0.75)
	2	2010-2021	0.25 (0.13 to 0.37)	2010-2021	0.42 (0.33 to 0.50)	2000-2004	-2.71 (-2.98 to -2.52)	2001-2010	1.30 (1.20 to 1.52)	2000-2004	-2.56 (-6.01 to -2.31)	2000-2004	-1.67 (-1.94 to -1.39)
	3					2004-2007	-6.30 (-6.50 to -6.07)	2010-2016	0.21 (-0.24 to 0.39)	2004-2007	-6.05 (-6.32 to -3.99)	2004-2007	-4.22 (-4.43 to -2.07)
	4					2007-2014	-4.15 (-4.24 to -4.06)	2016-2021	0.89 (0.67 to 1.45)	2007-2014	-3.99 (-4.13 to -1.88)	2007-2010	-2.13 (-2.78 to -1.92)
	5					2014-2021	-1.37 (-1.49 to -1.27)			2014-2021	-1.36 (-1.58 to -1.10)	2010-2014	-2.71 (-2.96 to -0.84)
	6											2014-2021	-0.53 (-0.65 to -0.38)
**Seven super regions**													
Southeast Asia, East Asia, and Oceania	0	1990-1999	0.13 (0.01 to 0.24)	1990-2000	-0.04 (-0.14 to 0.07)	1990-2000	-0.64 (-0.71 to -0.55)	1990-2000	-0.01 (-0.14 to 0.10)	1990-2001	-0.98 (-1.05 to -0.90)	1990-2001	-0.80 (-0.85 to -0.70)
	1	1999-2013	1.05 (0.99 to 1.13)	2000-2012	1.03 (0.95 to 1.16)	2000-2004	-2.43 (-2.62 to -2.23)	2000-2008	1.14 (0.96 to 1.67)	2001-2004	-2.74 (-2.99 to -2.30)	2001-2004	-1.92 (-2.16 to -1.47)
	2	2013-2021	0.14 (-0.04 to 0.28)	2012-2021	0.50 (0.27 to 0.62)	2004-2007	-5.58 (-5.78 to -5.33)	2008-2021	0.67 (0.52 to 0.75)	2004-2007	-5.26 (-5.48 to -4.95)	2004-2007	-3.87 (-4.10 to -3.50)
	3					2007-2013	-3.80 (-3.89 to -3.69)			2007-2013	-3.54 (-3.63 to -3.42)	2007-2013	-2.41 (-2.52 to -2.22)
	4					2013-2021	-1.63 (-1.72 to -1.55)			2013-2021	-1.52 (-1.61 to -1.45)	2013-2021	-0.83 (-0.92 to -0.72)
Central Europe, Eastern Europe, and Central Asia	0	1990-1993	0.10 (-0.13 to 0.28)	1990-1996	0.29 (0.18 to 0.37)	1990-1994	6.16 (5.43 to 7.02)	1990-1996	0.32 (0.24 to 0.39)	1990-1994	7.59 (6.84 to 8.50)	1990-1994	5.99 (5.30 to 6.80)
	1	1993-1996	0.42 (0.31 to 1.71)	1996-1999	1.91 (0.37 to 2.03)	1994-2005	-3.13 (-3.31 to -2.88)	1996-1999	1.92 (0.44 to 2.03)	1994-2003	-2.73 (-3.00 to -2.40)	1994-2011	-2.04 (-2.21 to -1.92)
	2	1996-1999	1.62 (0.72 to 1.72)	1999-2003	0.69 (0.54 to 1.84)	2005-2011	-4.41 (-5.97 to -3.85)	1999-2003	0.71 (0.57 to 1.82)	2003-2011	-4.23 (-5.08 to -3.78)	2011-2021	0.70 (0.22 to 1.27)
	3	1999-2006	0.75 (0.70 to 1.70)	2003-2006	0.93 (0.79 to 2.28)	2011-2021	-0.58 (-1.04 to -0.12)	2003-2006	0.95 (0.82 to 2.32)	2011-2017	0.68 (-0.35 to 2.53)		
	4	2006-2012	1.75 (1.61 to 1.87)	2006-2015	2.13 (1.96 to 2.18)			2006-2015	2.18 (2.06 to 2.22)	2017-2021	-2.69 (-7.04 to -0.79)		
	5	2012-2019	2.03 (1.97 to 2.19)	2015-2019	2.45 (2.34 to 2.62)			2015-2019	2.51 (2.40 to 2.67)				
	6	2019-2021	1.68 (1.46 to 1.93)	2019-2021	1.79 (1.53 to 2.05)			2019-2021	1.63 (1.39 to 1.92)				
South Asia	0	1990-1999	0.29 (0.25 to 0.38)	1990-2006	0.26 (0.19 to 0.29)	1990-1995	0.35 (-0.61 to 3.51)	1990-1992	1.63 (1.35 to 1.91)	1990-1996	0.66 (-0.05 to 1.94)	1990-1995	0.65 (-0.00 to 1.92)
	1	1999-2004	0.14 (-0.01 to 0.29)	2006-2016	0.37 (0.29 to 0.45)	1995-2021	-0.72 (-1.32 to -0.64)	1992-1995	0.68 (0.36 to 0.83)	1996-2021	-0.99 (-1.06 to -0.92)	1995-2021	-0.65 (-0.71 to -0.60)
	2	2004-2015	0.34 (0.31 to 0.44)	2016-2021	0.61 (0.48 to 0.90)			1995-2000	-0.78 (-0.93 to -0.68)				
	3	2015-2021	0.80 (0.73 to 0.93)					2000-2005	0.01 (-0.11 to 0.16)				
	4							2005-2013	0.94 (0.88 to 1.00)				

Location	Segment	Incidence		Prevalence		Mortality		YLD		YLL		DALY	
		Period	APC (95%CI)	Period	APC (95%CI)	Period	APC (95%CI)	Period	APC (95%CI)	Period	APC (95%CI)	Period	APC (95%CI)
	5							2013-2021	0.30 (0.24 to 0.36)				
High-income	0	1990-2003	1.20 (1.16 to 1.25)	1990-2000	1.27 (1.20 to 1.38)	1990-2003	-1.60 (-1.70 to -1.49)	1990-2000	1.42 (1.35 to 1.53)	1990-1997	-1.57 (-2.14 to -1.27)	1990-1997	-0.04 (-0.27 to 0.08)
	1	2003-2011	0.90 (0.76 to 1.25)	2000-2021	1.02 (0.99 to 1.04)	2003-2014	-3.74 (-3.86 to -3.61)	2000-2021	1.05 (1.02 to 1.08)	1997-2003	-0.50 (-0.78 to -0.09)	1997-2002	0.63 (0.37 to 1.00)
	2	2011-2016	1.39 (0.87 to 1.67)			2014-2017	1.32 (0.62 to 1.75)			2003-2014	-3.45 (-3.56 to -3.35)	2002-2013	-0.66 (-0.74 to -0.58)
	3	2016-2019	0.86 (0.66 to 1.43)			2017-2021	-2.10 (-2.67 to -1.71)			2014-2017	2.46 (2.01 to 2.89)	2013-2017	1.09 (0.76 to 1.64)
	4	2019-2021	1.55 (1.02 to 1.87)							2017-2021	-2.15 (-2.67 to -1.72)	2017-2021	0.08 (-0.43 to 0.36)
Latin America and Caribbean	0	1990-1995	-0.12 (-0.49 to 0.04)	1990-1996	-0.04 (-0.40 to 0.10)	1990-1995	0.35 (-0.19 to 0.85)	1990-1996	-0.05 (-0.43 to 0.10)	1990-1996	0.07 (-0.30 to 0.53)	1990-1996	0.03 (-0.18 to 0.29)
	1	1995-2001	0.25 (0.10 to 0.57)	1996-2001	0.36 (0.11 to 0.64)	1995-2002	-2.72 (-3.09 to -2.45)	1996-2001	0.32 (0.10 to 0.69)	1996-2002	-2.19 (-2.98 to -1.83)	1996-2002	-1.38 (-1.84 to -1.14)
	2	2001-2014	0.59 (0.39 to 0.67)	2001-2013	0.63 (-0.29 to 0.79)	2002-2012	-1.07 (-1.34 to -0.82)	2001-2014	0.68 (-0.34 to 0.82)	2002-2012	-0.68 (-0.95 to -0.38)	2002-2012	-0.16 (-0.33 to 0.01)
	3	2014-2021	-0.18 (-0.31 to -0.07)	2013-2021	-0.21 (-0.36 to -0.07)	2012-2019	0.92 (0.66 to 1.54)	2014-2021	-0.26 (-0.45 to -0.06)	2012-2019	1.26 (1.00 to 1.92)	2012-2019	0.82 (0.64 to 1.33)
	4					2019-2021	-1.47 (-2.68 to -0.04)			2019-2021	-1.85 (-3.22 to -0.29)	2019-2021	-1.46 (-2.25 to -0.45)
North Africa and Middle East	0	1990-1992	-0.39 (-0.47 to -0.25)	1990-2001	-0.17 (-0.19 to -0.16)	1990-2002	-1.53 (-1.70 to -1.39)	1990-1998	-0.26 (-0.32 to -0.23)	1990-2002	-1.48 (-1.63 to -1.36)	1990-1992	-2.08 (-2.60 to -1.01)
	1	1992-2000	-0.14 (-0.16 to -0.11)	2001-2004	0.73 (0.64 to 0.78)	2002-2005	1.89 (0.94 to 2.31)	1998-2001	-0.02 (-0.14 to 0.04)	2002-2005	2.01 (1.02 to 2.42)	1992-2002	-0.94 (-1.05 to 1.84)
	2	2000-2009	0.50 (0.48 to 0.51)	2004-2015	0.51 (0.49 to 0.53)	2005-2015	-0.95 (-1.09 to -0.81)	2001-2004	1.11 (1.00 to 1.20)	2005-2015	-1.13 (-1.28 to -0.96)	2002-2005	1.74 (-0.82 to 1.99)
	3	2009-2015	0.26 (0.22 to 0.29)	2015-2021	1.01 (0.97 to 1.05)	2015-2021	-2.12 (-2.47 to -1.88)	2004-2009	0.58 (0.52 to 0.63)	2015-2021	-2.19 (-2.58 to -1.92)	2005-2016	-0.62 (-0.70 to -0.39)
	4	2015-2021	0.69 (0.66 to 0.72)					2009-2015	0.24 (0.19 to 0.28)			2016-2021	-1.08 (-1.52 to -0.86)
	5							2015-2021	0.80 (0.75 to 0.84)				
Sub-Saharan Africa	0	1990-1994	-0.41 (-0.47 to -0.38)	1990-1992	-0.53 (-0.61 to -0.45)	1990-1994	-1.10 (-1.29 to -0.89)	1990-1993	-0.46 (-0.57 to -0.41)	1990-1994	-0.96 (-1.17 to -0.70)	1990-1994	-0.83 (-0.99 to -0.59)
	1	1994-1999	-0.05 (-0.12 to -0.02)	1992-1995	-0.29 (-0.33 to -0.12)	1994-1998	-2.11 (-2.35 to -1.90)	1993-1996	-0.15 (-0.21 to -0.01)	1994-1999	-1.92 (-2.18 to -1.77)	1994-1999	-1.40 (-1.66 to -1.29)
	2	1999-2006	0.03 (-0.01 to 0.06)	1995-2006	0.10 (0.09 to 0.11)	1998-2001	-1.44 (-1.80 to -0.59)	1996-2006	0.13 (0.12 to 0.15)	1999-2004	-0.67 (-0.83 to -0.47)	1999-2004	-0.45 (-0.58 to -0.26)
	3	2006-2010	0.10 (0.01 to 0.14)	2006-2010	0.25 (0.22 to 0.30)	2001-2004	-0.50 (-1.51 to -0.34)	2006-2010	0.28 (0.25 to 0.34)	2004-2011	-1.56 (-1.66 to -1.48)	2004-2011	-1.04 (-1.15 to -0.96)
	4	2010-2016	-0.16 (-0.21 to -0.12)	2010-2019	-0.24 (-0.26 to -0.23)	2004-2011	-1.48 (-1.58 to -0.34)	2010-2018	-0.17 (-0.19 to -0.15)	2011-2014	-0.11 (-0.36 to 0.05)	2011-2015	-0.24 (-0.42 to -0.03)
	5	2016-2019	-0.00 (-0.15 to 0.03)	2019-2021	0.25 (0.17 to 0.31)	2011-2015	-0.31 (-1.57 to -0.16)	2018-2021	0.05 (-0.01 to 0.17)	2014-2017	-1.30 (-1.61 to -1.12)	2015-2021	-1.32 (-1.41 to -1.25)
	6	2019-2021	0.31 (0.19 to 0.39)			2015-2021	-1.68 (-1.78 to -1.59)			2017-2021	-2.09 (-2.32 to -1.97)		
**21 regions**													
Andean Latin America	0	1990-1995	-0.06 (-0.35 to 0.08)	1990-1996	-0.05 (-0.27 to 0.10)	1990-1993	2.52 (0.07 to 6.05)	1990-1996	-0.07 (-0.32 to 0.09)	1990-1994	1.71 (1.00 to 2.63)	1990-1994	1.25 (0.88 to 1.87)
	1	1995-2002	0.37 (0.21 to 0.49)	1996-2021	0.56 (0.54 to 0.58)	1993-2009	-0.53 (-0.95 to -0.31)	1996-2021	0.59 (0.56 to 0.61)	1994-1998	-1.59 (-2.57 to -0.87)	1994-1997	-1.37 (-1.83 to -0.64)
	2	2002-2015	0.56 (0.53 to 0.79)			2009-2021	-1.77 (-2.38 to -1.45)			1998-2002	0.49 (-0.10 to 1.40)	1997-2002	0.33 (0.05 to 1.05)
	3	2015-2021	0.29 (-0.02 to 0.42)							2002-2012	-1.17 (-1.36 to -0.96)	2002-2012	-0.58 (-0.71 to -0.42)
	4									2012-2015	-3.33 (-3.88 to -2.45)	2012-2015	-1.90 (-2.30 to -1.30)
	5									2015-2019	-0.53 (-1.05 to 0.51)	2015-2019	-0.13 (-0.46 to 0.60)
	6									2019-2021	-5.75 (-7.35 to -3.82)	2019-2021	-3.37 (-4.42 to -2.14)
Australasia	0	1990-1994	1.96 (1.68 to 2.10)	1990-1996	2.12 (1.92 to 2.22)	1990-1995	0.29 (-0.51 to 1.58)	1990-1995	1.35 (0.99 to 1.57)	1990-1997	-0.28 (-0.91 to 0.47)	1990-1992	1.47 (0.82 to 1.92)
	1	1994-2003	2.26 (2.20 to 2.38)	1996-2004	2.41 (2.35 to 2.53)	1995-1998	-4.73 (-9.38 to -2.20)	1995-2000	2.42 (2.17 to 2.88)	1997-2001	-9.54 (-11.54 to -7.62)	1992-1998	0.60 (-1.78 to 0.70)
	2	2003-2007	1.87 (1.77 to 2.30)	2004-2010	1.44 (1.35 to 1.53)	1998-2001	-11.85 (-12.81 to -2.98)	2000-2006	1.08 (0.88 to 1.24)	2001-2015	-2.31 (-3.24 to -1.99)	1998-2001	-1.90 (-2.14 to 0.05)
	3	2007-2010	1.31 (1.17 to 1.88)	2010-2015	-1.03 (-1.16 to -0.92)	2001-2015	-2.47 (-2.93 to -2.21)	2006-2009	3.84 (3.57 to 4.12)	2015-2021	-0.46 (-1.54 to 2.15)	2001-2005	0.19 (-0.23 to 1.69)
	4	2010-2015	-0.87 (-1.03 to 1.33)	2015-2021	0.77 (0.62 to 0.90)	2015-2021	-0.61 (-1.42 to 1.29)	2009-2016	-0.58 (-0.74 to -0.46)			2005-2010	1.78 (-0.17 to 2.12)
	5	2015-2019	0.73 (-0.91 to 0.85)					2016-2021	0.99 (0.75 to 1.31)			2010-2015	-1.40 (-1.70 to -1.05)
	6	2019-2021	1.42 (0.85 to 1.75)									2015-2021	0.69 (0.48 to 0.97)
Caribbean	0	1990-1999	-0.28 (-0.40 to -0.18)	1990-1999	-0.51 (-0.63 to -0.41)	1990-1995	-0.27 (-0.65 to 0.17)	1990-1999	-0.55 (-0.68 to -0.44)	1990-1995	-0.28 (-0.65 to 0.12)	1990-1995	-0.38 (-0.68 to -0.02)
	1	1999-2011	0.34 (0.27 to 0.43)	1999-2010	0.36 (0.28 to 0.47)	1995-2002	-2.62 (-2.90 to -2.40)	1999-2010	0.35 (0.27 to 0.46)	1995-2002	-2.17 (-2.52 to -1.94)	1995-2002	-1.75 (-2.06 to -1.56)
	2	2011-2021	-0.43 (-0.54 to -0.34)	2010-2021	-0.62 (-0.73 to -0.54)	2002-2005	-0.13 (-1.09 to 0.29)	2010-2021	-0.64 (-0.74 to -0.55)	2002-2005	0.34 (-0.49 to 0.80)	2002-2005	0.41 (-0.26 to 0.78)
	3					2005-2010	-1.64 (-2.46 to -1.08)			2005-2010	-1.39 (-2.34 to -1.01)	2005-2010	-1.00 (-1.73 to -0.70)
	4					2010-2021	0.15 (-0.00 to 0.31)			2010-2019	0.77 (0.61 to 1.09)	2010-2019	0.45 (0.33 to 0.71)
	5									2019-2021	-1.69 (-2.77 to -0.41)	2019-2021	-1.48 (-2.34 to -0.47)
Central Asia	0	1990-2006	0.45 (0.44 to 0.46)	1990-2001	0.44 (0.40 to 0.46)	1990-1995	9.44 (8.94 to 9.99)	1990-2003	0.47 (0.44 to 0.49)	1990-1994	11.83 (11.03 to 12.92)	1990-1994	9.61 (8.95 to 10.52)
	1	2006-2011	1.11 (1.08 to 1.15)	2001-2005	0.65 (0.43 to 0.74)	1995-2016	-1.72 (-1.80 to -1.63)	2003-2007	0.90 (0.47 to 1.07)	1994-1997	0.07 (-1.11 to 1.01)	1994-1997	-0.01 (-0.94 to 0.71)
	2	2011-2018	1.97 (1.94 to 2.01)	2005-2008	1.09 (0.65 to 1.16)	2016-2021	-4.36 (-5.71 to -3.50)	2007-2011	1.54 (0.91 to 1.67)	1997-2011	-1.77 (-2.77 to -1.66)	1997-2011	-1.26 (-2.13 to -1.17)
	3	2018-2021	1.67 (1.48 to 1.79)	2008-2011	1.54 (1.19 to 1.63)			2011-2015	2.29 (1.52 to 2.36)	2011-2015	-0.52 (-1.46 to 0.59)	2011-2015	0.27 (-0.69 to 1.23)
	4			2011-2015	2.25 (1.97 to 2.30)			2015-2018	2.59 (2.40 to 2.69)	2015-2021	-4.12 (-5.21 to -3.49)	2015-2021	-2.38 (-3.30 to -1.87)
	5			2015-2018	2.47 (2.36 to 2.53)			2018-2021	1.83 (1.66 to 1.94)				
	6			2018-2021	1.82 (1.73 to 1.89)								
Central Europe	0	1990-1994	0.41 (0.34 to 0.51)	1990-1994	0.54 (0.45 to 0.63)	1990-1992	-1.31 (-2.35 to -0.49)	1990-1994	0.63 (0.53 to 0.73)	1990-1992	-1.45 (-2.76 to -0.51)	1990-1992	-0.96 (-1.88 to -0.29)
	1	1994-2000	0.09 (0.01 to 0.13)	1994-2000	0.13 (0.06 to 0.17)	1992-1996	-4.03 (-4.68 to -3.67)	1994-2000	0.15 (0.06 to 0.21)	1992-1996	-3.84 (-6.19 to -3.40)	1992-1996	-2.81 (-4.00 to -2.50)
	2	2000-2003	0.64 (0.36 to 0.73)	2000-2003	0.69 (0.43 to 0.77)	1996-2001	-6.55 (-7.11 to -6.17)	2000-2003	0.75 (0.40 to 0.89)	1996-2001	-6.50 (-7.29 to -3.71)	1996-2001	-4.57 (-5.05 to -4.03)
	3	2003-2006	1.18 (1.10 to 1.53)	2003-2006	1.27 (1.19 to 1.39)	2001-2007	-3.05 (-3.35 to -2.40)	2003-2006	1.28 (1.18 to 1.98)	2001-2007	-2.95 (-3.47 to -2.08)	2001-2012	-1.43 (-1.64 to -1.25)
	4	2006-2014	1.91 (1.88 to 1.94)	2006-2014	2.00 (1.97 to 2.03)	2007-2014	-4.34 (-5.37 to -4.00)	2006-2014	2.10 (2.07 to 2.15)	2007-2013	-4.10 (-5.20 to -3.52)	2012-2021	-0.46 (-0.74 to 0.00)
	5	2014-2019	1.33 (1.29 to 1.39)	2014-2019	1.56 (1.50 to 1.64)	2014-2021	-2.46 (-2.94 to -1.58)	2014-2019	1.66 (1.59 to 1.74)	2013-2021	-2.50 (-2.97 to -1.65)		
	6	2019-2021	0.52 (0.35 to 0.75)	2019-2021	0.81 (0.64 to 1.05)			2019-2021	0.52 (0.33 to 0.70)				
Central Latin America	0	1990-2000	0.02 (-0.09 to 0.10)	1990-2011	0.25 (0.22 to 0.29)	1990-1996	0.18 (-0.33 to 0.99)	1990-1999	0.12 (-0.06 to 0.20)	1990-1995	0.25 (-0.75 to 2.64)	1990-1995	0.18 (-0.50 to 1.59)
	1	2000-2010	0.35 (0.28 to 0.47)	2011-2016	-2.06 (-2.39 to -1.81)	1996-1999	-4.87 (-5.55 to -3.14)	1999-2010	0.38 (0.32 to 0.50)	1995-2008	-1.72 (-2.21 to -1.49)	1995-2007	-1.21 (-1.95 to -1.00)
	2	2010-2017	-1.18 (-1.34 to -1.07)	2016-2021	-0.30 (-0.60 to 0.09)	1999-2011	-1.80 (-2.07 to -1.42)	2010-2017	-1.55 (-1.68 to -1.45)	2008-2021	0.45 (0.08 to 0.87)	2007-2021	-0.01 (-0.21 to 0.24)

Location	Segment	Incidence		Prevalence		Mortality		YLD		YLL		DALY	
		Period	APC (95%CI)	Period	APC (95%CI)	Period	APC (95%CI)	Period	APC (95%CI)	Period	APC (95%CI)	Period	APC (95%CI)
	3	2017-2021	-0.31 (-0.55 to 0.05)			2011-2021	0.56 (0.04 to 1.23)	2017-2021	-0.26 (-0.56 to 0.13)				
Central Sub-Saharan Africa	0	1990-1993	-0.53 (-0.63 to -0.46)	1990-1993	-0.55 (-0.65 to -0.50)	1990-1997	-0.99 (-1.26 to -0.77)	1990-1994	-0.55 (-0.78 to -0.42)	1990-1997	-1.23 (-1.54 to -1.00)	1990-1997	-1.04 (-1.25 to -0.87)
	1	1993-1997	-0.32 (-0.40 to -0.23)	1993-1998	-0.30 (-0.34 to -0.26)	1997-2003	0.69 (0.34 to 1.07)	1994-2001	-0.15 (-0.23 to -0.05)	1997-2003	0.84 (0.53 to 1.28)	1997-2003	0.65 (0.43 to 0.97)
	2	1997-2000	-0.19 (-0.23 to 0.19)	1998-2001	-0.04 (-0.16 to 0.09)	2003-2009	-1.05 (-1.59 to -0.73)	2001-2008	0.30 (0.19 to 0.39)	2003-2009	-1.17 (-1.76 to -0.89)	2003-2009	-0.81 (-1.27 to -0.60)
	3	2000-2005	0.17 (0.13 to 0.34)	2001-2006	0.20 (0.16 to 0.24)	2009-2014	-0.38 (-0.69 to 0.16)	2008-2019	0.55 (0.53 to 0.65)	2009-2015	-0.38 (-0.63 to 0.25)	2009-2015	-0.14 (-0.34 to 0.35)
	4	2005-2011	0.34 (0.29 to 0.43)	2006-2011	0.37 (0.33 to 0.42)	2014-2021	-1.14 (-1.53 to -0.98)	2019-2021	0.19 (-0.02 to 0.46)	2015-2021	-1.42 (-1.82 to -1.19)	2015-2021	-0.91 (-1.24 to -0.74)
	5	2011-2021	0.42 (0.41 to 0.45)	2011-2018	0.49 (0.48 to 0.54)								
	6			2018-2021	0.39 (0.31 to 0.44)								
East Asia	0	1990-1999	-0.38 (-0.52 to -0.23)	1990-2000	-0.29 (-0.42 to -0.17)	1990-2000	-1.05 (-1.20 to -0.86)	1990-1999	-0.48 (-0.70 to -0.30)	1990-2001	-1.65 (-1.74 to -1.52)	1990-2001	-1.47 (-1.57 to -1.26)
	1	1999-2012	1.29 (1.21 to 1.43)	2000-2010	1.51 (1.37 to 1.68)	2000-2004	-2.98 (-3.47 to -2.55)	1999-2009	1.69 (1.52 to 1.92)	2001-2004	-3.33 (-3.66 to -2.70)	2001-2004	-2.48 (-5.22 to -1.89)
	2	2012-2021	0.74 (0.42 to 0.90)	2010-2021	0.62 (0.47 to 0.73)	2004-2007	-7.71 (-8.05 to -7.16)	2009-2016	0.34 (-0.38 to 0.61)	2004-2007	-7.82 (-8.20 to -7.36)	2004-2007	-6.14 (-6.52 to -4.42)
	3					2007-2014	-5.43 (-5.59 to -5.19)	2016-2021	1.52 (1.08 to 2.42)	2007-2010	-4.76 (-5.18 to -4.40)	2007-2015	-3.85 (-4.02 to -3.58)
	4					2014-2021	-2.47 (-2.71 to -2.20)			2010-2013	-6.23 (-6.56 to -5.75)	2015-2021	-1.06 (-1.37 to -0.58)
	5									2013-2018	-3.19 (-3.73 to -2.92)		
	6									2018-2021	-1.58 (-2.34 to -0.43)		
Eastern Europe	0	1990-1993	-0.33 (-0.73 to -0.03)	1990-1996	0.12 (0.01 to 0.24)	1990-1994	11.74 (7.47 to 15.60)	1990-1996	0.15 (0.03 to 0.27)	1990-1994	11.95 (7.97 to 15.91)	1990-1994	8.48 (7.21 to 9.80)
	1	1993-1996	0.34 (0.15 to 2.96)	1996-1999	3.47 (3.26 to 3.68)	1994-2003	-2.72 (-3.41 to -1.98)	1996-1999	3.43 (3.22 to 3.65)	1994-2003	-2.71 (-3.46 to -1.89)	1994-2001	-1.28 (-1.84 to -0.57)
	2	1996-1999	2.92 (0.73 to 3.09)	1999-2006	0.76 (0.66 to 0.85)	2003-2011	-7.26 (-8.39 to -6.69)	1999-2006	0.78 (0.67 to 0.87)	2003-2011	-7.24 (-8.74 to -6.55)	2001-2011	-3.83 (-4.70 to -3.37)
	3	1999-2006	0.70 (0.64 to 0.83)	2006-2015	2.33 (2.19 to 2.41)	2011-2021	2.20 (1.34 to 2.86)	2006-2015	2.36 (2.20 to 2.44)	2011-2021	2.78 (1.70 to 3.71)	2011-2021	2.37 (1.60 to 3.30)
	4	2006-2015	2.09 (2.00 to 2.15)	2015-2021	2.78 (2.62 to 3.07)			2015-2021	2.78 (2.63 to 3.12)				
	5	2015-2021	2.47 (2.36 to 2.68)										
Eastern Sub-Saharan Africa	0	1990-1995	-0.49 (-0.59 to -0.37)	1990-1994	-0.53 (-0.78 to -0.36)	1990-1995	-0.30 (-0.40 to -0.17)	1990-1994	-0.51 (-0.82 to -0.30)	1990-1995	-0.34 (-0.46 to -0.20)	1990-1995	-0.35 (-0.44 to -0.25)
	1	1995-2011	0.17 (0.15 to 0.18)	1994-2002	0.32 (0.26 to 0.48)	1995-1999	-1.67 (-1.77 to -1.46)	1994-2010	0.30 (0.27 to 0.33)	1995-1999	-1.84 (-1.95 to -1.20)	1995-1999	-1.32 (-1.41 to -0.93)
	2	2011-2018	-0.55 (-0.66 to -0.49)	2002-2011	0.15 (0.04 to 0.20)	1999-2002	-2.39 (-2.51 to -2.16)	2010-2021	-0.64 (-0.68 to -0.60)	1999-2002	-2.39 (-2.54 to -2.11)	1999-2002	-1.72 (-1.83 to -1.53)
	3	2018-2021	-0.20 (-0.42 to 0.09)	2011-2018	-0.90 (-1.03 to -0.84)	2002-2011	-1.81 (-1.87 to -1.74)			2002-2007	-1.52 (-1.63 to -1.30)	2002-2007	-1.04 (-1.11 to -0.88)
	4			2018-2021	-0.40 (-0.66 to -0.02)	2011-2016	-1.09 (-1.19 to -0.88)			2007-2011	-1.78 (-1.97 to -1.58)	2007-2011	-1.27 (-1.42 to -1.18)
	5					2016-2021	-1.65 (-1.78 to -1.55)			2011-2016	-1.00 (-1.15 to -0.86)	2011-2015	-0.82 (-0.97 to -0.67)
	6									2016-2021	-2.00 (-2.10 to -1.89)	2015-2021	-1.50 (-1.57 to -1.44)
High-income Asia Pacific	0	1990-2001	0.34 (0.28 to 0.38)	1990-1994	0.72 (0.57 to 0.94)	1990-1993	-5.13 (-5.68 to -4.51)	1990-2005	0.79 (0.73 to 0.83)	1990-1993	-5.06 (-5.87 to -4.08)	1990-1993	-2.20 (-2.43 to -1.93)
	1	2001-2013	0.84 (0.80 to 0.90)	1994-2001	0.20 (0.09 to 0.26)	1993-1996	-11.65 (-12.15 to -11.20)	2005-2011	1.44 (1.23 to 1.81)	1993-1996	-10.87 (-11.47 to -9.97)	1993-1996	-4.68 (-4.88 to -4.48)
	2	2013-2019	0.09 (-0.15 to 0.21)	2001-2010	1.05 (1.00 to 1.11)	1996-2002	-4.98 (-5.22 to -4.51)	2011-2021	0.25 (0.16 to 0.34)	1996-2007	-5.31 (-5.73 to -5.09)	1996-2006	-1.15 (-1.23 to -1.09)
	3	2019-2021	1.12 (0.59 to 1.43)	2010-2015	0.46 (0.35 to 0.62)	2002-2006	-6.22 (-6.94 to -5.65)			2007-2016	-4.58 (-4.94 to -3.00)	2006-2009	0.54 (0.09 to 0.78)
	4			2015-2019	-0.13 (-0.37 to 0.02)	2006-2016	-4.35 (-4.59 to -4.06)			2016-2021	-0.07 (-0.97 to 1.22)	2009-2016	-0.49 (-0.92 to -0.37)
	5			2019-2021	1.08 (0.69 to 1.37)	2016-2021	-0.16 (-0.81 to 0.74)					2016-2021	0.11 (-0.13 to 0.65)
High-income North America	0	1990-1994	1.43 (1.36 to 1.51)	1990-1994	1.59 (1.49 to 1.69)	1990-2002	1.13 (1.02 to 1.27)	1990-1994	1.80 (1.72 to 1.89)	1990-1998	1.20 (0.22 to 1.50)	1990-2002	1.20 (1.13 to 1.27)
	1	1994-2000	0.58 (0.53 to 0.64)	1994-1999	0.65 (0.57 to 0.76)	2002-2006	-2.62 (-3.04 to -1.68)	1994-1999	0.64 (0.57 to 0.73)	1998-2002	2.16 (-1.66 to 2.86)	2002-2006	-1.01 (-1.25 to -0.08)
	2	2000-2008	0.25 (0.14 to 0.32)	1999-2011	0.30 (0.28 to 0.32)	2006-2013	-4.54 (-4.81 to -4.31)	1999-2011	0.33 (0.31 to 0.35)	2002-2006	-2.28 (-3.57 to -1.40)	2006-2013	-1.72 (-1.97 to -1.58)
	3	2008-2011	0.39 (0.29 to 1.38)	2011-2016	1.07 (1.01 to 1.15)	2013-2017	1.09 (0.62 to 1.61)	2011-2017	1.08 (1.02 to 1.13)	2006-2014	-3.82 (-4.17 to -3.57)	2013-2017	1.62 (1.27 to 1.99)
	4	2011-2017	1.28 (1.20 to 1.37)	2016-2021	0.74 (0.63 to 0.82)	2017-2021	-1.97 (-2.64 to -1.38)	2017-2021	0.62 (0.49 to 0.73)	2014-2017	3.81 (3.04 to 4.49)	2017-2021	-0.52 (-0.96 to -0.14)
	5	2017-2021	0.82 (0.70 to 0.92)							2017-2021	-2.39 (-3.23 to -1.74)		
North Africa and Middle East	0	1990-1992	-0.39 (-0.47 to -0.25)	1990-2001	-0.17 (-0.19 to -0.16)	1990-2002	-1.53 (-1.70 to -1.39)	1990-1998	-0.26 (-0.32 to -0.23)	1990-2002	-1.48 (-1.63 to -1.36)	1990-1992	-2.08 (-2.60 to -1.01)
	1	1992-2000	-0.14 (-0.16 to -0.11)	2001-2004	0.73 (0.64 to 0.78)	2002-2005	1.89 (0.94 to 2.31)	1998-2001	-0.02 (-0.14 to 0.04)	2002-2005	2.01 (1.02 to 2.42)	1992-2002	-0.94 (-1.05 to 1.84)
	2	2000-2009	0.50 (0.48 to 0.51)	2004-2015	0.51 (0.49 to 0.53)	2005-2015	-0.95 (-1.09 to -0.81)	2001-2004	1.11 (1.00 to 1.20)	2005-2015	-1.13 (-1.28 to -0.96)	2002-2005	1.74 (-0.82 to 1.99)
	3	2009-2015	0.26 (0.22 to 0.29)	2015-2021	1.01 (0.97 to 1.05)	2015-2021	-2.12 (-2.47 to -1.88)	2004-2009	0.58 (0.52 to 0.63)	2015-2021	-2.19 (-2.58 to -1.92)	2005-2016	-0.62 (-0.70 to -0.39)
	4	2015-2021	0.69 (0.66 to 0.72)					2009-2015	0.24 (0.19 to 0.28)			2016-2021	-1.08 (-1.52 to -0.86)
	5							2015-2021	0.80 (0.75 to 0.84)				
Oceania	0	1990-1994	-0.20 (-0.23 to -0.17)	1990-2021	-0.18 (-0.19 to -0.17)	1990-2001	0.73 (0.46 to 1.17)	1990-2009	-0.13 (-0.15 to -0.10)	1990-2002	0.79 (0.50 to 1.27)	1990-2002	0.59 (0.36 to 0.96)
	1	1994-2000	-0.44 (-0.46 to -0.42)			2001-2021	-0.15 (-0.28 to -0.04)	2009-2014	-0.31 (-0.50 to -0.20)	2002-2021	-0.13 (-0.31 to 0.01)	2002-2021	-0.13 (-0.28 to -0.03)
	2	2000-2006	-0.25 (-0.28 to -0.23)					2014-2019	-0.01 (-0.10 to 0.23)				
	3	2006-2011	-0.11 (-0.15 to -0.08)					2019-2021	-0.72 (-1.03 to -0.38)				
	4	2011-2015	0.04 (-0.01 to 0.09)										
	5	2015-2019	0.22 (0.19 to 0.27)										
	6	2019-2021	-0.21 (-0.28 to -0.13)										
South Asia	0	1990-1999	0.29 (0.25 to 0.38)	1990-2006	0.26 (0.19 to 0.29)	1990-1995	0.35 (-0.61 to 3.51)	1990-1992	1.63 (1.35 to 1.91)	1990-1996	0.66 (-0.05 to 1.94)	1990-1995	0.65 (-0.00 to 1.92)
	1	1999-2004	0.14 (-0.01 to 0.29)	2006-2016	0.37 (0.29 to 0.45)	1995-2021	-0.72 (-1.32 to -0.64)	1992-1995	0.68 (0.36 to 0.83)	1996-2021	-0.99 (-1.06 to -0.92)	1995-2021	-0.65 (-0.71 to -0.60)
	2	2004-2015	0.34 (0.31 to 0.44)	2016-2021	0.61 (0.48 to 0.90)			1995-2000	-0.78 (-0.93 to -0.68)				
	3	2015-2021	0.80 (0.73 to 0.93)					2000-2005	0.01 (-0.11 to 0.16)				
	4							2005-2013	0.94 (0.88 to 1.00)				
	5							2013-2021	0.30 (0.24 to 0.36)				
Southeast Asia	0	1990-2001	-0.01 (-0.08 to 0.05)	1990-2001	-0.17 (-0.31 to -0.07)	1990-2000	-0.27 (-0.35 to -0.21)	1990-2002	-0.07 (-0.14 to -0.02)	1990-2000	-0.21 (-0.30 to -0.11)	1990-2000	-0.18 (-0.23 to -0.12)
	1	2001-2011	0.24 (0.17 to 0.51)	2001-2012	0.36 (0.27 to 0.59)	2000-2003	-1.62 (-1.85 to -0.27)	2002-2010	0.46 (0.34 to 0.81)	2000-2003	-1.61 (-1.92 to -0.22)	2000-2003	-1.12 (-1.33 to -0.63)
	2	2011-2019	-0.13 (-0.27 to 0.12)	2012-2021	-0.26 (-0.44 to -0.15)	2003-2009	-2.61 (-2.82 to -1.74)	2010-2019	-0.02 (-0.15 to 0.23)	2003-2009	-2.72 (-3.02 to -1.76)	2003-2007	-1.90 (-2.08 to -1.74)

Location	Segment	Incidence		Prevalence		Mortality		YLD		YLL		DALY	
		Period	APC (95%CI)	Period	APC (95%CI)	Period	APC (95%CI)	Period	APC (95%CI)	Period	APC (95%CI)	Period	APC (95%CI)
	3	2019-2021	-1.00 (-1.35 to -0.37)			2009-2012	-2.27 (-2.55 to -1.41)	2019-2021	-1.06 (-1.45 to -0.29)	2009-2012	-2.22 (-2.66 to -1.42)	2007-2012	-1.56 (-1.68 to -1.13)
	4					2012-2019	-1.24 (-1.30 to -1.03)			2012-2019	-1.19 (-1.26 to -0.94)	2012-2019	-0.80 (-0.85 to -0.70)
	5					2019-2021	-1.96 (-2.28 to -1.58)			2019-2021	-2.28 (-2.68 to -1.84)	2019-2021	-1.86 (-2.12 to -1.51)
Southern Latin America	0	1990-1995	-0.16 (-0.31 to -0.07)	1990-2000	0.06 (0.02 to 0.10)	1990-1996	-0.42 (-0.89 to 0.10)	1990-2000	0.04 (-0.02 to 0.09)	1990-1996	-0.11 (-0.63 to 0.50)	1990-1996	-0.12 (-0.41 to 0.20)
	1	1995-2000	0.15 (0.06 to 0.34)	2000-2010	0.59 (0.53 to 0.63)	1996-2001	-3.82 (-4.92 to -3.20)	2000-2010	0.60 (0.53 to 0.66)	1996-2001	-3.73 (-4.90 to -3.05)	1996-2002	-2.12 (-2.68 to -1.78)
	2	2000-2011	0.70 (0.68 to 0.73)	2010-2014	0.94 (0.77 to 1.13)	2001-2014	-1.85 (-2.29 to -1.68)	2010-2014	1.27 (1.05 to 1.51)	2001-2014	-1.70 (-2.06 to -1.51)	2002-2019	-0.44 (-0.51 to -0.31)
	3	2011-2014	1.78 (1.66 to 1.87)	2014-2021	0.13 (0.04 to 0.20)	2014-2019	-0.93 (-1.49 to 0.00)	2014-2021	0.18 (0.09 to 0.26)	2014-2019	-0.55 (-1.22 to 0.64)	2019-2021	-2.63 (-3.92 to -0.97)
	4	2014-2021	0.06 (0.00 to 0.12)			2019-2021	-5.97 (-7.63 to -4.04)			2019-2021	-6.77 (-8.72 to -4.29)		
Southern Sub-Saharan Africa	0	1990-1993	-0.43 (-0.58 to -0.30)	1990-1993	-0.51 (-0.60 to -0.46)	1990-1992	-0.03 (-2.55 to 2.05)	1990-1997	-0.40 (-0.48 to -0.37)	1990-1997	-3.62 (-4.26 to -2.98)	1990-1998	-1.81 (-2.21 to -1.46)
	1	1993-1999	-0.25 (-0.30 to 0.00)	1993-1999	-0.31 (-0.33 to -0.29)	1992-1996	-5.00 (-6.44 to -3.95)	1997-2000	-0.22 (-0.36 to 0.02)	1997-2004	6.37 (5.52 to 7.22)	1998-2004	4.50 (3.80 to 5.18)
	2	1999-2010	-0.00 (-0.02 to 0.58)	1999-2008	0.02 (0.01 to 0.03)	1996-1999	3.01 (-0.15 to 6.76)	2000-2010	0.04 (0.02 to 0.08)	2004-2014	-2.95 (-3.99 to -2.49)	2004-2013	-1.94 (-2.75 to -1.57)
	3	2010-2018	0.65 (0.62 to 0.68)	2008-2011	0.17 (0.12 to 0.20)	1999-2004	7.42 (-2.19 to 8.70)	2010-2015	0.68 (0.64 to 0.74)	2014-2021	-1.32 (-1.96 to 0.13)	2013-2021	-0.61 (-1.03 to 0.33)
	4	2018-2021	0.17 (0.05 to 0.30)	2011-2015	0.73 (0.69 to 0.76)	2004-2013	-2.96 (-3.70 to -2.42)	2015-2019	0.45 (0.38 to 0.51)				
	5			2015-2019	0.47 (0.43 to 0.50)	2013-2021	-1.35 (-1.80 to -0.70)	2019-2021	-0.26 (-0.39 to -0.14)				
	6			2019-2021	-0.20 (-0.26 to -0.13)								
Tropical Latin America	0	1990-1996	0.07 (-0.05 to 0.15)	1990-1996	0.19 (0.08 to 0.26)	1990-1996	0.70 (0.09 to 1.47)	1990-1996	0.17 (0.05 to 0.25)	1990-1993	-0.54 (-2.57 to 1.12)	1990-1993	-0.29 (-1.33 to 0.57)
	1	1996-2001	0.52 (0.35 to 0.68)	1996-2001	0.63 (0.48 to 0.77)	1996-2004	-2.29 (-3.06 to -1.91)	1996-2002	0.65 (0.53 to 0.77)	1993-1996	2.26 (-2.75 to 3.11)	1993-1996	1.41 (-1.13 to 1.84)
	2	2001-2009	0.90 (0.84 to 0.98)	2001-2010	0.97 (0.93 to 1.02)	2004-2014	0.52 (-0.16 to 0.89)	2002-2010	1.07 (1.02 to 1.14)	1996-2003	-2.46 (-3.08 to 0.71)	1996-2003	-1.11 (-1.49 to 0.91)
	3	2009-2015	1.72 (1.65 to 1.78)	2010-2014	2.09 (2.01 to 2.16)	2014-2017	3.69 (2.09 to 4.43)	2010-2014	2.20 (2.11 to 2.29)	2003-2014	0.44 (-0.00 to 0.75)	2003-2014	0.93 (0.54 to 1.09)
	4	2015-2019	0.24 (0.15 to 0.39)	2014-2019	0.38 (0.31 to 0.46)	2017-2021	0.05 (-1.68 to 0.77)	2014-2019	0.46 (0.39 to 0.55)	2014-2017	4.11 (2.89 to 4.80)	2014-2017	2.66 (1.75 to 3.07)
	5	2019-2021	-0.63 (-0.86 to -0.31)	2019-2021	-0.82 (-1.02 to -0.54)			2019-2021	-0.98 (-1.22 to -0.71)	2017-2021	0.12 (-1.03 to 0.73)	2017-2021	-0.11 (-0.77 to 0.25)
Western Europe	0	1990-1995	1.35 (0.69 to 1.62)	1990-1995	1.45 (0.74 to 1.70)	1990-1993	-2.70 (-3.35 to -1.69)	1990-1995	1.47 (0.70 to 1.78)	1990-2014	-3.90 (-4.01 to -3.82)	1990-1996	-0.76 (-1.07 to -0.57)
	1	1995-2003	1.95 (1.81 to 2.46)	1995-2003	2.08 (1.96 to 2.53)	1993-1998	-4.89 (-5.61 to -4.55)	1995-2001	2.51 (2.26 to 3.18)	2014-2021	-2.83 (-3.42 to -1.53)	1996-2012	0.16 (0.04 to 0.23)
	2	2003-2021	1.45 (1.39 to 1.49)	2003-2016	1.48 (1.41 to 1.58)	1998-2002	-3.45 (-3.95 to -2.80)	2001-2016	1.54 (1.47 to 1.62)			2012-2021	0.56 (0.38 to 1.02)
	3			2016-2019	0.78 (0.39 to 1.53)	2002-2013	-4.32 (-4.70 to -4.19)	2016-2019	0.55 (0.14 to 1.50)				
	4			2019-2021	2.59 (1.27 to 3.28)	2013-2021	-2.75 (-3.02 to -2.43)	2019-2021	2.62 (1.32 to 3.34)				
Western Sub-Saharan Africa	0	1990-1997	-0.26 (-0.57 to 0.14)	1990-1995	-0.35 (-0.76 to -0.10)	1990-1995	-1.92 (-2.12 to -1.69)	1990-1995	-0.29 (-0.72 to -0.13)	1990-1995	-1.57 (-1.74 to -1.39)	1990-1994	-1.12 (-1.31 to -0.85)
	1	1997-2005	0.03 (-0.37 to 0.25)	1995-2005	-0.00 (-0.37 to 0.29)	1995-2000	-3.36 (-3.53 to -3.16)	1995-2005	0.04 (-0.05 to 0.19)	1995-2000	-2.74 (-2.93 to -2.57)	1994-2000	-1.88 (-2.13 to -1.78)
	2	2005-2010	0.35 (-0.23 to 0.61)	2005-2010	0.47 (-0.32 to 0.77)	2000-2011	-0.80 (-0.94 to -0.74)	2005-2010	0.49 (0.34 to 0.81)	2000-2006	-0.76 (-0.87 to -0.50)	2000-2011	-0.56 (-0.65 to -0.51)
	3	2010-2015	-0.10 (-0.37 to 0.56)	2010-2015	-0.07 (-0.34 to 0.71)	2011-2014	0.93 (-0.80 to 1.16)	2010-2015	-0.09 (-0.43 to 0.08)	2006-2011	-1.13 (-1.40 to -0.99)	2011-2014	0.80 (-0.54 to 0.97)
	4	2015-2019	0.23 (-0.23 to 0.40)	2015-2019	0.29 (-0.26 to 0.42)	2014-2017	-1.40 (-1.70 to 0.74)	2015-2021	0.44 (0.31 to 0.79)	2011-2014	1.39 (1.12 to 1.65)	2014-2017	-0.83 (-1.08 to 0.58)
	5	2019-2021	0.90 (0.35 to 1.26)	2019-2021	0.86 (0.35 to 1.21)	2017-2021	-2.18 (-2.63 to -1.93)			2014-2017	-1.33 (-1.66 to -1.10)	2017-2021	-1.51 (-1.86 to -1.33)
	6									2017-2021	-2.50 (-2.80 to -2.33)		
**204 countries and territories**													
Afghanistan	0	1990-2000	-0.38 (-0.54 to -0.25)	1990-1995	0.30 (0.02 to 0.94)	1990-1993	-0.39 (-1.42 to 0.53)	1990-1995	0.29 (0.05 to 0.72)	1990-1994	-0.73 (-2.18 to 0.05)	1990-1994	-0.54 (-1.71 to 0.17)
	1	2000-2016	0.30 (-0.05 to 0.37)	1995-2000	-0.91 (-1.49 to -0.60)	1993-2002	0.72 (0.51 to 2.60)	1995-1999	-1.21 (-1.66 to -0.82)	1994-2002	0.75 (0.38 to 1.54)	1994-2002	0.49 (0.21 to 2.68)
	2	2016-2019	1.11 (0.17 to 1.40)	2000-2012	0.26 (0.17 to 0.35)	2002-2005	2.55 (-1.53 to 2.98)	1999-2012	0.26 (0.20 to 0.33)	2002-2005	3.26 (1.57 to 3.86)	2002-2005	2.83 (-1.40 to 3.32)
	3	2019-2021	-0.75 (-1.55 to 0.53)	2012-2019	1.48 (0.47 to 1.92)	2005-2011	-1.40 (-1.97 to -0.13)	2012-2019	1.44 (1.28 to 1.84)	2005-2011	-1.61 (-2.42 to -1.09)	2005-2011	-1.30 (-1.97 to 0.08)
	4			2019-2021	0.18 (-0.44 to 1.24)	2011-2021	-0.30 (-0.51 to -0.07)	2019-2021	-0.25 (-0.78 to 0.62)	2011-2021	-0.23 (-0.42 to 0.01)	2011-2021	0.04 (-0.16 to 0.25)
Albania	0	1990-2001	0.51 (0.48 to 0.53)	1990-2001	0.61 (0.57 to 0.64)	1990-1995	-4.41 (-5.95 to -3.56)	1990-2002	0.71 (0.67 to 0.74)	1990-1994	-4.11 (-6.76 to -2.69)	1990-1994	-2.62 (-4.16 to -1.78)
	1	2001-2005	1.04 (0.86 to 1.22)	2001-2005	1.14 (0.87 to 1.40)	1995-2003	-1.59 (-2.09 to -0.70)	2002-2006	1.31 (0.92 to 1.57)	1994-2003	-1.51 (-2.00 to -0.06)	1994-2003	-0.74 (-1.00 to 0.06)
	2	2005-2015	1.52 (1.50 to 1.56)	2005-2015	1.74 (1.70 to 1.79)	2003-2009	-5.50 (-6.31 to -4.85)	2006-2015	1.79 (1.74 to 1.86)	2003-2009	-5.41 (-6.83 to -4.46)	2003-2009	-2.38 (-3.37 to -1.89)
	3	2015-2018	2.73 (2.60 to 2.83)	2015-2018	2.97 (2.77 to 3.08)	2009-2016	1.16 (0.61 to 1.90)	2015-2019	2.83 (2.72 to 3.11)	2009-2017	0.59 (-0.08 to 1.62)	2009-2018	1.22 (0.94 to 1.65)
	4	2018-2021	2.03 (1.84 to 2.15)	2018-2021	2.18 (1.93 to 2.30)	2016-2021	-3.52 (-4.74 to -2.53)	2019-2021	1.90 (1.56 to 2.25)	2017-2021	-5.98 (-8.38 to -4.30)	2018-2021	-2.15 (-4.27 to -0.85)
Algeria	0	1990-2000	-0.23 (-0.25 to -0.22)	1990-2000	-0.30 (-0.32 to -0.28)	1990-1993	-2.78 (-3.70 to -2.08)	1990-2000	-0.26 (-0.33 to -0.21)	1990-1994	-2.62 (-3.57 to -2.13)	1990-1994	-1.87 (-2.61 to -1.52)
	1	2000-2004	0.64 (0.59 to 0.72)	2000-2005	0.57 (0.53 to 0.64)	1993-1999	-1.24 (-1.62 to -0.62)	2000-2013	0.51 (0.43 to 0.54)	1994-2000	-1.09 (-1.64 to -0.63)	1994-2000	-0.77 (-1.14 to -0.41)
	2	2004-2011	0.39 (0.35 to 0.42)	2005-2011	0.40 (0.29 to 0.44)	1999-2007	0.09 (-0.09 to 0.49)	2013-2019	1.47 (0.53 to 1.72)	2000-2006	-0.16 (-0.43 to 0.40)	2000-2006	0.05 (-0.13 to 0.49)
	3	2011-2015	0.98 (0.89 to 1.05)	2011-2015	0.95 (0.84 to 1.04)	2007-2014	-1.03 (-1.17 to -0.75)	2019-2021	0.81 (0.48 to 1.33)	2006-2014	-1.39 (-1.50 to -1.19)	2006-2017	-0.64 (-0.81 to -0.58)
	4	2015-2019	1.52 (1.45 to 1.63)	2015-2019	1.59 (1.52 to 1.71)	2014-2017	-1.99 (-2.25 to -1.52)			2014-2017	-2.29 (-2.56 to -1.87)	2017-2021	-0.13 (-0.38 to 0.28)
	5	2019-2021	0.77 (0.63 to 0.96)	2019-2021	0.79 (0.62 to 1.01)	2017-2021	-0.97 (-1.28 to -0.35)			2017-2021	-1.11 (-1.38 to -0.47)		
American Samoa	0	1990-2001	0.18 (0.03 to 0.66)	1990-2001	0.15 (-0.02 to 0.65)	1990-1998	0.07 (-0.43 to 0.48)	1990-2003	0.18 (0.12 to 0.28)	1990-1998	-0.26 (-0.67 to 0.07)	1990-1998	-0.14 (-0.40 to 0.08)
	1	2001-2011	0.03 (-0.44 to 0.63)	2001-2011	0.03 (-0.37 to 0.62)	1998-2003	6.43 (5.66 to 7.33)	2003-2010	-0.08 (-0.49 to 0.13)	1998-2003	5.12 (4.45 to 6.05)	1998-2003	3.71 (3.26 to 4.32)
	2	2011-2019	0.63 (0.03 to 0.79)	2011-2019	0.56 (0.03 to 0.70)	2003-2007	2.03 (0.32 to 3.48)	2010-2019	0.50 (0.06 to 0.61)	2003-2007	1.84 (0.29 to 2.95)	2003-2007	1.26 (0.32 to 1.95)
	3	2019-2021	1.59 (0.71 to 2.00)	2019-2021	1.39 (0.62 to 1.77)	2007-2016	-2.97 (-3.36 to -2.68)	2019-2021	1.46 (0.64 to 1.85)	2007-2016	-2.50 (-2.84 to -2.26)	2007-2016	-1.73 (-1.97 to -1.56)
	4					2016-2021	0.57 (-0.19 to 1.63)			2016-2021	0.42 (-0.24 to 1.32)	2016-2021	0.57 (0.10 to 1.18)
Andorra	0	1990-1995	1.38 (1.08 to 1.55)	1990-1995	1.38 (1.03 to 1.57)	1990-2009	-1.91 (-2.17 to -1.70)	1990-1995	1.37 (0.94 to 1.57)	1990-2009	-2.02 (-2.22 to -1.85)	1990-2009	0.11 (0.04 to 0.17)
	1	1995-2000	2.36 (2.17 to 2.76)	1995-2000	2.41 (2.19 to 2.84)	2009-2019	-0.36 (-0.74 to 0.60)	1995-2001	2.29 (2.13 to 2.69)	2009-2019	-0.44 (-0.78 to 0.26)	2009-2018	1.53 (1.33 to 1.83)
	2	2000-2010	1.55 (1.47 to 1.62)	2000-2010	1.45 (1.36 to 1.51)	2019-2021	-11.28 (-14.61 to -7.18)	2001-2010	1.46 (1.34 to 1.54)	2019-2021	-11.15 (-14.11 to -7.21)	2018-2021	-0.88 (-2.21 to -0.02)
	3	2010-2015	2.81 (2.64 to 3.18)	2010-2015	2.64 (2.47 to 3.09)			2010-2016	2.60 (2.47 to 2.97)				
	4	2015-2021	1.87 (1.71 to 2.00)	2015-2021	2.01 (1.80 to 2.13)			2016-2021	1.83 (1.57 to 2.01)				
Angola	0	1990-1994	-0.68 (-0.75 to -0.61)	1990-1994	-0.63 (-0.77 to -0.56)	1990-1994	0.54 (-0.37 to 2.39)	1990-1993	-0.74 (-1.05 to -0.56)	1990-1994	0.65 (-0.45 to 2.95)	1990-1994	0.30 (-0.61 to 2.23)
	1	1994-1999	-0.17 (-0.25 to -0.12)	1994-1998	-0.16 (-0.37 to -0.04)	1994-1997	-3.73 (-4.70 to -1.89)	1993-2000	-0.15 (-0.26 to -0.07)	1994-1997	-4.67 (-5.82 to -2.56)	1994-1997	-3.51 (-4.40 to -1.72)

Location	Segment	Incidence		Prevalence		Mortality		YLD		YLL		DALY	
		Period	APC (95%CI)	Period	APC (95%CI)	Period	APC (95%CI)	Period	APC (95%CI)	Period	APC (95%CI)	Period	APC (95%CI)
	2	1999-2005	0.05 (0.02 to 0.11)	1998-2005	0.01 (-0.01 to 0.33)	1997-2004	0.87 (0.30 to 2.20)	2000-2006	0.16 (0.04 to 0.30)	1997-2004	1.43 (0.78 to 2.84)	1997-2004	1.04 (0.50 to 2.17)
	3	2005-2015	0.49 (0.47 to 0.51)	2005-2015	0.56 (0.53 to 0.58)	2004-2021	-1.53 (-1.71 to -1.37)	2006-2015	0.66 (0.61 to 0.71)	2004-2021	-1.66 (-1.87 to -1.49)	2004-2021	-1.07 (-1.23 to -0.93)
	4	2015-2021	0.24 (0.18 to 0.28)	2015-2021	0.32 (0.25 to 0.37)			2015-2021	0.21 (0.14 to 0.28)				
Antigua and Barbuda	0	1990-1999	-0.95 (-1.10 to -0.82)	1990-1999	-1.27 (-1.41 to -1.12)	1990-1996	1.26 (0.51 to 1.88)	1990-1998	-1.24 (-1.48 to -1.05)	1990-1997	-0.44 (-0.94 to 0.12)	1990-1993	-1.22 (-2.22 to -0.48)
	1	1999-2007	0.19 (0.02 to 0.65)	1999-2008	0.09 (-0.07 to 0.50)	1996-2002	-4.11 (-5.51 to -3.40)	1998-2013	-0.07 (-0.15 to 0.03)	1997-2001	-4.82 (-5.92 to -3.75)	1993-1996	0.34 (-3.32 to 0.82)
	2	2007-2014	-0.32 (-0.85 to -0.08)	2008-2014	-0.52 (-1.06 to -0.17)	2002-2011	-2.19 (-2.92 to -1.65)	2013-2021	-1.52 (-1.78 to -1.31)	2001-2012	-2.20 (-2.62 to -1.90)	1996-2002	-3.42 (-3.79 to -0.97)
	3	2014-2021	-1.41 (-1.80 to -1.21)	2014-2021	-1.59 (-2.07 to -1.37)	2011-2019	-0.71 (-1.14 to 0.77)			2012-2019	-1.00 (-1.46 to 0.34)	2002-2006	-0.71 (-2.17 to 0.07)
	4					2019-2021	-5.93 (-7.76 to -3.48)			2019-2021	-5.68 (-7.36 to -3.35)	2006-2009	-2.61 (-3.02 to -1.43)
	5											2009-2019	-1.13 (-1.27 to -0.77)
	6											2019-2021	-4.76 (-5.79 to -3.37)
Argentina	0	1990-1999	-0.13 (-0.21 to -0.06)	1990-1998	-0.13 (-0.28 to -0.04)	1990-1997	-0.49 (-0.91 to 0.02)	1990-1998	-0.15 (-0.30 to -0.05)	1990-1997	-0.11 (-0.68 to 0.59)	1990-1997	-0.12 (-0.39 to 0.21)
	1	1999-2010	0.46 (0.40 to 0.52)	1998-2011	0.37 (0.11 to 0.42)	1997-2001	-3.83 (-4.71 to -2.95)	1998-2011	0.42 (0.34 to 0.47)	1997-2001	-3.87 (-4.97 to -2.58)	1997-2001	-2.53 (-3.31 to -1.82)
	2	2010-2014	1.60 (1.35 to 1.95)	2011-2014	0.82 (0.41 to 1.00)	2001-2014	-1.66 (-2.01 to -1.46)	2011-2014	1.29 (0.82 to 1.50)	2001-2014	-1.52 (-2.21 to -1.25)	2001-2019	-0.50 (-0.59 to -0.36)
	3	2014-2021	-0.17 (-0.28 to -0.07)	2014-2021	-0.26 (-0.46 to -0.14)	2014-2019	-0.48 (-1.08 to 0.57)	2014-2021	-0.19 (-0.35 to -0.07)	2014-2019	-0.22 (-1.07 to 1.15)	2019-2021	-3.14 (-4.58 to -1.23)
	4					2019-2021	-6.35 (-8.12 to -4.26)			2019-2021	-7.15 (-9.52 to -4.38)		
Armenia	0	1990-2000	-0.36 (-0.42 to -0.31)	1990-2001	-0.24 (-0.28 to -0.20)	1990-1996	5.58 (4.27 to 7.61)	1990-2001	-0.19 (-0.26 to -0.13)	1990-1993	7.59 (4.88 to 11.60)	1990-1993	6.27 (4.18 to 9.26)
	1	2000-2006	0.52 (0.33 to 0.65)	2001-2007	0.87 (0.68 to 0.97)	1996-2003	-0.42 (-1.26 to 0.85)	2001-2006	0.84 (0.33 to 1.04)	1993-1997	1.54 (-1.50 to 3.49)	1993-1997	1.30 (-1.09 to 2.81)
	2	2006-2012	1.23 (1.07 to 1.40)	2007-2014	1.54 (1.43 to 1.65)	2003-2006	-7.98 (-9.52 to -4.28)	2006-2012	1.38 (1.20 to 1.64)	1997-2003	-1.45 (-7.24 to -0.19)	1997-2003	-1.23 (-5.71 to -0.45)
	3	2012-2019	1.87 (1.77 to 2.13)	2014-2019	2.24 (2.10 to 2.55)	2006-2009	1.17 (-2.27 to 3.13)	2012-2019	2.16 (2.06 to 2.45)	2003-2006	-6.50 (-7.89 to 3.27)	2003-2006	-5.33 (-6.51 to 2.81)
	4	2019-2021	0.08 (-0.33 to 0.77)	2019-2021	0.28 (-0.09 to 0.80)	2009-2015	-3.40 (-4.88 to -2.20)	2019-2021	0.29 (-0.13 to 0.99)	2006-2009	2.65 (-4.44 to 4.21)	2006-2009	2.50 (-3.50 to 3.88)
	5					2015-2021	-7.72 (-9.60 to -6.78)			2009-2016	-2.73 (-8.80 to -1.15)	2009-2016	-1.90 (-5.43 to -0.34)
	6									2016-2021	-8.75 (-12.14 to -7.00)	2016-2021	-5.97 (-8.58 to -4.49)
Australia	0	1990-1994	2.37 (2.07 to 2.52)	1990-1995	2.46 (2.16 to 2.59)	1990-1993	1.23 (0.13 to 2.92)	1990-1995	1.58 (1.24 to 1.81)	1990-1997	-0.14 (-1.49 to 0.66)	1990-1998	1.12 (1.02 to 1.24)
	1	1994-2003	2.65 (2.60 to 2.77)	1995-2004	2.73 (2.67 to 2.83)	1993-1997	-1.72 (-3.83 to -1.01)	1995-2000	2.77 (2.46 to 3.17)	1997-2001	-7.50 (-9.08 to 0.86)	1998-2001	-0.74 (-1.00 to -0.19)
	2	2003-2007	2.07 (1.92 to 2.31)	2004-2010	1.56 (1.46 to 1.65)	1997-2001	-7.75 (-8.56 to -6.79)	2000-2006	1.21 (1.02 to 1.49)	2001-2005	-0.01 (-7.81 to 1.99)	2001-2006	0.88 (0.53 to 1.26)
	3	2007-2010	1.44 (1.26 to 1.64)	2010-2015	-1.21 (-1.35 to -1.07)	2001-2011	-1.65 (-1.87 to -1.28)	2006-2009	4.14 (1.24 to 4.44)	2005-2016	-2.42 (-3.79 to 0.25)	2006-2010	2.19 (1.86 to 2.63)
	4	2010-2015	-1.09 (-1.22 to -0.98)	2015-2021	0.98 (0.81 to 1.14)	2011-2015	-3.56 (-4.65 to -2.72)	2009-2012	-0.33 (-0.69 to 4.22)	2016-2019	1.39 (-3.00 to 2.46)	2010-2015	-1.57 (-1.89 to -1.35)
	5	2015-2021	1.17 (1.03 to 1.30)			2015-2019	0.49 (-0.18 to 1.73)	2012-2015	-1.33 (-1.60 to -0.66)	2019-2021	-3.44 (-5.81 to -0.19)	2015-2021	0.87 (0.66 to 1.10)
	6					2019-2021	-3.07 (-4.55 to -1.50)	2015-2021	1.08 (0.91 to 1.30)				
Austria	0	1990-1995	1.63 (1.43 to 1.76)	1990-1996	1.79 (1.73 to 1.85)	1990-1993	-1.94 (-3.02 to -0.88)	1990-1995	1.71 (1.57 to 1.85)	1990-1993	-1.43 (-2.60 to -0.42)	1990-1992	0.63 (0.01 to 1.14)
	1	1995-2005	2.62 (2.58 to 2.66)	1996-2004	2.74 (2.71 to 2.78)	1993-1998	-6.19 (-7.16 to -5.70)	1995-2000	3.50 (3.35 to 3.69)	1993-1998	-6.32 (-7.07 to -5.89)	1992-1997	-1.14 (-1.35 to -0.99)
	2	2005-2015	1.49 (1.45 to 1.53)	2004-2008	1.75 (1.66 to 2.02)	1998-2004	-2.22 (-2.73 to -1.54)	2000-2004	2.92 (2.70 to 3.10)	1998-2003	-1.92 (-2.48 to -1.02)	1997-2004	1.30 (1.15 to 1.46)
	3	2015-2021	2.55 (2.46 to 2.64)	2008-2015	1.43 (1.37 to 1.48)	2004-2012	-4.62 (-5.43 to -4.21)	2004-2012	0.98 (0.86 to 1.06)	2003-2012	-4.41 (-5.12 to -4.17)	2004-2013	-0.30 (-0.46 to -0.17)
	4			2015-2021	2.60 (2.54 to 2.65)	2012-2019	-3.35 (-4.02 to -2.24)	2012-2015	1.61 (1.10 to 2.24)	2012-2019	-3.35 (-3.67 to -2.36)	2013-2021	1.45 (1.28 to 1.66)
	5					2019-2021	-5.85 (-7.50 to -3.86)	2015-2021	2.60 (2.49 to 2.81)	2019-2021	-7.93 (-9.44 to -6.15)		
Azerbaijan	0	1990-1995	0.37 (0.30 to 0.46)	1990-1994	0.48 (0.40 to 0.56)	1990-1993	9.85 (7.71 to 11.18)	1990-1994	0.51 (0.41 to 0.64)	1990-1993	10.86 (8.22 to 12.56)	1990-1993	8.92 (7.10 to 10.16)
	1	1995-2002	0.11 (-0.00 to 0.15)	1994-2001	0.10 (0.05 to 0.13)	1993-2003	1.31 (1.08 to 1.53)	1994-2002	0.14 (0.08 to 0.17)	1993-2003	0.50 (0.18 to 0.80)	1993-2003	0.52 (0.25 to 0.79)
	2	2002-2006	0.45 (0.37 to 0.60)	2001-2005	0.50 (0.42 to 0.60)	2003-2021	-3.05 (-3.18 to -2.95)	2002-2006	0.62 (0.54 to 0.75)	2003-2021	-3.07 (-3.24 to -2.92)	2003-2008	-2.75 (-3.71 to -1.96)
	3	2006-2011	1.39 (1.34 to 1.45)	2005-2008	1.23 (1.14 to 1.31)			2006-2011	1.76 (1.70 to 1.81)			2008-2021	-1.77 (-2.01 to -1.11)
	4	2011-2018	2.14 (2.10 to 2.18)	2008-2011	1.80 (1.74 to 1.93)			2011-2018	2.43 (2.39 to 2.47)				
	5	2018-2021	1.20 (1.08 to 1.31)	2011-2018	2.38 (2.35 to 2.41)			2018-2021	1.41 (1.30 to 1.51)				
	6			2018-2021	1.38 (1.30 to 1.45)								
Bahamas	0	1990-1996	-0.88 (-1.26 to -0.65)	1990-1997	-1.07 (-1.37 to -0.87)	1990-1994	2.18 (1.61 to 2.73)	1990-1995	-1.29 (-1.73 to -1.01)	1990-1994	2.30 (1.51 to 3.15)	1990-1994	1.54 (0.91 to 2.21)
	1	1996-2014	-0.12 (-0.17 to -0.06)	1997-2014	-0.20 (-0.26 to -0.12)	1994-1999	-2.59 (-2.98 to -1.97)	1995-2015	-0.23 (-0.28 to -0.18)	1994-1999	-2.60 (-3.18 to -1.79)	1994-1999	-2.18 (-2.58 to -1.57)
	2	2014-2021	-1.29 (-1.55 to -1.10)	2014-2021	-1.29 (-1.58 to -1.08)	1999-2002	-6.26 (-6.80 to -5.24)	2015-2021	-1.40 (-1.77 to -1.14)	1999-2002	-7.08 (-7.74 to -5.79)	1999-2002	-5.61 (-6.14 to -4.60)
	3					2002-2011	-1.42 (-1.59 to -1.09)			2002-2011	-1.13 (-1.38 to -0.79)	2002-2011	-0.89 (-1.08 to -0.60)
	4					2011-2015	-2.73 (-3.51 to -2.08)			2011-2015	-3.99 (-5.14 to -3.10)	2011-2015	-2.96 (-3.88 to -2.23)
	5					2015-2021	-0.37 (-0.95 to 0.71)			2015-2021	0.47 (-0.39 to 1.80)	2015-2021	-0.09 (-0.63 to 0.84)
Bahrain	0	1990-2000	-0.23 (-0.25 to -0.21)	1990-2000	-0.22 (-0.25 to -0.19)	1990-1995	-1.61 (-3.08 to -1.01)	1990-1995	-0.19 (-0.29 to -0.03)	1990-1995	-2.62 (-4.04 to -1.97)	1990-1995	-1.95 (-2.71 to -1.53)
	1	2000-2011	0.34 (0.32 to 0.35)	2000-2004	0.59 (0.06 to 0.68)	1995-1998	1.37 (-0.11 to 2.15)	1995-1999	-0.62 (-0.81 to -0.49)	1995-1998	2.14 (0.37 to 3.07)	1995-1998	1.26 (-0.02 to 1.82)
	2	2011-2014	0.97 (0.82 to 1.03)	2004-2011	0.41 (0.30 to 0.45)	1998-2003	-4.50 (-5.54 to -4.00)	1999-2011	0.42 (0.39 to 0.46)	1998-2003	-4.71 (-5.99 to -4.05)	1998-2003	-3.19 (-3.91 to -2.72)
	3	2014-2019	0.54 (0.44 to 0.60)	2011-2014	1.03 (0.86 to 1.11)	2003-2006	-1.67 (-2.81 to -0.94)	2011-2014	1.34 (1.10 to 1.46)	2003-2021	-2.63 (-2.75 to -2.49)	2003-2011	-1.62 (-2.49 to -1.36)
	4	2019-2021	-0.61 (-0.77 to -0.42)	2014-2019	0.68 (0.56 to 0.75)	2006-2016	-3.32 (-4.45 to -3.12)	2014-2019	0.73 (0.61 to 0.82)			2011-2021	-1.14 (-1.33 to -0.44)
	5			2019-2021	-0.39 (-0.56 to -0.15)	2016-2021	-1.54 (-2.39 to 0.09)	2019-2021	-0.72 (-1.02 to -0.29)				
Bangladesh	0	1990-2007	0.32 (0.26 to 0.34)	1990-2015	0.39 (0.37 to 0.42)	1990-1998	-1.44 (-1.98 to -1.01)	1990-2013	0.47 (0.44 to 0.49)	1990-1999	-2.16 (-2.66 to -1.73)	1990-1999	-1.59 (-1.99 to -1.27)
	1	2007-2015	0.46 (0.38 to 0.76)	2015-2021	-0.16 (-0.46 to 0.03)	1998-2008	0.76 (0.48 to 1.19)	2013-2021	-0.05 (-0.21 to 0.05)	1999-2008	1.06 (0.72 to 1.59)	1999-2008	0.93 (0.66 to 1.33)
	2	2015-2021	0.02 (-0.23 to 0.15)			2008-2013	-3.09 (-4.38 to -2.36)			2008-2013	-2.82 (-4.25 to -2.03)	2008-2013	-2.05 (-3.14 to -1.44)
	3					2013-2021	-0.45 (-0.82 to 0.02)			2013-2021	-0.21 (-0.62 to 0.41)	2013-2021	-0.16 (-0.46 to 0.30)
Barbados	0	1990-2000	-0.89 (-0.97 to -0.80)	1990-1995	-0.93 (-1.15 to -0.42)	1990-1992	0.95 (-1.42 to 2.46)	1990-2000	-1.11 (-1.24 to -1.00)	1990-1992	1.15 (-1.44 to 2.97)	1990-2021	-1.51 (-1.59 to -1.45)
	1	2000-2011	0.04 (-0.03 to 0.12)	1995-1999	-1.58 (-1.85 to -1.22)	1992-2021	-1.63 (-1.83 to -1.55)	2000-2010	0.06 (-0.07 to 0.22)	1992-2021	-1.91 (-2.06 to -1.83)		
	2	2011-2021	-1.01 (-1.12 to -0.92)	1999-2011	0.00 (-0.05 to 0.06)			2010-2021	-1.01 (-1.14 to -0.91)				
	3			2011-2018	-1.36 (-1.66 to -1.25)								

Location	Segment	Incidence		Prevalence		Mortality		YLD		YLL		DALY	
		Period	APC (95%CI)	Period	APC (95%CI)	Period	APC (95%CI)	Period	APC (95%CI)	Period	APC (95%CI)	Period	APC (95%CI)
	4			2018-2021	-0.36 (-0.85 to 0.40)								
Belarus	0	1990-2002	0.17 (0.12 to 0.21)	1990-2002	0.24 (0.20 to 0.28)	1990-1995	6.10 (1.39 to 13.94)	1990-2002	0.27 (0.21 to 0.31)	1990-1995	6.78 (2.40 to 13.98)	1990-1995	4.67 (2.66 to 7.25)
	1	2002-2006	0.81 (0.48 to 1.10)	2002-2006	0.85 (0.64 to 1.11)	1995-2012	-5.63 (-6.36 to -5.11)	2002-2006	0.91 (0.58 to 1.24)	1995-2011	-5.95 (-6.81 to -5.36)	1995-2010	-4.04 (-4.62 to -3.64)
	2	2006-2010	1.95 (1.63 to 2.25)	2006-2010	2.15 (1.90 to 2.36)	2012-2021	8.50 (6.26 to 11.19)	2006-2010	2.21 (1.83 to 2.49)	2011-2021	8.15 (6.06 to 10.58)	2010-2021	4.99 (3.96 to 6.23)
	3	2010-2014	2.94 (2.71 to 3.26)	2010-2014	3.15 (2.96 to 3.44)			2010-2014	3.17 (2.93 to 3.49)				
	4	2014-2019	1.60 (1.40 to 1.83)	2014-2019	1.65 (1.48 to 1.86)			2014-2019	1.75 (1.55 to 1.98)				
	5	2019-2021	0.02 (-0.42 to 0.66)	2019-2021	-0.11 (-0.48 to 0.50)			2019-2021	0.14 (-0.25 to 0.73)				
Belgium	0	1990-1996	1.44 (1.39 to 1.48)	1990-1994	1.32 (1.16 to 1.43)	1990-2009	-3.87 (-4.15 to -3.69)	1990-1993	2.25 (2.02 to 2.62)	1990-2009	-3.67 (-4.18 to -3.34)	1990-2000	-0.62 (-0.80 to -0.52)
	1	1996-2004	1.70 (1.68 to 1.72)	1994-2004	1.69 (1.66 to 1.72)	2009-2014	-5.51 (-7.03 to -3.47)	1993-2001	1.73 (1.61 to 1.78)	2009-2012	-6.82 (-7.70 to -2.77)	2000-2008	-0.03 (-0.21 to 0.52)
	2	2004-2011	0.93 (0.91 to 0.95)	2004-2011	0.95 (0.91 to 0.99)	2014-2021	-3.72 (-4.56 to -1.61)	2001-2004	2.84 (2.62 to 2.98)	2012-2021	-4.24 (-4.97 to -2.22)	2008-2014	-1.28 (-2.05 to -0.96)
	3	2011-2014	1.80 (1.73 to 1.86)	2011-2014	1.80 (1.65 to 1.89)			2004-2010	0.93 (0.84 to 1.04)			2014-2021	-0.07 (-0.36 to 0.49)
	4	2014-2018	1.20 (1.14 to 1.25)	2014-2018	1.23 (1.06 to 1.36)			2010-2015	0.21 (-0.00 to 0.36)				
	5	2018-2021	0.41 (0.33 to 0.49)	2018-2021	0.59 (0.33 to 0.76)			2015-2018	1.26 (1.00 to 1.43)				
	6							2018-2021	0.55 (0.19 to 0.73)				
Belize	0	1990-2000	-1.12 (-1.23 to -1.00)	1990-2000	-1.34 (-1.45 to -1.19)	1990-1998	2.52 (1.73 to 3.53)	1990-2000	-1.31 (-1.45 to -1.16)	1990-1998	1.86 (0.97 to 3.14)	1990-1998	1.01 (0.38 to 1.94)
	1	2000-2011	0.30 (0.18 to 0.48)	2000-2011	0.34 (0.19 to 0.54)	1998-2021	-2.56 (-2.74 to -2.39)	2000-2011	0.35 (0.21 to 0.60)	1998-2021	-2.64 (-2.88 to -2.45)	1998-2021	-1.95 (-2.12 to -1.81)
	2	2011-2019	-0.42 (-0.70 to -0.13)	2011-2019	-0.55 (-0.81 to -0.09)			2011-2019	-0.50 (-0.81 to -0.04)				
	3	2019-2021	-2.55 (-3.14 to -1.35)	2019-2021	-2.58 (-3.26 to -1.23)			2019-2021	-2.65 (-3.37 to -1.24)				
Benin	0	1990-1994	-0.42 (-0.49 to -0.37)	1990-1993	-0.49 (-0.62 to -0.42)	1990-1996	-1.02 (-1.29 to -0.71)	1990-1994	-0.28 (-0.59 to -0.11)	1990-1996	-0.54 (-0.84 to -0.12)	1990-1996	-0.49 (-0.77 to -0.18)
	1	1994-1999	-0.12 (-0.20 to -0.08)	1993-1996	-0.18 (-0.33 to -0.02)	1996-2001	-2.95 (-3.70 to -2.50)	1994-2005	-0.03 (-0.07 to 0.13)	1996-2001	-2.51 (-3.16 to -0.79)	1996-2003	-1.66 (-2.23 to -0.79)
	2	1999-2005	-0.03 (-0.05 to 0.02)	1996-2005	0.00 (-0.01 to 0.05)	2001-2008	-1.15 (-1.58 to -0.82)	2005-2009	0.65 (0.41 to 0.90)	2001-2008	-1.48 (-2.56 to -1.02)	2003-2008	-0.78 (-1.67 to -0.02)
	3	2005-2010	0.38 (0.36 to 0.41)	2005-2010	0.47 (0.43 to 0.49)	2008-2013	1.51 (-0.13 to 2.10)	2009-2016	-0.29 (-0.47 to -0.21)	2008-2013	1.30 (-1.59 to 1.94)	2008-2013	0.94 (-0.75 to 1.49)
	4	2010-2015	-0.33 (-0.37 to -0.31)	2010-2015	-0.40 (-0.44 to -0.36)	2013-2019	-2.10 (-2.63 to 1.23)	2016-2021	0.00 (-0.12 to 0.27)	2013-2016	-1.42 (-1.80 to 1.49)	2013-2019	-1.51 (-2.05 to 0.92)
	5	2015-2021	-0.10 (-0.13 to -0.07)	2015-2021	-0.09 (-0.12 to -0.05)	2019-2021	-0.32 (-1.73 to 0.37)			2016-2019	-2.93 (-3.44 to -2.20)	2019-2021	-0.27 (-1.35 to 0.32)
	6									2019-2021	0.18 (-0.83 to 1.03)		
Bermuda	0	1990-1999	-0.67 (-0.85 to -0.49)	1990-2000	-0.80 (-0.89 to -0.70)	1990-1992	0.69 (-1.28 to 2.26)	1990-1999	-0.79 (-0.95 to -0.64)	1990-1993	-1.10 (-2.84 to 1.63)	1990-1992	-0.30 (-1.58 to 0.68)
	1	1999-2011	0.54 (-0.69 to 0.74)	2000-2010	0.53 (0.43 to 0.70)	1992-1997	-4.33 (-5.09 to -3.64)	1999-2010	0.50 (-0.47 to 0.77)	1993-2007	-5.41 (-6.03 to -5.18)	1992-1998	-3.42 (-4.01 to -2.92)
	2	2011-2015	-0.17 (-0.61 to 0.54)	2010-2015	-0.21 (-0.57 to 0.28)	1997-2007	-5.86 (-6.95 to -5.61)	2010-2015	-0.10 (-0.58 to 0.41)	2007-2015	-3.58 (-4.85 to -2.68)	1998-2001	-4.74 (-5.22 to -2.77)
	3	2015-2021	-0.98 (-1.52 to -0.74)	2015-2021	-1.03 (-1.43 to -0.85)	2007-2014	-3.94 (-4.66 to -2.98)	2015-2021	-1.01 (-1.48 to -0.79)	2015-2021	-0.87 (-2.14 to 1.83)	2001-2007	-2.65 (-4.89 to -1.64)
	4					2014-2021	-0.93 (-1.58 to 0.17)					2007-2014	-1.92 (-2.99 to -0.28)
	5											2014-2021	-1.08 (-2.55 to 0.37)
Bhutan	0	1990-1994	-0.09 (-0.19 to -0.03)	1990-1993	-0.22 (-0.27 to -0.19)	1990-1996	1.37 (0.82 to 1.88)	1990-1998	-0.02 (-0.14 to 0.03)	1990-1995	2.30 (1.25 to 3.72)	1990-1995	1.63 (0.79 to 2.84)
	1	1994-2000	0.02 (-0.00 to 0.21)	1993-1996	-0.02 (-0.07 to 0.04)	1996-2010	-1.76 (-2.03 to -1.64)	1998-2003	0.26 (0.09 to 0.45)	1995-2021	-1.83 (-1.91 to -1.77)	1995-2021	-1.32 (-1.38 to -1.27)
	2	2000-2006	0.33 (0.28 to 0.39)	1996-2001	0.15 (0.13 to 0.18)	2010-2021	-1.30 (-1.46 to -0.85)	2003-2021	0.51 (0.49 to 0.53)				
	3	2006-2019	0.46 (0.45 to 0.47)	2001-2006	0.36 (0.34 to 0.38)								
	4	2019-2021	0.75 (0.61 to 0.82)	2006-2009	0.60 (0.56 to 0.63)								
	5			2009-2016	0.37 (0.36 to 0.38)								
	6			2016-2021	0.55 (0.53 to 0.57)								
Bolivia (Plurinational State of)	0	1990-2000	-0.11 (-0.22 to -0.03)	1990-2000	-0.08 (-0.19 to 0.00)	1990-1994	0.24 (-0.09 to 0.51)	1990-2000	-0.05 (-0.18 to 0.04)	1990-1994	0.02 (-0.26 to 0.26)	1990-1994	-0.03 (-0.24 to 0.16)
	1	2000-2011	0.34 (0.27 to 0.46)	2000-2010	0.43 (0.35 to 0.59)	1994-2003	-1.35 (-1.43 to -1.25)	2000-2011	0.43 (0.35 to 0.61)	1994-2003	-1.47 (-1.55 to -1.38)	1994-2003	-1.10 (-1.15 to -1.02)
	2	2011-2021	-0.19 (-0.29 to -0.11)	2010-2021	-0.08 (-0.17 to -0.01)	2003-2006	-2.78 (-3.00 to -2.32)	2011-2021	-0.10 (-0.22 to -0.02)	2003-2006	-2.96 (-3.16 to -2.42)	2003-2006	-1.97 (-2.12 to -1.65)
	3					2006-2014	-1.35 (-1.45 to -1.04)			2006-2014	-1.65 (-1.74 to -1.25)	2006-2014	-1.12 (-1.18 to -0.86)
	4					2014-2021	-1.73 (-1.97 to -1.61)			2014-2021	-1.90 (-2.19 to -1.79)	2014-2021	-1.34 (-1.51 to -1.26)
Bosnia and Herzegovina	0	1990-1996	-0.20 (-0.47 to -0.06)	1990-1995	-0.26 (-0.42 to -0.17)	1990-1994	3.34 (1.92 to 4.28)	1990-1995	-0.20 (-0.44 to -0.07)	1990-1992	5.06 (2.63 to 6.94)	1990-1994	3.24 (2.46 to 3.86)
	1	1996-2004	0.31 (0.19 to 0.47)	1995-2001	0.30 (0.12 to 0.39)	1994-2005	-4.74 (-5.11 to -4.44)	1995-2001	0.37 (0.00 to 0.50)	1992-1995	1.27 (-5.55 to 2.04)	1994-2003	-3.63 (-4.06 to -3.37)
	2	2004-2016	1.02 (0.96 to 1.08)	2001-2005	0.59 (0.47 to 1.19)	2005-2021	-1.81 (-2.06 to -1.55)	2001-2005	0.63 (0.45 to 1.36)	1995-2003	-4.99 (-5.95 to -2.79)	2003-2011	-1.70 (-2.29 to -1.21)
	3	2016-2021	2.05 (1.85 to 2.36)	2005-2015	1.28 (1.25 to 1.33)			2005-2015	1.26 (1.19 to 2.09)	2003-2011	-2.82 (-3.82 to -1.92)	2011-2019	-0.07 (-0.39 to 1.06)
	4			2015-2021	2.12 (2.06 to 2.18)			2015-2021	2.17 (2.07 to 2.33)	2011-2019	-1.06 (-1.52 to 0.44)	2019-2021	-3.88 (-5.56 to -1.71)
	5									2019-2021	-8.19 (-10.93 to -5.10)		
Botswana	0	1990-2002	-0.04 (-0.08 to -0.00)	1990-1997	-0.14 (-0.50 to -0.03)	1990-1995	-1.28 (-2.01 to -0.13)	1990-1995	-0.32 (-0.81 to -0.09)	1990-1995	-1.24 (-1.86 to -0.26)	1990-1995	-0.82 (-1.20 to -0.17)
	1	2002-2019	0.26 (0.24 to 0.29)	1997-2005	0.07 (-0.03 to 0.43)	1995-1998	-7.19 (-8.27 to -4.91)	1995-2005	0.00 (-0.08 to 0.41)	1995-1998	-6.07 (-6.99 to -4.09)	1995-1998	-3.51 (-4.04 to -2.30)
	2	2019-2021	-0.76 (-1.00 to -0.34)	2005-2015	0.40 (-0.03 to 0.68)	1998-2006	-1.27 (-1.73 to -0.56)	2005-2015	0.42 (0.25 to 0.76)	1998-2006	-1.24 (-1.65 to -0.55)	1998-2006	-0.59 (-0.99 to -0.19)
	3			2015-2021	-0.05 (-0.30 to 0.15)	2006-2010	8.11 (6.97 to 9.20)	2015-2021	-0.08 (-0.40 to 0.12)	2006-2010	7.30 (6.29 to 8.21)	2006-2010	4.05 (-0.38 to 4.69)
	4					2010-2014	-0.58 (-1.59 to 0.78)			2010-2014	-0.55 (-1.42 to 0.81)	2010-2014	-0.08 (-0.54 to 3.92)
	5					2014-2018	-6.03 (-7.34 to -5.02)			2014-2018	-5.42 (-6.55 to -4.50)	2014-2017	-3.12 (-3.62 to -0.25)
	6					2018-2021	-2.22 (-3.55 to 0.05)			2018-2021	-2.35 (-3.58 to -0.45)	2017-2021	-1.56 (-2.17 to -0.47)
Brazil	0	1990-1996	0.09 (-0.03 to 0.17)	1990-1996	0.19 (0.09 to 0.27)	1990-1996	0.69 (0.02 to 1.53)	1990-1996	0.17 (0.06 to 0.25)	1990-1993	-0.57 (-2.59 to 1.07)	1990-1993	-0.30 (-1.33 to 0.53)
	1	1996-2001	0.55 (0.37 to 0.71)	1996-2001	0.65 (0.49 to 0.79)	1996-2004	-2.37 (-3.27 to -1.95)	1996-2002	0.67 (0.55 to 0.79)	1993-1996	2.30 (-2.77 to 3.16)	1993-1996	1.44 (-1.02 to 1.86)
	2	2001-2009	0.91 (0.86 to 1.00)	2001-2010	0.98 (0.94 to 1.03)	2004-2014	0.55 (-0.34 to 0.94)	2002-2010	1.08 (1.03 to 1.15)	1996-2003	-2.55 (-3.15 to 0.70)	1996-2003	-1.15 (-1.52 to 0.29)
	3	2009-2015	1.74 (1.67 to 1.81)	2010-2014	2.11 (2.03 to 2.19)	2014-2017	3.81 (2.03 to 4.57)	2010-2014	2.22 (2.13 to 2.31)	2003-2014	0.48 (0.11 to 0.77)	2003-2014	0.96 (0.64 to 1.12)
	4	2015-2019	0.21 (0.12 to 0.37)	2014-2019	0.37 (0.30 to 0.45)	2017-2021	0.08 (-1.71 to 0.93)	2014-2019	0.45 (0.38 to 0.54)	2014-2017	4.24 (3.04 to 4.91)	2014-2017	2.71 (1.82 to 3.12)
	5	2019-2021	-0.68 (-0.92 to -0.35)	2019-2021	-0.86 (-1.07 to -0.59)			2019-2021	-1.02 (-1.26 to -0.75)	2017-2021	0.16 (-0.93 to 0.79)	2017-2021	-0.10 (-0.72 to 0.26)

Location	Segment	Incidence		Prevalence		Mortality		YLD		YLL		DALY	
		Period	APC (95%CI)	Period	APC (95%CI)	Period	APC (95%CI)	Period	APC (95%CI)	Period	APC (95%CI)	Period	APC (95%CI)
Brunei Darussalam	0	1990-2011	0.13 (0.10 to 0.16)	1990-2003	0.25 (0.21 to 0.32)	1990-2011	-3.51 (-3.63 to -3.12)	1990-2006	0.17 (0.13 to 0.28)	1990-1997	-3.93 (-4.78 to -3.61)	1990-1997	-2.75 (-3.29 to -2.51)
	1	2011-2021	-0.92 (-1.04 to -0.81)	2003-2011	0.01 (-0.16 to 0.13)	2011-2016	-0.88 (-3.87 to -0.12)	2006-2012	-0.16 (-0.68 to 0.07)	1997-2004	-3.13 (-3.39 to -2.38)	1997-2010	-2.13 (-2.23 to -2.01)
	2			2011-2019	-0.70 (-0.80 to -0.37)	2016-2019	-2.51 (-3.01 to -0.89)	2012-2021	-0.96 (-1.17 to -0.86)	2004-2010	-3.61 (-4.29 to -1.91)	2010-2019	-1.27 (-1.39 to -1.07)
	3			2019-2021	-1.55 (-1.90 to -0.90)	2019-2021	-6.14 (-7.41 to -4.44)			2010-2019	-1.57 (-1.77 to -1.24)	2019-2021	-5.01 (-5.82 to -3.85)
	4									2019-2021	-7.63 (-8.76 to -6.01)		
Bulgaria	0	1990-1995	0.71 (0.63 to 0.80)	1990-1994	1.03 (0.89 to 1.19)	1990-1997	0.51 (-0.08 to 1.19)	1990-1994	1.14 (0.99 to 1.28)	1990-1997	0.85 (0.12 to 1.67)	1990-1997	0.82 (0.41 to 1.27)
	1	1995-2000	-0.42 (-0.52 to -0.33)	1994-2001	-0.47 (-0.59 to -0.38)	1997-2002	-3.96 (-5.64 to -3.07)	1994-2000	-0.51 (-0.64 to -0.41)	1997-2003	-3.99 (-6.17 to -3.13)	1997-2002	-3.30 (-4.68 to -2.57)
	2	2000-2006	0.43 (0.33 to 0.51)	2001-2006	0.64 (-0.34 to 0.78)	2002-2006	-0.50 (-1.81 to 1.17)	2000-2006	0.52 (0.30 to 0.65)	2003-2006	2.25 (-0.38 to 3.73)	2002-2006	0.57 (-0.45 to 1.96)
	3	2006-2011	1.40 (1.22 to 1.49)	2006-2011	1.37 (0.75 to 1.51)	2006-2015	-4.33 (-5.53 to -3.92)	2006-2011	1.50 (0.88 to 1.64)	2006-2009	-6.69 (-7.76 to -4.29)	2006-2012	-2.71 (-4.10 to -2.14)
	4	2011-2015	1.90 (1.67 to 2.05)	2011-2015	1.76 (1.54 to 1.95)	2015-2021	-1.82 (-2.88 to 0.34)	2011-2015	2.03 (1.73 to 2.23)	2009-2021	-3.09 (-3.48 to -2.24)	2012-2021	-0.42 (-0.86 to 0.26)
	5	2015-2018	2.66 (2.45 to 2.80)	2015-2018	3.12 (2.89 to 3.30)			2015-2018	3.13 (2.88 to 3.31)				
	6	2018-2021	1.21 (1.03 to 1.38)	2018-2021	1.29 (1.04 to 1.49)			2018-2021	1.22 (1.00 to 1.43)				
Burkina Faso	0	1990-1994	-0.48 (-0.67 to -0.39)	1990-1995	-0.42 (-0.57 to -0.33)	1990-1997	-3.53 (-3.97 to -3.18)	1990-1997	-0.28 (-0.50 to -0.18)	1990-2001	-3.27 (-3.47 to -3.12)	1990-2001	-2.50 (-2.62 to -2.41)
	1	1994-1999	-0.16 (-0.32 to -0.07)	1995-2005	-0.01 (-0.10 to 0.04)	1997-2001	-4.78 (-5.46 to -3.13)	1997-2005	0.07 (-0.07 to 0.20)	2001-2010	-2.01 (-2.20 to -1.74)	2001-2010	-1.30 (-1.41 to -1.16)
	2	1999-2005	0.01 (-0.06 to 0.16)	2005-2010	0.43 (-0.02 to 0.55)	2001-2006	-1.62 (-4.61 to -0.81)	2005-2010	0.48 (0.31 to 0.75)	2010-2016	2.75 (2.48 to 3.04)	2010-2016	1.71 (1.56 to 1.85)
	3	2005-2010	0.29 (0.24 to 0.40)	2010-2015	-0.76 (-0.88 to 0.44)	2006-2010	-2.37 (-2.85 to 2.19)	2010-2014	-0.87 (-1.14 to -0.60)	2016-2021	-2.27 (-2.66 to -1.91)	2016-2021	-1.61 (-1.84 to -1.41)
	4	2010-2015	-0.76 (-0.85 to -0.71)	2015-2019	-0.16 (-0.78 to -0.08)	2010-2016	2.29 (-1.62 to 2.65)	2014-2021	-0.18 (-0.27 to -0.02)				
	5	2015-2019	-0.21 (-0.43 to -0.09)	2019-2021	0.28 (-0.05 to 0.46)	2016-2021	-2.11 (-2.51 to -1.79)						
	6	2019-2021	0.30 (0.06 to 0.44)										
Burundi	0	1990-1997	-0.32 (-0.78 to -0.17)	1990-1994	-0.22 (-0.84 to 0.12)	1990-1996	0.49 (0.28 to 0.73)	1990-1996	-0.26 (-1.00 to 0.01)	1990-1994	0.74 (0.23 to 1.41)	1990-1994	0.48 (0.12 to 0.97)
	1	1997-2012	0.04 (-0.01 to 0.15)	1994-2011	0.14 (-1.24 to 0.40)	1996-2001	-1.63 (-2.13 to -1.38)	1996-2011	0.19 (0.12 to 0.39)	1994-2001	-1.31 (-2.01 to -1.09)	1994-2001	-0.99 (-1.44 to -0.82)
	2	2012-2021	-0.81 (-0.96 to -0.70)	2011-2021	-1.01 (-1.25 to -0.76)	2001-2005	-0.69 (-0.99 to -0.20)	2011-2021	-0.85 (-0.99 to -0.72)	2001-2005	-0.05 (-0.47 to 0.57)	2001-2005	0.03 (-0.31 to 0.50)
	3					2005-2008	-2.23 (-2.58 to -1.65)			2005-2008	-1.74 (-2.16 to -1.12)	2005-2008	-1.26 (-1.58 to -0.79)
	4					2008-2013	-0.14 (-0.40 to 0.31)			2008-2013	0.18 (-0.13 to 0.80)	2008-2013	0.09 (-0.11 to 0.51)
	5					2013-2021	-1.85 (-2.02 to -1.70)			2013-2021	-2.55 (-2.73 to -2.38)	2013-2021	-2.13 (-2.26 to -2.02)
Cabo Verde	0	1990-1996	-0.18 (-0.40 to -0.09)	1990-1996	-0.26 (-0.47 to -0.17)	1990-1996	0.40 (-0.08 to 1.08)	1990-1996	-0.18 (-0.53 to -0.05)	1990-1997	0.75 (0.28 to 1.32)	1990-1996	0.58 (0.22 to 1.04)
	1	1996-2004	0.13 (0.04 to 0.25)	1996-2004	0.13 (0.03 to 0.23)	1996-2021	-1.64 (-1.70 to -1.58)	1996-2006	0.18 (0.09 to 0.28)	1997-2021	-2.04 (-2.11 to -1.97)	1996-2021	-1.05 (-1.10 to -1.01)
	2	2004-2011	0.46 (0.37 to 0.69)	2004-2012	0.54 (0.45 to 0.65)			2006-2009	0.97 (0.59 to 1.12)				
	3	2011-2019	0.17 (-0.04 to 0.23)	2012-2019	0.05 (-0.15 to 0.31)			2009-2021	0.17 (0.11 to 0.22)				
	4	2019-2021	0.72 (0.29 to 0.91)	2019-2021	0.62 (0.16 to 0.84)								
Cambodia	0	1990-2001	-0.21 (-0.35 to -0.12)	1990-2001	-0.24 (-0.39 to -0.12)	1990-1996	-0.47 (-0.67 to -0.22)	1990-2002	-0.15 (-0.26 to -0.05)	1990-1996	-0.38 (-0.67 to -0.02)	1990-1999	-0.62 (-0.84 to -0.34)
	1	2001-2015	0.16 (0.10 to 0.28)	2001-2014	0.46 (0.36 to 0.60)	1996-2001	-1.62 (-1.92 to -1.27)	2002-2014	0.53 (0.43 to 0.68)	1996-2002	-1.72 (-2.05 to -1.30)	1999-2009	-2.05 (-2.30 to -1.90)
	2	2015-2021	-0.83 (-1.17 to -0.60)	2014-2021	-0.91 (-1.23 to -0.66)	2001-2009	-2.68 (-2.83 to -2.57)	2014-2021	-0.75 (-1.03 to -0.53)	2002-2009	-2.86 (-3.21 to -2.08)	2009-2021	-0.97 (-1.07 to -0.85)
	3					2009-2015	-1.31 (-1.62 to -1.18)			2009-2016	-1.47 (-2.84 to -1.31)		
	4					2015-2019	-0.51 (-0.75 to -0.19)			2016-2019	-0.40 (-1.50 to -0.14)		
	5					2019-2021	-1.64 (-2.14 to -1.12)			2019-2021	-1.80 (-2.40 to -0.91)		
Cameroon	0	1990-2000	-0.30 (-0.31 to -0.30)	1990-1995	-0.32 (-0.35 to -0.26)	1990-1997	-1.32 (-1.63 to -1.01)	1990-1995	-0.28 (-0.54 to -0.17)	1990-2008	-1.15 (-1.26 to -1.08)	1990-2008	-0.89 (-0.96 to -0.84)
	1	2000-2005	0.02 (0.00 to 0.04)	1995-1999	-0.39 (-0.43 to -0.20)	1997-2000	-2.24 (-2.53 to -0.92)	1995-2014	-0.08 (-0.10 to -0.06)	2008-2014	-0.38 (-0.77 to 0.44)	2008-2014	-0.29 (-0.56 to 0.30)
	2	2005-2010	0.33 (0.31 to 0.35)	1999-2005	-0.07 (-0.09 to -0.05)	2000-2008	-0.90 (-1.84 to -0.57)	2014-2021	0.74 (0.69 to 0.78)	2014-2021	-2.79 (-3.14 to -2.49)	2014-2021	-1.76 (-2.01 to -1.56)
	3	2010-2015	0.04 (0.00 to 0.06)	2005-2010	0.43 (0.41 to 0.45)	2008-2014	-0.40 (-2.50 to 0.10)						
	4	2015-2019	0.21 (0.17 to 0.26)	2010-2014	0.04 (0.01 to 0.08)	2014-2021	-2.53 (-2.79 to -2.22)						
	5	2019-2021	0.60 (0.52 to 0.67)	2014-2019	0.24 (0.22 to 0.28)								
	6			2019-2021	0.70 (0.63 to 0.77)								
Canada	0	1990-2000	2.28 (2.25 to 2.30)	1990-2000	2.36 (2.33 to 2.39)	1990-1994	1.79 (1.09 to 2.89)	1990-1995	2.30 (2.13 to 2.37)	1990-1994	1.43 (0.47 to 3.29)	1990-1995	1.91 (1.69 to 2.19)
	1	2000-2008	0.95 (0.91 to 0.99)	2000-2008	0.97 (0.92 to 1.00)	1994-2002	-0.98 (-1.31 to -0.70)	1995-1999	2.49 (2.38 to 2.60)	1994-2002	-0.62 (-1.33 to -0.23)	1995-2002	0.93 (0.71 to 1.06)
	2	2008-2011	1.34 (1.17 to 1.76)	2008-2011	1.30 (1.14 to 1.98)	2002-2009	-4.43 (-4.74 to -4.08)	1999-2002	1.26 (1.09 to 1.35)	2002-2009	-3.67 (-4.22 to -3.12)	2002-2013	-0.56 (-0.64 to -0.48)
	3	2011-2019	2.19 (2.15 to 2.25)	2011-2019	2.16 (2.11 to 2.23)	2009-2013	-7.40 (-8.15 to -6.84)	2002-2009	0.96 (0.88 to 1.01)	2009-2013	-7.21 (-8.40 to -6.33)	2013-2018	2.41 (2.18 to 2.74)
	4	2019-2021	0.87 (0.61 to 1.32)	2019-2021	0.93 (0.65 to 1.38)	2013-2018	0.77 (0.34 to 1.34)	2009-2012	1.60 (1.43 to 1.97)	2013-2018	2.33 (1.64 to 3.45)	2018-2021	0.44 (-0.25 to 0.97)
	5					2018-2021	-2.59 (-4.01 to -1.72)	2012-2018	2.29 (2.24 to 2.36)	2018-2021	-2.31 (-4.35 to -0.94)		
	6							2018-2021	1.23 (1.12 to 1.32)				
Central African Republic	0	1990-1995	-0.14 (-0.21 to -0.10)	1990-1994	-0.33 (-0.39 to -0.27)	1990-1999	0.34 (-0.02 to 1.16)	1990-1994	-0.38 (-0.79 to -0.19)	1990-2021	-0.18 (-0.25 to -0.11)	1990-2021	-0.13 (-0.18 to -0.07)
	1	1995-2000	0.14 (0.09 to 0.24)	1994-2000	0.17 (0.13 to 0.22)	1999-2021	-0.37 (-0.52 to -0.27)	1994-2021	0.08 (0.07 to 0.10)				
	2	2000-2005	-0.02 (-0.11 to 0.02)	2000-2006	-0.10 (-0.19 to -0.06)								
	3	2005-2016	0.09 (0.07 to 0.10)	2006-2015	0.05 (0.03 to 0.08)								
	4	2016-2019	0.25 (0.17 to 0.31)	2015-2019	0.25 (0.19 to 0.36)								
	5	2019-2021	-0.38 (-0.50 to -0.22)	2019-2021	-0.40 (-0.52 to -0.24)								
Chad	0	1990-1994	-0.40 (-0.52 to -0.35)	1990-1994	-0.46 (-0.59 to -0.38)	1990-1995	-0.99 (-2.10 to -0.44)	1990-1994	-0.40 (-0.76 to -0.19)	1990-1995	-0.54 (-2.12 to 0.11)	1990-1995	-0.50 (-1.64 to -0.00)
	1	1994-2005	-0.10 (-0.12 to -0.09)	1994-2005	-0.12 (-0.14 to -0.10)	1995-2009	0.50 (0.37 to 0.76)	1994-2005	-0.12 (-0.17 to 0.16)	1995-2008	0.58 (0.41 to 1.33)	1995-2008	0.42 (0.30 to 0.96)
	2	2005-2010	0.19 (0.16 to 0.30)	2005-2021	0.19 (0.18 to 0.21)	2009-2021	-0.41 (-0.62 to -0.24)	2005-2021	0.20 (0.17 to 0.24)	2008-2021	-0.42 (-0.65 to -0.24)	2008-2021	-0.26 (-0.43 to -0.12)
	3	2010-2015	0.06 (-0.03 to 0.10)										
	4	2015-2021	0.20 (0.16 to 0.30)										
Chile	0	1990-1999	0.62 (0.48 to 0.75)	1990-2000	0.66 (0.65 to 0.67)	1990-1995	-0.11 (-0.74 to 0.76)	1990-2002	0.69 (0.64 to 0.74)	1990-1995	-0.34 (-1.01 to 0.68)	1990-1995	0.03 (-0.32 to 0.50)

Location	Segment	Incidence		Prevalence		Mortality		YLD		YLL		DALY	
		Period	APC (95%CI)	Period	APC (95%CI)	Period	APC (95%CI)	Period	APC (95%CI)	Period	APC (95%CI)	Period	APC (95%CI)
	1	1999-2005	0.96 (0.48 to 1.07)	2000-2003	1.06 (0.67 to 1.10)	1995-1998	-6.73 (-7.46 to -4.91)	2002-2008	1.31 (1.05 to 1.48)	1995-1998	-6.13 (-6.90 to -4.43)	1995-1998	-3.83 (-4.34 to -2.78)
	2	2005-2008	1.51 (0.90 to 1.64)	2003-2006	1.29 (1.14 to 1.42)	1998-2008	-3.25 (-3.91 to -2.83)	2008-2014	1.81 (1.69 to 2.13)	1998-2008	-3.19 (-3.75 to -2.69)	1998-2006	-1.43 (-1.83 to -0.99)
	3	2008-2011	1.13 (0.97 to 1.36)	2006-2011	1.52 (1.47 to 1.56)	2008-2012	-1.43 (-2.53 to -0.31)	2014-2021	1.17 (1.06 to 1.26)	2008-2016	-1.93 (-2.36 to -0.29)	2006-2021	-0.33 (-0.48 to -0.12)
	4	2011-2014	2.33 (2.03 to 2.49)	2011-2014	1.76 (1.71 to 1.81)	2012-2021	-3.33 (-4.05 to -3.02)			2016-2021	-3.94 (-5.53 to -3.15)		
	5	2014-2019	0.89 (0.69 to 0.99)	2014-2019	1.04 (1.01 to 1.06)								
	6	2019-2021	1.57 (1.15 to 1.81)	2019-2021	1.68 (1.59 to 1.76)								
China	0	1990-1999	-0.44 (-0.59 to -0.28)	1990-2000	-0.30 (-0.44 to -0.16)	1990-2001	-1.34 (-1.47 to -1.14)	1990-1995	-0.94 (-1.87 to -0.56)	1990-2001	-1.83 (-1.97 to -1.63)	1990-2003	-1.63 (-1.74 to -1.49)
	1	1999-2012	1.34 (1.25 to 1.52)	2000-2010	1.55 (1.40 to 1.76)	2001-2004	-3.47 (-3.97 to -2.61)	1995-2001	0.13 (-0.29 to 0.78)	2001-2004	-3.27 (-3.71 to -1.87)	2003-2007	-5.69 (-6.31 to -4.93)
	2	2012-2021	0.86 (0.46 to 1.02)	2010-2021	0.66 (0.49 to 0.78)	2004-2007	-7.87 (-8.30 to -7.07)	2001-2004	2.85 (1.85 to 3.27)	2004-2007	-8.18 (-8.67 to -3.76)	2007-2015	-4.06 (-4.35 to -3.59)
	3					2007-2014	-5.63 (-5.83 to -5.27)	2004-2010	1.32 (0.78 to 1.82)	2007-2010	-4.90 (-7.50 to -4.40)	2015-2021	-1.04 (-1.49 to -0.33)
	4					2014-2021	-2.58 (-2.87 to -2.13)	2010-2016	0.32 (-0.53 to 0.67)	2010-2014	-6.15 (-6.70 to -3.57)		
	5							2016-2021	1.74 (1.30 to 2.71)	2014-2021	-2.57 (-2.88 to -2.11)		
Colombia	0	1990-1994	-0.38 (-0.59 to -0.26)	1990-1994	-0.32 (-0.57 to -0.17)	1990-1994	1.64 (0.54 to 2.88)	1990-1995	-0.23 (-0.53 to -0.10)	1990-1994	1.70 (0.19 to 4.13)	1990-1993	1.73 (0.60 to 3.48)
	1	1994-2000	0.05 (-0.04 to 0.15)	1994-2000	0.11 (0.00 to 0.25)	1994-2011	-2.33 (-2.48 to -2.20)	1995-2001	0.28 (0.04 to 0.44)	1994-2010	-2.38 (-2.65 to -2.18)	1993-2010	-1.38 (-1.52 to -1.28)
	2	2000-2009	0.51 (0.48 to 0.57)	2000-2010	0.56 (0.53 to 0.60)	2011-2019	0.67 (0.26 to 1.29)	2001-2009	0.59 (0.51 to 0.84)	2010-2019	0.77 (0.32 to 1.51)	2010-2019	0.33 (0.06 to 0.98)
	3	2009-2012	-0.01 (-0.34 to 0.30)	2010-2018	-0.52 (-0.57 to -0.48)	2019-2021	-3.99 (-7.18 to -0.16)	2009-2012	0.00 (-0.38 to 0.42)	2019-2021	-5.71 (-9.81 to -0.31)	2019-2021	-3.09 (-4.92 to -0.79)
	4	2012-2018	-0.62 (-0.77 to -0.57)	2018-2021	0.09 (-0.14 to 0.46)			2012-2018	-0.62 (-0.86 to -0.52)				
	5	2018-2021	0.04 (-0.17 to 0.37)					2018-2021	0.42 (0.15 to 0.82)				
Comoros	0	1990-2000	0.06 (0.01 to 0.11)	1990-2001	0.23 (0.03 to 0.30)	1990-1997	0.81 (-0.44 to 3.25)	1990-2002	0.22 (0.16 to 0.26)	1990-1997	0.87 (-0.77 to 4.71)	1990-1997	0.64 (-0.49 to 3.29)
	1	2000-2010	0.43 (0.37 to 0.50)	2001-2010	0.47 (0.36 to 0.77)	1997-2012	-2.72 (-3.43 to -2.33)	2002-2010	0.51 (0.38 to 0.68)	1997-2012	-2.94 (-4.11 to -2.44)	1997-2012	-2.03 (-2.80 to -1.70)
	2	2010-2019	-0.36 (-0.40 to 0.31)	2010-2021	-0.63 (-0.70 to -0.55)	2012-2015	8.36 (3.46 to 10.63)	2010-2019	-0.47 (-0.53 to 0.46)	2012-2015	9.75 (3.61 to 12.76)	2012-2015	6.57 (2.48 to 8.52)
	3	2019-2021	-0.91 (-1.13 to -0.44)			2015-2021	-1.96 (-4.87 to -0.60)	2019-2021	-1.08 (-1.37 to -0.55)	2015-2021	-2.22 (-6.70 to -0.42)	2015-2021	-1.70 (-4.65 to -0.52)
Congo	0	1990-2000	-0.40 (-0.46 to -0.33)	1990-1999	-0.43 (-0.51 to -0.37)	1990-1992	-1.64 (-3.52 to 1.25)	1990-2001	-0.35 (-0.45 to -0.27)	1990-1992	-1.77 (-4.14 to 2.01)	1990-1992	-1.50 (-3.25 to 1.26)
	1	2000-2012	0.41 (0.35 to 0.48)	1999-2005	0.19 (-0.01 to 0.34)	1992-1997	4.11 (3.07 to 5.81)	2001-2013	0.56 (0.49 to 0.70)	1992-1997	4.70 (3.35 to 6.88)	1992-1997	3.43 (2.44 to 5.09)
	2	2012-2019	0.08 (-0.05 to 0.35)	2005-2011	0.66 (0.55 to 0.95)	1997-2002	-2.94 (-4.59 to -1.96)	2013-2021	0.08 (-0.11 to 0.20)	1997-2002	-3.29 (-5.34 to -2.01)	1997-2002	-2.54 (-4.12 to -1.58)
	3	2019-2021	-0.74 (-1.05 to -0.16)	2011-2019	0.19 (0.10 to 0.31)	2002-2005	1.48 (-0.27 to 2.35)			2002-2005	2.38 (0.03 to 3.48)	2002-2005	1.95 (0.20 to 2.77)
	4			2019-2021	-0.66 (-0.96 to -0.17)	2005-2021	-1.79 (-1.97 to -1.65)			2005-2021	-1.86 (-2.08 to -1.68)	2005-2021	-1.26 (-1.42 to -1.13)
Cook Islands	0	1990-2000	-0.20 (-0.22 to -0.17)	1990-2000	-0.20 (-0.22 to -0.18)	1990-2000	-3.92 (-4.53 to -3.42)	1990-2000	-0.20 (-0.25 to -0.16)	1990-2000	-3.89 (-4.79 to -3.34)	1990-1999	-3.34 (-3.85 to -2.91)
	1	2000-2010	0.27 (0.24 to 0.29)	2000-2010	0.34 (0.32 to 0.36)	2000-2003	5.72 (-2.90 to 6.94)	2000-2009	0.38 (0.33 to 0.45)	2000-2003	3.89 (0.48 to 5.32)	1999-2004	1.18 (0.13 to 3.07)
	2	2010-2015	-0.80 (-0.88 to -0.74)	2010-2015	-0.92 (-0.97 to -0.87)	2003-2015	-3.98 (-4.52 to 4.16)	2009-2012	-0.32 (-0.44 to 0.26)	2003-2018	-3.69 (-4.05 to -3.43)	2004-2015	-3.05 (-3.51 to -2.79)
	3	2015-2021	-0.35 (-0.41 to -0.26)	2015-2019	-0.29 (-0.38 to -0.16)	2015-2019	-0.93 (-3.78 to 0.70)	2012-2015	-1.17 (-1.31 to -0.93)	2018-2021	12.94 (8.87 to 21.84)	2015-2019	-0.66 (-1.96 to 0.63)
	4			2019-2021	-0.75 (-0.93 to -0.54)	2019-2021	10.46 (5.35 to 13.83)	2015-2018	-0.06 (-0.25 to 0.08)			2019-2021	12.49 (8.35 to 15.85)
	5							2018-2021	-0.59 (-0.86 to -0.45)				
Costa Rica	0	1990-1994	-0.69 (-0.75 to -0.63)	1990-1994	-0.67 (-0.75 to -0.60)	1990-1996	2.85 (1.47 to 4.73)	1990-1994	-0.66 (-1.11 to -0.43)	1990-1996	2.53 (0.71 to 5.06)	1990-1996	1.72 (0.84 to 2.99)
	1	1994-2000	-0.19 (-0.23 to -0.15)	1994-2000	-0.14 (-0.18 to -0.09)	1996-2005	-2.13 (-2.95 to 1.50)	1994-2002	-0.02 (-0.15 to 0.12)	1996-2005	-2.16 (-3.19 to 2.87)	1996-2009	-1.68 (-2.28 to -1.35)
	2	2000-2005	0.29 (0.24 to 0.38)	2000-2005	0.41 (0.35 to 0.52)	2005-2008	-5.53 (-6.97 to 1.90)	2002-2006	0.63 (0.41 to 0.89)	2005-2008	-5.83 (-7.48 to 3.09)	2009-2021	0.72 (0.30 to 1.30)
	3	2005-2010	0.12 (0.05 to 0.18)	2005-2010	0.19 (0.11 to 0.26)	2008-2021	1.02 (-0.28 to 1.90)	2006-2016	-0.07 (-0.12 to -0.01)	2008-2021	1.40 (-0.63 to 2.68)		
	4	2010-2014	-0.18 (-0.27 to -0.10)	2010-2014	-0.17 (-0.27 to -0.07)			2016-2019	-0.78 (-0.99 to -0.52)				
	5	2014-2019	-0.47 (-0.54 to -0.43)	2014-2019	-0.48 (-0.55 to -0.43)			2019-2021	1.57 (1.06 to 1.93)				
	6	2019-2021	1.41 (1.26 to 1.54)	2019-2021	1.50 (1.34 to 1.63)								
Croatia	0	1990-1992	2.26 (2.05 to 2.45)	1990-1994	2.08 (1.93 to 2.21)	1990-1995	-2.63 (-3.50 to -1.47)	1990-1995	2.04 (1.85 to 2.21)	1990-1995	-2.82 (-3.91 to -0.93)	1990-1995	-1.44 (-2.14 to -0.29)
	1	1992-1995	1.55 (1.27 to 1.65)	1994-2003	0.90 (0.81 to 0.95)	1995-2005	-5.08 (-5.89 to -4.72)	1995-2001	0.79 (0.50 to 0.94)	1995-2005	-4.89 (-6.47 to -4.41)	1995-2004	-2.82 (-3.95 to -2.44)
	2	1995-2002	0.80 (0.74 to 0.85)	2003-2006	1.46 (1.04 to 1.96)	2005-2010	-2.03 (-2.81 to -0.22)	2001-2006	1.33 (1.04 to 1.72)	2005-2010	-1.16 (-2.32 to 1.06)	2004-2021	-0.16 (-0.39 to 0.08)
	3	2002-2006	1.30 (1.21 to 1.45)	2006-2010	2.44 (2.19 to 2.79)	2010-2013	-8.46 (-9.37 to -6.67)	2006-2010	2.50 (1.84 to 2.84)	2010-2013	-8.18 (-9.29 to -5.88)		
	4	2006-2010	2.32 (2.17 to 2.42)	2010-2018	3.03 (2.97 to 3.13)	2013-2021	-2.49 (-2.96 to -1.80)	2010-2018	3.08 (3.00 to 3.29)	2013-2021	-2.80 (-3.42 to -1.79)		
	5	2010-2018	2.91 (2.87 to 2.96)	2018-2021	1.52 (1.28 to 1.78)			2018-2021	1.60 (1.28 to 1.97)				
	6	2018-2021	1.69 (1.54 to 1.84)										
Cuba	0	1990-1998	-1.04 (-1.27 to -0.85)	1990-1999	-1.10 (-1.28 to -0.95)	1990-1994	1.23 (-0.05 to 3.28)	1990-1999	-1.02 (-1.29 to -0.81)	1990-1994	1.41 (-0.36 to 4.72)	1990-1994	0.57 (-0.34 to 2.02)
	1	1998-2010	0.18 (0.06 to 0.35)	1999-2010	0.14 (0.00 to 0.37)	1994-1999	-5.13 (-6.42 to -3.34)	1999-2012	0.14 (0.01 to 0.31)	1994-1999	-5.00 (-7.16 to -2.82)	1994-1998	-3.56 (-5.23 to -2.12)
	2	2010-2015	-1.10 (-1.69 to -0.37)	2010-2015	-1.21 (-1.81 to -0.35)	1999-2002	-9.03 (-9.97 to -4.99)	2012-2021	-2.39 (-2.67 to -2.16)	1999-2002	-9.98 (-11.13 to -3.32)	1998-2002	-6.34 (-7.80 to -1.83)
	3	2015-2018	-3.94 (-4.42 to -3.11)	2015-2018	-3.91 (-4.40 to -3.06)	2002-2014	-2.91 (-3.46 to -2.54)			2002-2013	-3.18 (-4.03 to -2.55)	2002-2021	-1.90 (-2.11 to -1.70)
	4	2018-2021	-1.52 (-2.15 to -0.22)	2018-2021	-1.52 (-2.13 to -0.25)	2014-2021	-1.02 (-1.89 to 0.96)			2013-2021	-1.12 (-2.07 to 1.02)		
Cyprus	0	1990-1997	2.18 (2.04 to 2.44)	1990-1997	2.29 (2.17 to 2.51)	1990-1992	2.11 (-1.21 to 4.83)	1990-1997	2.35 (2.21 to 2.58)	1990-2000	-1.27 (-2.08 to -0.26)	1990-2000	-0.38 (-0.67 to -0.04)
	1	1997-2005	1.71 (1.49 to 1.81)	1997-2005	1.82 (1.66 to 1.90)	1992-2001	-3.65 (-4.28 to -3.26)	1997-2005	1.76 (1.58 to 1.85)	2000-2011	-7.03 (-7.83 to -6.49)	2000-2008	-3.46 (-4.09 to -3.07)
	2	2005-2015	3.42 (3.34 to 3.50)	2005-2015	3.42 (3.36 to 3.48)	2001-2008	-7.64 (-8.45 to -7.01)	2005-2015	3.44 (3.37 to 3.51)	2011-2021	-3.49 (-4.14 to -2.66)	2008-2014	-0.16 (-1.24 to 0.68)
	3	2015-2019	5.54 (5.21 to 5.99)	2015-2019	5.44 (5.20 to 5.71)	2008-2016	-4.10 (-5.94 to -3.68)	2015-2019	5.56 (5.31 to 5.82)			2014-2021	2.21 (1.61 to 3.27)
	4	2019-2021	2.23 (1.53 to 3.13)	2019-2021	2.13 (1.58 to 2.80)	2016-2019	-0.69 (-3.93 to 0.18)	2019-2021	1.97 (1.48 to 2.63)				
	5					2019-2021	-5.97 (-8.03 to -2.87)						
Czechia	0	1990-1994	1.07 (0.89 to 1.38)	1990-1994	1.14 (0.92 to 1.53)	1990-1996	-9.92 (-11.19 to -9.07)	1990-1994	1.25 (1.02 to 1.61)	1990-1996	-8.92 (-10.36 to -8.00)	1990-1992	-4.29 (-5.95 to -3.07)
	1	1994-2001	0.51 (0.24 to 0.59)	1994-2002	0.60 (0.39 to 0.68)	1996-2005	-2.15 (-2.64 to -0.62)	1994-2001	0.61 (0.30 to 0.71)	1996-2008	-2.37 (-6.95 to -1.75)	1992-1996	-6.60 (-7.26 to -0.68)
	2	2001-2005	1.21 (0.87 to 1.61)	2002-2006	1.54 (1.17 to 1.88)	2005-2021	-3.77 (-4.12 to -3.53)	2001-2005	1.12 (0.91 to 1.71)	2008-2014	-4.26 (-6.32 to -1.26)	1996-2021	-0.74 (-0.86 to -0.60)
	3	2005-2010	2.54 (2.39 to 2.78)	2006-2009	3.17 (2.71 to 3.38)			2005-2009	2.91 (2.63 to 3.17)	2014-2021	-2.28 (-3.42 to 0.45)		

Location	Segment	Incidence		Prevalence		Mortality		YLD		YLL		DALY	
		Period	APC (95%CI)	Period	APC (95%CI)	Period	APC (95%CI)	Period	APC (95%CI)	Period	APC (95%CI)	Period	APC (95%CI)
	4	2010-2015	0.93 (0.66 to 1.08)	2009-2021	1.12 (1.07 to 1.17)			2009-2021	1.30 (1.24 to 1.34)				
	5	2015-2021	1.43 (1.31 to 1.75)										
CÃ□Â´te d'Ivoire	0	1990-1999	-0.11 (-0.21 to -0.08)	1990-1997	-0.19 (-0.40 to -0.12)	1990-1992	-4.32 (-5.12 to -2.97)	1990-2002	-0.06 (-0.12 to -0.00)	1990-1992	-4.46 (-5.79 to -2.09)	1990-1992	-3.00 (-3.58 to -1.97)
	1	1999-2005	0.08 (-0.04 to 0.22)	1997-2005	0.04 (-0.04 to 0.20)	1992-2000	-1.18 (-1.48 to -0.78)	2002-2010	0.48 (0.38 to 0.67)	1992-2011	-0.44 (-0.54 to -0.31)	1992-2002	-0.43 (-0.67 to -0.25)
	2	2005-2010	0.33 (0.21 to 0.47)	2005-2010	0.51 (0.38 to 0.73)	2000-2006	0.59 (-0.90 to 1.24)	2010-2014	-0.40 (-0.73 to -0.12)	2011-2015	3.41 (2.42 to 4.74)	2002-2007	0.30 (-0.41 to 0.89)
	3	2010-2015	-0.28 (-0.40 to -0.18)	2010-2015	-0.30 (-0.50 to -0.18)	2006-2011	-0.95 (-1.44 to 0.67)	2014-2021	0.43 (0.32 to 0.63)	2015-2021	-3.75 (-4.24 to -3.33)	2007-2011	-0.91 (-1.47 to 0.26)
	4	2015-2021	0.45 (0.38 to 0.52)	2015-2021	0.47 (0.37 to 0.61)	2011-2014	4.05 (-1.07 to 4.55)					2011-2014	3.31 (-0.86 to 3.78)
	5					2014-2017	-2.24 (-2.70 to 4.41)					2014-2017	-1.37 (-1.81 to 3.43)
	6					2017-2021	-3.42 (-4.28 to -2.91)					2017-2021	-2.48 (-3.16 to -2.07)
Democratic People's Republic of Korea	0	1990-2000	-0.17 (-0.20 to -0.15)	1990-2001	-0.17 (-0.19 to -0.14)	1990-1992	-0.44 (-0.70 to 0.10)	1990-2000	-0.21 (-0.27 to -0.15)	1990-1993	-0.27 (-0.70 to 0.01)	1990-1992	-0.51 (-0.77 to -0.06)
	1	2000-2009	0.89 (0.86 to 0.91)	2001-2009	1.10 (1.07 to 1.14)	1992-2000	0.34 (0.28 to 0.49)	2000-2009	1.06 (0.99 to 1.16)	1993-2000	0.31 (0.24 to 0.46)	1992-2000	0.22 (0.16 to 0.33)
	2	2009-2021	0.29 (0.27 to 0.31)	2009-2021	0.27 (0.24 to 0.29)	2000-2006	-1.17 (-1.27 to -1.08)	2009-2021	0.31 (0.26 to 0.36)	2000-2006	-1.24 (-1.33 to -1.16)	2000-2006	-0.93 (-1.01 to -0.84)
	3					2006-2009	0.58 (0.34 to 0.73)			2006-2009	1.31 (1.13 to 1.50)	2006-2009	1.25 (1.09 to 1.42)
	4					2009-2021	-1.35 (-1.39 to -1.31)			2009-2014	-1.59 (-1.82 to -1.46)	2009-2014	-1.31 (-1.51 to -1.19)
	5									2014-2021	-1.12 (-1.22 to -0.96)	2014-2021	-0.90 (-0.98 to -0.75)
Democratic Republic of the Congo	0	1990-1994	-0.49 (-0.61 to -0.43)	1990-1994	-0.49 (-0.66 to -0.40)	1990-1997	-1.32 (-1.66 to -1.02)	1990-1994	-0.56 (-0.90 to -0.39)	1990-1997	-1.66 (-2.05 to -1.32)	1990-1997	-1.32 (-1.65 to -1.05)
	1	1994-2000	-0.31 (-0.37 to -0.25)	1994-2000	-0.31 (-0.34 to 0.14)	1997-2003	1.07 (0.68 to 1.79)	1994-2001	-0.19 (-0.29 to 0.05)	1997-2002	1.49 (0.88 to 2.63)	1997-2003	0.90 (0.55 to 1.52)
	2	2000-2006	0.23 (-0.32 to 0.25)	2000-2011	0.24 (0.22 to 0.26)	2003-2009	-1.07 (-1.94 to -0.73)	2001-2010	0.30 (0.22 to 0.40)	2002-2010	-1.03 (-1.62 to -0.77)	2003-2010	-0.85 (-1.41 to -0.60)
	3	2006-2011	0.30 (0.22 to 0.43)	2011-2021	0.58 (0.56 to 0.61)	2009-2015	0.17 (-0.17 to 1.03)	2010-2021	0.64 (0.60 to 0.70)	2010-2015	0.47 (-0.06 to 1.45)	2010-2015	0.56 (0.16 to 1.38)
	4	2011-2015	0.46 (0.35 to 0.65)			2015-2021	-1.08 (-1.92 to -0.74)			2015-2021	-1.29 (-2.02 to -0.90)	2015-2021	-0.80 (-1.35 to -0.51)
	5	2015-2021	0.62 (0.55 to 0.71)										
Denmark	0	1990-1995	1.19 (0.97 to 1.34)	1990-1995	1.20 (0.98 to 1.35)	1990-1993	-0.37 (-1.44 to 1.46)	1990-1994	1.14 (0.81 to 1.34)	1990-2002	-2.62 (-3.06 to -2.09)	1990-1993	0.21 (-0.28 to 1.13)
	1	1995-2004	1.78 (1.70 to 1.92)	1995-2004	1.81 (1.75 to 1.93)	1993-1996	-4.37 (-5.11 to -3.03)	1994-2001	1.73 (1.64 to 1.96)	2002-2016	-5.80 (-6.60 to -5.44)	1993-1996	-2.28 (-2.72 to -1.53)
	2	2004-2015	1.12 (1.05 to 1.53)	2004-2014	1.14 (1.07 to 1.24)	1996-2002	-2.42 (-5.23 to -1.21)	2001-2019	1.10 (1.08 to 1.13)	2016-2021	-2.11 (-3.98 to 1.68)	1996-2002	-0.03 (-0.28 to 0.68)
	3	2015-2019	0.26 (0.10 to 1.13)	2014-2019	0.41 (0.22 to 0.94)	2002-2016	-5.24 (-5.50 to -4.99)	2019-2021	-0.68 (-0.99 to -0.10)			2002-2005	-2.65 (-3.09 to -1.77)
	4	2019-2021	-0.72 (-1.10 to -0.04)	2019-2021	-0.63 (-1.03 to -0.00)	2016-2021	-2.13 (-2.97 to -0.89)					2005-2015	-1.25 (-1.50 to -0.89)
	5											2015-2021	-0.04 (-0.54 to 1.22)
Djibouti	0	1990-1994	-0.26 (-0.74 to 0.00)	1990-2010	0.16 (0.12 to 0.38)	1990-1996	2.77 (1.86 to 4.79)	1990-1993	-0.39 (-1.01 to 0.08)	1990-1996	3.28 (2.10 to 5.79)	1990-1996	2.29 (1.40 to 4.29)
	1	1994-2014	0.10 (0.08 to 0.15)	2010-2015	-0.19 (-0.85 to 0.13)	1996-2007	0.49 (-0.22 to 0.96)	1993-2011	0.18 (0.15 to 0.48)	1996-2008	0.41 (-0.33 to 0.98)	1996-2008	0.37 (-0.24 to 0.75)
	2	2014-2021	-0.65 (-0.82 to -0.52)	2015-2021	-1.17 (-1.72 to -0.91)	2007-2021	-2.02 (-2.46 to -1.70)	2011-2016	-0.27 (-0.70 to 0.11)	2008-2021	-2.53 (-3.16 to -2.06)	2008-2021	-1.89 (-2.31 to -1.57)
	3							2016-2021	-1.13 (-1.66 to -0.89)				
Dominica	0	1990-1997	-0.68 (-0.97 to -0.50)	1990-1996	-0.87 (-1.35 to -0.59)	1990-1992	1.66 (0.72 to 2.40)	1990-1996	-0.82 (-1.26 to -0.57)	1990-1992	1.40 (0.49 to 2.16)	1990-1997	-0.21 (-0.42 to 0.01)
	1	1997-2011	-0.10 (-0.17 to 0.13)	1996-2012	-0.19 (-0.27 to 0.18)	1992-1997	-0.38 (-0.78 to -0.18)	1996-2012	-0.18 (-0.25 to 0.21)	1992-1997	-0.45 (-0.91 to -0.26)	1997-2001	-2.88 (-3.50 to -2.29)
	2	2011-2019	-0.59 (-0.90 to -0.23)	2012-2019	-0.66 (-1.04 to -0.23)	1997-2002	-3.15 (-3.40 to -2.92)	2012-2019	-0.63 (-1.00 to -0.26)	1997-2002	-3.31 (-3.56 to -3.05)	2001-2010	-0.32 (-0.70 to -0.15)
	3	2019-2021	-2.34 (-2.99 to -1.13)	2019-2021	-2.46 (-3.15 to -1.11)	2002-2006	-0.06 (-0.34 to 0.40)	2019-2021	-2.48 (-3.13 to -1.30)	2002-2006	0.20 (-0.17 to 0.65)	2010-2014	0.79 (0.26 to 1.36)
	4					2006-2009	-1.66 (-1.96 to -1.07)			2006-2010	-0.75 (-1.35 to -0.37)	2014-2021	-0.21 (-0.67 to -0.01)
	5					2009-2021	0.24 (0.15 to 0.35)			2010-2013	1.67 (0.90 to 2.09)		
	6									2013-2021	0.16 (-0.09 to 0.31)		
Dominican Republic	0	1990-1994	-0.14 (-0.25 to 0.04)	1990-2000	-0.40 (-0.41 to -0.38)	1990-1993	-0.94 (-1.89 to 0.49)	1990-2000	-0.37 (-0.42 to -0.33)	1990-1993	-0.94 (-1.91 to 0.54)	1990-2001	-1.73 (-1.92 to -1.56)
	1	1994-1999	-0.34 (-0.50 to 0.03)	2000-2005	0.16 (0.12 to 0.21)	1993-2001	-2.63 (-3.24 to -2.40)	2000-2010	0.13 (0.08 to 0.18)	1993-2001	-2.62 (-3.31 to -2.38)	2001-2007	0.51 (0.10 to 1.53)
	2	1999-2010	0.08 (0.04 to 0.12)	2005-2010	0.02 (-0.06 to 0.06)	2001-2006	1.45 (-0.09 to 2.12)	2010-2014	-0.69 (-0.86 to -0.52)	2001-2007	0.75 (0.29 to 1.54)	2007-2010	-1.96 (-2.53 to -0.74)
	3	2010-2015	-0.36 (-0.48 to 0.05)	2010-2015	-0.46 (-0.50 to -0.42)	2006-2011	-2.05 (-2.81 to 0.93)	2014-2021	0.38 (0.31 to 0.45)	2007-2011	-2.36 (-3.49 to -1.64)	2010-2018	1.34 (1.08 to 1.75)
	4	2015-2019	0.35 (-0.33 to 0.41)	2015-2019	0.27 (0.19 to 0.32)	2011-2014	2.76 (-1.73 to 3.38)			2011-2014	4.21 (3.01 to 4.99)	2018-2021	-4.01 (-5.22 to -2.82)
	5	2019-2021	0.78 (0.50 to 0.94)	2019-2021	0.78 (0.62 to 0.88)	2014-2018	0.85 (-0.18 to 1.55)			2014-2019	0.37 (-0.27 to 0.81)		
	6					2018-2021	-4.28 (-5.59 to -3.40)			2019-2021	-8.25 (-9.74 to -6.56)		
Ecuador	0	1990-1997	-0.06 (-0.25 to 0.03)	1990-1995	-0.06 (-0.45 to 0.09)	1990-1992	8.63 (5.94 to 10.74)	1990-1996	-0.08 (-0.49 to 0.08)	1990-1992	8.82 (6.13 to 11.02)	1990-2002	2.21 (1.89 to 2.63)
	1	1997-2010	0.22 (0.18 to 0.27)	1995-2009	0.25 (0.21 to 0.31)	1992-1998	1.65 (0.38 to 2.09)	1996-2010	0.30 (0.23 to 0.39)	1992-1998	1.88 (0.58 to 2.43)	2002-2012	-0.03 (-0.51 to 0.50)
	2	2010-2014	0.89 (0.65 to 1.15)	2009-2014	0.83 (0.64 to 1.16)	1998-2001	5.37 (3.29 to 6.21)	2010-2015	0.83 (0.53 to 1.21)	1998-2001	4.97 (0.28 to 5.87)	2012-2021	-2.36 (-3.17 to -1.87)
	3	2014-2021	0.27 (-0.01 to 0.38)	2014-2021	0.35 (-0.03 to 0.47)	2001-2012	0.11 (-0.34 to 0.49)	2015-2021	0.34 (-0.12 to 0.53)	2001-2012	-0.16 (-0.70 to 0.28)		
	4					2012-2021	-3.19 (-3.98 to -2.65)			2012-2021	-3.57 (-4.42 to -2.97)		
Egypt	0	1990-1994	-0.62 (-1.21 to -0.30)	1990-1994	-0.49 (-1.09 to -0.12)	1990-2000	-2.20 (-2.87 to -1.68)	1990-1997	-0.33 (-0.84 to -0.15)	1990-2000	-2.62 (-3.31 to -2.11)	1990-2000	-1.72 (-2.07 to -1.43)
	1	1994-2010	-0.14 (-0.17 to 0.06)	1994-2015	-0.06 (-0.09 to 0.06)	2000-2006	7.23 (6.55 to 8.06)	1997-2009	0.09 (0.02 to 0.47)	2000-2006	5.54 (4.70 to 6.97)	2000-2006	3.56 (3.04 to 4.41)
	2	2010-2015	-0.52 (-1.03 to -0.30)	2015-2021	1.71 (1.47 to 2.12)	2006-2015	0.55 (0.25 to 0.82)	2009-2015	-0.46 (-1.05 to -0.26)	2006-2014	0.36 (-0.05 to 0.83)	2006-2014	0.09 (-0.16 to 0.42)
	3	2015-2021	0.83 (0.64 to 1.12)			2015-2021	-3.48 (-4.11 to -2.84)	2015-2021	1.53 (1.30 to 1.87)	2014-2021	-3.03 (-3.63 to -2.52)	2014-2021	-1.52 (-1.94 to -1.22)
El Salvador	0	1990-1995	0.26 (0.02 to 0.37)	1990-1994	0.34 (0.04 to 0.51)	1990-1994	1.04 (-0.78 to 4.94)	1990-2004	0.53 (0.47 to 0.60)	1990-1994	0.60 (-1.06 to 4.11)	1990-1994	0.49 (-0.71 to 3.06)
	1	1995-2003	0.45 (0.21 to 0.67)	1994-2004	0.56 (0.28 to 0.72)	1994-2003	-2.11 (-3.54 to -1.63)	2004-2021	0.24 (0.19 to 0.28)	1994-2002	-2.52 (-4.33 to -1.96)	1994-2002	-1.76 (-3.14 to -1.32)
	2	2003-2021	0.17 (0.15 to 0.19)	2004-2018	0.18 (0.12 to 0.20)	2003-2021	-0.15 (-0.40 to 0.13)			2002-2021	-0.35 (-0.59 to -0.08)	2002-2021	-0.18 (-0.35 to 0.02)
	3			2018-2021	0.57 (0.35 to 0.90)								
Equatorial Guinea	0	1990-1993	-0.38 (-0.55 to -0.30)	1990-1993	-0.41 (-0.60 to -0.32)	1990-1996	-0.69 (-1.18 to -0.15)	1990-1996	-0.20 (-0.29 to -0.10)	1990-1996	-0.60 (-1.17 to 0.09)	1990-1996	-0.52 (-0.94 to 0.03)
	1	1993-1996	0.05 (-0.11 to 0.26)	1993-1996	0.02 (-0.06 to 0.22)	1996-2002	-4.08 (-4.93 to -3.59)	1996-2011	0.97 (0.94 to 1.00)	1996-2002	-4.05 (-5.19 to -3.49)	1996-2002	-2.90 (-3.73 to -2.49)
	2	1996-2001	0.47 (0.40 to 0.53)	1996-2001	0.73 (0.64 to 0.80)	2002-2011	-2.05 (-2.31 to -1.47)	2011-2021	0.38 (0.31 to 0.44)	2002-2011	-2.28 (-2.59 to -1.36)	2002-2011	-1.32 (-1.53 to -0.81)
	3	2001-2009	0.77 (0.74 to 0.81)	2001-2009	0.95 (0.92 to 1.01)	2011-2014	-5.35 (-6.07 to -3.83)			2011-2014	-5.96 (-6.84 to -4.26)	2011-2014	-3.74 (-4.32 to -2.55)

Location	Segment	Incidence		Prevalence		Mortality		YLD		YLL		DALY	
		Period	APC (95%CI)	Period	APC (95%CI)	Period	APC (95%CI)	Period	APC (95%CI)	Period	APC (95%CI)	Period	APC (95%CI)
	4	2009-2014	0.54 (0.49 to 0.61)	2009-2013	0.61 (0.51 to 0.73)	2014-2021	-0.46 (-0.87 to 0.11)			2014-2021	-0.43 (-0.91 to 0.30)	2014-2021	-0.12 (-0.44 to 0.36)
	5	2014-2019	0.34 (0.24 to 0.38)	2013-2021	0.38 (0.34 to 0.41)								
	6	2019-2021	0.67 (0.52 to 0.78)										
Eritrea	0	1990-2005	0.22 (0.19 to 0.25)	1990-2005	0.38 (0.34 to 0.42)	1990-1995	-1.30 (-1.78 to -1.03)	1990-2005	0.34 (0.30 to 0.38)	1990-1996	-0.98 (-2.06 to -0.55)	1990-1998	-0.47 (-0.70 to -0.29)
	1	2005-2019	-0.05 (-0.08 to -0.01)	2005-2019	-0.20 (-0.25 to -0.15)	1995-2001	0.12 (-0.36 to 0.42)	2005-2019	-0.11 (-0.15 to -0.06)	1996-2001	0.48 (-0.40 to 1.58)	1998-2007	1.10 (0.94 to 1.34)
	2	2019-2021	-0.73 (-0.96 to -0.29)	2019-2021	-1.33 (-1.66 to -0.70)	2001-2004	1.86 (1.12 to 2.19)	2019-2021	-1.29 (-1.67 to -0.74)	2001-2006	1.86 (-0.43 to 2.79)	2007-2021	-0.31 (-0.40 to -0.23)
	3					2004-2008	0.58 (-0.31 to 0.97)			2006-2021	-0.31 (-0.46 to -0.16)		
	4					2008-2021	-0.46 (-0.55 to -0.40)						
Estonia	0	1990-1995	0.13 (-0.18 to 0.30)	1990-1995	0.22 (0.08 to 0.36)	1990-1994	6.56 (3.27 to 12.12)	1990-1995	0.25 (-0.21 to 0.52)	1990-1994	7.64 (3.98 to 13.05)	1990-1994	5.38 (3.32 to 6.87)
	1	1995-2005	1.19 (1.11 to 1.46)	1995-2003	1.34 (1.27 to 1.44)	1994-2002	-4.02 (-5.24 to -2.56)	1995-2003	1.38 (1.24 to 1.80)	1994-2007	-5.53 (-6.05 to -4.43)	1994-2011	-3.55 (-3.86 to -3.33)
	2	2005-2010	0.90 (0.42 to 2.42)	2003-2011	0.64 (0.53 to 0.74)	2002-2011	-8.23 (-10.19 to -7.36)	2003-2011	0.83 (0.55 to 0.96)	2007-2011	-9.68 (-12.88 to -6.70)	2011-2017	7.64 (5.94 to 11.18)
	3	2010-2021	2.46 (2.33 to 2.62)	2011-2021	2.35 (2.27 to 2.43)	2011-2017	8.83 (7.04 to 12.61)	2011-2021	2.53 (2.43 to 2.64)	2011-2016	14.90 (12.01 to 19.29)	2017-2021	0.38 (-3.41 to 2.64)
	4					2017-2021	-1.08 (-6.68 to 2.14)			2016-2021	0.23 (-3.67 to 3.26)		
Eswatini	0	1990-2000	0.21 (0.19 to 0.23)	1990-1995	-0.08 (-0.15 to -0.05)	1990-1993	-1.30 (-2.13 to -0.76)	1990-2000	-0.02 (-0.13 to 0.04)	1990-1993	-1.04 (-2.13 to -0.33)	1990-1993	-0.63 (-1.30 to -0.21)
	1	2000-2010	-0.05 (-0.06 to -0.03)	1995-2000	0.06 (0.02 to 0.13)	1993-1998	1.02 (0.77 to 1.32)	2000-2008	-0.23 (-0.42 to 0.08)	1993-1998	0.99 (0.70 to 1.55)	1993-1998	0.54 (0.36 to 0.96)
	2	2010-2019	0.52 (0.50 to 0.53)	2000-2004	-0.24 (-0.30 to -0.18)	1998-2005	5.73 (5.53 to 5.93)	2008-2011	0.20 (-0.24 to 0.55)	1998-2005	5.29 (5.05 to 5.55)	1998-2005	3.09 (2.94 to 3.27)
	3	2019-2021	-0.27 (-0.40 to -0.13)	2004-2008	-0.09 (-0.16 to -0.02)	2005-2008	-0.20 (-0.94 to 0.33)	2011-2015	0.68 (0.41 to 0.88)	2005-2009	-0.40 (-0.96 to 0.49)	2005-2008	0.06 (-0.47 to 1.18)
	4			2008-2011	0.14 (0.09 to 0.19)	2008-2021	-2.86 (-2.97 to -2.77)	2015-2021	0.24 (0.12 to 0.32)	2009-2021	-2.69 (-2.84 to -2.56)	2008-2021	-1.46 (-1.54 to -1.40)
	5			2011-2015	0.78 (0.74 to 0.81)								
	6			2015-2021	0.22 (0.19 to 0.24)								
Ethiopia	0	1990-1998	-0.66 (-0.96 to -0.47)	1990-1998	-0.33 (-1.20 to -0.04)	1990-1995	-1.11 (-1.43 to -0.72)	1990-1997	-0.43 (-1.06 to -0.16)	1990-1995	-1.13 (-1.46 to -0.82)	1990-1995	-1.08 (-1.32 to -0.77)
	1	1998-2012	0.24 (0.14 to 0.35)	1998-2011	0.38 (0.22 to 0.76)	1995-2001	-3.03 (-3.21 to -2.86)	1997-2012	0.35 (0.25 to 0.51)	1995-2001	-3.18 (-3.41 to -2.99)	1995-2000	-2.50 (-2.76 to -2.20)
	2	2012-2021	-1.46 (-1.68 to -1.27)	2011-2021	-1.61 (-1.85 to -1.39)	2001-2012	-5.14 (-5.20 to -5.08)	2012-2021	-1.54 (-1.79 to -1.33)	2001-2012	-4.93 (-4.98 to -4.87)	2000-2012	-3.59 (-3.64 to -3.55)
	3					2012-2015	-2.52 (-2.80 to -2.20)			2012-2016	-2.34 (-2.66 to -2.19)	2012-2016	-2.00 (-2.22 to -1.89)
	4					2015-2021	-1.24 (-1.39 to -1.01)			2016-2019	-0.68 (-1.06 to -0.40)	2016-2019	-0.86 (-1.13 to -0.67)
	5									2019-2021	-2.43 (-2.97 to -1.90)	2019-2021	-2.45 (-2.81 to -2.09)
Fiji	0	1990-2000	-0.49 (-0.74 to -0.36)	1990-2000	-0.50 (-0.82 to -0.35)	1990-1997	1.39 (1.04 to 1.81)	1990-2000	-0.50 (-0.84 to -0.35)	1990-1997	1.18 (0.78 to 1.75)	1990-1997	0.79 (0.49 to 1.12)
	1	2000-2006	0.23 (-0.07 to 0.85)	2000-2006	0.31 (-0.05 to 1.13)	1997-2005	-0.74 (-1.35 to -0.52)	2000-2006	0.34 (-0.05 to 1.19)	1997-2009	-0.48 (-0.65 to -0.29)	1997-2005	-0.53 (-1.11 to -0.17)
	2	2006-2019	-0.28 (-0.52 to -0.17)	2006-2019	-0.50 (-0.71 to -0.37)	2005-2008	0.82 (-0.13 to 1.31)	2006-2019	-0.47 (-0.75 to -0.32)	2009-2012	-2.92 (-3.43 to -1.79)	2005-2008	0.53 (-0.38 to 0.92)
	3	2019-2021	-3.37 (-4.26 to -1.74)	2019-2021	-4.22 (-5.30 to -2.18)	2008-2012	-2.91 (-3.68 to -2.28)	2019-2021	-4.21 (-5.11 to -2.49)	2012-2021	-0.94 (-1.22 to -0.34)	2008-2012	-2.24 (-2.85 to -1.50)
	4					2012-2021	-1.00 (-1.25 to -0.60)					2012-2019	-0.70 (-0.95 to 0.12)
	5											2019-2021	-2.73 (-3.92 to -1.30)
Finland	0	1990-1995	1.34 (1.19 to 1.47)	1990-1995	1.35 (1.23 to 1.46)	1990-1993	-2.64 (-4.82 to 0.39)	1990-1995	1.35 (1.17 to 1.48)	1990-1995	-3.72 (-4.98 to -2.01)	1990-2000	-0.73 (-1.01 to -0.59)
	1	1995-1999	2.52 (2.37 to 2.77)	1995-1999	2.57 (2.42 to 2.70)	1993-1999	-10.27 (-11.50 to -9.51)	1995-1999	2.50 (2.31 to 2.76)	1995-1999	-10.33 (-12.61 to -8.50)	2000-2004	0.59 (-0.06 to 1.24)
	2	1999-2003	0.98 (0.81 to 1.16)	1999-2003	1.02 (0.89 to 1.18)	1999-2008	-2.65 (-3.07 to -1.63)	1999-2003	1.04 (0.83 to 1.23)	1999-2008	-1.77 (-2.33 to -0.86)	2004-2013	-0.57 (-1.06 to -0.39)
	3	2003-2011	0.09 (-0.04 to 0.16)	2003-2011	0.07 (-0.02 to 0.17)	2008-2016	-4.29 (-6.46 to -3.73)	2003-2011	0.07 (-0.01 to 0.14)	2008-2016	-4.74 (-6.64 to -4.05)	2013-2021	1.69 (1.40 to 2.06)
	4	2011-2015	1.24 (0.52 to 1.70)	2011-2015	1.21 (0.07 to 1.54)	2016-2021	-1.16 (-2.43 to 1.51)	2011-2015	1.20 (1.00 to 1.38)	2016-2021	-0.76 (-2.23 to 1.86)		
	5	2015-2021	2.32 (2.08 to 2.79)	2015-2019	2.78 (1.28 to 3.24)			2015-2018	2.93 (2.67 to 3.11)				
	6			2019-2021	1.55 (0.90 to 2.43)			2018-2021	1.87 (1.54 to 2.05)				
France	0	1990-1995	1.33 (1.22 to 1.42)	1990-1996	1.41 (1.35 to 1.46)	1990-1997	-2.54 (-3.27 to -2.23)	1990-1995	1.34 (1.14 to 1.51)	1990-1998	-2.69 (-3.18 to -2.38)	1990-1997	-0.44 (-0.57 to -0.32)
	1	1995-2002	1.86 (1.81 to 2.01)	1996-1999	2.04 (1.88 to 2.11)	1997-2002	-1.10 (-1.77 to 0.05)	1995-2000	2.41 (2.24 to 2.76)	1998-2002	0.15 (-1.03 to 1.45)	1997-2003	1.11 (0.95 to 1.34)
	2	2002-2010	1.72 (1.62 to 1.75)	1999-2010	1.79 (1.74 to 1.81)	2002-2014	-4.58 (-4.80 to -4.39)	2000-2009	1.71 (1.59 to 1.78)	2002-2013	-4.35 (-4.78 to -4.10)	2003-2006	-1.36 (-1.61 to -0.88)
	3	2010-2015	2.48 (2.36 to 2.56)	2010-2015	2.46 (2.35 to 2.53)	2014-2021	-2.73 (-3.13 to -2.20)	2009-2015	2.38 (2.21 to 2.56)	2013-2021	-2.85 (-3.26 to -2.18)	2006-2013	0.22 (0.04 to 0.43)
	4	2015-2019	3.26 (3.10 to 3.48)	2015-2019	3.24 (3.09 to 3.43)			2015-2019	3.30 (3.11 to 3.65)			2013-2021	1.68 (1.52 to 1.88)
	5	2019-2021	2.09 (1.76 to 2.53)	2019-2021	2.07 (1.80 to 2.46)			2019-2021	1.93 (1.50 to 2.45)				
Gabon	0	1990-1994	-0.53 (-0.81 to -0.40)	1990-1994	-0.55 (-0.79 to -0.44)	1990-1993	-1.97 (-3.78 to -0.96)	1990-1996	-0.50 (-0.68 to -0.39)	1990-2003	0.74 (0.39 to 1.13)	1990-2003	0.46 (0.20 to 0.74)
	1	1994-2000	-0.19 (-0.30 to 0.01)	1994-2000	-0.19 (-0.27 to -0.08)	1993-1996	2.94 (1.78 to 3.67)	1996-2004	0.09 (-0.03 to 0.20)	2003-2021	-2.62 (-2.86 to -2.41)	2003-2021	-1.63 (-1.80 to -1.47)
	2	2000-2006	0.27 (-0.01 to 0.37)	2000-2007	0.32 (0.21 to 0.40)	1996-2003	0.08 (-0.36 to 0.41)	2004-2019	0.52 (0.49 to 0.56)				
	3	2006-2019	0.41 (0.38 to 0.65)	2007-2019	0.49 (0.47 to 0.56)	2003-2008	-1.81 (-2.47 to -1.11)	2019-2021	-0.53 (-0.84 to -0.08)				
	4	2019-2021	-0.60 (-0.87 to -0.18)	2019-2021	-0.52 (-0.74 to -0.14)	2008-2013	-3.53 (-4.55 to -2.93)						
	5					2013-2021	-1.90 (-2.23 to -1.23)						
Gambia	0	1990-1995	-0.25 (-0.52 to -0.14)	1990-1994	-0.44 (-0.82 to -0.28)	1990-2002	-1.82 (-3.59 to -1.16)	1990-1996	-0.36 (-0.56 to -0.21)	1990-1999	-0.75 (-2.02 to 0.51)	1990-1999	-0.59 (-1.24 to 0.40)
	1	1995-2004	0.13 (-0.05 to 0.19)	1994-2005	0.04 (-0.02 to 0.11)	2002-2006	3.28 (-0.05 to 6.72)	1996-2021	0.21 (0.19 to 0.23)	1999-2002	-4.99 (-6.94 to 1.97)	1999-2002	-3.34 (-4.62 to 1.39)
	2	2004-2021	0.21 (0.19 to 0.29)	2005-2011	0.34 (0.07 to 0.56)	2006-2011	-4.15 (-8.59 to -1.49)			2002-2005	7.24 (-4.95 to 9.62)	2002-2005	4.88 (-3.12 to 6.36)
	3			2011-2021	0.19 (0.08 to 0.24)	2011-2021	2.08 (0.96 to 4.42)			2005-2012	-3.95 (-5.95 to 6.22)	2005-2012	-2.59 (-3.87 to 3.13)
	4									2012-2019	3.48 (-3.85 to 6.74)	2012-2019	2.39 (-2.32 to 4.33)
	5									2019-2021	-4.05 (-8.77 to 2.35)	2019-2021	-2.73 (-5.66 to 1.51)
Georgia	0	1990-1994	0.55 (0.35 to 0.90)	1990-1994	0.64 (0.41 to 1.07)	1990-1992	-3.38 (-6.93 to 0.85)	1990-1994	0.68 (0.48 to 1.02)	1990-1992	0.75 (-3.36 to 7.40)	1990-1998	5.00 (3.72 to 6.40)
	1	1994-2006	0.17 (0.05 to 0.21)	1994-2005	0.12 (0.00 to 0.18)	1992-1995	11.57 (8.58 to 13.95)	1994-2005	0.15 (0.05 to 0.20)	1992-1995	11.42 (1.50 to 14.07)	1998-2006	-3.88 (-6.36 to -2.89)
	2	2006-2011	0.86 (0.54 to 1.13)	2005-2011	0.85 (0.61 to 1.08)	1995-2000	2.18 (0.21 to 3.67)	2005-2010	0.76 (0.47 to 1.06)	1995-2000	0.78 (-5.53 to 2.51)	2006-2009	5.83 (1.13 to 8.13)
	3	2011-2015	1.97 (1.54 to 2.36)	2011-2015	2.10 (1.63 to 2.53)	2000-2006	-5.35 (-6.89 to -4.48)	2010-2014	1.79 (1.40 to 2.26)	2000-2006	-5.64 (-7.72 to 4.02)	2009-2021	-2.25 (-3.11 to -1.73)
	4	2015-2021	2.79 (2.67 to 3.06)	2015-2021	3.05 (2.90 to 3.29)	2006-2009	5.70 (-2.41 to 7.57)	2014-2021	2.95 (2.85 to 3.09)	2006-2009	6.89 (-2.05 to 8.97)		

Location	Segment	Incidence		Prevalence		Mortality		YLD		YLL		DALY	
		Period	APC (95%CI)	Period	APC (95%CI)	Period	APC (95%CI)	Period	APC (95%CI)	Period	APC (95%CI)	Period	APC (95%CI)
	5					2009-2015	-2.87 (-4.18 to 1.96)			2009-2015	-2.32 (-4.47 to -0.15)		
	6					2015-2021	-5.69 (-8.86 to -4.56)			2015-2021	-6.00 (-9.24 to -4.69)		
Germany	0	1990-1992	1.53 (1.25 to 1.98)	1990-1995	2.02 (1.89 to 2.15)	1990-1994	-2.34 (-3.24 to -1.43)	1990-1995	1.83 (1.69 to 1.97)	1990-1994	-2.97 (-4.20 to -0.63)	1990-1994	-1.03 (-1.34 to -0.48)
	1	1992-1995	2.10 (2.00 to 3.24)	1995-2004	3.21 (3.16 to 3.26)	1994-2000	-7.11 (-8.08 to -6.59)	1995-2002	3.19 (3.12 to 3.34)	1994-1998	-6.74 (-8.31 to -5.18)	1994-1998	-2.08 (-2.68 to -1.64)
	2	1995-2004	3.11 (0.84 to 3.19)	2004-2011	0.89 (0.80 to 0.95)	2000-2003	-3.50 (-5.03 to -2.73)	2002-2005	2.55 (2.01 to 2.80)	1998-2007	-4.43 (-5.00 to -2.87)	1998-2004	0.18 (-0.19 to 0.91)
	3	2004-2011	0.88 (0.80 to 2.02)	2011-2014	2.05 (1.74 to 2.20)	2003-2007	-5.54 (-6.65 to -2.41)	2005-2010	0.81 (0.58 to 0.93)	2007-2021	-2.68 (-3.00 to -2.19)	2004-2008	-0.73 (-1.33 to 0.59)
	4	2011-2015	2.00 (1.08 to 2.23)	2014-2021	1.07 (0.92 to 1.16)	2007-2021	-2.83 (-3.19 to -2.57)	2010-2014	2.09 (1.88 to 2.40)			2008-2021	0.43 (0.14 to 0.64)
	5	2015-2021	1.19 (0.98 to 1.38)					2014-2021	1.08 (0.98 to 1.18)				
Ghana	0	1990-1994	-0.41 (-0.63 to -0.30)	1990-1993	-0.51 (-0.86 to -0.28)	1990-1997	-0.19 (-0.30 to -0.02)	1990-1994	-0.47 (-0.89 to -0.29)	1990-1997	-0.33 (-0.47 to -0.04)	1990-1997	-0.27 (-0.39 to -0.02)
	1	1994-2005	-0.03 (-0.07 to 0.01)	1993-2007	-0.03 (-0.06 to 0.01)	1997-2000	-1.34 (-1.57 to -0.84)	1994-1998	0.24 (0.06 to 0.48)	1997-2000	-1.46 (-1.76 to -0.86)	1997-2000	-0.91 (-1.14 to -0.47)
	2	2005-2014	0.21 (0.08 to 0.25)	2007-2021	0.32 (0.29 to 0.35)	2000-2007	0.28 (0.17 to 0.47)	1998-2006	-0.10 (-0.34 to -0.03)	2000-2007	0.12 (-0.03 to 0.41)	2000-2007	0.05 (-0.09 to 0.30)
	3	2014-2021	0.34 (0.28 to 0.50)			2007-2010	-2.53 (-2.78 to -2.14)	2006-2021	0.35 (0.32 to 0.38)	2007-2010	-3.54 (-3.86 to -2.96)	2007-2010	-2.13 (-2.38 to -1.47)
	4					2010-2017	-0.55 (-0.67 to -0.37)			2010-2017	-0.58 (-0.77 to -0.37)	2010-2017	-0.26 (-0.41 to -0.03)
	5					2017-2021	-2.84 (-3.17 to -2.42)			2017-2021	-3.38 (-3.83 to -2.83)	2017-2021	-1.90 (-2.26 to -1.55)
Greece	0	1990-1996	2.06 (1.92 to 2.18)	1990-1996	2.04 (1.91 to 2.14)	1990-1993	-2.29 (-3.15 to -0.93)	1990-2001	2.72 (2.45 to 2.81)	1990-2021	-3.20 (-3.30 to -3.10)	1990-2001	1.10 (1.03 to 1.15)
	1	1996-2005	2.89 (2.82 to 3.04)	1996-2004	2.92 (2.86 to 3.02)	1993-2001	-3.99 (-4.59 to -3.79)	2001-2004	3.25 (1.88 to 3.44)			2001-2004	2.46 (1.98 to 2.67)
	2	2005-2010	2.52 (2.27 to 2.67)	2004-2010	2.45 (2.30 to 2.55)	2001-2005	-1.98 (-2.70 to -1.16)	2004-2011	1.86 (1.64 to 3.77)			2004-2010	1.04 (0.75 to 1.18)
	3	2010-2015	3.35 (3.22 to 3.52)	2010-2014	3.43 (3.28 to 3.64)	2005-2014	-4.08 (-4.38 to -3.87)	2011-2014	4.05 (0.90 to 4.32)			2010-2015	2.37 (2.13 to 2.71)
	4	2015-2021	0.78 (0.66 to 0.89)	2014-2021	0.59 (0.50 to 0.67)	2014-2021	-1.99 (-2.34 to -1.58)	2014-2021	0.64 (0.47 to 0.79)			2015-2021	0.20 (-0.00 to 0.37)
Greenland	0	1990-1995	-0.03 (-0.15 to 0.04)	1990-1995	-0.21 (-0.33 to -0.13)	1990-2004	-2.64 (-2.92 to -2.27)	1990-1993	-0.34 (-0.69 to -0.08)	1990-2004	-2.58 (-2.84 to -2.25)	1990-1998	-0.78 (-1.02 to -0.68)
	1	1995-2000	0.35 (0.11 to 0.43)	1995-2000	0.19 (0.06 to 0.31)	2004-2013	-4.66 (-5.85 to -4.17)	1993-1999	0.17 (0.04 to 0.43)	2004-2014	-4.56 (-5.23 to -4.15)	1998-2005	-0.36 (-0.52 to -0.01)
	2	2000-2005	0.51 (0.45 to 0.60)	2000-2004	0.60 (0.49 to 0.72)	2013-2021	-0.58 (-1.29 to 0.36)	1999-2005	0.57 (0.42 to 0.77)	2014-2021	-0.10 (-0.93 to 1.09)	2005-2011	-1.26 (-1.67 to -1.03)
	3	2005-2010	-0.38 (-0.42 to -0.32)	2004-2011	-0.13 (-0.17 to -0.10)			2005-2011	-0.23 (-0.33 to -0.09)			2011-2015	0.01 (-0.93 to 0.39)
	4	2010-2015	0.57 (0.46 to 0.62)	2011-2019	0.77 (0.73 to 0.80)			2011-2021	0.84 (0.80 to 0.89)			2015-2021	0.76 (0.57 to 1.20)
	5	2015-2019	0.86 (0.70 to 0.97)	2019-2021	1.34 (1.08 to 1.49)								
	6	2019-2021	1.38 (1.16 to 1.51)										
Grenada	0	1990-1995	-0.74 (-1.01 to -0.60)	1990-1995	-0.84 (-1.17 to -0.69)	1990-1994	0.02 (-0.60 to 1.30)	1990-2000	-0.66 (-0.89 to -0.53)	1990-1996	-1.57 (-2.04 to -1.06)	1990-1996	-1.49 (-2.04 to -0.83)
	1	1995-2003	-0.43 (-0.50 to -0.30)	1995-2004	-0.51 (-0.58 to -0.38)	1994-1997	-4.26 (-5.16 to -2.49)	2000-2012	-0.17 (-0.33 to 0.02)	1996-2000	-8.21 (-8.74 to -7.08)	1996-2000	-6.11 (-7.66 to -5.00)
	2	2003-2011	0.00 (-0.07 to 0.12)	2004-2011	-0.02 (-0.13 to 0.16)	1997-2000	-8.85 (-9.58 to -7.81)	2012-2018	-1.78 (-2.31 to -0.23)	2000-2006	-0.56 (-1.38 to 0.23)	2000-2021	-1.04 (-1.15 to -0.92)
	3	2011-2015	-1.37 (-1.48 to -0.41)	2011-2015	-1.45 (-1.60 to -0.49)	2000-2007	-0.54 (-0.86 to 0.25)	2018-2021	-0.81 (-1.51 to -0.05)	2006-2016	-1.76 (-2.39 to -0.84)		
	4	2015-2018	-2.06 (-2.23 to -1.77)	2015-2018	-2.27 (-2.47 to -1.91)	2007-2016	-1.35 (-2.32 to -1.15)			2016-2019	1.60 (-1.76 to 2.54)		
	5	2018-2021	-0.64 (-0.85 to -0.32)	2018-2021	-0.62 (-0.87 to -0.24)	2016-2019	2.62 (1.23 to 3.46)			2019-2021	-3.65 (-5.85 to -0.16)		
	6					2019-2021	-3.05 (-5.24 to -0.91)						
Guam	0	1990-1995	-0.41 (-0.54 to -0.29)	1990-1995	-0.47 (-0.57 to -0.36)	1990-2000	-2.68 (-6.69 to -1.12)	1990-1994	-0.58 (-0.72 to -0.43)	1990-2001	-2.66 (-6.07 to -1.18)	1990-2001	-1.18 (-2.43 to -0.56)
	1	1995-2000	0.80 (0.64 to 0.91)	1995-2000	0.87 (0.71 to 0.97)	2000-2018	0.64 (0.05 to 2.27)	1994-2001	0.71 (0.62 to 0.79)	2001-2018	1.10 (0.35 to 2.92)	2001-2018	0.56 (0.23 to 1.27)
	2	2000-2014	-0.10 (-0.14 to -0.06)	2000-2013	-0.11 (-0.17 to -0.08)	2018-2021	-22.37 (-31.30 to -15.76)	2001-2011	-0.15 (-0.24 to -0.10)	2018-2021	-26.74 (-36.43 to -19.33)	2018-2021	-11.67 (-16.39 to -7.93)
	3	2014-2019	0.27 (-0.05 to 0.46)	2013-2019	0.18 (-0.03 to 0.32)			2011-2019	0.10 (0.00 to 0.20)				
	4	2019-2021	1.13 (0.62 to 1.41)	2019-2021	1.01 (0.52 to 1.28)			2019-2021	0.97 (0.53 to 1.23)				
Guatemala	0	1990-2000	1.81 (1.41 to 2.08)	1990-2000	2.01 (1.56 to 2.30)	1990-1992	10.07 (2.50 to 16.30)	1990-2000	1.96 (1.57 to 2.22)	1990-1992	7.13 (-1.59 to 15.20)	1990-1992	6.19 (-0.63 to 11.70)
	1	2000-2010	0.21 (-0.26 to 2.23)	2000-2011	0.41 (-0.09 to 2.46)	1992-2001	-1.83 (-5.59 to -0.88)	2000-2011	0.44 (0.04 to 2.38)	1992-2001	-2.29 (-7.97 to 4.60)	1992-2001	-1.50 (-5.27 to 2.11)
	2	2010-2019	1.61 (0.13 to 1.85)	2011-2019	1.85 (0.30 to 2.14)	2001-2021	0.94 (0.56 to 1.42)	2011-2019	1.84 (0.17 to 2.07)	2001-2021	1.29 (-5.15 to 2.14)	2001-2021	1.17 (0.44 to 1.67)
	3	2019-2021	3.26 (1.68 to 4.02)	2019-2021	3.63 (1.95 to 4.48)			2019-2021	3.24 (1.89 to 3.94)				
Guinea	0	1990-1994	-0.14 (-0.32 to -0.05)	1990-1995	-0.11 (-0.26 to -0.04)	1990-1994	-1.42 (-1.77 to -0.74)	1990-1996	-0.09 (-0.29 to -0.01)	1990-1994	-1.02 (-1.68 to 0.11)	1990-2002	-1.16 (-1.29 to -1.05)
	1	1994-2006	0.02 (0.00 to 0.05)	1995-2000	0.22 (0.15 to 0.36)	1994-2001	-2.17 (-2.44 to -2.02)	1996-2000	0.40 (0.24 to 0.60)	1994-2001	-1.99 (-2.80 to 0.95)	2002-2006	1.22 (0.65 to 2.01)
	2	2006-2010	0.25 (0.19 to 0.35)	2000-2005	-0.09 (-0.23 to -0.02)	2001-2007	1.02 (0.85 to 1.25)	2000-2004	-0.22 (-0.41 to -0.07)	2001-2006	1.23 (-0.82 to 2.18)	2006-2011	-0.73 (-1.47 to -0.37)
	3	2010-2015	-0.56 (-0.61 to -0.51)	2005-2010	0.29 (0.23 to 0.39)	2007-2010	-1.15 (-1.50 to -0.45)	2004-2011	0.22 (0.16 to 0.37)	2006-2011	-0.98 (-1.96 to 0.52)	2011-2015	0.57 (0.03 to 1.20)
	4	2015-2021	0.01 (-0.03 to 0.06)	2010-2015	-0.56 (-0.65 to -0.51)	2010-2015	0.73 (0.49 to 1.19)	2011-2015	-0.76 (-0.97 to -0.58)	2011-2015	1.00 (-1.29 to 1.78)	2015-2021	-1.64 (-1.97 to -1.36)
	5			2015-2021	0.02 (-0.03 to 0.08)	2015-2021	-1.94 (-2.14 to -1.75)	2015-2018	0.35 (0.14 to 0.50)	2015-2021	-2.28 (-2.67 to -1.87)		
	6							2018-2021	-0.35 (-0.66 to -0.20)				
Guinea-Bissau	0	1990-2000	-0.09 (-0.11 to -0.08)	1990-1994	-0.19 (-0.26 to -0.16)	1990-1994	-1.18 (-1.67 to -0.33)	1990-2001	-0.10 (-0.14 to -0.07)	1990-2000	-1.36 (-1.76 to -1.10)	1990-2000	-1.10 (-1.40 to -0.90)
	1	2000-2005	0.10 (0.04 to 0.14)	1994-2000	-0.11 (-0.13 to -0.08)	1994-2000	-2.00 (-2.65 to -0.06)	2001-2011	0.25 (0.21 to 0.30)	2000-2009	0.04 (-0.30 to 0.95)	2000-2009	0.08 (-0.17 to 0.66)
	2	2005-2010	0.21 (0.18 to 0.25)	2000-2005	0.15 (0.11 to 0.17)	2000-2008	0.37 (0.10 to 0.70)	2011-2014	-0.56 (-0.68 to -0.32)	2009-2021	-1.72 (-1.96 to -1.51)	2009-2021	-1.33 (-1.51 to -1.18)
	3	2010-2015	-0.24 (-0.27 to -0.22)	2005-2010	0.22 (0.20 to 0.26)	2008-2017	-1.09 (-1.26 to -0.76)	2014-2019	0.13 (0.07 to 0.33)				
	4	2015-2019	-0.01 (-0.04 to 0.05)	2010-2015	-0.27 (-0.29 to -0.26)	2017-2021	-2.08 (-2.99 to -1.61)	2019-2021	-0.60 (-0.84 to -0.31)				
	5	2019-2021	-0.23 (-0.31 to -0.14)	2015-2019	0.03 (0.01 to 0.06)								
	6			2019-2021	-0.27 (-0.34 to -0.18)								
Guyana	0	1990-2000	-0.78 (-0.92 to -0.64)	1990-2000	-0.96 (-1.08 to -0.85)	1990-2000	0.42 (0.11 to 0.88)	1990-1999	-0.98 (-1.18 to -0.84)	1990-1992	-2.43 (-4.45 to 1.08)	1990-1992	-2.09 (-3.66 to 0.57)
	1	2000-2010	-0.05 (-0.77 to 0.39)	2000-2019	-0.21 (-0.26 to -0.16)	2000-2003	-3.09 (-4.09 to -1.13)	1999-2019	-0.23 (-0.27 to -0.17)	1992-2000	1.52 (-2.67 to 3.86)	1992-2000	0.93 (-2.04 to 2.66)
	2	2010-2019	-0.34 (-0.64 to -0.14)	2019-2021	-2.82 (-3.52 to -1.52)	2003-2007	0.67 (-0.74 to 2.33)	2019-2021	-2.84 (-3.49 to -1.58)	2000-2003	-3.23 (-4.46 to 0.81)	2000-2003	-2.52 (-3.54 to 0.58)
	3	2019-2021	-2.42 (-3.10 to -1.15)			2007-2013	-3.86 (-5.32 to -3.13)			2003-2008	0.68 (-5.08 to 2.73)	2003-2008	0.47 (-3.84 to 2.02)
	4					2013-2021	-1.49 (-2.26 to 0.03)			2008-2013	-5.03 (-6.90 to -1.15)	2008-2013	-3.78 (-5.29 to -0.83)
	5									2013-2021	-1.24 (-2.55 to 0.68)	2013-2021	-1.08 (-2.04 to 0.28)

Location	Segment	Incidence		Prevalence		Mortality		YLD		YLL		DALY	
		Period	APC (95%CI)	Period	APC (95%CI)	Period	APC (95%CI)	Period	APC (95%CI)	Period	APC (95%CI)	Period	APC (95%CI)
Haiti	0	1990-1999	-0.28 (-0.59 to -0.18)	1990-1994	-0.61 (-1.24 to -0.26)	1990-1995	-1.21 (-1.62 to -0.42)	1990-1996	-0.42 (-1.03 to -0.20)	1990-1998	-1.61 (-2.82 to -1.09)	1990-1998	-1.40 (-2.55 to -0.95)
	1	1999-2012	-0.05 (-0.11 to 0.16)	1994-2012	-0.15 (-0.18 to -0.07)	1995-1998	-2.61 (-3.03 to -0.28)	1996-2012	-0.11 (-0.17 to 0.09)	1998-2021	-0.56 (-0.66 to -0.40)	1998-2021	-0.54 (-0.62 to -0.39)
	2	2012-2021	-0.80 (-0.95 to -0.69)	2012-2021	-0.99 (-1.16 to -0.86)	1998-2005	0.16 (-0.35 to 0.55)	2012-2021	-0.96 (-1.13 to -0.82)				
	3					2005-2010	-1.65 (-2.43 to -1.07)						
	4					2010-2013	0.36 (-0.56 to 0.81)						
	5					2013-2021	-0.86 (-1.24 to -0.68)						
Honduras	0	1990-1997	0.04 (-0.17 to 0.11)	1990-1999	0.12 (0.02 to 0.16)	1990-1997	0.85 (0.35 to 1.57)	1990-1999	0.09 (-0.11 to 0.17)	1990-1998	-0.09 (-0.50 to 0.49)	1990-1997	0.06 (-0.33 to 0.74)
	1	1997-2011	0.17 (0.15 to 0.23)	1999-2004	0.39 (0.28 to 0.57)	1997-2021	-0.67 (-0.81 to -0.56)	1999-2003	0.44 (0.23 to 0.65)	1998-2012	-1.43 (-2.74 to -1.22)	1997-2012	-0.87 (-1.58 to -0.73)
	2	2011-2021	1.05 (1.01 to 1.08)	2004-2011	0.16 (0.06 to 0.21)			2003-2011	0.17 (-0.09 to 1.18)	2012-2021	-0.44 (-0.90 to 0.91)	2012-2021	0.14 (-0.22 to 0.87)
	3			2011-2021	1.19 (1.15 to 1.23)			2011-2021	1.17 (1.08 to 1.29)				
Hungary	0	1990-1994	0.72 (0.62 to 0.88)	1990-1994	0.83 (0.69 to 1.09)	1990-1993	-1.18 (-2.82 to 1.54)	1990-1993	1.05 (0.74 to 1.50)	1990-1993	-1.09 (-2.70 to 1.68)	1990-1993	-0.61 (-1.26 to 0.20)
	1	1994-2000	0.32 (0.22 to 0.38)	1994-2001	0.48 (0.36 to 0.54)	1993-1996	-7.55 (-8.64 to -5.47)	1993-2001	0.55 (0.33 to 0.63)	1993-1996	-7.47 (-8.63 to -5.42)	1993-1996	-5.37 (-5.93 to -4.41)
	2	2000-2003	1.18 (0.85 to 1.29)	2001-2005	1.70 (1.59 to 1.80)	1996-2002	-4.58 (-5.28 to 0.20)	2001-2005	1.74 (1.45 to 2.21)	1996-2002	-4.60 (-5.30 to -0.83)	1996-2002	-2.89 (-3.37 to -2.08)
	3	2003-2006	2.06 (1.94 to 2.18)	2005-2010	3.03 (2.95 to 3.11)	2002-2006	-0.34 (-7.62 to 1.31)	2005-2010	3.15 (2.97 to 3.31)	2002-2007	-1.43 (-7.73 to 0.58)	2002-2007	0.00 (-0.64 to 1.37)
	4	2006-2009	3.25 (3.12 to 3.37)	2010-2021	1.44 (1.40 to 1.48)	2006-2013	-7.00 (-8.01 to -2.55)	2010-2021	1.50 (1.45 to 1.55)	2007-2012	-6.90 (-9.00 to -1.27)	2007-2013	-2.27 (-3.81 to -1.69)
	5	2009-2012	1.97 (1.79 to 2.07)			2013-2021	-2.78 (-3.72 to -1.73)			2012-2021	-2.39 (-4.68 to -0.84)	2013-2021	0.01 (-0.49 to 0.85)
	6	2012-2021	1.35 (1.30 to 1.39)										
Iceland	0	1990-1995	0.79 (0.55 to 0.96)	1990-1994	0.81 (0.31 to 1.09)	1990-2006	-3.86 (-4.09 to -3.76)	1990-1995	0.90 (0.66 to 1.08)	1990-2000	-4.06 (-4.32 to -3.87)	1990-1994	-1.14 (-1.67 to -0.85)
	1	1995-2000	1.75 (1.59 to 1.98)	1994-2000	1.74 (1.58 to 2.05)	2006-2012	-3.03 (-3.53 to -1.87)	1995-1999	2.17 (1.91 to 2.45)	2000-2003	-0.36 (-3.90 to 0.16)	1994-2003	-0.20 (-0.30 to -0.04)
	2	2000-2005	-0.77 (-0.92 to -0.63)	2000-2006	-0.02 (-0.24 to 0.20)	2012-2019	-4.97 (-5.89 to -4.59)	1999-2006	-0.02 (-0.13 to 0.08)	2003-2006	-7.92 (-8.47 to -0.59)	2003-2006	-2.20 (-2.47 to -1.69)
	3	2005-2010	1.13 (0.71 to 1.27)	2006-2015	1.42 (0.85 to 1.53)	2019-2021	3.11 (0.14 to 5.08)	2006-2015	1.47 (1.40 to 1.55)	2006-2012	-2.67 (-7.82 to -2.11)	2006-2014	0.52 (0.40 to 0.69)
	4	2010-2015	1.65 (1.49 to 1.96)	2015-2018	-0.20 (-0.46 to 1.41)			2015-2018	-0.35 (-0.56 to 0.13)	2012-2016	-5.85 (-6.82 to -2.71)	2014-2018	-0.91 (-1.47 to -0.50)
	5	2015-2018	-0.81 (-1.07 to -0.47)	2018-2021	0.86 (0.35 to 1.46)			2018-2021	0.77 (0.45 to 1.31)	2016-2019	-3.48 (-5.40 to -2.66)	2018-2021	1.05 (0.47 to 2.02)
	6	2018-2021	0.58 (0.30 to 1.16)							2019-2021	6.08 (3.65 to 7.83)		
India	0	1990-2015	0.24 (0.23 to 0.26)	1990-2004	0.23 (0.02 to 0.47)	1990-1996	0.22 (-0.58 to 2.39)	1990-1992	2.10 (1.61 to 2.56)	1990-1996	0.19 (-0.72 to 2.27)	1990-1996	0.45 (-0.13 to 1.59)
	1	2015-2021	0.81 (0.68 to 0.98)	2004-2016	0.31 (0.17 to 0.48)	1996-2003	-1.77 (-3.43 to -1.17)	1992-1995	0.80 (-0.57 to 1.03)	1996-2021	-1.33 (-1.44 to -1.24)	1996-2003	-1.38 (-2.51 to -0.97)
	2			2016-2021	0.60 (0.39 to 0.99)	2003-2021	-0.63 (-0.75 to -0.46)	1995-2000	-1.14 (-1.42 to -0.94)			2003-2021	-0.80 (-0.88 to -0.54)
	3							2000-2005	-0.07 (-0.33 to 0.30)				
	4							2005-2013	1.06 (0.95 to 1.19)				
	5							2013-2021	0.19 (0.09 to 0.28)				
Indonesia	0	1990-1993	0.48 (0.44 to 0.55)	1990-1994	0.35 (0.24 to 0.54)	1990-1994	0.03 (-0.47 to 0.30)	1990-1993	0.50 (0.40 to 0.60)	1990-1994	0.00 (-0.83 to 0.38)	1990-1994	0.13 (-0.41 to 0.38)
	1	1993-1996	0.32 (0.22 to 0.37)	1994-2001	-0.06 (-0.13 to -0.00)	1994-2000	1.23 (1.13 to 1.41)	1993-2003	0.14 (0.12 to 0.16)	1994-2000	1.17 (1.04 to 1.46)	1994-2000	0.84 (0.75 to 1.04)
	2	1996-2000	0.12 (0.08 to 0.15)	2001-2015	0.32 (0.31 to 0.35)	2000-2012	-1.80 (-1.88 to -1.76)	2003-2011	0.33 (0.29 to 0.36)	2000-2012	-1.87 (-2.02 to -1.82)	2000-2012	-1.22 (-1.28 to -1.18)
	3	2000-2011	0.21 (0.20 to 0.22)	2015-2021	0.17 (0.03 to 0.23)	2012-2015	-1.09 (-1.50 to -0.92)	2011-2015	0.52 (0.46 to 0.60)	2012-2015	-1.19 (-1.66 to -0.99)	2012-2015	-0.53 (-0.87 to -0.39)
	4	2011-2014	0.36 (0.31 to 0.39)			2015-2021	-2.08 (-2.29 to -1.96)	2015-2019	0.29 (0.21 to 0.35)	2015-2021	-2.32 (-2.57 to -2.18)	2015-2021	-1.44 (-1.60 to -1.35)
	5	2014-2019	0.24 (0.22 to 0.27)					2019-2021	-0.22 (-0.33 to -0.08)				
	6	2019-2021	0.04 (-0.01 to 0.11)										
Iran (Islamic Republic of)	0	1990-1996	-0.30 (-0.32 to -0.27)	1990-1995	-0.11 (-0.17 to -0.02)	1990-2002	-2.07 (-2.32 to -1.82)	1990-1996	-0.19 (-0.24 to -0.11)	1990-2002	-2.32 (-2.54 to -2.10)	1990-2002	-1.29 (-1.39 to -1.20)
	1	1996-2000	-0.44 (-0.49 to -0.41)	1995-2000	-0.35 (-0.42 to -0.31)	2002-2006	3.85 (2.75 to 5.49)	1996-2000	-0.42 (-0.52 to -0.34)	2002-2006	3.94 (2.98 to 5.24)	2002-2006	2.34 (1.87 to 2.76)
	2	2000-2010	0.18 (0.17 to 0.19)	2000-2011	0.34 (0.32 to 0.36)	2006-2017	-0.60 (-0.91 to -0.31)	2000-2004	0.55 (0.48 to 0.65)	2006-2014	-0.45 (-0.86 to 0.06)	2006-2014	-0.06 (-0.24 to 0.36)
	3	2010-2015	0.51 (0.49 to 0.53)	2011-2015	0.74 (0.65 to 0.84)	2017-2021	-6.00 (-8.04 to -4.61)	2004-2015	0.41 (0.37 to 0.43)	2014-2019	-3.30 (-4.16 to -2.34)	2014-2019	-1.05 (-1.49 to -0.58)
	4	2015-2019	1.02 (0.99 to 1.05)	2015-2019	1.39 (1.32 to 1.47)			2015-2019	1.16 (1.10 to 1.24)	2019-2021	-10.13 (-12.20 to -7.47)	2019-2021	-4.18 (-5.14 to -2.98)
	5	2019-2021	0.26 (0.18 to 0.34)	2019-2021	0.42 (0.27 to 0.57)			2019-2021	0.25 (0.09 to 0.44)				
Iraq	0	1990-2000	-0.28 (-0.35 to -0.20)	1990-2000	-0.29 (-0.37 to -0.19)	1990-1997	1.49 (1.32 to 1.70)	1990-2000	-0.31 (-0.46 to -0.06)	1990-1997	1.49 (1.28 to 1.71)	1990-1997	1.24 (1.09 to 1.41)
	1	2000-2010	0.56 (0.47 to 0.72)	2000-2009	0.64 (-0.18 to 0.93)	1997-2002	-2.42 (-2.83 to -0.03)	2000-2008	0.65 (-0.51 to 1.05)	1997-2007	-2.12 (-2.27 to -1.97)	1997-2002	-1.97 (-2.35 to -1.64)
	2	2010-2019	0.26 (0.12 to 0.43)	2009-2019	0.38 (0.21 to 0.54)	2002-2006	-1.69 (-2.79 to -1.27)	2008-2019	0.39 (0.21 to 0.64)	2007-2012	-4.23 (-4.96 to -3.80)	2002-2006	-1.20 (-1.66 to -0.75)
	3	2019-2021	-0.73 (-1.08 to -0.03)	2019-2021	-0.73 (-1.14 to 0.10)	2006-2013	-3.80 (-4.05 to -3.59)	2019-2021	-0.77 (-1.23 to 0.18)	2012-2018	-2.73 (-3.06 to -2.04)	2006-2013	-3.05 (-3.30 to -2.87)
	4					2013-2018	-2.36 (-2.65 to -1.83)			2018-2021	-5.07 (-6.16 to -4.34)	2013-2018	-1.84 (-2.11 to -1.39)
	5					2018-2021	-4.00 (-4.67 to -3.53)					2018-2021	-3.81 (-4.36 to -3.34)
Ireland	0	1990-1998	2.10 (2.03 to 2.16)	1990-1999	2.32 (2.27 to 2.37)	1990-2003	-3.67 (-3.81 to -3.52)	1990-1997	2.22 (2.11 to 2.30)	1990-2001	-3.07 (-3.33 to -2.60)	1990-1996	-0.11 (-0.35 to 0.04)
	1	1998-2005	2.66 (2.59 to 2.76)	1999-2005	2.73 (2.65 to 2.84)	2003-2014	-5.63 (-5.85 to -5.45)	1997-2006	2.62 (2.56 to 2.73)	2001-2008	-4.24 (-5.11 to -3.69)	1996-2010	0.60 (0.43 to 0.66)
	2	2005-2010	2.12 (2.01 to 2.21)	2005-2010	2.20 (2.11 to 2.27)	2014-2019	-1.69 (-2.21 to -0.59)	2006-2010	2.16 (1.98 to 2.30)	2008-2014	-6.76 (-7.87 to -6.20)	2010-2013	1.63 (0.64 to 2.45)
	3	2010-2018	3.72 (3.68 to 3.77)	2010-2018	3.76 (3.72 to 3.79)	2019-2021	-7.40 (-8.89 to -5.32)	2010-2018	3.80 (3.75 to 3.86)	2014-2019	-1.57 (-2.31 to 0.04)	2013-2019	2.92 (2.74 to 3.40)
	4	2018-2021	2.11 (1.95 to 2.27)	2018-2021	2.08 (1.96 to 2.21)			2018-2021	2.02 (1.84 to 2.20)	2019-2021	-7.81 (-10.07 to -5.00)	2019-2021	0.43 (-0.35 to 1.39)
Israel	0	1990-1993	-0.53 (-0.77 to -0.40)	1990-1993	-0.35 (-0.58 to -0.20)	1990-2003	-3.66 (-3.84 to -3.44)	1990-1996	-0.20 (-0.26 to -0.14)	1990-1999	-3.99 (-4.35 to -3.79)	1990-1998	-2.02 (-2.21 to -1.88)
	1	1993-1996	0.11 (-0.04 to 0.36)	1993-1996	0.03 (-0.06 to 0.86)	2003-2012	-5.21 (-6.17 to -4.86)	1996-2005	1.11 (1.08 to 1.15)	1999-2002	-1.68 (-2.95 to -1.17)	1998-2002	-0.29 (-0.74 to 0.33)
	2	1996-2002	0.91 (0.80 to 0.97)	1996-2003	0.98 (0.92 to 1.03)	2012-2021	-3.83 (-4.21 to -3.18)	2005-2012	2.11 (2.05 to 2.19)	2002-2012	-5.66 (-5.95 to -5.46)	2002-2007	-1.43 (-2.14 to -1.09)
	3	2002-2005	1.18 (1.07 to 1.93)	2003-2006	1.49 (1.31 to 1.85)			2012-2017	1.51 (1.38 to 1.74)	2012-2019	-3.24 (-3.57 to -2.64)	2007-2021	0.08 (-0.03 to 0.21)
	4	2005-2011	2.10 (2.03 to 2.19)	2006-2010	2.27 (2.16 to 2.39)			2017-2021	1.09 (0.84 to 1.23)	2019-2021	-7.72 (-9.20 to -5.81)		
	5	2011-2016	1.66 (1.56 to 1.75)	2010-2015	1.71 (1.62 to 1.79)								
	6	2016-2021	1.14 (1.06 to 1.22)	2015-2021	1.24 (1.18 to 1.30)								
Italy	0	1990-2011	0.54 (-0.08 to 1.71)	1990-2013	0.67 (0.44 to 0.92)	1990-2005	-4.63 (-4.85 to -4.45)	1990-2014	1.04 (0.90 to 1.17)	1990-1998	-4.30 (-4.79 to -3.19)	1990-1999	-0.55 (-2.12 to -0.04)

Location	Segment	Incidence		Prevalence		Mortality		YLD		YLL		DALY	
		Period	APC (95%CI)	Period	APC (95%CI)	Period	APC (95%CI)	Period	APC (95%CI)	Period	APC (95%CI)	Period	APC (95%CI)
	1	2011-2015	2.63 (0.14 to 6.24)	2013-2019	5.01 (-0.28 to 5.96)	2005-2021	-3.09 (-3.33 to -2.81)	2014-2021	5.61 (4.70 to 6.75)	1998-2002	-6.72 (-8.11 to -5.26)	1999-2013	0.32 (-0.54 to 1.37)
	2	2015-2021	6.68 (5.22 to 9.71)	2019-2021	9.72 (5.49 to 11.92)					2002-2021	-3.43 (-3.63 to -3.21)	2013-2019	3.64 (-0.73 to 4.60)
	3											2019-2021	8.64 (4.24 to 10.93)
Jamaica	0	1990-1999	-0.95 (-1.47 to -0.68)	1990-2000	-1.12 (-1.49 to -0.89)	1990-1992	-3.61 (-6.21 to 0.50)	1990-2000	-1.06 (-1.35 to -0.85)	1990-1992	-4.20 (-6.74 to -0.80)	1990-1993	-2.32 (-5.19 to -0.50)
	1	1999-2010	0.10 (-0.08 to 0.60)	2000-2010	0.09 (-0.13 to 0.71)	1992-1999	2.58 (1.44 to 5.96)	2000-2010	0.21 (-0.01 to 0.64)	1992-1999	2.72 (1.86 to 5.47)	1993-1998	2.83 (1.58 to 5.16)
	2	2010-2021	-1.15 (-1.36 to -0.97)	2010-2021	-1.23 (-1.45 to -1.06)	1999-2007	-4.28 (-5.21 to -3.47)	2010-2021	-1.31 (-1.52 to -1.14)	1999-2007	-4.64 (-5.45 to -4.05)	1998-2007	-3.00 (-3.75 to -2.57)
	3					2007-2021	-0.76 (-1.30 to -0.21)			2007-2010	0.97 (-2.12 to 2.12)	2007-2021	-0.82 (-1.14 to -0.46)
	4									2010-2013	-3.93 (-5.03 to -1.70)		
	5									2013-2021	0.73 (-0.42 to 3.38)		
Japan	0	1990-2004	0.17 (0.06 to 0.25)	1990-2004	0.18 (0.05 to 0.28)	1990-1993	-4.17 (-5.32 to -2.94)	1990-2006	0.66 (0.50 to 0.75)	1990-1993	-3.88 (-6.45 to -1.45)	1990-1993	-1.18 (-1.72 to -0.48)
	1	2004-2013	1.32 (1.16 to 1.65)	2004-2012	1.07 (0.85 to 1.89)	1993-1996	-15.98 (-16.94 to -14.95)	2006-2010	1.81 (1.17 to 2.42)	1993-1997	-13.99 (-15.30 to -12.73)	1993-1996	-5.48 (-5.95 to -4.78)
	2	2013-2019	0.41 (-0.43 to 0.67)	2012-2019	0.50 (-0.40 to 0.73)	1996-2000	-6.41 (-7.75 to -5.38)	2010-2021	0.70 (0.38 to 0.83)	1997-2016	-3.46 (-3.73 to -3.27)	1996-2004	-0.59 (-0.97 to -0.33)
	3	2019-2021	2.65 (1.35 to 3.41)	2019-2021	2.66 (1.21 to 3.48)	2000-2016	-3.31 (-3.52 to -3.08)			2016-2021	-0.18 (-1.83 to 4.04)	2004-2021	0.38 (0.28 to 0.50)
	4					2016-2021	0.01 (-1.15 to 2.14)						
Jordan	0	1990-2000	-1.29 (-1.48 to -1.13)	1990-2000	-1.42 (-1.58 to -1.29)	1990-1992	-2.21 (-2.99 to -0.74)	1990-1995	-1.36 (-1.84 to -0.52)	1990-1999	-1.22 (-1.43 to -0.94)	1990-1998	-1.41 (-1.53 to -1.22)
	1	2000-2013	0.20 (0.08 to 0.35)	2000-2015	0.05 (-0.02 to 0.12)	1992-2000	-0.59 (-2.99 to 0.07)	1995-2000	-3.08 (-4.01 to -2.62)	1999-2004	-2.81 (-3.52 to -2.38)	1998-2001	-2.49 (-2.81 to -1.92)
	2	2013-2021	-1.56 (-1.82 to -1.35)	2015-2018	-4.13 (-4.45 to -3.70)	2000-2005	-3.04 (-8.99 to -2.68)	2000-2010	1.82 (1.63 to 2.03)	2004-2009	-7.98 (-8.57 to -7.62)	2001-2004	-1.19 (-1.87 to -0.83)
	3			2018-2021	-1.40 (-1.86 to -0.51)	2005-2009	-9.01 (-9.30 to -5.05)	2010-2021	-2.23 (-2.40 to -2.07)	2009-2018	-4.76 (-4.99 to -4.54)	2004-2008	-4.03 (-4.48 to -3.67)
	4					2009-2018	-4.95 (-5.14 to -4.75)			2018-2021	-1.38 (-2.34 to 0.35)	2008-2015	-3.02 (-3.14 to -2.49)
	5					2018-2021	-0.93 (-1.69 to -0.14)					2015-2018	-4.26 (-4.63 to -3.72)
	6											2018-2021	-1.25 (-1.77 to -0.25)
Kazakhstan	0	1990-2001	-0.29 (-0.39 to -0.16)	1990-2001	-0.29 (-0.40 to -0.18)	1990-1995	12.55 (11.44 to 13.92)	1990-2001	-0.24 (-0.34 to -0.15)	1990-1992	18.71 (13.02 to 23.14)	1990-1992	14.74 (10.54 to 18.04)
	1	2001-2010	1.24 (-0.20 to 1.36)	2001-2009	1.35 (0.90 to 1.50)	1995-2000	-5.44 (-7.01 to -4.65)	2001-2009	1.42 (1.05 to 1.58)	1992-1995	10.90 (-5.85 to 12.52)	1992-1995	9.06 (-4.94 to 10.24)
	2	2010-2014	2.46 (1.24 to 2.95)	2009-2014	2.48 (1.40 to 3.06)	2000-2003	-1.82 (-3.66 to -0.97)	2009-2014	2.52 (1.56 to 3.08)	1995-2000	-5.00 (-6.62 to -0.39)	1995-2000	-4.25 (-5.56 to -0.75)
	3	2014-2019	1.09 (0.68 to 2.33)	2014-2019	1.43 (0.98 to 2.26)	2003-2021	-4.30 (-4.70 to -4.12)	2014-2019	1.58 (1.17 to 2.21)	2000-2003	-0.78 (-5.36 to 0.07)	2000-2003	-1.04 (-4.23 to -0.27)
	4	2019-2021	-0.71 (-1.36 to 0.50)	2019-2021	-0.65 (-1.30 to 0.53)			2019-2021	-0.49 (-1.13 to 0.47)	2003-2021	-4.55 (-5.65 to -3.87)	2003-2021	-2.94 (-4.55 to -2.09)
Kenya	0	1990-1994	-0.35 (-0.60 to -0.23)	1990-1994	-0.31 (-0.47 to -0.23)	1990-1996	-0.98 (-1.36 to -0.75)	1990-1994	-0.30 (-0.45 to -0.21)	1990-1996	-1.07 (-1.55 to -0.81)	1990-1994	-0.89 (-1.38 to -0.66)
	1	1994-2011	0.03 (-0.05 to 0.17)	1994-2002	0.10 (0.06 to 0.16)	1996-2001	0.13 (-0.26 to 0.68)	1994-2004	0.12 (0.09 to 0.18)	1996-2001	0.06 (-0.38 to 0.74)	1994-1999	-0.29 (-0.54 to 0.19)
	2	2011-2015	0.50 (-0.10 to 0.55)	2002-2010	-0.06 (-0.16 to 0.07)	2001-2007	2.65 (2.41 to 2.93)	2004-2011	0.02 (-0.08 to 0.05)	2001-2007	2.66 (2.39 to 3.13)	1999-2002	0.79 (0.17 to 1.66)
	3	2015-2019	0.69 (0.48 to 0.81)	2010-2013	0.27 (-0.05 to 0.44)	2007-2011	0.95 (0.62 to 1.60)	2011-2019	0.55 (0.51 to 0.58)	2007-2011	1.21 (0.71 to 1.92)	2002-2007	1.79 (0.95 to 2.12)
	4	2019-2021	1.36 (1.03 to 1.56)	2013-2019	0.53 (0.47 to 0.63)	2011-2014	-0.62 (-1.52 to 0.10)	2019-2021	1.24 (1.02 to 1.37)	2011-2014	-0.83 (-1.92 to 0.21)	2007-2011	0.83 (-0.27 to 1.09)
	5			2019-2021	1.31 (1.09 to 1.44)	2014-2021	-2.35 (-2.57 to -2.20)			2014-2021	-2.69 (-2.99 to -2.50)	2011-2014	-0.45 (-1.43 to 0.01)
	6											2014-2021	-1.50 (-1.66 to -1.39)
Kiribati	0	1990-2010	0.04 (0.02 to 0.06)	1990-2011	0.07 (0.05 to 0.10)	1990-1997	0.12 (-0.06 to 0.24)	1990-2002	0.15 (-0.07 to 0.35)	1990-1997	0.11 (-0.10 to 0.26)	1990-1997	0.10 (-0.32 to 0.25)
	1	2010-2021	-0.07 (-0.17 to -0.03)	2011-2021	-0.29 (-0.40 to -0.21)	1997-2000	1.40 (0.95 to 1.67)	2002-2010	0.05 (-0.29 to 0.23)	1997-2001	1.26 (0.90 to 1.74)	1997-2000	1.23 (0.69 to 1.53)
	2					2000-2005	0.17 (-0.34 to 0.37)	2010-2019	-0.15 (-0.26 to 0.10)	2001-2005	-0.34 (-0.86 to 0.03)	2000-2005	-0.02 (-0.60 to 0.18)
	3					2005-2010	1.32 (0.99 to 1.85)	2019-2021	-0.77 (-1.04 to -0.31)	2005-2009	1.74 (1.30 to 2.32)	2005-2010	1.04 (0.72 to 1.66)
	4					2010-2021	-0.50 (-0.62 to -0.40)			2009-2021	-0.55 (-0.64 to -0.46)	2010-2021	-0.54 (-0.66 to -0.44)
Kuwait	0	1990-2018	0.43 (0.27 to 0.64)	1990-2018	0.38 (0.22 to 0.56)	1990-1996	7.58 (4.46 to 10.98)	1990-2018	0.39 (0.24 to 0.56)	1990-1996	8.94 (5.90 to 12.19)	1990-1996	5.82 (4.15 to 7.68)
	1	2018-2021	-4.59 (-11.08 to -0.74)	2018-2021	-5.59 (-12.38 to -1.53)	1996-2002	-14.45 (-17.04 to 4.65)	2018-2021	-5.21 (-11.57 to -1.87)	1996-2002	-14.21 (-16.73 to 6.68)	1996-2002	-8.95 (-11.59 to 3.94)
	2					2002-2009	0.15 (-14.72 to 4.11)			2002-2009	0.00 (-14.85 to 3.64)	2002-2008	0.41 (-9.06 to 6.05)
	3					2009-2012	-10.67 (-13.01 to 1.33)			2009-2012	-10.01 (-12.31 to 1.63)	2008-2021	-2.59 (-6.64 to -1.70)
	4					2012-2021	-4.14 (-6.10 to 0.86)			2012-2021	-4.01 (-5.85 to 1.09)		
Kyrgyzstan	0	1990-2000	-0.13 (-0.24 to -0.05)	1990-2001	-0.06 (-0.14 to 0.00)	1990-1994	10.02 (7.35 to 14.01)	1990-2001	-0.01 (-0.09 to 0.05)	1990-1994	10.44 (7.32 to 14.50)	1990-1995	5.75 (3.70 to 8.80)
	1	2000-2006	0.67 (0.39 to 0.94)	2001-2007	0.96 (0.67 to 1.20)	1994-2002	-0.71 (-1.88 to 1.74)	2001-2007	1.01 (0.70 to 1.25)	1994-2015	-1.60 (-1.86 to -1.30)	1995-2021	-1.20 (-1.43 to -1.02)
	2	2006-2014	1.62 (1.47 to 1.79)	2007-2014	1.85 (1.66 to 2.06)	2002-2015	-2.41 (-4.76 to -1.84)	2007-2014	1.89 (1.68 to 2.10)	2015-2021	-5.60 (-8.78 to -3.91)		
	3	2014-2021	2.55 (2.39 to 2.76)	2014-2021	2.71 (2.56 to 2.91)	2015-2021	-5.33 (-9.77 to -2.98)	2014-2021	2.74 (2.61 to 2.95)				
Lao People's Democratic Republic	0	1990-2000	-0.44 (-0.58 to -0.27)	1990-2001	-0.45 (-0.67 to -0.29)	1990-1994	0.61 (0.32 to 1.13)	1990-2000	-0.43 (-0.64 to -0.28)	1990-1998	0.31 (0.16 to 0.51)	1990-1995	0.46 (0.14 to 1.03)
	1	2000-2012	0.24 (-0.53 to 0.43)	2001-2012	0.60 (0.43 to 0.88)	1994-1999	-0.16 (-0.56 to 0.06)	2000-2013	0.47 (0.36 to 0.64)	1998-2002	-1.07 (-1.54 to -0.53)	1995-2001	-0.41 (-0.85 to -0.16)
	2	2012-2019	-0.95 (-1.10 to 0.30)	2012-2021	-1.13 (-1.41 to -0.92)	1999-2002	-1.23 (-2.34 to -0.80)	2013-2021	-1.18 (-1.47 to -0.95)	2002-2009	-2.26 (-2.42 to -2.08)	2001-2009	-1.69 (-1.82 to -1.53)
	3	2019-2021	-2.12 (-2.71 to -1.05)			2002-2009	-2.39 (-2.81 to -2.27)			2009-2014	-2.97 (-3.35 to -2.78)	2009-2014	-2.32 (-2.66 to -2.13)
	4					2009-2014	-2.90 (-3.17 to -2.33)			2014-2021	-1.84 (-1.95 to -1.69)	2014-2021	-1.69 (-1.80 to -1.51)
	5					2014-2021	-1.86 (-1.96 to -1.72)						
Latvia	0	1990-1995	0.54 (0.38 to 0.63)	1990-1995	0.59 (0.46 to 0.68)	1990-1994	5.27 (1.27 to 10.86)	1990-1995	0.58 (0.36 to 0.72)	1990-1993	8.72 (3.09 to 17.20)	1990-1994	5.27 (3.38 to 7.06)
	1	1995-1999	1.07 (0.92 to 1.24)	1995-1999	1.17 (1.02 to 1.36)	1994-2011	-6.31 (-6.79 to -5.91)	1995-1999	1.34 (1.12 to 1.60)	1993-2011	-6.15 (-6.64 to -5.73)	1994-2000	-6.36 (-8.41 to -5.34)
	2	1999-2006	0.67 (0.57 to 0.72)	1999-2006	0.63 (0.54 to 0.69)	2011-2018	9.61 (7.70 to 13.38)	1999-2006	0.70 (0.54 to 0.78)	2011-2018	11.71 (9.59 to 15.82)	2000-2011	-3.41 (-4.12 to -1.97)
	3	2006-2014	2.32 (2.27 to 2.45)	2006-2014	2.40 (2.35 to 2.54)	2018-2021	-5.64 (-17.34 to 1.77)	2006-2013	2.53 (1.02 to 2.72)	2018-2021	-6.89 (-20.04 to 1.24)	2011-2018	8.35 (6.98 to 11.26)
	4	2014-2021	2.09 (1.84 to 2.18)	2014-2021	2.21 (1.94 to 2.30)			2013-2021	2.33 (2.07 to 2.42)			2018-2021	-3.70 (-9.35 to 0.14)
Lebanon	0	1990-1999	-0.65 (-0.81 to -0.51)	1990-1999	-0.56 (-0.73 to -0.42)	1990-1993	-2.06 (-2.86 to -0.84)	1990-2000	-0.54 (-0.67 to -0.42)	1990-1993	-2.10 (-2.92 to -0.78)	1990-1993	-1.81 (-2.71 to -0.62)
	1	1999-2010	0.49 (0.39 to 0.63)	1999-2010	0.55 (0.45 to 0.69)	1993-2002	-4.20 (-4.73 to -4.02)	2000-2005	2.14 (1.78 to 2.77)	1993-2002	-4.35 (-4.94 to -4.15)	1993-2000	-3.20 (-3.91 to -2.89)
	2	2010-2021	-0.43 (-0.55 to -0.33)	2010-2021	-0.31 (-0.43 to -0.21)	2002-2009	-3.49 (-3.89 to -2.98)	2005-2011	0.34 (0.05 to 0.65)	2002-2008	-3.68 (-4.07 to -2.72)	2000-2018	-1.76 (-1.87 to -1.70)
	3					2009-2018	-2.63 (-2.89 to -2.27)	2011-2014	-2.26 (-2.62 to -1.47)	2008-2018	-2.50 (-2.76 to -2.21)	2018-2021	-0.55 (-1.26 to 0.36)

Location	Segment	Incidence		Prevalence		Mortality		YLD		YLL		DALY	
		Period	APC (95%CI)	Period	APC (95%CI)	Period	APC (95%CI)	Period	APC (95%CI)	Period	APC (95%CI)	Period	APC (95%CI)
	4					2018-2021	-0.75 (-1.56 to 0.47)	2014-2021	-0.49 (-0.67 to -0.14)	2018-2021	-0.63 (-1.53 to 0.69)		
Lesotho	0	1990-1994	1.19 (0.87 to 1.75)	1990-1994	0.93 (0.76 to 1.27)	1990-1998	-2.14 (-2.52 to -1.77)	1990-1993	1.06 (0.87 to 1.35)	1990-1998	-2.29 (-2.63 to -1.97)	1990-1998	-0.73 (-0.90 to -0.57)
	1	1994-1999	0.69 (-0.39 to 0.82)	1994-2000	0.47 (0.26 to 0.56)	1998-2005	9.06 (8.54 to 9.61)	1993-2000	0.49 (0.42 to 0.55)	1998-2001	9.69 (8.34 to 10.52)	1998-2005	4.19 (3.99 to 4.42)
	2	1999-2010	-0.26 (-0.42 to 0.78)	2000-2006	-0.54 (-0.78 to -0.40)	2005-2014	1.46 (0.96 to 2.16)	2000-2004	-0.74 (-0.91 to -0.61)	2001-2006	6.71 (4.64 to 7.29)	2005-2014	1.01 (0.80 to 1.27)
	3	2010-2019	0.66 (0.22 to 0.78)	2006-2011	-0.11 (-0.34 to 0.35)	2014-2021	-1.69 (-2.96 to -0.93)	2004-2010	-0.32 (-0.42 to -0.15)	2006-2014	1.33 (0.80 to 1.79)	2014-2021	-0.70 (-1.13 to -0.39)
	4	2019-2021	1.67 (0.87 to 2.04)	2011-2019	0.58 (0.39 to 0.69)			2010-2019	0.49 (0.43 to 0.54)	2014-2021	-1.47 (-2.12 to -0.97)		
	5			2019-2021	1.42 (0.88 to 1.68)			2019-2021	1.28 (0.91 to 1.47)				
Liberia	0	1990-2004	-0.05 (-0.16 to 0.30)	1990-1994	-0.23 (-0.61 to -0.02)	1990-1996	-1.26 (-1.64 to -0.89)	1990-2004	0.03 (-0.01 to 0.07)	1990-1996	-1.24 (-1.81 to -0.48)	1990-1996	-0.94 (-1.38 to -0.37)
	1	2004-2010	0.49 (-0.27 to 0.72)	1994-2005	0.11 (0.03 to 0.36)	1996-2002	-4.04 (-4.47 to -3.64)	2004-2010	0.64 (0.49 to 0.91)	1996-2002	-4.14 (-5.55 to -3.50)	1996-2002	-3.06 (-4.07 to -2.55)
	2	2010-2015	-0.29 (-0.55 to 0.66)	2005-2010	0.59 (-0.15 to 0.87)	2002-2007	-0.17 (-1.39 to 0.41)	2010-2016	-0.25 (-0.52 to -0.10)	2002-2007	-0.56 (-2.40 to 0.77)	2002-2007	-0.22 (-1.68 to 0.68)
	3	2015-2019	0.17 (-0.40 to 0.33)	2010-2015	-0.27 (-0.50 to 0.74)	2007-2013	2.15 (1.53 to 2.81)	2016-2021	0.44 (0.28 to 0.75)	2007-2013	2.25 (1.45 to 4.02)	2007-2013	1.61 (1.05 to 2.81)
	4	2019-2021	0.90 (0.30 to 1.23)	2015-2019	0.13 (-0.34 to 0.30)	2013-2018	-2.55 (-3.37 to -1.75)			2013-2021	-2.13 (-2.72 to -1.63)	2013-2021	-1.47 (-1.89 to -1.11)
	5			2019-2021	0.82 (0.26 to 1.15)	2018-2021	-0.57 (-1.69 to 0.93)						
Libya	0	1990-2001	0.24 (0.20 to 0.29)	1990-2001	0.24 (0.19 to 0.29)	1990-1993	-3.09 (-5.80 to -0.80)	1990-2001	0.23 (0.16 to 0.30)	1990-1992	-4.41 (-6.15 to -1.13)	1990-1993	-2.04 (-4.08 to -0.18)
	1	2001-2010	1.54 (1.48 to 1.60)	2001-2010	1.58 (1.51 to 1.64)	1993-2006	-0.15 (-0.37 to 0.54)	2001-2010	1.62 (1.54 to 1.71)	1992-2006	-0.45 (-0.66 to 0.17)	1993-2006	0.07 (-0.15 to 3.13)
	2	2010-2015	-1.12 (-1.23 to -1.02)	2010-2015	-1.75 (-1.87 to -1.64)	2006-2016	4.32 (3.99 to 4.73)	2010-2015	-1.74 (-1.87 to -1.61)	2006-2011	4.81 (3.78 to 6.20)	2006-2010	3.88 (1.40 to 5.08)
	3	2015-2021	1.59 (1.47 to 1.70)	2015-2021	1.60 (1.48 to 1.72)	2016-2021	-0.17 (-1.66 to 0.90)	2015-2021	1.57 (1.43 to 1.69)	2011-2017	2.74 (1.80 to 3.61)	2010-2021	1.13 (0.83 to 1.39)
	4									2017-2021	-0.73 (-2.82 to 0.43)		
Lithuania	0	1990-1995	0.57 (-0.00 to 0.79)	1990-1995	0.65 (0.10 to 0.89)	1990-1995	4.34 (2.14 to 6.83)	1990-2006	1.19 (1.09 to 1.28)	1990-1995	4.52 (2.00 to 7.48)	1990-1995	3.61 (1.82 to 5.29)
	1	1995-2000	1.42 (1.15 to 1.92)	1995-2000	1.59 (1.36 to 2.13)	1995-2003	-7.12 (-8.85 to -6.34)	2006-2015	2.57 (2.37 to 3.01)	1995-2003	-7.36 (-9.88 to -6.44)	1995-2003	-5.20 (-7.22 to -4.29)
	2	2000-2005	0.82 (0.28 to 1.98)	2000-2005	0.77 (0.21 to 1.03)	2003-2007	-0.35 (-2.86 to 2.81)	2015-2021	1.33 (0.73 to 1.70)	2003-2007	1.12 (-2.05 to 5.03)	2003-2006	2.66 (-1.95 to 4.76)
	3	2005-2015	2.35 (2.22 to 2.56)	2005-2015	2.39 (2.28 to 2.54)	2007-2011	-10.16 (-14.09 to -8.15)			2007-2011	-10.43 (-15.38 to -7.97)	2006-2011	-4.17 (-9.10 to -1.50)
	4	2015-2021	1.15 (0.68 to 1.44)	2015-2021	1.19 (0.74 to 1.48)	2011-2017	9.91 (8.44 to 12.06)			2011-2017	12.60 (10.70 to 15.82)	2011-2018	7.33 (5.87 to 11.47)
	5					2017-2021	-1.58 (-5.28 to 1.33)			2017-2021	-2.10 (-6.92 to 1.41)	2018-2021	-3.46 (-9.14 to 0.27)
Luxembourg	0	1990-1993	0.99 (0.83 to 1.08)	1990-1993	0.93 (0.74 to 1.03)	1990-1992	-0.81 (-2.41 to 0.18)	1990-1995	1.14 (1.01 to 1.24)	1990-1992	-1.50 (-4.71 to 1.46)	1990-1992	-0.11 (-0.80 to 0.39)
	1	1993-1996	1.31 (1.24 to 1.58)	1993-1996	1.42 (1.33 to 1.71)	1992-2000	-5.47 (-5.71 to -5.29)	1995-2004	1.99 (1.94 to 2.05)	1992-2021	-4.75 (-7.60 to -4.61)	1992-1999	-1.84 (-2.04 to -1.73)
	2	1996-2005	1.99 (1.97 to 2.01)	1996-2005	1.95 (1.93 to 1.98)	2000-2003	-1.22 (-2.28 to -0.73)	2004-2016	1.42 (1.39 to 1.46)			1999-2004	0.07 (-0.20 to 0.55)
	3	2005-2011	1.29 (1.26 to 1.32)	2005-2010	1.21 (1.14 to 1.25)	2003-2010	-5.53 (-5.92 to -5.28)	2016-2021	1.02 (0.87 to 1.13)			2004-2009	-1.00 (-1.59 to -0.71)
	4	2011-2014	1.80 (1.71 to 1.86)	2010-2014	1.51 (1.43 to 1.62)	2010-2014	-2.64 (-3.31 to -1.74)					2009-2021	0.03 (-0.07 to 0.17)
	5	2014-2019	0.97 (0.88 to 1.01)	2014-2019	0.98 (0.88 to 1.03)	2014-2021	-4.75 (-5.24 to -4.45)						
	6	2019-2021	1.33 (1.16 to 1.45)	2019-2021	1.43 (1.23 to 1.55)								
Madagascar	0	1990-1995	-0.43 (-0.56 to -0.32)	1990-1994	-0.40 (-0.68 to -0.24)	1990-2006	-0.86 (-0.97 to -0.76)	1990-1995	-0.25 (-0.63 to -0.09)	1990-2004	-0.92 (-1.06 to -0.82)	1990-2002	-0.73 (-0.83 to -0.65)
	1	1995-2011	0.07 (0.06 to 0.09)	1994-2010	0.13 (0.10 to 0.18)	2006-2017	-0.17 (-0.34 to 0.42)	1995-2010	0.16 (0.13 to 0.21)	2004-2017	-0.18 (-0.29 to 0.11)	2002-2017	-0.20 (-0.26 to -0.11)
	2	2011-2016	-0.47 (-0.66 to -0.37)	2010-2017	-0.61 (-0.82 to 0.12)	2017-2021	-1.01 (-2.16 to -0.42)	2010-2021	-0.40 (-0.46 to -0.35)	2017-2021	-1.21 (-2.32 to -0.62)	2017-2021	-1.01 (-1.59 to -0.65)
	3	2016-2021	-0.19 (-0.28 to 0.02)	2017-2021	-0.33 (-0.52 to 0.02)								
Malawi	0	1990-1995	-0.39 (-1.06 to -0.13)	1990-2012	0.15 (0.10 to 0.19)	1990-1992	-0.74 (-1.37 to 0.07)	1990-1997	-0.05 (-0.26 to 0.09)	1990-1992	-1.29 (-2.14 to -0.25)	1990-1992	-0.99 (-1.73 to 0.17)
	1	1995-2012	0.12 (0.07 to 0.19)	2012-2021	-1.20 (-1.42 to -1.03)	1992-1996	1.07 (0.70 to 1.60)	1997-2005	0.46 (0.32 to 0.88)	1992-1995	1.44 (0.71 to 1.95)	1992-1995	1.03 (-0.15 to 1.47)
	2	2012-2021	-0.82 (-0.97 to -0.70)			1996-2003	-0.77 (-1.03 to -0.53)	2005-2012	0.03 (-0.29 to 0.32)	1995-2003	-0.73 (-0.97 to -0.54)	1995-2003	-0.46 (-0.72 to -0.30)
	3					2003-2008	-2.02 (-2.53 to -0.86)	2012-2019	-0.88 (-1.05 to -0.26)	2003-2008	-2.31 (-2.98 to -1.96)	2003-2008	-1.63 (-2.23 to -1.33)
	4					2008-2011	-0.56 (-2.04 to -0.30)	2019-2021	-1.97 (-2.40 to -1.15)	2008-2011	0.13 (-0.58 to 0.53)	2008-2011	0.10 (-0.53 to 0.44)
	5					2011-2019	-1.76 (-1.89 to -0.70)			2011-2016	-1.69 (-2.16 to -1.29)	2011-2016	-1.39 (-1.80 to -1.11)
	6					2019-2021	-2.90 (-3.58 to -1.93)			2016-2021	-3.00 (-3.65 to -2.66)	2016-2021	-2.49 (-2.96 to -2.22)
Malaysia	0	1990-1998	-0.71 (-0.91 to -0.56)	1990-1999	-0.57 (-0.81 to -0.41)	1990-1994	-4.29 (-7.13 to -2.65)	1990-1995	0.29 (-0.13 to 0.91)	1990-1994	-4.14 (-7.01 to -2.49)	1990-2003	-2.11 (-2.39 to -1.89)
	1	1998-2010	0.23 (0.13 to 0.39)	1999-2011	0.42 (0.30 to 0.60)	1994-2008	-2.16 (-2.52 to -1.20)	1995-2000	-1.68 (-2.58 to -1.17)	1994-2008	-2.24 (-2.56 to -0.22)	2003-2021	-0.45 (-0.61 to -0.28)
	2	2010-2019	-0.54 (-0.71 to 0.00)	2011-2021	-0.64 (-0.83 to -0.50)	2008-2021	-0.61 (-0.99 to 0.05)	2000-2005	-0.10 (-0.91 to 1.05)	2008-2021	-0.37 (-0.90 to 0.38)		
	3	2019-2021	-2.09 (-2.71 to -0.81)					2005-2010	1.55 (-0.55 to 2.52)				
	4							2010-2021	-0.69 (-0.88 to -0.50)				
Maldives	0	1990-1999	-0.63 (-1.06 to -0.39)	1990-2001	-0.47 (-0.64 to -0.32)	1990-1997	-4.66 (-5.35 to -3.69)	1990-2000	-0.46 (-0.68 to -0.28)	1990-1998	-5.30 (-5.76 to -4.63)	1990-1996	-4.18 (-4.52 to -3.28)
	1	1999-2011	0.50 (0.32 to 0.77)	2001-2010	0.97 (0.77 to 1.26)	1997-2007	-7.47 (-7.91 to -7.25)	2000-2010	0.95 (0.74 to 1.24)	1998-2007	-7.20 (-7.68 to -6.98)	1996-2005	-4.92 (-5.20 to -4.79)
	2	2011-2021	-1.36 (-1.59 to -1.16)	2010-2021	-1.45 (-1.60 to -1.31)	2007-2010	-5.08 (-6.66 to -3.48)	2010-2021	-1.33 (-1.50 to -1.16)	2007-2010	-5.38 (-6.70 to -3.71)	2005-2009	-3.49 (-3.87 to -3.06)
	3					2010-2015	-2.24 (-2.63 to -1.34)			2010-2015	-2.00 (-2.41 to -1.15)	2009-2015	-1.74 (-1.90 to -1.45)
	4					2015-2021	-3.53 (-4.15 to -3.23)			2015-2021	-3.88 (-4.36 to -3.55)	2015-2021	-2.69 (-2.91 to -2.52)
Mali	0	1990-1993	-0.55 (-0.68 to -0.49)	1990-1994	-0.56 (-0.62 to -0.51)	1990-1995	-1.54 (-1.96 to -1.08)	1990-1993	-0.70 (-1.11 to -0.43)	1990-1995	-1.26 (-1.71 to -0.68)	1990-1995	-1.09 (-1.43 to -0.67)
	1	1993-1999	-0.25 (-0.29 to -0.22)	1994-2005	-0.10 (-0.12 to -0.09)	1995-2000	-3.71 (-4.42 to -3.17)	1993-2005	-0.10 (-0.16 to -0.05)	1995-2000	-3.44 (-4.48 to -3.01)	1995-2000	-2.71 (-3.45 to -2.39)
	2	1999-2005	-0.09 (-0.12 to -0.05)	2005-2010	0.21 (0.17 to 0.29)	2000-2003	0.25 (-1.65 to 0.84)	2005-2011	0.24 (0.12 to 0.50)	2000-2003	0.76 (-0.26 to 1.40)	2000-2003	0.54 (-0.19 to 1.01)
	3	2005-2010	0.17 (0.14 to 0.22)	2010-2014	-0.29 (-0.35 to -0.19)	2003-2011	-0.72 (-1.53 to -0.51)	2011-2021	-0.46 (-0.51 to -0.40)	2003-2011	-1.26 (-2.10 to -1.04)	2003-2011	-0.92 (-1.52 to -0.76)
	4	2010-2013	-0.42 (-0.47 to -0.37)	2014-2019	-0.51 (-0.60 to -0.47)	2011-2018	-0.00 (-0.26 to 0.74)			2011-2018	0.11 (-0.25 to 0.97)	2011-2018	-0.03 (-0.27 to 0.51)
	5	2013-2019	-0.55 (-0.59 to -0.53)	2019-2021	-0.02 (-0.18 to 0.09)	2018-2021	-2.39 (-3.41 to -1.60)			2018-2021	-2.91 (-4.19 to -1.93)	2018-2021	-2.27 (-3.11 to -1.58)
	6	2019-2021	-0.17 (-0.27 to -0.09)										
Malta	0	1990-1994	1.41 (1.32 to 1.49)	1990-1994	1.44 (1.33 to 1.54)	1990-1999	-4.81 (-5.35 to -4.46)	1990-1994	1.47 (1.36 to 1.58)	1990-1996	-4.84 (-8.46 to -3.37)	1990-1998	-1.47 (-1.90 to -1.24)
	1	1994-2003	0.61 (0.57 to 0.64)	1994-2003	0.68 (0.63 to 0.70)	1999-2003	-1.81 (-3.05 to -0.47)	1994-2001	0.63 (0.51 to 0.68)	1996-2021	-3.24 (-4.86 to -2.27)	1998-2007	-0.30 (-0.62 to 0.09)
	2	2003-2006	1.17 (1.06 to 1.24)	2003-2006	1.15 (1.04 to 1.23)	2003-2017	-4.15 (-4.67 to -3.95)	2001-2005	0.87 (0.75 to 1.07)			2007-2021	1.27 (1.12 to 1.44)

Location	Segment	Incidence		Prevalence		Mortality		YLD		YLL		DALY	
		Period	APC (95%CI)	Period	APC (95%CI)	Period	APC (95%CI)	Period	APC (95%CI)	Period	APC (95%CI)	Period	APC (95%CI)
	3	2006-2019	2.60 (2.59 to 2.62)	2006-2019	2.62 (2.60 to 2.64)	2017-2021	-1.22 (-2.93 to 2.14)	2005-2008	2.16 (2.05 to 2.46)				
	4	2019-2021	1.71 (1.49 to 2.05)	2019-2021	1.85 (1.63 to 2.15)			2008-2018	2.65 (2.62 to 2.70)				
	5							2018-2021	2.06 (1.86 to 2.24)				
Marshall Islands	0	1990-1995	0.17 (0.05 to 0.45)	1990-2008	0.02 (-0.04 to 0.06)	1990-1993	2.57 (2.33 to 2.79)	1990-2011	0.01 (-0.03 to 0.03)	1990-1994	2.45 (2.30 to 2.59)	1990-1994	1.91 (1.78 to 2.02)
	1	1995-2000	-0.19 (-0.39 to -0.09)	2008-2016	0.27 (0.15 to 0.70)	1993-1998	4.14 (4.03 to 4.35)	2011-2015	0.61 (0.27 to 0.96)	1994-1997	4.28 (4.01 to 4.46)	1994-1997	3.30 (3.07 to 3.45)
	2	2000-2010	0.06 (0.01 to 0.11)	2016-2021	-0.56 (-0.93 to -0.33)	1998-2001	3.27 (2.82 to 3.55)	2015-2021	-0.44 (-0.70 to -0.27)	1997-2001	3.22 (2.96 to 3.35)	1997-2001	2.53 (2.25 to 2.66)
	3	2010-2015	0.45 (0.07 to 0.66)			2001-2005	1.31 (1.14 to 1.44)			2001-2005	1.32 (1.17 to 1.46)	2001-2005	1.06 (0.89 to 1.18)
	4	2015-2019	0.05 (-0.10 to 0.39)			2005-2015	-0.85 (-0.89 to -0.74)			2005-2013	-0.83 (-0.90 to -0.71)	2005-2014	-0.64 (-0.70 to -0.55)
	5	2019-2021	-0.73 (-0.96 to -0.38)			2015-2021	-1.09 (-1.33 to -0.98)			2013-2021	-1.10 (-1.24 to -1.02)	2014-2021	-0.93 (-1.08 to -0.85)
Mauritania	0	1990-1994	-0.42 (-0.50 to -0.37)	1990-1994	-0.36 (-0.49 to -0.30)	1990-1999	-3.51 (-4.16 to -2.75)	1990-1994	-0.38 (-0.73 to -0.22)	1990-1999	-2.89 (-3.52 to -2.55)	1990-1997	-2.25 (-2.95 to -1.87)
	1	1994-2000	-0.20 (-0.23 to -0.16)	1994-1999	-0.12 (-0.21 to -0.04)	1999-2006	-0.84 (-4.11 to 0.26)	1994-2005	-0.00 (-0.05 to 0.07)	1999-2006	-0.92 (-1.55 to 1.01)	1997-2013	-0.90 (-1.21 to -0.62)
	2	2000-2005	0.11 (0.07 to 0.15)	1999-2005	0.05 (0.01 to 0.14)	2006-2013	-1.63 (-2.78 to -0.32)	2005-2010	0.59 (0.45 to 0.86)	2006-2021	-2.20 (-2.57 to -2.00)	2013-2016	-2.47 (-3.08 to -0.96)
	3	2005-2010	0.42 (0.39 to 0.45)	2005-2010	0.48 (0.43 to 0.53)	2013-2016	-3.22 (-3.92 to -1.05)	2010-2015	-0.03 (-0.26 to 0.10)			2016-2021	-0.35 (-1.18 to 1.19)
	4	2010-2015	-0.16 (-0.19 to -0.14)	2010-2015	-0.04 (-0.10 to 0.00)	2016-2021	-0.38 (-1.27 to 1.32)	2015-2021	0.30 (0.21 to 0.53)				
	5	2015-2021	0.25 (0.23 to 0.27)	2015-2021	0.33 (0.30 to 0.38)								
Mauritius	0	1990-1998	0.41 (0.27 to 0.65)	1990-1995	0.50 (0.31 to 0.88)	1990-1992	3.46 (-0.88 to 7.10)	1990-1995	0.47 (0.28 to 0.99)	1990-1992	3.44 (-1.95 to 8.08)	1990-1992	3.02 (-0.80 to 6.18)
	1	1998-2010	0.03 (-0.08 to 0.14)	1995-2010	0.04 (-0.02 to 0.08)	1992-1999	-4.14 (-5.74 to -3.41)	1995-2010	0.10 (0.03 to 0.15)	1992-1999	-4.35 (-6.77 to -3.34)	1992-1999	-3.60 (-5.05 to -2.93)
	2	2010-2014	-0.87 (-1.32 to 0.06)	2010-2015	-1.62 (-1.84 to -1.37)	1999-2006	0.84 (0.09 to 2.77)	2010-2015	-1.25 (-1.68 to -0.96)	1999-2006	1.63 (0.52 to 4.27)	1999-2006	1.36 (0.61 to 3.29)
	3	2014-2019	0.65 (-0.72 to 0.99)	2015-2019	0.63 (0.17 to 0.84)	2006-2021	-0.65 (-1.09 to -0.41)	2015-2019	0.58 (-0.52 to 1.04)	2006-2021	-0.27 (-0.82 to 0.06)	2006-2021	-0.25 (-0.63 to -0.01)
	4	2019-2021	2.14 (1.08 to 2.63)	2019-2021	2.32 (1.60 to 2.76)			2019-2021	2.35 (1.42 to 2.90)				
Mexico	0	1990-2011	0.12 (0.06 to 0.17)	1990-2011	0.20 (0.14 to 0.26)	1990-1996	-0.06 (-0.69 to 1.03)	1990-2011	0.19 (0.14 to 0.25)	1990-2012	-1.37 (-1.60 to -1.19)	1990-1995	-0.18 (-0.90 to 1.81)
	1	2011-2015	-2.71 (-3.45 to -1.97)	2011-2015	-3.62 (-4.39 to -2.83)	1996-1999	-5.32 (-6.09 to -3.33)	2011-2015	-3.32 (-4.03 to -2.51)	2012-2021	0.81 (-0.15 to 2.49)	1995-2013	-1.04 (-2.59 to -0.08)
	2	2015-2021	-1.44 (-1.76 to -0.69)	2015-2021	-1.39 (-1.71 to -0.84)	1999-2012	-2.17 (-2.45 to -1.74)	2015-2021	-1.44 (-1.78 to -0.73)			2013-2021	-0.02 (-0.78 to 1.43)
	3					2012-2021	0.34 (-0.25 to 1.22)						
Micronesia	0	1990-2001	0.07 (-0.08 to 0.13)	1990-2001	0.14 (-0.22 to 0.22)	1990-1994	-0.43 (-0.58 to -0.29)	1990-2015	0.23 (0.20 to 0.26)	1990-1994	-0.52 (-0.87 to -0.33)	1990-1994	-0.29 (-0.54 to -0.14)
(Federated States of)	1	2001-2014	0.23 (0.18 to 0.40)	2001-2014	0.29 (-0.38 to 0.60)	1994-2001	0.88 (0.81 to 0.96)	2015-2021	-0.74 (-1.08 to -0.49)	1994-2001	0.51 (0.37 to 0.64)	1994-2001	0.39 (0.33 to 0.49)
	2	2014-2021	-0.36 (-0.53 to -0.24)	2014-2021	-0.73 (-0.99 to -0.53)	2001-2004	-0.30 (-0.47 to 0.00)			2001-2005	-0.70 (-0.84 to 0.51)	2001-2005	-0.43 (-0.54 to -0.26)
	3					2004-2010	-1.77 (-1.94 to -1.68)			2005-2008	-2.11 (-2.25 to -0.75)	2005-2008	-1.44 (-1.54 to -1.21)
	4					2010-2015	-1.40 (-1.61 to -1.28)			2008-2015	-1.59 (-1.89 to -1.48)	2008-2019	-1.09 (-1.12 to -1.04)
	5					2015-2018	-0.88 (-1.11 to -0.74)			2015-2018	-1.14 (-1.43 to -1.01)	2019-2021	-2.00 (-2.24 to -1.66)
	6					2018-2021	-1.73 (-2.07 to -1.55)			2018-2021	-2.09 (-2.44 to -1.89)		
Monaco	0	1990-1996	1.19 (1.00 to 1.32)	1990-1996	1.19 (1.00 to 1.32)	1990-1994	-1.39 (-1.54 to -1.17)	1990-1996	1.17 (0.95 to 1.32)	1990-1994	-1.19 (-1.52 to -0.92)	1990-1996	0.68 (0.53 to 0.76)
	1	1996-2004	2.25 (2.17 to 2.34)	1996-2004	2.19 (2.10 to 2.29)	1994-1997	-2.87 (-3.14 to -2.41)	1996-2004	2.22 (2.13 to 2.35)	1994-2001	-3.03 (-3.16 to -2.92)	1996-2001	1.36 (1.01 to 1.52)
	2	2004-2012	0.63 (0.52 to 0.71)	2004-2012	0.71 (0.59 to 0.80)	1997-2001	-3.62 (-3.79 to -3.50)	2004-2011	0.62 (0.46 to 0.73)	2001-2007	-1.08 (-1.34 to -0.96)	2001-2004	1.96 (1.59 to 2.09)
	3	2012-2021	1.52 (1.44 to 1.62)	2012-2021	1.71 (1.63 to 1.81)	2001-2007	-1.16 (-1.27 to -1.07)	2011-2021	1.61 (1.54 to 1.69)	2007-2011	-0.08 (-0.37 to 0.29)	2004-2010	0.44 (0.28 to 0.54)
	4					2007-2011	-0.03 (-0.20 to 0.22)			2011-2018	-0.89 (-1.14 to -0.78)	2010-2014	1.09 (0.67 to 1.34)
	5					2011-2018	-0.66 (-0.82 to -0.59)			2018-2021	0.97 (0.52 to 1.83)	2014-2021	1.50 (1.42 to 1.66)
	6					2018-2021	0.20 (-0.06 to 0.63)						
Mongolia	0	1990-2000	-0.24 (-0.32 to -0.18)	1990-1999	-0.18 (-0.21 to -0.15)	1990-1992	4.70 (2.69 to 6.24)	1990-1994	-0.28 (-0.70 to -0.04)	1990-1992	4.72 (2.40 to 6.37)	1990-1992	4.13 (1.79 to 5.87)
	1	2000-2005	1.08 (0.71 to 1.38)	1999-2002	0.54 (0.36 to 0.64)	1992-1998	0.47 (-0.03 to 0.75)	1994-2001	0.09 (-0.00 to 1.21)	1992-1997	1.04 (-2.08 to 1.44)	1992-1997	0.71 (-3.13 to 1.12)
	2	2005-2021	1.80 (1.76 to 1.84)	2002-2006	1.58 (1.47 to 1.67)	1998-2008	-4.94 (-5.10 to -4.79)	2001-2005	1.41 (0.92 to 2.13)	1997-2008	-4.54 (-4.74 to -4.36)	1997-2008	-3.59 (-3.83 to -3.39)
	3			2006-2010	2.19 (2.08 to 2.32)	2008-2011	0.78 (0.20 to 1.27)	2005-2016	2.10 (1.89 to 2.15)	2008-2011	2.08 (1.38 to 2.70)	2008-2011	2.40 (1.61 to 2.99)
	4			2010-2015	1.87 (1.72 to 1.94)	2011-2014	-4.60 (-5.11 to -3.86)	2016-2019	2.54 (2.25 to 2.72)	2011-2014	-5.31 (-5.95 to -4.40)	2011-2014	-3.70 (-4.32 to -2.82)
	5			2015-2019	2.39 (2.29 to 2.55)	2014-2019	-2.20 (-2.48 to -1.31)	2019-2021	1.14 (0.76 to 1.60)	2014-2019	-1.70 (-2.06 to -0.64)	2014-2019	-0.55 (-0.88 to 0.56)
	6			2019-2021	1.42 (1.23 to 1.68)	2019-2021	-5.27 (-6.42 to -3.92)			2019-2021	-5.60 (-6.92 to -3.86)	2019-2021	-3.43 (-4.75 to -2.00)
Montenegro	0	1990-1994	0.42 (0.28 to 0.69)	1990-1994	0.35 (0.19 to 0.65)	1990-1992	0.55 (-0.61 to 2.83)	1990-1994	0.45 (0.29 to 0.76)	1990-1992	-1.04 (-2.99 to 3.34)	1990-1992	-0.62 (-1.89 to 2.02)
	1	1994-2001	0.06 (-0.09 to 0.12)	1994-2001	0.07 (-0.12 to 0.14)	1992-2000	3.05 (-1.41 to 3.94)	1994-2001	0.06 (-0.13 to 0.13)	1992-2000	4.03 (-0.55 to 5.29)	1992-2000	3.03 (2.72 to 3.76)
	2	2001-2006	0.63 (0.51 to 0.76)	2001-2006	0.74 (0.60 to 0.91)	2000-2003	-1.60 (-3.98 to -0.06)	2001-2006	0.78 (0.58 to 0.97)	2000-2010	-3.21 (-3.68 to -2.76)	2000-2009	-2.25 (-2.57 to -1.99)
	3	2006-2011	1.48 (1.35 to 1.60)	2006-2010	1.54 (1.38 to 1.82)	2003-2008	-3.98 (-4.72 to -0.78)	2006-2011	1.63 (1.44 to 1.82)	2010-2017	-0.35 (-1.00 to 1.61)	2009-2019	0.20 (-0.04 to 0.69)
	4	2011-2019	2.19 (2.14 to 2.25)	2010-2019	2.40 (2.36 to 2.46)	2008-2019	-0.35 (-0.51 to 0.10)	2011-2019	2.49 (2.43 to 2.56)	2017-2021	-3.34 (-6.00 to -1.97)	2019-2021	-3.45 (-5.18 to -1.19)
	5	2019-2021	0.17 (-0.09 to 0.48)	2019-2021	0.33 (0.07 to 0.66)	2019-2021	-3.36 (-4.86 to -1.43)	2019-2021	0.05 (-0.25 to 0.31)				
Morocco	0	1990-1995	0.10 (0.03 to 0.18)	1990-1995	0.13 (0.04 to 0.20)	1990-2000	-0.00 (-0.17 to 0.16)	1990-1995	0.05 (-0.04 to 0.20)	1990-2000	-0.15 (-0.35 to 0.02)	1990-2000	-0.15 (-0.28 to -0.03)
	1	1995-2000	-0.34 (-0.42 to -0.28)	1995-2000	-0.37 (-0.46 to -0.26)	2000-2005	1.98 (1.63 to 2.73)	1995-2000	-0.30 (-0.50 to -0.20)	2000-2006	1.51 (1.23 to 2.14)	2000-2006	1.12 (0.87 to 1.56)
	2	2000-2011	0.25 (0.24 to 0.27)	2000-2011	0.34 (-0.01 to 0.37)	2005-2008	0.41 (-0.89 to 0.91)	2000-2011	0.27 (0.24 to 0.30)	2006-2009	-0.73 (-1.71 to 0.32)	2006-2009	-0.29 (-0.77 to 0.60)
	3	2011-2021	0.84 (0.82 to 0.86)	2011-2015	0.91 (0.27 to 0.97)	2008-2021	-2.11 (-2.19 to -2.05)	2011-2021	0.99 (0.96 to 1.03)	2009-2021	-2.67 (-2.78 to -2.59)	2009-2012	-2.06 (-2.29 to -1.58)
	4			2015-2021	1.06 (0.99 to 1.22)							2012-2021	-1.37 (-1.45 to -1.15)
Mozambique	0	1990-1994	0.34 (0.09 to 0.48)	1990-2001	1.03 (0.96 to 1.12)	1990-2004	-1.13 (-1.23 to -1.04)	1990-2001	0.93 (0.84 to 1.04)	1990-2004	-1.33 (-1.52 to -1.17)	1990-2004	-0.75 (-0.88 to -0.64)
	1	1994-1999	0.88 (0.77 to 1.08)	2001-2010	-0.06 (-0.20 to 0.07)	2004-2007	3.59 (2.72 to 4.01)	2001-2009	0.07 (-0.13 to 0.26)	2004-2007	4.31 (2.68 to 4.97)	2004-2007	3.01 (1.90 to 3.46)
	2	1999-2005	0.20 (0.09 to 0.38)	2010-2021	-0.95 (-1.05 to -0.86)	2007-2015	0.23 (-0.02 to 0.46)	2009-2021	-0.83 (-0.93 to -0.75)	2007-2016	-0.22 (-0.55 to 0.11)	2007-2016	-0.33 (-0.56 to -0.10)
	3	2005-2011	-0.15 (-0.31 to -0.02)			2015-2021	-1.94 (-2.35 to -1.60)			2016-2021	-2.16 (-3.27 to -1.51)	2016-2021	-1.86 (-2.60 to -1.41)
	4	2011-2017	-0.68 (-0.91 to -0.59)										
	5	2017-2021	-0.37 (-0.51 to -0.07)										

Location	Segment	Incidence		Prevalence		Mortality		YLD		YLL		DALY	
		Period	APC (95%CI)	Period	APC (95%CI)	Period	APC (95%CI)	Period	APC (95%CI)	Period	APC (95%CI)	Period	APC (95%CI)
Myanmar	0	1990-2000	-0.44 (-0.60 to -0.31)	1990-2000	-0.68 (-0.90 to -0.51)	1990-1995	0.26 (0.02 to 0.56)	1990-2000	-0.59 (-0.97 to -0.37)	1990-1995	0.26 (-0.08 to 0.77)	1990-1994	0.36 (-0.11 to 1.24)
	1	2000-2011	0.26 (0.13 to 0.51)	2000-2013	0.41 (0.28 to 0.60)	1995-2000	-1.27 (-1.51 to -0.95)	2000-2012	0.50 (0.32 to 0.85)	1995-2000	-1.29 (-1.71 to -0.75)	1994-2000	-1.03 (-1.39 to -0.67)
	2	2011-2019	-0.88 (-1.08 to -0.19)	2013-2021	-1.25 (-1.61 to -0.99)	2000-2004	-2.79 (-3.11 to -2.46)	2012-2021	-1.04 (-1.37 to -0.80)	2000-2004	-2.90 (-3.58 to -2.16)	2000-2004	-2.52 (-3.22 to -1.90)
	3	2019-2021	-2.80 (-3.53 to -1.34)			2004-2008	-4.69 (-4.96 to -4.42)			2004-2012	-4.72 (-4.89 to -4.56)	2004-2012	-3.74 (-3.90 to -3.63)
	4					2008-2012	-4.24 (-4.36 to -1.76)			2012-2016	-1.55 (-4.58 to -1.30)	2012-2016	-1.34 (-1.97 to -1.10)
	5					2012-2021	-1.73 (-1.80 to -1.67)			2016-2021	-1.99 (-2.44 to -1.78)	2016-2021	-1.84 (-2.24 to -1.67)
Namibia	0	1990-2001	-0.39 (-0.48 to -0.31)	1990-2002	-0.29 (-0.36 to -0.23)	1990-2003	-0.41 (-0.61 to -0.18)	1990-2001	-0.36 (-0.47 to -0.26)	1990-2003	-0.43 (-0.68 to -0.08)	1990-2015	-0.45 (-0.51 to -0.37)
	1	2001-2011	0.32 (0.22 to 0.61)	2002-2011	0.37 (0.26 to 0.70)	2003-2021	-1.60 (-1.76 to -1.46)	2001-2011	0.36 (0.24 to 0.74)	2003-2021	-1.37 (-1.62 to -1.21)	2015-2021	-1.30 (-2.36 to -0.85)
	2	2011-2019	-0.13 (-0.41 to 0.06)	2011-2019	0.02 (-0.23 to 0.19)			2011-2019	-0.00 (-0.27 to 0.18)				
	3	2019-2021	-1.92 (-2.45 to -0.96)	2019-2021	-1.50 (-1.97 to -0.71)			2019-2021	-1.84 (-2.42 to -0.99)				
Nauru	0	1990-2000	-0.56 (-0.76 to -0.42)	1990-2000	-0.52 (-0.87 to -0.34)	1990-1993	0.20 (-0.07 to 0.48)	1990-2001	-0.52 (-0.74 to -0.38)	1990-1993	0.21 (-0.10 to 0.54)	1990-1993	0.12 (-0.12 to 0.37)
	1	2000-2019	-0.03 (-0.07 to 0.05)	2000-2019	-0.03 (-0.09 to 0.10)	1993-1998	3.23 (3.05 to 3.48)	2001-2019	-0.01 (-0.07 to 0.11)	1993-1998	3.15 (2.94 to 3.42)	1993-1998	2.31 (2.13 to 2.53)
	2	2019-2021	-2.27 (-2.95 to -1.23)	2019-2021	-2.95 (-3.86 to -1.55)	1998-2001	2.44 (0.43 to 2.74)	2019-2021	-3.04 (-3.91 to -1.61)	1998-2001	2.35 (-0.10 to 2.66)	1998-2001	1.77 (-0.28 to 1.99)
	3					2001-2009	-0.08 (-0.19 to 0.04)			2001-2009	-0.08 (-0.20 to 0.05)	2001-2009	-0.04 (-0.19 to 0.12)
	4					2009-2012	-2.33 (-2.61 to -1.73)			2009-2012	-2.32 (-2.63 to -1.75)	2009-2012	-1.87 (-2.20 to -1.37)
	5					2012-2015	-4.06 (-4.30 to -3.66)			2012-2015	-4.44 (-4.70 to -4.00)	2012-2015	-3.48 (-3.70 to -3.06)
	6					2015-2021	-2.45 (-2.58 to -2.25)			2015-2021	-2.59 (-2.74 to -2.37)	2015-2021	-2.19 (-2.31 to -2.00)
Nepal	0	1990-2000	-0.18 (-0.39 to -0.08)	1990-2000	-0.12 (-0.32 to -0.03)	1990-1994	0.05 (-0.46 to 0.44)	1990-1999	-0.08 (-0.40 to 0.03)	1990-1994	0.00 (-0.58 to 0.62)	1990-1994	-0.03 (-0.51 to 0.55)
	1	2000-2011	0.27 (-0.04 to 0.40)	2000-2011	0.28 (0.05 to 0.40)	1994-2002	-2.32 (-2.44 to -2.21)	1999-2007	0.23 (-0.00 to 0.44)	1994-2002	-2.34 (-2.54 to -2.18)	1994-2002	-1.93 (-2.15 to -1.79)
	2	2011-2019	0.79 (0.26 to 0.97)	2011-2019	0.70 (0.32 to 0.89)	2002-2012	-0.28 (-0.39 to -0.19)	2007-2016	0.52 (0.34 to 0.79)	2002-2012	-0.42 (-0.67 to -0.32)	2002-2012	-0.26 (-0.53 to -0.17)
	3	2019-2021	1.86 (0.90 to 2.25)	2019-2021	1.80 (0.89 to 2.18)	2012-2018	0.63 (0.44 to 0.99)	2016-2021	1.10 (0.86 to 1.64)	2012-2018	0.08 (-0.15 to 0.60)	2012-2018	0.21 (-0.00 to 0.68)
	4					2018-2021	-1.17 (-1.78 to -0.69)			2018-2021	-1.28 (-2.15 to -0.75)	2018-2021	-0.75 (-1.56 to -0.30)
Netherlands	0	1990-1995	1.63 (1.43 to 1.72)	1990-1995	1.74 (1.52 to 1.84)	1990-1994	-2.37 (-2.94 to -1.34)	1990-1994	1.70 (1.36 to 1.85)	1990-1994	-1.95 (-2.74 to -0.38)	1990-1994	-0.41 (-0.66 to -0.04)
	1	1995-2000	1.97 (1.83 to 2.16)	1995-2001	2.15 (2.01 to 2.27)	1994-1997	-6.37 (-7.09 to -5.08)	1994-2001	2.09 (1.97 to 2.21)	1994-1997	-6.64 (-7.62 to -4.91)	1994-1997	-2.63 (-2.97 to -2.08)
	2	2000-2004	2.61 (2.50 to 2.77)	2001-2004	2.72 (2.54 to 2.83)	1997-2002	-1.32 (-1.93 to -0.40)	2001-2004	2.56 (2.32 to 2.68)	1997-2002	-1.13 (-2.07 to 0.49)	1997-2002	0.70 (0.38 to 1.16)
	3	2004-2011	0.89 (0.83 to 0.94)	2004-2011	0.93 (0.84 to 0.98)	2002-2012	-6.01 (-6.31 to -5.78)	2004-2011	0.93 (0.83 to 0.99)	2002-2012	-6.05 (-6.54 to -5.68)	2002-2010	-1.56 (-1.87 to -1.33)
	4	2011-2014	1.90 (1.76 to 2.01)	2011-2014	1.71 (1.47 to 1.83)	2012-2021	-2.87 (-3.18 to -2.47)	2011-2014	1.81 (1.56 to 1.93)	2012-2021	-3.05 (-3.48 to -2.49)	2010-2019	-0.26 (-0.48 to 0.28)
	5	2014-2019	0.36 (0.28 to 0.45)	2014-2019	0.47 (0.36 to 0.56)			2014-2019	0.44 (0.33 to 0.53)			2019-2021	-2.08 (-3.12 to -0.62)
	6	2019-2021	-1.50 (-1.77 to -1.26)	2019-2021	-1.93 (-2.26 to -1.68)			2019-2021	-1.82 (-2.12 to -1.57)				
New Zealand	0	1990-2000	-0.02 (-0.05 to 0.02)	1990-2000	-0.01 (-0.04 to 0.01)	1990-1997	-1.09 (-4.06 to 1.44)	1990-2001	0.11 (0.08 to 0.14)	1990-1997	-0.84 (-2.70 to 1.02)	1990-1995	0.16 (-0.15 to 0.59)
	1	2000-2008	0.29 (0.25 to 0.43)	2000-2009	0.40 (0.37 to 0.43)	1997-2004	-18.67 (-22.73 to -2.41)	2001-2009	0.39 (0.35 to 0.51)	1997-2004	-16.72 (-18.80 to -15.10)	1995-1998	-2.95 (-3.53 to -2.05)
	2	2008-2015	0.18 (0.09 to 0.23)	2009-2015	0.16 (0.11 to 0.20)	2004-2014	-3.46 (-19.40 to -2.40)	2009-2015	0.22 (0.12 to 0.28)	2004-2014	-3.65 (-6.67 to -2.65)	1998-2001	-8.07 (-8.78 to -7.09)
	3	2015-2019	-1.55 (-1.64 to -1.48)	2015-2019	-1.31 (-1.37 to -1.25)	2014-2021	-0.14 (-2.47 to 5.59)	2015-2019	-1.39 (-1.48 to -1.31)	2014-2021	0.22 (-1.65 to 4.13)	2001-2004	-3.79 (-4.39 to -1.27)
	4	2019-2021	-0.54 (-0.79 to -0.36)	2019-2021	-0.16 (-0.32 to -0.01)			2019-2021	-0.25 (-0.49 to -0.07)			2004-2021	-0.55 (-0.66 to -0.40)
Nicaragua	0	1990-1994	-0.50 (-0.75 to -0.36)	1990-1998	-0.26 (-0.38 to -0.16)	1990-1996	0.29 (-0.49 to 1.93)	1990-1994	-0.42 (-0.85 to -0.20)	1990-1996	0.17 (-0.51 to 1.21)	1990-1996	0.02 (-0.50 to 0.82)
	1	1994-2000	-0.08 (-0.19 to 0.07)	1998-2015	0.36 (0.33 to 0.39)	1996-2006	-1.52 (-3.01 to -1.21)	1994-2001	0.02 (-0.10 to 0.29)	1996-2006	-2.07 (-3.08 to -1.75)	1996-2006	-1.52 (-2.15 to -1.28)
	2	2000-2015	0.30 (0.28 to 0.32)	2015-2018	-0.98 (-1.16 to -0.56)	2006-2019	-0.80 (-0.99 to 0.17)	2001-2015	0.42 (0.38 to 0.49)	2006-2019	-0.69 (-0.92 to -0.29)	2006-2019	-0.49 (-0.65 to -0.22)
	3	2015-2018	-0.87 (-0.99 to -0.70)	2018-2021	-0.01 (-0.33 to 0.49)	2019-2021	-7.82 (-10.83 to -4.58)	2015-2019	-0.96 (-1.25 to -0.74)	2019-2021	-8.91 (-11.39 to -5.53)	2019-2021	-6.76 (-8.43 to -4.23)
	4	2018-2021	-0.14 (-0.30 to 0.20)					2019-2021	0.51 (-0.03 to 0.88)				
Niger	0	1990-1994	-0.36 (-0.71 to -0.20)	1990-1994	-0.43 (-0.92 to -0.21)	1990-1994	-0.87 (-1.36 to -0.14)	1990-1995	-0.40 (-0.72 to -0.24)	1990-2005	-1.84 (-1.98 to -1.63)	1990-2005	-1.38 (-1.48 to -1.22)
	1	1994-2005	0.01 (-0.05 to 0.07)	1994-2005	0.03 (-0.05 to 0.15)	1994-1998	-3.06 (-3.77 to -2.47)	1995-2001	0.19 (0.01 to 0.50)	2005-2010	-3.42 (-5.06 to -2.73)	2005-2010	-2.21 (-3.23 to -1.74)
	2	2005-2010	0.26 (0.12 to 0.44)	2005-2010	0.45 (0.02 to 0.71)	1998-2005	-1.51 (-1.74 to -0.85)	2001-2005	-0.08 (-0.33 to 0.52)	2010-2015	3.00 (2.17 to 4.49)	2010-2015	1.94 (1.38 to 2.83)
	3	2010-2019	-0.31 (-0.45 to -0.26)	2010-2019	-0.31 (-0.52 to 0.41)	2005-2010	-2.83 (-3.29 to -2.47)	2005-2011	0.49 (-0.74 to 0.74)	2015-2021	-1.43 (-2.13 to -0.84)	2015-2021	-1.04 (-1.52 to -0.64)
	4	2019-2021	0.23 (-0.20 to 0.43)	2019-2021	0.31 (-0.25 to 0.56)	2010-2014	3.09 (2.56 to 3.49)	2011-2014	-0.71 (-0.87 to 0.05)				
	5					2014-2021	-0.55 (-0.80 to -0.33)	2014-2021	-0.14 (-0.34 to 0.11)				
Nigeria	0	1990-1999	-0.22 (-0.43 to -0.10)	1990-1999	-0.27 (-0.47 to -0.14)	1990-1995	-2.04 (-2.39 to -1.72)	1990-1999	-0.20 (-0.45 to -0.05)	1990-1995	-1.69 (-2.04 to -1.26)	1990-1995	-1.34 (-1.60 to -1.00)
	1	1999-2019	0.26 (0.20 to 0.30)	1999-2019	0.33 (0.22 to 0.38)	1995-2000	-4.62 (-5.20 to -4.30)	1999-2019	0.37 (-0.07 to 0.42)	1995-2000	-3.74 (-4.53 to -3.37)	1995-2000	-2.73 (-3.35 to -2.47)
	2	2019-2021	1.49 (0.51 to 1.90)	2019-2021	1.48 (0.40 to 1.91)	2000-2010	-1.38 (-1.55 to -1.25)	2019-2021	1.25 (0.38 to 1.70)	2000-2010	-1.34 (-1.55 to -1.15)	2000-2010	-0.76 (-0.92 to -0.62)
	3					2010-2014	0.77 (0.19 to 1.48)			2010-2014	1.05 (0.39 to 1.85)	2010-2014	0.72 (0.26 to 1.26)
	4					2014-2021	-2.06 (-2.29 to -1.88)			2014-2021	-2.10 (-2.37 to -1.88)	2014-2021	-1.15 (-1.36 to -0.99)
Niue	0	1990-2000	-0.08 (-0.20 to -0.01)	1990-2001	-0.01 (-0.28 to 0.06)	1990-2018	-0.65 (-1.16 to -0.20)	1990-2017	0.07 (0.05 to 0.10)	1990-2018	-0.87 (-1.49 to -0.32)	1990-2019	-0.45 (-0.80 to -0.14)
	1	2000-2016	0.12 (0.09 to 0.20)	2001-2015	0.14 (0.09 to 0.46)	2018-2021	36.99 (21.42 to 68.03)	2017-2021	-0.57 (-1.24 to -0.24)	2018-2021	47.80 (26.68 to 90.29)	2019-2021	60.86 (35.17 to 82.22)
	2	2016-2021	-0.35 (-0.67 to -0.18)	2015-2021	-0.46 (-0.79 to -0.27)								
North Macedonia	0	1990-2002	0.12 (0.05 to 0.17)	1990-2002	0.18 (0.12 to 0.23)	1990-1998	0.44 (0.05 to 0.82)	1990-2005	0.26 (0.21 to 0.33)	1990-1998	0.38 (0.07 to 0.71)	1990-1998	0.34 (0.11 to 0.61)
	1	2002-2006	0.51 (0.22 to 1.27)	2002-2006	0.69 (0.35 to 0.96)	1998-2001	-4.68 (-5.19 to 0.28)	2005-2019	1.67 (0.28 to 1.82)	1998-2001	-6.25 (-6.84 to 0.09)	1998-2001	-4.99 (-5.45 to -3.79)
	2	2006-2019	1.40 (1.36 to 1.54)	2006-2016	1.62 (1.51 to 1.66)	2001-2010	-2.76 (-3.54 to -2.23)	2019-2021	0.93 (0.48 to 1.64)	2001-2007	-2.29 (-5.70 to -1.19)	2001-2011	-1.88 (-2.15 to -1.66)
	3	2019-2021	0.48 (0.13 to 1.15)	2016-2019	2.04 (1.77 to 2.26)	2010-2021	-1.91 (-2.23 to -0.98)			2007-2010	-4.05 (-4.87 to -2.56)	2011-2015	-0.06 (-0.83 to 0.84)
	4			2019-2021	0.68 (0.33 to 1.18)					2010-2015	-0.77 (-1.74 to 0.75)	2015-2021	-1.97 (-3.02 to -1.55)
	5									2015-2021	-3.54 (-4.49 to -2.86)		
Northern Mariana Islands	0	1990-1995	0.29 (0.16 to 0.41)	1990-1995	0.24 (0.13 to 0.34)	1990-2012	-0.27 (-0.94 to 3.32)	1990-1994	0.48 (0.25 to 0.76)	1990-2015	-0.55 (-1.19 to 18.17)	1990-1998	-1.55 (-6.71 to 0.14)
	1	1995-2000	-0.72 (-0.83 to -0.62)	1995-2000	-0.53 (-0.62 to -0.44)	2012-2021	-4.21 (-16.21 to -1.71)	1994-2000	-0.43 (-0.72 to -0.30)	2015-2021	-6.56 (-27.05 to -1.62)	1998-2008	0.89 (-6.29 to 6.01)
	2	2000-2005	1.43 (1.34 to 1.52)	2000-2005	1.05 (0.96 to 1.13)			2000-2004	1.09 (0.81 to 1.47)			2008-2021	-1.47 (-4.30 to 2.63)

Location	Segment	Incidence		Prevalence		Mortality		YLD		YLL		DALY	
		Period	APC (95%CI)	Period	APC (95%CI)	Period	APC (95%CI)	Period	APC (95%CI)	Period	APC (95%CI)	Period	APC (95%CI)
	3	2005-2010	-0.02 (-0.13 to 0.14)	2005-2010	0.03 (-0.08 to 0.16)			2004-2008	0.40 (-0.07 to 0.65)				
	4	2010-2015	-0.61 (-0.85 to -0.48)	2010-2015	-0.60 (-0.76 to -0.50)			2008-2015	-0.48 (-0.76 to -0.35)				
	5	2015-2021	0.26 (0.16 to 0.39)	2015-2021	0.32 (0.24 to 0.41)			2015-2021	0.25 (0.09 to 0.50)				
Norway	0	1990-1993	1.19 (1.14 to 1.29)	1990-1994	1.21 (1.16 to 1.34)	1990-2007	-0.97 (-1.22 to -0.71)	1990-2000	1.18 (1.13 to 1.21)	1990-2008	-0.90 (-1.19 to -0.61)	1990-1992	1.30 (-0.06 to 2.23)
	1	1993-2000	1.07 (1.04 to 1.09)	1994-2000	1.08 (0.96 to 1.11)	2007-2021	-6.81 (-7.21 to -6.42)	2000-2006	1.38 (1.31 to 1.51)	2008-2018	-8.17 (-9.76 to -7.41)	1992-1995	-1.03 (-1.53 to 1.21)
	2	2000-2005	1.35 (1.32 to 1.39)	2000-2005	1.39 (1.30 to 1.48)			2006-2021	0.96 (0.95 to 0.98)	2018-2021	-2.02 (-6.13 to 3.72)	1995-1998	1.58 (0.48 to 2.05)
	3	2005-2009	1.08 (1.04 to 1.13)	2005-2009	1.08 (1.02 to 1.27)							1998-2007	0.51 (-0.81 to 0.67)
	4	2009-2015	0.87 (0.83 to 0.90)	2009-2015	0.90 (0.79 to 0.96)							2007-2015	-1.22 (-1.80 to -0.92)
	5	2015-2018	1.07 (1.01 to 1.11)	2015-2018	1.11 (0.94 to 1.17)							2015-2021	-0.02 (-0.45 to 0.85)
	6	2018-2021	0.87 (0.78 to 0.91)	2018-2021	0.89 (0.72 to 0.98)								
Oman	0	1990-2000	-0.09 (-0.32 to 0.03)	1990-2000	-0.12 (-0.29 to -0.01)	1990-1998	-1.30 (-1.76 to -0.62)	1990-2000	-0.06 (-0.26 to 0.05)	1990-2016	-1.92 (-2.05 to -1.73)	1990-1992	-3.21 (-4.49 to -1.60)
	1	2000-2019	0.19 (0.14 to 0.28)	2000-2019	0.21 (0.17 to 0.29)	1998-2003	-3.67 (-5.14 to -2.80)	2000-2019	0.22 (0.18 to 0.30)	2016-2021	-5.53 (-7.86 to -4.19)	1992-1996	0.31 (-0.31 to 1.31)
	2	2019-2021	-1.82 (-2.50 to -0.72)	2019-2021	-1.51 (-2.11 to -0.42)	2003-2010	-0.87 (-1.33 to 0.46)	2019-2021	-1.73 (-2.28 to -0.71)			1996-2002	-1.89 (-2.86 to -1.48)
	3					2010-2016	-2.28 (-3.08 to -1.70)					2002-2016	-0.87 (-0.99 to -0.70)
	4					2016-2021	-5.01 (-6.07 to -4.37)					2016-2021	-3.10 (-3.77 to -2.60)
Pakistan	0	1990-1999	0.38 (0.30 to 0.43)	1990-2000	0.40 (0.38 to 0.41)	1990-1996	5.45 (5.20 to 5.73)	1990-2001	0.23 (0.21 to 0.25)	1990-1995	5.89 (5.69 to 6.15)	1990-1995	4.61 (4.43 to 4.83)
	1	1999-2006	0.10 (-0.00 to 0.45)	2000-2005	0.02 (-0.04 to 0.06)	1996-2003	1.31 (1.01 to 1.56)	2001-2005	-0.03 (-0.15 to 0.05)	1995-1998	2.65 (2.14 to 3.77)	1995-1998	2.24 (1.79 to 3.10)
	2	2006-2011	0.27 (0.08 to 0.37)	2005-2015	0.29 (0.28 to 0.31)	2003-2021	-1.00 (-1.06 to -0.93)	2005-2009	0.56 (0.48 to 0.69)	1998-2003	1.08 (0.58 to 1.30)	1998-2003	0.88 (0.49 to 1.07)
	3	2011-2015	0.42 (0.30 to 0.85)	2015-2021	0.85 (0.83 to 0.88)			2009-2015	0.34 (0.25 to 0.38)	2003-2012	-1.07 (-1.45 to -0.96)	2003-2012	-0.80 (-1.11 to -0.71)
	4	2015-2021	0.81 (0.73 to 0.93)					2015-2021	0.82 (0.78 to 0.88)	2012-2015	0.01 (-0.61 to 0.29)	2012-2015	0.08 (-0.42 to 0.31)
	5									2015-2021	-1.39 (-1.71 to -1.21)	2015-2021	-0.93 (-1.22 to -0.79)
Palau	0	1990-1995	0.13 (0.09 to 0.18)	1990-1994	0.20 (0.14 to 0.31)	1990-1995	-0.61 (-1.52 to -0.24)	1990-2005	0.02 (-0.01 to 0.04)	1990-1996	-0.74 (-1.10 to -0.51)	1990-1996	-0.44 (-0.61 to -0.33)
	1	1995-2000	-0.18 (-0.24 to -0.14)	1994-2000	-0.10 (-0.19 to -0.07)	1995-2003	0.50 (0.17 to 0.81)	2005-2009	0.34 (0.17 to 0.50)	1996-2003	0.54 (0.23 to 0.75)	1996-2003	0.33 (0.20 to 0.44)
	2	2000-2006	0.09 (0.03 to 0.12)	2000-2005	0.10 (0.03 to 0.16)	2003-2006	1.75 (1.11 to 2.05)	2009-2021	-0.21 (-0.25 to -0.18)	2003-2006	1.82 (1.26 to 2.09)	2003-2006	1.29 (0.96 to 1.45)
	3	2006-2010	0.22 (0.16 to 0.27)	2005-2010	0.29 (0.26 to 0.34)	2006-2014	0.68 (0.40 to 0.80)			2006-2014	0.68 (0.45 to 0.80)	2006-2014	0.43 (0.33 to 0.49)
	4	2010-2021	-0.11 (-0.13 to -0.10)	2010-2021	-0.28 (-0.30 to -0.27)	2014-2021	-0.99 (-1.21 to -0.79)			2014-2021	-0.93 (-1.14 to -0.76)	2014-2021	-0.70 (-0.79 to -0.61)
Palestine	0	1990-1999	-0.17 (-0.23 to -0.13)	1990-1999	-0.29 (-0.44 to -0.14)	1990-2003	-2.47 (-2.77 to -2.19)	1990-1998	-0.34 (-0.52 to -0.23)	1990-2003	-3.01 (-3.71 to -2.43)	1990-2002	-1.81 (-1.98 to -1.66)
	1	1999-2012	0.18 (0.15 to 0.21)	1999-2011	0.25 (-0.36 to 0.40)	2003-2007	2.07 (0.73 to 3.77)	1998-2012	0.22 (0.10 to 0.28)	2003-2007	2.71 (-3.93 to 4.97)	2002-2007	1.01 (0.54 to 1.92)
	2	2012-2019	0.61 (0.52 to 0.69)	2011-2015	0.69 (0.04 to 0.86)	2007-2010	-4.32 (-5.10 to -2.59)	2012-2019	0.93 (0.23 to 1.07)	2007-2010	-5.91 (-7.40 to 3.13)	2007-2010	-2.46 (-2.95 to -1.40)
	3	2019-2021	1.47 (1.09 to 1.69)	2015-2019	1.20 (0.63 to 1.37)	2010-2021	-2.03 (-2.27 to -1.53)	2019-2021	1.86 (1.08 to 2.21)	2010-2013	-0.03 (-5.08 to 1.74)	2010-2021	-0.34 (-0.48 to -0.12)
	4			2019-2021	1.94 (1.35 to 2.25)					2013-2021	-2.43 (-4.02 to -1.80)		
Panama	0	1990-2015	0.22 (0.20 to 0.24)	1990-1994	-0.03 (-0.44 to 0.20)	1990-1996	4.02 (2.96 to 5.54)	1990-1994	-0.08 (-0.65 to 0.24)	1990-1996	3.74 (3.18 to 4.37)	1990-1996	2.61 (1.89 to 3.51)
	1	2015-2021	1.00 (0.84 to 1.21)	1994-2014	0.28 (0.25 to 0.35)	1996-2000	-3.49 (-5.27 to -1.56)	1994-2015	0.28 (0.25 to 0.48)	1996-2000	-4.23 (-5.52 to -3.01)	1996-2000	-2.74 (-4.06 to -1.41)
	2			2014-2019	0.86 (0.28 to 1.10)	2000-2021	-0.57 (-0.75 to -0.26)	2015-2021	1.21 (0.99 to 1.50)	2000-2005	0.10 (-0.68 to 1.64)	2000-2021	-0.23 (-0.37 to -0.04)
	3			2019-2021	1.88 (1.17 to 2.22)					2005-2009	-2.14 (-3.45 to -1.08)		
	4									2009-2014	1.19 (0.36 to 2.63)		
	5									2014-2021	-1.90 (-2.70 to -1.35)		
Papua New Guinea	0	1990-1997	0.13 (0.11 to 0.16)	1990-1993	0.28 (0.22 to 0.36)	1990-2001	0.80 (0.41 to 1.97)	1990-1999	0.21 (0.18 to 0.27)	1990-2001	0.95 (0.54 to 1.85)	1990-2001	0.78 (0.44 to 1.55)
	1	1997-2005	-0.01 (-0.04 to 0.01)	1993-1999	0.15 (0.09 to 0.18)	2001-2021	0.06 (-0.24 to 0.20)	1999-2002	-0.13 (-0.21 to 0.05)	2001-2021	0.02 (-0.23 to 0.18)	2001-2021	0.04 (-0.18 to 0.17)
	2	2005-2014	0.11 (0.10 to 0.13)	1999-2005	-0.02 (-0.07 to 0.01)			2002-2014	0.08 (0.05 to 0.16)				
	3	2014-2021	0.43 (0.41 to 0.44)	2005-2010	0.17 (0.14 to 0.22)			2014-2021	0.28 (0.22 to 0.39)				
	4			2010-2015	-0.00 (-0.04 to 0.03)								
	5			2015-2019	0.41 (0.38 to 0.46)								
	6			2019-2021	0.13 (0.06 to 0.23)								
Paraguay	0	1990-1998	-0.07 (-0.12 to -0.06)	1990-1999	-0.01 (-0.02 to 0.00)	1990-1996	1.23 (0.54 to 2.24)	1990-2000	-0.03 (-0.08 to -0.01)	1990-2006	0.08 (-0.23 to 0.68)	1990-2021	-0.01 (-0.09 to 0.07)
	1	1998-2001	0.05 (-0.02 to 0.35)	1999-2002	0.24 (0.18 to 0.28)	1996-2001	-0.82 (-2.26 to 1.09)	2000-2003	0.40 (-0.02 to 0.62)	2006-2021	-1.00 (-1.76 to -0.66)		
	2	2001-2006	0.49 (0.47 to 0.56)	2002-2006	0.58 (0.56 to 0.61)	2001-2004	2.57 (-0.86 to 3.29)	2003-2007	0.66 (0.54 to 1.02)				
	3	2006-2012	0.90 (0.84 to 0.93)	2006-2015	1.03 (1.02 to 1.04)	2004-2019	-0.49 (-0.66 to 1.75)	2007-2014	1.01 (0.96 to 1.36)				
	4	2012-2016	1.09 (1.00 to 1.25)	2015-2021	1.52 (1.50 to 1.54)	2019-2021	-3.32 (-5.28 to -0.65)	2014-2021	1.44 (1.39 to 1.50)				
	5	2016-2021	1.41 (1.37 to 1.48)										
Peru	0	1990-1996	-0.07 (-0.21 to 0.03)	1990-1996	-0.02 (-0.10 to 0.07)	1990-2015	-1.72 (-3.00 to -1.19)	1990-1996	-0.03 (-0.23 to 0.11)	1990-2015	-1.92 (-3.07 to -1.60)	1990-1994	0.71 (-0.23 to 2.60)
	1	1996-2006	0.54 (0.47 to 0.59)	1996-2006	0.63 (0.58 to 0.67)	2015-2021	1.21 (-1.63 to 8.94)	1996-2007	0.70 (0.58 to 0.77)	2015-2021	0.99 (-1.77 to 7.83)	1994-1997	-3.19 (-3.95 to -1.44)
	2	2006-2021	0.80 (0.77 to 0.83)	2006-2021	0.85 (0.83 to 0.88)			2007-2021	0.90 (0.86 to 0.98)			1997-2016	-0.73 (-0.93 to 0.41)
	3											2016-2019	3.23 (-1.75 to 4.32)
	4											2019-2021	-3.83 (-6.86 to 0.86)
Philippines	0	1990-1996	-0.57 (-1.19 to -0.27)	1990-2011	-0.17 (-0.27 to 0.04)	1990-1996	-0.67 (-1.17 to -0.32)	1990-1999	-0.38 (-1.07 to -0.13)	1990-1997	-0.77 (-1.10 to -0.53)	1990-1996	-0.77 (-1.03 to -0.59)
	1	1996-2011	-0.10 (-0.31 to 0.42)	2011-2019	-0.78 (-1.17 to -0.00)	1996-2006	0.68 (0.51 to 0.93)	1999-2010	-0.01 (-0.92 to 0.84)	1997-2005	0.77 (0.56 to 1.10)	1996-2006	0.34 (0.25 to 0.47)
	2	2011-2019	-0.52 (-0.80 to -0.09)	2019-2021	-2.68 (-3.58 to -1.01)	2006-2014	-0.59 (-0.99 to -0.39)	2010-2019	-0.72 (-1.03 to 0.04)	2005-2014	-0.60 (-0.89 to -0.44)	2006-2016	-0.50 (-0.64 to -0.41)
	3	2019-2021	-1.86 (-2.53 to -0.71)			2014-2019	0.48 (0.16 to 1.16)	2019-2021	-2.52 (-3.38 to -0.97)	2014-2019	0.58 (0.24 to 1.30)	2016-2019	0.58 (0.12 to 0.84)
	4					2019-2021	-1.98 (-3.09 to -0.77)			2019-2021	-2.33 (-3.53 to -1.00)	2019-2021	-2.65 (-3.30 to -2.00)
Poland	0	1990-2001	-0.08 (-0.14 to -0.04)	1990-2002	0.08 (-0.01 to 0.13)	1990-1992	-1.12 (-3.41 to 0.89)	1990-2002	0.10 (0.05 to 0.14)	1990-1992	-1.31 (-4.15 to 1.07)	1990-1992	-1.14 (-2.47 to -0.17)
	1	2001-2006	0.39 (0.19 to 0.63)	2002-2006	0.60 (0.19 to 1.12)	1992-1996	-6.98 (-8.28 to -6.22)	2002-2007	0.71 (0.51 to 0.93)	1992-1996	-7.23 (-9.54 to -6.28)	1992-1996	-5.45 (-5.90 to -5.00)

Location	Segment	Incidence		Prevalence		Mortality		YLD		YLL		DALY	
		Period	APC (95%CI)	Period	APC (95%CI)	Period	APC (95%CI)	Period	APC (95%CI)	Period	APC (95%CI)	Period	APC (95%CI)
	2	2006-2011	1.42 (1.30 to 1.59)	2006-2010	1.46 (1.12 to 2.20)	1996-2000	-13.25 (-14.79 to -12.27)	2007-2011	1.92 (1.63 to 2.18)	1996-2000	-12.77 (-14.48 to -11.00)	1996-1999	-9.30 (-10.06 to -8.18)
	3	2011-2014	3.31 (3.13 to 3.48)	2010-2015	2.68 (2.50 to 2.90)	2000-2003	-6.99 (-10.23 to -3.64)	2011-2014	3.47 (3.24 to 3.66)	2000-2003	-6.64 (-9.50 to -1.82)	1999-2002	-5.72 (-6.36 to -0.64)
	4	2014-2019	0.47 (0.37 to 0.60)	2015-2021	0.46 (0.34 to 0.57)	2003-2010	-2.17 (-2.67 to -0.65)	2014-2019	0.93 (0.83 to 1.07)	2003-2009	-1.63 (-3.77 to 0.62)	2002-2021	-0.58 (-0.75 to -0.39)
	5	2019-2021	-0.72 (-1.03 to -0.28)			2010-2014	-6.00 (-7.37 to -4.67)	2019-2021	-0.49 (-0.85 to 0.01)	2009-2021	-3.71 (-4.53 to -3.27)		
	6					2014-2021	-3.30 (-4.08 to -0.95)						
Portugal	0	1990-1995	1.55 (1.47 to 1.63)	1990-1996	1.66 (1.54 to 1.74)	1990-2003	-3.96 (-4.09 to -3.83)	1990-1996	1.72 (1.55 to 1.82)	1990-2003	-3.99 (-4.17 to -3.78)	1990-1995	-1.28 (-1.54 to -1.10)
	1	1995-2003	2.12 (2.00 to 2.17)	1996-2010	2.21 (2.19 to 2.24)	2003-2006	-9.82 (-10.44 to -9.08)	1996-2005	2.15 (1.76 to 2.26)	2003-2006	-10.71 (-11.75 to -8.87)	1995-2003	-0.42 (-0.52 to -0.22)
	2	2003-2010	2.31 (2.12 to 2.39)	2010-2015	0.65 (0.56 to 0.73)	2006-2009	-2.39 (-3.44 to -1.59)	2005-2009	2.45 (2.03 to 2.59)	2006-2009	-1.89 (-3.47 to -0.48)	2003-2006	-1.74 (-2.04 to -1.26)
	3	2010-2015	1.34 (1.25 to 2.31)	2015-2021	1.66 (1.57 to 1.76)	2009-2014	-6.04 (-7.16 to -5.52)	2009-2016	1.08 (0.98 to 2.51)	2009-2014	-6.50 (-8.62 to -5.73)	2006-2009	1.37 (0.87 to 1.74)
	4	2015-2019	1.68 (1.32 to 1.83)			2014-2021	-2.51 (-2.93 to -1.99)	2016-2019	1.79 (1.00 to 1.90)	2014-2021	-1.99 (-2.66 to -1.02)	2009-2014	-0.59 (-1.13 to -0.34)
	5	2019-2021	1.27 (1.06 to 1.57)					2019-2021	1.36 (1.10 to 1.67)			2014-2021	0.97 (0.76 to 1.22)
Puerto Rico	0	1990-1999	-0.73 (-0.88 to -0.60)	1990-1994	-1.30 (-1.93 to -0.97)	1990-1995	1.14 (-0.04 to 2.58)	1990-1999	-0.90 (-1.09 to -0.74)	1990-1995	1.20 (-0.08 to 2.81)	1990-1995	0.55 (-0.26 to 1.59)
	1	1999-2010	0.30 (0.19 to 0.45)	1994-2000	-0.66 (-0.86 to 0.17)	1995-2002	-4.56 (-6.03 to -3.91)	1999-2010	0.28 (0.16 to 0.46)	1995-2002	-4.70 (-6.54 to -3.94)	1995-2002	-3.48 (-4.58 to -2.96)
	2	2010-2021	-0.67 (-0.79 to -0.56)	2000-2011	0.26 (0.13 to 0.38)	2002-2015	-2.47 (-2.81 to -2.07)	2010-2021	-0.81 (-0.95 to -0.69)	2002-2015	-2.29 (-2.71 to -1.82)	2002-2015	-1.51 (-1.92 to -1.17)
	3			2011-2015	-1.44 (-1.89 to -1.02)	2015-2021	0.95 (-0.14 to 2.84)			2015-2021	1.48 (0.18 to 3.90)	2015-2021	0.38 (-0.49 to 2.37)
	4			2015-2021	-0.53 (-0.73 to -0.18)								
Qatar	0	1990-1994	0.34 (0.24 to 0.44)	1990-1994	0.05 (-0.05 to 0.20)	1990-1996	0.44 (-0.49 to 1.61)	1990-1995	0.02 (-0.09 to 0.16)	1990-1996	0.95 (-0.32 to 2.78)	1990-1996	0.51 (-0.21 to 1.52)
	1	1994-2000	-0.36 (-0.42 to -0.32)	1994-2000	-0.37 (-0.43 to -0.32)	1996-2000	-5.23 (-7.09 to -3.65)	1995-2000	-0.52 (-0.68 to -0.42)	1996-2000	-6.86 (-9.40 to -4.66)	1996-2000	-4.19 (-5.82 to -2.87)
	2	2000-2011	0.37 (0.33 to 0.40)	2000-2011	0.53 (0.51 to 0.55)	2000-2007	-0.25 (-0.93 to 0.82)	2000-2004	0.73 (0.59 to 0.92)	2000-2007	-0.84 (-1.60 to 1.10)	2000-2006	-0.15 (-0.81 to 1.61)
	3	2011-2014	1.27 (0.41 to 1.34)	2011-2015	1.48 (1.40 to 1.58)	2007-2012	-6.51 (-8.09 to -5.44)	2004-2010	0.36 (0.18 to 0.43)	2007-2019	-4.48 (-4.84 to -4.10)	2006-2021	-1.46 (-1.77 to -1.29)
	4	2014-2021	1.05 (0.97 to 1.11)	2015-2019	1.17 (1.02 to 1.26)	2012-2019	-4.48 (-4.91 to -2.86)	2010-2021	1.29 (1.26 to 1.33)	2019-2021	-13.66 (-16.66 to -9.53)		
	5			2019-2021	1.77 (1.51 to 1.96)	2019-2021	-9.96 (-12.55 to -7.23)						
Republic of Korea	0	1990-2000	0.36 (0.26 to 0.45)	1990-1994	0.82 (0.49 to 1.43)	1990-1997	-6.88 (-7.19 to -6.63)	1990-2001	0.52 (0.33 to 0.72)	1990-1997	-7.24 (-7.53 to -7.01)	1990-1996	-3.99 (-4.25 to -3.80)
	1	2000-2009	1.26 (1.14 to 1.42)	1994-2000	0.33 (-0.13 to 1.24)	1997-2001	-4.03 (-5.00 to -3.30)	2001-2009	1.19 (0.36 to 1.49)	1997-2002	-5.25 (-6.65 to -4.78)	1996-2006	-2.30 (-3.82 to -2.16)
	2	2009-2015	0.21 (-0.04 to 0.54)	2000-2009	1.19 (-0.01 to 1.41)	2001-2006	-8.99 (-9.49 to -7.45)	2009-2013	0.14 (-0.07 to 1.38)	2002-2006	-9.02 (-9.63 to -5.32)	2006-2009	-0.80 (-2.46 to -0.46)
	3	2015-2021	-0.61 (-0.95 to -0.41)	2009-2015	-0.03 (-0.61 to 0.30)	2006-2014	-6.65 (-8.67 to -6.32)	2013-2019	-0.34 (-0.58 to 0.06)	2006-2009	-4.72 (-8.92 to -4.06)	2009-2014	-1.69 (-2.27 to -1.27)
	4			2015-2021	-0.96 (-1.36 to -0.70)	2014-2017	-3.82 (-6.53 to -1.86)	2019-2021	-1.57 (-2.06 to -0.78)	2009-2013	-7.42 (-8.41 to -5.30)	2014-2021	-0.74 (-0.97 to -0.35)
	5					2017-2021	-0.40 (-1.29 to 1.09)			2013-2016	-4.74 (-6.84 to -1.79)		
	6									2016-2021	-0.12 (-0.68 to 0.80)		
Republic of Moldova	0	1990-2000	0.08 (-0.01 to 0.13)	1990-2005	0.21 (0.17 to 0.24)	1990-1995	8.15 (4.50 to 12.96)	1990-2005	0.23 (0.17 to 0.28)	1990-1995	7.50 (3.89 to 12.32)	1990-1995	5.70 (3.60 to 7.89)
	1	2000-2006	0.40 (0.28 to 0.59)	2005-2009	1.71 (1.26 to 1.93)	1995-2012	-5.60 (-6.20 to -5.19)	2005-2008	1.48 (0.51 to 2.32)	1995-2012	-5.55 (-6.20 to -5.09)	1995-2011	-4.09 (-4.62 to -3.74)
	2	2006-2010	1.90 (1.55 to 2.11)	2009-2019	2.62 (2.55 to 2.74)	2012-2016	14.39 (9.45 to 22.00)	2008-2019	2.63 (2.56 to 2.95)	2012-2016	18.48 (12.66 to 27.79)	2011-2017	9.47 (7.08 to 14.95)
	3	2010-2019	2.45 (2.39 to 2.62)	2019-2021	1.28 (0.85 to 2.01)	2016-2021	-0.36 (-6.72 to 2.91)	2019-2021	1.46 (0.87 to 2.36)	2016-2021	-0.57 (-7.78 to 3.43)	2017-2021	-1.00 (-6.69 to 2.11)
	4	2019-2021	1.15 (0.78 to 1.76)										
Romania	0	1990-2001	0.35 (0.28 to 0.41)	1990-1993	0.70 (0.51 to 1.01)	1990-1996	-2.82 (-3.26 to -2.40)	1990-1993	0.90 (0.67 to 1.26)	1990-1996	-3.04 (-3.64 to -2.39)	1990-1996	-2.26 (-2.51 to -1.99)
	1	2001-2006	1.70 (0.30 to 1.81)	1993-2001	0.41 (0.33 to 0.46)	1996-2001	-8.65 (-9.17 to -8.10)	1993-2000	0.34 (0.16 to 0.41)	1996-2001	-8.49 (-9.55 to -7.72)	1996-2001	-5.80 (-6.27 to -5.37)
	2	2006-2010	2.05 (1.39 to 2.26)	2001-2006	1.68 (1.55 to 1.77)	2001-2004	-1.62 (-2.77 to -0.99)	2000-2005	1.65 (1.30 to 1.79)	2001-2004	-1.62 (-3.22 to -0.81)	2001-2004	-0.44 (-1.62 to 0.18)
	3	2010-2015	-0.03 (-0.16 to 0.14)	2006-2009	2.41 (2.09 to 2.53)	2004-2014	-5.20 (-5.52 to -4.99)	2005-2010	2.31 (2.15 to 2.56)	2004-2013	-5.76 (-6.25 to -5.46)	2004-2013	-2.33 (-3.16 to -2.08)
	4	2015-2021	2.24 (2.10 to 2.36)	2009-2015	1.20 (1.11 to 1.28)	2014-2021	-0.90 (-1.50 to -0.09)	2010-2015	0.88 (0.73 to 1.01)	2013-2021	-1.03 (-1.66 to -0.29)	2013-2021	0.67 (0.15 to 1.29)
	5			2015-2021	2.21 (2.13 to 2.29)			2015-2018	2.61 (2.35 to 2.79)				
	6							2018-2021	1.77 (1.40 to 1.94)				
Russian Federation	0	1990-1993	-0.41 (-0.90 to -0.11)	1990-1993	-0.30 (-0.86 to 0.18)	1990-1994	10.64 (6.80 to 15.28)	1990-1996	0.17 (0.01 to 0.33)	1990-1994	13.31 (10.34 to 18.09)	1990-1994	8.97 (7.74 to 10.79)
	1	1993-1996	0.49 (0.24 to 0.78)	1993-1996	0.54 (0.28 to 5.07)	1994-2000	-1.52 (-2.37 to 0.91)	1996-1999	4.91 (4.61 to 5.20)	1994-1997	-5.55 (-7.92 to -0.42)	1994-1997	-3.40 (-4.89 to -0.68)
	2	1996-1999	4.07 (3.83 to 4.29)	1996-1999	4.82 (0.67 to 5.07)	2000-2005	-4.58 (-5.45 to -3.69)	1999-2006	0.75 (0.55 to 0.90)	1997-2000	2.40 (-6.80 to 4.20)	1997-2000	2.20 (-3.94 to 3.77)
	3	1999-2006	0.65 (0.56 to 0.76)	1999-2006	0.76 (0.64 to 2.31)	2005-2008	-9.58 (-10.55 to -7.87)	2006-2015	2.34 (2.16 to 2.47)	2000-2011	-6.54 (-6.97 to -4.56)	2000-2010	-3.88 (-4.80 to -0.62)
	4	2006-2015	2.05 (1.96 to 2.12)	2006-2015	2.32 (2.19 to 2.51)	2008-2011	-5.43 (-6.44 to 2.42)	2015-2021	3.01 (2.80 to 3.37)	2011-2017	5.13 (4.22 to 6.53)	2010-2021	2.62 (1.82 to 3.56)
	5	2015-2021	2.68 (2.54 to 2.88)	2015-2021	3.04 (2.86 to 3.30)	2011-2017	4.56 (3.86 to 5.61)			2017-2021	-1.64 (-7.79 to 1.31)		
	6					2017-2021	-1.26 (-4.32 to 0.95)						
Rwanda	0	1990-1999	-0.36 (-0.53 to -0.22)	1990-1997	-0.17 (-1.02 to 0.13)	1990-1995	1.64 (1.25 to 2.06)	1990-1998	-0.24 (-0.84 to 0.01)	1990-1995	1.91 (1.28 to 2.44)	1990-1995	1.54 (0.99 to 2.00)
	1	1999-2011	0.31 (0.14 to 0.48)	1997-2012	0.36 (0.27 to 0.63)	1995-1999	-2.87 (-3.42 to -2.02)	1998-2012	0.45 (0.33 to 0.68)	1995-2005	-3.68 (-3.92 to -3.25)	1995-2010	-2.96 (-3.08 to -2.83)
	2	2011-2019	-0.90 (-1.05 to 0.29)	2012-2021	-1.26 (-1.50 to -1.06)	1999-2009	-4.67 (-4.84 to -4.54)	2012-2021	-1.07 (-1.31 to -0.87)	2005-2009	-5.29 (-6.29 to -3.60)	2010-2015	-0.91 (-2.52 to 0.04)
	3	2019-2021	-2.29 (-2.90 to -1.05)			2009-2021	-1.65 (-1.78 to -1.52)			2009-2015	-1.33 (-4.89 to -0.07)	2015-2021	-2.06 (-2.99 to -1.63)
	4									2015-2021	-2.41 (-3.60 to -1.87)		
Saint Kitts and Nevis	0	1990-1999	-0.85 (-1.18 to -0.65)	1990-1998	-1.06 (-1.35 to -0.86)	1990-1992	-6.07 (-7.00 to -5.06)	1990-1998	-0.95 (-1.30 to -0.72)	1990-1998	-4.33 (-4.54 to -4.11)	1990-1998	-3.41 (-3.60 to -3.21)
	1	1999-2011	0.07 (-0.09 to 0.33)	1998-2011	0.00 (-0.11 to 0.17)	1992-1995	-1.39 (-2.15 to -0.75)	1998-2011	0.03 (-0.10 to 0.22)	1998-2002	-9.54 (-9.90 to -9.16)	1998-2001	-7.67 (-8.07 to -6.82)
	2	2011-2021	-2.24 (-2.48 to -2.03)	2011-2021	-2.48 (-2.69 to -2.31)	1995-1998	-4.40 (-6.31 to -3.86)	2011-2021	-2.38 (-2.59 to -2.20)	2002-2015	-3.02 (-3.18 to -2.89)	2001-2021	-2.09 (-2.16 to -2.01)
	3					1998-2002	-7.96 (-8.44 to -7.51)			2015-2021	-1.00 (-1.61 to -0.16)		
	4					2002-2016	-2.70 (-2.83 to -2.59)						
	5					2016-2021	-0.68 (-1.38 to 0.34)						
Saint Lucia	0	1990-1999	-0.95 (-1.35 to -0.73)	1990-1999	-1.10 (-1.39 to -0.90)	1990-1995	-0.71 (-1.26 to -0.18)	1990-2000	-0.98 (-1.29 to -0.78)	1990-1995	-0.75 (-1.44 to -0.23)	1990-1995	-0.88 (-1.27 to -0.53)
	1	1999-2011	0.08 (-0.08 to 0.34)	1999-2012	-0.08 (-0.20 to 0.11)	1995-2001	-6.15 (-6.76 to -5.72)	2000-2011	0.13 (-0.06 to 0.45)	1995-2002	-5.69 (-6.16 to -5.36)	1995-2002	-4.58 (-4.83 to -4.35)
	2	2011-2021	-1.68 (-1.90 to -1.48)	2012-2021	-1.92 (-2.17 to -1.71)	2001-2016	-3.05 (-3.21 to -2.88)	2011-2021	-1.79 (-2.02 to -1.59)	2002-2012	-1.77 (-2.00 to -1.39)	2002-2012	-1.28 (-1.44 to -1.07)

Location	Segment	Incidence		Prevalence		Mortality		YLD		YLL		DALY	
		Period	APC (95%CI)	Period	APC (95%CI)	Period	APC (95%CI)	Period	APC (95%CI)	Period	APC (95%CI)	Period	APC (95%CI)
	3					2016-2021	0.77 (-0.46 to 2.57)			2012-2016	-5.08 (-6.53 to -4.02)	2012-2016	-4.11 (-5.09 to -3.39)
	4									2016-2021	0.92 (-0.22 to 2.92)	2016-2021	0.03 (-0.60 to 0.97)
Saint Vincent and the Grenadines	0	1990-2000	-0.89 (-1.03 to -0.77)	1990-2000	-1.05 (-1.25 to -0.88)	1990-1996	1.09 (0.71 to 1.52)	1990-2000	-1.00 (-1.22 to -0.81)	1990-1996	2.10 (1.68 to 2.56)	1990-1996	1.54 (1.19 to 1.95)
	1	2000-2011	-0.01 (-0.13 to 0.16)	2000-2011	-0.09 (-0.80 to 0.17)	1996-2002	-4.85 (-5.42 to -4.45)	2000-2011	-0.08 (-0.89 to 0.23)	1996-2002	-5.44 (-6.03 to -4.98)	1996-2002	-4.64 (-5.14 to -4.26)
	2	2011-2019	-1.16 (-1.34 to -0.78)	2011-2019	-1.43 (-1.62 to -0.06)	2002-2012	-2.26 (-2.72 to -1.98)	2011-2019	-1.38 (-1.56 to -0.02)	2002-2009	-1.90 (-2.22 to -1.10)	2002-2008	-1.21 (-1.60 to -0.16)
	3	2019-2021	-3.06 (-3.84 to -1.78)	2019-2021	-3.15 (-4.09 to -1.65)	2012-2021	-1.21 (-1.56 to -0.58)	2019-2021	-2.96 (-3.79 to -1.57)	2009-2012	-4.69 (-5.47 to -3.49)	2008-2011	-3.93 (-4.70 to -2.68)
	4									2012-2016	1.10 (0.22 to 2.44)	2011-2016	-0.10 (-0.67 to 1.32)
	5									2016-2021	-2.55 (-3.90 to -1.75)	2016-2021	-2.19 (-3.35 to -1.59)
Samoa	0	1990-1999	-0.08 (-0.10 to -0.05)	1990-1998	-0.05 (-0.13 to -0.00)	1990-2000	0.24 (0.14 to 0.37)	1990-1996	-0.13 (-0.45 to 0.00)	1990-1997	-0.22 (-0.63 to -0.03)	1990-1997	-0.18 (-0.39 to -0.07)
	1	1999-2010	0.14 (0.13 to 0.16)	1998-2010	0.17 (0.14 to 0.22)	2000-2007	-0.66 (-0.91 to -0.50)	1996-2010	0.16 (0.12 to 0.25)	1997-2000	0.75 (-0.09 to 1.04)	1997-2000	0.56 (0.10 to 0.75)
	2	2010-2016	-0.13 (-0.20 to -0.10)	2010-2021	-0.19 (-0.22 to -0.15)	2007-2015	1.08 (0.95 to 1.22)	2010-2021	-0.19 (-0.25 to -0.14)	2000-2003	-1.65 (-1.97 to -0.52)	2000-2003	-1.12 (-1.33 to -0.68)
	3	2016-2019	0.15 (0.06 to 0.22)			2015-2021	-1.22 (-1.38 to -1.06)			2003-2007	-0.72 (-1.09 to 1.01)	2003-2007	-0.42 (-0.76 to 0.27)
	4	2019-2021	-0.28 (-0.39 to -0.13)							2007-2015	0.94 (-0.72 to 1.15)	2007-2014	0.66 (0.56 to 0.87)
	5									2015-2021	-1.36 (-1.55 to -1.15)	2014-2017	-0.37 (-0.70 to 0.28)
	6											2017-2021	-1.20 (-1.53 to -1.03)
San Marino	0	1990-1995	1.42 (1.22 to 1.53)	1990-1995	1.40 (1.15 to 1.54)	1990-2012	-2.46 (-2.63 to -2.32)	1990-1995	1.41 (1.16 to 1.54)	1990-2013	-1.95 (-2.11 to -1.84)	1990-1996	0.44 (0.06 to 0.64)
	1	1995-2000	2.52 (2.34 to 2.75)	1995-2004	2.25 (2.17 to 2.34)	2012-2019	0.10 (-0.67 to 2.10)	1995-2001	2.40 (2.26 to 2.67)	2013-2019	-0.10 (-0.99 to 1.89)	1996-2002	1.45 (1.26 to 2.06)
	2	2000-2005	2.17 (1.94 to 2.34)	2004-2015	1.54 (1.49 to 1.60)	2019-2021	-18.25 (-22.41 to -12.20)	2001-2005	1.97 (1.67 to 2.23)	2019-2021	-17.85 (-21.73 to -12.28)	2002-2018	0.96 (0.90 to 1.00)
	3	2005-2014	1.63 (1.55 to 1.73)	2015-2019	0.86 (0.55 to 1.06)			2005-2015	1.56 (1.48 to 1.61)			2018-2021	-0.53 (-1.37 to -0.01)
	4	2014-2019	0.74 (0.54 to 1.61)	2019-2021	1.91 (1.35 to 2.27)			2015-2019	0.79 (0.53 to 0.97)				
	5	2019-2021	1.34 (0.87 to 1.58)					2019-2021	1.75 (1.31 to 2.06)				
Sao Tome and Principe	0	1990-1994	-0.25 (-0.28 to -0.21)	1990-1994	-0.40 (-0.53 to -0.33)	1990-2000	1.93 (1.53 to 2.51)	1990-1992	-0.47 (-0.62 to -0.22)	1990-1999	1.73 (1.03 to 3.32)	1990-2001	1.05 (0.62 to 1.64)
	1	1994-2005	0.05 (0.04 to 0.06)	1994-2006	0.06 (0.05 to 0.08)	2000-2008	-0.73 (-1.40 to 0.35)	1992-1996	-0.17 (-0.24 to 0.32)	1999-2008	-0.94 (-1.80 to 0.36)	2001-2021	-1.26 (-1.48 to -1.08)
	2	2005-2012	0.38 (0.35 to 0.40)	2006-2015	0.46 (0.35 to 0.48)	2008-2021	-2.06 (-2.78 to -1.77)	1996-2000	0.26 (-0.14 to 0.38)	2008-2021	-2.54 (-4.55 to -2.11)		
	3	2012-2019	0.46 (0.44 to 0.50)	2015-2018	0.65 (0.51 to 0.71)			2000-2005	-0.06 (-0.15 to 0.54)				
	4	2019-2021	-0.16 (-0.24 to -0.08)	2018-2021	0.02 (-0.10 to 0.11)			2005-2019	0.48 (0.46 to 0.52)				
	5							2019-2021	-0.24 (-0.42 to 0.03)				
Saudi Arabia	0	1990-1995	0.60 (0.48 to 0.75)	1990-1995	0.66 (0.53 to 0.84)	1990-1999	-1.88 (-2.04 to -1.57)	1990-1994	-0.52 (-1.05 to -0.27)	1990-2003	-2.49 (-2.62 to -2.35)	1990-1993	-1.71 (-2.21 to -1.30)
	1	1995-2000	-0.02 (-0.27 to 0.10)	1995-2000	0.01 (-0.31 to 0.14)	1999-2002	-3.75 (-4.25 to -2.88)	1994-2001	0.18 (0.06 to 0.41)	2003-2006	3.37 (2.18 to 3.87)	1993-2002	-1.20 (-1.27 to 2.11)
	2	2000-2010	1.45 (1.37 to 1.51)	2000-2010	1.49 (1.41 to 1.56)	2002-2007	1.81 (1.41 to 2.17)	2001-2004	3.04 (2.60 to 3.25)	2006-2021	-2.09 (-2.26 to -1.97)	2002-2007	2.09 (-0.58 to 2.23)
	3	2010-2015	2.18 (2.00 to 2.57)	2010-2014	2.42 (2.13 to 2.78)	2007-2021	-2.08 (-2.23 to -1.96)	2004-2015	1.44 (1.36 to 1.50)			2007-2012	-0.68 (-0.97 to -0.03)
	4	2015-2021	1.62 (1.36 to 1.74)	2014-2021	1.48 (1.30 to 1.60)			2015-2018	3.45 (2.97 to 3.70)			2012-2021	0.66 (0.51 to 0.83)
	5							2018-2021	1.70 (1.11 to 2.03)				
Senegal	0	1990-2006	-0.01 (-0.03 to 0.01)	1990-2000	0.06 (0.04 to 0.09)	1990-2000	-3.24 (-4.41 to -1.58)	1990-1993	0.43 (0.19 to 0.80)	1990-2000	-3.07 (-4.34 to -1.21)	1990-2000	-2.19 (-3.02 to -0.92)
	1	2006-2010	0.39 (-0.02 to 0.56)	2000-2005	-0.11 (-0.27 to -0.05)	2000-2003	3.21 (-4.78 to 4.72)	1993-2006	-0.02 (-0.08 to 0.01)	2000-2003	3.40 (-4.59 to 5.00)	2000-2003	2.35 (-3.27 to 3.44)
	2	2010-2015	-0.33 (-0.49 to 0.38)	2005-2010	0.37 (0.31 to 0.48)	2003-2016	0.16 (-0.76 to 1.86)	2006-2010	0.48 (0.29 to 0.70)	2003-2016	-0.05 (-1.31 to 1.21)	2003-2016	-0.02 (-0.85 to 0.65)
	3	2015-2019	0.06 (-0.29 to 0.21)	2010-2015	-0.34 (-0.45 to -0.28)	2016-2021	-2.30 (-6.27 to -0.36)	2010-2015	-0.35 (-0.59 to -0.22)	2016-2021	-3.00 (-7.28 to -0.84)	2016-2021	-2.07 (-4.87 to -0.71)
	4	2019-2021	0.70 (0.36 to 0.90)	2015-2019	0.06 (-0.14 to 0.20)			2015-2021	0.20 (0.11 to 0.35)				
	5			2019-2021	0.73 (0.47 to 0.88)								
Serbia	0	1990-2001	-0.09 (-0.15 to -0.04)	1990-2001	0.01 (-0.07 to 0.07)	1990-1997	0.93 (0.07 to 1.69)	1990-2001	0.02 (-0.09 to 0.11)	1990-1997	0.74 (-0.15 to 1.66)	1990-1996	0.84 (0.15 to 1.66)
	1	2001-2005	1.04 (0.60 to 1.45)	2001-2005	1.08 (0.60 to 1.55)	1997-2014	-4.35 (-4.64 to -4.14)	2001-2005	1.09 (0.48 to 1.72)	1997-2013	-4.28 (-4.75 to -4.03)	1996-2011	-2.76 (-2.96 to -2.59)
	2	2005-2013	2.11 (2.01 to 2.30)	2005-2013	2.24 (2.13 to 2.45)	2014-2021	-2.03 (-2.89 to 0.18)	2005-2013	2.36 (2.23 to 2.73)	2013-2021	-2.50 (-3.32 to -0.18)	2011-2021	-1.00 (-1.34 to -0.58)
	3	2013-2019	1.36 (1.15 to 1.57)	2013-2019	1.45 (1.24 to 1.71)			2013-2019	1.57 (1.32 to 1.89)				
	4	2019-2021	-0.48 (-1.02 to 0.25)	2019-2021	-0.16 (-0.73 to 0.60)			2019-2021	-0.31 (-0.93 to 0.53)				
Seychelles	0	1990-1995	-0.05 (-0.09 to 0.01)	1990-1995	-0.13 (-0.17 to -0.07)	1990-1995	-4.92 (-5.08 to -4.75)	1990-1995	-0.01 (-0.08 to 0.12)	1990-1995	-5.15 (-5.90 to -4.55)	1990-1998	-2.80 (-3.39 to -2.36)
	1	1995-2000	-0.19 (-0.27 to -0.15)	1995-2000	-0.35 (-0.41 to -0.31)	1995-2001	-1.75 (-1.87 to -1.63)	1995-1998	-0.42 (-0.52 to -0.24)	1995-2001	-2.01 (-2.48 to -1.45)	1998-2021	0.33 (0.22 to 0.44)
	2	2000-2006	0.21 (0.11 to 0.24)	2000-2005	0.18 (0.11 to 0.22)	2001-2007	1.31 (1.17 to 1.44)	1998-2005	0.14 (0.05 to 0.20)	2001-2007	1.56 (1.07 to 2.86)		
	3	2006-2010	0.33 (0.28 to 0.40)	2005-2010	0.41 (0.37 to 0.47)	2007-2010	-1.55 (-1.85 to -1.14)	2005-2008	0.62 (0.43 to 0.73)	2007-2010	-1.40 (-2.12 to -0.01)		
	4	2010-2015	-0.70 (-0.74 to -0.67)	2010-2015	-0.55 (-0.60 to -0.51)	2010-2014	1.59 (1.30 to 1.99)	2008-2011	0.05 (-0.31 to 0.20)	2010-2019	1.24 (1.02 to 2.63)		
	5	2015-2019	-0.20 (-0.29 to -0.15)	2015-2019	-0.18 (-0.24 to -0.10)	2014-2019	0.68 (0.25 to 0.90)	2011-2015	-0.75 (-0.92 to -0.63)	2019-2021	-3.56 (-5.19 to -0.50)		
	6	2019-2021	0.18 (0.02 to 0.29)	2019-2021	0.70 (0.57 to 0.80)	2019-2021	-4.84 (-5.54 to -4.16)	2015-2021	0.08 (0.01 to 0.16)				
Sierra Leone	0	1990-2000	0.08 (0.04 to 0.16)	1990-2000	0.05 (0.00 to 0.17)	1990-2000	-1.70 (-1.84 to -1.56)	1990-1998	0.08 (0.00 to 0.28)	1990-2000	-1.57 (-1.81 to -1.35)	1990-2000	-1.08 (-1.25 to -0.92)
	1	2000-2005	-0.19 (-0.45 to -0.05)	2000-2005	-0.21 (-0.51 to -0.05)	2000-2006	3.07 (2.77 to 3.34)	1998-2007	-0.11 (-0.42 to -0.04)	2000-2006	2.87 (2.40 to 3.59)	2000-2006	1.94 (1.62 to 2.49)
	2	2005-2015	0.17 (0.08 to 0.29)	2005-2015	0.22 (0.08 to 0.34)	2006-2009	-1.37 (-1.91 to -0.12)	2007-2016	0.30 (0.11 to 0.40)	2006-2009	-2.28 (-3.05 to -0.77)	2006-2009	-1.54 (-2.09 to -0.50)
	3	2015-2021	0.64 (0.50 to 0.96)	2015-2021	0.63 (0.48 to 1.03)	2009-2014	0.42 (-0.09 to 1.40)	2016-2021	0.68 (0.50 to 1.09)	2009-2014	0.68 (0.07 to 2.07)	2009-2014	0.55 (0.14 to 1.51)
	4					2014-2021	-1.54 (-1.88 to -1.25)			2014-2021	-1.62 (-2.11 to -1.25)	2014-2021	-0.93 (-1.30 to -0.68)
Singapore	0	1990-1993	1.26 (1.16 to 1.40)	1990-1994	1.27 (1.11 to 1.42)	1990-1993	-4.70 (-5.87 to -2.44)	1990-1994	1.30 (1.13 to 1.47)	1990-1993	-5.38 (-7.39 to -2.60)	1990-1993	-1.70 (-1.95 to -1.25)
	1	1993-1996	0.71 (0.56 to 0.87)	1994-2000	0.44 (0.27 to 0.53)	1993-1996	-10.22 (-11.25 to -8.62)	1994-1999	0.41 (0.19 to 0.54)	1993-1996	-10.35 (-11.57 to -7.83)	1993-1996	-3.65 (-3.98 to -3.18)
	2	1996-2001	0.33 (0.22 to 0.38)	2000-2009	0.70 (0.66 to 0.78)	1996-2000	-3.26 (-4.38 to -1.70)	1999-2009	0.69 (0.66 to 0.77)	1996-2000	-3.01 (-5.68 to -1.13)	1996-2000	-0.64 (-0.87 to -0.06)
	3	2001-2005	0.89 (0.80 to 1.01)	2009-2019	-0.14 (-0.18 to -0.10)	2000-2018	-7.96 (-8.12 to -7.83)	2009-2019	-0.11 (-0.16 to -0.07)	2000-2019	-7.74 (-7.92 to -7.60)	2000-2003	-1.85 (-2.10 to -1.36)
	4	2005-2010	0.52 (0.43 to 0.57)	2019-2021	1.28 (0.98 to 1.49)	2018-2021	-2.82 (-4.88 to 0.74)	2019-2021	1.24 (0.90 to 1.46)	2019-2021	0.26 (-3.32 to 2.31)	2003-2018	-1.13 (-1.20 to -0.96)
	5	2010-2019	-0.09 (-0.12 to -0.07)									2018-2021	0.59 (-0.15 to 1.54)

Location	Segment	Incidence		Prevalence		Mortality		YLD		YLL		DALY	
		Period	APC (95%CI)	Period	APC (95%CI)	Period	APC (95%CI)	Period	APC (95%CI)	Period	APC (95%CI)	Period	APC (95%CI)
	6	2019-2021	1.34 (1.16 to 1.47)										
Slovakia	0	1990-2003	0.51 (0.47 to 0.53)	1990-1994	0.74 (0.61 to 0.99)	1990-2012	-3.06 (-3.22 to -2.93)	1990-1994	0.89 (0.77 to 1.07)	1990-1994	-4.39 (-6.97 to -3.05)	1990-1994	-3.17 (-5.17 to -1.89)
	1	2003-2006	1.46 (1.23 to 1.60)	1994-2001	0.46 (0.27 to 0.52)	2012-2021	-1.14 (-1.75 to -0.19)	1994-1999	0.43 (0.20 to 0.53)	1994-2013	-2.69 (-2.88 to -1.65)	1994-2004	-1.66 (-2.31 to -0.06)
	2	2006-2010	3.20 (3.07 to 3.34)	2001-2005	0.90 (0.61 to 1.06)			1999-2005	0.83 (0.76 to 0.95)	2013-2018	0.19 (-3.35 to 2.35)	2004-2012	-0.68 (-2.47 to 1.37)
	3	2010-2015	1.32 (1.10 to 1.44)	2005-2010	2.92 (0.96 to 3.00)			2005-2010	3.09 (3.02 to 3.16)	2018-2021	-3.83 (-7.53 to -0.96)	2012-2021	0.33 (-2.08 to 2.03)
	4	2015-2021	1.81 (1.69 to 2.06)	2010-2015	1.09 (0.98 to 2.89)			2010-2016	1.44 (1.35 to 1.50)				
	5			2015-2019	2.27 (1.14 to 2.53)			2016-2019	2.45 (2.25 to 2.59)				
	6			2019-2021	1.62 (1.25 to 2.08)			2019-2021	1.44 (1.14 to 1.72)				
Slovenia	0	1990-2001	0.21 (0.10 to 0.35)	1990-2001	0.22 (0.14 to 0.31)	1990-1992	6.22 (-0.03 to 10.99)	1990-2001	0.28 (0.15 to 0.44)	1990-1992	3.85 (-2.04 to 8.31)	1990-1992	3.79 (1.20 to 5.75)
	1	2001-2015	1.98 (0.28 to 2.11)	2001-2014	2.10 (2.02 to 2.19)	1992-2021	-5.99 (-6.16 to -5.85)	2001-2015	2.09 (0.29 to 2.23)	1992-2021	-5.51 (-5.70 to -5.37)	1992-1997	-4.67 (-5.80 to -4.07)
	2	2015-2019	0.71 (0.29 to 2.00)	2014-2019	0.78 (0.37 to 1.89)			2015-2019	0.67 (0.34 to 2.15)			1997-2006	-2.81 (-3.48 to -2.11)
	3	2019-2021	-1.71 (-2.62 to -0.15)	2019-2021	-1.43 (-2.14 to -0.09)			2019-2021	-1.23 (-2.02 to 0.14)			2006-2021	-1.52 (-1.83 to -0.80)
Solomon Islands	0	1990-1999	0.55 (0.48 to 0.62)	1990-1999	0.56 (0.48 to 0.66)	1990-2010	0.59 (0.34 to 0.70)	1990-1999	0.55 (0.47 to 0.66)	1990-2010	0.69 (0.35 to 0.81)	1990-2005	0.70 (0.55 to 1.20)
	1	1999-2011	0.03 (-0.02 to 0.07)	1999-2011	-0.00 (-0.08 to 0.05)	2010-2014	1.84 (0.98 to 2.71)	1999-2011	0.02 (-0.07 to 0.09)	2010-2014	1.88 (0.97 to 2.79)	2005-2008	-0.55 (-1.21 to 1.27)
	2	2011-2021	0.62 (0.56 to 0.69)	2011-2021	0.69 (0.61 to 0.78)	2014-2021	-0.20 (-0.72 to 0.11)	2011-2021	0.66 (0.58 to 0.76)	2014-2021	-0.18 (-0.79 to 0.16)	2008-2014	1.45 (0.14 to 2.63)
	3											2014-2021	0.06 (-0.34 to 0.41)
Somalia	0	1990-2000	0.32 (0.28 to 0.37)	1990-2000	0.75 (0.67 to 0.83)	1990-1995	1.02 (0.71 to 1.45)	1990-2001	0.63 (0.58 to 0.70)	1990-1993	2.06 (1.41 to 2.62)	1990-1994	1.28 (0.82 to 2.00)
	1	2000-2012	-0.01 (-0.05 to 0.03)	2000-2011	0.02 (-0.05 to 0.09)	1995-2006	-0.07 (-0.21 to 0.03)	2001-2012	-0.04 (-0.11 to 0.03)	1993-2006	-0.14 (-0.23 to -0.06)	1994-2006	-0.02 (-0.16 to 0.08)
	2	2012-2021	-0.60 (-0.66 to -0.55)	2011-2021	-1.00 (-1.08 to -0.92)	2006-2010	1.76 (1.39 to 2.26)	2012-2021	-0.92 (-1.01 to -0.84)	2006-2009	2.71 (0.14 to 3.02)	2006-2011	1.59 (1.27 to 2.00)
	3					2010-2021	-0.96 (-1.04 to -0.88)			2009-2012	0.70 (0.06 to 2.02)	2011-2021	-1.33 (-1.43 to -1.24)
	4									2012-2016	-1.90 (-2.30 to -1.51)		
	5									2016-2021	-1.16 (-1.44 to -0.56)		
South Africa	0	1990-1993	-0.66 (-0.81 to -0.57)	1990-1995	-0.64 (-0.77 to -0.58)	1990-1992	0.22 (-3.32 to 2.82)	1990-1997	-0.58 (-0.66 to -0.56)	1990-1992	0.10 (-3.71 to 3.00)	1990-1992	-0.23 (-1.65 to 1.11)
	1	1993-2000	-0.42 (-0.44 to -0.38)	1995-2000	-0.42 (-0.48 to -0.25)	1992-1996	-5.03 (-6.80 to 1.89)	1997-2000	-0.42 (-0.49 to 0.03)	1992-1996	-4.92 (-6.98 to 2.85)	1992-1996	-3.26 (-4.24 to -2.73)
	2	2000-2011	0.12 (0.11 to 0.13)	2000-2010	0.10 (0.07 to 0.13)	1996-1999	2.83 (-0.78 to 7.13)	2000-2010	0.11 (0.09 to 0.13)	1996-1999	2.60 (-1.01 to 7.57)	1996-1999	1.22 (-1.00 to 3.59)
	3	2011-2014	0.90 (0.82 to 0.94)	2010-2015	0.69 (0.63 to 0.78)	1999-2004	6.98 (-4.08 to 8.45)	2010-2015	0.73 (0.69 to 0.77)	1999-2004	6.85 (-4.76 to 8.50)	1999-2004	4.75 (4.13 to 5.50)
	4	2014-2019	0.59 (0.53 to 0.62)	2015-2019	0.48 (0.39 to 0.57)	2004-2013	-3.92 (-4.63 to -1.88)	2015-2019	0.45 (0.38 to 0.50)	2004-2013	-4.20 (-5.20 to -1.26)	2004-2007	-3.74 (-4.33 to -2.47)
	5	2019-2021	-0.40 (-0.51 to -0.29)	2019-2021	-0.59 (-0.75 to -0.42)	2013-2021	-1.42 (-1.98 to -0.73)	2019-2021	-0.56 (-0.68 to -0.45)	2013-2021	-1.66 (-2.42 to -0.66)	2007-2014	-2.07 (-2.46 to -1.26)
	6											2014-2021	-0.75 (-1.09 to -0.02)
South Sudan	0	1990-1994	0.01 (-0.18 to 0.13)	1990-1995	0.40 (0.11 to 0.57)	1990-1995	0.05 (-0.79 to 1.44)	1990-1996	0.37 (0.10 to 0.48)	1990-1995	0.03 (-0.70 to 0.98)	1990-1995	0.13 (-0.42 to 0.87)
	1	1994-2000	0.45 (0.38 to 0.61)	1995-2000	0.73 (0.00 to 1.04)	1995-2001	-2.97 (-4.60 to -2.24)	1996-1999	0.95 (0.61 to 1.09)	1995-2002	-3.18 (-3.90 to -2.69)	1995-2002	-2.14 (-2.69 to -1.79)
	2	2000-2011	0.02 (-0.01 to 0.06)	2000-2011	0.03 (-0.72 to 0.08)	2001-2021	1.03 (0.90 to 1.18)	1999-2011	0.08 (0.01 to 0.12)	2002-2007	2.74 (1.91 to 4.68)	2002-2007	1.94 (1.38 to 3.22)
	3	2011-2021	-0.49 (-0.54 to -0.46)	2011-2021	-0.83 (-0.92 to -0.77)			2011-2021	-0.74 (-0.81 to -0.68)	2007-2011	-0.64 (-2.06 to 0.59)	2007-2011	-0.48 (-1.45 to 0.36)
	4									2011-2018	2.27 (1.80 to 4.07)	2011-2018	1.45 (1.13 to 2.70)
	5									2018-2021	-0.46 (-2.88 to 1.05)	2018-2021	-0.52 (-2.35 to 0.55)
Spain	0	1990-1995	2.91 (2.47 to 3.17)	1990-1995	2.93 (2.57 to 3.14)	1990-2004	-4.80 (-4.92 to -4.55)	1990-1995	3.29 (2.21 to 3.70)	1990-2003	-4.76 (-4.98 to -4.27)	1990-1995	0.69 (-0.05 to 1.01)
	1	1995-2000	4.19 (3.91 to 4.77)	1995-1999	4.46 (4.14 to 4.88)	2004-2014	-5.26 (-6.28 to -5.03)	1995-1999	4.51 (3.91 to 5.17)	2003-2014	-5.53 (-7.27 to -5.22)	1995-2000	2.15 (1.75 to 2.91)
	2	2000-2005	2.87 (2.33 to 3.12)	1999-2005	3.02 (2.60 to 3.19)	2014-2021	-2.46 (-2.92 to -1.86)	1999-2005	2.70 (1.94 to 3.04)	2014-2021	-2.56 (-3.26 to -1.64)	2000-2005	1.29 (0.50 to 1.76)
	3	2005-2009	4.28 (3.93 to 4.73)	2005-2009	4.13 (3.82 to 4.56)			2005-2010	3.86 (3.35 to 4.65)			2005-2010	2.81 (2.22 to 3.75)
	4	2009-2015	1.70 (1.51 to 1.86)	2009-2013	2.22 (1.96 to 2.58)			2010-2015	1.42 (0.51 to 1.81)			2010-2015	0.89 (-0.36 to 1.28)
	5	2015-2021	-2.80 (-3.04 to -2.59)	2013-2016	0.25 (-0.26 to 0.76)			2015-2021	-2.73 (-3.06 to -2.47)			2015-2021	-2.69 (-3.06 to -2.26)
	6			2016-2021	-2.90 (-3.23 to -2.66)								
Sri Lanka	0	1990-1994	0.23 (0.15 to 0.39)	1990-2000	-0.10 (-0.14 to -0.07)	1990-2011	-0.37 (-0.49 to -0.20)	1990-1997	0.16 (0.01 to 0.28)	1990-2010	-0.58 (-0.73 to -0.37)	1990-2010	-0.20 (-0.33 to -0.02)
	1	1994-2000	-0.06 (-0.18 to -0.01)	2000-2005	0.35 (0.21 to 0.47)	2011-2021	-2.27 (-2.91 to -1.86)	1997-2010	0.96 (0.91 to 1.01)	2010-2021	-2.13 (-2.85 to -1.72)	2010-2021	-1.38 (-1.94 to -1.04)
	2	2000-2006	0.35 (0.28 to 0.39)	2005-2010	0.89 (0.80 to 1.02)			2010-2015	-0.51 (-0.73 to -0.22)				
	3	2006-2009	0.70 (0.59 to 0.77)	2010-2015	0.11 (-0.02 to 0.20)			2015-2019	0.88 (0.62 to 1.27)				
	4	2009-2015	0.04 (-0.02 to 0.08)	2015-2019	0.74 (0.61 to 0.93)			2019-2021	-0.30 (-0.76 to 0.26)				
	5	2015-2019	0.64 (0.55 to 0.75)	2019-2021	0.11 (-0.12 to 0.40)								
	6	2019-2021	-0.37 (-0.53 to -0.15)										
Sudan	0	1990-1997	-0.19 (-0.20 to -0.18)	1990-2000	-0.21 (-0.21 to -0.20)	1990-1996	-0.87 (-1.16 to -0.36)	1990-1993	-1.02 (-1.25 to -0.87)	1990-2002	-1.28 (-1.53 to -1.07)	1990-2002	-0.98 (-1.12 to -0.87)
	1	1997-2000	-0.06 (-0.10 to -0.03)	2000-2010	0.24 (0.22 to 0.25)	1996-2001	-1.91 (-2.64 to -1.51)	1993-1996	-0.05 (-0.29 to 0.15)	2002-2007	2.15 (1.38 to 3.36)	2002-2007	1.82 (1.38 to 2.37)
	2	2000-2008	0.21 (0.20 to 0.22)	2010-2013	0.53 (0.25 to 0.56)	2001-2007	1.66 (1.30 to 2.05)	1996-2000	0.79 (0.67 to 0.98)	2007-2015	-1.45 (-1.73 to -0.88)	2007-2018	-1.15 (-1.24 to -0.97)
	3	2008-2011	0.38 (0.34 to 0.40)	2013-2016	0.73 (0.67 to 0.77)	2007-2015	-1.26 (-1.45 to -0.97)	2000-2011	0.23 (0.19 to 0.26)	2015-2021	-2.69 (-3.52 to -2.25)	2018-2021	-2.28 (-3.23 to -1.64)
	4	2011-2016	0.69 (0.67 to 0.71)	2016-2019	1.14 (1.08 to 1.18)	2015-2021	-2.18 (-2.61 to -1.91)	2011-2017	0.83 (0.69 to 0.92)				
	5	2016-2019	0.92 (0.88 to 0.95)	2019-2021	1.62 (1.54 to 1.69)			2017-2021	1.27 (1.14 to 1.50)				
	6	2019-2021	1.34 (1.29 to 1.40)										
Suriname	0	1990-2000	-0.73 (-0.78 to -0.69)	1990-1999	-0.97 (-1.06 to -0.89)	1990-1993	3.28 (1.51 to 4.97)	1990-1999	-0.91 (-1.05 to -0.81)	1990-1993	4.01 (1.84 to 6.14)	1990-1993	2.83 (1.25 to 4.20)
	1	2000-2005	0.33 (0.16 to 0.57)	1999-2011	0.04 (-0.02 to 0.11)	1993-1997	-4.25 (-5.62 to -3.17)	1999-2010	0.10 (0.02 to 0.22)	1993-2001	-3.81 (-4.47 to -3.34)	1993-2001	-3.08 (-3.54 to -2.73)
	2	2005-2012	-0.08 (-0.23 to 0.07)	2011-2021	-0.48 (-0.56 to -0.41)	1997-2013	-1.35 (-1.53 to -1.17)	2010-2021	-0.44 (-0.54 to -0.37)	2001-2008	0.08 (-0.45 to 1.24)	2001-2008	0.14 (-0.28 to 1.07)
	3	2012-2021	-0.40 (-0.53 to -0.35)			2013-2019	1.90 (1.34 to 3.13)			2008-2012	-2.98 (-4.45 to -1.79)	2008-2012	-2.25 (-3.36 to -1.29)
	4					2019-2021	-4.49 (-6.72 to -1.74)			2012-2019	2.17 (1.70 to 3.41)	2012-2019	1.46 (1.10 to 2.40)
	5									2019-2021	-3.79 (-6.35 to -0.42)	2019-2021	-2.97 (-4.80 to -0.49)

Location	Segment	Incidence		Prevalence		Mortality		YLD		YLL		DALY	
		Period	APC (95%CI)	Period	APC (95%CI)	Period	APC (95%CI)	Period	APC (95%CI)	Period	APC (95%CI)	Period	APC (95%CI)
Sweden	0	1990-1996	0.18 (0.07 to 0.24)	1990-1995	0.25 (0.10 to 0.32)	1990-1994	-4.72 (-7.98 to -3.02)	1990-1996	0.25 (0.15 to 0.31)	1990-1994	-5.40 (-9.54 to -3.19)	1990-1994	-2.00 (-3.46 to -1.35)
	1	1996-2001	0.38 (0.16 to 0.49)	1995-2002	0.40 (0.35 to 0.56)	1994-2002	-1.32 (-1.94 to 1.34)	1996-2005	0.53 (0.48 to 0.58)	1994-2002	-1.28 (-2.17 to 1.98)	1994-2021	-0.31 (-0.36 to -0.26)
	2	2001-2006	0.60 (0.39 to 0.72)	2002-2011	0.71 (0.65 to 0.75)	2002-2021	-2.75 (-3.02 to -2.56)	2005-2015	0.81 (0.78 to 0.85)	2002-2021	-2.76 (-3.25 to -2.52)		
	3	2006-2011	0.77 (0.63 to 0.99)	2011-2014	0.96 (0.80 to 1.02)			2015-2021	0.36 (0.29 to 0.42)				
	4	2011-2014	0.99 (0.30 to 1.05)	2014-2021	0.42 (0.38 to 0.46)								
	5	2014-2021	0.36 (0.28 to 0.44)										
Switzerland	0	1990-1995	1.41 (1.02 to 1.60)	1990-1994	1.23 (0.59 to 1.53)	1990-1994	-3.32 (-4.10 to -2.28)	1990-1995	1.38 (0.96 to 1.57)	1990-1994	-2.60 (-3.49 to -1.03)	1990-1994	-0.58 (-0.80 to -0.28)
	1	1995-2003	2.21 (2.05 to 2.39)	1994-2003	2.13 (2.02 to 2.32)	1994-1998	-7.32 (-8.58 to -6.39)	1995-2003	2.21 (1.99 to 2.40)	1994-1997	-9.10 (-10.14 to -6.98)	1994-1997	-2.60 (-2.88 to -2.12)
	2	2003-2009	1.25 (1.09 to 2.19)	2003-2009	1.23 (1.05 to 2.10)	1998-2005	-3.41 (-3.87 to -2.40)	2003-2009	1.20 (1.05 to 2.22)	1997-2005	-3.67 (-4.16 to -2.17)	1997-2005	0.25 (0.10 to 0.43)
	3	2009-2015	0.28 (-0.07 to 1.23)	2009-2015	0.36 (-0.02 to 1.22)	2005-2015	-5.31 (-6.20 to -5.01)	2009-2015	0.33 (-0.01 to 1.20)	2005-2015	-5.95 (-7.24 to -5.54)	2005-2015	-0.78 (-0.97 to -0.65)
	4	2015-2019	1.01 (0.34 to 1.30)	2015-2019	1.13 (0.41 to 1.43)	2015-2021	-3.30 (-4.07 to -1.73)	2015-2019	1.14 (0.36 to 1.44)	2015-2021	-3.38 (-4.36 to -1.08)	2015-2021	0.74 (0.44 to 1.15)
	5	2019-2021	2.33 (1.65 to 2.72)	2019-2021	2.46 (1.70 to 2.90)			2019-2021	2.32 (1.57 to 2.73)				
	0	1990-2000	0.17 (0.08 to 0.24)	1990-2000	0.12 (0.06 to 0.17)	1990-2000	-2.23 (-2.40 to -2.05)	1990-2001	0.14 (0.09 to 0.19)	1990-2000	-2.72 (-2.89 to -2.53)	1990-2000	-1.43 (-1.56 to -1.22)
	1	2000-2009	0.53 (0.36 to 0.74)	2000-2010	0.60 (0.55 to 0.66)	2000-2003	-6.91 (-7.33 to -6.27)	2001-2009	0.67 (0.57 to 0.78)	2000-2003	-7.44 (-7.86 to -6.89)	2000-2003	-3.41 (-3.81 to -2.52)
	2	2009-2019	-0.06 (-0.13 to 0.50)	2010-2015	-0.40 (-0.65 to -0.29)	2003-2012	-1.38 (-1.62 to -1.20)	2009-2016	-0.22 (-0.43 to -0.12)	2003-2012	-1.72 (-1.94 to -1.54)	2003-2021	-0.34 (-0.42 to -0.25)
Syrian Arab	3	2019-2021	-0.74 (-1.06 to -0.12)	2015-2019	0.23 (0.07 to 0.52)	2012-2016	0.72 (-0.06 to 1.70)	2016-2019	0.31 (0.04 to 0.52)	2012-2016	1.17 (0.29 to 2.32)		
Republic	4			2019-2021	-0.80 (-1.10 to -0.37)	2016-2021	-1.72 (-3.11 to -1.12)	2019-2021	-0.81 (-1.17 to -0.37)	2016-2021	-1.94 (-3.03 to -1.32)		
Taiwan (Province of	0	1990-2001	-0.02 (-0.16 to 0.12)	1990-1995	0.25 (-0.13 to 1.26)	1990-1995	3.02 (2.06 to 4.28)	1990-1994	1.47 (0.93 to 2.33)	1990-1995	2.98 (1.85 to 4.61)	1990-1995	2.81 (2.05 to 3.74)
China)	1	2001-2011	0.99 (0.85 to 1.23)	1995-2000	-1.05 (-1.69 to -0.65)	1995-2001	-1.58 (-2.47 to -0.51)	1994-2000	-0.55 (-1.16 to -0.25)	1995-2000	-1.21 (-3.04 to 0.35)	1995-2001	-1.42 (-1.98 to -0.28)
	2	2011-2019	-0.14 (-0.33 to 0.19)	2000-2011	0.96 (0.85 to 1.13)	2001-2006	-4.53 (-7.26 to -3.51)	2000-2014	0.79 (0.70 to 0.89)	2000-2006	-4.13 (-8.73 to -3.37)	2001-2006	-3.53 (-5.39 to -2.63)
	3	2019-2021	-2.14 (-2.86 to -0.98)	2011-2019	0.07 (-0.12 to 0.50)	2006-2010	-9.36 (-11.39 to -4.58)	2014-2021	-0.89 (-1.19 to -0.64)	2006-2010	-9.48 (-11.51 to -2.95)	2006-2010	-6.93 (-8.60 to -2.89)
	4			2019-2021	-1.50 (-2.05 to -0.47)	2010-2021	-2.70 (-3.11 to -2.23)			2010-2021	-2.19 (-2.65 to -1.69)	2010-2021	-1.59 (-2.01 to -1.10)
Tajikistan	0	1990-1993	0.78 (0.70 to 0.87)	1990-1994	0.78 (0.73 to 0.83)	1990-1994	7.60 (7.03 to 8.26)	1990-1994	0.88 (0.74 to 1.00)	1990-1995	6.80 (6.27 to 7.32)	1990-1995	5.67 (5.23 to 6.09)
	1	1993-1996	0.34 (0.23 to 0.44)	1994-2002	0.10 (0.07 to 0.12)	1994-1998	-2.31 (-3.16 to -1.84)	1994-2004	0.11 (0.05 to 0.16)	1995-1998	-4.45 (-5.13 to -3.66)	1995-1998	-3.68 (-4.27 to -3.02)
	2	1996-2003	0.08 (0.02 to 0.10)	2002-2006	0.44 (0.38 to 0.53)	1998-2001	0.35 (-0.44 to 0.94)	2004-2008	0.89 (0.08 to 0.99)	1998-2001	0.26 (-0.57 to 0.98)	1998-2001	0.23 (-0.47 to 0.86)
	3	2003-2006	0.42 (0.25 to 0.63)	2006-2011	1.17 (1.13 to 1.20)	2001-2004	-3.76 (-4.28 to -2.83)	2008-2012	1.37 (0.89 to 1.56)	2001-2004	-4.65 (-5.33 to -3.66)	2001-2004	-3.76 (-4.34 to -2.92)
	4	2006-2011	0.87 (0.84 to 0.92)	2011-2019	1.69 (1.66 to 1.71)	2004-2016	-0.82 (-0.94 to -0.62)	2012-2018	1.75 (1.46 to 1.84)	2004-2009	-1.16 (-1.89 to -0.49)	2004-2009	-0.71 (-1.33 to -0.11)
	5	2011-2019	1.50 (1.48 to 1.52)	2019-2021	2.18 (2.00 to 2.29)	2016-2021	-2.60 (-3.33 to -2.11)	2018-2021	2.07 (1.87 to 2.32)	2009-2015	0.35 (-0.10 to 1.45)	2009-2015	0.62 (0.27 to 1.54)
	6	2019-2021	2.05 (1.88 to 2.14)							2015-2021	-2.43 (-3.08 to -1.97)	2015-2021	-1.33 (-1.87 to -0.97)
Thailand	0	1990-1999	-0.86 (-1.09 to -0.67)	1990-1999	-0.90 (-1.16 to -0.66)	1990-2002	0.40 (0.13 to 0.67)	1990-2000	-0.76 (-0.97 to -0.58)	1990-2002	0.94 (0.57 to 1.30)	1990-1998	0.17 (-1.09 to 0.63)
	1	1999-2012	0.29 (0.13 to 0.59)	1999-2012	0.39 (-0.27 to 0.74)	2002-2007	-5.83 (-7.13 to -4.75)	2000-2010	0.58 (0.37 to 1.22)	2002-2007	-6.68 (-8.70 to -5.18)	1998-2002	1.66 (-5.91 to 2.84)
	2	2012-2019	-0.68 (-1.13 to 0.18)	2012-2019	-0.64 (-1.12 to 0.34)	2007-2014	-3.74 (-4.24 to -2.76)	2010-2019	-0.41 (-0.80 to -0.01)	2007-2014	-3.37 (-4.24 to -2.34)	2002-2006	-5.71 (-7.00 to -2.30)
	3	2019-2021	-3.21 (-4.17 to -1.31)	2019-2021	-3.17 (-4.18 to -1.17)	2014-2021	-0.77 (-1.20 to -0.13)	2019-2021	-3.44 (-4.38 to -1.60)	2014-2021	-0.58 (-1.18 to 0.45)	2006-2013	-2.52 (-3.33 to -0.84)
	4											2013-2021	-0.85 (-1.33 to -0.10)
Timor-Leste	0	1990-1995	0.42 (0.35 to 0.52)	1990-1994	0.34 (0.26 to 0.44)	1990-2000	-1.46 (-1.78 to -0.97)	1990-1994	0.47 (0.34 to 0.65)	1990-2000	-1.54 (-1.97 to -0.85)	1990-1999	-1.09 (-1.49 to -0.30)
	1	1995-2006	0.14 (0.11 to 0.16)	1994-2003	0.04 (-0.01 to 0.06)	2000-2008	-3.11 (-3.99 to -2.73)	1994-2000	0.01 (-0.19 to 0.08)	2000-2008	-3.64 (-4.70 to -3.15)	1999-2008	-2.45 (-3.26 to -2.12)
	2	2006-2010	0.61 (0.54 to 0.74)	2003-2006	0.30 (0.19 to 0.36)	2008-2013	2.85 (2.05 to 4.85)	2000-2005	0.31 (0.18 to 0.52)	2008-2013	4.07 (3.00 to 6.45)	2008-2013	2.92 (2.14 to 4.49)
	3	2010-2019	-0.43 (-0.46 to -0.40)	2006-2009	1.08 (0.99 to 1.16)	2013-2021	0.29 (-0.28 to 0.67)	2005-2009	0.95 (0.82 to 1.14)	2013-2021	0.31 (-0.36 to 0.81)	2013-2021	0.14 (-0.39 to 0.52)
	4	2019-2021	0.37 (0.15 to 0.50)	2009-2012	-0.04 (-0.16 to 0.03)			2009-2012	-0.05 (-0.27 to 0.31)				
	5			2012-2019	-0.55 (-0.58 to -0.52)			2012-2019	-0.52 (-0.63 to -0.48)				
	6			2019-2021	0.68 (0.53 to 0.81)			2019-2021	0.46 (0.12 to 0.66)				
Togo	0	1990-1994	-0.45 (-0.65 to -0.33)	1990-1995	-0.46 (-0.69 to -0.36)	1990-1995	-1.57 (-1.83 to -1.12)	1990-1995	-0.42 (-0.76 to -0.30)	1990-1995	-1.31 (-1.63 to -0.53)	1990-1995	-1.06 (-1.31 to -0.46)
	1	1994-1999	-0.23 (-0.33 to -0.04)	1995-2005	-0.14 (-0.18 to -0.08)	1995-2000	-2.60 (-3.32 to -2.30)	1995-2005	-0.12 (-0.18 to -0.02)	1995-2000	-2.34 (-3.15 to -1.94)	1995-2000	-1.69 (-2.24 to -1.38)
	2	1999-2005	-0.04 (-0.11 to 0.38)	2005-2010	0.48 (0.38 to 0.65)	2000-2006	-0.37 (-0.95 to -0.01)	2005-2009	0.55 (0.34 to 0.79)	2000-2007	-0.63 (-1.20 to -0.28)	2000-2007	-0.41 (-0.77 to -0.16)
	3	2005-2010	0.36 (-0.20 to 0.48)	2010-2015	-0.16 (-0.35 to -0.05)	2006-2010	1.08 (0.54 to 1.67)	2009-2016	0.00 (-0.21 to 0.09)	2007-2010	1.00 (0.09 to 1.42)	2007-2010	0.86 (0.16 to 1.15)
	4	2010-2015	-0.18 (-0.29 to 0.19)	2015-2021	0.25 (0.18 to 0.41)	2010-2017	-0.85 (-1.07 to -0.53)	2016-2021	0.25 (0.12 to 0.54)	2010-2017	-0.86 (-1.25 to -0.51)	2010-2017	-0.58 (-0.83 to -0.27)
	5	2015-2021	0.23 (0.16 to 0.35)			2017-2021	-1.95 (-2.81 to -1.53)			2017-2021	-2.51 (-3.60 to -1.97)	2017-2021	-1.55 (-2.31 to -1.18)
Tokelau	0	1990-1998	-0.24 (-0.34 to -0.15)	1990-1998	-0.17 (-0.32 to -0.06)	1990-2019	-1.51 (-2.01 to -1.06)	1990-1997	-0.19 (-0.38 to -0.06)	1990-2019	-1.68 (-2.25 to -1.17)	1990-2019	-1.21 (-1.58 to -0.87)
	1	1998-2010	0.31 (0.24 to 0.37)	1998-2010	0.39 (0.32 to 0.49)	2019-2021	98.53 (53.87 to 138.27)	1997-2011	0.36 (0.31 to 0.43)	2019-2021	131.98 (69.59 to 189.69)	2019-2021	95.38 (50.64 to 133.25)
	2	2010-2016	-0.28 (-0.65 to 0.29)	2010-2019	-0.24 (-0.33 to 0.10)			2011-2015	-0.53 (-0.88 to -0.26)				
	3	2016-2019	0.23 (-0.12 to 0.50)	2019-2021	-1.29 (-1.73 to -0.48)			2015-2019	0.25 (0.04 to 0.66)				
	4	2019-2021	-1.38 (-1.75 to -0.88)					2019-2021	-1.79 (-2.34 to -1.12)				
Tonga	0	1990-1995	-0.02 (-0.05 to 0.02)	1990-1994	-0.02 (-0.09 to 0.12)	1990-1992	-0.90 (-1.26 to -0.39)	1990-2006	-0.08 (-0.11 to -0.06)	1990-1995	-0.19 (-0.68 to 0.11)	1990-1995	-0.14 (-0.60 to 0.09)
	1	1995-2000	-0.17 (-0.23 to -0.14)	1994-2005	-0.15 (-0.18 to -0.13)	1992-1996	0.29 (0.03 to 0.69)	2006-2009	0.54 (0.30 to 0.64)	1995-2000	1.36 (1.11 to 1.84)	1995-2000	0.99 (0.78 to 1.39)
	2	2000-2005	-0.05 (-0.07 to -0.00)	2005-2010	0.42 (0.39 to 0.46)	1996-2000	1.12 (0.93 to 1.40)	2009-2015	-0.22 (-0.40 to -0.15)	2000-2004	-2.17 (-2.68 to -1.85)	2000-2004	-1.61 (-2.09 to -1.36)
	3	2005-2010	0.35 (0.32 to 0.37)	2010-2015	-0.36 (-0.42 to -0.32)	2000-2004	-1.86 (-2.03 to -1.69)	2015-2021	0.06 (-0.02 to 0.23)	2004-2008	0.67 (0.21 to 1.31)	2004-2009	0.30 (0.05 to 0.69)
	4	2010-2015	-0.29 (-0.31 to -0.27)	2015-2019	0.18 (0.13 to 0.29)	2004-2008	0.34 (0.08 to 0.56)			2008-2021	-1.21 (-1.30 to -1.13)	2009-2021	-0.96 (-1.03 to -0.89)
	5	2015-2019	0.23 (0.20 to 0.26)	2019-2021	-0.30 (-0.45 to -0.13)	2008-2014	-0.84 (-0.99 to -0.62)						
	6	2019-2021	-0.17 (-0.26 to -0.05)			2014-2021	-1.25 (-1.44 to -1.15)						
Trinidad and Tobago	0	1990-1999	-1.19 (-1.35 to -1.05)	1990-1999	-1.42 (-1.51 to -1.32)	1990-1994	1.79 (0.83 to 3.54)	1990-1999	-1.33 (-1.48 to -1.21)	1990-1994	2.04 (1.21 to 3.21)	1990-1995	1.02 (0.50 to 1.76)
	1	1999-2010	0.19 (0.06 to 0.39)	1999-2010	0.22 (0.14 to 0.33)	1994-1999	-2.09 (-2.89 to -0.98)	1999-2010	0.22 (0.12 to 0.39)	1994-1999	-1.55 (-2.20 to -0.79)	1995-1999	-1.93 (-2.61 to -0.82)
	2	2010-2015	-0.71 (-1.10 to 0.00)	2010-2015	-0.79 (-1.22 to -0.20)	1999-2002	-6.11 (-6.96 to -4.54)	2010-2015	-0.68 (-1.13 to -0.10)	1999-2002	-6.13 (-6.87 to -4.78)	1999-2002	-4.95 (-5.61 to -3.82)

Location	Segment	Incidence		Prevalence		Mortality		YLD		YLL		DALY	
		Period	APC (95%CI)	Period	APC (95%CI)	Period	APC (95%CI)	Period	APC (95%CI)	Period	APC (95%CI)	Period	APC (95%CI)
	3	2015-2021	-1.60 (-2.15 to -1.38)	2015-2021	-1.80 (-2.26 to -1.59)	2002-2008	-2.39 (-2.96 to -0.86)	2015-2021	-1.73 (-2.18 to -1.51)	2002-2008	-2.11 (-2.52 to -1.06)	2002-2008	-1.67 (-2.04 to -0.57)
	4					2008-2012	-4.17 (-5.48 to -0.46)			2008-2013	-4.35 (-5.60 to -3.68)	2008-2013	-3.39 (-4.61 to -2.78)
	5					2012-2021	0.25 (-0.53 to 1.18)			2013-2016	3.01 (0.88 to 4.09)	2013-2016	1.88 (0.29 to 2.84)
	6									2016-2021	-1.46 (-3.35 to -0.59)	2016-2021	-1.39 (-3.04 to -0.77)
Tunisia	0	1990-1994	-0.10 (-0.17 to 0.02)	1990-1995	-0.25 (-0.30 to -0.18)	1990-2001	-1.41 (-1.62 to -1.23)	1990-2000	-0.28 (-0.34 to -0.23)	1990-1996	-1.44 (-1.74 to -0.80)	1990-2001	-1.14 (-1.24 to -1.04)
	1	1994-2000	-0.30 (-0.36 to -0.27)	1995-2000	-0.47 (-0.53 to -0.43)	2001-2006	0.65 (0.09 to 1.80)	2000-2015	0.46 (0.42 to 0.49)	1996-2000	-2.41 (-3.16 to -0.85)	2001-2006	0.59 (0.26 to 1.06)
	2	2000-2004	0.66 (0.61 to 0.73)	2000-2006	0.57 (0.53 to 0.61)	2006-2021	-1.39 (-1.50 to -1.29)	2015-2021	1.05 (0.93 to 1.21)	2000-2006	0.41 (-0.05 to 0.91)	2006-2011	-0.89 (-1.35 to -0.64)
	3	2004-2011	0.50 (0.42 to 0.53)	2006-2015	0.38 (0.36 to 0.40)					2006-2011	-1.91 (-2.52 to -1.48)	2011-2021	-0.39 (-0.47 to -0.24)
	4	2011-2015	0.68 (0.58 to 0.84)	2015-2019	0.95 (0.85 to 0.99)					2011-2017	-1.07 (-1.35 to -0.38)		
	5	2015-2021	1.03 (0.99 to 1.09)	2019-2021	1.38 (1.21 to 1.51)					2017-2021	-2.45 (-3.16 to -2.04)		
Turkmenistan	0	1990-1993	1.01 (0.88 to 1.20)	1990-1993	1.07 (0.96 to 1.23)	1990-1996	5.93 (4.81 to 7.84)	1990-1999	0.94 (0.88 to 1.01)	1990-1994	7.94 (5.26 to 12.86)	1990-1994	6.74 (4.65 to 10.52)
	1	1993-1999	0.76 (0.61 to 0.80)	1993-1999	0.86 (0.73 to 0.90)	1996-1999	-5.83 (-7.11 to -2.62)	1999-2006	0.43 (0.26 to 0.52)	1994-1997	2.74 (-6.75 to 4.82)	1994-1997	2.54 (-5.87 to 4.31)
	2	1999-2006	0.28 (0.24 to 0.32)	1999-2006	0.41 (0.37 to 0.46)	1999-2021	-0.44 (-0.74 to -0.06)	2006-2011	1.22 (0.99 to 1.41)	1997-2000	-5.77 (-6.82 to 2.57)	1997-2000	-5.00 (-5.93 to 2.56)
	3	2006-2011	0.93 (0.89 to 0.97)	2006-2010	1.15 (1.02 to 1.20)			2011-2018	2.12 (1.92 to 2.21)	2000-2021	0.27 (-5.20 to 0.65)	2000-2021	0.44 (-2.71 to 0.81)
	4	2011-2019	1.84 (1.81 to 1.87)	2010-2013	1.77 (1.47 to 1.91)			2018-2021	2.68 (2.37 to 3.07)				
	5	2019-2021	2.53 (2.28 to 2.67)	2013-2019	2.18 (2.09 to 2.24)								
	6			2019-2021	2.63 (2.40 to 2.75)								
Tuvalu	0	1990-1994	0.18 (0.01 to 0.44)	1990-2005	0.09 (0.06 to 0.11)	1990-1994	-1.09 (-2.14 to -0.56)	1990-2005	0.09 (0.05 to 0.11)	1990-1994	-1.24 (-2.63 to -0.57)	1990-1994	-0.87 (-1.84 to -0.43)
	1	1994-2002	-0.05 (-0.23 to 0.19)	2005-2010	0.36 (0.23 to 0.63)	1994-2001	-0.02 (-0.21 to 0.56)	2005-2010	0.45 (0.30 to 0.70)	1994-2001	0.10 (-0.10 to 0.80)	1994-2001	0.08 (-0.06 to 0.54)
	2	2002-2010	0.19 (-0.22 to 0.30)	2010-2019	-0.60 (-0.64 to -0.43)	2001-2005	-2.41 (-3.00 to -1.94)	2010-2019	-0.52 (-0.57 to -0.43)	2001-2004	-3.12 (-3.67 to -2.22)	2001-2004	-2.18 (-2.58 to -1.56)
	3	2010-2019	-0.38 (-0.42 to -0.20)	2019-2021	-1.28 (-1.55 to -0.77)	2005-2008	0.29 (-0.35 to 0.65)	2019-2021	-1.21 (-1.49 to -0.79)	2004-2007	0.37 (-0.29 to 0.90)	2004-2008	0.20 (-0.12 to 0.78)
	4	2019-2021	-0.93 (-1.15 to -0.53)			2008-2017	-0.80 (-1.01 to -0.67)			2007-2016	-0.85 (-1.08 to -0.71)	2008-2016	-0.72 (-0.90 to -0.57)
	5					2017-2021	-2.17 (-2.87 to -1.79)			2016-2021	-2.10 (-2.73 to -1.76)	2016-2021	-1.70 (-2.14 to -1.46)
TÃ□Â¼rkiye	0	1990-1993	-0.59 (-0.79 to -0.45)	1990-1998	-0.37 (-0.47 to -0.34)	1990-2000	-4.36 (-4.72 to -3.32)	1990-1998	-0.41 (-0.49 to -0.37)	1990-2000	-4.43 (-4.81 to -3.47)	1990-1992	-5.51 (-6.49 to -3.86)
	1	1993-2000	-0.33 (-0.37 to -0.25)	1998-2001	-0.08 (-0.23 to 0.00)	2000-2006	-5.83 (-7.40 to -5.10)	1998-2001	0.14 (-0.06 to 0.26)	2000-2006	-6.09 (-7.78 to -5.32)	1992-2006	-2.81 (-2.94 to -2.24)
	2	2000-2005	0.69 (0.62 to 0.77)	2001-2004	1.11 (0.96 to 1.22)	2006-2015	-2.20 (-2.56 to -1.34)	2001-2004	2.37 (2.22 to 2.52)	2006-2021	-3.32 (-3.50 to -3.10)	2006-2015	-1.28 (-3.19 to -0.94)
	3	2005-2010	0.52 (0.05 to 0.57)	2004-2009	0.63 (0.52 to 0.68)	2015-2021	-3.73 (-4.93 to -3.14)	2004-2009	0.84 (0.74 to 0.93)			2015-2018	-2.62 (-3.13 to -0.79)
	4	2010-2017	-0.20 (-0.26 to -0.16)	2009-2017	-0.07 (-0.12 to -0.04)			2009-2015	-0.13 (-0.22 to -0.05)			2018-2021	-1.06 (-2.04 to 0.14)
	5	2017-2021	0.23 (0.13 to 0.38)	2017-2021	0.47 (0.37 to 0.64)			2015-2019	-1.06 (-1.20 to -0.91)				
	6							2019-2021	0.72 (0.44 to 0.97)				
Uganda	0	1990-1998	-0.11 (-0.19 to -0.04)	1990-1998	0.09 (-0.25 to 0.20)	1990-1996	3.59 (3.12 to 4.46)	1990-1998	0.11 (-0.13 to 0.22)	1990-1996	3.72 (3.08 to 4.74)	1990-1996	2.61 (2.14 to 3.35)
	1	1998-2009	0.30 (0.25 to 0.40)	1998-2010	0.35 (0.29 to 0.58)	1996-2000	1.71 (-0.24 to 2.67)	1998-2010	0.43 (0.37 to 0.57)	1996-2001	1.28 (0.25 to 2.50)	1996-2001	1.01 (0.24 to 1.97)
	2	2009-2019	0.08 (0.02 to 0.21)	2010-2021	-0.18 (-0.26 to -0.11)	2000-2005	-1.08 (-2.62 to -0.49)	2010-2021	-0.15 (-0.23 to -0.08)	2001-2006	-1.31 (-2.39 to -0.17)	2001-2006	-0.80 (-1.74 to 0.21)
	3	2019-2021	-0.51 (-0.77 to -0.04)			2005-2010	-3.20 (-4.23 to -1.78)			2006-2010	-3.37 (-4.65 to 1.40)	2006-2010	-2.27 (-3.22 to 1.17)
	4					2010-2015	1.24 (0.46 to 2.34)			2010-2015	1.89 (-0.78 to 3.35)	2010-2015	1.28 (-1.19 to 2.36)
	5					2015-2021	-1.74 (-2.34 to -1.25)			2015-2021	-2.15 (-2.91 to -1.50)	2015-2021	-1.54 (-2.11 to -1.04)
Ukraine	0	1990-2001	-0.02 (-0.07 to 0.02)	1990-2001	-0.02 (-0.07 to 0.03)	1990-1995	10.13 (7.59 to 12.17)	1990-2001	0.00 (-0.05 to 0.06)	1990-1995	11.01 (8.87 to 12.88)	1990-1995	8.91 (8.24 to 9.63)
	1	2001-2006	0.89 (0.71 to 1.10)	2001-2006	1.00 (0.76 to 1.23)	1995-2007	-3.67 (-4.16 to -3.18)	2001-2006	1.08 (0.81 to 1.31)	1995-2007	-3.88 (-4.40 to -3.35)	1995-1998	-4.69 (-5.50 to -3.79)
	2	2006-2014	2.00 (1.92 to 2.19)	2006-2021	2.14 (2.11 to 2.18)	2007-2013	-9.74 (-12.02 to -8.59)	2006-2021	2.20 (2.17 to 2.24)	2007-2013	-9.90 (-12.37 to -8.73)	1998-2001	-0.05 (-1.30 to 0.80)
	3	2014-2021	1.68 (1.46 to 1.77)			2013-2021	-3.12 (-5.25 to -0.56)			2013-2021	-2.24 (-4.53 to 0.48)	2001-2008	-4.21 (-4.71 to -3.71)
	4											2008-2013	-6.10 (-8.10 to -5.03)
	5											2013-2021	0.37 (-0.57 to 1.51)
United Arab Emirates	0	1990-1994	-0.80 (-1.83 to -0.26)	1990-1999	-0.41 (-0.50 to -0.33)	1990-1994	-5.02 (-10.38 to -2.43)	1990-2000	-0.58 (-0.67 to -0.50)	1990-1995	-3.07 (-6.48 to -1.57)	1990-1994	-2.07 (-3.46 to -1.44)
	1	1994-2015	-0.04 (-0.09 to 0.05)	1999-2005	0.38 (0.22 to 0.73)	1994-2003	0.78 (-0.63 to 2.24)	2000-2014	0.34 (0.28 to 0.39)	1995-2007	0.43 (0.03 to 1.14)	1994-2002	-0.30 (-0.74 to 0.02)
	2	2015-2019	1.51 (1.02 to 2.29)	2005-2011	-0.11 (-0.45 to 0.04)	2003-2008	5.98 (3.88 to 9.49)	2014-2019	1.70 (1.46 to 2.13)	2007-2012	-4.33 (-5.86 to -3.30)	2002-2007	0.85 (0.37 to 1.66)
	3	2019-2021	-1.12 (-2.10 to 0.05)	2011-2016	0.91 (0.66 to 1.08)	2008-2012	-6.81 (-9.13 to -4.62)	2019-2021	-0.09 (-0.58 to 0.65)	2012-2015	10.82 (8.06 to 12.43)	2007-2012	-2.18 (-2.62 to -1.76)
	4			2016-2019	2.05 (1.70 to 2.32)	2012-2015	16.18 (11.95 to 18.58)			2015-2019	-1.71 (-3.10 to -0.30)	2012-2015	5.65 (4.94 to 6.25)
	5			2019-2021	-0.00 (-0.45 to 0.54)	2015-2019	-0.77 (-2.56 to 1.19)			2019-2021	-13.70 (-16.53 to -9.65)	2015-2019	0.02 (-0.50 to 0.51)
	6					2019-2021	-14.27 (-18.28 to -9.27)					2019-2021	-6.50 (-7.70 to -5.13)
United Kingdom	0	1990-1994	1.16 (0.51 to 1.48)	1990-2004	1.71 (1.62 to 1.79)	1990-1994	-4.73 (-7.25 to -3.50)	1990-1995	1.40 (1.02 to 1.69)	1990-1994	-5.51 (-9.12 to -3.82)	1990-1995	-1.54 (-2.11 to -1.16)
	1	1994-2003	1.80 (1.70 to 2.05)	2004-2011	0.38 (-0.18 to 0.69)	1994-2008	-2.30 (-2.54 to -1.83)	1995-2000	3.50 (3.26 to 3.83)	1994-2008	-1.91 (-2.22 to -1.30)	1995-2000	1.74 (1.24 to 2.53)
	2	2003-2011	0.46 (0.17 to 0.60)	2011-2015	1.17 (0.72 to 1.66)	2008-2014	-5.43 (-7.52 to -4.60)	2000-2010	0.20 (0.06 to 0.30)	2008-2014	-5.24 (-8.17 to -4.13)	2000-2016	-0.68 (-0.78 to -0.58)
	3	2011-2015	1.29 (0.98 to 1.69)	2015-2019	-5.13 (-5.47 to -4.78)	2014-2017	1.60 (-0.92 to 3.07)	2010-2015	1.15 (0.88 to 1.58)	2014-2018	0.77 (-1.15 to 3.46)	2016-2019	-4.60 (-5.24 to -3.40)
	4	2015-2019	-4.30 (-4.55 to -4.04)	2019-2021	2.59 (1.76 to 3.38)	2017-2021	-4.62 (-7.27 to -3.47)	2015-2019	-4.96 (-5.25 to -4.67)	2018-2021	-6.56 (-11.51 to -4.13)	2019-2021	0.14 (-1.46 to 1.43)
	5	2019-2021	2.27 (1.60 to 2.88)					2019-2021	2.36 (1.62 to 3.12)				
United Republic of Tanzania	0	1990-1994	-1.36 (-2.62 to -0.80)	1990-1994	-2.13 (-4.21 to -1.21)	1990-1998	-0.29 (-0.40 to -0.13)	1990-1994	-1.80 (-3.81 to -0.89)	1990-1998	-0.55 (-0.68 to -0.33)	1990-2001	-0.64 (-0.76 to -0.53)
	1	1994-2000	0.61 (0.37 to 1.46)	1994-2000	1.03 (0.67 to 2.34)	1998-2001	-2.43 (-2.75 to -1.84)	1994-2002	0.94 (0.67 to 2.38)	1998-2001	-2.19 (-2.55 to -1.56)	2001-2007	1.13 (0.81 to 1.68)
	2	2000-2011	0.11 (-0.07 to 0.21)	2000-2011	0.11 (-0.14 to 0.28)	2001-2007	1.17 (0.92 to 1.44)	2002-2011	0.17 (-0.24 to 0.43)	2001-2007	1.67 (1.39 to 1.97)	2007-2015	-0.57 (-0.78 to -0.33)
	3	2011-2019	-0.51 (-1.16 to -0.35)	2011-2019	-0.96 (-1.86 to -0.72)	2007-2017	-0.79 (-0.90 to -0.68)	2011-2019	-0.96 (-2.10 to -0.69)	2007-2017	-0.80 (-0.92 to -0.67)	2015-2021	-1.87 (-2.25 to -1.59)
	4	2019-2021	3.44 (1.70 to 4.34)	2019-2021	4.44 (1.62 to 5.87)	2017-2021	-2.99 (-3.37 to -2.55)	2019-2021	4.49 (2.22 to 5.72)	2017-2021	-3.71 (-4.10 to -3.18)		
United States Virgin Islands	0	1990-2000	-0.74 (-0.81 to -0.68)	1990-2000	-0.97 (-1.04 to -0.91)	1990-2000	-1.39 (-1.69 to -1.01)	1990-2000	-0.89 (-0.96 to -0.82)	1990-1998	-0.61 (-1.22 to 0.42)	1990-1998	-0.72 (-1.30 to 0.60)
	1	2000-2010	0.20 (0.13 to 0.28)	2000-2010	0.10 (0.03 to 0.18)	2000-2011	-3.38 (-3.73 to -3.11)	2000-2010	0.10 (0.03 to 0.19)	1998-2010	-2.81 (-4.15 to -2.45)	1998-2010	-2.10 (-3.00 to -1.20)

Location	Segment	Incidence		Prevalence		Mortality		YLD		YLL		DALY	
		Period	APC (95%CI)	Period	APC (95%CI)	Period	APC (95%CI)	Period	APC (95%CI)	Period	APC (95%CI)	Period	APC (95%CI)
	2	2010-2021	-0.49 (-0.56 to -0.43)	2010-2021	-0.58 (-0.64 to -0.52)	2011-2019	0.21 (-0.37 to 1.49)	2010-2021	-0.52 (-0.58 to -0.46)	2010-2021	-0.41 (-1.18 to 1.02)	2010-2019	-0.15 (-2.25 to 1.76)
	3					2019-2021	-6.45 (-9.27 to -1.95)					2019-2021	-4.14 (-6.56 to -0.29)
United States of America	0	1990-1994	1.27 (1.20 to 1.34)	1990-1993	1.60 (1.43 to 1.81)	1990-2002	1.30 (1.19 to 1.43)	1990-1993	1.87 (1.73 to 2.05)	1990-1997	1.20 (0.19 to 1.55)	1990-2002	1.16 (1.09 to 1.23)
	1	1994-2002	0.24 (0.21 to 0.29)	1993-1996	0.61 (0.36 to 0.89)	2002-2006	-2.43 (-2.85 to -1.47)	1993-1996	0.62 (0.44 to 0.79)	1997-2002	2.30 (1.75 to 3.14)	2002-2006	-1.04 (-1.29 to -0.38)
	2	2002-2011	0.12 (0.08 to 0.15)	1996-2011	0.10 (0.08 to 0.12)	2006-2013	-4.37 (-4.64 to -4.15)	1996-2011	0.12 (0.10 to 0.14)	2002-2006	-2.15 (-2.81 to -0.99)	2006-2013	-1.95 (-2.15 to -1.81)
	3	2011-2016	1.06 (1.00 to 1.12)	2011-2015	0.76 (0.62 to 0.96)	2013-2017	1.18 (0.71 to 1.65)	2011-2015	0.81 (0.69 to 1.00)	2006-2014	-3.71 (-4.05 to -3.48)	2013-2017	1.46 (1.09 to 1.83)
	4	2016-2021	0.61 (0.52 to 0.69)	2015-2021	0.44 (0.31 to 0.51)	2017-2021	-2.04 (-2.67 to -1.43)	2015-2021	0.41 (0.32 to 0.48)	2014-2017	3.85 (3.03 to 4.56)	2017-2021	-0.84 (-1.26 to -0.42)
	5									2017-2021	-2.52 (-3.41 to -1.84)		
Uruguay	0	1990-1994	0.42 (0.24 to 0.73)	1990-1995	0.41 (0.28 to 0.64)	1990-1994	1.88 (0.79 to 2.95)	1990-2006	0.20 (0.16 to 0.23)	1990-1995	1.30 (0.20 to 2.79)	1990-1994	1.24 (0.67 to 1.76)
	1	1994-2006	0.14 (0.06 to 0.17)	1995-2005	0.13 (-0.01 to 0.18)	1994-2001	-3.28 (-4.34 to -2.77)	2006-2013	0.98 (0.88 to 1.27)	1995-2001	-3.91 (-6.04 to -3.06)	1994-2002	-1.44 (-1.85 to -1.16)
	2	2006-2014	1.06 (1.00 to 1.21)	2005-2014	0.88 (0.28 to 1.13)	2001-2013	-1.64 (-2.42 to -1.37)	2013-2021	0.70 (0.54 to 0.78)	2001-2021	-1.52 (-1.69 to -1.31)	2002-2013	-0.20 (-1.33 to -0.05)
	3	2014-2021	0.69 (0.56 to 0.78)	2014-2021	0.72 (0.45 to 0.82)	2013-2021	-0.82 (-1.28 to 0.62)					2013-2021	0.19 (-0.06 to 1.03)
Uzbekistan	0	1990-1999	0.82 (0.73 to 0.96)	1990-2000	0.83 (0.74 to 1.04)	1990-1994	15.10 (13.74 to 16.76)	1990-1999	0.90 (0.79 to 1.17)	1990-1994	15.60 (12.69 to 17.94)	1990-1994	12.11 (11.12 to 13.37)
	1	1999-2007	0.40 (-0.06 to 0.53)	2000-2007	0.52 (0.01 to 0.70)	1994-2004	-0.59 (-1.21 to -0.25)	1999-2007	0.55 (0.02 to 0.70)	1994-2011	-0.47 (-1.40 to -0.21)	1994-2004	-0.43 (-1.19 to -0.15)
	2	2007-2012	1.19 (0.80 to 1.80)	2007-2012	1.41 (1.02 to 2.29)	2004-2007	4.85 (2.46 to 6.14)	2007-2012	1.46 (1.02 to 2.17)	2011-2015	2.23 (0.07 to 4.46)	2004-2007	2.30 (0.49 to 3.32)
	3	2012-2021	2.50 (2.39 to 2.65)	2012-2021	2.75 (2.63 to 2.90)	2007-2010	-4.25 (-5.55 to -2.14)	2012-2021	2.80 (2.67 to 2.98)	2015-2021	-4.73 (-6.77 to -3.51)	2007-2010	-3.00 (-4.17 to -1.04)
	4					2010-2015	1.54 (0.65 to 3.74)					2010-2015	2.39 (1.40 to 4.48)
	5					2015-2021	-4.40 (-5.39 to -3.61)					2015-2021	-2.66 (-3.70 to -1.86)
Vanuatu	0	1990-1994	0.36 (0.33 to 0.39)	1990-1994	0.43 (0.33 to 0.59)	1990-1992	4.42 (2.31 to 6.10)	1990-1992	1.00 (0.53 to 1.36)	1990-2001	2.04 (1.81 to 2.33)	1990-2001	1.56 (1.38 to 1.78)
	1	1994-2002	-0.01 (-0.03 to 0.01)	1994-2003	-0.00 (-0.05 to 0.04)	1992-2001	1.57 (0.99 to 1.80)	1992-2002	-0.02 (-0.15 to 0.03)	2001-2004	-4.73 (-5.63 to -2.96)	2001-2004	-3.62 (-4.35 to -2.26)
	2	2002-2011	0.12 (0.10 to 0.13)	2003-2015	0.19 (-0.01 to 0.25)	2001-2004	-4.11 (-4.79 to -2.67)	2002-2015	0.20 (0.16 to 0.27)	2004-2007	3.92 (2.34 to 5.02)	2004-2007	3.07 (1.85 to 3.93)
	3	2011-2015	0.24 (0.19 to 0.29)	2015-2019	-0.12 (-0.18 to 0.21)	2004-2007	3.71 (2.32 to 4.52)	2015-2021	-0.19 (-0.34 to -0.08)	2007-2013	-1.45 (-2.80 to -1.00)	2007-2013	-1.12 (-2.17 to -0.78)
	4	2015-2021	0.02 (-0.01 to 0.04)	2019-2021	-0.48 (-0.65 to -0.21)	2007-2012	-1.26 (-2.31 to -0.76)			2013-2019	0.15 (-0.30 to 1.55)	2013-2018	0.35 (-0.16 to 1.41)
	5					2012-2019	-0.00 (-0.28 to 1.16)			2019-2021	-4.50 (-6.27 to -2.43)	2018-2021	-2.41 (-3.92 to -1.51)
	6					2019-2021	-3.38 (-4.77 to -1.67)						
Venezuela (Bolivarian Republic of)	0	1990-1994	0.60 (0.47 to 0.85)	1990-1994	0.81 (0.68 to 1.04)	1990-1996	1.20 (-0.11 to 2.80)	1990-1997	0.54 (0.42 to 0.71)	1990-1996	2.68 (-0.55 to 5.07)	1990-1996	2.30 (0.99 to 3.60)
	1	1994-2000	0.17 (0.08 to 0.32)	1994-1999	0.31 (0.16 to 0.47)	1996-2007	-2.71 (-4.29 to -2.13)	1997-2006	0.05 (-0.10 to 0.17)	1996-2007	-2.67 (-4.93 to 4.96)	1996-2006	-2.18 (-3.68 to 1.57)
	2	2000-2005	-0.07 (-0.22 to 0.02)	1999-2005	-0.04 (-0.17 to 0.02)	2007-2013	0.34 (-2.30 to 2.30)	2006-2010	1.05 (0.10 to 1.37)	2007-2013	0.88 (-3.18 to 3.37)	2006-2014	0.51 (-2.35 to 1.70)
	3	2005-2010	0.68 (0.59 to 0.77)	2005-2010	0.85 (0.77 to 0.94)	2013-2021	4.11 (2.28 to 6.87)	2010-2019	-0.84 (-1.03 to 0.95)	2013-2021	4.85 (-2.83 to 9.82)	2014-2017	5.68 (0.25 to 7.39)
	4	2010-2015	-0.74 (-0.81 to -0.57)	2010-2014	-0.66 (-0.77 to -0.49)			2019-2021	-0.09 (-0.77 to 0.29)			2017-2021	-0.28 (-6.41 to 2.84)
	5	2015-2018	-1.19 (-1.31 to -0.99)	2014-2018	-1.20 (-1.38 to -1.05)								
	6	2018-2021	-0.19 (-0.35 to 0.10)	2018-2021	-0.16 (-0.34 to 0.08)								
Viet Nam	0	1990-1999	0.31 (0.27 to 0.37)	1990-1995	0.19 (0.14 to 0.29)	1990-1995	-1.91 (-2.01 to -1.81)	1990-1994	0.37 (0.23 to 0.66)	1990-1994	-2.10 (-2.23 to -1.98)	1990-1994	-1.42 (-1.56 to -1.18)
	1	1999-2005	0.01 (-0.22 to 0.08)	1995-2001	0.04 (-0.05 to 0.07)	1995-2000	-2.55 (-2.65 to -2.45)	1994-2002	0.07 (-0.14 to 0.13)	1994-2000	-2.76 (-2.83 to -2.69)	1994-2000	-1.88 (-2.01 to -1.80)
	2	2005-2010	0.43 (0.32 to 0.67)	2001-2005	0.15 (0.11 to 0.36)	2000-2005	-1.34 (-1.44 to -1.25)	2002-2006	0.34 (0.13 to 0.83)	2000-2005	-1.46 (-1.54 to -1.37)	2000-2011	-0.91 (-0.95 to -0.87)
	3	2010-2021	-0.02 (-0.06 to 0.02)	2005-2010	0.63 (0.60 to 0.67)	2005-2009	-1.96 (-2.13 to -1.82)	2006-2009	0.90 (0.04 to 1.04)	2005-2009	-1.95 (-2.12 to -1.83)	2011-2021	-0.27 (-0.32 to -0.22)
	4			2010-2017	0.10 (0.06 to 0.12)	2009-2012	-1.47 (-1.68 to -1.00)	2009-2014	0.06 (-0.14 to 0.40)	2009-2012	-1.23 (-1.59 to -0.81)		
	5			2017-2021	0.28 (0.22 to 0.38)	2012-2017	-0.59 (-0.67 to -0.38)	2014-2021	0.30 (0.19 to 0.48)	2012-2018	-0.44 (-0.51 to -0.31)		
	6					2017-2021	-1.07 (-1.24 to -0.95)			2018-2021	-1.02 (-1.27 to -0.84)		
Yemen	0	1990-2000	-0.24 (-0.33 to -0.17)	1990-2000	-0.24 (-0.34 to -0.16)	1990-1996	-0.22 (-0.76 to 1.16)	1990-2000	-0.25 (-0.36 to -0.16)	1990-2001	-0.77 (-1.27 to -0.42)	1990-1996	-0.16 (-0.59 to 0.80)
	1	2000-2014	0.31 (0.25 to 0.36)	2000-2014	0.41 (0.35 to 0.47)	1996-2001	-1.45 (-2.65 to 0.70)	2000-2014	0.42 (0.35 to 0.49)	2001-2008	1.55 (0.94 to 2.79)	1996-2001	-1.08 (-2.04 to 1.01)
	2	2014-2021	0.97 (0.84 to 1.16)	2014-2021	1.13 (0.99 to 1.33)	2001-2008	1.44 (0.96 to 2.01)	2014-2021	1.08 (0.92 to 1.34)	2008-2012	-4.13 (-5.54 to -2.85)	2001-2008	1.46 (-0.46 to 1.95)
	3					2008-2011	-4.45 (-5.00 to -2.54)			2012-2021	-0.49 (-0.84 to 0.01)	2008-2011	-3.45 (-3.88 to 1.14)
	4					2011-2017	-0.99 (-1.78 to -0.49)					2011-2017	-0.58 (-2.63 to -0.25)
	5					2017-2021	0.77 (0.03 to 2.20)					2017-2021	0.56 (-0.04 to 1.69)
Zambia	0	1990-1998	-0.49 (-0.66 to -0.37)	1990-1997	-0.37 (-0.84 to -0.18)	1990-1996	1.26 (1.03 to 1.52)	1990-1996	-0.48 (-1.03 to -0.23)	1990-1995	1.29 (0.99 to 1.71)	1990-1996	0.89 (0.58 to 1.16)
	1	1998-2013	0.11 (0.06 to 0.18)	1997-2012	0.14 (0.07 to 0.25)	1996-1999	-1.85 (-2.37 to -0.47)	1996-2013	0.13 (0.07 to 0.23)	1995-1998	-0.88 (-2.08 to 0.28)	1996-2003	-1.95 (-2.27 to -1.72)
	2	2013-2021	-0.95 (-1.13 to -0.80)	2012-2021	-1.19 (-1.37 to -1.04)	1999-2003	-3.15 (-3.72 to -2.63)	2013-2021	-1.08 (-1.30 to -0.91)	1998-2003	-2.97 (-3.54 to -2.68)	2003-2010	-0.73 (-0.96 to -0.37)
	3					2003-2010	-1.16 (-1.39 to -0.93)			2003-2006	-0.24 (-0.91 to 0.15)	2010-2021	-2.26 (-2.37 to -2.16)
	4					2010-2021	-2.62 (-2.71 to -2.53)			2006-2011	-1.72 (-2.22 to -1.43)		
	5									2011-2021	-2.81 (-2.95 to -2.70)		
Zimbabwe	0	1990-2000	0.21 (0.06 to 0.46)	1990-2000	0.29 (0.15 to 0.47)	1990-1997	-5.13 (-5.69 to -4.60)	1990-2000	0.26 (0.11 to 0.46)	1990-1994	-4.09 (-5.20 to -1.72)	1990-1997	-2.67 (-3.19 to -2.17)
	1	2000-2011	-0.40 (-0.68 to -0.27)	2000-2011	-0.55 (-0.75 to -0.43)	1997-2003	14.63 (13.54 to 15.90)	2000-2011	-0.57 (-0.81 to -0.43)	1994-1997	-9.70 (-11.27 to -7.62)	1997-2003	6.72 (5.93 to 8.16)
	2	2011-2021	1.48 (1.33 to 1.66)	2011-2021	1.35 (1.20 to 1.52)	2003-2009	4.47 (2.43 to 6.93)	2011-2021	1.33 (1.16 to 1.54)	1997-2002	17.34 (15.48 to 19.14)	2003-2009	2.73 (1.45 to 4.45)
	3					2009-2021	-0.60 (-1.19 to -0.09)			2002-2009	5.64 (4.00 to 7.44)	2009-2021	0.29 (-0.23 to 0.63)
	4									2009-2021	-0.27 (-0.86 to 0.25)		

APC: annual percentage change; CI: confidence interval; DALY: Disability adjusted life years; SDI: Sociodemographic index; YLD: Years lived with disability; YLL: Years of life lost.

Table S8. Annual percentage changes estimated from joinpoint regression for age-standardized **incidence, prevalence, mortality, YLD, YLL, and DALY** of type 2 diabetes among **women** globally and by different SDI, regions, countries, and territories, 1990-2021

Location	Segment	Incidence		Prevalence		Mortality		YLD		YLL		DALY	
		Period	APC (95%CI)	Period	APC (95%CI)	Period	APC (95%CI)	Period	APC (95%CI)	Period	APC (95%CI)	Period	APC (95%CI)
Global	0	1990-1993	2.24 (2.14 to 2.45)	1990-1996	2.07 (1.96 to 2.15)	1990-1995	1.04 (0.85 to 1.22)	1990-1993	2.38 (2.28 to 2.59)	1990-1995	1.12 (0.92 to 1.29)	1990-1994	1.60 (1.43 to 1.82)
	1	1993-1996	1.61 (1.53 to 1.71)	1996-1999	3.25 (3.03 to 3.38)	1995-2000	-0.18 (-0.50 to -0.03)	1993-1996	2.02 (1.92 to 2.15)	1995-2000	-0.34 (-0.65 to -0.20)	1994-2004	0.96 (0.90 to 1.01)
	2	1996-1999	2.05 (1.94 to 2.13)	1999-2009	1.75 (1.70 to 1.79)	2000-2003	0.99 (0.57 to 1.17)	1996-1999	2.77 (2.58 to 2.86)	2000-2003	0.54 (0.18 to 0.70)	2004-2011	0.42 (0.22 to 0.51)
	3	1999-2007	1.50 (1.43 to 1.52)	2009-2019	2.07 (2.01 to 2.12)	2003-2012	-0.59 (-0.67 to -0.53)	1999-2004	2.08 (2.00 to 2.16)	2003-2012	-0.67 (-0.74 to -0.61)	2011-2021	1.42 (1.36 to 1.49)
	4	2007-2011	1.69 (1.55 to 1.81)	2019-2021	2.81 (2.43 to 2.99)	2012-2019	0.85 (0.77 to 0.99)	2004-2007	1.71 (1.65 to 1.88)	2012-2019	0.97 (0.88 to 1.08)		
	5	2011-2014	1.95 (1.73 to 2.01)			2019-2021	-0.67 (-1.19 to -0.06)	2007-2016	1.93 (1.90 to 2.05)	2019-2021	-0.37 (-0.89 to 0.27)		
	6	2014-2021	1.61 (1.55 to 1.64)					2016-2021	2.33 (2.26 to 2.45)				
**By SDI**													
High SDI	0	1990-1993	1.38 (1.12 to 1.52)	1990-1993	1.52 (1.21 to 1.67)	1990-1994	0.26 (-0.08 to 0.74)	1990-1993	1.59 (1.30 to 1.73)	1990-1994	0.29 (-0.08 to 0.76)	1990-1997	0.79 (0.55 to 0.92)
	1	1993-1996	1.86 (1.73 to 1.98)	1993-1996	2.20 (2.05 to 2.34)	1994-1998	-1.53 (-2.11 to -1.13)	1993-1996	2.44 (2.28 to 2.56)	1994-1998	-1.73 (-2.27 to -1.31)	1997-2003	1.48 (1.27 to 1.80)
	2	1996-1999	3.51 (3.33 to 3.63)	1996-1999	3.89 (3.69 to 4.02)	1998-2003	-0.03 (-0.34 to 0.56)	1996-1999	4.58 (4.46 to 4.70)	1998-2003	-0.57 (-0.92 to 0.03)	2003-2013	-0.27 (-0.36 to -0.18)
	3	1999-2004	2.61 (2.55 to 2.66)	1999-2004	3.03 (2.96 to 3.08)	2003-2007	-3.24 (-3.71 to -2.54)	1999-2004	2.87 (2.79 to 2.94)	2003-2007	-3.23 (-3.79 to -2.58)	2013-2021	2.10 (1.97 to 2.22)
	4	2004-2010	1.71 (1.66 to 1.76)	2004-2010	2.05 (2.00 to 2.10)	2007-2013	-4.67 (-5.08 to -4.43)	2004-2010	1.99 (1.90 to 2.06)	2007-2013	-4.82 (-5.13 to -4.57)		
	5	2010-2016	2.31 (2.24 to 2.36)	2010-2016	2.69 (2.64 to 2.74)	2013-2021	-0.87 (-1.06 to -0.69)	2010-2015	2.45 (2.33 to 2.59)	2013-2021	-0.50 (-0.70 to -0.33)		
	6	2016-2021	2.75 (2.68 to 2.86)	2016-2021	3.44 (3.37 to 3.51)			2015-2021	3.18 (3.12 to 3.26)				
High-middle SDI	0	1990-1993	2.69 (2.47 to 2.95)	1990-1995	2.13 (1.85 to 2.34)	1990-1995	0.56 (0.26 to 1.11)	1990-1996	2.17 (2.12 to 2.21)	1990-1995	0.93 (0.68 to 1.30)	1990-1994	1.52 (1.27 to 1.99)
	1	1993-2000	1.94 (1.88 to 2.00)	1995-2000	3.37 (3.21 to 3.60)	1995-2001	-0.47 (-1.11 to -0.19)	1996-1999	3.34 (3.25 to 3.42)	1995-1998	-1.34 (-1.65 to -0.71)	1994-2004	1.02 (0.82 to 1.10)
	2	2000-2006	0.87 (0.73 to 0.94)	2000-2009	1.24 (1.15 to 1.31)	2001-2004	0.62 (-1.28 to 1.00)	1999-2004	1.98 (1.93 to 2.04)	1998-2005	-0.29 (-0.45 to 0.17)	2004-2012	-0.15 (-0.33 to 0.96)
	3	2006-2011	1.21 (1.06 to 1.38)	2009-2021	1.90 (1.85 to 1.96)	2004-2013	-1.54 (-1.73 to -1.35)	2004-2010	1.19 (1.11 to 1.24)	2005-2008	-2.73 (-3.10 to -1.97)	2012-2017	1.65 (-0.20 to 2.10)
	4	2011-2014	1.99 (1.74 to 2.10)			2013-2016	3.41 (2.45 to 3.88)	2010-2016	1.50 (1.44 to 1.58)	2008-2013	-1.54 (-1.85 to -0.90)	2017-2021	1.06 (0.47 to 1.42)
	5	2014-2021	1.00 (0.94 to 1.05)			2016-2021	-0.51 (-1.04 to -0.14)	2016-2021	2.08 (2.02 to 2.16)	2013-2016	2.68 (1.84 to 3.13)		
	6									2016-2021	-0.49 (-1.01 to -0.17)		
Middle SDI	0	1990-1993	2.68 (2.56 to 2.84)	1990-1993	2.25 (2.11 to 2.52)	1990-1996	1.30 (1.10 to 1.59)	1990-1993	2.26 (2.06 to 2.47)	1990-1996	1.15 (0.86 to 1.40)	1990-1995	1.56 (1.44 to 1.67)
	1	1993-2006	1.00 (0.97 to 1.03)	1993-1996	1.49 (1.36 to 1.66)	1996-1999	-1.15 (-1.50 to -0.46)	1993-2001	1.56 (1.39 to 1.64)	1996-2000	-0.83 (-1.25 to 1.04)	1995-2001	0.32 (0.18 to 0.40)
	2	2006-2015	1.47 (1.04 to 1.55)	1996-1999	2.33 (2.21 to 2.45)	1999-2004	0.78 (0.53 to 1.36)	2001-2004	1.77 (1.27 to 1.87)	2000-2004	0.54 (-0.80 to 0.96)	2001-2004	1.18 (0.89 to 1.33)
	3	2015-2019	1.24 (1.05 to 1.51)	1999-2008	1.16 (1.12 to 1.20)	2004-2008	-0.68 (-1.08 to -0.27)	2004-2016	1.35 (1.18 to 1.76)	2004-2007	-1.21 (-1.47 to 0.46)	2004-2007	-0.22 (-0.38 to 0.06)
	4	2019-2021	1.75 (1.35 to 1.97)	2008-2013	1.68 (1.60 to 1.84)	2008-2021	-0.04 (-0.12 to 0.13)	2016-2019	1.95 (1.28 to 2.03)	2007-2012	-0.44 (-0.81 to 0.21)	2007-2013	0.38 (0.25 to 0.68)
	5			2013-2019	1.48 (1.37 to 1.53)			2019-2021	2.45 (2.08 to 2.68)	2012-2021	0.28 (0.15 to 0.55)	2013-2021	1.00 (0.91 to 1.13)
	6			2019-2021	2.82 (2.61 to 3.02)								
Low-middle SDI	0	1990-1996	1.67 (1.59 to 1.72)	1990-1993	2.49 (2.37 to 2.75)	1990-1996	1.53 (1.20 to 2.31)	1990-1993	3.31 (3.21 to 3.45)	1990-1995	1.33 (0.97 to 1.99)	1990-1995	1.81 (1.58 to 2.07)
	1	1996-1999	2.38 (2.17 to 2.47)	1993-1996	1.90 (1.77 to 2.06)	1996-2001	0.27 (-0.53 to 0.73)	1993-2002	1.73 (1.69 to 1.77)	1995-2005	0.53 (0.23 to 0.66)	1995-2000	0.72 (0.38 to 1.38)
	2	1999-2005	1.76 (1.68 to 1.82)	1996-1999	2.74 (2.54 to 2.86)	2001-2019	1.28 (1.22 to 1.40)	2002-2006	2.17 (2.03 to 2.34)	2005-2019	1.12 (1.05 to 1.22)	2000-2005	1.14 (0.88 to 1.88)
	3	2005-2010	2.45 (1.90 to 2.56)	1999-2005	2.00 (1.91 to 2.06)	2019-2021	-0.86 (-1.99 to 0.67)	2006-2009	3.26 (3.10 to 3.37)	2019-2021	-0.84 (-1.69 to 0.39)	2005-2018	1.70 (1.63 to 1.95)
	4	2010-2014	2.20 (2.06 to 2.38)	2005-2013	2.67 (2.64 to 2.73)			2009-2014	2.90 (2.73 to 2.96)			2018-2021	0.67 (-0.02 to 1.12)
	5	2014-2021	1.96 (1.89 to 2.00)	2013-2016	2.11 (2.02 to 2.29)			2014-2019	2.01 (1.86 to 2.07)				
	6			2016-2021	2.55 (2.48 to 2.69)			2019-2021	2.61 (2.32 to 2.78)				
Low SDI	0	1990-1996	1.37 (1.25 to 1.42)	1990-1993	2.40 (2.32 to 2.57)	1990-1995	1.36 (1.01 to 1.81)	1990-1994	2.64 (2.58 to 2.71)	1990-1996	0.91 (0.80 to 1.04)	1990-1996	1.23 (1.17 to 1.31)
	1	1996-1999	1.58 (1.39 to 1.64)	1993-1996	1.91 (1.82 to 2.05)	1995-2010	-0.20 (-0.26 to -0.14)	1994-2001	1.61 (1.56 to 1.65)	1996-2000	-0.55 (-0.79 to -0.16)	1996-2000	-0.03 (-0.20 to 0.07)
	2	1999-2005	1.34 (1.24 to 1.56)	1996-1999	2.27 (2.12 to 2.36)	2010-2014	1.85 (1.44 to 2.43)	2001-2006	1.96 (1.87 to 2.07)	2000-2003	0.04 (-0.49 to 0.21)	2000-2003	0.50 (0.31 to 0.62)
	3	2005-2014	1.79 (1.35 to 1.82)	1999-2005	1.88 (1.78 to 1.93)	2014-2021	-0.18 (-0.38 to -0.01)	2006-2014	2.45 (2.42 to 2.49)	2003-2007	-0.96 (-1.20 to -0.38)	2003-2007	-0.11 (-0.30 to 0.01)
	4	2014-2019	1.52 (1.42 to 1.83)	2005-2013	2.24 (2.21 to 2.29)			2014-2019	1.65 (1.57 to 1.70)	2007-2010	-0.26 (-0.59 to 0.53)	2007-2010	0.59 (0.19 to 1.04)
	5	2019-2021	1.74 (1.55 to 1.85)	2013-2019	1.90 (1.80 to 1.95)			2019-2021	2.12 (1.91 to 2.26)	2010-2014	1.08 (0.71 to 1.34)	2010-2014	1.49 (1.31 to 1.70)
	6			2019-2021	2.41 (2.18 to 2.52)					2014-2021	-0.07 (-0.18 to 0.03)	2014-2021	0.54 (0.46 to 0.61)
**Four world regions**													
Africa	0	1990-1996	1.91 (1.88 to 1.92)	1990-1992	2.79 (2.68 to 2.87)	1990-1995	0.92 (-0.17 to 1.40)	1990-1993	2.65 (2.60 to 2.72)	1990-1995	0.76 (-0.20 to 1.23)	1990-1995	1.18 (0.60 to 1.45)
	1	1996-2001	2.19 (2.12 to 2.21)	1992-2000	2.64 (2.60 to 2.66)	1995-2006	1.78 (1.64 to 2.21)	1993-1997	2.49 (2.44 to 2.53)	1995-2006	1.43 (1.26 to 1.90)	1995-2006	1.77 (1.68 to 2.00)
	2	2001-2004	2.33 (2.27 to 2.36)	2000-2004	2.94 (2.87 to 3.00)	2006-2015	0.52 (0.35 to 0.71)	1997-2001	2.70 (2.65 to 2.75)	2006-2015	0.30 (0.11 to 0.51)	2006-2015	0.99 (0.85 to 1.14)
	3	2004-2009	2.21 (2.16 to 2.23)	2004-2008	2.76 (2.69 to 2.83)	2015-2021	-0.89 (-1.29 to -0.61)	2001-2005	2.90 (2.87 to 2.94)	2015-2021	-1.03 (-1.47 to -0.73)	2015-2021	0.04 (-0.29 to 0.26)
	4	2009-2014	2.01 (1.98 to 2.03)	2008-2014	2.64 (2.60 to 2.66)			2005-2014	2.63 (2.62 to 2.64)				
	5	2014-2018	1.67 (1.64 to 1.71)	2014-2018	2.35 (2.31 to 2.41)			2014-2018	2.28 (2.26 to 2.31)				
	6	2018-2021	1.49 (1.42 to 1.54)	2018-2021	2.16 (2.08 to 2.21)			2018-2021	1.96 (1.92 to 2.00)				
America	0	1990-1996	1.83 (1.68 to 1.92)	1990-1996	2.19 (2.11 to 2.27)	1990-1995	2.27 (1.95 to 2.69)	1990-1996	2.38 (2.30 to 2.43)	1990-1995	2.17 (1.78 to 2.59)	1990-1995	2.26 (2.08 to 2.45)
	1	1996-2000	2.69 (2.55 to 2.94)	1996-1999	3.43 (3.21 to 3.56)	1995-1998	-1.29 (-1.75 to -0.25)	1996-1999	3.07 (2.88 to 3.17)	1995-1999	-1.83 (-2.60 to -1.09)	1995-1998	-0.22 (-0.48 to 0.27)
	2	2000-2005	2.07 (1.95 to 2.16)	1999-2004	2.60 (2.50 to 2.66)	1998-2004	0.24 (-1.79 to 1.06)	1999-2004	2.55 (2.44 to 2.61)	1999-2004	-0.11 (-0.63 to 0.83)	1998-2004	0.89 (0.71 to 1.30)
	3	2005-2010	1.19 (1.05 to 1.27)	2004-2010	1.53 (1.47 to 1.59)	2004-2013	-2.07 (-2.40 to -1.74)	2004-2010	1.54 (1.49 to 1.60)	2004-2012	-2.34 (-2.80 to -2.04)	2004-2012	-0.38 (-0.59 to -0.23)
	4	2010-2015	1.96 (1.88 to 2.12)	2010-2015	2.24 (2.16 to 2.37)	2013-2021	0.04 (-0.29 to 0.44)	2010-2015	2.18 (2.09 to 2.30)	2012-2021	0.28 (-0.04 to 0.66)	2012-2021	1.22 (1.08 to 1.39)
	5	2015-2019	1.13 (0.97 to 1.27)	2015-2019	1.61 (1.43 to 1.72)			2015-2019	1.55 (1.39 to 1.65)				
	6	2019-2021	2.62 (2.28 to 2.84)	2019-2021	2.74 (2.45 to 2.95)			2019-2021	2.64 (2.38 to 2.83)				
Asia	0	1990-1993	2.54 (2.31 to 2.71)	1990-1993	2.18 (2.02 to 2.51)	1990-1995	1.06 (0.84 to 1.37)	1990-1993	2.37 (2.22 to 2.65)	1990-1995	0.97 (0.81 to 1.18)	1990-1994	1.48 (1.35 to 1.61)

Location	Segment	Incidence		Prevalence		Mortality		YLD		YLL		DALY	
		Period	APC (95%CI)	Period	APC (95%CI)	Period	APC (95%CI)	Period	APC (95%CI)	Period	APC (95%CI)	Period	APC (95%CI)
	1	1993-1999	1.40 (1.33 to 1.50)	1993-1996	1.57 (1.42 to 1.75)	1995-2000	0.10 (-0.27 to 0.31)	1993-1996	1.52 (1.36 to 1.76)	1995-2004	0.20 (0.14 to 0.26)	1994-2003	0.93 (0.89 to 0.98)
	2	1999-2006	1.09 (0.97 to 1.14)	1996-1999	2.90 (2.75 to 3.04)	2000-2004	0.71 (0.41 to 1.00)	1996-1999	2.14 (1.91 to 2.27)	2004-2010	-0.75 (-0.86 to -0.66)	2003-2010	0.41 (0.31 to 0.49)
	3	2006-2015	1.74 (1.71 to 1.82)	1999-2006	1.20 (1.09 to 1.27)	2004-2011	-0.48 (-0.63 to -0.34)	1999-2016	1.69 (1.66 to 1.71)	2010-2014	0.26 (0.03 to 0.51)	2010-2015	0.97 (0.81 to 1.13)
	4	2015-2021	1.57 (1.45 to 1.64)	2006-2016	1.83 (1.24 to 1.87)	2011-2019	0.78 (0.69 to 0.94)	2016-2021	2.52 (2.42 to 2.62)	2014-2019	1.08 (0.96 to 1.32)	2015-2019	1.77 (1.64 to 1.99)
	5			2016-2019	2.12 (1.79 to 2.21)	2019-2021	-0.66 (-1.23 to 0.08)			2019-2021	-0.68 (-1.11 to -0.09)	2019-2021	0.96 (0.67 to 1.29)
	6			2019-2021	2.85 (2.49 to 3.08)								
Europe	0	1990-1995	1.49 (1.39 to 1.57)	1990-1995	1.97 (1.77 to 2.10)	1990-1994	-0.21 (-0.66 to 0.29)	1990-1995	2.18 (2.10 to 2.25)	1990-1995	-0.16 (-0.48 to 0.29)	1990-1994	0.95 (0.71 to 1.31)
	1	1995-2000	2.43 (1.60 to 2.50)	1995-2000	2.88 (2.74 to 3.06)	1994-2000	-2.25 (-2.87 to -2.01)	1995-1999	3.17 (3.08 to 3.27)	1995-1998	-3.53 (-3.92 to -2.53)	1994-2007	0.17 (0.03 to 0.22)
	2	2000-2008	1.50 (1.41 to 2.42)	2000-2011	1.82 (1.77 to 1.86)	2000-2003	-0.26 (-1.05 to 0.15)	1999-2002	2.17 (2.02 to 2.44)	1998-2007	-2.02 (-2.48 to -1.67)	2007-2012	0.65 (0.34 to 1.28)
	3	2008-2011	1.67 (1.46 to 1.75)	2011-2021	2.54 (2.49 to 2.60)	2003-2006	-2.53 (-2.97 to -1.79)	2002-2011	1.74 (1.69 to 1.77)	2007-2013	-1.19 (-1.59 to -0.25)	2012-2016	2.94 (2.56 to 3.47)
	4	2011-2014	2.53 (2.33 to 2.65)			2006-2013	-1.07 (-1.28 to -0.43)	2011-2014	2.80 (2.63 to 2.90)	2013-2016	5.09 (3.68 to 5.73)	2016-2021	1.49 (1.15 to 1.74)
	5	2014-2019	1.99 (1.83 to 2.05)			2013-2016	4.78 (3.82 to 5.26)	2014-2019	2.08 (1.92 to 2.14)	2016-2021	0.04 (-0.58 to 0.47)		
	6	2019-2021	2.44 (2.15 to 2.60)			2016-2021	0.03 (-0.39 to 0.36)	2019-2021	2.57 (2.29 to 2.74)				
**Six WHO regions**													
African Region	0	1990-1996	1.44 (1.39 to 1.47)	1990-2000	2.16 (2.12 to 2.18)	1990-1995	1.01 (0.10 to 1.45)	1990-1993	2.24 (2.17 to 2.37)	1990-1995	0.73 (0.03 to 1.00)	1990-1995	1.06 (0.49 to 1.27)
	1	1996-2001	1.72 (1.63 to 1.77)	2000-2005	2.49 (2.41 to 2.62)	1995-1998	2.25 (1.23 to 2.62)	1993-1997	2.01 (1.93 to 2.06)	1995-1998	1.88 (1.32 to 2.18)	1995-1998	1.90 (1.44 to 2.13)
	2	2001-2004	1.94 (1.84 to 1.98)	2005-2011	2.08 (1.97 to 2.13)	1998-2006	1.16 (0.46 to 1.30)	1997-2001	2.19 (2.14 to 2.28)	1998-2006	0.83 (0.56 to 0.96)	1998-2006	1.18 (0.95 to 1.28)
	3	2004-2015	1.62 (1.61 to 1.63)	2011-2014	2.44 (2.28 to 2.50)	2006-2015	0.10 (-0.05 to 0.26)	2001-2004	2.62 (2.50 to 2.68)	2006-2016	-0.12 (-0.22 to 0.78)	2006-2015	0.50 (0.37 to 0.63)
	4	2015-2019	1.02 (0.96 to 1.05)	2014-2021	1.81 (1.77 to 1.84)	2015-2021	-0.80 (-1.12 to -0.58)	2004-2011	2.10 (2.07 to 2.13)	2016-2019	-1.56 (-1.89 to -0.10)	2015-2021	-0.16 (-0.43 to 0.02)
	5	2019-2021	1.32 (1.20 to 1.41)					2011-2014	2.48 (2.40 to 2.53)	2019-2021	0.01 (-1.01 to 0.58)		
	6							2014-2021	1.70 (1.67 to 1.72)				
Region of the Americas	0	1990-1996	1.83 (1.68 to 1.92)	1990-1996	2.19 (2.11 to 2.27)	1990-1995	2.27 (1.95 to 2.69)	1990-1996	2.38 (2.30 to 2.43)	1990-1995	2.17 (1.78 to 2.59)	1990-1995	2.26 (2.08 to 2.45)
	1	1996-2000	2.69 (2.55 to 2.94)	1996-1999	3.43 (3.21 to 3.56)	1995-1998	-1.29 (-1.75 to -0.25)	1996-1999	3.07 (2.88 to 3.17)	1995-1999	-1.83 (-2.60 to -1.09)	1995-1998	-0.22 (-0.48 to 0.27)
	2	2000-2005	2.07 (1.95 to 2.16)	1999-2004	2.60 (2.50 to 2.66)	1998-2004	0.24 (-1.79 to 1.06)	1999-2004	2.55 (2.44 to 2.61)	1999-2004	-0.11 (-0.63 to 0.83)	1998-2004	0.89 (0.71 to 1.30)
	3	2005-2010	1.19 (1.05 to 1.27)	2004-2010	1.53 (1.47 to 1.59)	2004-2013	-2.07 (-2.40 to -1.74)	2004-2010	1.54 (1.49 to 1.60)	2004-2012	-2.34 (-2.80 to -2.04)	2004-2012	-0.38 (-0.59 to -0.23)
	4	2010-2015	1.96 (1.88 to 2.12)	2010-2015	2.24 (2.16 to 2.37)	2013-2021	0.04 (-0.29 to 0.44)	2010-2015	2.18 (2.09 to 2.30)	2012-2021	0.28 (-0.04 to 0.66)	2012-2021	1.22 (1.08 to 1.39)
	5	2015-2019	1.13 (0.97 to 1.27)	2015-2019	1.61 (1.43 to 1.72)			2015-2019	1.55 (1.39 to 1.65)				
	6	2019-2021	2.62 (2.28 to 2.84)	2019-2021	2.74 (2.45 to 2.95)			2019-2021	2.64 (2.38 to 2.83)				
South-East Asia Region	0	1990-1996	1.54 (1.42 to 1.60)	1990-1993	2.71 (2.52 to 2.99)	1990-1995	1.77 (0.84 to 3.18)	1990-1992	4.42 (4.08 to 4.76)	1990-1996	1.09 (0.65 to 1.62)	1990-1995	1.75 (1.35 to 2.24)
	1	1996-1999	1.98 (1.76 to 2.08)	1993-1996	1.57 (1.40 to 1.89)	1995-2004	-0.45 (-1.34 to 0.17)	1992-1995	2.27 (2.03 to 2.45)	1996-2005	-0.53 (-0.86 to 0.67)	1995-2005	0.07 (-0.15 to 0.22)
	2	1999-2005	1.19 (1.13 to 1.23)	1996-1999	2.12 (1.53 to 2.28)	2004-2007	2.52 (-0.51 to 3.18)	1995-2001	0.76 (0.62 to 0.85)	2005-2008	1.54 (-0.65 to 1.91)	2005-2021	1.76 (1.67 to 1.85)
	3	2005-2014	2.10 (2.06 to 2.14)	1999-2006	1.59 (1.45 to 2.51)	2007-2010	-0.04 (-0.63 to 1.24)	2001-2006	1.64 (1.53 to 1.80)	2008-2011	0.15 (-0.18 to 1.17)		
	4	2014-2019	1.81 (1.64 to 1.90)	2006-2012	2.52 (2.29 to 2.70)	2010-2019	1.97 (1.79 to 2.86)	2006-2014	3.03 (2.97 to 3.09)	2011-2019	1.80 (1.66 to 2.17)		
	5	2019-2021	2.27 (1.98 to 2.45)	2012-2019	2.09 (2.00 to 2.17)	2019-2021	-1.38 (-3.13 to 0.66)	2014-2019	1.61 (1.51 to 1.70)	2019-2021	-1.15 (-2.21 to 0.22)		
	6			2019-2021	3.97 (3.63 to 4.21)			2019-2021	3.62 (3.28 to 3.91)				
European Region	0	1990-1995	1.56 (1.41 to 1.65)	1990-1995	2.02 (1.93 to 2.11)	1990-1995	-0.09 (-0.45 to 0.52)	1990-1995	2.22 (2.15 to 2.29)	1990-1995	0.37 (0.05 to 0.78)	1990-1994	1.28 (1.02 to 1.60)
	1	1995-2000	2.49 (2.05 to 2.57)	1995-1999	2.97 (2.84 to 3.13)	1995-1998	-2.62 (-3.02 to -1.75)	1995-1999	3.19 (3.10 to 3.28)	1995-1998	-3.41 (-3.79 to -2.41)	1994-2006	0.27 (0.10 to 0.33)
	2	2000-2011	1.66 (1.62 to 2.46)	1999-2003	2.24 (2.06 to 2.45)	1998-2013	-1.21 (-1.30 to -1.07)	1999-2002	2.28 (2.13 to 2.60)	1998-2007	-1.78 (-2.18 to -1.48)	2006-2012	0.71 (0.45 to 1.23)
	3	2011-2014	2.58 (1.64 to 2.68)	2003-2010	1.81 (1.73 to 1.87)	2013-2016	4.98 (3.93 to 5.52)	2002-2011	1.84 (1.80 to 1.87)	2007-2013	-0.92 (-1.30 to -0.08)	2012-2016	3.00 (2.62 to 3.57)
	4	2014-2019	1.95 (1.78 to 2.57)	2010-2021	2.47 (2.44 to 2.50)	2016-2021	-0.04 (-0.63 to 0.42)	2011-2014	2.78 (2.50 to 2.88)	2013-2016	5.09 (3.87 to 5.68)	2016-2021	1.42 (1.09 to 1.67)
	5	2019-2021	2.34 (2.01 to 2.51)					2014-2019	2.07 (1.92 to 2.16)	2016-2021	-0.04 (-0.57 to 0.35)		
	6							2019-2021	2.49 (2.20 to 2.65)				
Eastern Mediterranean Region	0	1990-1996	2.86 (2.75 to 2.94)	1990-1997	3.38 (3.28 to 3.44)	1990-1997	2.19 (2.07 to 2.42)	1990-1996	3.17 (3.02 to 3.24)	1990-1996	2.43 (2.24 to 2.67)	1990-1996	2.66 (2.56 to 2.79)
	1	1996-2010	3.28 (3.25 to 3.30)	1997-2009	3.60 (3.53 to 3.63)	1997-2000	1.11 (0.82 to 1.65)	1996-2000	3.56 (3.26 to 3.72)	1996-2000	1.13 (0.63 to 1.52)	1996-2001	2.06 (1.74 to 2.20)
	2	2010-2015	2.46 (2.32 to 3.19)	2009-2012	2.84 (2.77 to 3.67)	2000-2005	2.63 (2.31 to 3.17)	2000-2009	3.78 (3.74 to 3.88)	2000-2005	2.32 (2.02 to 2.91)	2001-2004	3.20 (2.86 to 3.39)
	3	2015-2018	2.83 (2.61 to 2.94)	2012-2015	2.54 (2.43 to 2.70)	2005-2015	1.02 (0.91 to 1.13)	2009-2012	2.71 (2.61 to 3.80)	2005-2015	0.82 (0.71 to 0.95)	2004-2009	2.09 (1.92 to 2.25)
	4	2018-2021	1.55 (1.37 to 1.69)	2015-2018	3.12 (2.99 to 3.22)	2015-2021	-0.05 (-0.37 to 0.18)	2012-2015	2.33 (2.21 to 2.50)	2015-2021	-0.20 (-0.56 to 0.05)	2009-2016	1.52 (1.39 to 1.65)
	5			2018-2021	2.05 (1.92 to 2.17)			2015-2018	3.24 (3.10 to 3.36)			2016-2021	0.97 (0.69 to 1.13)
	6							2018-2021	1.83 (1.68 to 1.96)				
Western Pacific Region	0	1990-1993	2.87 (2.50 to 3.15)	1990-1996	1.50 (1.39 to 1.61)	1990-1997	-0.65 (-0.82 to -0.44)	1990-1996	1.26 (1.16 to 1.35)	1990-1997	-0.51 (-0.70 to -0.32)	1990-1996	0.36 (0.12 to 0.49)
	1	1993-1999	1.18 (1.05 to 1.32)	1996-1999	3.64 (3.47 to 3.82)	1997-2004	1.22 (1.07 to 1.37)	1996-1999	3.31 (2.96 to 3.47)	1997-2004	0.99 (0.86 to 1.14)	1996-1999	1.98 (1.50 to 2.20)
	2	1999-2007	0.43 (0.31 to 0.50)	1999-2008	0.75 (0.68 to 0.81)	2004-2007	-3.07 (-3.34 to -2.68)	1999-2004	1.71 (1.58 to 1.87)	2004-2007	-3.38 (-3.63 to -3.04)	1999-2004	1.38 (-1.09 to 1.52)
	3	2007-2021	1.17 (1.14 to 1.20)	2008-2016	1.46 (1.38 to 1.54)	2007-2013	-1.72 (-1.89 to -1.46)	2004-2011	0.76 (0.70 to 1.10)	2007-2013	-2.08 (-2.20 to -1.85)	2004-2008	-1.00 (-1.28 to -0.29)
	4			2016-2021	2.31 (2.17 to 2.52)	2013-2021	-0.53 (-0.71 to -0.32)	2011-2015	0.48 (0.29 to 0.63)	2013-2021	-0.31 (-0.46 to -0.15)	2008-2015	-0.25 (-0.38 to 0.19)
	5							2015-2019	3.03 (2.91 to 3.21)			2015-2021	1.76 (1.58 to 2.00)
	6							2019-2021	2.09 (1.74 to 2.47)				
**Seven super regions**													
Southeast Asia, East Asia, and Oceania	0	1990-1993	3.38 (3.21 to 3.58)	1990-1996	1.76 (1.32 to 1.92)	1990-2004	1.14 (1.08 to 1.20)	1990-2004	1.75 (1.70 to 1.80)	1990-2004	0.86 (0.80 to 0.92)	1990-2004	1.24 (1.20 to 1.28)
	1	1993-1999	0.95 (0.87 to 1.05)	1996-1999	2.83 (2.26 to 3.08)	2004-2008	-2.46 (-2.75 to -2.22)	2004-2016	0.89 (0.83 to 0.95)	2004-2008	-2.89 (-3.19 to -2.66)	2004-2008	-1.20 (-1.44 to -1.01)
	2	1999-2005	0.50 (0.30 to 0.57)	1999-2007	0.69 (0.53 to 0.79)	2008-2013	-1.42 (-1.74 to -1.03)	2016-2021	2.92 (2.76 to 3.06)	2008-2013	-1.69 (-1.99 to -1.29)	2008-2015	-0.31 (-0.44 to -0.15)
	3	2005-2010	1.10 (0.75 to 1.24)	2007-2019	1.51 (1.45 to 1.57)	2013-2021	-0.50 (-0.65 to -0.24)			2013-2021	-0.36 (-0.53 to -0.16)	2015-2021	1.32 (1.15 to 1.50)
	4	2010-2014	1.45 (1.31 to 1.61)	2019-2021	3.42 (2.69 to 3.81)								

Location	Segment	Incidence Period	Incidence APC (95%CI)	Prevalence Period	Prevalence APC (95%CI)	Mortality Period	Mortality APC (95%CI)	YLD Period	YLD APC (95%CI)	YLL Period	YLL APC (95%CI)	DALY Period	DALY APC (95%CI)
	5	2014-2019	0.85 (0.64 to 0.93)										
	6	2019-2021	1.52 (1.18 to 1.73)										
Central Europe, Eastern Europe, and Central Asia	0	1990-1995	1.86 (1.80 to 1.93)	1990-1992	2.03 (1.87 to 2.27)	1990-1994	5.64 (4.81 to 6.51)	1990-1992	1.97 (1.81 to 2.22)	1990-1994	6.39 (5.51 to 7.38)	1990-1994	4.00 (3.66 to 4.36)
	1	1995-1999	2.98 (2.87 to 3.07)	1992-1995	2.37 (2.30 to 2.99)	1994-2001	-2.04 (-2.71 to -1.67)	1992-1995	2.35 (2.27 to 3.00)	1994-2001	-2.58 (-3.37 to -2.17)	1994-2001	0.44 (0.05 to 0.63)
	2	1999-2003	2.36 (2.25 to 2.53)	1995-2000	2.95 (2.24 to 3.05)	2001-2012	0.38 (0.10 to 0.76)	1995-2000	2.98 (2.26 to 3.09)	2001-2012	-0.24 (-0.54 to 0.15)	2001-2012	1.25 (1.12 to 1.44)
	3	2003-2009	1.95 (1.88 to 2.04)	2000-2004	2.26 (1.99 to 2.38)	2012-2016	11.94 (10.75 to 13.01)	2000-2004	2.26 (2.04 to 2.39)	2012-2016	10.63 (9.49 to 11.71)	2012-2017	4.57 (4.24 to 5.04)
	4	2009-2018	1.71 (1.68 to 1.77)	2004-2010	2.01 (1.74 to 2.08)	2016-2021	2.36 (1.16 to 3.40)	2004-2011	2.05 (1.74 to 2.09)	2016-2021	1.83 (0.63 to 2.88)	2017-2021	1.21 (0.78 to 1.63)
	5	2018-2021	1.49 (1.27 to 1.62)	2010-2021	1.78 (1.74 to 1.82)			2011-2021	1.74 (1.71 to 1.77)				
South Asia	0	1990-1995	1.34 (1.02 to 1.50)	1990-1996	1.89 (1.61 to 2.03)	1990-1996	2.07 (1.06 to 3.96)	1990-1993	3.90 (3.66 to 4.18)	1990-1996	1.36 (0.63 to 2.76)	1990-1995	2.06 (1.61 to 2.60)
	1	1995-1999	2.40 (1.74 to 2.70)	1996-1999	3.12 (2.63 to 3.35)	1996-2001	-1.87 (-4.38 to -0.46)	1993-2005	1.21 (1.16 to 1.25)	1996-2004	-0.67 (-1.85 to -0.21)	1995-2004	-0.01 (-0.33 to 0.18)
	2	1999-2005	1.14 (0.92 to 2.33)	1999-2006	1.57 (1.35 to 1.68)	2001-2021	1.81 (1.64 to 2.03)	2005-2014	3.18 (3.12 to 3.25)	2004-2021	1.81 (1.66 to 1.97)	2004-2019	2.19 (2.10 to 2.33)
	3	2005-2010	2.26 (1.14 to 2.63)	2006-2011	2.69 (2.49 to 3.11)			2014-2021	1.55 (1.45 to 1.66)			2019-2021	-0.37 (-1.59 to 1.15)
	4	2010-2017	1.87 (1.79 to 2.29)	2011-2021	2.10 (2.01 to 2.17)								
	5	2017-2021	1.55 (1.12 to 1.76)										
High-income	0	1990-1996	1.75 (1.68 to 1.83)	1990-1993	1.88 (1.57 to 2.14)	1990-1993	-0.38 (-0.73 to 0.16)	1990-1993	2.13 (1.88 to 2.25)	1990-1994	-0.61 (-0.88 to -0.28)	1990-1997	0.58 (0.50 to 0.66)
	1	1996-1999	3.53 (3.30 to 3.66)	1993-1996	2.33 (2.18 to 3.87)	1993-1998	-1.59 (-2.06 to -1.38)	1993-1996	2.66 (2.53 to 2.77)	1994-1998	-2.00 (-2.41 to -1.68)	1997-2003	1.29 (1.19 to 1.40)
	2	1999-2004	2.45 (2.35 to 2.52)	1996-1999	3.79 (2.94 to 3.91)	1998-2003	-0.46 (-0.68 to -0.12)	1996-1999	4.52 (4.42 to 4.63)	1998-2003	-0.90 (-1.12 to -0.53)	2003-2013	-0.31 (-0.35 to -0.26)
	3	2004-2010	1.54 (1.46 to 1.59)	1999-2004	2.95 (1.90 to 3.00)	2003-2009	-3.59 (-3.78 to -3.39)	1999-2004	2.79 (2.73 to 2.85)	2003-2008	-3.54 (-3.80 to -3.25)	2013-2021	1.76 (1.68 to 1.83)
	4	2010-2019	2.30 (2.24 to 2.33)	2004-2010	1.92 (1.84 to 2.60)	2009-2013	-5.10 (-5.49 to -4.77)	2004-2010	1.89 (1.81 to 1.94)	2008-2013	-5.09 (-5.35 to -4.86)		
	5	2019-2021	3.00 (2.64 to 3.17)	2010-2016	2.65 (2.55 to 2.83)	2013-2021	-1.57 (-1.71 to -1.43)	2010-2015	2.49 (2.36 to 2.61)	2013-2021	-1.27 (-1.40 to -1.14)		
	6			2016-2021	3.08 (2.98 to 3.24)			2015-2021	2.82 (2.76 to 2.92)				
Latin America and Caribbean	0	1990-1993	1.58 (1.48 to 1.69)	1990-1993	1.97 (1.73 to 2.33)	1990-1996	0.47 (0.19 to 0.81)	1990-1993	1.98 (1.55 to 2.66)	1990-1995	0.95 (0.25 to 1.53)	1990-1995	1.16 (0.92 to 1.42)
	1	1993-2001	0.57 (0.53 to 0.61)	1993-2001	0.99 (0.89 to 1.04)	1996-1999	-4.15 (-4.70 to -3.13)	1993-2019	0.98 (0.95 to 1.00)	1995-2000	-3.16 (-3.95 to 0.88)	1995-2000	-1.80 (-2.21 to -1.39)
	2	2001-2005	1.06 (0.95 to 1.20)	2001-2004	1.44 (1.21 to 1.57)	1999-2004	0.33 (-0.06 to 1.18)	2019-2021	2.05 (1.16 to 2.42)	2000-2004	0.31 (-3.32 to 1.39)	2000-2004	0.72 (-0.00 to 1.39)
	3	2005-2010	0.58 (0.45 to 0.65)	2004-2010	0.81 (0.59 to 0.88)	2004-2007	-2.30 (-2.82 to -1.26)			2004-2007	-2.79 (-3.37 to 0.45)	2004-2007	-1.36 (-1.73 to -0.48)
	4	2010-2015	0.94 (0.87 to 1.09)	2010-2019	1.01 (0.97 to 1.08)	2007-2021	-0.39 (-0.53 to -0.20)			2007-2015	-0.58 (-2.72 to -0.13)	2007-2015	0.07 (-0.51 to 0.32)
	5	2015-2019	0.59 (0.46 to 0.68)	2019-2021	2.29 (1.86 to 2.56)					2015-2021	0.40 (-0.32 to 2.11)	2015-2021	0.76 (0.44 to 1.62)
	6	2019-2021	2.28 (2.09 to 2.47)										
North Africa and Middle East	0	1990-2001	2.64 (2.59 to 2.70)	1990-1994	3.35 (3.26 to 3.44)	1990-1992	-2.89 (-3.59 to -1.67)	1990-1993	3.23 (3.13 to 3.37)	1990-2001	-0.42 (-0.79 to -0.17)	1990-1992	0.23 (-0.10 to 0.80)
	1	2001-2006	2.88 (2.59 to 2.94)	1994-2001	2.91 (2.82 to 2.95)	1992-2001	-0.38 (-0.53 to -0.17)	1993-2001	2.92 (2.90 to 2.95)	2001-2006	1.64 (-0.58 to 2.24)	1992-2001	0.97 (0.86 to 1.39)
	2	2006-2011	3.04 (2.87 to 3.09)	2001-2011	3.25 (2.99 to 3.28)	2001-2006	1.86 (1.32 to 2.27)	2001-2004	3.65 (3.57 to 3.71)	2006-2009	2.95 (0.99 to 3.47)	2001-2006	2.45 (0.73 to 2.79)
	3	2011-2014	3.22 (3.11 to 3.28)	2011-2014	3.80 (3.22 to 3.85)	2006-2009	4.11 (3.46 to 4.55)	2004-2011	3.20 (3.17 to 3.22)	2009-2015	0.83 (-0.46 to 1.21)	2006-2009	3.00 (2.03 to 3.25)
	4	2014-2018	2.84 (2.75 to 2.91)	2014-2018	3.01 (2.97 to 3.85)	2009-2012	0.42 (0.01 to 0.91)	2011-2014	3.74 (3.67 to 3.80)	2015-2021	-0.76 (-1.51 to -0.31)	2009-2015	2.07 (0.88 to 2.24)
	5	2018-2021	2.37 (2.25 to 2.47)	2018-2021	2.82 (2.65 to 2.95)	2012-2015	2.25 (1.57 to 2.66)	2014-2018	2.82 (2.77 to 2.90)			2015-2021	1.03 (0.75 to 1.33)
	6					2015-2021	-0.71 (-1.01 to -0.49)	2018-2021	2.57 (2.44 to 2.66)				
Sub-Saharan Africa	0	1990-1996	1.28 (1.23 to 1.32)	1990-2000	1.99 (1.96 to 2.02)	1990-1995	1.13 (0.59 to 1.35)	1990-2000	1.96 (1.92 to 1.99)	1990-1995	0.82 (0.29 to 1.05)	1990-1995	1.07 (0.60 to 1.25)
	1	1996-2001	1.57 (1.48 to 1.63)	2000-2005	2.36 (2.29 to 2.46)	1995-1998	2.27 (1.73 to 2.55)	2000-2005	2.37 (2.28 to 2.54)	1995-1998	1.95 (1.34 to 2.19)	1995-1998	1.92 (1.52 to 2.13)
	2	2001-2004	1.81 (1.70 to 1.86)	2005-2010	1.82 (1.73 to 1.90)	1998-2003	1.23 (0.85 to 1.49)	2005-2011	1.88 (1.75 to 1.94)	1998-2004	0.82 (0.56 to 1.12)	1998-2005	1.12 (0.89 to 1.24)
	3	2004-2015	1.48 (1.46 to 1.49)	2010-2015	2.21 (2.15 to 2.29)	2003-2009	0.53 (0.26 to 0.75)	2011-2014	2.46 (2.25 to 2.56)	2004-2009	0.19 (-0.04 to 0.75)	2005-2016	0.43 (0.35 to 0.74)
	4	2015-2019	0.80 (0.74 to 0.83)	2015-2019	1.43 (1.32 to 1.49)	2009-2016	-0.07 (-0.21 to 0.23)	2014-2021	1.49 (1.44 to 1.54)	2009-2016	-0.22 (-0.38 to -0.04)	2016-2019	-0.82 (-1.09 to 0.45)
	5	2019-2021	1.28 (1.13 to 1.38)	2019-2021	1.90 (1.70 to 2.03)	2016-2019	-1.27 (-1.54 to -0.17)			2016-2019	-1.53 (-1.82 to -1.08)	2019-2021	0.49 (-0.38 to 0.97)
	6					2019-2021	0.01 (-0.78 to 0.49)			2019-2021	-0.04 (-0.74 to 0.46)		
**21 regions**													
Andean Latin America	0	1990-1993	3.11 (2.90 to 3.25)	1990-1992	3.48 (3.29 to 3.68)	1990-1995	3.16 (1.76 to 5.83)	1990-1993	3.42 (3.31 to 3.56)	1990-1995	3.12 (1.54 to 5.92)	1990-1995	2.90 (1.93 to 4.51)
	1	1993-2001	2.14 (2.08 to 2.18)	1992-1995	2.68 (2.50 to 2.77)	1995-2011	0.15 (-0.11 to 1.16)	1993-1996	2.47 (2.32 to 2.60)	1995-2021	-0.36 (-0.55 to -0.22)	1995-2021	0.63 (0.50 to 0.72)
	2	2001-2005	2.55 (2.42 to 2.69)	1995-2001	1.98 (1.94 to 2.01)	2011-2021	-0.78 (-4.67 to -0.21)	1996-2000	1.90 (1.79 to 1.97)				
	3	2005-2014	2.01 (1.98 to 2.04)	2001-2004	2.88 (2.77 to 2.93)			2000-2009	2.69 (2.66 to 2.73)				
	4	2014-2021	1.23 (1.18 to 1.27)	2004-2014	2.49 (2.46 to 2.50)			2009-2014	2.44 (2.36 to 2.52)				
	5			2014-2017	1.74 (1.66 to 2.04)			2014-2017	1.72 (1.59 to 1.99)				
	6			2017-2021	1.46 (1.33 to 1.52)			2017-2021	1.31 (1.18 to 1.38)				
Australasia	0	1990-1992	0.59 (0.39 to 0.86)	1990-1995	0.95 (0.72 to 1.12)	1990-1995	2.17 (1.36 to 2.88)	1990-1995	0.24 (-0.04 to 0.45)	1990-1996	1.46 (0.70 to 2.29)	1990-1996	1.05 (0.74 to 1.37)
	1	1992-1995	1.22 (1.12 to 2.02)	1995-2004	2.20 (2.15 to 2.28)	1995-2001	-2.94 (-3.79 to -2.14)	1995-2000	2.28 (2.04 to 2.63)	1996-2001	-4.11 (-6.13 to -3.01)	1996-2002	-1.19 (-1.65 to -0.87)
	2	1995-2000	2.11 (1.93 to 2.22)	2004-2007	1.19 (1.06 to 1.54)	2001-2008	0.99 (-0.71 to 2.12)	2000-2006	0.68 (0.52 to 0.86)	2001-2010	-0.24 (-0.74 to 0.65)	2002-2010	1.02 (0.78 to 1.37)
	3	2000-2018	1.66 (1.64 to 1.68)	2007-2021	2.24 (2.20 to 2.28)	2008-2011	-2.80 (-4.27 to 0.42)	2006-2010	3.60 (3.29 to 3.92)	2010-2014	-8.50 (-10.70 to -6.61)	2010-2014	-1.71 (-2.63 to -0.94)
	4	2018-2021	2.06 (1.86 to 2.33)			2011-2014	-8.56 (-9.55 to -6.10)	2010-2021	2.18 (2.09 to 2.25)	2014-2021	-0.09 (-0.87 to 0.89)	2014-2021	1.43 (1.07 to 1.91)
	5					2014-2021	-0.61 (-1.16 to 0.12)						
Caribbean	0	1990-1994	1.85 (1.71 to 1.99)	1990-1994	2.38 (2.23 to 2.50)	1990-1995	-0.31 (-0.75 to 0.27)	1990-1993	2.55 (2.24 to 2.77)	1990-1996	-0.32 (-0.63 to 0.03)	1990-1995	0.65 (0.42 to 0.91)
	1	1994-2000	1.12 (0.98 to 1.20)	1994-2001	1.59 (1.45 to 1.64)	1995-2002	-2.42 (-3.05 to -2.12)	1993-2021	1.61 (1.59 to 1.62)	1996-2002	-2.51 (-3.18 to -2.19)	1995-2002	-0.91 (-1.14 to -0.77)
	2	2000-2021	1.37 (1.36 to 1.39)	2001-2004	1.83 (1.67 to 1.90)	2002-2005	1.61 (0.28 to 2.31)			2002-2005	1.13 (0.00 to 1.73)	2002-2005	1.32 (0.63 to 1.64)
	3			2004-2021	1.64 (1.61 to 1.65)	2005-2015	-1.57 (-2.32 to -1.36)			2005-2013	-1.66 (-2.48 to -1.42)	2005-2013	-0.25 (-0.61 to -0.11)
	4					2015-2021	0.04 (-0.69 to 1.50)			2013-2021	0.00 (-0.42 to 0.63)	2013-2021	0.78 (0.59 to 1.01)
Central Asia	0	1990-1995	2.71 (2.37 to 2.90)	1990-1995	3.21 (3.03 to 3.31)	1990-1994	12.16 (11.20 to 13.26)	1990-1992	2.93 (2.74 to 3.21)	1990-1994	12.69 (11.53 to 14.05)	1990-1994	8.55 (8.07 to 9.18)

Location	Segment	Incidence		Prevalence		Mortality		YLD		YLL		DALY	
		Period	APC (95%CI)	Period	APC (95%CI)	Period	APC (95%CI)	Period	APC (95%CI)	Period	APC (95%CI)	Period	APC (95%CI)
	1	1995-2001	3.76 (3.37 to 3.95)	1995-2001	3.89 (3.51 to 3.96)	1994-2005	1.23 (0.29 to 1.50)	1992-1996	3.41 (3.33 to 3.96)	1994-2005	0.84 (-0.24 to 1.15)	1994-2015	2.11 (2.04 to 2.18)
	2	2001-2004	4.40 (2.92 to 4.56)	2001-2004	4.12 (3.96 to 4.20)	2005-2015	1.91 (1.57 to 3.00)	1996-2005	3.99 (3.95 to 4.03)	2005-2015	1.55 (0.62 to 2.84)	2015-2021	-0.64 (-1.28 to -0.12)
	3	2004-2015	2.58 (2.53 to 2.63)	2004-2007	3.04 (2.87 to 3.13)	2015-2021	-3.11 (-4.12 to -2.24)	2005-2014	2.69 (2.66 to 2.72)	2015-2021	-2.84 (-4.15 to -1.88)		
	4	2015-2021	1.37 (1.27 to 1.46)	2007-2014	2.65 (2.61 to 2.69)			2014-2017	1.96 (1.81 to 2.25)				
	5			2014-2017	1.96 (1.84 to 2.11)			2017-2021	1.32 (1.20 to 1.41)				
	6			2017-2021	1.40 (1.31 to 1.46)								
Central Europe	0	1990-1995	0.73 (0.38 to 0.94)	1990-1995	1.04 (0.57 to 1.27)	1990-2002	-1.31 (-1.61 to -1.04)	1990-1995	1.02 (0.67 to 1.22)	1990-2002	-2.03 (-2.29 to -1.79)	1990-1996	-0.26 (-1.13 to 0.13)
	1	1995-1999	2.65 (2.35 to 3.12)	1995-2000	2.68 (1.38 to 3.09)	2002-2008	1.53 (0.74 to 3.43)	1995-2000	2.79 (2.54 to 3.13)	2002-2008	0.81 (0.20 to 2.41)	1996-2021	0.77 (0.71 to 0.84)
	2	1999-2004	1.60 (1.29 to 1.91)	2000-2004	1.49 (1.25 to 2.93)	2008-2011	-1.70 (-2.60 to 0.14)	2000-2011	1.40 (1.33 to 1.56)	2008-2012	-1.75 (-3.04 to -0.64)		
	3	2004-2021	1.11 (1.06 to 1.14)	2004-2021	1.23 (0.79 to 1.27)	2011-2021	1.00 (0.56 to 2.31)	2011-2021	1.19 (1.01 to 1.26)	2012-2021	0.72 (0.31 to 1.60)		
Central Latin America	0	1990-1994	2.26 (2.03 to 2.48)	1990-1994	2.66 (2.33 to 3.15)	1990-1996	0.50 (-0.13 to 1.19)	1990-1994	2.82 (2.54 to 3.10)	1990-1996	0.71 (-0.01 to 1.48)	1990-1995	1.70 (1.17 to 2.21)
	1	1994-2001	0.02 (-0.12 to 0.14)	1994-1999	0.47 (0.01 to 0.70)	1996-2000	-5.07 (-6.68 to -3.88)	1994-2000	0.35 (0.07 to 0.52)	1996-2000	-5.62 (-7.33 to -4.27)	1995-2000	-2.90 (-3.70 to -2.36)
	2	2001-2014	0.72 (0.68 to 0.79)	1999-2012	0.96 (0.90 to 1.11)	2000-2004	2.17 (0.84 to 4.12)	2000-2013	0.94 (0.88 to 1.04)	2000-2004	2.07 (0.62 to 4.09)	2000-2004	1.47 (0.60 to 2.71)
	3	2014-2019	0.10 (-0.24 to 0.27)	2012-2019	0.39 (0.12 to 0.52)	2004-2008	-4.08 (-5.83 to -2.72)	2013-2019	0.29 (-0.06 to 0.44)	2004-2008	-4.75 (-6.59 to -3.33)	2004-2008	-2.33 (-3.51 to -1.44)
	4	2019-2021	3.16 (2.62 to 3.70)	2019-2021	3.43 (2.43 to 4.07)	2008-2021	0.98 (0.67 to 1.33)	2019-2021	3.33 (2.36 to 3.97)	2008-2021	1.25 (0.89 to 1.64)	2008-2021	1.01 (0.83 to 1.21)
Central Sub-Saharan Africa	0	1990-1993	0.77 (0.61 to 0.86)	1990-1996	1.81 (1.77 to 1.85)	1990-1996	0.50 (0.24 to 1.27)	1990-1996	1.70 (1.67 to 1.73)	1990-2003	0.25 (0.15 to 0.63)	1990-2021	0.62 (0.59 to 0.65)
	1	1993-1996	1.02 (0.96 to 1.64)	1996-2000	2.36 (2.23 to 2.42)	1996-2021	0.19 (0.00 to 0.22)	1996-2001	2.43 (2.35 to 2.47)	2003-2021	0.01 (-0.16 to 0.07)		
	2	1996-2005	1.80 (1.76 to 1.82)	2000-2007	2.58 (2.55 to 2.65)			2001-2009	2.58 (2.55 to 2.62)				
	3	2005-2009	1.96 (1.90 to 2.05)	2007-2010	2.38 (2.25 to 2.51)			2009-2014	2.11 (2.07 to 2.16)				
	4	2009-2012	1.59 (1.48 to 1.70)	2010-2014	2.06 (1.98 to 2.11)			2014-2019	1.73 (1.68 to 1.77)				
	5	2012-2019	1.30 (1.26 to 1.32)	2014-2019	1.74 (1.69 to 1.77)			2019-2021	3.20 (3.07 to 3.30)				
	6	2019-2021	2.51 (2.37 to 2.62)	2019-2021	3.36 (3.24 to 3.47)								
East Asia	0	1990-1993	3.65 (3.38 to 3.96)	1990-1996	1.61 (1.41 to 1.76)	1990-1997	1.00 (-0.16 to 1.39)	1990-1996	1.28 (1.17 to 1.38)	1990-2004	1.04 (0.93 to 1.17)	1990-1996	0.99 (0.68 to 1.14)
	1	1993-1999	1.04 (0.90 to 1.15)	1996-1999	3.80 (3.51 to 4.05)	1997-2004	1.82 (1.15 to 2.46)	1996-1999	2.86 (2.48 to 3.02)	2004-2008	-4.40 (-4.88 to 0.54)	1996-1999	2.09 (1.59 to 2.33)
	2	1999-2005	-0.03 (-0.27 to 0.09)	1999-2007	0.38 (0.25 to 0.47)	2004-2007	-4.63 (-5.12 to -1.81)	1999-2004	1.53 (1.38 to 1.72)	2008-2013	-2.88 (-3.78 to -2.18)	1999-2004	1.36 (-1.83 to 1.49)
	3	2005-2009	0.80 (0.38 to 1.11)	2007-2017	1.45 (1.37 to 1.53)	2007-2013	-2.38 (-3.22 to -1.86)	2004-2012	0.54 (0.49 to 0.80)	2013-2021	-1.50 (-1.84 to -0.72)	2004-2008	-1.72 (-2.05 to -0.83)
	4	2009-2015	1.38 (1.27 to 1.67)	2017-2021	2.24 (1.97 to 2.71)	2013-2021	-1.37 (-1.75 to -0.41)	2012-2015	0.15 (-0.01 to 0.45)			2008-2015	-0.75 (-0.91 to -0.23)
	5	2015-2021	0.80 (0.66 to 0.90)					2015-2019	2.95 (2.79 to 3.14)			2015-2021	1.38 (1.16 to 1.70)
	6							2019-2021	2.18 (1.81 to 2.57)				
Eastern Europe	0	1990-1996	2.53 (2.44 to 2.57)	1990-1999	2.81 (2.79 to 2.83)	1990-1994	11.69 (8.97 to 14.14)	1990-2000	2.80 (2.78 to 2.82)	1990-1994	11.29 (7.56 to 14.14)	1990-1994	6.67 (5.97 to 7.43)
	1	1996-1999	2.96 (2.77 to 3.04)	1999-2002	2.28 (2.19 to 2.52)	1994-2000	-4.72 (-7.10 to -3.57)	2000-2010	2.06 (2.04 to 2.09)	1994-2011	-3.68 (-4.00 to -3.38)	1994-2000	-0.42 (-1.31 to 0.04)
	2	1999-2002	2.20 (2.08 to 2.32)	2002-2009	2.00 (1.96 to 2.03)	2000-2011	-2.63 (-3.11 to -0.68)	2010-2015	1.77 (1.64 to 1.84)	2011-2016	21.42 (19.45 to 23.66)	2000-2012	0.63 (0.44 to 1.12)
	3	2002-2010	2.01 (1.95 to 2.04)	2009-2016	1.74 (1.69 to 1.78)	2011-2016	24.95 (22.29 to 26.97)	2015-2021	2.04 (1.99 to 2.12)	2016-2021	5.11 (-0.34 to 8.77)	2012-2016	9.65 (8.76 to 10.38)
	4	2010-2015	1.67 (1.57 to 1.73)	2016-2021	2.07 (2.02 to 2.15)	2016-2021	5.68 (1.00 to 9.42)					2016-2021	2.75 (2.13 to 3.30)
	5	2015-2018	2.38 (2.25 to 2.46)										
	6	2018-2021	1.64 (1.51 to 1.73)										
Eastern Sub-Saharan Africa	0	1990-2002	0.40 (0.35 to 0.43)	1990-1994	1.57 (1.43 to 1.80)	1990-1998	0.18 (0.10 to 0.27)	1990-1994	1.57 (1.46 to 1.68)	1990-1995	0.25 (-0.00 to 0.66)	1990-1995	0.43 (0.30 to 0.67)
	1	2002-2006	0.59 (0.39 to 0.77)	1994-2001	1.10 (0.91 to 1.16)	1998-2008	-1.34 (-1.41 to -1.28)	1994-2001	1.07 (1.00 to 1.13)	1995-1998	-0.37 (-1.91 to -0.11)	1995-1998	-0.05 (-1.20 to 0.19)
	2	2006-2011	0.94 (0.64 to 1.01)	2001-2004	1.62 (1.39 to 1.74)	2008-2014	-0.59 (-0.77 to -0.41)	2001-2004	1.71 (1.54 to 1.79)	1998-2008	-1.75 (-1.88 to -1.00)	1998-2001	-1.41 (-1.55 to -1.03)
	3	2011-2015	1.11 (1.02 to 1.20)	2004-2010	1.32 (1.09 to 1.41)	2014-2021	0.28 (0.16 to 0.44)	2004-2010	1.40 (1.26 to 1.46)	2008-2014	-0.94 (-1.11 to -0.25)	2001-2008	-1.07 (-1.14 to -0.43)
	4	2015-2019	0.42 (0.33 to 0.48)	2010-2015	1.90 (1.79 to 2.08)			2010-2015	1.93 (1.85 to 2.06)	2014-2021	0.05 (-0.09 to 0.25)	2008-2013	-0.43 (-0.55 to 0.30)
	5	2019-2021	1.26 (1.07 to 1.40)	2015-2019	1.27 (1.08 to 1.40)			2015-2019	1.12 (0.98 to 1.26)			2013-2021	0.32 (0.24 to 0.42)
	6			2019-2021	1.95 (1.62 to 2.16)			2019-2021	1.65 (1.37 to 1.82)				
High-income Asia Pacific	0	1990-1996	1.39 (1.15 to 1.49)	1990-2001	1.64 (1.55 to 1.72)	1990-1993	-1.09 (-2.02 to -0.13)	1990-1996	1.86 (1.74 to 1.98)	1990-1993	-1.38 (-2.31 to -0.36)	1990-1993	0.41 (0.07 to 0.80)
	1	1996-2001	1.85 (1.61 to 2.11)	2001-2005	3.32 (2.87 to 3.82)	1993-1996	-10.80 (-11.50 to -10.06)	1996-1999	3.86 (3.53 to 4.06)	1993-1996	-10.31 (-11.06 to -9.47)	1993-1996	-2.60 (-2.93 to -2.22)
	2	2001-2005	2.51 (2.27 to 2.74)	2005-2010	1.15 (0.81 to 1.41)	1996-2005	-1.54 (-1.86 to -1.16)	1999-2005	2.68 (2.50 to 2.76)	1996-2005	-1.93 (-2.28 to -1.55)	1996-1999	2.43 (1.94 to 2.77)
	3	2005-2011	0.96 (0.78 to 1.04)	2010-2015	1.66 (1.50 to 2.82)	2005-2016	-4.50 (-5.01 to -4.18)	2005-2015	1.44 (1.38 to 1.49)	2005-2016	-5.40 (-5.84 to -5.06)	1999-2005	1.36 (0.91 to 1.55)
	4	2011-2015	1.24 (1.11 to 1.41)	2015-2021	4.02 (3.86 to 4.20)	2016-2021	-1.38 (-2.50 to 0.54)	2015-2019	4.26 (4.12 to 4.44)	2016-2021	-1.35 (-2.52 to 0.49)	2005-2015	0.30 (0.16 to 0.40)
	5	2015-2018	4.12 (3.90 to 4.28)					2019-2021	2.58 (2.13 to 3.17)			2015-2021	3.31 (3.09 to 3.51)
	6	2018-2021	3.23 (2.91 to 3.38)										
High-income North America	0	1990-1995	1.71 (1.56 to 1.88)	1990-1995	2.09 (1.92 to 2.26)	1990-1992	1.43 (0.63 to 2.67)	1990-1995	2.42 (2.27 to 2.57)	1990-1995	2.80 (2.26 to 3.36)	1990-1992	1.66 (1.30 to 2.07)
	1	1995-2000	5.05 (4.91 to 5.21)	1995-2000	5.33 (5.20 to 5.49)	1992-1995	3.52 (0.73 to 4.00)	1995-2000	5.25 (5.03 to 5.40)	1995-2003	-0.12 (-0.41 to 0.19)	1992-1995	3.13 (2.84 to 3.34)
	2	2000-2004	3.29 (3.07 to 3.49)	2000-2004	3.50 (3.30 to 3.72)	1995-2002	0.65 (-1.47 to 0.86)	2000-2004	3.54 (3.31 to 4.19)	2003-2008	-3.60 (-4.17 to -2.91)	1995-2002	2.08 (1.99 to 2.17)
	3	2004-2010	1.96 (1.84 to 2.06)	2004-2010	2.29 (2.17 to 2.39)	2002-2005	-1.91 (-4.24 to -1.00)	2004-2010	2.26 (2.05 to 2.72)	2008-2013	-6.85 (-7.33 to -6.36)	2002-2005	0.53 (0.16 to 0.93)
	4	2010-2015	3.15 (3.01 to 3.31)	2010-2015	3.47 (3.34 to 3.61)	2005-2009	-4.56 (-7.10 to -4.16)	2010-2015	3.37 (2.27 to 3.65)	2013-2021	-0.29 (-0.56 to -0.04)	2005-2012	-0.92 (-1.03 to -0.84)
	5	2015-2019	1.92 (1.66 to 2.11)	2015-2019	2.32 (2.09 to 2.50)	2009-2013	-7.33 (-7.69 to -1.34)	2015-2019	2.17 (1.86 to 3.38)			2012-2021	1.67 (1.59 to 1.75)
	6	2019-2021	3.08 (2.64 to 3.40)	2019-2021	3.19 (2.80 to 3.47)	2013-2021	-0.80 (-1.01 to -0.59)	2019-2021	3.03 (2.42 to 3.39)				
North Africa and Middle East	0	1990-2001	2.64 (2.59 to 2.70)	1990-1994	3.35 (3.26 to 3.44)	1990-1992	-2.89 (-3.59 to -1.67)	1990-1993	3.23 (3.13 to 3.37)	1990-2001	-0.42 (-0.79 to -0.17)	1990-1992	0.23 (-0.10 to 0.80)
	1	2001-2006	2.88 (2.59 to 2.94)	1994-2001	2.91 (2.82 to 2.95)	1992-2001	-0.38 (-0.53 to -0.17)	1993-2001	2.92 (2.90 to 2.95)	2001-2006	1.64 (-0.58 to 2.24)	1992-2001	0.97 (0.86 to 1.39)
	2	2006-2011	3.04 (2.87 to 3.09)	2001-2011	3.25 (2.99 to 3.28)	2001-2006	1.86 (1.32 to 2.27)	2001-2004	3.65 (3.57 to 3.71)	2006-2009	2.95 (0.99 to 3.47)	2001-2006	2.45 (0.73 to 2.79)
	3	2011-2014	3.22 (3.11 to 3.28)	2011-2014	3.80 (3.22 to 3.85)	2006-2009	4.11 (3.46 to 4.55)	2004-2011	3.20 (3.17 to 3.22)	2009-2015	0.83 (-0.46 to 1.21)	2006-2009	3.00 (2.03 to 3.25)
	4	2014-2018	2.84 (2.75 to 2.91)	2014-2018	3.01 (2.97 to 3.85)	2009-2012	0.42 (0.01 to 0.91)	2011-2014	3.74 (3.67 to 3.80)	2015-2021	-0.76 (-1.51 to -0.31)	2009-2015	2.07 (0.88 to 2.24)

Location	Segment	Incidence		Prevalence		Mortality		YLD		YLL		DALY	
		Period	APC (95%CI)	Period	APC (95%CI)	Period	APC (95%CI)	Period	APC (95%CI)	Period	APC (95%CI)	Period	APC (95%CI)
	5	2018-2021	2.37 (2.25 to 2.47)	2018-2021	2.82 (2.65 to 2.95)	2012-2015	2.25 (1.57 to 2.66)	2014-2018	2.82 (2.77 to 2.90)			2015-2021	1.03 (0.75 to 1.33)
	6					2015-2021	-0.71 (-1.01 to -0.49)	2018-2021	2.57 (2.44 to 2.66)				
Oceania	0	1990-1995	1.91 (1.71 to 2.01)	1990-2011	2.62 (2.60 to 2.64)	1990-1997	1.48 (0.89 to 2.15)	1990-1995	2.52 (2.20 to 2.64)	1990-2008	1.10 (1.02 to 1.19)	1990-2008	1.41 (1.36 to 1.48)
	1	1995-2000	2.48 (2.22 to 2.67)	2011-2021	2.26 (2.20 to 2.31)	1997-2008	1.01 (0.77 to 1.82)	1995-2000	2.85 (2.75 to 3.09)	2008-2021	-0.38 (-0.52 to -0.26)	2008-2021	0.28 (0.17 to 0.38)
	2	2000-2005	1.94 (1.80 to 2.44)			2008-2011	0.39 (-0.83 to 1.11)	2000-2005	2.47 (2.25 to 2.59)				
	3	2005-2010	2.11 (1.55 to 2.24)			2011-2014	-0.92 (-1.21 to 0.33)	2005-2010	2.77 (2.60 to 2.99)				
	4	2010-2018	1.56 (1.52 to 1.71)			2014-2021	-0.02 (-0.51 to 0.47)	2010-2021	2.21 (2.17 to 2.26)				
	5	2018-2021	1.25 (0.89 to 1.42)										
South Asia	0	1990-1995	1.34 (1.02 to 1.50)	1990-1996	1.89 (1.61 to 2.03)	1990-1996	2.07 (1.06 to 3.96)	1990-1993	3.90 (3.66 to 4.18)	1990-1996	1.36 (0.63 to 2.76)	1990-1995	2.06 (1.61 to 2.60)
	1	1995-1999	2.40 (1.74 to 2.70)	1996-1999	3.12 (2.63 to 3.35)	1996-2001	-1.87 (-4.38 to -0.46)	1993-2005	1.21 (1.16 to 1.25)	1996-2004	-0.67 (-1.85 to -0.21)	1995-2004	-0.01 (-0.33 to 0.18)
	2	1999-2005	1.14 (0.92 to 2.33)	1999-2006	1.57 (1.35 to 1.68)	2001-2021	1.81 (1.64 to 2.03)	2005-2014	3.18 (3.12 to 3.25)	2004-2021	1.81 (1.66 to 1.97)	2004-2019	2.19 (2.10 to 2.33)
	3	2005-2010	2.26 (1.14 to 2.63)	2006-2011	2.69 (2.49 to 3.11)			2014-2021	1.55 (1.45 to 1.66)			2019-2021	-0.37 (-1.59 to 1.15)
	4	2010-2017	1.87 (1.79 to 2.29)	2011-2021	2.10 (2.01 to 2.17)								
	5	2017-2021	1.55 (1.12 to 1.76)										
Southeast Asia	0	1990-1993	1.91 (1.75 to 2.05)	1990-1994	2.88 (2.42 to 3.72)	1990-1999	0.97 (0.89 to 1.08)	1990-1994	2.84 (2.54 to 3.35)	1990-2000	0.83 (0.76 to 0.91)	1990-1995	1.35 (1.21 to 1.55)
	1	1993-1998	1.25 (1.18 to 1.30)	1994-2000	0.83 (0.33 to 1.09)	1999-2006	0.29 (0.16 to 0.41)	1994-2000	1.06 (0.80 to 1.26)	2000-2005	-0.18 (-0.34 to 0.06)	1995-2003	0.75 (0.65 to 0.85)
	2	1998-2011	1.89 (1.87 to 1.90)	2000-2019	2.15 (2.11 to 2.19)	2006-2012	-0.49 (-0.68 to -0.36)	2000-2019	2.24 (2.20 to 2.27)	2005-2012	-0.80 (-0.90 to -0.71)	2003-2012	0.21 (0.11 to 0.70)
	3	2011-2014	2.23 (2.08 to 2.30)	2019-2021	6.20 (4.70 to 6.98)	2012-2019	0.66 (0.58 to 0.83)	2019-2021	5.30 (4.19 to 5.90)	2012-2019	0.48 (0.40 to 0.61)	2012-2019	1.09 (0.20 to 1.17)
	4	2014-2019	1.73 (1.62 to 1.78)			2019-2021	-0.45 (-0.93 to 0.13)			2019-2021	-0.46 (-0.90 to 0.11)	2019-2021	1.80 (1.18 to 2.12)
	5	2019-2021	2.57 (2.34 to 2.72)										
Southern Latin America	0	1990-2000	2.31 (2.20 to 2.36)	1990-2005	2.83 (2.80 to 2.85)	1990-1993	1.96 (0.39 to 4.64)	1990-2005	2.78 (2.74 to 2.81)	1990-1994	1.38 (-0.26 to 4.57)	1990-1994	2.26 (1.79 to 3.02)
	1	2000-2004	2.66 (2.45 to 2.86)	2005-2010	1.57 (1.40 to 1.69)	1993-2004	-0.71 (-1.13 to -0.42)	2005-2010	1.52 (1.27 to 1.78)	1994-2004	-1.35 (-2.10 to -0.62)	1994-1997	-0.75 (-1.28 to 0.14)
	2	2004-2010	1.33 (1.15 to 1.43)	2010-2015	2.49 (2.32 to 2.80)	2004-2009	-3.47 (-5.07 to -2.56)	2010-2015	2.46 (2.26 to 2.86)	2004-2021	-2.28 (-3.57 to -2.04)	1997-2004	0.84 (0.60 to 1.78)
	3	2010-2015	2.03 (1.87 to 2.36)	2015-2019	1.51 (1.19 to 1.72)	2009-2016	-1.03 (-1.62 to 0.86)	2015-2019	1.48 (1.10 to 1.70)			2004-2008	-1.03 (-1.84 to -0.25)
	4	2015-2019	1.11 (0.76 to 1.31)	2019-2021	2.78 (2.26 to 3.15)	2016-2021	-3.95 (-5.62 to -2.97)	2019-2021	2.63 (2.00 to 3.03)			2008-2021	0.25 (0.09 to 0.55)
	5	2019-2021	3.12 (2.55 to 3.51)										
Southern Sub-Saharan Africa	0	1990-1996	1.57 (1.14 to 1.74)	1990-1999	2.14 (1.93 to 2.25)	1990-1995	1.65 (-2.50 to 3.19)	1990-1999	2.12 (1.96 to 2.23)	1990-1995	0.86 (-3.51 to 2.47)	1990-1995	1.12 (-1.88 to 2.29)
	1	1996-2001	2.33 (1.70 to 2.59)	1999-2005	2.88 (2.40 to 3.38)	1995-1998	8.74 (5.98 to 10.38)	1999-2005	2.95 (2.70 to 3.42)	1995-1998	8.07 (4.79 to 9.90)	1995-1998	6.62 (4.39 to 7.99)
	2	2001-2004	2.98 (2.54 to 3.21)	2005-2010	2.03 (1.51 to 2.85)	1998-2007	3.83 (3.20 to 4.27)	2005-2010	2.13 (1.61 to 2.44)	1998-2007	3.99 (0.64 to 4.45)	1998-2006	3.96 (0.13 to 4.37)
	3	2004-2010	1.77 (1.39 to 1.90)	2010-2015	3.10 (2.12 to 3.62)	2007-2015	0.55 (-0.05 to 1.20)	2010-2015	3.22 (2.95 to 3.76)	2007-2015	0.45 (-0.41 to 1.30)	2006-2015	1.13 (-1.96 to 2.18)
	4	2010-2014	2.43 (2.22 to 2.76)	2015-2018	2.12 (1.62 to 2.88)	2015-2021	-2.35 (-3.51 to -1.67)	2015-2018	2.18 (1.60 to 2.80)	2015-2021	-2.57 (-4.01 to -1.71)	2015-2021	-1.70 (-2.95 to -0.56)
	5	2014-2018	1.76 (1.40 to 1.94)	2018-2021	0.44 (-0.07 to 0.80)			2018-2021	0.15 (-0.35 to 0.53)				
	6	2018-2021	-0.00 (-0.36 to 0.31)										
Tropical Latin America	0	1990-1996	-0.11 (-0.25 to -0.00)	1990-1995	0.13 (0.04 to 0.23)	1990-1995	0.78 (0.50 to 1.14)	1990-1993	-0.05 (-0.28 to 0.08)	1990-1995	0.65 (0.32 to 1.06)	1990-1993	0.71 (-0.39 to 2.37)
	1	1996-2005	0.97 (0.91 to 1.04)	1995-2005	1.16 (1.12 to 1.20)	1995-1998	-2.72 (-3.23 to -1.94)	1993-1996	0.43 (0.33 to 0.93)	1995-1998	-2.99 (-3.54 to -2.15)	1993-2021	-0.53 (-0.71 to -0.48)
	2	2005-2011	0.02 (-0.13 to 0.14)	2005-2010	-0.20 (-0.26 to -0.14)	1998-2001	0.77 (0.07 to 1.36)	1996-2005	1.16 (1.13 to 1.20)	1998-2001	-0.00 (-0.70 to 0.60)		
	3	2011-2021	1.23 (1.17 to 1.28)	2010-2021	1.44 (1.41 to 1.47)	2001-2005	-1.67 (-2.49 to -1.23)	2005-2010	-0.08 (-0.14 to -0.03)	2001-2005	-2.18 (-3.02 to -1.69)		
	4					2005-2010	0.37 (-0.14 to 1.36)	2010-2013	1.25 (1.15 to 1.37)	2005-2010	-0.07 (-0.59 to 1.02)		
	5					2010-2017	-2.64 (-3.42 to -2.32)	2013-2019	1.53 (1.49 to 1.68)	2010-2017	-2.83 (-3.55 to -2.48)		
	6					2017-2021	-0.80 (-1.53 to 0.47)	2019-2021	1.02 (0.82 to 1.25)	2017-2021	-0.21 (-1.08 to 1.14)		
Western Europe	0	1990-1995	1.25 (1.19 to 1.32)	1990-1993	1.64 (1.41 to 1.83)	1990-2000	-2.39 (-2.56 to -2.27)	1990-1996	2.25 (2.19 to 2.31)	1990-2000	-2.87 (-3.05 to -2.75)	1990-1995	-0.75 (-1.02 to -0.61)
	1	1995-2000	2.27 (2.23 to 2.32)	1993-1996	2.01 (1.89 to 3.31)	2000-2003	-0.12 (-1.11 to 0.27)	1996-1999	3.86 (3.75 to 3.98)	2000-2003	-0.97 (-1.92 to -0.59)	1995-1998	0.02 (-0.59 to 0.46)
	2	2000-2010	1.31 (1.28 to 1.34)	1996-1999	3.27 (2.14 to 3.38)	2003-2013	-3.50 (-3.78 to -3.33)	1999-2002	1.95 (1.79 to 2.06)	2003-2013	-3.91 (-4.17 to -3.75)	1998-2003	0.50 (-1.21 to 0.75)
	3	2010-2015	1.67 (1.45 to 1.78)	1999-2003	2.13 (1.77 to 2.26)	2013-2021	-1.97 (-2.27 to -1.57)	2002-2011	1.59 (1.51 to 1.62)	2013-2021	-2.07 (-2.38 to -1.69)	2003-2006	-1.20 (-1.39 to -0.03)
	4	2015-2019	2.27 (1.84 to 2.37)	2003-2011	1.77 (1.70 to 1.88)			2011-2015	2.08 (1.87 to 2.30)			2006-2014	-0.17 (-0.31 to 1.51)
	5	2019-2021	2.83 (2.52 to 3.02)	2011-2015	2.21 (2.10 to 2.47)			2015-2021	2.76 (2.69 to 2.86)			2014-2021	1.47 (1.26 to 1.71)
	6			2015-2021	3.09 (3.02 to 3.16)								
Western Sub-Saharan Africa	0	1990-2000	1.70 (1.59 to 1.75)	1990-2000	2.22 (2.07 to 2.27)	1990-1995	1.50 (1.41 to 1.67)	1990-2001	2.23 (2.15 to 2.27)	1990-2002	1.32 (1.25 to 1.38)	1990-2003	1.56 (1.43 to 1.71)
	1	2000-2005	1.98 (1.84 to 2.22)	2000-2004	2.58 (2.35 to 2.80)	1995-1999	1.14 (0.96 to 1.25)	2001-2004	2.69 (2.38 to 2.82)	2002-2014	0.34 (0.28 to 0.41)	2003-2009	0.72 (0.53 to 1.67)
	2	2005-2015	1.55 (1.50 to 1.60)	2004-2015	1.99 (1.93 to 2.05)	1999-2002	1.73 (1.56 to 1.84)	2004-2015	1.99 (1.93 to 2.04)	2014-2021	-0.53 (-0.68 to -0.40)	2009-2014	0.96 (0.60 to 1.20)
	3	2015-2018	0.64 (0.49 to 0.93)	2015-2018	1.15 (0.98 to 1.47)	2002-2013	0.61 (0.59 to 0.64)	2015-2018	1.09 (0.93 to 1.40)			2014-2019	0.02 (-0.26 to 1.04)
	4	2018-2021	1.69 (1.46 to 2.15)	2018-2021	2.17 (1.92 to 2.67)	2013-2021	-0.29 (-0.32 to -0.25)	2018-2021	2.09 (1.86 to 2.53)			2019-2021	0.56 (0.07 to 0.82)
**204 countries and territories**													
Afghanistan	0	1990-1997	2.92 (2.85 to 3.09)	1990-1998	3.60 (3.54 to 3.67)	1990-1998	1.26 (0.97 to 1.35)	1990-1994	3.66 (3.57 to 3.82)	1990-2000	1.32 (1.21 to 1.41)	1990-2000	2.12 (2.04 to 2.19)
	1	1997-2005	2.73 (2.57 to 2.79)	1998-2005	3.08 (3.01 to 3.15)	1998-2001	1.70 (1.33 to 2.42)	1994-1999	3.40 (3.28 to 3.48)	2000-2006	2.94 (2.74 to 3.19)	2000-2008	2.93 (2.83 to 3.07)
	2	2005-2010	3.52 (3.40 to 3.68)	2005-2010	4.46 (4.39 to 4.54)	2001-2005	3.39 (3.08 to 3.71)	1999-2005	3.05 (2.99 to 3.09)	2006-2014	1.05 (0.83 to 1.18)	2008-2021	2.06 (2.01 to 2.10)
	3	2010-2015	2.67 (2.57 to 2.91)	2010-2014	3.11 (3.00 to 3.26)	2005-2008	2.02 (1.49 to 2.52)	2005-2010	4.29 (4.24 to 4.34)	2014-2021	1.66 (1.51 to 1.91)		
	4	2015-2021	2.37 (2.21 to 2.45)	2014-2021	2.72 (2.65 to 2.78)	2008-2014	1.13 (0.88 to 1.24)	2010-2014	3.07 (3.00 to 3.18)				
	5					2014-2018	2.00 (1.80 to 2.30)	2014-2019	2.74 (2.67 to 2.85)				
	6					2018-2021	1.20 (0.78 to 1.45)	2019-2021	2.34 (2.18 to 2.54)				
Albania	0	1990-1995	1.24 (1.16 to 1.32)	1990-1992	1.23 (1.03 to 1.57)	1990-1994	-5.80 (-7.47 to -4.51)	1990-1992	1.21 (0.99 to 1.58)	1990-1994	-5.73 (-8.41 to -4.08)	1990-1994	-0.76 (-1.05 to -0.46)
	1	1995-1999	2.66 (2.56 to 2.77)	1992-1995	1.69 (1.60 to 2.69)	1994-2004	2.28 (1.92 to 2.69)	1992-1995	1.71 (1.61 to 2.69)	1994-2004	1.88 (1.46 to 2.45)	1994-1999	2.48 (2.24 to 2.86)

Location	Segment	Incidence		Prevalence		Mortality		YLD		YLL		DALY	
		Period	APC (95%CI)	Period	APC (95%CI)	Period	APC (95%CI)	Period	APC (95%CI)	Period	APC (95%CI)	Period	APC (95%CI)
	2	1999-2003	1.48 (1.36 to 1.81)	1995-2000	2.63 (1.32 to 2.72)	2004-2008	-6.32 (-7.41 to -4.74)	1995-2000	2.62 (1.33 to 2.74)	2004-2008	-6.98 (-9.07 to -5.21)	1999-2004	1.61 (1.11 to 1.86)
	3	2003-2016	1.22 (1.19 to 1.25)	2000-2015	1.35 (1.19 to 1.40)	2008-2017	3.18 (2.67 to 4.02)	2000-2015	1.37 (1.08 to 1.42)	2008-2017	2.72 (2.15 to 3.98)	2004-2008	-0.63 (-1.17 to -0.20)
	4	2016-2019	0.56 (0.43 to 0.78)	2015-2021	1.05 (0.94 to 1.13)	2017-2021	-1.48 (-4.23 to 0.11)	2015-2021	0.99 (0.88 to 1.09)	2017-2021	-1.98 (-5.29 to -0.18)	2008-2017	1.65 (1.53 to 1.84)
	5	2019-2021	1.35 (1.04 to 1.59)									2017-2021	0.22 (-0.32 to 0.60)
Algeria	0	1990-2005	2.61 (2.60 to 2.62)	1990-1994	3.40 (3.30 to 3.53)	1990-1992	-4.53 (-5.61 to -2.57)	1990-1994	3.27 (3.19 to 3.38)	1990-1998	-0.68 (-1.10 to -0.43)	1990-1999	1.54 (1.44 to 1.64)
	1	2005-2009	3.36 (3.32 to 3.40)	1994-2004	3.00 (2.97 to 3.03)	1992-1998	-0.67 (-1.33 to 0.06)	1994-2004	2.96 (2.93 to 2.98)	1998-2001	2.26 (0.36 to 3.95)	1999-2009	3.47 (3.37 to 3.59)
	2	2009-2015	2.48 (2.45 to 2.51)	2004-2010	3.40 (3.37 to 3.45)	1998-2001	2.36 (0.33 to 4.48)	2004-2010	3.38 (3.34 to 3.41)	2001-2005	4.85 (4.13 to 5.71)	2009-2021	2.27 (2.19 to 2.34)
	3	2015-2018	3.23 (3.16 to 3.30)	2010-2015	2.70 (2.66 to 2.74)	2001-2005	5.63 (3.95 to 6.62)	2010-2015	2.71 (2.67 to 2.75)	2005-2011	2.82 (2.34 to 3.14)		
	4	2018-2021	2.33 (2.24 to 2.42)	2015-2018	3.52 (3.44 to 3.59)	2005-2011	3.80 (3.08 to 4.25)	2015-2018	3.55 (3.49 to 3.62)	2011-2021	0.87 (0.72 to 1.01)		
	5			2018-2021	2.62 (2.52 to 2.70)	2011-2019	1.09 (0.95 to 3.82)	2018-2021	2.49 (2.41 to 2.57)				
	6					2019-2021	-0.35 (-1.42 to 0.88)						
American Samoa	0	1990-1992	3.52 (3.33 to 3.71)	1990-1993	4.61 (4.41 to 4.85)	1990-2005	3.48 (3.34 to 3.62)	1990-1993	4.48 (4.30 to 4.70)	1990-2005	3.54 (3.39 to 3.71)	1990-1997	3.92 (3.49 to 4.66)
	1	1992-1995	2.96 (2.66 to 3.06)	1993-1996	3.59 (3.29 to 3.95)	2005-2017	-0.89 (-1.21 to -0.71)	1993-1996	3.52 (3.23 to 3.75)	2005-2017	-0.85 (-1.30 to -0.62)	1997-2005	3.11 (2.19 to 3.71)
	2	1995-2005	2.28 (2.25 to 2.30)	1996-2005	2.92 (2.86 to 2.97)	2017-2021	1.39 (0.00 to 4.25)	1996-2005	2.84 (2.79 to 2.88)	2017-2021	1.30 (-0.27 to 4.31)	2005-2018	0.26 (-0.25 to 3.10)
	3	2005-2010	2.02 (1.88 to 2.09)	2005-2010	2.48 (2.27 to 2.59)			2005-2010	2.40 (2.20 to 2.49)			2018-2021	1.50 (0.33 to 2.93)
	4	2010-2014	2.37 (2.30 to 2.49)	2010-2014	2.85 (2.73 to 3.02)			2010-2014	2.77 (2.66 to 2.93)				
	5	2014-2019	0.82 (0.77 to 0.86)	2014-2019	0.83 (0.75 to 0.91)			2014-2019	0.86 (0.78 to 0.94)				
	6	2019-2021	2.34 (2.18 to 2.47)	2019-2021	2.88 (2.60 to 3.09)			2019-2021	2.75 (2.49 to 2.96)				
Andorra	0	1990-1992	1.51 (1.35 to 1.78)	1990-1992	1.84 (1.69 to 2.06)	1990-2010	-0.74 (-1.10 to -0.56)	1990-1992	1.79 (1.63 to 2.09)	1990-1994	0.62 (-0.89 to 5.84)	1990-2009	0.94 (0.87 to 1.00)
	1	1992-1995	1.93 (1.85 to 2.75)	1992-1995	2.22 (2.14 to 2.89)	2010-2019	0.44 (-0.06 to 2.18)	1992-1996	2.35 (2.21 to 2.75)	1994-2008	-1.15 (-3.27 to -0.41)	2009-2019	1.91 (1.77 to 2.13)
	2	1995-2000	2.72 (2.17 to 2.83)	1995-2000	2.89 (2.44 to 3.01)	2019-2021	-11.13 (-14.93 to -6.85)	1996-2000	2.96 (2.81 to 3.12)	2008-2019	0.20 (-0.20 to 1.14)	2019-2021	-1.69 (-2.94 to -0.25)
	3	2000-2005	2.17 (2.05 to 2.42)	2000-2005	2.43 (2.33 to 2.55)			2000-2004	2.34 (2.23 to 2.50)	2019-2021	-11.11 (-14.73 to -6.91)		
	4	2005-2015	2.46 (2.42 to 2.50)	2005-2015	2.56 (2.54 to 2.63)			2004-2015	2.57 (2.54 to 2.63)				
	5	2015-2019	3.00 (2.92 to 3.16)	2015-2019	3.21 (3.15 to 3.30)			2015-2019	3.22 (3.15 to 3.42)				
	6	2019-2021	2.46 (2.27 to 2.66)	2019-2021	2.27 (2.10 to 2.43)			2019-2021	2.16 (1.93 to 2.41)				
Angola	0	1990-1996	0.96 (0.87 to 1.05)	1990-1995	1.45 (1.22 to 1.60)	1990-2003	0.27 (0.12 to 0.46)	1990-1995	1.37 (1.16 to 1.50)	1990-2004	0.09 (-0.08 to 0.34)	1990-2005	0.68 (0.57 to 0.82)
	1	1996-2009	1.90 (1.87 to 1.93)	1995-1999	2.37 (2.02 to 2.68)	2003-2015	-0.59 (-0.85 to -0.43)	1995-2000	2.47 (2.18 to 2.66)	2004-2015	-0.86 (-1.61 to -0.63)	2005-2015	0.04 (-0.65 to 0.23)
	2	2009-2019	1.17 (1.10 to 1.22)	1999-2005	3.13 (3.01 to 3.34)	2015-2021	1.61 (1.08 to 2.36)	2000-2005	3.10 (2.94 to 3.38)	2015-2021	1.31 (0.56 to 2.66)	2015-2021	1.50 (1.01 to 2.38)
	3	2019-2021	2.20 (1.71 to 2.44)	2005-2009	2.20 (1.96 to 2.50)			2005-2009	2.24 (1.99 to 2.50)				
	4			2009-2016	1.63 (1.41 to 1.72)			2009-2016	1.61 (1.38 to 1.70)				
	5			2016-2021	2.17 (2.02 to 2.44)			2016-2021	2.06 (1.91 to 2.35)				
Antigua and Barbuda	0	1990-1994	2.00 (1.79 to 2.41)	1990-1994	2.65 (2.39 to 2.84)	1990-1994	6.43 (3.36 to 11.11)	1990-1994	2.63 (2.37 to 2.86)	1990-1994	5.25 (2.84 to 9.16)	1990-1994	4.23 (2.66 to 6.59)
	1	1994-2006	1.36 (1.31 to 1.42)	1994-2007	1.59 (1.57 to 1.75)	1994-2021	-1.17 (-1.42 to -0.98)	1994-2008	1.58 (1.55 to 1.67)	1994-2021	-1.34 (-1.52 to -1.18)	1994-2021	-0.29 (-0.43 to -0.17)
	2	2006-2017	1.13 (1.07 to 1.20)	2007-2018	1.49 (1.42 to 1.54)			2008-2018	1.45 (1.36 to 1.51)				
	3	2017-2021	0.68 (0.03 to 0.89)	2018-2021	0.96 (0.38 to 1.20)			2018-2021	0.83 (0.23 to 1.09)				
Argentina	0	1990-1997	2.00 (1.87 to 2.05)	1990-1995	2.44 (2.26 to 2.50)	1990-1999	0.34 (-0.36 to 1.27)	1990-2004	2.48 (2.47 to 2.50)	1990-1999	0.16 (-0.55 to 1.17)	1990-1993	2.06 (1.09 to 3.45)
	1	1997-2004	2.14 (2.10 to 2.24)	1995-2004	2.57 (2.55 to 2.63)	1999-2021	-2.21 (-2.45 to -2.02)	2004-2011	1.19 (1.16 to 1.22)	1999-2021	-2.14 (-2.39 to -1.95)	1993-2004	0.45 (0.11 to 0.65)
	2	2004-2010	0.71 (0.65 to 0.76)	2004-2011	1.27 (1.23 to 1.30)			2011-2014	2.42 (2.30 to 2.50)			2004-2009	-1.55 (-2.76 to -0.84)
	3	2010-2019	1.62 (1.57 to 1.66)	2011-2014	2.43 (2.29 to 2.53)			2014-2019	1.98 (1.85 to 2.03)			2009-2021	0.32 (0.09 to 0.65)
	4	2019-2021	3.03 (2.71 to 3.25)	2014-2019	2.01 (1.81 to 2.06)			2019-2021	2.65 (2.42 to 2.81)				
	5			2019-2021	2.79 (2.48 to 2.99)								
Armenia	0	1990-2002	2.84 (2.81 to 2.91)	1990-1994	3.40 (3.21 to 3.73)	1990-1996	9.69 (8.24 to 11.63)	1990-1994	3.43 (3.23 to 3.83)	1990-1996	9.08 (7.37 to 11.62)	1990-1996	7.16 (6.05 to 8.57)
	1	2002-2005	2.28 (2.00 to 2.56)	1994-2004	2.99 (2.80 to 3.04)	1996-2003	3.11 (2.01 to 4.36)	1994-2004	3.02 (2.82 to 3.08)	1996-2003	2.18 (0.90 to 4.06)	1996-2003	2.44 (1.53 to 6.30)
	2	2005-2010	-0.69 (-0.85 to -0.56)	2004-2007	0.21 (0.04 to 3.18)	2003-2006	-8.99 (-10.98 to -5.22)	2004-2007	0.22 (0.03 to 3.21)	2003-2006	-9.13 (-11.50 to 1.89)	2003-2006	-5.80 (-7.20 to 2.82)
	3	2010-2015	1.03 (0.91 to 1.15)	2007-2010	-0.39 (-0.59 to -0.06)	2006-2009	3.69 (0.59 to 6.25)	2007-2010	-0.49 (-0.71 to -0.13)	2006-2009	3.62 (-6.64 to 6.77)	2006-2009	2.00 (-5.14 to 3.79)
	4	2015-2019	-0.82 (-1.06 to -0.66)	2010-2015	1.24 (1.13 to 1.41)	2009-2015	-2.22 (-3.85 to -1.14)	2010-2015	1.26 (1.13 to 1.49)	2009-2015	-2.69 (-4.77 to -0.91)	2009-2015	-1.28 (-2.58 to 0.02)
	5	2019-2021	1.14 (0.50 to 1.54)	2015-2019	-0.54 (-0.76 to -0.41)	2015-2018	-19.42 (-21.30 to -16.58)	2015-2019	-0.63 (-0.91 to -0.46)	2015-2018	-17.59 (-21.13 to -15.02)	2015-2018	-11.07 (-12.85 to -8.20)
	6			2019-2021	1.37 (0.83 to 1.71)	2018-2021	-4.62 (-8.19 to 2.20)	2019-2021	1.20 (0.64 to 1.55)	2019-2021	0.33 (-9.40 to 6.26)	2018-2021	-2.06 (-5.12 to 3.40)
Australia	0	1990-1995	1.99 (1.91 to 2.06)	1990-1996	2.19 (2.09 to 2.27)	1990-1995	1.55 (0.69 to 2.43)	1990-1995	1.12 (0.86 to 1.32)	1990-1995	1.70 (0.69 to 2.83)	1990-1994	1.76 (1.21 to 2.47)
	1	1995-2000	2.56 (2.51 to 2.61)	1996-1999	2.88 (2.27 to 2.99)	1995-2001	-3.28 (-4.25 to -2.57)	1995-2000	2.78 (2.62 to 3.00)	1995-2001	-3.73 (-5.08 to -2.90)	1994-2004	-0.51 (-0.77 to -0.34)
	2	2000-2014	1.56 (1.55 to 1.58)	1999-2003	2.25 (2.05 to 2.78)	2001-2008	1.85 (1.20 to 3.01)	2000-2006	0.19 (0.07 to 0.31)	2001-2010	0.50 (0.03 to 1.16)	2004-2010	1.75 (1.33 to 2.53)
	3	2014-2018	1.31 (1.17 to 1.42)	2003-2007	1.21 (1.03 to 2.13)	2008-2011	-2.29 (-3.58 to 0.63)	2006-2009	4.29 (3.84 to 4.54)	2010-2014	-9.13 (-10.89 to -7.48)	2010-2014	-2.29 (-3.38 to -1.33)
	4	2018-2021	1.91 (1.75 to 2.20)	2007-2011	1.93 (1.31 to 2.11)	2011-2014	-9.43 (-10.50 to -7.26)	2009-2013	2.38 (2.11 to 2.72)	2014-2021	-0.17 (-0.89 to 0.63)	2014-2021	1.17 (0.76 to 1.84)
	5			2011-2014	2.34 (2.00 to 2.46)	2014-2021	-0.66 (-1.25 to 0.12)	2013-2021	1.82 (1.62 to 1.92)				
	6			2014-2021	1.81 (1.69 to 1.88)								
Austria	0	1990-1993	3.34 (3.22 to 3.43)	1990-1993	4.22 (3.96 to 4.48)	1990-2001	-4.05 (-4.68 to -3.47)	1990-1996	3.96 (3.88 to 4.04)	1990-2001	-4.25 (-5.08 to -3.56)	1990-1993	-2.86 (-4.65 to -1.44)
	1	1993-1999	2.60 (2.56 to 2.64)	1993-2000	3.24 (3.15 to 3.31)	2001-2005	13.74 (10.05 to 19.89)	1996-1999	5.59 (5.43 to 5.74)	2001-2005	12.48 (8.38 to 18.98)	1993-2001	-0.37 (-0.78 to 1.20)
	2	1999-2006	1.64 (1.60 to 1.67)	2000-2006	2.10 (1.92 to 2.20)	2005-2010	-7.78 (-11.61 to -5.18)	1999-2002	2.81 (2.63 to 2.98)	2005-2010	-8.51 (-12.82 to -5.38)	2001-2005	7.22 (5.76 to 9.10)
	3	2006-2009	2.13 (1.99 to 2.20)	2006-2014	2.44 (2.37 to 2.62)	2010-2021	-1.95 (-2.81 to -0.23)	2002-2005	1.80 (1.48 to 1.95)	2010-2021	-2.05 (-3.05 to 0.21)	2005-2010	-4.16 (-5.46 to -3.15)
	4	2009-2014	1.72 (1.62 to 1.77)	2014-2021	1.70 (1.61 to 1.78)			2005-2010	0.70 (0.59 to 0.80)			2010-2021	0.31 (-0.15 to 0.85)
	5	2014-2019	1.05 (0.97 to 1.11)					2010-2014	2.40 (2.23 to 2.60)				
	6	2019-2021	1.87 (1.63 to 2.02)					2014-2021	1.65 (1.55 to 1.73)				

Location	Segment	Incidence		Prevalence		Mortality		YLD		YLL		DALY	
		Period	APC (95%CI)	Period	APC (95%CI)	Period	APC (95%CI)	Period	APC (95%CI)	Period	APC (95%CI)	Period	APC (95%CI)
Azerbaijan	0	1990-1995	3.25 (2.96 to 3.45)	1990-1995	3.72 (3.47 to 3.88)	1990-1994	9.72 (8.67 to 10.51)	1990-1995	3.72 (3.49 to 3.87)	1990-1993	12.34 (11.10 to 13.54)	1990-1993	8.76 (8.16 to 9.37)
	1	1995-2005	4.13 (4.07 to 4.21)	1995-2005	4.24 (4.19 to 4.31)	1994-2004	3.28 (2.99 to 3.52)	1995-2005	4.29 (4.24 to 4.35)	1993-2003	3.34 (3.03 to 3.60)	1993-2003	3.70 (3.56 to 3.82)
	2	2005-2010	2.02 (1.81 to 2.15)	2005-2010	2.37 (2.20 to 2.47)	2004-2017	-0.64 (-0.81 to -0.42)	2005-2010	2.31 (2.13 to 2.42)	2003-2017	-1.06 (-1.21 to -0.36)	2003-2017	0.62 (0.54 to 0.81)
	3	2010-2014	3.08 (2.78 to 3.40)	2010-2015	2.94 (2.79 to 3.17)	2017-2021	-3.02 (-4.51 to -2.05)	2010-2015	2.98 (2.83 to 3.22)	2017-2021	-2.63 (-4.84 to -1.46)	2017-2021	-0.38 (-1.59 to 0.27)
	4	2014-2021	1.64 (1.53 to 1.75)	2015-2021	1.79 (1.68 to 1.89)			2015-2021	1.71 (1.60 to 1.81)				
Bahamas	0	1990-2005	1.40 (1.38 to 1.43)	1990-2004	1.97 (1.94 to 2.00)	1990-1996	3.88 (1.97 to 7.20)	1990-1994	2.05 (1.90 to 2.38)	1990-1996	2.71 (0.99 to 4.72)	1990-1996	2.46 (1.66 to 3.56)
	1	2005-2021	1.13 (1.10 to 1.15)	2004-2021	1.48 (1.46 to 1.51)	1996-1999	-5.87 (-7.56 to 0.50)	1994-2005	1.86 (1.40 to 1.89)	1996-2002	-4.70 (-8.10 to -0.16)	1996-2001	-2.48 (-4.32 to -1.35)
	2					1999-2021	-2.05 (-10.90 to 0.02)	2005-2021	1.43 (1.39 to 1.48)	2002-2010	-1.10 (-4.10 to 2.45)	2001-2021	-0.42 (-0.64 to -0.05)
	3									2010-2021	-2.97 (-7.96 to -2.13)		
Bahrain	0	1990-1995	3.73 (3.60 to 3.98)	1990-1993	4.60 (4.30 to 5.07)	1990-1997	4.33 (3.59 to 5.35)	1990-1993	5.00 (4.79 to 5.25)	1990-1997	4.05 (3.44 to 4.76)	1990-1997	3.93 (3.52 to 4.35)
	1	1995-2000	3.34 (2.87 to 3.44)	1993-2000	3.71 (3.56 to 3.80)	1997-2002	-0.31 (-2.36 to 0.62)	1993-1996	2.82 (2.55 to 3.02)	1997-2002	-0.73 (-2.32 to 0.05)	1997-2002	-0.03 (-0.75 to 0.50)
	2	2000-2008	2.30 (2.22 to 2.35)	2000-2008	2.76 (2.68 to 2.86)	2002-2005	6.02 (3.88 to 7.20)	1996-2001	2.38 (2.12 to 2.81)	2002-2005	4.65 (2.80 to 5.59)	2002-2005	4.28 (3.05 to 4.87)
	3	2008-2014	1.39 (1.31 to 1.47)	2008-2014	1.84 (1.74 to 2.67)	2005-2012	0.10 (-0.56 to 0.68)	2001-2005	2.94 (2.42 to 3.18)	2005-2012	-1.47 (-1.92 to -0.77)	2005-2013	-0.47 (-0.70 to -0.04)
	4	2014-2021	0.62 (0.56 to 0.68)	2014-2018	0.86 (0.64 to 1.77)	2012-2015	-7.18 (-8.17 to -5.07)	2005-2011	2.37 (2.21 to 2.53)	2012-2018	-3.32 (-4.94 to -2.77)	2013-2018	-1.78 (-3.03 to -1.21)
	5			2018-2021	1.38 (1.09 to 1.72)	2015-2021	-0.22 (-0.95 to 0.99)	2011-2014	3.47 (3.13 to 3.62)	2018-2021	2.07 (-0.09 to 5.48)	2018-2021	1.69 (0.30 to 3.81)
	6							2014-2021	1.01 (0.93 to 1.09)				
Bangladesh	0	1990-1996	1.96 (1.76 to 2.06)	1990-1995	2.22 (1.91 to 2.42)	1990-1998	2.07 (1.06 to 2.77)	1990-1995	2.25 (2.01 to 2.41)	1990-1998	1.08 (-0.02 to 1.57)	1990-1992	-0.17 (-1.20 to 1.63)
	1	1996-1999	2.78 (2.43 to 2.94)	1995-1999	3.61 (3.37 to 3.97)	1998-2002	7.13 (4.59 to 9.47)	1995-1999	3.51 (3.23 to 3.84)	1998-2002	4.39 (2.92 to 6.01)	1992-1998	1.92 (1.44 to 4.16)
	2	1999-2006	1.43 (1.36 to 1.50)	1999-2005	1.42 (1.30 to 1.53)	2002-2005	-2.85 (-4.44 to 0.47)	1999-2005	1.85 (1.73 to 1.95)	2002-2010	0.67 (0.11 to 1.29)	1998-2002	3.97 (-0.01 to 4.89)
	3	2006-2009	3.65 (3.37 to 3.81)	2005-2010	4.01 (3.84 to 4.18)	2005-2010	1.56 (-0.04 to 4.11)	2005-2010	3.91 (3.74 to 4.06)	2010-2013	-3.53 (-4.50 to -1.51)	2002-2005	0.12 (-0.59 to 2.61)
	4	2009-2018	2.44 (2.38 to 2.50)	2010-2015	2.86 (2.53 to 3.05)	2010-2013	-5.35 (-6.73 to -2.27)	2010-2015	2.55 (2.28 to 2.71)	2013-2021	0.84 (0.25 to 2.26)	2005-2010	2.22 (-1.89 to 3.29)
	5	2018-2021	1.58 (1.07 to 1.89)	2015-2018	3.92 (3.50 to 4.17)	2013-2021	1.78 (0.98 to 2.96)	2015-2018	3.95 (3.55 to 4.17)			2010-2013	-1.64 (-2.32 to 2.50)
	6			2018-2021	2.23 (1.77 to 2.52)			2018-2021	2.04 (1.70 to 2.30)			2013-2021	1.77 (0.51 to 2.59)
Barbados	0	1990-1994	2.37 (2.12 to 2.67)	1990-1994	3.13 (2.89 to 3.37)	1990-2001	-0.08 (-1.12 to 0.34)	1990-1994	3.17 (2.94 to 3.43)	1990-2001	-0.88 (-2.32 to 0.13)	1990-2001	-0.28 (-1.69 to 0.12)
	1	1994-2001	-0.16 (-0.38 to -0.02)	1994-2001	0.16 (0.00 to 0.27)	2001-2004	3.34 (0.46 to 4.69)	1994-2001	0.03 (-0.20 to 0.19)	2001-2004	2.96 (-2.68 to 4.35)	2001-2004	1.89 (-1.57 to 2.79)
	2	2001-2019	0.99 (0.68 to 1.03)	2001-2007	1.35 (0.67 to 1.51)	2004-2012	-3.04 (-5.10 to -2.23)	2001-2007	1.31 (-0.02 to 1.49)	2004-2013	-2.86 (-5.16 to 2.12)	2004-2013	-1.29 (-3.23 to 1.17)
	3	2019-2021	1.91 (1.02 to 2.28)	2007-2019	1.65 (1.58 to 1.75)	2012-2021	-0.60 (-1.75 to 1.93)	2007-2018	1.60 (1.46 to 1.73)	2013-2021	-0.72 (-2.51 to 4.87)	2013-2021	0.30 (-0.81 to 2.77)
	4			2019-2021	2.90 (2.24 to 3.38)			2018-2021	2.33 (1.87 to 2.96)				
Belarus	0	1990-1995	1.89 (1.49 to 2.07)	1990-1995	2.30 (1.93 to 2.48)	1990-1994	5.45 (2.81 to 9.78)	1990-2001	2.37 (2.31 to 2.42)	1990-1995	4.68 (2.39 to 8.01)	1990-1993	4.59 (3.11 to 6.84)
	1	1995-1999	2.32 (1.85 to 2.57)	1995-1999	2.73 (1.67 to 2.97)	1994-2002	-3.02 (-4.36 to -1.70)	2001-2013	0.97 (0.91 to 1.03)	1995-2003	-3.97 (-5.58 to -2.10)	1993-1999	0.89 (-0.00 to 1.59)
	2	1999-2004	1.37 (1.17 to 2.29)	1999-2002	1.54 (0.95 to 2.02)	2002-2012	-8.13 (-9.72 to -7.31)	2013-2021	1.72 (1.61 to 1.86)	2003-2012	-8.69 (-12.03 to -4.65)	1999-2012	-1.46 (-1.86 to -1.21)
	3	2004-2011	0.83 (0.52 to 1.34)	2002-2014	0.97 (0.89 to 1.60)	2012-2015	4.37 (-6.40 to 6.75)			2012-2015	3.50 (-8.60 to 5.97)	2012-2021	3.13 (2.60 to 3.81)
	4	2011-2015	1.26 (0.80 to 1.49)	2014-2021	1.82 (1.70 to 1.99)	2015-2018	21.47 (16.03 to 25.15)			2015-2018	18.77 (13.16 to 22.47)		
	5	2015-2018	2.07 (1.73 to 2.30)			2018-2021	-0.94 (-8.18 to 3.44)			2018-2021	-0.01 (-9.64 to 4.79)		
	6	2018-2021	1.11 (0.54 to 1.32)										
Belgium	0	1990-1995	1.60 (1.49 to 1.77)	1990-1994	2.31 (2.13 to 2.57)	1990-1999	-1.68 (-2.63 to 0.58)	1990-1994	3.78 (3.39 to 4.05)	1990-1999	-1.91 (-2.76 to -0.13)	1990-2016	-0.04 (-0.52 to 0.08)
	1	1995-2005	1.18 (1.11 to 1.22)	1994-2005	1.74 (1.66 to 1.79)	1999-2021	-3.92 (-4.47 to -3.61)	1994-2005	1.64 (1.54 to 1.71)	1999-2021	-4.12 (-4.54 to -3.85)	2016-2021	1.60 (0.10 to 5.77)
	2	2005-2010	2.21 (2.07 to 2.50)	2005-2009	2.61 (2.35 to 2.91)			2005-2009	2.79 (2.37 to 3.24)				
	3	2010-2015	1.45 (1.26 to 1.58)	2009-2015	1.82 (1.65 to 1.95)			2009-2015	0.66 (0.43 to 0.83)				
	4	2015-2021	2.83 (2.72 to 2.93)	2015-2018	3.47 (3.13 to 3.66)			2015-2021	3.08 (2.90 to 3.26)				
	5			2018-2021	2.67 (2.21 to 2.91)								
Belize	0	1990-2001	1.88 (1.81 to 1.98)	1990-2001	2.47 (2.39 to 2.57)	1990-1993	-4.24 (-10.40 to -0.17)	1990-2001	2.40 (2.32 to 2.54)	1990-1993	-3.81 (-9.29 to -0.47)	1990-1993	-2.05 (-5.52 to -0.07)
	1	2001-2012	1.54 (1.44 to 1.61)	2001-2012	2.02 (1.92 to 2.11)	1993-1999	6.64 (5.21 to 11.10)	2001-2012	2.00 (1.87 to 2.10)	1993-1999	6.52 (5.27 to 9.37)	1993-1999	5.36 (4.50 to 7.00)
	2	2012-2021	0.84 (0.68 to 0.95)	2012-2021	1.15 (1.01 to 1.26)	1999-2006	1.17 (-0.27 to 2.50)	2012-2021	1.11 (0.94 to 1.24)	1999-2006	0.59 (-0.44 to 1.76)	1999-2006	0.97 (0.23 to 1.97)
	3					2006-2015	-3.07 (-5.91 to -2.35)			2006-2015	-2.84 (-5.01 to -2.28)	2006-2015	-1.31 (-3.01 to -0.78)
	4					2015-2021	-0.15 (-1.91 to 5.19)			2015-2021	0.05 (-1.52 to 4.23)	2015-2021	0.30 (-0.70 to 3.00)
Benin	0	1990-1994	2.57 (2.20 to 3.04)	1990-1993	4.31 (3.43 to 4.90)	1990-1993	1.77 (1.13 to 2.26)	1990-1994	3.50 (3.02 to 3.92)	1990-1992	1.37 (0.84 to 2.45)	1990-1996	2.42 (2.25 to 2.59)
	1	1994-2000	0.42 (0.22 to 0.59)	1993-2001	0.89 (0.73 to 1.04)	1993-2000	2.77 (2.65 to 3.09)	1994-2001	0.67 (0.47 to 0.86)	1992-2000	2.55 (-0.21 to 3.01)	1996-2006	1.57 (1.42 to 1.67)
	2	2000-2005	3.62 (3.40 to 3.90)	2001-2005	5.70 (5.40 to 5.96)	2000-2005	0.32 (-0.03 to 1.86)	2001-2005	5.60 (5.13 to 5.93)	2000-2005	-0.25 (-0.76 to 1.57)	2006-2012	2.13 (1.61 to 2.43)
	3	2005-2012	2.23 (2.06 to 2.48)	2005-2013	2.51 (2.39 to 2.81)	2005-2012	1.71 (0.34 to 1.95)	2005-2013	2.53 (2.38 to 2.96)	2005-2013	1.61 (1.38 to 1.89)	2012-2015	0.61 (0.40 to 2.15)
	4	2012-2019	1.57 (1.29 to 1.72)	2013-2019	1.96 (1.49 to 2.17)	2012-2015	-0.31 (-0.52 to 1.99)	2013-2019	1.96 (1.43 to 2.19)	2013-2018	-1.33 (-1.89 to -0.99)	2015-2018	-0.33 (-0.61 to 0.12)
	5	2019-2021	5.22 (4.32 to 5.88)	2019-2021	6.36 (5.32 to 7.14)	2015-2018	-1.18 (-1.48 to -0.70)	2019-2021	6.18 (5.04 to 6.97)	2018-2021	0.40 (-0.27 to 1.36)	2018-2021	2.44 (2.07 to 2.85)
	6					2018-2021	0.52 (0.13 to 1.20)						
Bermuda	0	1990-1994	1.16 (1.06 to 1.30)	1990-1994	1.97 (1.89 to 2.11)	1990-1994	-1.96 (-3.32 to 0.50)	1990-1994	1.95 (1.86 to 2.09)	1990-1996	-2.25 (-2.87 to -1.25)	1990-1996	-0.83 (-1.45 to -0.29)
	1	1994-2002	0.82 (0.68 to 0.87)	1994-1999	1.65 (1.55 to 1.73)	1994-2014	-4.05 (-4.57 to -3.88)	1994-1998	1.57 (1.43 to 1.70)	1996-1999	-6.57 (-7.47 to -4.60)	1996-1999	-3.21 (-3.72 to -0.45)
	2	2002-2010	1.00 (0.94 to 1.11)	1999-2006	1.34 (1.21 to 1.38)	2014-2021	-0.33 (-1.81 to 2.63)	1998-2007	1.32 (1.22 to 1.35)	1999-2014	-4.21 (-4.51 to -2.92)	1999-2007	-1.64 (-2.76 to -0.58)
	3	2010-2015	1.55 (1.41 to 1.69)	2006-2011	1.63 (1.47 to 1.74)			2007-2012	1.67 (1.43 to 1.76)	2014-2021	-0.56 (-1.80 to 1.91)	2007-2014	-0.46 (-1.37 to 1.67)
	4	2015-2018	2.41 (2.19 to 2.53)	2011-2015	1.96 (1.86 to 2.08)			2012-2015	1.95 (1.81 to 2.04)			2014-2021	1.37 (0.51 to 3.01)
	5	2018-2021	1.43 (1.21 to 1.57)	2015-2018	3.03 (2.92 to 3.14)			2015-2018	2.97 (2.86 to 3.08)				
	6			2018-2021	1.69 (1.55 to 1.80)			2018-2021	1.60 (1.48 to 1.71)				
Bhutan	0	1990-1995	1.02 (0.86 to 1.18)	1990-1995	1.58 (1.30 to 1.79)	1990-1995	2.24 (1.97 to 2.51)	1990-1995	1.53 (1.22 to 1.73)	1990-1995	1.97 (1.54 to 2.46)	1990-1997	1.69 (1.53 to 1.86)
	1	1995-1999	2.67 (2.41 to 2.90)	1995-2000	3.32 (3.15 to 3.59)	1995-2003	0.43 (0.16 to 0.56)	1995-2000	3.20 (3.01 to 3.51)	1995-2008	0.00 (-0.15 to 0.11)	1997-2006	0.49 (0.31 to 0.60)

Location	Segment	Incidence		Prevalence		Mortality		YLD		YLL		DALY	
		Period	APC (95%CI)	Period	APC (95%CI)	Period	APC (95%CI)	Period	APC (95%CI)	Period	APC (95%CI)	Period	APC (95%CI)
	2	1999-2006	1.27 (1.19 to 1.35)	2000-2005	1.06 (0.79 to 1.24)	2003-2014	1.00 (0.86 to 1.10)	2000-2005	1.09 (0.79 to 1.30)	2008-2021	0.84 (0.74 to 0.96)	2006-2021	1.28 (1.23 to 1.34)
	3	2006-2009	3.19 (2.92 to 3.36)	2005-2014	2.33 (2.26 to 2.44)	2014-2018	1.65 (1.34 to 2.03)	2005-2013	2.51 (2.41 to 2.71)				
	4	2009-2014	2.08 (1.93 to 2.22)	2014-2021	1.78 (1.64 to 1.89)	2018-2021	0.44 (-0.26 to 0.82)	2013-2021	1.81 (1.68 to 1.92)				
	5	2014-2021	1.55 (1.43 to 1.63)										
Bolivia (Plurinational State of)	0	1990-1994	2.24 (2.15 to 2.33)	1990-1992	3.52 (2.97 to 3.94)	1990-1994	1.44 (1.17 to 1.68)	1990-1994	3.20 (3.08 to 3.34)	1990-1994	1.29 (1.03 to 1.52)	1990-1994	1.70 (1.52 to 1.87)
	1	1994-2000	1.33 (1.27 to 1.38)	1992-1995	2.66 (1.58 to 2.80)	1994-2001	-0.59 (-0.78 to -0.46)	1994-2000	1.72 (1.64 to 1.80)	1994-2003	-0.96 (-1.09 to -0.86)	1994-2001	-0.43 (-0.56 to -0.31)
	2	2000-2005	2.59 (2.52 to 2.66)	1995-2000	1.65 (1.50 to 3.21)	2001-2013	0.20 (0.13 to 0.29)	2000-2005	3.19 (3.08 to 3.28)	2003-2013	-0.10 (-0.19 to 0.13)	2001-2015	0.52 (0.48 to 0.60)
	3	2005-2011	1.60 (1.52 to 1.66)	2000-2005	3.19 (1.67 to 3.33)	2013-2021	-0.36 (-0.53 to -0.24)	2005-2010	1.76 (1.60 to 1.88)	2013-2021	-0.49 (-0.78 to -0.36)	2015-2021	0.20 (-0.12 to 0.34)
	4	2011-2014	2.48 (2.28 to 2.59)	2005-2010	1.68 (1.47 to 2.84)			2010-2014	2.86 (2.69 to 3.11)				
	5	2014-2021	1.39 (1.32 to 1.44)	2010-2015	2.84 (1.57 to 3.05)			2014-2017	2.00 (1.71 to 2.38)				
	6			2015-2021	1.50 (1.38 to 1.64)			2017-2021	1.25 (1.03 to 1.39)				
Bosnia and Herzegovina	0	1990-1992	1.93 (1.62 to 2.40)	1990-1995	2.66 (2.53 to 2.78)	1990-1993	4.16 (2.50 to 6.39)	1990-1993	2.45 (2.12 to 2.62)	1990-1993	4.39 (2.58 to 6.82)	1990-1992	4.25 (2.94 to 5.17)
	1	1992-1995	2.59 (2.47 to 3.74)	1995-2001	3.83 (3.73 to 3.98)	1993-1998	-1.37 (-3.49 to -0.42)	1993-1996	3.14 (2.75 to 3.60)	1993-1998	-1.53 (-4.15 to -0.41)	1992-1998	1.08 (0.58 to 1.33)
	2	1995-2004	3.59 (2.47 to 3.72)	2001-2007	3.14 (3.03 to 3.26)	1998-2007	5.44 (4.98 to 6.18)	1996-2000	3.93 (3.79 to 4.13)	1998-2007	4.85 (3.27 to 6.08)	1998-2007	4.14 (3.97 to 4.33)
	3	2004-2012	2.58 (2.53 to 2.76)	2007-2014	2.58 (2.52 to 2.65)	2007-2015	1.52 (1.06 to 2.30)	2000-2004	3.33 (3.13 to 3.49)	2007-2015	0.54 (0.20 to 5.87)	2007-2015	1.47 (1.27 to 1.73)
	4	2012-2015	1.91 (1.61 to 2.16)	2014-2021	0.97 (0.92 to 1.02)	2015-2021	-0.24 (-1.43 to 0.36)	2004-2009	3.02 (2.60 to 3.10)	2015-2021	-0.50 (-2.77 to 0.17)	2015-2021	0.10 (-0.35 to 0.41)
	5	2015-2019	0.46 (0.26 to 0.58)					2009-2014	2.55 (0.96 to 2.62)				
	6	2019-2021	1.22 (0.87 to 1.46)					2014-2021	0.93 (0.87 to 0.99)				
Botswana	0	1990-1995	1.44 (1.08 to 1.60)	1990-2007	2.49 (2.41 to 2.55)	1990-1995	0.56 (-0.85 to 3.00)	1990-1995	2.11 (1.70 to 2.26)	1990-2006	-1.46 (-2.65 to -0.68)	1990-1995	0.51 (-0.76 to 2.67)
	1	1995-1999	2.20 (2.00 to 2.48)	2007-2015	3.43 (2.43 to 3.70)	1995-1998	-9.68 (-12.18 to -5.39)	1995-2000	2.72 (2.57 to 3.06)	2006-2011	11.60 (7.41 to 20.07)	1995-1998	-8.42 (-10.57 to -4.66)
	2	1999-2007	1.87 (1.60 to 1.94)	2015-2018	2.33 (1.93 to 3.44)	1998-2003	5.24 (3.54 to 10.84)	2000-2005	2.19 (1.83 to 2.33)	2011-2021	-3.60 (-4.60 to -2.69)	1998-2003	4.77 (3.35 to 9.29)
	3	2007-2014	2.40 (2.32 to 2.57)	2018-2021	1.20 (0.60 to 1.65)	2003-2006	-6.19 (-8.82 to -2.55)	2005-2010	3.09 (2.68 to 3.34)			2003-2006	-4.86 (-6.95 to -1.90)
	4	2014-2018	1.53 (1.37 to 1.73)			2006-2009	15.27 (11.48 to 18.18)	2010-2014	3.79 (3.53 to 4.07)			2006-2009	13.55 (10.51 to 15.85)
	5	2018-2021	0.52 (0.26 to 0.77)			2009-2013	2.27 (0.08 to 4.26)	2014-2018	2.42 (2.20 to 2.64)			2009-2013	2.73 (0.88 to 4.42)
	6					2013-2021	-3.80 (-4.47 to -3.25)	2018-2021	0.95 (0.65 to 1.28)			2013-2021	-2.79 (-3.32 to -2.34)
Brazil	0	1990-1995	-0.28 (-0.52 to -0.14)	1990-1995	0.07 (-0.03 to 0.18)	1990-1995	0.67 (0.39 to 1.05)	1990-1993	-0.12 (-0.36 to 0.01)	1990-1995	0.54 (0.21 to 0.99)	1990-1995	0.36 (0.17 to 0.61)
	1	1995-2000	0.72 (0.14 to 0.90)	1995-2005	1.12 (1.08 to 1.16)	1995-1998	-2.85 (-3.38 to -2.05)	1993-1996	0.38 (0.27 to 0.93)	1995-1998	-3.11 (-3.70 to -2.23)	1995-1998	-1.74 (-2.06 to -1.27)
	2	2000-2005	1.02 (-0.01 to 1.21)	2005-2010	-0.26 (-0.32 to -0.19)	1998-2001	0.71 (-0.03 to 1.31)	1996-2005	1.12 (1.10 to 1.16)	1998-2001	-0.11 (-0.85 to 0.56)	1998-2001	0.35 (-0.09 to 0.71)
	3	2005-2011	-0.05 (-0.13 to 1.12)	2010-2021	1.44 (1.41 to 1.48)	2001-2005	-1.93 (-2.80 to -1.47)	2005-2010	-0.12 (-0.18 to -0.07)	2001-2005	-2.47 (-3.38 to -1.97)	2001-2005	-1.14 (-1.64 to -0.86)
	4	2011-2021	1.23 (1.18 to 1.29)			2005-2010	0.35 (-0.18 to 1.37)	2010-2013	1.23 (1.13 to 1.35)	2005-2010	-0.10 (-0.66 to 1.04)	2005-2010	-0.05 (-0.32 to 0.51)
	5					2010-2017	-2.78 (-3.57 to -2.46)	2013-2019	1.55 (1.51 to 1.70)	2010-2017	-2.98 (-3.76 to -2.62)	2010-2015	-1.40 (-2.01 to -1.10)
	6					2017-2021	-0.72 (-1.51 to 0.63)	2019-2021	1.01 (0.81 to 1.25)	2017-2021	-0.12 (-1.06 to 1.34)	2015-2021	0.15 (-0.12 to 0.54)
Brunei Darussalam	0	1990-1994	2.55 (2.43 to 2.69)	1990-1992	3.84 (3.57 to 4.11)	1990-2005	-0.48 (-0.63 to -0.31)	1990-1992	3.69 (3.42 to 3.95)	1990-2005	-0.92 (-1.05 to -0.76)	1990-2006	-0.28 (-0.36 to -0.18)
	1	1994-2007	0.84 (0.81 to 0.87)	1992-1995	2.55 (2.42 to 2.68)	2005-2010	-3.23 (-4.62 to -2.49)	1992-1995	2.47 (2.21 to 2.61)	2005-2010	-3.32 (-4.42 to -2.70)	2006-2009	-2.05 (-2.45 to -1.07)
	2	2007-2015	1.84 (1.74 to 1.93)	1995-2001	1.26 (1.17 to 1.33)	2010-2016	-1.09 (-1.67 to 0.17)	1995-2002	1.26 (1.16 to 1.32)	2010-2016	-1.74 (-2.20 to -0.59)	2009-2021	-0.29 (-0.45 to 0.02)
	3	2015-2021	3.03 (2.88 to 3.18)	2001-2006	1.85 (1.67 to 1.94)	2016-2021	-5.53 (-6.53 to -4.72)	2002-2006	1.74 (1.50 to 1.97)	2016-2021	-4.31 (-5.29 to -3.65)		
	4			2006-2011	2.65 (2.12 to 2.75)			2006-2012	2.44 (2.29 to 2.54)				
	5			2011-2015	2.96 (2.78 to 3.44)			2012-2016	2.93 (2.68 to 3.19)				
	6			2015-2021	3.59 (3.50 to 3.72)			2016-2021	3.49 (3.39 to 3.69)				
Bulgaria	0	1990-1995	1.88 (1.77 to 2.00)	1990-1995	1.94 (1.78 to 2.11)	1990-1993	-0.26 (-3.43 to 1.40)	1990-1992	1.50 (1.21 to 1.95)	1990-1993	0.31 (-2.74 to 3.01)	1990-1993	0.84 (-0.85 to 1.84)
	1	1995-2000	3.38 (3.30 to 3.48)	1995-2000	3.96 (3.85 to 4.07)	1993-1996	6.91 (4.48 to 8.43)	1992-1995	2.24 (2.09 to 3.92)	1993-1997	4.52 (-3.46 to 6.59)	1993-1997	4.01 (3.16 to 5.04)
	2	2000-2004	1.95 (1.85 to 2.11)	2000-2008	1.83 (1.78 to 1.90)	1996-2004	-1.87 (-3.49 to -1.33)	1995-2000	3.90 (1.91 to 4.01)	1997-2004	-3.10 (-5.00 to 1.92)	1997-2004	-0.30 (-1.08 to -0.01)
	3	2004-2009	1.45 (1.35 to 1.56)	2008-2014	1.37 (1.27 to 1.46)	2004-2008	0.60 (-1.10 to 2.44)	2000-2008	1.89 (1.83 to 1.96)	2004-2007	1.91 (-5.38 to 3.25)	2004-2007	1.66 (0.51 to 2.30)
	4	2009-2015	0.96 (0.88 to 1.02)	2014-2019	0.14 (0.03 to 0.24)	2008-2013	-5.21 (-7.35 to -3.74)	2008-2014	1.37 (1.27 to 1.46)	2007-2013	-4.93 (-6.47 to 0.91)	2007-2013	-1.26 (-2.56 to -0.76)
	5	2015-2019	-0.46 (-0.56 to -0.37)	2019-2021	2.83 (2.34 to 3.17)	2013-2021	0.53 (-0.22 to 1.39)	2014-2019	0.07 (-0.03 to 0.15)	2013-2021	0.72 (-0.45 to 1.82)	2013-2021	0.63 (0.24 to 1.32)
	6	2019-2021	2.94 (2.62 to 3.21)					2019-2021	2.62 (2.33 to 2.87)				
Burkina Faso	0	1990-1993	0.58 (0.36 to 0.72)	1990-1995	1.72 (1.62 to 1.86)	1990-1999	0.63 (0.45 to 0.85)	1990-1995	1.66 (1.56 to 1.77)	1990-1993	-0.31 (-1.10 to 0.19)	1990-1993	0.14 (-0.44 to 0.53)
	1	1993-1996	1.01 (0.90 to 1.62)	1995-2006	2.70 (2.66 to 2.74)	1999-2002	-1.87 (-2.33 to 0.44)	1995-2006	2.73 (2.68 to 2.77)	1993-1998	0.93 (0.67 to 1.41)	1993-1999	1.08 (0.95 to 1.34)
	2	1996-2006	1.64 (1.61 to 1.70)	2006-2018	3.10 (3.06 to 3.13)	2002-2006	-0.47 (-1.15 to 0.23)	2006-2018	3.06 (3.02 to 3.10)	1998-2003	-1.48 (-1.97 to -1.20)	1999-2002	-0.96 (-1.21 to -0.57)
	3	2006-2013	2.06 (1.97 to 2.12)	2018-2021	4.29 (4.03 to 4.46)	2006-2009	-2.17 (-2.59 to -0.54)	2018-2021	4.14 (3.87 to 4.41)	2003-2006	-0.37 (-0.80 to -0.04)	2002-2006	0.37 (0.20 to 0.73)
	4	2013-2018	2.34 (2.26 to 2.50)			2009-2016	0.35 (0.10 to 0.90)			2006-2009	-2.72 (-3.02 to -2.24)	2006-2009	-0.95 (-1.18 to -0.61)
	5	2018-2021	3.47 (3.32 to 3.65)			2016-2021	-1.03 (-1.66 to -0.62)			2009-2016	0.58 (0.41 to 0.76)	2009-2016	1.38 (1.27 to 1.53)
	6									2016-2021	-1.29 (-1.63 to -0.96)	2016-2021	0.60 (0.32 to 0.80)
Burundi	0	1990-1996	0.55 (0.45 to 0.59)	1990-2003	1.62 (1.59 to 1.65)	1990-1996	2.38 (2.17 to 2.64)	1990-2003	1.59 (1.57 to 1.62)	1990-1996	2.45 (2.21 to 2.73)	1990-1996	2.32 (2.17 to 2.47)
	1	1996-2001	0.71 (0.65 to 0.81)	2003-2010	1.15 (1.03 to 1.22)	1996-1999	-0.34 (-0.98 to 0.34)	2003-2010	1.22 (1.09 to 1.29)	1996-1999	-0.73 (-1.43 to 0.42)	1996-1999	-0.28 (-0.55 to 0.16)
	2	2001-2011	0.43 (0.41 to 0.45)	2010-2015	1.76 (1.64 to 1.95)	1999-2008	-2.43 (-2.62 to -2.31)	2010-2015	1.78 (1.66 to 1.95)	1999-2008	-2.89 (-3.09 to -2.76)	1999-2002	-2.67 (-2.96 to -2.33)
	3	2011-2015	0.94 (0.88 to 0.99)	2015-2019	1.08 (0.87 to 1.22)	2008-2016	-0.74 (-0.99 to -0.54)	2015-2019	0.98 (0.78 to 1.13)	2008-2016	-0.95 (-1.23 to -0.75)	2002-2005	-1.56 (-1.94 to -1.28)
	4	2015-2019	0.19 (0.12 to 0.24)	2019-2021	1.95 (1.61 to 2.18)	2016-2021	0.38 (0.05 to 0.92)	2019-2021	1.69 (1.34 to 1.92)	2016-2021	0.15 (-0.20 to 0.73)	2005-2008	-2.64 (-2.86 to -2.22)
	5	2019-2021	1.29 (1.13 to 1.42)									2008-2016	-0.38 (-0.51 to -0.26)
	6											2016-2021	0.36 (0.16 to 0.67)
Cabo Verde	0	1990-1992	2.00 (1.82 to 2.25)	1990-1993	2.93 (2.66 to 3.08)	1990-1992	7.86 (6.59 to 10.00)	1990-1993	2.83 (2.58 to 2.97)	1990-1992	8.06 (7.00 to 9.75)	1990-1993	5.72 (5.17 to 6.23)
	1	1992-1996	2.66 (2.56 to 2.82)	1993-1996	3.56 (3.26 to 3.90)	1992-1998	12.70 (12.30 to 13.62)	1993-1996	3.52 (3.25 to 3.84)	1992-1998	12.66 (12.31 to 13.27)	1993-1998	8.60 (8.36 to 8.90)

Location	Segment	Incidence		Prevalence		Mortality		YLD		YLL		DALY	
		Period	APC (95%CI)	Period	APC (95%CI)	Period	APC (95%CI)	Period	APC (95%CI)	Period	APC (95%CI)	Period	APC (95%CI)
	2	1996-1999	3.65 (3.47 to 3.74)	1996-2000	4.21 (4.10 to 4.34)	1998-2001	3.01 (1.95 to 6.17)	1996-2000	4.24 (4.16 to 4.36)	1998-2001	2.86 (1.95 to 3.53)	1998-2001	3.52 (3.02 to 3.82)
	3	1999-2010	3.09 (3.06 to 3.11)	2000-2009	3.31 (3.28 to 3.34)	2001-2013	1.12 (0.92 to 1.25)	2000-2009	3.37 (3.34 to 3.40)	2001-2013	0.61 (0.47 to 0.72)	2001-2013	1.71 (1.60 to 1.77)
	4	2010-2014	1.89 (1.81 to 1.99)	2009-2013	2.27 (2.18 to 2.36)	2013-2016	5.52 (4.51 to 6.05)	2009-2013	2.30 (2.22 to 2.38)	2013-2016	3.70 (2.81 to 4.11)	2013-2016	2.53 (2.00 to 2.75)
	5	2014-2019	1.31 (1.23 to 1.41)	2013-2018	1.55 (1.47 to 1.64)	2016-2021	-0.52 (-1.04 to -0.10)	2013-2018	1.55 (1.48 to 1.63)	2016-2021	-0.72 (-1.18 to -0.38)	2016-2021	0.13 (-0.12 to 0.33)
	6	2019-2021	0.45 (0.20 to 0.76)	2018-2021	0.72 (0.58 to 0.89)			2018-2021	0.63 (0.51 to 0.75)				
Cambodia	0	1990-2000	2.36 (2.32 to 2.39)	1990-1993	3.74 (3.50 to 4.11)	1990-1992	-0.05 (-0.30 to 0.27)	1990-1994	3.62 (3.38 to 4.05)	1990-1992	-0.20 (-0.50 to 0.25)	1990-1992	0.60 (0.40 to 0.84)
	1	2000-2005	2.91 (2.78 to 3.14)	1993-2005	3.17 (3.13 to 3.23)	1992-1995	0.75 (0.50 to 0.90)	1994-2011	3.21 (3.16 to 3.24)	1992-1995	0.67 (0.28 to 0.85)	1992-1995	1.26 (1.09 to 1.38)
	2	2005-2010	2.47 (2.27 to 2.58)	2005-2010	2.91 (2.66 to 3.04)	1995-2000	-0.34 (-0.46 to -0.23)	2011-2014	4.00 (3.62 to 4.15)	1995-2000	-0.55 (-0.84 to -0.43)	1995-2000	0.32 (0.24 to 0.41)
	3	2010-2014	3.43 (3.24 to 3.70)	2010-2014	3.80 (3.59 to 4.07)	2000-2006	-0.96 (-1.05 to -0.87)	2014-2021	3.00 (2.86 to 3.09)	2000-2006	-1.36 (-1.51 to -1.21)	2000-2006	-0.05 (-0.13 to 0.01)
	4	2014-2021	2.44 (2.35 to 2.51)	2014-2021	2.98 (2.87 to 3.06)	2006-2010	0.51 (0.40 to 0.63)			2006-2010	-0.16 (-0.32 to 0.23)	2006-2010	0.94 (0.86 to 1.04)
	5					2010-2019	1.84 (1.79 to 1.90)			2010-2019	1.47 (1.41 to 1.59)	2010-2019	2.18 (2.15 to 2.23)
	6					2019-2021	0.52 (0.15 to 1.02)			2019-2021	0.47 (0.01 to 1.05)	2019-2021	1.26 (1.01 to 1.60)
Cameroon	0	1990-1995	0.86 (0.71 to 1.03)	1990-1995	1.47 (1.29 to 1.65)	1990-1996	2.27 (1.96 to 2.45)	1990-1995	1.42 (1.12 to 1.74)	1990-1996	2.27 (2.15 to 2.38)	1990-1996	2.18 (2.07 to 2.28)
	1	1995-2000	3.71 (3.57 to 3.85)	1995-2000	4.83 (4.68 to 4.98)	1996-2000	3.66 (3.36 to 4.14)	1995-2000	4.99 (4.69 to 5.26)	1996-2000	3.69 (3.51 to 3.92)	1996-2000	4.12 (3.97 to 4.29)
	2	2000-2005	1.54 (1.27 to 1.71)	2000-2005	2.09 (1.86 to 2.27)	2000-2003	1.52 (0.98 to 2.09)	2000-2021	2.31 (2.27 to 2.35)	2000-2003	1.35 (1.05 to 1.54)	2000-2003	1.42 (1.15 to 1.61)
	3	2005-2011	2.46 (2.28 to 2.79)	2005-2010	3.06 (2.82 to 3.45)	2003-2013	0.28 (0.16 to 0.37)			2003-2014	0.11 (0.06 to 0.16)	2003-2014	0.66 (0.60 to 0.71)
	4	2011-2021	1.52 (1.44 to 1.59)	2010-2017	2.07 (1.75 to 2.20)	2013-2021	-0.91 (-1.09 to -0.76)			2014-2018	-1.62 (-1.91 to -1.35)	2014-2017	-0.69 (-0.87 to -0.28)
	5			2017-2021	2.62 (2.34 to 3.13)					2018-2021	-0.58 (-0.94 to 0.02)	2017-2021	0.24 (0.03 to 0.68)
Canada	0	1990-1994	3.44 (3.33 to 3.55)	1990-1994	4.68 (4.50 to 4.87)	1990-1994	2.85 (2.00 to 4.57)	1990-1994	4.61 (4.43 to 4.79)	1990-1995	2.51 (1.82 to 3.36)	1990-1995	3.11 (2.78 to 3.46)
	1	1994-2000	2.24 (2.13 to 2.33)	1994-2000	2.71 (2.57 to 2.83)	1994-1999	0.18 (-1.41 to 0.87)	1994-2000	2.67 (2.54 to 2.79)	1995-1999	-1.07 (-2.37 to -0.06)	1995-1999	0.12 (-0.66 to 0.63)
	2	2000-2005	3.37 (2.33 to 3.50)	2000-2005	4.82 (2.86 to 4.96)	1999-2002	3.53 (1.39 to 4.60)	2000-2005	4.66 (3.10 to 4.80)	1999-2002	2.86 (0.83 to 3.84)	1999-2002	3.42 (2.40 to 3.89)
	3	2005-2011	2.21 (2.03 to 3.37)	2005-2011	2.85 (2.61 to 4.76)	2002-2008	-4.38 (-5.25 to -3.77)	2005-2011	2.78 (2.53 to 4.55)	2002-2008	-4.40 (-5.13 to -3.63)	2002-2008	-0.95 (-1.24 to -0.51)
	4	2011-2016	2.47 (2.27 to 2.63)	2011-2016	3.21 (2.94 to 3.43)	2008-2013	-8.99 (-10.29 to -8.00)	2011-2016	3.19 (2.88 to 3.42)	2008-2013	-8.98 (-9.92 to -4.78)	2008-2013	-2.37 (-3.47 to -1.88)
	5	2016-2019	3.26 (3.02 to 3.43)	2016-2019	4.30 (3.98 to 4.52)	2013-2021	-1.81 (-2.28 to -1.27)	2016-2019	4.10 (3.78 to 4.32)	2013-2018	-0.55 (-8.62 to 0.86)	2013-2019	2.30 (0.61 to 2.75)
	6	2019-2021	8.10 (7.71 to 8.45)	2019-2021	9.94 (9.41 to 10.41)			2019-2021	9.68 (9.21 to 10.14)	2018-2021	-2.86 (-5.01 to -1.42)	2019-2021	6.12 (3.59 to 7.49)
Central African Republic	0	1990-1994	1.53 (1.02 to 1.76)	1990-1995	2.18 (1.82 to 2.38)	1990-1992	-0.25 (-0.73 to 0.29)	1990-1993	1.89 (1.24 to 2.33)	1990-1999	1.56 (1.27 to 1.90)	1990-1992	0.28 (-0.46 to 1.77)
	1	1994-2011	1.97 (1.94 to 2.01)	1995-1999	3.12 (2.84 to 3.43)	1992-1995	2.33 (1.94 to 2.64)	1993-2010	2.69 (2.65 to 2.75)	1999-2021	-0.39 (-0.47 to -0.30)	1992-1998	2.10 (0.41 to 2.90)
	2	2011-2019	0.93 (0.79 to 1.03)	1999-2010	2.64 (2.51 to 2.69)	1995-1999	1.12 (0.64 to 1.40)	2010-2021	1.55 (1.48 to 1.64)			1998-2013	0.47 (0.36 to 0.68)
	3	2019-2021	1.77 (1.18 to 2.05)	2010-2021	1.60 (1.53 to 1.67)	1999-2006	0.11 (-0.07 to 0.27)					2013-2021	-0.01 (-0.39 to 0.19)
	4					2006-2009	-0.98 (-1.27 to -0.51)						
	5					2009-2013	0.44 (0.10 to 0.88)						
	6					2013-2021	-0.60 (-0.75 to -0.48)						
Chad	0	1990-1999	3.00 (2.97 to 3.05)	1990-1999	3.67 (3.64 to 3.70)	1990-1995	2.85 (2.63 to 3.05)	1990-1999	3.68 (3.65 to 3.72)	1990-1995	2.79 (2.40 to 3.06)	1990-1995	3.06 (2.76 to 3.27)
	1	1999-2003	2.61 (2.44 to 2.80)	1999-2003	3.13 (3.01 to 3.29)	1995-2000	4.60 (4.42 to 5.03)	1999-2003	3.09 (3.00 to 3.27)	1995-2000	4.77 (4.50 to 5.39)	1995-2000	4.42 (4.21 to 4.87)
	2	2003-2009	2.21 (2.11 to 2.29)	2003-2009	2.73 (2.65 to 2.78)	2000-2003	3.42 (2.46 to 3.76)	2003-2009	2.72 (2.65 to 2.78)	2000-2003	3.47 (1.59 to 3.92)	2000-2003	3.30 (2.17 to 3.70)
	3	2009-2014	1.75 (1.65 to 1.83)	2009-2014	2.00 (1.95 to 2.06)	2003-2010	1.08 (0.93 to 1.22)	2009-2014	2.05 (1.99 to 2.11)	2003-2010	0.88 (0.66 to 1.08)	2003-2010	1.46 (1.26 to 1.61)
	4	2014-2019	0.09 (0.03 to 0.16)	2014-2019	0.62 (0.56 to 0.67)	2010-2021	0.22 (0.15 to 0.29)	2014-2019	0.52 (0.46 to 0.58)	2010-2021	0.16 (0.05 to 0.25)	2010-2021	0.52 (0.44 to 0.59)
	5	2019-2021	2.32 (2.09 to 2.50)	2019-2021	2.89 (2.69 to 3.05)			2019-2021	2.74 (2.54 to 2.90)				
Chile	0	1990-2006	3.08 (3.00 to 3.17)	1990-2005	3.40 (3.28 to 3.52)	1990-1995	4.70 (3.46 to 6.17)	1990-2005	3.42 (3.31 to 3.53)	1990-1995	4.18 (2.58 to 6.33)	1990-1995	3.99 (3.27 to 4.85)
	1	2006-2015	2.27 (2.07 to 2.43)	2005-2015	2.48 (2.27 to 3.38)	1995-1998	-8.70 (-10.21 to -4.67)	2005-2015	2.46 (2.22 to 3.33)	1995-1998	-11.15 (-13.47 to -7.18)	1995-1998	-5.02 (-6.23 to -2.91)
	2	2015-2019	-0.03 (-0.74 to 0.54)	2015-2019	0.28 (-0.49 to 2.55)	1998-2004	2.95 (1.05 to 5.37)	2015-2019	0.20 (-0.63 to 2.50)	1998-2003	2.56 (0.72 to 7.07)	1998-2003	3.18 (2.27 to 5.45)
	3	2019-2021	3.19 (1.65 to 4.12)	2019-2021	2.63 (0.81 to 3.60)	2004-2008	-4.60 (-7.14 to 2.59)	2019-2021	2.62 (0.80 to 3.62)	2003-2016	-1.98 (-2.72 to -1.33)	2003-2016	0.60 (0.24 to 0.99)
	4					2008-2016	0.24 (-4.38 to 2.70)			2016-2021	-7.62 (-11.69 to -5.38)	2016-2021	-1.93 (-4.92 to -0.73)
	5					2016-2019	-11.60 (-13.68 to 0.44)						
	6					2019-2021	-1.81 (-8.49 to 2.79)						
China	0	1990-1993	3.73 (3.47 to 4.04)	1990-1996	1.61 (1.40 to 1.77)	1990-1997	0.69 (0.17 to 1.04)	1990-1996	1.24 (1.13 to 1.34)	1990-1997	0.47 (-0.08 to 0.76)	1990-1996	0.83 (0.52 to 0.99)
	1	1993-1999	1.01 (0.86 to 1.12)	1996-1999	3.85 (3.53 to 4.11)	1997-2004	2.07 (1.78 to 2.47)	1996-1999	2.88 (2.51 to 3.05)	1997-2004	1.53 (1.31 to 1.92)	1996-1999	2.19 (1.66 to 2.45)
	2	1999-2005	-0.11 (-0.34 to 0.01)	1999-2007	0.32 (0.18 to 0.41)	2004-2007	-4.66 (-5.08 to -3.84)	1999-2004	1.53 (1.37 to 1.69)	2004-2007	-4.85 (-5.24 to -4.32)	1999-2004	1.50 (-1.99 to 1.66)
	3	2005-2009	0.79 (0.35 to 1.11)	2007-2018	1.48 (1.40 to 1.55)	2007-2014	-2.09 (-2.67 to -1.81)	2004-2012	0.51 (0.46 to 0.72)	2007-2013	-2.88 (-3.18 to -2.48)	2004-2007	-1.85 (-2.06 to -0.69)
	4	2009-2015	1.41 (1.29 to 1.70)	2018-2021	2.49 (2.07 to 3.12)	2014-2021	-1.17 (-1.57 to -0.16)	2012-2015	0.10 (-0.07 to 0.36)	2013-2021	-1.38 (-1.66 to -0.99)	2007-2015	-0.76 (-0.88 to 0.44)
	5	2015-2021	0.77 (0.64 to 0.88)					2015-2019	2.99 (2.84 to 3.19)			2015-2021	1.49 (1.27 to 1.84)
	6							2019-2021	2.17 (1.79 to 2.56)				
Colombia	0	1990-1994	2.83 (1.92 to 4.26)	1990-1994	3.10 (2.07 to 4.86)	1990-1995	1.05 (0.31 to 2.17)	1990-1994	3.26 (2.29 to 4.89)	1990-1995	0.92 (0.22 to 1.85)	1990-1995	1.69 (1.34 to 2.09)
	1	1994-2000	-1.50 (-2.53 to -0.97)	1994-2000	-1.47 (-2.48 to -0.91)	1995-2004	-1.45 (-1.99 to -1.05)	1994-2000	-1.51 (-2.55 to -0.98)	1995-2004	-2.31 (-2.76 to -1.92)	1995-1998	-2.74 (-3.23 to -1.96)
	2	2000-2019	0.77 (0.67 to 0.87)	2000-2019	1.05 (0.96 to 1.14)	2004-2011	-6.34 (-7.66 to -5.66)	2000-2019	1.03 (0.93 to 1.12)	2004-2012	-6.24 (-7.10 to -5.74)	1998-2003	-0.91 (-1.28 to -0.12)
	3	2019-2021	7.63 (5.03 to 9.23)	2019-2021	8.49 (5.67 to 10.28)	2011-2017	-3.20 (-4.46 to -1.97)	2019-2021	8.15 (5.47 to 9.87)	2012-2017	-2.25 (-4.08 to -0.97)	2003-2011	-2.35 (-2.89 to -2.07)
	4					2017-2021	1.74 (0.01 to 4.57)			2017-2021	2.60 (0.86 to 5.45)	2011-2018	-0.28 (-0.75 to 0.26)
	5											2018-2021	4.77 (3.52 to 6.75)
Comoros	0	1990-2001	0.94 (0.89 to 0.98)	1990-2000	1.85 (1.79 to 1.90)	1990-2000	0.14 (-0.01 to 0.33)	1990-2000	1.84 (1.80 to 1.88)	1990-1997	0.25 (-0.21 to 1.01)	1990-1997	0.61 (0.24 to 1.48)
	1	2001-2012	1.58 (1.55 to 1.63)	2000-2008	2.71 (2.65 to 2.81)	2000-2004	-1.34 (-1.90 to -0.62)	2000-2009	2.66 (2.61 to 2.72)	1997-2010	-1.08 (-1.48 to -0.90)	1997-2006	-0.41 (-1.60 to -0.13)
	2	2012-2015	1.08 (0.73 to 1.32)	2008-2014	2.22 (2.08 to 2.32)	2004-2013	-0.24 (-0.67 to -0.00)	2009-2014	2.17 (2.03 to 2.28)	2010-2021	0.29 (0.03 to 0.66)	2006-2021	0.51 (0.36 to 0.71)
	3	2015-2019	0.18 (-0.05 to 0.29)	2014-2019	0.88 (0.64 to 0.99)	2013-2021	0.34 (0.11 to 1.01)	2014-2019	0.79 (0.59 to 0.88)				

Location	Segment	Incidence		Prevalence		Mortality		YLD		YLL		DALY	
		Period	APC (95%CI)	Period	APC (95%CI)	Period	APC (95%CI)	Period	APC (95%CI)	Period	APC (95%CI)	Period	APC (95%CI)
	4	2019-2021	1.18 (0.80 to 1.43)	2019-2021	1.94 (1.46 to 2.23)			2019-2021	1.61 (1.21 to 1.85)				
Congo	0	1990-1995	1.26 (1.07 to 1.37)	1990-1994	2.02 (1.61 to 2.27)	1990-1992	0.45 (-0.43 to 1.64)	1990-1995	2.10 (1.81 to 2.28)	1990-1992	0.20 (-0.92 to 1.71)	1990-1992	0.45 (-0.55 to 1.83)
	1	1995-2000	2.04 (1.94 to 2.29)	1994-2010	2.80 (2.77 to 2.85)	1992-1996	3.69 (3.27 to 4.39)	1995-2010	2.81 (2.77 to 2.87)	1992-1996	4.39 (3.87 to 5.26)	1992-1996	4.07 (3.57 to 5.01)
	2	2000-2005	1.79 (1.57 to 1.89)	2010-2014	1.91 (1.66 to 2.24)	1996-1999	0.10 (-1.12 to 1.36)	2010-2014	1.94 (1.62 to 2.30)	1996-1999	-0.05 (-1.40 to 1.52)	1996-1999	0.40 (-0.90 to 1.79)
	3	2005-2010	2.25 (2.14 to 2.45)	2014-2019	1.06 (0.83 to 1.21)	1999-2002	-1.69 (-2.16 to 0.62)	2014-2019	1.01 (0.70 to 1.18)	1999-2002	-2.26 (-2.84 to 0.80)	1999-2002	-1.34 (-1.92 to 1.32)
	4	2010-2014	1.22 (1.09 to 1.40)	2019-2021	3.20 (2.68 to 3.57)	2002-2005	0.94 (-0.56 to 1.33)	2019-2021	3.01 (2.42 to 3.43)	2002-2005	1.19 (-0.79 to 1.69)	2002-2006	1.19 (-0.42 to 1.98)
	5	2014-2019	0.61 (0.46 to 0.71)			2005-2021	-0.51 (-0.61 to -0.44)			2005-2021	-0.73 (-0.85 to -0.63)	2006-2021	-0.16 (-0.33 to 0.08)
	6	2019-2021	2.11 (1.74 to 2.34)										
Cook Islands	0	1990-1996	1.69 (1.53 to 1.74)	1990-2000	2.62 (2.59 to 2.65)	1990-2002	-1.06 (-1.12 to -1.00)	1990-2000	2.53 (2.48 to 2.57)	1990-2002	-1.48 (-1.54 to -1.43)	1990-2002	-0.51 (-0.63 to -0.40)
	1	1996-1999	1.92 (1.74 to 1.99)	2000-2010	1.89 (1.86 to 1.92)	2002-2005	0.13 (-0.25 to 0.35)	2000-2010	1.81 (1.77 to 1.86)	2002-2005	-0.36 (-0.66 to -0.19)	2002-2005	0.28 (-0.77 to 0.43)
	2	1999-2006	1.12 (1.06 to 1.16)	2010-2019	1.35 (1.29 to 1.39)	2005-2008	-2.88 (-3.11 to -2.54)	2010-2019	1.40 (1.30 to 1.45)	2005-2008	-2.74 (-2.96 to -2.51)	2005-2008	-1.28 (-1.49 to 0.28)
	3	2006-2010	1.49 (1.39 to 1.61)	2019-2021	2.36 (1.97 to 2.55)	2008-2014	-2.14 (-2.25 to -1.75)	2019-2021	2.24 (1.80 to 2.46)	2008-2011	-1.55 (-1.81 to -1.34)	2008-2011	-0.44 (-1.22 to -0.17)
	4	2010-2019	1.15 (1.11 to 1.18)			2014-2021	-0.08 (-0.23 to 0.09)			2011-2014	-2.48 (-2.69 to -2.22)	2011-2014	-1.09 (-1.30 to 0.55)
	5	2019-2021	1.92 (1.66 to 2.06)							2014-2021	-0.17 (-0.29 to -0.05)	2014-2021	0.52 (0.31 to 0.84)
Costa Rica	0	1990-1994	3.83 (3.35 to 4.52)	1990-1994	4.45 (3.96 to 4.99)	1990-2004	-1.55 (-2.77 to 3.26)	1990-1994	4.65 (4.05 to 5.52)	1990-2013	-3.51 (-4.23 to -2.90)	1990-1996	1.95 (1.09 to 3.15)
	1	1994-2000	0.64 (0.20 to 0.89)	1994-2000	1.00 (0.49 to 1.26)	2004-2012	-5.74 (-16.40 to 5.15)	1994-2000	0.85 (0.09 to 1.18)	2013-2021	9.60 (5.94 to 14.89)	1996-2000	-3.83 (-6.19 to -2.12)
	2	2000-2015	1.59 (1.52 to 1.68)	2000-2014	1.90 (1.81 to 2.03)	2012-2021	9.82 (5.66 to 16.05)	2000-2014	1.89 (1.79 to 2.05)			2000-2003	3.63 (0.85 to 5.45)
	3	2015-2019	-1.00 (-1.86 to -0.50)	2014-2019	-0.05 (-0.66 to 0.32)			2014-2019	-0.14 (-0.87 to 0.27)			2003-2011	-1.04 (-3.60 to -0.25)
	4	2019-2021	5.17 (3.38 to 6.46)	2019-2021	4.89 (3.22 to 6.06)			2019-2021	4.88 (3.16 to 6.13)			2011-2021	3.05 (2.35 to 3.95)
Croatia	0	1990-1996	1.16 (1.04 to 1.29)	1990-1995	1.21 (1.03 to 1.40)	1990-1992	9.07 (5.70 to 11.93)	1990-1995	1.25 (1.07 to 1.41)	1990-1997	3.01 (2.16 to 3.97)	1990-1997	2.29 (2.00 to 2.65)
	1	1996-1999	3.81 (3.54 to 4.04)	1995-2000	3.48 (3.35 to 3.62)	1992-1997	2.65 (0.85 to 3.43)	1995-2000	3.42 (2.58 to 3.57)	1997-2000	-12.09 (-13.53 to -8.31)	1997-2000	-3.38 (-4.00 to -1.77)
	2	1999-2004	1.37 (1.18 to 1.59)	2000-2019	1.04 (1.00 to 1.06)	1997-2000	-13.12 (-14.29 to -10.87)	2000-2005	1.20 (1.06 to 3.48)	2000-2010	-0.21 (-0.67 to 0.59)	2000-2010	0.59 (0.39 to 1.16)
	3	2004-2019	0.83 (0.78 to 0.87)	2019-2021	2.26 (1.46 to 2.59)	2000-2008	1.37 (0.71 to 2.10)	2005-2019	0.99 (0.72 to 1.03)	2010-2013	-8.25 (-9.81 to -5.48)	2010-2013	-2.24 (-3.14 to -0.75)
	4	2019-2021	2.27 (1.57 to 2.58)			2008-2013	-5.35 (-6.71 to -4.15)	2019-2021	2.33 (1.69 to 2.69)	2013-2017	10.44 (8.47 to 13.24)	2013-2017	4.05 (3.15 to 5.68)
	5					2013-2017	11.56 (10.02 to 13.02)			2017-2021	-2.02 (-4.16 to -0.14)	2017-2021	0.34 (-1.08 to 1.13)
	6					2017-2021	-1.68 (-3.23 to -0.29)						
Cuba	0	1990-1994	1.77 (1.50 to 2.07)	1990-1994	2.17 (1.94 to 2.41)	1990-1996	-2.21 (-3.25 to -1.20)	1990-1994	2.14 (1.87 to 2.52)	1990-1996	-1.61 (-2.60 to -0.56)	1990-1996	-0.20 (-0.98 to 0.71)
	1	1994-2000	0.61 (0.27 to 0.79)	1994-2005	1.29 (1.23 to 1.34)	1996-2002	-13.47 (-14.44 to -12.18)	1994-2005	1.19 (1.12 to 1.26)	1996-2002	-13.07 (-13.95 to -12.15)	1996-2001	-5.96 (-8.89 to -4.75)
	2	2000-2004	1.09 (0.78 to 1.37)	2005-2010	0.53 (0.25 to 0.79)	2002-2005	6.95 (-10.59 to 8.76)	2005-2011	0.63 (0.26 to 0.78)	2002-2009	2.75 (1.85 to 3.87)	2001-2021	0.61 (0.36 to 0.89)
	3	2004-2011	0.49 (0.22 to 0.63)	2010-2013	1.61 (0.69 to 2.11)	2005-2010	1.10 (-1.36 to 3.09)	2011-2018	2.30 (2.18 to 2.52)	2009-2014	-5.66 (-8.57 to -4.07)		
	4	2011-2018	1.95 (1.84 to 2.34)	2013-2018	2.43 (2.27 to 2.80)	2010-2014	-6.19 (-8.52 to -3.72)	2018-2021	1.32 (0.73 to 1.69)	2014-2021	-0.13 (-1.28 to 2.07)		
	5	2018-2021	1.14 (0.35 to 1.51)	2018-2021	1.35 (0.96 to 1.66)	2014-2021	-0.08 (-1.28 to 2.04)						
Cyprus	0	1990-1993	0.47 (0.30 to 0.70)	1990-1993	1.11 (1.04 to 1.25)	1990-1992	5.15 (0.66 to 8.70)	1990-1995	1.00 (0.92 to 1.08)	1990-1992	4.02 (0.53 to 6.89)	1990-1992	3.53 (1.15 to 5.15)
	1	1993-1996	0.12 (-0.94 to 0.22)	1993-1996	0.74 (0.60 to 0.83)	1992-1999	-3.57 (-4.69 to -2.66)	1995-2000	0.08 (0.01 to 0.14)	1992-1999	-3.09 (-4.04 to -2.42)	1992-1999	-2.36 (-2.92 to -1.83)
	2	1996-2001	-0.90 (-0.99 to -0.17)	1996-2000	0.10 (0.04 to 0.15)	1999-2006	-7.19 (-9.02 to -6.44)	2000-2010	0.97 (0.94 to 1.00)	1999-2006	-6.60 (-8.25 to -5.89)	1999-2006	-4.48 (-5.54 to -4.04)
	3	2001-2010	0.00 (-0.05 to 0.13)	2000-2010	1.06 (1.03 to 1.09)	2006-2021	-2.49 (-2.76 to -2.20)	2010-2015	0.33 (0.25 to 0.46)	2006-2016	-3.70 (-5.31 to -3.32)	2006-2021	-1.74 (-1.93 to -1.56)
	4	2010-2015	-0.37 (-0.52 to -0.14)	2010-2015	0.36 (0.28 to 0.50)			2015-2019	-0.38 (-0.50 to -0.25)	2016-2021	-2.03 (-3.15 to 0.54)		
	5	2015-2019	-0.83 (-1.02 to -0.69)	2015-2019	-0.31 (-0.42 to -0.19)			2019-2021	1.41 (1.16 to 1.62)				
	6	2019-2021	1.23 (0.88 to 1.52)	2019-2021	1.54 (1.31 to 1.74)								
Czechia	0	1990-1996	0.99 (0.80 to 1.13)	1990-1995	1.19 (0.88 to 1.38)	1990-1996	-12.44 (-17.14 to -9.12)	1990-1995	1.19 (0.87 to 1.42)	1990-1996	-12.35 (-16.87 to -9.23)	1990-1995	-4.71 (-6.12 to -3.80)
	1	1996-1999	3.23 (2.53 to 3.46)	1995-2000	2.69 (2.38 to 2.99)	1996-2021	4.59 (4.05 to 5.16)	1995-2000	2.72 (2.48 to 3.08)	1996-2021	3.57 (3.08 to 4.12)	1995-2010	2.00 (1.63 to 2.25)
	2	1999-2006	0.97 (0.82 to 1.12)	2000-2005	0.75 (0.59 to 1.04)			2000-2005	0.74 (0.47 to 0.95)			2010-2013	5.36 (3.35 to 6.26)
	3	2006-2009	2.92 (1.09 to 3.12)	2005-2015	2.48 (2.09 to 2.55)			2005-2014	2.58 (2.49 to 2.70)			2013-2021	1.78 (0.99 to 2.18)
	4	2009-2015	2.41 (2.15 to 2.57)	2015-2019	1.32 (0.97 to 2.48)			2014-2021	1.70 (1.52 to 1.84)				
	5	2015-2019	1.03 (0.65 to 1.31)	2019-2021	2.43 (1.65 to 2.86)								
	6	2019-2021	2.55 (1.91 to 3.00)										
CÃ□Â´te d'Ivoire	0	1990-1994	1.81 (1.51 to 2.00)	1990-1995	2.70 (2.53 to 2.80)	1990-1992	-0.92 (-1.55 to 0.06)	1990-1995	2.81 (2.60 to 2.91)	1990-1992	-1.46 (-2.01 to -0.74)	1990-1992	-0.21 (-0.62 to 0.39)
	1	1994-2010	2.41 (2.38 to 2.44)	1995-2002	3.09 (3.02 to 3.32)	1992-1997	1.98 (1.67 to 2.42)	1995-2002	3.09 (3.02 to 3.31)	1992-1997	1.77 (1.55 to 2.06)	1992-1997	2.15 (1.98 to 2.38)
	2	2010-2021	1.52 (1.48 to 1.56)	2002-2009	2.77 (2.68 to 2.89)	1997-2001	5.51 (5.06 to 6.13)	2002-2009	2.84 (2.68 to 2.94)	1997-2001	5.74 (5.43 to 6.05)	1997-2001	4.91 (4.68 to 5.15)
	3			2009-2013	2.33 (2.10 to 2.55)	2001-2006	2.88 (2.60 to 3.24)	2009-2013	2.34 (2.06 to 2.58)	2001-2006	2.92 (2.70 to 3.14)	2001-2006	2.91 (2.75 to 3.08)
	4			2013-2021	1.92 (1.82 to 1.97)	2006-2011	0.31 (-0.13 to 0.71)	2013-2021	1.89 (1.77 to 1.95)	2006-2011	0.07 (-0.24 to 0.25)	2006-2011	0.90 (0.62 to 1.03)
	5					2011-2014	1.23 (-0.52 to 1.55)			2011-2014	2.09 (1.58 to 2.39)	2011-2014	2.08 (1.59 to 2.31)
	6					2014-2021	-0.82 (-1.07 to -0.62)			2014-2021	-1.13 (-1.30 to -0.99)	2014-2021	-0.07 (-0.20 to 0.03)
Democratic People's Republic of Korea	0	1990-1995	2.13 (2.06 to 2.31)	1990-1994	2.55 (2.44 to 2.62)	1990-2001	0.87 (0.83 to 0.90)	1990-1994	2.50 (2.41 to 2.63)	1990-1994	0.81 (0.61 to 0.90)	1990-2009	1.50 (1.49 to 1.50)
	1	1995-2004	1.97 (1.91 to 2.01)	1994-2005	2.10 (2.09 to 2.13)	2001-2009	1.23 (1.18 to 1.30)	1994-2005	2.13 (2.11 to 2.15)	1994-2005	1.01 (0.97 to 1.04)	2009-2012	0.80 (0.74 to 0.85)
	2	2004-2010	1.69 (1.54 to 1.75)	2005-2010	1.93 (1.79 to 1.99)	2009-2012	-0.26 (-0.38 to -0.13)	2005-2010	1.92 (1.77 to 1.99)	2005-2009	1.16 (1.08 to 1.27)	2012-2018	0.41 (0.37 to 0.43)
	3	2010-2015	2.09 (2.02 to 2.28)	2010-2015	2.28 (2.23 to 2.42)	2012-2017	-1.54 (-1.70 to -1.45)	2010-2015	2.26 (2.21 to 2.42)	2009-2012	-0.38 (-0.50 to -0.29)	2018-2021	1.00 (0.93 to 1.07)
	4	2015-2018	1.73 (1.63 to 1.87)	2015-2018	1.87 (1.79 to 1.99)	2017-2021	-1.02 (-1.17 to -0.75)	2015-2018	1.87 (1.77 to 2.00)	2012-2017	-1.33 (-1.44 to -1.26)		
	5	2018-2021	2.50 (2.36 to 2.79)	2018-2021	2.65 (2.54 to 2.79)			2018-2021	2.64 (2.52 to 2.86)	2017-2021	-0.89 (-1.00 to -0.72)		
Democratic Republic of the Congo	0	1990-1992	0.57 (0.48 to 0.71)	1990-1997	1.86 (1.81 to 1.89)	1990-1999	0.10 (-0.57 to 0.30)	1990-1997	1.76 (1.73 to 1.79)	1990-1998	-0.00 (-1.03 to 0.26)	1990-1998	0.32 (-0.13 to 0.53)
	1	1992-1996	0.83 (0.79 to 0.91)	1997-2001	2.18 (2.05 to 2.29)	1999-2021	0.48 (0.42 to 0.59)	1997-2004	2.31 (2.26 to 2.34)	1998-2021	0.36 (0.30 to 0.64)	1998-2021	0.82 (0.77 to 0.88)
	2	1996-2005	1.65 (1.62 to 1.66)	2001-2009	2.42 (2.39 to 2.48)			2004-2009	2.51 (2.45 to 2.62)				

Location	Segment	Incidence		Prevalence		Mortality		YLD		YLL		DALY	
		Period	APC (95%CI)	Period	APC (95%CI)	Period	APC (95%CI)	Period	APC (95%CI)	Period	APC (95%CI)	Period	APC (95%CI)
	3	2005-2010	1.86 (1.81 to 1.95)	2009-2014	2.28 (2.20 to 2.32)			2009-2014	2.28 (2.19 to 2.33)				
	4	2010-2014	1.58 (1.52 to 1.67)	2014-2019	1.76 (1.72 to 1.80)			2014-2019	1.80 (1.75 to 1.85)				
	5	2014-2019	1.40 (1.34 to 1.44)	2019-2021	3.86 (3.73 to 3.98)			2019-2021	3.64 (3.49 to 3.75)				
	6	2019-2021	2.88 (2.76 to 2.99)										
Denmark	0	1990-1995	1.93 (1.69 to 2.04)	1990-2005	2.20 (2.17 to 2.23)	1990-1993	1.51 (-2.09 to 6.63)	1990-1999	2.28 (2.22 to 2.35)	1990-1993	1.28 (-1.11 to 5.51)	1990-1993	1.60 (0.31 to 3.99)
	1	1995-2005	2.11 (2.07 to 2.24)	2005-2010	3.81 (3.69 to 3.95)	1993-1996	-5.04 (-7.31 to 12.12)	1999-2005	1.57 (1.46 to 1.67)	1993-1996	-6.70 (-8.83 to -3.76)	1993-1996	-3.18 (-4.36 to -1.63)
	2	2005-2010	3.47 (3.35 to 3.54)	2010-2015	2.90 (2.58 to 3.08)	1996-2000	11.63 (-2.54 to 15.33)	2005-2010	3.83 (3.71 to 3.96)	1996-1999	14.53 (8.96 to 16.68)	1996-1999	8.96 (6.37 to 10.15)
	3	2010-2015	2.36 (2.21 to 2.49)	2015-2021	3.60 (3.47 to 3.79)	2000-2021	-2.04 (-3.03 to -1.55)	2010-2015	2.83 (2.68 to 2.96)	1999-2021	-2.50 (-2.76 to -2.28)	1999-2013	-0.34 (-0.70 to -0.12)
	4	2015-2021	3.24 (3.13 to 3.36)					2015-2018	5.00 (4.74 to 5.19)			2013-2021	1.52 (0.80 to 2.84)
	5							2018-2021	3.57 (3.25 to 3.78)				
Djibouti	0	1990-1995	0.90 (0.76 to 0.98)	1990-1995	1.83 (1.60 to 1.95)	1990-2005	1.28 (1.21 to 1.33)	1990-1996	1.87 (1.74 to 1.95)	1990-2005	1.22 (1.13 to 1.31)	1990-2005	1.39 (1.31 to 1.47)
	1	1995-2006	1.44 (1.38 to 1.47)	1995-2005	2.28 (2.13 to 2.34)	2005-2021	0.12 (0.05 to 0.18)	1996-2005	2.23 (2.02 to 2.30)	2005-2021	-0.18 (-0.28 to -0.09)	2005-2021	0.31 (0.23 to 0.38)
	2	2006-2009	2.23 (1.44 to 2.31)	2005-2014	2.41 (2.27 to 2.54)			2005-2009	2.56 (2.19 to 2.70)				
	3	2009-2014	1.59 (1.53 to 2.22)	2014-2019	1.50 (1.34 to 2.42)			2009-2014	2.37 (1.36 to 2.45)				
	4	2014-2019	0.69 (0.58 to 1.57)	2019-2021	1.93 (1.55 to 2.12)			2014-2021	1.46 (1.39 to 1.56)				
	5	2019-2021	1.08 (0.76 to 1.23)										
Dominica	0	1990-1994	2.07 (1.96 to 2.17)	1990-1994	2.74 (2.52 to 2.88)	1990-1992	2.47 (1.78 to 2.98)	1990-1994	2.71 (2.59 to 2.83)	1990-1992	2.40 (1.63 to 3.03)	1990-1992	2.52 (1.88 to 3.03)
	1	1994-2014	1.14 (1.12 to 1.15)	1994-2006	1.63 (1.59 to 1.66)	1992-1997	0.87 (0.56 to 1.06)	1994-2006	1.58 (1.54 to 1.62)	1992-1997	0.47 (0.16 to 0.70)	1992-1996	1.15 (0.65 to 1.35)
	2	2014-2021	0.95 (0.82 to 1.02)	2006-2021	1.32 (1.28 to 1.34)	1997-2001	-0.91 (-1.31 to -0.65)	2006-2018	1.34 (1.30 to 1.44)	1997-2001	-1.32 (-1.82 to -0.98)	1996-2001	-0.23 (-0.63 to 0.05)
	3					2001-2004	0.43 (-0.06 to 0.71)	2018-2021	1.03 (0.66 to 1.26)	2001-2004	0.16 (-0.41 to 0.50)	2001-2004	0.66 (-0.46 to 0.90)
	4					2004-2009	-1.73 (-2.07 to -1.52)			2004-2009	-1.88 (-2.40 to -1.67)	2004-2009	-0.71 (-1.13 to 0.17)
	5					2009-2016	-0.07 (-0.29 to 0.10)			2009-2016	0.15 (-0.75 to 0.30)	2009-2021	0.72 (0.64 to 0.81)
	6					2016-2021	0.81 (0.55 to 1.27)			2016-2021	0.68 (0.35 to 1.43)		
Dominican Republic	0	1990-1993	2.79 (2.45 to 3.13)	1990-1994	3.06 (2.83 to 3.30)	1990-2000	-1.52 (-1.78 to -1.27)	1990-1994	3.06 (2.81 to 3.32)	1990-2001	-0.77 (-0.95 to -0.61)	1990-2001	0.68 (0.57 to 0.78)
	1	1993-2001	1.70 (1.58 to 1.77)	1994-2001	2.10 (1.93 to 2.21)	2000-2006	4.82 (4.19 to 5.46)	1994-2001	2.00 (1.81 to 2.12)	2001-2006	4.29 (3.70 to 4.90)	2001-2006	3.45 (3.06 to 3.93)
	2	2001-2010	2.59 (2.51 to 2.69)	2001-2010	2.80 (2.73 to 2.90)	2006-2011	-1.65 (-3.39 to -0.51)	2001-2010	2.80 (2.73 to 2.93)	2006-2011	-1.56 (-2.86 to -0.71)	2006-2011	0.65 (-0.02 to 1.04)
	3	2010-2018	1.87 (1.79 to 1.97)	2010-2018	2.12 (2.05 to 2.21)	2011-2021	0.90 (0.56 to 1.40)	2010-2018	2.12 (2.03 to 2.22)	2011-2018	2.08 (1.69 to 2.94)	2011-2018	2.09 (1.86 to 2.67)
	4	2018-2021	1.01 (0.54 to 1.30)	2018-2021	1.16 (0.76 to 1.45)			2018-2021	1.01 (0.60 to 1.34)	2018-2021	-0.88 (-3.08 to 0.50)	2018-2021	0.14 (-0.99 to 0.88)
Ecuador	0	1990-1993	3.85 (3.72 to 3.96)	1990-1993	4.04 (3.85 to 4.35)	1990-1996	4.25 (3.25 to 5.90)	1990-1994	4.09 (3.95 to 4.26)	1990-1996	4.42 (3.39 to 5.95)	1990-1996	4.36 (3.62 to 5.40)
	1	1993-1999	3.17 (3.12 to 3.21)	1993-1999	3.48 (3.41 to 3.53)	1996-2011	1.14 (0.60 to 1.53)	1994-1999	3.42 (3.31 to 3.50)	1996-2011	0.56 (0.05 to 0.98)	1996-2011	1.35 (1.06 to 1.62)
	2	1999-2003	2.39 (2.33 to 2.98)	1999-2006	2.29 (2.25 to 2.33)	2011-2021	-3.75 (-5.05 to -2.79)	1999-2006	2.41 (2.36 to 2.46)	2011-2021	-4.10 (-5.67 to -3.04)	2011-2021	-1.32 (-2.02 to -0.82)
	3	2003-2006	2.20 (2.13 to 2.66)	2006-2009	3.65 (3.54 to 3.75)			2006-2009	3.92 (3.80 to 4.04)				
	4	2006-2009	2.75 (2.21 to 2.82)	2009-2013	2.66 (2.58 to 2.75)			2009-2013	2.64 (2.54 to 2.77)				
	5	2009-2014	2.18 (1.24 to 2.21)	2013-2016	1.97 (1.82 to 2.13)			2013-2016	1.91 (1.63 to 2.17)				
	6	2014-2021	1.17 (1.14 to 1.21)	2016-2021	1.36 (1.26 to 1.43)			2016-2021	1.24 (1.13 to 1.32)				
Egypt	0	1990-1992	2.32 (2.12 to 2.62)	1990-1995	3.34 (3.09 to 3.48)	1990-1992	-3.42 (-4.67 to -0.96)	1990-1992	2.92 (2.63 to 3.34)	1990-2000	0.72 (0.26 to 1.08)	1990-1992	-0.79 (-1.85 to 1.31)
	1	1992-1996	3.12 (3.00 to 3.32)	1995-2000	4.43 (3.92 to 4.64)	1992-2000	1.22 (0.89 to 1.82)	1992-1996	3.64 (3.50 to 4.45)	2000-2003	9.58 (1.89 to 10.68)	1992-2000	1.73 (1.39 to 6.70)
	2	1996-2000	4.12 (3.92 to 4.34)	2000-2005	5.33 (4.75 to 5.63)	2000-2003	11.98 (10.30 to 12.85)	1996-2000	4.59 (4.34 to 5.13)	2003-2009	4.56 (3.53 to 6.93)	2000-2003	8.34 (4.68 to 9.06)
	3	2000-2009	4.64 (4.60 to 4.70)	2005-2009	4.91 (4.53 to 5.19)	2003-2009	5.23 (4.60 to 5.88)	2000-2008	5.17 (5.05 to 5.30)	2009-2015	2.22 (0.64 to 3.09)	2003-2009	4.68 (3.60 to 5.32)
	4	2009-2015	3.86 (3.78 to 3.94)	2009-2015	4.27 (4.09 to 4.37)	2009-2015	2.89 (1.93 to 3.41)	2008-2015	4.29 (4.20 to 4.36)	2015-2021	-1.99 (-3.02 to -1.26)	2009-2015	2.70 (1.50 to 3.33)
	5	2015-2019	5.02 (4.91 to 5.13)	2015-2018	6.09 (5.88 to 6.27)	2015-2021	-1.85 (-2.47 to -1.28)	2015-2018	6.00 (5.84 to 6.16)			2015-2021	0.06 (-0.84 to 0.60)
	6	2019-2021	2.93 (2.65 to 3.16)	2018-2021	4.00 (3.75 to 4.21)			2018-2021	3.85 (3.66 to 4.04)				
El Salvador	0	1990-1994	5.14 (4.73 to 5.55)	1990-1994	5.65 (5.24 to 6.05)	1990-1997	4.06 (2.88 to 6.56)	1990-1994	5.88 (5.47 to 6.27)	1990-1997	3.76 (2.67 to 6.19)	1990-1994	4.87 (2.98 to 8.40)
	1	1994-2001	0.60 (0.32 to 0.81)	1994-2001	0.46 (0.19 to 0.68)	1997-2021	1.79 (1.44 to 1.98)	1994-2001	0.45 (0.18 to 0.70)	1997-2021	1.66 (1.35 to 1.85)	1994-2021	1.88 (1.71 to 2.00)
	2	2001-2010	2.48 (1.95 to 2.64)	2001-2010	2.61 (2.33 to 2.77)			2001-2010	2.66 (2.34 to 2.82)				
	3	2010-2014	3.29 (2.79 to 3.82)	2010-2014	3.66 (3.16 to 4.23)			2010-2014	3.72 (3.18 to 4.37)				
	4	2014-2019	0.13 (-0.34 to 0.43)	2014-2019	0.23 (-0.22 to 0.55)			2014-2019	0.16 (-0.29 to 0.49)				
	5	2019-2021	6.52 (5.52 to 7.50)	2019-2021	7.20 (6.19 to 8.25)			2019-2021	7.00 (6.13 to 8.00)				
Equatorial Guinea	0	1990-1995	1.68 (1.42 to 1.84)	1990-1994	2.18 (1.70 to 2.42)	1990-1996	0.64 (0.15 to 1.44)	1990-1994	2.18 (1.56 to 2.45)	1990-1996	0.50 (-0.06 to 1.47)	1990-1996	0.87 (0.32 to 1.81)
	1	1995-2005	2.35 (2.29 to 2.42)	1994-2005	2.88 (2.80 to 2.96)	1996-2001	-1.42 (-2.83 to -0.64)	1994-2005	2.93 (2.85 to 3.03)	1996-2001	-2.04 (-3.80 to -1.08)	1996-2001	-0.93 (-2.39 to 0.29)
	2	2005-2010	3.66 (3.50 to 3.87)	2005-2010	3.69 (2.94 to 4.02)	2001-2011	1.51 (1.11 to 2.23)	2005-2010	3.79 (3.57 to 4.26)	2001-2011	1.23 (0.79 to 2.51)	2001-2011	1.80 (-0.03 to 2.77)
	3	2010-2014	2.30 (2.09 to 2.57)	2010-2015	2.71 (2.53 to 3.68)	2011-2014	-2.29 (-3.20 to -0.19)	2010-2014	2.81 (2.39 to 3.20)	2011-2014	-3.11 (-4.23 to -0.63)	2011-2014	-1.27 (-2.12 to 1.84)
	4	2014-2021	1.39 (1.28 to 1.48)	2015-2019	1.73 (1.43 to 2.67)	2014-2021	0.77 (0.16 to 2.28)	2014-2021	1.91 (1.72 to 2.03)	2014-2021	0.79 (0.04 to 2.96)	2014-2021	1.17 (0.51 to 2.74)
	5			2019-2021	2.60 (1.97 to 2.97)								
Eritrea	0	1990-2001	1.13 (1.08 to 1.16)	1990-2001	2.13 (2.07 to 2.18)	1990-1993	-1.16 (-1.36 to -0.97)	1990-2002	2.10 (2.05 to 2.14)	1990-1993	-2.03 (-2.29 to -1.81)	1990-1993	-1.40 (-1.64 to -1.20)
	1	2001-2005	1.45 (1.24 to 1.70)	2001-2014	2.49 (2.46 to 2.54)	1993-1999	0.62 (0.50 to 0.72)	2002-2014	2.53 (2.48 to 2.58)	1993-1997	-0.01 (-0.28 to 0.17)	1993-1997	0.31 (0.05 to 0.47)
	2	2005-2010	1.93 (1.84 to 2.12)	2014-2021	1.78 (1.66 to 1.88)	1999-2004	1.49 (1.33 to 1.77)	2014-2021	1.66 (1.57 to 1.77)	1997-2001	0.68 (0.39 to 1.24)	1997-2001	0.92 (0.68 to 1.23)
	3	2010-2015	1.51 (1.41 to 1.60)			2004-2009	0.74 (0.52 to 0.97)			2001-2004	1.55 (0.58 to 1.75)	2001-2004	1.70 (1.32 to 1.86)
	4	2015-2019	0.66 (0.50 to 0.75)			2009-2015	-0.03 (-0.33 to 0.09)			2004-2010	0.51 (-0.23 to 0.69)	2004-2010	0.89 (0.65 to 1.03)
	5	2019-2021	1.37 (1.09 to 1.56)			2015-2021	0.44 (0.33 to 0.63)			2010-2015	-0.31 (-0.67 to 0.09)	2010-2015	0.23 (-0.05 to 0.40)
	6									2015-2021	0.34 (0.19 to 0.56)	2015-2021	0.63 (0.52 to 0.86)
Estonia	0	1990-2000	2.90 (2.80 to 3.02)	1990-1999	3.26 (3.10 to 3.40)	1990-1994	7.20 (4.68 to 12.11)	1990-1999	3.24 (3.03 to 3.41)	1990-1994	8.18 (5.34 to 13.51)	1990-1994	4.93 (4.16 to 6.22)

Location	Segment	Incidence		Prevalence		Mortality		YLD		YLL		DALY	
		Period	APC (95%CI)	Period	APC (95%CI)	Period	APC (95%CI)	Period	APC (95%CI)	Period	APC (95%CI)	Period	APC (95%CI)
	1	2000-2017	2.16 (2.11 to 2.21)	1999-2012	2.45 (2.39 to 3.36)	1994-1997	-5.49 (-7.97 to -0.72)	1999-2012	2.46 (2.40 to 3.44)	1994-1997	-6.28 (-8.84 to -1.22)	1994-1997	-0.04 (-0.83 to 1.48)
	2	2017-2021	0.75 (0.26 to 1.13)	2012-2017	2.01 (1.68 to 2.41)	1997-2007	3.35 (2.55 to 5.56)	2012-2017	2.05 (1.67 to 2.42)	1997-2007	2.10 (1.31 to 4.95)	1997-2006	2.51 (2.20 to 3.53)
	3			2017-2021	1.08 (0.48 to 1.39)	2007-2010	-19.15 (-21.64 to 0.22)	2017-2021	1.02 (0.39 to 1.35)	2007-2010	-18.79 (-21.25 to 0.22)	2006-2011	-1.31 (-2.79 to -0.46)
	4					2010-2013	7.95 (-13.12 to 12.12)			2010-2013	8.94 (-15.38 to 13.44)	2011-2018	5.61 (5.11 to 6.42)
	5					2013-2016	22.98 (17.02 to 26.98)			2013-2016	22.83 (15.96 to 26.99)	2018-2021	0.74 (-1.28 to 2.17)
	6					2016-2021	5.56 (1.58 to 7.69)			2016-2021	5.31 (1.57 to 7.35)		
Eswatini	0	1990-1995	2.18 (1.81 to 2.32)	1990-1995	2.91 (2.47 to 3.11)	1990-1994	1.25 (0.38 to 1.71)	1990-1995	2.82 (2.43 to 3.00)	1990-1994	0.91 (0.20 to 1.33)	1990-1994	1.18 (0.06 to 1.69)
	1	1995-2010	2.47 (2.44 to 2.51)	1995-2000	3.55 (3.32 to 3.91)	1994-1998	3.92 (3.45 to 4.68)	1995-2000	3.48 (3.29 to 3.83)	1994-1998	3.79 (3.34 to 4.46)	1994-1998	3.71 (3.12 to 4.65)
	2	2010-2014	2.95 (2.79 to 3.17)	2000-2010	3.12 (2.93 to 3.25)	1998-2002	9.62 (9.21 to 10.10)	2000-2010	3.14 (2.94 to 3.21)	1998-2002	10.81 (10.36 to 11.25)	1998-2002	9.76 (9.24 to 10.41)
	3	2014-2021	0.20 (0.12 to 0.28)	2010-2014	4.29 (3.12 to 4.67)	2002-2005	4.91 (4.39 to 5.80)	2010-2014	4.34 (4.10 to 4.67)	2002-2005	5.40 (4.86 to 6.26)	2002-2005	5.16 (4.32 to 6.45)
	4			2014-2019	0.46 (0.20 to 4.24)	2005-2008	-0.92 (-1.60 to -0.46)	2014-2021	0.52 (0.42 to 0.62)	2005-2008	-1.25 (-1.94 to -0.66)	2005-2008	-0.81 (-1.59 to -0.23)
	5			2019-2021	1.14 (0.56 to 1.47)	2008-2021	-2.25 (-2.38 to -2.17)			2008-2018	-2.73 (-3.45 to -2.61)	2008-2021	-1.80 (-2.16 to -1.71)
	6									2018-2021	-1.68 (-2.48 to -0.62)		
Ethiopia	0	1990-1994	0.30 (0.26 to 0.35)	1990-1994	1.48 (1.37 to 1.59)	1990-1996	-0.41 (-0.75 to 0.13)	1990-1994	1.47 (1.37 to 1.58)	1990-1996	-0.63 (-1.07 to 0.06)	1990-1996	-0.36 (-0.63 to 0.02)
	1	1994-2008	-0.24 (-0.26 to -0.23)	1994-2000	0.46 (0.35 to 0.52)	1996-1999	-1.76 (-3.17 to -0.62)	1994-2001	0.44 (0.34 to 0.51)	1996-1999	-2.29 (-3.74 to -0.67)	1996-1999	-1.93 (-2.82 to -0.25)
	2	2008-2011	0.06 (-0.04 to 0.15)	2000-2005	0.94 (0.86 to 1.08)	1999-2005	-3.68 (-4.01 to -3.25)	2001-2004	1.20 (0.43 to 1.30)	1999-2005	-4.37 (-4.72 to -3.60)	1999-2005	-3.41 (-3.68 to -2.21)
	3	2011-2015	0.71 (0.64 to 0.78)	2005-2010	-0.05 (-0.12 to 0.02)	2005-2009	-4.94 (-5.40 to -4.52)	2004-2010	0.16 (0.08 to 1.19)	2005-2009	-5.56 (-6.06 to -4.63)	2005-2011	-4.05 (-4.35 to -3.41)
	4	2015-2019	0.21 (0.15 to 0.27)	2010-2015	1.28 (1.22 to 1.39)	2009-2013	-4.04 (-4.35 to -3.60)	2010-2015	1.36 (0.14 to 1.46)	2009-2013	-4.69 (-5.07 to -4.17)	2011-2014	-1.67 (-4.12 to -1.34)
	5	2019-2021	1.14 (1.01 to 1.26)	2015-2019	0.94 (0.78 to 1.04)	2013-2018	0.21 (-0.38 to 0.41)	2015-2019	0.85 (0.67 to 1.41)	2013-2017	-0.39 (-1.03 to 0.01)	2014-2018	0.34 (-1.76 to 0.58)
	6			2019-2021	1.59 (1.31 to 1.80)	2018-2021	1.34 (0.69 to 2.26)	2019-2021	1.28 (0.94 to 1.50)	2017-2021	1.04 (0.50 to 2.17)	2018-2021	1.20 (0.65 to 1.87)
Fiji	0	1990-1993	2.96 (2.88 to 3.05)	1990-1993	3.46 (3.33 to 3.65)	1990-1997	4.20 (3.38 to 5.67)	1990-1993	3.64 (3.53 to 3.78)	1990-1996	3.80 (2.60 to 6.85)	1990-1996	3.74 (2.81 to 5.48)
	1	1993-1999	2.04 (2.01 to 2.07)	1993-1996	2.74 (2.55 to 2.96)	1997-2006	1.21 (0.28 to 1.49)	1993-1996	2.81 (2.66 to 2.97)	1996-2006	1.28 (-0.03 to 1.55)	1996-2006	1.36 (0.31 to 1.63)
	2	1999-2003	1.34 (1.30 to 1.42)	1996-1999	2.32 (1.92 to 2.39)	2006-2009	3.39 (1.99 to 4.09)	1996-1999	2.31 (2.00 to 2.39)	2006-2009	3.24 (1.60 to 3.98)	2006-2009	3.01 (0.28 to 3.65)
	3	2003-2009	1.16 (1.12 to 1.20)	1999-2015	1.89 (1.88 to 1.90)	2009-2021	-0.88 (-1.33 to -0.57)	1999-2009	1.89 (1.86 to 1.92)	2009-2021	-1.06 (-1.63 to -0.67)	2009-2021	-0.58 (-1.03 to -0.28)
	4	2009-2016	0.89 (0.85 to 1.16)	2015-2019	2.40 (2.23 to 2.49)			2009-2015	1.78 (1.63 to 1.83)				
	5	2016-2019	1.19 (0.89 to 1.24)	2019-2021	3.19 (2.87 to 3.37)			2015-2019	2.32 (2.14 to 2.46)				
	6	2019-2021	1.66 (1.44 to 1.80)					2019-2021	3.06 (2.80 to 3.20)				
Finland	0	1990-1993	2.16 (1.98 to 2.41)	1990-1994	2.52 (2.36 to 2.81)	1990-2011	-4.39 (-4.73 to -4.09)	1990-1994	2.45 (2.30 to 2.72)	1990-2011	-4.21 (-4.57 to -3.90)	1990-2006	0.20 (0.08 to 0.28)
	1	1993-1999	1.81 (1.58 to 1.86)	1994-1999	2.04 (1.80 to 2.19)	2011-2021	1.82 (0.64 to 3.45)	1994-1999	1.95 (1.75 to 2.10)	2011-2021	1.56 (0.39 to 3.27)	2006-2011	1.50 (0.53 to 2.32)
	2	1999-2006	1.05 (0.99 to 1.09)	1999-2006	1.55 (1.43 to 1.63)			1999-2006	1.51 (1.36 to 1.58)			2011-2021	2.85 (2.65 to 3.21)
	3	2006-2011	2.10 (2.01 to 2.17)	2006-2010	2.48 (2.25 to 2.67)			2006-2010	2.52 (2.19 to 2.78)				
	4	2011-2018	2.59 (2.52 to 2.64)	2010-2021	3.03 (2.99 to 3.07)			2010-2021	3.00 (2.96 to 3.05)				
	5	2018-2021	3.02 (2.87 to 3.27)										
France	0	1990-1996	1.08 (1.02 to 1.13)	1990-1996	1.12 (0.98 to 1.24)	1990-1996	-1.94 (-2.70 to -1.34)	1990-1996	1.22 (1.08 to 1.35)	1990-1996	-2.16 (-2.83 to -1.57)	1990-1996	-0.74 (-1.92 to -0.17)
	1	1996-2000	2.04 (1.94 to 2.17)	1996-1999	3.27 (2.90 to 3.48)	1996-1999	8.45 (6.53 to 9.65)	1996-1999	4.00 (3.58 to 4.25)	1996-1999	8.75 (6.92 to 9.97)	1996-1999	6.73 (0.83 to 7.76)
	2	2000-2005	1.37 (1.25 to 1.46)	1999-2005	2.03 (1.76 to 2.15)	1999-2002	2.73 (-1.85 to 3.88)	1999-2005	2.01 (1.66 to 2.14)	1999-2002	2.35 (-1.29 to 3.48)	1999-2002	1.92 (-0.76 to 5.02)
	3	2005-2010	1.96 (1.88 to 2.07)	2005-2010	2.97 (2.77 to 3.28)	2002-2014	-4.00 (-4.61 to -3.73)	2005-2010	2.80 (2.57 to 3.23)	2002-2014	-4.27 (-4.75 to -4.00)	2002-2013	-1.04 (-2.71 to -0.68)
	4	2010-2015	0.96 (0.89 to 1.04)	2010-2015	1.44 (1.18 to 1.62)	2014-2021	-1.81 (-2.60 to -0.30)	2010-2015	1.40 (1.07 to 1.59)	2014-2021	-1.80 (-2.53 to -0.54)	2013-2021	0.58 (-0.13 to 2.31)
	5	2015-2018	2.63 (2.49 to 2.77)	2015-2018	4.01 (3.65 to 4.31)			2015-2018	3.84 (3.43 to 4.15)				
	6	2018-2021	0.57 (0.40 to 0.74)	2018-2021	0.07 (-0.30 to 0.43)			2018-2021	0.06 (-0.30 to 0.42)				
Gabon	0	1990-1995	2.03 (1.93 to 2.10)	1990-1995	2.60 (2.48 to 2.69)	1990-1992	-0.56 (-1.37 to 0.82)	1990-1995	2.54 (2.45 to 2.62)	1990-1993	0.22 (-1.19 to 1.17)	1990-1993	0.63 (-0.55 to 1.38)
	1	1995-2000	2.51 (2.43 to 2.62)	1995-2000	3.24 (3.15 to 3.36)	1992-2001	2.54 (2.40 to 2.85)	1995-2000	3.25 (3.16 to 3.36)	1993-2001	2.71 (2.49 to 3.25)	1993-2001	2.78 (2.62 to 3.16)
	2	2000-2006	2.08 (1.98 to 2.13)	2000-2009	2.71 (2.67 to 2.74)	2001-2004	0.72 (-0.76 to 1.85)	2000-2010	2.69 (2.66 to 2.72)	2001-2004	0.78 (-1.57 to 2.04)	2001-2004	1.15 (-0.34 to 2.25)
	3	2006-2009	2.45 (2.34 to 2.54)	2009-2014	2.05 (1.99 to 2.12)	2004-2013	-1.76 (-2.05 to -1.57)	2010-2014	1.95 (1.87 to 2.06)	2004-2013	-2.17 (-2.54 to -1.92)	2004-2013	-1.01 (-1.31 to -0.83)
	4	2009-2014	1.42 (1.37 to 1.47)	2014-2019	1.11 (1.05 to 1.17)	2013-2021	0.03 (-0.22 to 0.34)	2014-2019	1.04 (0.98 to 1.10)	2013-2021	-0.24 (-0.56 to 0.13)	2013-2021	0.28 (0.06 to 0.56)
	5	2014-2019	0.49 (0.44 to 0.54)	2019-2021	3.12 (2.91 to 3.29)			2019-2021	2.95 (2.75 to 3.13)				
	6	2019-2021	2.11 (1.92 to 2.26)										
Gambia	0	1990-1993	1.92 (1.78 to 2.00)	1990-1995	2.80 (2.73 to 2.85)	1990-2006	2.77 (2.56 to 2.99)	1990-1993	2.59 (2.45 to 2.69)	1990-2007	2.65 (2.04 to 3.09)	1990-2007	2.76 (2.57 to 2.98)
	1	1993-1996	2.25 (2.15 to 2.46)	1995-2004	3.14 (3.12 to 3.18)	2006-2012	-0.44 (-2.49 to 0.45)	1993-1996	2.90 (2.75 to 3.10)	2007-2012	-2.21 (-4.98 to 3.33)	2007-2011	-0.85 (-2.39 to 0.65)
	2	1996-2000	2.70 (2.63 to 2.77)	2004-2009	2.89 (2.83 to 2.95)	2012-2021	2.08 (1.57 to 3.36)	1996-2004	3.15 (3.12 to 3.21)	2012-2017	3.76 (-2.89 to 6.99)	2011-2021	2.27 (1.89 to 3.20)
	3	2000-2009	2.59 (2.55 to 2.60)	2009-2014	2.57 (2.53 to 2.62)			2004-2009	2.89 (2.83 to 2.96)	2017-2021	0.68 (-2.52 to 2.61)		
	4	2009-2014	2.14 (2.11 to 2.17)	2014-2019	1.95 (1.89 to 1.99)			2009-2014	2.58 (2.54 to 2.63)				
	5	2014-2018	1.57 (1.51 to 1.62)	2019-2021	2.32 (2.16 to 2.41)			2014-2019	1.93 (1.86 to 1.97)				
	6	2018-2021	1.79 (1.71 to 1.92)					2019-2021	2.18 (2.01 to 2.27)				
Georgia	0	1990-1992	1.82 (1.37 to 2.51)	1990-1994	2.55 (1.85 to 2.97)	1990-1992	-9.90 (-14.93 to -0.06)	1990-1992	2.05 (1.64 to 2.55)	1990-1992	-7.11 (-11.98 to 2.19)	1990-1992	-3.85 (-6.86 to 2.07)
	1	1992-1995	2.82 (2.61 to 4.83)	1994-2000	4.95 (4.63 to 5.28)	1992-2001	7.34 (5.41 to 12.15)	1992-1995	3.28 (3.06 to 4.85)	1992-2001	6.74 (4.99 to 12.11)	1992-2000	6.11 (5.18 to 8.48)
	2	1995-2000	4.65 (2.64 to 4.90)	2000-2017	2.85 (2.80 to 2.90)	2001-2007	-6.65 (-10.73 to 6.82)	1995-2000	5.14 (4.99 to 5.38)	2001-2007	-6.41 (-10.20 to 5.23)	2000-2007	-1.06 (-3.02 to 0.11)
	3	2000-2014	2.75 (2.71 to 2.94)	2017-2021	1.38 (0.88 to 1.76)	2007-2012	19.63 (-7.61 to 24.29)	2000-2005	2.66 (2.41 to 2.82)	2007-2012	18.14 (-7.24 to 22.17)	2007-2012	10.11 (8.00 to 12.76)
	4	2014-2018	2.35 (1.98 to 2.60)			2012-2015	-3.18 (-5.73 to 22.91)	2005-2014	2.97 (2.91 to 3.25)	2012-2015	-4.31 (-6.62 to 20.91)	2012-2021	-1.98 (-2.74 to -1.35)
	5	2018-2021	0.98 (0.50 to 1.33)			2015-2018	-11.94 (-14.87 to -7.37)	2014-2018	2.50 (2.12 to 2.72)	2015-2018	-12.57 (-15.25 to -8.38)		
	6					2018-2021	3.41 (-1.31 to 12.49)	2018-2021	1.01 (0.67 to 1.35)	2018-2021	2.85 (-1.65 to 11.30)		
Germany	0	1990-1995	1.40 (0.93 to 1.64)	1990-1995	2.11 (1.52 to 2.37)	1990-1993	3.48 (2.09 to 5.78)	1990-1995	2.20 (1.42 to 2.54)	1990-1993	2.10 (0.66 to 4.50)	1990-1993	2.22 (1.60 to 3.34)

Location	Segment	Incidence		Prevalence		Mortality		YLD		YLL		DALY	
		Period	APC (95%CI)	Period	APC (95%CI)	Period	APC (95%CI)	Period	APC (95%CI)	Period	APC (95%CI)	Period	APC (95%CI)
	1	1995-1999	2.62 (2.22 to 3.09)	1995-2000	3.16 (2.85 to 3.78)	1993-1996	-3.09 (-5.59 to -0.71)	1995-2000	3.00 (2.65 to 3.61)	1993-1996	-2.83 (-9.39 to -1.12)	1993-1996	-1.41 (-2.10 to -0.41)
	2	1999-2010	1.49 (1.39 to 1.56)	2000-2009	2.17 (1.89 to 2.28)	1996-1999	-10.17 (-11.57 to -7.52)	2000-2009	2.05 (1.51 to 2.26)	1996-1999	-10.97 (-12.22 to -0.48)	1996-1999	-6.08 (-6.83 to -5.21)
	3	2010-2015	3.32 (2.82 to 3.72)	2009-2013	3.49 (2.79 to 4.14)	1999-2003	0.92 (-0.95 to 3.12)	2009-2012	3.31 (2.29 to 4.73)	1999-2003	0.18 (-4.94 to 2.06)	1999-2003	1.13 (0.40 to 2.21)
	4	2015-2021	4.46 (4.20 to 4.84)	2013-2021	4.78 (4.62 to 5.03)	2003-2006	-5.62 (-6.64 to -2.69)	2012-2021	4.73 (4.53 to 5.06)	2003-2007	-5.36 (-7.05 to -2.05)	2003-2007	-2.08 (-3.28 to -1.16)
	5					2006-2021	-1.97 (-2.25 to -1.53)			2007-2021	-1.80 (-2.12 to -1.44)	2007-2014	0.83 (-0.07 to 1.65)
	6											2014-2021	2.74 (2.12 to 4.30)
Ghana	0	1990-1994	3.43 (3.15 to 3.64)	1990-1993	3.57 (3.35 to 3.75)	1990-1999	1.28 (1.08 to 1.40)	1990-1994	3.80 (3.61 to 3.95)	1990-1999	1.17 (0.75 to 1.31)	1990-2009	1.75 (1.71 to 1.79)
	1	1994-2006	2.23 (2.17 to 2.29)	1993-2004	2.82 (2.79 to 2.84)	1999-2010	1.71 (1.62 to 1.88)	1994-2004	2.79 (2.74 to 2.84)	1999-2009	1.45 (1.31 to 1.79)	2009-2015	0.61 (0.38 to 0.91)
	2	2006-2015	1.76 (1.67 to 1.83)	2004-2014	2.18 (2.15 to 2.20)	2010-2016	0.12 (-0.02 to 1.48)	2004-2014	2.23 (2.18 to 2.27)	2009-2016	-0.05 (-0.25 to 0.24)	2015-2021	-0.29 (-0.59 to -0.09)
	3	2015-2018	-1.09 (-1.30 to -0.85)	2014-2019	0.74 (0.68 to 0.80)	2016-2021	-0.45 (-1.06 to -0.16)	2014-2019	0.43 (0.33 to 0.51)	2016-2021	-0.95 (-1.48 to -0.64)		
	4	2018-2021	1.24 (0.92 to 1.60)	2019-2021	2.23 (1.97 to 2.46)			2019-2021	2.03 (1.62 to 2.30)				
Greece	0	1990-1992	3.69 (3.10 to 4.32)	1990-1994	3.07 (2.92 to 3.25)	1990-1992	3.25 (-2.28 to 8.47)	1990-1992	5.60 (5.20 to 6.00)	1990-1992	3.06 (-1.53 to 7.18)	1990-1992	4.30 (2.55 to 5.50)
	1	1992-1995	1.80 (1.09 to 2.10)	1994-2000	0.40 (0.26 to 0.50)	1992-1995	-12.45 (-14.49 to -9.14)	1992-1995	2.57 (2.35 to 2.79)	1992-1995	-9.42 (-11.07 to -6.66)	1992-1998	-1.47 (-2.17 to -1.10)
	2	1995-2000	-0.53 (-0.87 to -0.35)	2000-2005	1.79 (1.40 to 2.00)	1995-2001	-1.26 (-3.86 to 1.32)	1995-2000	0.02 (-0.11 to 0.14)	1995-2001	-2.73 (-4.36 to -0.16)	1998-2001	0.08 (-1.17 to 1.95)
	3	2000-2005	1.16 (0.63 to 1.57)	2005-2010	2.44 (2.23 to 2.75)	2001-2007	3.20 (-8.43 to 6.62)	2000-2004	1.82 (1.63 to 2.05)	2001-2007	2.19 (0.92 to 4.90)	2001-2007	1.77 (-1.97 to 2.66)
	4	2005-2010	2.25 (1.94 to 2.68)	2010-2019	1.35 (1.23 to 1.42)	2007-2010	-12.63 (-14.48 to -1.80)	2004-2015	1.42 (1.36 to 1.50)	2007-2010	-10.61 (-12.11 to -6.38)	2007-2010	-1.76 (-2.28 to 2.21)
	5	2010-2019	0.73 (0.57 to 0.81)	2019-2021	2.48 (1.86 to 2.82)	2010-2021	1.43 (0.67 to 2.37)	2015-2019	1.07 (0.79 to 1.25)	2010-2021	1.71 (1.15 to 2.40)	2010-2021	1.46 (0.66 to 1.97)
	6	2019-2021	2.25 (1.39 to 2.66)					2019-2021	2.50 (1.98 to 2.90)				
Greenland	0	1990-1996	1.17 (0.98 to 1.27)	1990-1996	3.28 (3.11 to 3.37)	1990-1997	-1.03 (-1.64 to -0.33)	1990-1995	3.08 (2.74 to 3.23)	1990-1997	-0.93 (-1.44 to -0.20)	1990-1997	-0.24 (-0.42 to -0.06)
	1	1996-2000	1.64 (1.27 to 1.91)	1996-2000	3.90 (3.64 to 4.23)	1997-2013	-4.37 (-4.60 to -4.19)	1995-2000	3.67 (3.48 to 4.03)	1997-2006	-4.14 (-4.56 to -2.24)	1997-2000	-2.65 (-2.89 to -1.88)
	2	2000-2005	3.03 (1.68 to 3.23)	2000-2005	4.91 (4.77 to 5.11)	2013-2021	0.24 (-0.35 to 0.99)	2000-2005	4.82 (4.68 to 5.10)	2006-2013	-5.05 (-6.45 to -0.78)	2000-2011	-1.81 (-1.95 to -1.46)
	3	2005-2010	2.55 (2.36 to 3.03)	2005-2010	4.04 (3.92 to 4.15)			2005-2010	3.98 (3.79 to 4.12)	2013-2021	0.22 (-0.32 to 0.87)	2011-2014	1.38 (-0.59 to 2.89)
	4	2010-2013	4.44 (2.50 to 4.69)	2010-2014	5.85 (5.64 to 5.99)			2010-2014	5.75 (5.41 to 5.93)			2014-2021	3.46 (3.18 to 4.04)
	5	2013-2018	5.39 (4.52 to 5.71)	2014-2018	6.63 (6.47 to 6.87)			2014-2018	6.54 (6.34 to 6.82)				
	6	2018-2021	4.99 (4.58 to 5.26)	2018-2021	5.60 (5.34 to 5.80)			2018-2021	5.45 (5.08 to 5.69)				
Grenada	0	1990-1999	1.58 (1.52 to 1.67)	1990-1998	2.08 (1.98 to 2.20)	1990-1992	-10.14 (-13.92 to -4.87)	1990-1998	2.07 (1.96 to 2.22)	1990-1992	-7.04 (-9.71 to -3.33)	1990-1992	-4.60 (-6.36 to -2.14)
	1	1999-2011	1.16 (0.97 to 1.20)	1998-2013	1.44 (1.37 to 1.48)	1992-1996	3.62 (1.49 to 7.36)	1998-2013	1.44 (1.33 to 1.49)	1992-1996	3.16 (1.67 to 5.69)	1992-1996	2.68 (1.62 to 4.39)
	2	2011-2018	1.44 (1.32 to 1.80)	2013-2018	1.98 (1.78 to 2.37)	1996-2002	-3.38 (-6.78 to -2.13)	2013-2018	1.93 (1.68 to 2.34)	1996-2000	-5.34 (-7.34 to -3.73)	1996-2001	-2.85 (-4.80 to -1.92)
	3	2018-2021	0.47 (-0.05 to 0.80)	2018-2021	0.56 (0.02 to 0.98)	2002-2011	2.13 (1.35 to 3.49)	2018-2021	0.54 (-0.05 to 0.96)	2000-2006	-0.12 (-1.92 to 1.26)	2001-2011	1.34 (0.95 to 2.03)
	4					2011-2015	-5.47 (-8.15 to -3.08)			2006-2010	3.11 (1.16 to 4.87)	2011-2015	-2.41 (-4.13 to -0.93)
	5					2015-2021	2.68 (1.16 to 5.40)			2010-2016	-3.84 (-5.15 to -2.81)	2015-2021	1.98 (1.12 to 4.03)
	6									2016-2021	3.52 (1.95 to 5.78)		
Guam	0	1990-1998	0.29 (0.27 to 0.30)	1990-1999	1.29 (1.26 to 1.31)	1990-1998	-6.84 (-8.00 to -6.05)	1990-2000	1.20 (1.17 to 1.22)	1990-1992	-1.11 (-5.03 to 1.89)	1990-1992	-0.40 (-1.71 to 0.62)
	1	1998-2001	0.55 (0.48 to 0.62)	1999-2005	1.60 (1.36 to 1.69)	1998-2010	-3.07 (-4.63 to -2.41)	2000-2005	1.56 (1.49 to 1.69)	1992-1998	-6.13 (-8.32 to -3.16)	1992-1997	-4.10 (-4.89 to -3.75)
	2	2001-2007	0.91 (0.88 to 0.93)	2005-2010	1.41 (1.31 to 1.59)	2010-2019	-0.97 (-3.49 to 1.73)	2005-2009	1.38 (1.26 to 1.46)	1998-2003	-3.05 (-4.70 to -0.51)	1997-2001	-1.94 (-3.00 to -0.66)
	3	2007-2010	1.20 (1.14 to 1.25)	2010-2013	2.05 (1.39 to 2.14)	2019-2021	-7.19 (-11.11 to -1.65)	2009-2012	1.81 (1.56 to 2.10)	2003-2021	-1.32 (-2.37 to -0.66)	2001-2011	-0.31 (-0.64 to 0.28)
	4	2010-2013	1.73 (1.68 to 1.79)	2013-2016	2.47 (2.17 to 2.72)			2012-2016	2.40 (2.27 to 2.56)			2011-2021	0.82 (0.56 to 1.36)
	5	2013-2016	2.13 (2.08 to 2.19)	2016-2021	2.87 (2.81 to 2.97)			2016-2021	2.85 (2.79 to 2.95)				
	6	2016-2021	2.66 (2.63 to 2.70)										
Guatemala	0	1990-2003	4.18 (4.12 to 4.26)	1990-2000	4.25 (4.12 to 4.66)	1990-1992	35.70 (24.60 to 42.44)	1990-2000	4.33 (4.20 to 4.68)	1990-1992	34.89 (22.05 to 44.24)	1990-1992	22.47 (14.27 to 28.15)
	1	2003-2009	3.15 (2.87 to 3.46)	2000-2009	3.71 (3.33 to 3.93)	1992-2006	3.60 (3.07 to 4.24)	2000-2009	3.72 (3.35 to 3.97)	1992-2006	3.72 (3.08 to 4.56)	1992-2006	3.91 (3.52 to 4.34)
	2	2009-2014	1.75 (1.33 to 2.22)	2009-2014	2.33 (1.66 to 2.96)	2006-2021	0.92 (0.23 to 1.41)	2009-2014	2.33 (1.65 to 3.04)	2006-2021	1.49 (0.48 to 2.03)	2006-2021	1.56 (1.07 to 1.94)
	3	2014-2019	0.26 (-0.24 to 0.50)	2014-2019	0.49 (-0.21 to 0.79)			2014-2019	0.46 (-0.35 to 0.76)				
	4	2019-2021	3.63 (2.45 to 4.54)	2019-2021	4.34 (2.90 to 5.53)			2019-2021	3.88 (2.64 to 5.02)				
Guinea	0	1990-1994	2.18 (2.04 to 2.30)	1990-2000	1.79 (1.73 to 1.86)	1990-1996	1.62 (1.50 to 1.74)	1990-2000	1.92 (1.85 to 1.99)	1990-1996	1.44 (1.30 to 1.58)	1990-1996	1.56 (1.44 to 1.66)
	1	1994-2000	1.48 (1.37 to 1.55)	2000-2009	2.75 (2.69 to 2.82)	1996-1999	3.70 (3.45 to 3.90)	2000-2009	2.71 (2.64 to 2.79)	1996-1999	3.54 (3.19 to 3.76)	1996-1999	3.15 (2.80 to 3.32)
	2	2000-2009	2.16 (2.07 to 2.20)	2009-2015	1.34 (1.27 to 1.42)	1999-2002	2.65 (2.12 to 2.85)	2009-2015	1.27 (1.19 to 1.36)	1999-2002	2.43 (1.74 to 2.66)	1999-2002	2.43 (1.85 to 2.63)
	3	2009-2012	0.83 (0.72 to 2.22)	2015-2021	3.84 (3.73 to 3.94)	2002-2008	1.14 (1.04 to 1.24)	2015-2021	3.57 (3.47 to 3.67)	2002-2008	0.99 (0.83 to 1.14)	2002-2008	1.40 (1.26 to 1.50)
	4	2012-2015	0.47 (0.37 to 0.67)			2008-2016	0.72 (0.63 to 0.79)			2008-2016	0.60 (0.44 to 0.69)	2008-2016	0.84 (0.78 to 1.04)
	5	2015-2021	2.25 (2.18 to 2.32)			2016-2021	-0.41 (-0.56 to -0.24)			2016-2021	-0.59 (-0.82 to -0.37)	2016-2021	0.59 (0.30 to 0.72)
Guinea-Bissau	0	1990-1995	1.96 (1.81 to 2.06)	1990-2004	2.86 (2.84 to 2.90)	1990-1996	2.47 (2.10 to 2.78)	1990-2005	2.86 (2.83 to 2.90)	1990-1996	2.30 (1.95 to 2.46)	1990-1996	2.42 (2.20 to 2.54)
	1	1995-2008	2.33 (2.31 to 2.38)	2004-2009	2.54 (2.38 to 2.77)	1996-2000	3.41 (2.31 to 3.76)	2005-2009	2.54 (2.35 to 2.83)	1996-1999	3.46 (2.98 to 3.72)	1996-1999	3.36 (2.98 to 3.54)
	2	2008-2013	1.76 (1.60 to 2.03)	2009-2014	2.08 (1.92 to 2.29)	2000-2004	2.27 (2.01 to 3.52)	2009-2014	2.08 (1.91 to 2.29)	1999-2004	2.28 (1.98 to 2.47)	1999-2004	2.39 (2.21 to 2.57)
	3	2013-2018	1.27 (1.01 to 1.38)	2014-2018	1.58 (1.39 to 1.72)	2004-2007	1.55 (0.72 to 2.26)	2014-2018	1.59 (1.39 to 1.72)	2004-2008	1.05 (0.70 to 1.38)	2004-2007	1.60 (1.04 to 2.04)
	4	2018-2021	1.87 (1.67 to 2.21)	2018-2021	2.35 (2.17 to 2.65)	2007-2014	0.60 (0.46 to 1.48)	2018-2021	2.27 (2.10 to 2.55)	2008-2018	0.09 (0.02 to 0.18)	2007-2014	0.74 (0.61 to 0.85)
	5					2014-2019	0.12 (-0.03 to 0.61)			2018-2021	-0.66 (-1.32 to -0.29)	2014-2021	0.38 (0.22 to 0.47)
	6					2019-2021	-0.69 (-1.14 to -0.11)						
Guyana	0	1990-1994	2.38 (2.30 to 2.48)	1990-1992	3.14 (2.98 to 3.30)	1990-2002	3.64 (2.80 to 4.64)	1990-1992	3.16 (3.04 to 3.29)	1990-2002	3.78 (2.93 to 4.78)	1990-2002	3.19 (2.64 to 3.86)
	1	1994-2010	1.28 (1.26 to 1.30)	1992-1995	2.45 (2.36 to 2.54)	2002-2021	-2.45 (-3.07 to -1.99)	1992-1995	2.44 (2.37 to 2.51)	2002-2021	-2.81 (-3.42 to -2.33)	2002-2021	-1.26 (-1.64 to -0.96)
	2	2010-2021	0.70 (0.67 to 0.72)	1995-2000	1.62 (1.54 to 1.67)			1995-2000	1.61 (1.55 to 1.65)				
	3			2000-2004	2.15 (2.07 to 2.24)			2000-2004	2.05 (1.98 to 2.13)				
	4			2004-2009	1.85 (1.80 to 1.89)			2004-2009	1.80 (1.75 to 1.84)				

Location	Segment	Incidence		Prevalence		Mortality		YLD		YLL		DALY	
		Period	APC (95%CI)	Period	APC (95%CI)	Period	APC (95%CI)	Period	APC (95%CI)	Period	APC (95%CI)	Period	APC (95%CI)
	5			2009-2018	1.17 (1.15 to 1.20)			2009-2018	1.12 (1.10 to 1.14)				
	6			2018-2021	0.98 (0.80 to 1.08)			2018-2021	0.84 (0.69 to 0.94)				
Haiti	0	1990-1992	1.97 (1.85 to 2.08)	1990-1993	2.47 (2.42 to 2.53)	1990-1998	-0.45 (-0.61 to -0.32)	1990-1992	2.53 (2.43 to 2.63)	1990-1998	-0.73 (-0.92 to -0.59)	1990-1995	0.13 (-0.11 to 0.48)
	1	1992-1995	1.36 (1.28 to 1.43)	1993-1996	1.80 (1.72 to 1.85)	1998-2005	1.11 (0.92 to 1.36)	1992-1995	1.99 (1.93 to 2.04)	1998-2005	0.96 (0.73 to 1.27)	1995-1998	-0.52 (-0.79 to 1.21)
	2	1995-2001	0.83 (0.78 to 0.86)	1996-2005	1.43 (1.41 to 1.44)	2005-2009	-0.93 (-1.37 to -0.49)	1995-2004	1.42 (1.39 to 1.44)	2005-2009	-1.24 (-1.74 to -0.73)	1998-2005	1.15 (-0.50 to 1.41)
	3	2001-2012	1.08 (1.06 to 1.11)	2005-2010	1.61 (1.56 to 1.68)	2009-2021	-0.23 (-0.31 to -0.05)	2004-2009	1.61 (1.55 to 1.68)	2009-2021	-0.34 (-0.44 to -0.15)	2005-2009	-0.48 (-0.82 to 0.27)
	4	2012-2021	0.97 (0.94 to 1.00)	2010-2021	1.37 (1.36 to 1.38)			2009-2018	1.35 (1.34 to 1.41)			2009-2021	0.18 (0.07 to 0.31)
	5							2018-2021	1.23 (1.12 to 1.31)				
Honduras	0	1990-2014	2.24 (2.20 to 2.29)	1990-2013	2.39 (2.33 to 2.44)	1990-1994	3.49 (-1.83 to 6.82)	1990-1994	2.96 (2.54 to 3.71)	1990-1994	2.91 (-1.58 to 5.66)	1990-1993	2.24 (-0.33 to 4.02)
	1	2014-2019	0.30 (-0.54 to 0.70)	2013-2019	0.92 (-0.03 to 1.25)	1994-1997	11.06 (0.60 to 13.54)	1994-2003	2.15 (1.51 to 2.27)	1994-1997	9.08 (0.04 to 11.23)	1993-1996	6.44 (3.29 to 7.61)
	2	2019-2021	4.72 (2.99 to 5.70)	2019-2021	4.74 (2.89 to 5.74)	1997-2021	1.66 (-0.79 to 2.53)	2003-2013	2.54 (2.44 to 2.85)	1997-2021	1.53 (-1.33 to 2.53)	1996-2009	1.81 (0.78 to 2.05)
	3							2013-2019	0.79 (0.42 to 0.99)			2009-2012	3.56 (1.07 to 4.24)
	4							2019-2021	4.68 (3.35 to 5.58)			2012-2021	0.98 (0.28 to 1.43)
Hungary	0	1990-1995	2.44 (1.51 to 2.80)	1990-1994	2.49 (1.47 to 2.95)	1990-2002	-1.02 (-1.96 to -0.39)	1990-1994	2.51 (1.38 to 3.01)	1990-2002	-1.63 (-2.53 to -0.98)	1990-1996	0.03 (-0.87 to 0.46)
	1	1995-2000	3.54 (3.03 to 4.11)	1994-2000	3.67 (3.32 to 4.19)	2002-2005	10.02 (3.98 to 12.69)	1994-2000	3.80 (3.41 to 4.39)	2002-2005	9.20 (3.66 to 11.70)	1996-1999	3.74 (2.25 to 4.60)
	2	2000-2015	0.87 (0.80 to 0.95)	2000-2015	0.99 (0.86 to 1.75)	2005-2013	-4.40 (-7.47 to -3.26)	2000-2015	1.03 (0.92 to 1.44)	2005-2013	-4.62 (-7.22 to -3.57)	1999-2002	-0.60 (-1.68 to 0.72)
	3	2015-2019	-1.06 (-1.77 to -0.48)	2015-2019	-0.77 (-1.54 to 1.13)	2013-2021	2.13 (0.45 to 4.78)	2015-2019	-0.83 (-1.52 to 1.11)	2013-2021	2.09 (0.59 to 4.30)	2002-2005	4.40 (2.70 to 5.56)
	4	2019-2021	1.35 (0.00 to 2.20)	2019-2021	1.12 (-0.36 to 2.04)			2019-2021	0.99 (-0.43 to 1.84)			2005-2011	-1.33 (-2.72 to -0.72)
	5											2011-2021	0.38 (0.05 to 0.97)
Iceland	0	1990-1995	2.36 (2.27 to 2.42)	1990-1995	2.53 (2.41 to 2.60)	1990-2009	-1.13 (-1.31 to -0.94)	1990-1994	2.47 (2.33 to 2.55)	1990-2008	-1.49 (-1.68 to -1.25)	1990-1992	-0.03 (-0.46 to 0.75)
	1	1995-2000	2.88 (2.81 to 2.99)	1995-2000	3.09 (3.01 to 3.25)	2009-2021	-2.87 (-3.41 to -2.49)	1994-2002	2.98 (2.95 to 3.02)	2008-2021	-2.95 (-3.48 to -2.61)	1992-2006	1.54 (1.48 to 1.65)
	2	2000-2004	2.42 (2.30 to 2.50)	2000-2004	2.77 (2.57 to 2.86)			2002-2005	2.57 (2.36 to 2.73)			2006-2011	0.98 (0.46 to 1.28)
	3	2004-2011	1.87 (1.82 to 1.91)	2004-2011	2.11 (2.05 to 2.16)			2005-2010	2.04 (1.95 to 2.10)			2011-2021	1.92 (1.80 to 2.08)
	4	2011-2015	2.49 (2.36 to 2.56)	2011-2015	2.76 (2.59 to 2.85)			2010-2015	2.67 (2.60 to 2.73)				
	5	2015-2018	3.05 (2.91 to 3.14)	2015-2018	3.32 (3.15 to 3.41)			2015-2018	3.38 (3.26 to 3.46)				
	6	2018-2021	2.53 (2.36 to 2.62)	2018-2021	2.54 (2.38 to 2.65)			2018-2021	2.50 (2.39 to 2.60)				
India	0	1990-1995	1.16 (0.91 to 1.30)	1990-1993	2.14 (1.94 to 2.58)	1990-1996	1.58 (0.45 to 3.42)	1990-1993	4.07 (3.81 to 4.40)	1990-1995	1.08 (-0.09 to 3.54)	1990-1995	1.66 (0.99 to 2.58)
	1	1995-1999	2.18 (1.90 to 2.46)	1993-1996	1.28 (1.08 to 1.55)	1996-2001	-3.22 (-5.97 to -1.67)	1993-1996	1.11 (0.78 to 1.38)	1995-2004	-1.23 (-2.53 to -0.75)	1995-2004	-0.59 (-1.20 to -0.28)
	2	1999-2005	0.79 (0.62 to 0.93)	1996-1999	3.12 (2.74 to 3.32)	2001-2019	2.52 (2.34 to 2.94)	1996-2002	0.56 (0.19 to 0.71)	2004-2019	2.44 (2.25 to 2.90)	2004-2018	2.54 (2.39 to 2.84)
	3	2005-2012	1.86 (1.75 to 2.14)	1999-2006	1.27 (1.17 to 1.35)	2019-2021	-1.93 (-4.85 to 2.11)	2002-2006	1.23 (1.04 to 3.06)	2019-2021	-1.19 (-3.68 to 2.12)	2018-2021	0.41 (-1.77 to 1.88)
	4	2012-2021	1.69 (1.51 to 1.75)	2006-2013	2.39 (2.32 to 2.52)			2006-2014	3.30 (3.22 to 3.40)				
	5			2013-2016	1.56 (1.41 to 1.86)			2014-2019	1.11 (0.86 to 1.23)				
	6			2016-2021	2.22 (2.09 to 2.57)			2019-2021	2.30 (1.73 to 2.67)				
Indonesia	0	1990-1993	3.28 (3.04 to 3.55)	1990-1995	3.63 (2.77 to 5.06)	1990-1995	1.87 (1.60 to 2.04)	1990-1995	3.45 (2.77 to 4.45)	1990-1995	1.53 (1.25 to 1.70)	1990-1994	2.23 (1.87 to 2.96)
	1	1993-1999	0.61 (0.50 to 0.71)	1995-2000	-3.60 (-4.81 to -2.52)	1995-2000	2.72 (2.60 to 2.90)	1995-2000	-2.75 (-3.69 to -1.85)	1995-2000	2.53 (2.42 to 2.73)	1994-2000	1.20 (0.90 to 1.45)
	2	1999-2008	2.37 (2.21 to 2.44)	2000-2013	2.78 (2.58 to 3.29)	2000-2005	-0.15 (-0.33 to -0.04)	2000-2014	2.81 (2.66 to 3.13)	2000-2005	-0.62 (-0.81 to -0.50)	2000-2006	0.26 (-0.15 to 0.43)
	3	2008-2015	2.69 (2.59 to 2.91)	2013-2019	1.39 (0.12 to 2.02)	2005-2009	0.49 (0.08 to 0.78)	2014-2019	1.41 (0.33 to 2.10)	2005-2010	0.10 (-0.22 to 0.34)	2006-2015	1.10 (1.01 to 1.44)
	4	2015-2019	1.00 (0.69 to 1.21)	2019-2021	14.55 (10.22 to 17.43)	2009-2016	0.95 (0.85 to 1.16)	2019-2021	12.26 (8.95 to 14.55)	2010-2016	0.64 (0.51 to 0.94)	2015-2019	0.56 (0.18 to 0.85)
	5	2019-2021	2.64 (2.08 to 3.03)			2016-2021	0.12 (-0.08 to 0.28)			2016-2021	-0.17 (-0.41 to 0.01)	2019-2021	3.69 (2.68 to 4.40)
Iran (Islamic Republic of)	0	1990-2001	2.56 (2.45 to 2.65)	1990-1994	4.07 (3.51 to 4.94)	1990-2002	0.32 (0.13 to 0.49)	1990-1993	4.23 (3.55 to 5.43)	1990-2002	0.14 (-0.03 to 0.30)	1990-2001	1.33 (1.21 to 1.44)
	1	2001-2010	3.50 (3.37 to 3.68)	1994-2001	2.20 (1.71 to 2.47)	2002-2006	4.05 (3.18 to 5.07)	1993-2000	2.38 (1.76 to 2.62)	2002-2006	4.18 (3.50 to 4.77)	2001-2006	3.95 (3.57 to 4.56)
	2	2010-2015	2.05 (1.57 to 2.32)	2001-2009	3.80 (3.58 to 4.20)	2006-2011	0.19 (-0.22 to 0.71)	2000-2009	4.09 (3.13 to 4.38)	2006-2011	0.30 (-0.06 to 0.67)	2006-2012	2.00 (1.37 to 2.30)
	3	2015-2018	4.13 (3.53 to 4.48)	2009-2021	2.74 (2.61 to 2.85)	2011-2016	5.17 (4.77 to 5.56)	2009-2015	1.76 (1.22 to 4.41)	2011-2016	5.01 (4.68 to 5.36)	2012-2017	3.42 (3.00 to 4.12)
	4	2018-2021	1.58 (1.02 to 1.96)			2016-2019	-0.42 (-1.05 to 1.82)	2015-2018	3.76 (1.52 to 4.24)	2016-2019	-0.69 (-1.29 to 0.17)	2017-2021	0.25 (-0.30 to 0.80)
	5					2019-2021	-4.00 (-5.38 to -2.64)	2018-2021	2.25 (1.05 to 3.07)	2019-2021	-4.38 (-5.61 to -3.26)		
Iraq	0	1990-1998	1.88 (1.84 to 1.90)	1990-2000	2.15 (2.10 to 2.20)	1990-2017	0.11 (0.02 to 0.18)	1990-2000	2.15 (2.11 to 2.18)	1990-1996	0.51 (0.26 to 0.95)	1990-1995	1.18 (1.03 to 1.44)
	1	1998-2001	2.39 (2.29 to 2.45)	2000-2005	4.48 (4.39 to 4.57)	2017-2021	4.35 (3.08 to 6.30)	2000-2005	4.30 (4.23 to 4.37)	1996-2001	-0.90 (-1.70 to -0.50)	1995-2001	0.61 (0.37 to 0.74)
	2	2001-2004	3.71 (3.63 to 3.80)	2005-2014	2.01 (1.94 to 2.07)			2005-2015	2.05 (2.00 to 2.08)	2001-2008	0.42 (0.17 to 1.22)	2001-2004	2.53 (2.10 to 2.75)
	3	2004-2007	2.27 (2.17 to 2.34)	2014-2021	2.64 (2.53 to 2.77)			2015-2018	2.96 (2.69 to 3.10)	2008-2017	-0.66 (-0.94 to -0.48)	2004-2008	1.47 (1.14 to 1.76)
	4	2007-2015	1.99 (1.95 to 2.01)					2018-2021	2.12 (1.76 to 2.31)	2017-2021	3.01 (2.41 to 3.64)	2008-2013	0.53 (0.15 to 0.73)
	5	2015-2018	2.72 (2.63 to 2.79)									2013-2017	1.34 (0.94 to 1.91)
	6	2018-2021	1.92 (1.82 to 2.01)									2017-2021	2.56 (2.29 to 3.07)
Ireland	0	1990-1995	0.62 (0.50 to 0.75)	1990-1993	0.31 (0.16 to 0.42)	1990-1992	-1.34 (-5.58 to 1.55)	1990-1993	0.35 (0.08 to 0.48)	1990-1992	-1.91 (-5.97 to 1.04)	1990-1992	-0.97 (-2.72 to 0.24)
	1	1995-2000	2.01 (1.89 to 2.19)	1993-1996	0.99 (0.88 to 1.08)	1992-1995	-8.08 (-9.28 to -0.61)	1993-1996	1.02 (0.90 to 1.15)	1992-1995	-8.07 (-9.23 to -1.02)	1992-1995	-3.78 (-4.32 to 0.51)
	2	2000-2007	1.36 (1.16 to 1.45)	1996-2000	2.36 (2.29 to 2.43)	1995-2006	-0.69 (-5.73 to 0.08)	1996-2000	2.34 (2.26 to 2.43)	1995-2006	-1.26 (-7.72 to -0.57)	1995-2006	0.48 (-1.24 to 0.78)
	3	2007-2014	1.77 (1.66 to 1.92)	2000-2006	1.51 (1.43 to 1.57)	2006-2009	-6.85 (-8.01 to -1.84)	2000-2006	1.47 (1.37 to 1.52)	2006-2009	-7.99 (-9.08 to -2.80)	2006-2010	-1.61 (-2.60 to 0.42)
	4	2014-2021	2.38 (2.29 to 2.49)	2006-2015	1.83 (1.80 to 1.87)	2009-2021	-3.12 (-3.82 to -1.57)	2006-2015	1.90 (1.86 to 1.94)	2009-2021	-3.48 (-4.17 to -2.62)	2010-2021	0.82 (0.55 to 1.19)
	5			2015-2019	2.54 (2.00 to 2.64)			2015-2019	2.61 (2.52 to 2.78)				
	6			2019-2021	2.12 (1.95 to 2.37)			2019-2021	1.96 (1.74 to 2.21)				
Israel	0	1990-1995	2.67 (2.39 to 2.81)	1990-2000	2.66 (2.57 to 2.74)	1990-1994	-1.20 (-3.25 to 0.55)	1990-2000	2.77 (2.69 to 2.84)	1990-1994	-2.68 (-4.40 to -1.01)	1990-1994	-0.66 (-1.78 to 0.38)
	1	1995-1999	3.27 (3.11 to 3.47)	2000-2010	1.58 (1.51 to 1.66)	1994-1997	19.14 (15.27 to 21.49)	2000-2010	1.58 (1.51 to 1.65)	1994-1997	19.37 (16.49 to 21.67)	1994-1997	12.80 (11.21 to 14.20)
	2	1999-2010	1.43 (1.39 to 1.47)	2010-2014	-0.90 (-1.19 to -0.73)	1997-2003	-0.23 (-1.46 to 1.14)	2010-2014	-0.92 (-1.22 to -0.72)	1997-2003	-1.49 (-2.60 to -0.10)	1997-2001	0.60 (-0.51 to 2.02)

Location	Segment	Incidence		Prevalence		Mortality		YLD		YLL		DALY	
		Period	APC (95%CI)	Period	APC (95%CI)	Period	APC (95%CI)	Period	APC (95%CI)	Period	APC (95%CI)	Period	APC (95%CI)
	3	2010-2015	-1.04 (-1.19 to -0.95)	2014-2021	0.30 (0.18 to 0.46)	2003-2011	-5.58 (-8.32 to -4.70)	2014-2021	0.23 (0.12 to 0.38)	2003-2011	-6.18 (-8.95 to -5.30)	2001-2014	-2.64 (-3.67 to -2.40)
	4	2015-2021	-0.13 (-0.24 to 0.02)			2011-2021	-3.27 (-4.05 to -0.90)			2011-2021	-3.36 (-4.15 to -1.70)	2014-2021	-1.27 (-2.08 to 1.28)
Italy	0	1990-1995	0.38 (0.28 to 0.48)	1990-1995	0.76 (0.67 to 0.85)	1990-1993	-5.73 (-8.35 to -3.42)	1990-1995	0.72 (0.61 to 0.83)	1990-1993	-6.37 (-8.98 to -3.97)	1990-1994	-3.35 (-3.61 to -3.09)
	1	1995-2000	2.05 (1.95 to 2.14)	1995-2000	2.46 (2.38 to 2.53)	1993-2021	-2.54 (-2.68 to -2.36)	1995-2004	2.82 (2.72 to 2.88)	1993-2021	-3.08 (-3.23 to -2.91)	1994-2011	-0.39 (-0.45 to -0.32)
	2	2000-2005	-0.29 (-0.46 to -0.17)	2000-2009	0.65 (0.61 to 0.70)			2004-2007	1.10 (0.90 to 2.87)			2011-2019	-1.53 (-2.07 to -1.31)
	3	2005-2019	0.22 (0.19 to 0.25)	2009-2019	0.22 (0.17 to 0.26)			2007-2011	0.45 (0.14 to 0.79)			2019-2021	1.41 (-0.21 to 2.47)
	4	2019-2021	3.48 (3.13 to 3.78)	2019-2021	2.28 (1.98 to 2.53)			2011-2019	-0.08 (-0.19 to -0.01)				
	5							2019-2021	2.14 (1.76 to 2.44)				
Jamaica	0	1990-1994	2.41 (2.25 to 2.57)	1990-1994	3.18 (2.91 to 3.47)	1990-2000	1.82 (1.35 to 2.41)	1990-1994	3.36 (3.06 to 3.65)	1990-2000	1.58 (1.10 to 2.25)	1990-2000	1.69 (1.25 to 2.04)
	1	1994-2000	0.04 (-0.06 to 0.14)	1994-2001	0.25 (0.10 to 0.38)	2000-2013	-2.01 (-2.56 to -1.71)	1994-2001	0.06 (-0.15 to 0.23)	2000-2013	-2.59 (-3.09 to -2.29)	2000-2003	-2.90 (-3.57 to 1.84)
	2	2000-2006	1.30 (1.04 to 1.39)	2001-2011	2.09 (1.98 to 2.24)	2013-2021	1.27 (-0.28 to 4.23)	2001-2011	1.98 (1.87 to 2.13)	2013-2021	1.47 (-0.34 to 4.73)	2003-2013	-1.03 (-1.52 to 0.19)
	3	2006-2009	2.10 (1.73 to 2.29)	2011-2019	0.95 (0.71 to 1.10)			2011-2018	0.96 (0.62 to 1.15)			2013-2021	1.01 (0.20 to 2.75)
	4	2009-2014	0.96 (0.77 to 1.21)	2019-2021	3.85 (2.62 to 4.56)			2018-2021	2.77 (2.22 to 3.73)				
	5	2014-2019	0.38 (0.04 to 0.55)										
	6	2019-2021	2.26 (1.59 to 2.74)										
Japan	0	1990-2000	1.51 (1.44 to 1.60)	1990-1992	2.47 (1.77 to 2.99)	1990-1993	-1.71 (-3.55 to 0.13)	1990-1996	2.03 (1.92 to 2.15)	1990-1993	-1.69 (-3.24 to -0.25)	1990-1993	0.45 (0.06 to 0.87)
	1	2000-2005	1.11 (0.71 to 1.27)	1992-2005	1.64 (1.40 to 1.68)	1993-1996	-19.42 (-20.83 to -17.54)	1996-1999	4.70 (4.48 to 4.92)	1993-1996	-20.45 (-21.57 to -19.23)	1993-1996	-4.02 (-4.39 to -3.63)
	2	2005-2011	-0.21 (-0.51 to -0.08)	2005-2010	0.06 (-0.26 to 0.37)	1996-2001	-7.01 (-8.83 to -5.24)	1999-2004	1.78 (1.58 to 1.93)	1996-2001	-7.19 (-8.42 to -6.08)	1996-1999	2.65 (2.10 to 3.05)
	3	2011-2015	0.39 (0.17 to 0.66)	2010-2015	0.87 (0.70 to 1.38)	2001-2021	-2.29 (-2.55 to -1.98)	2004-2015	0.65 (0.59 to 0.70)	2001-2011	-2.37 (-2.89 to -0.36)	1999-2005	1.02 (0.69 to 1.27)
	4	2015-2019	4.10 (3.93 to 4.35)	2015-2021	3.16 (3.01 to 3.33)			2015-2019	3.52 (3.36 to 3.69)	2011-2015	-5.12 (-7.19 to -3.26)	2005-2015	0.27 (0.03 to 0.38)
	5	2019-2021	2.36 (1.90 to 2.96)					2019-2021	0.81 (0.36 to 1.26)	2015-2021	0.23 (-0.86 to 2.36)	2015-2019	3.31 (2.97 to 4.10)
	6											2019-2021	0.65 (-0.32 to 1.69)
Jordan	0	1990-1999	1.96 (1.85 to 2.07)	1990-1999	2.76 (2.51 to 3.09)	1990-2006	2.19 (1.78 to 2.70)	1990-1993	2.91 (2.24 to 3.98)	1990-2001	2.28 (1.13 to 3.86)	1990-2006	1.72 (1.50 to 1.97)
	1	1999-2006	0.82 (0.45 to 1.01)	1999-2021	1.75 (1.67 to 1.82)	2006-2009	-15.22 (-16.83 to -10.48)	1993-2002	1.28 (1.08 to 1.41)	2001-2006	-0.18 (-13.64 to 3.44)	2006-2009	-8.93 (-10.07 to -5.81)
	2	2006-2016	1.48 (1.38 to 1.89)			2009-2021	-3.26 (-3.94 to -2.43)	2002-2009	3.85 (3.60 to 4.04)	2006-2009	-13.93 (-15.02 to -0.11)	2009-2021	-1.24 (-1.70 to -0.62)
	3	2016-2021	0.76 (-0.04 to 1.08)					2009-2016	0.71 (0.40 to 0.96)	2009-2019	-4.27 (-13.98 to -3.64)		
	4							2016-2019	2.31 (1.47 to 2.78)	2019-2021	2.73 (-3.94 to 5.78)		
	5							2019-2021	-0.27 (-1.13 to 0.87)				
Kazakhstan	0	1990-1995	2.44 (1.82 to 2.70)	1990-1996	2.91 (2.73 to 3.03)	1990-1992	18.76 (13.46 to 22.32)	1990-1995	2.81 (2.67 to 2.90)	1990-1992	19.39 (12.84 to 23.94)	1990-1994	9.35 (8.46 to 10.34)
	1	1995-2005	3.17 (3.09 to 3.39)	1996-2005	3.45 (3.39 to 3.53)	1992-1995	10.21 (-3.88 to 11.85)	1995-2005	3.34 (3.31 to 3.39)	1992-1995	9.71 (-4.30 to 11.48)	1994-2004	0.07 (-0.45 to 0.36)
	2	2005-2012	2.58 (2.49 to 2.75)	2005-2010	2.92 (2.79 to 3.00)	1995-2005	-3.86 (-5.25 to -2.82)	2005-2010	2.92 (2.79 to 3.01)	1995-2008	-3.79 (-4.37 to -2.68)	2004-2021	1.35 (1.16 to 1.60)
	3	2012-2018	2.25 (2.11 to 2.34)	2010-2018	2.24 (2.20 to 2.27)	2005-2009	-1.41 (-3.43 to 5.08)	2010-2018	2.26 (2.22 to 2.30)	2008-2012	2.20 (-0.34 to 4.67)		
	4	2018-2021	1.05 (0.75 to 1.39)	2018-2021	1.22 (1.09 to 1.33)	2009-2012	5.24 (-2.58 to 6.70)	2018-2021	1.14 (1.01 to 1.26)	2012-2021	-1.78 (-2.90 to -1.17)		
	5					2012-2021	-1.34 (-2.90 to 0.18)						
Kenya	0	1990-1998	0.07 (0.03 to 0.10)	1990-2007	0.66 (0.64 to 0.67)	1990-1996	-1.57 (-1.94 to -1.38)	1990-2001	0.64 (0.60 to 0.67)	1990-1996	-1.46 (-1.81 to -1.26)	1990-1997	-0.90 (-1.01 to -0.80)
	1	1998-2006	0.31 (0.27 to 0.36)	2007-2010	1.03 (0.81 to 1.31)	1996-2000	-0.08 (-0.66 to 0.66)	2001-2007	0.83 (0.68 to 0.91)	1996-2000	0.20 (-0.45 to 0.99)	1997-2001	0.95 (0.37 to 1.12)
	2	2006-2009	1.07 (0.25 to 1.15)	2010-2015	1.66 (1.61 to 1.72)	2000-2004	3.21 (2.81 to 3.79)	2007-2010	1.14 (0.88 to 1.46)	2000-2004	3.42 (3.00 to 4.03)	2001-2004	3.21 (2.65 to 3.44)
	3	2009-2015	1.24 (1.19 to 1.31)	2015-2019	0.59 (0.52 to 0.65)	2004-2012	1.93 (1.75 to 2.07)	2010-2015	1.74 (1.68 to 1.83)	2004-2012	1.69 (1.55 to 1.84)	2004-2008	1.27 (0.98 to 1.53)
	4	2015-2019	0.10 (0.04 to 0.15)	2019-2021	1.86 (1.70 to 2.00)	2012-2021	0.23 (0.09 to 0.35)	2015-2019	0.44 (0.36 to 0.50)	2012-2021	-0.25 (-0.38 to -0.13)	2008-2011	1.98 (1.57 to 2.19)
	5	2019-2021	1.31 (1.17 to 1.44)					2019-2021	1.59 (1.39 to 1.76)			2011-2014	0.64 (0.20 to 1.22)
	6											2014-2021	-0.07 (-0.26 to 0.03)
Kiribati	0	1990-1996	1.40 (1.37 to 1.43)	1990-1995	1.76 (1.66 to 1.86)	1990-2005	1.88 (1.83 to 1.94)	1990-1995	1.83 (1.74 to 1.91)	1990-2005	1.65 (1.61 to 1.71)	1990-1997	1.67 (1.43 to 1.77)
	1	1996-2000	1.97 (1.89 to 2.02)	1995-2000	2.74 (2.61 to 2.84)	2005-2009	0.95 (0.56 to 1.69)	1995-1999	2.73 (2.54 to 2.87)	2005-2010	0.86 (0.54 to 1.32)	1997-2005	1.94 (1.86 to 2.18)
	2	2000-2005	2.16 (2.12 to 2.22)	2000-2004	3.34 (3.22 to 3.50)	2009-2021	0.42 (0.28 to 0.49)	1999-2005	3.23 (3.16 to 3.34)	2010-2021	0.28 (0.14 to 0.36)	2005-2010	1.10 (0.86 to 1.37)
	3	2005-2009	1.71 (1.66 to 1.75)	2004-2008	2.51 (2.39 to 2.64)			2005-2009	2.33 (2.20 to 2.48)			2010-2021	0.49 (0.41 to 0.55)
	4	2009-2013	1.15 (1.11 to 1.19)	2008-2013	1.73 (1.63 to 1.84)			2009-2014	1.59 (1.49 to 1.71)				
	5	2013-2019	0.62 (0.59 to 0.65)	2013-2019	1.13 (0.99 to 1.21)			2014-2019	1.06 (0.89 to 1.15)				
	6	2019-2021	1.26 (1.13 to 1.36)	2019-2021	1.95 (1.59 to 2.14)			2019-2021	1.87 (1.57 to 2.08)				
Kuwait	0	1990-2000	2.32 (2.18 to 2.95)	1990-2010	2.44 (2.01 to 2.62)	1990-1993	-8.99 (-20.93 to 2.52)	1990-2019	2.45 (2.42 to 2.51)	1990-1993	-10.04 (-22.44 to 1.18)	1990-1993	-2.49 (-6.77 to 0.17)
	1	2000-2006	1.94 (1.35 to 2.54)	2010-2019	2.60 (2.48 to 3.08)	1993-1996	15.99 (-1.34 to 22.32)	2019-2021	1.03 (-0.06 to 2.35)	1993-1996	16.75 (4.13 to 23.01)	1993-1998	6.27 (4.96 to 9.55)
	2	2006-2015	2.46 (1.52 to 2.99)	2019-2021	1.06 (0.09 to 2.24)	1996-2021	-1.80 (-2.49 to -1.31)			1996-2021	-2.26 (-2.94 to -1.80)	1998-2001	-2.91 (-4.16 to 0.13)
	3	2015-2021	1.66 (1.23 to 2.00)									2001-2021	0.99 (0.77 to 1.44)
Kyrgyzstan	0	1990-1995	2.85 (2.51 to 3.03)	1990-1995	3.27 (3.02 to 3.42)	1990-1992	17.68 (11.63 to 22.56)	1990-1996	3.34 (3.18 to 3.44)	1990-1994	16.10 (13.91 to 18.37)	1990-1994	9.71 (8.88 to 10.67)
	1	1995-2000	3.54 (3.13 to 3.87)	1995-2005	3.79 (3.74 to 3.85)	1992-1995	9.74 (-5.51 to 11.27)	1996-2005	3.88 (3.82 to 3.95)	1994-2003	-3.45 (-5.00 to -2.75)	1994-2001	-0.08 (-1.03 to 0.35)
	2	2000-2004	4.03 (1.83 to 4.25)	2005-2010	1.83 (1.74 to 1.92)	1995-2003	-3.94 (-5.86 to 2.00)	2005-2010	1.86 (1.76 to 1.96)	2003-2012	0.08 (-1.94 to 0.96)	2001-2012	1.40 (0.80 to 1.87)
	3	2004-2010	1.80 (1.65 to 2.97)	2010-2014	3.22 (3.07 to 3.38)	2003-2012	0.33 (-1.88 to 6.26)	2010-2014	3.30 (3.15 to 3.48)	2012-2015	5.53 (1.69 to 7.38)	2012-2015	4.18 (2.21 to 5.12)
	4	2010-2015	3.03 (1.33 to 3.22)	2014-2017	1.64 (1.40 to 2.07)	2012-2015	6.64 (-3.21 to 8.44)	2014-2017	1.60 (1.35 to 1.92)	2015-2021	-1.91 (-4.97 to -0.52)	2015-2021	-0.16 (-1.34 to 0.49)
	5	2015-2021	0.72 (0.59 to 0.85)	2017-2021	0.81 (0.56 to 0.95)	2015-2021	-2.03 (-4.54 to 0.22)	2017-2021	0.70 (0.49 to 0.83)				
Lao People's Democratic Republic	0	1990-1995	2.44 (2.41 to 2.46)	1990-1999	3.51 (3.50 to 3.53)	1990-1998	1.17 (1.10 to 1.27)	1990-1996	3.48 (3.37 to 3.52)	1990-1993	1.28 (1.01 to 1.65)	1990-1999	1.46 (1.41 to 1.52)
	1	1995-2010	2.55 (2.55 to 2.55)	1999-2002	3.04 (2.99 to 3.29)	1998-2001	0.07 (-0.22 to 0.89)	1996-1999	3.62 (3.05 to 3.66)	1993-1998	0.85 (-0.03 to 0.95)	1999-2012	0.15 (0.11 to 0.18)
	2	2010-2013	2.63 (2.59 to 2.65)	2002-2005	2.84 (2.80 to 2.91)	2001-2008	-0.54 (-0.65 to -0.42)	1999-2018	3.05 (3.05 to 3.06)	1998-2001	-0.18 (-0.89 to 0.20)	2012-2021	0.82 (0.75 to 0.91)
	3	2013-2018	2.51 (2.49 to 2.53)	2005-2018	3.01 (3.00 to 3.02)	2008-2011	-1.00 (-1.13 to -0.63)	2018-2021	2.61 (2.53 to 2.73)	2001-2008	-0.90 (-0.98 to -0.80)		

Location	Segment	Incidence		Prevalence		Mortality		YLD		YLL		DALY	
		Period	APC (95%CI)	Period	APC (95%CI)	Period	APC (95%CI)	Period	APC (95%CI)	Period	APC (95%CI)	Period	APC (95%CI)
	4	2018-2021	2.11 (2.08 to 2.14)	2018-2021	2.71 (2.63 to 2.79)	2011-2021	-0.07 (-0.13 to -0.00)			2008-2011	-1.36 (-1.47 to -1.10)		
	5									2011-2017	-0.54 (-0.72 to -0.42)		
	6									2017-2021	-0.14 (-0.31 to 0.17)		
Latvia	0	1990-1995	2.20 (2.04 to 2.35)	1990-1992	2.21 (1.93 to 2.66)	1990-1994	7.42 (3.37 to 13.73)	1990-1992	2.15 (1.86 to 2.62)	1990-1994	8.28 (3.48 to 15.35)	1990-1994	5.62 (4.04 to 8.36)
	1	1995-2000	4.08 (3.89 to 4.35)	1992-1995	2.89 (2.74 to 4.23)	1994-2005	-2.13 (-5.60 to -1.15)	1992-1995	2.84 (2.69 to 4.27)	1994-2005	-3.20 (-8.11 to -0.55)	1994-2000	0.08 (-2.12 to 1.10)
	2	2000-2004	2.72 (2.30 to 3.22)	1995-2000	4.16 (2.98 to 4.40)	2005-2009	9.27 (4.46 to 15.53)	1995-2000	4.20 (3.02 to 4.39)	2005-2008	8.18 (-6.50 to 12.54)	2000-2013	1.82 (1.35 to 3.19)
	3	2004-2015	2.03 (1.93 to 2.09)	2000-2004	2.98 (2.05 to 3.18)	2009-2013	-2.65 (-9.01 to 0.97)	2000-2004	3.02 (2.14 to 3.20)	2008-2013	-1.47 (-9.31 to 2.34)	2013-2018	6.75 (5.29 to 9.85)
	4	2015-2018	3.41 (3.04 to 3.63)	2004-2015	2.10 (2.04 to 2.16)	2013-2017	14.12 (9.91 to 21.90)	2004-2015	2.15 (2.09 to 2.20)	2013-2017	16.37 (11.29 to 25.00)	2018-2021	-0.74 (-4.57 to 1.57)
	5	2018-2021	0.61 (0.28 to 0.92)	2015-2018	3.13 (2.90 to 3.27)	2017-2021	-0.05 (-7.98 to 3.49)	2015-2018	3.20 (2.97 to 3.34)	2017-2021	-0.23 (-9.21 to 3.85)		
	6			2018-2021	1.18 (0.94 to 1.37)			2018-2021	0.96 (0.76 to 1.15)				
Lebanon	0	1990-1995	2.18 (1.81 to 2.37)	1990-1994	2.45 (1.76 to 2.78)	1990-2014	-1.18 (-1.26 to -1.09)	1990-1994	2.43 (1.73 to 2.77)	1990-2021	-1.73 (-1.81 to -1.66)	1990-2000	0.18 (-0.05 to 0.31)
	1	1995-1999	3.08 (2.77 to 3.53)	1994-2000	3.27 (3.08 to 3.86)	2014-2018	-2.81 (-3.98 to -1.21)	1994-2003	3.16 (3.05 to 3.71)			2000-2006	0.98 (0.68 to 1.67)
	2	1999-2007	1.52 (1.34 to 1.63)	2000-2005	1.44 (0.86 to 1.77)	2018-2021	0.11 (-1.65 to 2.25)	2003-2009	2.51 (2.02 to 2.79)			2006-2021	0.35 (0.20 to 0.43)
	3	2007-2019	2.20 (2.14 to 2.29)	2005-2019	2.43 (2.37 to 2.53)			2009-2016	1.21 (0.89 to 1.33)				
	4	2019-2021	0.45 (0.03 to 1.19)	2019-2021	0.62 (0.13 to 1.57)			2016-2019	2.90 (2.39 to 3.30)				
	5							2019-2021	0.04 (-0.60 to 0.84)				
Lesotho	0	1990-1995	2.19 (1.88 to 2.40)	1990-1995	2.86 (2.03 to 3.19)	1990-1997	-1.38 (-1.85 to -0.93)	1990-1994	2.67 (1.88 to 3.01)	1990-1997	-1.94 (-2.47 to -1.45)	1990-1997	-1.06 (-1.42 to -0.72)
	1	1995-2005	3.12 (3.04 to 3.20)	1995-2006	3.60 (3.49 to 3.76)	1997-2000	16.80 (15.16 to 17.98)	1994-2006	3.50 (3.43 to 3.59)	1997-2000	17.52 (15.68 to 18.87)	1997-2000	14.76 (13.49 to 15.60)
	2	2005-2013	4.26 (4.19 to 4.36)	2006-2014	5.11 (5.01 to 5.29)	2000-2005	9.22 (8.53 to 9.72)	2006-2014	5.19 (5.09 to 5.34)	2000-2005	10.18 (9.26 to 10.74)	2000-2005	9.55 (8.94 to 10.06)
	3	2013-2017	2.40 (2.25 to 2.56)	2014-2017	3.41 (2.95 to 3.91)	2005-2015	1.40 (1.14 to 1.68)	2014-2017	3.39 (2.91 to 3.92)	2005-2015	1.36 (1.07 to 1.69)	2005-2011	1.32 (0.62 to 1.71)
	4	2017-2021	0.68 (0.49 to 0.86)	2017-2021	1.46 (1.14 to 1.70)	2015-2021	-1.29 (-2.09 to -0.71)	2017-2021	1.28 (0.96 to 1.53)	2015-2021	-1.38 (-2.32 to -0.71)	2011-2014	3.45 (2.16 to 4.07)
	5											2014-2021	-0.78 (-1.23 to -0.44)
Liberia	0	1990-1995	1.56 (1.44 to 1.68)	1990-1995	2.39 (2.29 to 2.49)	1990-2000	2.64 (2.55 to 2.74)	1990-1995	2.35 (2.24 to 2.43)	1990-1999	2.79 (2.64 to 2.94)	1990-1999	2.75 (2.65 to 2.86)
	1	1995-2009	2.22 (2.20 to 2.25)	1995-2003	2.97 (2.93 to 3.05)	2000-2007	1.21 (0.97 to 1.37)	1995-2004	2.91 (2.88 to 3.00)	1999-2007	0.95 (0.70 to 1.14)	1999-2007	1.45 (1.28 to 1.58)
	2	2009-2014	1.66 (1.59 to 2.20)	2003-2009	2.74 (2.64 to 2.80)	2007-2010	4.23 (3.64 to 4.59)	2004-2009	2.71 (2.59 to 2.83)	2007-2010	4.41 (3.39 to 4.90)	2007-2010	3.90 (3.34 to 4.23)
	3	2014-2018	1.38 (1.22 to 1.51)	2009-2014	2.14 (2.06 to 2.25)	2010-2014	0.51 (0.11 to 0.98)	2009-2013	2.20 (2.05 to 2.38)	2010-2013	1.37 (-0.10 to 2.10)	2010-2013	1.56 (0.62 to 1.95)
	4	2018-2021	1.85 (1.68 to 2.16)	2014-2018	1.75 (1.62 to 1.85)	2014-2018	-1.88 (-2.53 to -1.42)	2013-2018	1.79 (1.61 to 1.86)	2013-2018	-1.86 (-2.69 to -1.54)	2013-2018	-0.84 (-1.37 to -0.62)
	5			2018-2021	2.32 (2.18 to 2.57)	2018-2021	0.37 (-0.22 to 1.35)	2018-2021	2.23 (2.08 to 2.47)	2018-2021	0.22 (-0.57 to 1.54)	2018-2021	0.85 (0.29 to 1.73)
Libya	0	1990-2009	3.70 (3.63 to 3.77)	1990-2008	4.20 (3.98 to 4.37)	1990-1996	-1.04 (-2.31 to -0.35)	1990-2007	4.19 (4.08 to 4.31)	1990-1996	-0.94 (-1.30 to -0.56)	1990-1996	1.56 (1.03 to 1.85)
	1	2009-2016	2.05 (1.47 to 3.13)	2008-2016	2.29 (1.53 to 4.44)	1996-2011	4.90 (4.70 to 5.09)	2007-2019	2.57 (2.42 to 3.65)	1996-1999	5.75 (4.74 to 6.27)	1996-1999	5.27 (4.25 to 5.77)
	2	2016-2019	3.20 (2.47 to 3.70)	2016-2019	3.38 (2.49 to 4.00)	2011-2021	-0.08 (-0.56 to 0.43)	2019-2021	0.35 (-0.94 to 2.29)	1999-2010	4.38 (3.90 to 4.54)	1999-2009	4.19 (3.21 to 4.33)
	3	2019-2021	0.06 (-0.88 to 1.23)	2019-2021	-0.07 (-1.27 to 1.46)					2010-2018	0.91 (0.56 to 1.99)	2009-2019	1.82 (1.68 to 2.05)
	4									2018-2021	-1.69 (-3.84 to -0.04)	2019-2021	-1.06 (-2.06 to 0.18)
Lithuania	0	1990-1995	1.81 (1.39 to 2.07)	1990-1995	2.26 (1.88 to 2.50)	1990-1995	3.45 (1.82 to 5.41)	1990-1995	2.17 (1.80 to 2.39)	1990-1995	3.61 (1.68 to 5.98)	1990-1993	3.28 (1.42 to 6.02)
	1	1995-2000	3.31 (3.02 to 3.69)	1995-2000	3.69 (3.40 to 3.97)	1995-2002	-4.34 (-6.86 to -3.24)	1995-2000	3.72 (3.42 to 4.02)	1995-2003	-4.84 (-7.03 to -3.83)	1993-2013	1.14 (0.25 to 1.26)
	2	2000-2015	1.73 (1.68 to 1.77)	2000-2015	1.91 (1.86 to 1.94)	2002-2007	2.48 (0.82 to 6.43)	2000-2015	1.89 (1.83 to 1.93)	2003-2007	4.50 (1.46 to 8.58)	2013-2018	7.43 (6.32 to 9.99)
	3	2015-2018	3.69 (3.13 to 3.96)	2015-2018	3.57 (3.11 to 3.79)	2007-2010	-6.31 (-8.38 to -2.97)	2015-2018	3.75 (3.25 to 3.99)	2007-2010	-7.78 (-10.08 to -3.60)	2018-2021	1.64 (-0.99 to 3.34)
	4	2018-2021	1.08 (0.49 to 1.45)	2018-2021	1.56 (1.07 to 1.88)	2010-2014	7.15 (4.11 to 10.98)	2018-2021	1.37 (0.89 to 1.72)	2010-2014	7.76 (3.43 to 12.10)		
	5					2014-2018	19.54 (16.78 to 23.20)			2014-2018	18.67 (15.81 to 22.66)		
	6					2018-2021	1.40 (-2.88 to 5.23)			2018-2021	1.13 (-3.74 to 5.05)		
Luxembourg	0	1990-1995	1.48 (1.43 to 1.51)	1990-1995	1.99 (1.94 to 2.01)	1990-1992	4.54 (-0.54 to 8.41)	1990-1996	1.97 (1.90 to 1.99)	1990-1992	3.18 (-1.12 to 6.49)	1990-1992	2.37 (0.62 to 3.65)
	1	1995-1999	1.66 (1.61 to 1.72)	1995-1999	2.09 (2.06 to 2.14)	1992-1998	-5.27 (-8.14 to -4.11)	1996-1999	2.09 (2.02 to 2.12)	1992-1999	-5.34 (-7.66 to -4.66)	1992-1998	-1.73 (-2.61 to -1.30)
	2	1999-2005	1.57 (1.51 to 1.59)	1999-2006	1.94 (1.91 to 1.95)	1998-2014	-1.45 (-1.83 to -0.79)	1999-2006	1.91 (1.87 to 1.93)	1999-2003	0.43 (-1.72 to 2.67)	1998-2021	0.63 (0.54 to 0.72)
	3	2005-2012	1.78 (1.61 to 1.80)	2006-2012	2.11 (2.08 to 2.13)	2014-2021	-4.75 (-7.35 to -3.43)	2006-2012	2.14 (2.07 to 2.17)	2003-2015	-2.35 (-3.35 to -1.79)		
	4	2012-2015	1.87 (1.80 to 1.90)	2012-2015	2.25 (2.20 to 2.28)			2012-2015	2.30 (2.23 to 2.36)	2015-2021	-5.12 (-9.19 to -3.71)		
	5	2015-2019	2.38 (2.35 to 2.43)	2015-2019	2.72 (2.69 to 2.75)			2015-2019	2.75 (2.71 to 2.79)				
	6	2019-2021	2.16 (2.07 to 2.25)	2019-2021	2.14 (2.07 to 2.20)			2019-2021	2.03 (1.94 to 2.09)				
Madagascar	0	1990-1994	0.81 (0.46 to 1.00)	1990-2009	1.72 (1.70 to 1.75)	1990-2004	0.16 (0.06 to 0.32)	1990-2009	1.76 (1.73 to 1.78)	1990-2004	0.01 (-0.10 to 0.20)	1990-2004	0.32 (0.23 to 0.50)
	1	1994-2010	1.09 (1.05 to 1.13)	2009-2015	2.12 (1.98 to 2.36)	2004-2007	-1.45 (-1.88 to -0.45)	2009-2015	2.18 (1.85 to 2.42)	2004-2007	-1.62 (-2.09 to -0.51)	2004-2007	-0.85 (-1.23 to 0.02)
	2	2010-2015	1.32 (1.19 to 1.52)	2015-2019	1.08 (0.80 to 1.30)	2007-2021	0.42 (0.28 to 0.64)	2015-2019	0.93 (0.63 to 2.08)	2007-2021	0.31 (0.15 to 0.56)	2007-2021	0.64 (0.52 to 0.83)
	3	2015-2019	0.29 (0.03 to 0.47)	2019-2021	2.10 (1.57 to 2.41)			2019-2021	1.85 (1.22 to 2.19)				
	4	2019-2021	1.30 (0.86 to 1.58)										
Malawi	0	1990-1993	0.41 (0.26 to 0.63)	1990-1994	1.41 (1.28 to 1.59)	1990-1992	-0.16 (-0.82 to 1.09)	1990-1994	1.41 (1.26 to 1.62)	1990-1992	-0.13 (-0.77 to 0.94)	1990-1992	0.14 (-0.41 to 1.02)
	1	1993-2011	0.17 (0.15 to 0.18)	1994-2000	0.75 (0.62 to 0.84)	1992-1998	2.18 (1.91 to 2.78)	1994-2000	0.74 (0.60 to 0.82)	1992-1998	2.21 (2.01 to 2.64)	1992-1998	2.01 (1.82 to 2.38)
	2	2011-2015	1.01 (0.93 to 1.09)	2000-2004	1.31 (1.14 to 1.48)	1998-2004	-0.19 (-0.43 to 0.23)	2000-2004	1.45 (1.28 to 1.65)	1998-2004	-0.47 (-0.68 to 0.03)	1998-2004	-0.23 (-0.42 to 0.17)
	3	2015-2019	0.33 (0.18 to 0.44)	2004-2011	0.45 (0.40 to 0.51)	2004-2010	-1.10 (-1.41 to -0.84)	2004-2011	0.47 (0.41 to 0.53)	2004-2010	-1.37 (-1.66 to -1.09)	2004-2010	-1.11 (-1.34 to -0.81)
	4	2019-2021	0.98 (0.72 to 1.15)	2011-2014	2.28 (2.06 to 2.39)	2010-2014	-2.41 (-2.96 to -2.01)	2011-2014	2.23 (2.04 to 2.36)	2010-2014	-2.63 (-3.10 to -2.27)	2010-2014	-1.89 (-2.25 to -1.54)
	5			2014-2019	1.46 (1.32 to 1.54)	2014-2021	0.47 (0.25 to 0.73)	2014-2021	1.37 (1.29 to 1.42)	2014-2021	0.22 (0.02 to 0.42)	2014-2021	0.45 (0.28 to 0.64)
	6			2019-2021	1.87 (1.59 to 2.05)								
Malaysia	0	1990-1994	1.04 (0.88 to 1.12)	1990-1993	2.78 (2.63 to 2.94)	1990-1994	-3.21 (-6.91 to -1.35)	1990-1994	3.61 (3.38 to 3.86)	1990-2003	-0.91 (-1.38 to -0.26)	1990-1996	-0.70 (-2.21 to -0.01)
	1	1994-2000	1.23 (1.18 to 1.33)	1993-1996	1.34 (1.22 to 1.49)	1994-2002	0.50 (-0.22 to 3.22)	1994-2005	0.37 (0.31 to 0.44)	2003-2011	-4.10 (-6.03 to -3.17)	1996-1999	1.63 (-1.44 to 2.40)
	2	2000-2005	0.86 (0.79 to 0.92)	1996-2000	2.16 (2.08 to 2.29)	2002-2012	-3.33 (-4.36 to -1.52)	2005-2011	3.43 (3.24 to 3.58)	2011-2021	0.75 (0.02 to 1.65)	1999-2008	-1.37 (-2.00 to 0.58)

Location	Segment	Incidence		Prevalence		Mortality		YLD		YLL		DALY	
		Period	APC (95%CI)	Period	APC (95%CI)	Period	APC (95%CI)	Period	APC (95%CI)	Period	APC (95%CI)	Period	APC (95%CI)
	3	2005-2011	2.09 (2.03 to 2.15)	2000-2006	1.14 (1.09 to 1.20)	2012-2015	3.06 (-3.74 to 4.51)	2011-2021	1.70 (1.60 to 1.79)			2008-2021	1.03 (0.71 to 1.38)
	4	2011-2015	1.78 (1.66 to 1.93)	2006-2009	2.62 (2.47 to 2.73)	2015-2021	-0.84 (-3.88 to 0.40)						
	5	2015-2018	2.34 (2.11 to 2.44)	2009-2014	2.11 (1.98 to 2.19)								
	6	2018-2021	2.00 (1.78 to 2.13)	2014-2021	1.55 (1.48 to 1.61)								
Maldives	0	1990-2000	1.06 (1.02 to 1.10)	1990-2005	1.62 (1.57 to 1.65)	1990-1997	-1.50 (-2.46 to -0.20)	1990-2005	1.67 (1.65 to 1.70)	1990-1997	-2.29 (-3.30 to -0.89)	1990-1997	-1.46 (-2.17 to -0.34)
	1	2000-2004	1.40 (1.26 to 1.56)	2005-2010	1.30 (1.12 to 1.66)	1997-2006	-5.55 (-6.92 to -4.92)	2005-2010	1.34 (1.18 to 1.43)	1997-2007	-6.24 (-7.25 to -5.68)	1997-2007	-3.77 (-4.96 to -3.29)
	2	2004-2010	0.92 (0.83 to 0.98)	2010-2019	2.80 (1.32 to 2.92)	2006-2021	-1.71 (-2.05 to -1.30)	2010-2019	2.76 (2.73 to 2.84)	2007-2021	-1.96 (-2.31 to -1.56)	2007-2021	0.29 (-0.08 to 0.72)
	3	2010-2019	2.34 (2.31 to 2.42)	2019-2021	2.36 (2.06 to 2.76)			2019-2021	2.13 (1.92 to 2.51)				
	4	2019-2021	1.81 (1.59 to 2.18)										
Mali	0	1990-1992	1.79 (1.62 to 2.04)	1990-1995	2.63 (2.27 to 2.85)	1990-1993	1.72 (1.08 to 2.05)	1990-1992	2.14 (1.86 to 2.54)	1990-1993	1.51 (0.84 to 1.85)	1990-1993	1.81 (1.32 to 2.09)
	1	1992-1995	2.14 (2.06 to 2.90)	1995-2000	4.18 (3.76 to 4.41)	1993-1996	3.24 (2.82 to 3.48)	1992-1995	2.79 (2.65 to 3.89)	1993-1996	3.21 (2.77 to 3.46)	1993-1997	3.06 (2.88 to 3.34)
	2	1995-2000	2.86 (2.22 to 2.97)	2000-2014	2.40 (2.35 to 3.47)	1996-2002	2.00 (1.82 to 2.13)	1995-2000	3.87 (2.60 to 4.05)	1996-2002	1.65 (1.51 to 1.78)	1997-2002	2.20 (2.02 to 2.34)
	3	2000-2005	2.23 (1.94 to 2.32)	2014-2019	0.48 (0.20 to 2.41)	2002-2005	0.27 (0.09 to 0.68)	2000-2004	2.59 (2.27 to 2.99)	2002-2005	-0.16 (-0.35 to 0.27)	2002-2005	0.73 (0.58 to 1.06)
	4	2005-2014	2.00 (1.96 to 2.04)	2019-2021	1.40 (0.60 to 1.77)	2005-2014	0.90 (0.82 to 1.09)	2004-2014	2.35 (2.29 to 2.42)	2005-2014	0.71 (0.63 to 0.86)	2005-2014	1.33 (1.28 to 1.47)
	5	2014-2019	0.90 (0.84 to 0.95)			2014-2021	-0.19 (-0.34 to -0.04)	2014-2019	0.64 (0.50 to 0.72)	2014-2021	-0.26 (-0.43 to -0.12)	2014-2021	0.17 (0.06 to 0.29)
	6	2019-2021	1.74 (1.51 to 1.88)					2019-2021	1.42 (1.06 to 1.64)				
Malta	0	1990-1995	1.18 (1.07 to 1.29)	1990-1993	2.26 (1.99 to 2.67)	1990-1992	-0.00 (-4.61 to 4.02)	1990-1993	2.22 (1.99 to 2.59)	1990-1992	-1.11 (-7.35 to 3.27)	1990-1992	-0.27 (-2.58 to 1.71)
	1	1995-2000	0.12 (-0.03 to 0.23)	1993-2001	1.66 (1.54 to 1.72)	1992-1996	-10.53 (-13.81 to -8.62)	1993-2001	1.52 (1.41 to 1.59)	1992-1996	-10.35 (-13.86 to -0.16)	1992-1996	-6.89 (-8.17 to -6.28)
	2	2000-2003	1.38 (0.77 to 1.54)	2001-2005	2.90 (2.62 to 3.10)	1996-2008	0.08 (-0.42 to 0.70)	2001-2005	2.76 (1.53 to 2.88)	1996-2008	-0.13 (-1.24 to 0.72)	1996-2008	0.91 (0.67 to 1.26)
	3	2003-2006	2.18 (1.98 to 2.87)	2005-2019	3.81 (3.78 to 3.85)	2008-2011	-12.37 (-13.94 to -8.28)	2005-2010	3.90 (2.83 to 4.10)	2008-2011	-12.37 (-13.96 to -6.63)	2008-2011	-3.61 (-4.65 to -1.24)
	4	2006-2010	3.67 (3.46 to 3.92)	2019-2021	2.93 (2.60 to 3.34)	2011-2021	0.63 (-0.18 to 1.63)	2010-2019	3.74 (3.63 to 3.80)	2011-2021	0.13 (-0.78 to 1.32)	2011-2021	2.22 (1.70 to 3.10)
	5	2010-2021	3.28 (3.21 to 3.33)					2019-2021	2.90 (2.63 to 3.28)				
Marshall Islands	0	1990-1998	2.48 (2.42 to 2.55)	1990-1996	3.37 (3.23 to 3.56)	1990-1996	5.29 (5.18 to 5.41)	1990-1996	3.33 (3.20 to 3.50)	1990-1996	5.53 (5.45 to 5.61)	1990-1995	5.16 (5.07 to 5.24)
	1	1998-2005	2.16 (2.04 to 2.23)	1996-2005	2.73 (2.62 to 2.81)	1996-2001	4.49 (4.34 to 4.64)	1996-2005	2.69 (2.58 to 2.77)	1996-2000	4.63 (4.52 to 4.80)	1995-2000	4.41 (4.34 to 4.51)
	2	2005-2009	3.19 (3.06 to 3.35)	2005-2010	4.27 (4.12 to 4.43)	2001-2005	2.80 (2.68 to 2.96)	2005-2010	4.01 (3.86 to 4.27)	2000-2003	3.43 (3.27 to 3.64)	2000-2003	3.20 (3.06 to 3.39)
	3	2009-2013	1.94 (1.79 to 2.05)	2010-2015	1.95 (1.78 to 2.15)	2005-2011	0.14 (-0.07 to 0.24)	2010-2015	1.88 (1.72 to 2.09)	2003-2006	2.02 (1.85 to 2.18)	2003-2006	2.20 (2.06 to 2.33)
	4	2013-2021	1.03 (0.98 to 1.08)	2015-2021	1.06 (0.90 to 1.20)	2011-2015	0.63 (0.39 to 0.92)	2015-2021	1.09 (0.92 to 1.21)	2006-2010	-0.21 (-0.43 to -0.05)	2006-2016	0.67 (0.63 to 0.71)
	5					2015-2021	-0.03 (-0.31 to 0.10)			2010-2016	0.40 (0.29 to 0.70)	2016-2021	0.04 (-0.11 to 0.17)
	6									2016-2021	-0.27 (-0.50 to -0.11)		
Mauritania	0	1990-1994	1.88 (1.75 to 2.02)	1990-1994	3.27 (3.09 to 3.45)	1990-1994	1.14 (0.25 to 1.66)	1990-1992	4.01 (3.65 to 4.38)	1990-1995	0.91 (0.07 to 1.29)	1990-2003	1.28 (1.19 to 1.36)
	1	1994-2001	0.15 (0.09 to 0.22)	1994-2001	0.27 (0.20 to 0.36)	1994-2002	1.80 (-0.23 to 2.27)	1992-1995	1.95 (1.73 to 2.15)	1995-2001	1.71 (1.41 to 2.37)	2003-2016	0.27 (0.17 to 0.35)
	2	2001-2005	1.74 (1.56 to 1.87)	2001-2009	2.40 (2.20 to 2.45)	2002-2016	-0.18 (-0.32 to 0.09)	1995-2000	0.05 (-0.08 to 0.16)	2001-2005	0.21 (-0.47 to 0.98)	2016-2021	1.88 (1.53 to 2.40)
	3	2005-2014	2.08 (2.04 to 2.16)	2009-2014	2.69 (2.55 to 2.91)	2016-2021	1.85 (1.38 to 2.55)	2000-2003	1.73 (1.44 to 2.00)	2005-2016	-0.56 (-0.96 to 0.17)		
	4	2014-2019	0.96 (0.89 to 1.03)	2014-2019	1.61 (1.49 to 1.72)			2003-2014	2.60 (2.56 to 2.66)	2016-2021	1.78 (1.28 to 2.53)		
	5	2019-2021	2.95 (2.73 to 3.13)	2019-2021	3.87 (3.59 to 4.12)			2014-2019	1.70 (1.52 to 1.85)				
	6							2019-2021	3.68 (3.26 to 3.99)				
Mauritius	0	1990-1993	4.08 (3.73 to 4.58)	1990-1994	4.22 (3.86 to 4.92)	1990-1992	5.90 (-4.08 to 13.30)	1990-1994	4.22 (3.88 to 4.92)	1990-1992	5.51 (-1.29 to 12.47)	1990-1992	5.10 (0.30 to 9.42)
	1	1993-1999	3.18 (2.93 to 3.38)	1994-2000	3.21 (2.76 to 3.51)	1992-1995	-10.81 (-13.31 to 4.33)	1994-2000	3.17 (2.81 to 3.48)	1992-1995	-12.30 (-14.64 to -8.07)	1992-1995	-7.07 (-8.88 to -3.80)
	2	1999-2008	2.40 (2.31 to 2.53)	2000-2011	2.47 (2.33 to 2.56)	1995-2003	5.16 (3.40 to 8.20)	2000-2012	2.47 (2.35 to 2.56)	1995-2003	4.84 (3.38 to 6.56)	1995-2003	4.07 (2.89 to 5.56)
	3	2008-2014	1.92 (1.75 to 2.21)	2011-2019	1.78 (1.63 to 1.92)	2003-2006	28.48 (17.77 to 31.46)	2012-2019	1.60 (1.42 to 1.97)	2003-2006	25.28 (17.09 to 27.92)	2003-2006	18.17 (12.65 to 19.99)
	4	2014-2019	1.17 (0.99 to 1.70)	2019-2021	0.02 (-0.39 to 0.73)	2006-2021	-1.98 (-2.51 to -1.50)	2019-2021	0.24 (-0.22 to 1.05)	2006-2021	-1.70 (-2.16 to -1.29)	2006-2021	-0.70 (-1.02 to -0.44)
	5	2019-2021	0.35 (0.07 to 0.85)										
Mexico	0	1990-1992	2.75 (2.51 to 2.99)	1990-1993	3.00 (2.82 to 3.21)	1990-1996	0.16 (-0.68 to 1.17)	1990-1993	3.10 (2.93 to 3.30)	1990-1996	0.52 (-0.42 to 1.64)	1990-1996	0.99 (0.55 to 1.49)
	1	1992-1995	1.15 (1.01 to 1.28)	1993-2004	0.82 (0.78 to 0.87)	1996-2000	-6.49 (-8.54 to -4.96)	1993-1996	0.99 (0.81 to 1.16)	1996-2000	-6.78 (-9.04 to -4.91)	1996-2000	-4.55 (-5.75 to -3.61)
	2	1995-2000	-0.14 (-0.26 to -0.05)	2004-2010	0.38 (0.25 to 0.52)	2000-2004	3.20 (1.66 to 5.75)	1996-2000	0.40 (0.20 to 0.51)	2000-2005	1.80 (0.68 to 4.18)	2000-2004	2.12 (1.07 to 3.59)
	3	2000-2015	0.19 (0.17 to 0.21)	2010-2019	-0.04 (-0.11 to 0.02)	2004-2007	-6.39 (-7.69 to -3.66)	2000-2005	0.76 (0.65 to 0.98)	2005-2008	-7.04 (-8.32 to -4.22)	2004-2008	-3.16 (-4.58 to -2.04)
	4	2015-2019	-0.42 (-0.60 to -0.29)	2019-2021	1.72 (1.29 to 2.00)	2007-2021	0.88 (0.54 to 1.31)	2005-2012	0.28 (0.19 to 0.37)	2008-2021	1.41 (0.99 to 1.90)	2008-2021	0.79 (0.58 to 1.04)
	5	2019-2021	1.58 (1.36 to 1.79)					2012-2019	-0.09 (-0.18 to -0.03)				
	6							2019-2021	1.56 (1.25 to 1.80)				
Micronesia (Federated States of)	0	1990-2000	2.88 (2.82 to 2.94)	1990-2000	3.89 (3.76 to 4.06)	1990-1995	1.70 (1.42 to 1.98)	1990-2000	3.86 (3.54 to 4.28)	1990-1995	1.53 (1.27 to 1.79)	1990-1995	1.96 (1.51 to 2.22)
	1	2000-2005	2.06 (1.89 to 2.21)	2000-2005	2.37 (1.98 to 2.68)	1995-2002	4.44 (4.30 to 4.61)	2000-2005	2.41 (2.11 to 4.14)	1995-2002	4.24 (4.11 to 4.40)	1995-2001	4.19 (4.02 to 4.50)
	2	2005-2009	4.06 (3.91 to 4.22)	2005-2009	5.71 (5.36 to 6.10)	2002-2005	1.84 (1.49 to 2.37)	2005-2009	5.48 (2.24 to 5.75)	2002-2005	1.65 (1.32 to 2.16)	2001-2004	2.77 (2.14 to 3.27)
	3	2009-2012	1.82 (1.53 to 2.00)	2009-2012	1.75 (1.29 to 3.09)	2005-2011	-1.31 (-1.62 to -1.15)	2009-2012	1.82 (1.49 to 5.74)	2005-2011	-1.31 (-1.62 to -1.15)	2004-2012	0.19 (-0.12 to 0.32)
	4	2012-2021	0.66 (0.60 to 0.72)	2012-2021	0.86 (0.63 to 0.96)	2011-2021	0.41 (0.30 to 0.53)	2012-2018	1.05 (0.87 to 1.95)	2011-2021	0.37 (0.26 to 0.48)	2012-2021	0.50 (0.37 to 0.86)
	5							2018-2021	0.42 (-0.25 to 0.89)				
Monaco	0	1990-1993	1.95 (1.73 to 2.09)	1990-1992	2.10 (1.97 to 2.28)	1990-1997	-0.49 (-0.66 to -0.32)	1990-1994	2.22 (2.13 to 2.30)	1990-1997	-0.56 (-0.77 to -0.36)	1990-1993	1.20 (1.07 to 1.30)
	1	1993-1996	2.47 (2.23 to 2.84)	1992-1995	2.50 (2.43 to 2.99)	1997-2002	-2.11 (-2.33 to -1.89)	1994-2001	2.99 (2.93 to 3.04)	1997-2002	-2.39 (-2.65 to -2.14)	1993-1998	1.86 (1.82 to 1.95)
	2	1996-2000	3.04 (2.86 to 3.15)	1995-2001	3.03 (2.75 to 3.09)	2002-2010	0.38 (0.28 to 0.53)	2001-2012	2.51 (2.46 to 2.78)	2002-2011	0.26 (0.16 to 0.39)	1998-2001	1.44 (1.37 to 1.57)
	3	2000-2015	2.38 (2.36 to 2.39)	2001-2012	2.52 (2.49 to 2.55)	2010-2021	-0.01 (-0.11 to 0.06)	2012-2015	2.76 (2.52 to 3.00)	2011-2021	-0.26 (-0.38 to -0.18)	2001-2008	1.98 (1.89 to 2.03)
	4	2015-2019	3.24 (3.18 to 3.31)	2012-2015	2.74 (2.54 to 2.95)			2015-2019	3.34 (3.25 to 3.48)			2008-2015	2.11 (2.07 to 2.20)
	5	2019-2021	2.45 (2.24 to 2.71)	2015-2019	3.32 (3.25 to 3.42)			2019-2021	2.29 (2.10 to 2.48)			2015-2019	2.81 (2.74 to 2.92)
	6			2019-2021	2.31 (2.17 to 2.44)							2019-2021	1.83 (1.65 to 2.03)

Location	Segment	Incidence		Prevalence		Mortality		YLD		YLL		DALY	
		Period	APC (95%CI)	Period	APC (95%CI)	Period	APC (95%CI)	Period	APC (95%CI)	Period	APC (95%CI)	Period	APC (95%CI)
Mongolia	0	1990-1995	1.83 (1.64 to 1.96)	1990-1995	2.19 (2.07 to 2.27)	1990-1992	5.49 (1.61 to 8.90)	1990-1995	2.15 (1.95 to 2.25)	1990-1992	6.93 (2.62 to 10.28)	1990-1992	4.29 (2.32 to 5.86)
	1	1995-2004	2.43 (2.37 to 2.52)	1995-1999	2.71 (2.59 to 2.85)	1992-2017	1.46 (1.19 to 1.59)	1995-1999	2.68 (2.55 to 2.85)	1992-2017	1.09 (0.96 to 1.21)	1992-2010	1.81 (1.52 to 1.88)
	2	2004-2010	2.03 (1.91 to 2.12)	1999-2008	2.43 (2.38 to 2.46)	2017-2021	-2.55 (-4.82 to -1.18)	1999-2010	2.47 (2.42 to 2.49)	2017-2021	-2.63 (-4.79 to -1.31)	2010-2017	2.68 (2.28 to 3.85)
	3	2010-2015	4.02 (3.93 to 4.11)	2008-2011	3.05 (2.87 to 3.13)			2010-2015	3.98 (3.92 to 4.06)			2017-2021	0.16 (-1.24 to 0.97)
	4	2015-2021	2.30 (2.21 to 2.37)	2011-2014	4.09 (3.96 to 4.20)			2015-2018	2.80 (2.64 to 3.21)				
	5			2014-2017	3.20 (3.06 to 3.31)			2018-2021	2.14 (1.87 to 2.27)				
	6			2017-2021	2.32 (2.22 to 2.40)								
Montenegro	0	1990-1995	1.49 (1.21 to 1.63)	1990-1994	1.81 (1.23 to 2.10)	1990-2003	3.02 (2.75 to 3.31)	1990-1994	1.79 (1.23 to 2.05)	1990-2002	3.27 (3.05 to 3.50)	1990-1992	1.49 (1.00 to 2.50)
	1	1995-1999	2.31 (2.10 to 2.59)	1994-2001	2.34 (2.20 to 2.69)	2003-2007	-3.84 (-5.65 to -2.20)	1994-2001	2.34 (2.22 to 2.68)	2002-2008	-2.01 (-2.72 to -1.43)	1992-2002	2.73 (2.64 to 2.94)
	2	1999-2004	1.68 (1.51 to 1.84)	2001-2014	1.54 (1.49 to 1.60)	2007-2016	2.99 (2.54 to 3.92)	2001-2014	1.54 (1.48 to 1.61)	2008-2018	1.33 (1.05 to 1.78)	2002-2007	-0.12 (-0.47 to 0.15)
	3	2004-2016	1.30 (1.27 to 1.34)	2014-2019	0.80 (0.38 to 1.01)	2016-2021	0.05 (-1.66 to 1.08)	2014-2019	0.81 (0.40 to 1.54)	2018-2021	-1.94 (-4.36 to -0.45)	2007-2017	1.29 (1.19 to 1.46)
	4	2016-2019	0.16 (-0.03 to 0.49)	2019-2021	1.91 (1.22 to 2.31)			2019-2021	1.70 (0.99 to 2.10)			2017-2021	0.20 (-0.38 to 0.57)
	5	2019-2021	2.40 (1.96 to 2.74)										
Morocco	0	1990-1998	3.64 (3.59 to 3.73)	1990-1999	4.03 (4.00 to 4.07)	1990-1998	1.40 (1.29 to 1.48)	1990-1998	4.02 (3.98 to 4.07)	1990-1998	1.39 (1.09 to 1.52)	1990-2000	2.96 (2.91 to 3.01)
	1	1998-2010	3.46 (3.43 to 3.49)	1999-2005	3.68 (3.57 to 3.74)	1998-2001	2.45 (2.08 to 2.66)	1998-2005	3.67 (3.58 to 3.72)	1998-2001	2.40 (1.84 to 2.75)	2000-2007	4.28 (4.21 to 4.36)
	2	2010-2019	2.72 (2.69 to 2.76)	2005-2010	4.28 (4.22 to 4.36)	2001-2004	6.20 (5.87 to 6.49)	2005-2010	4.13 (4.06 to 4.22)	2001-2004	6.38 (5.67 to 6.82)	2007-2010	3.28 (2.99 to 3.75)
	3	2019-2021	2.12 (1.93 to 2.40)	2010-2019	2.96 (2.93 to 2.99)	2004-2007	4.33 (3.96 to 4.63)	2010-2019	2.94 (2.91 to 2.97)	2004-2008	3.83 (3.25 to 4.13)	2010-2019	2.42 (2.37 to 2.48)
	4			2019-2021	2.53 (2.36 to 2.75)	2007-2011	2.27 (2.01 to 2.62)	2019-2021	2.28 (2.09 to 2.54)	2008-2019	1.56 (1.46 to 1.70)	2019-2021	1.43 (1.06 to 1.87)
	5					2011-2019	1.63 (1.47 to 1.74)			2019-2021	-0.24 (-1.04 to 0.80)		
	6					2019-2021	-0.26 (-0.76 to 0.47)						
Mozambique	0	1990-1996	0.72 (0.52 to 0.84)	1990-1995	1.92 (1.22 to 2.23)	1990-1994	-0.92 (-1.50 to -0.57)	1990-2001	2.11 (1.83 to 2.60)	1990-1994	-1.23 (-1.79 to -0.93)	1990-1994	-0.72 (-1.23 to -0.45)
	1	1996-2005	1.26 (1.11 to 1.36)	1995-2004	2.46 (2.27 to 2.78)	1994-2000	0.18 (0.02 to 0.66)	2001-2014	2.88 (1.76 to 3.25)	1994-1998	0.30 (-0.04 to 0.77)	1994-1998	0.60 (0.31 to 1.00)
	2	2005-2009	2.09 (1.35 to 2.39)	2004-2016	2.84 (2.74 to 3.13)	2000-2003	-1.05 (-1.40 to -0.53)	2014-2017	2.25 (1.74 to 3.00)	1998-2003	-0.87 (-1.36 to -0.64)	1998-2003	-0.24 (-0.71 to -0.05)
	3	2009-2015	1.69 (1.40 to 1.85)	2016-2021	1.71 (1.34 to 1.99)	2003-2007	1.53 (0.98 to 2.19)	2017-2021	1.42 (0.75 to 1.81)	2003-2007	1.55 (0.79 to 2.12)	2003-2007	1.82 (1.19 to 2.34)
	4	2015-2021	0.95 (0.78 to 1.08)			2007-2012	3.84 (3.58 to 4.23)			2007-2011	3.88 (3.42 to 4.55)	2007-2011	3.69 (3.32 to 4.23)
	5					2012-2021	-0.31 (-0.46 to -0.18)			2011-2014	0.86 (0.03 to 2.17)	2011-2014	1.28 (0.61 to 2.28)
	6									2014-2021	-0.65 (-1.01 to -0.48)	2014-2021	-0.09 (-0.34 to 0.05)
Myanmar	0	1990-2003	2.13 (2.08 to 2.18)	1990-1994	3.55 (3.35 to 3.91)	1990-1993	1.66 (1.31 to 2.06)	1990-1994	3.46 (3.27 to 3.81)	1990-1994	1.25 (0.99 to 1.55)	1990-1994	1.67 (1.47 to 1.89)
	1	2003-2015	1.79 (1.72 to 1.84)	1994-1999	2.86 (2.59 to 3.08)	1993-1997	0.29 (0.02 to 0.60)	1994-1999	2.88 (2.58 to 3.08)	1994-1999	-0.25 (-0.42 to 0.01)	1994-1999	0.41 (0.28 to 0.61)
	2	2015-2021	2.59 (2.46 to 2.75)	1999-2004	2.25 (1.92 to 2.49)	1997-2003	-0.35 (-0.59 to -0.27)	1999-2004	2.39 (1.94 to 2.56)	1999-2004	-0.88 (-1.24 to -0.75)	1999-2004	-0.13 (-0.37 to -0.03)
	3			2004-2015	1.82 (1.77 to 1.86)	2003-2012	-1.71 (-1.76 to -1.65)	2004-2015	1.92 (1.86 to 2.07)	2004-2012	-2.45 (-2.53 to -2.38)	2004-2012	-1.21 (-1.28 to -1.16)
	4			2015-2021	3.13 (3.07 to 3.20)	2012-2015	0.21 (-1.37 to 0.39)	2015-2021	3.11 (3.01 to 3.22)	2012-2015	-0.30 (-2.06 to -0.08)	2012-2015	0.52 (-0.32 to 0.72)
	5					2015-2021	0.67 (0.57 to 0.97)			2015-2021	0.28 (0.15 to 0.64)	2015-2021	1.27 (1.17 to 1.50)
Namibia	0	1990-2006	1.01 (0.98 to 1.04)	1990-2000	1.82 (1.65 to 1.86)	1990-1997	2.00 (1.71 to 2.23)	1990-2000	1.77 (1.63 to 1.81)	1990-1997	1.76 (1.52 to 1.93)	1990-1997	1.76 (1.61 to 1.90)
	1	2006-2010	0.47 (0.16 to 0.67)	2000-2005	1.99 (1.87 to 2.17)	1997-2000	4.26 (1.91 to 4.57)	2000-2005	1.95 (1.84 to 2.13)	1997-2000	4.64 (4.03 to 5.04)	1997-2000	4.22 (3.75 to 4.50)
	2	2010-2015	1.95 (1.81 to 2.16)	2005-2010	1.22 (1.09 to 1.34)	2000-2003	1.96 (1.57 to 4.25)	2005-2010	1.25 (1.14 to 1.35)	2000-2003	1.89 (1.31 to 2.43)	2000-2003	1.90 (1.55 to 2.31)
	3	2015-2021	0.50 (0.38 to 0.60)	2010-2014	3.13 (3.01 to 3.27)	2003-2008	-3.15 (-3.63 to 2.01)	2010-2014	3.18 (3.06 to 3.31)	2003-2008	-3.68 (-4.33 to -3.34)	2003-2008	-2.89 (-3.29 to -2.62)
	4			2014-2017	1.82 (1.59 to 2.13)	2008-2012	-1.73 (-3.17 to -0.87)	2014-2017	1.81 (1.56 to 2.04)	2008-2012	-1.87 (-2.76 to -0.69)	2008-2012	-1.26 (-2.03 to -0.59)
	5			2017-2021	0.97 (0.77 to 1.08)	2012-2016	0.72 (-1.67 to 1.43)	2017-2021	0.75 (0.59 to 0.87)	2012-2021	0.15 (-0.07 to 0.49)	2012-2016	0.99 (0.55 to 1.59)
	6					2016-2021	-0.14 (-1.01 to 0.28)					2016-2021	0.02 (-0.59 to 0.29)
Nauru	0	1990-1994	3.17 (3.06 to 3.27)	1990-1994	4.79 (4.63 to 4.96)	1990-1993	0.79 (0.04 to 1.26)	1990-1994	4.78 (4.63 to 4.93)	1990-1994	1.27 (0.71 to 1.57)	1990-1993	1.67 (1.09 to 2.04)
	1	1994-1999	2.04 (1.95 to 2.11)	1994-1999	2.78 (2.63 to 2.90)	1993-1998	2.72 (2.50 to 3.14)	1994-1999	2.76 (2.64 to 2.87)	1994-1998	3.27 (2.96 to 3.69)	1993-1998	3.01 (2.84 to 3.33)
	2	1999-2005	1.16 (1.11 to 1.21)	1999-2005	1.49 (1.38 to 1.60)	1998-2001	2.04 (0.67 to 2.38)	1999-2005	1.44 (1.30 to 1.54)	1998-2002	1.78 (1.39 to 2.13)	1998-2001	2.06 (1.21 to 2.56)
	3	2005-2010	2.09 (2.03 to 2.15)	2005-2010	2.55 (2.43 to 2.70)	2001-2007	0.64 (0.02 to 0.86)	2005-2010	2.57 (2.44 to 2.80)	2002-2009	0.39 (0.21 to 0.57)	2001-2010	0.77 (0.66 to 0.86)
	4	2010-2014	1.18 (1.08 to 1.27)	2010-2014	1.76 (1.57 to 1.93)	2007-2015	-0.11 (-0.44 to 0.00)	2010-2014	1.67 (1.43 to 1.89)	2009-2015	-0.44 (-0.89 to -0.27)	2010-2014	-0.04 (-0.32 to 0.23)
	5	2014-2019	0.49 (0.41 to 0.57)	2014-2019	0.80 (0.67 to 0.91)	2015-2018	1.13 (0.65 to 1.43)	2014-2019	0.75 (0.54 to 0.88)	2015-2018	0.96 (0.44 to 1.28)	2014-2021	0.48 (0.37 to 0.73)
	6	2019-2021	1.59 (1.35 to 1.76)	2019-2021	2.37 (2.02 to 2.63)	2018-2021	-0.40 (-1.20 to -0.03)	2019-2021	2.29 (1.89 to 2.59)	2018-2021	-0.53 (-1.33 to -0.14)		
Nepal	0	1990-1993	2.57 (2.08 to 3.03)	1990-1994	3.00 (2.57 to 3.37)	1990-1994	1.31 (1.06 to 1.55)	1990-1994	2.99 (2.68 to 3.25)	1990-1994	1.12 (0.90 to 1.34)	1990-1994	1.80 (1.65 to 1.97)
	1	1993-2001	0.99 (0.78 to 1.10)	1994-2001	0.35 (0.12 to 0.51)	1994-2002	-0.37 (-0.60 to -0.23)	1994-2001	0.53 (0.34 to 0.66)	1994-2002	-0.89 (-1.01 to -0.81)	1994-2001	-0.36 (-0.48 to -0.28)
	2	2001-2005	1.88 (1.40 to 2.66)	2001-2005	2.28 (1.81 to 2.77)	2002-2005	0.44 (-0.49 to 1.00)	2001-2005	2.24 (1.76 to 2.79)	2002-2006	0.29 (0.05 to 0.65)	2001-2005	0.81 (0.56 to 1.13)
	3	2005-2010	3.45 (3.22 to 3.81)	2005-2010	4.52 (4.21 to 4.87)	2005-2008	1.60 (0.78 to 2.29)	2005-2010	4.36 (4.06 to 4.72)	2006-2014	2.13 (1.99 to 2.21)	2005-2010	2.91 (2.77 to 3.16)
	4	2010-2021	2.30 (2.22 to 2.37)	2010-2021	2.69 (2.60 to 2.77)	2008-2014	2.46 (2.22 to 2.70)	2010-2021	2.66 (2.58 to 2.74)	2014-2017	3.10 (2.60 to 3.33)	2010-2018	2.56 (2.42 to 2.66)
	5					2014-2017	3.47 (3.01 to 3.69)			2017-2021	1.01 (0.66 to 1.26)	2018-2021	1.65 (1.07 to 1.99)
	6					2017-2021	1.28 (0.97 to 1.50)						
Netherlands	0	1990-1994	2.12 (1.92 to 2.35)	1990-1994	2.60 (2.42 to 2.89)	1990-1992	-6.49 (-8.11 to -3.55)	1990-1994	2.68 (2.44 to 3.05)	1990-1998	-4.37 (-4.80 to -3.97)	1990-1998	-1.95 (-2.34 to -1.67)
	1	1994-2000	1.22 (1.13 to 1.32)	1994-2000	1.88 (1.75 to 2.00)	1992-1998	-3.26 (-3.80 to 5.09)	1994-2000	1.83 (1.67 to 1.95)	1998-2002	3.82 (2.76 to 4.79)	1998-2001	3.62 (1.89 to 4.37)
	2	2000-2005	-0.36 (-0.43 to -0.28)	2000-2005	0.53 (0.32 to 0.86)	1998-2002	4.24 (-7.59 to 6.04)	2000-2005	-0.56 (-0.67 to -0.45)	2002-2012	-6.97 (-7.40 to -6.64)	2001-2011	-3.02 (-3.41 to -2.78)
	3	2005-2019	0.85 (0.81 to 0.87)	2005-2009	1.46 (0.64 to 1.67)	2002-2011	-6.84 (-7.56 to -1.87)	2005-2019	1.58 (1.53 to 1.63)	2012-2021	-2.33 (-2.89 to -1.66)	2011-2021	0.29 (-0.13 to 0.80)
	4	2019-2021	2.56 (2.07 to 2.84)	2009-2019	1.77 (1.70 to 1.90)	2011-2021	-2.14 (-2.95 to -1.20)	2019-2021	3.16 (2.44 to 3.51)				
	5			2019-2021	3.32 (2.73 to 3.64)								
New Zealand	0	1990-1994	-3.09 (-3.31 to -2.86)	1990-1994	-3.83 (-4.08 to -3.56)	1990-1998	3.64 (2.45 to 5.03)	1990-1994	-3.59 (-3.79 to -3.38)	1990-1998	2.13 (0.90 to 3.63)	1990-1998	0.47 (0.09 to 1.12)
	1	1994-1998	-0.38 (-0.74 to -0.21)	1994-1998	-0.53 (-0.95 to -0.34)	1998-2013	-4.14 (-5.66 to -3.59)	1994-1998	-0.42 (-0.77 to -0.23)	1998-2013	-4.67 (-5.62 to -4.14)	1998-2001	-3.01 (-3.76 to -1.32)

Location	Segment	Incidence		Prevalence		Mortality		YLD		YLL		DALY	
		Period	APC (95%CI)	Period	APC (95%CI)	Period	APC (95%CI)	Period	APC (95%CI)	Period	APC (95%CI)	Period	APC (95%CI)
	2	1998-2001	0.79 (0.55 to 0.98)	1998-2001	0.69 (0.43 to 0.93)	2013-2021	-0.80 (-2.45 to 3.41)	1998-2001	0.76 (0.42 to 1.63)	2013-2021	-0.33 (-1.85 to 2.35)	2001-2013	-0.41 (-0.75 to 0.25)
	3	2001-2004	2.56 (2.33 to 2.71)	2001-2005	3.26 (3.06 to 3.43)			2001-2005	3.09 (2.81 to 3.47)			2013-2021	2.46 (1.77 to 3.63)
	4	2004-2011	1.80 (1.74 to 1.84)	2005-2010	2.12 (1.97 to 2.23)			2005-2011	2.05 (1.81 to 2.21)				
	5	2011-2021	2.86 (2.82 to 2.88)	2010-2014	3.20 (2.98 to 3.37)			2011-2021	3.44 (3.37 to 3.52)				
	6			2014-2021	3.70 (3.63 to 3.79)								
Nicaragua	0	1990-1994	2.90 (2.64 to 3.19)	1990-1994	3.40 (3.10 to 3.71)	1990-2009	1.32 (1.04 to 1.75)	1990-1994	3.48 (3.20 to 3.76)	1990-1993	4.66 (1.33 to 10.09)	1990-1996	3.02 (2.47 to 3.93)
	1	1994-2000	0.92 (0.70 to 1.08)	1994-2000	0.97 (0.71 to 1.16)	2009-2021	-0.61 (-1.67 to 0.01)	1994-2000	0.95 (0.69 to 1.14)	1993-2016	0.58 (0.30 to 0.82)	1996-1999	-1.49 (-2.37 to 0.26)
	2	2000-2005	2.07 (1.85 to 2.42)	2000-2013	2.09 (2.03 to 2.17)			2000-2013	2.12 (2.05 to 2.20)	2016-2021	-2.32 (-8.12 to -0.37)	1999-2009	1.72 (1.42 to 2.84)
	3	2005-2014	1.70 (1.45 to 1.80)	2013-2019	1.10 (0.82 to 1.28)			2013-2019	1.06 (0.77 to 1.24)			2009-2021	0.44 (0.04 to 0.71)
	4	2014-2019	0.68 (0.38 to 0.87)	2019-2021	3.90 (2.98 to 4.51)			2019-2021	3.68 (2.81 to 4.26)				
	5	2019-2021	3.64 (3.04 to 4.18)										
Niger	0	1990-1995	1.64 (1.59 to 1.70)	1990-1994	2.39 (2.29 to 2.45)	1990-1998	2.48 (2.09 to 2.61)	1990-1995	2.35 (2.32 to 2.39)	1990-2001	2.45 (2.33 to 2.54)	1990-2001	2.50 (2.41 to 2.56)
	1	1995-2004	2.21 (2.19 to 2.25)	1994-2003	2.69 (2.66 to 2.72)	1998-2001	2.95 (0.12 to 3.15)	1995-1999	2.79 (2.74 to 2.85)	2001-2004	0.07 (-0.26 to 2.68)	2001-2004	1.01 (0.80 to 2.64)
	2	2004-2010	2.03 (1.96 to 2.08)	2003-2009	2.46 (2.42 to 2.49)	2001-2010	0.07 (-0.06 to 2.06)	1999-2004	2.64 (2.58 to 2.69)	2004-2010	-0.52 (-0.83 to -0.07)	2004-2010	0.62 (0.39 to 0.78)
	3	2010-2019	1.53 (1.48 to 1.55)	2009-2013	2.11 (2.06 to 2.19)	2010-2014	2.13 (0.48 to 2.50)	2004-2009	2.47 (2.41 to 2.51)	2010-2014	2.19 (1.55 to 2.57)	2010-2014	2.12 (1.80 to 2.39)
	4	2019-2021	1.87 (1.62 to 2.00)	2013-2018	1.87 (1.78 to 1.92)	2014-2021	0.36 (0.20 to 0.54)	2009-2013	2.14 (2.08 to 2.21)	2014-2021	0.33 (0.15 to 0.49)	2014-2021	1.01 (0.89 to 1.12)
	5			2018-2021	2.18 (2.09 to 2.35)			2013-2018	1.88 (1.80 to 1.92)				
	6							2018-2021	2.07 (2.00 to 2.19)				
Nigeria	0	1990-1993	1.75 (1.36 to 2.36)	1990-2011	1.35 (0.78 to 1.45)	1990-1995	1.32 (1.17 to 1.48)	1990-1994	2.03 (1.70 to 2.68)	1990-1994	1.38 (1.12 to 1.60)	1990-1994	1.59 (1.38 to 1.78)
	1	1993-2001	0.63 (0.47 to 0.72)	2011-2021	1.66 (1.42 to 2.49)	1995-1999	-0.24 (-0.53 to -0.00)	1994-2000	0.77 (0.31 to 0.96)	1994-2004	0.06 (-0.01 to 0.15)	1994-2000	0.14 (-0.10 to 0.24)
	2	2001-2004	2.07 (1.56 to 2.28)			1999-2003	0.67 (0.44 to 1.01)	2000-2005	1.99 (1.72 to 2.52)	2004-2008	-0.72 (-1.07 to -0.43)	2000-2003	0.84 (0.50 to 1.01)
	3	2004-2011	0.93 (0.70 to 1.03)			2003-2008	-0.05 (-0.41 to 0.10)	2005-2010	0.78 (0.33 to 1.04)	2008-2013	0.58 (0.37 to 0.99)	2003-2009	-0.11 (-0.37 to -0.00)
	4	2011-2014	1.94 (1.50 to 2.17)			2008-2012	0.71 (0.45 to 1.03)	2010-2015	2.18 (1.96 to 2.71)	2013-2021	-0.44 (-0.56 to -0.33)	2009-2013	1.05 (0.80 to 1.38)
	5	2014-2018	0.74 (0.38 to 0.95)			2012-2021	-0.24 (-0.33 to -0.16)	2015-2018	0.79 (0.48 to 1.25)			2013-2021	0.15 (0.05 to 0.23)
	6	2018-2021	1.45 (1.14 to 1.98)					2018-2021	2.18 (1.83 to 2.89)				
Niue	0	1990-1995	2.09 (1.96 to 2.14)	1990-1994	2.71 (2.38 to 2.87)	1990-1992	4.83 (4.00 to 5.42)	1990-1993	2.65 (2.41 to 2.85)	1990-1992	5.34 (4.20 to 6.23)	1990-1992	4.59 (3.69 to 5.29)
	1	1995-1999	2.40 (2.17 to 2.50)	1994-2005	3.03 (3.00 to 3.10)	1992-1997	2.58 (2.23 to 2.91)	1993-2004	2.95 (2.93 to 3.07)	1992-1997	2.38 (1.95 to 2.77)	1992-1997	2.55 (2.20 to 2.83)
	2	1999-2002	2.10 (1.99 to 2.41)	2005-2011	2.37 (2.30 to 2.52)	1997-2007	1.68 (1.53 to 1.80)	2004-2008	2.47 (2.39 to 2.86)	1997-2008	1.43 (1.27 to 1.55)	1997-2008	1.80 (1.67 to 1.88)
	3	2002-2009	1.88 (1.82 to 2.08)	2011-2019	2.07 (1.91 to 2.12)	2007-2017	0.76 (0.65 to 1.01)	2008-2014	2.20 (2.13 to 2.30)	2008-2021	0.52 (0.41 to 0.62)	2008-2021	1.01 (0.93 to 1.09)
	4	2009-2013	1.73 (1.65 to 1.89)	2019-2021	2.79 (2.37 to 2.99)	2017-2021	0.10 (-0.66 to 0.49)	2014-2019	1.95 (1.73 to 2.03)				
	5	2013-2019	1.59 (1.50 to 1.64)					2019-2021	2.80 (2.48 to 3.01)				
	6	2019-2021	2.41 (2.23 to 2.54)										
North Macedonia	0	1990-1995	1.97 (1.83 to 2.12)	1990-1992	1.85 (1.62 to 2.11)	1990-1998	4.94 (4.54 to 5.40)	1990-1992	1.84 (1.58 to 2.16)	1990-1998	3.71 (2.96 to 4.26)	1990-1995	2.93 (1.87 to 3.40)
	1	1995-2000	3.56 (3.45 to 3.66)	1992-1995	2.55 (2.43 to 2.97)	1998-2005	1.52 (0.60 to 1.94)	1992-1995	2.57 (2.44 to 3.55)	1998-2004	0.78 (-0.58 to 4.31)	1995-1998	4.27 (1.34 to 4.75)
	2	2000-2015	1.76 (1.73 to 1.79)	1995-2000	3.81 (3.73 to 3.90)	2005-2008	5.18 (3.32 to 5.92)	1995-2000	3.81 (3.72 to 3.93)	2004-2007	3.94 (0.57 to 4.72)	1998-2004	1.38 (0.61 to 2.82)
	3	2015-2019	0.20 (0.00 to 0.35)	2000-2005	1.76 (1.61 to 1.85)	2008-2021	-1.61 (-1.80 to -1.44)	2000-2005	1.79 (1.64 to 1.89)	2007-2012	-0.68 (-1.40 to 3.53)	2004-2007	2.98 (0.58 to 3.41)
	4	2019-2021	1.92 (1.38 to 2.24)	2005-2014	2.01 (1.97 to 2.07)			2005-2014	2.04 (2.00 to 2.12)	2012-2021	-1.86 (-2.89 to -1.52)	2007-2012	0.57 (-0.74 to 0.96)
	5			2014-2019	0.79 (0.69 to 0.86)			2014-2019	0.75 (0.60 to 0.81)			2012-2021	-0.43 (-0.77 to -0.12)
	6			2019-2021	1.61 (1.27 to 1.82)			2019-2021	1.42 (1.08 to 1.63)				
Northern Mariana Islands	0	1990-2000	1.74 (1.72 to 1.77)	1990-1999	2.30 (2.28 to 2.32)	1990-1999	-0.95 (-3.75 to 0.17)	1990-2000	2.28 (2.26 to 2.30)	1990-1999	-1.14 (-1.63 to -0.75)	1990-1999	-0.34 (-0.61 to -0.10)
	1	2000-2004	1.54 (1.43 to 1.63)	1999-2004	2.12 (2.07 to 2.16)	1999-2004	3.82 (0.10 to 7.35)	2000-2004	2.05 (1.92 to 2.13)	1999-2004	3.45 (2.37 to 4.62)	1999-2004	2.97 (2.37 to 3.65)
	2	2004-2011	1.23 (1.18 to 1.27)	2004-2011	1.53 (1.51 to 1.55)	2004-2019	-2.33 (-3.98 to 2.96)	2004-2011	1.55 (1.50 to 1.59)	2004-2017	-2.34 (-2.60 to -2.15)	2004-2017	-1.12 (-1.27 to -0.99)
	3	2011-2015	1.65 (1.58 to 1.75)	2011-2015	1.97 (1.93 to 2.02)	2019-2021	2.96 (-2.07 to 5.27)	2011-2015	1.98 (1.89 to 2.09)	2017-2021	0.60 (-0.39 to 3.18)	2017-2021	1.55 (0.85 to 2.67)
	4	2015-2021	2.34 (2.30 to 2.38)	2015-2021	2.70 (2.67 to 2.73)			2015-2021	2.66 (2.62 to 2.70)				
Norway	0	1990-1994	-0.47 (-0.84 to -0.24)	1990-2005	0.31 (0.26 to 0.34)	1990-2007	1.18 (0.62 to 1.77)	1990-2005	0.32 (0.28 to 0.35)	1990-2007	0.66 (0.11 to 1.26)	1990-2008	0.47 (0.33 to 0.64)
	1	1994-2015	0.22 (0.20 to 0.24)	2005-2010	0.95 (0.77 to 1.30)	2007-2021	-4.35 (-5.33 to -3.54)	2005-2010	0.86 (0.70 to 1.21)	2007-2021	-4.59 (-5.62 to -3.76)	2008-2015	-1.64 (-3.27 to -1.02)
	2	2015-2019	3.45 (3.32 to 3.59)	2010-2015	0.05 (-0.13 to 0.19)			2010-2015	0.10 (-0.07 to 0.23)			2015-2021	1.68 (0.80 to 3.17)
	3	2019-2021	1.13 (0.68 to 1.73)	2015-2019	3.52 (3.36 to 3.72)			2015-2019	3.54 (3.40 to 3.73)				
	4			2019-2021	1.50 (1.04 to 2.14)			2019-2021	1.38 (0.93 to 2.04)				
Oman	0	1990-1993	2.51 (1.60 to 3.78)	1990-1994	3.40 (2.77 to 3.94)	1990-1999	0.50 (-0.01 to 1.21)	1990-1994	3.22 (2.61 to 3.77)	1990-1999	0.63 (-0.11 to 1.13)	1990-1998	1.06 (0.40 to 1.53)
	1	1993-2004	1.18 (0.80 to 1.31)	1994-2001	0.34 (-0.21 to 0.57)	1999-2005	-2.66 (-4.32 to -1.87)	1994-2004	0.63 (0.41 to 0.78)	1999-2004	-2.20 (-3.14 to 1.25)	1998-2004	-1.15 (-1.93 to 1.57)
	2	2004-2021	1.87 (1.80 to 1.95)	2001-2006	1.30 (0.77 to 1.96)	2005-2016	2.03 (1.73 to 2.38)	2004-2010	2.78 (2.44 to 3.60)	2004-2007	-0.76 (-2.25 to 2.06)	2004-2007	0.15 (-1.32 to 2.29)
	3			2006-2009	3.58 (2.86 to 3.97)	2016-2021	-3.61 (-4.71 to -2.58)	2010-2019	2.22 (1.53 to 2.38)	2007-2010	2.43 (-2.23 to 3.01)	2007-2010	2.71 (-0.57 to 3.25)
	4			2009-2019	2.23 (2.05 to 2.33)			2019-2021	5.43 (4.01 to 6.26)	2010-2013	-2.33 (-2.86 to 1.96)	2010-2013	-0.81 (-1.36 to 1.81)
	5			2019-2021	5.92 (4.60 to 6.63)					2013-2016	2.04 (-3.82 to 2.60)	2013-2016	1.90 (-0.84 to 2.46)
	6									2016-2021	-3.99 (-4.50 to -3.52)	2016-2021	-1.05 (-1.68 to -0.67)
Pakistan	0	1990-1995	2.59 (2.26 to 2.83)	1990-1995	3.03 (2.54 to 3.34)	1990-1995	5.64 (5.48 to 5.82)	1990-1996	2.81 (2.50 to 3.00)	1990-1995	6.04 (5.86 to 6.20)	1990-1995	5.16 (5.04 to 5.27)
	1	1995-2000	4.58 (4.33 to 5.02)	1995-2001	4.67 (4.45 to 5.31)	1995-1998	2.93 (2.62 to 4.03)	1996-1999	5.11 (4.52 to 5.42)	1995-1998	2.75 (2.45 to 3.06)	1995-2000	3.10 (3.01 to 3.32)
	2	2000-2010	3.54 (3.44 to 3.62)	2001-2009	3.74 (3.54 to 3.88)	1998-2003	2.44 (0.65 to 2.54)	1999-2010	4.01 (3.88 to 4.08)	1998-2003	2.29 (0.45 to 2.39)	2000-2003	2.62 (1.68 to 2.84)
	3	2010-2018	1.69 (1.60 to 1.79)	2009-2018	2.04 (1.94 to 2.16)	2003-2010	0.72 (0.45 to 0.87)	2010-2018	1.99 (1.86 to 2.13)	2003-2010	0.51 (0.28 to 0.69)	2003-2010	1.55 (1.40 to 1.64)
	4	2018-2021	-0.39 (-0.78 to 0.14)	2018-2021	-0.30 (-0.76 to 0.33)	2010-2021	0.38 (0.29 to 0.44)	2018-2021	-0.33 (-0.88 to 0.28)	2010-2021	0.26 (0.14 to 0.34)	2010-2018	0.82 (0.74 to 0.94)
	5											2018-2021	0.17 (-0.32 to 0.46)

Location	Segment	Incidence		Prevalence		Mortality		YLD		YLL		DALY	
		Period	APC (95%CI)	Period	APC (95%CI)	Period	APC (95%CI)	Period	APC (95%CI)	Period	APC (95%CI)	Period	APC (95%CI)
Palau	0	1990-1993	2.41 (2.31 to 2.52)	1990-1994	2.92 (2.82 to 3.01)	1990-2014	2.09 (2.03 to 2.16)	1990-1993	3.08 (2.93 to 3.25)	1990-2006	1.73 (1.63 to 2.24)	1990-2013	1.83 (1.80 to 1.87)
	1	1993-1998	1.39 (1.35 to 1.46)	1994-2001	1.66 (1.62 to 1.70)	2014-2021	-0.49 (-0.99 to -0.07)	1993-1996	2.03 (1.85 to 2.22)	2006-2013	1.36 (-0.57 to 1.62)	2013-2021	0.20 (-0.01 to 0.40)
	2	1998-2001	1.12 (1.05 to 1.21)	2001-2004	3.15 (3.03 to 3.25)			1996-2000	1.43 (1.32 to 1.51)	2013-2021	-0.49 (-0.93 to -0.16)		
	3	2001-2009	2.12 (2.09 to 2.15)	2004-2009	2.73 (2.64 to 2.80)			2000-2009	2.76 (2.72 to 2.80)				
	4	2009-2012	1.75 (1.64 to 1.97)	2009-2013	2.26 (2.15 to 2.39)			2009-2014	2.16 (2.08 to 2.32)				
	5	2012-2018	1.31 (1.21 to 1.36)	2013-2021	1.95 (1.91 to 1.99)			2014-2021	1.87 (1.80 to 1.93)				
	6	2018-2021	1.48 (1.36 to 1.64)										
Palestine	0	1990-1995	1.33 (1.22 to 1.41)	1990-1996	1.94 (1.86 to 1.99)	1990-2002	0.40 (-0.57 to 0.80)	1990-1996	1.90 (1.82 to 1.96)	1990-2002	0.27 (-0.84 to 0.69)	1990-2002	0.83 (0.46 to 1.07)
	1	1995-2000	1.74 (1.64 to 1.86)	1996-2001	2.41 (2.30 to 2.50)	2002-2011	1.74 (1.31 to 2.90)	1996-2001	2.45 (2.28 to 2.56)	2002-2011	1.49 (1.07 to 2.66)	2002-2011	2.02 (1.73 to 2.66)
	2	2000-2005	2.37 (2.27 to 2.46)	2001-2005	2.81 (2.65 to 2.97)	2011-2021	-1.98 (-2.38 to -1.62)	2001-2005	2.82 (2.64 to 3.03)	2011-2021	-2.08 (-2.50 to -1.71)	2011-2021	-0.34 (-0.60 to -0.09)
	3	2005-2009	3.06 (2.94 to 3.21)	2005-2014	3.19 (3.16 to 3.23)			2005-2014	3.23 (3.19 to 3.27)				
	4	2009-2014	2.64 (2.52 to 2.74)	2014-2019	2.55 (2.49 to 2.64)			2014-2019	2.36 (2.30 to 2.43)				
	5	2014-2018	2.04 (1.89 to 2.22)	2019-2021	1.68 (1.49 to 1.97)			2019-2021	1.32 (1.14 to 1.60)				
	6	2018-2021	1.44 (1.16 to 1.61)										
Panama	0	1990-1994	2.59 (2.18 to 3.37)	1990-1994	3.09 (2.67 to 3.86)	1990-1994	4.71 (2.70 to 8.73)	1990-1994	3.15 (2.67 to 4.06)	1990-1994	4.32 (2.30 to 8.28)	1990-1993	4.80 (1.75 to 9.76)
	1	1994-2000	1.03 (0.49 to 1.26)	1994-2001	1.25 (0.91 to 1.45)	1994-2005	0.72 (-0.00 to 1.32)	1994-2001	1.23 (0.78 to 1.45)	1994-2006	0.14 (-0.43 to 0.75)	1993-2021	0.79 (0.57 to 0.92)
	2	2000-2013	2.00 (1.94 to 2.10)	2001-2013	2.30 (2.21 to 2.44)	2005-2009	-4.01 (-6.97 to -1.85)	2001-2013	2.32 (2.21 to 2.47)	2006-2009	-5.06 (-6.88 to -1.84)		
	3	2013-2019	0.77 (0.45 to 0.95)	2013-2019	1.11 (0.71 to 1.30)	2009-2013	5.25 (2.63 to 8.91)	2013-2019	1.09 (0.60 to 1.30)	2009-2013	5.39 (2.79 to 8.99)		
	4	2019-2021	4.95 (3.54 to 5.85)	2019-2021	5.16 (3.74 to 6.12)	2013-2021	-2.49 (-3.77 to -1.49)	2019-2021	5.00 (3.64 to 5.99)	2013-2021	-2.39 (-3.73 to -1.34)		
Papua New Guinea	0	1990-1995	1.76 (1.52 to 1.89)	1990-1994	2.48 (1.96 to 2.72)	1990-1995	0.17 (-1.00 to 0.64)	1990-1995	2.46 (2.11 to 2.63)	1990-1995	0.06 (-1.28 to 0.64)	1990-1995	0.56 (-0.45 to 0.96)
	1	1995-2000	2.90 (2.77 to 3.21)	1994-2012	2.98 (2.96 to 3.02)	1995-2006	1.03 (0.90 to 1.39)	1995-2000	3.35 (2.81 to 3.72)	1995-2006	0.85 (0.29 to 1.45)	1995-2006	1.35 (1.25 to 1.68)
	2	2000-2005	2.31 (2.05 to 2.43)	2012-2021	2.61 (2.52 to 2.67)	2006-2014	-0.23 (-0.60 to -0.04)	2000-2005	2.83 (2.55 to 3.33)	2006-2014	-0.30 (-0.97 to 0.00)	2006-2014	0.60 (0.11 to 0.76)
	3	2005-2010	2.71 (2.60 to 2.97)			2014-2021	0.73 (0.48 to 1.16)	2005-2010	3.13 (2.81 to 3.42)	2014-2021	0.65 (0.32 to 1.37)	2014-2021	1.23 (1.03 to 1.72)
	4	2010-2017	2.30 (2.20 to 2.39)					2010-2017	2.73 (2.62 to 2.92)				
	5	2017-2021	1.70 (1.38 to 1.86)					2017-2021	2.29 (1.90 to 2.47)				
Paraguay	0	1990-1994	2.98 (2.85 to 3.14)	1990-1995	2.89 (2.78 to 3.10)	1990-1995	5.51 (4.63 to 7.48)	1990-1994	3.01 (2.82 to 3.34)	1990-1994	5.89 (4.57 to 8.61)	1990-1994	4.96 (4.03 to 6.83)
	1	1994-2004	2.51 (2.48 to 2.54)	1995-2004	2.65 (2.57 to 2.68)	1995-2001	2.93 (0.94 to 3.60)	1994-2004	2.69 (2.53 to 2.73)	1994-2001	3.03 (1.11 to 3.58)	1994-2001	2.91 (1.65 to 3.28)
	2	2004-2011	1.64 (1.56 to 1.67)	2004-2014	1.77 (1.74 to 1.82)	2001-2005	6.02 (4.58 to 7.77)	2004-2014	1.76 (1.70 to 1.81)	2001-2005	6.35 (4.76 to 8.04)	2001-2005	5.19 (4.12 to 6.31)
	3	2011-2014	1.97 (1.83 to 2.06)	2014-2019	0.91 (0.78 to 1.65)	2005-2016	1.10 (0.77 to 1.47)	2014-2021	0.96 (0.87 to 1.05)	2005-2016	0.63 (0.31 to 1.08)	2005-2016	0.93 (0.71 to 1.19)
	4	2014-2019	0.56 (0.43 to 0.63)	2019-2021	1.41 (1.06 to 1.61)	2016-2021	-1.99 (-3.61 to -1.02)			2016-2021	-1.83 (-3.64 to -0.83)	2016-2021	-0.99 (-2.06 to -0.36)
	5	2019-2021	1.19 (0.88 to 1.36)										
Peru	0	1990-1993	2.76 (2.36 to 3.15)	1990-1994	2.56 (2.34 to 2.79)	1990-1995	4.35 (2.58 to 6.68)	1990-1992	3.11 (2.70 to 3.50)	1990-1994	5.14 (2.71 to 8.91)	1990-1995	3.65 (2.18 to 5.87)
	1	1993-2001	1.47 (1.36 to 1.54)	1994-2000	0.64 (0.54 to 0.73)	1995-2007	-1.71 (-2.58 to -1.21)	1992-1995	2.00 (1.33 to 2.19)	1994-2007	-1.76 (-2.62 to -1.24)	1995-2001	-1.82 (-4.71 to -0.56)
	2	2001-2005	2.58 (2.37 to 2.88)	2000-2005	2.68 (2.55 to 2.83)	2007-2010	5.64 (1.20 to 7.60)	1995-2000	0.63 (0.50 to 0.75)	2007-2010	6.24 (-1.84 to 8.28)	2001-2021	1.44 (1.19 to 1.75)
	3	2005-2015	1.75 (1.69 to 1.81)	2005-2016	2.09 (2.04 to 2.15)	2010-2015	-2.56 (-6.05 to -0.70)	2000-2005	2.74 (2.58 to 2.89)	2010-2015	-2.80 (-6.40 to 5.50)		
	4	2015-2021	1.32 (1.14 to 1.44)	2016-2021	1.65 (1.39 to 1.80)	2015-2019	7.32 (4.41 to 11.27)	2005-2016	2.09 (2.03 to 2.14)	2015-2019	7.13 (-3.13 to 11.21)		
	5					2019-2021	-5.49 (-11.10 to 0.59)	2016-2021	1.48 (1.30 to 1.63)	2019-2021	-4.75 (-10.85 to 3.73)		
Philippines	0	1990-1995	-0.65 (-1.16 to -0.40)	1990-1995	-1.02 (-1.67 to -0.65)	1990-1992	-2.17 (-3.57 to 0.96)	1990-1995	-0.91 (-1.39 to -0.59)	1990-1999	0.87 (0.21 to 1.12)	1990-1994	-0.11 (-1.35 to 0.49)
	1	1995-2000	1.71 (1.31 to 2.18)	1995-2000	2.58 (2.09 to 3.22)	1992-2021	1.09 (1.00 to 1.26)	1995-2000	2.64 (2.18 to 3.20)	1999-2005	1.90 (1.44 to 3.00)	1994-2005	1.47 (1.33 to 1.73)
	2	2000-2010	0.49 (0.18 to 0.64)	2000-2008	0.25 (-0.27 to 0.50)			2000-2009	0.44 (0.09 to 0.63)	2005-2010	0.09 (-0.96 to 0.70)	2005-2011	0.35 (-0.43 to 0.72)
	3	2010-2015	1.30 (0.57 to 1.62)	2008-2014	1.42 (0.71 to 1.93)			2009-2015	1.66 (0.85 to 2.04)	2010-2021	1.67 (1.44 to 1.98)	2011-2019	2.07 (1.87 to 2.56)
	4	2015-2018	2.48 (1.83 to 2.80)	2014-2018	3.03 (2.48 to 3.72)			2015-2018	3.23 (2.43 to 3.64)			2019-2021	-0.58 (-1.95 to 1.12)
	5	2018-2021	0.24 (-0.39 to 0.62)	2018-2021	1.25 (0.21 to 1.83)			2018-2021	0.89 (-0.05 to 1.38)				
Poland	0	1990-1993	-1.33 (-2.20 to -0.60)	1990-1996	-0.29 (-0.60 to -0.07)	1990-1992	0.35 (-1.89 to 2.34)	1990-1996	-0.31 (-0.57 to -0.10)	1990-1992	-1.47 (-3.83 to 0.49)	1990-1998	-2.18 (-2.91 to -1.86)
	1	1993-1996	-0.07 (-0.38 to 2.00)	1996-2005	1.90 (1.77 to 2.07)	1992-1995	-5.90 (-6.64 to -4.62)	1996-2005	1.98 (1.84 to 2.15)	1992-2002	-5.59 (-6.57 to -5.34)	1998-2002	-0.26 (-1.84 to 1.03)
	2	1996-2005	1.89 (0.51 to 2.19)	2005-2015	0.73 (0.55 to 0.85)	1995-2004	-2.98 (-3.38 to -2.32)	2005-2015	0.82 (0.62 to 0.95)	2002-2005	-0.63 (-5.26 to 2.86)	2002-2021	1.08 (0.95 to 1.41)
	3	2005-2015	0.61 (0.39 to 1.12)	2015-2021	1.82 (1.57 to 2.21)	2004-2008	3.80 (2.44 to 5.85)	2015-2021	1.76 (1.50 to 2.24)	2005-2008	3.82 (-0.37 to 5.17)		
	4	2015-2021	1.72 (1.44 to 2.25)			2008-2021	0.92 (0.50 to 1.19)			2008-2021	0.51 (-0.79 to 1.47)		
Portugal	0	1990-1993	4.11 (4.03 to 4.21)	1990-1994	3.86 (3.79 to 3.92)	1990-1992	4.06 (0.26 to 7.23)	1990-1993	4.15 (4.06 to 4.25)	1990-2003	-0.78 (-1.21 to -0.29)	1990-1992	3.95 (2.35 to 5.32)
	1	1993-1999	3.10 (3.06 to 3.13)	1994-1999	3.28 (3.24 to 3.32)	1992-2000	-1.17 (-4.04 to -0.58)	1993-1998	3.41 (3.35 to 3.46)	2003-2006	-8.32 (-9.68 to -4.64)	1992-1996	-0.36 (-1.23 to 0.24)
	2	1999-2002	2.33 (2.23 to 2.40)	1999-2003	2.68 (2.61 to 2.76)	2000-2003	3.57 (-5.00 to 5.03)	1998-2002	2.81 (2.72 to 2.91)	2006-2021	-3.76 (-4.26 to -2.59)	1996-2003	1.27 (0.97 to 2.15)
	3	2002-2009	1.93 (1.89 to 1.96)	2003-2008	2.27 (2.20 to 2.34)	2003-2006	-8.49 (-9.76 to -4.02)	2002-2008	2.29 (2.23 to 2.34)			2003-2006	-3.49 (-4.14 to -1.95)
	4	2009-2014	1.60 (1.53 to 1.66)	2008-2013	1.91 (1.85 to 1.96)	2006-2021	-3.30 (-3.75 to -2.67)	2008-2014	1.84 (1.80 to 1.89)			2006-2021	-0.26 (-0.42 to -0.02)
	5	2014-2019	0.52 (0.47 to 0.58)	2013-2019	1.30 (1.26 to 1.34)			2014-2019	1.10 (1.05 to 1.15)				
	6	2019-2021	2.61 (2.43 to 2.76)	2019-2021	2.44 (2.28 to 2.58)			2019-2021	2.31 (2.16 to 2.44)				
Puerto Rico	0	1990-1996	1.80 (1.63 to 1.87)	1990-1995	2.19 (2.09 to 2.27)	1990-1995	1.15 (-1.75 to 4.03)	1990-1995	2.22 (2.12 to 2.29)	1990-1995	2.29 (0.91 to 4.32)	1990-1995	2.37 (1.67 to 3.33)
	1	1996-2000	2.26 (2.07 to 2.44)	1995-2000	2.86 (2.79 to 2.96)	1995-2011	-1.61 (-4.86 to 2.60)	1995-1999	2.89 (2.80 to 3.05)	1995-2011	-1.79 (-2.20 to -1.44)	1995-2011	-0.10 (-0.49 to 0.12)
	2	2000-2009	1.31 (1.27 to 1.34)	2000-2003	1.96 (1.85 to 2.35)	2011-2015	-5.32 (-8.10 to 1.61)	1999-2002	2.17 (2.00 to 2.36)	2011-2015	-5.86 (-8.25 to -1.79)	2011-2015	-2.24 (-3.60 to 0.37)
	3	2009-2016	0.66 (0.62 to 0.73)	2003-2009	1.74 (1.65 to 1.78)	2015-2021	-2.15 (-4.71 to 3.07)	2002-2009	1.70 (1.64 to 1.74)	2015-2021	-1.19 (-3.27 to 3.65)	2015-2021	-0.01 (-1.04 to 2.19)
	4	2016-2019	-0.28 (-0.43 to -0.05)	2009-2014	1.22 (1.16 to 1.28)			2009-2014	1.15 (1.07 to 1.22)				
	5	2019-2021	1.40 (1.04 to 1.66)	2014-2019	0.50 (0.43 to 0.56)			2014-2019	0.36 (0.29 to 0.41)				
	6			2019-2021	1.42 (1.16 to 1.59)			2019-2021	1.39 (1.18 to 1.54)				

Location	Segment	Incidence		Prevalence		Mortality		YLD		YLL		DALY	
		Period	APC (95%CI)	Period	APC (95%CI)	Period	APC (95%CI)	Period	APC (95%CI)	Period	APC (95%CI)	Period	APC (95%CI)
Qatar	0	1990-1995	4.11 (3.54 to 4.33)	1990-1995	4.84 (4.47 to 5.05)	1990-2000	2.57 (1.09 to 3.70)	1990-1995	4.83 (4.51 to 4.96)	1990-2000	2.71 (1.28 to 3.73)	1990-1996	2.71 (-0.50 to 4.05)
	1	1995-1999	4.72 (4.51 to 4.97)	1995-1999	5.30 (2.16 to 5.55)	2000-2003	12.51 (3.26 to 15.02)	1995-1999	5.32 (5.14 to 5.55)	2000-2003	10.91 (3.41 to 13.05)	1996-2006	5.08 (4.34 to 6.77)
	2	1999-2010	1.13 (1.08 to 1.17)	1999-2002	1.99 (1.43 to 2.16)	2003-2007	4.09 (-5.97 to 7.65)	1999-2002	1.86 (1.62 to 2.03)	2003-2007	3.29 (-4.84 to 6.61)	2006-2017	-3.41 (-4.74 to -2.79)
	3	2010-2021	0.60 (0.56 to 0.65)	2002-2009	1.44 (1.29 to 1.56)	2007-2017	-7.04 (-9.11 to -6.19)	2002-2010	1.37 (1.27 to 1.45)	2007-2017	-6.53 (-8.79 to -5.88)	2017-2021	-0.15 (-2.59 to 4.24)
	4			2009-2021	1.27 (1.18 to 1.31)	2017-2021	0.49 (-3.01 to 7.92)	2010-2021	1.17 (1.08 to 1.21)	2017-2021	-1.65 (-4.77 to 4.57)		
Republic of Korea	0	1990-1998	1.64 (1.48 to 1.74)	1990-2000	1.34 (1.23 to 1.45)	1990-1997	1.97 (1.52 to 2.24)	1990-2000	1.43 (1.34 to 1.53)	1990-1997	0.79 (0.42 to 1.01)	1990-1996	0.93 (0.76 to 1.06)
	1	1998-2001	2.60 (2.27 to 2.80)	2000-2005	6.72 (6.51 to 6.92)	1997-2000	4.79 (3.47 to 5.40)	2000-2005	6.23 (6.01 to 6.43)	1997-2000	3.13 (2.23 to 3.64)	1996-2002	2.03 (1.88 to 2.23)
	2	2001-2004	5.51 (5.17 to 5.78)	2005-2009	3.06 (2.77 to 3.94)	2000-2004	-1.18 (-1.82 to -0.61)	2005-2010	2.84 (2.63 to 3.47)	2000-2004	-2.61 (-3.04 to -2.18)	2002-2005	0.68 (0.36 to 1.24)
	3	2004-2008	2.98 (2.73 to 3.24)	2009-2015	2.23 (1.72 to 2.44)	2004-2009	-6.56 (-7.31 to -6.03)	2010-2015	2.07 (1.51 to 2.34)	2004-2014	-7.12 (-7.43 to -6.97)	2005-2009	-1.54 (-1.90 to -1.22)
	4	2008-2016	1.95 (1.84 to 2.05)	2015-2021	4.46 (4.21 to 4.78)	2009-2019	-5.61 (-5.87 to -5.07)	2015-2021	4.51 (4.28 to 4.80)	2014-2019	-6.09 (-6.72 to -5.20)	2009-2015	-0.58 (-0.88 to -0.29)
	5	2016-2021	3.82 (3.62 to 4.00)			2019-2021	0.91 (-1.31 to 2.15)			2019-2021	1.22 (-0.71 to 2.63)	2015-2019	2.27 (-0.09 to 2.51)
	6											2019-2021	4.06 (3.01 to 4.71)
Republic of Moldova	0	1990-1997	2.16 (1.98 to 2.28)	1990-1995	2.38 (1.92 to 2.59)	1990-1994	5.91 (2.45 to 12.88)	1990-1995	2.36 (2.11 to 2.49)	1990-1994	6.35 (2.61 to 13.45)	1990-1994	3.88 (2.27 to 7.14)
	1	1997-2004	2.91 (2.82 to 3.04)	1995-2000	2.97 (2.69 to 3.63)	1994-2006	-3.27 (-4.09 to -2.02)	1995-2000	3.03 (2.77 to 3.23)	1994-2006	-3.25 (-4.12 to -2.17)	1994-2006	0.54 (-0.07 to 1.42)
	2	2004-2015	1.12 (1.07 to 1.16)	2000-2004	3.73 (1.12 to 3.96)	2006-2011	-7.48 (-12.79 to -5.08)	2000-2004	3.58 (3.43 to 3.77)	2006-2011	-8.60 (-13.98 to -6.26)	2006-2010	-1.83 (-4.12 to 4.93)
	3	2015-2018	3.02 (2.68 to 3.20)	2004-2014	1.12 (1.03 to 1.85)	2011-2016	15.14 (11.83 to 22.36)	2004-2015	1.16 (1.12 to 1.20)	2011-2016	14.87 (11.75 to 21.75)	2010-2021	3.31 (1.57 to 4.64)
	4	2018-2021	1.33 (0.93 to 1.54)	2014-2021	2.20 (2.06 to 2.39)	2016-2021	1.13 (-3.17 to 4.16)	2015-2018	2.77 (2.54 to 2.92)	2016-2021	0.92 (-3.30 to 3.89)		
	5							2018-2021	1.67 (1.34 to 1.85)				
Romania	0	1990-1995	1.07 (0.82 to 1.29)	1990-1995	1.36 (1.09 to 1.55)	1990-1992	3.17 (-0.13 to 6.08)	1990-1995	1.37 (1.08 to 1.58)	1990-1992	1.43 (-1.48 to 3.75)	1990-1992	1.66 (0.87 to 2.45)
	1	1995-2000	2.94 (2.69 to 3.15)	1995-2000	3.08 (2.92 to 3.28)	1992-1996	-5.81 (-7.40 to -4.68)	1995-2000	3.16 (2.98 to 3.48)	1992-2000	-4.43 (-5.27 to -4.03)	1992-1995	-1.81 (-2.16 to -1.19)
	2	2000-2010	0.93 (0.83 to 1.01)	2000-2009	0.93 (0.82 to 1.01)	1996-2001	-2.63 (-3.79 to 0.33)	2000-2006	0.93 (0.59 to 1.09)	2000-2010	0.30 (-0.08 to 0.91)	1995-2013	0.72 (0.65 to 0.79)
	3	2010-2016	1.77 (1.58 to 2.11)	2009-2017	1.66 (1.55 to 1.84)	2001-2010	1.19 (0.73 to 2.88)	2006-2018	1.62 (1.57 to 1.71)	2010-2013	-4.51 (-5.63 to -2.36)	2013-2018	1.78 (1.35 to 2.58)
	4	2016-2021	0.50 (0.26 to 0.72)	2017-2021	0.53 (0.21 to 0.80)	2010-2013	-3.92 (-5.31 to -1.60)	2018-2021	-0.21 (-0.58 to 0.27)	2013-2016	4.55 (2.37 to 5.95)	2018-2021	-0.22 (-1.43 to 0.49)
	5					2013-2016	4.94 (2.59 to 6.59)			2016-2021	0.21 (-1.95 to 1.13)		
	6					2016-2021	0.14 (-2.20 to 1.05)						
Russian Federation	0	1990-1999	2.77 (2.73 to 2.81)	1990-1993	3.24 (3.02 to 3.53)	1990-1994	13.02 (10.68 to 15.35)	1990-1996	3.03 (2.92 to 3.16)	1990-1994	13.64 (11.27 to 16.30)	1990-1994	7.36 (6.49 to 8.26)
	1	1999-2010	2.31 (2.28 to 2.34)	1993-1997	2.76 (2.35 to 2.91)	1994-2001	-4.71 (-6.89 to -3.82)	1996-2010	2.39 (2.36 to 2.42)	1994-2000	-4.82 (-7.02 to -3.66)	1994-2001	-0.56 (-1.53 to -0.12)
	2	2010-2015	1.55 (1.45 to 1.60)	1997-2010	2.33 (2.24 to 2.36)	2001-2011	-2.15 (-2.77 to -0.54)	2010-2016	1.76 (1.53 to 1.88)	2000-2011	-2.94 (-3.43 to 0.02)	2001-2012	1.03 (0.75 to 1.51)
	3	2015-2018	2.40 (2.21 to 2.51)	2010-2017	1.71 (1.58 to 1.79)	2011-2016	30.68 (28.01 to 32.94)	2016-2021	2.11 (1.98 to 2.37)	2011-2016	26.53 (23.45 to 28.54)	2012-2016	12.13 (11.35 to 12.91)
	4	2018-2021	1.71 (1.43 to 1.84)	2017-2021	2.23 (2.08 to 2.46)	2016-2021	4.57 (0.87 to 8.50)			2016-2021	4.09 (-0.50 to 8.01)	2016-2021	2.71 (2.10 to 3.26)
Rwanda	0	1990-2000	0.00 (-0.02 to 0.03)	1990-2000	1.15 (1.11 to 1.20)	1990-1994	2.84 (2.34 to 3.36)	1990-2000	1.04 (1.00 to 1.08)	1990-1994	3.09 (2.54 to 3.69)	1990-1994	2.86 (2.52 to 3.20)
	1	2000-2005	-0.79 (-0.86 to -0.72)	2000-2005	-0.62 (-0.72 to -0.52)	1994-1998	0.00 (-0.39 to 0.41)	2000-2006	-0.34 (-0.41 to -0.26)	1994-1998	-0.49 (-0.97 to -0.02)	1994-1998	-0.28 (-0.54 to 0.01)
	2	2005-2010	0.17 (0.05 to 0.26)	2005-2010	0.83 (0.63 to 0.95)	1998-2005	-5.44 (-5.68 to -5.26)	2006-2011	1.25 (1.03 to 1.35)	1998-2005	-6.23 (-6.58 to -6.01)	1998-2004	-5.54 (-5.78 to -5.38)
	3	2010-2015	0.73 (0.63 to 0.93)	2010-2014	1.73 (1.56 to 2.00)	2005-2009	-3.07 (-3.90 to -2.27)	2011-2014	1.91 (1.62 to 2.07)	2005-2009	-3.68 (-4.96 to -2.81)	2004-2007	-3.99 (-4.83 to -3.10)
	4	2015-2019	0.23 (0.05 to 0.35)	2014-2019	1.27 (1.04 to 1.37)	2009-2015	-0.47 (-0.96 to -0.07)	2014-2019	1.11 (0.89 to 1.22)	2009-2014	-0.93 (-2.12 to -0.31)	2007-2011	-1.68 (-2.17 to -1.20)
	5	2019-2021	1.56 (1.25 to 1.79)	2019-2021	2.88 (2.47 to 3.17)	2015-2021	0.92 (0.60 to 1.47)	2019-2021	2.59 (2.17 to 2.90)	2014-2021	0.39 (0.10 to 1.12)	2011-2019	0.23 (-0.02 to 0.36)
	6											2019-2021	1.69 (0.75 to 2.27)
Saint Kitts and Nevis	0	1990-1994	1.29 (1.19 to 1.41)	1990-1994	1.92 (1.76 to 2.06)	1990-1992	-16.44 (-20.46 to -11.11)	1990-1994	1.92 (1.71 to 2.10)	1990-1992	-14.04 (-16.83 to -10.58)	1990-1992	-9.46 (-11.18 to -7.67)
	1	1994-2000	0.30 (0.17 to 0.41)	1994-2001	0.92 (0.76 to 1.03)	1992-1996	8.07 (5.22 to 12.33)	1994-2000	0.85 (0.60 to 0.96)	1992-1996	5.51 (3.52 to 7.95)	1992-1996	4.13 (3.06 to 5.28)
	2	2000-2010	0.89 (0.29 to 0.94)	2001-2012	1.07 (0.90 to 1.16)	1996-2003	-4.85 (-7.62 to -3.61)	2000-2014	1.12 (1.08 to 1.17)	1996-2002	-5.95 (-7.58 to -4.77)	1996-2002	-3.67 (-4.56 to -2.93)
	3	2010-2015	1.07 (0.90 to 1.22)	2012-2015	1.43 (1.02 to 1.54)	2003-2007	4.95 (1.77 to 8.89)	2014-2018	2.05 (1.86 to 2.35)	2002-2009	1.61 (0.49 to 3.32)	2002-2008	1.75 (0.87 to 3.22)
	4	2015-2018	1.65 (1.38 to 1.80)	2015-2018	2.16 (1.90 to 2.32)	2007-2021	-2.88 (-3.65 to -2.36)	2018-2021	0.92 (0.62 to 1.20)	2009-2016	-4.55 (-7.08 to -3.51)	2008-2016	-1.71 (-3.28 to -1.16)
	5	2018-2021	0.71 (0.38 to 0.89)	2018-2021	0.98 (0.71 to 1.15)					2016-2021	-0.25 (-2.44 to 5.30)	2016-2021	0.62 (-0.61 to 3.59)
Saint Lucia	0	1990-1992	1.83 (1.66 to 2.01)	1990-1992	2.74 (2.57 to 2.92)	1990-1995	1.66 (0.41 to 3.32)	1990-1994	2.22 (2.13 to 2.34)	1990-1992	5.08 (1.39 to 7.89)	1990-1992	4.35 (2.46 to 6.11)
	1	1992-1995	1.10 (1.01 to 1.19)	1992-1995	1.57 (1.48 to 1.66)	1995-1999	-6.96 (-9.24 to -4.83)	1994-2004	0.41 (0.36 to 0.45)	1992-1995	-0.36 (-7.94 to 0.81)	1992-1995	0.21 (-4.20 to 0.99)
	2	1995-2003	-0.10 (-0.13 to -0.07)	1995-2000	0.37 (0.27 to 0.42)	1999-2004	-0.42 (-2.21 to 2.39)	2004-2012	0.99 (0.92 to 1.09)	1995-1999	-7.11 (-9.02 to -0.14)	1995-1999	-4.74 (-6.00 to -0.57)
	3	2003-2011	0.51 (0.47 to 0.57)	2000-2005	0.56 (0.50 to 0.73)	2004-2008	-9.12 (-11.52 to -6.31)	2012-2021	0.51 (0.45 to 0.56)	1999-2004	-0.96 (-8.74 to 1.24)	1999-2004	-0.43 (-4.19 to 0.98)
	4	2011-2021	0.31 (0.27 to 0.34)	2005-2010	1.14 (1.07 to 1.24)	2008-2013	-4.25 (-6.73 to -0.23)			2004-2008	-8.04 (-9.96 to -3.43)	2004-2008	-4.44 (-5.76 to -1.45)
	5			2010-2014	0.76 (0.64 to 0.93)	2013-2021	1.53 (0.07 to 4.79)			2008-2013	-4.04 (-5.50 to 2.72)	2008-2015	-1.13 (-2.32 to 0.03)
	6			2014-2021	0.55 (0.46 to 0.59)					2013-2021	0.82 (-2.95 to 4.29)	2015-2021	1.26 (0.36 to 3.36)
Saint Vincent and the Grenadines	0	1990-1993	1.49 (1.43 to 1.55)	1990-1994	2.13 (2.07 to 2.19)	1990-1994	3.32 (2.02 to 5.34)	1990-1994	2.12 (2.04 to 2.21)	1990-1994	3.83 (2.65 to 5.46)	1990-1994	3.37 (2.69 to 4.24)
	1	1993-1999	0.89 (0.86 to 0.92)	1994-1999	1.53 (1.47 to 1.58)	1994-1997	-6.89 (-8.09 to -4.75)	1994-1999	1.46 (1.39 to 1.54)	1994-1997	-6.09 (-7.16 to -4.07)	1994-1997	-4.12 (-4.85 to -2.60)
	2	1999-2006	0.66 (0.59 to 0.69)	1999-2006	1.14 (1.07 to 1.17)	1997-2004	0.14 (-0.46 to 1.12)	1999-2006	1.08 (0.95 to 1.12)	1997-2004	-0.72 (-1.24 to 0.22)	1997-2004	-0.12 (-0.50 to 0.69)
	3	2006-2010	0.77 (0.70 to 0.84)	2006-2010	1.30 (1.20 to 1.38)	2004-2008	-8.89 (-10.16 to -7.46)	2006-2010	1.29 (1.19 to 1.40)	2004-2008	-8.37 (-9.43 to -7.14)	2004-2008	-5.58 (-6.71 to -4.54)
	4	2010-2015	0.43 (0.38 to 0.48)	2010-2015	0.78 (0.71 to 0.84)	2008-2012	8.74 (7.62 to 9.90)	2010-2015	0.76 (0.67 to 0.82)	2008-2012	7.23 (6.29 to 8.17)	2008-2011	7.09 (5.32 to 8.35)
	5	2015-2018	1.32 (1.25 to 1.39)	2015-2018	1.77 (1.68 to 1.85)	2012-2016	-10.82 (-11.91 to -9.76)	2015-2018	1.74 (1.61 to 1.83)	2012-2016	-9.04 (-10.07 to -8.07)	2011-2016	-3.86 (-5.31 to -3.05)
	6	2018-2021	0.64 (0.55 to 0.72)	2018-2021	0.90 (0.80 to 0.99)	2016-2021	2.99 (1.57 to 4.31)	2018-2021	0.79 (0.65 to 0.89)	2016-2021	1.88 (0.53 to 3.12)	2016-2021	1.20 (0.05 to 3.16)
Samoa	0	1990-1994	2.41 (2.33 to 2.52)	1990-1994	3.22 (3.11 to 3.33)	1990-2001	2.32 (2.30 to 2.35)	1990-1994	3.14 (3.04 to 3.28)	1990-2000	2.40 (2.37 to 2.44)	1990-1999	2.55 (2.53 to 2.58)
	1	1994-2006	2.25 (2.22 to 2.27)	1994-2000	2.72 (2.66 to 2.78)	2001-2004	1.34 (1.24 to 1.43)	1994-2001	2.75 (2.66 to 2.80)	2000-2003	1.66 (1.51 to 1.87)	1999-2003	2.21 (2.10 to 2.29)
	2	2006-2009	2.48 (2.37 to 2.54)	2000-2004	3.52 (3.43 to 3.62)	2004-2007	0.41 (0.31 to 0.53)	2001-2004	3.40 (2.78 to 3.49)	2003-2007	0.61 (0.41 to 0.71)	2003-2006	1.27 (1.19 to 1.38)
	3	2009-2012	1.59 (1.52 to 1.68)	2004-2007	3.02 (2.89 to 3.25)	2007-2011	1.31 (1.21 to 1.42)	2004-2007	2.91 (2.63 to 3.32)	2007-2011	1.51 (1.28 to 1.74)	2006-2011	1.72 (1.64 to 1.81)
	4	2012-2015	1.31 (1.25 to 1.40)	2007-2010	2.53 (2.14 to 2.64)	2011-2016	2.07 (2.00 to 2.15)	2007-2010	2.52 (1.53 to 2.64)	2011-2016	2.22 (2.13 to 2.37)	2011-2016	2.02 (1.96 to 2.11)

Location	Segment	Incidence		Prevalence		Mortality		YLD		YLL		DALY	
		Period	APC (95%CI)	Period	APC (95%CI)	Period	APC (95%CI)	Period	APC (95%CI)	Period	APC (95%CI)	Period	APC (95%CI)
	5	2015-2021	1.65 (1.61 to 1.71)	2010-2015	1.59 (1.47 to 1.67)	2016-2019	0.57 (0.44 to 0.70)	2010-2015	1.59 (1.46 to 1.85)	2016-2019	0.50 (0.34 to 0.67)	2016-2019	0.96 (0.87 to 1.06)
	6			2015-2021	1.90 (1.84 to 1.99)	2019-2021	-0.42 (-0.68 to -0.19)	2015-2021	1.89 (1.81 to 2.01)	2019-2021	-0.47 (-0.81 to -0.18)	2019-2021	0.35 (0.18 to 0.51)
San Marino	0	1990-1995	2.03 (1.95 to 2.11)	1990-1995	2.29 (2.22 to 2.35)	1990-2002	-1.78 (-2.19 to -1.31)	1990-1993	2.15 (2.02 to 2.25)	1990-2012	-2.10 (-2.25 to -1.96)	1990-2006	0.70 (0.62 to 0.74)
	1	1995-2000	2.51 (2.44 to 2.63)	1995-2000	2.74 (2.67 to 2.83)	2002-2005	-6.02 (-7.13 to -1.92)	1993-1996	2.50 (2.35 to 2.71)	2012-2019	0.71 (-0.03 to 2.02)	2006-2013	1.31 (1.02 to 1.67)
	2	2000-2005	2.09 (1.94 to 2.16)	2000-2010	2.39 (2.35 to 2.41)	2005-2012	-1.42 (-3.66 to -0.28)	1996-2000	2.75 (2.34 to 2.85)	2019-2021	-17.59 (-21.47 to -11.49)	2013-2019	2.49 (2.27 to 2.94)
	3	2005-2015	2.30 (2.28 to 2.33)	2010-2015	2.55 (2.48 to 2.66)	2012-2019	1.05 (0.25 to 3.03)	2000-2006	2.34 (2.24 to 2.46)			2019-2021	-1.97 (-2.85 to -0.56)
	4	2015-2019	2.89 (2.78 to 3.03)	2015-2019	3.17 (3.10 to 3.25)	2019-2021	-16.98 (-20.96 to -10.90)	2006-2014	2.47 (2.44 to 2.52)				
	5	2019-2021	2.21 (2.03 to 2.46)	2019-2021	2.11 (1.95 to 2.26)			2014-2019	3.07 (3.03 to 3.11)				
	6							2019-2021	2.12 (2.00 to 2.26)				
Sao Tome and Principe	0	1990-1997	1.94 (1.86 to 2.00)	1990-2001	2.56 (2.54 to 2.58)	1990-1998	3.36 (2.61 to 3.88)	1990-1996	2.43 (2.34 to 2.47)	1990-1999	3.65 (3.44 to 3.89)	1990-1999	3.02 (2.91 to 3.15)
	1	1997-2008	2.27 (2.24 to 2.31)	2001-2010	2.80 (2.78 to 2.83)	1998-2003	1.33 (0.62 to 4.06)	1996-2001	2.53 (2.48 to 2.67)	1999-2004	0.86 (0.22 to 1.64)	1999-2004	1.79 (1.49 to 2.23)
	2	2008-2019	1.96 (1.92 to 1.99)	2010-2019	2.39 (2.37 to 2.41)	2003-2010	0.39 (-0.89 to 1.10)	2001-2010	2.71 (2.69 to 2.74)	2004-2021	-0.50 (-0.60 to -0.41)	2004-2021	1.26 (1.19 to 1.30)
	3	2019-2021	2.86 (2.50 to 3.09)	2019-2021	2.95 (2.75 to 3.06)	2010-2021	-0.38 (-1.12 to 0.09)	2010-2015	2.36 (2.33 to 2.46)				
	4							2015-2018	2.18 (2.13 to 2.26)				
	5							2018-2021	2.78 (2.71 to 2.86)				
Saudi Arabia	0	1990-1995	2.70 (2.64 to 2.74)	1990-1995	3.36 (3.27 to 3.46)	1990-2000	1.61 (1.39 to 1.86)	1990-1996	2.27 (1.99 to 2.46)	1990-2000	1.52 (1.36 to 1.71)	1990-2000	1.93 (1.84 to 2.03)
	1	1995-2005	2.39 (2.36 to 2.41)	1995-2006	2.99 (2.89 to 3.02)	2000-2003	-3.78 (-4.32 to -2.03)	1996-2009	3.33 (3.26 to 3.40)	2000-2003	-3.33 (-3.73 to -2.89)	2000-2003	-0.62 (-0.90 to -0.06)
	2	2005-2009	2.58 (2.50 to 2.67)	2006-2009	3.20 (3.02 to 3.28)	2003-2007	1.86 (1.14 to 2.63)	2009-2016	1.11 (1.00 to 3.24)	2003-2008	1.55 (1.23 to 1.90)	2003-2009	2.14 (1.98 to 2.39)
	3	2009-2012	1.56 (1.49 to 1.67)	2009-2012	2.08 (1.92 to 2.27)	2007-2012	-0.38 (-1.34 to 0.12)	2016-2019	4.47 (1.18 to 4.76)	2008-2012	-0.88 (-1.53 to -0.39)	2009-2012	-0.24 (-0.51 to 0.43)
	4	2012-2016	1.35 (1.25 to 1.40)	2012-2016	1.20 (1.07 to 1.29)	2012-2015	1.42 (0.34 to 2.02)	2019-2021	2.92 (2.29 to 3.92)	2012-2015	1.54 (0.69 to 2.04)	2012-2021	1.26 (1.14 to 1.44)
	5	2016-2019	1.86 (1.77 to 1.94)	2016-2019	2.28 (1.74 to 2.40)	2015-2021	-0.68 (-1.53 to -0.32)			2015-2021	-0.77 (-1.31 to -0.48)		
	6	2019-2021	3.01 (2.88 to 3.13)	2019-2021	3.28 (2.98 to 3.48)								
Senegal	0	1990-1995	2.09 (1.88 to 2.28)	1990-1995	2.70 (2.36 to 2.99)	1990-1999	2.35 (1.54 to 2.67)	1990-1995	2.64 (2.39 to 2.88)	1990-1999	2.00 (0.52 to 2.61)	1990-1999	2.46 (1.73 to 2.74)
	1	1995-2000	3.95 (3.81 to 4.12)	1995-2000	4.20 (3.97 to 4.68)	1999-2002	4.91 (3.22 to 5.61)	1995-2000	4.34 (4.12 to 4.71)	1999-2002	5.25 (1.14 to 6.20)	1999-2002	4.78 (3.31 to 5.39)
	2	2000-2012	2.20 (2.14 to 2.24)	2000-2011	2.48 (2.40 to 2.57)	2002-2013	1.13 (0.84 to 1.46)	2000-2011	2.51 (2.44 to 2.60)	2002-2014	0.93 (0.57 to 2.17)	2002-2014	1.41 (1.20 to 1.66)
	3	2012-2021	1.34 (1.25 to 1.43)	2011-2019	1.81 (1.63 to 1.98)	2013-2021	0.04 (-0.90 to 0.42)	2011-2019	1.77 (1.63 to 1.93)	2014-2021	-0.42 (-3.03 to 0.36)	2014-2021	0.24 (-0.63 to 0.63)
	4			2019-2021	-0.09 (-0.51 to 0.67)			2019-2021	0.11 (-0.34 to 0.84)				
Serbia	0	1990-1995	1.72 (1.62 to 1.81)	1990-1995	2.05 (1.81 to 2.20)	1990-1996	4.09 (2.96 to 5.25)	1990-1995	2.05 (1.84 to 2.18)	1990-1996	4.15 (2.84 to 5.67)	1990-1997	3.01 (2.58 to 3.61)
	1	1995-1999	2.88 (2.79 to 2.99)	1995-1999	2.87 (2.68 to 3.13)	1996-2002	-2.95 (-4.73 to -2.03)	1995-1999	2.88 (2.71 to 3.12)	1996-2002	-2.75 (-4.75 to 1.44)	1997-2000	-1.40 (-1.95 to 0.11)
	2	1999-2006	0.99 (0.92 to 1.04)	1999-2006	1.10 (0.95 to 1.17)	2002-2008	0.38 (-0.48 to 2.43)	1999-2006	1.10 (0.98 to 1.17)	2002-2008	0.26 (-2.76 to 2.50)	2000-2021	0.28 (0.17 to 0.55)
	3	2006-2009	1.89 (1.65 to 2.02)	2006-2009	1.79 (1.50 to 1.93)	2008-2021	-1.00 (-1.75 to -0.75)	2006-2009	1.93 (1.65 to 2.07)	2008-2013	-2.33 (-4.06 to 0.16)		
	4	2009-2016	0.97 (0.83 to 1.03)	2009-2021	1.27 (1.22 to 1.31)			2009-2021	1.27 (1.22 to 1.30)	2013-2021	-0.62 (-1.33 to 1.50)		
	5	2016-2021	1.25 (1.14 to 1.52)										
Seychelles	0	1990-1996	4.94 (4.79 to 5.14)	1990-1996	5.71 (5.53 to 5.92)	1990-2018	2.28 (2.13 to 2.45)	1990-1994	5.91 (5.72 to 6.25)	1990-1995	-0.04 (-3.77 to 1.44)	1990-1994	2.67 (1.56 to 3.15)
	1	1996-2004	4.35 (4.19 to 4.44)	1996-2004	4.71 (4.58 to 4.83)	2018-2021	-5.03 (-10.47 to -1.64)	1994-1998	5.21 (4.83 to 5.49)	1995-2007	2.85 (2.54 to 4.67)	1994-2007	3.76 (3.67 to 3.94)
	2	2004-2015	3.16 (3.11 to 3.21)	2004-2015	3.51 (3.46 to 3.58)			1998-2004	4.67 (3.82 to 4.76)	2007-2011	-0.17 (-1.94 to 1.31)	2007-2010	1.24 (0.77 to 2.41)
	3	2015-2018	1.55 (1.39 to 1.80)	2015-2018	1.91 (1.72 to 2.38)			2004-2015	3.50 (3.45 to 3.55)	2011-2018	2.99 (2.41 to 5.12)	2010-2015	3.33 (2.89 to 4.26)
	4	2018-2021	2.40 (2.19 to 2.81)	2018-2021	2.82 (2.47 to 3.30)			2015-2018	1.84 (1.67 to 2.12)	2018-2021	-5.10 (-8.57 to -2.73)	2015-2019	1.85 (1.45 to 2.65)
	5							2018-2021	2.70 (2.46 to 3.11)			2019-2021	-1.07 (-2.31 to 0.17)
Sierra Leone	0	1990-1994	2.05 (1.74 to 2.24)	1990-1995	2.95 (2.82 to 3.03)	1990-1994	1.45 (1.22 to 1.68)	1990-1994	2.81 (2.61 to 2.93)	1990-1994	1.39 (0.98 to 1.69)	1990-1994	1.85 (1.62 to 2.03)
	1	1994-2010	2.68 (2.65 to 2.71)	1995-2000	3.33 (3.24 to 3.48)	1994-2003	4.42 (4.33 to 4.53)	1994-2009	3.21 (3.18 to 3.24)	1994-1999	4.38 (3.81 to 4.61)	1994-1999	4.00 (3.65 to 4.16)
	2	2010-2021	1.64 (1.60 to 1.68)	2000-2009	3.16 (3.06 to 3.20)	2003-2006	2.51 (2.06 to 3.44)	2009-2013	2.44 (2.33 to 3.23)	1999-2003	5.06 (4.66 to 5.42)	1999-2003	4.47 (3.50 to 4.73)
	3			2009-2013	2.48 (2.29 to 2.90)	2006-2011	1.11 (0.74 to 1.33)	2013-2018	1.86 (1.67 to 2.48)	2003-2006	2.71 (1.59 to 3.18)	2003-2006	2.90 (1.63 to 3.21)
	4			2013-2018	1.90 (1.70 to 2.00)	2011-2021	-0.32 (-0.42 to -0.22)	2018-2021	2.15 (1.95 to 2.39)	2006-2012	0.92 (0.60 to 1.10)	2006-2012	1.53 (1.28 to 1.64)
	5			2018-2021	2.21 (2.06 to 2.44)					2012-2021	-0.46 (-0.60 to -0.34)	2012-2021	0.40 (0.32 to 0.48)
Singapore	0	1990-1994	1.61 (1.48 to 1.74)	1990-1994	1.83 (1.63 to 2.06)	1990-1995	-7.52 (-11.10 to -5.37)	1990-1993	1.87 (1.66 to 2.21)	1990-1996	-6.40 (-9.80 to -4.48)	1990-1992	-5.42 (-6.83 to -2.92)
	1	1994-2000	0.41 (0.34 to 0.49)	1994-1999	0.86 (0.69 to 0.99)	1995-2007	-0.16 (-0.93 to 0.86)	1993-1999	1.28 (1.20 to 1.35)	1996-2007	-0.57 (-1.45 to 0.93)	1992-2007	-0.29 (-0.46 to -0.05)
	2	2000-2005	-0.55 (-0.64 to -0.45)	1999-2005	-0.28 (-0.42 to -0.19)	2007-2013	-20.02 (-22.32 to -18.05)	1999-2006	-0.17 (-0.22 to -0.13)	2007-2013	-20.82 (-23.11 to -18.93)	2007-2010	-6.62 (-7.53 to -4.29)
	3	2005-2010	0.71 (0.51 to 0.83)	2005-2010	1.02 (0.71 to 1.18)	2013-2021	-8.51 (-10.27 to -6.14)	2006-2015	1.11 (1.07 to 1.14)	2013-2021	-9.24 (-10.79 to -7.25)	2010-2021	-0.79 (-1.17 to -0.24)
	4	2010-2015	1.18 (1.07 to 1.35)	2010-2015	1.74 (1.59 to 2.01)			2015-2019	-0.15 (-0.25 to -0.05)				
	5	2015-2019	-0.53 (-0.67 to -0.41)	2015-2019	0.04 (-0.17 to 0.22)			2019-2021	2.61 (2.33 to 2.86)				
	6	2019-2021	2.56 (2.20 to 2.87)	2019-2021	2.71 (2.14 to 3.08)								
Slovakia	0	1990-1996	1.03 (0.92 to 1.13)	1990-1995	1.20 (1.03 to 1.36)	1990-2010	-1.43 (-1.95 to -1.26)	1990-1995	1.18 (1.01 to 1.34)	1990-2013	-1.69 (-2.01 to -1.54)	1990-1995	-0.68 (-1.59 to -0.31)
	1	1996-1999	3.00 (2.77 to 3.18)	1995-1999	2.97 (2.79 to 3.19)	2010-2021	-0.62 (-1.10 to 1.15)	1995-1999	2.95 (2.76 to 3.19)	2013-2021	-0.41 (-1.30 to 2.41)	1995-1998	1.73 (0.73 to 2.21)
	2	1999-2005	0.67 (0.46 to 0.79)	1999-2006	0.78 (0.56 to 0.87)			1999-2006	0.77 (0.55 to 0.87)			1998-2011	-0.07 (-0.37 to 0.04)
	3	2005-2015	0.93 (0.83 to 1.03)	2006-2015	1.12 (1.01 to 1.23)			2006-2015	1.14 (1.02 to 1.26)			2011-2021	0.74 (0.51 to 1.08)
	4	2015-2018	1.47 (1.17 to 1.62)	2015-2018	1.85 (1.50 to 2.02)			2015-2018	1.88 (1.53 to 2.06)				
	5	2018-2021	0.74 (0.31 to 0.96)	2018-2021	0.66 (0.20 to 0.90)			2018-2021	0.63 (0.19 to 0.86)				
Slovenia	0	1990-1995	0.70 (0.30 to 0.95)	1990-1995	1.06 (0.68 to 1.28)	1990-1992	24.23 (14.02 to 32.90)	1990-1995	1.11 (0.67 to 1.33)	1990-1992	21.70 (12.11 to 29.38)	1990-1992	12.16 (9.96 to 14.19)
	1	1995-2000	2.45 (2.23 to 2.80)	1995-1999	2.98 (2.71 to 3.48)	1992-2004	-1.56 (-2.37 to -0.87)	1995-1999	2.98 (2.67 to 3.53)	1992-2004	-2.33 (-3.06 to -1.66)	1992-1995	-2.04 (-2.87 to -0.83)
	2	2000-2005	0.24 (0.04 to 0.76)	1999-2002	1.15 (0.69 to 1.84)	2004-2008	-15.60 (-20.08 to -11.75)	1999-2002	1.03 (0.49 to 1.86)	2004-2008	-13.09 (-17.00 to -9.70)	1995-2003	0.55 (0.25 to 1.42)
	3	2005-2019	-0.19 (-0.27 to -0.14)	2002-2019	0.19 (0.15 to 0.22)	2008-2021	-0.17 (-1.11 to 0.94)	2002-2019	0.16 (0.10 to 0.19)	2008-2021	-0.47 (-1.31 to 0.55)	2003-2008	-4.63 (-5.97 to -3.66)

Location	Segment	Incidence		Prevalence		Mortality		YLD		YLL		DALY	
		Period	APC (95%CI)	Period	APC (95%CI)	Period	APC (95%CI)	Period	APC (95%CI)	Period	APC (95%CI)	Period	APC (95%CI)
	4	2019-2021	2.23 (1.45 to 2.67)	2019-2021	2.29 (1.49 to 2.66)			2019-2021	2.22 (1.46 to 2.58)			2008-2021	0.08 (-0.16 to 0.37)
Solomon Islands	0	1990-1994	1.87 (1.59 to 2.05)	1990-1994	2.48 (2.08 to 2.75)	1990-2008	0.39 (0.25 to 0.50)	1990-1994	2.50 (2.09 to 2.74)	1990-2008	0.26 (0.11 to 0.40)	1990-2008	0.66 (0.51 to 0.79)
	1	1994-2000	2.76 (2.67 to 2.88)	1994-2000	3.55 (3.43 to 3.71)	2008-2014	2.63 (2.14 to 3.95)	1994-2000	3.57 (3.45 to 3.76)	2008-2014	3.08 (2.53 to 4.43)	2008-2014	3.17 (2.69 to 4.58)
	2	2000-2005	1.55 (1.45 to 1.64)	2000-2005	1.23 (1.11 to 1.34)	2014-2021	1.18 (0.57 to 1.52)	2000-2005	1.25 (1.12 to 1.37)	2014-2021	1.07 (0.48 to 1.46)	2014-2021	1.24 (0.75 to 1.57)
	3	2005-2010	3.51 (3.43 to 3.60)	2005-2010	4.16 (4.03 to 4.30)			2005-2010	4.18 (4.03 to 4.33)				
	4	2010-2017	1.69 (1.62 to 1.77)	2010-2017	2.54 (2.45 to 2.68)			2010-2017	2.54 (2.43 to 2.71)				
	5	2017-2021	1.11 (0.90 to 1.25)	2017-2021	1.92 (1.58 to 2.11)			2017-2021	1.87 (1.47 to 2.10)				
Somalia	0	1990-1996	0.84 (0.74 to 0.90)	1990-2003	1.84 (1.81 to 1.88)	1990-1992	0.43 (0.15 to 0.96)	1990-2002	1.79 (1.76 to 1.82)	1990-1992	0.35 (-0.04 to 1.22)	1990-1993	0.78 (0.32 to 1.07)
	1	1996-2005	1.01 (0.98 to 1.07)	2003-2014	2.16 (1.85 to 2.21)	1992-1998	1.51 (1.38 to 1.73)	2002-2014	2.17 (2.15 to 2.20)	1992-1998	1.44 (0.37 to 1.85)	1993-1997	1.70 (1.45 to 1.97)
	2	2005-2010	1.50 (1.44 to 1.64)	2014-2019	1.35 (1.20 to 2.18)	1998-2010	0.59 (0.55 to 0.63)	2014-2021	1.31 (1.27 to 1.35)	1998-2010	0.34 (0.25 to 0.42)	1997-2010	0.64 (0.59 to 0.68)
	3	2010-2015	1.19 (1.13 to 1.24)	2019-2021	1.78 (1.39 to 1.97)	2010-2014	-0.57 (-0.77 to -0.33)			2010-2021	-0.39 (-0.47 to -0.31)	2010-2021	-0.02 (-0.07 to 0.04)
	4	2015-2019	0.38 (0.28 to 0.43)			2014-2019	0.03 (-0.07 to 0.33)						
	5	2019-2021	1.02 (0.82 to 1.15)			2019-2021	-1.19 (-1.57 to -0.77)						
South Africa	0	1990-1997	1.58 (1.37 to 1.74)	1990-1999	2.04 (1.76 to 2.21)	1990-1995	2.72 (-2.09 to 5.28)	1990-1999	2.03 (1.77 to 2.18)	1990-2007	3.82 (3.46 to 4.40)	1990-1995	1.76 (-1.39 to 3.14)
	1	1997-2005	2.62 (2.49 to 2.82)	1999-2005	2.78 (1.92 to 3.30)	1995-1998	8.77 (2.79 to 10.51)	1999-2005	2.86 (1.97 to 3.35)	2007-2015	0.71 (-0.33 to 2.09)	1995-1998	6.69 (3.97 to 7.95)
	2	2005-2009	1.48 (1.13 to 1.82)	2005-2010	1.78 (1.22 to 2.91)	1998-2008	3.07 (2.03 to 3.97)	2005-2010	1.88 (1.32 to 2.99)	2015-2021	-2.90 (-5.98 to -1.67)	1998-2008	2.93 (2.12 to 3.72)
	3	2009-2018	2.05 (1.96 to 2.27)	2010-2015	2.92 (1.72 to 3.47)	2008-2015	0.71 (-0.82 to 1.78)	2010-2015	3.05 (1.81 to 3.63)			2008-2015	1.12 (-0.42 to 2.17)
	4	2018-2021	-0.20 (-0.58 to 0.36)	2015-2018	2.19 (1.58 to 2.82)	2015-2021	-2.60 (-4.75 to -1.66)	2015-2018	2.27 (1.52 to 2.91)			2015-2021	-1.77 (-4.19 to -0.80)
	5			2018-2021	0.34 (-0.22 to 0.73)			2018-2021	0.04 (-0.53 to 0.45)				
South Sudan	0	1990-1996	0.58 (0.52 to 0.62)	1990-1999	1.60 (1.56 to 1.62)	1990-1996	0.27 (0.12 to 0.50)	1990-2001	1.60 (1.57 to 1.62)	1990-1995	0.25 (-0.01 to 0.64)	1990-1995	0.45 (0.22 to 0.78)
	1	1996-2002	0.87 (0.72 to 0.91)	1999-2010	1.91 (1.88 to 1.93)	1996-2001	-0.51 (-0.95 to -0.28)	2001-2010	1.96 (1.90 to 2.01)	1995-2002	-0.77 (-1.13 to -0.59)	1995-2002	-0.38 (-0.69 to -0.23)
	2	2002-2006	0.99 (0.91 to 1.24)	2010-2014	2.23 (1.98 to 2.34)	2001-2011	0.43 (0.32 to 0.55)	2010-2014	2.24 (2.10 to 2.35)	2002-2011	0.47 (0.29 to 0.67)	2002-2011	0.78 (0.63 to 0.93)
	3	2006-2015	1.29 (1.27 to 1.31)	2014-2019	1.58 (1.49 to 2.15)	2011-2021	1.88 (1.79 to 1.98)	2014-2021	1.49 (1.44 to 1.53)	2011-2021	2.13 (2.01 to 2.28)	2011-2021	2.05 (1.95 to 2.17)
	4	2015-2019	0.59 (0.53 to 0.62)	2019-2021	1.88 (1.66 to 1.98)								
	5	2019-2021	1.09 (0.97 to 1.18)										
Spain	0	1990-1996	2.20 (1.82 to 2.41)	1990-1995	2.45 (1.96 to 2.66)	1990-2001	-3.67 (-4.25 to -2.83)	1990-1997	3.77 (3.65 to 3.97)	1990-2001	-4.48 (-4.87 to -3.91)	1990-1992	-0.96 (-1.35 to -0.30)
	1	1996-2000	3.38 (3.07 to 3.76)	1995-1999	3.21 (2.94 to 3.49)	2001-2004	-1.56 (-4.57 to -0.92)	1997-2000	2.85 (1.72 to 3.20)	2001-2004	-2.25 (-5.06 to -1.64)	1992-1998	0.29 (0.13 to 0.78)
	2	2000-2005	1.30 (1.09 to 1.49)	1999-2004	2.00 (1.77 to 2.13)	2004-2008	-3.85 (-6.88 to -1.92)	2000-2010	0.22 (0.12 to 0.29)	2004-2008	-4.19 (-7.45 to -2.97)	1998-2003	-0.46 (-0.95 to -0.15)
	3	2005-2010	0.06 (-0.32 to 0.27)	2004-2010	0.38 (0.28 to 0.48)	2008-2013	-6.28 (-7.79 to -1.66)	2010-2015	2.42 (2.20 to 2.95)	2008-2013	-6.93 (-8.27 to -1.96)	2003-2011	-1.09 (-1.49 to 0.55)
	4	2010-2015	1.92 (1.71 to 2.20)	2010-2015	2.63 (2.49 to 2.77)	2013-2021	-2.55 (-3.94 to -1.41)	2015-2021	1.29 (1.06 to 1.46)	2013-2021	-2.54 (-3.49 to -1.72)	2011-2021	0.85 (0.72 to 1.03)
	5	2015-2021	0.24 (0.08 to 0.39)	2015-2021	1.63 (1.48 to 1.76)								
Sri Lanka	0	1990-1998	3.28 (3.24 to 3.34)	1990-1993	4.20 (4.00 to 4.54)	1990-1993	-1.94 (-4.70 to -0.23)	1990-1996	3.99 (3.93 to 4.02)	1990-2002	1.40 (0.91 to 1.82)	1990-1993	0.74 (-0.61 to 1.54)
	1	1998-2001	2.74 (2.56 to 3.04)	1993-1999	3.73 (3.61 to 3.80)	1993-1997	2.51 (1.42 to 3.99)	1996-1999	4.35 (4.27 to 4.41)	2002-2009	4.30 (3.51 to 6.42)	1993-1997	3.51 (2.82 to 4.38)
	2	2001-2008	2.31 (2.26 to 2.37)	1999-2004	3.21 (3.08 to 3.28)	1997-2002	0.79 (-0.67 to 5.19)	1999-2004	3.53 (3.50 to 3.57)	2009-2021	-0.19 (-0.95 to 0.33)	1997-2001	2.11 (1.37 to 3.53)
	3	2008-2015	2.12 (2.06 to 2.17)	2004-2015	2.47 (2.45 to 2.49)	2002-2008	4.72 (0.49 to 5.96)	2004-2009	2.60 (2.56 to 2.64)			2001-2009	3.49 (1.49 to 4.17)
	4	2015-2018	1.34 (1.25 to 1.70)	2015-2021	1.55 (1.50 to 1.59)	2008-2015	0.84 (-1.88 to 1.79)	2009-2014	2.01 (1.97 to 2.07)			2009-2015	1.10 (0.58 to 1.87)
	5	2018-2021	0.98 (0.78 to 1.08)			2015-2021	-2.02 (-5.01 to -0.57)	2014-2019	1.67 (1.63 to 1.75)			2015-2021	-0.20 (-1.14 to 0.29)
	6							2019-2021	1.28 (1.14 to 1.44)				
Sudan	0	1990-1999	2.92 (2.88 to 2.96)	1990-1993	4.01 (3.73 to 4.31)	1990-2001	0.05 (-0.01 to 0.11)	1990-1996	3.03 (2.72 to 3.18)	1990-1995	0.20 (0.10 to 0.37)	1990-1992	1.67 (1.38 to 1.89)
	1	1999-2006	1.80 (1.76 to 1.84)	1993-2000	3.04 (2.95 to 3.11)	2001-2004	4.26 (3.94 to 4.49)	1996-2000	4.07 (3.76 to 4.44)	1995-1998	-0.50 (-0.67 to -0.24)	1992-1996	1.28 (1.11 to 1.85)
	2	2006-2014	3.04 (2.99 to 3.08)	2000-2005	1.85 (1.76 to 1.95)	2004-2009	1.96 (1.75 to 2.24)	2000-2005	1.77 (1.45 to 1.95)	1998-2001	0.23 (0.02 to 0.41)	1996-2001	1.81 (1.73 to 2.52)
	3	2014-2019	1.67 (1.53 to 2.07)	2005-2014	3.32 (3.28 to 3.37)	2009-2021	1.44 (1.36 to 1.50)	2005-2014	3.29 (3.20 to 3.41)	2001-2004	3.97 (3.73 to 4.15)	2001-2010	2.51 (2.29 to 2.62)
	4	2019-2021	2.25 (1.86 to 2.46)	2014-2019	1.80 (1.62 to 1.89)			2014-2021	1.91 (1.78 to 2.05)	2004-2009	1.78 (1.65 to 1.98)	2010-2014	2.26 (1.58 to 2.38)
	5			2019-2021	2.62 (2.19 to 2.88)					2009-2019	1.32 (1.28 to 1.45)	2014-2021	1.64 (1.55 to 1.72)
	6									2019-2021	0.84 (0.53 to 1.23)		
Suriname	0	1990-1994	2.21 (2.02 to 2.38)	1990-1994	2.61 (2.42 to 2.79)	1990-1993	2.62 (1.25 to 4.73)	1990-1994	2.63 (2.44 to 2.79)	1990-1993	2.96 (1.66 to 4.66)	1990-1993	2.83 (2.30 to 3.39)
	1	1994-2000	1.29 (1.18 to 1.38)	1994-2000	1.75 (1.58 to 1.86)	1993-1996	-7.36 (-8.48 to -5.41)	1994-2000	1.68 (1.59 to 1.76)	1993-1996	-7.53 (-8.52 to -6.20)	1993-1996	-3.89 (-4.37 to -3.44)
	2	2000-2008	2.61 (2.55 to 2.69)	2000-2009	2.93 (2.75 to 3.01)	1996-2002	2.51 (1.83 to 3.73)	2000-2010	2.91 (2.87 to 2.96)	1996-2002	2.78 (2.11 to 3.64)	1996-2002	2.43 (2.08 to 2.81)
	3	2008-2013	2.21 (2.08 to 2.40)	2009-2013	2.51 (2.38 to 2.92)	2002-2011	-0.69 (-1.64 to -0.27)	2010-2018	2.25 (2.19 to 2.31)	2002-2007	-1.88 (-3.23 to 1.08)	2002-2007	0.37 (-0.31 to 2.18)
	4	2013-2018	1.85 (1.67 to 1.96)	2013-2018	2.19 (2.05 to 2.29)	2011-2019	1.66 (1.20 to 3.05)	2018-2021	0.66 (0.48 to 0.81)	2007-2012	-0.17 (-1.42 to 1.68)	2007-2012	1.22 (0.46 to 2.30)
	5	2018-2021	0.75 (0.51 to 1.00)	2018-2021	0.86 (0.66 to 1.03)	2019-2021	-5.07 (-8.69 to -1.24)			2012-2018	2.45 (1.78 to 4.03)	2012-2018	2.34 (1.86 to 3.07)
	6									2018-2021	-2.66 (-5.84 to -0.81)	2018-2021	-1.13 (-2.28 to -0.21)
Sweden	0	1990-2001	1.22 (0.99 to 1.29)	1990-2001	1.35 (0.85 to 1.46)	1990-1993	-0.92 (-2.42 to 1.43)	1990-2002	1.25 (0.98 to 1.37)	1990-1992	0.48 (-2.50 to 3.02)	1990-1997	-0.35 (-0.81 to -0.02)
	1	2001-2010	1.46 (1.35 to 1.69)	2001-2011	1.67 (1.50 to 1.94)	1993-1997	-3.35 (-4.86 to 4.22)	2002-2011	1.95 (1.22 to 2.12)	1992-1997	-3.58 (-5.64 to 3.12)	1997-2005	1.45 (1.15 to 2.12)
	2	2010-2014	2.57 (2.23 to 3.07)	2011-2014	3.03 (2.34 to 3.41)	1997-2001	4.37 (-1.34 to 6.30)	2011-2014	2.90 (2.14 to 3.31)	1997-2000	4.31 (-0.24 to 5.67)	2005-2013	0.07 (-0.95 to 0.38)
	3	2014-2019	0.77 (0.28 to 0.94)	2014-2018	0.70 (-0.03 to 1.08)	2001-2009	-0.54 (-1.19 to 0.38)	2014-2018	0.64 (-0.09 to 1.07)	2000-2008	-0.37 (-1.29 to 0.26)	2013-2021	1.16 (0.81 to 1.92)
	4	2019-2021	3.39 (2.44 to 4.23)	2018-2021	3.28 (2.55 to 4.60)	2009-2014	-5.26 (-6.81 to -4.33)	2018-2021	3.11 (2.41 to 4.39)	2008-2014	-5.11 (-6.91 to -4.20)		
	5					2014-2017	5.63 (3.23 to 7.08)			2014-2017	5.72 (2.71 to 7.43)		
	6					2017-2021	-3.26 (-4.94 to -2.24)			2017-2021	-4.18 (-6.33 to -2.90)		
Switzerland	0	1990-1999	1.80 (1.69 to 1.95)	1990-2001	1.92 (1.86 to 2.00)	1990-2003	-2.48 (-2.89 to -2.00)	1990-2001	1.93 (1.88 to 2.00)	1990-1996	-2.02 (-3.06 to 0.68)	1990-1996	0.09 (-0.36 to 1.20)
	1	1999-2010	1.26 (1.13 to 1.32)	2001-2010	1.63 (1.50 to 1.69)	2003-2011	-6.19 (-9.13 to -5.26)	2001-2010	1.59 (1.47 to 1.65)	1996-2003	-3.64 (-6.78 to -2.62)	1996-2011	-0.52 (-1.53 to 1.31)
	2	2010-2015	2.16 (1.91 to 2.42)	2010-2015	2.86 (2.64 to 3.01)	2011-2021	-3.48 (-4.19 to -2.06)	2010-2015	2.76 (2.54 to 2.93)	2003-2012	-5.82 (-8.39 to -1.75)	2011-2021	1.92 (1.48 to 2.44)

Location	Segment	Incidence		Prevalence		Mortality		YLD		YLL		DALY	
		Period	APC (95%CI)	Period	APC (95%CI)	Period	APC (95%CI)	Period	APC (95%CI)	Period	APC (95%CI)	Period	APC (95%CI)
	3	2015-2019	3.74 (3.44 to 4.19)	2015-2019	3.94 (3.72 to 4.25)			2015-2019	3.95 (3.72 to 4.25)	2012-2021	-3.64 (-6.79 to -1.22)		
	4	2019-2021	0.87 (0.39 to 1.55)	2019-2021	1.25 (0.86 to 1.73)			2019-2021	1.11 (0.73 to 1.63)				
Syrian Arab Republic	0	1990-1995	2.30 (2.24 to 2.34)	1990-2000	2.76 (2.74 to 2.77)	1990-1992	7.16 (3.85 to 9.56)	1990-2000	2.75 (2.74 to 2.77)	1990-1992	6.00 (3.17 to 8.21)	1990-1992	4.55 (3.40 to 5.50)
	1	1995-2000	2.61 (2.56 to 2.67)	2000-2004	2.40 (2.35 to 2.46)	1992-2000	1.42 (0.73 to 1.87)	2000-2005	2.36 (2.33 to 2.39)	1992-1999	1.26 (0.23 to 1.61)	1992-1999	1.93 (1.54 to 2.13)
	2	2000-2004	2.22 (2.17 to 2.28)	2004-2012	2.01 (1.98 to 2.02)	2000-2007	-2.79 (-3.74 to -2.23)	2005-2011	1.94 (1.90 to 1.97)	1999-2006	-3.97 (-4.52 to -3.55)	1999-2003	-1.25 (-1.76 to 2.17)
	3	2004-2012	1.78 (1.75 to 1.80)	2012-2015	2.20 (2.12 to 2.29)	2007-2021	0.30 (0.04 to 0.58)	2011-2015	2.17 (2.11 to 2.26)	2006-2021	0.25 (0.05 to 0.44)	2003-2007	-0.35 (-1.03 to 1.70)
	4	2012-2015	2.00 (1.85 to 2.14)	2015-2019	2.70 (2.65 to 2.76)			2015-2019	2.64 (2.59 to 2.70)			2007-2021	1.40 (0.95 to 1.75)
	5	2015-2019	2.34 (2.29 to 2.42)	2019-2021	1.75 (1.65 to 1.84)			2019-2021	1.60 (1.49 to 1.69)				
	6	2019-2021	1.39 (1.28 to 1.48)										
Taiwan (Province of China)	0	1990-1993	1.68 (1.54 to 1.82)	1990-1995	0.96 (0.77 to 1.06)	1990-1994	6.28 (4.80 to 8.29)	1990-1993	1.96 (1.80 to 2.19)	1990-1995	4.70 (3.48 to 6.34)	1990-1995	3.91 (2.94 to 5.17)
	1	1993-1998	1.12 (1.05 to 1.20)	1995-1998	1.63 (1.42 to 1.75)	1994-2000	-0.02 (-1.04 to 1.34)	1993-1999	1.53 (1.44 to 1.59)	1995-2000	-1.22 (-2.74 to 1.26)	1995-2000	-0.52 (-1.65 to 1.59)
	2	1998-2004	0.92 (0.80 to 0.96)	1998-2004	1.24 (1.08 to 1.28)	2000-2006	-3.30 (-4.56 to -2.36)	1999-2004	0.88 (0.79 to 1.00)	2000-2006	-3.92 (-11.96 to -3.05)	2000-2006	-2.49 (-7.96 to -1.77)
	3	2004-2011	0.32 (0.26 to 0.36)	2004-2015	0.61 (0.58 to 0.64)	2006-2009	-11.86 (-13.09 to -8.82)	2004-2010	0.43 (0.29 to 0.50)	2006-2009	-12.99 (-14.19 to -3.48)	2006-2009	-8.27 (-9.30 to -0.47)
	4	2011-2016	0.74 (0.66 to 0.85)	2015-2021	2.53 (2.45 to 2.59)	2009-2021	-2.99 (-3.47 to -2.39)	2010-2015	0.61 (0.55 to 0.74)	2009-2021	-3.55 (-4.16 to -2.95)	2009-2021	-1.01 (-1.81 to -0.42)
	5	2016-2021	1.66 (1.57 to 1.74)					2015-2021	2.35 (2.29 to 2.40)				
Tajikistan	0	1990-1995	2.82 (2.53 to 2.98)	1990-1995	3.29 (3.01 to 3.45)	1990-1994	8.40 (7.88 to 8.88)	1990-1995	3.28 (2.96 to 3.44)	1990-1994	8.39 (7.92 to 8.89)	1990-1994	6.71 (6.44 to 6.99)
	1	1995-2005	3.89 (3.80 to 3.96)	1995-2005	3.91 (3.86 to 3.97)	1994-1997	-3.42 (-3.93 to -2.85)	1995-2005	3.99 (3.78 to 4.07)	1994-1997	-3.15 (-3.67 to -2.57)	1994-1997	-0.89 (-1.18 to -0.63)
	2	2005-2014	2.76 (2.70 to 3.08)	2005-2018	2.67 (2.64 to 2.70)	1997-2000	3.09 (2.43 to 3.55)	2005-2013	2.81 (2.76 to 4.09)	1997-2001	2.10 (1.69 to 2.74)	1997-2005	2.56 (2.45 to 2.74)
	3	2014-2018	2.16 (1.94 to 2.63)	2018-2021	1.38 (1.14 to 1.76)	2000-2009	2.15 (1.51 to 2.28)	2013-2018	2.58 (2.32 to 2.75)	2001-2009	1.36 (0.60 to 1.51)	2005-2009	1.72 (1.05 to 2.19)
	4	2018-2021	1.27 (0.82 to 1.60)			2009-2013	-3.39 (-4.10 to -2.85)	2018-2021	1.33 (0.99 to 1.64)	2009-2013	-2.80 (-3.65 to -1.97)	2009-2013	-0.44 (-0.99 to -0.10)
	5					2013-2016	0.74 (-0.47 to 1.36)			2013-2016	0.74 (-0.34 to 1.31)	2013-2016	1.66 (1.01 to 2.02)
	6					2016-2021	-1.98 (-2.98 to -1.51)			2016-2021	-2.07 (-2.78 to -1.69)	2016-2021	-0.20 (-0.64 to 0.03)
Thailand	0	1990-1995	2.16 (2.05 to 2.41)	1990-2000	2.91 (2.82 to 3.02)	1990-1995	2.83 (2.02 to 3.93)	1990-2000	2.92 (2.83 to 3.03)	1990-1996	2.63 (2.00 to 3.47)	1990-1996	2.77 (2.34 to 3.35)
	1	1995-2004	1.89 (1.79 to 1.97)	2000-2012	2.14 (1.93 to 2.21)	1995-2005	-0.34 (-0.69 to -0.05)	2000-2011	2.13 (1.86 to 2.21)	1996-2004	-0.33 (-0.88 to 0.09)	1996-2004	0.41 (0.03 to 0.76)
	2	2004-2010	1.68 (1.43 to 2.14)	2012-2021	2.44 (2.34 to 2.67)	2005-2013	-4.00 (-4.35 to -3.67)	2011-2021	2.48 (2.38 to 2.70)	2004-2013	-4.24 (-4.55 to -3.95)	2004-2013	-1.88 (-2.19 to -1.65)
	3	2010-2021	2.24 (2.19 to 2.29)			2013-2021	1.08 (0.72 to 1.42)			2013-2021	0.50 (0.12 to 0.87)	2013-2021	1.54 (1.20 to 1.92)
Timor-Leste	0	1990-1995	2.34 (2.24 to 2.44)	1990-1995	3.62 (3.46 to 3.78)	1990-2006	-0.42 (-0.50 to -0.34)	1990-1994	3.42 (3.16 to 3.63)	1990-2006	-0.83 (-0.96 to -0.71)	1990-2006	0.67 (0.61 to 0.73)
	1	1995-2000	3.74 (3.63 to 3.91)	1995-2000	4.70 (4.56 to 4.91)	2006-2019	1.75 (1.66 to 1.89)	1994-2000	4.50 (4.37 to 4.70)	2006-2018	1.75 (1.60 to 2.06)	2006-2019	2.71 (2.64 to 2.80)
	2	2000-2006	2.83 (2.63 to 2.93)	2000-2006	3.27 (3.07 to 3.37)	2019-2021	-1.14 (-2.13 to 0.20)	2000-2007	3.52 (3.26 to 3.63)	2018-2021	-0.09 (-1.81 to 1.01)	2019-2021	0.70 (-0.07 to 1.76)
	3	2006-2014	3.33 (3.23 to 3.41)	2006-2014	3.84 (3.74 to 3.92)			2007-2015	3.94 (3.76 to 4.02)				
	4	2014-2019	3.69 (3.56 to 3.97)	2014-2019	4.29 (4.16 to 4.60)			2015-2019	4.32 (4.13 to 4.62)				
	5	2019-2021	1.88 (1.52 to 2.36)	2019-2021	2.27 (1.89 to 2.80)			2019-2021	2.14 (1.76 to 2.62)				
Togo	0	1990-1993	1.63 (1.55 to 1.71)	1990-1994	2.63 (2.51 to 2.75)	1990-1995	1.73 (1.33 to 1.95)	1990-1994	2.51 (2.37 to 2.63)	1990-1995	1.55 (1.04 to 1.80)	1990-1996	1.78 (1.40 to 1.92)
	1	1993-1998	1.27 (1.19 to 1.30)	1994-2000	1.23 (1.16 to 1.29)	1995-2002	2.57 (2.42 to 2.85)	1994-2001	1.47 (1.41 to 1.53)	1995-2002	2.38 (2.20 to 2.76)	1996-2002	2.21 (2.05 to 2.55)
	2	1998-2001	1.53 (1.47 to 1.76)	2000-2010	2.57 (2.53 to 2.61)	2002-2005	0.27 (-0.02 to 0.88)	2001-2010	2.63 (2.58 to 2.68)	2002-2005	0.01 (-0.33 to 0.88)	2002-2005	0.72 (0.47 to 1.26)
	3	2001-2010	1.98 (1.97 to 2.00)	2010-2021	2.13 (2.10 to 2.17)	2005-2009	1.95 (1.57 to 2.42)	2010-2021	2.10 (2.06 to 2.14)	2005-2009	2.01 (1.48 to 2.56)	2005-2009	2.15 (1.77 to 2.56)
	4	2010-2013	1.67 (1.61 to 1.80)			2009-2014	-0.09 (-0.48 to 0.16)			2009-2014	-0.26 (-0.70 to 0.35)	2009-2014	0.42 (0.13 to 0.72)
	5	2013-2019	1.47 (1.42 to 1.49)			2014-2021	0.44 (0.29 to 0.83)			2014-2021	0.25 (0.06 to 0.73)	2014-2021	0.79 (0.65 to 1.15)
	6	2019-2021	1.92 (1.79 to 2.00)										
Tokelau	0	1990-1994	2.40 (2.31 to 2.49)	1990-1994	3.23 (3.07 to 3.38)	1990-1995	1.23 (1.05 to 1.31)	1990-1994	3.24 (3.09 to 3.38)	1990-2002	1.17 (1.13 to 1.24)	1990-2002	1.58 (1.55 to 1.63)
	1	1994-2000	1.89 (1.78 to 1.93)	1994-2006	2.54 (2.51 to 2.57)	1995-2002	1.36 (0.20 to 1.46)	1994-2006	2.54 (2.51 to 2.57)	2002-2005	0.05 (-0.41 to 0.78)	2002-2005	0.82 (0.37 to 1.30)
	2	2000-2005	2.09 (2.02 to 2.21)	2006-2010	2.03 (1.84 to 2.22)	2002-2005	0.12 (-0.40 to 0.19)	2006-2010	2.01 (1.84 to 2.24)	2005-2015	-0.54 (-0.68 to -0.47)	2005-2018	0.22 (0.17 to 0.25)
	3	2005-2009	1.81 (1.65 to 1.90)	2010-2014	1.43 (1.26 to 1.62)	2005-2015	-0.43 (-0.46 to -0.40)	2010-2014	1.42 (1.24 to 1.62)	2015-2021	0.32 (0.16 to 0.54)	2018-2021	1.02 (0.73 to 1.50)
	4	2009-2014	1.22 (1.14 to 1.29)	2014-2019	0.92 (0.78 to 1.01)	2015-2018	0.35 (0.20 to 0.44)	2014-2019	0.87 (0.71 to 0.96)				
	5	2014-2019	0.68 (0.61 to 0.74)	2019-2021	2.25 (1.95 to 2.46)	2018-2021	-0.14 (-0.38 to -0.02)	2019-2021	2.17 (1.85 to 2.38)				
	6	2019-2021	1.78 (1.60 to 1.94)										
Tonga	0	1990-1996	0.82 (0.79 to 0.84)	1990-1993	1.11 (0.89 to 1.22)	1990-1995	-0.20 (-0.91 to 0.17)	1990-1993	1.05 (0.91 to 1.14)	1990-1996	0.35 (-0.02 to 0.55)	1990-1992	-0.01 (-0.35 to 0.59)
	1	1996-2000	1.80 (1.76 to 1.84)	1993-1996	1.73 (1.63 to 1.84)	1995-1998	2.34 (1.43 to 2.76)	1993-1996	1.71 (1.62 to 1.79)	1996-1999	1.86 (0.65 to 2.12)	1992-1996	0.79 (0.61 to 2.12)
	2	2000-2005	1.47 (1.43 to 1.51)	1996-2000	2.82 (2.74 to 2.88)	1998-2021	0.57 (0.50 to 0.62)	1996-2000	2.76 (2.70 to 2.82)	1999-2003	-0.42 (-0.79 to 1.75)	1996-1999	1.99 (0.16 to 2.18)
	3	2005-2009	1.89 (1.84 to 1.94)	2000-2010	2.22 (2.20 to 2.24)			2000-2010	2.19 (2.17 to 2.21)	2003-2017	0.39 (-0.35 to 0.86)	1999-2003	0.26 (-0.00 to 1.01)
	4	2009-2015	1.43 (1.40 to 1.46)	2010-2015	1.73 (1.67 to 1.81)			2010-2015	1.73 (1.68 to 1.79)	2017-2021	-0.09 (-1.26 to 0.33)	2003-2021	0.81 (0.59 to 0.88)
	5	2015-2019	2.01 (1.97 to 2.06)	2015-2018	2.71 (2.57 to 2.79)			2015-2018	2.66 (2.55 to 2.73)				
	6	2019-2021	1.55 (1.42 to 1.72)	2018-2021	1.99 (1.85 to 2.08)			2018-2021	1.95 (1.83 to 2.04)				
Trinidad and Tobago	0	1990-1992	1.28 (0.98 to 1.50)	1990-1994	1.89 (1.82 to 1.98)	1990-2005	-1.17 (-1.39 to -0.90)	1990-1994	1.84 (1.76 to 1.92)	1990-1995	0.12 (-0.86 to 2.25)	1990-1994	0.76 (-0.08 to 2.33)
	1	1992-1995	0.81 (-0.18 to 0.90)	1994-2001	0.57 (0.51 to 0.61)	2005-2012	-3.45 (-5.66 to -2.70)	1994-2001	0.46 (0.42 to 0.50)	1995-2008	-2.31 (-2.65 to -1.95)	1994-2008	-1.38 (-1.62 to -0.96)
	2	1995-2000	-0.13 (-0.20 to 0.81)	2001-2014	1.20 (1.17 to 1.23)	2012-2021	0.15 (-0.80 to 1.50)	2001-2014	1.17 (1.15 to 1.19)	2008-2012	-5.05 (-6.83 to -3.61)	2008-2012	-2.69 (-4.07 to 1.17)
	3	2000-2015	0.82 (0.79 to 0.85)	2014-2019	0.58 (0.42 to 0.71)			2014-2019	0.54 (0.47 to 0.64)	2012-2021	0.71 (-0.25 to 2.13)	2012-2021	0.59 (-0.79 to 1.60)
	4	2015-2019	-0.01 (-0.28 to 0.20)	2019-2021	2.05 (1.72 to 2.30)			2019-2021	1.87 (1.64 to 2.06)				
	5	2019-2021	1.64 (1.21 to 1.95)										
Tunisia	0	1990-1992	6.72 (6.47 to 7.00)	1990-1992	7.17 (6.91 to 7.45)	1990-1996	1.56 (1.19 to 2.30)	1990-1993	6.82 (6.66 to 6.99)	1990-1996	1.44 (0.94 to 2.45)	1990-1992	5.05 (4.50 to 5.47)
	1	1992-1995	5.08 (4.92 to 5.24)	1992-1995	5.49 (5.33 to 5.65)	1996-2000	-0.23 (-1.31 to 0.62)	1993-1996	4.85 (4.70 to 5.00)	1996-2000	-0.04 (-1.14 to 2.67)	1992-1996	3.63 (3.28 to 3.85)
	2	1995-1999	3.54 (3.37 to 3.66)	1995-1999	3.88 (3.71 to 3.99)	2000-2008	2.93 (2.54 to 3.76)	1996-2000	3.57 (3.44 to 3.74)	2000-2008	2.71 (0.84 to 3.79)	1996-2000	2.27 (1.96 to 2.53)

Location	Segment	Incidence		Prevalence		Mortality		YLD		YLL		DALY	
		Period	APC (95%CI)	Period	APC (95%CI)	Period	APC (95%CI)	Period	APC (95%CI)	Period	APC (95%CI)	Period	APC (95%CI)
	3	1999-2011	2.77 (2.74 to 2.81)	1999-2009	3.03 (3.00 to 3.08)	2008-2021	0.90 (0.63 to 1.12)	2000-2010	2.98 (2.94 to 3.03)	2008-2021	0.82 (0.51 to 1.11)	2000-2008	2.88 (2.79 to 3.13)
	4	2011-2014	2.49 (2.37 to 2.65)	2009-2014	2.75 (2.64 to 2.83)			2010-2014	2.68 (2.52 to 2.79)			2008-2014	2.20 (1.96 to 2.40)
	5	2014-2019	0.85 (0.71 to 0.92)	2014-2019	1.25 (1.14 to 1.33)			2014-2019	1.18 (1.05 to 1.26)			2014-2021	1.07 (0.93 to 1.20)
	6	2019-2021	1.96 (1.61 to 2.19)	2019-2021	2.30 (1.96 to 2.50)			2019-2021	1.99 (1.66 to 2.20)				
Turkmenistan	0	1990-1995	3.05 (2.69 to 3.23)	1990-2005	3.90 (3.86 to 3.93)	1990-1994	14.88 (13.18 to 17.23)	1990-2005	3.85 (3.82 to 3.89)	1990-1994	17.08 (12.92 to 21.81)	1990-1994	12.17 (11.03 to 13.75)
	1	1995-2004	3.58 (3.51 to 3.70)	2005-2010	2.14 (1.95 to 2.28)	1994-1997	-0.22 (-3.16 to 6.05)	2005-2010	2.07 (1.88 to 2.19)	1994-2000	-3.59 (-7.81 to -1.40)	1994-1997	0.50 (-1.41 to 5.05)
	2	2004-2010	1.76 (1.60 to 1.87)	2010-2016	3.00 (2.85 to 3.22)	1997-2000	-6.43 (-8.18 to 3.52)	2010-2016	3.06 (2.93 to 3.25)	2000-2021	2.81 (2.38 to 3.29)	1997-2000	-3.62 (-4.93 to 4.48)
	3	2010-2015	2.97 (2.73 to 3.28)	2016-2021	1.77 (1.60 to 1.94)	2000-2004	4.40 (0.96 to 7.00)	2016-2021	1.69 (1.53 to 1.87)			2000-2004	4.74 (1.57 to 6.75)
	4	2015-2021	1.65 (1.49 to 1.80)			2004-2012	0.92 (-1.17 to 2.51)					2004-2012	1.70 (-0.03 to 5.21)
	5					2012-2015	8.75 (4.45 to 10.52)					2012-2015	6.87 (1.93 to 8.17)
	6					2015-2021	0.03 (-2.27 to 1.31)					2015-2021	0.39 (-1.09 to 1.31)
Tuvalu	0	1990-1994	2.05 (1.91 to 2.14)	1990-1994	2.98 (2.55 to 3.24)	1990-1994	1.12 (0.60 to 1.34)	1990-1994	2.79 (2.38 to 3.01)	1990-1994	0.89 (0.38 to 1.10)	1990-1994	1.20 (0.83 to 1.36)
	1	1994-2000	2.50 (2.46 to 2.56)	1994-2000	3.36 (1.73 to 3.58)	1994-1997	2.10 (1.78 to 2.32)	1994-2000	3.43 (3.33 to 3.63)	1994-1997	1.95 (1.61 to 2.17)	1994-1997	2.26 (1.96 to 2.44)
	2	2000-2005	1.39 (1.34 to 1.43)	2000-2005	1.67 (1.50 to 2.74)	1997-2001	1.36 (0.80 to 1.51)	2000-2005	1.66 (1.50 to 1.78)	1997-2001	1.08 (0.70 to 1.24)	1997-2001	1.41 (1.17 to 1.54)
	3	2005-2010	2.30 (2.25 to 2.35)	2005-2011	2.76 (2.31 to 2.98)	2001-2005	-0.12 (-0.39 to 0.20)	2005-2010	3.00 (2.83 to 3.23)	2001-2005	-0.55 (-0.86 to -0.31)	2001-2005	-0.10 (-0.34 to 0.06)
	4	2010-2014	1.52 (1.45 to 1.63)	2011-2021	2.20 (2.12 to 2.27)	2005-2017	0.54 (0.49 to 0.63)	2010-2021	2.17 (2.11 to 2.22)	2005-2017	0.51 (0.46 to 0.58)	2005-2016	0.99 (0.94 to 1.05)
	5	2014-2019	1.21 (1.09 to 1.27)			2017-2021	-0.46 (-0.81 to -0.20)			2017-2021	-0.58 (-0.95 to -0.29)	2016-2021	0.24 (0.05 to 0.40)
	6	2019-2021	1.76 (1.55 to 1.90)										
TÃ□Â¼rkiye	0	1990-2001	1.49 (1.39 to 1.61)	1990-1999	2.24 (2.05 to 2.53)	1990-2006	-3.51 (-4.05 to -3.06)	1990-2011	2.12 (2.04 to 2.19)	1990-2006	-3.49 (-3.78 to -3.23)	1990-1999	-1.49 (-1.64 to -1.14)
	1	2001-2008	1.06 (0.54 to 1.46)	1999-2010	1.44 (1.26 to 1.58)	2006-2009	12.09 (-4.40 to 13.50)	2011-2014	11.07 (2.52 to 11.67)	2006-2010	7.84 (5.55 to 10.89)	1999-2003	-2.30 (-3.01 to -1.70)
	2	2008-2011	2.19 (1.05 to 2.49)	2010-2014	8.85 (8.46 to 9.25)	2009-2016	-0.05 (-0.68 to 13.26)	2014-2019	0.41 (-0.38 to 10.15)	2010-2021	-2.25 (-2.74 to -1.85)	2003-2006	-0.27 (-1.42 to 0.45)
	3	2011-2014	8.30 (2.25 to 8.74)	2014-2021	2.11 (1.80 to 2.40)	2016-2021	-2.41 (-6.35 to -0.79)	2019-2021	3.29 (1.19 to 4.42)			2006-2009	5.39 (4.30 to 6.01)
	4	2014-2019	1.08 (0.61 to 8.05)									2009-2015	2.69 (2.25 to 3.01)
	5	2019-2021	2.71 (1.34 to 3.37)									2015-2019	-1.74 (-2.61 to -1.10)
	6											2019-2021	1.98 (0.31 to 3.12)
Uganda	0	1990-1999	0.78 (0.74 to 0.81)	1990-1999	1.73 (1.70 to 1.76)	1990-2001	2.81 (2.72 to 2.90)	1990-1999	1.74 (1.71 to 1.77)	1990-2000	3.21 (3.09 to 3.35)	1990-2000	2.94 (2.83 to 3.07)
	1	1999-2003	0.16 (0.06 to 0.76)	1999-2006	0.73 (0.69 to 0.77)	2001-2004	-0.70 (-1.13 to -0.36)	1999-2006	0.69 (0.66 to 0.73)	2000-2004	-0.28 (-0.60 to 0.12)	2000-2003	0.28 (-0.27 to 1.28)
	2	2003-2006	0.38 (0.26 to 0.48)	2006-2009	2.72 (2.58 to 2.82)	2004-2008	-2.79 (-3.28 to -2.48)	2006-2009	2.82 (2.68 to 2.91)	2004-2008	-3.69 (-4.27 to -3.23)	2003-2008	-2.44 (-2.92 to -2.11)
	3	2006-2010	1.78 (1.66 to 1.85)	2009-2014	2.12 (2.01 to 2.19)	2008-2012	-0.97 (-1.54 to -0.41)	2009-2014	2.20 (2.11 to 2.26)	2008-2012	-1.27 (-2.06 to -0.55)	2008-2011	-0.93 (-1.93 to 0.08)
	4	2010-2015	1.07 (1.00 to 1.13)	2014-2019	1.35 (1.24 to 1.42)	2012-2021	0.29 (0.17 to 0.45)	2014-2019	1.22 (1.11 to 1.28)	2012-2021	0.18 (0.02 to 0.39)	2011-2021	0.40 (0.29 to 0.58)
	5	2015-2019	0.32 (0.24 to 0.39)	2019-2021	2.27 (1.97 to 2.47)			2019-2021	1.82 (1.56 to 2.00)				
	6	2019-2021	1.40 (1.20 to 1.56)										
Ukraine	0	1990-1995	1.73 (1.61 to 1.85)	1990-1995	2.24 (2.10 to 2.38)	1990-1995	8.14 (6.61 to 10.09)	1990-1995	2.10 (1.88 to 2.26)	1990-1995	9.14 (7.66 to 10.94)	1990-1995	4.89 (4.45 to 5.40)
	1	1995-1999	3.35 (3.21 to 3.51)	1995-2000	3.40 (3.28 to 3.59)	1995-1998	-10.02 (-11.72 to -5.93)	1995-1999	3.64 (3.43 to 4.00)	1995-1998	-9.66 (-11.24 to -6.23)	1995-1998	-1.65 (-2.26 to -0.59)
	2	1999-2002	1.96 (1.68 to 2.23)	2000-2004	1.69 (1.52 to 1.95)	1998-2007	-2.26 (-2.90 to 0.65)	1999-2003	2.03 (1.79 to 2.29)	1998-2007	-2.24 (-2.87 to -0.42)	1998-2005	0.62 (0.27 to 1.95)
	3	2002-2010	1.00 (0.92 to 1.06)	2004-2010	0.90 (0.72 to 1.01)	2007-2014	-5.42 (-7.98 to -4.46)	2003-2010	1.03 (0.84 to 1.13)	2007-2014	-6.17 (-7.85 to -5.31)	2005-2013	-0.47 (-1.77 to -0.12)
	4	2010-2018	2.06 (2.00 to 2.18)	2010-2021	1.82 (1.77 to 1.87)	2014-2021	6.08 (3.23 to 8.82)	2010-2021	1.90 (1.84 to 1.97)	2014-2021	4.87 (2.29 to 7.09)	2013-2021	2.27 (1.82 to 2.85)
	5	2018-2021	1.61 (1.21 to 1.85)										
United Arab Emirates	0	1990-1999	3.22 (3.16 to 3.30)	1990-1999	3.78 (3.69 to 3.89)	1990-1995	-4.89 (-14.24 to -0.78)	1990-1993	4.47 (3.94 to 5.22)	1990-1995	-3.57 (-9.59 to -0.87)	1990-1995	-0.96 (-5.07 to 0.76)
	1	1999-2005	0.28 (0.16 to 0.38)	1999-2005	1.09 (0.95 to 2.98)	1995-2003	5.16 (1.62 to 9.06)	1993-1999	2.93 (2.53 to 3.11)	1995-2003	4.18 (1.70 to 6.33)	1995-2003	3.30 (1.84 to 4.87)
	2	2005-2010	-1.25 (-1.41 to -1.03)	2005-2009	-0.37 (-0.73 to 1.02)	2003-2008	14.37 (10.50 to 20.60)	1999-2019	0.77 (0.66 to 0.82)	2003-2008	10.63 (7.98 to 14.46)	2003-2008	7.49 (5.59 to 10.35)
	3	2010-2015	0.78 (0.60 to 1.08)	2009-2019	0.83 (-0.31 to 0.92)	2008-2012	-6.98 (-12.85 to -2.22)	2019-2021	1.70 (0.80 to 2.12)	2008-2012	-7.23 (-10.59 to -4.15)	2008-2012	-4.98 (-7.75 to -2.73)
	4	2015-2018	-0.38 (-0.70 to 0.09)	2019-2021	1.89 (0.93 to 2.32)	2012-2016	17.68 (11.67 to 25.05)			2012-2015	17.66 (12.16 to 20.72)	2012-2015	12.93 (9.20 to 15.09)
	5	2018-2021	1.15 (0.68 to 2.00)			2016-2021	-6.19 (-9.93 to -2.93)			2015-2019	-0.16 (-2.38 to 2.74)	2015-2019	0.06 (-1.52 to 2.02)
	6									2019-2021	-14.31 (-19.05 to -8.65)	2019-2021	-10.50 (-14.07 to -6.34)
United Kingdom	0	1990-1995	0.69 (-0.03 to 1.13)	1990-1995	0.81 (-0.43 to 1.34)	1990-1995	-7.25 (-8.52 to -6.23)	1990-1995	0.64 (0.22 to 1.08)	1990-1994	-7.71 (-9.31 to -6.54)	1990-1995	-2.54 (-2.98 to -2.09)
	1	1995-2004	2.80 (2.65 to 3.10)	1995-1999	4.80 (2.26 to 5.87)	1995-2003	-0.99 (-1.59 to 0.11)	1995-1999	7.58 (7.09 to 8.01)	1994-2003	-2.14 (-2.58 to -1.38)	1995-2000	3.79 (3.16 to 4.66)
	2	2004-2015	1.89 (1.77 to 1.99)	1999-2004	3.76 (1.78 to 4.26)	2003-2012	-4.33 (-5.59 to -3.65)	1999-2015	2.02 (1.92 to 2.11)	2003-2012	-4.70 (-5.83 to -4.09)	2000-2013	0.38 (0.14 to 0.57)
	3	2015-2021	4.77 (4.53 to 4.99)	2004-2015	2.15 (1.69 to 5.55)	2012-2021	0.73 (-0.05 to 1.75)	2015-2021	5.53 (5.08 to 6.14)	2012-2021	0.45 (-0.26 to 1.29)	2013-2021	4.19 (3.73 to 4.77)
	4			2015-2021	5.54 (4.90 to 6.39)								
United Republic of Tanzania	0	1990-1998	0.79 (0.77 to 0.81)	1990-1994	2.23 (2.05 to 2.51)	1990-1998	1.15 (1.00 to 1.33)	1990-1994	2.18 (2.05 to 2.32)	1990-1998	1.08 (0.92 to 1.26)	1990-1998	1.18 (1.05 to 1.35)
	1	1998-2001	1.40 (1.34 to 1.46)	1994-2000	1.51 (1.40 to 1.60)	1998-2001	-1.99 (-2.35 to -1.25)	1994-1998	1.51 (1.32 to 1.63)	1998-2001	-2.40 (-2.75 to -1.69)	1998-2001	-1.64 (-1.96 to -0.97)
	2	2001-2004	2.50 (2.40 to 2.57)	2000-2005	4.04 (3.95 to 4.13)	2001-2008	0.64 (0.45 to 1.16)	1998-2001	2.25 (2.09 to 2.38)	2001-2008	0.77 (0.59 to 1.08)	2001-2008	1.31 (1.13 to 1.61)
	3	2004-2009	2.00 (1.94 to 2.05)	2005-2014	2.63 (2.58 to 2.69)	2008-2021	0.08 (-0.07 to 0.17)	2001-2004	4.69 (4.55 to 4.84)	2008-2021	-0.12 (-0.24 to -0.03)	2008-2021	0.39 (0.29 to 0.48)
	4	2009-2015	1.58 (1.54 to 1.61)	2014-2021	2.18 (2.08 to 2.25)			2004-2007	3.23 (3.03 to 3.36)				
	5	2015-2019	0.98 (0.92 to 1.04)					2007-2014	2.53 (2.45 to 2.58)				
	6	2019-2021	1.70 (1.54 to 1.82)					2014-2021	1.99 (1.92 to 2.06)				
United States Virgin Islands	0	1990-1993	2.47 (2.39 to 2.58)	1990-1994	2.62 (2.46 to 2.88)	1990-1995	1.01 (0.10 to 2.82)	1990-1994	2.63 (2.48 to 2.82)	1990-1995	0.79 (0.02 to 2.00)	1990-1995	1.52 (1.24 to 1.80)
	1	1993-2001	1.46 (1.41 to 1.49)	1994-2005	1.99 (1.95 to 2.05)	1995-2003	-2.07 (-2.56 to -1.36)	1994-2007	1.91 (1.88 to 1.95)	1995-2003	-2.20 (-2.63 to -1.76)	1995-2004	-0.24 (-0.34 to -0.06)
	2	2001-2005	1.69 (1.59 to 1.79)	2005-2010	1.68 (1.46 to 1.84)	2003-2007	-4.78 (-6.22 to -3.39)	2007-2011	1.49 (1.30 to 1.83)	2003-2007	-5.28 (-6.63 to -4.28)	2004-2007	-1.36 (-1.62 to -0.74)
	3	2005-2010	1.35 (1.25 to 1.41)	2010-2019	1.21 (1.16 to 1.26)	2007-2019	-1.64 (-1.96 to -0.97)	2011-2019	1.17 (1.07 to 1.22)	2007-2019	-1.42 (-1.67 to -1.06)	2007-2019	0.34 (0.25 to 0.46)
	4	2010-2019	0.95 (0.92 to 0.98)	2019-2021	-0.40 (-0.61 to -0.15)	2019-2021	-11.45 (-15.16 to -7.19)	2019-2021	-0.41 (-0.63 to -0.15)	2019-2021	-11.27 (-14.18 to -7.22)	2019-2021	-3.75 (-4.54 to -2.29)

Location	Segment	Incidence		Prevalence		Mortality		YLD		YLL		DALY	
		Period	APC (95%CI)	Period	APC (95%CI)	Period	APC (95%CI)	Period	APC (95%CI)	Period	APC (95%CI)	Period	APC (95%CI)
	5	2019-2021	-0.12 (-0.27 to 0.01)										
United States of America	0	1990-1995	1.60 (1.43 to 1.77)	1990-1995	2.02 (1.79 to 2.26)	1990-1992	1.34 (0.53 to 2.53)	1990-1995	2.37 (2.17 to 2.58)	1990-1996	2.47 (2.06 to 2.93)	1990-1992	1.58 (1.24 to 1.97)
	1	1995-2000	5.26 (5.10 to 5.42)	1995-2000	5.50 (5.32 to 5.72)	1992-1995	3.71 (2.76 to 4.19)	1995-2000	5.41 (5.24 to 5.63)	1996-2003	-0.36 (-0.74 to 0.04)	1992-1995	3.20 (2.91 to 3.41)
	2	2000-2004	3.27 (3.05 to 3.52)	2000-2004	3.44 (3.19 to 3.76)	1995-2002	0.60 (0.37 to 0.81)	2000-2004	3.49 (3.20 to 3.86)	2003-2008	-3.41 (-4.09 to -2.78)	1995-2002	2.15 (2.05 to 2.23)
	3	2004-2010	1.94 (1.82 to 2.06)	2004-2010	2.27 (2.07 to 2.40)	2002-2005	-1.84 (-3.20 to -0.77)	2004-2010	2.24 (1.80 to 2.41)	2008-2013	-6.73 (-7.23 to -6.25)	2002-2005	0.52 (0.18 to 0.93)
	4	2010-2015	3.20 (3.03 to 3.35)	2010-2015	3.46 (3.29 to 3.72)	2005-2009	-4.47 (-5.40 to -4.02)	2010-2015	3.36 (3.13 to 3.85)	2013-2021	-0.22 (-0.49 to 0.04)	2005-2012	-0.82 (-0.92 to -0.73)
	5	2015-2019	1.85 (1.58 to 2.10)	2015-2021	2.37 (2.23 to 2.50)	2009-2013	-7.14 (-7.54 to -6.58)	2015-2021	2.21 (2.01 to 2.38)			2012-2021	1.64 (1.56 to 1.72)
	6	2019-2021	2.72 (2.26 to 3.04)			2013-2021	-0.73 (-0.93 to -0.52)						
Uruguay	0	1990-1998	2.04 (1.71 to 2.23)	1990-1999	2.75 (2.49 to 2.93)	1990-2004	-0.98 (-2.25 to -0.70)	1990-1999	2.69 (2.45 to 2.87)	1990-2005	-1.35 (-2.13 to -1.00)	1990-1993	1.67 (0.95 to 2.92)
	1	1998-2004	3.42 (3.14 to 4.21)	1999-2004	4.51 (4.07 to 5.39)	2004-2021	-0.39 (-0.63 to 0.52)	1999-2004	4.38 (3.97 to 5.25)	2005-2021	-0.16 (-0.51 to 0.59)	1993-1996	-1.29 (-1.87 to -0.24)
	2	2004-2017	2.79 (2.67 to 2.90)	2004-2017	3.12 (3.02 to 3.23)			2004-2017	3.11 (3.00 to 3.21)			1996-2002	0.65 (0.08 to 1.40)
	3	2017-2021	1.69 (0.61 to 2.16)	2017-2021	1.60 (0.72 to 2.09)			2017-2021	1.60 (0.79 to 2.08)			2002-2021	1.40 (1.11 to 1.92)
Uzbekistan	0	1990-1996	3.62 (3.28 to 3.82)	1990-1996	4.15 (3.99 to 4.25)	1990-1994	16.09 (14.76 to 17.58)	1990-1996	4.09 (3.81 to 4.22)	1990-1994	16.53 (15.14 to 18.12)	1990-1994	11.97 (11.07 to 12.94)
	1	1996-2000	5.21 (4.07 to 5.67)	1996-2000	4.84 (4.64 to 5.12)	1994-1998	-1.58 (-3.42 to -0.23)	1996-2000	4.94 (4.58 to 5.45)	1994-1998	-1.43 (-3.27 to -0.03)	1994-1998	0.47 (-0.75 to 1.34)
	2	2000-2004	6.79 (5.45 to 7.17)	2000-2004	6.20 (6.07 to 6.37)	1998-2004	3.28 (1.71 to 5.01)	2000-2004	6.50 (6.32 to 6.84)	1998-2004	2.92 (1.36 to 5.05)	1998-2004	4.09 (3.07 to 4.91)
	3	2004-2007	3.66 (3.26 to 6.90)	2004-2007	4.33 (4.10 to 4.48)	2004-2007	12.39 (8.72 to 14.31)	2004-2007	4.29 (3.96 to 4.59)	2004-2007	11.33 (7.43 to 13.28)	2004-2007	8.78 (6.98 to 9.89)
	4	2007-2015	3.08 (1.65 to 3.21)	2007-2014	3.25 (3.16 to 3.31)	2007-2010	-2.74 (-4.36 to 0.15)	2007-2014	3.30 (3.19 to 3.37)	2007-2010	-3.03 (-4.62 to 0.10)	2007-2010	-0.93 (-2.03 to 0.66)
	5	2015-2021	1.25 (1.03 to 1.48)	2014-2021	1.54 (1.46 to 1.61)	2010-2016	3.33 (2.52 to 5.73)	2014-2021	1.51 (1.42 to 1.59)	2010-2016	3.36 (2.46 to 5.74)	2010-2015	3.62 (2.87 to 5.49)
	6					2016-2021	-2.70 (-4.63 to -1.32)			2016-2021	-2.98 (-5.02 to -1.51)	2015-2021	-0.61 (-1.55 to 0.06)
Vanuatu	0	1990-1996	2.04 (1.92 to 2.09)	1990-1994	2.86 (2.76 to 2.99)	1990-2000	1.44 (1.26 to 1.69)	1990-1994	2.82 (2.74 to 2.91)	1990-1999	1.55 (1.24 to 1.92)	1990-1999	1.74 (1.52 to 2.00)
	1	1996-2001	2.17 (2.13 to 2.73)	1994-2000	2.51 (2.45 to 2.55)	2000-2003	-1.43 (-1.98 to -0.19)	1994-2000	2.47 (2.38 to 2.52)	1999-2003	-1.64 (-2.80 to -0.43)	1999-2003	-0.76 (-1.68 to -0.02)
	2	2001-2010	2.81 (2.78 to 2.83)	2000-2010	3.26 (3.23 to 3.28)	2003-2006	1.88 (0.96 to 2.43)	2000-2003	3.15 (2.60 to 3.26)	2003-2007	1.85 (0.98 to 2.80)	2003-2007	2.22 (1.61 to 2.93)
	3	2010-2019	2.24 (2.22 to 2.26)	2010-2019	2.84 (2.81 to 2.87)	2006-2021	0.84 (0.68 to 0.92)	2003-2009	3.35 (3.26 to 3.44)	2007-2021	0.89 (0.58 to 1.00)	2007-2021	1.39 (1.23 to 1.47)
	4	2019-2021	2.65 (2.44 to 2.75)	2019-2021	3.33 (3.11 to 3.45)			2009-2019	2.83 (2.79 to 2.86)				
	5							2019-2021	3.21 (2.99 to 3.32)				
Venezuela (Bolivarian Republic of)	0	1990-1993	2.06 (1.49 to 2.93)	1990-1993	2.25 (1.56 to 3.19)	1990-1994	0.76 (-0.51 to 3.18)	1990-1994	2.09 (1.57 to 3.09)	1990-1994	1.17 (0.02 to 3.38)	1990-1994	1.51 (0.90 to 2.59)
	1	1993-2002	0.59 (0.36 to 0.73)	1993-2002	0.93 (0.56 to 1.08)	1994-1997	-5.50 (-7.11 to -3.09)	1994-2002	0.80 (0.32 to 0.99)	1994-1997	-6.34 (-7.77 to -4.04)	1994-1997	-3.92 (-4.84 to -2.42)
	2	2002-2013	1.85 (1.76 to 1.97)	2002-2012	2.35 (2.23 to 2.52)	1997-2002	0.44 (-0.80 to 3.57)	2002-2012	2.33 (2.18 to 2.55)	1997-2002	0.00 (-0.93 to 2.69)	1997-2002	0.31 (-0.50 to 2.22)
	3	2013-2019	0.79 (0.37 to 0.98)	2012-2019	1.24 (0.88 to 1.41)	2002-2006	-4.41 (-6.93 to -2.14)	2012-2019	1.16 (0.72 to 1.34)	2002-2006	-4.59 (-7.01 to -3.02)	2002-2006	-1.57 (-3.03 to 3.07)
	4	2019-2021	4.12 (2.85 to 4.97)	2019-2021	4.36 (3.09 to 5.35)	2006-2021	2.98 (2.49 to 3.45)	2019-2021	4.10 (2.97 to 5.05)	2006-2021	2.84 (2.43 to 3.27)	2006-2021	2.35 (1.80 to 2.72)
Viet Nam	0	1990-2000	1.01 (0.88 to 1.09)	1990-1998	1.27 (1.02 to 1.42)	1990-1995	0.01 (-0.14 to 0.22)	1990-1999	1.35 (1.20 to 1.45)	1990-1994	-0.47 (-0.64 to -0.16)	1990-1994	-0.06 (-0.15 to 0.13)
	1	2000-2004	2.64 (1.00 to 2.95)	1998-2012	2.04 (1.95 to 2.11)	1995-2000	-0.50 (-0.63 to -0.01)	1999-2005	2.37 (2.12 to 2.82)	1994-2000	-0.90 (-1.01 to -0.83)	1994-2000	-0.28 (-0.35 to -0.24)
	2	2004-2011	1.73 (1.39 to 2.64)	2012-2019	2.69 (2.35 to 2.90)	2000-2006	1.19 (-0.53 to 1.29)	2005-2011	1.92 (1.45 to 2.13)	2000-2006	1.09 (1.02 to 1.18)	2000-2005	1.51 (1.43 to 1.57)
	3	2011-2019	2.14 (1.71 to 2.27)	2019-2021	4.18 (3.24 to 4.61)	2006-2013	0.68 (0.56 to 1.25)	2011-2019	2.65 (2.40 to 2.83)	2006-2013	0.56 (0.42 to 0.62)	2005-2013	1.03 (0.98 to 1.07)
	4	2019-2021	2.88 (2.22 to 3.20)			2013-2016	1.17 (0.60 to 1.28)	2019-2021	4.03 (3.16 to 4.42)	2013-2017	0.92 (0.76 to 1.11)	2013-2016	1.57 (1.40 to 1.65)
	5					2016-2019	0.56 (0.43 to 1.20)			2017-2021	0.08 (-0.15 to 0.24)	2016-2021	1.18 (1.06 to 1.24)
	6					2019-2021	-0.13 (-0.42 to 0.27)						
Yemen	0	1990-1992	2.75 (2.04 to 3.35)	1990-1995	2.70 (2.22 to 3.31)	1990-2001	-0.07 (-0.15 to 0.01)	1990-1995	2.72 (2.42 to 3.07)	1990-2001	-0.16 (-0.39 to 0.06)	1990-1994	1.43 (1.12 to 1.85)
	1	1992-2001	1.97 (1.71 to 2.91)	1995-2000	0.33 (-0.38 to 0.78)	2001-2004	3.27 (0.10 to 3.52)	1995-2000	0.74 (0.35 to 1.03)	2001-2004	3.31 (-0.25 to 3.58)	1994-2000	0.23 (0.00 to 0.39)
	2	2001-2019	2.80 (2.76 to 2.85)	2000-2019	2.97 (2.88 to 3.01)	2004-2008	1.26 (0.97 to 2.88)	2000-2021	3.00 (2.96 to 3.03)	2004-2008	1.18 (0.86 to 3.39)	2000-2006	2.56 (2.39 to 2.78)
	3	2019-2021	-0.89 (-1.53 to -0.48)	2019-2021	4.76 (3.04 to 5.60)	2008-2017	0.73 (0.34 to 0.83)			2008-2012	0.11 (-0.30 to 1.26)	2006-2017	1.76 (1.64 to 1.84)
	4					2017-2021	1.88 (1.48 to 2.51)			2012-2018	0.79 (0.30 to 1.23)	2017-2021	2.63 (2.29 to 3.27)
	5									2018-2021	2.09 (1.40 to 2.93)		
Zambia	0	1990-1995	0.71 (0.37 to 0.87)	1990-2001	1.94 (1.78 to 2.10)	1990-1995	2.51 (2.31 to 2.72)	1990-2001	1.92 (1.84 to 2.02)	1990-1995	2.68 (2.45 to 2.91)	1990-1995	2.49 (2.33 to 2.64)
	1	1995-2000	1.42 (1.24 to 1.79)	2001-2005	1.43 (1.07 to 2.08)	1995-1999	-0.56 (-0.79 to -0.30)	2001-2007	1.52 (1.14 to 1.73)	1995-1999	-0.89 (-1.17 to -0.59)	1995-1999	-0.28 (-0.45 to -0.09)
	2	2000-2009	0.73 (0.48 to 0.79)	2005-2014	1.88 (1.72 to 2.38)	1999-2005	-3.13 (-3.38 to -2.96)	2007-2015	1.86 (1.72 to 2.25)	1999-2005	-3.51 (-3.80 to -3.30)	1999-2005	-2.29 (-2.43 to -2.15)
	3	2009-2015	1.05 (0.92 to 1.37)	2014-2021	1.44 (1.13 to 1.61)	2005-2008	-0.31 (-2.60 to 0.01)	2015-2021	1.19 (0.95 to 1.36)	2005-2008	-0.40 (-3.03 to -0.09)	2005-2008	0.22 (-1.79 to 0.44)
	4	2015-2021	0.52 (0.32 to 0.63)			2008-2011	1.20 (0.71 to 1.54)			2008-2011	0.91 (0.34 to 1.25)	2008-2011	1.17 (0.79 to 1.41)
	5					2011-2016	-0.59 (-1.07 to -0.37)			2011-2016	-1.06 (-1.59 to -0.83)	2011-2016	-0.19 (-0.53 to -0.03)
	6					2016-2021	0.20 (-0.08 to 0.89)			2016-2021	-0.08 (-0.40 to 0.68)	2016-2021	0.32 (0.12 to 0.80)
Zimbabwe	0	1990-1996	1.52 (1.40 to 1.59)	1990-1997	2.12 (1.99 to 2.19)	1990-1994	-3.20 (-4.49 to -2.62)	1990-1997	2.09 (1.97 to 2.15)	1990-1994	-3.84 (-4.89 to -3.31)	1990-1994	-2.63 (-4.11 to -1.99)
	1	1996-2001	1.87 (1.72 to 2.03)	1997-2002	2.58 (2.40 to 2.79)	1994-1997	0.34 (-0.89 to 1.22)	1997-2001	2.51 (2.19 to 2.72)	1994-1997	-0.17 (-1.37 to 0.69)	1994-1997	0.32 (-0.78 to 1.19)
	2	2001-2006	2.44 (2.21 to 2.56)	2002-2010	3.11 (3.05 to 3.21)	1997-2000	18.20 (17.33 to 19.29)	2001-2006	2.99 (2.75 to 3.12)	1997-2000	20.16 (19.26 to 21.22)	1997-2000	16.34 (15.20 to 17.43)
	3	2006-2010	2.82 (2.64 to 3.03)	2010-2014	4.29 (4.17 to 4.44)	2000-2004	8.46 (7.96 to 8.94)	2006-2010	3.34 (3.21 to 3.58)	2000-2004	9.63 (9.13 to 10.12)	2000-2004	8.66 (8.03 to 9.14)
	4	2010-2014	3.35 (3.25 to 3.50)	2014-2017	2.39 (2.21 to 2.55)	2004-2009	1.70 (1.37 to 2.10)	2010-2014	4.36 (4.25 to 4.50)	2004-2009	1.94 (1.61 to 2.31)	2004-2009	2.08 (1.71 to 2.54)
	5	2014-2018	1.46 (1.38 to 1.55)	2017-2021	1.07 (0.92 to 1.18)	2009-2012	-2.30 (-2.71 to -1.32)	2014-2017	2.43 (2.30 to 2.56)	2009-2012	-2.85 (-3.27 to -1.98)	2009-2012	-1.65 (-2.06 to -0.58)
	6	2018-2021	0.33 (0.21 to 0.45)			2012-2021	-0.49 (-0.68 to -0.14)	2017-2021	0.88 (0.78 to 0.99)	2012-2021	-0.57 (-0.75 to -0.30)	2012-2021	-0.06 (-0.26 to 0.53)

APC: annual percentage change; CI: confidence interval; DALY: Disability adjusted life years; SDI: Sociodemographic index; YLD: Years lived with disability; YLL: Years of life lost.

Table S9. DALY counts, YLD counts, and proportions of DALY due to YLD for type 1 and type 2 diabetes among women and men at global and SDI levels, forecasted from 2022 to 2050.

	**Forecasted all-age counts (000s) in 2022**			**Forecasted all-age counts (000s) in 2050**			**Relative increase in proportions from 2022 to 2050**
	DALY	YLD	Proportion of DALY due to YLD, %	DALY	YLD	Proportion of DALY due to YLD, %	
**Type 1 diabetes, women**							
Global	1688 (1399 to 1986)	699 (452 to 1010)	41.42	1623 (1172 to 2147)	1104 (733 to 1577)	68.06	64.29
High SDI	326 (252 to 416)	224 (149 to 314)	68.86	307 (217 to 411)	269 (179 to 372)	87.79	27.50
High-middle SDI	178 (141 to 223)	107 (70 to 154)	60.05	158 (113 to 211)	134 (90 to 190)	84.64	40.94
Middle SDI	468 (388 to 559)	172 (109 to 254)	36.70	384 (283 to 515)	263 (171 to 380)	68.46	86.56
Low-middle SDI	515 (417 to 615)	149 (94 to 221)	28.90	508 (360 to 684)	301 (194 to 439)	59.11	104.56
Low SDI	200 (156 to 249)	47 (31 to 68)	23.62	263 (183 to 376)	137 (93 to 197)	51.85	119.53
**Type 1 diabetes, men**							
Global	1919 (1602 to 2263)	691 (446 to 990)	35.99	2115 (1531 to 2741)	1119 (741 to 1618)	52.91	47.01
High SDI	408 (332 to 498)	224 (149 to 313)	54.86	379 (281 to 488)	276 (183 to 383)	72.76	32.63
High-middle SDI	213 (176 to 263)	110 (72 to 159)	51.70	191 (143 to 254)	138 (92 to 196)	72.19	39.63
Middle SDI	457 (387 to 543)	159 (103 to 234)	34.85	432 (322 to 564)	244 (158 to 356)	56.37	61.75
Low-middle SDI	539 (421 to 653)	139 (88 to 208)	25.80	654 (448 to 894)	291 (188 to 425)	44.51	72.51
Low SDI	301 (235 to 367)	58 (39 to 84)	19.32	457 (307 to 649)	169 (113 to 244)	37.11	92.08
**Type 2 diabetes, women**							
Global	37985 (31556 to 45691)	20174 (14116 to 28001)	53.11	75112 (57667 to 95925)	52111 (36517 to 71804)	69.38	30.63
High SDI	5834 (4681 to 7369)	3884 (2740 to 5340)	66.59	8768 (6649 to 11376)	6908 (4899 to 9517)	78.79	18.32
High-middle SDI	6016 (4704 to 7697)	4176 (2933 to 5798)	69.41	10339 (7713 to 13475)	8413 (5889 to 11708)	81.37	17.23
Middle SDI	14016 (11841 to 16692)	6870 (4776 to 9525)	49.02	26522 (20750 to 33495)	18016 (12591 to 24742)	67.93	38.58
Low-middle SDI	9490 (8010 to 11193)	4208 (2896 to 5888)	44.35	22487 (17251 to 28481)	14649 (10343 to 20500)	65.14	46.90
Low SDI	2594 (2158 to 3037)	1019 (688 to 1429)	39.27	6926 (5320 to 8711)	4083 (2815 to 5687)	58.95	50.09
**Type 2 diabetes, men**							
Global	39059 (32468 to 47357)	21029 (14666 to 29414)	53.84	80831 (64513 to 101999)	51215 (35745 to 70917)	63.36	17.68
High SDI	6551 (5249 to 8273)	4290 (2988 to 5972)	65.49	10541 (8164 to 13634)	7810 (5463 to 10894)	74.09	13.14
High-middle SDI	6397 (4973 to 8107)	4462 (3113 to 6221)	69.75	11322 (8664 to 14644)	8705 (6075 to 12191)	76.89	10.24
Middle SDI	13651 (11561 to 16368)	6728 (4667 to 9417)	49.29	26654 (21711 to 33369)	16179 (11165 to 22370)	60.70	23.16
Low-middle SDI	9586 (8027 to 11465)	4480 (3119 to 6278)	46.73	24137 (19275 to 29894)	14269 (9884 to 20222)	59.11	26.49
Low SDI	2841 (2405 to 3335)	1054 (713 to 1482)	37.11	8108 (6506 to 9807)	4213 (2905 to 5851)	51.96	40.01

DALYs: Disability adjusted life years; YLD: Years lived with disability; SDI: Sociodemographic index.
