# Supplementary material for: Global burden of diabetes in women from 1990 to 2021, with projections to 2050: population-based study
Source: BMC Med. 2025 Oct 8;23:538. doi: 10.1186/s12916-025-04361-y (PMC12506318; doi:10.1186/s12916-025-04361-y)
Supplement: Supplementary file 3 — Additional file 3: Fig S1. Temporal trends in total and type-specific diabetes prevalence, YLD, and YLL among women at global and SDI level, 1990-2021. Fig S2. Comparison of the global diabetes burden across the full spectrum of diseases between women and men, 1990-2021. Fig S3-S4. Ranking of type 1 diabetes burden across super-regions, sub-regions, and High-income countries and territories, 1990-2021. Fig S5-S6. Maps showing global burden of type 1 diabetes in women and corresponding female-to-male ratios or differences, 1990-2021. Fig S7. Ranking of type 2 diabetes burden across super-regions and sub-regions, 1990-2021. Fig S8-S9. Maps showing global burden of type 2 diabetes in women and corresponding female-to-male ratios or differences, 1990-2021. Fig S10. The age-specific patterns of diabetes burden among women in 1990, 2005, and 2021, along with population structures. Fig S11-S14. Female-to-male ratios of type 1 diabetes burden by age groups, 1990-2021. Fig S15. Comparison of the age-specific type 2 diabetes incidence patterns between men and women in 1990, 2005, and 2021. Fig S16. Correlations between age and AAPCs in the burden of type 1 and type 2 diabetes among women, at global and SDI levels. Fig S17-S20. Female-to-male ratios of type 2 diabetes burden by age groups, 1990-2021. Fig S21. Contributions of detailed risk factors to diabetes DALY among women in 2021 and corresponding AAPCs from 1990 to 2021. Fig S22. Trends of contributions of specific risk factors to diabetes DALY among women at global and SDI levels, 1990-2021. Fig S23. Age-specific differences between women and men in proportional DALY attributable to specific risk factors for diabetes in 2021, globally and by SDI. Fig S24. Age-related characteristics in terms of proportional DALY attributable to specific risk factors for diabetes among women in 2021, globally and by SDI. Fig S25. Diabetes DALY, YLD, and YLL among women globally, for the past and for five future scenarios, 1990-2050. Fig S [file 12916_2025_4361_MOESM3_ESM.pdf]

Fig S1. Temporal trends in age-standardized **prevalence**, **YLD**, and **YLL** of total and type-specific diabetes among women at global and SDI level, 1990-2021

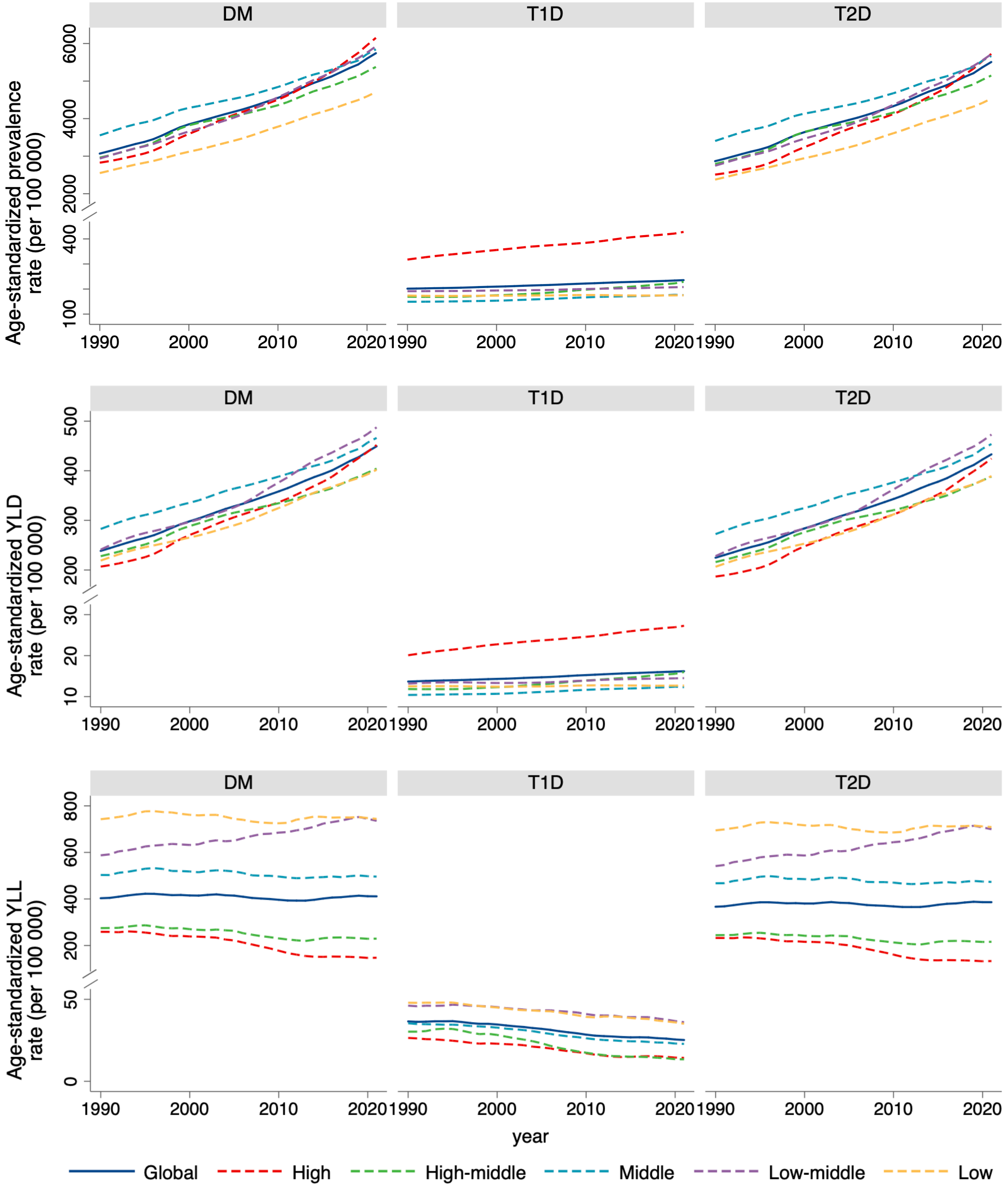

DM: Diabetes mellitus; T1D: Type 1 diabetes; T2D: Type 2 diabetes; YLD: Years lived with disability; YLL: Years of life lost.

Fig S2. Comparison of the global burden of diabetes across the full spectrum of diseases between women and men, 1990-2021

(A) The disease burden (all cause DALY) from diabetes among women and men

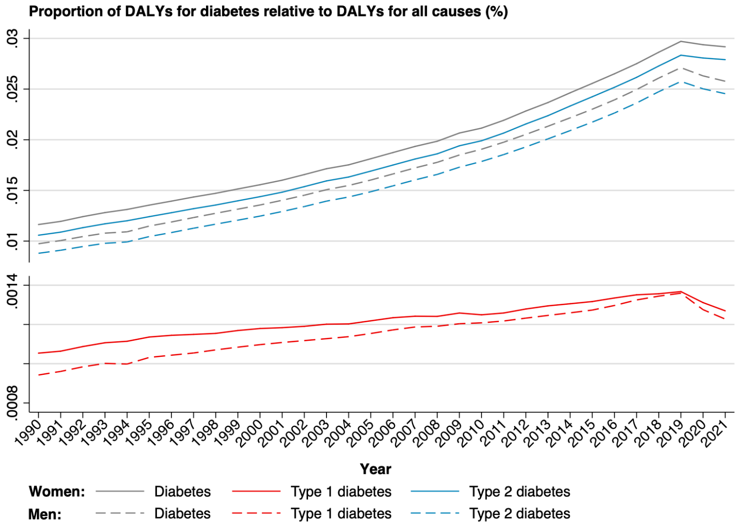

(B) Ranking of diabetes burden across the disease spectrum for women and men, respectively

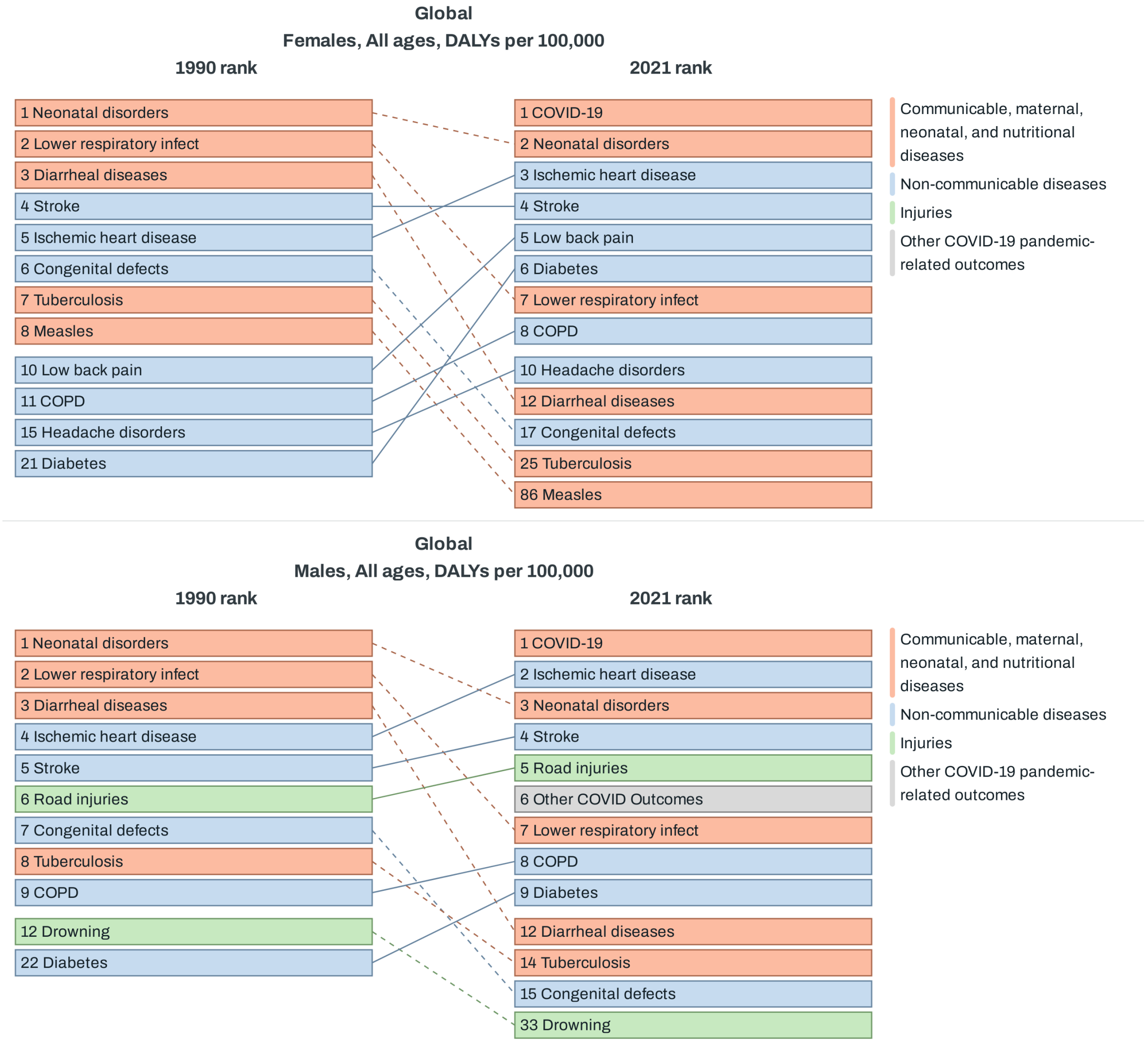

The bottom part was generated and extracted from https://vizhub.healthdata.org/gbd-compare/.
Similar findings on higher ranks of diabetes among females than males were noted for all-age DALY number (ranked among females vs males: 21st vs 22nd in 1990 and 6th vs 9th in 2021) and age-standardized DALY rate (ranked among females vs males: 19th vs 20th in 1990 and 7th vs 9th in 2021).

Fig S3. Proportion relative to all causes (%) and age-standardized rate (per 100 000) of **type 1 diabetes** incidence, DALY, and mortality in 2021, as well as corresponding average annual percentage change (%) in age-standardized rate from 1990 to 2021, across 7 super-regions and 21 sub-regions in women and men

Incidence

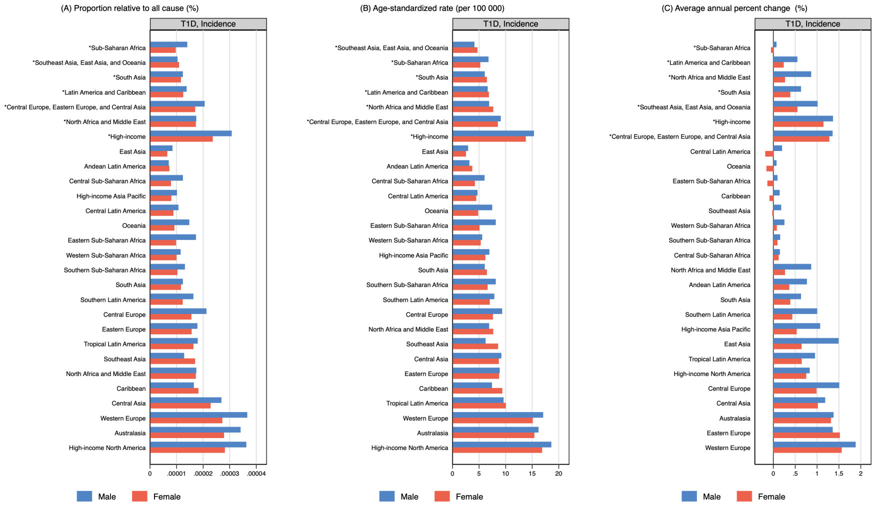

DALY

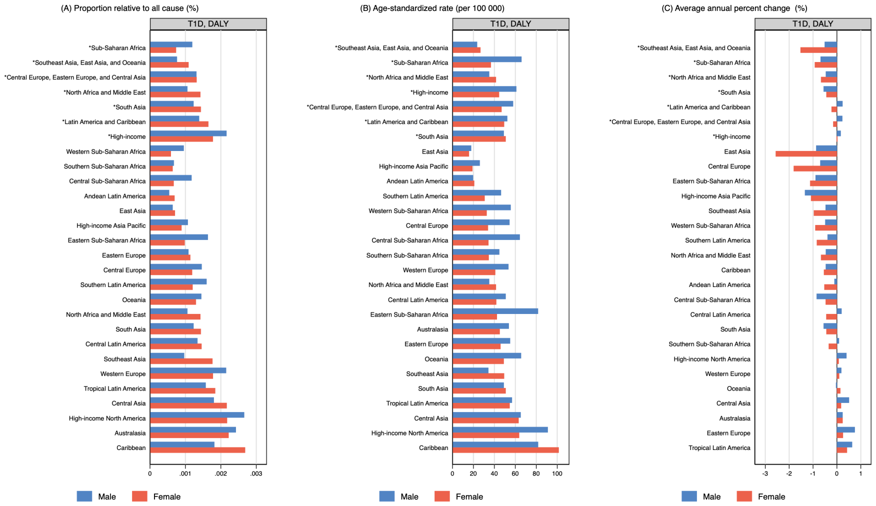

Mortality

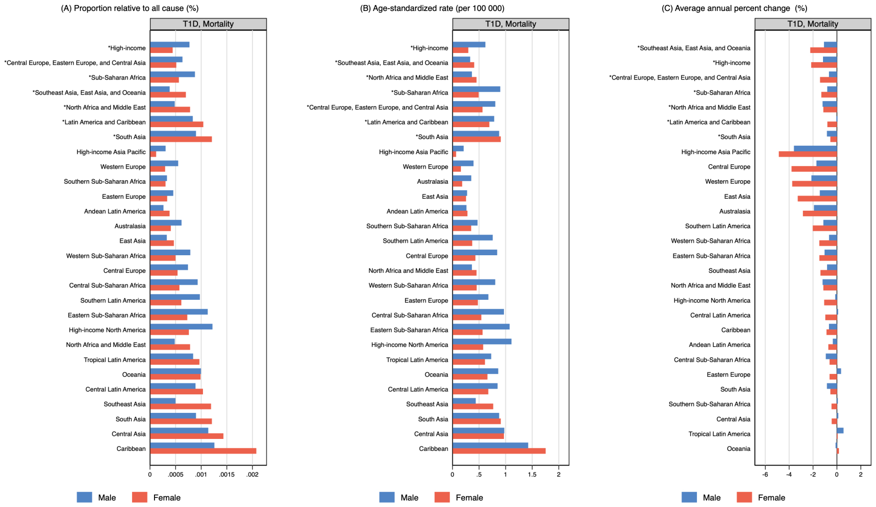

DALY: Disability adjusted life years; T1D: Type 1 diabetes. * Super-region.

Fig S4. Ranking of High-income countries and territories for **type 1 diabetes** incidence in 2021 (A), as well as AAPCs in incidence (B), mortality (C), and DALY (D) from 1990 to 2021, in women

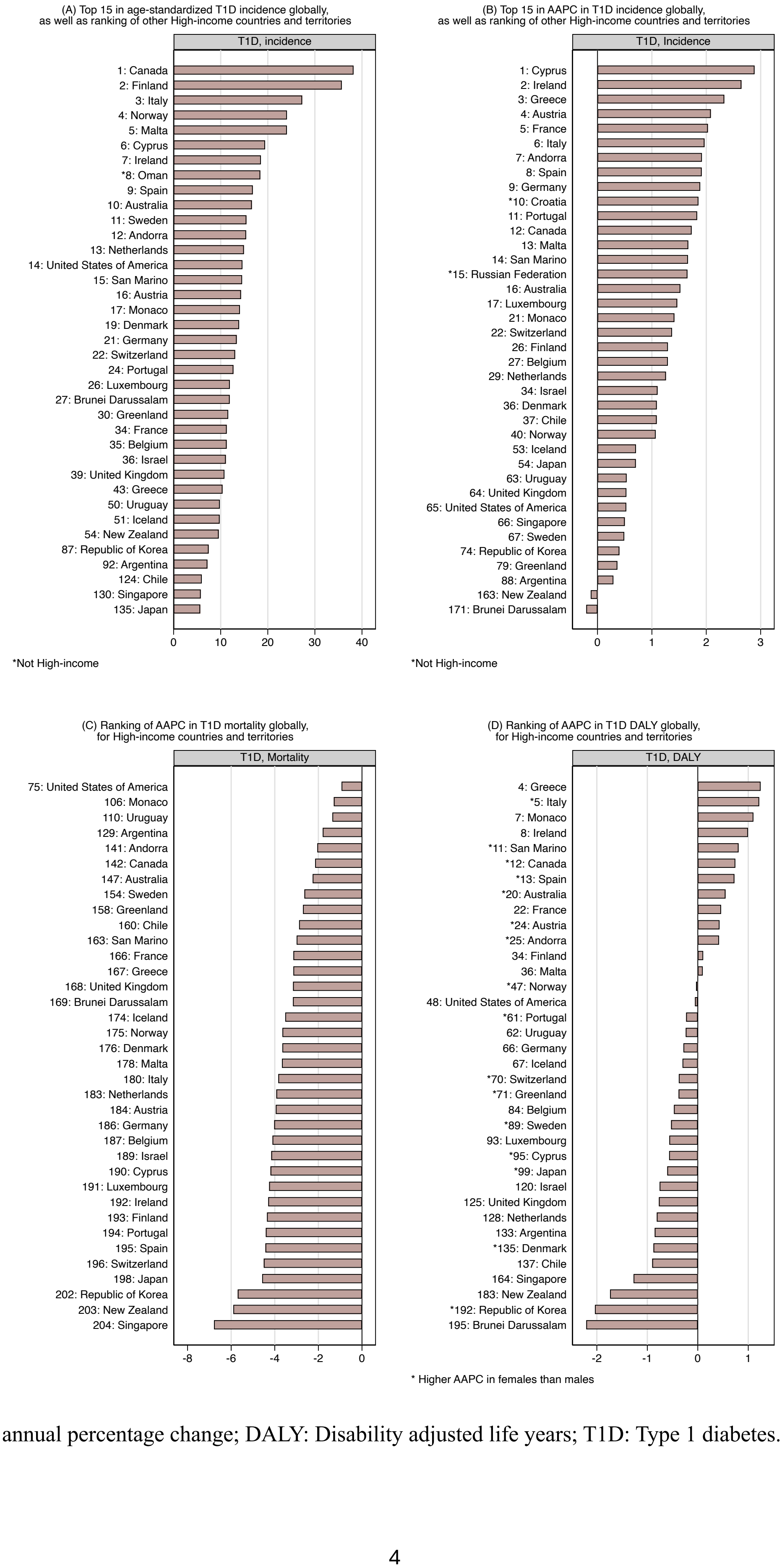

AAPC: average annual percentage change; DALY: Disability adjusted life years; T1D: Type 1 diabetes.

Fig S5. Maps showing global age-standardized **prevalence, mortality, YLD and YLL rate** (per 100, 000) in 2021, as well as corresponding AAPC from 1990 to 2021, of **type 1 diabetes** in women

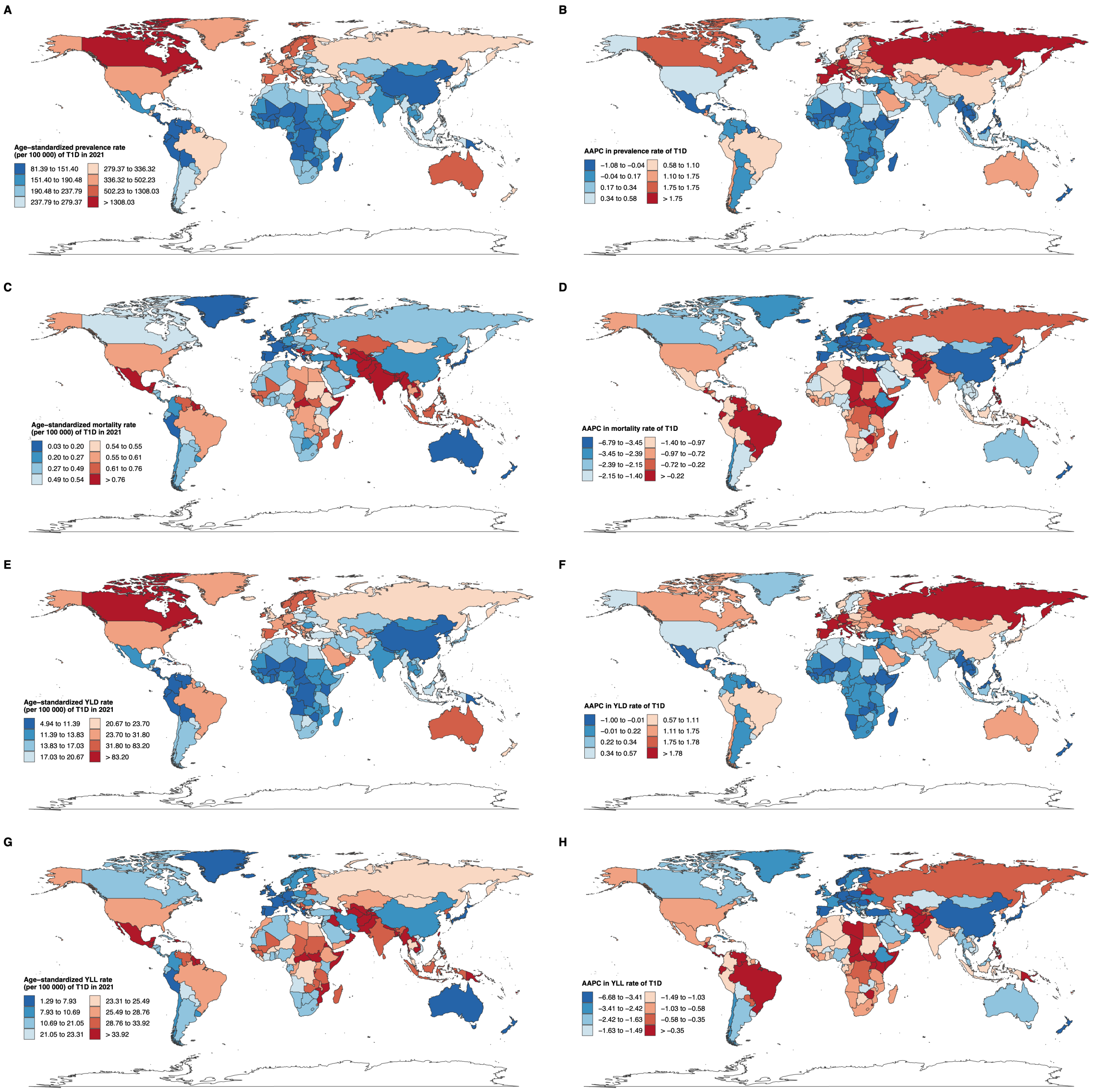

AAPC: average annual percentage change; T1D: Type 1 diabetes; YLD: Years lived with disability; YLL: Years of life lost.

Fig S6. Maps showing global **female-to-male ratio** of age-standardized incidence, prevalence, mortality and DALY rate in 2021, as well as **female-to-male difference** in corresponding AAPC from 1990 to 2021, of **type 1 diabetes**

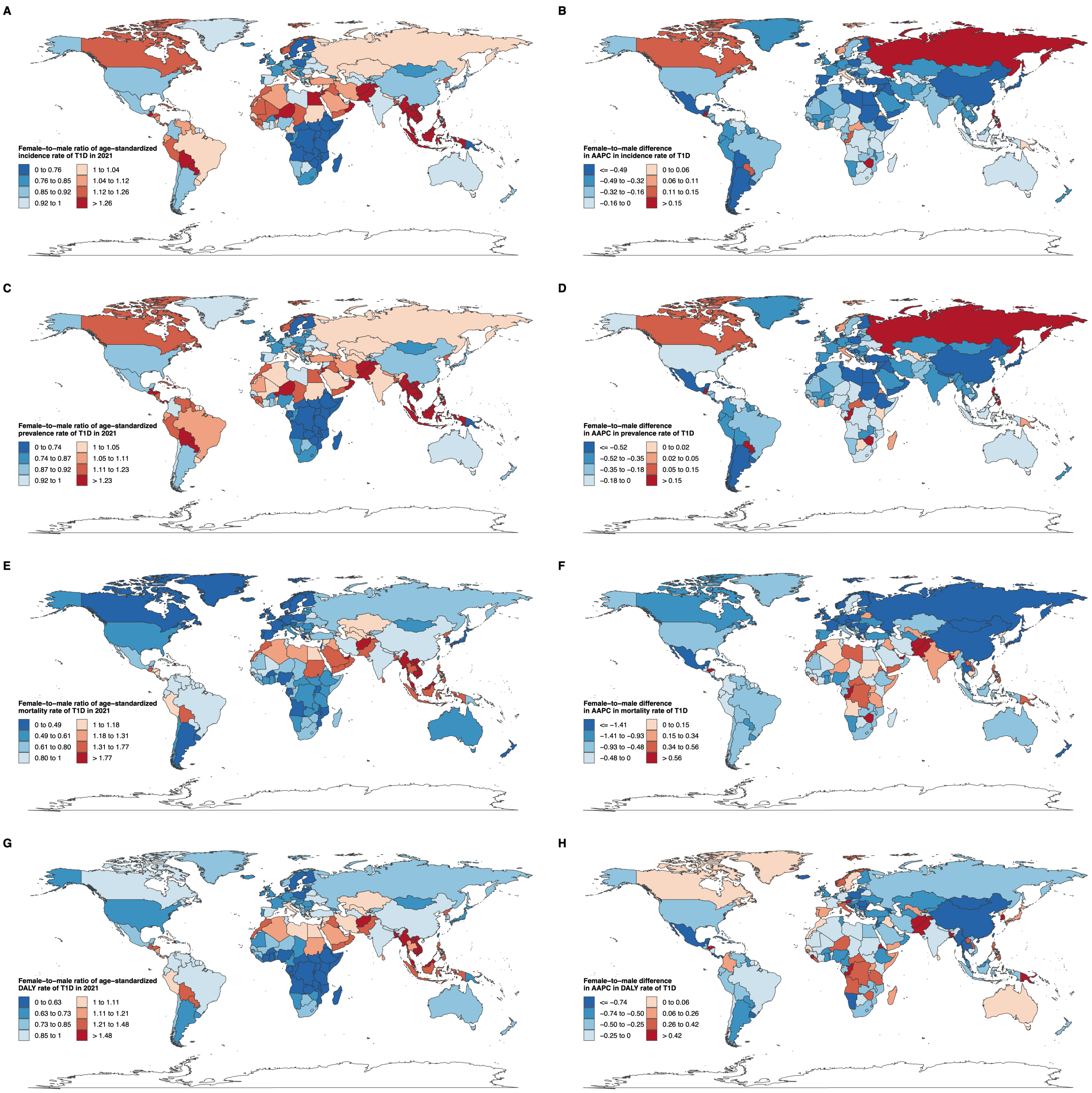

AAPC: Average annual percentage change; DALY: Disability adjusted life years; T1D: Type 1 diabetes.

Fig S7. Proportion relative to all causes (%) and age-standardized rate (per 100 000) of **type 2 diabetes** incidence, DALY, and mortality in 2021, as well as corresponding average annual percentage change (%) in age-standardized rate from 1990 to 2021, across 7 super-regions and 21 sub-regions in women and men

Incidence

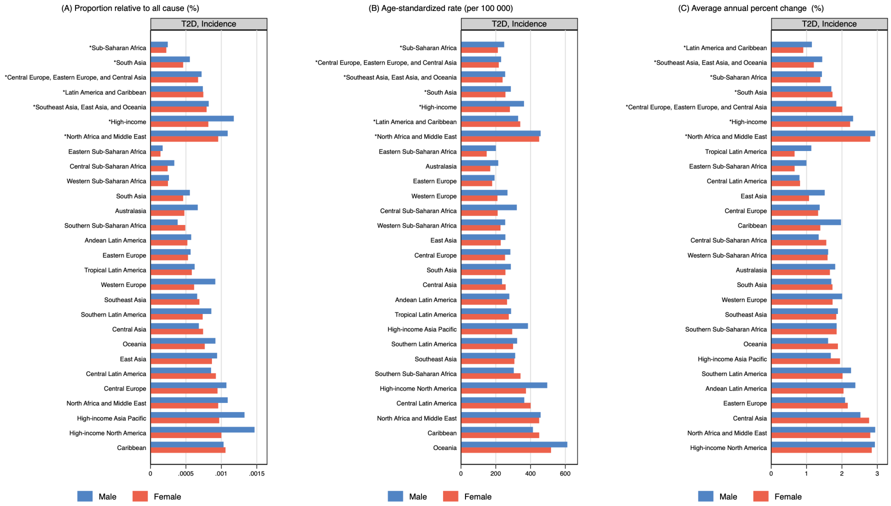

DALY

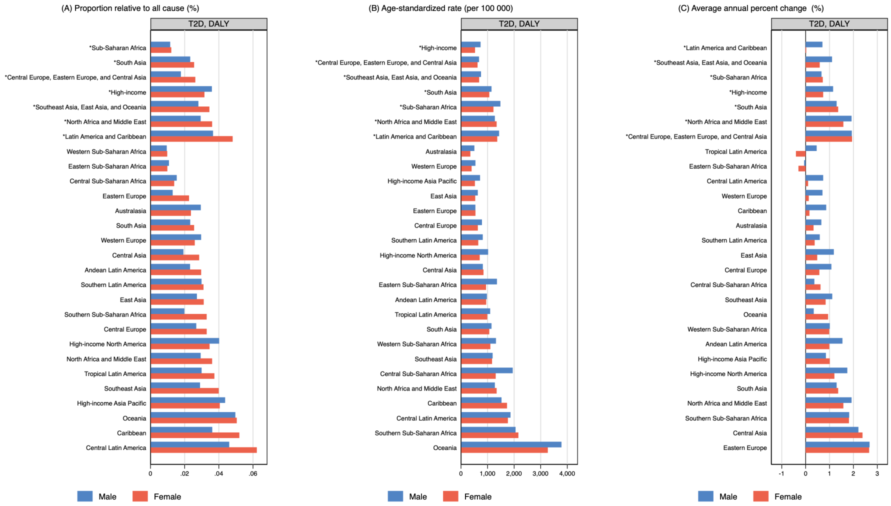

Mortality

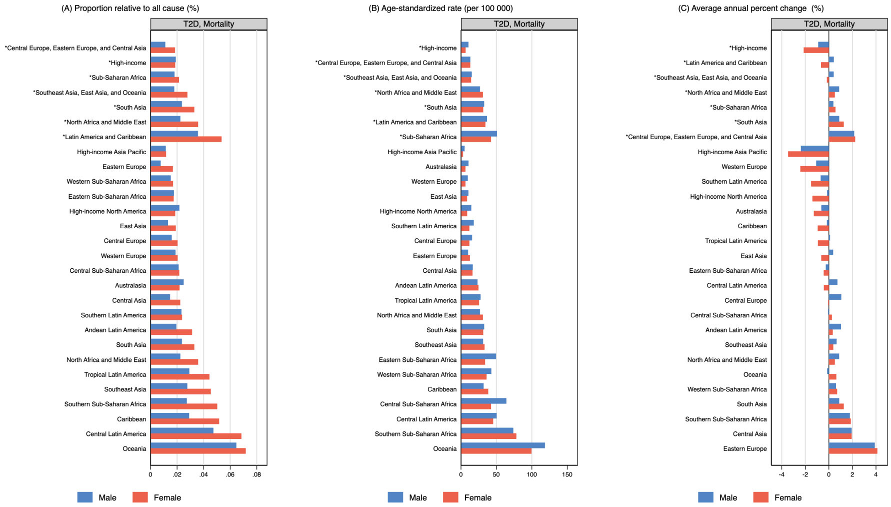

DALY: Disability adjusted life years; T2D: Type 2 diabetes. * Super-region.

Fig S8. Maps showing global age-standardized **prevalence, mortality, YLD and YLL rate** (per 100, 000) in 2021, as well as AAPC from 1990 to 2021, of **type 2 diabetes** in women

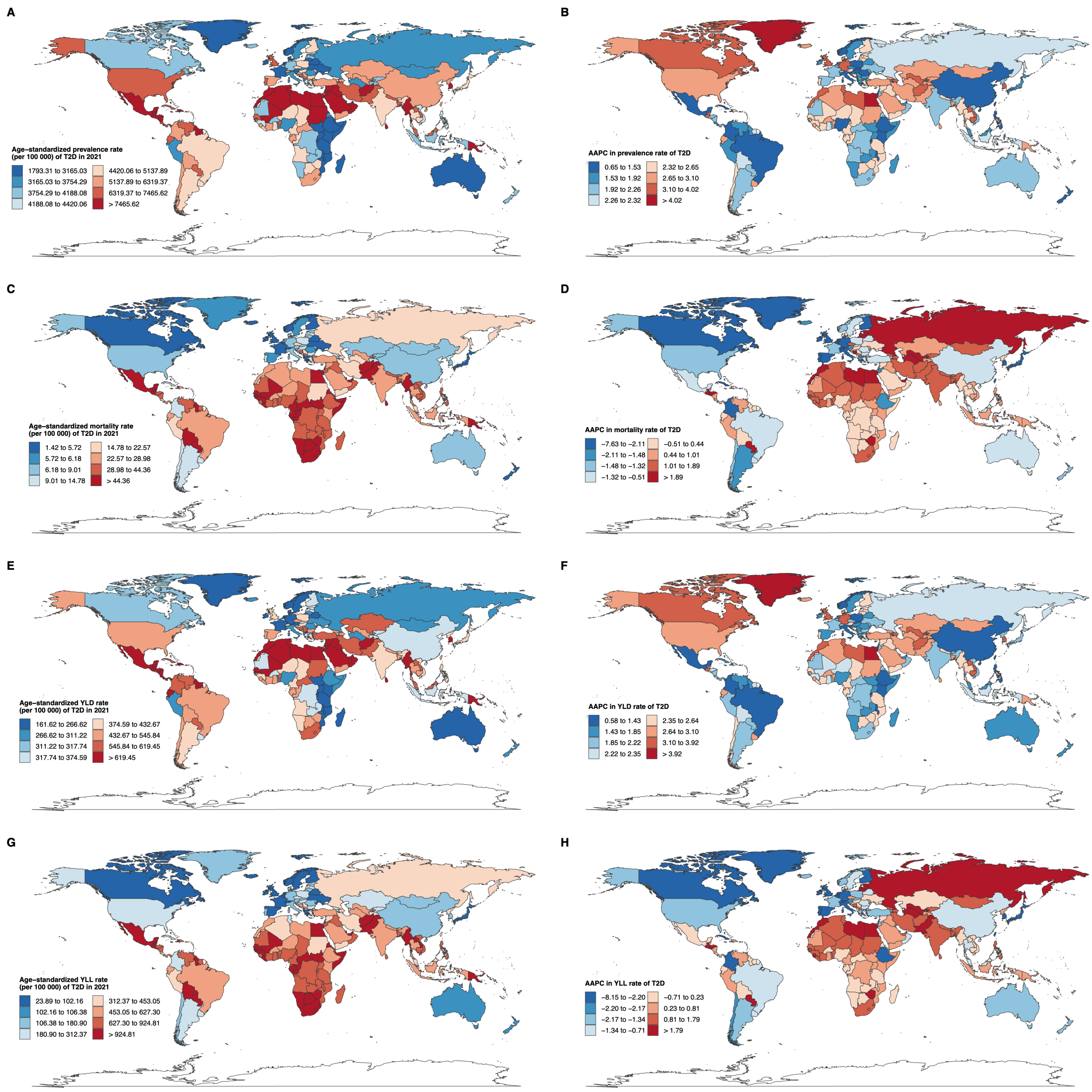

AAPC: average annual percentage change; T2D: Type 2 diabetes; YLD: Years lived with disability; YLL: Years of life lost.

Fig S9. Maps showing global **female-to-male ratio** of age-standardized incidence, prevalence, mortality and DALY rate in 2021, as well as **female-to-male difference** in corresponding AAPC from 1990 to 2021, of **type 2 diabetes**

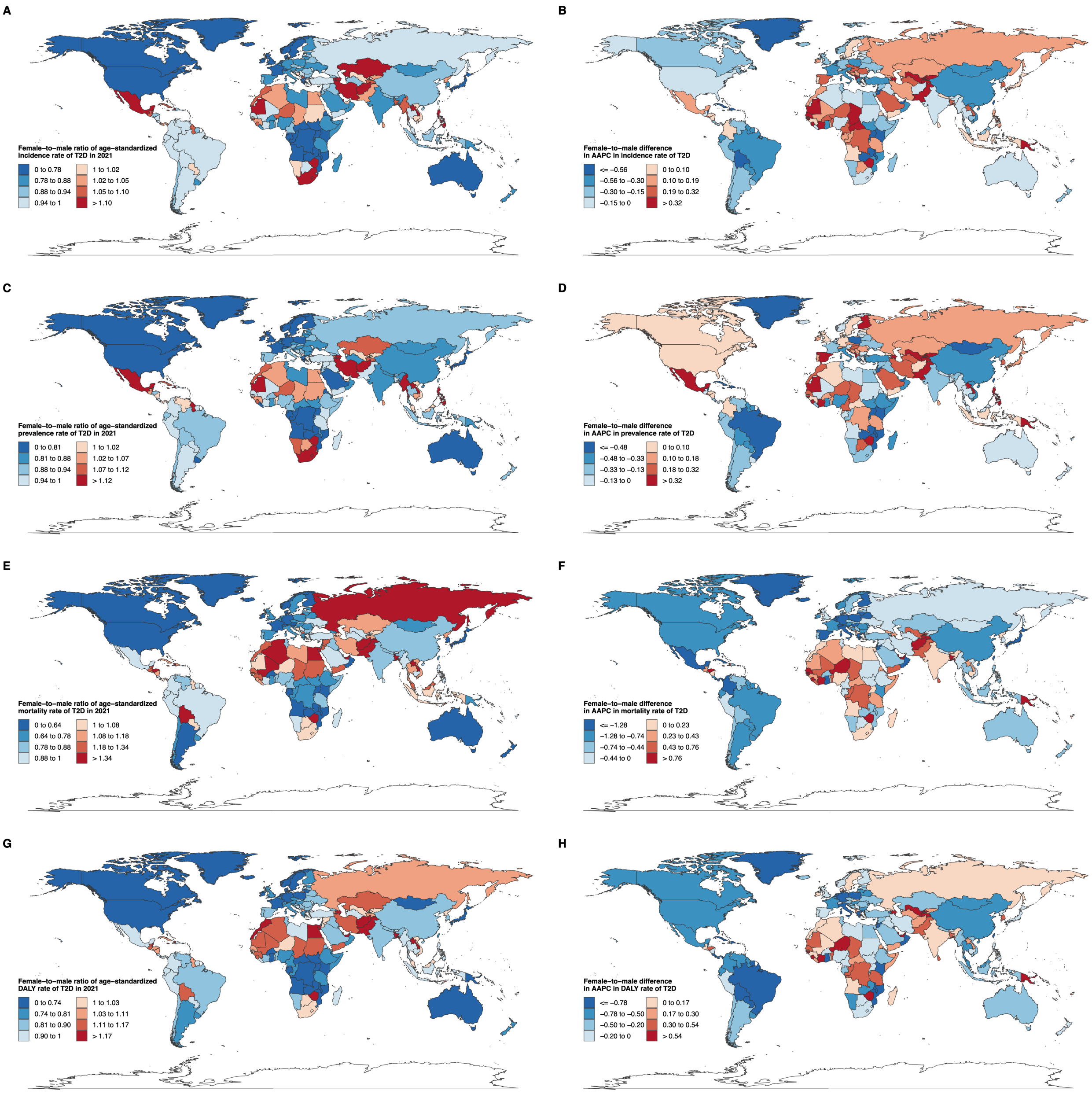

AAPC: Average annual percentage change; DALY: Disability adjusted life years; T2D: Type 2 diabetes.

Fig S10. The age-specific number, rate, and proportion relative to all causes across six metrics of diabetes for women in 1990, 2005, and 2021, along with population structures.

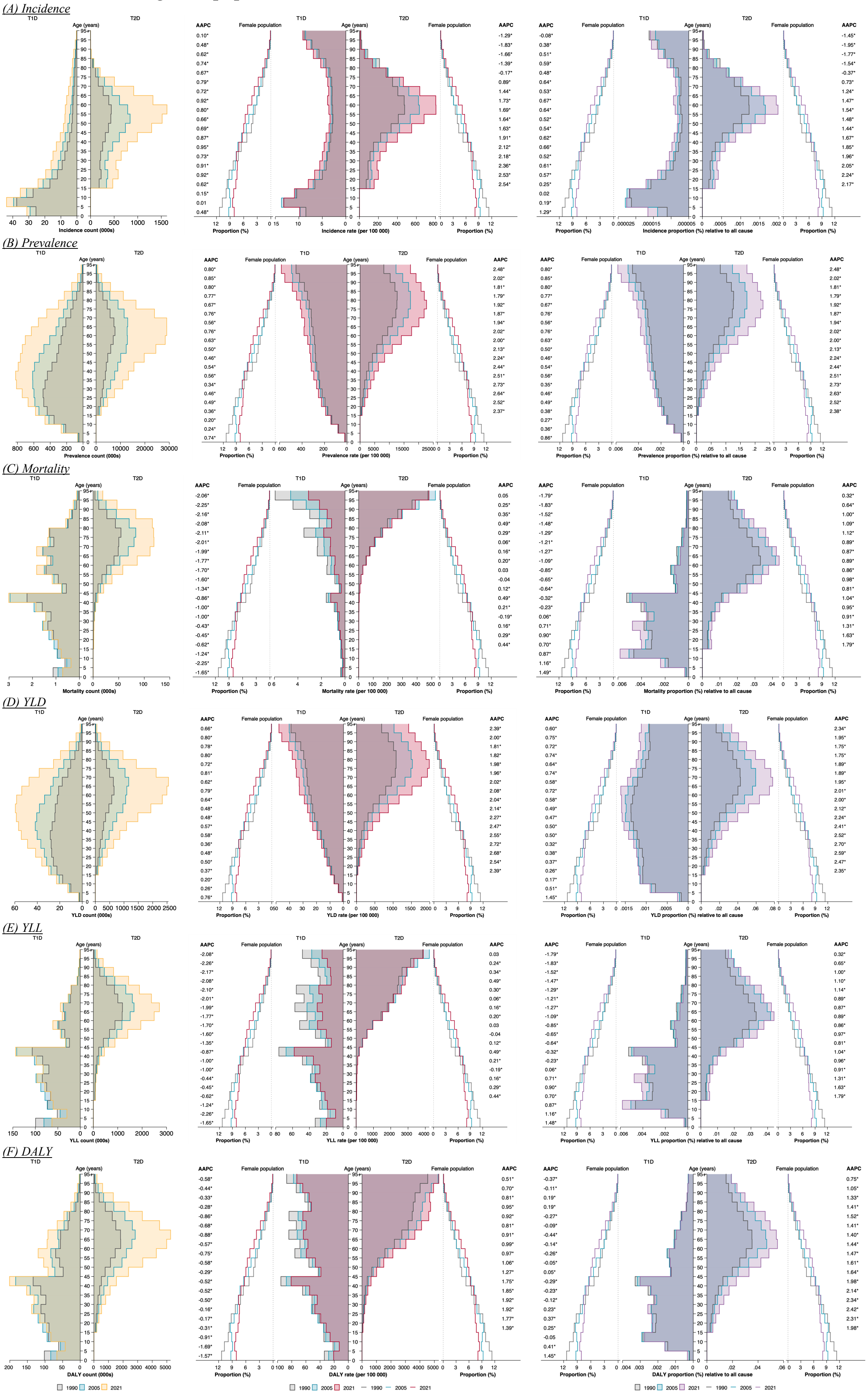

AAPC: Average annual percentage change; DALY: Disability adjusted; T1D: Type 1 diabetes; T2D: Type 2 diabetes; YLD: Years lived with disability; YLL: Years of life lost. *p<0.05.

Fig S11. Female-to-male ratio of **incidence** counts and rates for **type 1 diabetes** by age groups, 1990-2021

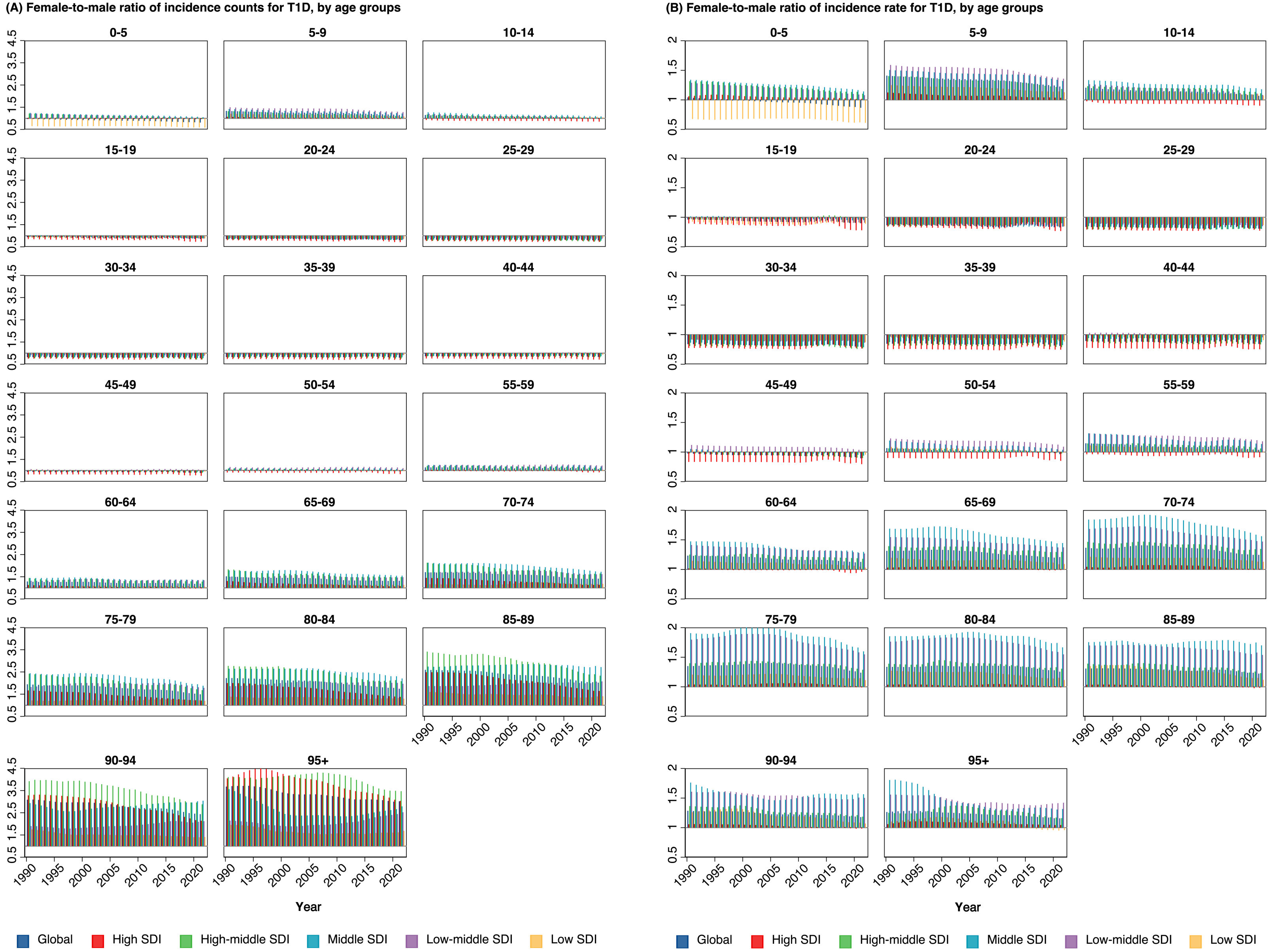

SDI: Sociodemographic index; T1D: Type 1 diabetes.

Fig S12. Female-to-male ratio of **prevalence** counts and rates for **type 1 diabetes** by age groups, 1990-2021

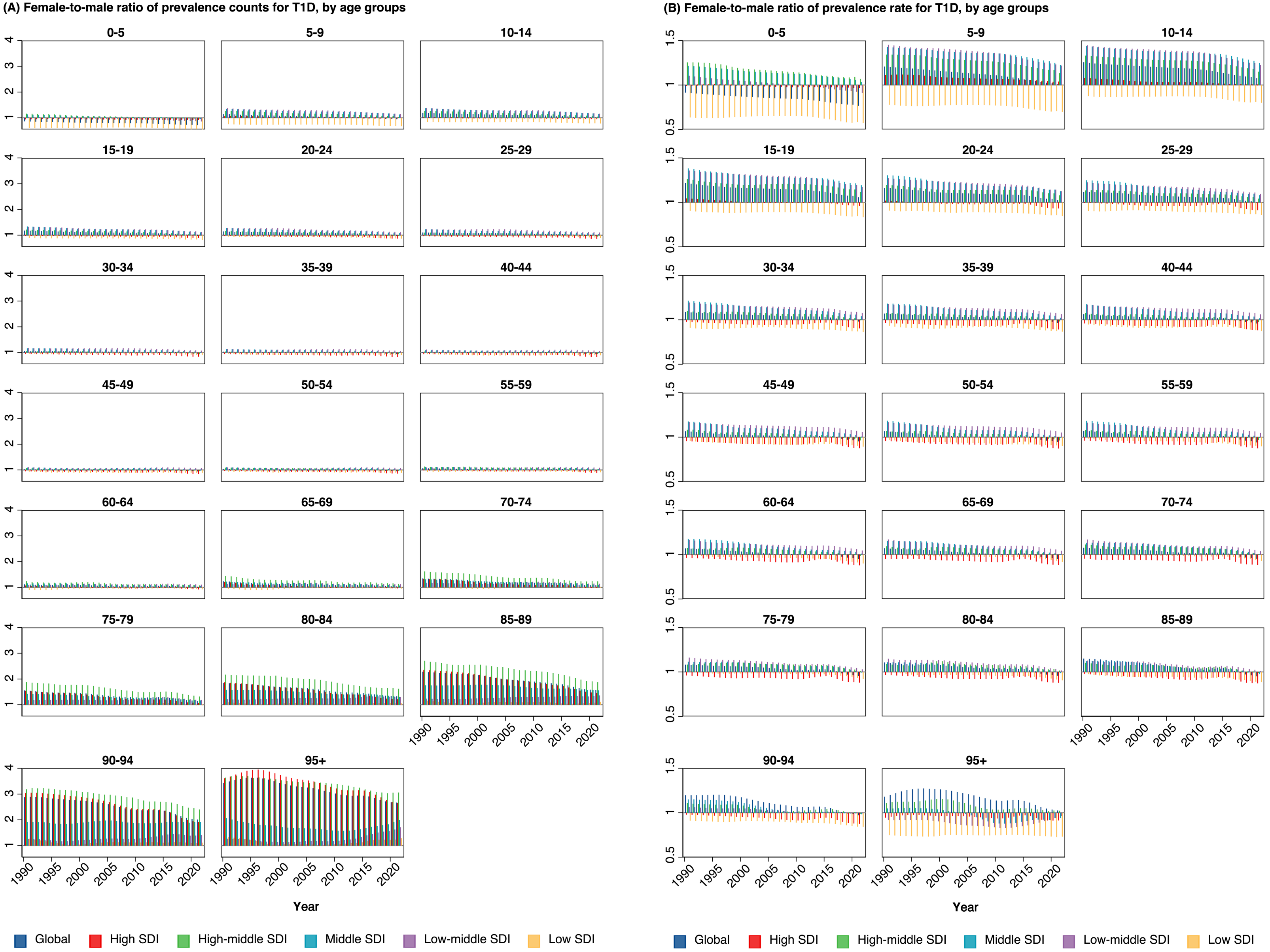

SDI: Sociodemographic index; T1D: Type 1 diabetes.

Fig S13. Female-to-male ratio of **mortality** counts and rates for **type 1 diabetes** by age groups, 1990-2021

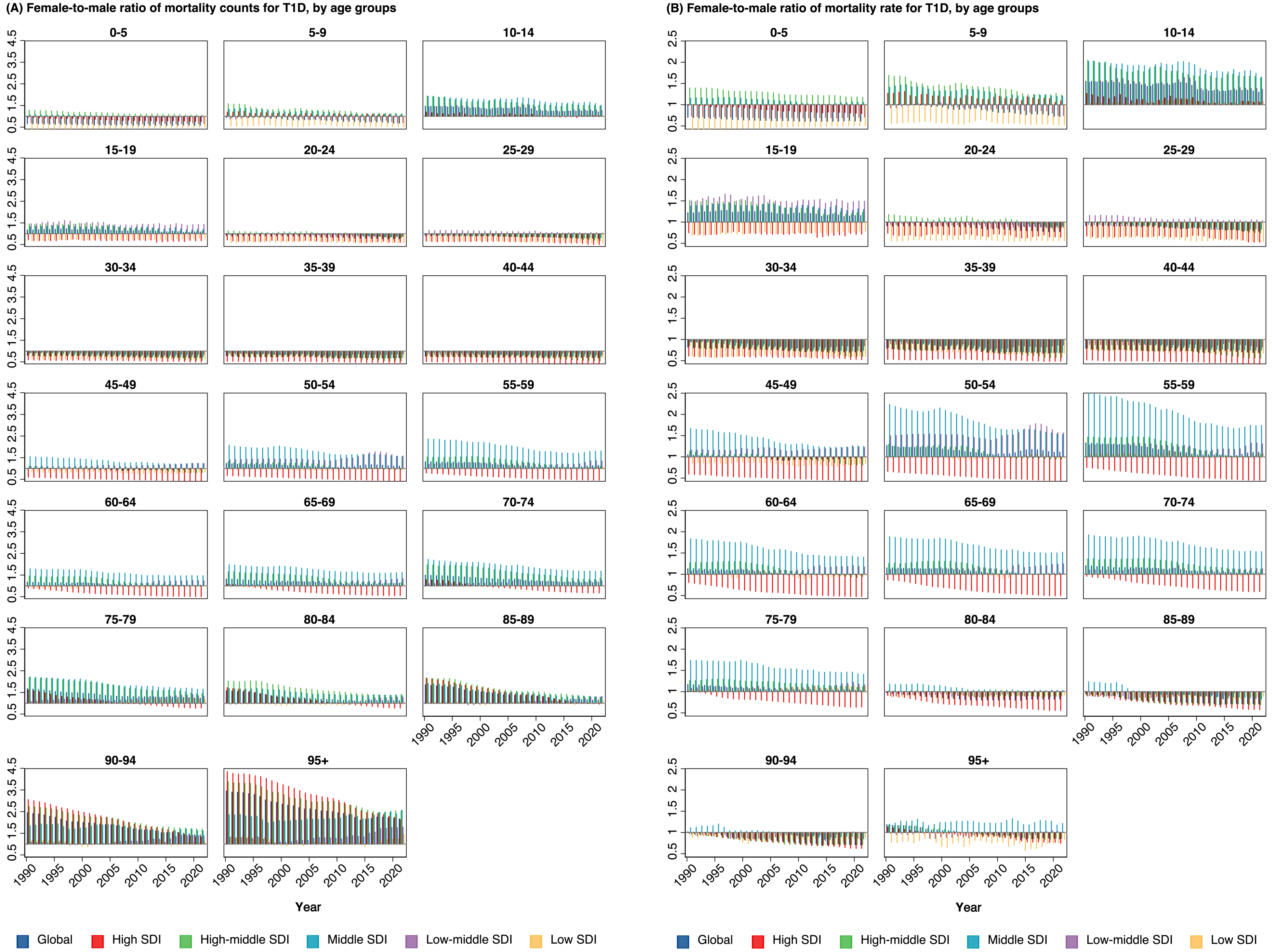

SDI: Sociodemographic index; T1D: Type 1 diabetes.

Fig S14. Female-to-male ratio of **DALY** counts and rates for **type 1 diabetes** by age groups, 1990-2021

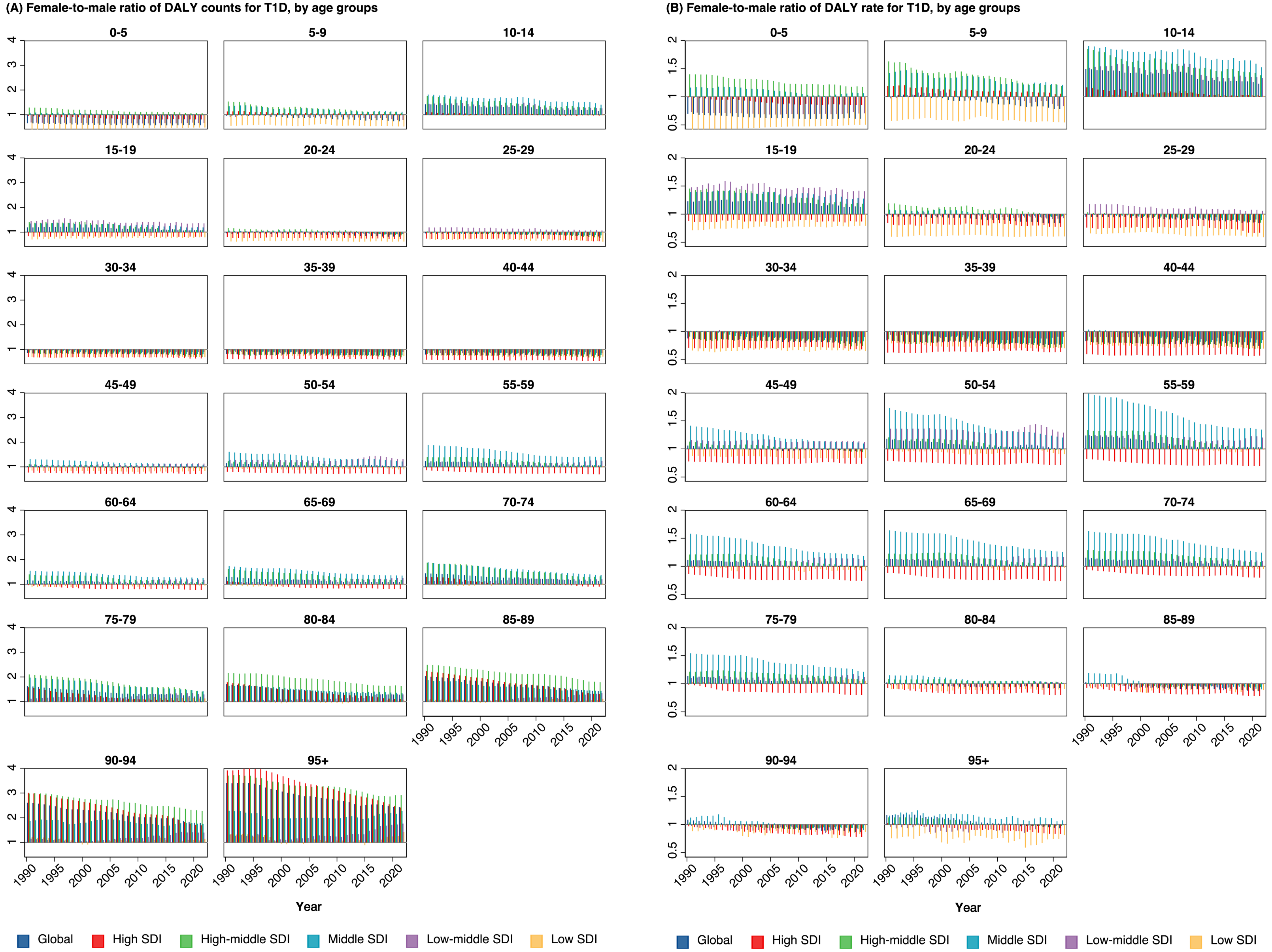

DALY: Disability adjusted life years; SDI: Sociodemographic index; T1D: Type 1 diabetes.

Fig S15. Comparison of the age-specific incidence rate of type 2 diabetes between men and women in 1990, 2005, and 2021, along with population structures

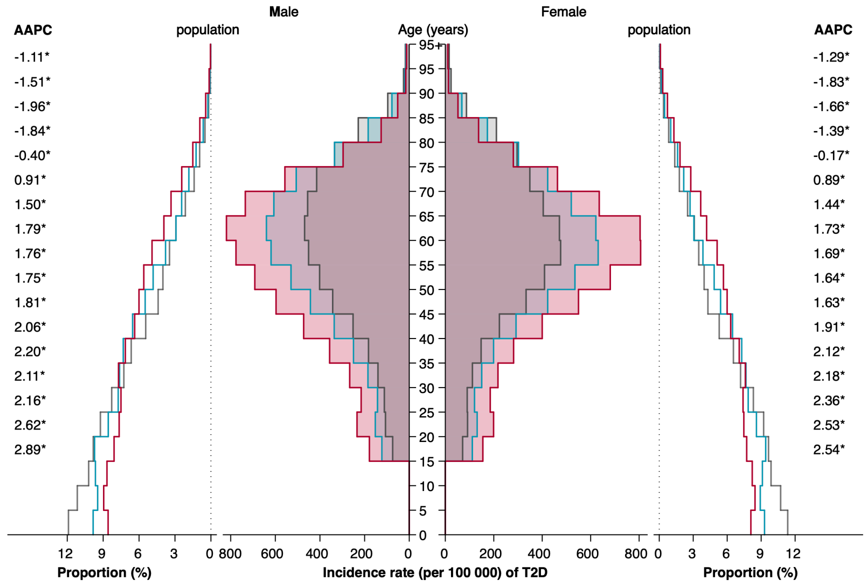

AAPC: Average annual percentage change; T2D: Type 2 diabetes. *p<0.05.

Fig S16. Correlations between age and AAPC in **incidence, DALY, and mortality** (rate and proportion) of type 1 and type 2 diabetes in women, at global and SDI levels

(A) Incidence

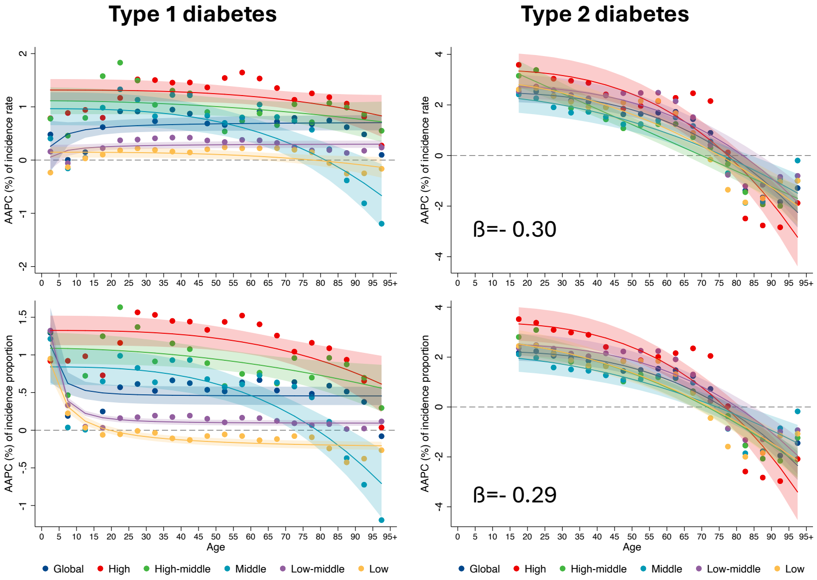

(B) DALY (Disability adjusted life years)

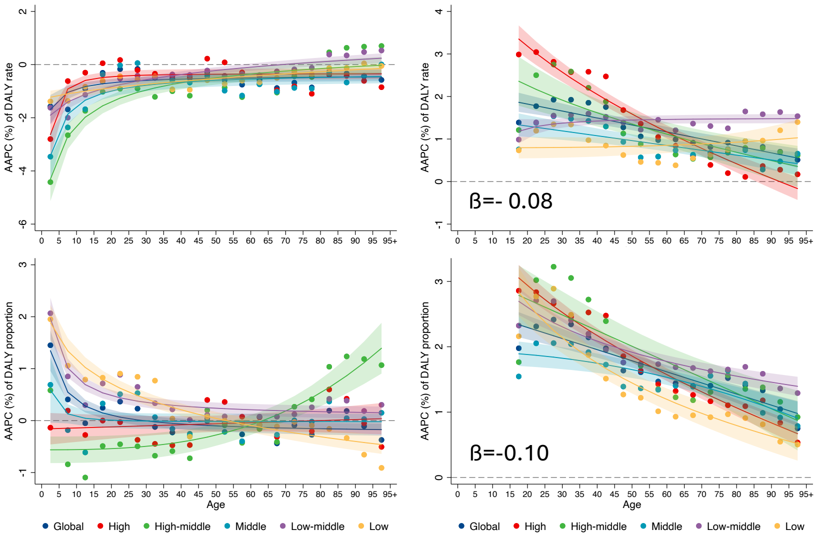

(C) Mortality

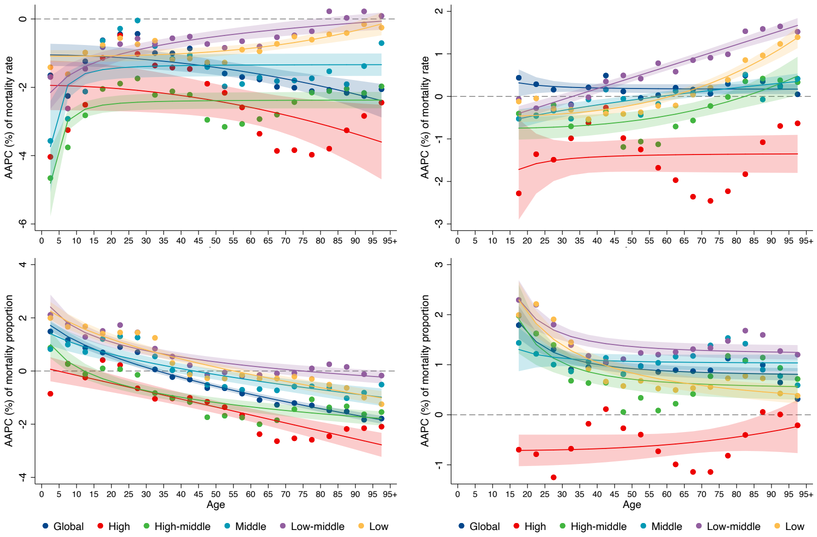

AAPC: Average annual percentage change; SDI: Sociodemographic index. For the incidence and DALY of type 2 diabetes, approximately linear associations were recorded between AAPC and age, with coefficients estimated by regressing AAPC on age groups after adjusting for the global and SDI levels.

Fig S17. Female-to-male ratio of **mortality** counts and rates for **type 2 diabetes** by age groups, 1990-2021

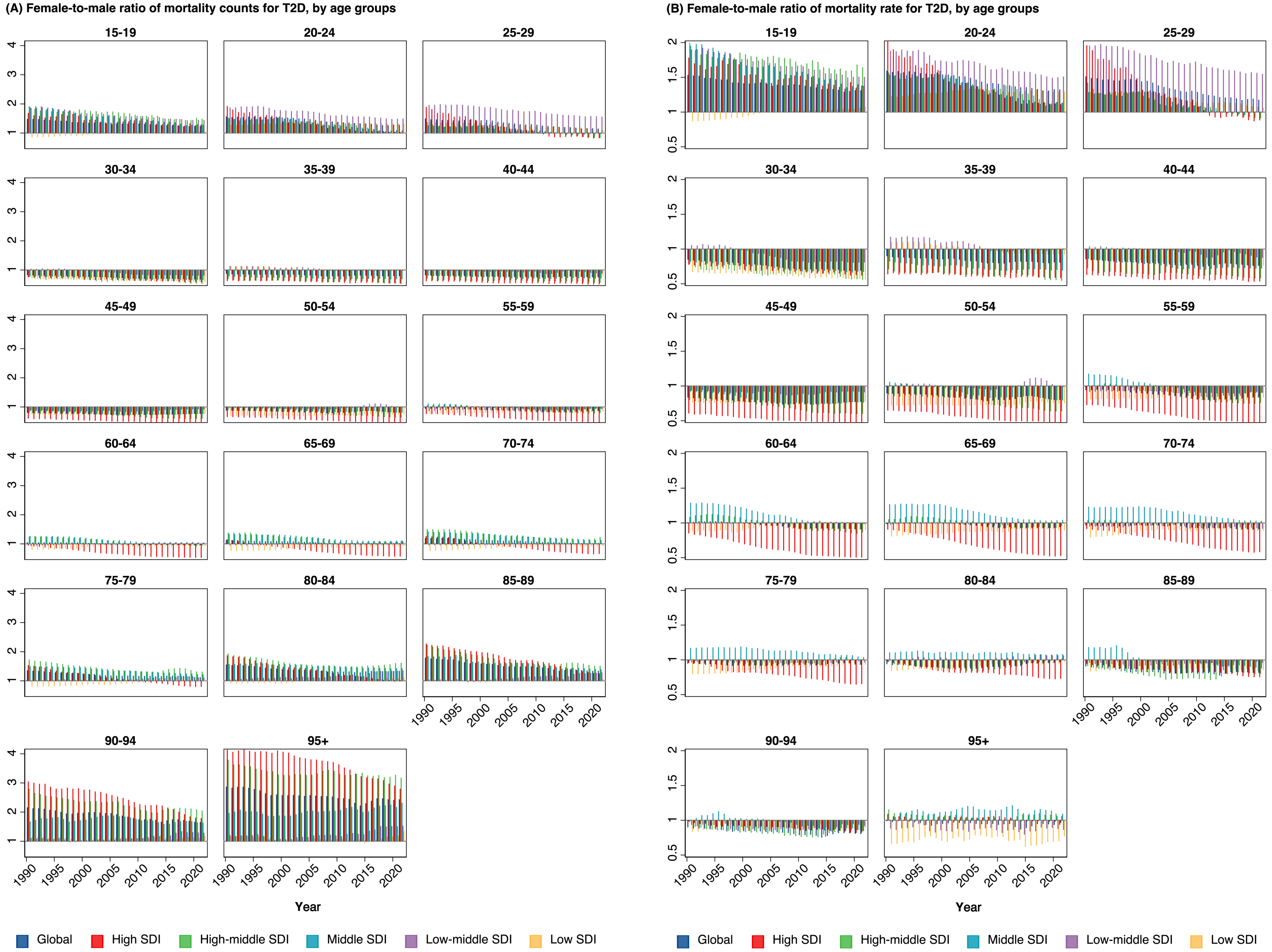

SDI: Sociodemographic index; T2D: Type 2 diabetes.

Fig S18. Female-to-male ratio of **incidence** counts and rates for **type 2 diabetes** by age groups, 1990-2021

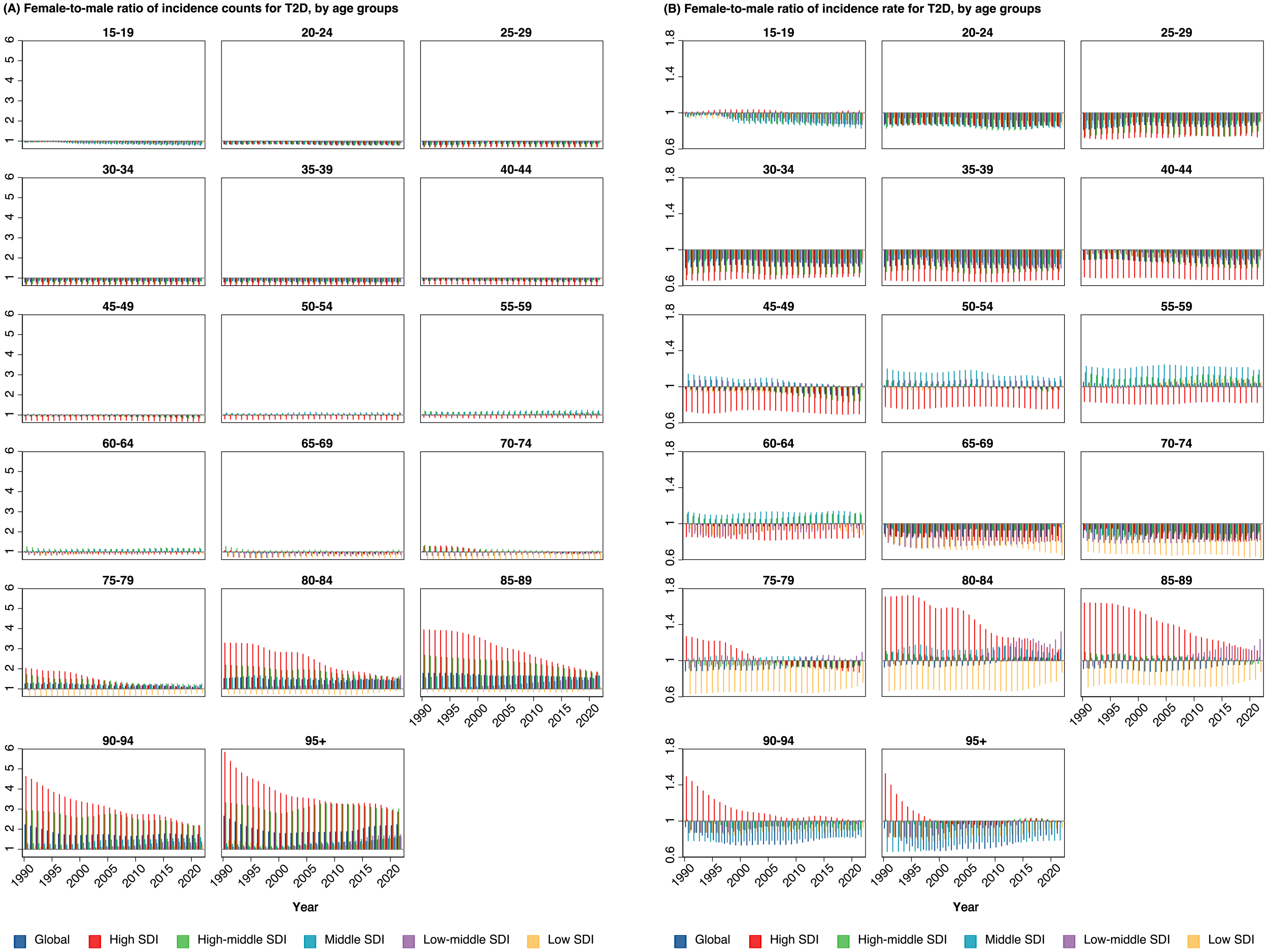

SDI: Sociodemographic index; T2D: Type 2 diabetes.

Fig S19. Female-to-male ratio of **prevalence** counts and rates for **type 2 diabetes** by age groups, 1990-2021

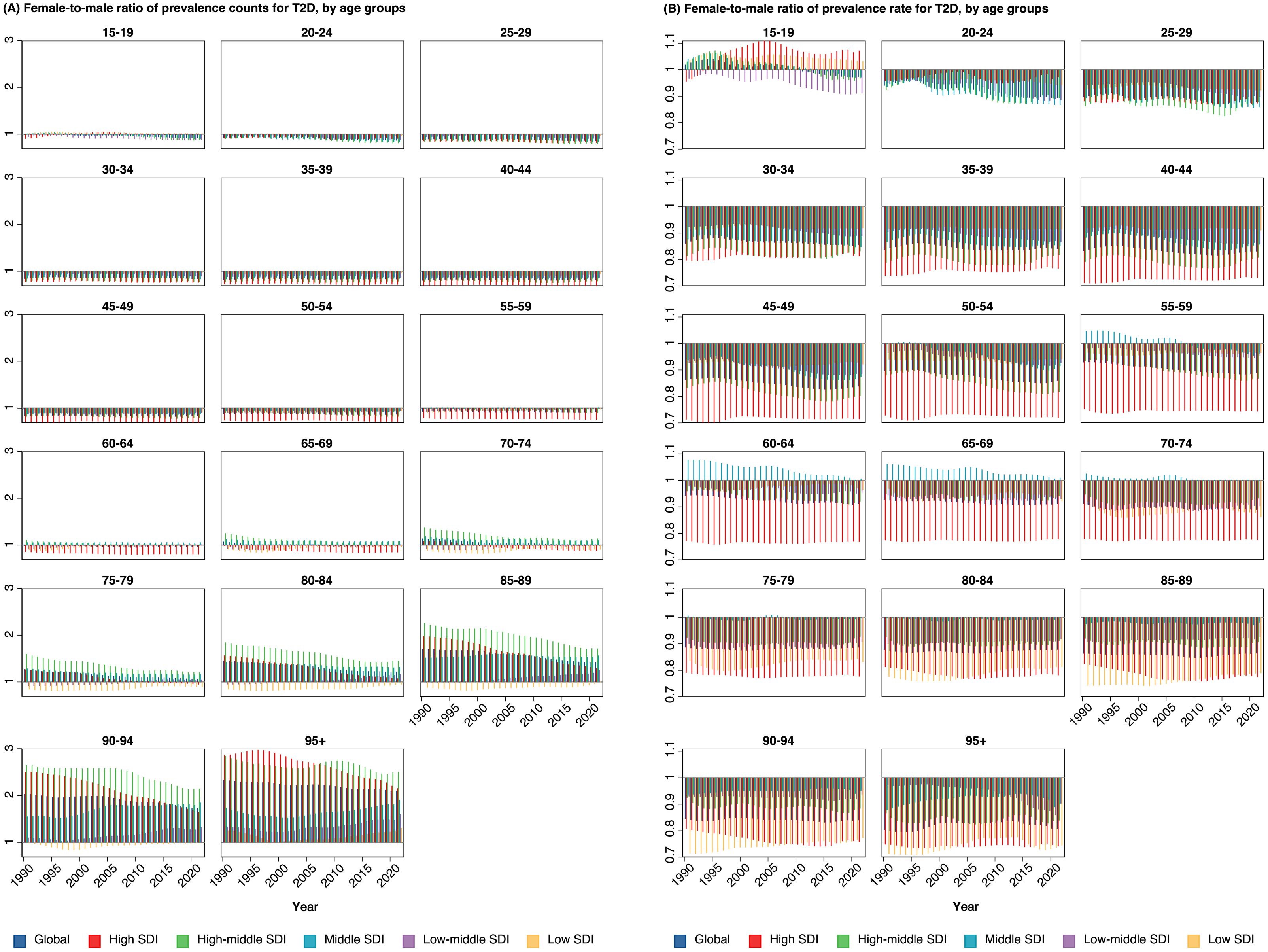

SDI: Sociodemographic index; T2D: Type 2 diabetes.

Fig S20. Female-to-male ratio of **DALY** counts and rates for **type 2 diabetes** by age groups, 1990-2021

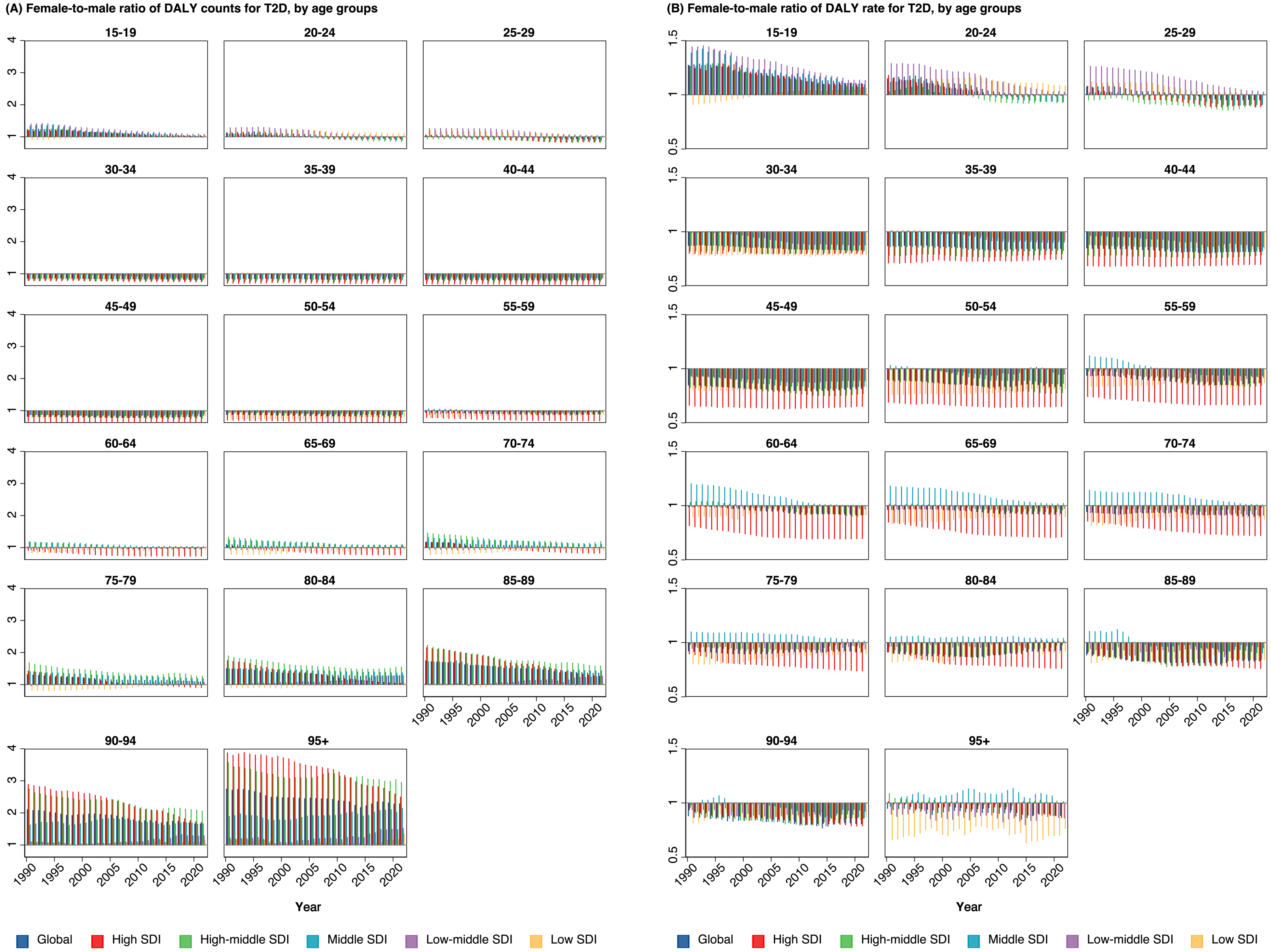

DALY: Disability adjusted life years; SDI: Sociodemographic index; T2D: Type 2 diabetes.

Fig S21. Contributions of detailed risk factors to DALY of type 1 and type 2 diabetes among women in 2021 and corresponding AAPCs from 1990 to 2021

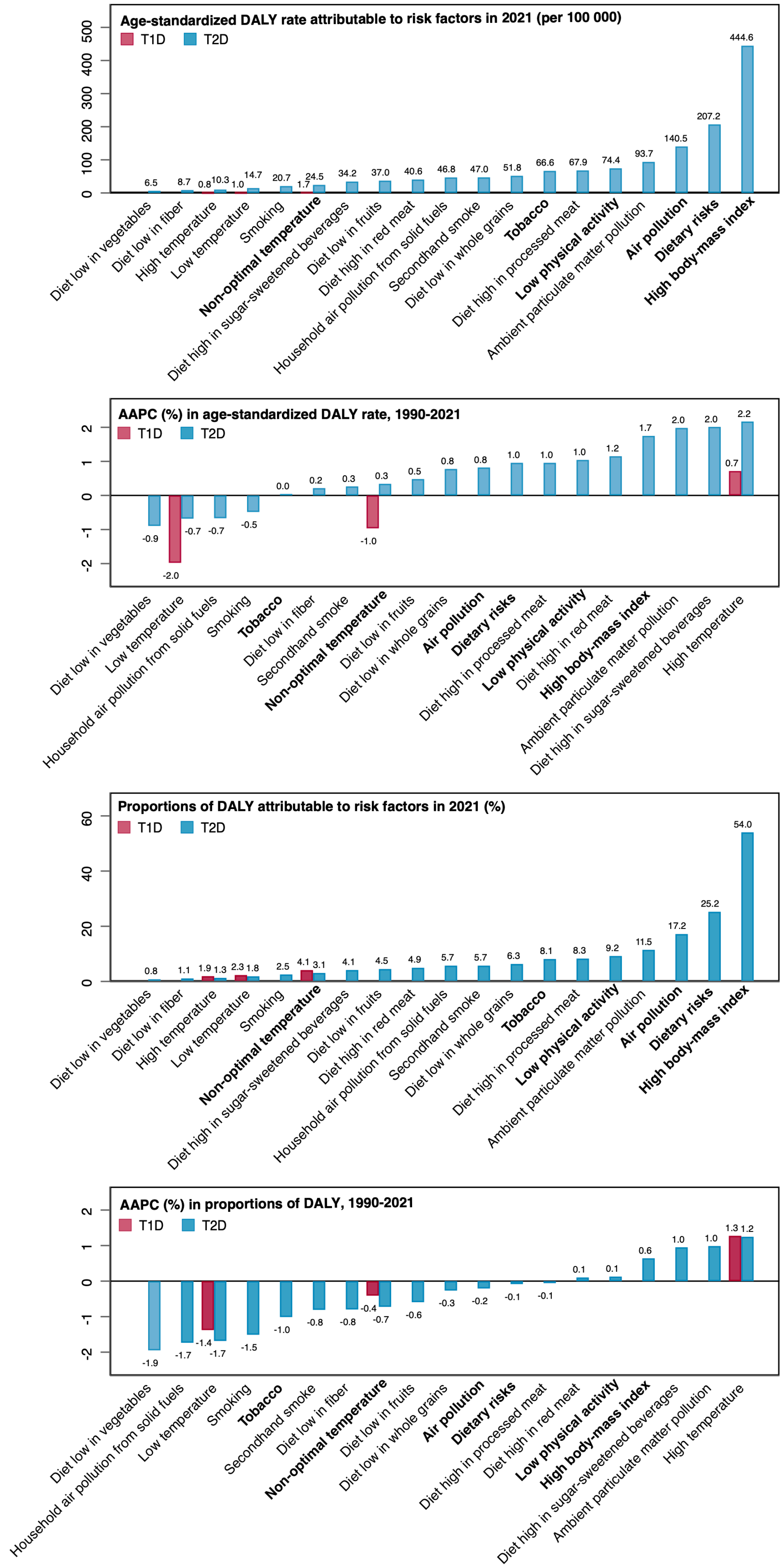

AAPC: Average annual percentage change; DALY: Disability adjusted life years; T1D: Type 1 diabetes; T2D: Type 2 diabetes.

Fig S22. Trends of contributions of more specific risk factors to type 1 and type 2 diabetes among women globally and by SDI, 1990-2021

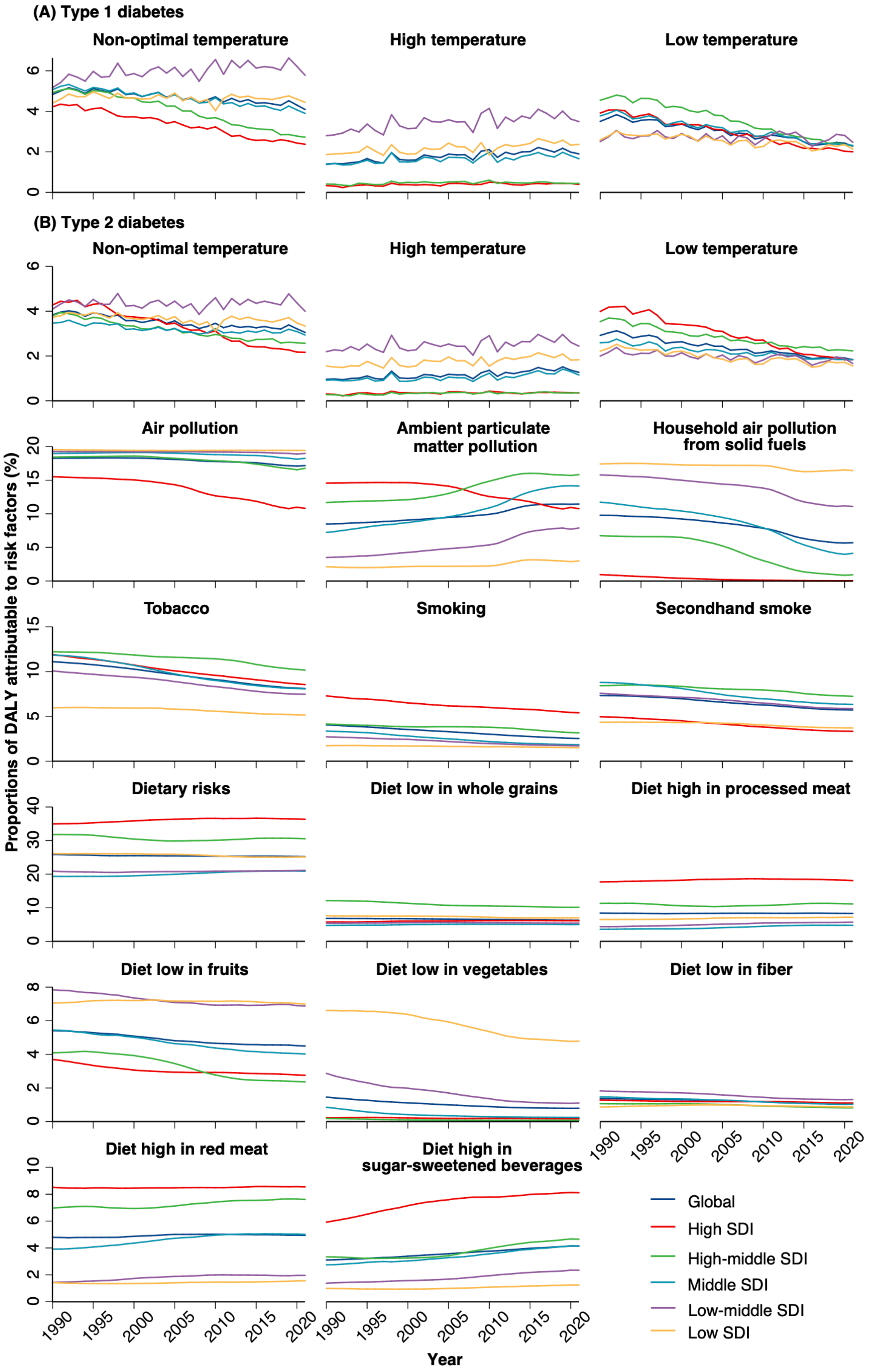

DALY: Disability adjusted life years; SDI: Sociodemographic index.

Fig S23. Age-specific differences between women and men in proportional DALY attributable to more specific risk factors (%) for type 1 and type 2 diabetes in 2021, globally and by SDI

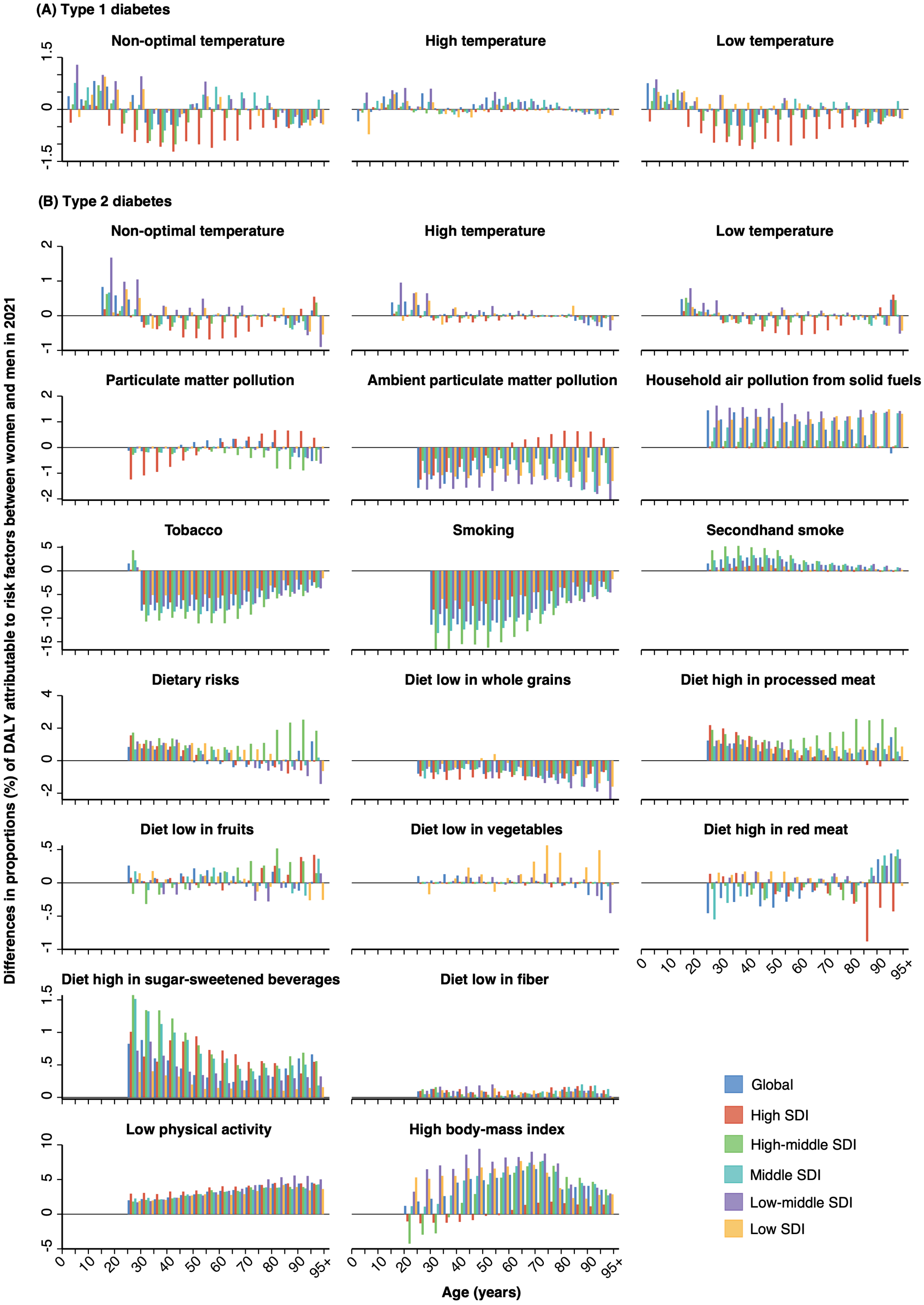

DALY: Disability adjusted life years; SDI: Sociodemographic index.

Fig S24. Age-related characteristics in terms of proportions of DALY attributable to more specific risk factors (%) for type 1 and type 2 diabetes among women in 2021, globally and by SDI

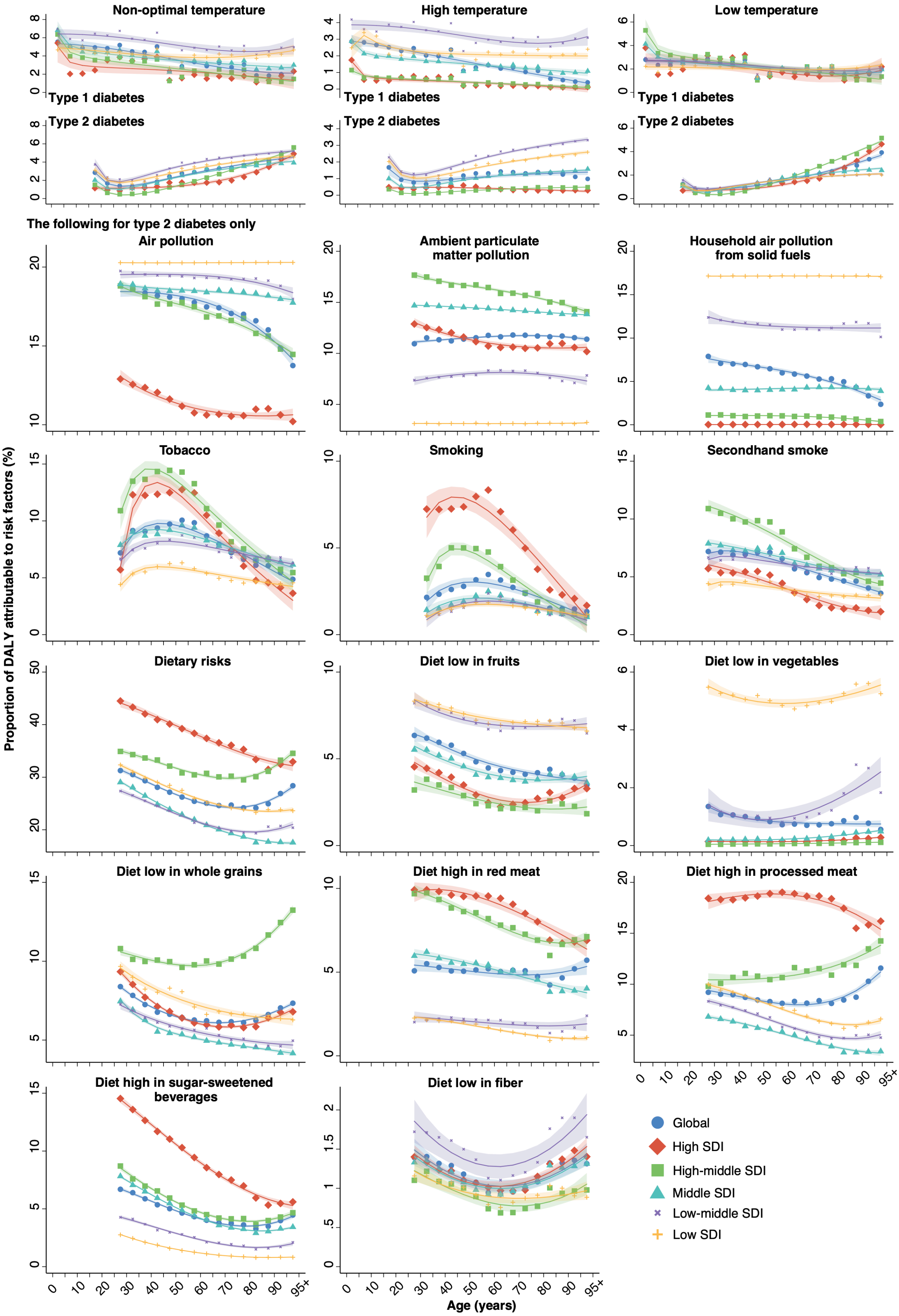

DALY: Disability adjusted life years; SDI: Sociodemographic index.

Fig S25. Global all-age counts and age-standardized rates of DALY, YLD, and YLL for **type 1 diabetes** and **type 2 diabetes** among women, for the past and for five future scenarios, 1990–2050

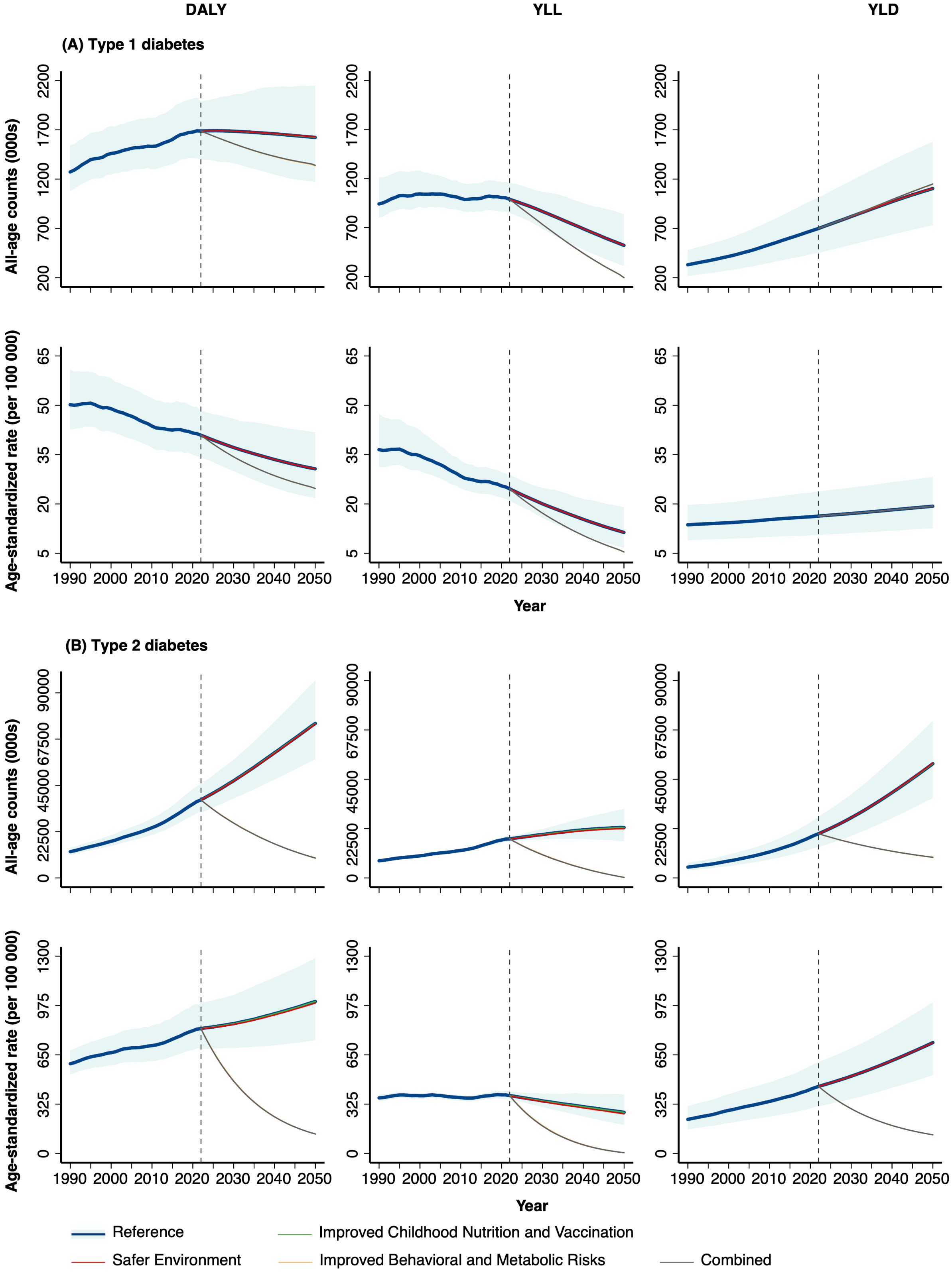

DALY: Disability adjusted life years; SDI: Sociodemographic index; YLD: Years lived with disability.

Fig S26. All-age counts (A) and age-standardized rates (B) of DALY, YLD, and YLL for **type 1 diabetes** among women by SDI levels, for the past and for five future scenarios, 1990–2050

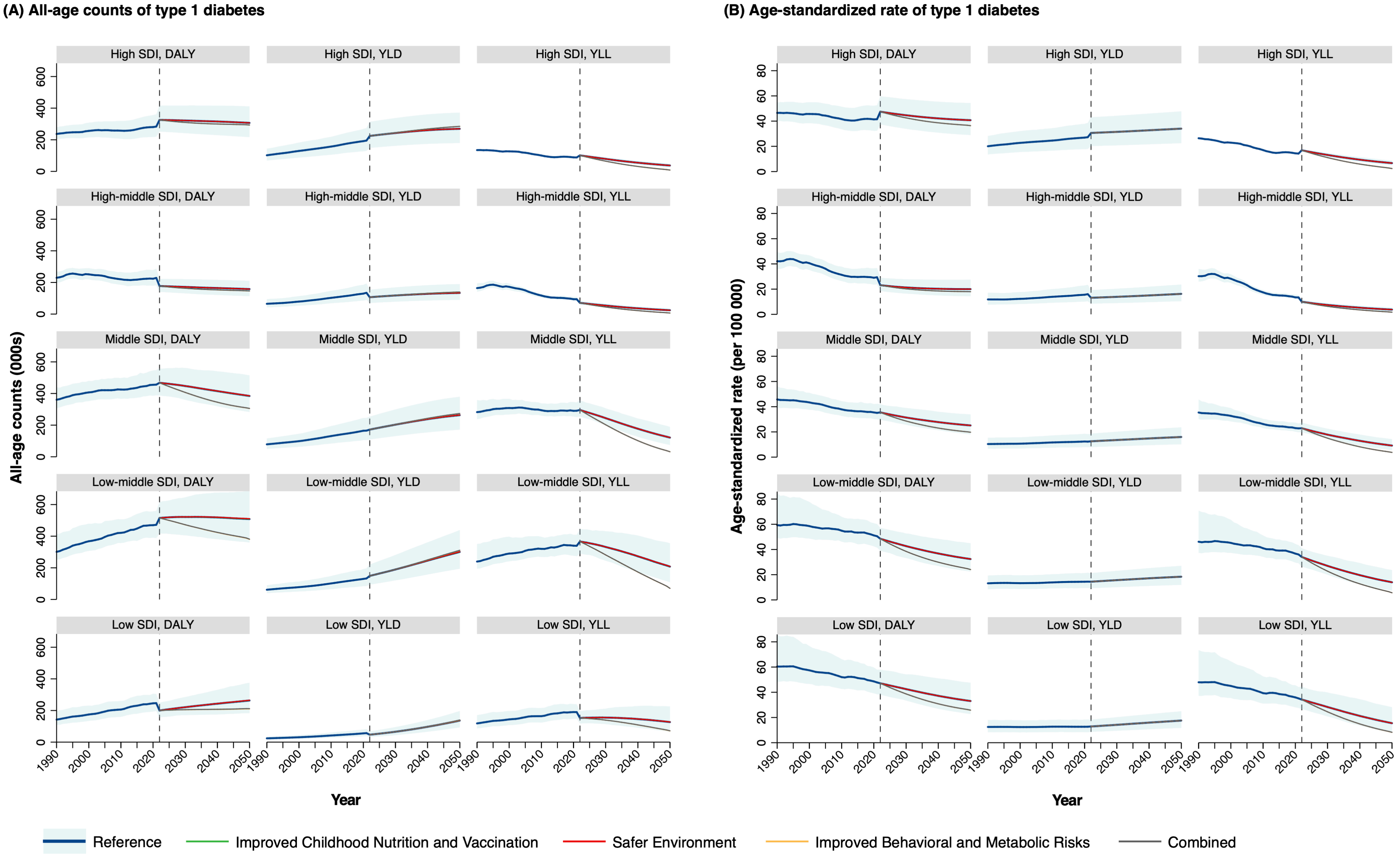

DALY: Disability adjusted life years; SDI: Sociodemographic index; YLD: Years lived with disability; YLL: Years of life lost.

Fig S27. All-age counts (A) and age-standardized rates (B) of DALY, YLD, and YLL for **type 2 diabetes** among women by SDI levels, for the past and for five future scenarios, 1990–2050

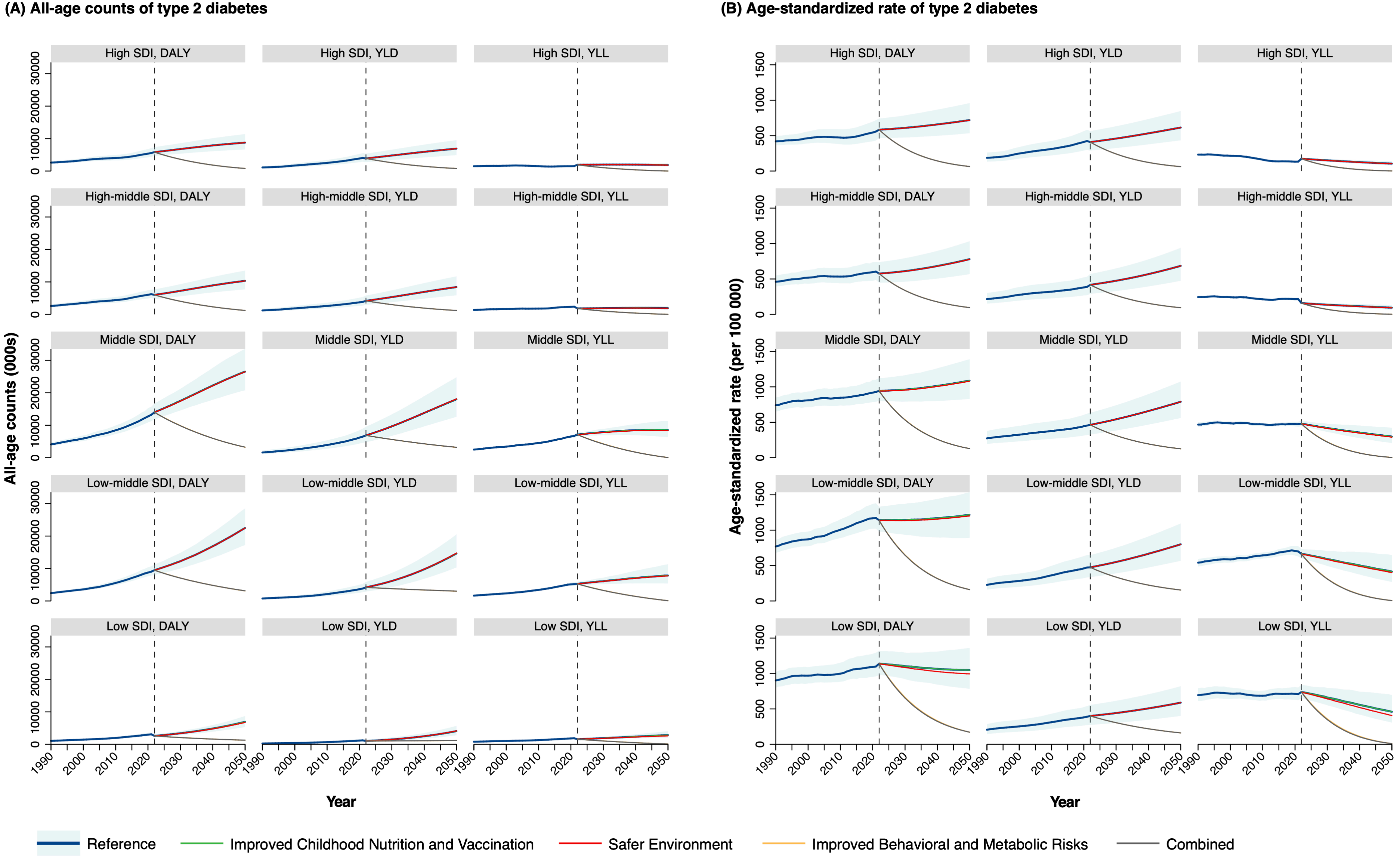

DALY: Disability adjusted life years; SDI: Sociodemographic index; YLD: Years lived with disability; YLL: Years of life lost.
